# Combinations of Protein-Chemical Complex Structures Reveal New Targets for Established Drugs

**DOI:** 10.1371/journal.pcbi.1002043

**Published:** 2011-05-05

**Authors:** Olga V. Kalinina, Oliver Wichmann, Gordana Apic, Robert B. Russell

**Affiliations:** 1 Cell Networks, University of Heidelberg, Heidelberg, Germany; 2 Institute for Information Transmission Problems, RAS, Moscow, Russia; 3 Cambridge Cell Networks Ltd, St. John's Innovation Centre, Cambridge, United Kingdom; Fox Chase Cancer Center, United States of America

## Abstract

Biological networks are powerful tools for predicting undocumented relationships between molecules. The underlying principle is that existing interactions between molecules can be used to predict new interactions. Here we use this principle to suggest new protein-chemical interactions via the network derived from three-dimensional structures. For pairs of proteins sharing a common ligand, we use protein and chemical superimpositions combined with fast structural compatibility screens to predict whether additional compounds bound by one protein would bind the other. The method reproduces 84% of complexes in a benchmark, and we make many predictions that would not be possible using conventional modeling techniques. Within 19,578 novel predicted interactions are 7,793 involving 718 drugs, including filaminast, coumarin, alitretonin and erlotinib. The growth rate of confident predictions is twice that of experimental complexes, meaning that a complete structural drug-protein repertoire will be available at least ten years earlier than by X-ray and NMR techniques alone.

## Author Summary

Predicting drug-target interactions is a hot topic, and many efforts have been undertaken to do this, many using large interaction networks. We take a novel approach using protein-chemical interactions derived from 3D structures. The basic premise is that two proteins sharing a common bound chemical will likely share others. We use protein and chemical superimpositions and physical tests of chemical-protein compatibility to identify the most likely candidates among the nearly one million potential interactions. We show for a benchmark that known protein-chemical structures are reconstructed with good accuracy and sometimes via very different proteins and chemicals. We make thousands of confident predictions, including structures for known protein-drug interactions lacking a structure (e.g. topoisomerase-2/radicicol) and many new interactions. The number of confident predictions grows faster than the number of known structures, suggesting that this approach will play a key role in completing the protein-chemical interaction repertoire.

## Introduction

Large biological networks have been used previously to suggest protein-protein interactions [1], phosphorylation events [2] and most recently drug-protein interactions. New drug-protein relationships have been proposed from the analysis of shared side-effects [3], by comparing sets of protein targets according to drug pairs [4], or sets of targets for particular drugs [5], [6]. Though not always considered as such, the database of protein three-dimensional (3D) structures is also a large network, where links are physical associations between molecules within structurally determined complexes. The network contains many thousands of protein-protein and protein-chemical interactions, of which several hundred involve drugs. In this paper we explored this large network systematically to predict new potential protein-chemical interactions.

**Figure 1:**
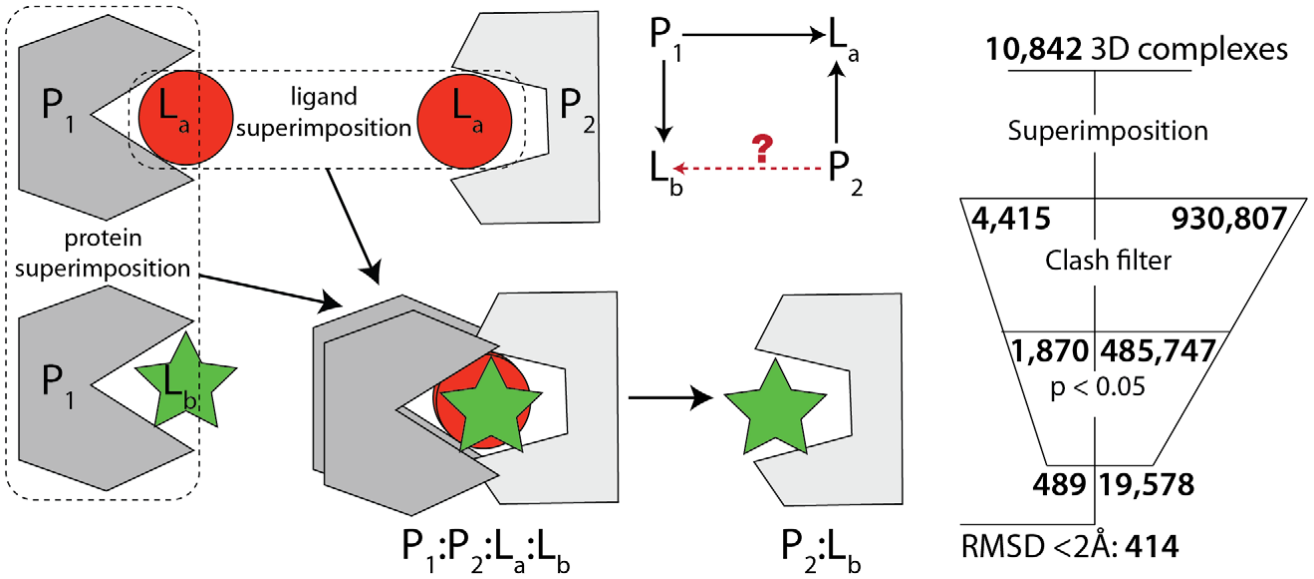
Schematic outlining the method used to predict protein-chemical interactions (left), and summary of how prediction candidates survive the clash filter and how many have statistically significant scores (right).

We exploited the basic premise that if two proteins in the network share one bound chemical they are likely to share others. Considering protein-chemical interactions alone would lead to many thousands of predictions including mostly false positives. However, we profit here from the use of 3D structures, where we can use physicochemical criteria to remove false predictions. A single prediction candidate (Figure 1) involves combining three protein-chemical complex structures, two of which involve two distinct proteins (P_1_ and P_2_) binding a common ligand (L_a_) and a third where one protein (P_1_) binds another ligand (L_b_). By superimpositions based on the common protein and the common ligand, we obtain an additional putative complex (P_2_ with L_b_). We then used several criteria to decide whether or not these new complexes were structurally viable and evaluated the statistical significance using a p-value (see Methods). From 10,842 complexes forming the network of known structures, we identified 907,827 potential interactions, of which 20,067 (including 19,578 novel structures and 489 complexes with a previously determined structure) were significant (p≤0.05). Note that we ignored trivial candidates where the two proteins (P_1_ and P_2_) shared ≥80% identity (i.e. where ligand transference would be very likely due to orthology). The predictions include enzyme/substrate, enzyme/product, target/inhibitor and target/activator structures (Table S1 in Text S1).

## Results

### Benchmarking

For the benchmark, we selected those protein-chemical complexes of known structure that could, in principle, be predicted by superimpositions of chemicals and proteins in a non-obvious fashion. Specifically, we considered only pairs of proteins with less than 80% sequence identity; and non-identical chemicals. Predictions made with identical (or very similar) chemicals or proteins are less interesting as they represent cases where transference is more obvious: for instance testing a chemical inhibitor of a human protein in a mouse orthologue, or inferring that a slightly modified chemical compound might have a similar activity. The number of these complexes is small relative to the total number of predictions owing to these requirements. That is, it is currently relatively unlikely that these combinations exist given the frequency of chemicals solved in complex with multiple distinct protein structures. There were thus only 376 complexes of known structure that were also predicted significant (p≤0.05) by our method using different protein-chemical complexes in the network. For 270 (72%) of them, the ligand in the predicted complex had an RMSD <2 Å when compared to the known structure, which matches standards acceptable for docking solutions (e.g. [7]).

**Figure 2:**
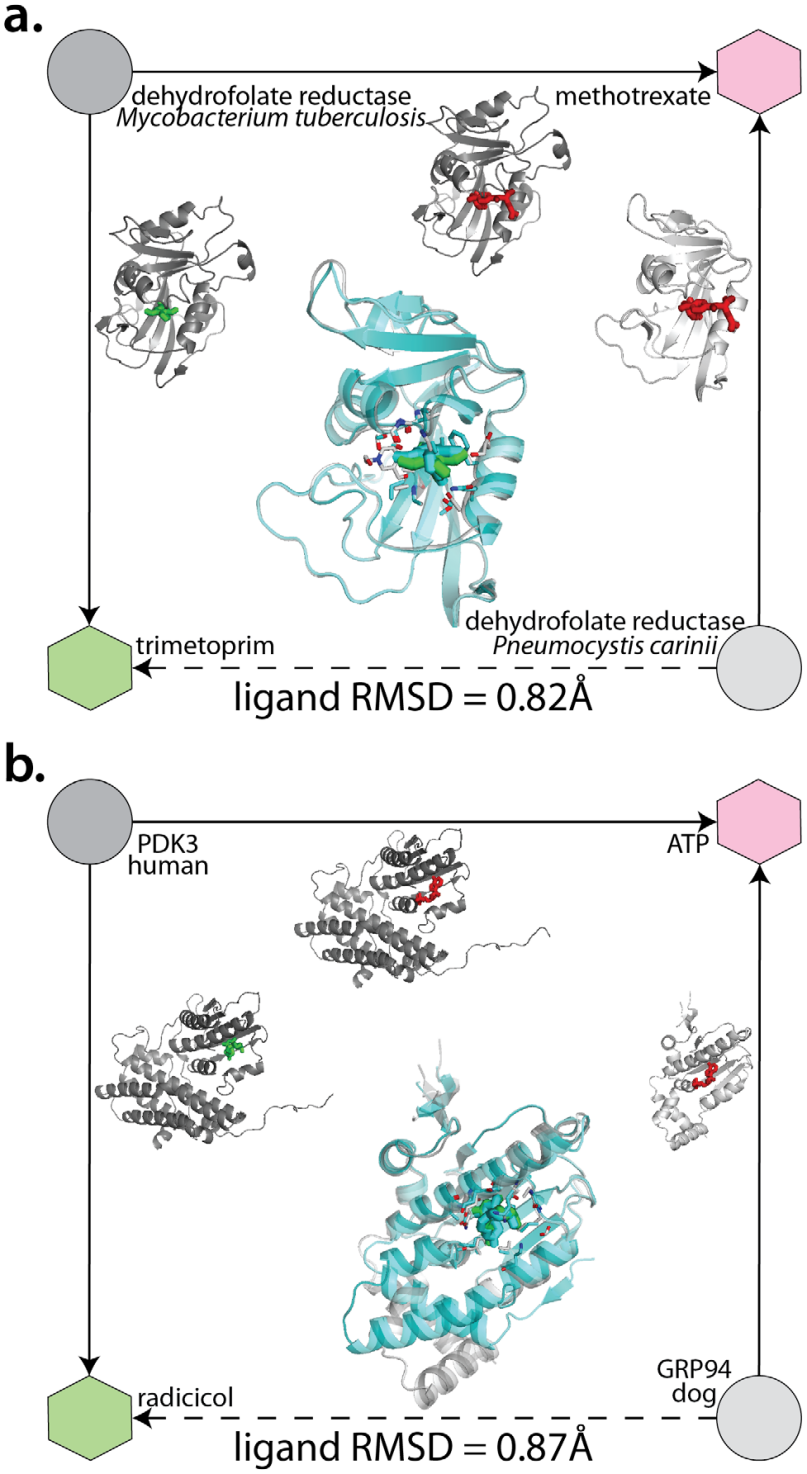
Examples from the benchmark dataset of high-quality predictions. (A): High-identity subset: the complex of dihydrofolate reductase from *Pneumocystis carinii* with trimethoprim predicted using complexes with dihydrofolate reductase from *Mycobacterium tuberculosis* (with trimethoprim, PDB code 1DG5) and methotrexate (with dihydrofolate reductase from *Pneumocystis carinii*, 1DF7, with dihydrofolate reductase from *Mycobacterium tuberculosis*, PDB code 3CD2). RMSD from a known 3D structure (PDB code 1DYR) is 0.82 Å. (B): Low-identity or non-homology subset: the complex of endoplasmin GRP94 with radicicol using complexes with pyruvate dehydrogenase kinase isoform 3 (PDK3) (with radicicol, PDB code 2Q8I) and ATP (with PDK3, PDB 1Y8O, with CRP94, PDB 1TC6). RMSD from a known 3D structure (PDB 1QY8) is 0.87 Å.

There are several revealing instances where interactions already of known structure are predicted correctly via complex paths. For instance, we accurately predicted the complex of *Pneumocystis carinii* dihydrofolate reductase (DHFR) with trimethoprim. This prediction was made since both this DHFR and a remote homolog (36% identity) from *Mycobacterium tuberculosis* have known structures in complex with methotrexate, and the latter has been solved with trimethoprim (Figure 2A). We also made accurate predictions using radically different proteins that nevertheless share a common ligand. For example, we successfully reconstruct the complex of the endoplasmic reticulum paralog of the chaperone Hsp90 (GRP94) with its high-affinity inhibitor radicicol, by exploiting the known complexes of pyruvate dehydrogenase kinase (PDK3) with radicicol and both proteins with ATP (Figure 2B). This prediction is remarkable in that neither the protein sequences nor the chemicals involved are detectably similar (though PDK3 is a remote homologue of GRP94, detectable only via structure comparison).

The difficulty in establishing false-positive rates in molecular interaction studies is well established (e.g. ref [8], [9]), and is principally due to a lack of known negatives: pairs of molecules known not to interact. We suffer from the same situation here, as there is currently no standardized set of protein-chemical interactions known not to interact. We can, however, get a set of such interactions from screening studies, where it is at least known that the chemicals and the proteins did not interact under the conditions used. Extracting these from PubChem gives 63,218,098 potential negative interactions, of which only 172 overlap with the predictions made here. The low number is due to the fact that very few of the screens in PubChem can be unambiguously assigned to a single protein structure, and that chemicals both in screens and known structures are very often unique. We obtained positive predictions for just 17 of these giving a false positive rate of 9.9%.

A natural question is how conformational flexibility impacts on our predictions. We do not attempt to explore alternative conformations, instead letting the structural integrity filter implicitly test for this – predictions will only be made when the conformation from the original structure fits into the new. In practice, the method tends to predict best in situations where ligands are similar in size to that in the original structure and/or conformationally rigid.

### Comparison with protein-chemical interaction databases

There are many well-established protein-chemical interactions lacking a 3D structure in databases including STITCH [10], DrugBank [11], BindingDB [12] and ChEMBL [13]. The nature of the chemicals and proteins contained in these databases differs greatly from those in our set, being mostly dedicated to drugs and mammalian proteins, and including many membrane proteins, thus leading to a potential overlap of at most a few hundreds of drug/target pairs. Nevertheless, 222 of 312 predictions (71%) have a p-value≤0.05 for STITCH, 131 of 185 (71%) for DrugBank, 52 of 71 (73%) for BindingDB, and 573 of 975 (59%) for ChEMBL. The numbers improve considerably, when we adopt a more lenient p-value≤0.2 (86%, 85%, 94%, and 85% predictions, respectively) suggesting that the p-value threshold ≤0.05 might be too stringent and thus miss some true positives. Overall, majority of predicted structures overlapping with these databases are significant, providing further support for our approach. As the number of solved structures grows, we expect the overlap with the potential complexes to grow accordingly. Both known structures and predictions are biased against proteins that are difficult to solve, most notably membrane proteins. This is in sharp contrast with the representation of certain protein classes, such as GPCRs or Ion channels, among the drug targets [14] (Table S2, Figure S1 in Text S1). However, new innovations in the solution of membrane proteins will likely make this disparity diminish over time.

A natural question is whether or not our approach can tell anything about the relative or absolute affinity of protein-chemical interactions predicted. Here we are limited by the fact that the protein databank contains protein-chemical interactions at a broad range of affinities: from millimolar to picomolar, and affinities for all protein-chemical structures are not systematically available. We thus believe that we are predicting essentially whether a crystal structure of a protein/chemical interaction is possible, which means we expect our predictions to have a similar range of affinities. This has some potential impact on our attempts to predict selectivity (see below).

**Figure 3:**
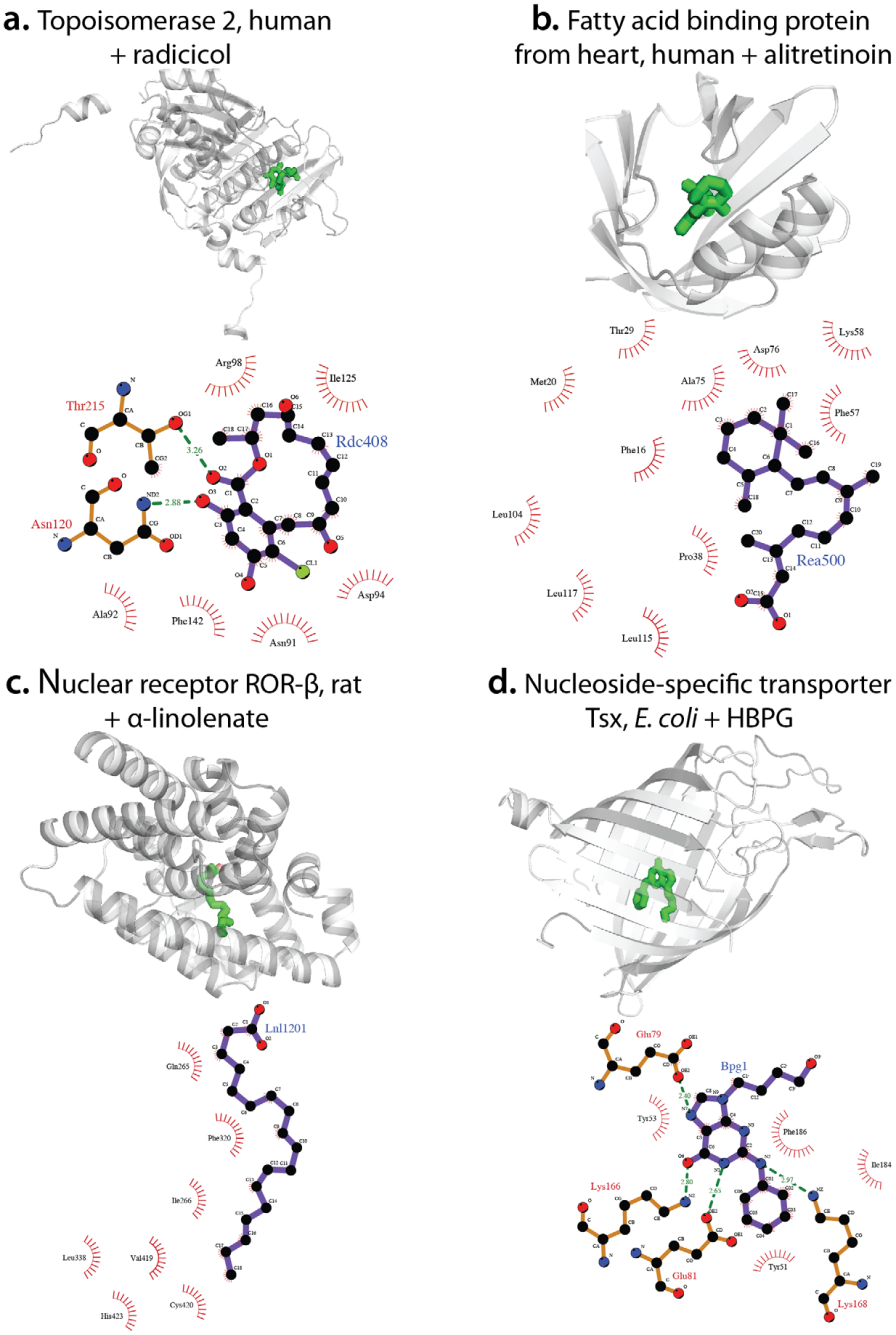
Examples of new predictions with limited literature evidence. (A): the complex of human topoisomerase 2α with its inhibitor radicicol, predicted via complexes of topoisomerase 2α with adenosine (PDB code 1ZXN), yeast chaperone HSP82 with adenosine (PDB code 1AMW) and radicicol (PDB code 1BGQ). (B): the complex of human fatty acid binding protein FABP3 with alitretinoin, built using complexes of FABP3 with stearic acid (PDB code 1HMR), mouse retinoic acid receptor RXRα with stearic acid (PDB code 1DKF) and alitretinoin (PDB code 1XDK). (C): the complex of rat nuclear receptor RORβ with α-linolenate, predicted using complexes of RORβ with stearic acid (PDB code 1K4W), and maize non-specific lipid-transfer protein with stearic acid (PDB code 1FK4) and with α-linolenate (PDB code 1FK6). (D): the complex of *E. coli* channel-forming protein Tsx with anti-viral agent HBPG, predicted via structures of Tsx in complex with thimidine (PDB code 1TLW) and of herpes virus Thimidine kinase with thimidine (PDB code 1P7C) and HBPG (PDB code 1QHI).

Among predictions involving drugs are several established relationships that lack an experimental structure. For example, we predicted complexes between DNA topoisomerase 2 and radicicol [15] (Figure 3A), and between pentoxifyline and human chitotriosidase, a recently established and surprising finding for this and several other methylxanthine drugs [16]. Here again predictions could be made using disparate routes, for instance the prediction of the known interaction [17] between flurbiprofen and aldo-keto reductase C3 (3-alpha-hydroxysteroid dehydrogenase) was made via the non-homologous protein prostaglandin G/H synthase 2 and the very dissimilar chemical indomethacin.

### New targets for known chemicals

There are 19,578 new predicted interactions, including 7806 involving known drugs. Of the total, 4738 were made using intermediate proteins sharing ≥30% identity to the target protein or one in the same Pfam [18] family. These predictions can be considered easier, since the protein sequences can be readily aligned, and the resulting structure could be obtained by conventional modeling techniques. Similarly there were 3,782 predictions made using chemicals that were ≥90% identical to the intermediate, which could also be made by simple chemical similarity searching and superimposition. Lastly, 1,200 novel predictions involved solvents and common buffer components. Ignoring these three cases left 10,668 non-trivial predicted complexes, of which 4,240 involved drugs. We interrogated this list for interactions supported by literature or other evidence, though for the majority the relative obscurity of the chemicals means that no evidence could be found. The full list of predictions is given in Table S1 in Text S1; we discuss several highlights below.

Several predictions are made using what appear to be convergently evolved binding pockets. That is, the proteins sharing the common ligand share no sequence or structural similarity, and we exploit the common ligand (and thus the two binding pockets) to predict a new ligand for one of the proteins. More exactly, there are 112,546 total protein pairs from the potential predictions that share <30% sequence identity and for which a SCOP [19] fold assignment is available, of which 14,931 (13%) are significant. Of these 94,955 (10,883, 11%, significant) pairs do not share any SCOP fold assignment. There are, thus, more predictions made using weakly homologous proteins, but nevertheless convergences still play an important role. This is perhaps not surprising considering the number of compounds, such as ATP analogs, that are known to bind distinct ATP binding folds.

We predict a complex between the heart-specific fatty acid binding protein (FABP3) and alitretinoin, using structures of FABP3 with stearic acid and of mouse RXRα with strearic acid and alitretinoin. There is only indirect evidence in support of this interaction: proteins in the wider superfamily of lipid binding proteins show some ligand promiscuity [20]. However, the structural fit of the alitretinoin into FABP3 is striking (Figure 3B). Elsewhere, we predicted a complex between orphan retinoic acid receptor (ROR) β and α-linolenate made by virtue of a complex between this protein and stearic acid, which also binds to maize non-specific lipid-transfer protein, which in turn binds to linolenate (Figure 3C). The natural ligand of RORβ is not known; stearate was observed in complex fortuitously owing to the expression of the protein in *E. coli* [21]. In contrast to RORα, the expression of RORβ is highly restricted to parts of the brain, the retina, and pineal gland [22]. α-linolenate is an essential fatty acid and in humans is a precursor for eicosapentaenoic acid (EPA) and docosahexaenoic acid (DHA), and deficiencies in dietary α-linolenate result in various problems, including learning [23] or vision [24]. Although these observations could be coincidental, they support the possibility of an interaction between linolenate and RORβ.

Several predictions involve anti-viral compounds in complex with the *E. coli* transporter Tsx, mostly based on structures of herpes virus thymidine kinase with thymidine, which also binds to Tsx. The kinase has been solved in complex with 12 anti-viral compounds, of which eight fit well into the Tsx structure (e.g., HBPG, Figure 3D). The structure of *E. coli* Tsx was proposed [25] to be a possible model for drug transport via the eukaryotic equilibrative nucleoside transporters [26]. Our predictions support this possibility, though obviously additional structures of eukaryotic equivalents in complex with model compounds are needed.

There are also hundreds of predictions involving drugs that apparently lack supporting evidence from the literature. These include the phosphodiesterase inhibitor filaminast binding to 5′-AMP-activated protein kinase, zanamivir binding to mammalian sialoadhesin, and coumarin binding to dipeptidyl peptidase 4 (see Table S1 in Text S1).

### Inhibitor selectivity

Protein-drug selectivity is an important issue, since non-selective drugs can have undesired side-effects. The problem is particularly acute for drugs designed to target one member of a large homologous family of proteins, such as GPCRs or protein kinases. We predicted many protein-kinase inhibitor complexes (12% of confident predictions). They offered an opportunity to test whether our approach could say anything about inhibitor selectivity. There are currently 766 unique human protein kinase-inhibitor crystal structures (284 kinases and 627 inhibitors). We compared our predictions with systematic screens for 127 human kinases and 33 inhibitors. The overlap of kinase/compound pairs in the screens (i.e. whether interacting or not) and the known or predicted structures is low: only 21 complexes overlap with our set of potential structures, and we get significant predictions for six of these (see Supplementary information). Despite the low overlap, we saw a correlation between the tendency for an inhibitor to be predicted to bind many kinases and the tendency to interact with many kinases in the screens, even if the particular kinases differ (see Table S3 in Text S1). For example, known promiscuous inhibitors, such as staurosporine are both predicted and observed to bind dozens of kinases, in contrast to imatinib where despite 70 potential predictions, none are significant. This set also contains numerous predictions of kinase-inhibitor complexes that have not, to our knowledge, been tested, for instance binding of nilotinib to KIT and LCK, or of erlotinib to SRC, HCK or PKR.

Data related to the predictions presented here are available as an online resource at http://pcidb.russelllab.org/.

## Discussion

We have demonstrated that using protein and chemical superimpositions and structural compatibility screens can reproduce known protein-chemical interactions, and suggest many novel relationships. The prospect of using databases or networks of known biomolecular interactions to predict additional relationships is not new (e.g. [3], [5]) though to our knowledge this is the first attempt to use 3D structures in the network context in this way to link disparate chemicals and targets. Like other methods based on large experimentally determined networks, the approach here has the advantage that it will improve in terms of coverage and accuracy as the number of interactions grows. The number of protein-chemical interactions of known 3D structure has been growing exponentially since the late 1980s, and with the increasing number grows the potential to infer new relationships.

**Figure 4:**
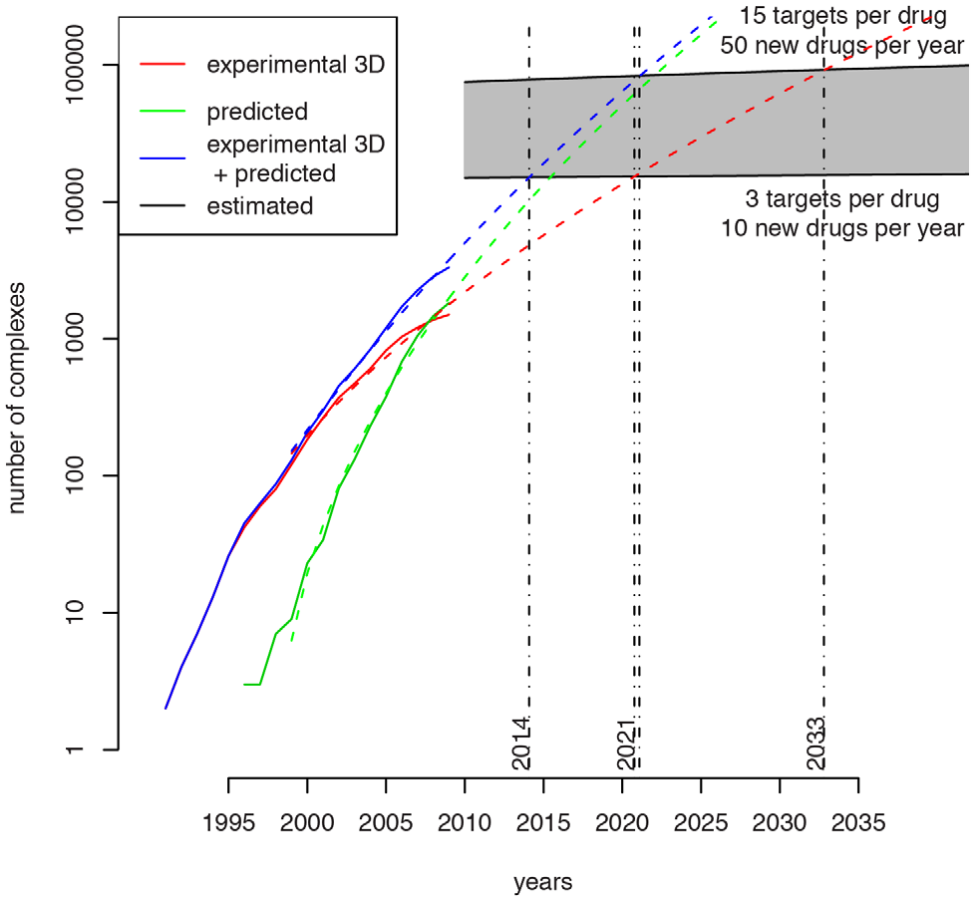
Extrapolation of growth rates for complexes of human proteins with drugs, resolved using experimental methods (red) and amenable to prediction using our method (green) over time. The sum of the two curves is shown in blue. The total number of existing drug-target complexes is shown in gray and estimated given the average number of targets per drug to be between 3 and 15, the number of targets to be fixed at 5000 and the number of drugs to be increasing linearly at the rate of between 10 and 50 per year (see text for details).

The growth in the number of confident predictions is steeper than that for known structures (Figure S2 in Text S1). This raises the question as to when we will we be able to predict most protein-chemical interactions confidently based on available data. For such an estimate, it is simplest to consider a smaller subset of interactions, and for this purpose, we considered the set of human protein-drug interactions. There are currently 4,774 distinct drugs known (in DrugBank [11]), with 14–44 new drugs appearing each year (see http://www.vfa.de/en/statistics/innovation/). Range estimates as to the average number of proteins to which a typical drug will bind can come from known 3D structures (3 proteins per compound), or drug-target databases such as SuperTarget/Matador [27] (4.8) or DrugBank [11] (2.7). As we know these are either conservative or based on missing data, we also considered an upper figure of 15 proteins. We thus estimate between roughly 20,000 and 80,000 protein-chemical interactions within the human system, a number that would be reached by 2022 according to the extrapolation in Figure 4, or perhaps later if to the apparent decrease in the growth rate in the last two years holds. This estimate presumes that protein-chemical structures are similar in terms of the ease with which they are solved; something that is obviously false for membrane proteins that make up more than 40% of drug targets [14]. Thus the estimate is probably over-optimistic, though we anticipate that additional breakthroughs in structural biology will also ultimately make membrane protein – ligand complexes more commonplace.

Regardless of the precise details of this estimate, it is not fanciful to imagine a time when there will be sufficiently determined 3D structures to predict accurately most known protein-chemical interactions using methods like that described here. Twenty years ago the prospect of having structural information for most globular protein domains seemed very distant, but today, thanks to structural genomics and modeling techniques, it is difficult to find proteins for which structural information is unavailable [28]. Predicted protein-chemical structures will have limitations in the same way that modeled individual structures are not as accurate as those experimentally determined, but they will similarly provide a great deal of useful information. As with all estimates of this sort, we suspect that advances in structure determination methods will probably make the time shorter, though it is also likely that the number of known protein-chemical interactions will also increase greatly. It is clear that the continued study of the structural database by approaches like that discussed here will deliver a growing set of novel and highly relevant protein-chemical complexes for use in biomedicine, biotechnology and beyond.

## Methods

### Model construction

Given three complexes, involving two common proteins and two common ligands as detailed in Figure 1, we superimpose the two complexes involving P_1_ using structure superimposition [29]. The resulting transformation produces a superimposition of the two non-identical ligands L_a_ and L_b_. We then use ligand L_a_ to superimpose the complexes it makes with P_1_ and P_2_, producing a superimposition of the non-identical proteins. We combine these two superimpositions by re-centering on the common ligand L_a_, producing a final superimposed complex, involving all proteins and ligands, and including the new complex P_2_:L_b_.

### Scoring system

We considered seven structural criteria to judge the predicted complexes: a) the clash volume; b) the number of ligand-protein contacts; c) the number of potential hydrogen bonds; d) the number of potential Van der Waals contacts; e) the number of un-satsified ligand hydrogen bond donors and acceptors; f) the number of ligand carbon atoms not involved in Van der Waals contacts; and g) the number of potential hydrogen donor and acceptor atoms exposed to the solvent. The raw values were normalized to ranges from 0 to 1: for a specific value of the parameter *ξ_i_* for the model *i*, we calculate the fraction *s_i_* of values *ξ*<*ξ_i_*, where *ξ* represents values of the same parameter in the negative dataset composed of random complexes (see below). For a, e, f and g we take the fraction of *ξ>ξ_i_*, as these terms are detrimental to binding. We sum *s_i_* for all the parameters and obtain a combined score *S_i_* that can range from 0 to 7, greater scores corresponding to better models.

We convert the scores into p-values by considering scores for a negative dataset of 100,000 random structures (see below). For a given model score *S_i_* we calculate *p* as *p* = (#*σ*: *σ*>*S_i_*)/#*σ*, where *σ* are the scores from the negative dataset. Note, that only 24% of real structures, when scored by this scheme, have p≤0.05, which we believe not to be due to crystal packing or non-specific binding of buffer components, as a solvent-free subset, and a subset where promiscuous small molecules (those seen in more than 20 structures) were removed gave similar ratios. Instead, we believe this to reflect the stringency of our approach; p<0.2 clearly separates the positive and negative datasets (see below) but produces many false positives and predicted complexes with poor RMSDs in the benchmark (Figure S3 in Text S1). When limiting the set of negatives to structures where the modeled random ligand has the same number of atoms as the cognate, and/or to modeled ligands having the same number of hydrogen bond donors and acceptors, we see little differences in the score distributions compared to the initial negative set (correlations R = 0.99, p = 0 and R = 0.94, p = 0 respectively).

### Dataset construction

We consider all pairwise complexes extracted from the Protein Data Bank [30] that consists of a protein annotated in Uniprot [31] and a compound annotated in PubChem [32], as of mid-2009. Only contacting pairs were considered, i.e. at least two heavy atoms, one from the proteins and one from the compound, were required to be closer than 5 Å. There were thus 45,455 protein-small-molecule complexes of known structure as the positive dataset. If a structure included more than one instance of either protein chain or chemical, we considered each chain-small molecule pair as a separate entry. We constructed a negative dataset of 100,000 complexes by randomly selecting two complexes from the positive dataset, and substituting their ligands according to their geometrical centers (without any rotation to optimize binding). We then removed all complexes involving either protein-ligand clashes, or those lacking protein-ligand contacts. This set represents random fits of small molecules into protein pockets.

For the benchmark dataset and the dataset of potential complexes, we selected all combinations of three complexes as shown in Figure 1. We excluded instances where the proteins were ≥80% identical or the ligands had a Tanimoto score of 1. This set includes all possible predictions (even those that are wrong) made using our approach. For 907,827 potential structures, we reconstructed 194,317 3D predicted complexes, of which 20,067 scored with a p-value≤0.05. Within this set, we defined the benchmark dataset as those 376 significantly predicted complexes for which a structure for the predicted complex was already of known structure. For 703 of those we reconstructed a 3D structure using superimposition. We applied our scoring system to these complexes and excluded all predictions with a p-value>0.05, thus yielding 376 significant predictions. We also excluded predictions where the number of heavy atoms in L_a_ and L_b_ differs more than two fold.

## Supporting Information

Text S1 Includes a more detailed comparison of the predictions with BindingDB [12] and STITCH [10], including various affinity cutoffs; rationale for the cutoff choice; analysis of kinase-inhibitor complexes; and four supplementary figure and three supplementary tables.
(PDF)
