# Supplementary material for: Combinations of Protein-Chemical Complex Structures Reveal New Targets for Established Drugs
Source: PLoS Comput Biol. 2011 May 5;7(5):e1002043. doi: 10.1371/journal.pcbi.1002043 (PMC3088657; doi:10.1371/journal.pcbi.1002043)
Supplement: Text S1 — Includes a more detailed comparison of the predictions with BindingDB [12] and STITCH [10], including various affinity cutoffs; rationale for the cutoff choice; analysis of kinase-inhibitor complexes; and four supplementary figure and three supplementary tables. (PDF) [file pcbi.1002043.s001.pdf]

Comparison with other sources of protein-chemical interactions

For an additional test of this approach, we compared our set of candidate complexes to interactions with measured affinities from BindingDB[7], that includes 1277 protein-chemical pairs, 323 with high (<50 nM) affinity. 71 of those were in our candidate set, including 27 high-affinity complexes. For 34 complexes we obtain confident predictions. For high-affinity complexes the corresponding numbers are 20 and 10. When considering all predictions, there is no correlation between measured affinity and p-value (data not shown); this is not surprising as all affinities are considered together when constructing our dataset (i.e. all interactions of known structure regardless of affinity, and the range of affinities ranges from high micro to picomolar). However, if we restrict the set of potential complexes to those where all the complexes used to make the predictions have a measured high affinity (54 cases where all three intermediate complexes have IC50 or Ki <50 nM and 172, <1000nM), the affinity of the resulting complex is no more than an order of magnitude lower than the original values (data not shown). Thus high-affinity known complexes tend to predict other high-affinity complexes.

We tested our predictions against the STITCH database that includes 1,322,502 high-confidence protein-chemical interactions supported by experimental or literature evidence[8] that involve 305,953 proteins and 6589 distinct chemicals. Of those, 1879 proteins and 655 chemicals appear in resolved 3D structures, and 341 protein-chemical pairs are included in our candidate dataset, and 173 of them (51%) have a p-value <0.05.

Rationale for cutoff choice

As can be seen from Figure S3, the cutoff that best separates the positive and the negative datasets corresponds to p=0.2. However, we chose to apply a more stringent cutoff of p=0.05 to avoid high false positive rate. Indeed, for p=0.2, sensitivity (number of real complexes from the positive dataset that score better than this threshold) is 43%, while false discovery rate (number of random complexes from the negative dataset that score better than the threshold) is, by definition, 20%. For p=0.05, sensitivity is 23% (drops roughly twice), while false discovery rate is 5% (drops 4 times).

Kinase-inhibitor complexes

In our candidate dataset, we found 15 potential kinase-inhibitor complexes, for which there is experimental evidence of their existence, but no 3D structure have been determined. They are listed in the table below together with corresponding p-values and minimal distance of the modelled inhibitor to the protein. For 7 of 15 complexes, p-value is less than 0.05 and minimal distance is greater that 2 Å, indicating structurally very sound prediction. For additional 4, minimal distance is less that 2 Å, which means that some adjustment of the side chains may be needed to accommodate the inhibitor. For one complex, p-value is 0.11, and minimal distance is less that 2 Å, and for three, we cannot construct any plausible structure.

Kinase	Inhibitor	Reference	P-value	Minimal distance (Å)
ROCK2	H89		0	2.2
KPCT	Ruboxistaurosporin		0	1.3
CSK21	LY294002		–	–
CSK21	Quercitin		–	–
AAKG1	Quercitin		0	0.9
PIM1	Gefitinib		0.11	0.7
LCK	Indirubine-3'-monoxime		0.02	1.4
CDK2	Purvalanol A		0	1.9
SRC	PD173955		0.04	2.6
SGK1	Indirubine-3'-monoxime		0	2.2
CSK	PP2		0	2.8
CDK2	Indirubine-3'-monoxime		0	2.6
ERBB2	Lapatinib		–	–
MAPK2	Fasudil		0.02	2.7
EGFR	Staurosporine		0	2.3

Our method can assist in understanding the structural basis of inhibitor selectivity. For example, for fasudil, a Rho-associated kinase inhibitor (and its analog H-1152P), which are known and predicted by our method to have a wide spectrum of kinase inihibition, an explanation comes at least partly from the 3D structure of the inhibitor. Although quite

dissimilar to ATP in 2D representations, Fasudil occupies a very similar volume (Fig. S4A). In contrast we predict flavopiridol to have a smaller spectrum, which can possibly be explained by the extra aromatic ring extruding from the ATP volume (Fig. S4B).

Supplementary figure legends

Figure S1.

Fraction of drugs (as defined in DrugBank) in different target sets.

Figure S2.

Growth of experimentally identified (red) and confidently predicted (green) 3D structures of proteins-chemical complexes over last 30 years. The number of experimentally identified complexes was calculated as the total number of complexes, for which the PDB entry dates not later that the corresponding year, the number of predicted complexes as those, for which all three intermediate PDB entries date not later than the corresponding year.

Figure S3.

Score distributions for the positive (red) and the negative (black) datasets. Vertical lines denote scores corresponding to cutoffs $p<0.05$ and $p<0.2$.

Figure S4.

Structural comparison of predicted conformations of selected kinase inhibitors with ATP. (A): A promiscuous inhibitor fasudil adopts a similar conformation. Superimposition of ATP co-crystallized with Mevalonate kinase from rat (PDB 1KVK) with fasudil modeled into the structure of Mevalonate kinase using complexes of bovine cAMP-dependent protein kinase with ATP (PDB 1Q24) and fasudil (PDB 1Q8W). (B): A selective inhibitor flavopiridol adopts a distinct conformation. Superimposition of ATP co-crystallized with bovine rhodopsin kinase (PDB code 3C4W) with flavopiridol modeled into the structure of bovine rhodopsin kinase using complexes of human cell division protein kinase 9 with ATP (PDB code 3BLQ) and flavopiridol (PDB code 3BLR).

Figure S1

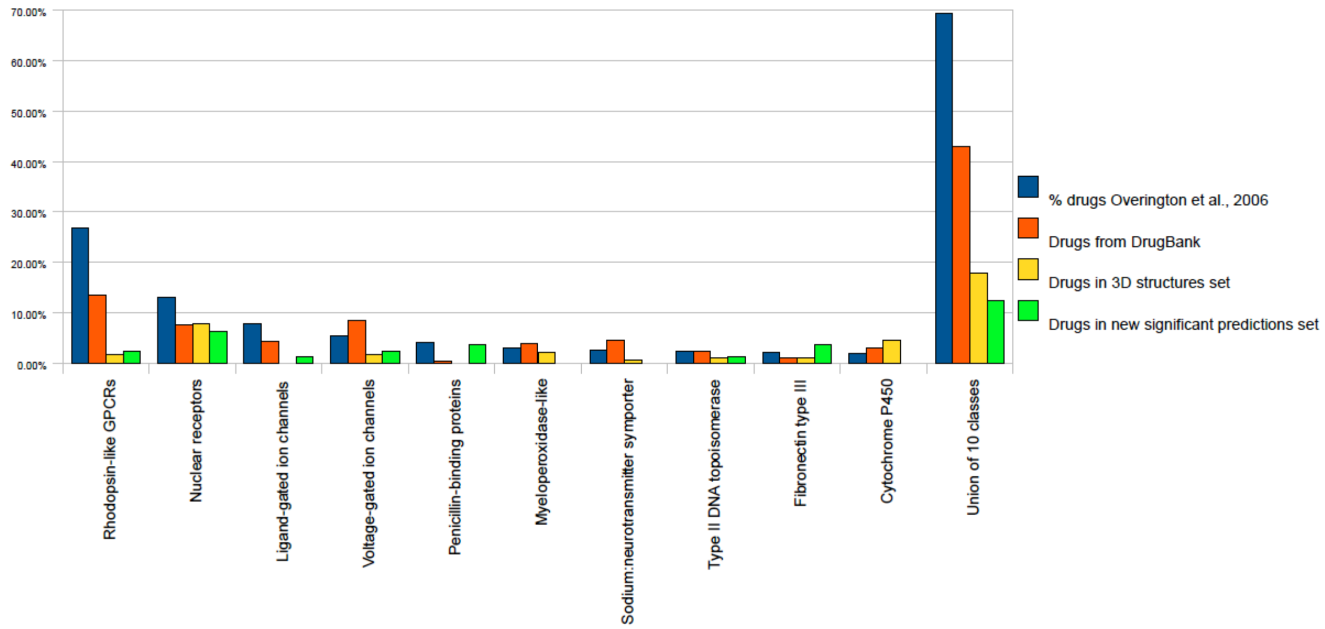
70.00%
60.00%
50.00%
40.00%
30.00%
20.00%
10.00%
0.00%
Rhodopsin-like GPCRs
Nuclear receptors
Ligand-gated ion channels
Voltage-gated ion channels
Penicillin-binding proteins
Myeloperoxidase-like
Sodium:neurotransmitter symporter
Type II DNA topoisomerase
Fibronectin type III
Cytochrome P450
Union of 10 classes
% drugs Overington et al., 2006
Drugs from DrugBank
Drugs in 3D structures set
Drugs in new significant predictions set

Figure S2

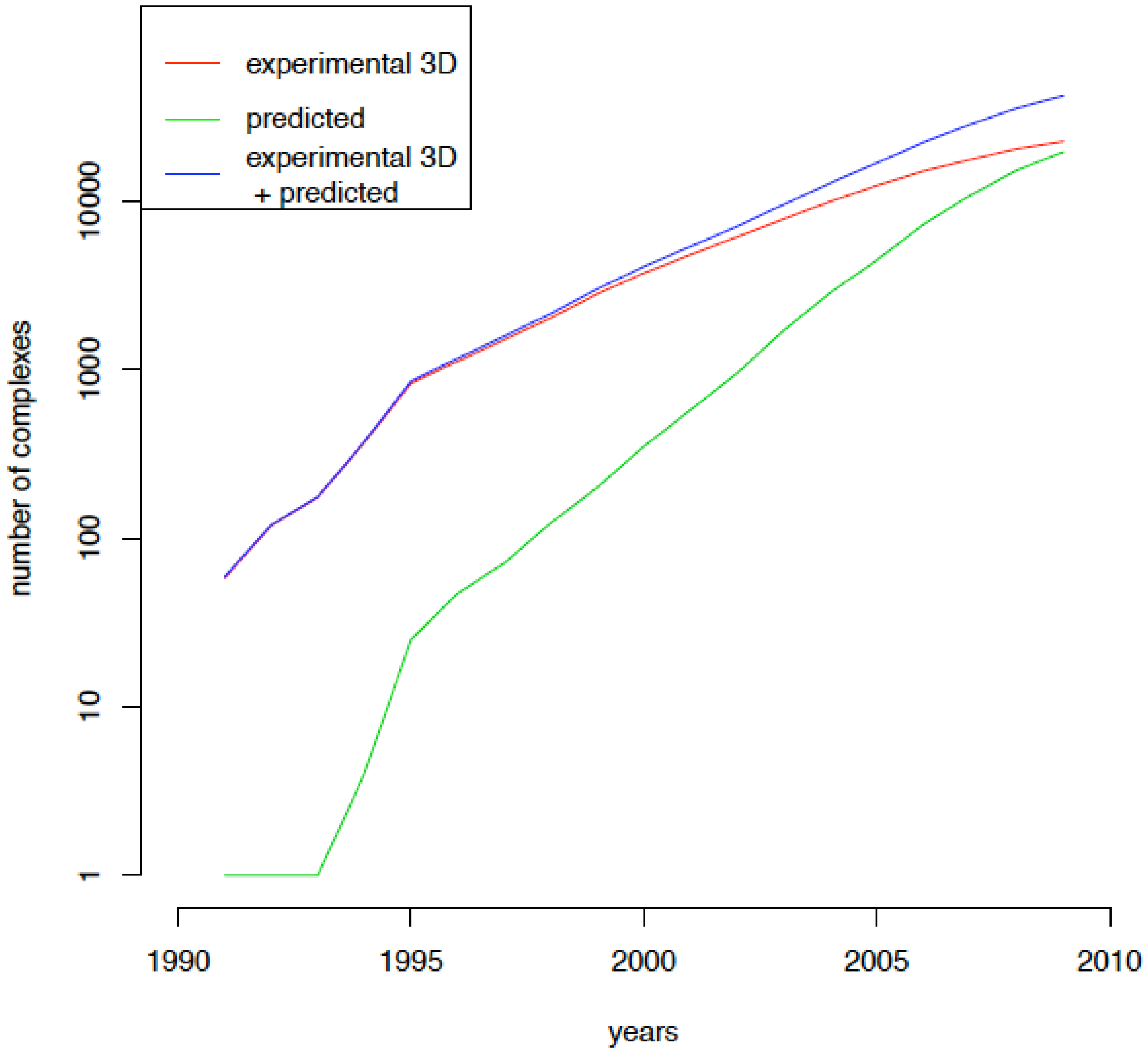
experimental 3D
predicted
experimental 3D
+ predicted
number of complexes
1
10
100
1000
10000
1990
1995
2000
2005
2010
years

Figure S3

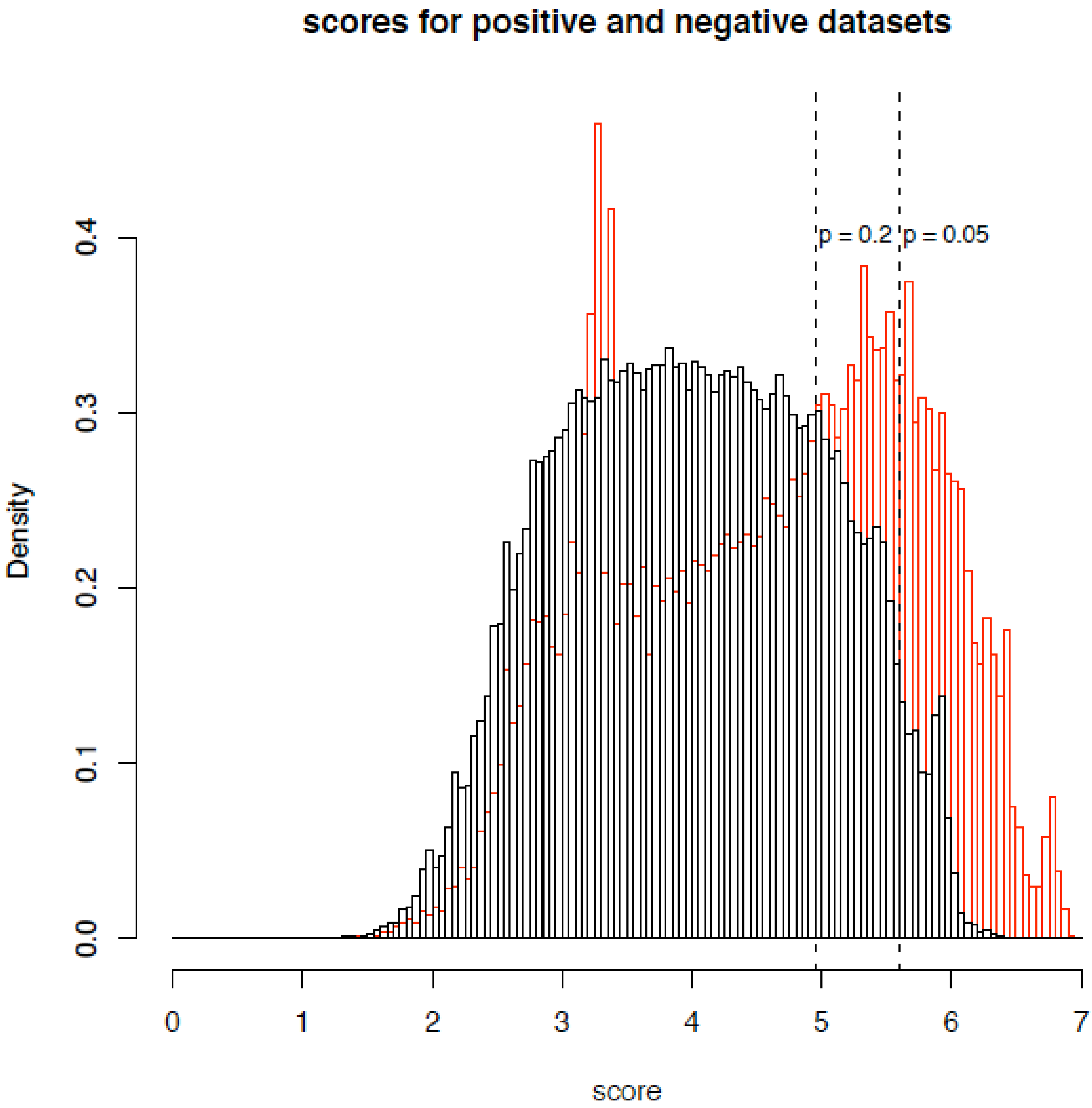
scores for positive and negative datasets
p = 0.2
p = 0.05
Density
score
0.0
0.1
0.2
0.3
0.4
0
1
2
3
4
5
6
7

Figure S4

A.

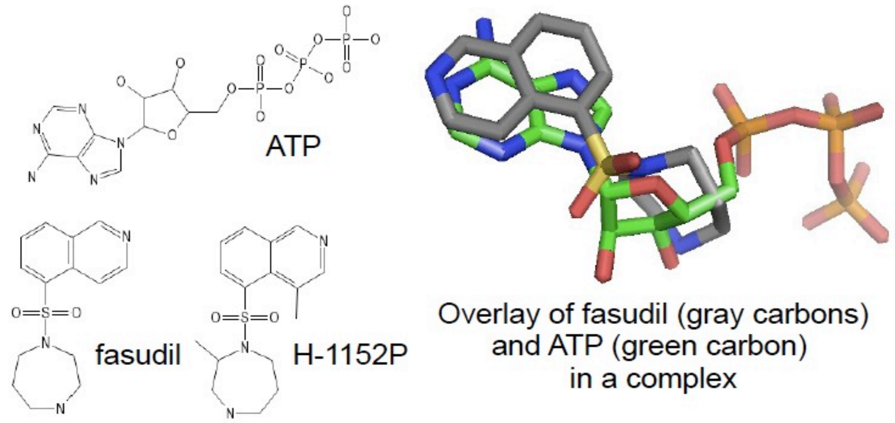

ATP
fasudil
H-1152P
Overlay of fasudil (gray carbons)
and ATP (green carbon)
in a complex

B.

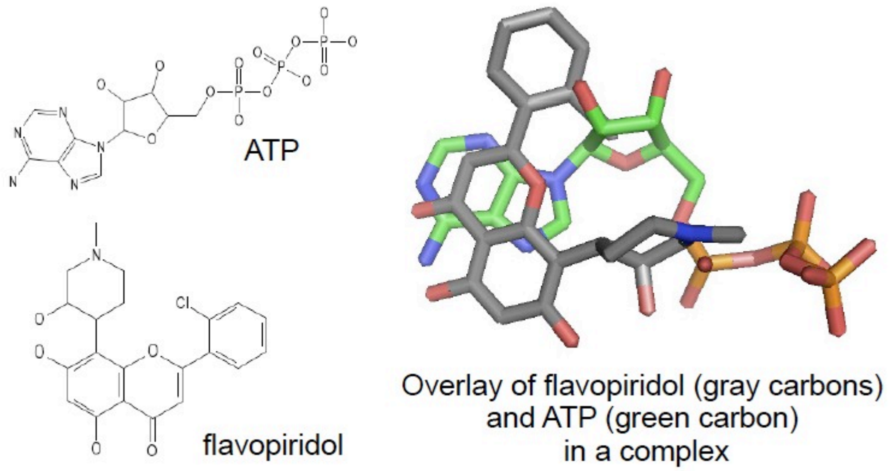

ATP
flavopiridol
Overlay of flavopiridol (gray carbons)
and ATP (green carbon)
in a complex

Table S1. Full list of novel predictions.

Prediction		Intermediates							
Protein2	Ligand2	Protein1	Ligand1	Structure1	Structure2	Structure3	protein identity	chemicals Tanimoto score	Score; minimal distance to ligand
1A02_HUMAN F	439353 be	O96048_LUMTE	8172 Trig	2DRY	2PYE	2DS0			Sc=5.63826, min distance = 2.494918
1A1D_PYRHO F	445005 D-	DGDA_BURCE Fu	4324 PLP-	1D7R	1J0B	1D7S		0.89	Sc=6.45422, min distance = 2.350048
1A1D_PYRHO F	445008 DE	DGDA_BURCE Fu	4324 PLP-	1D7R	1J0B	1D7U		0.88	Sc=6.45534, min distance = 2.375275
1B15_HUMAN F	447364 4-	Q0VDX6_MOUSE	8200 TETR	1Q0X	3C9N	1NGP	28.95		Sc=5.77711, min distance = 2.379363
3HAO_RALME F	448825 CI	PHZF_PSEFL Fu	86 3-Hydr	1U1W	1YFY	1U1X			Sc=5.77279, min distance = 2.639662
3HIDH_HUMAN	439153 Di	GALE_ECOLI Fu	5893 nadi	1UDC	2I9P	1UDB		0.79	Sc=6.75687, min distance = 1.904539
3HIDH_HUMAN	446050 CI	G3P_HUMAN Ful	5893 nadi	1U8F	2I9P	3GPD		0.95	Sc=6.74527, min distance = 1.044449
5NT3_MOUSE F	5962 L-ly	RBCMT_PEA Ful	23831 HEF	1MLV	2G0A	2H2E			Sc=5.61263, min distance = 1.163558
5NT3_MOUSE F	854023 Ep	Q8WSF8_APLCA	23831 HEF	2BR7	2G0A	2BYQ			Sc=5.70454, min distance = 1.885160
5NTC_HUMAN F	8975 2-Fl	SAHH_MYCTU Fu	60961 ade	3CE6	2JC9	2ZJ0		0.99	Sc=6.15835, min distance = 2.387620
6PGD1_YEAST	16750041	PYRB_ECOLI Fu	311 citri	1R0B	2P4Q	2IPO			Sc=6.09896, min distance = 0.891414
6PGD1_YEAST	445905 1g	AROQ_HELPY Fu	311 citri	2C4V	2P4Q	2C57			Sc=5.77923, min distance = 2.049610
6PGD1_YEAST	448573 FI	CASP7_HUMAN F	311 citri	2QL9	2P4Q	1SHL			Sc=5.78706, min distance = 2.125534
6PGD1_YEAST	51 2-Oxop	SERA_ECOLI Fu	311 citri	2P9E	2P4Q	1YBA		0.76	Sc=5.83812, min distance = 1.309822
6PGD1_YEAST	6102712 C	PYRB_ECOLI Fu	311 citri	1R0B	2P4Q	2AIR			Sc=5.83651, min distance = 1.234648
6PGD_LACLM F	440641 1m	G6PI_RABIT Fu	91493 1pg	1DQR	2IYO	1HOX			Sc=5.85675, min distance = 2.161876
6PGD_SHEEP F	15942680	6PGD_LACLM Fu	5886 NADF	2IZ0	1PGO	2IZ1	56.81		Sc=6.2135, min distance = 2.1006070
6PGL_THEMA F	11957399	PYRB_ECOLI Fu	311 citri	1R0B	1VL1	2H3E			Sc=5.96466, min distance = 1.028965
6PGL_THEMA F	16750041	PYRB_ECOLI Fu	311 citri	1R0B	1VL1	2IPO			Sc=5.9564, min distance = 1.0220616

6PGL_THEMA F	445607 AT	ACON_BOVIN Fu	311 citri	1C96	1VL1	1FGH			Sc=5.78972, min distance = 2.086955
6PGL_THEMA F	445905 1g	AROQ_HELPY Fu	311 citri	2C4V	1VL1	2C57			Sc=5.7407, min distance = 2.7428909
6PGL_THEMA F	447376 2-	ACON_BOVIN Fu	311 citri	1C96	1VL1	1NIS			Sc=5.67516, min distance = 2.521406
6PGL_THEMA F	447537 1c	SRC_HUMAN Ful	311 citri	1O4L	1VL1	1O4R			Sc=5.84158, min distance = 2.291082
6PGL_THEMA F	449604 NI	ACON_BOVIN Fu	311 citri	1C96	1VL1	8ACN			Sc=5.68885, min distance = 2.182604
6PGL_THEMA F	51 2-Oxop	SERA_ECOLI Fu	311 citri	2P9E	1VL1	1YBA		0.76	Sc=5.60249, min distance = 1.426700
A0EKU1_9FLAV	5326531 C	CHOMT_MEDSA F	34756 Acy	1FPQ	2OXT	1FP1		0.92	Sc=6.5315, min distance = 1.922203
A0EKU1_9FLAV	6852187 2	Q70GK9_STRCT	34756 Acy	1RQP	2OXT	2CBX		0.84	Sc=6.09466, min distance = 1.965190
A1AT_HUMAN F	439353 be	ANT3_HUMAN Fu	311 citri	2ZNH	3CWM	1E05			Sc=5.78164, min distance = 2.191806
A1AT_HUMAN F	439459 Hc	NIFD_KLEPN Fu	311 citri	1H1L	3CWM	1QGU		0.92	Sc=6.12521, min distance = 2.052945
A1AT_HUMAN F	439459 Hc	NIFK_KLEPN Fu	311 citri	1H1L	3CWM	1QGU		0.92	Sc=6.12133, min distance = 2.019036
A1AT_HUMAN F	51 2-Oxop	SERA_ECOLI Fu	311 citri	2P9E	3CWM	1YBA		0.76	Sc=5.78932, min distance = 2.161804
A3F9D6_CAPHI	11957353	HBA_HORSE Ful	444098 HE	2D5X	2EFB	1IWH		0.83	Sc=6.79842, min distance = 1.988848
A3F9D6_CAPHI	11957363	HBB_HUMAN Ful	444098 HE	1J40	2EFB	1RQA		0.77	Sc=6.80662, min distance = 1.843561
A3F9D6_CAPHI	11957385	MYG_PHYCA Ful	444098 HE	1A6M	2EFB	2CMM			Sc=6.33305, min distance = 2.059932
A3F9D6_CAPHI	16741061	MYG_PHYCA Ful	444098 HE	1A6M	2EFB	1MBC		0.83	Sc=6.48916, min distance = 2.020704
A3F9D6_CAPHI	16741062	MYG_PHYCA Ful	444098 HE	1A6M	2EFB	1MBN		0.84	Sc=6.44563, min distance = 2.029637
A3F9D6_CAPHI	24883662	MYG_HORSE Ful	444098 HE	2FRF	2EFB	1YMC		0.82	Sc=6.76289, min distance = 2.056286
A3F9D6_CAPHI	4369122 C	GLB1_LUCPE Fu	444098 HE	1FLP	2EFB	1B0B		0.96	Sc=6.79214, min distance = 2.139712
A3F9D6_CAPHI	444668 CI	MYG_PHYCA Ful	444098 HE	1A6M	2EFB	1BVD		0.8	Sc=6.34186, min distance = 2.030057
A3F9D6_CAPHI	446406 CZ	MYG_PHYCA Ful	444098 HE	1A6M	2EFB	1J3F			Sc=6.40566, min distance = 1.883639
A4H1Z0_9PICO	164628 de	NDKC_DICDI Fu	122108 Ri	1MN9	2E9R	1NDC			Sc=5.80138, min distance = 2.498192
A4H1Z0_9PICO	446090 1h	NDKC_DICDI Fu	122108 Ri	1MN9	2E9R	1HIY			Sc=5.87489, min distance = 1.910593
A4H1Z0_9PICO	448505 AD	NDKC_DICDI Fu	122108 Ri	1MN9	2E9R	1S5Z			Sc=6.34804, min distance = 2.101825
A4H1Z0_9PICO	6022 Aden	NDKC_DICDI Fu	122108 Ri	1MN9	2E9R	1KDN			Sc=6.33851, min distance = 1.863258
A4H1Z0_9PICO	6030 Uric	PYRH_SULSO Fu	6133 urid	2J4L	2E9Z	2J4J		0.99	Sc=6.15398, min distance = 1.592652
A5JUY8_BUBBU	11957353	HBA_HORSE Ful	444098 HE	2D5X	2Z5Z	1IWH		0.83	Sc=6.79378, min distance = 0
A5JUY8_BUBBU	11957363	HBB_HUMAN Ful	444098 HE	1J40	2Z5Z	1RQA		0.77	Sc=6.79973, min distance = 0
A5K709_PLAVI	101543 3'	RNMC_MOMCH Fu	6804 guan	1J1F	2QOR	1UCC			Sc=6.26983, min distance = 2.046831
A5K709_PLAVI	445463 B-	TGM3_HUMAN Fu	6804 guan	1SGX	2QOR	1L9N			Sc=6.23866, min distance = 1.759465
A5K709_PLAVI	65059 Dec	RNAS1_BOVIN F	6804 guan	1RNC	2QOR	2QCA		0.97	Sc=6.35774, min distance = 2.023965
A5K709_PLAVI	8582 Inos	HPRT_ECOLI Fu	6804 guan	1G9T	2QOR	1G9S		0.99	Sc=6.38508, min distance = 2.124591
A5KBL5_PLAVI	6804 guar	PDE10_HUMAN F	6083 aden	2OUN	2QGA	2OUQ		0.8	Sc=5.83473, min distance = 0
A5KBL5_PLAVI	8582 Inos	PURA1_MOUSE F	6083 aden	1MF0	2QGA	1IWE		0.79	Sc=6.31605, min distance = 1.895384
A5KE01_PLAVI	6021 inos	DEOD_ECOLI Fu	60961 ade	1PK7	2PGF	1PR0		0.79	Sc=6.32735, min distance = 1.607873
A5KE01_PLAVI	8975 2-Fl	SAHH_MYCTU Fu	60961 ade	3CE6	2PGF	2ZJ0		0.99	Sc=6.33761, min distance = 2.167787
A5KE01_PLAVI	97184 6-M	DEOD_ECOLI Fu	60961 ade	1PK7	2PGF	1OVG		0.9	Sc=6.26373, min distance = 1.980169

A5KE56_PLAVI	150856 5-	PYR5_HUMAN Fu	6030 Urid	2V30	2FFC	2QCF		0.94	Sc=6.23512, min distance = 2.50723
A5KE56_PLAVI	150973 5-	PYR5_HUMAN Fu	6030 Urid	2V30	2FFC	2QCG		0.91	Sc=6.24285, min distance = 2.40453
A5KE56_PLAVI	160617 Or	PYR5_HUMAN Fu	6030 Urid	2V30	2FFC	2QCL		0.86	Sc=6.01392, min distance = 2.38583
A5KE56_PLAVI	17749721	PYR5_HUMAN Fu	6030 Urid	2V30	2FFC	2QCM		0.88	Sc=6.29572, min distance = 2.52565
A5KE56_PLAVI	445132 1x	PYR5_HUMAN Fu	6030 Urid	2V30	2FFC	3BGG		0.81	Sc=5.91677, min distance = 2.36110
A5KE56_PLAVI	449489 Ic	PYR5_HUMAN Fu	6030 Urid	2V30	2FFC	2QCH		0.9	Sc=6.1976, min distance = 2.240243
A5KE56_PLAVI	65063 dUM	PYR5_HUMAN Fu	6030 Urid	2V30	2FFC	2QCH		0.97	Sc=6.17647, min distance = 2.24251
A5KE56_PLAVI	6804 guar	RNMC_MOMCH Fu	6030 Urid	1UCD	2FFC	1J1F			Sc=6.35774, min distance = 2.06389
A7A026_YEAS7	11957353	HBA_HORSE Full	444124 HE	1Y8I	2IA8	1IWH		0.87	Sc=6.71026, min distance = 2.07317
A7A026_YEAS7	11957363	HBA_HUMAN Full	444124 HE	1NQP	2IA8	1RQA		0.81	Sc=6.72131, min distance = 2.43464
A7A026_YEAS7	11957364	HMOX1_HUMAN Fu	444124 HE	1OZW	2IA8	1S13		0.86	Sc=6.66447, min distance = 1.69920
A7A026_YEAS7	11957370	HMOX1_HUMAN Fu	444124 HE	1OZW	2IA8	1TWN			Sc=6.51673, min distance = 2.19585
A7A026_YEAS7	11957385	MYG_PHYCA Full	444124 HE	1U7R	2IA8	2CMM			Sc=6.10071, min distance = 2.26902
A7A026_YEAS7	4369001 C	HBA_HUMAN Full	444124 HE	1NQP	2IA8	4HHB		0.8	Sc=6.76325, min distance = 2.05540
A7A026_YEAS7	444522 HE	BFR_ECOLI Full	444124 HE	1BFR	2IA8	1BCF		0.99	Sc=6.7724, min distance = 2.197215
A7A026_YEAS7	444522 HE	CP2C9_HUMAN Fu	444124 HE	1OG2	2IA8	1R9O		0.99	Sc=6.77274, min distance = 2.02426
A7A026_YEAS7	444522 HE	HBA_HORSE Full	444124 HE	1Y8I	2IA8	1Y8K		0.99	Sc=6.71505, min distance = 2.13042
A7A026_YEAS7	444522 HE	HBA_PAGBE Full	444124 HE	1S5X	2IA8	1PBX		0.99	Sc=6.71214, min distance = 2.21495
AAC2_MYCTU Fu	449233 CI	O53831_MYCTU I	87642 coe	1P0H	1M4I	2C27			Sc=6.04652, min distance = 2.43333
AADAT_HUMAN I	3744 Indo	AAT_ECOLI Full	1053 pyri	2Q7W	2R2N	1AHF			Sc=5.89782, min distance = 1.37048
AADAT_HUMAN I	444286 N-	AAT_ECOLI Full	1053 pyri	2Q7W	2R2N	1ASL		0.9	Sc=6.00106, min distance = 2.39568
AAKG1_HUMAN I	11963492	PUR9_CHICK Fu	65110 AIC	1M9N	2UV5	2B1I		0.93	Sc=6.17907, min distance = 2.00606
AAKG1_HUMAN I	24180721	PIM1_HUMAN Fu	6083 aden	1YXU	2UV4	3C4E			Sc=6.0102, min distance = 1.908056
AAKG1_HUMAN I	6804 guar	PDE10_HUMAN Fu	6083 aden	2OUN	2UV4	2OUQ		0.8	Sc=5.87391, min distance = 0
AAKG1_HUMAN I	8582 Inos	PURA1_MOUSE Fu	6083 aden	1MF0	2UV4	1IWE		0.79	Sc=6.37563, min distance = 1.57745
AAKG1_RAT Fu	101609 Ur	Q9SSV1_NICGU I	6083 aden	1VD1	2V8Q	1VD3			Sc=6.21164, min distance = 1.88056
AAKG1_RAT Fu	16122632	KAPCA_BOVIN Fu	5957 Aden	1Q24	2V92	2UVZ			Sc=6.38199, min distance = 1.15929
AAKG1_RAT Fu	16122635	KAPCA_BOVIN Fu	5957 Aden	1Q24	2V92	2UW8			Sc=5.65282, min distance = 1.17414
AAKG1_RAT Fu	166760 Fa	PURP_METJA Fu	6083 aden	2R7M	2V8Q	2R7N		0.76	Sc=5.90433, min distance = 2.27988
AAKG1_RAT Fu	3758 IBMX	PDE4D_HUMAN Fu	6083 aden	1TB7	2V8Q	1ZKN			Sc=6.05565, min distance = 1.40158
AAKG1_RAT Fu	440317 AT	SRC_CHICK Full	6083 aden	3DQX	2V8Q	3DQW		0.97	Sc=6.49541, min distance = 0.64586
AAKG1_RAT Fu	444564 AD	BIOD_ECOLI Fu	5957 Aden	1A82	2V92	1BS1		0.91	Sc=6.4262, min distance = 1.642586
AAKG1_RAT Fu	444852 CI	ATPB_BOVIN Fu	5957 Aden	2V7Q	2V92	1COW		0.91	Sc=6.51016, min distance = 1.46343
AAKG1_RAT Fu	445708 AM	NADE_ECOLI Fu	6083 aden	1WXI	2V8Q	1WXE		0.91	Sc=6.38128, min distance = 1.06007
AAKG1_RAT Fu	445736 5'	SYS_PYRHO Full	6083 aden	2ZR2	2V8Q	2DQ0		0.86	Sc=6.48577, min distance = 1.33367
AAKG1_RAT Fu	445736 5'	SYS_THET2 Full	6083 aden	1SES	2V8Q	1SET		0.86	Sc=6.48507, min distance = 1.07652
AAKG1_RAT Fu	5327121 I	PIM1_HUMAN Fu	6083 aden	1YXU	2V8Q	2C3I			Sc=6.33065, min distance = 0.85680

AAKG1_RAT Fu	6022 Aden	ARSA1_ECOLX Fu	5957 Aden	1II0	2V92	1IHU		0.99	Sc=6.42539, min distance = 1.82252
AAKG1_RAT Fu	6022 Aden	BIOD_ECOLI Fu	5957 Aden	1A82	2V92	1DAD		0.99	Sc=6.41927, min distance = 1.61983
AAKG1_RAT Fu	6022 Aden	HSP7F_YEAST Fu	5957 Aden	3D2F	2V92	3C7N		0.99	Sc=6.43155, min distance = 2.03872
AAKG1_RAT Fu	6022 Aden	MUTS_ECOLI Fu	5957 Aden	1W7A	2V92	1OH7		0.99	Sc=6.42759, min distance = 1.68023
AAKG1_RAT Fu	6022 Aden	Y059_METJA Fu	5957 Aden	2J9C	2V92	2J9D		0.99	Sc=6.4389, min distance = 1.422386
AAKG1_RAT Fu	6076 Cycl	PDE10_HUMAN Fu	6083 aden	2OUN	2V8Q	2OUR		0.99	Sc=6.27193, min distance = 2.230920
AAKG1_RAT Fu	611002 Op	PIM1_HUMAN Fu	6083 aden	1YXU	2V8Q	1YXX			Sc=6.1891, min distance = 1.416367
AAKG1_RAT Fu	65110 AIC	PURP_METJA Fu	6083 aden	2R7M	2V8Q	2R7K		0.79	Sc=6.27987, min distance = 2.15222
AAKG1_RAT Fu	65110 AIC	YL28_SCHPO Fu	6083 aden	2OOX	2V8Q	2QRE		0.79	Sc=6.28503, min distance = 1.88764
AAKG1_RAT Fu	656965 1y	PDE4D_HUMAN Fu	6083 aden	1TB7	2V8Q	1Y2D			Sc=6.03514, min distance = 1.48467
AAKG1_RAT Fu	656966 1y	PDE4D_HUMAN Fu	6083 aden	1TB7	2V8Q	1Y2E			Sc=6.0852, min distance = 1.732791
AAKG1_RAT Fu	656969 1y	PDE4B_HUMAN Fu	6083 aden	1ROR	2V8Q	1Y2J			Sc=6.20316, min distance = 1.76714
AAKG1_RAT Fu	656969 1y	PDE4D_HUMAN Fu	6083 aden	1TB7	2V8Q	1Y2K			Sc=6.21358, min distance = 1.83525
AAKG1_RAT Fu	657135 Op	PIM1_HUMAN Fu	6083 aden	1YXU	2V8Q	1YXV			Sc=5.86976, min distance = 1.83468
AAKG1_RAT Fu	6804 guar	Q9SSV1_NICGU	6083 aden	1VD1	2V8Q	1VCZ		0.8	Sc=5.82848, min distance = 2.06508
AAKG1_RAT Fu	8582 Inos	PYGM_RABIT Fu	6083 aden	8GPB	2V8Q	2QN7		0.79	Sc=6.37713, min distance = 2.23071
AAKG1_RAT Fu	91532 AMP	NIFH1_AZOVI Fu	5957 Aden	2C8V	2V92	2AFK		0.99	Sc=6.51596, min distance = 1.92170
AAKG1_RAT Fu	9578243 F	PDE4B_HUMAN Fu	6083 aden	1ROR	2V8Q	1XLZ			Sc=6.17738, min distance = 1.28435
AATC_PIG Ful	1053 pyri	AAT_ECOLI Ful	444286 N-	1ASL	1AJS	2Q7W		0.9	Sc=6.14226, min distance = 2.04449
AATM_CHICK Fu	3744 Indo	AAT_ECOLI Ful	444286 N-	1ASL	1AMA	1AHF	41.14		Sc=5.93639, min distance = 2.07264
AATM_CHICK Fu	3744 Indo	AAT_ECOLI Ful	444292 PI	1ARG	1AKB	1AHF	41.14		Sc=5.93112, min distance = 2.09010
AATM_CHICK Fu	4369488 E	AAT_ECOLI Ful	444292 PI	1ARG	1AKB	1X29	41.14	0.94	Sc=6.07375, min distance = 2.30038
AATM_CHICK Fu	4369489 D	AAT_ECOLI Ful	444292 PI	1ARG	1AKB	1X2A	41.14	0.96	Sc=5.75043, min distance = 2.19670
AAT_ECOLI Fu	446383 PY	AATM_CHICK Fu	444286 N-	1AMA	1ASL	1MAQ	41.14	0.94	Sc=5.73951, min distance = 2.17342
AAT_ECOLI Fu	447109 KE	AATM_CHICK Fu	444293 PI	1AKC	1X28	1MAP	41.14	0.96	Sc=5.70556, min distance = 2.40960
AAT_ECOLI Fu	845 histi	HIS8_ECOLI Fu	1053 pyri	1FG7	2Q7W	1GEX			Sc=5.8177, min distance = 2.023363
AAT_ECOLI Fu	845 histi	HIS8_ECOLI Fu	444293 PI	1GEY	1X28	1GEX			Sc=5.82551, min distance = 2.02159
AAT_THET8 Fu	5962 L-ly	THER_BACTH Fu	6305 L-tr	1THL	1GC3	1KEI			Sc=5.66571, min distance = 2.33314
AAT_THET8 Fu	6057 L-ty	CHMU_YEAST Fu	6305 L-tr	5CSM	1GC3	4CSM			Sc=5.95408, min distance = 2.01148
AAT_THET8 Fu	6140 L-ph	APX_STRGR Ful	6305 L-tr	1TF8	1GC3	1F2P			Sc=5.75079, min distance = 2.47529
AAT_THET8 Fu	71567 D-p	APX_STRGR Ful	6305 L-tr	1TF8	1GC3	1TKH			Sc=5.74847, min distance = 2.54329
AAUA_ALCFA Fu	5326783 F	AOFB_HUMAN Fu	29147 4-N	2C70	2HJ4	1S3E			Sc=5.75235, min distance = 1.83744
AAUA_ALCFA Fu	6102778 C	AOFB_HUMAN Fu	29147 4-N	2C70	2HJ4	2C64			Sc=5.64708, min distance = 2.14980
ABDH_ECOLI Fu	6022 Aden	Q5SI02_THET8	5893 nadi	2BJK	1WNB	2BJA			Sc=6.352, min distance = 2.0948398
ABDH_ECOLI Fu	9548000 G	CD38_HUMAN Fu	5893 nadi	2I65	1WNB	2I66			Sc=6.07211, min distance = 2.19653
ABL1_HUMAN Fu	23653515	CDK2_HUMAN Fu	6022 Aden	1GY3	2G2I	2R3F	29.44		Sc=5.95868, min distance = 1.84958
ABL1_HUMAN Fu	23653516	CDK2_HUMAN Fu	6022 Aden	1GY3	2G2I	2R3G	29.44		Sc=6.23002, min distance = 2.23336

ABL1_HUMAN F	23653518	CDK2_HUMAN Fu	6022 Aden	1GY3	2G2I	2R3J	29.44		Sc=6.12466, min distance = 1.72870
ABL1_HUMAN F	23653519	CDK2_HUMAN Fu	6022 Aden	1GY3	2G2I	2R3K	29.44		Sc=6.20786, min distance = 2.05746
ABL1_HUMAN F	23653520	CDK2_HUMAN Fu	6022 Aden	1GY3	2G2I	2R3L	29.44		Sc=6.22132, min distance = 1.64234
ABL1_HUMAN F	23653521	CDK2_HUMAN Fu	6022 Aden	1GY3	2G2I	2R3M	29.44		Sc=6.32155, min distance = 2.31249
ABL1_HUMAN F	23653524	CDK2_HUMAN Fu	6022 Aden	1GY3	2G2I	2R3P	29.44		Sc=6.04661, min distance = 1.76663
ABL1_HUMAN F	24871491	INSR_HUMAN Fu	5287416 C	1RQQ	2G1T	2Z8C	41.34		Sc=6.44482, min distance = 2.35059
ABL1_HUMAN F	2608 1jsv	CDK2_HUMAN Fu	6022 Aden	1GY3	2G2I	1JSV	29.44		Sc=5.79394, min distance = 2.15473
ABL1_HUMAN F	398148 1e	CDK2_HUMAN Fu	6022 Aden	1GY3	2G2I	1E1X	29.44		Sc=6.09034, min distance = 2.13097
ABL1_HUMAN F	444345 1c	GSK3B_HUMAN F	6022 Aden	1J1C	2G2I	1Q3D	26.34		Sc=6.24676, min distance = 2.11130
ABL1_HUMAN F	445940 O6	CDK2_HUMAN Fu	6022 Aden	1GY3	2G2I	1GZ8	29.44		Sc=5.92595, min distance = 2.58056
ABL1_HUMAN F	447656 1c	CDK2_HUMAN Fu	6022 Aden	1GY3	2G2I	1OIY	29.44		Sc=6.33633, min distance = 1.59936
ABL1_HUMAN F	447962 1p	CDK2_HUMAN Fu	6022 Aden	1GY3	2G2I	2C5N	29.44		Sc=6.05943, min distance = 2.42399
ABL1_HUMAN F	4564 1e1v	CDK2_HUMAN Fu	6022 Aden	1GY3	2G2I	1E1V	29.44		Sc=5.9261, min distance = 2.825989
ABL1_HUMAN F	4565 1h1r	CDK2_HUMAN Fu	6022 Aden	1GY3	2G2I	1H1R	29.44		Sc=5.69541, min distance = 1.94359
ABL1_HUMAN F	5287844 i	GSK3B_HUMAN F	6022 Aden	1J1C	2G2I	1UV5	26.34		Sc=6.27097, min distance = 1.53417
ABL1_HUMAN F	5288641 i	CDK2_HUMAN Fu	6022 Aden	1GY3	2G2I	1E9H	29.44		Sc=6.17339, min distance = 2.29792
ABL1_HUMAN F	5326739 i	GSK3B_HUMAN F	6022 Aden	1J1C	2G2I	1Q41	26.34		Sc=6.18028, min distance = 2.20626
ABL1_HUMAN F	5327097 2	CDK2_HUMAN Fu	6022 Aden	1GY3	2G2I	2BTS	29.44		Sc=5.86359, min distance = 2.06396
ABL1_HUMAN F	5327130 C	CDK2_HUMAN Fu	6022 Aden	1GY3	2G2I	2C68	29.44		Sc=6.09239, min distance = 2.16619
ABL1_HUMAN F	5327131 T	CDK2_HUMAN Fu	6022 Aden	1GY3	2G2I	2C69	29.44		Sc=6.07393, min distance = 2.26455
ABL1_HUMAN F	5331010 c	CDK2_HUMAN Fu	6022 Aden	1GY3	2G2I	2BKZ	29.44		Sc=5.99154, min distance = 1.66436
ABL1_HUMAN F	5494414 C	CDK2_HUMAN Fu	6022 Aden	1GY3	2G2I	2A0C	29.44		Sc=6.39682, min distance = 2.26490
ABL1_HUMAN F	6420138 2	CDK2_HUMAN Fu	6022 Aden	1GY3	2G2I	2UUE	29.44		Sc=6.05196, min distance = 2.45584
ABL1_HUMAN F	6420139 C	CDK2_HUMAN Fu	6022 Aden	1GY3	2G2I	2C5V	29.44		Sc=6.07124, min distance = 1.76654
ABL1_HUMAN F	6539118 g	CDK2_HUMAN Fu	6022 Aden	1GY3	2G2I	1FVV	29.44		Sc=6.25425, min distance = 2.24112
ABL1_HUMAN F	6852187 2	O57883_PYRHO I	6022 Aden	1WNL	2G2I	2DTH		0.89	Sc=5.92764, min distance = 1.55246
ABL1_HUMAN F	6918710 5	CDK2_HUMAN Fu	6022 Aden	1GY3	2G2I	3EJ1	29.44		Sc=5.9324, min distance = 2.998814
ABL1_HUMAN F	9817550 V	CDK2_HUMAN Fu	6022 Aden	1GY3	2G2I	3BHV	29.44		Sc=6.16369, min distance = 2.92193
ABL_AGABI Fu	101798 Me	MBL1_RAT Full	82313 1rd	1KWV	1Y2W	1KWU			Sc=5.76236, min distance = 1.76013
ABL_AGABI Fu	185698 al	MBL1_RAT Full	82313 1rd	1KWV	1Y2W	2MSB			Sc=5.67761, min distance = 2.32880
ABL_AGABI Fu	439710 rh	PGRP_CAMDR Fu	82313 1rd	3C93	1Y2W	3CG9			Sc=5.67529, min distance = 2.02685
ABL_AGABI Fu	444863 is	MBL1_RAT Full	82313 1rd	1KWV	1Y2W	1KWX			Sc=5.69101, min distance = 2.40269
ABL_AGABI Fu	94214 Met	MBL1_RAT Full	82313 1rd	1KWV	1Y2W	1AFA			Sc=5.7749, min distance = 1.558431
ABP1_MAIZE F	24768551	TIR1_ARATH Fu	6862 Tran	2P1O	1LRH	3C6P			Sc=6.03992, min distance = 2.22638
ABP1_MAIZE F	445184 1,	EGFR_HUMAN Fu	185698 al	1MOX	1LR5	3C09		0.89	Sc=5.64849, min distance = 1.86858
ABP1_MAIZE F	445184 1,	Q6PYX1_HUMAN I	185698 al	1ZLS	1LR5	1ZLW		0.89	Sc=5.76222, min distance = 1.39589
ABP1_MAIZE F	5326557 C	QDOI_ASPJA Fu	439680 be	1JUH	1LR5	1GQH			Sc=5.6718, min distance = 0.764244

ACAC_YEAST F	5287830 6	Q8RLY5_LACHE I	190 adeni	1S2D	1OD2	1S2I		0.93	Sc=5.60724, min distance = 2.29385
ACAC_YEAST F	764 guani	PURR_ECOLI Fu	190 adeni	2PUB	1OD2	1WET			Sc=5.61763, min distance = 2.84114
ACCC_ECOLI F	11608401	KAPCA_BOVIN F	5957 Aden	1Q24	1DV2	2UVY			Sc=6.18428, min distance = 1.71121
ACCC_ECOLI F	3540 1yds	KAPCA_BOVIN F	5957 Aden	1Q24	1DV2	1YDS			Sc=5.83364, min distance = 2.00341
ACCC_ECOLI F	3547 Fasu	KAPCA_BOVIN F	5957 Aden	1Q24	1DV2	1Q8W			Sc=5.92246, min distance = 2.02259
ACCC_ECOLI F	3547 Fasu	ROCK1_HUMAN F	33113 gam	2V55	2J9G	2ESM			Sc=5.93592, min distance = 2.09696
ACCC_ECOLI F	36735 Gpp	Q9RVK2_DEIRA I	5957 Aden	1SU2	1DV2	1SZ3		0.8	Sc=6.44609, min distance = 1.89603
ACCC_ECOLI F	440317 AT	FTSK_PSEAE Fu	6022 Aden	2IUU	2J9G	2IUT		0.99	Sc=5.71634, min distance = 1.96593
ACCC_ECOLI F	440317 AT	PURT_ECOLI Fu	6022 Aden	1KJQ	2J9G	1KJJ		0.99	Sc=5.99741, min distance = 2.52562
ACCC_ECOLI F	444345 1c	PDPK1_HUMAN F	5957 Aden	2BIY	1DV2	1OKY			Sc=6.35042, min distance = 2.07158
ACCC_ECOLI F	444564 AD	MYS2_DICDI Fu	6022 Aden	1VOM	2J9G	1W9I		0.91	Sc=5.96309, min distance = 2.14824
ACCC_ECOLI F	444852 CI	ATPB_BOVIN Fu	5957 Aden	2V7Q	1DV2	1COW		0.91	Sc=6.02859, min distance = 0.55691
ACCC_ECOLI F	447916 ac	RIO1_ARCFU Fu	6022 Aden	1ZTH	2J9G	1ZTF		0.95	Sc=6.2074, min distance = 2.007985
ACCC_ECOLI F	449240 1y	KAPCA_BOVIN F	5957 Aden	1Q24	1DV2	1YDR			Sc=5.91374, min distance = 1.84552
ACCC_ECOLI F	5326976 1	CSK2A_MAIZE F	33113 gam	1LP4	2J9G	1ZOE			Sc=5.68088, min distance = 2.09632
ACCC_ECOLI F	5327148 C	IPKA_RABIT Fu	6022 Aden	1JBP	2J9G	2ERZ			Sc=6.13286, min distance = 2.19075
ACCC_ECOLI F	5789 thym	KITH_EHV4 Ful	6022 Aden	1P72	2J9G	1P6X			Sc=5.91546, min distance = 2.41554
ACCC_ECOLI F	6083 aden	AAKG1_RAT Ful	5957 Aden	2V92	1DV2	2V8Q		0.98	Sc=5.87576, min distance = 1.50513
ACCC_ECOLI F	6083 aden	PSPF_ECOLI Fu	5957 Aden	2C96	1DV2	2VII		0.98	Sc=5.83918, min distance = 2.33972
ACCC_ECOLI F	6083 aden	PURP_METJA Fu	6022 Aden	2R7N	2J9G	2R7M		0.99	Sc=5.84279, min distance = 2.63043
ACCC_ECOLI F	60961 ade	IPKA_RABIT Fu	6022 Aden	1JBP	2J9G	1FMO		0.95	Sc=6.20436, min distance = 2.35483
ACCC_ECOLI F	6132 Cyti	O33839_THEMA I	6022 Aden	1XJK	2J9G	1XJN			Sc=6.26782, min distance = 1.98174
ACCC_ECOLI F	6852207 M	KAPCA_BOVIN F	5957 Aden	1Q24	1DV2	2GNI			Sc=5.89862, min distance = 1.84903
ACCC_ECOLI F	72194 2-C	ENPL_CANFA Fu	6022 Aden	1TC6	2J9G	1QYE		0.89	Sc=6.10338, min distance = 2.20082
ACCC_ECOLI F	8977 1dar	O33839_THEMA I	6022 Aden	1XJK	2J9G	1XJE		0.8	Sc=5.66755, min distance = 2.13023
ACCC_ECOLI F	91532 AMP	MTNK_BACSU Fu	6022 Aden	2OLC	2J9G	2PUL		0.99	Sc=6.47483, min distance = 1.61607
ACCC_ECOLI F	91557 1tm	Q94M05_9VIRU I	6022 Aden	1W44	2J9G	1W48		0.99	Sc=5.75227, min distance = 1.66357
ACCC_PSEAE F	60961 ade	USHA_ECOLI Fu	92199 AMP	1HPU	2VQD	1HO5		0.93	Sc=6.25934, min distance = 2.36910
ACE2_HUMAN F	16741014	CARP_YEAST Fu	82313 1rd	1FMU	3D0G	1FQ7			Sc=5.71526, min distance = 2.01970
ACES_MOUSE F	3202 edro	ACES_TORCA Fu	2968 deca	1ACL	1MAA	2ACK	59.66		Sc=5.77444, min distance = 1.71676
ACES_MOUSE F	444211 th	ACES_TORCA Fu	20544 ACE	2C4H	2HA5	1ACJ	59.66		Sc=6.0504, min distance = 0.835120
ACES_MOUSE F	445892 1g	ACES_TORCA Fu	20544 ACE	2C4H	2HA5	1GQR	59.66		Sc=5.77201, min distance = 0.92171
ACES_MOUSE F	445892 1g	ACES_TORCA Fu	2968 deca	1ACL	1MAA	1GQR	59.66		Sc=5.64256, min distance = 2.13784
ACES_TORCA F	13455857	IGKC_MOUSE Fu	62551 Pen	1P7K	2BAG	3C6S			Sc=5.65594, min distance = 0.84745
ACES_TORCA F	187 acety	ACES_MOUSE Fu	20544 ACE	2HA5	2C4H	2HA4	59.66		Sc=5.78634, min distance = 0.88882
ACES_TORCA F	5327028 C	HNMT_HUMAN Fu	444211 th	2AOW	1ACJ	2AOX			Sc=5.94917, min distance = 1.48362
ACES_TORCA F	91420 5-T	ACES_MOUSE Fu	2968 deca	1MAA	1ACL	2HA0	59.66		Sc=5.64926, min distance = 0.66988

ACE_DROME Fu	16122592	ACE_HUMAN Ful	5362119 l	1O86	1J36	2OC2	44.25		Sc=6.77735, min distance = 2.30003
ACE_DROME Fu	24808492	ACE_HUMAN Ful	5362119 l	1O86	1J36	3BKK	44.25	0.78	Sc=6.6756, min distance = 2.498358
ACE_DROME Fu	24808493	ACE_HUMAN Ful	5362119 l	1O86	1J36	3BKL	44.25		Sc=6.27344, min distance = 2.48028
ACE_DROME Fu	5462501 E	ACE_HUMAN Ful	5362119 l	1O86	1J36	1UZE	44.25	0.99	Sc=6.38623, min distance = 2.48477
ACHP_LYMST F	190 adeni	PPAL_YEAST Fu	23831 HEP	1D1P	1UX2	1D2A			Sc=5.73246, min distance = 1.32334
ACHP_LYMST F	445939 1g	PPTA_ECOLI Fu	23831 HEP	1GYX	1UX2	1GYY			Sc=5.81517, min distance = 1.42067
ACHP_LYMST F	447414 5-	CPXA_PSEPU Fu	89594 nic	1P2Y	1UW6	1NOO			Sc=5.77793, min distance = 1.52521
ACHP_LYMST F	69590 4-E	CPXA_PSEPU Fu	89594 nic	1P2Y	1UW6	1PHD			Sc=5.63272, min distance = 2.54993
ACHP_LYMST F	854023 Ep	Q8WSF8_APLCA	23831 HEP	2BR7	1UX2	2BYQ	38.04		Sc=6.02395, min distance = 0.38422
ACKA_METTE F	23727981	CDK2_HUMAN Fu	6022 Aden	1GY3	1TUU	3BHT			Sc=6.16942, min distance = 2.05971
ACKA_METTE F	23727982	CDK2_HUMAN Fu	6022 Aden	1GY3	1TUU	3BHU			Sc=6.08151, min distance = 2.60660
ACKA_METTE F	398148 1e	CDK2_HUMAN Fu	6022 Aden	1GY3	1TUU	1E1X			Sc=6.29056, min distance = 2.17168
ACKA_METTE F	445966 O6	CDK2_HUMAN Fu	6022 Aden	1GY3	1TUU	1H0V			Sc=6.30787, min distance = 2.41515
ACKA_METTE F	446090 1h	NDKC_DICDI Fu	6022 Aden	1KDN	1TUU	1HIY		0.97	Sc=5.93109, min distance = 2.02709
ACKA_METTE F	447955 1p	CDK2_HUMAN Fu	6022 Aden	1GY3	1TUU	1PXI			Sc=5.77737, min distance = 2.47329
ACKA_METTE F	4565 1h1r	CDK2_HUMAN Fu	6022 Aden	1GY3	1TUU	1H1R			Sc=6.05981, min distance = 1.96406
ACKA_METTE F	5327148 C	IPKA_RABIT Fu	6022 Aden	1JBP	1TUU	2ERZ			Sc=6.17673, min distance = 2.27521
ACKA_METTE F	6030 Urid	ECX1_SULSO Fu	6083 aden	2C38	1TUU	2C37			Sc=6.25827, min distance = 1.74963
ACKA_METTE F	6031 Urid	RIR1_YEAST Fu	6022 Aden	2CVX	1TUU	2CVV			Sc=5.84739, min distance = 2.21631
ACKA_METTE F	60961 ade	IPKA_RABIT Fu	6022 Aden	1JBP	1TUU	1FMO		0.95	Sc=6.24577, min distance = 2.96260
ACKA_METTE F	611002 Op	PIM1_HUMAN Fu	6083 aden	1YXU	1TUU	1YXX			Sc=6.18446, min distance = 1.12016
ACKA_METTE F	65110 AIC	AAKG1_HUMAN F	6083 aden	2UV4	1TUU	2UV5		0.79	Sc=5.80714, min distance = 2.48475
ACKA_METTE F	657135 Op	PIM1_HUMAN Fu	6083 aden	1YXU	1TUU	1YXV			Sc=5.87164, min distance = 1.67495
ACKA_METTE F	8977 1dar	O33839_THEMA	6022 Aden	1XJK	1TUU	1XJE		0.8	Sc=5.97528, min distance = 2.37663
ACKA_METTE F	8977 1dar	PARM_ECOLX Fu	6022 Aden	1MWM	1TUU	2ZGY		0.8	Sc=5.64334, min distance = 2.31246
ACKA_METTE F	9547921 C	SYQ_ECOLI Ful	6083 aden	1ZJW	1TUU	2RE8		0.82	Sc=6.05199, min distance = 2.38471
ACKA_METTE F	9817550 V	CDK2_HUMAN Fu	6022 Aden	1GY3	1TUU	3BHV			Sc=6.40458, min distance = 1.97812
ACON2_ECOLI	311 citri	ACON_BOVIN Fu	444212 tr	1ACO	1L5J	1C96			Sc=6.04864, min distance = 2.33264
ACON2_ECOLI	445607 AT	ACON_BOVIN Fu	444212 tr	1ACO	1L5J	1FGH		0.8	Sc=6.04542, min distance = 2.35236
ACON2_ECOLI	447376 2-	ACON_BOVIN Fu	444212 tr	1ACO	1L5J	1NIS			Sc=5.95237, min distance = 2.31525
ACON2_ECOLI	449604 NI	ACON_BOVIN Fu	444212 tr	1ACO	1L5J	8ACN			Sc=5.95336, min distance = 2.46783
ACON2_ECOLI	5318532 i	ACON_BOVIN Fu	444212 tr	1ACO	1L5J	1C97			Sc=6.05503, min distance = 2.24941
ACON2_ECOLI	5459784 m	ACON_BOVIN Fu	444212 tr	1ACO	1L5J	1AMI			Sc=6.10707, min distance = 1.63813
ACON_BOVIN F	444591 CI	IDH_ECOLI Ful	5318532 i	1P8F	1C97	1BL5			Sc=5.69603, min distance = 2.03719
ACON_PIG Ful	444591 CI	IDH_ECOLI Ful	5318532 i	1P8F	7ACN	1BL5			Sc=5.70204, min distance = 2.33168
ACON_PIG Ful	51 2-Oxop	SERA_ECOLI Fu	311 citri	2P9E	1B0K	1YBA		0.76	Sc=5.89032, min distance = 0.893525
ACRB_ECOLI F	444305 L-	Q3XZW8_ENTFC	311 citri	2JFV	2GIF	2JFW			Sc=5.6374, min distance = 1.122574

ACRB_ECOLI F	446944 CI	MIF_HUMAN Ful	311 citri	1GD0	2GIF	1LJT			Sc=6.03521, min distance = 1.222804
ACRB_ECOLI F	51 2-Oxop	SERA_ECOLI Fu	311 citri	2P9E	2GIF	1YBA		0.76	Sc=5.85509, min distance = 0.672127
ACRB_ECOLI F	5354052 p	MIF_HUMAN Ful	311 citri	1GD0	2GIF	2OOZ			Sc=5.64728, min distance = 1.192200
ACS1_YEAST F	24316 cyc	PDE10_HUMAN F	6083 aden	2OUN	1RY2	2OUU		0.81	Sc=6.37349, min distance = 2.295464
ACS1_YEAST F	440641 1m	O57693_THETE	6083 aden	1UXU	1RY2	1UXR			Sc=5.90497, min distance = 2.359319
ACS1_YEAST F	445708 AM	NADE_ECOLI Fu	6083 aden	1WXI	1RY2	1WXE		0.91	Sc=6.39829, min distance = 2.036588
ACS1_YEAST F	447832 AD	ACSA_SALTY Fu	6083 aden	2P2F	1RY2	1PG4		0.99	Sc=6.47725, min distance = 2.468098
ACS1_YEAST F	5327121 I	PIM1_HUMAN Fu	6083 aden	1YXU	1RY2	2C3I			Sc=6.36032, min distance = 1.167598
ACS1_YEAST F	6076 Cycl	PDE10_HUMAN F	6083 aden	2OUN	1RY2	2OUR		0.99	Sc=6.31932, min distance = 2.033618
ACS1_YEAST F	611002 Op	PIM1_HUMAN Fu	6083 aden	1YXU	1RY2	1YXX			Sc=6.24196, min distance = 1.825887
ACS1_YEAST F	657135 Op	PIM1_HUMAN Fu	6083 aden	1YXU	1RY2	1YXV			Sc=5.96072, min distance = 2.142645
ACS1_YEAST F	6804 guar	PDE10_HUMAN F	6083 aden	2OUN	1RY2	2OUQ		0.8	Sc=6.39965, min distance = 0
ACS2A_HUMAN	3540 1yds	KAPCA_BOVIN F	5957 Aden	1Q24	3C5E	1YDS			Sc=5.96099, min distance = 2.168378
ACS2A_HUMAN	449240 1y	KAPCA_BOVIN F	5957 Aden	1Q24	3C5E	1YDR			Sc=6.04164, min distance = 2.135043
ACSA_SALTY F	101609 Ur	Q9SSV1_NICGU	6083 aden	1VD1	2P2F	1VD3			Sc=6.29854, min distance = 2.007964
ACSA_SALTY F	24180721	PIM1_HUMAN Fu	6083 aden	1YXU	2P2F	3C4E			Sc=6.08229, min distance = 1.900135
ACSA_SALTY F	440641 1m	O57693_THETE	6083 aden	1UXU	2P2F	1UXR			Sc=5.98054, min distance = 2.025103
ACSA_SALTY F	445708 AM	NADE_ECOLI Fu	6083 aden	1WXI	2P2F	1WXE		0.91	Sc=6.39217, min distance = 2.246473
ACSA_SALTY F	446916 1l	F16P1_PIG Ful	6083 aden	2F3D	2P2F	1LEV			Sc=6.27671, min distance = 1.836150
ACSA_SALTY F	5327121 I	PIM1_HUMAN Fu	6083 aden	1YXU	2P2F	2C3I			Sc=6.3652, min distance = 1.3802108
ACSA_SALTY F	6076 Cycl	PDE10_HUMAN F	6083 aden	2OUN	2P2F	2OUR		0.99	Sc=6.3563, min distance = 2.1474480
ACSA_SALTY F	65110 AIC	PURP_METJA Fu	6083 aden	2R7M	2P2F	2R7K		0.79	Sc=6.33475, min distance = 2.196191
ACSA_SALTY F	65533 cor	O57693_THETE	6083 aden	1UXU	2P2F	1UXT			Sc=5.97436, min distance = 2.028202
ACSA_SALTY F	657135 Op	PIM1_HUMAN Fu	6083 aden	1YXU	2P2F	1YXV			Sc=5.97463, min distance = 2.055515
ACSA_SALTY F	8582 Inos	PYGM_RABIT Fu	6083 aden	8GPB	2P2F	2QN7		0.79	Sc=6.42061, min distance = 2.094676
ACT1_CAEEL F	444852 CI	ATPA1_BOVIN F	5957 Aden	2V7Q	1D4X	1COW		0.91	Sc=6.53366, min distance = 2.096723
ACT1_CAEEL F	444852 CI	ATPB_BOVIN Fu	5957 Aden	2V7Q	1D4X	1COW		0.91	Sc=6.52703, min distance = 2.182128
ACT1_CAEEL F	449240 1y	KAPCA_BOVIN F	5957 Aden	1Q24	1D4X	1YDR			Sc=6.02282, min distance = 2.026184
ACT1_CAEEL F	6022 Ader	BIOD_ECOLI Fu	5957 Aden	1A82	1D4X	1DAD		0.99	Sc=6.46469, min distance = 2.233815
ACT1_CAEEL F	6022 Ader	HSP7F_YEAST F	5957 Aden	3D2F	1D4X	3C7N		0.99	Sc=6.4803, min distance = 2.1327222
ACT1_CAEEL F	6022 Ader	MUTS_ECOLI Fu	5957 Aden	1W7A	1D4X	1OH7		0.99	Sc=6.45867, min distance = 1.240013
ACT1_CAEEL F	6022 Ader	PURK_ECOLI Fu	5957 Aden	3ETH	1D4X	3ETJ		0.99	Sc=6.45701, min distance = 2.093752
ACT1_CAEEL F	6022 Ader	Q72H90_THET2	5957 Aden	2BEK	1D4X	2BEJ		0.99	Sc=6.46179, min distance = 2.203094
ACT1_DICDI F	11608401	KAPCA_BOVIN F	5957 Aden	1Q24	3CIP	2UVY			Sc=6.25916, min distance = 2.168943
ACT1_DICDI F	11708454	KAPCA_BOVIN F	5957 Aden	1Q24	3CIP	2VNW			Sc=6.15219, min distance = 2.563482
ACT1_DICDI F	199528 D-	SYW2_DEIRA Fu	5957 Aden	1YID	3CIP	1YIA			Sc=6.15749, min distance = 2.105704
ACT1_DICDI F	3540 1yds	KAPCA_BOVIN F	5957 Aden	1Q24	3CIP	1YDS			Sc=5.93725, min distance = 2.177964

ACT1_DICDI F	3547 Fasu	KAPCA_BOVIN F	5957 Aden	1Q24	3CIP	1Q8W		Sc=5.99721, min distance = 2.02760
ACT1_DICDI F	444564 AD	BIOD_ECOLI Fu	5957 Aden	1A82	3CIP	1BS1	0.91	Sc=6.44573, min distance = 2.15044
ACT1_DICDI F	444852 CI	ATPB_BOVIN Fu	5957 Aden	2V7Q	3CIP	1COW	0.91	Sc=6.50029, min distance = 2.01222
ACT1_DICDI F	6022 Aden	BIOD_ECOLI Fu	5957 Aden	1A82	3CIP	1DAD	0.99	Sc=6.4463, min distance = 2.151525
ACT1_DICDI F	6022 Aden	KTHY_HUMAN Fu	5957 Aden	1E2Q	3CIP	1NN3	0.99	Sc=6.4389, min distance = 2.103409
ACT1_DICDI F	6022 Aden	MUTS_ECOLI Fu	5957 Aden	1W7A	3CIP	1OH7	0.99	Sc=5.97842, min distance = 2.07792
ACT1_DICDI F	6022 Aden	PURK_ECOLI Fu	5957 Aden	3ETH	3CIP	3ETJ	0.99	Sc=6.42829, min distance = 2.19263
ACT1_DICDI F	6022 Aden	Q72H90_THET2	5957 Aden	2BEK	3CIP	2BEJ	0.99	Sc=6.44051, min distance = 2.34373
ACT1_DICDI F	6022 Aden	REX_BACSU Full	5957 Aden	2VT3	3CIP	2VT2	0.99	Sc=6.47359, min distance = 1.70153
ACT1_DICDI F	6022 Aden	SECA_ECOLI Fu	5957 Aden	2FSG	3CIP	2FSI	0.99	Sc=6.45105, min distance = 2.04600
ACT1_DICDI F	6102784 A	PSPF_ECOLI Fu	5957 Aden	2C96	3CIP	2C99	0.99	Sc=6.05155, min distance = 2.06828
ACT1_DROME F	3540 1yds	KAPCA_BOVIN F	5957 Aden	1Q24	2HF4	1YDS		Sc=5.94888, min distance = 2.01709
ACT1_DROME F	3547 Fasu	KAPCA_BOVIN F	5957 Aden	1Q24	2HF4	1Q8W		Sc=6.0109, min distance = 2.012260
ACT1_DROME F	36735 Gpp	PARM_ECOLX Fu	6022 Aden	1MWM	2HF3	2ZGZ	0.79	Sc=6.08198, min distance = 1.89667
ACT1_DROME F	444564 AD	BIOD_ECOLI Fu	5957 Aden	1A82	2HF4	1BS1	0.91	Sc=6.45364, min distance = 2.26763
ACT1_DROME F	444842 CI	CDK2_HUMAN Fu	6022 Aden	1GY3	2HF3	1CKP		Sc=5.60314, min distance = 2.29722
ACT1_DROME F	444852 CI	ATPA1_BOVIN F	5957 Aden	2V7Q	2HF4	1COW	0.91	Sc=6.52484, min distance = 2.02265
ACT1_DROME F	444852 CI	ATPB_BOVIN Fu	5957 Aden	2V7Q	2HF4	1COW	0.91	Sc=6.51235, min distance = 2.25202
ACT1_DROME F	446090 1h	NDKC_DICDI Fu	6022 Aden	1KDN	2HF3	1HIY	0.97	Sc=6.48312, min distance = 2.00535
ACT1_DROME F	447955 1p	CDK2_HUMAN Fu	6022 Aden	1GY3	2HF3	1PXI		Sc=5.80402, min distance = 2.36688
ACT1_DROME F	448505 AD	NDKC_DICDI Fu	6022 Aden	1KDN	2HF3	1S5Z	0.93	Sc=6.50746, min distance = 2.27988
ACT1_DROME F	449240 1y	KAPCA_BOVIN F	5957 Aden	1Q24	2HF4	1YDR		Sc=6.02133, min distance = 2.09576
ACT1_DROME F	4565 1h1r	CDK2_HUMAN Fu	6022 Aden	1GY3	2HF3	1H1R		Sc=6.13568, min distance = 2.06844
ACT1_DROME F	6083 aden	PURP_METJA Fu	5957 Aden	2R7L	2HF4	2R7M	0.98	Sc=6.41811, min distance = 2.01170
ACT1_DROME F	6083 aden	PURP_METJA Fu	6022 Aden	2R7N	2HF3	2R7M	0.99	Sc=6.404, min distance = 2.2284508
ACT1_DROME F	60961 ade	IPKA_RABIT Fu	6022 Aden	1JBP	2HF3	1FMO	0.95	Sc=6.27292, min distance = 2.49239
ACT1_DROME F	6804 guar	NDK_PYRHO Full	6022 Aden	2DYA	2HF3	2DXF	0.8	Sc=6.43092, min distance = 2.08340
ACT1_DROME F	6852187 2	O57883_PYRHO	6022 Aden	1WNL	2HF3	2DTH	0.89	Sc=6.1598, min distance = 1.695416
ACT1_DROME F	8977 1dar	NDK_PYRHO Full	6022 Aden	2DYA	2HF3	2DXE	0.8	Sc=6.4911, min distance = 2.251535
ACT1_TETTH F	11708454	KAPCA_BOVIN F	5957 Aden	1Q24	1DEJ	2VNW		Sc=6.16825, min distance = 2.58644
ACT1_TETTH F	3540 1yds	KAPCA_BOVIN F	5957 Aden	1Q24	1DEJ	1YDS		Sc=5.93948, min distance = 2.05884
ACT1_TETTH F	3547 Fasu	KAPCA_BOVIN F	5957 Aden	1Q24	1DEJ	1Q8W		Sc=6.00009, min distance = 2.08620
ACT1_TETTH F	444564 AD	BIOD_ECOLI Fu	5957 Aden	1A82	1DEJ	1BS1	0.91	Sc=6.45576, min distance = 2.36364
ACT1_TETTH F	444852 CI	ATPA1_BOVIN F	5957 Aden	2V7Q	1DEJ	1COW	0.91	Sc=6.52616, min distance = 2.02894
ACT1_TETTH F	444852 CI	ATPB_BOVIN Fu	5957 Aden	2V7Q	1DEJ	1COW	0.91	Sc=6.51458, min distance = 2.19817
ACT1_TETTH F	6022 Aden	ACT1_DROME Fu	5957 Aden	2HF4	1DEJ	2HF3	0.99	Sc=6.46298, min distance = 2.63912
ACT1_TETTH F	6022 Aden	ARSA1_ECOLX F	5957 Aden	1II0	1DEJ	1IHU	0.99	Sc=6.45867, min distance = 2.18929

ACT1_TETTH F	6022 Ader	BIOD_ECOLI Fu	5957 Aden	1A82	1DEJ	1DAD		0.99	Sc=6.45175, min distance = 2.03817
ACT1_TETTH F	6022 Ader	HSP7F_YEAST F	5957 Aden	3D2F	1DEJ	3C7N		0.99	Sc=6.46696, min distance = 1.99229
ACT1_TETTH F	6022 Ader	PURK_ECOLI Fu	5957 Aden	3ETH	1DEJ	3ETJ		0.99	Sc=6.44188, min distance = 1.95708
ACT1_TETTH F	6022 Ader	Q72H90_THET2 I	5957 Aden	2BEK	1DEJ	2BEJ		0.99	Sc=6.45175, min distance = 2.22878
ACT3_STRCO F	15942680	6PGD_LACLM Fu	5886 NADF	2IZ0	2RHC	2IZ1			Sc=6.24645, min distance = 2.19073
ACT3_STRCO F	440141 9i	DHB1_HUMAN Fu	5886 NADF	1QYV	2RHC	1QYW	31.63		Sc=6.4169, min distance = 2.294048
ACT3_STRCO F	440141 9i	G6PD_LEUME Fu	5886 NADF	1H9A	2RHC	1E7Y			Sc=6.43049, min distance = 2.01293
ACT3_STRCO F	6022 Ader	HMDH_HUMAN Fu	5886 NADF	1DQA	2RHC	1HW8			Sc=5.72219, min distance = 1.92302
ACTB_BOVIN F	11708454	KAPCA_BOVIN F	5957 Aden	1Q24	2BTF	2VNW			Sc=6.14809, min distance = 2.49539
ACTB_BOVIN F	3540 1yds	KAPCA_BOVIN F	5957 Aden	1Q24	2BTF	1YDS			Sc=5.9272, min distance = 2.087855
ACTB_BOVIN F	444564 AI	BIOD_ECOLI Fu	5957 Aden	1A82	2BTF	1BS1		0.91	Sc=6.44521, min distance = 2.21809
ACTB_BOVIN F	444852 CI	ATPA1_BOVIN F	5957 Aden	2V7Q	2BTF	1COW		0.91	Sc=6.51606, min distance = 1.77040
ACTB_BOVIN F	6022 Ader	ACT1_DROME Fu	5957 Aden	2HF4	2BTF	2HF3		0.99	Sc=6.45105, min distance = 2.60886
ACTB_BOVIN F	6022 Ader	ARSA1_ECOLX F	5957 Aden	1II0	2BTF	1IHU		0.99	Sc=6.45044, min distance = 2.31463
ACTB_BOVIN F	6022 Ader	BIOD_ECOLI Fu	5957 Aden	1A82	2BTF	1DAD		0.99	Sc=6.44446, min distance = 2.35943
ACTB_BOVIN F	6022 Ader	HSP7F_YEAST F	5957 Aden	3D2F	2BTF	3C7N		0.99	Sc=6.45927, min distance = 2.09830
ACTB_BOVIN F	6022 Ader	MUTS_ECOLI Fu	5957 Aden	1W7A	2BTF	1OH7		0.99	Sc=6.45436, min distance = 1.04401
ACTB_BOVIN F	6022 Ader	PURK_ECOLI Fu	5957 Aden	3ETH	2BTF	3ETJ		0.99	Sc=6.44264, min distance = 2.04731
ACTB_BOVIN F	6022 Ader	Q72H90_THET2 I	5957 Aden	2BEK	2BTF	2BEJ		0.99	Sc=6.44521, min distance = 2.26065
ACTB_BOVIN F	6022 Ader	REX_BACSU Ful	5957 Aden	2VT3	2BTF	2VT2		0.99	Sc=6.48088, min distance = 1.62951
ACTB_BOVIN F	6083 ader	PURP_METJA Fu	5957 Aden	2R7L	2BTF	2R7M		0.98	Sc=6.40532, min distance = 2.00328
ACTB_BOVIN F	6852207 M	KAPCA_BOVIN F	5957 Aden	1Q24	2BTF	2GNI			Sc=5.97731, min distance = 2.09495
ACTB_BOVIN F	91532 AMI	PURT_ECOLI Fu	5957 Aden	1KJ8	2BTF	1KJI		0.99	Sc=6.53207, min distance = 2.00780
ACTH_THEAC F	1540 1h1c	CDK2_HUMAN Fu	6022 Aden	1GY3	2FSN	1H1Q			Sc=6.26795, min distance = 2.01901
ACTH_THEAC F	36735 Gpp	PARM_ECOLX Fu	6022 Aden	1MWM	2FSN	2ZGZ		0.79	Sc=6.45963, min distance = 1.91317
ACTH_THEAC F	444564 AI	BIOD_ECOLI Fu	6022 Aden	1DAD	2FSN	1BS1		0.91	Sc=6.38671, min distance = 1.93888
ACTH_THEAC F	444852 CI	ATPB_BOVIN Fu	6022 Aden	2CK3	2FSN	1COW		0.9	Sc=6.45963, min distance = 2.11321
ACTH_THEAC F	445966 O6	CDK2_HUMAN Fu	6022 Aden	1GY3	2FSN	1H0V			Sc=6.06802, min distance = 1.65827
ACTH_THEAC F	446090 1h	NDKC_DICDI Fu	6022 Aden	1KDN	2FSN	1HIY		0.97	Sc=6.41587, min distance = 2.31922
ACTH_THEAC F	447649 1c	CDK2_HUMAN Fu	6022 Aden	1GY3	2FSN	1OI9			Sc=6.37965, min distance = 1.66756
ACTH_THEAC F	447916 ac	RIO1_ARCFU Fu	6022 Aden	1ZTH	2FSN	1ZTF		0.95	Sc=6.17584, min distance = 2.66778
ACTH_THEAC F	447955 1p	CDK2_HUMAN Fu	6022 Aden	1GY3	2FSN	1PXI			Sc=5.68534, min distance = 1.87331
ACTH_THEAC F	448505 AI	NDKC_DICDI Fu	6022 Aden	1KDN	2FSN	1S5Z		0.93	Sc=6.44809, min distance = 2.05413
ACTH_THEAC F	4564 1e1v	CDK2_HUMAN Fu	6022 Aden	1GY3	2FSN	1E1V			Sc=5.99673, min distance = 2.12136
ACTH_THEAC F	4565 1h1r	CDK2_HUMAN Fu	6022 Aden	1GY3	2FSN	1H1R			Sc=6.27341, min distance = 2.09386
ACTH_THEAC F	5957 Ader	ARP3_BOVIN Fu	6022 Aden	2P9I	2FSN	1TYQ		0.99	Sc=6.46674, min distance = 2.30025
ACTH_THEAC F	5957 Ader	HSP7F_YEAST F	6022 Aden	3C7N	2FSN	3D2F		0.99	Sc=6.45463, min distance = 1.81043

ACTH_THEAC Fu	5957 Aden	MUTS_ECOLI Ful	6022 Aden	1OH7	2FSN	1W7A	0.99	Sc=6.45463, min distance = 1.730232
ACTH_THEAC Fu	5957 Aden	PPCK_ECOLI Ful	6022 Aden	1K3C	2FSN	2OLR	0.99	Sc=5.98974, min distance = 2.268700
ACTH_THEAC Fu	6083 aden	PURP_METJA Ful	6022 Aden	2R7N	2FSN	2R7M	0.99	Sc=6.30457, min distance = 2.223230
ACTH_THEAC Fu	60961 ade	IPKA_RABIT Ful	6022 Aden	1JBP	2FSN	1FMO	0.95	Sc=6.16697, min distance = 2.061535
ACTH_THEAC Fu	6338562 C	MYS2_DICDI Ful	6022 Aden	1VOM	2FSN	1D0Y		Sc=6.21437, min distance = 2.012246
ACTH_THEAC Fu	6804 guar	NDK_PYRHO Ful	6022 Aden	2DYA	2FSN	2DXF	0.8	Sc=6.3274, min distance = 2.334451
ACTH_THEAC Fu	6852187 2	O57883_PYRHO I	6022 Aden	1WNL	2FSN	2DTH	0.89	Sc=5.98944, min distance = 2.072242
ACTH_THEAC Fu	8977 1dar	NDK_PYRHO Ful	6022 Aden	2DYA	2FSN	2DXE	0.8	Sc=6.39617, min distance = 2.172688
ACTH_THEAC Fu	8977 1dar	O33839_THEMA I	6022 Aden	1XJK	2FSN	1XJE	0.8	Sc=5.97799, min distance = 1.944681
ACTH_THEAC Fu	8977 1dar	RIR1_YEAST Ful	6022 Aden	2CVX	2FSN	2CVW	0.8	Sc=6.43253, min distance = 1.834388
ACTH_THEAC Fu	91532 AMP	NIFH1_AZOVI Fu	6022 Aden	1FP6	2FSN	2AFK	0.99	Sc=6.47861, min distance = 1.293337
ACTH_THEAC Fu	9547890 I	CDK2_HUMAN Ful	6022 Aden	1GY3	2FSN	1W8C		Sc=6.05347, min distance = 2.284503
ACTH_THEAC Fu	9991833 S	CDK2_HUMAN Ful	6022 Aden	1GY3	2FSN	2R3H		Sc=6.13164, min distance = 1.013101
ACTNA_DICDI I	1540 1h1q	CDK2_HUMAN Ful	6022 Aden	1GY3	1G8X	1H1Q		Sc=6.36505, min distance = 1.932771
ACTNA_DICDI I	160355 rc	CDK2_HUMAN Ful	6022 Aden	1GY3	1G8X	3DDQ		Sc=6.45007, min distance = 1.282803
ACTNA_DICDI I	23653515	CDK2_HUMAN Ful	6022 Aden	1GY3	1G8X	2R3F		Sc=6.05632, min distance = 1.993488
ACTNA_DICDI I	23653518	CDK2_HUMAN Ful	6022 Aden	1GY3	1G8X	2R3J		Sc=6.25142, min distance = 1.630301
ACTNA_DICDI I	23653519	CDK2_HUMAN Ful	6022 Aden	1GY3	1G8X	2R3K		Sc=6.25142, min distance = 1.761540
ACTNA_DICDI I	23653520	CDK2_HUMAN Ful	6022 Aden	1GY3	1G8X	2R3L		Sc=6.24677, min distance = 1.821338
ACTNA_DICDI I	23653524	CDK2_HUMAN Ful	6022 Aden	1GY3	1G8X	2R3P		Sc=6.32019, min distance = 1.734336
ACTNA_DICDI I	24864078	CDK2_HUMAN Ful	6022 Aden	1GY3	1G8X	2VTO		Sc=6.43407, min distance = 1.658217
ACTNA_DICDI I	24916751	CDK2_HUMAN Ful	6022 Aden	1GY3	1G8X	3DOG		Sc=6.45472, min distance = 1.501571
ACTNA_DICDI I	444564 AD	BIOD_ECOLI Ful	6022 Aden	1DAD	1G8X	1BS1	0.91	Sc=5.99201, min distance = 2.362302
ACTNA_DICDI I	444564 AD	MYS2_DICDI Ful	6022 Aden	1VOM	1G8X	1W9I	0.91	Sc=5.97786, min distance = 2.300487
ACTNA_DICDI I	445940 O6	CDK2_HUMAN Ful	6022 Aden	1GY3	1G8X	1GZ8		Sc=6.17772, min distance = 2.236998
ACTNA_DICDI I	445966 O6	CDK2_HUMAN Ful	6022 Aden	1GY3	1G8X	1H0V		Sc=6.15212, min distance = 2.118268
ACTNA_DICDI I	447004 CI	MYS2_DICDI Ful	6022 Aden	1VOM	1G8X	1LVK		Sc=6.16766, min distance = 2.245732
ACTNA_DICDI I	447656 1c	CDK2_HUMAN Ful	6022 Aden	1GY3	1G8X	1OIY		Sc=6.49394, min distance = 1.683820
ACTNA_DICDI I	447955 1p	CDK2_HUMAN Ful	6022 Aden	1GY3	1G8X	1PXI		Sc=5.70708, min distance = 1.732209
ACTNA_DICDI I	447962 1p	CDK2_HUMAN Ful	6022 Aden	1GY3	1G8X	2C5N		Sc=6.29686, min distance = 1.779745
ACTNA_DICDI I	448014 1c	GSK3B_HUMAN Fu	6022 Aden	1J1C	1G8X	1Q5K		Sc=6.30404, min distance = 1.432196
ACTNA_DICDI I	4565 1h1r	CDK2_HUMAN Ful	6022 Aden	1GY3	1G8X	1H1R		Sc=6.35603, min distance = 2.079322
ACTNA_DICDI I	5327130 C	CDK2_HUMAN Ful	6022 Aden	1GY3	1G8X	2C68		Sc=6.2584, min distance = 1.790960
ACTNA_DICDI I	5327133 C	CDK2_HUMAN Ful	6022 Aden	1GY3	1G8X	2C6K		Sc=6.20587, min distance = 2.315640
ACTNA_DICDI I	5957 Aden	BIOD_ECOLI Ful	6022 Aden	1DAD	1G8X	1A82	0.99	Sc=6.50791, min distance = 2.405166
ACTNA_DICDI I	6031 Uric	DCK_HUMAN Ful	6022 Aden	1P5Z	1G8X	2ZIA		Sc=6.34458, min distance = 2.080465
ACTNA_DICDI I	6132 Cyti	ECX1_PYRAB Ful	6022 Aden	2PO0	1G8X	2PO2		Sc=5.89408, min distance = 2.190709

ACTNA_DICDI I	6132 Cyti	ECX2_PYRAB Fu	6022 Aden	2PO0	1G8X	2PO2			Sc=5.87962, min distance = 2.47202
ACTNA_DICDI I	6338562 C	MYS2_DICDI Fu	6022 Aden	1VOM	1G8X	1D0Y			Sc=6.2615, min distance = 1.886945
ACTNA_DICDI I	6420139 C	CDK2_HUMAN Fu	6022 Aden	1GY3	1G8X	2C5V			Sc=6.2694, min distance = 1.694899
ACTNA_DICDI I	91532 AMP	BIOD_ECOLI Fu	6022 Aden	1DAD	1G8X	1DAG		0.99	Sc=6.52984, min distance = 1.98265
ACTNA_DICDI I	9547890 I	CDK2_HUMAN Fu	6022 Aden	1GY3	1G8X	1W8C			Sc=6.27284, min distance = 1.53020
ACTNA_DICDI I	9994066 4	CDK2_HUMAN Fu	6022 Aden	1GY3	1G8X	2VTJ			Sc=5.83618, min distance = 2.18017
ACTS_CHICK F	11708454	KAPCA_BOVIN F	5957 Aden	1Q24	1MDU	2VNW			Sc=6.15219, min distance = 2.19642
ACTS_CHICK F	3540 1yds	KAPCA_BOVIN F	5957 Aden	1Q24	1MDU	1YDS			Sc=5.93846, min distance = 1.95039
ACTS_CHICK F	3547 Fasu	KAPCA_BOVIN F	5957 Aden	1Q24	1MDU	1Q8W			Sc=6.00204, min distance = 2.05212
ACTS_CHICK F	444564 AD	BIOD_ECOLI Fu	5957 Aden	1A82	1MDU	1BS1		0.91	Sc=6.44841, min distance = 2.21856
ACTS_CHICK F	444852 CI	ATPB_BOVIN Fu	5957 Aden	2V7Q	1MDU	1COW		0.91	Sc=6.51235, min distance = 2.20924
ACTS_CHICK F	447004 CI	MYS2_DICDI Fu	5957 Aden	1FMW	1MDU	1LVK			Sc=6.53064, min distance = 0
ACTS_CHICK F	449240 1y	KAPCA_BOVIN F	5957 Aden	1Q24	1MDU	1YDR			Sc=6.01252, min distance = 2.26761
ACTS_CHICK F	6022 Aden	HSP7F_YEAST F	5957 Aden	3D2F	1MDU	3C7N		0.99	Sc=6.46586, min distance = 2.37685
ACTS_CHICK F	6022 Aden	MUTS_ECOLI Fu	5957 Aden	1W7A	1MDU	1OH7		0.99	Sc=6.44981, min distance = 1.03942
ACTS_CHICK F	6022 Aden	PURK_ECOLI Fu	5957 Aden	3ETH	1MDU	3ETJ		0.99	Sc=6.4389, min distance = 2.106864
ACTS_CHICK F	6022 Aden	Q72H90_THET2 I	5957 Aden	2BEK	1MDU	2BEJ		0.99	Sc=6.44573, min distance = 2.16400
ACTS_CHICK F	6022 Aden	REX_BACSU Ful	5957 Aden	2VT3	1MDU	2VT2		0.99	Sc=6.48088, min distance = 1.52011
ACTS_CHICK F	6022 Aden	Y059_METJA Fu	5957 Aden	2J9C	1MDU	2J9D		0.99	Sc=6.44981, min distance = 1.08835
ACTS_RABIT F	11708454	KAPCA_BOVIN F	5957 Aden	1Q24	2FXU	2VNW			Sc=6.17821, min distance = 2.46814
ACTS_RABIT F	17727 Do	FMS1_YEAST Fu	1102 sper	3CN8	2HMP	3BI2			Sc=5.75009, min distance = 2.15251
ACTS_RABIT F	3540 1yds	KAPCA_BOVIN F	5957 Aden	1Q24	2FXU	1YDS			Sc=5.93948, min distance = 2.12143
ACTS_RABIT F	3547 Fasu	KAPCA_BOVIN F	5957 Aden	1Q24	2FXU	1Q8W			Sc=6.00767, min distance = 2.07817
ACTS_RABIT F	36735 Gpp	PARM_ECOLX Fu	6022 Aden	1MWM	1J6Z	2ZGZ		0.79	Sc=6.53236, min distance = 1.71276
ACTS_RABIT F	444564 AD	BIOD_ECOLI Fu	5957 Aden	1A82	2FXU	1BS1		0.91	Sc=6.46237, min distance = 2.14664
ACTS_RABIT F	444852 CI	ATPA1_BOVIN F	5957 Aden	2V7Q	2FXU	1COW		0.91	Sc=6.5299, min distance = 2.019638
ACTS_RABIT F	444852 CI	ATPB_BOVIN Fu	5957 Aden	2V7Q	2FXU	1COW		0.91	Sc=6.51557, min distance = 2.05205
ACTS_RABIT F	446090 1h	NDKC_DICDI Fu	6022 Aden	1KDN	1J6Z	1HIY		0.97	Sc=6.48679, min distance = 1.84539
ACTS_RABIT F	447652 In	CDK2_HUMAN Fu	6022 Aden	1GY3	1J6Z	1OIQ			Sc=6.30432, min distance = 2.01464
ACTS_RABIT F	447955 1p	CDK2_HUMAN Fu	6022 Aden	1GY3	1J6Z	1PXI			Sc=5.79875, min distance = 2.35435
ACTS_RABIT F	449240 1y	KAPCA_BOVIN F	5957 Aden	1Q24	2FXU	1YDR			Sc=6.01898, min distance = 2.17088
ACTS_RABIT F	4565 1h1r	CDK2_HUMAN Fu	6022 Aden	1GY3	1J6Z	1H1R			Sc=6.15359, min distance = 1.92257
ACTS_RABIT F	6083 aden	ENTP2_RAT Ful	33113 gam	3CJA	1NWK	3CJ7		0.97	Sc=6.41036, min distance = 2.37991
ACTS_RABIT F	6083 aden	HSP71_HUMAN F	33113 gam	2E8A	1NWK	1XQS		0.97	Sc=6.39311, min distance = 2.60450
ACTS_RABIT F	6083 aden	HSP71_HUMAN F	6022 Aden	1S3X	1J6Z	1XQS		0.99	Sc=6.3991, min distance = 2.226881
ACTS_RABIT F	6083 aden	PURP_METJA Fu	6022 Aden	2R7N	1J6Z	2R7M		0.99	Sc=6.40468, min distance = 2.13914
ACTS_RABIT F	60961 ade	IPKA_RABIT Fu	6022 Aden	1JBP	1J6Z	1FMO		0.95	Sc=6.28497, min distance = 2.59227

ACTS_RABIT F	6804 guar	NDK_PYRHO Ful	6022 Aden	2DYA	1J6Z	2DXF	0.8	Sc=6.42525, min distance = 2.203467
ACTS_RABIT F	6852187 2	O57883_PYRHO	6022 Aden	1WNL	1J6Z	2DTH	0.89	Sc=6.16781, min distance = 1.505582
ACTS_RABIT F	8977 1dar	NDK_PYRHO Ful	6022 Aden	2DYA	1J6Z	2DXE	0.8	Sc=6.49642, min distance = 2.108994
ACTS_RABIT F	8977 1dar	PARM_ECOLX Fu	6022 Aden	1MWM	1J6Z	2ZGY	0.8	Sc=6.4895, min distance = 2.2297842
ACT_YEAST Fu	3540 1yds	KAPCA_BOVIN F	5957 Aden	1Q24	1YAG	1YDS		Sc=5.9509, min distance = 2.1201653
ACT_YEAST Fu	3547 Fasu	KAPCA_BOVIN F	5957 Aden	1Q24	1YAG	1Q8W		Sc=6.01664, min distance = 2.040275
ACT_YEAST Fu	444564 AD	BIOD_ECOLI Fu	5957 Aden	1A82	1YAG	1BS1	0.91	Sc=6.46179, min distance = 2.406539
ACT_YEAST Fu	444852 CI	ATPB_BOVIN Fu	5957 Aden	2V7Q	1YAG	1COW	0.91	Sc=6.51709, min distance = 2.154017
ACT_YEAST Fu	447004 CI	MYS2_DICDI Fu	5957 Aden	1FMW	1YAG	1LVK		Sc=6.54024, min distance = 0
ACT_YEAST Fu	449240 1y	KAPCA_BOVIN F	5957 Aden	1Q24	1YAG	1YDR		Sc=6.02048, min distance = 2.014196
ACT_YEAST Fu	6022 Aden	BIOD_ECOLI Fu	5957 Aden	1A82	1YAG	1DAD	0.99	Sc=6.46179, min distance = 2.173615
ACT_YEAST Fu	6022 Aden	HSP7F_YEAST F	5957 Aden	3D2F	1YAG	3C7N	0.99	Sc=6.47542, min distance = 2.072206
ACT_YEAST Fu	6022 Aden	MUTS_ECOLI Fu	5957 Aden	1W7A	1YAG	1OH7	0.99	Sc=6.45576, min distance = 2.048447
ACT_YEAST Fu	6022 Aden	PURK_ECOLI Fu	5957 Aden	3ETH	1YAG	3ETJ	0.99	Sc=6.44981, min distance = 2.221154
ACT_YEAST Fu	6022 Aden	Q72H90_THET2	5957 Aden	2BEK	1YAG	2BEJ	0.99	Sc=6.46179, min distance = 2.299069
ACT_YEAST Fu	6022 Aden	Y059_METJA Fu	5957 Aden	2J9C	1YAG	2J9D	0.99	Sc=6.45761, min distance = 2.126733
ACT_YEAST Fu	91532 AMP	BIOD_ECOLI Fu	5957 Aden	1A82	1YAG	1DAG	0.99	Sc=6.5483, min distance = 2.0534453
ADA_BOVIN Fu	14257 Unc	BACR_HALSA Fu	185698 al	1IW6	1W1I	1CWQ		Sc=5.63955, min distance = 1.276998
ADA_BOVIN Fu	444205 CI	PGH1_SHEEP Fu	439680 be	1Q4G	1W1I	1EQG		Sc=6.04328, min distance = 0.833770
ADF1_ARATH F	445070 fa	AOFB_HUMAN Fu	444279 1s	1OJD	1F7S	2BK3		Sc=5.69613, min distance = 2.515192
ADH1A_HUMAN	11987786	LDH_PLAFD Ful	5893 nadi	1T2D	1U3T	1T2E	0.77	Sc=6.02484, min distance = 2.244400
ADH1A_HUMAN	123927 A3	DAPB_ECOLI Fu	5893 nadi	1DRU	1U3T	1DRV	0.97	Sc=6.30678, min distance = 2.178563
ADH1A_HUMAN	439153 Di	ADH1B_HUMAN F	5893 nadi	1U3U	1U3T	1DEH	0.79	Sc=6.76419, min distance = 1.274646
ADH1A_HUMAN	439153 Di	ADH1E_HORSE F	5893 nadi	1MGO	1U3T	2JHF	0.79	Sc=6.3114, min distance = 2.5833081
ADH1A_HUMAN	440141 9i	G6PD_LEUME Fu	5893 nadi	1H94	1U3T	1E7Y		Sc=5.62387, min distance = 2.685464
ADH1B_HUMAN	11987786	LDH_PLAFD Ful	5893 nadi	1T2D	1U3U	1T2E	0.77	Sc=6.32095, min distance = 2.218233
ADH1B_HUMAN	123927 A3	DAPB_ECOLI Fu	5893 nadi	1DRU	1U3U	1DRV	0.97	Sc=6.7644, min distance = 2.1757311
ADH1E_HORSE	11987786	LDH_PLAFD Ful	5893 nadi	1T2D	1MGO	1T2E	0.77	Sc=6.75312, min distance = 2.244898
ADH1E_HORSE	123927 A3	DAPB_ECOLI Fu	5893 nadi	1DRU	1MGO	1DRV	0.97	Sc=6.75536, min distance = 2.004813
ADH1E_HORSE	16129587	G3PA_SPIOL Fu	5893 nadi	1NBO	1MGO	2PKR		Sc=6.79124, min distance = 1.600864
ADH1E_HORSE	4369002 C	LDHA_PIG Full	5893 nadi	9LDB	1MGO	9LDT	0.77	Sc=6.75832, min distance = 2.507805
ADH1E_HORSE	440516 CI	MDH_THETH Ful	5893 nadi	1BMD	1MGO	1BDM	0.79	Sc=6.32292, min distance = 2.550483
ADH1E_HORSE	5957 Aden	REX_BACSU Ful	6022 Aden	2VT2	1JU9	2VT3	0.99	Sc=6.43591, min distance = 1.606127
ADH1G_HUMAN	123927 A3	DAPB_ECOLI Fu	5893 nadi	1DRU	1U3W	1DRV	0.97	Sc=6.77055, min distance = 2.116721
ADH1G_HUMAN	446288 NA	G3P_PALVE Ful	5893 nadi	1DSS	1U3W	1IHX		Sc=6.77404, min distance = 1.648782
ADH1G_HUMAN	5957 Aden	KTRA_BACSU Fu	5893 nadi	1LSU	1U3W	2HMW		Sc=6.03402, min distance = 2.044054
ADH1S_HORSE	11987786	LDH_PLAFD Ful	5893 nadi	1T2D	1EE2	1T2E	0.77	Sc=6.75955, min distance = 2.000625

ADH1S_HORSE I	4369002 C	LDHA_PIG Full=	5893 nadi	9LDB	1EE2	9LDT		0.77	Sc=6.76187, min distance = 2.30926
ADH1S_HORSE I	439153 Di	CTBP1_RAT Ful	5893 nadi	1HKU	1EE2	1HL3		0.79	Sc=6.76266, min distance = 2.10946
ADH1S_HORSE I	439153 Di	GALE_ECOLI Fu	5893 nadi	1UDC	1EE2	1UDB		0.79	Sc=6.76665, min distance = 2.05941
ADH1S_HORSE I	446288 NA	G3P_PALVE Ful	5893 nadi	1DSS	1EE2	1IHX			Sc=6.7662, min distance = 1.690069
ADH1S_HORSE I	5957 Ader	MAOM_HUMAN Fu	5893 nadi	1PJ3	1EE2	1GZ4			Sc=6.49587, min distance = 0.92998
ADH1_ENTHI Fu	23624247	WRBA_ECOLI Fu	62551 Pen	3B6K	2OUI	3B6I			Sc=5.73537, min distance = 2.22528
ADH1_ENTHI Fu	444211 th	ACES_TORCA Fu	8200 TETR	1DX6	2OUI	1ACJ			Sc=5.87388, min distance = 2.14050
ADH1_GADCA Fu	11987786	LDH_PLAFD Ful	5893 nadi	1T2D	1CDO	1T2E		0.77	Sc=6.27166, min distance = 2.55218
ADH1_GADCA Fu	123927 A3	DAPB_ECOLI Fu	5893 nadi	1DRU	1CDO	1DRV		0.97	Sc=6.72015, min distance = 2.14801
ADH1_GADCA Fu	123927 A3	Q9GT92_CRYPV I	5893 nadi	2FM3	1CDO	2EWD		0.97	Sc=6.71837, min distance = 2.80074
ADH1_GADCA Fu	439153 Di	ADH1E_HORSE Fu	5893 nadi	1MGO	1CDO	2JHF		0.79	Sc=6.72252, min distance = 2.76695
ADH1_GADCA Fu	439153 Di	CTBP1_RAT Ful	5893 nadi	1HKU	1CDO	1HL3		0.79	Sc=6.70927, min distance = 2.24352
ADH1_GADCA Fu	440516 CI	MDH_THETH Ful	5893 nadi	1BMD	1CDO	1BDM		0.79	Sc=6.28122, min distance = 2.12608
ADH1_GADCA Fu	445794 AI	ADHX_HUMAN Fu	5893 nadi	2FZW	1CDO	2FZE			Sc=6.57162, min distance = 2.19119
ADH1_GADCA Fu	446288 NA	G3P_PALVE Ful	5893 nadi	1DSS	1CDO	1IHX			Sc=6.73138, min distance = 2.23923
ADH4_HUMAN Fu	123927 A3	Q9GT92_CRYPV I	5893 nadi	2FM3	3COS	2EWD		0.97	Sc=6.74547, min distance = 2.44036
ADH4_HUMAN Fu	439153 Di	ADH1B_HUMAN Fu	5893 nadi	1U3U	3COS	1DEH		0.79	Sc=6.75142, min distance = 2.22191
ADH4_HUMAN Fu	439153 Di	CTBP1_RAT Ful	5893 nadi	1HKU	3COS	1HL3		0.79	Sc=6.7321, min distance = 2.343432
ADH4_HUMAN Fu	6102710 A	Q4PRK9_PLAVI I	5893 nadi	2A92	3COS	2AA3		0.95	Sc=6.74507, min distance = 2.46587
ADH4_MOUSE Fu	11987786	LDH_PLAFD Ful	5893 nadi	1T2D	1E3I	1T2E		0.77	Sc=5.99634, min distance = 2.66371
ADH4_MOUSE Fu	123927 A3	Q9GT92_CRYPV I	5893 nadi	2FM3	1E3I	2EWD		0.97	Sc=5.99898, min distance = 2.63357
ADH4_MOUSE Fu	439153 Di	ADH1B_HUMAN Fu	5893 nadi	1U3U	1E3I	1DEH		0.79	Sc=6.28621, min distance = 2.38315
ADH4_MOUSE Fu	439153 Di	CTBP1_RAT Ful	5893 nadi	1HKU	1E3I	1HL3		0.79	Sc=6.27297, min distance = 2.71864
ADH4_MOUSE Fu	440516 CI	MDH_THETH Ful	5893 nadi	1BMD	1E3I	1BDM		0.79	Sc=5.83413, min distance = 2.61481
ADH4_MOUSE Fu	445794 AI	ADHX_HUMAN Fu	5893 nadi	2FZW	1E3I	2FZE			Sc=5.82652, min distance = 2.82534
ADH4_MOUSE Fu	446050 CI	G3P_HUMAN Ful	5893 nadi	1U8F	1E3I	3GPD		0.95	Sc=6.28117, min distance = 2.19675
ADH4_MOUSE Fu	446288 NA	G3P_PALVE Ful	5893 nadi	1DSS	1E3I	1IHX			Sc=6.27783, min distance = 2.12651
ADH4_MOUSE Fu	6102710 A	Q4PRK9_PLAVI I	5893 nadi	2A92	1E3I	2AA3		0.95	Sc=6.28577, min distance = 2.66299
ADH6_YEAST Fu	15942680	6PGD_LACLM Fu	5886 NADP	2IZ0	1PS0	2IZ1			Sc=6.27282, min distance = 0.81549
ADH6_YEAST Fu	440141 9i	DHB1_HUMAN Fu	5886 NADP	1QYV	1PS0	1QYW			Sc=5.632, min distance = 2.1011323
ADH7_HUMAN Fu	11987786	LDH_PLAFD Ful	5893 nadi	1T2D	1D1T	1T2E		0.77	Sc=6.74779, min distance = 2.15772
ADH7_HUMAN Fu	123927 A3	DAPB_ECOLI Fu	5893 nadi	1DRU	1D1T	1DRV		0.97	Sc=6.7577, min distance = 1.922683
ADH7_HUMAN Fu	4369002 C	LDHA_PIG Full=	5893 nadi	9LDB	1D1T	9LDT		0.77	Sc=6.7446, min distance = 2.424180
ADH7_HUMAN Fu	439153 Di	ADH1B_HUMAN Fu	5893 nadi	1U3U	1D1T	1DEH		0.79	Sc=6.80093, min distance = 1.47006
ADH7_HUMAN Fu	439153 Di	CTBP1_RAT Ful	5893 nadi	1HKU	1D1T	1HL3		0.79	Sc=6.74683, min distance = 1.86893
ADH7_HUMAN Fu	439153 Di	GALE_ECOLI Fu	5893 nadi	1UDC	1D1T	1UDB		0.79	Sc=6.75575, min distance = 2.00275
ADH7_HUMAN Fu	440516 CI	MDH_THETH Ful	5893 nadi	1BMD	1D1T	1BDM		0.79	Sc=6.319, min distance = 2.3770675

ADH7_HUMAN Fu	6083 ader	NADE_ECOLI Ful	5893 nadi	1WXH	1D1T	1WXI			Sc=5.78628, min distance = 2.31096
ADH7_HUMAN Fu	6420113 C	MDH_THETH Ful	5893 nadi	1BMD	1D1T	1WZI		0.77	Sc=6.22271, min distance = 0.75045
ADH8_RANPE Fu	440141 9i	G6PD_LEUME Fu	5886 NADF	1H9A	1P0F	1E7Y			Sc=5.88887, min distance = 2.55701
ADHX_HUMAN Fu	11987786	LDH_PLAFD Ful	5893 nadi	1T2D	2FZW	1T2E		0.77	Sc=6.00278, min distance = 2.34303
ADHX_HUMAN Fu	15942665	INHA_MYCTU Fu	5893 nadi	2H7I	2FZW	2H9I		0.88	Sc=6.83624, min distance = 2.08332
ADHX_HUMAN Fu	15942690	INHA_MYCTU Fu	5893 nadi	2H7I	2FZW	2NTJ			Sc=6.11107, min distance = 2.08940
ADHX_HUMAN Fu	16129587	G3PA_SPIOL Fu	5893 nadi	1NBO	2FZW	2PKR			Sc=6.05605, min distance = 1.74547
ADHX_HUMAN Fu	169266 1,	GALE_HUMAN Fu	5893 nadi	1HZJ	2FZW	1I3K		0.79	Sc=6.75455, min distance = 1.87426
ADHX_HUMAN Fu	4369002 C	LDHA_PIG Full=	5893 nadi	9LDB	2FZW	9LDT		0.77	Sc=6.7497, min distance = 1.134301
ADHX_HUMAN Fu	439153 Di	ADH1E_HORSE Fu	5893 nadi	1MGO	2FZW	2JHF	62.2	0.79	Sc=6.73586, min distance = 2.46002
ADHX_HUMAN Fu	439153 Di	CTBP1_RAT Ful	5893 nadi	1HKU	2FZW	1HL3		0.79	Sc=6.7248, min distance = 2.253640
ADHX_HUMAN Fu	440516 CI	MDH_THETH Ful	5893 nadi	1BMD	2FZW	1BDM		0.79	Sc=6.3109, min distance = 1.242383
ADHX_HUMAN Fu	6022 Ader	ADH1E_HORSE Fu	445794 AD	5ADH	2FZE	1JU9	62.2	0.95	Sc=5.92857, min distance = 2.55337
ADHX_HUMAN Fu	6022 Ader	YMX7_YEAST Fu	445794 AD	1TXZ	2FZE	1TY8		0.95	Sc=6.38455, min distance = 2.38023
ADH_DROLE Fu	165230 1a	G6PD_LEUME Fu	5893 nadi	1H94	1SBY	2DPG			Sc=6.53008, min distance = 1.66522
ADH_DROLE Fu	439153 Di	Q9BJJ9_PLAFA	5893 nadi	1UH5	1SBY	1V35		0.79	Sc=6.76955, min distance = 2.08420
ADH_DROLE Fu	440141 9i	G6PD_LEUME Fu	5893 nadi	1H94	1SBY	1E7Y			Sc=6.47978, min distance = 2.25578
ADH_DROLE Fu	6083 ader	NADE_ECOLI Fu	5893 nadi	1WXH	1SBY	1WXI			Sc=6.33616, min distance = 2.11631
ADH_DROME Fu	5893 nadi	GALE_HUMAN Fu	169266 1,	1I3K	1MG5	1HZJ		0.79	Sc=6.77643, min distance = 2.25973
ADH_SULSO Fu	11987786	LDH_PLAFD Ful	5893 nadi	1T2D	1R37	1T2E		0.77	Sc=6.78175, min distance = 2.58107
ADH_SULSO Fu	123927 A3	DAPB_ECOLI Fu	5893 nadi	1DRU	1R37	1DRV		0.97	Sc=6.32361, min distance = 2.08861
ADH_SULSO Fu	4369002 C	LDHA_PIG Full=	5893 nadi	9LDB	1R37	9LDT		0.77	Sc=6.77582, min distance = 2.62703
ADH_SULSO Fu	439153 Di	ADH1B_HUMAN Fu	5893 nadi	1U3U	1R37	1DEH		0.79	Sc=6.79947, min distance = 1.37771
ADH_SULSO Fu	439153 Di	ADH1E_HORSE Fu	5893 nadi	1MGO	1R37	2JHF		0.79	Sc=6.77549, min distance = 2.51563
ADH_SULSO Fu	439153 Di	CTBP1_RAT Ful	5893 nadi	1HKU	1R37	1HL3		0.79	Sc=6.77175, min distance = 2.15228
ADH_SULSO Fu	440516 CI	MDH_THETH Ful	5893 nadi	1BMD	1R37	1BDM		0.79	Sc=6.34771, min distance = 2.19548
ADH_SULSO Fu	445794 AD	ADHX_HUMAN Fu	5893 nadi	2FZW	1R37	2FZE			Sc=6.6338, min distance = 2.314121
ADH_SULSO Fu	446288 NA	G3P_PALVE Ful	5893 nadi	1DSS	1R37	1IHX			Sc=6.77777, min distance = 1.73991
ADH_SULTO Fu	123927 A3	DAPB_ECOLI Fu	5893 nadi	1DRU	2EER	1DRV		0.97	Sc=6.32297, min distance = 2.10205
ADH_SULTO Fu	439153 Di	ADH1B_HUMAN Fu	5893 nadi	1U3U	2EER	1DEH		0.79	Sc=6.80188, min distance = 1.15969
ADH_SULTO Fu	439153 Di	ADH1E_HORSE Fu	5893 nadi	1MGO	2EER	2JHF		0.79	Sc=6.77549, min distance = 2.55424
ADH_SULTO Fu	440516 CI	MDH_THETH Ful	5893 nadi	1BMD	2EER	1BDM		0.79	Sc=6.34661, min distance = 1.96801
ADH_THEBR Fu	15942680	6PGD_LACLM Fu	5886 NADF	2IZ0	2NVB	2IZ1			Sc=6.20541, min distance = 0.98756
ADH_THEBR Fu	16741136	DYR_ECOLI Ful	5886 NADF	1RA2	2NVB	1RX9	1		Sc=5.90584, min distance = 2.00029
ADH_THEBR Fu	440141 9i	G6PD_LEUME Fu	5886 NADF	1H9A	2NVB	1E7Y			Sc=6.24492, min distance = 2.17679
ADK_HUMAN Fu	24180721	PIM1_HUMAN Fu	60961 ade	1YI4	1BX4	3C4E			Sc=5.95519, min distance = 2.57710
ADK_HUMAN Fu	5281701 T	PIM1_HUMAN Fu	60961 ade	1YI4	1BX4	2O65			Sc=6.31553, min distance = 2.45687

ADK_HUMAN Fu	5327148	KAPCA_MOUSE F	60961 ade	1FMO	1BX4	2ERZ		Sc=6.17763, min distance = 2.05373
ADK_HUMAN Fu	6083 ade	SYQ_ECOLI Ful	60961 ade	1O0B	1BX4	1ZJW	0.96	Sc=6.32397, min distance = 1.66416
ADK_HUMAN Fu	611002 Op	PIM1_HUMAN Fu	60961 ade	1YI4	1BX4	1YXX		Sc=6.22907, min distance = 1.21875
ADK_HUMAN Fu	6802 gua	A5KE01_PLAVI	60961 ade	2PGF	1BX4	2QVN	0.79	Sc=6.2993, min distance = 2.290676
ADK_HUMAN Fu	8975 2-F	SAHH_MYCTU Fu	60961 ade	3CE6	1BX4	2ZJ0	0.99	Sc=6.24562, min distance = 1.91236
ADK_TOXGO Fu	21704 Vi	DEOD_ECOLI Fu	9570 6-Me	1PR4	2AA0	1PW7	0.88	Sc=6.16907, min distance = 2.18059
ADK_TOXGO Fu	439176 M	MTAP_SULSO Fu	60961 ade	1JDV	1DGM	1JDT	0.91	Sc=6.15601, min distance = 1.97206
ADK_TOXGO Fu	447722 9-	DEOD_ECOLI Fu	60961 ade	1PK7	1DGM	1OUM	0.9	Sc=6.20594, min distance = 1.70725
ADK_TOXGO Fu	447722 9-	DEOD_ECOLI Fu	9570 6-Me	1PR4	2AA0	1OUM	0.84	Sc=6.1842, min distance = 1.970005
ADK_TOXGO Fu	447724 9-	DEOD_ECOLI Fu	60961 ade	1PK7	1DGM	1OV6	0.9	Sc=6.1949, min distance = 0.604048
ADK_TOXGO Fu	447864 2	DEOD_ECOLI Fu	9570 6-Me	1PR4	2AA0	1PKE	0.85	Sc=6.1141, min distance = 1.986004
ADK_TOXGO Fu	447916 a	DEOD_ECOLI Fu	9570 6-Me	1PR4	2AA0	1PR6	0.88	Sc=6.23841, min distance = 1.73929
ADK_TOXGO Fu	5327148	KAPCA_MOUSE F	60961 ade	1FMO	1DGM	2ERZ		Sc=6.12828, min distance = 2.13607
ADK_TOXGO Fu	6021 inos	DEOD_ECOLI Fu	9570 6-Me	1PR4	2AA0	1PR0		Sc=6.19256, min distance = 1.86685
ADK_TOXGO Fu	6021 inos	TMPC_TREPA Fu	60961 ade	2FQY	1DGM	2FQW	0.79	Sc=5.8074, min distance = 2.883030
ADK_TOXGO Fu	6083 ade	SYQ_ECOLI Ful	60961 ade	1O0B	1DGM	1ZJW	0.96	Sc=6.20403, min distance = 2.86314
ADK_TOXGO Fu	6245 tube	DEOD_ECOLI Fu	9570 6-Me	1PR4	2AA0	1PR5		Sc=6.20716, min distance = 2.41266
ADK_TOXGO Fu	6802 gua	TMPC_TREPA Fu	60961 ade	2FQY	1DGM	2FQX	0.79	Sc=5.86362, min distance = 2.05471
ADK_TOXGO Fu	72443 for	DEOD_ECOLI Fu	9570 6-Me	1PR4	2AA0	1A69		Sc=6.27849, min distance = 2.05792
ADK_TOXGO Fu	8975 2-F	DEOD_ECOLI Fu	9570 6-Me	1PR4	2AA0	1PK9	0.87	Sc=6.20895, min distance = 1.49981
ADK_TOXGO Fu	8975 2-F	SAHH_MYCTU Fu	60961 ade	3CE6	1DGM	2ZJ0	0.99	Sc=5.8408, min distance = 2.182479
ADK_TOXGO Fu	97184 6-	DEOD_ECOLI Fu	60961 ade	1PK7	1DGM	1OVG	0.9	Sc=6.08846, min distance = 1.75996
ADK_TOXGO Fu	97184 6-	DEOD_ECOLI Fu	9570 6-Me	1PR4	2AA0	1OVG	0.83	Sc=6.13917, min distance = 1.69555
ADOK_MYCTU F	33113 ga	PURT_ECOLI Fu	91532 AM	1KJI	2PKN	1EYZ	0.99	Sc=5.97261, min distance = 2.13718
ADOK_MYCTU F	440317 A	PKNB_MYCTU Fu	91532 AM	1O6Y	2PKN	1MRU	0.99	Sc=6.42238, min distance = 2.29890
ADOK_MYCTU F	444564 A	BIOD_ECOLI Fu	91532 AM	1DAG	2PKN	1BS1	0.91	Sc=5.90565, min distance = 2.35876
ADOK_MYCTU F	5957 Ade	AROK_MYCTU Fu	91532 AM	1ZYU	2PKN	2IYW	0.99	Sc=5.97002, min distance = 2.41225
ADOK_MYCTU F	5957 Ade	BIOD_ECOLI Fu	91532 AM	1DAG	2PKN	1A82	0.99	Sc=5.77037, min distance = 2.35076
ADOK_MYCTU F	6022 Ade	BIOD_ECOLI Fu	91532 AM	1DAG	2PKN	1DAD	0.99	Sc=5.91218, min distance = 2.23088
ADOK_MYCTU F	6022 Ade	DNAA_AQUAE Fu	91532 AM	2HCB	2PKN	1L8Q	0.99	Sc=5.87978, min distance = 2.12498
ADOK_MYCTU F	6022 Ade	PURP_METJA Fu	91532 AM	2R7K	2PKN	2R7N	0.99	Sc=5.90213, min distance = 2.13890
ADOK_MYCTU F	6022 Ade	PURT_ECOLI Fu	91532 AM	1KJI	2PKN	1KJQ	0.99	Sc=6.36524, min distance = 2.10289
ADOK_MYCTU F	6022 Ade	RBSK_ECOLI Fu	91532 AM	1GQT	2PKN	1RKD	0.99	Sc=6.40192, min distance = 2.45347
ADOK_MYCTU F	6083 ade	PURP_METJA Fu	91532 AM	2R7K	2PKN	2R7M	0.97	Sc=5.84529, min distance = 2.27528
ADPP_ECOLI F	6083 ade	Q84CU3_THETH	445794 A	1V8M	1G9Q	1V8S	0.94	Sc=5.99031, min distance = 2.40128
ADRO_BOVIN F	11987634	TYTR_TRYCR Fu	444188 C	1BZL	1CJC	1AOG	0.97	Sc=6.43069, min distance = 1.80951
ADRO_BOVIN F	15942680	6PGD_LACLM Fu	5886 NAD	2IZ0	1E1L	2IZ1		Sc=6.26898, min distance = 1.38142

ADRO_BOVIN F	23631921	LSD1_HUMAN Fu	444188 CI 2DW4	1CJC	2Z3Y	0.91	Sc=6.48997, min distance = 2.04609
ADRO_BOVIN F	444502 CI	GSHR_HUMAN Fu	444188 CI 3DK9	1CJC	1BWC	0.98	Sc=6.42459, min distance = 2.12351
ADRO_BOVIN F	444502 CI	TYTR_TRYCR Fu	444188 CI 1BZL	1CJC	1GXF	0.98	Sc=6.42818, min distance = 2.21352
ADRO_BOVIN F	446013 1,	O28603_ARCFU	444188 CI 1JNR	1CJC	1JNZ	0.94	Sc=6.42696, min distance = 2.05418
ADRO_BOVIN F	449465 CI	GSHR_HUMAN Fu	444188 CI 3DK9	1CJC	3GRT	0.98	Sc=6.42774, min distance = 2.34221
ADRO_BOVIN F	6420174 C	O28603_ARCFU	444188 CI 1JNR	1CJC	2FJB	0.92	Sc=6.43622, min distance = 2.01139
ADRO_BOVIN F	6420174 C	O28604_ARCFU	444188 CI 1JNR	1CJC	2FJB	0.92	Sc=6.43622, min distance = 2.05802
AGAL_HUMAN F	15133 m-X	CARP_YEAST Fu	439680 be 1DPJ	1R46	1FQ5		Sc=5.69304, min distance = 2.14086
AGAL_HUMAN F	439731 al	ARAF_ECOLI Fu	439357 ga 8ABP	1R47	6ABP	0.93	Sc=5.65485, min distance = 2.37728
AGAL_HUMAN F	439764 be	ARAF_ECOLI Fu	439357 ga 8ABP	1R47	6ABP	0.93	Sc=5.64101, min distance = 2.47397
AGAL_ORYSJ F	444635 O3	ATLE_AGRAE Fu	439353 be 1WW6	1UAS	1WW5	0.9	Sc=5.78694, min distance = 2.02190
AGAL_ORYSJ F	444635 O3	MBL1_RAT Full	439353 be 2KMB	1UAS	3KMB	0.9	Sc=5.80192, min distance = 1.61330
AGAL_ORYSJ F	445340 1e	ELBP_ECOLX Fu	439353 be 1LT3	1UAS	1EFI		Sc=6.25204, min distance = 1.95393
AGAL_ORYSJ F	446996 GA	Q9HYN5_PSEAE	439353 be 1W8F	1UAS	1W8H	0.89	Sc=5.80655, min distance = 1.96248
AGAL_ORYSJ F	657082 GA	LEG3_HUMAN Fu	439353 be 1KJL	1UAS	1KJR	0.75	Sc=5.65947, min distance = 2.53230
AGGL_RICCO F	439731 al	ARAF_ECOLI Fu	439353 be 8ABP	1RZO	6ABP	0.93	Sc=5.61389, min distance = 2.51097
AGGL_RICCO F	439764 be	ARAF_ECOLI Fu	439353 be 8ABP	1RZO	6ABP	0.93	Sc=5.60269, min distance = 2.45479
AGGL_RICCO F	7027 gluc	Q72KX2_THET2	439353 be 2B3F	1RZO	2B3B		Sc=5.83805, min distance = 2.24153
AGI1_WHEAT F	8761 Bici	MLTB_ECOLI Fu	24139 ace 1QUT	2UVO	1QUS		Sc=5.61263, min distance = 2.07025
AGI2_WHEAT F	985 palmi	EST1_HUMAN Fu	444885 O- 2H7C	2WGC	2DQY		Sc=5.88534, min distance = 2.31834
AGLA_THEMA F	123927 A3	DAPB_ECOLI Fu	5893 nadi 1DRU	1OBB	1DRV	0.97	Sc=6.31378, min distance = 1.24334
AGLA_THEMA F	439153 Di	ADH1B_HUMAN F	5893 nadi 1U3U	1OBB	1DEH	0.79	Sc=6.40404, min distance = 1.28991
AGLA_THEMA F	439153 Di	ADH1E_HORSE F	5893 nadi 1MGO	1OBB	2JHF	0.79	Sc=6.70429, min distance = 1.04459
AGLA_THEMA F	445794 AD	ADHX_HUMAN Fu	5893 nadi 2FZW	1OBB	2FZE		Sc=6.60603, min distance = 2.14001
AGLA_THEMA F	6022 Ader	Q5SI02_THET8	5893 nadi 2BJK	1OBB	2BJA		Sc=6.2882, min distance = 2.331489
AGLA_THEMA F	6102710 A	Q4PRK9_PLAVI	5893 nadi 2A92	1OBB	2AA3	0.95	Sc=6.41208, min distance = 1.15172
AGO2_DROME F	444915 Gl	CDGT2_BACCI F	439341 ma 1KCL	1R6Z	1CXL		Sc=5.81074, min distance = 1.15569
AGO2_DROME F	447139 CI	MALE_ECOLI Fu	439341 ma 1JVY	1R6Z	1MDQ	0.91	Sc=6.38652, min distance = 2.49652
AHPF_ECOLI F	16740985	FRDA_SHEFR Fu	444188 CI 1M64	1FL2	1E39	0.93	Sc=6.43837, min distance = 2.23358
AHPF_ECOLI F	444502 CI	FRDA_SHEFR Fu	444188 CI 1M64	1FL2	1QJD	0.98	Sc=6.43899, min distance = 2.30118
AHPF_ECOLI F	446013 1,	O28603_ARCFU	444188 CI 1JNR	1FL2	1JNZ	0.94	Sc=6.4433, min distance = 2.287864
AHPF_ECOLI F	446013 1,	O28604_ARCFU	444188 CI 1JNR	1FL2	1JNZ	0.94	Sc=6.44358, min distance = 2.25397
AHPF_ECOLI F	449465 CI	GSHR_HUMAN Fu	444188 CI 3DK9	1FL2	3GRT	0.98	Sc=6.43856, min distance = 2.32331
AHPF_SALTY F	16740985	FRDA_SHEFR Fu	444188 CI 1M64	1HYU	1E39	0.93	Sc=6.86079, min distance = 2.24604
AHPF_SALTY F	444502 CI	FRDA_SHEFR Fu	444188 CI 1M64	1HYU	1QJD	0.98	Sc=6.86228, min distance = 2.31542
AHPF_SALTY F	445794 AD	PHHY_PSEFL Fu	444188 CI 1PBE	1HYU	2PHH		Sc=6.21202, min distance = 2.09537
AHPF_SALTY F	446013 1,	O28604_ARCFU	444188 CI 1JNR	1HYU	1JNZ	0.94	Sc=6.86696, min distance = 2.36407

AHPF_SALTY F	449465 CI	GSHR_HUMAN Fu	444188 CI	3DK9	1HYU	3GRT		0.98	Sc=6.86991, min distance = 2.449002
AIFM1_HUMAN I	11987634	GLPD_ECOLI Fu	444188 CI	2QCU	1M6I	2R4E		0.97	Sc=6.44053, min distance = 2.011351
AIFM1_HUMAN I	16740985	FRDA_SHEFR Fu	444188 CI	1M64	1M6I	1E39		0.93	Sc=6.43415, min distance = 2.412416
AIFM1_HUMAN I	446013 1,	O28603_ARCFU I	444188 CI	1JNR	1M6I	1JNZ		0.94	Sc=6.43692, min distance = 2.204610
AIFM1_HUMAN I	446013 1,	O28604_ARCFU I	444188 CI	1JNR	1M6I	1JNZ		0.94	Sc=6.4354, min distance = 2.2550957
AIFM1_HUMAN I	448054 CI	FRDA_SHEFR Fu	444188 CI	1M64	1M6I	1Y0P		0.97	Sc=6.43663, min distance = 2.456107
AIFM1_HUMAN I	449465 CI	GSHR_HUMAN Fu	444188 CI	3DK9	1M6I	3GRT		0.98	Sc=6.43415, min distance = 2.125440
AIFM1_HUMAN I	6420174 C	O28604_ARCFU I	444188 CI	1JNR	1M6I	2FJB		0.92	Sc=6.4469, min distance = 2.1690398
AIFM1_MOUSE I	11987634	GLPD_ECOLI Fu	444188 CI	2QCU	1GV4	2R4E		0.97	Sc=6.16011, min distance = 1.956745
AIFM1_MOUSE I	16740985	FRDA_SHEFR Fu	444188 CI	1M64	1GV4	1E39		0.93	Sc=6.43744, min distance = 2.377218
AIFM1_MOUSE I	444502 CI	FRDA_SHEFR Fu	444188 CI	1M64	1GV4	1QJD		0.98	Sc=6.15909, min distance = 2.400942
AIFM1_MOUSE I	446013 1,	O28603_ARCFU I	444188 CI	1JNR	1GV4	1JNZ		0.94	Sc=6.44027, min distance = 2.094517
AIFM1_MOUSE I	6420174 C	O28603_ARCFU I	444188 CI	1JNR	1GV4	2FJB		0.92	Sc=6.44905, min distance = 2.080704
AIFM1_MOUSE I	6420174 C	O28604_ARCFU I	444188 CI	1JNR	1GV4	2FJB		0.92	Sc=6.44883, min distance = 2.079913
AK1C1_HUMAN I	15942680	6PGD_LACLM Fu	5886 NADF	2IZ0	1MRQ	2IZ1			Sc=6.14145, min distance = 2.104644
AK1C1_HUMAN I	440141 9i	DHB1_HUMAN Fu	5886 NADF	1QYV	1MRQ	1QYW			Sc=6.44121, min distance = 2.044627
AK1C3_HUMAN I	15942680	6PGD_LACLM Fu	5886 NADF	2IZ0	1S1P	2IZ1			Sc=6.15447, min distance = 2.184379
AK1C3_HUMAN I	72099 flu	PGH2_MOUSE Fu	3715 indo	4COX	1S2A	3PGH			Sc=5.87132, min distance = 2.297516
AK1C4_HUMAN I	15942680	6PGD_LACLM Fu	5886 NADF	2IZ0	2FVL	2IZ1			Sc=6.14754, min distance = 2.067981
AK1CL_MOUSE I	15942680	6PGD_LACLM Fu	5886 NADF	2IZ0	2HEJ	2IZ1			Sc=6.15706, min distance = 2.321637
AK1D1_HUMAN I	13455857	IGKC_MOUSE Fu	23831 HEF	1P7K	3BUV	3C6S			Sc=5.61411, min distance = 1.151636
AK1D1_HUMAN I	15942680	6PGD_LACLM Fu	5886 NADF	2IZ0	3BUV	2IZ1			Sc=6.14754, min distance = 2.113649
AK1D1_HUMAN I	164795 N(	RBCMT_PEA Ful	23831 HEF	1MLV	3BUV	2H2J			Sc=5.63353, min distance = 1.496731
AK1D1_HUMAN I	222865 Ar	DHB1_HUMAN Fu	11987715	1JTV	3BUR	1QYW			Sc=5.98824, min distance = 2.337300
AK1D1_HUMAN I	222865 Ar	DHB1_HUMAN Fu	6128 andr	1QYX	3CAS	1QYW		0.77	Sc=6.01096, min distance = 2.299820
AK1D1_HUMAN I	245468 6-	DHB1_HUMAN Fu	11987715	1JTV	3BUR	1FDU			Sc=5.99704, min distance = 1.832955
AK1D1_HUMAN I	439710 rh	IGKC_MOUSE Fu	23831 HEF	1P7K	3BUV	3BZ4			Sc=5.60997, min distance = 2.232189
AK1D1_HUMAN I	440141 9i	DHB1_HUMAN Fu	5886 NADF	1QYV	3BUV	1QYW			Sc=6.43626, min distance = 2.019254
AK1D1_HUMAN I	445939 1g	PPTA_ECOLI Fu	23831 HEF	1GYX	3BUV	1GYY			Sc=5.77347, min distance = 2.243326
AK1_ARATH Fu	21945395	SETD7_HUMAN F	34756 Acy	1N6A	2CDQ	1XQH		0.88	Sc=6.00343, min distance = 2.575240
AK3_ECOLI Fu	5957 Ader	PURL_THEMA Fu	6022 Aden	2HRU	2J0W	2HS0		0.99	Sc=6.16317, min distance = 2.469400
AKT2_HUMAN F	11608401	IPKA_HUMAN Fu	11175137	2UW7	2UW9	2UVY			Sc=6.19838, min distance = 2.395854
AKT2_HUMAN F	11608401	KAPCA_BOVIN F	11175137	2UW7	2UW9	2UVY	44.86		Sc=6.20623, min distance = 2.362310
AKT2_HUMAN F	11608401	KAPCA_BOVIN F	11857236	3E8C	3E88	2UVY	44.86		Sc=6.21795, min distance = 2.049289
AKT2_HUMAN F	11696113	KAPCA_BOVIN F	11175137	2UW7	2UW9	2VO3	44.86		Sc=6.23085, min distance = 2.256017
AKT2_HUMAN F	11696113	KAPCA_BOVIN F	11857236	3E8C	3E88	2VO3	44.86		Sc=6.28925, min distance = 1.851773
AKT2_HUMAN F	11708454	KAPCA_BOVIN F	11175137	2UW7	2UW9	2VNW	44.86		Sc=6.15749, min distance = 2.250099

AKT2_HUMAN F	11708454	KAPCA_BOVIN F	11857236	3E8C	3E88	2VNW	44.86		Sc=6.17565, min distance = 2.020453
AKT2_HUMAN F	15602982	KAPCA_BOVIN F	11857236	3E8C	3E88	2UW6	44.86		Sc=6.0985, min distance = 2.0792991
AKT2_HUMAN F	15602983	KAPCA_BOVIN F	11175137	2UW7	2UW9	2UW5	44.86	0.92	Sc=6.13935, min distance = 2.687688
AKT2_HUMAN F	15602983	KAPCA_BOVIN F	11857236	3E8C	3E88	2UW5	44.86		Sc=6.08465, min distance = 2.168831
AKT2_HUMAN F	16122632	KAPCA_BOVIN F	11175137	2UW7	2UW9	2UVZ	44.86		Sc=6.35603, min distance = 2.415487
AKT2_HUMAN F	16122632	KAPCA_BOVIN F	11857236	3E8C	3E88	2UVZ	44.86		Sc=6.31667, min distance = 2.160376
AKT2_HUMAN F	16122632	KAPCA_BOVIN F	24963049	3E8E	3E8D	2UVZ	44.86		Sc=6.34131, min distance = 2.061922
AKT2_HUMAN F	16122633	IPKA_HUMAN Fu	11175137	2UW7	2UW9	2UW0			Sc=6.45483, min distance = 2.305843
AKT2_HUMAN F	16122633	KAPCA_BOVIN F	11175137	2UW7	2UW9	2UW0	44.86		Sc=6.46385, min distance = 2.370301
AKT2_HUMAN F	16122634	KAPCA_BOVIN F	11175137	2UW7	2UW9	2UW4	44.86	0.77	Sc=5.95528, min distance = 2.187563
AKT2_HUMAN F	16122635	KAPCA_BOVIN F	11175137	2UW7	2UW9	2UW8	44.86		Sc=5.72884, min distance = 2.715653
AKT2_HUMAN F	16122635	KAPCA_BOVIN F	11857236	3E8C	3E88	2UW8	44.86		Sc=5.74295, min distance = 1.186438
AKT2_HUMAN F	16122635	KAPCA_BOVIN F	24963049	3E8E	3E8D	2UW8	44.86		Sc=5.65697, min distance = 2.367325
AKT2_HUMAN F	16122642	KAPCA_BOVIN F	24963049	3E8E	3E8D	2UZW	44.86		Sc=6.39759, min distance = 1.922086
AKT2_HUMAN F	17751819	KAPCA_BOVIN F	11175137	2UW7	2UW9	2VO6	44.86		Sc=6.29397, min distance = 2.390028
AKT2_HUMAN F	17751819	KAPCA_BOVIN F	11857236	3E8C	3E88	2VO6	44.86		Sc=6.26075, min distance = 2.086427
AKT2_HUMAN F	24701731	KAPCA_BOVIN F	11175137	2UW7	2UW9	2VNY	44.86		Sc=6.10488, min distance = 2.292956
AKT2_HUMAN F	24762195	KAPCA_BOVIN F	11175137	2UW7	2UW9	2VO0	44.86		Sc=6.30293, min distance = 2.215672
AKT2_HUMAN F	3064778 H	ROCK1_HUMAN F	33113 gam	2V55	1O6L	2ETK	38.11		Sc=6.09235, min distance = 2.040300
AKT2_HUMAN F	3540 1yds	KAPCA_BOVIN F	11857236	3E8C	3E88	1YDS	44.86		Sc=5.90241, min distance = 1.981285
AKT2_HUMAN F	3540 1yds	KAPCA_BOVIN F	24963049	3E8E	3E8D	1YDS	44.86		Sc=5.91835, min distance = 1.970145
AKT2_HUMAN F	3547 Fasu	KAPCA_BOVIN F	11857236	3E8C	3E88	1Q8W	44.86		Sc=5.9594, min distance = 2.2885973
AKT2_HUMAN F	3547 Fasu	KAPCA_BOVIN F	24963049	3E8E	3E8D	1Q8W	44.86		Sc=5.98489, min distance = 2.185886
AKT2_HUMAN F	3547 Fasu	ROCK1_HUMAN F	33113 gam	2V55	1O6L	2ESM	38.11		Sc=5.96602, min distance = 1.977197
AKT2_HUMAN F	445479 CI	CSK2A_MAIZE F	33113 gam	1LP4	1O6L	1F0Q	26.21		Sc=6.27946, min distance = 2.017450
AKT2_HUMAN F	448042 2e	KAPCA_BOVIN F	11175137	2UW7	2UW9	1Q8T	44.86		Sc=6.01809, min distance = 2.317795
AKT2_HUMAN F	448042 2e	KAPCA_BOVIN F	11857236	3E8C	3E88	1Q8T	44.86		Sc=6.04838, min distance = 1.797898
AKT2_HUMAN F	448042 2e	KAPCA_BOVIN F	24963049	3E8E	3E8D	1Q8T	44.86		Sc=6.08984, min distance = 1.733348
AKT2_HUMAN F	448043 2g	KAPCA_BOVIN F	11857236	3E8C	3E88	1Q8U	44.86		Sc=6.01578, min distance = 2.222077
AKT2_HUMAN F	448043 2g	KAPCA_BOVIN F	24963049	3E8E	3E8D	1Q8U	44.86		Sc=6.04484, min distance = 2.256976
AKT2_HUMAN F	448043 2g	ROCK1_HUMAN F	33113 gam	2V55	1O6L	3D9V	38.11		Sc=6.01162, min distance = 2.037061
AKT2_HUMAN F	449240 1y	KAPCA_BOVIN F	11857236	3E8C	3E88	1YDR	44.86		Sc=5.96825, min distance = 2.079449
AKT2_HUMAN F	449240 1y	KAPCA_BOVIN F	24963049	3E8E	3E8D	1YDR	44.86		Sc=5.98489, min distance = 1.741211
AKT2_HUMAN F	5327109 2	KAPCA_BOVIN F	11175137	2UW7	2UW9	2C1A	44.86		Sc=6.14477, min distance = 2.047717
AKT2_HUMAN F	5327109 2	KAPCA_BOVIN F	11857236	3E8C	3E88	2C1A	44.86		Sc=6.11879, min distance = 2.252639
AKT2_HUMAN F	5327110 i	IPKA_HUMAN Fu	11175137	2UW7	2UW9	2C1B			Sc=6.14289, min distance = 2.137375
AKT2_HUMAN F	5327110 i	KAPCA_BOVIN F	11175137	2UW7	2UW9	2C1B	44.86		Sc=6.15316, min distance = 2.122157

AKT2_HUMAN F	5327121 I	PIM1_HUMAN Ful	33113 gam	1XR1	1O6L	2C3I	30.68		Sc=6.28937, min distance = 2.33218
AKT2_HUMAN F	5327148 C	IPKA_RABIT Ful	33113 gam	1CDK	1O6L	2ERZ			Sc=6.1787, min distance = 2.186804
AKT2_HUMAN F	5957 Aden	KAPCA_BOVIN F	11857236	3E8C	3E88	1Q24	44.86		Sc=5.67974, min distance = 2.10952
AKT2_HUMAN F	60961 ade	SKY1_YEAST Ful	33113 gam	1Q99	1O6L	1Q97	23.89	0.93	Sc=6.23274, min distance = 1.77067
AKT2_HUMAN F	657001 1s	KAPCA_BOVIN F	11857236	3E8C	3E88	1SVE	44.86		Sc=6.62031, min distance = 1.80641
AKT2_HUMAN F	657002 1s	KAPCA_BOVIN F	11857236	3E8C	3E88	1SVG	44.86		Sc=6.6209, min distance = 1.944464
AKT2_HUMAN F	6852207 M	IPKA_HUMAN Ful	11175137	2UW7	2UW9	2GNI			Sc=5.92379, min distance = 2.13223
AKT2_HUMAN F	6914614 p	IPKA_RABIT Ful	33113 gam	1CDK	1O6L	2F7Z			Sc=6.5026, min distance = 1.953825
AKT2_HUMAN F	91532 AMP	PURT_ECOLI Ful	33113 gam	1EYZ	1O6L	1KJI		0.99	Sc=5.99336, min distance = 2.41863
AK_METJA Ful	3044933 A	ENPL_CANFA Ful	33113 gam	2O1U	3C1M	1U0Y		0.81	Sc=6.42768, min distance = 1.89865
AK_METJA Ful	444564 AD	MYS2_DICDI Ful	33113 gam	1MMN	3C1M	1W9I		0.91	Sc=5.9934, min distance = 2.130344
AK_METJA Ful	448968 1u	HS90A_HUMAN F	6022 Aden	1BYQ	2HMF	1UY9			Sc=6.29281, min distance = 2.18125
AK_METJA Ful	448973 1u	HS90A_HUMAN F	6022 Aden	1BYQ	2HMF	1UYG			Sc=6.31247, min distance = 2.05387
AK_METJA Ful	448974 1u	HS90A_HUMAN F	6022 Aden	1BYQ	2HMF	1UYH			Sc=6.32785, min distance = 1.97695
AK_METJA Ful	448976 1u	HS90A_HUMAN F	6022 Aden	1BYQ	2HMF	1UYK			Sc=6.31602, min distance = 2.05870
AK_METJA Ful	5496922 C	HS90A_HUMAN F	6022 Aden	1BYQ	2HMF	1UY7			Sc=6.28728, min distance = 2.36620
AK_METJA Ful	5496923 C	HS90A_HUMAN F	6022 Aden	1BYQ	2HMF	1UY8			Sc=6.29353, min distance = 2.18635
AK_METJA Ful	5957 Aden	ARP3_BOVIN Ful	6022 Aden	2P9I	2HMF	1TYQ		0.99	Sc=6.00603, min distance = 2.32670
AK_METJA Ful	5957 Aden	HSP7F_YEAST F	6022 Aden	3C7N	2HMF	3D2F		0.99	Sc=6.46543, min distance = 1.72595
AK_METJA Ful	6852187 2	O57883_PYRHO	6022 Aden	1WNL	2HMF	2DTH		0.89	Sc=6.17555, min distance = 2.48226
AL1A1_SHEEP	6022 Aden	Q5SI02_THET8	5893 nadi	2BJK	1BXS	2BJA			Sc=6.1964, min distance = 2.132145
AL7A1_HUMAN	446006 CI	O57693_THETE	5893 nadi	1UXT	2J6L	1UXU			Sc=5.90133, min distance = 2.02183
ALBU_HUMAN F	10831270	TTHY_HUMAN Ful	3059 difl	3D2T	2BXE	3CN3			Sc=5.75563, min distance = 1.63299
ALBU_HUMAN F	1184681 C	TTHY_HUMAN Ful	3059 difl	3D2T	2BXE	3CN4			Sc=6.09755, min distance = 1.55441
ALBU_HUMAN F	119346 3,	TTHY_HUMAN Ful	8631 3,5-	3B56	2BXL	2GAB			Sc=5.64261, min distance = 2.02211
ALBU_HUMAN F	11957353	HBB_HORSE Ful	444124 HE	1Y8I	1O9X	1IWH		0.87	Sc=6.60007, min distance = 1.99215
ALBU_HUMAN F	11957355	CY1_BOVIN Ful	444098 HE	1L0N	1N5U	1L0L		0.77	Sc=6.69005, min distance = 2.34635
ALBU_HUMAN F	11957360	CY1_BOVIN Ful	444098 HE	1L0N	1N5U	1NTM			Sc=6.63235, min distance = 2.02033
ALBU_HUMAN F	11957363	HBA_HUMAN Ful	444124 HE	1NQP	1O9X	1RQA		0.81	Sc=6.58602, min distance = 2.24354
ALBU_HUMAN F	11957364	HMOX1_HUMAN F	444124 HE	1OZW	1O9X	1S13		0.86	Sc=6.41642, min distance = 1.99067
ALBU_HUMAN F	11957370	HMOX1_HUMAN F	444124 HE	1OZW	1O9X	1TWN			Sc=6.35457, min distance = 2.12972
ALBU_HUMAN F	11957371	HMOX1_HUMAN F	444124 HE	1OZW	1O9X	1TWR			Sc=6.3533, min distance = 2.431699
ALBU_HUMAN F	11957373	CATE_ECOLI Ful	444124 HE	1IPH	1O9X	1P81		0.79	Sc=6.59134, min distance = 2.57693
ALBU_HUMAN F	11957385	MYG_PHYCA Ful	444098 HE	1A6M	1N5U	2CMM			Sc=6.04136, min distance = 2.28148
ALBU_HUMAN F	14368760	LPXC_AQUAE Fu	11005 Tet	1P42	1N5U	2O3Z			Sc=6.01753, min distance = 0.89718
ALBU_HUMAN F	1493 2,4-	TTHY_HUMAN Ful	8631 3,5-	3B56	2BXL	2B15			Sc=5.77335, min distance = 2.28997
ALBU_HUMAN F	16741061	MYG_PHYCA Ful	444098 HE	1A6M	1N5U	1MBC		0.83	Sc=6.69005, min distance = 2.15993

ALBU_HUMAN F	16741062	MYG_PHYCA Ful	444124 HE	1U7R	1O9X	1MBN	0.87	Sc=6.60007, min distance = 2.24395
ALBU_HUMAN F	177880 2'	TTHY_HUMAN Fu	8631 3,5-	3B56	2BXL	2G9K		Sc=5.65312, min distance = 2.58603
ALBU_HUMAN F	23722944	TTHY_HUMAN Fu	3059 difl	3D2T	2BXE	2QGE		Sc=5.88284, min distance = 1.17934
ALBU_HUMAN F	23722944	TTHY_HUMAN Fu	8631 3,5-	3B56	2BXL	2QGE		Sc=5.73074, min distance = 2.36135
ALBU_HUMAN F	24893347	CATE_ECOLI Fu	444124 HE	1IPH	1O9X	1GGJ		Sc=6.5878, min distance = 2.075466
ALBU_HUMAN F	25011739	TTHY_HUMAN Fu	8631 3,5-	3B56	2BXL	3CN0		Sc=5.73044, min distance = 2.33515
ALBU_HUMAN F	3033 dicl	TTHY_HUMAN Fu	3059 difl	3D2T	2BXE	1DVX		Sc=6.15122, min distance = 0.58415
ALBU_HUMAN F	3033 dicl	TTHY_HUMAN Fu	8631 3,5-	3B56	2BXL	1DVX		Sc=6.02271, min distance = 2.31750
ALBU_HUMAN F	30951 alc	PGH1_SHEEP Fu	338 salic	1PTH	2I2Z	1HT8		Sc=5.8288, min distance = 2.391532
ALBU_HUMAN F	3371 fluf	TTHY_HUMAN Fu	3059 difl	3D2T	2BXE	1BM7		Sc=6.06153, min distance = 1.52780
ALBU_HUMAN F	4369477 D	TTHY_HUMAN Fu	8631 3,5-	3B56	2BXL	1U21		Sc=6.02677, min distance = 2.90085
ALBU_HUMAN F	444207 He	CP119_SULTO F	444124 HE	3B4X	1O9X	1UE8	0.99	Sc=6.59193, min distance = 2.02558
ALBU_HUMAN F	444207 He	PRXC_CALFU Fu	444124 HE	2CPO	1O9X	1CPO	0.99	Sc=6.58061, min distance = 2.60439
ALBU_HUMAN F	444522 HE	HBB_PAGBE Ful	444124 HE	1S5X	1O9X	1PBX	0.99	Sc=6.59485, min distance = 2.35542
ALBU_HUMAN F	444522 HE	MYG_PIG Full=I	444124 HE	1MYH	1O9X	1MNI	0.99	Sc=6.60007, min distance = 2.07595
ALBU_HUMAN F	445154 re	TTHY_HUMAN Fu	3059 difl	3D2T	2BXE	1DVS		Sc=6.08409, min distance = 1.43717
ALBU_HUMAN F	445155 O-	TTHY_HUMAN Fu	8631 3,5-	3B56	2BXL	1DVZ		Sc=6.0305, min distance = 2.391777
ALBU_HUMAN F	445302 AC	PGH1_SHEEP Fu	39912 ibu	1EQG	2BXG	1EBV		Sc=5.72421, min distance = 2.45880
ALBU_HUMAN F	445529 CI	TTHY_HUMAN Fu	3059 difl	3D2T	2BXE	1F86		Sc=5.74569, min distance = 1.96525
ALBU_HUMAN F	445638 pa	NLTP_MAIZE Fu	11005 Tet	1FK2	1N5U	1FK3	0.75	Sc=6.01162, min distance = 2.52981
ALBU_HUMAN F	445638 pa	NLTP_MAIZE Fu	2969 Deca	1FK0	2VDB	1FK3	0.75	Sc=6.02428, min distance = 1.48225
ALBU_HUMAN F	445638 pa	NLTP_MAIZE Fu	3893 laur	1FK1	1E7G	1FK3	0.75	Sc=5.97603, min distance = 2.06244
ALBU_HUMAN F	445641 Ri	NLTP_MAIZE Fu	11005 Tet	1FK2	1N5U	1FK7		Sc=6.21234, min distance = 2.07614
ALBU_HUMAN F	446189 CI	HBG1_HUMAN Fu	444124 HE	1I3E	1O9X	1I3D	0.89	Sc=6.59898, min distance = 2.31493
ALBU_HUMAN F	447739 CI	PA28_DABRR Fu	3715 indc	2OTH	2BXK	1OXL		Sc=6.13165, min distance = 2.45097
ALBU_HUMAN F	448537 di	TTHY_HUMAN Fu	3059 difl	3D2T	2BXE	1TT6		Sc=6.08387, min distance = 1.15205
ALBU_HUMAN F	448708 CI	TTHY_HUMAN Fu	3059 difl	3D2T	2BXE	1THC		Sc=6.3774, min distance = 1.715856
ALBU_HUMAN F	448734 CI	TTHY_HUMAN Fu	3059 difl	3D2T	2BXE	1TLM		Sc=6.09464, min distance = 1.54994
ALBU_HUMAN F	448734 CI	TTHY_HUMAN Fu	8631 3,5-	3B56	2BXL	1TLM		Sc=6.0291, min distance = 2.521293
ALBU_HUMAN F	5280934 l	NLTP_MAIZE Fu	11005 Tet	1FK2	1N5U	1FK6	0.75	Sc=6.07981, min distance = 1.10614
ALBU_HUMAN F	5287495 2	TTHY_HUMAN Fu	8631 3,5-	3B56	2BXL	2B77		Sc=5.97812, min distance = 2.82863
ALBU_HUMAN F	5327160 2	TTHY_HUMAN Fu	3059 difl	3D2T	2BXE	2F8I		Sc=6.08632, min distance = 1.42026
ALBU_HUMAN F	6031 Uric	LPXC_AQUAE Fu	11005 Tet	1P42	1N5U	2J65		Sc=6.28874, min distance = 2.04579
ALBU_HUMAN F	6420167 M	CPXA_PSEPU Fu	444124 HE	1RE9	1O9X	2FE6	0.89	Sc=6.58236, min distance = 2.20972
ALBU_HUMAN F	65552 Tet	TTHY_RAT Full=	5819 L-th	1IE4	1HK4	1KGI	0.87	Sc=6.10915, min distance = 0.80740
ALBU_HUMAN F	6914652 C	FHUA_ECOLI Fu	3893 laur	1QFG	1E7G	2GRX		Sc=5.74996, min distance = 1.03714
ALBU_HUMAN F	72099 flu	PGH2_MOUSE Fu	3715 indc	4COX	2BXK	3PGH		Sc=5.96698, min distance = 1.97480

ALBU_HUMAN F	72099 flu	TTHY_HUMAN Fu	3059 difl	3D2T	2BXE	1DVT		Sc=6.00232, min distance = 1.26749
ALBU_HUMAN F	8209 Myri	ABL1_MOUSE Fu	11005 Tet	1OPK	1N5U	1OPJ		Sc=5.76567, min distance = 0.95457
ALD2_MOUSE F	157839 Li	ALDR_HUMAN Fu	1613 Alon	2FZ8	1FRB	1Z3N		Sc=6.20662, min distance = 2.65331
ALD2_MOUSE F	15942680	6PGD_LACLM Fu	5886 NADP	2IZ0	1FRB	2IZ1		Sc=6.17177, min distance = 2.13251
ALDH1_ELEED	6022 Aden	Q5SI02_THET8	5893 nadi	2BJK	1O9J	2BJA		Sc=6.20512, min distance = 2.27378
ALDH2_BOVIN	6022 Aden	Q5SI02_THET8	5893 nadi	2BJK	1A4Z	2BJA		Sc=6.21483, min distance = 2.46522
ALDH2_HUMAN	16214828	CDK2_HUMAN Fu	6022 Aden	1GY3	1NZX	2UZO		Sc=5.84498, min distance = 2.24030
ALDH2_HUMAN	23653526	CDK2_HUMAN Fu	6022 Aden	1GY3	1NZX	2R3R		Sc=5.7457, min distance = 2.029377
ALDH2_HUMAN	4566 1h1s	CDK2_HUMAN Fu	6022 Aden	1GY3	1NZX	1H1S		Sc=6.16893, min distance = 2.30154
ALDH2_HUMAN	5288641 i	CDK2_HUMAN Fu	6022 Aden	1GY3	1NZX	1E9H		Sc=6.05359, min distance = 2.48839
ALDR_HORVU F	15942680	6PGD_LACLM Fu	5886 NADP	2IZ0	2BGS	2IZ1		Sc=6.19826, min distance = 2.18809
ALDR_HORVU F	440141 9i	DHB1_HUMAN Fu	5886 NADP	1QYV	2BGS	1QYW		Sc=6.45812, min distance = 2.04059
ALDR_HUMAN F	15942680	6PGD_LACLM Fu	5886 NADP	2IZ0	1US0	2IZ1		Sc=6.21836, min distance = 2.09910
ALDR_HUMAN F	445607 AT	ACON_BOVIN Fu	311 citri	1C96	1US0	1FGH		Sc=5.87949, min distance = 2.22804
ALDR_HUMAN F	51 2-Oxop	SERA_ECOLI Fu	311 citri	2P9E	1US0	1YBA	0.76	Sc=5.7414, min distance = 1.463511
ALF1_THETE F	107737 ir	SUHB_ARCFU Fu	10267 Har	1LBZ	1W8S	1LBX	0.75	Sc=6.09129, min distance = 1.37645
ALF1_THETE F	439427 Ro	CCPA_BACME Fu	10267 Har	2NZV	1W8S	2NZU	0.87	Sc=5.94815, min distance = 1.93704
ALF_ECOLI Fu	445225 CI	FUCA_ECOLI Fu	4797 1tpw	4FUA	1B57	1E47		Sc=5.73213, min distance = 2.09632
ALF_ECOLI Fu	754 3-Gly	FUCA_ECOLI Fu	4797 1tpw	4FUA	1B57	1E48		Sc=5.72286, min distance = 2.20622
ALF_HELPY Fu	439183 1i	TPIS_TRYBB Fu	4797 1tpw	1TTJ	3C52	1IIH		Sc=5.77693, min distance = 1.98413
ALF_HELPY Fu	445225 CI	FUCA_ECOLI Fu	4797 1tpw	4FUA	3C52	1E47		Sc=5.71367, min distance = 1.94317
ALF_HELPY Fu	754 3-Gly	FUCA_ECOLI Fu	4797 1tpw	4FUA	3C52	1E48		Sc=5.72075, min distance = 2.03226
ALKB2_HUMAN	3080614 C	HIF1N_HUMAN F	51 2-Oxop	1H2L	3BUC	1H2K		Sc=5.66767, min distance = 2.03297
ALKB2_HUMAN	3080614 C	JHD3A_HUMAN F	51 2-Oxop	2Q8C	3BUC	2OX0		Sc=5.69658, min distance = 2.22633
ALKB3_HUMAN	161166 L-	AADAT_HUMAN F	51 2-Oxop	3DC1	2IUW	2R2N		Sc=6.13326, min distance = 1.75872
ALKB3_HUMAN	3080614 C	JHD3A_HUMAN F	51 2-Oxop	2Q8C	2IUW	2OX0		Sc=5.64715, min distance = 2.29059
ALKB3_HUMAN	33032 L-g	DHE3_BOVIN Fu	51 2-Oxop	1HWY	2IUW	1HWX		Sc=5.67313, min distance = 2.44926
ALKB3_HUMAN	444591 CI	IDH_ECOLI Ful	51 2-Oxop	1CW4	2IUW	1BL5		Sc=5.65027, min distance = 2.21436
ALKB_ECOLI F	3080614 C	HIF1A_HUMAN F	51 2-Oxop	1H2L	3BKZ	1H2K		Sc=5.70487, min distance = 2.64304
ALKB_ECOLI F	3080614 C	HIF1N_HUMAN F	51 2-Oxop	1H2L	3BKZ	1H2K		Sc=5.70487, min distance = 2.65873
ALKB_ECOLI F	3080614 C	JHD3A_HUMAN F	51 2-Oxop	2Q8C	3BKZ	2OX0		Sc=5.6839, min distance = 2.174963
ALKB_ECOLI F	33032 L-g	DHE3_BOVIN Fu	51 2-Oxop	1HWY	3BKZ	1HWX		Sc=5.70422, min distance = 2.77054
ALKB_ECOLI F	444591 CI	IDH_ECOLI Ful	51 2-Oxop	1CW4	3BKZ	1BL5		Sc=5.67557, min distance = 2.42842
ALKH_ECOLI F	12136 Ber	POL_HV1N5 Ful	311 citri	2GON	1FQ0	9HVP		Sc=5.66951, min distance = 0.53876
ALKH_ECOLI F	12793 Phe	SRC_HUMAN Ful	311 citri	1O4L	1FQ0	1O4O		Sc=5.66817, min distance = 0.90686
ALKH_ECOLI F	444212 tr	ACON_BOVIN Fu	311 citri	1C96	1FQ0	1ACO		Sc=5.90001, min distance = 1.93583
ALKH_ECOLI F	447376 2-	ACON_BOVIN Fu	311 citri	1C96	1FQ0	1NIS		Sc=5.86907, min distance = 1.75612

ALKH_ECOLI Fu	447536 1d	SRC_HUMAN Ful	311 citri	1O4L	1FQ0	1O4Q			Sc=5.75831, min distance = 0.98078
AMPM1_HUMAN I	854023 Ep	Q8WSF8_APLCA I	23831 HEP	2BR7	2G6P	2BYQ			Sc=5.67938, min distance = 2.32529
AMPS2_RANPI I	447376 2-	ACON_BOVIN Ful	311 citri	1C96	2P6Z	1NIS			Sc=5.80152, min distance = 2.18413
AMPS2_RANPI I	51 2-Oxop	SERA_ECOLI Ful	311 citri	2P9E	2P6Z	1YBA		0.76	Sc=5.87183, min distance = 0.95358
AMPX_VIBPR Fu	11987881	AMPL_BOVIN Ful	445715 1l	1LCP	3B3C	2J9A			Sc=5.96826, min distance = 2.04632
AMT4_PSEST Fu	444139 CI	CDGT2_BACCI Fu	79025 alp	1D3C	1JDC	1DTU	24.78		Sc=5.80567, min distance = 0.38183
AMT4_PSEST Fu	446564 LI	BACR_HALSA Ful	79025 alp	1DZE	1JDC	1JV7			Sc=5.79693, min distance = 0.19782
AMT4_PSEST Fu	446685 Va	CDGT2_BACCI Fu	79025 alp	1D3C	1JDC	1KCK	24.78		Sc=5.95806, min distance = 0.93200
AMT4_PSEST Fu	447600 CI	AMY1_HORVU Ful	79025 alp	1RP8	1JDC	1P6W	30.26	0.83	Sc=5.75584, min distance = 1.07982
AMT4_PSEST Fu	447607 1d	PYGM_RABIT Ful	79025 alp	1H5U	1JDC	2G9V			Sc=5.69101, min distance = 2.47150
AMT6_BACS7 Fu	445237 1,	AMY1_HORVU Ful	444440 AC	1RP9	2GJP	1P6W	27.72		Sc=5.62251, min distance = 2.18669
AMTB_ECOLI Fu	155448 B-	GLPG_ECOLI Ful	444279 1s	2IRV	3C1H	3B44			Sc=5.76886, min distance = 0.95247
AMTB_ECOLI Fu	4369597 C	MK14_HUMAN Ful	62852 B-C	2FST	1U7G	2FSO			Sc=6.1869, min distance = 2.380017
AMY1_HORVU Fu	193758 Va	CDGT2_BACCI Fu	79025 alp	1D3C	1RP8	1DTU	24.63		Sc=5.88391, min distance = 0.90200
AMY1_HORVU Fu	439692 D-	AMYB_BACCE Ful	79025 alp	1VEM	1RP8	1J10			Sc=5.70741, min distance = 1.05666
AMY1_HORVU Fu	444139 CI	AMYP_PIG Full	79025 alp	1HX0	1RP8	1PIG			Sc=5.65947, min distance = 0.84982
AMY1_HORVU Fu	444139 CI	CDGT1_BACCI Fu	79025 alp	1CGU	1RP8	6CGT	22.83		Sc=5.70271, min distance = 0.66368
AMY1_HORVU Fu	444139 CI	CDGT2_BACCI Fu	79025 alp	1D3C	1RP8	1DTU	24.63		Sc=5.84043, min distance = 0.68888
AMY1_HORVU Fu	444140 CI	CDGT_THETU Ful	79025 alp	3BMW	1RP8	1A47			Sc=5.78081, min distance = 1.21163
AMY1_HORVU Fu	446757 CI	IGKC_MOUSE Ful	79025 alp	3BZ4	1RP8	1KNO			Sc=5.84879, min distance = 1.79385
AMY1_HORVU Fu	448009 TF	AMY1_HUMAN Ful	79025 alp	3DHP	1RP8	1Q4N			Sc=5.72842, min distance = 1.46187
AMY1_HORVU Fu	449571 CI	CDGT1_BACCI Fu	79025 alp	1CGU	1RP8	6CGT	22.83		Sc=5.93933, min distance = 0.48500
AMY1_HORVU Fu	5327083 C	GUN2_THEFU Ful	64689 bet	2BOF	1RP9	2BOD			Sc=5.74199, min distance = 0.79340
AMY1_HORVU Fu	82313 1rd	Q9HYN5_PSEAE I	64689 bet	1W8F	1RP9	1W8H			Sc=5.88163, min distance = 2.13848
AMY1_HORVU Fu	892 myo-i	SFTPD_HUMAN Fu	79025 alp	1PWB	1RP8	2OS9			Sc=5.88942, min distance = 2.17484
AMY1_HUMAN Fu	446685 Va	NEPU1_THEVU Fu	79025 alp	1UH4	3DHP	1UH3			Sc=5.93419, min distance = 1.64919
AMYA1_ASPOR I	29435 1-D	CDGT_BACS0 Ful	64689 bet	1UKS	2GVY	1I75	27.74		Sc=5.75727, min distance = 2.11785
AMYA1_ASPOR I	445999 CI	Q79G13_MYCTU I	64689 bet	1UP0	2GVY	1UOZ		0.77	Sc=5.61221, min distance = 2.25232
AMYA1_ASPOR I	447601 CI	GUX6_HUMIN Ful	64689 bet	1OCN	2GVY	1OCB			Sc=5.63307, min distance = 2.55195
AMYA1_ASPOR I	447607 1d	Q79G13_MYCTU I	64689 bet	1UP0	2GVY	1UP2			Sc=5.68216, min distance = 2.34309
AMYA1_ASPOR I	6027 xylo	Q8XXK6_RALSO I	64689 bet	2BS5	2GVY	2BS6		0.93	Sc=5.7838, min distance = 0.519023
AMYA1_ASPOR I	82313 1rd	Q9HYN5_PSEAE I	64689 bet	1W8F	2GVY	1W8H			Sc=5.96856, min distance = 1.47330
AMYB_BACCE Fu	124823 6g	PYGM_RABIT Ful	79025 alp	1H5U	1VEM	6GPB		0.78	Sc=6.05563, min distance = 2.14515
AMYB_BACCE Fu	193758 Va	AMY1_HUMAN Ful	79025 alp	3DHP	1VEM	1MFU			Sc=5.98061, min distance = 1.02112
AMYB_BACCE Fu	439357 ga	FCN2_HUMAN Ful	64960 Pol	2J0Y	1VEN	2J3U		0.89	Sc=5.72108, min distance = 0.97059
AMYB_BACCE Fu	444139 CI	CDGT2_BACCI Fu	79025 alp	1D3C	1VEM	1DTU	37.84		Sc=5.91052, min distance = 0.56517
AMYB_BACCE Fu	444175 CI	HBSAG_HBVD3 Fu	6255 malt	1IUD	1B9Z	1A7L		0.94	Sc=6.33071, min distance = 2.24498

AMYB_BACCE F	444175 CI	MALE_ECOLI Fu	6255 malt	1IUD	1B9Z	1A7L		0.94	Sc=6.33071, min distance = 2.24498
AMYB_BACCE F	444175 CI	MALE_ECOLI Fu	64960 Pol	1LAX	1VEN	1A7L			Sc=6.26211, min distance = 2.07415
AMYB_BACCE F	444685 2'	AMYB_SOYBN Fu	79025 alp	1V3H	1VEM	1BYD		0.87	Sc=6.35446, min distance = 2.30182
AMYB_BACCE F	444915 Gl	CDGT2_BACCI F	79025 alp	1D3C	1VEM	1CXL	37.84	0.85	Sc=5.82255, min distance = 0.81356
AMYB_BACCE F	446000 CI	AMYP_PIG Full	79025 alp	1HX0	1VEM	1JFH		0.83	Sc=5.70362, min distance = 1.91238
AMYB_BACCE F	446685 Va	CDGT2_BACCI F	79025 alp	1D3C	1VEM	1KCK	37.84		Sc=5.78719, min distance = 1.44683
AMYB_BACCE F	447139 CI	MALE_ECOLI Fu	6255 malt	1IUD	1B9Z	1MDQ		0.91	Sc=6.28496, min distance = 2.50742
AMYB_BACCE F	447607 1c	PYGM_RABIT Fu	79025 alp	1H5U	1VEM	2G9V			Sc=5.61263, min distance = 2.72559
AMYB_BACCE F	448009 TF	AMY1_HUMAN Fu	79025 alp	3DHP	1VEM	1Q4N			Sc=5.72372, min distance = 2.12658
AMYB_BACCE F	449554 CI	PYGM_RABIT Fu	79025 alp	1H5U	1VEM	5GPB			Sc=6.03367, min distance = 2.05750
AMYB_BACCE F	5780 D-Sc	TRFL_BOVIN Fu	79025 alp	2DWJ	1VEM	2DXR		0.78	Sc=5.78599, min distance = 2.25572
AMYB_BACCE F	6027 xylc	Q9ZB17_9LACT	79025 alp	1NSZ	1VEM	1MN0		0.93	Sc=5.61389, min distance = 1.91995
AMYB_BACCE F	7048614 Z	XYLA_STRRU Fu	79025 alp	1XIF	1VEM	1XID			Sc=5.76203, min distance = 2.22345
AMYB_HORVU F	444139 CI	CDGT2_BACCI F	79025 alp	1D3C	1B1Y	1DTU			Sc=5.81381, min distance = 0.67248
AMYB_HORVU F	444685 2'	AMYB_SOYBN Fu	79025 alp	1V3H	1B1Y	1BYD		0.87	Sc=6.30448, min distance = 2.32595
AMYB_HORVU F	446685 Va	NEPU1_THEVU F	79025 alp	1UH4	1B1Y	1UH3			Sc=5.83554, min distance = 2.01962
AMYB_HORVU F	448009 TF	AMY1_HUMAN Fu	79025 alp	3DHP	1B1Y	1Q4N			Sc=5.64982, min distance = 2.57560
AMYB_HORVU F	449571 CI	CDGT1_BACCI F	79025 alp	1CGU	1B1Y	6CGT			Sc=5.64122, min distance = 2.22689
AMYG_ASPAW F	54445 cas	Q2PS28_9PSED	29435 1-D	2PWD	1DOG	2PWG		0.75	Sc=5.92763, min distance = 2.14310
AMYG_ASPAW F	64689 bet	Q2PS28_9PSED	29435 1-D	2PWD	1DOG	2PWF			Sc=5.87651, min distance = 2.30740
AMYM_BACST F	79025 alp	CDGT2_BACCI F	439341 ma	1KCL	1QHO	1D3C	45.8	0.87	Sc=5.90855, min distance = 0.77257
AMYP_HUMAN F	4369386 A	AMY1_HUMAN Fu	193758 Va	1MFU	1CPU	1Z32			Sc=5.63281, min distance = 0.71603
AMYP_HUMAN F	444140 CI	CDGT_THETU Fu	193758 Va	3BMW	1CPU	1A47	23.88		Sc=5.68793, min distance = 2.02821
AMYP_HUMAN F	446685 Va	CDGT2_BACCI F	79025 alp	1D3C	3CPU	1KCK	23.96		Sc=5.93408, min distance = 1.15080
AMYP_HUMAN F	448009 TF	AMY1_HUMAN Fu	193758 Va	1MFU	1CPU	1Q4N			Sc=5.70416, min distance = 2.69461
AMYP_HUMAN F	448009 TF	AMY1_HUMAN Fu	444139 CI	3DHP	1CPU	1Q4N			Sc=5.69139, min distance = 2.61599
AMYP_PIG Ful	446685 Va	CDGT2_BACCI F	193758 Va	1DTU	1PIG	1KCK	27.63		Sc=5.73948, min distance = 2.01919
AMYP_PIG Ful	446685 Va	NEPU1_THEVU F	79025 alp	1UH4	1HX0	1UH3			Sc=5.73805, min distance = 2.61204
AMYP_PIG Ful	448009 TF	AMY1_HUMAN Fu	193758 Va	1MFU	1PIG	1Q4N			Sc=5.67106, min distance = 2.80331
AMYP_PIG Ful	448009 TF	AMY1_HUMAN Fu	444139 CI	3DHP	1PIG	1Q4N			Sc=5.67355, min distance = 2.88331
AMYP_PIG Ful	448009 TF	AMY1_HUMAN Fu	79025 alp	3DHP	1HX0	1Q4N			Sc=5.70416, min distance = 2.74001
AMYS_NEIPO F	125409 be	XYNA1_CLOSR F	64689 bet	1OD3	1JG9	1UY4		0.93	Sc=5.84717, min distance = 1.06343
AMYS_NEIPO F	16058615	LYSC_CHICK Fu	5988 sucr	1JJ0	1JGI	2H9J			Sc=5.7192, min distance = 2.654052
AMYS_NEIPO F	164795 N(	RBCMT_PEA Ful	23831 HEF	1MLV	1G5A	2H2J			Sc=5.6862, min distance = 2.380447
AMYS_NEIPO F	24139 ace	LYSC_CHICK Fu	5988 sucr	1JJ0	1JGI	1LZB			Sc=5.97562, min distance = 2.09993
AMYS_NEIPO F	29435 1-D	Q2PS28_9PSED	64689 bet	2PWF	1JG9	2PWD	30.77		Sc=5.90153, min distance = 1.58033
AMYS_NEIPO F	378 4-nit	PPAL_YEAST Fu	23831 HEF	1D1P	1G5A	1D1Q			Sc=5.83805, min distance = 1.91940

AMYS_NEIPO Fu	440115 N-	SFTPD_HUMAN Fu	79025 alp	1PWB	1MVY	2ORJ			Sc=5.87394, min distance = 2.77096(
AMYS_NEIPO Fu	444139 CI	CDGT2_BACCI Fu	79025 alp	1D3C	1MVY	1DTU	32.26		Sc=5.82491, min distance = 0.33277:
AMYS_NEIPO Fu	444915 Gl	CDGT2_BACCI Fu	79025 alp	1D3C	1MVY	1CXL	32.26	0.85	Sc=5.78587, min distance = 1.22605(
AMYS_NEIPO Fu	445238 O1	GUN2_THEFU Fu:	64689 bet	2BOF	1JG9	2BOG		0.94	Sc=5.88632, min distance = 2.313528
AMYS_NEIPO Fu	445318 n-	ECOT_ECOLI Fu:	79025 alp	1ECY	1MVY	1ECZ		0.85	Sc=6.27546, min distance = 0.67014‹
AMYS_NEIPO Fu	445999 CI	Q79G13_MYCTU l	64689 bet	1UP0	1JG9	1UOZ		0.77	Sc=5.67342, min distance = 2.04530!
AMYS_NEIPO Fu	447026 NA	LYSC_CHICK Fu:	5988 sucr	1JJ0	1JGI	1LZG			Sc=5.98285, min distance = 2.01475!
AMYS_NEIPO Fu	447600 CI	AMY1_HORVU Fu:	79025 alp	1RP8	1MVY	1P6W		0.83	Sc=5.62916, min distance = 1.23601:
AMYS_NEIPO Fu	447607 1c	Q79G13_MYCTU l	64689 bet	1UP0	1JG9	1UP2			Sc=5.93486, min distance = 1.02269(
AMYS_NEIPO Fu	54445 cas	Q2PS28_9PSED l	64689 bet	2PWF	1JG9	2PWG	30.77		Sc=6.09467, min distance = 0.78615(
AMYS_NEIPO Fu	6027 xylc	O93715_SULSO l	64689 bet	2CDB	1JG9	2CDC		0.93	Sc=5.63483, min distance = 2.36959:
AMYS_NEIPO Fu	6027 xylc	Q8XXK6_RALSO l	64689 bet	2BS5	1JG9	2BS6		0.93	Sc=5.66739, min distance = 2.43160:
AMYS_NEIPO Fu	6102767 C	Q9RX51_DEIRA l	64689 bet	2BY0	1JG9	2BY3	29.49		Sc=5.8976, min distance = 2.430910!
AMYS_NEIPO Fu	62551 Per	Q8WSF8_APLCA l	23831 HEF	2BR7	1G5A	2BYN			Sc=5.68142, min distance = 2.42036:
AMYS_NEIPO Fu	656981 CU	LYSC_CHICK Fu:	5988 sucr	1JJ0	1JGI	1YIL			Sc=5.86611, min distance = 2.55672:
AMY_BACAM Fu:	444440 AC	AMY1_HORVU Fu:	79025 alp	1RP8	1E3Z	1RP9			Sc=5.8924, min distance = 2.490621(
AMY_BACAM Fu:	444915 Gl	CDGT2_BACCI Fu	79025 alp	1D3C	1E3Z	1CXL		0.85	Sc=5.80266, min distance = 1.12795‹
AMY_BACAM Fu:	445237 1,	AMY1_HORVU Fu:	79025 alp	1RP8	1E3Z	1P6W		0.77	Sc=5.95114, min distance = 0.77897(
AMY_BACAM Fu:	446685 Va	CDGT2_BACCI Fu	79025 alp	1D3C	1E3Z	1KCK			Sc=5.86523, min distance = 0.847498
AMY_BACAM Fu:	446685 Va	NEPU1_THEVU Fu	79025 alp	1UH4	1E3Z	1UH3			Sc=5.89118, min distance = 1.01860!
AMY_BACLI Fu:	444915 Gl	CDGT2_BACCI Fu	79025 alp	1D3C	1E3Z	1CXL		0.85	Sc=5.80266, min distance = 1.12795‹
AMY_BACLI Fu:	446685 Va	CDGT2_BACCI Fu	79025 alp	1D3C	1E3Z	1KCK			Sc=5.86523, min distance = 0.847498
AMY_BACLI Fu:	446685 Va	NEPU1_THEVU Fu	79025 alp	1UH4	1E3Z	1UH3			Sc=5.89118, min distance = 1.01860!
AMY_BACSU Fu:	444140 CI	CDGT_THETU Fu:	193758 Va	3BMW	1UA7	1A47			Sc=5.70907, min distance = 2.48965:
AMY_BACSU Fu:	444140 CI	CDGT_THETU Fu:	79025 alp	3BMW	1UA7	1A47			Sc=5.6049, min distance = 2.3113288
AMY_BACSU Fu:	445237 1,	AMY1_HORVU Fu:	79025 alp	1RP8	1UA7	1P6W		0.77	Sc=5.91072, min distance = 0.96879:
AMY_BACSU Fu:	446685 Va	CDGT2_BACCI Fu	193758 Va	1DTU	1UA7	1KCK			Sc=5.77061, min distance = 2.69099‹
AMY_PSEHA Fu:	125409 be	AMYB_BACCE Fu:	79025 alp	1VEM	1G94	1J10		0.93	Sc=5.74014, min distance = 1.04382!
AMY_PSEHA Fu:	444139 CI	CDGT2_BACCI Fu	79025 alp	1D3C	1G94	1DTU	23.24		Sc=5.82415, min distance = 0.84301:
AMY_PSEHA Fu:	445237 1,	AMY1_HORVU Fu:	444440 AC	1RP9	1G94	1P6W			Sc=5.64084, min distance = 2.30347(
AMY_PSEHA Fu:	448009 TF	AMY1_HUMAN Fu:	79025 alp	3DHP	1G94	1Q4N			Sc=5.70162, min distance = 2.68538:
ANDR_HUMAN Fu	1184681 C	TTHY_HUMAN Fu:	3371 fluf	1BM7	2PIX	3CN4			Sc=5.97472, min distance = 1.17760(
ANDR_HUMAN Fu	11987726	SHBG_HUMAN Fu:	10635 And	1D2S	1T65	1LHV			Sc=6.09276, min distance = 2.45051:
ANDR_HUMAN Fu	11987785	PRGR_HUMAN Fu:	261000 Me	1E3K	1XOW	1SQN	55.69	0.91	Sc=6.11254, min distance = 2.07079'
ANDR_HUMAN Fu	242332 3k	SHBG_HUMAN Fu:	10635 And	1D2S	1T65	1LHO		0.85	Sc=5.99752, min distance = 2.57556(
ANDR_HUMAN Fu	4369524 T	PRGR_HUMAN Fu:	261000 Me	1E3K	1XOW	1ZUC	55.69		Sc=6.02822, min distance = 1.89553'
ANDR_HUMAN Fu	446934 CH	SHBG_HUMAN Fu:	10635 And	1D2S	1T65	1LHN		0.85	Sc=5.99889, min distance = 3.04724:

ANDR_HUMAN Fu	5757 estr	SHBG_HUMAN Ful	10635 And	1D2S	1T65	1LHU			Sc=5.95698, min distance = 2.26287
ANDR_HUMAN Fu	5994 prog	PRGR_HUMAN Ful	261000 Me	1E3K	1XOW	1A28	55.69		Sc=6.13651, min distance = 1.77860
ANDR_MOUSE Fu	11560224	ANDR_HUMAN Ful	10635 And	1T65	2QPY	2HVC			Sc=6.02133, min distance = 2.09447
ANDR_MOUSE Fu	11987726	SHBG_HUMAN Ful	10635 And	1D2S	2QPY	1LHV			Sc=6.10344, min distance = 2.49537
ANDR_MOUSE Fu	242332 3b	SHBG_HUMAN Ful	10635 And	1D2S	2QPY	1LHO		0.85	Sc=6.00178, min distance = 3.03035
ANDR_MOUSE Fu	261000 Me	ANDR_HUMAN Ful	10635 And	1T65	2QPY	1XOW			Sc=6.02661, min distance = 2.75408
ANDR_MOUSE Fu	446188 CI	ANDR_HUMAN Ful	10635 And	1T65	2QPY	2AM9			Sc=5.99752, min distance = 2.75324
ANDR_MOUSE Fu	446934 CH	SHBG_HUMAN Ful	10635 And	1D2S	2QPY	1LHN		0.85	Sc=5.99573, min distance = 2.91162
ANDR_MOUSE Fu	5757 estr	SHBG_HUMAN Ful	10635 And	1D2S	2QPY	1LHU			Sc=5.95869, min distance = 2.57561
ANDR_MOUSE Fu	66414 Par	SHBG_HUMAN Ful	10635 And	1D2S	2QPY	1LHW			Sc=6.02051, min distance = 2.59742
ANDR_MOUSE Fu	9880 Andr	ANDR_HUMAN Ful	10635 And	1T65	2QPY	2OZ7			Sc=6.31924, min distance = 2.13576
ANDR_PANTR Fu	11560224	ANDR_HUMAN Ful	10635 And	1T65	1T7R	2HVC			Sc=6.04164, min distance = 2.42581
ANDR_PANTR Fu	11987726	SHBG_HUMAN Ful	10635 And	1D2S	1T7R	1LHV			Sc=6.11167, min distance = 2.38914
ANDR_PANTR Fu	242332 3b	SHBG_HUMAN Ful	10635 And	1D2S	1T7R	1LHO		0.85	Sc=6.02661, min distance = 2.51668
ANDR_PANTR Fu	261000 Me	ANDR_HUMAN Ful	10635 And	1T65	1T7R	1XOW			Sc=6.04229, min distance = 2.64676
ANDR_PANTR Fu	31378 flu	ANDR_HUMAN Ful	10635 And	1T65	1T7R	1GS4			Sc=6.45185, min distance = 2.32560
ANDR_PANTR Fu	3371 fluf	ANDR_HUMAN Ful	10635 And	1T65	1T7R	2PIX			Sc=6.04312, min distance = 2.22066
ANDR_PANTR Fu	446188 CI	ANDR_HUMAN Ful	10635 And	1T65	1T7R	2AM9			Sc=6.01791, min distance = 2.64117
ANDR_PANTR Fu	446934 CH	SHBG_HUMAN Ful	10635 And	1D2S	1T7R	1LHN		0.85	Sc=6.03047, min distance = 3.01160
ANDR_PANTR Fu	5757 estr	SHBG_HUMAN Ful	10635 And	1D2S	1T7R	1LHU			Sc=5.97902, min distance = 2.18923
ANDR_PANTR Fu	66414 Par	SHBG_HUMAN Ful	10635 And	1D2S	1T7R	1LHW			Sc=6.0343, min distance = 2.279204
ANM1_RAT Ful	188380 Ad	MCES_ENCCU Ful	439155 Ad	1RI1	1OR8	1Z3C		0.91	Sc=6.49843, min distance = 2.06687
ANM1_RAT Ful	446535 CI	HNMT_HUMAN Ful	439155 Ad	2AOT	1OR8	1JQE		0.95	Sc=6.46311, min distance = 2.53782
ANM1_RAT Ful	60961 ade	PIMT_PYRFU Ful	439155 Ad	1JG1	1OR8	1JG2		0.84	Sc=6.19103, min distance = 2.64076
ANM3_HUMAN Fu	188380 Ad	MCES_ENCCU Ful	439155 Ad	1RI1	2FYT	1Z3C		0.91	Sc=6.56303, min distance = 2.47590
ANM3_HUMAN Fu	446535 CI	HNMT_HUMAN Ful	439155 Ad	2AOT	2FYT	1JQE		0.95	Sc=6.51919, min distance = 2.80130
ANM3_HUMAN Fu	60961 ade	PIMT_PYRFU Ful	439155 Ad	1JG1	2FYT	1JG2		0.84	Sc=6.30224, min distance = 2.65560
ANM3_HUMAN Fu	65482 sir	ERM_BACSU Ful	439155 Ad	1QAN	2FYT	1QAQ		0.88	Sc=6.57185, min distance = 2.11155
ANM3_HUMAN Fu	65482 sir	MCES_ENCCU Ful	439155 Ad	1RI1	2FYT	2HV9		0.88	Sc=6.10353, min distance = 2.40904
ANM3_RAT Ful	446535 CI	HNMT_HUMAN Ful	439155 Ad	2AOT	1F3L	1JQE		0.95	Sc=6.50668, min distance = 2.58253
ANM3_RAT Ful	60961 ade	PIMT_PYRFU Ful	439155 Ad	1JG1	1F3L	1JG2		0.84	Sc=6.30021, min distance = 2.73407
ANM3_RAT Ful	65482 sir	MCES_ENCCU Ful	439155 Ad	1RI1	1F3L	2HV9		0.88	Sc=6.09493, min distance = 2.44800
ANT3_HUMAN Fu	5354052 p	MIF_HUMAN Ful	311 citri	1GD0	2ZNH	2OOZ			Sc=5.64244, min distance = 0.45030
ANT3_HUMAN Fu	6102777 2	AROQ_HELPY Ful	311 citri	2C4V	2ZNH	2C4W			Sc=5.91836, min distance = 2.05315
AOFA_RAT Ful	5280953 H	AOFA_HUMAN Ful	5326701 C	2BXR	1O5W	2Z5Y			Sc=5.89529, min distance = 2.12869
AOFB_HUMAN Fu	1150 tryp	AAUB_ALCFA Ful	29147 4-N	2HJ4	2C70	2AGY			Sc=5.76911, min distance = 2.58075
AOFB_HUMAN Fu	11963950	FMS1_YEAST Ful	444188 CI	1RSG	2V5Z	3BI4	45.83	0.99	Sc=6.89573, min distance = 1.80812

AOFB_HUMAN F	241868 4-	AAUA_ALCFA Fu	29147 4-N	2HJ4	2C70	2HKR			Sc=5.70006, min distance = 1.92236
AOFB_HUMAN F	241868 4-	AAUB_ALCFA Fu	29147 4-N	2HJ4	2C70	2HKR			Sc=5.71163, min distance = 1.85504
AOSL_PLEHO F	11957353	HBB_HORSE Ful	444098 HE	2D5X	1U5U	1IWH		0.83	Sc=6.80259, min distance = 2.09602
AOSL_PLEHO F	11957355	CY1_BOVIN Ful	444098 HE	1L0N	1U5U	1L0L		0.77	Sc=6.81003, min distance = 2.05942
AOSL_PLEHO F	11957360	CY1_BOVIN Ful	444098 HE	1L0N	1U5U	1NTM			Sc=6.80697, min distance = 2.19187
AOSL_PLEHO F	11957361	CY1_BOVIN Ful	444098 HE	1L0N	1U5U	1NTZ		0.8	Sc=6.80855, min distance = 2.03115
AOSL_PLEHO F	11957385	MYG_PHYCA Ful	444098 HE	1A6M	1U5U	2CMM			Sc=6.31032, min distance = 2.16477
AOXB_ALCFA F	5326487 c	FDHF_ECOLI Fu	4369050 F	2IV2	1G8J	1FDO	20.79	0.98	Sc=6.81521, min distance = 2.19012
AP4A_CAEEL F	449190 1x	PDE4B_HUMAN F	6083 aden	1ROR	1KTG	1XM6			Sc=5.94217, min distance = 2.27648
AP4A_CAEEL F	449193 Ro	PDE4B_HUMAN F	6083 aden	1ROR	1KTG	1XMU			Sc=6.05711, min distance = 1.89075
AP4A_CAEEL F	449193 Ro	PDE4D_HUMAN F	6083 aden	1TB7	1KTG	1XOQ			Sc=6.07953, min distance = 1.84569
AP4A_CAEEL F	6022 Aden	PURP_METJA Fu	6083 aden	2R7M	1KTG	2R7N		0.99	Sc=5.89012, min distance = 2.36998
AP4A_CAEEL F	6076 Cycl	PDE10_HUMAN F	6083 aden	2OUN	1KTG	2OUR		0.99	Sc=6.16543, min distance = 2.35086
AP4A_CAEEL F	656966 1y	PDE4D_HUMAN F	6083 aden	1TB7	1KTG	1Y2E			Sc=6.04966, min distance = 1.79520
AP4A_CAEEL F	656968 1y	PDE4B_HUMAN F	6083 aden	1ROR	1KTG	1Y2H			Sc=5.94712, min distance = 2.03668
APAF_HUMAN F	188966 dA	PAPS1_HUMAN F	6022 Aden	1X6V	1Z6T	2PEZ		0.98	Sc=5.93617, min distance = 2.36860
APAF_HUMAN F	444564 AD	BIOD_ECOLI Fu	6022 Aden	1DAD	1Z6T	1BS1		0.91	Sc=5.70698, min distance = 2.53320
APAF_HUMAN F	444564 AD	MYS2_DICDI Fu	6022 Aden	1VOM	1Z6T	1W9I		0.91	Sc=5.97862, min distance = 2.14128
APAF_HUMAN F	4565 1h1r	CDK2_HUMAN Fu	6022 Aden	1GY3	1Z6T	1H1R			Sc=6.40101, min distance = 1.42188
APAF_HUMAN F	5957 Aden	BIOD_ECOLI Fu	6022 Aden	1DAD	1Z6T	1A82		0.99	Sc=5.77525, min distance = 2.64255
APAF_HUMAN F	5957 Aden	MALK_ECOLI Fu	6022 Aden	2AWN	1Z6T	1Q12		0.99	Sc=6.52683, min distance = 0.59741
APAF_HUMAN F	5957 Aden	NIFH1_AZOVI F	6022 Aden	1FP6	1Z6T	2C8V		0.99	Sc=6.06813, min distance = 1.74678
APAF_HUMAN F	5957 Aden	RK_BOVIN Full	6022 Aden	3C4Z	1Z6T	3C4W		0.99	Sc=6.04171, min distance = 2.07033
APAF_HUMAN F	6031 Uric	ECX1_PYRAB Fu	6022 Aden	2PO0	1Z6T	2PNZ			Sc=5.85201, min distance = 2.39027
APAF_HUMAN F	91532 AM	BIOD_ECOLI Fu	6022 Aden	1DAD	1Z6T	1DAG		0.99	Sc=5.78966, min distance = 2.38379
APAF_HUMAN F	91532 AM	KIF1A_MOUSE F	6022 Aden	1I5S	1Z6T	1I6I		0.99	Sc=6.52788, min distance = 2.39723
APAF_HUMAN F	91532 AM	MTNK_BACSU Fu	6022 Aden	2OLC	1Z6T	2PUL		0.99	Sc=6.07666, min distance = 1.98250
APAF_HUMAN F	91532 AM	NIFH1_AZOVI F	6022 Aden	1FP6	1Z6T	2AFK		0.99	Sc=6.09252, min distance = 2.17854
APEH_AERPE F	134497 4-	APX_STRGR Ful	6140 L-ph	1F2P	2HU5	1TF9		0.97	Sc=5.83362, min distance = 2.03099
APEH_AERPE F	71083 D-	CAH2_HUMAN Fu	6140 L-ph	2FMG	2HU5	2EZ7			Sc=5.71587, min distance = 2.57808
APHA_ECOLI F	24066 zal	DNK_DROME Ful	13711 dec	2VP5	1RMY	2VP9		0.96	Sc=5.98325, min distance = 0.91005
APHA_ECOLI F	24250 Oct	FMS1_YEAST Fu	1103 sper	1XPQ	2B8J	3BI5			Sc=5.66303, min distance = 2.42571
APHA_ECOLI F	514815 B	DNK_DROME Ful	13711 dec	2VP5	1RMY	2VQS			Sc=6.22168, min distance = 1.13563
APHA_ECOLI F	5790 flo	DNK_DROME Ful	13711 dec	2VP5	1RMY	2VP6		0.75	Sc=6.07699, min distance = 3.19246
APHA_ECOLI F	916 N1-ac	FMS1_YEAST Fu	1103 sper	1XPQ	2B8J	3CND		0.76	Sc=6.20372, min distance = 2.05986
APHA_SALTY F	24316 cyc	CNGK1_RHILO F	6076 Cycl	1VP6	1Z5U	3CL1		0.81	Sc=6.26828, min distance = 2.80104
APHA_SALTY F	656966 1y	PDE4D_HUMAN F	6076 Cycl	2PW3	1Z5U	1Y2E			Sc=6.07283, min distance = 2.60945

APHA_SALTY Fu	656969 1y	PDE4D_HUMAN Fu	6076 Cycl	2PW3	1Z5U	1Y2K		Sc=6.19033, min distance = 2.13310
APOD_HUMAN Fu	10472685	MCR_HUMAN Full	5994 prog	2AA6	2HZQ	2A3I	0.77	Sc=6.25158, min distance = 2.57006
APOD_HUMAN Fu	11987810	MCR_HUMAN Full	5994 prog	2AA6	2HZQ	2AA2		Sc=6.27771, min distance = 2.47908
APOD_HUMAN Fu	13472 Bol	P71278_ENTCL	5994 prog	2ABA	2HZQ	1H62	0.96	Sc=5.87063, min distance = 2.88125
APOD_HUMAN Fu	4369524 T	PRGR_HUMAN Fu	5994 prog	1A28	2HZQ	1ZUC		Sc=5.88123, min distance = 2.57346
APOD_HUMAN Fu	445033 CI	P71278_ENTCL	5994 prog	2ABA	2HZQ	1H60	0.8	Sc=5.99995, min distance = 2.71181
APOD_HUMAN Fu	5865 pred	MCR_HUMAN Full	5994 prog	2AA6	2HZQ	2AAX	0.76	Sc=6.35157, min distance = 2.61553
APT_HUMAN Fu	2519 caff	PYGM_RABIT Fu	6083 aden	8GPB	1ZN8	1GFZ		Sc=5.81757, min distance = 1.97714
APT_HUMAN Fu	6030 Uric	ECX1_SULSO Fu	6083 aden	2C38	1ZN8	2C37		Sc=6.23391, min distance = 2.29513
APT_HUMAN Fu	611002 Op	PIM1_HUMAN Fu	6083 aden	1YXU	1ZN8	1YXX		Sc=6.11595, min distance = 1.43055
APT_HUMAN Fu	65110 AIC	AAKG1_HUMAN Fu	6083 aden	2UV4	1ZN8	2UV5	0.79	Sc=6.27197, min distance = 2.39797
APT_HUMAN Fu	65533 cor	O57693_THETE	6083 aden	1UXU	1ZN8	1UXT		Sc=5.95532, min distance = 2.33849
APT_HUMAN Fu	656965 1y	PDE4D_HUMAN Fu	6083 aden	1TB7	1ZN8	1Y2D		Sc=6.03667, min distance = 1.45118
APT_HUMAN Fu	656968 1y	PDE4B_HUMAN Fu	6083 aden	1ROR	1ZN8	1Y2H		Sc=5.95231, min distance = 2.00494
APT_HUMAN Fu	657135 Op	PIM1_HUMAN Fu	6083 aden	1YXU	1ZN8	1YXV		Sc=5.84904, min distance = 1.80115
APT_HUMAN Fu	8582 Inos	PURA1_MOUSE Fu	6083 aden	1MF0	1ZN8	1IWE	0.79	Sc=6.40468, min distance = 1.25515
APX1_PEA Full	11957353	HBA_HORSE Full	444124 HE	1Y8I	1APX	1IWH	0.87	Sc=6.70151, min distance = 2.15679
APX1_PEA Full	11957363	HBA_HUMAN Full	444124 HE	1NQP	1APX	1RQA	0.81	Sc=6.71797, min distance = 2.27774
APX1_PEA Full	11957370	HMOX1_HUMAN Fu	444124 HE	1OZW	1APX	1TWN		Sc=6.51494, min distance = 1.98790
APX1_PEA Full	11957385	MYG_PHYCA Full	444124 HE	1U7R	1APX	2CMM		Sc=5.94417, min distance = 2.32011
APX1_PEA Full	444207 He	HBB_HORSE Full	444124 HE	1Y8I	1APX	2ZLW	0.99	Sc=6.75363, min distance = 1.66809
APX1_PEA Full	444522 HE	HBB_PAGBE Full	444124 HE	1S5X	1APX	1PBX	0.99	Sc=6.71699, min distance = 1.99497
APX1_PEA Full	446189 CI	HBG1_HUMAN Fu	444124 HE	1I3E	1APX	1I3D	0.89	Sc=6.70516, min distance = 1.11691
APX_STRGR Fu	5962 L-ly	THER_BACTH Fu	6140 L-ph	1OS0	1F2P	1KEI		Sc=5.69804, min distance = 2.06948
AQPZ_ECOLI Fu	444279 1s	OPSD_BOVIN Fu	445463 B-	3CAP	1RC2	1GZM		Sc=5.70849, min distance = 2.07351
ARAC_ECOLI Fu	323 couma	TRFL_BOVIN Fu	439731 al	2E1S	2ARC	3CRB		Sc=5.67732, min distance = 2.06364
ARAF_ECOLI Fu	15942683	FCN3_HUMAN Fu	439353 be	2J5Z	8ABP	2J60	0.77	Sc=5.62651, min distance = 2.13642
ARC3_CBDP Fu	164628 de	PMS2_HUMAN Fu	6022 Aden	1EA6	1GZF	1H7U		Sc=6.06264, min distance = 1.69260
ARC3_CBDP Fu	24832037	PGK1_HUMAN Fu	6022 Aden	2ZGV	1GZF	3C3C		Sc=6.30864, min distance = 1.70337
ARC3_CBDP Fu	36735 Gpp	PARM_ECOLX Fu	6022 Aden	1MWM	1GZF	2ZGZ	0.79	Sc=6.05757, min distance = 2.39870
ARC3_CBDP Fu	444503 FU	NDKC_DICDI Fu	6022 Aden	1KDN	1GZF	1B99		Sc=5.86071, min distance = 2.26170
ARC3_CBDP Fu	5957 Aden	HSP7F_YEAST Fu	6022 Aden	3C7N	1GZF	3D2F	0.99	Sc=5.7046, min distance = 2.514819
ARC3_CBDP Fu	5957 Aden	RK_BOVIN Full=	6022 Aden	3C4Z	1GZF	3C4W	0.99	Sc=6.03431, min distance = 1.33550
ARC3_CBDP Fu	5957 Aden	Y059_METJA Fu	6022 Aden	2J9D	1GZF	2J9C	0.99	Sc=6.44195, min distance = 1.89008
ARC3_CBDP Fu	6083 aden	PURP_METJA Fu	6022 Aden	2R7N	1GZF	2R7M	0.99	Sc=5.80799, min distance = 2.32022
ARC3_CBDP Fu	60961 ade	IPKA_RABIT Fu	6022 Aden	1JBP	1GZF	1FMO	0.95	Sc=5.85143, min distance = 2.25572
ARC3_CBDP Fu	6132 Cyti	RIR1_YEAST Fu	6022 Aden	2CVX	1GZF	2CVU		Sc=6.29835, min distance = 2.00304

ARC3_CBDP Fu	6852187 2	O57883_PYRHO I	6022 Aden	1WNL	1GZF	2DTH	0.89	Sc=6.13705, min distance = 1.97987
ARC3_CBDP Fu	91532 AMI	PURT_ECOLI Fu	6022 Aden	1KJQ	1GZF	1KJI	0.99	Sc=5.7777, min distance = 2.334992
ARCA_PSEAE F	123895 1h	NOS3_BOVIN Fu	6322 L-ar	4NSE	2A9G	5NSE	0.96	Sc=6.03636, min distance = 2.06028
ARCA_PSEAE F	123895 1h	NOSO_BACSU Fu	6322 L-ar	2FC1	2A9G	2FBZ	0.96	Sc=6.04111, min distance = 2.19477
ARCA_PSEAE F	126494 N(	NOS1_RAT Full	6322 L-ar	1OM4	2A9G	1K2S	0.76	Sc=6.21502, min distance = 1.61946
ARCA_PSEAE F	440005 L-	NOS1_RAT Full	6322 L-ar	1OM4	2A9G	1K2R	0.84	Sc=6.12461, min distance = 1.98618
ARCA_PSEAE F	444288 CI	KARG_LIMPO Fu	6322 L-ar	1M15	2A9G	1P52		Sc=5.87034, min distance = 2.12619
ARCA_PSEAE F	447180 To	NOS1_RAT Full	6322 L-ar	1OM4	2A9G	1MMV	0.83	Sc=6.21268, min distance = 1.77162
ARCA_PSEAE F	447181 VI	NOS1_RAT Full	6322 L-ar	1OM4	2A9G	1MMW	0.78	Sc=6.13625, min distance = 1.24122
ARCA_PSEAE F	5962 L-ly	ARGT_SALTY Fu	6322 L-ar	1LAF	2A9G	1LST	0.79	Sc=5.76391, min distance = 2.56042
ARCA_PSEAE F	6274 hist	ARGT_SALTY Fu	6322 L-ar	1LAF	2A9G	1LAG		Sc=5.85925, min distance = 2.43955
ARCA_PSEAE F	9085 homo	NOS3_BOVIN Fu	6322 L-ar	4NSE	2A9G	1DM7	0.9	Sc=6.03492, min distance = 2.05164
ARCA_PSEAE F	9547942 C	NOS3_BOVIN Fu	6322 L-ar	4NSE	2A9G	2G6O	0.84	Sc=5.95799, min distance = 2.14978
ARF1_HUMAN F	51 2-Oxop	SERA_ECOLI Fu	311 citri	2P9E	1RE0	1YBA	0.76	Sc=5.71862, min distance = 2.41383
ARF1_MOUSE F	445871 CI	RASH_HUMAN Fu	6830 guan	2CL7	1O3Y	1GNP		Sc=5.67283, min distance = 2.22367
ARF1_MOUSE F	8582 Inos	PURA1_MOUSE F	6830 guan	1LOO	1O3Y	1IWE	0.98	Sc=6.34047, min distance = 2.52514
ARF2_MOUSE F	445871 CI	RASH_HUMAN Fu	6830 guan	2CL7	1J2J	1GNP		Sc=5.64628, min distance = 2.19002
ARF6_HUMAN F	445871 CI	RASH_HUMAN Fu	6830 guan	2CL7	2A5D	1GNP		Sc=5.74797, min distance = 2.22475
ARGB_ECOLI F	36735 Gpp	PARM_ECOLX Fu	6022 Aden	1MWM	1OHA	2ZGZ	0.79	Sc=6.47174, min distance = 1.67308
ARGB_ECOLI F	447955 1p	CDK2_HUMAN Fu	6022 Aden	1GY3	1OHA	1PXI		Sc=5.70913, min distance = 2.28809
ARGB_ECOLI F	5957 Aden	ACTS_RABIT Fu	6022 Aden	1J6Z	1OHA	2FXU	0.99	Sc=5.94833, min distance = 2.14788
ARGB_ECOLI F	5957 Aden	MUTS_ECOLI Fu	6022 Aden	1OH7	1OHA	1W7A	0.99	Sc=5.925, min distance = 2.22641258
ARGB_ECOLI F	6083 aden	ENTP2_RAT Ful	33113 gan	3CJA	1GS5	3CJ7	0.97	Sc=5.64792, min distance = 2.44172
ARGB_ECOLI F	8977 1dan	NDK_THET8 Ful	6022 Aden	1WKL	1OHA	1WKK	0.8	Sc=5.97528, min distance = 2.03202
ARGB_ECOLI F	8977 1dan	PARM_ECOLX Fu	6022 Aden	1MWM	1OHA	2ZGY	0.8	Sc=6.43762, min distance = 1.62054
ARGB_PSEAE F	2608 1jsv	CDK2_HUMAN Fu	6022 Aden	1GY3	2BUF	1JSV		Sc=5.88645, min distance = 1.85733
ARGB_PSEAE F	3044933 A	ENPL_CANFA Fu	6022 Aden	1TC6	2BUF	1U0Y	0.81	Sc=6.30312, min distance = 2.21287
ARGB_PSEAE F	4564 1e1v	CDK2_HUMAN Fu	6022 Aden	1GY3	2BUF	1E1V		Sc=6.08253, min distance = 1.94945
ARGB_PSEAE F	5957 Aden	BIOD_ECOLI Fu	6022 Aden	1DAD	2BUF	1A82	0.99	Sc=6.3595, min distance = 0.884065
ARGB_PSEAE F	5957 Aden	MALK_ECOLI Fu	6022 Aden	2AWN	2BUF	1Q12	0.99	Sc=6.45321, min distance = 1.17645
ARGB_PSEAE F	5957 Aden	MUTS_ECOLI Fu	6022 Aden	1OH7	2BUF	1W7A	0.99	Sc=5.89274, min distance = 2.08893
ARGB_PSEAE F	5957 Aden	RK_BOVIN Full	6022 Aden	3C4Z	2BUF	3C4W	0.99	Sc=6.41126, min distance = 1.30148
ARGC_MYCTU F	15942680	6PGD_LACLM Fu	5886 NADI	2IZ0	2I3G	2IZ1		Sc=6.13826, min distance = 2.00504
ARGC_MYCTU F	16741136	DYR_ECOLI Ful	5886 NADI	1RA2	2I3G	1RX9	1	Sc=5.941, min distance = 1.8238303
ARGC_MYCTU F	440141 9i	DHB1_HUMAN Fu	5886 NADI	1QYV	2I3G	1QYW		Sc=5.90579, min distance = 2.34184
ARGD_THEMA F	447173 AC	BIOA_ECOLI Fu	1051 Code	1S07	2ORD	1MLY		Sc=5.82113, min distance = 2.46110
ARGD_THET8 F	11987749	AATM_CHICK Fu	444293 PI	1AKC	1WKH	1OXO		Sc=6.29769, min distance = 2.07877

ARGD_THET8 Fu	3744 Indo	AAT_ECOLI Full	444293 PL	1X28	1WKH	1AHF			Sc=5.85285, min distance = 2.377518
ARGI1_RAT Fu	107984 L-	NOS3_BOVIN Fu	123895 1h	5NSE	1HQF	1ED6		0.88	Sc=5.9312, min distance = 2.2010365
ARGI1_RAT Fu	11149707	NOS2_HUMAN Fu	6322 L-ar	1NSI	1T5G	3E7G			Sc=6.02751, min distance = 2.104890
ARGI1_RAT Fu	126494 N(	NOS1_RAT Full	123895 1h	1LZX	1HQF	1K2S			Sc=6.18187, min distance = 2.102403
ARGI1_RAT Fu	1337 Lopa	NOS3_BOVIN Fu	6322 L-ar	4NSE	1T5G	1D1X			Sc=6.0669, min distance = 1.9602879
ARGI1_RAT Fu	439202 L-	NOS3_BOVIN Fu	123895 1h	5NSE	1HQF	6NSE		0.75	Sc=5.82763, min distance = 2.504959
ARGI1_RAT Fu	444288 CI	KARG_LIMPO Fu	6322 L-ar	1M15	1T5G	1P52			Sc=5.74332, min distance = 2.085573
ARGI1_RAT Fu	447181 VI	NOS1_RAT Full	123895 1h	1LZX	1HQF	1MMW		0.76	Sc=6.10047, min distance = 2.076177
ARGI1_RAT Fu	5962 L-ly	ARGI_BACCD Fu	6322 L-ar	3CEV	1T5G	5CEV	42.76	0.79	Sc=5.83814, min distance = 0.839776
ARGI1_RAT Fu	9085 homo	NOS3_BOVIN Fu	123895 1h	5NSE	1HQF	1DM7		0.87	Sc=6.01525, min distance = 1.922865
ARGI1_RAT Fu	9547942 C	NOS3_BOVIN Fu	123895 1h	5NSE	1HQF	2G6O		0.81	Sc=5.92222, min distance = 2.300840
ARGI2_HUMAN I	446124 CU	ARGI1_RAT Full	446122 S2	3E9B	1PQ3	1HQH			Sc=5.85859, min distance = 2.302480
ARGI2_HUMAN I	448254 CI	ARGI1_RAT Full	446122 S2	3E9B	1PQ3	1R1O		0.81	Sc=5.61707, min distance = 1.881781
ARGI_BACCD Fu	123831 N,	NOS3_BOVIN Fu	6322 L-ar	4NSE	3CEV	7NSE		0.89	Sc=6.06693, min distance = 1.748250
ARGI_BACCD Fu	123895 1h	NOS3_BOVIN Fu	6322 L-ar	4NSE	3CEV	5NSE		0.96	Sc=6.01028, min distance = 2.195594
ARGI_BACCD Fu	123895 1h	NOSO_BACSU Fu	6322 L-ar	2FC1	3CEV	2FBZ		0.96	Sc=5.99439, min distance = 2.138015
ARGI_BACCD Fu	24850913	NOS1_RAT Full	6322 L-ar	1OM4	3CEV	3B3M			Sc=6.31858, min distance = 0.633631
ARGI_BACCD Fu	444288 CI	KARG_LIMPO Fu	6322 L-ar	1M15	3CEV	1P52			Sc=5.85925, min distance = 2.416416
ARGI_BACCD Fu	444965 2(	ARGI1_RAT Full	6322 L-ar	1T5G	3CEV	1D3V	42.76		Sc=5.83911, min distance = 1.674537
ARGI_BACCD Fu	446719 2-	ARGI1_RAT Full	6322 L-ar	1T5G	3CEV	1T5F	42.76		Sc=5.98116, min distance = 1.988905
ARGI_BACCD Fu	447181 VI	NOS1_RAT Full	6322 L-ar	1OM4	3CEV	1MMW		0.78	Sc=6.11185, min distance = 1.677516
ARGI_BACCD Fu	6274 hist	ARGT_SALTY Fu	5962 L-ly	1LST	5CEV	1LAG			Sc=5.80766, min distance = 2.660687
ARGI_BACCD Fu	6274 hist	ARGT_SALTY Fu	6322 L-ar	1LAF	3CEV	1LAG			Sc=5.79509, min distance = 2.791538
ARGI_BACCD Fu	657085 DE	ARGI1_RAT Full	6322 L-ar	1T5G	3CEV	1T4P	42.76		Sc=5.81714, min distance = 1.315491
ARGI_BACCD Fu	9547942 C	NOS3_BOVIN Fu	6322 L-ar	4NSE	3CEV	2G6O		0.84	Sc=5.93913, min distance = 2.223945
ARGR_BACST Fu	123831 N,	NOS3_BOVIN Fu	6322 L-ar	4NSE	1B4B	7NSE		0.89	Sc=5.87663, min distance = 1.902821
ARGR_BACST Fu	126494 N(	NOS1_RAT Full	6322 L-ar	1OM4	1B4B	1K2S		0.76	Sc=5.93288, min distance = 2.013021
ARGR_BACST Fu	133246 di	NOS2_HUMAN Fu	6322 L-ar	1NSI	1B4B	4NOS			Sc=6.1399, min distance = 1.9021645
ARGR_BACST Fu	439202 L-	NOS3_BOVIN Fu	6322 L-ar	4NSE	1B4B	6NSE			Sc=5.6826, min distance = 2.3918095
ARGR_BACST Fu	444288 CI	KARG_LIMPO Fu	6322 L-ar	1M15	1B4B	1P52			Sc=5.62077, min distance = 2.084250
ARGR_BACST Fu	444951 CI	NOS3_BOVIN Fu	6322 L-ar	4NSE	1B4B	1Q2O			Sc=6.16634, min distance = 1.857849
ARGR_BACST Fu	445040 6-	NOS1_RAT Full	6322 L-ar	1OM4	1B4B	2G6I			Sc=6.00056, min distance = 2.185477
ARGR_BACST Fu	446719 2-	ARGI1_RAT Full	6322 L-ar	1T5G	1B4B	1T5F			Sc=5.76635, min distance = 2.149715
ARGR_BACST Fu	447180 To	NOS1_RAT Full	6322 L-ar	1OM4	1B4B	1MMV		0.83	Sc=6.0246, min distance = 2.0217123
ARGR_BACST Fu	447181 VI	NOS1_RAT Full	6322 L-ar	1OM4	1B4B	1MMW		0.78	Sc=5.92407, min distance = 2.431716
ARGR_BACST Fu	5962 L-ly	ARGT_SALTY Fu	6322 L-ar	1LAF	1B4B	1LST		0.79	Sc=5.66805, min distance = 2.506992
ARGR_BACST Fu	6274 hist	ARGT_SALTY Fu	6322 L-ar	1LAF	1B4B	1LAG			Sc=5.67761, min distance = 2.694810

ARGR_BACST Fι	9085 homo	NOS3_BOVIN Fu	6322 L-ar	4NSE	1B4B	1DM7	0.9	Sc=5.74414, min distance = 2.36389
ARGR_BACST Fι	9547942 C	NOS3_BOVIN Fu	6322 L-ar	4NSE	1B4B	2G6O	0.84	Sc=5.69291, min distance = 2.18633
ARGR_ECOLI Fι	123831 N,	NOS3_BOVIN Fu	6322 L-ar	4NSE	1XXA	7NSE	0.89	Sc=5.93639, min distance = 2.55812
ARGR_ECOLI Fι	123895 1h	NOSO_BACSU Fu	6322 L-ar	2FC1	1XXA	2FBZ	0.96	Sc=5.85006, min distance = 2.60854
ARGR_ECOLI Fι	445108 AE	NOS3_BOVIN Fu	6322 L-ar	4NSE	1XXA	1DMJ		Sc=6.11173, min distance = 1.02460
ARGR_ECOLI Fι	447181 VI	NOS1_RAT Full	6322 L-ar	1OM4	1XXA	1MMW	0.78	Sc=5.98791, min distance = 2.68418
ARGR_ECOLI Fι	5962 L-ly	ARGT_SALTY Fu	6322 L-ar	1LAF	1XXA	1LST	0.79	Sc=5.65587, min distance = 2.26591
ARGR_ECOLI Fι	9547942 C	NOS3_BOVIN Fu	6322 L-ar	4NSE	1XXA	2G6O	0.84	Sc=5.73209, min distance = 2.22723
ARGT_SALTY Fι	126494 N(	NOS1_RAT Full	6322 L-ar	1OM4	1LAF	1K2S	0.76	Sc=6.24588, min distance = 1.94054
ARGT_SALTY Fι	1649 3-br	NOS3_BOVIN Fu	6322 L-ar	4NSE	1LAF	1D0C		Sc=5.94907, min distance = 0.83807
ARGT_SALTY Fι	165271 L-	HISX_ECOLI Fu	6274 hist	1KAH	1LAG	1KAE	0.89	Sc=5.79273, min distance = 2.20766
ARGT_SALTY Fι	1894 7-ni	NOS3_BOVIN Fu	6322 L-ar	4NSE	1LAF	1FOJ		Sc=5.91557, min distance = 2.19337
ARGT_SALTY Fι	439202 L-	NOS3_BOVIN Fu	6322 L-ar	4NSE	1LAF	6NSE		Sc=5.91856, min distance = 2.38046
ARGT_SALTY Fι	444965 2(	ARGI1_RAT Ful	6322 L-ar	1T5G	1LAF	1D3V		Sc=5.81639, min distance = 2.12274
ARGT_SALTY Fι	446122 S2	ARGI1_RAT Ful	6322 L-ar	1T5G	1LAF	3E9B		Sc=5.71345, min distance = 2.04563
ARGT_SALTY Fι	446719 2-	ARGI1_RAT Ful	6322 L-ar	1T5G	1LAF	1T5F		Sc=6.01897, min distance = 2.18709
ARGT_SALTY Fι	447180 To	NOS1_RAT Full	6322 L-ar	1OM4	1LAF	1MMV	0.83	Sc=6.24785, min distance = 2.16218
ARGT_SALTY Fι	447181 VI	NOS1_RAT Full	6322 L-ar	1OM4	1LAF	1MMW	0.78	Sc=6.17753, min distance = 2.20706
ARGT_SALTY Fι	657087 CI	ARGI1_RAT Ful	6322 L-ar	1T5G	1LAF	1T4T	0.76	Sc=5.86311, min distance = 2.00152
ARGT_SALTY Fι	9085 homo	NOS3_BOVIN Fu	6322 L-ar	4NSE	1LAF	1DM7	0.9	Sc=6.08345, min distance = 2.03936
ARGT_SALTY Fι	9547942 C	NOS3_BOVIN Fu	6322 L-ar	4NSE	1LAF	2G6O	0.84	Sc=6.01662, min distance = 2.05521
ARIS_PENRO Fι	131682 Sa	AOFB_HUMAN Fu	445070 fa	2BK3	1DGP	2V5Z		Sc=6.23368, min distance = 1.16219
ARK72_HUMAN I	448573 FI	CASP7_HUMAN Fι	311 citri	2QL9	2BP1	1SHL		Sc=6.03541, min distance = 0.08914
ARK72_HUMAN I	51 2-Oxop	SERA_ECOLI Fu	311 citri	2P9E	2BP1	1YBA	0.76	Sc=5.60743, min distance = 2.13869
ARK72_MOUSE I	164795 N(	LMBL1_HUMAN Fι	78165 MES	1OZ2	2C91	2RHY		Sc=5.60861, min distance = 2.09785
ARL1_HUMAN Fι	445871 CI	RASH_HUMAN Fu	6830 guan	2CL7	1UPT	1GNP		Sc=5.67035, min distance = 2.19079
ARL1_RAT Ful	445873 CA	RASH_HUMAN Fu	36735 Gpp	1CTQ	1R4A	2CL6		Sc=6.28211, min distance = 2.13546
ARL1_RAT Ful	446248 CI	RASH_HUMAN Fu	36735 Gpp	1CTQ	1R4A	1IAQ		Sc=6.04226, min distance = 2.52137
ARL1_RAT Ful	5496555 C	RASH_HUMAN Fu	36735 Gpp	1CTQ	1R4A	2EVW		Sc=5.99201, min distance = 2.21342
ARL1_RAT Ful	93082 Gab	RAC3_HUMAN Fu	36735 Gpp	2IC5	1R4A	2QME	0.99	Sc=5.82295, min distance = 2.47359
ARL1_RAT Ful	93082 Gab	RASH_HUMAN Fu	36735 Gpp	1CTQ	1R4A	121P	0.99	Sc=5.80918, min distance = 2.19852
ARL1_RAT Ful	93082 Gab	SRP54_THEAQ Fι	36735 Gpp	1JPN	1R4A	1RJ9	0.99	Sc=6.11531, min distance = 1.60634
ARL2_MOUSE Fι	37792 gam	GNAT1_BOVIN Fι	8977 1dar	1TAD	1KSH	1TND	0.99	Sc=6.1567, min distance = 2.125956
ARL2_MOUSE Fι	37792 gam	RAB6B_HUMAN Fι	8977 1dar	2E9S	1KSH	2FFQ	0.99	Sc=6.50516, min distance = 2.68224
ARL2_MOUSE Fι	37792 gam	RB11A_HUMAN Fι	8977 1dar	1OIX	1KSH	1OIW	0.99	Sc=6.14437, min distance = 2.73171
ARL2_MOUSE Fι	37792 gam	RHOA_HUMAN Fu	8977 1dar	1TX4	1KSH	1CXZ	0.99	Sc=6.16314, min distance = 2.43011
ARL2_MOUSE Fι	445873 CA	RASH_HUMAN Fu	8977 1dar	2CE2	1KSH	2CL6		Sc=6.73264, min distance = 2.01596

ARL2_MOUSE F	446248 CI	RASH_HUMAN Fu	6830 guan	2CL7	1KSG	1IAQ			Sc=5.77389, min distance = 2.24354
ARL2_MOUSE F	446248 CI	RASH_HUMAN Fu	8977 1dar	2CE2	1KSH	1IAQ			Sc=6.14008, min distance = 2.59493
ARL2_MOUSE F	6804 guar	DPOL_BPR69 Fu	8977 1dar	1CLQ	1KSH	1WAJ		0.99	Sc=6.02439, min distance = 2.19394
ARL2_MOUSE F	6804 guar	EF1A_YEAST Fu	8977 1dar	2B7B	1KSH	1G7C		0.99	Sc=6.04768, min distance = 2.58838
ARL2_MOUSE F	8582 Inos	PURA1_MOUSE F	6830 guan	1LOO	1KSG	1IWE		0.98	Sc=6.3486, min distance = 2.4283910
ARL2_MOUSE F	8582 Inos	PURA1_MOUSE F	8977 1dar	1LON	1KSH	1IWE		0.99	Sc=6.36062, min distance = 2.72818
ARL2_MOUSE F	93082 Gab	CDC42_HUMAN F	8977 1dar	2NGR	1KSH	2ODB		0.99	Sc=6.23186, min distance = 2.17075
ARL2_MOUSE F	93082 Gab	EFG_THETH Ful	8977 1dar	2BM0	1KSH	2J7K		0.99	Sc=6.20382, min distance = 2.19389
ARL2_MOUSE F	93082 Gab	RAC3_HUMAN Fu	8977 1dar	2C2H	1KSH	2QME		0.99	Sc=6.22737, min distance = 2.65474
ARL3_MOUSE F	446248 CI	RAN_HUMAN Ful	36735 Gpp	1K5D	3BH6	1IBR			Sc=6.17875, min distance = 2.31414
ARL3_MOUSE F	93082 Gab	FTSY_THEAQ Fu	36735 Gpp	2J7P	3BH6	1RJ9		0.99	Sc=6.23186, min distance = 2.36787
ARL5B_HUMAN	6804 guar	EF1A_YEAST Fu	8977 1dar	2B7B	1YZG	1G7C		0.99	Sc=5.95006, min distance = 2.43672
ARL6_HUMAN F	65070 dUT	Q381M1_9TRYP	6830 guan	2Q0E	2H57	2NOM			Sc=5.90484, min distance = 1.94174
ARL8A_HUMAN	37792 gam	GNAI1_RAT Ful	8977 1dar	1SVK	1ZD9	1AS0		0.99	Sc=6.52447, min distance = 2.31270
ARL8A_HUMAN	37792 gam	RAB6B_HUMAN F	8977 1dar	2E9S	1ZD9	2FFQ		0.99	Sc=6.53713, min distance = 2.10657
ARL8A_HUMAN	37792 gam	RAC1_HUMAN Fu	8977 1dar	1RYF	1ZD9	2FJU		0.99	Sc=6.54409, min distance = 2.24656
ARL8A_HUMAN	37792 gam	RB11A_HUMAN F	8977 1dar	1OIX	1ZD9	1OIW		0.99	Sc=6.52447, min distance = 2.42828
ARL8A_HUMAN	6804 guar	EF1A_YEAST Fu	8977 1dar	2B7B	1ZD9	1G7C		0.99	Sc=6.43155, min distance = 2.36133
ARL8A_HUMAN	93082 Gab	CDC42_HUMAN F	8977 1dar	2NGR	1ZD9	2ODB		0.99	Sc=6.57453, min distance = 2.10347
ARO1_EMENI F	169266 1,	AROB_STAAR Fu	5893 nadi	1XAH	1SG6	1XAG	39.73	0.79	Sc=6.4338, min distance = 2.187607
ARO1_EMENI F	25023707	DNLJ_ENTFA Fu	5893 nadi	1TAE	1SG6	3BAB			Sc=6.56954, min distance = 1.93770
ARO1_EMENI F	445794 AD	FUCO_ECOLI Fu	5893 nadi	2BI4	1SG6	1RRM			Sc=6.59504, min distance = 2.24317
ARO1_EMENI F	5957 Ader	BIOD_ECOLI Fu	444564 AD	1BS1	1NVA	1A82		0.91	Sc=5.67546, min distance = 2.32412
ARO1_EMENI F	6022 Ader	BIOD_ECOLI Fu	444564 AD	1BS1	1NVA	1DAD		0.91	Sc=5.94562, min distance = 2.32953
ARO1_EMENI F	91532 AMF	BIOD_ECOLI Fu	444564 AD	1BS1	1NVA	1DAG		0.91	Sc=6.43932, min distance = 2.47523
AROA_STRPN F	8742 shik	AROA_ECOLI Fu	3496 glyp	1G6S	1RF6	2AA9	25.76		Sc=5.85455, min distance = 2.46252
AROB_STAAR F	440141 9i	GSHR_HUMAN Fu	5893 nadi	1GRB	1XAH	1GRA			Sc=5.62387, min distance = 2.36894
AROC_HELPY F	449551 FM	FLAV_DESVH Fu	444243 FA	1F4P	1UM0	5FX2		0.77	Sc=6.6025, min distance = 2.340497
AROC_MYCTU F	5326566 l	BLVRB_HUMAN F	444243 FA	1HE4	2O12	1HE5		0.82	Sc=6.21094, min distance = 2.17092
AROC_STRPN F	5326566 l	BLVRB_HUMAN F	444243 FA	1HE4	1QXO	1HE5		0.82	Sc=6.14451, min distance = 2.40606
AROE_AQUAE F	121947 sh	AROA_ECOLI Fu	8742 shik	2AA9	2HK9	1G6S		0.91	Sc=6.03219, min distance = 1.91397
AROE_AQUAE F	15942680	6PGD_LACLM Fu	5886 NADF	2IZ0	2HK9	2IZ1			Sc=6.12063, min distance = 1.98726
AROE_AQUAE F	440141 9i	G6PD_LEUME Fu	5886 NADF	1H9A	2HK9	1E7Y			Sc=6.36106, min distance = 2.32251
AROE_AQUAE F	445782 CI	AROA_ECOLI Fu	8742 shik	2AA9	2HK9	2QFQ		0.91	Sc=6.04503, min distance = 1.79107
AROE_AQUAE F	445904 DH	AROK_MYCTU Fu	8742 shik	2IYS	2HK9	2DFN			Sc=5.80603, min distance = 2.42902
AROE_AQUAE F	448155 AD	ILV5_SPIOL Fu	5886 NADF	1YVE	2HK9	1QMG			Sc=6.18045, min distance = 1.76093
AROE_ECOLI F	440141 9i	G6PD_LEUME Fu	5886 NADF	1H9A	1NYT	1E7Y			Sc=6.39142, min distance = 1.91662

AROE_ECOLI	F	440334 Ni	DAPB_ECOLI Fu	5886 NADF	1DIH	1NYT	1DRW	0.91	Sc=6.29966, min distance = 2.077618
AROE_ECOLI	F	448155 AD	ILV5_SPIOL Fu	5886 NADF	1YVE	1NYT	1QMG		Sc=6.17935, min distance = 1.887319
AROE_THET8	F	121947 sh	AROA_ECOLI Fu	8742 shik	2AA9	2D5C	1G6S	0.91	Sc=6.02445, min distance = 2.026645
AROE_THET8	F	121947 sh	AROK_MYCTU Fu	8742 shik	2IYS	2D5C	2IYY	0.91	Sc=5.95599, min distance = 2.033340
AROE_THET8	F	440141 9i	DHB1_HUMAN Fu	5886 NADF	1QYV	2EV9	1QYW		Sc=5.91537, min distance = 2.294635
AROE_THET8	F	440334 Ni	DAPB_ECOLI Fu	5886 NADF	1DIH	2EV9	1DRW	0.91	Sc=5.81478, min distance = 2.024351
AROE_THET8	F	445782 CI	AROA_ECOLI Fu	8742 shik	2AA9	2D5C	2QFQ	0.91	Sc=6.04629, min distance = 2.005322
AROE_THET8	F	445904 DH	AROK_MYCTU Fu	8742 shik	2IYS	2D5C	2DFN		Sc=5.79879, min distance = 2.436798
AROF_THEMA	F	188324 Ri	KDSA_AQUAE Fu	1005 phos	2NWR	1RZM	1FXQ		Sc=5.76165, min distance = 1.898578
AROF_THEMA	F	447634 RI	KDSA_AQUAE Fu	1005 phos	2NWR	1RZM	2NX1		Sc=5.73982, min distance = 2.141309
AROF_THEMA	F	447634 RI	KDSA_AQUAE Fu	122357 er	1FWT	1RZM	2NX1		Sc=5.83226, min distance = 1.721541
AROG_YEAST	F	439278 ph	KDSA_AQUAE Fu	446060 2-	2NX3	1HFB	1PE1	0.88	Sc=5.70644, min distance = 2.239969
AROG_YEAST	F	444881 L-	CHMU_YEAST Fu	6057 L-ty	4CSM	1OF6	1CSM		Sc=6.22822, min distance = 0.833767
AROG_YEAST	F	6140 L-ph	SYFA_THETH Fu	6057 L-ty	2AMC	1OF6	1B70	0.84	Sc=5.78012, min distance = 2.191230
AROG_YEAST	F	6305 L-tr	CHMU_YEAST Fu	6057 L-ty	4CSM	1OF6	5CSM		Sc=6.09558, min distance = 1.902222
AROG_YEAST	F	6420123 E	SYY_METJA Ful	6057 L-ty	1J1U	1OF6	1ZH6	0.77	Sc=6.08109, min distance = 1.290825
AROG_YEAST	F	671214 L-	SYY_METJA Ful	6057 L-ty	1J1U	1OF6	2AG6	0.76	Sc=5.84424, min distance = 1.802735
AROG_YEAST	F	736190 L-	SYFA_THETH Fu	6057 L-ty	2AMC	1OF6	2AKW	0.76	Sc=5.82658, min distance = 2.628016
AROK_HELPY	F	445904 DH	AROK_MYCTU Fu	8742 shik	2IYS	1ZUI	2DFN		Sc=5.89465, min distance = 2.501136
AROK_MYCTU	F	33113 gam	KIF1A_MOUSE F	91532 AMF	1I6I	1ZYU	1VFV	0.99	Sc=6.02665, min distance = 2.329790
AROK_MYCTU	F	3547 Fasu	KAPCA_BOVIN F	5957 Aden	1Q24	2IYW	1Q8W		Sc=5.92628, min distance = 2.173532
AROK_MYCTU	F	444564 AD	BIOD_ECOLI Fu	5957 Aden	1A82	2IYW	1BS1	0.91	Sc=5.96788, min distance = 2.454626
AROK_MYCTU	F	444564 AD	BIOD_ECOLI Fu	91532 AMF	1DAG	1ZYU	1BS1	0.91	Sc=6.00653, min distance = 2.125886
AROK_MYCTU	F	444564 AD	MYS2_DICDI Fu	5957 Aden	1FMW	2IYW	1W9I	0.91	Sc=5.96788, min distance = 0
AROK_MYCTU	F	444914 4-	CDGT2_BACCI F	23831 HEF	1OT1	1L4U	1CXL		Sc=5.64592, min distance = 2.253781
AROK_MYCTU	F	447004 CI	MYS2_DICDI Fu	5957 Aden	1FMW	2IYW	1LVK		Sc=5.75815, min distance = 0
AROK_MYCTU	F	449240 1y	KAPCA_BOVIN F	5957 Aden	1Q24	2IYW	1YDR		Sc=5.88549, min distance = 2.034630
AROK_MYCTU	F	6132 Cyti	ECX2_PYRAB Fu	6022 Aden	2PO0	2IYV	2PO2		Sc=5.72667, min distance = 2.011125
AROK_MYCTU	F	6338562 C	MYS2_DICDI Fu	5957 Aden	1FMW	2IYW	1D0Y		Sc=6.18879, min distance = 0
AROK_MYCTU	F	6338563 C	MYS2_DICDI Fu	5957 Aden	1FMW	2IYW	1D0Z		Sc=6.17112, min distance = 0
AROK_MYCTU	F	6338564 C	MYS2_DICDI Fu	5957 Aden	1FMW	2IYW	1D1A		Sc=5.78021, min distance = 0
AROK_MYCTU	F	65059 Dec	RNAS1_BOVIN F	6022 Aden	1O0H	2IYV	2QCA	0.77	Sc=5.92067, min distance = 2.015011
AROL_ERWCH	F	445940 O6	CDK2_HUMAN Fu	6022 Aden	1GY3	2SHK	1GZ8		Sc=6.05449, min distance = 2.529949
AROL_ERWCH	F	447004 CI	MYS2_DICDI Fu	6022 Aden	1VOM	2SHK	1LVK		Sc=5.7668, min distance = 2.1078944
AROQ_HELPY	F	1005 phos	F16PA_ECOLI F	311 citri	2OWZ	2C4V	2OX3		Sc=5.65805, min distance = 2.214518
AROQ_HELPY	F	439183 1i	TPIS_TRYBB Fu	311 citri	2VEK	2C4V	1IIH		Sc=5.70474, min distance = 2.233508
AROQ_HELPY	F	447550 ph	TPIS_TRYBB Fu	311 citri	2VEK	2C4V	4TIM		Sc=5.69042, min distance = 2.405668

AROQ_HELPY F	4797 1tpw	TPIS_TRYBB Fu	311 citri	2VEK	2C4V	1TTJ			Sc=5.61518, min distance = 2.11358
AROQ_HELPY F	51 2-Oxop	SERA_ECOLI Fu	311 citri	2P9E	2C4V	1YBA		0.76	Sc=5.77729, min distance = 1.33791
AROQ_STRCO F	121947 sh	AROK_MYCTU Fu	445904 DH	2DFN	1GTZ	2IYY			Sc=6.06972, min distance = 2.16546
AROQ_STRCO F	6102777 2	AROQ_HELPY Fu	445905 1g	2C57	1GU1	2C4W	43.28		Sc=6.47529, min distance = 0.67267
ARP2_BOVIN F	11708454	KAPCA_BOVIN F	5957 Aden	1Q24	1TYQ	2VNW			Sc=6.14663, min distance = 2.65320
ARP2_BOVIN F	3064778 H	ROCK1_HUMAN F	33113 gam	2V55	2P9U	2ETK			Sc=6.07038, min distance = 2.16434
ARP2_BOVIN F	3540 1yds	KAPCA_BOVIN F	5957 Aden	1Q24	1TYQ	1YDS			Sc=5.91714, min distance = 2.04042
ARP2_BOVIN F	3547 Fasu	KAPCA_BOVIN F	5957 Aden	1Q24	1TYQ	1Q8W			Sc=5.98898, min distance = 2.01394
ARP2_BOVIN F	36735 Gpp	PARM_ECOLX Fu	6022 Aden	1MWM	2P9I	2ZGZ		0.79	Sc=6.52334, min distance = 1.65342
ARP2_BOVIN F	440141 9i	RNAS1_BOVIN F	6022 Aden	1O0H	2P9I	1O0O	1		Sc=6.46696, min distance = 1.32987
ARP2_BOVIN F	444564 AD	BIOD_ECOLI Fu	5957 Aden	1A82	1TYQ	1BS1		0.91	Sc=5.97772, min distance = 2.22532
ARP2_BOVIN F	445966 O6	CDK2_HUMAN Fu	6022 Aden	1GY3	2P9I	1H0V			Sc=6.30021, min distance = 2.37218
ARP2_BOVIN F	447955 1p	CDK2_HUMAN Fu	6022 Aden	1GY3	2P9I	1PXI			Sc=5.8145, min distance = 2.304665
ARP2_BOVIN F	448042 2e	ROCK1_HUMAN F	33113 gam	2V55	2P9U	2ETR			Sc=6.07606, min distance = 2.25437
ARP2_BOVIN F	449240 1y	KAPCA_BOVIN F	5957 Aden	1Q24	1TYQ	1YDR			Sc=6.01319, min distance = 2.11939
ARP2_BOVIN F	6083 aden	GLNA1_MYCTU F	6022 Aden	2BVC	2P9I	1HTO		0.99	Sc=6.4118, min distance = 2.343476
ARP2_BOVIN F	6083 aden	PURP_METJA Fu	5957 Aden	2R7L	1TYQ	2R7M		0.98	Sc=6.38586, min distance = 2.20880
ARP2_BOVIN F	6083 aden	PURP_METJA Fu	6022 Aden	2R7N	2P9I	2R7M		0.99	Sc=6.40049, min distance = 2.19687
ARP2_BOVIN F	6804 guan	NDK_PYRHO Ful	6022 Aden	2DYA	2P9I	2DXF		0.8	Sc=6.42187, min distance = 2.20642
ARP2_BOVIN F	8977 1dan	NDK_PYRHO Ful	6022 Aden	2DYA	2P9I	2DXE		0.8	Sc=6.4884, min distance = 2.081461
ARP2_BOVIN F	8977 1dan	PARM_ECOLX Fu	6022 Aden	1MWM	2P9I	2ZGY		0.8	Sc=6.47986, min distance = 2.19955
ARP2_BOVIN F	9543433 T	NDKC_DICDI Fu	6022 Aden	1KDN	2P9I	1F6T			Sc=6.42799, min distance = 1.83483
ARP3_BOVIN F	11708454	KAPCA_BOVIN F	5957 Aden	1Q24	1TYQ	2VNW			Sc=6.18084, min distance = 2.20465
ARP3_BOVIN F	16750062	CSK2A_MAIZE F	33113 gam	1LP4	2P9U	2OXD			Sc=5.75433, min distance = 2.11775
ARP3_BOVIN F	16750063	CSK2A_MAIZE F	33113 gam	1LP4	2P9U	2OXX			Sc=5.65458, min distance = 2.00722
ARP3_BOVIN F	3540 1yds	KAPCA_BOVIN F	5957 Aden	1Q24	1TYQ	1YDS			Sc=5.9571, min distance = 2.070042
ARP3_BOVIN F	444852 CI	ATPB_BOVIN Fu	5957 Aden	2V7Q	1TYQ	1COW		0.91	Sc=6.51505, min distance = 2.12242
ARP3_BOVIN F	447955 1p	CDK2_HUMAN Fu	6022 Aden	1GY3	2P9I	1PXI			Sc=5.82051, min distance = 2.28773
ARP3_BOVIN F	448042 2e	ROCK1_HUMAN F	33113 gam	2V55	2P9U	2ETR			Sc=6.17763, min distance = 2.16444
ARP3_BOVIN F	448043 2g	ROCK1_HUMAN F	33113 gam	2V55	2P9U	3D9V			Sc=6.06706, min distance = 1.76835
ARP3_BOVIN F	449240 1y	KAPCA_BOVIN F	5957 Aden	1Q24	1TYQ	1YDR			Sc=6.03156, min distance = 2.14596
ARP3_BOVIN F	4565 1h1n	CDK2_HUMAN Fu	6022 Aden	1GY3	2P9I	1H1R			Sc=6.15265, min distance = 1.90802
ARP3_BOVIN F	6083 aden	GLNA1_MYCTU F	6022 Aden	2BVC	2P9I	1HTO		0.99	Sc=6.41341, min distance = 2.24356
ARP3_BOVIN F	6083 aden	PURP_METJA Fu	6022 Aden	2R7N	2P9I	2R7M		0.99	Sc=6.41112, min distance = 2.14572
ARP3_BOVIN F	8977 1dan	NDK_PYRHO Ful	6022 Aden	2DYA	2P9I	2DXE		0.8	Sc=6.49995, min distance = 1.99799
ARP3_BOVIN F	91532 AMP	KIF1A_MOUSE F	33113 gam	1VFV	2P9U	1I6I		0.99	Sc=6.49741, min distance = 2.08979
ARP3_BOVIN F	91532 AMP	PURT_ECOLI Fu	33113 gam	1EYZ	2P9U	1KJI		0.99	Sc=6.52393, min distance = 1.76111

ARP3_SCHPO Fı	11708454	KAPCA_BOVIN Fı	5957 Aden	1Q24	3DWL	2VNW			Sc=6.10799, min distance = 2.63777
ARP3_SCHPO Fı	3540 1yds	KAPCA_BOVIN Fı	5957 Aden	1Q24	3DWL	1YDS			Sc=5.89996, min distance = 2.12939
ARP3_SCHPO Fı	3547 Fasu	KAPCA_BOVIN Fı	5957 Aden	1Q24	3DWL	1Q8W			Sc=5.94712, min distance = 2.10696
ARP3_SCHPO Fı	440317 AT	PURT_ECOLI Fu	5957 Aden	1KJ8	3DWL	1KJJ		0.99	Sc=5.99931, min distance = 1.68063
ARP3_SCHPO Fı	444564 AD	BIOD_ECOLI Fu	5957 Aden	1A82	3DWL	1BS1		0.91	Sc=5.64007, min distance = 2.29486
ARP3_SCHPO Fı	449240 1y	KAPCA_BOVIN Fı	5957 Aden	1Q24	3DWL	1YDR			Sc=5.96825, min distance = 2.18956
ARP3_SCHPO Fı	5893 nadi	MAOM_HUMAN Fu	5957 Aden	1GZ4	3DWL	1PJ3			Sc=6.24557, min distance = 2.44701
ARP3_SCHPO Fı	6022 Aden	ACT1_DROME Fu	5957 Aden	2HF4	3DWL	2HF3		0.99	Sc=5.93592, min distance = 2.66187
ARP3_SCHPO Fı	6022 Aden	ARSA1_ECOLX Fı	5957 Aden	1II0	3DWL	1IHU		0.99	Sc=5.95052, min distance = 1.94715
ARP3_SCHPO Fı	6022 Aden	BIOD_ECOLI Fu	5957 Aden	1A82	3DWL	1DAD		0.99	Sc=5.64665, min distance = 2.23956
ARP3_SCHPO Fı	6022 Aden	CLCN5_HUMAN Fı	5957 Aden	2J9L	3DWL	2JA3		0.99	Sc=6.42245, min distance = 2.24013
ARP3_SCHPO Fı	6022 Aden	KTHY_HUMAN Fu	5957 Aden	1E2Q	3DWL	1NN3		0.99	Sc=5.94155, min distance = 2.43234
ARP3_SCHPO Fı	6022 Aden	MUTS_ECOLI Fu	5957 Aden	1W7A	3DWL	1OH7		0.99	Sc=5.62891, min distance = 1.40376
ARP3_SCHPO Fı	6022 Aden	PPCK_ECOLI Fu	5957 Aden	2OLR	3DWL	1K3C		0.99	Sc=5.63309, min distance = 1.70679
ARP3_SCHPO Fı	6022 Aden	PURP_METJA Fu	5957 Aden	2R7L	3DWL	2R7N		0.99	Sc=5.96591, min distance = 2.22992
ARP3_SCHPO Fı	6022 Aden	Q72H90_THET2 I	5957 Aden	2BEK	3DWL	2BEJ		0.99	Sc=5.62796, min distance = 2.72359
ARP3_SCHPO Fı	6083 aden	AAKG1_RAT Ful	5957 Aden	2V92	3DWL	2V8Q		0.98	Sc=6.36522, min distance = 1.93296
ARP3_SCHPO Fı	6083 aden	PURP_METJA Fu	5957 Aden	2R7L	3DWL	2R7M		0.98	Sc=6.36755, min distance = 1.97069
ARP3_SCHPO Fı	6083 aden	PURP_PYRFU Fu	5957 Aden	2R86	3DWL	2R85		0.98	Sc=6.36984, min distance = 2.12405
ARSA1_ECOLX I	11608401	KAPCA_BOVIN Fı	5957 Aden	1Q24	1II0	2UVY			Sc=6.19169, min distance = 2.46601
ARSA1_ECOLX I	16750062	CSK2A_MAIZE Fı	33113 gam	1LP4	1II9	2OXD			Sc=5.68594, min distance = 2.01469
ARSA1_ECOLX I	3064778 H	ROCK1_HUMAN Fı	33113 gam	2V55	1II9	2ETK			Sc=6.10296, min distance = 2.04247
ARSA1_ECOLX I	3540 1yds	KAPCA_BOVIN Fı	5957 Aden	1Q24	1II0	1YDS			Sc=5.85296, min distance = 2.20593
ARSA1_ECOLX I	398148 1e	CDK2_HUMAN Fu	6022 Aden	1GY3	1IHU	1E1X			Sc=6.26619, min distance = 1.80793
ARSA1_ECOLX I	444564 AD	BIOD_ECOLI Fu	5957 Aden	1A82	1II0	1BS1		0.91	Sc=6.42539, min distance = 2.68464
ARSA1_ECOLX I	444564 AD	BIOD_ECOLI Fu	6022 Aden	1DAD	1IHU	1BS1		0.91	Sc=6.429, min distance = 2.6030059
ARSA1_ECOLX I	445940 O6	CDK2_HUMAN Fu	6022 Aden	1GY3	1IHU	1GZ8			Sc=6.17038, min distance = 1.78634
ARSA1_ECOLX I	445966 O6	CDK2_HUMAN Fu	6022 Aden	1GY3	1IHU	1H0V			Sc=6.25591, min distance = 2.27621
ARSA1_ECOLX I	447004 CI	MYS2_DICDI Fu	33113 gam	1MMN	1II9	1LVK			Sc=6.16364, min distance = 2.44151
ARSA1_ECOLX I	447004 CI	MYS2_DICDI Fu	5957 Aden	1FMW	1II0	1LVK			Sc=6.05948, min distance = 0
ARSA1_ECOLX I	447052 CI	CSK2A_MAIZE Fı	33113 gam	1LP4	1II9	1M2R			Sc=6.35287, min distance = 1.93320
ARSA1_ECOLX I	449240 1y	KAPCA_BOVIN Fı	5957 Aden	1Q24	1II0	1YDR			Sc=5.92776, min distance = 2.33642
ARSA1_ECOLX I	4564 1e1v	CDK2_HUMAN Fu	6022 Aden	1GY3	1IHU	1E1V			Sc=6.21329, min distance = 1.53843
ARSA1_ECOLX I	4565 1h1r	CDK2_HUMAN Fu	6022 Aden	1GY3	1IHU	1H1R			Sc=6.09549, min distance = 1.21540
ARSA1_ECOLX I	5326977 1	CSK2A_MAIZE Fı	33113 gam	1LP4	1II9	1ZOG			Sc=5.67711, min distance = 1.84500
ARSA1_ECOLX I	5496923 C	HS90A_HUMAN Fı	6022 Aden	1BYQ	1IHU	1UY8			Sc=6.24576, min distance = 2.17355
ARSA1_ECOLX I	6031 Uric	DCK_HUMAN Ful	6022 Aden	1P5Z	1IHU	2ZIA			Sc=6.36658, min distance = 2.39193

ARSA1_ECOLX I	6083 ader	AAKG1_RAT Ful	5957 Aden	2V92	1II0	2V8Q		0.98	Sc=6.32965, min distance = 1.99460
ARSA1_ECOLX I	6083 ader	ENTP2_RAT Ful	33113 gam	3CJA	1II9	3CJ7		0.97	Sc=5.96721, min distance = 2.53505
ARSA1_ECOLX I	6083 ader	HSP71_HUMAN Fu	33113 gam	2E8A	1II9	1XQS		0.97	Sc=5.98059, min distance = 2.18102
ARSA1_ECOLX I	6083 ader	PSPF_ECOLI Fu	5957 Aden	2C96	1II0	2VII		0.98	Sc=6.3249, min distance = 2.305972
ARSA1_ECOLX I	60961 ade	SKY1_YEAST Fu	33113 gam	1Q99	1II9	1Q97		0.93	Sc=6.2121, min distance = 2.257368
ARSA1_ECOLX I	6102784 A	PSPF_ECOLI Fu	5957 Aden	2C96	1II0	2C99		0.99	Sc=6.46629, min distance = 2.36717
ARSA1_ECOLX I	6102784 A	PSPF_ECOLI Fu	6022 Aden	2C98	1IHU	2C99		0.99	Sc=6.50482, min distance = 1.90659
ARSA1_ECOLX I	6132 Cyti	ECX1_PYRAB Fu	6022 Aden	2PO0	1IHU	2PO2			Sc=5.97405, min distance = 1.91107
ARSA1_ECOLX I	6338562 C	MYS2_DICDI Fu	5957 Aden	1FMW	1II0	1D0Y			Sc=6.21003, min distance = 0
ARSA1_ECOLX I	6338566 C	MYS2_DICDI Fu	5957 Aden	1FMW	1II0	1D1C			Sc=6.20542, min distance = 0
ARSA1_ECOLX I	65051 ddT	NDKC_DICDI Fu	6022 Aden	1KDN	1IHU	1F3F			Sc=6.42879, min distance = 1.37051
ARSA1_ECOLX I	6852207 M	KAPCA_BOVIN Fu	5957 Aden	1Q24	1II0	2GNI			Sc=5.93726, min distance = 2.38643
ARSA1_ECOLX I	91532 AMI	AROK_MYCTU Fu	6022 Aden	2IYV	1IHU	1ZYU		0.99	Sc=6.51903, min distance = 2.13684
ARSA1_ECOLX I	91532 AMI	BIOD_ECOLI Fu	5957 Aden	1A82	1II0	1DAG		0.99	Sc=6.46232, min distance = 2.59466
ARSA1_ECOLX I	91532 AMI	BIOD_ECOLI Fu	6022 Aden	1DAD	1IHU	1DAG		0.99	Sc=6.52283, min distance = 2.40025
ARSA1_ECOLX I	91532 AMI	KIF1A_MOUSE Fu	33113 gam	1VFV	1II9	1I6I		0.99	Sc=6.48569, min distance = 2.22984
ARSA1_ECOLX I	91532 AMI	NIFH1_AZOVI Fu	5957 Aden	2C8V	1II0	2AFK	37.5	0.99	Sc=6.48692, min distance = 2.64419
ARSA1_ECOLX I	91532 AMI	NIFH1_AZOVI Fu	6022 Aden	1FP6	1IHU	2AFK	37.5	0.99	Sc=6.54319, min distance = 1.45368
ASNA_ECOLI Fu	166760 Fa	PURP_METJA Fu	6083 aden	2R7M	12AS	2R7N		0.76	Sc=6.41284, min distance = 2.38847
ASNA_ECOLI Fu	21826754	PYGM_RABIT Fu	6083 aden	8GPB	12AS	3BD6			Sc=6.02956, min distance = 2.21440
ASNA_ECOLI Fu	24752834	SYH_ECOLI Ful	6083 aden	1HTT	12AS	2EL9			Sc=6.65003, min distance = 1.48982
ASNA_ECOLI Fu	24851689	PIM1_HUMAN Fu	6083 aden	1YXU	12AS	3CY2			Sc=6.17936, min distance = 1.96148
ASNA_ECOLI Fu	24905143	SRC_CHICK Ful	6083 aden	3DQX	12AS	3EN7			Sc=6.29215, min distance = 2.04814
ASNA_ECOLI Fu	24905152	SRC_CHICK Ful	6083 aden	3DQX	12AS	3EN6			Sc=6.3791, min distance = 2.149241
ASNA_ECOLI Fu	440641 1m	O57693_THETE I	6083 aden	1UXU	12AS	1UXR			Sc=5.94452, min distance = 2.16957
ASNA_ECOLI Fu	445708 AM	NADE_ECOLI Fu	6083 aden	1WXI	12AS	1WXE		0.91	Sc=6.35869, min distance = 2.18179
ASNA_ECOLI Fu	445736 5'	SYS_THET2 Ful	6083 aden	1SES	12AS	1SET		0.86	Sc=6.50635, min distance = 2.08303
ASNA_ECOLI Fu	446724 AM	NUDT5_HUMAN Fu	6083 aden	2DSD	12AS	3BM4		0.92	Sc=6.60632, min distance = 1.89315
ASNA_ECOLI Fu	448895 AD	UMPK_YEAST Fu	6083 aden	1UKZ	12AS	1UKY		0.99	Sc=6.42391, min distance = 2.63210
ASNA_ECOLI Fu	5327121 I	PIM1_HUMAN Fu	6083 aden	1YXU	12AS	2C3I			Sc=6.26593, min distance = 2.22660
ASNA_ECOLI Fu	5957 Ader	AAKG1_RAT Ful	6083 aden	2V8Q	12AS	2V92		0.98	Sc=6.48439, min distance = 1.64673
ASNA_ECOLI Fu	6022 Ader	HSP71_HUMAN Fu	6083 aden	1XQS	12AS	1S3X		0.99	Sc=6.43746, min distance = 2.04677
ASNA_ECOLI Fu	611002 Op	PIM1_HUMAN Fu	6083 aden	1YXU	12AS	1YXX			Sc=6.24271, min distance = 1.46134
ASNA_ECOLI Fu	65533 cor	O57693_THETE I	6083 aden	1UXU	12AS	1UXT			Sc=5.9636, min distance = 2.013420
ASNA_ECOLI Fu	657135 Op	PIM1_HUMAN Fu	6083 aden	1YXU	12AS	1YXV			Sc=5.92188, min distance = 3.18829
ASNA_ECOLI Fu	8582 Inos	PURA1_MOUSE Fu	6083 aden	1MF0	12AS	1IWE		0.79	Sc=6.37139, min distance = 2.27122
ASNA_ECOLI Fu	8582 Inos	PYGM_RABIT Fu	6083 aden	8GPB	12AS	2QN7		0.79	Sc=6.37056, min distance = 2.64787

ASNA_ECOLI F	91557 1tm	BLS_STRCL Ful	6083 aden	1MC1	12AS	1JGT	0.97	Sc=6.04897, min distance = 1.350368
ASNA_ECOLI F	97297 5-I	HASP_HUMAN Fu	6083 aden	3DLZ	12AS	2VUW	0.75	Sc=6.3044, min distance = 2.3639437
ASNB_ECOLI F	11986113	PIM1_HUMAN Fu	6083 aden	1YXU	1CT9	2BZH		Sc=6.40795, min distance = 2.139454
ASNB_ECOLI F	1400 nche	RET_HUMAN Ful	6083 aden	2IVT	1CT9	2IVV		Sc=6.15985, min distance = 2.820901
ASNB_ECOLI F	16058647	PIM1_HUMAN Fu	6083 aden	1YXU	1CT9	2OI4		Sc=6.41427, min distance = 2.147689
ASNB_ECOLI F	24905143	SRC_CHICK Ful	6083 aden	3DQX	1CT9	3EN7		Sc=6.21999, min distance = 2.466027
ASNB_ECOLI F	24905152	SRC_CHICK Ful	6083 aden	3DQX	1CT9	3EN6		Sc=6.30498, min distance = 2.545628
ASNB_ECOLI F	24905153	SRC_CHICK Ful	6083 aden	3DQX	1CT9	3EN5		Sc=6.26141, min distance = 2.488187
ASNB_ECOLI F	33032 L-g	SYQ_ECOLI Ful	5961 L-gl	1ZJW	1CT9	1O0C	0.84	Sc=5.76107, min distance = 2.088911
ASNB_ECOLI F	3973 nche	PIM1_HUMAN Fu	6083 aden	1YXU	1CT9	1YI3		Sc=5.91778, min distance = 1.390735
ASNB_ECOLI F	445708 AM	NADE_ECOLI Fu	6083 aden	1WXI	1CT9	1WXE	0.91	Sc=5.8907, min distance = 2.5618401
ASNB_ECOLI F	6022 Ader	PSPF_ECOLI Fu	6083 aden	2VII	1CT9	2C98	0.99	Sc=6.42092, min distance = 2.061627
ASNB_ECOLI F	6076 Cycl	PDE10_HUMAN F	6083 aden	2OUN	1CT9	2OUR	0.99	Sc=6.28585, min distance = 2.164551
ASNB_ECOLI F	656964 1y	PDE4D_HUMAN F	6083 aden	1TB7	1CT9	1Y2C		Sc=5.973, min distance = 1.8732808
ASNB_ECOLI F	656968 1y	PDE4B_HUMAN F	6083 aden	1ROR	1CT9	1Y2H		Sc=6.01252, min distance = 2.069732
ASNB_ECOLI F	657135 Op	PIM1_HUMAN Fu	6083 aden	1YXU	1CT9	1YXV		Sc=5.87387, min distance = 1.850981
ASNB_ECOLI F	8582 Inos	PYGM_RABIT Fu	6083 aden	8GPB	1CT9	2QN7	0.79	Sc=5.88964, min distance = 2.893081
ASPD_ARCFU F	12136 Ber	POL_HV1N5 Ful	311 citri	2GON	2DC1	9HVP		Sc=5.63009, min distance = 62.96670
ASPD_ARCFU F	123927 A3	Q9GT92_CRYPV	5893 nadi	2FM3	2DC1	2EWD	0.97	Sc=6.74448, min distance = 2.277444
ASPD_ARCFU F	439153 Di	ADH1B_HUMAN F	5893 nadi	1U3U	2DC1	1DEH	0.79	Sc=6.74741, min distance = 1.950801
ASPD_ARCFU F	439153 Di	CTBP1_RAT Ful	5893 nadi	1HKU	2DC1	1HL3	0.79	Sc=6.73957, min distance = 2.161505
ASPD_ARCFU F	439183 1i	TPIS_TRYBB Fu	311 citri	2VEK	2DC1	1IIH		Sc=5.62225, min distance = 2.053911
ASPD_ARCFU F	440516 CI	MDH_THETH Ful	5893 nadi	1BMD	2DC1	1BDM	0.79	Sc=6.74727, min distance = 2.138712
ASPD_ARCFU F	444212 tr	ACON_BOVIN Fu	311 citri	1C96	2DC1	1ACO		Sc=5.86465, min distance = 2.086130
ASPD_ARCFU F	445607 AT	ACON_BOVIN Fu	311 citri	1C96	2DC1	1FGH		Sc=5.90497, min distance = 2.106146
ASPD_ARCFU F	445794 AD	ADHX_HUMAN Fu	5893 nadi	2FZW	2DC1	2FZE		Sc=6.61256, min distance = 1.733187
ASPD_ARCFU F	446288 NA	G3P_PALVE Ful	5893 nadi	1DSS	2DC1	1IHX		Sc=6.80976, min distance = 0.897636
ASPD_ARCFU F	447376 2-	ACON_BOVIN Fu	311 citri	1C96	2DC1	1NIS		Sc=5.83194, min distance = 1.884546
ASPD_ARCFU F	447550 ph	TPIS_TRYBB Fu	311 citri	2VEK	2DC1	4TIM		Sc=5.61286, min distance = 1.932350
ASPD_ARCFU F	449604 NI	ACON_BOVIN Fu	311 citri	1C96	2DC1	8ACN		Sc=5.83003, min distance = 2.040972
ASPD_ARCFU F	6083 ader	NADE_ECOLI Fu	5893 nadi	1WXH	2DC1	1WXI		Sc=5.85048, min distance = 2.519064
ASPD_THEMA F	11987786	LDH_PLAFD Ful	5893 nadi	1T2D	1J5P	1T2E	0.77	Sc=6.75356, min distance = 2.372302
ASPD_THEMA F	439153 Di	ADH1B_HUMAN F	5893 nadi	1U3U	1J5P	1DEH	0.79	Sc=6.44845, min distance = 1.971263
ASPD_THEMA F	439153 Di	ADH1E_HORSE F	5893 nadi	1MGO	1J5P	2JHF	0.79	Sc=6.31424, min distance = 2.134171
ASPD_THEMA F	439153 Di	CTBP1_RAT Ful	5893 nadi	1HKU	1J5P	1HL3	0.79	Sc=6.3224, min distance = 2.1120847
ASPD_THEMA F	440516 CI	MDH_THETH Ful	5893 nadi	1BMD	1J5P	1BDM	0.79	Sc=6.76115, min distance = 2.267669
ASPD_THEMA F	445794 AD	ADHX_HUMAN Fu	5893 nadi	2FZW	1J5P	2FZE		Sc=6.17683, min distance = 1.578493

ASSY_ECOLI F	11708454	KAPCA_BOVIN F	5957 Aden	1Q24	1KP2	2VNW			Sc=6.14068, min distance = 2.67485
ASSY_ECOLI F	3540 1yds	KAPCA_BOVIN F	5957 Aden	1Q24	1KP2	1YDS			Sc=5.88274, min distance = 2.27579
ASSY_ECOLI F	3547 Fasu	KAPCA_BOVIN F	5957 Aden	1Q24	1KP2	1Q8W			Sc=5.92776, min distance = 2.08902
ASSY_ECOLI F	3973 nche	PK3CG_PIG Ful	5957 Aden	1E8X	1KP2	1E7V			Sc=5.94727, min distance = 2.04007
ASSY_ECOLI F	444852 CI	ATPB_BOVIN Fu	5957 Aden	2V7Q	1KP2	1COW		0.91	Sc=5.68644, min distance = 1.92586
ASSY_ECOLI F	447916 ac	RIO1_ARCFU Fu	5957 Aden	1ZP9	1KP2	1ZTF		0.94	Sc=5.82148, min distance = 2.70292
ASSY_ECOLI F	448043 2g	KAPCA_BOVIN F	5957 Aden	1Q24	1KP2	1Q8U			Sc=6.03636, min distance = 2.01936
ASSY_ECOLI F	6022 Ader	BIOD_ECOLI Fu	5957 Aden	1A82	1KP2	1DAD		0.99	Sc=5.6439, min distance = 2.216491
ASSY_ECOLI F	6022 Ader	DDL_THET8 Ful	5957 Aden	2ZDQ	1KP2	2ZDH		0.99	Sc=5.65946, min distance = 1.50704
ASSY_ECOLI F	6022 Ader	PSPF_ECOLI Fu	5957 Aden	2C96	1KP2	2C98		0.99	Sc=5.62699, min distance = 2.35121
ASSY_ECOLI F	6022 Ader	PURP_METJA Fu	5957 Aden	2R7L	1KP2	2R7N		0.99	Sc=5.94707, min distance = 2.16720
ASSY_ECOLI F	6022 Ader	Y059_METJA Fu	5957 Aden	2J9C	1KP2	2J9D		0.99	Sc=5.93497, min distance = 1.89405
ASSY_ECOLI F	6083 ader	PURP_METJA Fu	5957 Aden	2R7L	1KP2	2R7M		0.98	Sc=6.3486, min distance = 2.004018
ASSY_ECOLI F	6083 ader	PURP_PYRFU Fu	5957 Aden	2R86	1KP2	2R85		0.98	Sc=6.31462, min distance = 2.46182
ASSY_ECOLI F	6322 L-ar	ASSY_THET8 Fu	9750 citr	1J1Z	1K97	1KOR		0.78	Sc=5.89649, min distance = 2.39803
ASSY_ECOLI F	6852207 M	KAPCA_BOVIN F	5957 Aden	1Q24	1KP2	2GNI			Sc=5.96825, min distance = 2.25433
ASSY_HUMAN F	6322 L-ar	ASSY_THET8 Fu	9750 citr	1J1Z	2NZ2	1KOR		0.78	Sc=5.89649, min distance = 2.63742
ASSY_THET8 F	123831 N,	NOS3_BOVIN Fu	6322 L-ar	4NSE	1KOR	7NSE		0.89	Sc=6.02895, min distance = 1.96263
ASSY_THET8 F	123895 1h	NOS3_BOVIN Fu	6322 L-ar	4NSE	1KOR	5NSE		0.96	Sc=5.96212, min distance = 2.19127
ASSY_THET8 F	15602983	KAPCA_BOVIN F	5957 Aden	1Q24	1J1Z	2UW5			Sc=6.12927, min distance = 2.43417
ASSY_THET8 F	16122632	KAPCA_BOVIN F	5957 Aden	1Q24	1J1Z	2UVZ			Sc=6.37391, min distance = 2.14177
ASSY_THET8 F	16122635	KAPCA_BOVIN F	5957 Aden	1Q24	1J1Z	2UW8			Sc=5.7236, min distance = 1.978789
ASSY_THET8 F	1893 7-ni	NOS3_BOVIN Fu	6322 L-ar	4NSE	1KOR	1FOJ			Sc=5.74654, min distance = 1.49868
ASSY_THET8 F	21826754	PYGM_RABIT Fu	6083 aden	8GPB	1J20	3BD6			Sc=5.6324, min distance = 2.105531
ASSY_THET8 F	24762195	KAPCA_BOVIN F	5957 Aden	1Q24	1J1Z	2VO0			Sc=6.30014, min distance = 1.88201
ASSY_THET8 F	24851689	PIM1_HUMAN Fu	6083 aden	1YXU	1J20	3CY2			Sc=6.17612, min distance = 2.18864
ASSY_THET8 F	24905143	SRC_CHICK Ful	6083 aden	3DQX	1J20	3EN7			Sc=6.27324, min distance = 1.73205
ASSY_THET8 F	2519 caff	PYGM_RABIT Fu	6083 aden	8GPB	1J20	1GFZ			Sc=5.80598, min distance = 2.20017
ASSY_THET8 F	439202 L-	NOS3_BOVIN Fu	6322 L-ar	4NSE	1KOR	6NSE			Sc=5.80881, min distance = 1.96389
ASSY_THET8 F	444288 CI	KARG_LIMPO Fu	6322 L-ar	1M15	1KOR	1P52			Sc=5.79287, min distance = 2.25945
ASSY_THET8 F	444564 AD	BIOD_ECOLI Fu	5957 Aden	1A82	1J1Z	1BS1		0.91	Sc=5.91571, min distance = 1.37635
ASSY_THET8 F	444797 Be	CBP2_WHEAT Fu	6322 L-ar	1BCS	1KOR	1WHT			Sc=6.11588, min distance = 2.04385
ASSY_THET8 F	445432 CI	SYQ_ECOLI Ful	6083 aden	1ZJW	1J20	1QTQ		0.84	Sc=6.09757, min distance = 1.86951
ASSY_THET8 F	445708 AM	NADE_ECOLI Fu	6083 aden	1WXI	1J20	1WXE		0.91	Sc=6.33718, min distance = 2.11700
ASSY_THET8 F	447181 VI	NOS1_RAT Full	6322 L-ar	1OM4	1KOR	1MMW		0.78	Sc=6.07004, min distance = 1.82123
ASSY_THET8 F	447916 ac	RIO1_ARCFU Fu	5957 Aden	1ZP9	1J1Z	1ZTF		0.94	Sc=6.24707, min distance = 2.25973
ASSY_THET8 F	448042 2e	KAPCA_BOVIN F	5957 Aden	1Q24	1J1Z	1Q8T			Sc=6.14764, min distance = 1.58813

ASSY_THET8 F	448895 AD	UMPK_YEAST Fu	6083 aden	1UKZ	1J20	1UKY		0.99	Sc=5.93294, min distance = 2.49471
ASSY_THET8 F	5326977 1	CSK2A_MAIZE F	33113 gam	1LP4	1KOR	1ZOG			Sc=5.67883, min distance = 2.27321
ASSY_THET8 F	6022 Ader	CLCN5_HUMAN F	5957 Aden	2J9L	1J1Z	2JA3		0.99	Sc=6.40508, min distance = 1.25256
ASSY_THET8 F	6022 Ader	DDL_THET8 Ful	5957 Aden	2ZDQ	1J1Z	2ZDH		0.99	Sc=6.3757, min distance = 2.475870
ASSY_THET8 F	6022 Ader	FAK1_HUMAN Fu	5957 Aden	2IJM	1J1Z	1MP8		0.99	Sc=5.91441, min distance = 2.53779
ASSY_THET8 F	6022 Ader	GLGS_SOLTU Fu	5957 Aden	1YP3	1J1Z	1YP4		0.99	Sc=6.35552, min distance = 1.95858
ASSY_THET8 F	6022 Ader	PDXK_SHEEP Fu	5957 Aden	1LHR	1J1Z	1RFU		0.99	Sc=5.9586, min distance = 1.861369
ASSY_THET8 F	6022 Ader	PSPF_ECOLI Fu	6083 aden	2VII	1J20	2C98		0.99	Sc=5.95358, min distance = 1.81030
ASSY_THET8 F	6022 Ader	PUR7_METJA Fu	5957 Aden	2Z02	1J1Z	2YZL		0.99	Sc=5.9208, min distance = 1.439952
ASSY_THET8 F	6022 Ader	PURP_METJA Fu	6083 aden	2R7M	1J20	2R7N		0.99	Sc=5.75369, min distance = 2.59449
ASSY_THET8 F	6022 Ader	SKY1_YEAST Fu	5957 Aden	1Q97	1J1Z	1Q8Y		0.99	Sc=5.92286, min distance = 2.05989
ASSY_THET8 F	6076 Cycl	PDE10_HUMAN F	6083 aden	2OUN	1J20	2OUR		0.99	Sc=6.30124, min distance = 2.03082
ASSY_THET8 F	60961 ade	SYQ_ECOLI Ful	5957 Aden	1GTR	1J1Z	1O0B		0.94	Sc=6.28604, min distance = 2.21708
ASSY_THET8 F	6102784 A	PSPF_ECOLI Fu	6083 aden	2VII	1J20	2C99		0.97	Sc=5.98679, min distance = 2.06604
ASSY_THET8 F	6176 Cyti	PYRI_ECOLI Fu	5957 Aden	7AT1	1J1Z	2FZC			Sc=6.37561, min distance = 1.51846
ASSY_THET8 F	6274 hist	ARGT_SALTY Fu	6322 L-ar	1LAF	1KOR	1LAG			Sc=5.83983, min distance = 2.70642
ASSY_THET8 F	6419789 C	HASP_HUMAN Fu	6083 aden	3DLZ	1J20	3E7V			Sc=6.29556, min distance = 1.82622
ASSY_THET8 F	656964 1y	PDE4D_HUMAN F	6083 aden	1TB7	1J20	1Y2C			Sc=5.94462, min distance = 1.90478
ASSY_THET8 F	656966 1y	PDE4D_HUMAN F	6083 aden	1TB7	1J20	1Y2E			Sc=6.08183, min distance = 1.96034
ASSY_THET8 F	656968 1y	PDE4B_HUMAN F	6083 aden	1ROR	1J20	1Y2H			Sc=5.973, min distance = 1.8883627
ASSY_THET8 F	6804 guar	PDE10_HUMAN F	6083 aden	2OUN	1J20	2OUQ		0.8	Sc=5.90232, min distance = 0
ASSY_THET8 F	6914614 p	KAPCA_BOVIN F	5957 Aden	1Q24	1J1Z	2F7Z			Sc=6.41552, min distance = 2.25203
ASSY_THET8 F	8582 Inos	PURA1_MOUSE F	6083 aden	1MF0	1J20	1IWE		0.79	Sc=5.94201, min distance = 0.95101
ASSY_THET8 F	8977 1dar	NDK_THET8 Ful	5957 Aden	1WKL	1J1Z	1WKK		0.8	Sc=5.98641, min distance = 1.76826
ASSY_THET8 F	9085 homo	NOS3_BOVIN Fu	6322 L-ar	4NSE	1KOR	1DM7		0.9	Sc=5.95314, min distance = 2.12071
ASSY_THET8 F	91532 AMP	INSR_HUMAN Fu	5957 Aden	3BU5	1J1Z	1I44		0.99	Sc=5.96167, min distance = 0.72665
ASSY_THET8 F	91532 AMP	SYK2_ECOLI Fu	5957 Aden1E24		1J1Z	1E22		0.99	Sc=6.00691, min distance = 1.99579
ASSY_THET8 F	91557 1tm	BLS_STRCL Ful	6083 aden	1MC1	1J20	1JGT		0.97	Sc=6.03178, min distance = 2.22000
ATGT_PYRHO F	100684 9-	TGT_ZYMMO Ful	446357 7-	1P0B	1IT8	1Q2R	28.41		Sc=5.87327, min distance = 2.53410
ATGT_PYRHO F	100684 9-	TGT_ZYMMO Ful	764 guani	2PWU	1IT7	1Q2R	28.41		Sc=5.85754, min distance = 2.49166
ATGT_PYRHO F	108714 9-	URIC_ASPFL Fu	764 guani	1XT4	1IT7	1R4S			Sc=5.93455, min distance = 2.48533
ATGT_PYRHO F	11287125	TGT_ZYMMO Ful	446357 7-	1P0B	1IT8	2BBF	28.41		Sc=6.19015, min distance = 2.33694
ATGT_PYRHO F	1188 xant	XGPT_ECOLI Fu	764 guani	1A95	1IT7	1A96		0.92	Sc=5.83226, min distance = 2.71061
ATGT_PYRHO F	15113 Xar	URIC_ASPFL Fu	764 guani	1XT4	1IT7	2IBA			Sc=5.78752, min distance = 2.77359
ATGT_PYRHO F	16758215	TGT_ZYMMO Ful	446357 7-	1P0B	1IT8	2PWV	28.41		Sc=6.03149, min distance = 2.65730
ATGT_PYRHO F	16758215	TGT_ZYMMO Ful	764 guani	2PWU	1IT7	2PWV	28.41		Sc=6.02019, min distance = 2.63749
ATGT_PYRHO F	242831 2-	TGT_ZYMMO Ful	446357 7-	1P0B	1IT8	1S39	28.41		Sc=5.96192, min distance = 2.71387

ATGT_PYRHO Fu	242831 2-	TGT_ZYMMO Full	764 guani	2PWU	1IT7	1S39	28.41		Sc=5.93349, min distance = 2.545878
ATGT_PYRHO Fu	24741806	TGT_ZYMMO Full	446357 7-	1P0B	1IT8	2Z7K	28.41		Sc=6.19713, min distance = 2.274391
ATGT_PYRHO Fu	24741806	TGT_ZYMMO Full	764 guani	2PWU	1IT7	2Z7K	28.41		Sc=6.17753, min distance = 2.031564
ATGT_PYRHO Fu	445390 CI	TGT_ZYMMO Full	446357 7-	1P0B	1IT8	1ENU	28.41		Sc=6.0599, min distance = 2.2508245
ATGT_PYRHO Fu	445497 CI	TGT_ZYMMO Full	446357 7-	1P0B	1IT8	1F3E	28.41		Sc=6.12964, min distance = 2.215216
ATGT_PYRHO Fu	448010 1q	TGT_ZYMMO Full	446357 7-	1P0B	1IT8	1R5Y	28.41		Sc=6.05555, min distance = 2.087695
ATGT_PYRHO Fu	448488 1s	TGT_ZYMMO Full	446357 7-	1P0B	1IT8	1S38	28.41		Sc=6.01577, min distance = 2.528787
ATGT_PYRHO Fu	448488 1s	TGT_ZYMMO Full	764 guani	2PWU	1IT7	1S38	28.41		Sc=5.99636, min distance = 2.499671
ATGT_PYRHO Fu	5287547 6	PURR_ECOLI Fu	764 guani	1WET	1IT7	2PUA			Sc=5.74009, min distance = 2.558081
ATGT_PYRHO Fu	5326622 C	URIC_ASPFL Fu	764 guani	1XT4	1IT7	3BJP			Sc=5.88294, min distance = 2.434548
ATGT_PYRHO Fu	5326930 E	TGT_ZYMMO Full	446357 7-	1P0B	1IT8	1Y5X	28.41		Sc=6.51401, min distance = 2.048017
ATGT_PYRHO Fu	76726 5,6	URIC_ASPFL Fu	764 guani	1XT4	1IT7	1WS2			Sc=5.727, min distance = 2.5255371
ATGT_PYRHO Fu	8646 Guar	PNPH_HUMAN Fu	764 guani	1V2H	1IT7	1V41			Sc=5.77524, min distance = 2.629669
ATLE_AGRAE Fu	440552 1b	LECA_ARTIN Fu	439353 be	1UGX	1WW6	1UH1			Sc=5.83941, min distance = 2.679142
ATPA1_BOVIN I	11608401	KAPCA_BOVIN Fu	5957 Aden	1Q24	2V7Q	2UVY			Sc=6.10197, min distance = 2.738410
ATPA1_BOVIN I	11708454	KAPCA_BOVIN Fu	5957 Aden	1Q24	2V7Q	2VNW			Sc=6.07469, min distance = 2.747813
ATPA1_BOVIN I	1400 nche	HCK_HUMAN Full	33113 gam	1AD5	2JDI	1QCF			Sc=6.22073, min distance = 1.727656
ATPA1_BOVIN I	16750062	CSK2A_MAIZE Fu	33113 gam	1LP4	2JDI	2OXD			Sc=5.65416, min distance = 2.679893
ATPA1_BOVIN I	24779678	CSK2A_MAIZE Fu	33113 gam	1LP4	2JDI	2PVM			Sc=6.05529, min distance = 2.291272
ATPA1_BOVIN I	3547 Fasu	KAPCA_BOVIN Fu	5957 Aden	1Q24	2V7Q	1Q8W			Sc=5.92628, min distance = 2.011188
ATPA1_BOVIN I	3547 Fasu	ROCK1_HUMAN Fu	33113 gam	2V55	2JDI	2ESM			Sc=5.88549, min distance = 2.325363
ATPA1_BOVIN I	444564 AD	BIOD_ECOLI Fu	5957 Aden	1A82	2V7Q	1BS1		0.91	Sc=6.39408, min distance = 2.389860
ATPA1_BOVIN I	444564 AD	MYS2_DICDI Fu	33113 gam	1MMN	2JDI	1W9I		0.91	Sc=5.9855, min distance = 2.6720510
ATPA1_BOVIN I	444564 AD	MYS2_DICDI Fu	5957 Aden	1FMW	2V7Q	1W9I		0.91	Sc=6.40759, min distance = 0
ATPA1_BOVIN I	447004 CI	MYS2_DICDI Fu	5957 Aden	1FMW	2V7Q	1LVK			Sc=5.9654, min distance = 0
ATPA1_BOVIN I	448042 2e	ROCK1_HUMAN Fu	33113 gam	2V55	2JDI	2ETR			Sc=6.16716, min distance = 1.443037
ATPA1_BOVIN I	448043 2g	ROCK1_HUMAN Fu	33113 gam	2V55	2JDI	3D9V			Sc=6.0221, min distance = 1.8105894
ATPA1_BOVIN I	449240 1y	KAPCA_BOVIN Fu	5957 Aden	1Q24	2V7Q	1YDR			Sc=5.87641, min distance = 1.972629
ATPA1_BOVIN I	5326976 1	CSK2A_MAIZE Fu	33113 gam	1LP4	2JDI	1ZOE			Sc=5.74615, min distance = 2.237759
ATPA1_BOVIN I	5326977 1	CSK2A_MAIZE Fu	33113 gam	1LP4	2JDI	1ZOG			Sc=5.60481, min distance = 1.903973
ATPA1_BOVIN I	6083 ader	AAKG1_RAT Full	5957 Aden	2V92	2V7Q	2V8Q		0.98	Sc=6.29766, min distance = 1.981800
ATPA1_BOVIN I	6083 ader	ENTP2_RAT Full	33113 gam	3CJA	2JDI	3CJ7		0.97	Sc=6.29104, min distance = 2.432431
ATPA1_BOVIN I	6083 ader	HSP71_HUMAN Fu	33113 gam	2E8A	2JDI	1XQS		0.97	Sc=6.32842, min distance = 2.298886
ATPA1_BOVIN I	6083 ader	Y059_METJA Fu	5957 Aden	2J9C	2V7Q	2J9D		0.98	Sc=5.82976, min distance = 1.865892
ATPA1_BOVIN I	60961 ade	SYQ_ECOLI Full	5957 Aden	1GTR	2V7Q	1O0B		0.94	Sc=5.78365, min distance = 2.341807
ATPA1_BOVIN I	6338561 C	MYS2_DICDI Fu	5957 Aden	1FMW	2V7Q	1D0X			Sc=6.18066, min distance = 0
ATPA1_BOVIN I	6338562 C	MYS2_DICDI Fu	5957 Aden	1FMW	2V7Q	1D0Y			Sc=6.18376, min distance = 0

ATPA1_BOVIN I	91532 AMF	BIOD_ECOLI Ful	5957 Aden	1A82	2V7Q	1DAG	0.99	Sc=6.4648, min distance = 2.367389
ATPA1_BOVIN I	91532 AMF	INSR_HUMAN Ful	5957 Aden	3BU5	2V7Q	1I44	0.99	Sc=6.05096, min distance = 2.150685
ATPA1_BOVIN I	91532 AMF	PURT_ECOLI Ful	33113 gam	1EYZ	2JDI	1KJI	0.99	Sc=6.06718, min distance = 1.459878
ATPA_RAT Ful	11608401	KAPCA_BOVIN Fu	5957 Aden	1Q24	1MAB	2UVY		Sc=5.99761, min distance = 2.141398
ATPA_RAT Ful	11708454	KAPCA_BOVIN Fu	5957 Aden	1Q24	1MAB	2VNW		Sc=5.9354, min distance = 2.760625
ATPA_RAT Ful	447840 CI	PHKG1_RABIT Fu	5957 Aden	1QL6	1MAB	1PHK		Sc=5.66514, min distance = 2.582360
ATPA_RAT Ful	6083 aden	PURP_PYRFU Ful	5957 Aden	2R86	1MAB	2R85	0.98	Sc=5.80817, min distance = 2.217727
ATPA_THEMA Fu	11608401	KAPCA_BOVIN Fu	5957 Aden	1Q24	2R9V	2UVY		Sc=6.09154, min distance = 2.662646
ATPA_THEMA Fu	11708454	KAPCA_BOVIN Fu	5957 Aden	1Q24	2R9V	2VNW		Sc=6.05018, min distance = 2.610401
ATPA_THEMA Fu	16231 AMI	UROK_HUMAN Ful	8200 TETR	2O8U	2R9V	1F5L		Sc=5.67712, min distance = 2.359512
ATPA_THEMA Fu	3540 1yds	KAPCA_BOVIN Fu	5957 Aden	1Q24	2R9V	1YDS		Sc=5.7897, min distance = 2.123766
ATPA_THEMA Fu	3547 Fasu	KAPCA_BOVIN Fu	5957 Aden	1Q24	2R9V	1Q8W		Sc=5.89227, min distance = 2.072873
ATPA_THEMA Fu	36735 Gpp	Q9RVK2_DEIRA I	5957 Aden	1SU2	2R9V	1SZ3	0.8	Sc=5.99947, min distance = 2.095901
ATPA_THEMA Fu	444852 CI	ATPA1_BOVIN Fu	5957 Aden	2V7Q	2R9V	1COW	0.91	Sc=6.47139, min distance = 2.177761
ATPA_THEMA Fu	449240 1y	KAPCA_BOVIN Fu	5957 Aden	1Q24	2R9V	1YDR		Sc=5.86061, min distance = 2.142927
ATPA_THEMA Fu	6022 Aden	ARSA1_ECOLX Fu	5957 Aden	1II0	2R9V	1IHU	0.99	Sc=6.39549, min distance = 2.545589
ATPA_THEMA Fu	6022 Aden	BIOD_ECOLI Ful	5957 Aden	1A82	2R9V	1DAD	0.99	Sc=6.0266, min distance = 2.585869
ATPA_THEMA Fu	6022 Aden	HSP7F_YEAST Fu	5957 Aden	3D2F	2R9V	3C7N	0.99	Sc=6.01522, min distance = 2.288992
ATPA_THEMA Fu	6022 Aden	MUTS_ECOLI Ful	5957 Aden	1W7A	2R9V	1OH7	0.99	Sc=6.03877, min distance = 2.488609
ATPA_THEMA Fu	6022 Aden	PURK_ECOLI Ful	5957 Aden	3ETH	2R9V	3ETJ	0.99	Sc=6.37786, min distance = 2.487029
ATPA_THEMA Fu	6022 Aden	Q72H90_THET2 I	5957 Aden	2BEK	2R9V	2BEJ	0.99	Sc=6.02948, min distance = 2.634783
ATPA_THEMA Fu	6022 Aden	Y059_METJA Ful	5957 Aden	2J9C	2R9V	2J9D	0.99	Sc=6.37893, min distance = 1.639604
ATPA_THEMA Fu	6083 aden	Y059_METJA Ful	5957 Aden	2J9C	2R9V	2J9D	0.98	Sc=5.82651, min distance = 1.972178
ATPA_THEMA Fu	6338561 C	MYS2_DICDI Ful	5957 Aden	1FMW	2R9V	1D0X		Sc=6.15821, min distance = 0
ATPA_THEMA Fu	91532 AMF	INSR_HUMAN Ful	5957 Aden	3BU5	2R9V	1I44	0.99	Sc=6.04483, min distance = 2.029575
ATPA_THEMA Fu	91532 AMF	NIFH1_AZOVI Fu	5957 Aden	2C8V	2R9V	2AFK	0.99	Sc=6.14514, min distance = 2.374001
ATPA_YEAST Fu	16750062	CSK2A_MAIZE Fu	33113 gam	1LP4	2HLD	2OXD		Sc=5.63148, min distance = 2.654609
ATPA_YEAST Fu	24779674	CSK2A_MAIZE Fu	33113 gam	1LP4	2HLD	2PVH		Sc=6.10623, min distance = 2.193431
ATPA_YEAST Fu	3064778 H	ROCK1_HUMAN Fu	33113 gam	2V55	2HLD	2ETK		Sc=6.08984, min distance = 1.754055
ATPA_YEAST Fu	3547 Fasu	ROCK1_HUMAN Fu	33113 gam	2V55	2HLD	2ESM		Sc=5.84496, min distance = 1.999226
ATPA_YEAST Fu	444564 AD	MYS2_DICDI Ful	33113 gam	1MMN	2HLD	1W9I	0.91	Sc=6.03955, min distance = 2.514058
ATPA_YEAST Fu	448042 2e	ROCK1_HUMAN Fu	33113 gam	2V55	2HLD	2ETR		Sc=6.14361, min distance = 2.055192
ATPA_YEAST Fu	5327148 C	IPKA_RABIT Ful	33113 gam	1CDK	2HLD	2ERZ		Sc=6.10086, min distance = 1.800872
ATPA_YEAST Fu	6083 aden	Q6L8F0_THETH I	33113 gam	1V25	2HLD	1V26	0.97	Sc=5.79679, min distance = 2.084673
ATPB_BOVIN Fu	1400 nche	HCK_HUMAN Ful	33113 gam	1AD5	2JDI	1QCF		Sc=6.29006, min distance = 2.275330
ATPB_BOVIN Fu	1540 1h1q	CDK2_HUMAN Ful	6022 Aden	1GY3	2CK3	1H1Q		Sc=6.29397, min distance = 2.035362
ATPB_BOVIN Fu	16750062	CSK2A_MAIZE Fu	33113 gam	1LP4	2JDI	2OXD		Sc=5.73038, min distance = 2.533661

ATPB_BOVIN F	188966 dA	HSLU_ECOLI Fu	33113 gam	1E94	2JDI	1G4A	0.97	Sc=5.9442, min distance = 2.309852
ATPB_BOVIN F	188966 dA	PAPS1_HUMAN F	6022 Aden	1X6V	2CK3	2PEZ	0.98	Sc=5.9266, min distance = 2.281785
ATPB_BOVIN F	24779675	CSK2A_MAIZE F	33113 gam	1LP4	2JDI	2PVJ		Sc=6.35842, min distance = 2.094279
ATPB_BOVIN F	24779676	CSK2A_MAIZE F	33113 gam	1LP4	2JDI	2PVK		Sc=6.36494, min distance = 2.159246
ATPB_BOVIN F	24779677	CSK2A_MAIZE F	33113 gam	1LP4	2JDI	2PVL		Sc=6.28446, min distance = 2.135414
ATPB_BOVIN F	3547 Fasu	ROCK1_HUMAN F	33113 gam	2V55	2JDI	2ESM		Sc=5.7673, min distance = 2.267712
ATPB_BOVIN F	440317 AT	FTSK_PSEAE Fu	6022 Aden	2IUU	2CK3	2IUT	0.99	Sc=5.73121, min distance = 2.26453
ATPB_BOVIN F	444564 AD	BIOD_ECOLI Fu	6022 Aden	1DAD	2CK3	1BS1	0.91	Sc=5.67454, min distance = 2.230654
ATPB_BOVIN F	444564 AD	MYS2_DICDI Fu	33113 gam	1MMN	2JDI	1W9I	0.91	Sc=5.68261, min distance = 2.298624
ATPB_BOVIN F	444564 AD	MYS2_DICDI Fu	6022 Aden	1VOM	2CK3	1W9I	0.91	Sc=5.66539, min distance = 2.306402
ATPB_BOVIN F	447052 CI	CSK2A_MAIZE F	33113 gam	1LP4	2JDI	1M2R		Sc=6.34712, min distance = 2.395820
ATPB_BOVIN F	447649 1c	CDK2_HUMAN Fu	6022 Aden	1GY3	2CK3	1OI9		Sc=6.36887, min distance = 2.08789
ATPB_BOVIN F	447655 1c	CDK2_HUMAN Fu	6022 Aden	1GY3	2CK3	1OIU		Sc=6.29307, min distance = 2.11397
ATPB_BOVIN F	447962 1p	CDK2_HUMAN Fu	6022 Aden	1GY3	2CK3	2C5N		Sc=6.21543, min distance = 1.633880
ATPB_BOVIN F	4565 1h1r	CDK2_HUMAN Fu	6022 Aden	1GY3	2CK3	1H1R		Sc=6.34884, min distance = 2.514644
ATPB_BOVIN F	4566 1h1s	CDK2_HUMAN Fu	6022 Aden	1GY3	2CK3	1H1S		Sc=6.3012, min distance = 2.1519549
ATPB_BOVIN F	5149739 2	CSK2A_MAIZE F	33113 gam	1LP4	2JDI	2OXY		Sc=5.60151, min distance = 2.370710
ATPB_BOVIN F	5326739 i	GSK3B_HUMAN F	33113 gam	1J1B	2JDI	1Q41		Sc=6.30549, min distance = 1.555838
ATPB_BOVIN F	5326739 i	GSK3B_HUMAN F	6022 Aden	1J1C	2CK3	1Q41		Sc=6.32019, min distance = 2.082512
ATPB_BOVIN F	5326976 1	CSK2A_MAIZE F	33113 gam	1LP4	2JDI	1ZOE		Sc=5.77241, min distance = 2.067602
ATPB_BOVIN F	5326977 1	CSK2A_MAIZE F	33113 gam	1LP4	2JDI	1ZOG		Sc=5.65339, min distance = 2.048559
ATPB_BOVIN F	5326978 1	CSK2A_MAIZE F	33113 gam	1LP4	2JDI	1ZOH		Sc=5.66786, min distance = 2.269170
ATPB_BOVIN F	6338561 C	MYS2_DICDI Fu	6022 Aden	1VOM	2CK3	1D0X		Sc=6.16624, min distance = 2.145475
ATPB_BOVIN F	6420139 C	CDK2_HUMAN Fu	6022 Aden	1GY3	2CK3	2C5V		Sc=6.20108, min distance = 1.351607
ATPB_BOVIN F	91532 AMF	BIOD_ECOLI Fu	6022 Aden	1DAD	2CK3	1DAG	0.99	Sc=5.75507, min distance = 2.36760
ATPB_BOVIN F	9547890 I	CDK2_HUMAN Fu	6022 Aden	1GY3	2CK3	1W8C		Sc=6.14957, min distance = 2.059049
ATPB_RAT Ful	4565 1h1r	CDK2_HUMAN Fu	6022 Aden	1GY3	1MAB	1H1R		Sc=6.08416, min distance = 1.869454
ATPB_RAT Ful	91532 AMF	BIOD_ECOLI Fu	6022 Aden	1DAD	1MAB	1DAG	0.99	Sc=6.05217, min distance = 2.017184
ATPB_RAT Ful	9547890 I	CDK2_HUMAN Fu	6022 Aden	1GY3	1MAB	1W8C		Sc=5.88621, min distance = 3.01764
ATPB_YEAST F	16750062	CSK2A_MAIZE F	33113 gam	1LP4	2HLD	2OXD		Sc=5.7338, min distance = 2.2161635
ATPB_YEAST F	24779674	CSK2A_MAIZE F	33113 gam	1LP4	2HLD	2PVH		Sc=6.22967, min distance = 2.487674
ATPB_YEAST F	24779675	CSK2A_MAIZE F	33113 gam	1LP4	2HLD	2PVJ		Sc=6.3487, min distance = 2.3577832
ATPB_YEAST F	24779676	CSK2A_MAIZE F	33113 gam	1LP4	2HLD	2PVK		Sc=6.40725, min distance = 2.36748
ATPB_YEAST F	24779677	CSK2A_MAIZE F	33113 gam	1LP4	2HLD	2PVL		Sc=6.34032, min distance = 2.344538
ATPB_YEAST F	3547 Fasu	ROCK1_HUMAN F	33113 gam	2V55	2HLD	2ESM		Sc=5.7673, min distance = 2.5769837
ATPB_YEAST F	447052 CI	CSK2A_MAIZE F	33113 gam	1LP4	2HLD	1M2R		Sc=6.33044, min distance = 2.095850
ATPB_YEAST F	448042 2e	ROCK1_HUMAN F	33113 gam	2V55	2HLD	2ETR		Sc=6.17552, min distance = 1.757079

ATPB_YEAST F	5149739 2	CSK2A_MAIZE F	33113 gam	1LP4	2HLD	2OXY			Sc=5.60848, min distance = 2.17524
ATPB_YEAST F	5326739 i	GSK3B_HUMAN F	33113 gam	1J1B	2HLD	1Q41			Sc=6.30199, min distance = 1.68716
ATPB_YEAST F	5326977 1	CSK2A_MAIZE F	33113 gam	1LP4	2HLD	1ZOG			Sc=5.67711, min distance = 1.98287
ATPB_YEAST F	5326978 1	CSK2A_MAIZE F	33113 gam	1LP4	2HLD	1ZOH			Sc=5.67079, min distance = 2.07627
ATPB_YEAST F	91532 AMP	KIF1A_MOUSE F	33113 gam	1VFV	2HLD	1I6I		0.99	Sc=5.60132, min distance = 1.97439
ATPE_GEOKA F	17754396	PDPK1_HUMAN F	5957 Aden	2BIY	2E5Y	2R7B			Sc=6.34386, min distance = 2.13096
ATPE_GEOKA F	440317 AT	PURT_ECOLI Fu	5957 Aden	1KJ8	2E5Y	1KJJ		0.99	Sc=6.37677, min distance = 1.75411
ATPE_GEOKA F	444564 AD	MYS2_DICDI Fu	5957 Aden	1FMW	2E5Y	1W9I		0.91	Sc=6.32569, min distance = 0
ATPE_GEOKA F	447840 CI	PHKG1_RABIT F	5957 Aden	1QL6	2E5Y	1PHK			Sc=5.95112, min distance = 2.07719
ATPE_GEOKA F	6022 Aden	DDL_THET8 Ful	5957 Aden	2ZDQ	2E5Y	2ZDH		0.99	Sc=6.33916, min distance = 2.09994
ATPE_GEOKA F	6022 Aden	FAK1_HUMAN Fu	5957 Aden	2IJM	2E5Y	1MP8		0.99	Sc=5.89975, min distance = 2.24576
ATPE_GEOKA F	6022 Aden	MUTS_ECOLI Fu	5957 Aden	1W7A	2E5Y	1OH7		0.99	Sc=6.37273, min distance = 1.74379
ATPE_GEOKA F	6022 Aden	PURK_ECOLI Fu	5957 Aden	3ETH	2E5Y	3ETJ		0.99	Sc=6.37893, min distance = 2.08686
ATPE_GEOKA F	6022 Aden	PURP_METJA Fu	5957 Aden	2R7L	2E5Y	2R7N		0.99	Sc=5.89487, min distance = 1.85408
ATPE_GEOKA F	6022 Aden	VPS4B_MOUSE F	5957 Aden	2ZAN	2E5Y	2ZAO		0.99	Sc=6.35332, min distance = 2.11248
ATPE_GEOKA F	6022 Aden	Y059_METJA Fu	5957 Aden	2J9C	2E5Y	2J9D		0.99	Sc=6.37893, min distance = 2.30723
ATPE_GEOKA F	6083 aden	AAKG1_RAT Ful	5957 Aden	2V92	2E5Y	2V8Q		0.98	Sc=6.29641, min distance = 2.02700
ATPE_GEOKA F	6133 uric	Q381M1_9TRYP	5957 Aden	2Q0D	2E5Y	2IKF			Sc=5.79206, min distance = 2.27008
ATPE_GEOKA F	6176 Cyti	Q381M1_9TRYP	5957 Aden	2Q0D	2E5Y	2Q0C			Sc=5.77664, min distance = 2.21960
ATPE_GEOKA F	6830 guan	Q381M1_9TRYP	5957 Aden	2Q0D	2E5Y	2Q0E		0.8	Sc=5.94057, min distance = 2.43511
ATPE_GEOKA F	91532 AMP	PURT_ECOLI Fu	5957 Aden	1KJ8	2E5Y	1KJI		0.99	Sc=6.10123, min distance = 2.01749
ATPE_GEOKA F	91532 AMP	Q5SJV7_THET8	5957 Aden	2Z08	2E5Y	2Z09		0.99	Sc=6.41788, min distance = 1.93713
ATPG_BOVIN F	10831270	TTHY_HUMAN Fu	445154 re	1DVS	2JIZ	3CN3			Sc=5.65312, min distance = 2.33861
ATPG_BOVIN F	1184681 C	TTHY_HUMAN Fu	445154 re	1DVS	2JIZ	3CN4			Sc=5.9395, min distance = 2.241295
ATPG_BOVIN F	119346 3,	TTHY_HUMAN Fu	445154 re	1DVS	2JIZ	2GAB			Sc=5.61177, min distance = 0
ATPG_BOVIN F	177880 2'	TTHY_HUMAN Fu	445154 re	1DVS	2JIZ	2G9K			Sc=5.6137, min distance = 0
ATPG_BOVIN F	20284644	TTHY_HUMAN Fu	445154 re	1DVS	2JIZ	2QGC			Sc=5.80277, min distance = 2.70949
ATPG_BOVIN F	23722943	TTHY_HUMAN Fu	445154 re	1DVS	2JIZ	2QGD			Sc=5.78311, min distance = 2.38212
ATPG_BOVIN F	23722944	TTHY_HUMAN Fu	445154 re	1DVS	2JIZ	2QGE			Sc=5.77341, min distance = 2.08366
ATPG_BOVIN F	25011739	TTHY_HUMAN Fu	445154 re	1DVS	2JIZ	3CN0		0.79	Sc=5.67526, min distance = 0
ATPG_BOVIN F	25011740	TTHY_HUMAN Fu	445154 re	1DVS	2JIZ	3CN1			Sc=5.63166, min distance = 0
ATPG_BOVIN F	97032 1g3	TTHY_HUMAN Fu	445154 re	1DVS	2JIZ	2G5U			Sc=5.79174, min distance = 0
AVID_CHICK F	11184136	SAV_STRAV Ful	446236 BI	1RXJ	1IJ8	1SWP	42.25		Sc=6.11111, min distance = 1.41369
AVID_CHICK F	11184136	SAV_STRAV Ful	446903 NC	1LCV	1LDO	1SWP	42.25	0.99	Sc=6.21024, min distance = 0.68626
AVID_CHICK F	11184136	SAV_STRAV Ful	446905 Bi	1LCZ	1LEL	1SWP	42.25	0.94	Sc=6.04471, min distance = 1.86327
AVID_CHICK F	5326799 1	SAV_STRAV Ful	171548 bi	2F01	2JGS	1SRI	42.25		Sc=6.28879, min distance = 1.15202
AVID_CHICK F	5326800 C	SAV_STRAV Ful	446904 HC	1LCW	1LDQ	1SRJ	42.25		Sc=6.16039, min distance = 2.13392

AVID_CHICK Fı	5326800 (	SAV_STRAV Ful	446905 Bi	1LCZ	1LEL	1SRJ	42.25		Sc=6.13209, min distance = 2.56111
AVR2_CHICK Fı	446903 NC	AVID_CHICK Fu	171548 bi	2JGS	1WBI	1LDO		0.99	Sc=6.13005, min distance = 1.586012
AVR4_CHICK Fı	11184136	SAV_STRAV Ful	446236 BI	1RXJ	2FHL	1SWP	32.5		Sc=6.16421, min distance = 1.94508
AVR4_CHICK Fı	24771754	SAV_STRAV Ful	446236 BI	1RXJ	2FHL	2QCB	32.5	0.78	Sc=6.05845, min distance = 2.87706
AVR4_CHICK Fı	446903 NC	SAV_STRAV Ful	171548 bi	2F01	1Y55	1LCV	32.5	0.99	Sc=6.09696, min distance = 2.51704
AVR4_CHICK Fı	5326799 1	SAV_STRAV Ful	171548 bi	2F01	1Y55	1SRI	32.5		Sc=6.36264, min distance = 1.46385
AVRB_PSESG Fı	444564 AI	BIOD_ECOLI Fu	6022 Aden	1DAD	2NUN	1BS1		0.91	Sc=6.36326, min distance = 2.49502
AVRB_PSESG Fı	445940 O6	CDK2_HUMAN Fu	6022 Aden	1GY3	2NUN	1GZ8			Sc=6.14213, min distance = 1.85988
AVRB_PSESG Fı	445966 O6	CDK2_HUMAN Fu	6022 Aden	1GY3	2NUN	1H0V			Sc=6.28916, min distance = 1.70361
AVRB_PSESG Fı	447916 ac	RIO1_ARCFU Fu	6022 Aden	1ZTH	2NUN	1ZTF		0.95	Sc=6.19342, min distance = 2.18305
AVRB_PSESG Fı	447955 1p	CDK2_HUMAN Fu	6022 Aden	1GY3	2NUN	1PXI			Sc=5.73641, min distance = 1.66149
AVRB_PSESG Fı	4564 1e1v	CDK2_HUMAN Fu	6022 Aden	1GY3	2NUN	1E1V			Sc=6.21643, min distance = 2.05188
AVRB_PSESG Fı	5957 Aden	BIOD_ECOLI Fu	6022 Aden	1DAD	2NUN	1A82		0.99	Sc=5.9565, min distance = 2.379417
AVRB_PSESG Fı	6031 Uric	O33839_THEMA I	6022 Aden	1XJK	2NUN	1XJG			Sc=6.27635, min distance = 2.14499
AVRB_PSESG Fı	6083 aden	PSPF_ECOLI Fu	6022 Aden	2C98	2NUN	2VII		0.99	Sc=6.24892, min distance = 2.62247
AVRB_PSESG Fı	60961 ade	IPKA_RABIT Fu	6022 Aden	1JBP	2NUN	1FMO		0.95	Sc=6.17038, min distance = 2.61439
AVRB_PSESG Fı	6804 guar	NDK_PYRHO Ful	6022 Aden	2DYA	2NUN	2DXF		0.8	Sc=6.27374, min distance = 2.61635
AVRB_PSESG Fı	6918710 5	CDK2_HUMAN Fu	6022 Aden	1GY3	2NUN	3EJ1			Sc=6.21059, min distance = 1.60576
AVRB_PSESG Fı	8977 1dar	O33839_THEMA I	6022 Aden	1XJK	2NUN	1XJE		0.8	Sc=6.39909, min distance = 2.19271
AVRB_PSESG Fı	8977 1dar	RIR1_YEAST Fu	6022 Aden	2CVX	2NUN	2CVW		0.8	Sc=5.96144, min distance = 2.06346
AVRB_PSESG Fı	91532 AMI	BIOD_ECOLI Fu	6022 Aden	1DAD	2NUN	1DAG		0.99	Sc=6.00612, min distance = 2.18097
AVRB_PSESG Fı	91532 AMI	DNAA_AQUAE Fu	6022 Aden	1L8Q	2NUN	2HCB		0.99	Sc=6.02682, min distance = 1.86661
AVRB_PSESG Fı	91532 AMI	PURP_METJA Fu	6022 Aden	2R7N	2NUN	2R7K		0.99	Sc=6.43999, min distance = 2.05150
AZOR1_PSEAE I	16741044	PYRDA_LACLC Fı	444243 FA	1JUB	2V9C	1JRB			Sc=6.17046, min distance = 2.28562
AZOR1_PSEAE I	446995 FM	CYB2_YEAST Fu	444243 FA	1KBI	2V9C	1LTD		0.77	Sc=6.1699, min distance = 1.903672
AZOR1_PSEAE I	5326566 1	Q9WZW1_THEMA I	444243 FA	1T6Y	2V9C	1S4M		0.82	Sc=6.19239, min distance = 1.52659
AZOR_ECOLI Fı	446995 FM	CYB2_YEAST Fu	444243 FA	1KBI	2Z98	1LTD		0.77	Sc=6.17046, min distance = 2.40094
AZOR_ECOLI Fı	448076 CI	CYB2_YEAST Fu	444243 FA	1KBI	2Z98	1SZG		0.76	Sc=6.17046, min distance = 2.22969
AZOR_ENTFA Fı	446995 FM	CYB2_YEAST Fu	444243 FA	1KBI	2HPV	1LTD		0.77	Sc=6.54697, min distance = 2.36655
AZOR_ENTFA Fı	448076 CI	CYB2_YEAST Fu	444243 FA	1KBI	2HPV	1SZG		0.76	Sc=6.10821, min distance = 1.64473
AZOR_SALTY Fı	446995 FM	CYB2_YEAST Fu	444243 FA	1KBI	1T5B	1LTD		0.77	Sc=6.1699, min distance = 2.317846
AZOR_SALTY Fı	448076 CI	CYB2_YEAST Fu	444243 FA	1KBI	1T5B	1SZG		0.76	Sc=6.16935, min distance = 2.26433
AZOR_SALTY Fı	9547941 4	NOS1_RAT Full	444243 FA	1TLL	1T5B	2G6J			Sc=5.68725, min distance = 0.58983
AZR_BACSU Fu	16741044	PYRDA_LACLC Fı	444243 FA	1JUB	1NNI	1JRB			Sc=6.16754, min distance = 1.95320
AZR_BACSU Fu	446995 FM	CYB2_YEAST Fu	444243 FA	1KBI	1NNI	1LTD		0.77	Sc=6.16518, min distance = 1.99422
AZR_BACSU Fu	448076 CI	CYB2_YEAST Fu	444243 FA	1KBI	1NNI	1SZG		0.76	Sc=6.15992, min distance = 2.02265
AZR_BACSU Fu	5326566 1	BLVRB_HUMAN Fı	444243 FA	1HE4	1NNI	1HE5		0.82	Sc=6.1479, min distance = 2.147101

B2LA1_MOUSE I	439183 1i	TPIS_TRYBB Fu	311 citri	2VEK	2VOH	1IIH		Sc=5.68695, min distance = 1.17393
B2LA1_MOUSE I	445905 1g	AROQ_HELPY Fu	311 citri	2C4V	2VOH	2C57		Sc=5.81931, min distance = 2.17090
B2LA1_MOUSE I	447550 ph	TPIS_TRYBB Fu	311 citri	2VEK	2VOH	4TIM		Sc=5.67814, min distance = 1.42419
B2LA1_MOUSE I	51 2-Oxop	SERA_ECOLI Fu	311 citri	2P9E	2VOH	1YBA	0.76	Sc=5.6826, min distance = 1.131176
B3GA1_HUMAN I	17473 UDI	B3GA3_HUMAN Fu	6031 Urid	3CU0	1V84	1KWS	0.78	Sc=6.6449, min distance = 2.430463
B3GA1_HUMAN I	185698 al	Q9HYN5_PSEAE I	82313 1rd	1W8H	1V84	1OVS		Sc=5.6304, min distance = 2.575557
B3GA1_HUMAN I	439357 ga	CGL2_COPCI Fu	24139 ace	1ULD	1V84	1ULE		Sc=5.83694, min distance = 0.80532
B3GA1_HUMAN I	445675 UI	EXTL2_MOUSE Fu	6031 Urid	1ON8	1V84	1ON6	0.82	Sc=6.25404, min distance = 2.06855
B3GA1_HUMAN I	445948 D-	Q9HYN5_PSEAE I	82313 1rd	1W8H	1V84	1OXC		Sc=5.65103, min distance = 2.44453
B3GA1_HUMAN I	446996 GA	Q9HYN5_PSEAE I	439353 be	1W8F	1V84	1W8H	0.89	Sc=5.67966, min distance = 2.07610
B3GA1_HUMAN I	448295 4-	BGAT_HUMAN Fu	6031 Urid	1LZJ	1V84	1R7Y		Sc=6.14805, min distance = 1.81930
B3GA1_HUMAN I	448923 2a	MGAT1_RABIT Fu	6031 Urid	2AM5	1V84	2AM4	0.87	Sc=6.15558, min distance = 2.49571
B3GA1_HUMAN I	449462 2-	LEG7_HUMAN Fu	439353 be	4GAL	1V84	3GAL		Sc=5.78908, min distance = 2.21302
B3GA1_HUMAN I	6030 Uric	GLMU_HAEIN Fu	6031 Urid	2V0K	1V84	2V0J	0.99	Sc=6.29236, min distance = 2.38980
B3GA1_HUMAN I	6030 Uric	O87988_BORBR I	6031 Urid	2PZM	1V84	2PZL	0.99	Sc=6.29325, min distance = 2.45494
B3GA1_HUMAN I	6030 Uric	PYRH_ECOLI Fu	6031 Urid	2BND	1V84	2BNE	0.99	Sc=6.30415, min distance = 1.90859
B3GA1_HUMAN I	6132 Cyti	ECX1_PYRAB Fu	6031 Urid	2PNZ	1V84	2PO2	0.83	Sc=5.9071, min distance = 2.104276
B3GA1_HUMAN I	6420194 N	B4GT1_BOVIN Fu	24139 ace	1NMM	1V84	2FYD		Sc=5.99137, min distance = 2.32564
B3GA3_HUMAN I	16058615	LYSC_CHICK Fu	439353 be	1UC0	3CU0	2H9J		Sc=5.91201, min distance = 2.13362
B3GA3_HUMAN I	445675 UI	EXTL2_MOUSE Fu	6031 Urid	1ON8	3CU0	1ON6	0.82	Sc=5.96453, min distance = 1.86420
B3GA3_HUMAN I	448923 2a	MGAT1_RABIT Fu	6031 Urid	2AM5	3CU0	2AM4	0.87	Sc=6.29338, min distance = 2.23848
B3GA3_HUMAN I	4495 nime	TRFL_BOVIN Fu	439353 be	2DWJ	3CU0	3E9X		Sc=5.79431, min distance = 2.27967
B3GA3_HUMAN I	6030 Uric	PYRH_ECOLI Fu	6031 Urid	2BND	3CU0	2BNE	0.99	Sc=6.30651, min distance = 1.46056
B3GA3_HUMAN I	6132 Cyti	ECX1_PYRAB Fu	6031 Urid	2PNZ	3CU0	2PO2	0.83	Sc=6.35388, min distance = 2.20239
B3GA3_HUMAN I	656981 CU	LYSC_CHICK Fu	439353 be	1UC0	3CU0	1YIL		Sc=5.93105, min distance = 2.17521
B4GT1_BOVIN I	23722946	BGAT_HUMAN Fu	18068 Udp	2RJ7	1YRO	2RJ9	0.86	Sc=6.37157, min distance = 2.21856
B4GT1_BOVIN I	439353 be	Q6PYX1_HUMAN I	24139 ace	1CLY	1NMM	1S3K		Sc=5.74199, min distance = 2.17391
B4GT1_BOVIN I	444205 CI	DPP4_HUMAN Fu	24139 ace	2RGU	1NMM	3BJM	0.91	Sc=5.97025, min distance = 2.49083
B4GT1_BOVIN I	446578 1r	MBL2_RAT Full	24139 ace	1KZD	1NMM	1RDI		Sc=5.67135, min distance = 2.29291
B4GT1_HUMAN I	104904 Me	IGKC_MOUSE Fu	24139 ace	3BZ4	2AGD	2AJZ		Sc=5.78142, min distance = 1.80043
B4GT1_HUMAN I	440271 N-	HYSA_STRPN Fu	24139 ace	1LXK	2AGD	1OJN	0.94	Sc=6.03588, min distance = 2.42852
BACE1_HUMAN I	24901727	RHAMD_SALTY Fu	444305 L-	3D46	2QP8	3CXO		Sc=5.6657, min distance = 2.511641
BACH_HALSA Fu	151008 N-	CPXB_BACME Fu	985 palmi	1FAG	2JAF	1JPZ		Sc=6.20786, min distance = 2.24807
BACH_HALSA Fu	175468 12	LACB_BOVIN Fu	985 palmi	1GXA	2JAF	1BSO	0.83	Sc=5.7238, min distance = 2.741801
BACH_HALSA Fu	91486 Sph	POLG_POL1M Fu	985 palmi	1HXS	2JAF	2PLV		Sc=6.15216, min distance = 2.09319
BADH_GADCA Fu	446006 CI	O57693_THETE I	5893 nadi	1UXT	1BPW	1UXU		Sc=5.89112, min distance = 2.29025
BADH_GADCA Fu	6022 Ader	ALDH2_HUMAN Fu	5893 nadi	1O04	1BPW	1NZX		Sc=5.60152, min distance = 2.76523

BADH_GADCA F	6022 Ader	Q5SI02_THET8	5893 nadi	2BJK	1BPW	2BJA		Sc=6.15835, min distance = 2.73115
BCAT2_HUMAN	164795 N	RBCMT_PEA Ful	23831 HEF	1MLV	2HGX	2H2J		Sc=5.66309, min distance = 2.77506
BCKD_RAT Ful	11963551	HSP82_YEAST F	6022 Aden	1AMW	1GKZ	2IWS		Sc=6.18952, min distance = 2.26373
BCKD_RAT Ful	11963552	HSP82_YEAST F	6022 Aden	1AMW	1GKZ	2IWU		Sc=6.19822, min distance = 2.65419
BCKD_RAT Ful	16058668	HS90A_HUMAN F	6022 Aden	1BYQ	1GKZ	2UWD		Sc=6.33512, min distance = 2.08785
BCKD_RAT Ful	24832021	HS90A_HUMAN F	6022 Aden	1BYQ	1GKZ	2QG0		Sc=6.39776, min distance = 1.97593
BCKD_RAT Ful	444503 FU	NDKC_DICDI Fu	6022 Aden	1KDN	1GKZ	1B99		Sc=6.29542, min distance = 2.23096
BCKD_RAT Ful	445940 O6	CDK2_HUMAN Fu	6022 Aden	1GY3	1GKZ	1GZ8		Sc=6.16452, min distance = 2.05521
BCKD_RAT Ful	446090 1h	NDKC_DICDI Fu	6022 Aden	1KDN	1GKZ	1HIY	0.97	Sc=5.98439, min distance = 2.03004
BCKD_RAT Ful	447955 1p	CDK2_HUMAN Fu	6022 Aden	1GY3	1GKZ	1PXI		Sc=5.71389, min distance = 2.24817
BCKD_RAT Ful	5326934 1	HS90A_HUMAN F	6022 Aden	1BYQ	1GKZ	1YC1		Sc=6.41141, min distance = 2.29211
BCKD_RAT Ful	5326935 1	HS90A_HUMAN F	6022 Aden	1BYQ	1GKZ	1YC4		Sc=6.2815, min distance = 2.219269
BCKD_RAT Ful	5327095 V	HS90A_HUMAN F	6022 Aden	1BYQ	1GKZ	2BSM		Sc=6.40921, min distance = 2.21111
BCKD_RAT Ful	5327103 2	HS90A_HUMAN F	6022 Aden	1BYQ	1GKZ	2BYH		Sc=6.46064, min distance = 2.35466
BCKD_RAT Ful	5327148 C	IPKA_RABIT Fu	6022 Aden	1JBP	1GKZ	2ERZ		Sc=6.17552, min distance = 2.13298
BCKD_RAT Ful	5403427 C	PDK4_HUMAN Fu	6022 Aden	2ZKJ	1GKZ	2ZDX		Sc=6.24785, min distance = 2.09099
BCKD_RAT Ful	6082103 2	HS90A_HUMAN F	6022 Aden	1BYQ	1GKZ	2BT0		Sc=6.29358, min distance = 2.16168
BCKD_RAT Ful	6082103 2	HSP82_YEAST F	6022 Aden	1AMW	1GKZ	2BRC		Sc=6.32174, min distance = 2.44180
BCKD_RAT Ful	6083 aden	SRC_CHICK Ful	440317 AT	3DQW	1GJV	3DQX	0.97	Sc=6.3137, min distance = 2.521396
BCKD_RAT Ful	60961 ade	IPKA_RABIT Fu	6022 Aden	1JBP	1GKZ	1FMO	0.95	Sc=6.17404, min distance = 2.14819
BCKD_RAT Ful	6102788 2	HS90A_HUMAN F	6022 Aden	1BYQ	1GKZ	2CCT		Sc=6.36921, min distance = 2.02239
BCKD_RAT Ful	6132 Cyti	O33839_THEMA	6022 Aden	1XJK	1GKZ	1XJN		Sc=6.3296, min distance = 1.828856
BCKD_RAT Ful	6323491 M	PDK3_HUMAN Fu	6022 Aden	1Y8O	1GKZ	2Q8I		Sc=6.29556, min distance = 2.45355
BCKD_RAT Ful	6323491 M	TOP6B_SULSH F	6022 Aden	1Z5B	1GKZ	2HKJ		Sc=6.30745, min distance = 2.18137
BCKD_RAT Ful	6804 guar	NDK_PYRHO Ful	6022 Aden	2DYA	1GKZ	2DXF	0.8	Sc=5.60973, min distance = 2.48810
BCKD_RAT Ful	72194 2-C	ENPL_CANFA Fu	6022 Aden	1TC6	1GKZ	1QYE	0.89	Sc=6.0852, min distance = 2.610860
BCKD_RAT Ful	8977 1dar	NDK_PYRHO Ful	6022 Aden	2DYA	1GKZ	2DXE	0.8	Sc=5.696, min distance = 2.2380469
BCKD_RAT Ful	9543433 T	NDKC_DICDI Fu	6022 Aden	1KDN	1GKZ	1F6T		Sc=6.36095, min distance = 2.03633
BDH2_HUMAN F	15942665	INHA_MYCTU Fu	5893 nadi	2H7I	2AG5	2H9I	0.88	Sc=6.87316, min distance = 2.07299
BDH2_HUMAN F	439153 Di	GALE_ECOLI Fu	5893 nadi	1UDC	2AG5	1UDB	0.79	Sc=6.74575, min distance = 2.30862
BDH2_HUMAN F	447049 OA	NPD_THEMA Ful	5893 nadi	2H4H	2AG5	2H4J		Sc=6.16854, min distance = 2.14664
BDH2_HUMAN F	449263 IN	INHA_MYCTU Fu	5893 nadi	2H7I	2AG5	2NV6	0.93	Sc=6.85254, min distance = 2.43000
BDH2_HUMAN F	6022 Ader	Q5SI02_THET8	5893 nadi	2BJK	2AG5	2BJA		Sc=6.24562, min distance = 2.33249
BEV1L_BETVE	5881 Dehy	SDIS_PSEPU Fu	222528 DE	1E3V	1FM4	1E3R		Sc=5.87732, min distance = 1.65437
BFRA_THEMA F	446944 CI	MIF_HUMAN Ful	311 citri	1GD0	1W2T	1LJT		Sc=5.76549, min distance = 2.26615
BFRA_THEMA F	448573 FI	CASP7_HUMAN F	311 citri	2QL9	1W2T	1SHL		Sc=5.77977, min distance = 2.63764
BGAL_ECOLI F	15942683	FCN3_HUMAN Fu	439353 be	2J5Z	1JZ7	2J60	0.77	Sc=5.67725, min distance = 2.24942

BGAL_ECOLI F	439764 be	ARAF_ECOLI Fu	439353 be	8ABP	1JZ7	6ABP		0.93	Sc=5.64704, min distance = 2.33531
BGAL_ECOLI F	6027 xylo	Q9ZB17_9LACT	439353 be	1NSX	1JZ7	1MN0		0.93	Sc=5.68518, min distance = 1.96120
BGAL_SULSO F	11957434	BGLA_THEMA Fu	445248 nc	2J78	1UWU	2J7G	29.47		Sc=6.11064, min distance = 2.37222
BGAL_SULSO F	11957435	BGLA_THEMA Fu	445248 nc	2J78	1UWU	2J7H	29.47		Sc=5.76279, min distance = 2.62564
BGAL_SULSO F	24963032	BGLA_THEMA Fu	445248 nc	2J78	1UWU	2VRJ	29.47		Sc=6.15777, min distance = 2.32116
BGAL_SULSO F	445257 1i	MYRA_SINAL Fu	445248 nc	1E6S	1UWU	1E70	24.28		Sc=5.71422, min distance = 2.03054
BGAL_SULSO F	447413 NT	BGLA_THEMA Fu	445248 nc	2J78	1UWU	2J7B	29.47		Sc=6.02287, min distance = 2.37356
BGAL_SULSO F	447413 NT	MYRA_SINAL Fu	445248 nc	1E6S	1UWU	1E6Q	24.28		Sc=6.02177, min distance = 2.41585
BGAL_SULSO F	5326892 C	BGLA_THEMA Fu	445248 nc	2J78	1UWU	2J7C	29.47		Sc=6.3685, min distance = 2.226358
BGAL_SULSO F	7027 gluc	MYRA_SINAL Fu	445248 nc	1E6S	1UWU	1E6X	24.28		Sc=5.91575, min distance = 2.46786
BGAL_SULSO F	9549193 C	BGLA_THEMA Fu	445248 nc	2J78	1UWU	2CES	29.47		Sc=6.06112, min distance = 2.56588
BGAT_HUMAN F	445795 1g	GGTA1_BOVIN F	18068 Udp	2VS5	2RJ7	2VFZ	46.89	0.98	Sc=6.63634, min distance = 2.51084
BGAT_HUMAN F	6030 Urid	GGTA1_BOVIN F	18068 Udp	2VS5	2RJ7	1G8O	46.89	0.88	Sc=6.31956, min distance = 2.45613
BGLA_PAEPO F	29435 1-D	XYLA_ARTS7 Fu	10690 glu	1XLF	1BGG	1DIE			Sc=5.8976, min distance = 1.679838
BGLA_PAEPO F	445074 2,	XYLA_ARTS7 Fu	10690 glu	1XLF	1BGG	1DID			Sc=5.92452, min distance = 1.25087
BGLA_THEMA F	445999 CI	Q79G13_MYCTU	447607 1c	1UP2	1OIF	1UOZ			Sc=5.7012, min distance = 2.282491
BGLA_THEMA F	64689 bet	Q79G13_MYCTU	447607 1c	1UP2	1OIF	1UP0			Sc=5.89491, min distance = 2.08505
BGLA_THEMA F	64960 Pol	Q9XEI3_HORVD	5326892 C	1X39	2J7C	1EX1			Sc=5.78964, min distance = 2.04210
BGLA_THEMA F	81462 Bis	GLCM_HUMAN Fu	447607 1c	2NSX	1OIF	2V3F			Sc=6.07036, min distance = 2.36558
BGLB_PAEPO F	446983 ID	Q9XEI3_HORVD	446272 Th	1IEX	2O9R	1X38			Sc=6.20119, min distance = 2.45442
BHMT1_HUMAN	51 2-Oxop	SERA_ECOLI Fu	311 citri	2P9E	1LT8	1YBA		0.76	Sc=5.87854, min distance = 0.41826
BIOB_ECOLI F	439182 5'	Q70GK9_STRCT	34756 Acy	1RQP	1R30	2CC2		0.86	Sc=6.21604, min distance = 1.46714
BIOB_ECOLI F	444178 CI	BIOD_ECOLI Fu	445027 d-	1DAM	1R30	1A82		0.78	Sc=5.87484, min distance = 2.42833
BIOB_ECOLI F	445025 7,	BIOD_ECOLI Fu	445027 d-	1DAM	1R30	1DAH		0.78	Sc=5.88992, min distance = 2.35962
BIOB_ECOLI F	445971 CI	COMT_RAT Full	34756 Acy	2CL5	1R30	1H1D		0.92	Sc=6.17749, min distance = 2.13706
BIOB_ECOLI F	6852187 2	MOAA_STAA8 Fu	34756 Acy	2FB2	1R30	2FB3		0.84	Sc=6.08325, min distance = 2.22145
BIOB_ECOLI F	6852187 2	Q70GK9_STRCT	34756 Acy	1RQP	1R30	2CBX		0.84	Sc=6.15423, min distance = 1.59302
BIOD_ECOLI F	16122607	PDPK1_HUMAN F	5957 Aden	2BIY	1A82	2PE0			Sc=6.18109, min distance = 2.06693
BIOD_ECOLI F	24864081	CDK2_HUMAN Fu	6022 Aden	1GY3	1DAD	2VTS			Sc=6.24065, min distance = 2.10866
BIOD_ECOLI F	2608 1jsv	CDK2_HUMAN Fu	6022 Aden	1GY3	1DAD	1JSV			Sc=5.91939, min distance = 2.85012
BIOD_ECOLI F	33113 gam	KIF1A_MOUSE F	91532 AMP	1I6I	1DAG	1VFV		0.99	Sc=6.10614, min distance = 2.40240
BIOD_ECOLI F	3540 1yds	KAPCA_BOVIN F	5957 Aden	1Q24	1A82	1YDS			Sc=5.85122, min distance = 2.39234
BIOD_ECOLI F	3547 Fasu	KAPCA_BOVIN F	5957 Aden	1Q24	1A82	1Q8W			Sc=5.92379, min distance = 2.16362
BIOD_ECOLI F	4369136 C	CDK2_HUMAN Fu	6022 Aden	1GY3	1DAD	1DM2			Sc=6.28951, min distance = 1.50167
BIOD_ECOLI F	444852 CI	ATPA1_BOVIN F	5957 Aden	2V7Q	1A82	1COW		0.91	Sc=6.5043, min distance = 2.434584
BIOD_ECOLI F	447649 1c	CDK2_HUMAN Fu	6022 Aden	1GY3	1DAD	1OI9			Sc=6.38174, min distance = 2.06594
BIOD_ECOLI F	447655 1c	CDK2_HUMAN Fu	6022 Aden	1GY3	1DAD	1OIU			Sc=6.33126, min distance = 2.10004

BIOD_ECOLI F	448968 1	HS90A_HUMAN F	6022 Aden	1BYQ	1DAD	1UY9			Sc=6.22967, min distance = 2.11142
BIOD_ECOLI F	449240 1	KAPCA_BOVIN F	5957 Aden	1Q24	1A82	1YDR			Sc=5.90464, min distance = 2.10208
BIOD_ECOLI F	4566 1h1s	CDK2_HUMAN Fu	6022 Aden	1GY3	1DAD	1H1S			Sc=6.32555, min distance = 2.04047
BIOD_ECOLI F	5496922	HS90A_HUMAN F	6022 Aden	1BYQ	1DAD	1UY7			Sc=6.21996, min distance = 2.09318
BIOD_ECOLI F	5496923	HS90A_HUMAN F	6022 Aden	1BYQ	1DAD	1UY8			Sc=6.22514, min distance = 2.02688
BIOD_ECOLI F	6083 ade	AAKG1_RAT Ful	5957 Aden	2V92	1A82	2V8Q		0.98	Sc=5.96483, min distance = 2.16192
BIOD_ECOLI F	6083 ade	Y059_METJA Fu	5957 Aden	2J9C	1A82	2J9D		0.98	Sc=6.30377, min distance = 1.95855
BIOD_ECOLI F	60961 ade	IPKA_RABIT Fu	6022 Aden	1JBP	1DAD	1FMO		0.95	Sc=6.13801, min distance = 2.84229
BIOI_BACSU F	11957330	CCPR_YEAST Fu	444098 HE	2EUT	3EJB	1BEQ		0.81	Sc=6.79973, min distance = 2.27322
BIOI_BACSU F	11957353	HBA_HORSE Ful	444098 HE	2D5X	3EJB	1IWH		0.83	Sc=6.7959, min distance = 2.152112
BIOI_BACSU F	11957360	CY1_BOVIN Ful	444098 HE	1L0N	3EJB	1NTM			Sc=6.80337, min distance = 2.05572
BIOI_BACSU F	11957361	CY1_BOVIN Ful	444098 HE	1L0N	3EJB	1NTZ		0.8	Sc=6.78024, min distance = 2.46506
BIOI_BACSU F	11957363	HBB_HUMAN Ful	444098 HE	1J40	3EJB	1RQA		0.77	Sc=6.79695, min distance = 2.03714
BIOI_BACSU F	11957385	MYG_PHYCA Ful	444098 HE	1A6M	3EJB	2CMM			Sc=6.2812, min distance = 2.422469
BIOI_BACSU F	11970219	CCPR_YEAST Fu	444098 HE	2EUT	3EJB	1BEM		0.83	Sc=6.78842, min distance = 1.75523
BIOI_BACSU F	11970220	CCPR_YEAST Fu	444098 HE	2EUT	3EJB	1BEP		0.8	Sc=6.80729, min distance = 2.22191
BIOI_BACSU F	11970221	CCPR_YEAST Fu	444098 HE	2EUT	3EJB	1BES		0.83	Sc=6.74682, min distance = 1.93079
BIOI_BACSU F	11970242	CCPR_YEAST Fu	444098 HE	2EUT	3EJB	1CPE		0.83	Sc=6.76248, min distance = 2.41256
BIOI_BACSU F	126994 C	CCPR_YEAST Fu	444098 HE	2EUT	3EJB	1Z53		0.77	Sc=6.79255, min distance = 2.28988
BIOI_BACSU F	4369260	KATG_BURPS Fu	444098 HE	2DV1	3EJB	1MWV		0.93	Sc=6.80071, min distance = 2.12007
BIOI_BACSU F	5326592	HMOX1_RAT Ful	444098 HE	1DVG	3EJB	1J2C		0.79	Sc=6.73083, min distance = 2.08229
BIOI_BACSU F	6420167	CPXA_PSEPU Fu	444098 HE	1YRC	3EJB	2FE6	28.62	0.86	Sc=6.80007, min distance = 2.13938
BIRA_ECOLI F	446903 N	AVID_CHICK Fu	171548 bi	2JGS	1HXD	1LDO		0.99	Sc=6.18491, min distance = 1.74002
BLAB_BACFR F	23831 HE	DEF_LEPIN Ful	78165 MES	1VEV	1A7T	1VEY		0.81	Sc=5.74278, min distance = 0.63441
BLAC_BACLI F	11869260	DGAL_ECOLI Fu	311 citri	2FW0	1I2S	2QW1			Sc=5.78318, min distance = 1.98319
BLAC_BACLI F	439183 1i	TPIS_TRYBB Fu	311 citri	2VEK	1I2S	1IIH			Sc=5.6018, min distance = 1.772203
BLAC_BACLI F	439459 H	NIFD_KLEPN Fu	311 citri	1H1L	1I2S	1QGU		0.92	Sc=5.98895, min distance = 2.07457
BLAC_BACLI F	439459 H	NIFK_KLEPN Fu	311 citri	1H1L	1I2S	1QGU		0.92	Sc=5.98895, min distance = 1.98982
BLAC_BACLI F	449604 N	ACON_BOVIN Fu	5318532 i	1C97	1W7F	8ACN			Sc=5.86733, min distance = 2.04583
BLAC_BACLI F	51 2-Oxo	IDHP_YEAST Fu	5318532 i	2QFW	1W7F	2QFY			Sc=5.77266, min distance = 1.53799
BLAC_BACLI F	51 2-Oxo	SERA_ECOLI Fu	311 citri	2P9E	1I2S	1YBA		0.76	Sc=5.6826, min distance = 1.042066
BLAR_STAAU F	64689 bet	O33897_RHOMR	23831 HE	1H0B	1XKZ	2BWA			Sc=5.60996, min distance = 2.11685
BLS_STRCL Fu	24851689	PIM1_HUMAN Fu	6083 aden	1YXU	1MC1	3CY2			Sc=6.29611, min distance = 1.66706
BLS_STRCL Fu	2519 caff	PYGM_RABIT Fu	6083 aden	8GPB	1MC1	1GFZ			Sc=5.84428, min distance = 2.02136
BLS_STRCL Fu	33113 ga	ASSY_THET8 Fu	6083 aden	1J20	1MC1	1KOR		0.97	Sc=6.55453, min distance = 1.40818
BLS_STRCL Fu	3973 nche	PIM1_HUMAN Fu	6083 aden	1YXU	1MC1	1YI3			Sc=5.98093, min distance = 1.21210
BLS_STRCL Fu	445321 C	NADE_BACSU Fu	6083 aden	2NSY	1MC1	1EE1		0.85	Sc=6.50024, min distance = 2.25579

BLS_STRCL Fu	447832 AD	ACSA_SALTY Fu	6083 aden	2P2F	1MC1	1PG4		0.99	Sc=6.45718, min distance = 1.148018
BLS_STRCL Fu	5957 Aden	PANC_MYCTU Fu	91557 1tm	1N2E	1JGT	2A84		0.99	Sc=6.02349, min distance = 2.023808
BLS_STRCL Fu	5957 Aden	PSPF_ECOLI Fu	6083 aden	2VII	1MC1	2C96		0.98	Sc=6.48623, min distance = 1.653390
BLS_STRCL Fu	6102784 A	PSPF_ECOLI Fu	6083 aden	2VII	1MC1	2C99		0.97	Sc=6.50779, min distance = 1.731780
BLS_STRCL Fu	611002 Op	PIM1_HUMAN Fu	6083 aden	1YXU	1MC1	1YXX			Sc=6.1937, min distance = 2.0779752
BLS_STRCL Fu	65110 AIC	AAKG1_HUMAN Fu	6083 aden	2UV4	1MC1	2UV5		0.79	Sc=5.83663, min distance = 2.021527
BLS_STRCL Fu	656964 1y	PDE4D_HUMAN Fu	6083 aden	1TB7	1MC1	1Y2C			Sc=5.973, min distance = 1.9368391
BLS_STRCL Fu	657135 Op	PIM1_HUMAN Fu	6083 aden	1YXU	1MC1	1YXV			Sc=5.88541, min distance = 2.048661
BLS_STRCL Fu	6804 guar	PDE10_HUMAN Fu	6083 aden	2OUN	1MC1	2OUQ		0.8	Sc=6.40204, min distance = 0
BLS_STRCL Fu	8582 Inos	PURA1_MOUSE Fu	6083 aden	1MF0	1MC1	1IWE		0.79	Sc=6.4339, min distance = 0.6733045
BPHB_BURXL Fu	11987635	ADH_DROLE Ful	5893 nadi	1SBY	1BDB	1B2L		0.76	Sc=6.86195, min distance = 1.956546
BPHB_BURXL Fu	169266 1,	GALE_HUMAN Fu	5893 nadi	1HZJ	1BDB	1I3K		0.79	Sc=6.7787, min distance = 2.1725731
BPHB_BURXL Fu	439153 Di	Q9BJJ9_PLAFA I	5893 nadi	1UH5	1BDB	1V35		0.79	Sc=6.77582, min distance = 2.146429
BPHC_BURXL Fu	6102772 C	ACES_MOUSE Fu	17472 Hex	2HA2	1LKD	2GYU		0.81	Sc=5.72623, min distance = 2.387015
BPHC_PSES1 Fu	11318 1,2	NAHC_PSEU8 Fu	254 3-phe	2EI3	1KW6	2EI1		0.92	Sc=5.83301, min distance = 2.117765
BPL_METJA Fu	446903 NC	AVID_CHICK Fu	171548 bi	2JGS	2EJ9	1LDO		0.99	Sc=6.17263, min distance = 1.992516
BPL_METJA Fu	5326799 1	SAV_STRAV Ful	171548 bi	2F01	2EJ9	1SRI			Sc=6.39854, min distance = 2.201386
BPNT1_RAT Fu	101812 2'	RET_HUMAN Ful	6083 aden	2IVT	1JP4	2IVS		0.97	Sc=6.24461, min distance = 2.088066
BPNT1_RAT Fu	3758 IBMX	PDE4D_HUMAN Fu	6083 aden	1TB7	1JP4	1ZKN			Sc=5.93998, min distance = 2.418770
BPNT1_RAT Fu	445708 AM	NADE_ECOLI Fu	6083 aden	1WXI	1JP4	1WXE		0.91	Sc=6.30589, min distance = 2.043524
BPNT1_RAT Fu	449190 1x	PDE4B_HUMAN Fu	6083 aden	1ROR	1JP4	1XM6			Sc=5.86061, min distance = 2.198480
BPNT1_RAT Fu	6022 Ader	PSPF_ECOLI Fu	6083 aden	2VII	1JP4	2C98		0.99	Sc=6.40508, min distance = 2.004788
BPNT1_RAT Fu	6030 Uric	ECX1_SULSO Fu	6083 aden	2C38	1JP4	2C37			Sc=6.29801, min distance = 0.549193
BPNT1_RAT Fu	6076 Cycl	PDE10_HUMAN Fu	6083 aden	2OUN	1JP4	2OUR		0.99	Sc=6.23146, min distance = 2.006166
BPNT1_RAT Fu	62551 Per	WRBA_ECOLI Fu	6083 aden	3B6J	1JP4	3B6K			Sc=5.66912, min distance = 1.691220
BPNT1_RAT Fu	65110 AIC	YL28_SCHPO Fu	6083 aden	2OOX	1JP4	2QRE		0.79	Sc=6.2023, min distance = 2.5763687
BPNT1_RAT Fu	656966 1y	PDE4D_HUMAN Fu	6083 aden	1TB7	1JP4	1Y2E			Sc=5.99448, min distance = 2.502682
BPNT1_RAT Fu	656968 1y	PDE4B_HUMAN Fu	6083 aden	1ROR	1JP4	1Y2H			Sc=5.85688, min distance = 1.976251
BPNT1_RAT Fu	656969 1y	PDE4D_HUMAN Fu	6083 aden	1TB7	1JP4	1Y2K			Sc=6.13107, min distance = 2.391935
BPNT1_RAT Fu	657135 Op	PIM1_HUMAN Fu	6083 aden	1YXU	1JP4	1YXV			Sc=5.85955, min distance = 2.403555
BPNT1_RAT Fu	6804 guar	NDK_PYRHO Ful	6083 aden	2DXD	1JP4	2DXF		0.8	Sc=6.29182, min distance = 2.339431
BPNT1_RAT Fu	8582 Inos	PURA1_MOUSE Fu	6083 aden	1MF0	1JP4	1IWE		0.79	Sc=6.35869, min distance = 2.152549
BPNT1_RAT Fu	8977 1dar	NDK_PYRHO Ful	6083 aden	2DXD	1JP4	2DXE		0.79	Sc=6.36106, min distance = 2.059986
BPNT1_RAT Fu	9578243 E	PDE4B_HUMAN Fu	6083 aden	1ROR	1JP4	1XLZ			Sc=6.05903, min distance = 1.891665
BPPS_SALOF Fu	51634 Mic	GLCM_HUMAN Fu	81462 Bis	2V3F	1N1Z	2V3D			Sc=6.05182, min distance = 1.246891
BRD2_HUMAN Fu	152217 2,	DYR_CANAL Ful	78165 MES	1M79	1X0J	1M78			Sc=5.886, min distance = 2.3112295
BTUB_ECOLI Fu	155448 B-	GLPG_ECOLI Fu	444279 1s	2IRV	1UJW	3B44			Sc=6.21413, min distance = 1.561213

BTUB_ECOLI F	350021 Op	AOFB_HUMAN Fu	444279 1s	1OJD	1UJW	1OJC		Sc=5.78241, min distance = 2.27520
BTUB_ECOLI F	445070 fa	AOFB_HUMAN Fu	444279 1s	1OJD	1UJW	2BK3		Sc=5.74563, min distance = 2.85717
BTUD_ECOLI F	79025 alp	MALE_ECOLI Fu	62551 Pen	3CSB	2QI9	1ANF		Sc=5.91455, min distance = 0.71238
BUDC_KLEPN F	169266 1,	GALE_HUMAN Fu	5893 nadi	1HZJ	1GEG	1I3K	0.79	Sc=6.79849, min distance = 2.24916
BUDC_KLEPN F	193758 Va	AMY1_HUMAN Fu	79025 alp	3DHP	1GEG	1MFU		Sc=5.68874, min distance = 1.16402
BUDC_KLEPN F	4369162 1	PYGM_RABIT Fu	79025 alp	1H5U	1GEG	1HLF		Sc=5.86082, min distance = 2.03076
BUDC_KLEPN F	447607 1c	PYGM_RABIT Fu	79025 alp	1H5U	1GEG	2G9V		Sc=5.61793, min distance = 2.73688
BUK2_THEMA F	33113 gam	IGF1R_HUMAN F	91532 AMP	1K3A	1SAZ	1JQH	0.99	Sc=6.00075, min distance = 1.79295
BUK2_THEMA F	338 salic	ALBU_HUMAN Fu	311 citri	1TF0	1X9J	2I2Z		Sc=5.85579, min distance = 0.90992
BUK2_THEMA F	444212 tr	ACON_BOVIN Fu	311 citri	1C96	1X9J	1ACO		Sc=5.70904, min distance = 2.28335
BUK2_THEMA F	447530	SRC_HUMAN Ful	311 citri	1O4L	1X9J	1O4F		Sc=5.93466, min distance = 2.46986
BUK2_THEMA F	447536 1c	SRC_HUMAN Ful	311 citri	1O4L	1X9J	1O4Q		Sc=5.65339, min distance = 2.21265
BUK2_THEMA F	6022 Aden	GLPK_ECOLI Fu	91532 AMP	1GLL	1SAZ	1GLB	0.99	Sc=6.35453, min distance = 1.99412
BUK2_THEMA F	6022 Aden	RBSK_ECOLI Fu	91532 AMP	1GQT	1SAZ	1RKD	0.99	Sc=5.65267, min distance = 2.05795
BUK2_THEMA F	6083 aden	PURP_METJA Fu	91532 AMP	2R7K	1SAZ	2R7M	0.97	Sc=5.80605, min distance = 2.70744
BUK2_THEMA F	8631 3,5-	ALBU_HUMAN Fu	311 citri	1TF0	1X9J	2BXL		Sc=5.71431, min distance = 0.99921
BZNB_PSEFL F	124687 1r	OXC_OXAFO Ful	1132 thia	2JI8	2UZ1	2C31	0.79	Sc=6.12812, min distance = 1.05174
BZNB_PSEFL F	448673 PE	ILVB_YEAST Fu	1132 thia	1N0H	2UZ1	1T9B		Sc=5.86359, min distance = 2.52268
BZNB_PSEFL F	448721 N3	TKT1_YEAST Fu	1132 thia	1TRK	2UZ1	1TKA		Sc=6.44081, min distance = 2.21298
BZNB_PSEFL F	448723 1t	TKT1_YEAST Fu	1132 thia	1TRK	2UZ1	1TKC	0.77	Sc=6.1549, min distance = 2.358772
C167_SORCE F	11957330	CCPR_YEAST Fu	444098 HE	2EUT	1Q5D	1BEQ	0.81	Sc=6.74023, min distance = 2.05731
C167_SORCE F	11957360	CY1_BOVIN Ful	444098 HE	1L0N	1Q5D	1NTM		Sc=6.79297, min distance = 2.08333
C167_SORCE F	11957385	MYG_PHYCA Ful	444098 HE	1A6M	1Q5D	2CMM		Sc=6.28364, min distance = 2.29090
C167_SORCE F	11970219	CCPR_YEAST Fu	444098 HE	2EUT	1Q5D	1BEM	0.83	Sc=6.73855, min distance = 2.01113
C167_SORCE F	11970220	CCPR_YEAST Fu	444098 HE	2EUT	1Q5D	1BEP	0.8	Sc=6.75895, min distance = 2.04276
C167_SORCE F	11970222	CCPR_YEAST Fu	444098 HE	2EUT	1Q5D	1BJ9	0.77	Sc=6.80635, min distance = 2.08899
C167_SORCE F	11970242	CCPR_YEAST Fu	444098 HE	2EUT	1Q5D	1CPE	0.83	Sc=6.74063, min distance = 2.14792
C167_SORCE F	126994 CI	CCPR_YEAST Fu	444098 HE	2EUT	1Q5D	1Z53	0.77	Sc=6.7758, min distance = 2.144094
C167_SORCE F	446406 CZ	MYG_PHYCA Ful	444098 HE	1A6M	1Q5D	1J3F		Sc=6.3797, min distance = 2.058438
C167_SORCE F	447168 ZE	CCPR_YEAST Fu	444098 HE	2EUT	1Q5D	1ML2	0.82	Sc=6.80635, min distance = 2.13681
C1R_HUMAN Fu	101798 Me	MBL1_RAT Full	439554 fu	3KMB	1GPZ	1KWU	0.96	Sc=5.7749, min distance = 2.428528
C1R_HUMAN Fu	446578 1r	MBL1_RAT Full	439554 fu	3KMB	1GPZ	1KWW	0.96	Sc=5.62446, min distance = 2.51918
C1R_HUMAN Fu	94214 Met	MBL1_RAT Full	439554 fu	3KMB	1GPZ	1AFA	0.96	Sc=5.73529, min distance = 2.42411
C1TC_HUMAN F	15942680	6PGD_LACLM Fu	5886 NADP	2IZ0	1A4I	2IZ1		Sc=6.18131, min distance = 2.63143
C1TC_HUMAN F	440141 9i	DHB1_HUMAN Fu	5886 NADP	1QYV	1A4I	1QYW		Sc=5.89975, min distance = 2.15748
C562_ECOLX F	11957385	MYG_PHYCA Ful	444124 HE	1U7R	1LM3	2CMM		Sc=6.07405, min distance = 2.28452
C5AP_STRPY F	12136 Ber	POL_HV1N5 Ful	311 citri	2GON	1XF1	9HVP		Sc=5.66676, min distance = 1.11075

C5AP_STRPY F	16740961	ELA1_PIG Full	311 citri	2G4U	1XF1	1BMA			Sc=5.61111, min distance = 1.76471
C5AP_STRPY F	439459 H	NIFK_KLEPN Fu	311 citri	1H1L	1XF1	1QGU		0.92	Sc=5.98706, min distance = 1.41759
C5AP_STRPY F	51 2-Oxop	SERA_ECOLI Fu	311 citri	2P9E	1XF1	1YBA		0.76	Sc=5.86456, min distance = 0.72049
C5AP_STRPY F	6102777 2	AROQ_HELPY Fu	311 citri	2C4V	1XF1	2C4W			Sc=6.2486, min distance = 1.940779
C5AP_STRPY F	69689 9-M	GSK3B_HUMAN F	311 citri	1R0E	1XF1	1O9U			Sc=5.7552, min distance = 1.648868
C5AP_STRPY F	8778 Laur	CTXA3_NAJAT F	311 citri	1XT3	1XF1	1H0J			Sc=5.73865, min distance = 1.43245
C5B3_AMYOR F	11957355	CY1_BOVIN Ful	444098 HE	1L0N	1LG9	1L0L		0.77	Sc=6.805, min distance = 2.2281126
C5B3_AMYOR F	11957355	CYB_BOVIN Ful	444522 HE	1BGY	1LFK	1L0L		0.81	Sc=6.78803, min distance = 1.28178
C5B3_AMYOR F	11957360	CYB_BOVIN Ful	444522 HE	1BGY	1LFK	1NTM		0.78	Sc=6.7783, min distance = 2.093308
C5B3_AMYOR F	11957363	HBB_HUMAN Ful	444522 HE	2DN1	1LFK	1RQA		0.81	Sc=6.78842, min distance = 2.16701
C5B3_AMYOR F	11957370	HMOX1_HUMAN F	444124 HE	1OZW	1LGF	1TWN			Sc=6.64799, min distance = 2.08683
C5B3_AMYOR F	11957371	HMOX1_HUMAN F	444124 HE	1OZW	1LGF	1TWR			Sc=6.67324, min distance = 1.86310
C5B3_AMYOR F	11957373	CATE_ECOLI Fu	444124 HE	1IPH	1LGF	1P81		0.79	Sc=6.8204, min distance = 1.773623
C5B3_AMYOR F	11957385	MYG_PHYCA Ful	444098 HE	1A6M	1LG9	2CMM			Sc=6.30099, min distance = 2.19537
C5B3_AMYOR F	11957385	MYG_PHYCA Ful	444124 HE	1U7R	1LGF	2CMM			Sc=6.29044, min distance = 2.27747
C5B3_AMYOR F	11957385	MYG_PHYCA Ful	444522 HE	1VXD	1LFK	2CMM			Sc=6.30208, min distance = 2.07069
C5B3_AMYOR F	4369228 C	PER_COPCI Ful	444124 HE	1LY9	1LGF	1LYC		0.95	Sc=6.80071, min distance = 1.78694
C5B3_AMYOR F	444095 HE	RCEM_RHOVI Fu	444124 HE	3D38	1LGF	1DXR		0.91	Sc=6.79973, min distance = 2.14809
C5B3_AMYOR F	444125 HE	RCEL_RHOVI Fu	444124 HE	3D38	1LGF	2I5N		0.91	Sc=6.82381, min distance = 1.48993
C5B3_AMYOR F	444207 He	CP119_SULTO F	444124 HE	3B4X	1LGF	1UE8		0.99	Sc=6.79552, min distance = 2.38773
C5B3_AMYOR F	444207 He	PETD_MASLA Fu	444522 HE2E76		1LFK	1VF5	1		Sc=6.81263, min distance = 1.53960
C5B3_AMYOR F	446332 CI	MYG_PHYCA Ful	444124 HE	1U7R	1LGF	1IOP		0.8	Sc=6.36556, min distance = 2.20942
C5B3_AMYOR F	446406 CZ	MYG_PHYCA Ful	444098 HE	1A6M	1LG9	1J3F			Sc=6.38905, min distance = 1.99568
C5B3_AMYOR F	446406 CZ	MYG_PHYCA Ful	444124 HE	1U7R	1LGF	1J3F			Sc=6.38334, min distance = 2.05575
C5C4_AMYOR F	11957385	MYG_PHYCA Ful	444522 HE	1VXD	1UED	2CMM			Sc=6.30208, min distance = 2.05807
C5C4_AMYOR F	162636 1f	UROK_HUMAN Fu	8200 TETR	2O8U	1UED	1FV9			Sc=5.76839, min distance = 2.03182
C5C4_AMYOR F	444124 HE	NOS3_BOVIN Fu	444522 HE	2HX2	1UED	1ZZS		0.99	Sc=6.77962, min distance = 2.04707
C5C4_AMYOR F	444207 He	PETD_MASLA Fu	444522 HE2E76		1UED	1VF5		1	Sc=6.8053, min distance = 1.736982
C74A2_PARAR	11957330	CCPR_YEAST Fu	444098 HE	2EUT	3DAN	1BEQ		0.81	Sc=6.77628, min distance = 2.00572
C74A2_PARAR	11957363	HBB_HUMAN Ful	444098 HE	1J40	3DAN	1RQA		0.77	Sc=6.78962, min distance = 0.94001
C74A2_PARAR	11957385	MYG_PHYCA Ful	444098 HE	1A6M	3DAN	2CMM			Sc=6.30208, min distance = 2.36870
C74A2_PARAR	11963498	CP74A_ARATH F	24875321	3DSI	3DBM	3DSK		0.9	Sc=5.93225, min distance = 2.29098
C74A2_PARAR	11970219	CCPR_YEAST Fu	444098 HE	2EUT	3DAN	1BEM		0.83	Sc=6.7754, min distance = 2.102927
C74A2_PARAR	11970221	CCPR_YEAST Fu	444098 HE	2EUT	3DAN	1BES		0.83	Sc=6.73898, min distance = 2.07410
C74A2_PARAR	11970242	CCPR_YEAST Fu	444098 HE	2EUT	3DAN	1CPE		0.83	Sc=6.78119, min distance = 2.20510
C74A2_PARAR	126994 CI	CCPR_YEAST Fu	444098 HE	2EUT	3DAN	1Z53		0.77	Sc=6.7783, min distance = 2.099898
C74A2_PARAR	4369260 C	KATG_BURPS Fu	444098 HE	2DV1	3DAN	1MWV		0.93	Sc=6.77684, min distance = 2.26537

C74A2_PARAR I	446406 CZ	MYG_PHYCA Ful	444098 HE	1A6M	3DAN	1J3F			Sc=6.37887, min distance = 2.26101
C74A2_PARAR I	447168 ZE	CCPR_YEAST Fu	444098 HE	2EUT	3DAN	1ML2		0.82	Sc=6.80477, min distance = 2.06694
C74A2_PARAR I	6420167 M	CPXA_PSEPU Fu	444098 HE	1YRC	3DAN	2FE6		0.86	Sc=6.7959, min distance = 2.158466
C74A2_PARAR I	6443013 1	CP74A_ARATH Fu	24875321	3DSI	3DBM	3DSJ			Sc=6.05556, min distance = 2.12094
CADH2_ARATH I	11970225	ETR1_CANTR Fu	5886 NADP	1GUF	2CF6	1N9G		0.78	Sc=6.74278, min distance = 1.73132
CAH12_HUMAN I	10661 8-C	CHIT_YEAST Fu	1986 acet	2UY4	1JD0	2UY3			Sc=5.70162, min distance = 2.49927
CAH12_HUMAN I	14611919	CAH2_HUMAN Fu	1986 acet	1YDB	1JD0	3B4F			Sc=6.12765, min distance = 2.60189
CAH12_HUMAN I	3161908 S	CAH1_HUMAN Fu	1986 acet	1AZM	1JD0	2NN1			Sc=5.71031, min distance = 1.96357
CAH13_HUMAN I	14611919	CAH2_HUMAN Fu	1986 acet	1YDB	3CZV	3B4F			Sc=6.13068, min distance = 1.74269
CAH13_HUMAN I	16122582	CAH1_HUMAN Fu	1986 acet	1AZM	3CZV	2NN7			Sc=5.71529, min distance = 2.17526
CAH13_HUMAN I	3161908 S	CAH1_HUMAN Fu	1986 acet	1AZM	3CZV	2NN1			Sc=5.70674, min distance = 2.43429
CAH13_HUMAN I	3440 furo	CAH2_HUMAN Fu	1986 acet	1YDB	3CZV	1Z9Y			Sc=6.01803, min distance = 2.45015
CAH13_HUMAN I	4288 2,6-	CAH2_HUMAN Fu	1986 acet	1YDB	3CZV	1G48			Sc=5.73231, min distance = 2.62547
CAH13_HUMAN I	444612 1b	CAH2_HUMAN Fu	1986 acet	1YDB	3CZV	1BNT			Sc=5.74569, min distance = 2.27654
CAH13_HUMAN I	444613 CI	CAH2_HUMAN Fu	1986 acet	1YDB	3CZV	1BNU			Sc=5.68142, min distance = 2.20857
CAH13_HUMAN I	444828 CI	CAH2_HUMAN Fu	1986 acet	1YDB	3CZV	1CIL			Sc=5.66617, min distance = 2.55524
CAH13_HUMAN I	446237 4-	CAH2_HUMAN Fu	1986 acet	1YDB	3CZV	1I9L			Sc=5.94848, min distance = 1.61359
CAH13_HUMAN I	446240 1i	CAH2_HUMAN Fu	1986 acet	1YDB	3CZV	1I9O			Sc=5.75804, min distance = 2.52061
CAH13_HUMAN I	446241 1i	CAH2_HUMAN Fu	1986 acet	1YDB	3CZV	1I9P			Sc=5.83772, min distance = 2.49568
CAH13_HUMAN I	462919 re	CAH2_HUMAN Fu	1986 acet	1YDB	3CZV	2HD6			Sc=5.68878, min distance = 2.81415
CAH13_HUMAN I	5289159 C	CAH2_HUMAN Fu	1986 acet	1YDB	3CZV	1ZE8			Sc=5.66679, min distance = 2.61352
CAH13_HUMAN I	6604229 N	CAH2_HUMAN Fu	1986 acet	1YDB	3CZV	1ZGF			Sc=5.60036, min distance = 2.09906
CAH14_MOUSE I	10661 8-C	CHIT_YEAST Fu	1986 acet	2UY4	1RJ6	2UY3			Sc=5.63148, min distance = 2.65167
CAH14_MOUSE I	11957408	CAH2_HUMAN Fu	1986 acet	1YDB	1RJ6	2HOC			Sc=5.86761, min distance = 1.39929
CAH14_MOUSE I	1700 2,3-	CAH2_HUMAN Fu	1986 acet	1YDB	1RJ6	1G52			Sc=5.90788, min distance = 2.25218
CAH14_MOUSE I	444613 CI	CAH2_HUMAN Fu	1986 acet	1YDB	1RJ6	1BNU			Sc=5.70795, min distance = 2.54101
CAH14_MOUSE I	444828 CI	CAH2_HUMAN Fu	1986 acet	1YDB	1RJ6	1CIL			Sc=5.62303, min distance = 2.72560
CAH14_MOUSE I	446237 4-	CAH2_HUMAN Fu	1986 acet	1YDB	1RJ6	1I9L			Sc=5.87826, min distance = 1.89022
CAH14_MOUSE I	446238 2,	CAH2_HUMAN Fu	1986 acet	1YDB	1RJ6	1I9M			Sc=5.95476, min distance = 1.49001
CAH14_MOUSE I	446241 1i	CAH2_HUMAN Fu	1986 acet	1YDB	1RJ6	1I9P			Sc=5.83772, min distance = 2.20880
CAH14_MOUSE I	6102834 C	CAH2_HUMAN Fu	1986 acet	1YDB	1RJ6	2FOQ			Sc=5.70486, min distance = 2.75434
CAH14_MOUSE I	745994 2n	CAH2_HUMAN Fu	1986 acet	1YDB	1RJ6	2NNS			Sc=5.63033, min distance = 2.58914
CAH1_HUMAN Fu	11957408	CAH2_HUMAN Fu	1986 acet	1YDB	1AZM	2HOC	61.72		Sc=5.85339, min distance = 2.06712
CAH1_HUMAN Fu	11957408	CAH2_HUMAN Fu	3161908 S	2NNO	2NN1	2HOC	61.72		Sc=5.8237, min distance = 1.995482
CAH1_HUMAN Fu	1700 2,3-	CAH2_HUMAN Fu	745994 2n	2NNS	2NMX	1G52	61.72	0.8	Sc=5.91553, min distance = 2.16782
CAH1_HUMAN Fu	17414308	CAH13_HUMAN Fu	1986 acet	3CZV	1AZM	3DA2			Sc=5.64443, min distance = 1.77416
CAH1_HUMAN Fu	2144 1cnx	CAH2_HUMAN Fu	3161908 S	2NNO	2NN1	1CNX	61.72		Sc=5.8724, min distance = 2.395993

CAH1_HUMAN F	2144 1cnx	CAH2_HUMAN Fu	745994 2n	2NNS	2NMX	1CNX	61.72	0.78	Sc=5.9087, min distance = 2.013213
CAH1_HUMAN F	3440 furo	CAH2_HUMAN Fu	1986 acet	1YDB	1AZM	1Z9Y	61.72		Sc=5.90743, min distance = 2.16059
CAH1_HUMAN F	3440 furo	CAH2_HUMAN Fu	3161908 S	2NNO	2NN1	1Z9Y	61.72		Sc=6.05237, min distance = 2.11333
CAH1_HUMAN F	4369031 S	CAH2_HUMAN Fu	16122582	2NNV	2NN7	1OKN	61.72		Sc=5.884, min distance = 1.9302492
CAH1_HUMAN F	444137 CI	CAH2_HUMAN Fu	1986 acet	1YDB	1AZM	1A42	61.72		Sc=5.76054, min distance = 2.34348
CAH1_HUMAN F	444611 CI	CAH2_HUMAN Fu	1986 acet	1YDB	1AZM	1BNQ	61.72		Sc=5.7258, min distance = 2.510829
CAH1_HUMAN F	444613 CI	CAH2_HUMAN Fu	3161908 S	2NNO	2NN1	1BNU	61.72		Sc=5.74897, min distance = 2.44683
CAH1_HUMAN F	444830 CI	CAH2_HUMAN Fu	3161908 S	2NNO	2NN1	1CIN	61.72		Sc=5.65921, min distance = 1.62371
CAH1_HUMAN F	446241 1i	CAH2_HUMAN Fu	3161908 S	2NNO	2NN1	1I9P	61.72		Sc=5.94983, min distance = 1.96979
CAH1_HUMAN F	448784	CAH2_HUMAN Fu	3161908 S	2NNO	2NN1	1TTM	61.72		Sc=5.78718, min distance = 2.06786
CAH1_HUMAN F	5143 sacc	CAH2_HUMAN Fu	16122582	2NNV	2NN7	2Q1B	61.72		Sc=5.62341, min distance = 1.02941
CAH1_HUMAN F	6102834 C	CAH2_HUMAN Fu	16122582	2NNV	2NN7	2FOQ	61.72		Sc=5.71555, min distance = 2.21446
CAH1_HUMAN F	6102834 C	CAH2_HUMAN Fu	745994 2n	2NNS	2NMX	2FOQ	61.72	0.93	Sc=5.67037, min distance = 2.64022
CAH1_HUMAN F	6420183 E	CAH2_HUMAN Fu	16122582	2NNV	2NN7	2FOS	61.72		Sc=5.87585, min distance = 2.00739
CAH1_HUMAN F	6420183 E	CAH2_HUMAN Fu	745994 2n	2NNS	2NMX	2FOS	61.72	0.79	Sc=5.68516, min distance = 2.83267
CAH1_HUMAN F	6852129 a	CAH2_HUMAN Fu	745994 2n	2NNS	2NMX	1ZFQ	61.72		Sc=5.61035, min distance = 2.41577
CAH1_HUMAN F	71083 D-H	CAH2_HUMAN Fu	745994 2n	2NNS	2NMX	2EZ7	61.72		Sc=5.95856, min distance = 1.64294
CAH2_HUMAN F	17414308	CAH13_HUMAN F	1986 acet	3CZV	1YDB	3DA2			Sc=5.69756, min distance = 2.25266
CAH2_HUMAN F	1798 CID1	CAH1_HUMAN Fu	16122582	2NN7	2NNV	1BZM	61.72		Sc=5.60966, min distance = 1.68676
CAH2_HUMAN F	3830 kine	CHIT_YEAST Fu	1986 acet	2UY4	1YDB	2UY5			Sc=5.99875, min distance = 2.13881
CAHC_PEA Ful	69689 9-M	GSK3B_HUMAN F	311 citri	1R0E	1EKJ	1O9U			Sc=5.78164, min distance = 0.42087
CAMA_PSEPU F	11987634	TYTR_TRYCR Fu	444188 CI	1BZL	1Q1R	1AOG		0.97	Sc=6.43107, min distance = 1.79009
CAMA_PSEPU F	444502 CI	GSHR_HUMAN Fu	444188 CI	3DK9	1Q1R	1BWC		0.98	Sc=6.42704, min distance = 2.01374
CAMA_PSEPU F	446013 1,	O28603_ARCFU	444188 CI	1JNR	1Q1R	1JNZ		0.94	Sc=6.43248, min distance = 2.14790
CAMA_PSEPU F	446013 1,	O28604_ARCFU	444188 CI	1JNR	1Q1R	1JNZ		0.94	Sc=6.43156, min distance = 2.15746
CAMA_PSEPU F	448054 CI	FRDA_SHEFR Fu	444188 CI	1M64	1Q1R	1Y0P		0.97	Sc=6.42656, min distance = 2.12637
CAMA_PSEPU F	449465 CI	GSHR_HUMAN Fu	444188 CI	3DK9	1Q1R	3GRT		0.98	Sc=6.42898, min distance = 2.08984
CAMA_PSEPU F	6420174 C	O28603_ARCFU	444188 CI	1JNR	1Q1R	2FJB		0.92	Sc=6.16095, min distance = 1.93559
CAMT_MEDSA F	188380 Ad	MCES_ENCCU Fu	439155 Ad	1RI1	1SUI	1Z3C		0.91	Sc=6.58317, min distance = 1.90376
CAMT_MEDSA F	446535 CI	HNMT_HUMAN Fu	439155 Ad	2AOT	1SUI	1JQE		0.95	Sc=6.55397, min distance = 2.25357
CAMT_MEDSA F	60961 ade	PIMT_PYRFU Fu	439155 Ad	1JG1	1SUI	1JG2		0.84	Sc=6.30319, min distance = 2.66870
CAMT_MEDSA F	65482 sir	ERM_BACSU Ful	439155 Ad	1QAN	1SUI	1QAQ		0.88	Sc=6.59404, min distance = 2.11465
CAMT_MEDSA F	65482 sir	MCES_ENCCU Fu	439155 Ad	1RI1	1SUI	2HV9		0.88	Sc=6.58093, min distance = 2.22049
CAMT_MEDSA F	65482 sir	MTR1_RHOSH Fu	439155 Ad	1NW7	1SUI	1NW6		0.88	Sc=6.57476, min distance = 1.45143
CAMT_MEDSA F	65482 sir	SETD7_HUMAN F	439155 Ad	2F69	1SUI	3CBP		0.88	Sc=6.58646, min distance = 1.75847
CARA_ECOLI F	33032 L-g	SYQ_ECOLI Ful	5961 L-gl	1ZJW	1C3O	1O0C		0.84	Sc=5.80334, min distance = 2.17523
CARB_ECOLI F	1855 CID1	KITH_HHV11 Fu	6022 Aden	2VTK	1A9X	1E2P			Sc=6.18597, min distance = 0.44630

CARB_ECOLI Fı	3064778 H	ROCK1_HUMAN Fı	33113 gam	2V55	1BXR	2ETK			Sc=6.12745, min distance = 1.83373:
CARB_ECOLI Fı	3547 Fasu	ROCK1_HUMAN Fı	33113 gam	2V55	1BXR	2ESM			Sc=6.04867, min distance = 2.14503!
CARB_ECOLI Fı	444564 AD	BIOD_ECOLI Fu:	6022 Aden	1DAD	1A9X	1BS1		0.91	Sc=6.46696, min distance = 1.67778!
CARB_ECOLI Fı	444674 CI	ACCC_ECOLI Fu:	6022 Aden	2J9G	1A9X	2VR1	25.95	0.96	Sc=6.56921, min distance = 1.36606:
CARB_ECOLI Fı	444842 CI	CDK2_HUMAN Fu:	6022 Aden	1GY3	1A9X	1CKP			Sc=5.60314, min distance = 2.38609:
CARB_ECOLI Fı	445212 1e	KITH_HHV11 Fu:	6022 Aden	2VTK	1A9X	1E2K			Sc=6.04048, min distance = 2.49123:
CARB_ECOLI Fı	447628 [(	KITH_HHV11 Fu:	6022 Aden	2VTK	1A9X	1OF1			Sc=6.29843, min distance = 0.90138(
CARB_ECOLI Fı	448042 2e	ROCK1_HUMAN Fı	33113 gam	2V55	1BXR	2ETR			Sc=6.19882, min distance = 1.81182:
CARB_ECOLI Fı	448043 2g	ROCK1_HUMAN Fı	33113 gam	2V55	1BXR	3D9V			Sc=6.05597, min distance = 2.28936
CARB_ECOLI Fı	448222 NE	ENPL_CANFA Fu:	6022 Aden	1TC6	1A9X	1QY5		0.81	Sc=6.42323, min distance = 1.91103{
CARB_ECOLI Fı	4565 1h1r	CDK2_HUMAN Fu:	6022 Aden	1GY3	1A9X	1H1R			Sc=6.13447, min distance = 2.31952(
CARB_ECOLI Fı	5326976 1	CSK2A_MAIZE Fı	33113 gam	1LP4	1BXR	1ZOE			Sc=5.76592, min distance = 1.91873!
CARB_ECOLI Fı	5326977 1	CSK2A_MAIZE Fı	33113 gam	1LP4	1BXR	1ZOG			Sc=5.75225, min distance = 0.87258(
CARB_ECOLI Fı	5327148 C	IPKA_RABIT Fu:	6022 Aden	1JBP	1A9X	2ERZ			Sc=6.22985, min distance = 2.24711!
CARB_ECOLI Fı	5905 idox	KITH_HHV11 Fu:	6022 Aden	2VTK	1A9X	1KI7			Sc=6.17263, min distance = 0.96838:
CARB_ECOLI Fı	5957 Aden	ARSA1_ECOLX Fı	6022 Aden	1IHU	1A9X	1II0		0.99	Sc=6.5338, min distance = 1.191614
CARB_ECOLI Fı	5957 Aden	MALK_ECOLI Fu:	6022 Aden	2AWN	1A9X	1Q12		0.99	Sc=6.54228, min distance = 1.58583:
CARB_ECOLI Fı	5957 Aden	MUTS_ECOLI Fu:	6022 Aden	1OH7	1A9X	1W7A		0.99	Sc=6.53647, min distance = 2.01032
CARB_ECOLI Fı	5957 Aden	RK_BOVIN Full:	6022 Aden	3C4Z	1A9X	3C4W		0.99	Sc=6.523, min distance = 1.6628686(
CARB_ECOLI Fı	5957 Aden	Y059_METJA Fu:	6022 Aden	2J9D	1A9X	2J9C		0.99	Sc=6.53713, min distance = 1.44956!
CARB_ECOLI Fı	6083 aden	NDK_PYRHO Ful:	6022 Aden	2DYA	1A9X	2DXD		0.99	Sc=6.3787, min distance = 1.456798!
CARB_ECOLI Fı	6083 aden	PYGM_RABIT Fu:	8582 Inos	2QN7	1CE8	8GPB		0.79	Sc=6.39535, min distance = 1.87802:
CARB_ECOLI Fı	65059 Dec	RNAS1_BOVIN Fı	6022 Aden	1O0H	1A9X	2QCA		0.77	Sc=6.38744, min distance = 0.73165!
CARB_ECOLI Fı	6804 guar	NDK_PYRHO Ful:	6022 Aden	2DYA	1A9X	2DXF		0.8	Sc=6.42187, min distance = 1.83195:
CARB_ECOLI Fı	91532 AME	MTNK_BACSU Fu:	6022 Aden	2OLC	1A9X	2PUL		0.99	Sc=6.56387, min distance = 1.90141
CARB_ECOLI Fı	9817550 V	CDK2_HUMAN Fu:	6022 Aden	1GY3	1A9X	3BHV			Sc=6.42842, min distance = 0.74395!
CARM1_MOUSE I	12771338	MCES_HUMAN Fu:	439155 Ad	3BGV	2V74	3EPP		0.88	Sc=6.57421, min distance = 2.19247:
CARM1_MOUSE I	188380 Ad	MCES_ENCCU Fu:	439155 Ad	1RI1	2V74	1Z3C		0.91	Sc=6.56242, min distance = 2.31331!
CARM1_MOUSE I	446535 CI	HNMT_HUMAN Fu:	439155 Ad	2AOT	2V74	1JQE		0.95	Sc=6.52717, min distance = 2.44020!
CARM1_MOUSE I	60961 ade	PIMT_PYRFU Fu:	439155 Ad	1JG1	2V74	1JG2		0.84	Sc=6.30787, min distance = 2.83453:
CARM1_MOUSE I	65482 sir	ERM_BACSU Ful:	439155 Ad	1QAN	2V74	1QAQ		0.88	Sc=6.57004, min distance = 2.12275:
CARM1_MOUSE I	65482 sir	MCES_ENCCU Fu:	439155 Ad	1RI1	2V74	2HV9		0.88	Sc=6.55706, min distance = 2.24735!
CARM1_MOUSE I	65482 sir	PRMA_THET8 Fu:	439155 Ad	3EGV	2V74	2ZBR		0.88	Sc=6.57075, min distance = 2.48995{
CARP2_CANAL I	444568 HI	RENI_HUMAN Fu:	444569 CI	1HRN	1EAG	1BIL	26.14		Sc=5.66534, min distance = 1.51719:
CARP3_CANAL I	446912 1l	PLM2_PLAFA Fu:	5478883 p	1XDH	2H6T	1LEE			Sc=6.67152, min distance = 1.90553!
CARP3_CANAL I	446917 1l	PLM2_PLAFA Fu:	5478883 p	1XDH	2H6T	1LF2			Sc=6.66365, min distance = 2.11126:
CARP5_CANAL I	446912 1l	PLM2_PLAFA Fu:	5478883 p	1XDH	2QZX	1LEE			Sc=6.66318, min distance = 1.81172

CARP_CRYPA Fu	445687 CI	CARP_YEAST Ful	445692 CI	1FQ5	1EPP	1FQ4			Sc=6.06285, min distance = 2.48923
CARP_CRYPA Fu	445696 CI	CARP_YEAST Ful	445692 CI	1FQ5	1EPP	1FQ6			Sc=6.01356, min distance = 2.34817
CARP_YEAST Fu	16740994	CARP_CRYPA Ful	445692 CI	1EPP	1FQ5	2JJJ		0.79	Sc=6.04613, min distance = 2.46825
CARP_YEAST Fu	445265 CI	CARP_CRYPA Ful	445692 CI	1EPP	1FQ5	1E81			Sc=6.03828, min distance = 2.48583
CARP_YEAST Fu	445266 CI	CARP_CRYPA Ful	445692 CI	1EPP	1FQ5	1E82			Sc=6.05347, min distance = 2.55703
CAS1_STRCL Fu	3080614 C	HIF1A_HUMAN Fu	51 2-Oxop	1H2L	1DS1	1H2K			Sc=5.60174, min distance = 2.60486
CAS1_STRCL Fu	3080614 C	HIF1N_HUMAN Fu	51 2-Oxop	1H2L	1DS1	1H2K			Sc=5.60174, min distance = 2.58624
CAS1_STRCL Fu	3080614 C	JHD3A_HUMAN Fu	51 2-Oxop	2Q8C	1DS1	2OX0			Sc=5.60629, min distance = 2.52187
CAS1_STRCL Fu	439655 d-	HIF1N_HUMAN Fu	51 2-Oxop	1H2L	1DS1	1MZE			Sc=5.62759, min distance = 2.43545
CATA_PROMI Fu	3739 Adip	ALBU_HUMAN Ful	444124 HE	1O9X	1M85	2BXN			Sc=5.87484, min distance = 2.01015
CATA_PROMI Fu	444207 He	CP119_SULTO Fu	444124 HE	3B4X	1M85	1UE8		0.99	Sc=6.79214, min distance = 2.05204
CATA_PROMI Fu	444522 HE	NOS3_BOVIN Ful	444124 HE	1ZZS	1M85	2HX2		0.99	Sc=6.79451, min distance = 2.41531
CATA_PROMI Fu	444522 HE	NOSO_BACSU Ful	444124 HE	2AN0	1M85	2FC1		0.99	Sc=6.7959, min distance = 2.061899
CATE_ECOLI Fu	11957370	HMOX1_HUMAN Fu	444124 HE	1OZW	1IPH	1TWN			Sc=6.66854, min distance = 1.30308
CBH_CLOPE Ful	5881 Dehy	SDIS_PSEPU Ful	222528 DE	1E3V	2BJF	1E3R			Sc=5.99573, min distance = 2.10371
CBIF_BACME Fu	16750069	DPHB_PYRHO Ful	439155 Ad	2Z6R	1CBF	2PCI		0.92	Sc=6.0529, min distance = 2.061490
CBIF_BACME Fu	4369533 C	SETD8_HUMAN Fu	439155 Ad	1ZKK	1CBF	2BQZ		0.95	Sc=6.49827, min distance = 2.21958
CBIT_METJA Fu	51 2-Oxop	LDOX_ARATH Ful	78165 MES	1GP6	2YXD	1GP4			Sc=5.63559, min distance = 2.17784
CBIT_METTH Fu	446535 CI	HNMT_HUMAN Ful	439155 Ad	2AOT	1L3I	1JQE		0.95	Sc=6.54813, min distance = 2.34985
CBIT_METTH Fu	60961 ade	PIMT_PYRFU Ful	439155 Ad	1JG1	1L3I	1JG2		0.84	Sc=6.31053, min distance = 2.69234
CBIT_METTH Fu	65482 sir	MTR1_RHOSH Ful	439155 Ad	1NW7	1L3I	1NW6		0.88	Sc=6.57075, min distance = 2.01236
CBP2_WHEAT Fu	123831 N,	NOS3_BOVIN Ful	6322 L-ar	4NSE	1BCS	7NSE		0.89	Sc=5.96507, min distance = 1.96861
CBP2_WHEAT Fu	126494 N(	NOS1_RAT Full=	6322 L-ar	1OM4	1BCS	1K2S		0.76	Sc=5.99795, min distance = 2.29446
CBP2_WHEAT Fu	3311 S-Et	NOS1_RAT Full=	6322 L-ar	1OM4	1BCS	1K2U			Sc=5.65655, min distance = 2.57253
CBP2_WHEAT Fu	444288 CI	KARG_LIMPO Ful	6322 L-ar	1M15	1BCS	1P52			Sc=5.66571, min distance = 2.52954
CBP2_WHEAT Fu	447181 VI	NOS1_RAT Full=	6322 L-ar	1OM4	1BCS	1MMW		0.78	Sc=5.97793, min distance = 2.16733
CBP2_WHEAT Fu	9085 homo	NOS3_BOVIN Ful	6322 L-ar	4NSE	1BCS	1DM7		0.9	Sc=5.846, min distance = 1.9696766
CBP2_WHEAT Fu	9547942 C	NOS3_BOVIN Ful	6322 L-ar	4NSE	1BCS	2G6O		0.84	Sc=5.80968, min distance = 2.12395
CBP2_WHEAT Fu	9750 citr	ASSY_THET8 Ful	6322 L-ar	1KOR	1BCS	1J1Z		0.78	Sc=5.80338, min distance = 2.12028
CBR1_HUMAN Fu	444104 II	GSTP1_MOUSE Fu	124886 gl	1GSY	3BHJ	1GLP		0.9	Sc=5.86419, min distance = 2.52452
CBR1_PIG Full	15942680	6PGD_LACLM Ful	5886 NADP	2IZ0	1N5D	2IZ1			Sc=6.36318, min distance = 1.11812
CBR3_HUMAN Fu	15942680	6PGD_LACLM Ful	5886 NADP	2IZ0	2HRB	2IZ1			Sc=6.35639, min distance = 1.20589
CBR3_HUMAN Fu	6022 Aden	HMDH_HUMAN Ful	5886 NADP	1DQA	2HRB	1HW8			Sc=5.99854, min distance = 1.98984
CBS_HUMAN Ful	11957353	HBA_HORSE Full	444124 HE	1Y8I	1JBQ	1IWH		0.87	Sc=6.40271, min distance = 2.19247
CBS_HUMAN Ful	11957353	HBB_HORSE Full	444124 HE	1Y8I	1JBQ	1IWH		0.87	Sc=6.80363, min distance = 2.12129
CBS_HUMAN Ful	11957371	HMOX1_HUMAN Fu	444124 HE	1OZW	1JBQ	1TWR			Sc=6.66234, min distance = 2.10081
CBS_HUMAN Ful	11957385	MYG_PHYCA Full	444124 HE	1U7R	1JBQ	2CMM			Sc=6.36081, min distance = 2.11917

CBS_HUMAN Fu	444097 He	HBA_HUMAN Ful	444124 HE	1NQP	1JBQ	1RPS	0.95	Sc=6.48162, min distance = 1.99123
CBS_HUMAN Fu	444207 He	CP119_SULTO Fu	444124 HE	3B4X	1JBQ	1UE8	0.99	Sc=6.77962, min distance = 2.18923
CBS_HUMAN Fu	444207 He	PRXC_CALFU Ful	444124 HE	2CPO	1JBQ	1CPO	0.99	Sc=6.4775, min distance = 2.025760
CCA_ARCFU Fu	11608401	KAPCA_BOVIN Fu	5957 Aden	1Q24	1R8B	2UVY		Sc=6.04068, min distance = 2.06850
CCA_ARCFU Fu	6022 Aden	CLCN5_HUMAN Fu	5957 Aden	2J9L	1R8B	2JA3	0.99	Sc=5.91441, min distance = 2.11348
CCA_ARCFU Fu	6022 Aden	Y059_METJA Ful	5957 Aden	2J9C	1R8B	2J9D	0.99	Sc=5.77732, min distance = 2.37519
CCA_BACST Fu	11708454	KAPCA_BOVIN Fu	5957 Aden	1Q24	1MIW	2VNW		Sc=6.17435, min distance = 1.89847
CCA_BACST Fu	3540 1yds	KAPCA_BOVIN Fu	5957 Aden	1Q24	1MIW	1YDS		Sc=5.86359, min distance = 2.24737
CCA_BACST Fu	3547 Fasu	KAPCA_BOVIN Fu	5957 Aden	1Q24	1MIW	1Q8W		Sc=5.89549, min distance = 2.18124
CCA_BACST Fu	444852 CI	ATPB_BOVIN Ful	5957 Aden	2V7Q	1MIW	1COW	0.91	Sc=6.45821, min distance = 1.70682
CCA_BACST Fu	447840 CI	PHKG1_RABIT Fu	5957 Aden	1QL6	1MIW	1PHK		Sc=5.98, min distance = 1.76976749
CCA_BACST Fu	447916 ac	RIO1_ARCFU Ful	5957 Aden	1ZP9	1MIW	1ZTF	0.94	Sc=6.24577, min distance = 2.33626
CCA_BACST Fu	449240 1y	KAPCA_BOVIN Fu	5957 Aden	1Q24	1MIW	1YDR		Sc=5.94592, min distance = 2.09393
CCA_BACST Fu	6022 Aden	DDL_THET8 Ful	5957 Aden	2ZDQ	1MIW	2ZDH	0.99	Sc=6.30754, min distance = 2.56069
CCA_BACST Fu	6022 Aden	HSP7F_YEAST Fu	5957 Aden	3D2F	1MIW	3C7N	0.99	Sc=6.40423, min distance = 2.13277
CCA_BACST Fu	6022 Aden	MUTS_ECOLI Ful	5957 Aden	1W7A	1MIW	1OH7	0.99	Sc=6.38867, min distance = 2.09985
CCA_BACST Fu	6022 Aden	PSPF_ECOLI Ful	5957 Aden	2C96	1MIW	2C98	0.99	Sc=6.43242, min distance = 2.14436
CCA_BACST Fu	6022 Aden	PURK_ECOLI Ful	5957 Aden	3ETH	1MIW	3ETJ	0.99	Sc=6.40508, min distance = 1.93757
CCA_BACST Fu	6022 Aden	PURP_METJA Ful	5957 Aden	2R7L	1MIW	2R7N	0.99	Sc=6.02241, min distance = 2.30284
CCA_BACST Fu	6022 Aden	Q72H90_THET2	5957 Aden	2BEK	1MIW	2BEJ	0.99	Sc=6.40345, min distance = 2.06108
CCA_BACST Fu	6022 Aden	REX_BACSU Ful	5957 Aden	2VT3	1MIW	2VT2	0.99	Sc=5.94395, min distance = 1.90064
CCA_BACST Fu	6022 Aden	Y059_METJA Ful	5957 Aden	2J9C	1MIW	2J9D	0.99	Sc=6.41137, min distance = 2.08263
CCA_BACST Fu	6083 aden	PURP_METJA Ful	5957 Aden	2R7L	1MIW	2R7M	0.98	Sc=6.31031, min distance = 2.56074
CCA_BACST Fu	6083 aden	Y059_METJA Ful	5957 Aden	2J9C	1MIW	2J9D	0.98	Sc=5.8653, min distance = 2.129442
CCA_BACST Fu	65070 dUT	Q381M1_9TRYP	6176 Cyti	2Q0C	1MIY	2NOM	0.8	Sc=6.35471, min distance = 2.03755
CCMH_PSEAE Fu	439353 be	O96048_LUMTE	8172 Trig	2DRY	2HL7	2DS0		Sc=5.75684, min distance = 1.70805
CCPR_YEAST Fu	11957353	HBA_HORSE Ful	444098 HE	2D5X	2EUT	1IWH	0.83	Sc=6.75934, min distance = 2.43456
CCPR_YEAST Fu	11957353	HBA_HORSE Ful	444522 HE	1Y8K	3E2O	1IWH	0.87	Sc=6.75861, min distance = 2.31772
CCPR_YEAST Fu	11957355	CYB_BOVIN Ful	444522 HE	1BGY	3E2O	1L0L	0.81	Sc=6.813, min distance = 2.2054137
CCPR_YEAST Fu	11957361	CYB_BOVIN Ful	444522 HE	1BGY	3E2O	1NTZ	0.84	Sc=6.77308, min distance = 2.23905
CCPR_YEAST Fu	11957363	HBA_HUMAN Ful	444522 HE	2DN1	3E2O	1RQA	0.81	Sc=6.77383, min distance = 1.97298
CCPR_YEAST Fu	11957385	MYG_PHYCA Ful	444098 HE	1A6M	2EUT	2CMM		Sc=6.21202, min distance = 2.44804
CCPR_YEAST Fu	11957385	MYG_PHYCA Ful	444207 He	1VXG	2CCP	2CMM		Sc=6.08782, min distance = 2.22808
CCPR_YEAST Fu	11957385	MYG_PHYCA Ful	444522 HE	1VXD	3E2O	2CMM		Sc=6.11427, min distance = 2.43692
CCPR_YEAST Fu	16741062	MYG_PHYCA Ful	444098 HE	1A6M	2EUT	1MBN	0.84	Sc=6.75934, min distance = 2.21793
CCPR_YEAST Fu	16741062	MYG_PHYCA Ful	444522 HE	1VXD	3E2O	1MBN	0.88	Sc=6.76669, min distance = 2.19347
CCPR_YEAST Fu	16741183	MYG_PHYCA Ful	444207 He	1VXG	2CCP	2EKT	0.93	Sc=6.52282, min distance = 2.18315

CCPR_YEAST Fu	24139 ace	Q309D1_9AGAR I	78165 MES	2BWR	1BES	2C4D			Sc=6.08392, min distance = 1.76732
CCPR_YEAST Fu	4369228 C	PER_COPCI Full	444522 HE	1H3J	3E2O	1LYC	24.12	0.96	Sc=6.77486, min distance = 2.01548
CCPR_YEAST Fu	8200 TETR	LMBL1_HUMAN Fu	78165 MES	1OZ2	1BES	2RHI			Sc=5.69304, min distance = 2.17704
CD1D1_MOUSE I	12389 Tet	LTC4S_HUMAN Fu	985 palmi	2UUH	2FIK	2UUI			Sc=6.02929, min distance = 2.02355
CD1D1_MOUSE I	14253 Cet	FABP4_MOUSE Fu	985 palmi	1LIE	2FIK	1LIC			Sc=5.73291, min distance = 2.09709
CD1D1_MOUSE I	440552 1b	MBL1_RAT Full=	439554 fu	3KMB	1ZHN	1AFB			Sc=6.02522, min distance = 2.20881
CD1D1_MOUSE I	446578 1r	MBL1_RAT Full=	439554 fu	3KMB	1ZHN	1KWW		0.96	Sc=5.62916, min distance = 2.47257
CD1D1_MOUSE I	447681 al	S14L2_HUMAN Fu	985 palmi	1O6U	2FIK	1OLM			Sc=6.43541, min distance = 2.04784
CD209_HUMAN I	445948 D-	Q9HYN5_PSEAE I	439353 be	1W8F	1SL5	1OXC			Sc=5.71337, min distance = 1.77187
CD209_HUMAN I	446578 1r	MBL1_RAT Full=	439554 fu	3KMB	1SL5	1KWW		0.96	Sc=5.62687, min distance = 2.31845
CDC2H_PLAFK I	10138993	CDK2_HUMAN Fu	5288641 i	1E9H	1V0O	2R64	63.31		Sc=6.38883, min distance = 2.50470
CDC2H_PLAFK I	10224714	CDK2_HUMAN Fu	5288641 i	1E9H	1V0O	3DDP	63.31		Sc=6.55888, min distance = 1.88558
CDC2H_PLAFK I	11957417	CDK2_HUMAN Fu	5288641 i	1E9H	1V0O	2I40	63.31		Sc=6.34181, min distance = 1.87858
CDC2H_PLAFK I	16214823	CDK2_HUMAN Fu	5288641 i	1E9H	1V0O	2UZB	63.31		Sc=6.14747, min distance = 2.17739
CDC2H_PLAFK I	16214825	CDK2_HUMAN Fu	5288641 i	1E9H	1V0O	2UZE	63.31		Sc=6.30452, min distance = 2.68075
CDC2H_PLAFK I	16214826	CDK2_HUMAN Fu	5288641 i	1E9H	1V0O	2UZL	63.31		Sc=6.18641, min distance = 2.09823
CDC2H_PLAFK I	1694 TBBt	CDK2_HUMAN Fu	5288641 i	1E9H	1V0O	1P5E	63.31		Sc=5.73522, min distance = 0.59135
CDC2H_PLAFK I	23653518	CDK2_HUMAN Fu	5288641 i	1E9H	1V0O	2R3J	63.31		Sc=6.30014, min distance = 2.45796
CDC2H_PLAFK I	23653520	CDK2_HUMAN Fu	5288641 i	1E9H	1V0O	2R3L	63.31		Sc=6.30014, min distance = 2.33659
CDC2H_PLAFK I	23653522	CDK2_HUMAN Fu	5288641 i	1E9H	1V0O	2R3N	63.31		Sc=6.35717, min distance = 2.25944
CDC2H_PLAFK I	23653526	CDK2_HUMAN Fu	5288641 i	1E9H	1V0O	2R3R	63.31		Sc=6.29556, min distance = 2.15251
CDC2H_PLAFK I	23727981	CDK2_HUMAN Fu	5288641 i	1E9H	1V0O	3BHT	63.31		Sc=6.1949, min distance = 2.215736
CDC2H_PLAFK I	24864080	CDK2_HUMAN Fu	5288641 i	1E9H	1V0O	2VTR	63.31		Sc=6.00955, min distance = 2.78309
CDC2H_PLAFK I	24901723	CDK2_HUMAN Fu	5288641 i	1E9H	1V0O	2W06	63.31		Sc=6.32861, min distance = 2.25829
CDC2H_PLAFK I	24916751	CDK2_HUMAN Fu	5288641 i	1E9H	1V0O	3DOG	63.31		Sc=6.40849, min distance = 2.04872
CDC2H_PLAFK I	24963033	CDK2_HUMAN Fu	5288641 i	1E9H	1V0O	2W05	63.31		Sc=6.27947, min distance = 1.78725
CDC2H_PLAFK I	25021197	CDK2_HUMAN Fu	5288641 i	1E9H	1V0O	3EOC	63.31		Sc=6.29719, min distance = 2.17095
CDC2H_PLAFK I	4369136 C	CDK2_HUMAN Fu	5288641 i	1E9H	1V0O	1DM2	63.31		Sc=6.25697, min distance = 2.90594
CDC2H_PLAFK I	445840 di	CDK2_HUMAN Fu	5288641 i	1E9H	1V0O	1GII	63.31		Sc=6.26256, min distance = 2.66934
CDC2H_PLAFK I	447652 Im	CDK2_HUMAN Fu	5288641 i	1E9H	1V0O	1OIQ	63.31		Sc=6.16445, min distance = 2.94581
CDC2H_PLAFK I	447653 CI	CDK2_HUMAN Fu	5288641 i	1E9H	1V0O	1OIR	63.31		Sc=6.45049, min distance = 2.44151
CDC2H_PLAFK I	447654 CI	CDK2_HUMAN Fu	5288641 i	1E9H	1V0O	1OIT	63.31		Sc=6.21174, min distance = 2.09536
CDC2H_PLAFK I	447655 1c	CDK2_HUMAN Fu	5288641 i	1E9H	1V0O	1OIU	63.31		Sc=6.34611, min distance = 1.00553
CDC2H_PLAFK I	447656 1c	CDK2_HUMAN Fu	5288641 i	1E9H	1V0O	1OIY	63.31		Sc=6.50391, min distance = 1.50327
CDC2H_PLAFK I	447962 1p	CDK2_HUMAN Fu	5288641 i	1E9H	1V0O	2C5N	63.31		Sc=6.17619, min distance = 2.46737
CDC2H_PLAFK I	447967 CI	CDK2_HUMAN Fu	5288641 i	1E9H	1V0O	1PYE	63.31		Sc=6.40312, min distance = 2.17050
CDC2H_PLAFK I	4566 1h1s	CDK2_HUMAN Fu	5288641 i	1E9H	1V0O	1H1S	63.31		Sc=6.35467, min distance = 1.48434

CDC2H_PLAFK	5287845 N	CDK2_HUMAN Fu	5288641 i	1E9H	1V0O	2BHE	63.31	0.8	Sc=6.20591, min distance = 3.11938
CDC2H_PLAFK	5326844 C	CDK2_HUMAN Fu	5288641 i	1E9H	1V0O	1URW	63.31		Sc=6.48377, min distance = 2.14609
CDC2H_PLAFK	5327096 2	CDK2_HUMAN Fu	5288641 i	1E9H	1V0O	2BTR	63.31		Sc=6.10815, min distance = 2.35535
CDC2H_PLAFK	5327097 2	CDK2_HUMAN Fu	5288641 i	1E9H	1V0O	2BTS	63.31		Sc=5.9415, min distance = 2.249018
CDC2H_PLAFK	5331010 c	CDK2_HUMAN Fu	5288641 i	1E9H	1V0O	2BKZ	63.31		Sc=6.32735, min distance = 1.67781
CDC2H_PLAFK	6420138 2	CDK2_HUMAN Fu	5288641 i	1E9H	1V0O	2UUE	63.31		Sc=6.35712, min distance = 1.48457
CDC2H_PLAFK	6420139 C	CDK2_HUMAN Fu	5288641 i	1E9H	1V0O	2C5V	63.31		Sc=6.17098, min distance = 1.93473
CDC2H_PLAFK	6918710 5	CDK2_HUMAN Fu	5288641 i	1E9H	1V0O	3EJ1	63.31		Sc=6.13424, min distance = 2.78929
CDC2H_PLAFK	9817550 V	CDK2_HUMAN Fu	5288641 i	1E9H	1V0O	3BHV	63.31		Sc=6.38435, min distance = 2.19147
CDC42_HUMAN	188347 pp	ARF1_BOVIN Fu	8977 1dar	1R8S	2NGR	1R8Q		0.99	Sc=6.05226, min distance = 1.97236
CDC42_HUMAN	188347 pp	ARL5A_HUMAN Fu	8977 1dar	2H17	2NGR	1ZJ6		0.99	Sc=6.05443, min distance = 2.41222
CDC42_HUMAN	37792 gam	GNA13_MOUSE Fu	8977 1dar	1ZCB	2NGR	3CX8		0.99	Sc=6.51572, min distance = 2.05979
CDC42_HUMAN	37792 gam	GNAI1_RAT Ful	8977 1dar	1SVK	2NGR	1AS0		0.99	Sc=6.05478, min distance = 2.41525
CDC42_HUMAN	37792 gam	RAB6B_HUMAN Fu	8977 1dar	2E9S	2NGR	2FFQ		0.99	Sc=6.51984, min distance = 2.51954
CDC42_HUMAN	37792 gam	RAC1_HUMAN Fu	8977 1dar	1RYF	2NGR	2FJU		0.99	Sc=6.0789, min distance = 2.349572
CDC42_HUMAN	37792 gam	RB11A_HUMAN Fu	8977 1dar	1OIX	2NGR	1OIW		0.99	Sc=5.7784, min distance = 2.616638
CDC42_HUMAN	37792 gam	RHOA_HUMAN Fu	8977 1dar	1TX4	2NGR	1CXZ		0.99	Sc=6.0761, min distance = 2.241561
CDC42_HUMAN	445871 CI	RASH_HUMAN Fu	36735 Gpp	1CTQ	1NF3	1GNP			Sc=6.71675, min distance = 2.47893
CDC42_HUMAN	445871 CI	RASH_HUMAN Fu	8977 1dar	2CE2	2NGR	1GNP			Sc=6.38819, min distance = 2.35840
CDC42_HUMAN	446248 CI	RAN_HUMAN Ful	8977 1dar	3CH5	2NGR	1IBR			Sc=6.53998, min distance = 2.28724
CDC42_HUMAN	446248 CI	RASH_HUMAN Fu	8977 1dar	2CE2	2NGR	1IAQ			Sc=6.5116, min distance = 2.450446
CDC42_HUMAN	446248 CI	RASH_HUMAN Fu	93082 Gab	121P	2ODB	1IAQ			Sc=6.49148, min distance = 2.73241
CDC42_HUMAN	6802 guar	DPOL_BPR69 Fu	8977 1dar	1CLQ	2NGR	1IH7		0.94	Sc=6.2848, min distance = 2.426892
CDC42_HUMAN	6804 guar	DPOL_BPR69 Fu	8977 1dar	1CLQ	2NGR	1WAJ		0.99	Sc=6.36681, min distance = 2.22069
CDC42_HUMAN	6804 guar	EF1A_YEAST Fu	8977 1dar	2B7B	2NGR	1G7C		0.99	Sc=5.94771, min distance = 2.82523
CDC61_SULSO	1694 TBBt	CDK2_HUMAN Fu	6022 Aden	1GY3	2QBY	1P5E			Sc=5.65758, min distance = 1.91455
CDC61_SULSO	398148 1e	CDK2_HUMAN Fu	6022 Aden	1GY3	2QBY	1E1X			Sc=6.245, min distance = 1.9008482
CDC61_SULSO	444564 AD	BIOD_ECOLI Fu	6022 Aden	1DAD	2QBY	1BS1		0.91	Sc=6.43242, min distance = 2.12186
CDC61_SULSO	445940 O6	CDK2_HUMAN Fu	6022 Aden	1GY3	2QBY	1GZ8			Sc=6.17643, min distance = 2.42290
CDC61_SULSO	445966 O6	CDK2_HUMAN Fu	6022 Aden	1GY3	2QBY	1H0V			Sc=6.23554, min distance = 1.84834
CDC61_SULSO	447916 ac	RIO1_ARCFU Fu	6022 Aden	1ZTH	2QBY	1ZTF		0.95	Sc=6.2074, min distance = 2.675788
CDC61_SULSO	447955 1p	CDK2_HUMAN Fu	6022 Aden	1GY3	2QBY	1PXI			Sc=5.6805, min distance = 1.906123
CDC61_SULSO	4564 1e1v	CDK2_HUMAN Fu	6022 Aden	1GY3	2QBY	1E1V			Sc=6.18981, min distance = 2.05953
CDC61_SULSO	4565 1h1r	CDK2_HUMAN Fu	6022 Aden	1GY3	2QBY	1H1R			Sc=6.09002, min distance = 2.23964
CDC61_SULSO	5957 Aden	BIOD_ECOLI Fu	6022 Aden	1DAD	2QBY	1A82		0.99	Sc=6.48858, min distance = 2.24243
CDC61_SULSO	5957 Aden	PURL_THEMA Fu	6022 Aden	2HRU	2QBY	2HS0		0.99	Sc=6.0743, min distance = 2.379921
CDC61_SULSO	6083 aden	PSPF_ECOLI Fu	6022 Aden	2C98	2QBY	2VII		0.99	Sc=6.34967, min distance = 2.51347

CDC61_SULSO I	60961 ade	SKY1_YEAST Fu	6022 Aden	1Q8Y	2QBY	1Q97		0.95	Sc=6.25346, min distance = 2.039479
CDC61_SULSO I	6132 Cyti	ECX2_PYRAB Fu	6022 Aden	2PO0	2QBY	2PO2			Sc=6.29835, min distance = 2.235796
CDC61_SULSO I	72194 2-C	ENPL_CANFA Fu	6022 Aden	1TC6	2QBY	1QYE		0.89	Sc=6.14143, min distance = 2.127781
CDC61_SULSO I	91532 AMP	AROK_MYCTU Fu	6022 Aden	2IYV	2QBY	1ZYU		0.99	Sc=6.51375, min distance = 2.207449
CDC63_SULSO I	1694 TBBt	CDK2_HUMAN Fu	6022 Aden	1GY3	2QBY	1P5E			Sc=5.68884, min distance = 1.827826
CDC63_SULSO I	444564 AD	BIOD_ECOLI Fu	6022 Aden	1DAD	2QBY	1BS1		0.91	Sc=5.71284, min distance = 2.333997
CDC63_SULSO I	447004 CI	MYS2_DICDI Fu	6022 Aden	1VOM	2QBY	1LVK			Sc=6.5341, min distance = 2.2356408
CDC63_SULSO I	5957 Aden	MUTS_ECOLI Fu	6022 Aden	1OH7	2QBY	1W7A		0.99	Sc=6.05478, min distance = 2.088644
CDC63_SULSO I	72194 2-C	ENPL_CANFA Fu	6022 Aden	1TC6	2QBY	1QYE		0.89	Sc=6.17804, min distance = 2.096188
CDC6_AERPE Fu	398148 1e	CDK2_HUMAN Fu	6022 Aden	1GY3	2V1U	1E1X			Sc=6.22816, min distance = 1.696172
CDC6_AERPE Fu	445966 O6	CDK2_HUMAN Fu	6022 Aden	1GY3	2V1U	1H0V			Sc=6.21668, min distance = 2.152910
CDC6_AERPE Fu	4564 1e1v	CDK2_HUMAN Fu	6022 Aden	1GY3	2V1U	1E1V			Sc=6.17225, min distance = 2.808673
CDC6_AERPE Fu	4565 1h1r	CDK2_HUMAN Fu	6022 Aden	1GY3	2V1U	1H1R			Sc=6.38583, min distance = 1.315041
CDC6_AERPE Fu	5957 Aden	BIOD_ECOLI Fu	6022 Aden	1DAD	2V1U	1A82		0.99	Sc=6.48384, min distance = 2.300607
CDC6_AERPE Fu	5957 Aden	KTHY_HUMAN Fu	6022 Aden	1NN3	2V1U	1E2Q		0.99	Sc=6.47797, min distance = 2.101660
CDC6_AERPE Fu	6083 aden	PSPF_ECOLI Fu	6022 Aden	2C98	2V1U	2VII		0.99	Sc=5.89926, min distance = 2.595281
CDC6_AERPE Fu	6132 Cyti	ECX2_PYRAB Fu	6022 Aden	2PO0	2V1U	2PO2			Sc=6.30041, min distance = 2.023843
CDC6_AERPE Fu	91532 AMP	BIOD_ECOLI Fu	6022 Aden	1DAD	2V1U	1DAG		0.99	Sc=6.50006, min distance = 2.362238
CDGT1_BACCI I	193758 Va	CDGT2_BACCI Fu	79025 alp	1D3C	1CGU	1DTU	74.31		Sc=5.90004, min distance = 0.764087
CDGT1_BACCI I	444915 Gl	CDGT2_BACCI Fu	79025 alp	1D3C	1CGU	1CXL	74.31	0.85	Sc=5.83046, min distance = 0.375776
CDGT1_BACCI I	446000 CI	AMYP_PIG Full	79025 alp	1HX0	1CGU	1JFH		0.83	Sc=5.62192, min distance = 2.790630
CDGT1_BACCI I	446564 LI	BACR_HALSA Fu	79025 alp	1DZE	1CGU	1JV7			Sc=5.71926, min distance = 0.626409
CDGT1_BACCI I	446685 Va	CDGT2_BACCI Fu	79025 alp	1D3C	1CGU	1KCK	74.31		Sc=5.93216, min distance = 1.384602
CDGT1_BACCI I	447600 CI	AMY1_HORVU Fu	79025 alp	1RP8	1CGU	1P6W	22.83	0.83	Sc=5.74491, min distance = 1.701705
CDGT1_BACCI I	448009 TF	AMY1_HUMAN Fu	444139 CI	3DHP	6CGT	1Q4N			Sc=5.66576, min distance = 2.764820
CDGT2_BACCI I	125409 be	AMYB_BACCE Fu	79025 alp	1VEM	1D3C	1J10	37.84	0.93	Sc=5.83878, min distance = 1.039352
CDGT2_BACCI I	13455857	IGKC_MOUSE Fu	79025 alp	3BZ4	1D3C	3C6S		0.83	Sc=5.82976, min distance = 0.883840
CDGT2_BACCI I	15600 DEC	BACR_HALSA Fu	79025 alp	1DZE	1D3C	1XJI			Sc=5.62596, min distance = 1.066885
CDGT2_BACCI I	164795 N(	RBCMT_PEA Full	23831 HEP	1MLV	1OT1	2H2J			Sc=5.6966, min distance = 2.0495394
CDGT2_BACCI I	1983 acet	TRFL_BOVIN Fu	79025 alp	2DWJ	1D3C	2OCU			Sc=5.79875, min distance = 1.171051
CDGT2_BACCI I	444140 CI	CDGT_THETU Fu	193758 Va	3BMW	1DTU	1A47	70.41		Sc=5.69173, min distance = 2.422170
CDGT2_BACCI I	444140 CI	CDGT_THETU Fu	444139 CI	3BMW	1DTU	1A47	70.41		Sc=5.6631, min distance = 2.4980110
CDGT2_BACCI I	444140 CI	CDGT_THETU Fu	79025 alp	3BMW	1D3C	1A47	70.41		Sc=5.63829, min distance = 1.101022
CDGT2_BACCI I	444809 Th	AMY1_HORVU Fu	79025 alp	1RP8	1D3C	1P6W	24.63	0.78	Sc=5.95248, min distance = 0.746045
CDGT2_BACCI I	446564 LI	BACR_HALSA Fu	79025 alp	1DZE	1D3C	1JV7			Sc=5.85492, min distance = 0.841958
CDGT2_BACCI I	449571 CI	CDGT1_BACCI Fu	79025 alp	1CGU	1D3C	6CGT	74.31		Sc=5.75553, min distance = 1.774496
CDGT2_BACCI I	5962 L-ly	RBCMT_PEA Full	23831 HEP	1MLV	1OT1	2H2E			Sc=5.6153, min distance = 2.2904975

CDGT2_BACCI	64960 Pol	AMYB_BACCE Fu	79025 alp	1VEM	1D3C	1VEN	37.84	0.89	Sc=5.73005, min distance = 1.26746
CDGT_BACS0 F	14257 Unc	BACR_HALSA Fu	79025 alp	1DZE	1UKS	1CWQ			Sc=5.6901, min distance = 1.280987
CDGT_BACS0 F	447607 1c	Q79G13_MYCTU	64689 bet	1UP0	1UKS	1UP2			Sc=5.62522, min distance = 2.21505
CDGT_THETU F	125409 be	AMYB_BACCE Fu	79025 alp	1VEM	3BMW	1J10	40	0.93	Sc=5.8193, min distance = 0.872594
CDGT_THETU F	444809 Th	AMY1_HORVU Fu	79025 alp	1RP8	3BMW	1P6W		0.78	Sc=5.90354, min distance = 1.00185
CDGT_THETU F	444915 Gl	CDGT2_BACCI F	79025 alp	1D3C	3BMW	1CXL	70.41	0.85	Sc=5.78478, min distance = 0.68144
CDGT_THETU F	446685 Va	CDGT2_BACCI F	193758 Va	1DTU	3BMW	1KCK	70.41		Sc=5.7665, min distance = 2.623224
CDGT_THETU F	446685 Va	CDGT2_BACCI F	444139 CI	1DTU	3BMW	1KCK	70.41		Sc=5.77279, min distance = 2.27456
CDGT_THETU F	446685 Va	CDGT2_BACCI F	79025 alp	1D3C	3BMW	1KCK	70.41		Sc=5.99282, min distance = 0.75885
CDH_PHACH Fu	11957370	HMOX1_HUMAN F	444124 HE	1OZW	1D7B	1TWN			Sc=6.64931, min distance = 2.07040
CDH_PHACH Fu	11957385	MYG_PHYCA Ful	444098 HE	1A6M	1PL3	2CMM			Sc=6.27294, min distance = 2.08341
CDH_PHACH Fu	11957385	MYG_PHYCA Ful	444124 HE	1U7R	1D7B	2CMM			Sc=6.25433, min distance = 2.00606
CDK2_HUMAN F	10109823	PIM1_HUMAN Fu	444345 1c	1YHS	1AQ1	3CY3	31.71		Sc=6.33385, min distance = 2.85946
CDK2_HUMAN F	10125830	CHK1_HUMAN Fu	444345 1c	1NVR	1AQ1	2AYP	35.05		Sc=6.36081, min distance = 2.33877
CDK2_HUMAN F	10156987	CHK1_HUMAN Fu	72271 7-H	1NVQ	1PKD	2HXQ	35.05		Sc=6.35263, min distance = 1.98802
CDK2_HUMAN F	11314340	KAPCA_BOVIN F	444345 1c	1STC	1AQ1	2UZT	34.78		Sc=6.32862, min distance = 2.26665
CDK2_HUMAN F	11348631	CHK1_HUMAN Fu	444345 1c	1NVR	1AQ1	2E9U	35.05		Sc=6.21736, min distance = 2.09677
CDK2_HUMAN F	11348631	CHK1_HUMAN Fu	72271 7-H	1NVQ	1PKD	2E9U	35.05		Sc=6.21852, min distance = 2.13037
CDK2_HUMAN F	11553058	LCK_HUMAN Ful	444345 1c	1QPD	1AQ1	2OF2	28.39		Sc=6.17416, min distance = 2.46301
CDK2_HUMAN F	15991572	LCK_HUMAN Ful	444345 1c	1QPD	1AQ1	2OF4	28.39		Sc=6.14432, min distance = 2.62041
CDK2_HUMAN F	16122608	PDPK1_HUMAN F	72271 7-H	1OKZ	1PKD	2PE1	29.3		Sc=6.32871, min distance = 2.24135
CDK2_HUMAN F	16122643	CHK1_HUMAN Fu	444345 1c	1NVR	1AQ1	2YWP	35.05		Sc=6.26319, min distance = 2.18017
CDK2_HUMAN F	16122643	CHK1_HUMAN Fu	72271 7-H	1NVQ	1PKD	2YWP	35.05		Sc=6.28602, min distance = 2.15148
CDK2_HUMAN F	1907917 2	CHK1_HUMAN Fu	444345 1c	1NVR	1AQ1	2CGW	35.05		Sc=6.10229, min distance = 2.81622
CDK2_HUMAN F	23657800	CHK1_HUMAN Fu	444345 1c	1NVR	1AQ1	2E9P	35.05		Sc=6.2584, min distance = 2.088740
CDK2_HUMAN F	23657800	CHK1_HUMAN Fu	72271 7-H	1NVQ	1PKD	2E9P	35.05		Sc=6.25731, min distance = 2.06697
CDK2_HUMAN F	24752838	CHK1_HUMAN Fu	444345 1c	1NVR	1AQ1	2QHM	35.05		Sc=6.03438, min distance = 2.20444
CDK2_HUMAN F	24752838	CHK1_HUMAN Fu	72271 7-H	1NVQ	1PKD	2QHM	35.05		Sc=6.0326, min distance = 2.708783
CDK2_HUMAN F	24905143	PK3CG_HUMAN F	444345 1c	1E8Z	1AQ1	2V4L			Sc=5.98927, min distance = 2.92128
CDK2_HUMAN F	24905144	PK3CG_HUMAN F	444345 1c	1E8Z	1AQ1	3ENE			Sc=6.0821, min distance = 2.587985
CDK2_HUMAN F	33113 gam	IRAK4_HUMAN F	444345 1c	2NRY	1AQ1	2OID	35.75		Sc=5.98891, min distance = 2.08027
CDK2_HUMAN F	3973 nche	PIM1_HUMAN Fu	444345 1c	1YHS	1AQ1	1YI3	31.71		Sc=5.87431, min distance = 2.40136
CDK2_HUMAN F	444564 AD	BIOD_ECOLI Fu	6022 Aden	1DAD	1GY3	1BS1		0.91	Sc=6.31795, min distance = 2.17932
CDK2_HUMAN F	444564 AD	MYS2_DICDI Fu	6022 Aden	1VOM	1GY3	1W9I		0.91	Sc=5.91756, min distance = 2.00593
CDK2_HUMAN F	448005 CI	GSK3B_HUMAN F	6022 Aden	1J1C	1GY3	1Q3W	35.44		Sc=6.34373, min distance = 0.79071
CDK2_HUMAN F	448043 2g	KAPCA_BOVIN F	444345 1c	1STC	1AQ1	1Q8U	34.78		Sc=5.97066, min distance = 2.50444
CDK2_HUMAN F	448222 NE	ENPL_CANFA Fu	6022 Aden	1TC6	1GY3	1QY5		0.81	Sc=5.92946, min distance = 2.45482

CDK2_HUMAN F	448991 Pu	CDC2H_PLAFK F	5288641 i	1V0O	1E9H	1V0P	63.31		Sc=6.52668, min distance = 1.54910
CDK2_HUMAN F	5287844 i	GSK3B_HUMAN F	6022 Aden	1J1C	1GY3	1UV5	35.44		Sc=6.2139, min distance = 2.344204
CDK2_HUMAN F	5326739 i	CDK5_HUMAN Fu	160355 rc	1UNL	3DDQ	1UNH	60		Sc=6.27969, min distance = 2.435242
CDK2_HUMAN F	5326739 i	GSK3B_HUMAN F	6022 Aden	1J1C	1GY3	1Q41	35.44		Sc=6.30199, min distance = 2.181817
CDK2_HUMAN F	5326843 A	CDK5_HUMAN Fu	160355 rc	1UNL	3DDQ	1UNG	60		Sc=6.09535, min distance = 2.347834
CDK2_HUMAN F	5326935 1	HS90A_HUMAN F	6022 Aden	1BYQ	1GY3	1YC4			Sc=6.34616, min distance = 2.213474
CDK2_HUMAN F	5327103 2	HS90A_HUMAN F	6022 Aden	1BYQ	1GY3	2BYH			Sc=6.41316, min distance = 2.003543
CDK2_HUMAN F	5327104 2	HS90A_HUMAN F	6022 Aden	1BYQ	1GY3	2BYI			Sc=6.43407, min distance = 2.206103
CDK2_HUMAN F	5327121 I	PIM1_HUMAN Fu	444345 1c	1YHS	1AQ1	2C3I	31.71		Sc=6.11904, min distance = 2.518049
CDK2_HUMAN F	5327122 C	CHK1_HUMAN Fu	444345 1c	1NVR	1AQ1	2C3K	35.05		Sc=6.40106, min distance = 2.543637
CDK2_HUMAN F	5327123 I	CHK1_HUMAN Fu	444345 1c	1NVR	1AQ1	2C3L	35.05		Sc=6.13712, min distance = 2.143430
CDK2_HUMAN F	5327148 C	IPKA_RABIT Fu	6022 Aden	1JBP	1GY3	2ERZ			Sc=6.05419, min distance = 2.379334
CDK2_HUMAN F	5403427 C	PDK4_HUMAN Fu	6022 Aden	2ZKJ	1GY3	2ZDX			Sc=6.32894, min distance = 1.724960
CDK2_HUMAN F	6082103 2	HSP82_YEAST F	6022 Aden	1AMW	1GY3	2BRC			Sc=6.36522, min distance = 2.073247
CDK2_HUMAN F	6083 ader	PURP_METJA Fu	6022 Aden	2R7N	1GY3	2R7M		0.99	Sc=6.24096, min distance = 2.315111
CDK2_HUMAN F	60961 ade	PIM1_HUMAN Fu	444345 1c	1YHS	1AQ1	1YI4	31.71		Sc=6.1349, min distance = 2.3034118
CDK2_HUMAN F	60961 ade	SKY1_YEAST Fu	6022 Aden	1Q8Y	1GY3	1Q97	30.75	0.95	Sc=6.0831, min distance = 2.9046869
CDK2_HUMAN F	6102787 2	HS90A_HUMAN F	6022 Aden	1BYQ	1GY3	2CCS			Sc=6.2347, min distance = 2.1903858
CDK2_HUMAN F	6540272 C	CHK1_HUMAN Fu	72271 7-H	1NVQ	1PKD	2CGU	35.05		Sc=6.38895, min distance = 2.251940
CDK2_HUMAN F	6540273 2	CHK1_HUMAN Fu	444345 1c	1NVR	1AQ1	2CGV	35.05		Sc=6.15391, min distance = 2.807122
CDK2_HUMAN F	657138 1z	PDPK1_HUMAN F	72271 7-H	1OKZ	1PKD	1Z5M	29.3		Sc=6.63523, min distance = 2.226622
CDK2_HUMAN F	6804 guar	NDK_PYRHO Ful	6022 Aden	2DYA	1GY3	2DXF		0.8	Sc=5.91324, min distance = 2.184601
CDK2_HUMAN F	6852207 M	KAPCA_BOVIN F	444345 1c	1STC	1AQ1	2GNI	34.78		Sc=5.90944, min distance = 2.259765
CDK2_HUMAN F	6914568 C	CHK1_HUMAN Fu	72271 7-H	1NVQ	1PKD	1ZYS	35.05		Sc=6.38166, min distance = 2.310457
CDK2_HUMAN F	6914614 p	IPKA_RABIT Fu	6022 Aden	1JBP	1GY3	2F7Z			Sc=6.35776, min distance = 1.858460
CDK2_HUMAN F	8977 1dar	NDK_THET8 Ful	6022 Aden	1WKL	1GY3	1WKK		0.8	Sc=6.02602, min distance = 2.003270
CDK2_HUMAN F	8977 1dar	RIR1_YEAST Fu	6022 Aden	2CVX	1GY3	2CVW		0.8	Sc=5.87555, min distance = 2.097240
CDK2_HUMAN F	91532 AMF	MTNK_BACSU Fu	6022 Aden	2OLC	1GY3	2PUL		0.99	Sc=5.96884, min distance = 1.905649
CDK2_HUMAN F	9543433 T	NDKC_DICDI Fu	6022 Aden	1KDN	1GY3	1F6T			Sc=6.23033, min distance = 1.374454
CDK5_HUMAN F	1540 1h1q	CDK2_HUMAN Fu	160355 rc	3DDQ	1UNL	1H1Q	60	0.76	Sc=6.43362, min distance = 1.294131
CDK5_HUMAN F	16113377	CDK2_HUMAN Fu	160355 rc	3DDQ	1UNL	2W17	60		Sc=6.53721, min distance = 1.496990
CDK5_HUMAN F	23653515	CDK2_HUMAN Fu	160355 rc	3DDQ	1UNL	2R3F	60		Sc=6.07949, min distance = 2.155800
CDK5_HUMAN F	23653518	CDK2_HUMAN Fu	160355 rc	3DDQ	1UNL	2R3J	60		Sc=6.25253, min distance = 2.079641
CDK5_HUMAN F	23653520	CDK2_HUMAN Fu	160355 rc	3DDQ	1UNL	2R3L	60		Sc=6.2584, min distance = 2.2227660
CDK5_HUMAN F	23653524	CDK2_HUMAN Fu	160355 rc	3DDQ	1UNL	2R3P	60		Sc=6.21614, min distance = 2.460955
CDK5_HUMAN F	24864080	CDK2_HUMAN Fu	160355 rc	3DDQ	1UNL	2VTR	60		Sc=6.00759, min distance = 2.046069
CDK5_HUMAN F	24864081	CDK2_HUMAN Fu	160355 rc	3DDQ	1UNL	2VTS	60		Sc=6.29375, min distance = 2.088904

CDK5_HUMAN F	25021197	CDK2_HUMAN Fu	160355 rc	3DDQ	1UNL	3EOC	60		Sc=6.43052, min distance = 1.283705
CDK5_HUMAN F	4369136 C	CDK2_HUMAN Fu	160355 rc	3DDQ	1UNL	1DM2	60		Sc=6.24611, min distance = 2.200505
CDK5_HUMAN F	445940 O6	CDK2_HUMAN Fu	160355 rc	3DDQ	1UNL	1GZ8	60		Sc=6.03031, min distance = 2.119739
CDK5_HUMAN F	447649 1c	CDK2_HUMAN Fu	160355 rc	3DDQ	1UNL	1OI9	60		Sc=6.4305, min distance = 2.3117584
CDK5_HUMAN F	447652 Im	CDK2_HUMAN Fu	160355 rc	3DDQ	1UNL	1OIQ	60	0.77	Sc=6.23595, min distance = 2.156032
CDK5_HUMAN F	447655 1c	CDK2_HUMAN Fu	160355 rc	3DDQ	1UNL	1OIU	60		Sc=6.3051, min distance = 1.6675221
CDK5_HUMAN F	447656 1c	CDK2_HUMAN Fu	160355 rc	3DDQ	1UNL	1OIY	60		Sc=6.52025, min distance = 1.356105
CDK5_HUMAN F	447956 1p	CDK2_HUMAN Fu	160355 rc	3DDQ	1UNL	2C5O	60		Sc=5.96517, min distance = 1.265246
CDK5_HUMAN F	447962 1p	CDK2_HUMAN Fu	160355 rc	3DDQ	1UNL	2C5N	60		Sc=6.30227, min distance = 1.748197
CDK5_HUMAN F	449087 1v	CDK2_HUMAN Fu	160355 rc	3DDQ	1UNL	1VYW	60		Sc=6.30924, min distance = 1.228464
CDK5_HUMAN F	4565 1h1r	CDK2_HUMAN Fu	160355 rc	3DDQ	1UNL	1H1R	60		Sc=6.34884, min distance = 2.002296
CDK5_HUMAN F	4566 1h1s	CDK2_HUMAN Fu	160355 rc	3DDQ	1UNL	1H1S	60		Sc=6.4219, min distance = 1.6677173
CDK5_HUMAN F	5327096 2	CDK2_HUMAN Fu	160355 rc	3DDQ	1UNL	2BTR	60		Sc=6.122, min distance = 2.12543548
CDK5_HUMAN F	5327130 C	CDK2_HUMAN Fu	160355 rc	3DDQ	1UNL	2C68	60		Sc=6.25618, min distance = 2.499877
CDK5_HUMAN F	5331010 c	CDK2_HUMAN Fu	160355 rc	3DDQ	1UNL	2BKZ	60		Sc=6.33549, min distance = 1.915501
CDK5_HUMAN F	6420138 2	CDK2_HUMAN Fu	160355 rc	3DDQ	1UNL	2UUE	60		Sc=6.42499, min distance = 1.998568
CDK5_HUMAN F	6420139 C	CDK2_HUMAN Fu	160355 rc	3DDQ	1UNL	2C5V	60		Sc=6.27565, min distance = 2.237673
CDK5_HUMAN F	6918710 5	CDK2_HUMAN Fu	160355 rc	3DDQ	1UNL	3EJ1	60		Sc=6.24046, min distance = 2.081447
CDK5_HUMAN F	9549254 2	CDK2_HUMAN Fu	160355 rc	3DDQ	1UNL	2IW6	60		Sc=6.54421, min distance = 2.128148
CDK5_HUMAN F	9817550 V	CDK2_HUMAN Fu	160355 rc	3DDQ	1UNL	3BHV	60		Sc=6.34808, min distance = 2.177300
CDK7_HUMAN F	11957393	FAK1_HUMAN Fu	5957 Aden	2IJM	1UA2	2ETM			Sc=6.34408, min distance = 2.031196
CDK7_HUMAN F	129236 2c	CKI1_SCHPO Fu	5957 Aden	1CSN	1UA2	2CSN			Sc=5.8288, min distance = 1.4363182
CDK7_HUMAN F	16122608	PDPK1_HUMAN F	5957 Aden	2BIY	1UA2	2PE1			Sc=6.35949, min distance = 2.242752
CDK7_HUMAN F	17751819	KAPCA_BOVIN F	5957 Aden	1Q24	1UA2	2VO6			Sc=6.30822, min distance = 1.370723
CDK7_HUMAN F	2396 bisi	PDPK1_HUMAN F	5957 Aden	2BIY	1UA2	1UU8			Sc=6.37624, min distance = 1.856304
CDK7_HUMAN F	2398 bisi	PDPK1_HUMAN F	5957 Aden	2BIY	1UA2	1UU9			Sc=6.41952, min distance = 2.044152
CDK7_HUMAN F	3547 Fasu	KAPCA_BOVIN F	5957 Aden	1Q24	1UA2	1Q8W			Sc=5.92897, min distance = 2.010163
CDK7_HUMAN F	444564 AD	MYS2_DICDI Fu	5957 Aden	1FMW	1UA2	1W9I		0.91	Sc=5.92054, min distance = 0
CDK7_HUMAN F	448042 2e	KAPCA_BOVIN F	5957 Aden	1Q24	1UA2	1Q8T			Sc=5.99448, min distance = 2.174269
CDK7_HUMAN F	449240 1y	KAPCA_BOVIN F	5957 Aden	1Q24	1UA2	1YDR			Sc=5.9329, min distance = 2.0449371
CDK7_HUMAN F	6022 Ader	BIOD_ECOLI Fu	5957 Aden	1A82	1UA2	1DAD		0.99	Sc=6.37067, min distance = 2.188475
CDK7_HUMAN F	6022 Ader	GLGS_SOLTU Fu	5957 Aden	1YP3	1UA2	1YP4		0.99	Sc=5.96229, min distance = 1.939930
CDK7_HUMAN F	6022 Ader	MUTS_ECOLI Fu	5957 Aden	1W7A	1UA2	1OH7		0.99	Sc=5.89581, min distance = 2.198883
CDK7_HUMAN F	6022 Ader	NIFH1_AZOVI F	5957 Aden	2C8V	1UA2	1FP6		0.99	Sc=6.33805, min distance = 2.143597
CDK7_HUMAN F	6022 Ader	PDXK_SHEEP Fu	5957 Aden	1LHR	1UA2	1RFU		0.99	Sc=6.34738, min distance = 1.903227
CDK7_HUMAN F	6022 Ader	PURK_ECOLI Fu	5957 Aden	3ETH	1UA2	3ETJ		0.99	Sc=6.33457, min distance = 2.454176
CDK7_HUMAN F	6022 Ader	Q72H90_THET2	5957 Aden	2BEK	1UA2	2BEJ		0.99	Sc=5.98592, min distance = 2.580859

CDK7_HUMAN F	6022 Aden	Y059_METJA Fu	5957 Aden	2J9C	1UA2	2J9D		0.99	Sc=6.01285, min distance = 1.99767
CDK7_HUMAN F	6083 aden	PURP_PYRFU Fu	5957 Aden	2R86	1UA2	2R85		0.98	Sc=5.93427, min distance = 2.05166
CDK7_HUMAN F	60961 ade	SYQ_ECOLI Ful	5957 Aden	1GTR	1UA2	1O0B		0.94	Sc=6.16533, min distance = 3.05647
CDK7_HUMAN F	6830 guar	Q381M1_9TRYP	5957 Aden	2Q0D	1UA2	2Q0E		0.8	Sc=6.00149, min distance = 2.08110
CDK9_HUMAN F	11708454	KAPCA_BOVIN F	5957 Aden	1Q24	3BLQ	2VNW	27.75		Sc=5.9635, min distance = 2.675920
CDK9_HUMAN F	11957393	FAK1_HUMAN Fu	5957 Aden	2IJM	3BLQ	2ETM	26.11		Sc=6.20589, min distance = 2.04244
CDK9_HUMAN F	6022 Aden	PURK_ECOLI Fu	5957 Aden	3ETH	3BLQ	3ETJ		0.99	Sc=5.73777, min distance = 2.20434
CDK9_HUMAN F	6022 Aden	REX_BACSU Ful	5957 Aden	2VT3	3BLQ	2VT2		0.99	Sc=5.88887, min distance = 2.79385
CDR_STAA8 Fu	16740985	FRDA_SHEFR Fu	444188 CI	1M64	1YQZ	1E39		0.93	Sc=6.42523, min distance = 2.20922
CDR_STAA8 Fu	24757937	LSD1_HUMAN Fu	444188 CI	2DW4	1YQZ	2Z5U			Sc=6.21849, min distance = 2.02958
CDR_STAA8 Fu	444502 CI	FRDA_SHEFR Fu	444188 CI	1M64	1YQZ	1QJD		0.98	Sc=6.4251, min distance = 2.287957
CDR_STAA8 Fu	444502 CI	GSHR_HUMAN Fu	444188 CI	3DK9	1YQZ	1BWC		0.98	Sc=6.42389, min distance = 2.27229
CEA3_ECOLX F	11957399	PYRB_ECOLI Fu	311 citri	1R0B	2B5U	2H3E			Sc=6.07592, min distance = 1.56863
CEA3_ECOLX F	39981 Spa	PYRB_ECOLI Fu	311 citri	1R0B	2B5U	1EKX			Sc=6.07592, min distance = 1.48646
CEA3_ECOLX F	439183 1i	TPIS_TRYBB Fu	311 citri	2VEK	2B5U	1IIH			Sc=5.65858, min distance = 0.88183
CEA3_ECOLX F	447532 1c	SRC_HUMAN Ful	311 citri	1O4L	2B5U	1O4H			Sc=5.95657, min distance = 2.19191
CEA3_ECOLX F	447550 ph	TPIS_TRYBB Fu	311 citri	2VEK	2B5U	4TIM			Sc=5.63574, min distance = 1.31801
CEA5_ECOLX F	101544 2'	RNAS1_BOVIN F	449392 1w	1W4P	2DJH	1ROB		0.8	Sc=6.2851, min distance = 2.485618
CEA5_ECOLX F	5326862 C	RNAS1_BOVIN F	449392 1w	1W4P	2DJH	1W4O		0.84	Sc=6.29236, min distance = 2.45611
CEA5_ECOLX F	656946 CI	RNAS1_BOVIN F	449392 1w	1W4P	2DJH	1W4Q		0.76	Sc=6.25719, min distance = 2.57079
CEA5_ECOLX F	66535 3'-	RNAS1_BOVIN F	449392 1w	1W4P	2DJH	1RPF		0.8	Sc=6.26874, min distance = 2.07929
CED4_CAEEL F	11708454	KAPCA_BOVIN F	5957 Aden	1Q24	2A5Y	2VNW			Sc=6.13144, min distance = 2.30811
CED4_CAEEL F	3547 Fasu	KAPCA_BOVIN F	5957 Aden	1Q24	2A5Y	1Q8W			Sc=5.96713, min distance = 2.44492
CED4_CAEEL F	440317 AT	PURT_ECOLI Fu	5957 Aden	1KJ8	2A5Y	1KJJ		0.99	Sc=6.51026, min distance = 2.08977
CED4_CAEEL F	444564 AD	BIOD_ECOLI Fu	5957 Aden	1A82	2A5Y	1BS1		0.91	Sc=6.44589, min distance = 2.56452
CED4_CAEEL F	444852 CI	ATPA1_BOVIN F	5957 Aden	2V7Q	2A5Y	1COW		0.91	Sc=6.51265, min distance = 1.91824
CED4_CAEEL F	447840 CI	PHKG1_RABIT F	5957 Aden	1QL6	2A5Y	1PHK			Sc=6.50825, min distance = 2.04164
CED4_CAEEL F	449240 1y	KAPCA_BOVIN F	5957 Aden	1Q24	2A5Y	1YDR			Sc=5.97535, min distance = 2.26319
CED4_CAEEL F	6022 Aden	ARSA1_ECOLX F	5957 Aden	1II0	2A5Y	1IHU		0.99	Sc=6.44404, min distance = 2.20394
CED4_CAEEL F	6022 Aden	BIOD_ECOLI Fu	5957 Aden	1A82	2A5Y	1DAD		0.99	Sc=5.9865, min distance = 2.562635
CED4_CAEEL F	6022 Aden	MUTS_ECOLI Fu	5957 Aden	1W7A	2A5Y	1OH7		0.99	Sc=6.45927, min distance = 2.08597
CED4_CAEEL F	6022 Aden	Q72H90_THET2	5957 Aden	2BEK	2A5Y	2BEJ		0.99	Sc=6.44771, min distance = 2.31035
CED4_CAEEL F	6022 Aden	RK_BOVIN Full=	5957 Aden	3C4W	2A5Y	3C4Z		0.99	Sc=6.46916, min distance = 2.24075
CED4_CAEEL F	6022 Aden	Y059_METJA Fu	5957 Aden	2J9C	2A5Y	2J9D		0.99	Sc=6.44981, min distance = 2.34657
CED4_CAEEL F	6083 aden	AAKG1_RAT Ful	5957 Aden	2V92	2A5Y	2V8Q		0.98	Sc=6.37563, min distance = 2.07677
CED4_CAEEL F	6083 aden	Y059_METJA Fu	5957 Aden	2J9C	2A5Y	2J9D		0.98	Sc=6.36984, min distance = 2.34934
CED4_CAEEL F	6852187 2	O57883_PYRHO	5957 Aden	2DTO	2A5Y	2DTH			Sc=6.16682, min distance = 1.44972

CED4_CAEEL F	6852207 M	KAPCA_BOVIN F	5957 Aden	1Q24	2A5Y	2GNI		Sc=5.973, min distance = 2.3310564
CENPE_HUMAN	188966 dA	PAPS1_HUMAN F	6022 Aden	1X6V	1T5C	2PEZ	0.98	Sc=5.91019, min distance = 2.280656
CENPE_HUMAN	447004 CI	MYS2_DICDI Fu	6022 Aden	1VOM	1T5C	1LVK		Sc=5.64868, min distance = 2.435720
CENPE_HUMAN	447955 1p	CDK2_HUMAN Fu	6022 Aden	1GY3	1T5C	1PXI		Sc=5.67796, min distance = 2.019342
CENPE_HUMAN	5957 Aden	RK_BOVIN Full-	6022 Aden	3C4Z	1T5C	3C4W	0.99	Sc=5.80377, min distance = 1.863647
CENPE_HUMAN	91557 1tm	Q94M05_9VIRU	6022 Aden	1W44	1T5C	1W48	0.99	Sc=5.98382, min distance = 1.887327
CFA1_MYCTU F	188380 Ad	MCES_ENCCU Fu	439155 Ad	1RI1	1KPG	1Z3C	0.91	Sc=6.58785, min distance = 2.142961
CFA1_MYCTU F	446535 CI	HNMT_HUMAN Fu	439155 Ad	2AOT	1KPG	1JQE	0.95	Sc=6.55048, min distance = 2.387744
CFA1_MYCTU F	60961 ade	PIMT_PYRFU Fu	439155 Ad	1JG1	1KPG	1JG2	0.84	Sc=6.32013, min distance = 2.256388
CFA2_MYCTU F	188380 Ad	MCES_ENCCU Fu	439155 Ad	1RI1	1KPI	1Z3C	0.91	Sc=6.57047, min distance = 2.064910
CFA2_MYCTU F	446535 CI	HNMT_HUMAN Fu	439155 Ad	2AOT	1KPI	1JQE	0.95	Sc=6.54159, min distance = 2.451339
CFA2_MYCTU F	60961 ade	PIMT_PYRFU Fu	439155 Ad	1JG1	1KPI	1JG2	0.84	Sc=6.26754, min distance = 2.196945
CFTR_HUMAN F	11608401	KAPCA_BOVIN F	5957 Aden	1Q24	2BBS	2UVY		Sc=5.72563, min distance = 2.944110
CFTR_HUMAN F	91532 AMP	INSR_HUMAN Fu	5957 Aden	3BU5	2BBS	1I44	0.99	Sc=5.97194, min distance = 2.111202
CFTR_MOUSE F	11708454	KAPCA_BOVIN F	5957 Aden	1Q24	1XFA	2VNW		Sc=5.81964, min distance = 2.088656
CFTR_MOUSE F	16122607	PDPK1_HUMAN F	5957 Aden	2BIY	1XFA	2PE0		Sc=5.99973, min distance = 2.159028
CFTR_MOUSE F	3547 Fasu	KAPCA_BOVIN F	5957 Aden	1Q24	1XFA	1Q8W		Sc=5.79114, min distance = 1.911071
CFTR_MOUSE F	444564 AD	BIOD_ECOLI Fu	5957 Aden	1A82	1XFA	1BS1	0.91	Sc=5.60247, min distance = 2.048600
CFTR_MOUSE F	444564 AD	MYS2_DICDI Fu	5957 Aden	1FMW	1XFA	1W9I	0.91	Sc=5.6106, min distance = 0
CFTR_MOUSE F	444564 AD	NIFH1_AZOVI F	5957 Aden	2C8V	1XFA	1NIP	0.91	Sc=5.74775, min distance = 2.045200
CFTR_MOUSE F	448043 2g	KAPCA_BOVIN F	5957 Aden	1Q24	1XFA	1Q8U		Sc=5.86491, min distance = 1.796246
CFTR_MOUSE F	449240 1y	KAPCA_BOVIN F	5957 Aden	1Q24	1XFA	1YDR		Sc=5.76001, min distance = 1.985218
CFTR_MOUSE F	6022 Aden	RK_BOVIN Full-	5957 Aden	3C4W	1XFA	3C4Z	0.99	Sc=5.85289, min distance = 2.020693
CFTR_MOUSE F	6083 aden	PURP_METJA Fu	5957 Aden	2R7L	1XFA	2R7M	0.98	Sc=6.1183, min distance = 2.4156866
CFTR_MOUSE F	91532 AMP	BIOD_ECOLI Fu	5957 Aden	1A82	1XFA	1DAG	0.99	Sc=5.68639, min distance = 2.031010
CGGR_BACSU F	1983 acet	PA28_DABRR Fu	439162 gl	1Y38	2OKG	2DPZ		Sc=5.71582, min distance = 1.324816
CH3L1_CAPHI	444863 is	IGHG1_HUMAN F	444205 CI	1HZH	1ZBW	1L6X		Sc=5.8035, min distance = 0.7934758
CH3L1_SHEEP	64689 bet	Q9HYN5_PSEAE	82313 1rd	1W8H	2DSU	1W8F		Sc=5.74975, min distance = 1.841206
CH60_ECOLI F	445940 O6	CDK2_HUMAN Fu	6022 Aden	1GY3	1PCQ	1GZ8		Sc=6.14726, min distance = 2.537635
CH60_ECOLI F	6083 aden	HSP71_HUMAN F	6022 Aden	1S3X	1PCQ	1XQS	0.99	Sc=6.3392, min distance = 2.0217932
CH60_ECOLI F	6083 aden	PSPF_ECOLI Fu	6022 Aden	2C98	1PCQ	2VII	0.99	Sc=6.32965, min distance = 2.218805
CH60_ECOLI F	8977 1dar	PARM_ECOLX Fu	6022 Aden	1MWM	1PCQ	2ZGY	0.8	Sc=6.43972, min distance = 1.742570
CH60_THET2 F	4565 1h1r	CDK2_HUMAN Fu	6022 Aden	1GY3	1WE3	1H1R		Sc=6.39616, min distance = 0.896036
CH60_THET2 F	6083 aden	HSP71_HUMAN F	6022 Aden	1S3X	1WE3	1XQS	0.99	Sc=6.33814, min distance = 2.164753
CH60_THET2 F	6083 aden	PSPF_ECOLI Fu	6022 Aden	2C98	1WE3	2VII	0.99	Sc=6.32378, min distance = 2.260056
CH60_THET2 F	6083 aden	PURP_METJA Fu	6022 Aden	2R7N	1WE3	2R7M	0.99	Sc=6.3392, min distance = 2.0776181
CH60_THET2 F	6804 guar	NDK_PYRHO Ful	6022 Aden	2DYA	1WE3	2DXF	0.8	Sc=6.37312, min distance = 2.299254

CH60_THET2 F	9817550 V	CDK2_HUMAN Fu	6022 Aden	1GY3	1WE3	3BHV			Sc=6.40114, min distance = 1.80642
CHEA_THEMA F	13373715	HS90A_HUMAN F	6022 Aden	1BYQ	1I58	2QF6			Sc=6.22316, min distance = 2.01235
CHEA_THEMA F	15993 dAT	RECA_MYCS2 Fu	6022 Aden	2ZRO	1I58	2ZR9		0.97	Sc=5.69388, min distance = 2.05396
CHEA_THEMA F	16750062	CSK2A_MAIZE F	33113 gam	1LP4	1I59	2OXD			Sc=5.73716, min distance = 2.34466
CHEA_THEMA F	444564 AD	MYS2_DICDI Fu	6022 Aden	1VOM	1I58	1W9I		0.91	Sc=5.97258, min distance = 2.09266
CHEA_THEMA F	444842 CI	CDK2_HUMAN Fu	6022 Aden	1GY3	1I58	1CKP			Sc=5.62862, min distance = 2.09801
CHEA_THEMA F	446090 1h	NDKC_DICDI Fu	6022 Aden	1KDN	1I58	1HIY		0.97	Sc=5.6618, min distance = 1.918656
CHEA_THEMA F	447017 CI	NDKC_DICDI Fu	6022 Aden	1KDN	1I58	1LWX			Sc=5.8787, min distance = 1.743049
CHEA_THEMA F	5326976 1	CSK2A_MAIZE F	33113 gam	1LP4	1I59	1ZOE			Sc=5.74615, min distance = 2.27432
CHEA_THEMA F	5326977 1	CSK2A_MAIZE F	33113 gam	1LP4	1I59	1ZOG			Sc=5.69469, min distance = 2.10786
CHEA_THEMA F	5326978 1	CSK2A_MAIZE F	33113 gam	1LP4	1I59	1ZOH			Sc=5.69905, min distance = 2.46874
CHEA_THEMA F	5327103 2	HS90A_HUMAN F	6022 Aden	1BYQ	1I58	2BYH			Sc=6.50774, min distance = 2.12699
CHEA_THEMA F	65051 ddT	NDKC_DICDI Fu	6022 Aden	1KDN	1I58	1F3F			Sc=5.89581, min distance = 2.27881
CHEA_THEMA F	6804 guar	NDK_PYRHO Full	6022 Aden	2DYA	1I58	2DXF		0.8	Sc=5.61974, min distance = 2.13191
CHER_SALTY F	446535 CI	HNMT_HUMAN Fu	439155 Ad	2AOT	1AF7	1JQE		0.95	Sc=6.52854, min distance = 2.46992
CHER_SALTY F	60961 ade	PIMT_PYRFU Fu	439155 Ad	1JG1	1AF7	1JG2		0.84	Sc=6.29836, min distance = 2.42669
CHER_SALTY F	65482 sir	PRMA_THET8 Fu	439155 Ad	3EGV	1AF7	2ZBR		0.88	Sc=6.57983, min distance = 2.40117
CHI1_COCPO F	119404 Ma	Q54276_SERMA	445246 N-	1E6R	1LL4	1W1Y			Sc=6.21144, min distance = 1.88339
CHIA_SERMA F	119404 Ma	Q54276_SERMA	445246 N-	1E6R	1FFQ	1W1Y	29.36		Sc=6.19684, min distance = 0.33623
CHIA_SERMA F	16058615	LYSC_CHICK Fu	24139 ace	1LZB	1K9T	2H9J			Sc=5.9127, min distance = 2.249900
CHIA_SERMA F	656981 CU	LYSC_CHICK Fu	24139 ace	1LZB	1K9T	1YIL			Sc=6.05565, min distance = 0.88471
CHIA_SERMA F	87901 EIN	Q54276_SERMA	445246 N-	1E6R	1FFQ	1UR8	29.36	0.79	Sc=6.05095, min distance = 2.39784
CHIT1_HUMAN	119404 Ma	Q54276_SERMA	24139 ace	1UR9	1HKI	1W1Y	29.52		Sc=6.19988, min distance = 2.03722
CHIT1_HUMAN	119404 Ma	Q54276_SERMA	445246 N-	1E6R	1HKK	1W1Y	29.52		Sc=6.23938, min distance = 1.00234
CHIT1_HUMAN	126154 Cy	Q54276_SERMA	445247 CI	1E6R	1HKK	1W1P	29.52		Sc=5.74491, min distance = 2.15187
CHIT1_HUMAN	444162 CI	Q54276_SERMA	445247 CI	1E6R	1HKK	1UR9	29.52		Sc=5.88395, min distance = 2.40201
CHIT1_HUMAN	445256 NG	Q54276_SERMA	445247 CI	1E6R	1HKK	1E6Z	29.52		Sc=5.79199, min distance = 2.34191
CHIT1_HUMAN	449094 Cy	Q54276_SERMA	445247 CI	1E6R	1HKK	1W1T	29.52		Sc=6.19686, min distance = 2.05750
CHIT1_HUMAN	4740 pent	Q873X9_ASPFU	445247 CI	2A3E	1HKK	2A3C	32.58		Sc=6.1868, min distance = 1.514142
CHIT_CARPA F	185698 al	MBL1_RAT Full	82313 1rd	1KWV	3CQL	2MSB			Sc=5.65103, min distance = 2.04114
CHIT_CARPA F	185698 al	MBL2_RAT Full	24139 ace	1KZD	3CQL	1RDL			Sc=5.80653, min distance = 1.64500
CHIT_CARPA F	185698 al	MBL2_RAT Full	82313 1rd	1RDN	3CQL	1RDL			Sc=5.75342, min distance = 1.77263
CHIT_CARPA F	24310 fru	Q9HYN5_PSEAE	82313 1rd	1W8H	3CQL	1OVP			Sc=5.72864, min distance = 2.46564
CHIT_CARPA F	24779679	AGI1_WHEAT Fu	24139 ace	2UVO	3CQL	2UWG		0.92	Sc=6.52666, min distance = 0.77240
CHIT_CARPA F	439353 be	AGI3_WHEAT Fu	24139 ace	1K7U	3CQL	1K7V			Sc=5.73207, min distance = 1.84097
CHIT_CARPA F	439353 be	LYSC_CHICK Fu	24139 ace	1LZB	3CQL	1UC0			Sc=5.66077, min distance = 2.57757
CHIT_CARPA F	439353 be	MBL2_RAT Full	24139 ace	1KZD	3CQL	1RDK			Sc=5.72108, min distance = 2.62126

CHIT_CARPA F	439353 be	Q6PYX1_HUMAN	24139 ace	1CLY	3CQL	1S3K			Sc=5.62522, min distance = 1.92617
CHIT_CARPA F	439554 fu	Q9HYN5_PSEAE	82313 1rd	1W8H	3CQL	1UZV			Sc=5.673, min distance = 2.4325071
CHIT_CARPA F	439583 L-	Q9HYN5_PSEAE	82313 1rd	1W8H	3CQL	2BP6			Sc=5.79098, min distance = 2.05977
CHIT_CARPA F	444205 CI	DPP4_HUMAN Fu	24139 ace	2RGU	3CQL	3BJM		0.91	Sc=6.03837, min distance = 2.08335
CHIT_CARPA F	444863 is	MBL2_RAT Full	24139 ace	1KZD	3CQL	1RDJ			Sc=5.64347, min distance = 2.38764
CHIT_CARPA F	445247 CI	CHLY_HEVBR Fu	24139 ace	1KQY	3CQL	1LLO			Sc=6.00447, min distance = 1.62934
CHIT_CARPA F	445948 D-	Q9HYN5_PSEAE	82313 1rd	1W8H	3CQL	1OXC			Sc=5.66805, min distance = 2.53752
CHIT_CARPA F	446101 AC	CSLA_PEDHE Fu	24139 ace	1HM3	3CQL	1HM2		0.94	Sc=5.93349, min distance = 2.79644
CHIT_CARPA F	446578 1r	MBL2_RAT Full	24139 ace	1KZD	3CQL	1RDI			Sc=5.68594, min distance = 1.73346
CHIT_CARPA F	446578 1r	Q9HYN5_PSEAE	82313 1rd	1W8H	3CQL	2JDP			Sc=5.64022, min distance = 2.29313
CHIT_CARPA F	6134 lact	GGTA1_BOVIN F	24139 ace	1O7Q	3CQL	1O7O			Sc=6.38575, min distance = 1.15943
CHIT_CARPA F	64689 bet	UGL_BACGL Ful	82313 1rd	2FV1	3CQL	2FV0			Sc=5.77455, min distance = 1.25033
CHIT_CARPA F	79025 alp	LEC_ERYCO Ful	82313 1rd	1AX2	3CQL	1FYU			Sc=5.71845, min distance = 2.38145
CHK1_HUMAN F	10224714	CDK2_HUMAN Fu	444345 1c	1AQ1	1NVR	3DDP	35.05		Sc=6.44119, min distance = 2.14347
CHK1_HUMAN F	10224714	CDK2_HUMAN Fu	72271 7-H	1PKD	1NVQ	3DDP	35.05		Sc=6.42523, min distance = 2.77820
CHK1_HUMAN F	11314340	KAPCA_BOVIN F	444345 1c	1STC	1NVR	2UZT	33.01		Sc=6.32659, min distance = 2.16857
CHK1_HUMAN F	11553058	LCK_HUMAN Ful	444345 1c	1QPD	1NVR	2OF2	30.2		Sc=6.28965, min distance = 2.71949
CHK1_HUMAN F	11957417	CDK2_HUMAN Fu	444345 1c	1AQ1	1NVR	2I40	35.05		Sc=6.28114, min distance = 2.07533
CHK1_HUMAN F	1540 1h1q	CDK2_HUMAN Fu	444345 1c	1AQ1	1NVR	1H1Q	35.05		Sc=6.22886, min distance = 2.57382
CHK1_HUMAN F	15602982	KAPCA_BOVIN F	444345 1c	1STC	1NVR	2UW6	33.01		Sc=6.03283, min distance = 2.61434
CHK1_HUMAN F	15991572	LCK_HUMAN Ful	444345 1c	1QPD	1NVR	2OF4	30.2		Sc=6.14337, min distance = 2.50896
CHK1_HUMAN F	160355 rc	CDK2_HUMAN Fu	444345 1c	1AQ1	1NVR	3DDQ	35.05		Sc=6.14854, min distance = 2.60661
CHK1_HUMAN F	160355 rc	CDK2_HUMAN Fu	72271 7-H	1PKD	1NVQ	3DDQ	35.05		Sc=6.33169, min distance = 2.70585
CHK1_HUMAN F	16046126	CDK2_HUMAN Fu	444345 1c	1AQ1	1NVR	2DS1	35.05		Sc=6.23237, min distance = 2.81196
CHK1_HUMAN F	16122608	PDPK1_HUMAN F	444345 1c	1OKY	1NVR	2PE1	33.65		Sc=6.32978, min distance = 2.72263
CHK1_HUMAN F	16122608	PDPK1_HUMAN F	72271 7-H	1OKZ	1NVQ	2PE1	33.65		Sc=6.3266, min distance = 2.721494
CHK1_HUMAN F	16214823	CDK2_HUMAN Fu	72271 7-H	1PKD	1NVQ	2UZB	35.05		Sc=6.10149, min distance = 1.78223
CHK1_HUMAN F	16214825	CDK2_HUMAN Fu	444345 1c	1AQ1	1NVR	2UZE	35.05		Sc=6.22439, min distance = 2.94772
CHK1_HUMAN F	16214825	CDK2_HUMAN Fu	72271 7-H	1PKD	1NVQ	2UZE	35.05		Sc=6.27284, min distance = 2.59696
CHK1_HUMAN F	16214826	CDK2_HUMAN Fu	444345 1c	1AQ1	1NVR	2UZL	35.05		Sc=6.11192, min distance = 2.43965
CHK1_HUMAN F	16214826	CDK2_HUMAN Fu	72271 7-H	1PKD	1NVQ	2UZL	35.05		Sc=6.13282, min distance = 2.26434
CHK1_HUMAN F	16214827	CDK2_HUMAN Fu	72271 7-H	1PKD	1NVQ	2UZN	35.05		Sc=6.10482, min distance = 2.35213
CHK1_HUMAN F	16214828	CDK2_HUMAN Fu	72271 7-H	1PKD	1NVQ	2UZO	35.05		Sc=6.09758, min distance = 1.91159
CHK1_HUMAN F	1707 1fvt	CDK2_HUMAN Fu	444345 1c	1AQ1	1NVR	1FVT	35.05		Sc=6.07837, min distance = 2.09170
CHK1_HUMAN F	1707 1fvt	CDK2_HUMAN Fu	72271 7-H	1PKD	1NVQ	1FVT	35.05		Sc=6.09956, min distance = 2.11085
CHK1_HUMAN F	17754396	PDPK1_HUMAN F	444345 1c	1OKY	1NVR	2R7B	33.65		Sc=6.27762, min distance = 2.71560
CHK1_HUMAN F	17754396	PDPK1_HUMAN F	72271 7-H	1OKZ	1NVQ	2R7B	33.65		Sc=6.28421, min distance = 2.86024

CHK1_HUMAN F	23536770	LCK_HUMAN Ful	444345 1c	1QPD	1NVR	3BYM	30.2	Sc=6.31744, min distance = 2.34807
CHK1_HUMAN F	23653515	CDK2_HUMAN Fu	444345 1c	1AQ1	1NVR	2R3F	35.05	Sc=5.99006, min distance = 2.54818
CHK1_HUMAN F	23653515	CDK2_HUMAN Fu	72271 7-H	1PKD	1NVQ	2R3F	35.05	Sc=6.00052, min distance = 2.30273
CHK1_HUMAN F	23653516	CDK2_HUMAN Fu	72271 7-H	1PKD	1NVQ	2R3G	35.05	Sc=6.1234, min distance = 2.100081
CHK1_HUMAN F	23653519	CDK2_HUMAN Fu	444345 1c	1AQ1	1NVR	2R3K	35.05	Sc=6.0678, min distance = 2.355199
CHK1_HUMAN F	23653519	CDK2_HUMAN Fu	72271 7-H	1PKD	1NVQ	2R3K	35.05	Sc=6.20786, min distance = 2.09432
CHK1_HUMAN F	23653520	CDK2_HUMAN Fu	72271 7-H	1PKD	1NVQ	2R3L	35.05	Sc=6.20376, min distance = 2.25485
CHK1_HUMAN F	23653521	CDK2_HUMAN Fu	444345 1c	1AQ1	1NVR	2R3M	35.05	Sc=6.32804, min distance = 2.53190
CHK1_HUMAN F	23653524	CDK2_HUMAN Fu	444345 1c	1AQ1	1NVR	2R3P	35.05	Sc=6.23123, min distance = 2.50211
CHK1_HUMAN F	23727982	CDK2_HUMAN Fu	444345 1c	1AQ1	1NVR	3BHU	35.05	Sc=6.20589, min distance = 2.73117
CHK1_HUMAN F	23727982	CDK2_HUMAN Fu	72271 7-H	1PKD	1NVQ	3BHU	35.05	Sc=6.23711, min distance = 2.61394
CHK1_HUMAN F	2396 bisi	PDPK1_HUMAN F	72271 7-H	1OKZ	1NVQ	1UU8	33.65	Sc=6.38656, min distance = 2.16343
CHK1_HUMAN F	2398 bisi	PDPK1_HUMAN F	444345 1c	1OKY	1NVR	1UU9	33.65	Sc=6.43611, min distance = 2.16322
CHK1_HUMAN F	2398 bisi	PDPK1_HUMAN F	72271 7-H	1OKZ	1NVQ	1UU9	33.65	Sc=6.43611, min distance = 2.37637
CHK1_HUMAN F	2403 1uvr	PDPK1_HUMAN F	444345 1c	1OKY	1NVR	1UVR	33.65	Sc=6.37213, min distance = 2.18586
CHK1_HUMAN F	24901723	CDK2_HUMAN Fu	444345 1c	1AQ1	1NVR	2W06	35.05	Sc=6.16565, min distance = 2.21648
CHK1_HUMAN F	24901723	CDK2_HUMAN Fu	72271 7-H	1PKD	1NVQ	2W06	35.05	Sc=6.2963, min distance = 2.012465
CHK1_HUMAN F	24905143	PK3CG_HUMAN F	444345 1c	1E8Z	1NVR	2V4L		Sc=5.99448, min distance = 3.01453
CHK1_HUMAN F	24905144	PK3CG_HUMAN F	444345 1c	1E8Z	1NVR	3ENE		Sc=6.07124, min distance = 2.76676
CHK1_HUMAN F	24916751	CDK2_HUMAN Fu	444345 1c	1AQ1	1NVR	3DOG	35.05	Sc=6.23784, min distance = 2.17653
CHK1_HUMAN F	24916751	CDK2_HUMAN Fu	72271 7-H	1PKD	1NVQ	3DOG	35.05	Sc=6.33169, min distance = 2.71163
CHK1_HUMAN F	24978497	CDK2_HUMAN Fu	72271 7-H	1PKD	1NVQ	3EID	35.05	Sc=6.54108, min distance = 2.04075
CHK1_HUMAN F	25021197	CDK2_HUMAN Fu	72271 7-H	1PKD	1NVQ	3EOC	35.05	Sc=6.21836, min distance = 2.70200
CHK1_HUMAN F	33113 gam	IRAK4_HUMAN F	444345 1c	2NRY	1NVR	2OID	36.48	Sc=5.6944, min distance = 2.189665
CHK1_HUMAN F	3973 nche	PIM1_HUMAN Fu	444345 1c	1YHS	1NVR	1YI3	34.71	Sc=5.84143, min distance = 2.38444
CHK1_HUMAN F	4369136 C	CDK2_HUMAN Fu	444345 1c	1AQ1	1NVR	1DM2	35.05	Sc=6.2247, min distance = 2.724031
CHK1_HUMAN F	4369136 C	CDK2_HUMAN Fu	72271 7-H	1PKD	1NVQ	1DM2	35.05	Sc=6.23709, min distance = 2.67654
CHK1_HUMAN F	446704 LS	CDK2_HUMAN Fu	444345 1c	1AQ1	1NVR	1KE6	35.05	Sc=6.24978, min distance = 2.33529
CHK1_HUMAN F	446704 LS	CDK2_HUMAN Fu	72271 7-H	1PKD	1NVQ	1KE6	35.05	Sc=6.26559, min distance = 1.90228
CHK1_HUMAN F	447649 1c	CDK2_HUMAN Fu	444345 1c	1AQ1	1NVR	1OI9	35.05	Sc=6.31532, min distance = 2.68582
CHK1_HUMAN F	447649 1c	CDK2_HUMAN Fu	72271 7-H	1PKD	1NVQ	1OI9	35.05	Sc=6.34131, min distance = 2.40713
CHK1_HUMAN F	447654 CI	CDK2_HUMAN Fu	444345 1c	1AQ1	1NVR	1OIT	35.05	Sc=6.13252, min distance = 2.16716
CHK1_HUMAN F	447655 1c	CDK2_HUMAN Fu	444345 1c	1AQ1	1NVR	1OIU	35.05	Sc=6.14644, min distance = 2.67073
CHK1_HUMAN F	447656 1c	CDK2_HUMAN Fu	444345 1c	1AQ1	1NVR	1OIY	35.05	Sc=6.33723, min distance = 2.67210
CHK1_HUMAN F	447656 1c	CDK2_HUMAN Fu	72271 7-H	1PKD	1NVQ	1OIY	35.05	Sc=6.42911, min distance = 2.41516
CHK1_HUMAN F	447962 1p	CDK2_HUMAN Fu	444345 1c	1AQ1	1NVR	2C5N	35.05	Sc=6.10381, min distance = 2.57363
CHK1_HUMAN F	456214 pu	KS6A1_HUMAN F	444345 1c	2Z7R	1NVR	2Z7S		Sc=6.27663, min distance = 2.27196

CHK1_HUMAN Fı 4565 1h1r	CDK2_HUMAN Fu 444345 1c 1AQ1	1NVR	1H1R	35.05	Sc=6.22641, min distance = 2.785625
CHK1_HUMAN Fı 4565 1h1r	CDK2_HUMAN Fu 72271 7-H 1PKD	1NVQ	1H1R	35.05	Sc=6.17832, min distance = 0.659309
CHK1_HUMAN Fı 4566 1h1s	CDK2_HUMAN Fu 444345 1c 1AQ1	1NVR	1H1S	35.05	Sc=6.27123, min distance = 2.252419
CHK1_HUMAN Fı 5288016 6	CDK2_HUMAN Fu 444345 1c 1AQ1	1NVR	2B53	35.05	Sc=6.00445, min distance = 2.362743
CHK1_HUMAN Fı 5288016 6	CDK2_HUMAN Fu 72271 7-H 1PKD	1NVQ	2B53	35.05	Sc=6.15711, min distance = 2.227556
CHK1_HUMAN Fı 5288708 1	CDK2_HUMAN Fu 444345 1c 1AQ1	1NVR	1KE5	35.05	Sc=6.10146, min distance = 2.321570
CHK1_HUMAN Fı 5288708 1	CDK2_HUMAN Fu 72271 7-H 1PKD	1NVQ	1KE5	35.05	Sc=6.10146, min distance = 2.490491
CHK1_HUMAN Fı 5288710 C	CDK2_HUMAN Fu 444345 1c 1AQ1	1NVR	1KE7	35.05	Sc=6.31611, min distance = 2.183215
CHK1_HUMAN Fı 5288710 C	CDK2_HUMAN Fu 72271 7-H 1PKD	1NVQ	1KE7	35.05	Sc=6.3387, min distance = 1.7650351
CHK1_HUMAN Fı 5288711 1	CDK2_HUMAN Fu 444345 1c 1AQ1	1NVR	1KE8	35.05	Sc=6.25032, min distance = 2.626485
CHK1_HUMAN Fı 5327130 C	CDK2_HUMAN Fu 444345 1c 1AQ1	1NVR	2C68	35.05	Sc=6.18089, min distance = 2.458398
CHK1_HUMAN Fı 5327131 T	CDK2_HUMAN Fu 444345 1c 1AQ1	1NVR	2C69	35.05	Sc=6.17773, min distance = 2.537223
CHK1_HUMAN Fı 5327132 C	CDK2_HUMAN Fu 444345 1c 1AQ1	1NVR	2C6I	35.05	Sc=6.26795, min distance = 2.156527
CHK1_HUMAN Fı 5327133 C	CDK2_HUMAN Fu 72271 7-H 1PKD	1NVQ	2C6K	35.05	Sc=6.2375, min distance = 2.2392291
CHK1_HUMAN Fı 5331010 c	CDK2_HUMAN Fu 444345 1c 1AQ1	1NVR	2BKZ	35.05	Sc=6.18566, min distance = 2.400710
CHK1_HUMAN Fı 5494414 C	CDK2_HUMAN Fu 444345 1c 1AQ1	1NVR	2A0C	35.05	Sc=6.39566, min distance = 2.661687
CHK1_HUMAN Fı 5687 1di8	CDK2_HUMAN Fu 444345 1c 1AQ1	1NVR	1DI8	35.05	Sc=6.21962, min distance = 2.399153
CHK1_HUMAN Fı 6083 aden	PIM1_HUMAN Fu 444345 1c 1YHS	1NVR	1YXU	34.71	Sc=6.28743, min distance = 2.219856
CHK1_HUMAN Fı 60961 ade	PIM1_HUMAN Fu 444345 1c 1YHS	1NVR	1YI4	34.71	Sc=6.17584, min distance = 2.447951
CHK1_HUMAN Fı 6102670	CDK2_HUMAN Fu 444345 1c 1AQ1	1NVR	1YKR	35.05	Sc=6.24881, min distance = 2.013764
CHK1_HUMAN Fı 6420138 2	CDK2_HUMAN Fu 444345 1c 1AQ1	1NVR	2UUE	35.05	Sc=6.23906, min distance = 2.307584
CHK1_HUMAN Fı 6420138 2	CDK2_HUMAN Fu 72271 7-H 1PKD	1NVQ	2UUE	35.05	Sc=6.3479, min distance = 2.2463452
CHK1_HUMAN Fı 6420139 C	CDK2_HUMAN Fu 444345 1c 1AQ1	1NVR	2C5V	35.05	Sc=6.10665, min distance = 2.140925
CHK1_HUMAN Fı 6420140 2	CDK2_HUMAN Fu 72271 7-H 1PKD	1NVQ	2C5Y	35.05	Sc=6.16594, min distance = 2.262937
CHK1_HUMAN Fı 6539118 g	CDK2_HUMAN Fu 444345 1c 1AQ1	1NVR	1FVV	35.05	Sc=6.32994, min distance = 2.192153
CHK1_HUMAN Fı 6539118 g	CDK2_HUMAN Fu 72271 7-H 1PKD	1NVQ	1FVV	35.05	Sc=6.42693, min distance = 2.312696
CHK1_HUMAN Fı 656971 CI	CDK2_HUMAN Fu 444345 1c 1AQ1	1NVR	1Y8Y	35.05	Sc=6.01625, min distance = 2.189690
CHK1_HUMAN Fı 656971 CI	CDK2_HUMAN Fu 72271 7-H 1PKD	1NVQ	1Y8Y	35.05	Sc=5.99508, min distance = 2.648876
CHK1_HUMAN Fı 657072 RY	CDK2_HUMAN Fu 444345 1c 1AQ1	1NVR	2BHH	35.05	Sc=6.41837, min distance = 2.032837
CHK1_HUMAN Fı 657072 RY	CDK2_HUMAN Fu 72271 7-H 1PKD	1NVQ	2BHH	35.05	Sc=6.42423, min distance = 2.110401
CHK1_HUMAN Fı 657138 1z	PDPK1_HUMAN Fı 444345 1c 1OKY	1NVR	1Z5M	33.65	Sc=6.63931, min distance = 2.043125
CHK1_HUMAN Fı 6852207 M	KAPCA_BOVIN Fı 444345 1c 1STC	1NVR	2GNI	33.01	Sc=5.91095, min distance = 2.525599
CHK1_HUMAN Fı 6918852 2	CDK2_HUMAN Fu 72271 7-H 1PKD	1NVQ	2FVD	35.05	Sc=6.34286, min distance = 1.999824
CHK1_HUMAN Fı 9547890 I	CDK2_HUMAN Fu 444345 1c 1AQ1	1NVR	1W8C	35.05	Sc=6.18464, min distance = 2.679161
CHK1_HUMAN Fı 9549254 2	CDK2_HUMAN Fu 444345 1c 1AQ1	1NVR	2IW6	35.05	Sc=6.43537, min distance = 2.020962
CHK1_HUMAN Fı 9817550 V	CDK2_HUMAN Fu 444345 1c 1AQ1	1NVR	3BHV	35.05	Sc=6.31821, min distance = 2.415825
CHK1_HUMAN Fı 9817550 V	CDK2_HUMAN Fu 72271 7-H 1PKD	1NVQ	3BHV	35.05	Sc=6.33904, min distance = 2.264583

CHK2_HUMAN Fu	10138993	CDK2_HUMAN Fu	6022 Aden	1GY3	2CN5	2R64	30.8		Sc=6.37774, min distance = 2.570491
CHK2_HUMAN Fu	10224714	CDK2_HUMAN Fu	6022 Aden	1GY3	2CN5	3DDP	30.8		Sc=6.53744, min distance = 1.627269
CHK2_HUMAN Fu	11268094	CDK2_HUMAN Fu	6022 Aden	1GY3	2CN5	2VTQ	30.8		Sc=6.41451, min distance = 1.828072
CHK2_HUMAN Fu	11957393	FAK1_HUMAN Fu	6022 Aden	1MP8	2CN5	2ETM	29.41		Sc=6.32423, min distance = 2.362513
CHK2_HUMAN Fu	11957417	CDK2_HUMAN Fu	6022 Aden	1GY3	2CN5	2I40	30.8		Sc=6.3064, min distance = 2.1104184
CHK2_HUMAN Fu	1540 1h1q	CDK2_HUMAN Fu	6022 Aden	1GY3	2CN5	1H1Q	30.8		Sc=6.40465, min distance = 1.784427
CHK2_HUMAN Fu	15942652	CDK2_HUMAN Fu	6022 Aden	1GY3	2CN5	2DUV	30.8		Sc=6.33088, min distance = 2.333291
CHK2_HUMAN Fu	160355 rc	CDK2_HUMAN Fu	6022 Aden	1GY3	2CN5	3DDQ	30.8		Sc=6.4811, min distance = 1.0512592
CHK2_HUMAN Fu	16214823	CDK2_HUMAN Fu	6022 Aden	1GY3	2CN5	2UZB	30.8		Sc=6.14831, min distance = 1.823017
CHK2_HUMAN Fu	16214825	CDK2_HUMAN Fu	6022 Aden	1GY3	2CN5	2UZE	30.8		Sc=6.28301, min distance = 2.518879
CHK2_HUMAN Fu	16214826	CDK2_HUMAN Fu	6022 Aden	1GY3	2CN5	2UZL	30.8		Sc=6.15474, min distance = 2.440815
CHK2_HUMAN Fu	16758212	GSK3B_HUMAN Fu	6022 Aden	1J1C	2CN5	2O5K	30.82		Sc=6.43006, min distance = 2.230163
CHK2_HUMAN Fu	23653515	CDK2_HUMAN Fu	6022 Aden	1GY3	2CN5	2R3F	30.8		Sc=6.03501, min distance = 2.402357
CHK2_HUMAN Fu	23653518	CDK2_HUMAN Fu	6022 Aden	1GY3	2CN5	2R3J	30.8		Sc=6.25968, min distance = 2.032335
CHK2_HUMAN Fu	23653519	CDK2_HUMAN Fu	6022 Aden	1GY3	2CN5	2R3K	30.8		Sc=6.26271, min distance = 1.874474
CHK2_HUMAN Fu	23653520	CDK2_HUMAN Fu	6022 Aden	1GY3	2CN5	2R3L	30.8		Sc=6.25497, min distance = 2.042985
CHK2_HUMAN Fu	23653521	CDK2_HUMAN Fu	6022 Aden	1GY3	2CN5	2R3M	30.8		Sc=6.44943, min distance = 2.351728
CHK2_HUMAN Fu	23653524	CDK2_HUMAN Fu	6022 Aden	1GY3	2CN5	2R3P	30.8		Sc=6.32019, min distance = 2.076026
CHK2_HUMAN Fu	23727981	CDK2_HUMAN Fu	6022 Aden	1GY3	2CN5	3BHT	30.8		Sc=6.21094, min distance = 1.824935
CHK2_HUMAN Fu	23727982	CDK2_HUMAN Fu	6022 Aden	1GY3	2CN5	3BHU	30.8		Sc=6.24206, min distance = 2.844249
CHK2_HUMAN Fu	24864080	CDK2_HUMAN Fu	6022 Aden	1GY3	2CN5	2VTR	30.8		Sc=5.98824, min distance = 1.940519
CHK2_HUMAN Fu	24864081	CDK2_HUMAN Fu	6022 Aden	1GY3	2CN5	2VTS	30.8		Sc=6.3161, min distance = 2.1929908
CHK2_HUMAN Fu	24864082	CDK2_HUMAN Fu	6022 Aden	1GY3	2CN5	2VTT	30.8		Sc=6.41218, min distance = 2.506957
CHK2_HUMAN Fu	24901723	CDK2_HUMAN Fu	6022 Aden	1GY3	2CN5	2W06	30.8		Sc=6.29307, min distance = 2.059900
CHK2_HUMAN Fu	25021197	CDK2_HUMAN Fu	6022 Aden	1GY3	2CN5	3EOC	30.8		Sc=6.36731, min distance = 1.664996
CHK2_HUMAN Fu	2608 1jsv	CDK2_HUMAN Fu	6022 Aden	1GY3	2CN5	1JSV	30.8		Sc=5.92801, min distance = 2.203494
CHK2_HUMAN Fu	3543 1g5s	CDK2_HUMAN Fu	6022 Aden	1GY3	2CN5	1G5S	30.8		Sc=6.47008, min distance = 2.592900
CHK2_HUMAN Fu	447649 1c	CDK2_HUMAN Fu	6022 Aden	1GY3	2CN5	1OI9	30.8		Sc=6.44418, min distance = 2.102577
CHK2_HUMAN Fu	447652 Im	CDK2_HUMAN Fu	6022 Aden	1GY3	2CN5	1OIQ	30.8		Sc=6.13841, min distance = 2.678571
CHK2_HUMAN Fu	447654 CI	CDK2_HUMAN Fu	6022 Aden	1GY3	2CN5	1OIT	30.8		Sc=6.17714, min distance = 2.223256
CHK2_HUMAN Fu	447655 1c	CDK2_HUMAN Fu	6022 Aden	1GY3	2CN5	1OIU	30.8		Sc=6.41977, min distance = 1.591941
CHK2_HUMAN Fu	447656 1c	CDK2_HUMAN Fu	6022 Aden	1GY3	2CN5	1OIY	30.8		Sc=6.49539, min distance = 2.137588
CHK2_HUMAN Fu	447766 1p	CDK2_HUMAN Fu	6022 Aden	1GY3	2CN5	1P2A	30.8		Sc=6.31511, min distance = 2.044871
CHK2_HUMAN Fu	447821 CI	CDK2_HUMAN Fu	6022 Aden	1GY3	2CN5	1PF8	30.8		Sc=6.10815, min distance = 2.231269
CHK2_HUMAN Fu	447916 ac	RIO1_ARCFU Fu	6022 Aden	1ZTH	2CN5	1ZTF		0.95	Sc=6.20117, min distance = 2.316784
CHK2_HUMAN Fu	447955 1p	CDK2_HUMAN Fu	6022 Aden	1GY3	2CN5	1PXI	30.8		Sc=5.62063, min distance = 2.222419
CHK2_HUMAN Fu	447962 1p	CDK2_HUMAN Fu	6022 Aden	1GY3	2CN5	2C5N	30.8		Sc=6.2584, min distance = 1.2955967

CHK2_HUMAN F	447967 CI	CDK2_HUMAN Fu	6022 Aden	1GY3	2CN5	1PYE	30.8		Sc=6.36731, min distance = 1.82887
CHK2_HUMAN F	449087 1v	CDK2_HUMAN Fu	6022 Aden	1GY3	2CN5	1VYW	30.8		Sc=6.27842, min distance = 1.06332
CHK2_HUMAN F	449088 1v	CDK2_HUMAN Fu	6022 Aden	1GY3	2CN5	1VYZ	30.8		Sc=6.08463, min distance = 2.30181
CHK2_HUMAN F	4565 1h1r	CDK2_HUMAN Fu	6022 Aden	1GY3	2CN5	1H1R	30.8		Sc=6.3479, min distance = 2.121745
CHK2_HUMAN F	4566 1h1s	CDK2_HUMAN Fu	6022 Aden	1GY3	2CN5	1H1S	30.8		Sc=6.41168, min distance = 2.00026
CHK2_HUMAN F	5287844 i	GSK3B_HUMAN F	6022 Aden	1J1C	2CN5	1UV5	30.82		Sc=6.31072, min distance = 2.09515
CHK2_HUMAN F	5287845 N	CDK2_HUMAN Fu	6022 Aden	1GY3	2CN5	2BHE	30.8		Sc=6.20208, min distance = 2.48011
CHK2_HUMAN F	5288018 C	CDK2_HUMAN Fu	6022 Aden	1GY3	2CN5	2B52	30.8		Sc=6.56634, min distance = 2.22163
CHK2_HUMAN F	5288708 1	CDK2_HUMAN Fu	6022 Aden	1GY3	2CN5	1KE5	30.8		Sc=6.1181, min distance = 2.091532
CHK2_HUMAN F	5288710 C	CDK2_HUMAN Fu	6022 Aden	1GY3	2CN5	1KE7	30.8		Sc=6.31804, min distance = 2.43810
CHK2_HUMAN F	5288711 1	CDK2_HUMAN Fu	6022 Aden	1GY3	2CN5	1KE8	30.8		Sc=6.25968, min distance = 2.45968
CHK2_HUMAN F	5288712 1	CDK2_HUMAN Fu	6022 Aden	1GY3	2CN5	1KE9	30.8		Sc=6.28487, min distance = 2.56400
CHK2_HUMAN F	5327096 2	CDK2_HUMAN Fu	6022 Aden	1GY3	2CN5	2BTR	30.8		Sc=6.08121, min distance = 2.61621
CHK2_HUMAN F	5327097 2	CDK2_HUMAN Fu	6022 Aden	1GY3	2CN5	2BTS	30.8		Sc=5.89334, min distance = 2.37155
CHK2_HUMAN F	5327124	CDK2_HUMAN Fu	6022 Aden	1GY3	2CN5	2C4G	30.8		Sc=6.22933, min distance = 2.70823
CHK2_HUMAN F	5327132 C	CDK2_HUMAN Fu	6022 Aden	1GY3	2CN5	2C6I	30.8		Sc=6.30673, min distance = 2.24939
CHK2_HUMAN F	5327133 C	CDK2_HUMAN Fu	6022 Aden	1GY3	2CN5	2C6K	30.8		Sc=6.28164, min distance = 2.30261
CHK2_HUMAN F	5327134 C	CDK2_HUMAN Fu	6022 Aden	1GY3	2CN5	2C6L	30.8		Sc=6.37671, min distance = 2.16561
CHK2_HUMAN F	5327135 D	CDK2_HUMAN Fu	6022 Aden	1GY3	2CN5	2C6M	30.8		Sc=6.28467, min distance = 1.85300
CHK2_HUMAN F	5327148 C	IPKA_RABIT Fu	6022 Aden	1JBP	2CN5	2ERZ			Sc=6.16325, min distance = 2.57018
CHK2_HUMAN F	5331010 c	CDK2_HUMAN Fu	6022 Aden	1GY3	2CN5	2BKZ	30.8		Sc=6.31548, min distance = 1.65962
CHK2_HUMAN F	5494414 C	CDK2_HUMAN Fu	6022 Aden	1GY3	2CN5	2A0C	30.8		Sc=6.43486, min distance = 2.07079
CHK2_HUMAN F	5957 Aden	RK_BOVIN Full	6022 Aden	3C4Z	2CN5	3C4W		0.99	Sc=6.46037, min distance = 2.38148
CHK2_HUMAN F	60961 ade	IPKA_RABIT Fu	6022 Aden	1JBP	2CN5	1FMO		0.95	Sc=6.20579, min distance = 2.37477
CHK2_HUMAN F	6102670	CDK2_HUMAN Fu	6022 Aden	1GY3	2CN5	1YKR	30.8		Sc=6.26662, min distance = 2.05625
CHK2_HUMAN F	6420138 2	CDK2_HUMAN Fu	6022 Aden	1GY3	2CN5	2UUE	30.8		Sc=6.29126, min distance = 2.04628
CHK2_HUMAN F	6420139 C	CDK2_HUMAN Fu	6022 Aden	1GY3	2CN5	2C5V	30.8		Sc=6.26751, min distance = 1.77710
CHK2_HUMAN F	6420140 2	CDK2_HUMAN Fu	6022 Aden	1GY3	2CN5	2C5Y	30.8		Sc=6.51041, min distance = 1.83192
CHK2_HUMAN F	6539118 g	CDK2_HUMAN Fu	6022 Aden	1GY3	2CN5	1FVV	30.8		Sc=6.4481, min distance = 1.595306
CHK2_HUMAN F	656971 CI	CDK2_HUMAN Fu	6022 Aden	1GY3	2CN5	1Y8Y	30.8		Sc=6.03085, min distance = 1.86429
CHK2_HUMAN F	657072 RY	CDK2_HUMAN Fu	6022 Aden	1GY3	2CN5	2BHH	30.8		Sc=6.48327, min distance = 2.24275
CHK2_HUMAN F	6852187 2	O57883_PYRHO	6022 Aden	1WNL	2CN5	2DTH		0.89	Sc=6.1144, min distance = 1.705257
CHK2_HUMAN F	6852201 2	CDK2_HUMAN Fu	6022 Aden	1GY3	2CN5	2G9X	30.8		Sc=6.59719, min distance = 1.26946
CHK2_HUMAN F	6918710 5	CDK2_HUMAN Fu	6022 Aden	1GY3	2CN5	3EJ1	30.8		Sc=6.19712, min distance = 2.37429
CHK2_HUMAN F	6918852 2	CDK2_HUMAN Fu	6022 Aden	1GY3	2CN5	2FVD	30.8		Sc=6.3954, min distance = 2.135726
CHK2_HUMAN F	72194 2-C	ENPL_CANFA Fu	6022 Aden	1TC6	2CN5	1QYE		0.89	Sc=6.07882, min distance = 2.05650
CHK2_HUMAN F	91532 AM	MTNK_BACSU Fu	6022 Aden	2OLC	2CN5	2PUL		0.99	Sc=6.50023, min distance = 2.34060

CHK2_HUMAN F	9547890 I	CDK2_HUMAN Fu	6022 Aden	1GY3	2CN5	1W8C	30.8		Sc=6.25936, min distance = 1.56097
CHK2_HUMAN F	9549254 2	CDK2_HUMAN Fu	6022 Aden	1GY3	2CN5	2IW6	30.8		Sc=6.50855, min distance = 1.78246
CHK2_HUMAN F	9817550 V	CDK2_HUMAN Fu	6022 Aden	1GY3	2CN5	3BHV	30.8		Sc=6.37834, min distance = 0.72819
CHK2_HUMAN F	9926933 i	CDK2_HUMAN Fu	6022 Aden	1GY3	2CN5	2VTI	30.8		Sc=6.18811, min distance = 1.99855
CHK2_HUMAN F	9991833 S	CDK2_HUMAN Fu	6022 Aden	1GY3	2CN5	2R3H	30.8		Sc=6.07213, min distance = 2.63714
CHK2_HUMAN F	9994066 4	CDK2_HUMAN Fu	6022 Aden	1GY3	2CN5	2VTJ	30.8		Sc=5.82047, min distance = 1.75716
CHMU_YEAST F	6140 L-ph	SYFA_THETH Fu	6057 L-ty	2AMC	4CSM	1B70		0.84	Sc=5.6984, min distance = 2.950383
CHOD_BREST F	16740985	FRDA_SHEFR Fu	444188 CI	1M64	1COY	1E39		0.93	Sc=6.44358, min distance = 2.05492
CHOD_BREST F	16741253	LSD1_HUMAN Fu	444188 CI	2DW4	1COY	2UXN		0.95	Sc=6.45278, min distance = 2.16643
CHOD_BREST F	444502 CI	FRDA_SHEFR Fu	444188 CI	1M64	1COY	1QJD		0.98	Sc=6.44661, min distance = 2.03783
CHOD_BREST F	444502 CI	GSHR_HUMAN Fu	444188 CI	3DK9	1COY	1BWC		0.98	Sc=6.44661, min distance = 2.31332
CHOD_BREST F	444502 CI	TYTR_TRYCR Fu	444188 CI	1BZL	1COY	1GXF		0.98	Sc=6.45032, min distance = 1.80955
CHOD_BREST F	448054 CI	FRDA_SHEFR Fu	444188 CI	1M64	1COY	1Y0P		0.97	Sc=6.44482, min distance = 2.08177
CHOD_BREST F	449465 CI	GSHR_HUMAN Fu	444188 CI	3DK9	1COY	3GRT		0.98	Sc=6.44561, min distance = 2.10968
CHOD_STRS0 F	16740985	FRDA_SHEFR Fu	444502 CI	1QJD	1B8S	1E39		0.92	Sc=6.44226, min distance = 2.11088
CHOD_STRS0 F	444563 CI	FRDA_SHEFR Fu	444502 CI	1QJD	1B8S	1P2E		0.98	Sc=6.8828, min distance = 2.072279
CHOD_STRS0 F	444969 CI	FRDA_SHEFR Fu	444502 CI	1QJD	1B8S	1JRY		0.98	Sc=6.44369, min distance = 2.08539
CHOD_STRS0 F	446546 CI	FRDA_SHEFR Fu	444502 CI	1QJD	1B8S	1KSS		0.98	Sc=6.44456, min distance = 2.10119
CHOD_STRS0 F	448054 CI	FRDA_SHEFR Fu	444502 CI	1QJD	1B8S	1Y0P		0.95	Sc=6.44402, min distance = 2.14037
CHOD_STRS0 F	449465 CI	GSHR_HUMAN Fu	444502 CI	1BWC	1B8S	3GRT		0.97	Sc=6.44848, min distance = 2.01237
CHOMT_MEDSA	5327118 5	Q70GK9_STRCT	34756 Acy	1RQP	1FPQ	2C2W		0.81	Sc=5.85611, min distance = 1.10643
CHS2_MEDSA F	10831270	TTHY_HUMAN Fu	445154 re	1DVS	1U0W	3CN3			Sc=5.74613, min distance = 2.05908
CHS2_MEDSA F	1184681 C	TTHY_HUMAN Fu	445154 re	1DVS	1U0W	3CN4			Sc=6.07377, min distance = 2.31998
CHS2_MEDSA F	119346 3,	TTHY_HUMAN Fu	445154 re	1DVS	1U0W	2GAB			Sc=5.78329, min distance = 2.58446
CHS2_MEDSA F	177880 2'	TTHY_HUMAN Fu	445154 re	1DVS	1U0W	2G9K			Sc=5.79667, min distance = 1.84003
CHS2_MEDSA F	20284644	TTHY_HUMAN Fu	445154 re	1DVS	1U0W	2QGC			Sc=6.01959, min distance = 2.11242
CHS2_MEDSA F	23722943	TTHY_HUMAN Fu	445154 re	1DVS	1U0W	2QGD			Sc=5.9579, min distance = 2.352433
CHS2_MEDSA F	243556 NS	TTHY_HUMAN Fu	445154 re	1DVS	1U0W	3CN2			Sc=5.72932, min distance = 2.38839
CHS2_MEDSA F	25011739	TTHY_HUMAN Fu	445154 re	1DVS	1U0W	3CN0		0.79	Sc=5.87824, min distance = 2.08686
CHS2_MEDSA F	25011740	TTHY_HUMAN Fu	445154 re	1DVS	1U0W	3CN1			Sc=5.81972, min distance = 2.69509
CHS2_MEDSA F	3033 dicl	TTHY_HUMAN Fu	445154 re	1DVS	1U0W	1DVX			Sc=6.14191, min distance = 0.97769
CHS2_MEDSA F	4369477 D	TTHY_HUMAN Fu	445154 re	1DVS	1U0W	1U21			Sc=6.14513, min distance = 1.81791
CHS2_MEDSA F	448708 CI	TTHY_HUMAN Fu	445154 re	1DVS	1U0W	1THC			Sc=6.35425, min distance = 2.11954
CHS2_MEDSA F	5327160 2	TTHY_HUMAN Fu	445154 re	1DVS	1U0W	2F8I			Sc=6.11622, min distance = 2.29375
CHS2_MEDSA F	667639 pi	ATPG_BOVIN Fu	445154 re	2JIZ	1U0W	2JJ1		0.93	Sc=6.22985, min distance = 1.55715
CHS2_MEDSA F	8631 3,5-	TTHY_HUMAN Fu	445154 re	1DVS	1U0W	3B56			Sc=5.7389, min distance = 2.218237
CHS2_MEDSA F	97032 1g3	TTHY_HUMAN Fu	445154 re	1DVS	1U0W	2G5U			Sc=5.916, min distance = 2.242632

CHS_ECOLX Fu	18068 Udp	GALE_ECOLI Fu	6031 Urid	1NAH	2Z86	1A9Z	0.89	Sc=5.82388, min distance = 2.12537
CHS_ECOLX Fu	8977 1dar	O33839_THEMA	6031 Urid	1XJG	2Z86	1XJE		Sc=5.9884, min distance = 1.903689
CHTA_VIBCH F	128225 be	NAR2B_RAT Ful	5893 nadi	1OG4	2A5F	1OG1		Sc=6.23164, min distance = 2.38332
CHTB_VIBCH F	444635 O3	MBL1_RAT Full	439353 be	2KMB	3CHB	3KMB	0.9	Sc=5.77431, min distance = 1.26010
CISY_ABDS2 F	439276 3-	GGGPS_ARCFU F	311 citri	2F6U	1A59	2F6X		Sc=5.60361, min distance = 1.88111
CISY_ABDS2 F	51 2-Oxop	SERA_ECOLI Fu	311 citri	2P9E	1A59	1YBA	0.76	Sc=5.75199, min distance = 0.78581
CISY_BACSU F	12136 Ber	POL_HV1N5 Ful	311 citri	2GON	2C6X	9HVP		Sc=5.67848, min distance = 1.73949
CISY_CHICK F	439276 3-	GGGPS_ARCFU F	311 citri	2F6U	6CSC	2F6X		Sc=5.6056, min distance = 1.667484
CISY_CHICK F	51 2-Oxop	SERA_ECOLI Fu	311 citri	2P9E	6CSC	1YBA	0.76	Sc=5.80063, min distance = 1.28523
CISY_CHICK F	6961 PYRO	FUMC_ECOLI Fu	311 citri	1FUO	6CSC	1FUP		Sc=6.31462, min distance = 0.98723
CISY_PIG Ful	439276 3-	GGGPS_ARCFU F	311 citri	2F6U	2CTS	2F6X		Sc=5.6094, min distance = 1.814163
CISY_PIG Ful	439554 fu	A2NHM3_MOUSE	311 citri	1ZEA	2CTS	1CLZ		Sc=5.71804, min distance = 1.20585
CISY_PIG Ful	447532 1c	SRC_HUMAN Ful	311 citri	1O4L	2CTS	1O4H		Sc=6.16659, min distance = 2.01934
CISY_PIG Ful	51 2-Oxop	SERA_ECOLI Fu	311 citri	2P9E	2CTS	1YBA	0.76	Sc=5.75199, min distance = 1.32573
CISY_PIG Ful	8158 Pela	ALBU_HUMAN Fu	311 citri	1TF0	2CTS	1E7E		Sc=5.62596, min distance = 1.09388
CISY_PIG Ful	8180 UNDE	ALBU_HUMAN Fu	311 citri	1TF0	2CTS	1E7F		Sc=5.65108, min distance = 2.06653
CISY_PYRFU F	447532 1c	SRC_HUMAN Ful	311 citri	1O4L	1AJ8	1O4H		Sc=6.15643, min distance = 2.05375
CISY_PYRFU F	449604 NI	ACON_BOVIN Fu	311 citri	1C96	1AJ8	8ACN		Sc=5.87055, min distance = 1.89497
CISY_PYRFU F	6852187 2	PPNK1_LISMO F	311 citri	2I29	1AJ8	2I2B		Sc=5.99795, min distance = 2.46270
CITA_KLEPN F	444212 tr	ACON_BOVIN Fu	311 citri	1C96	1P0Z	1ACO		Sc=5.90909, min distance = 2.27185
CKI1_SCHPO F	11314340	KAPCA_BOVIN F	5957 Aden	1Q24	1CSN	2UZT		Sc=6.34076, min distance = 2.14729
CKI1_SCHPO F	11608401	KAPCA_BOVIN F	5957 Aden	1Q24	1CSN	2UVY		Sc=6.07576, min distance = 2.15529
CKI1_SCHPO F	11957393	FAK1_HUMAN Fu	5957 Aden	2IJM	1CSN	2ETM		Sc=6.23145, min distance = 2.20455
CKI1_SCHPO F	153999 Ru	PDPK1_HUMAN F	5957 Aden	2BIY	1CSN	1UU3		Sc=6.37292, min distance = 2.20818
CKI1_SCHPO F	15602983	KAPCA_BOVIN F	5957 Aden	1Q24	1CSN	2UW5		Sc=6.14757, min distance = 2.17442
CKI1_SCHPO F	16122633	KAPCA_BOVIN F	5957 Aden	1Q24	1CSN	2UW0		Sc=6.38491, min distance = 2.08845
CKI1_SCHPO F	17751819	KAPCA_BOVIN F	5957 Aden	1Q24	1CSN	2VO6		Sc=6.29478, min distance = 2.39843
CKI1_SCHPO F	17754396	PDPK1_HUMAN F	5957 Aden	2BIY	1CSN	2R7B		Sc=6.2904, min distance = 2.535747
CKI1_SCHPO F	2396 bisi	PDPK1_HUMAN F	5957 Aden	2BIY	1CSN	1UU8		Sc=6.42499, min distance = 2.24301
CKI1_SCHPO F	2398 bisi	PDPK1_HUMAN F	5957 Aden	2BIY	1CSN	1UU9		Sc=6.47298, min distance = 2.41735
CKI1_SCHPO F	2403 1uvr	PDPK1_HUMAN F	5957 Aden	2BIY	1CSN	1UVR		Sc=6.41142, min distance = 2.18032
CKI1_SCHPO F	3540 1yds	KAPCA_BOVIN F	5957 Aden	1Q24	1CSN	1YDS		Sc=5.84248, min distance = 2.44622
CKI1_SCHPO F	3547 Fasu	KAPCA_BOVIN F	5957 Aden	1Q24	1CSN	1Q8W		Sc=5.9169, min distance = 2.289310
CKI1_SCHPO F	440317 AT	PURT_ECOLI Fu	5957 Aden	1KJ8	1CSN	1KJJ	0.99	Sc=5.96788, min distance = 2.47463
CKI1_SCHPO F	444345 1c	PDPK1_HUMAN F	5957 Aden	2BIY	1CSN	1OKY		Sc=6.34784, min distance = 2.24536
CKI1_SCHPO F	444345 1c	TAOK2_RAT Ful	5957 Aden	1U5R	1CSN	2GCD		Sc=6.36951, min distance = 1.52434
CKI1_SCHPO F	444564 AD	BIOD_ECOLI Fu	5957 Aden	1A82	1CSN	1BS1	0.91	Sc=6.35033, min distance = 2.32461

CKI1_SCHPO F	444564 AD	MYS2_DICDI Fu	5957 Aden	1FMW	1CSN	1W9I		0.91	Sc=6.33805, min distance = 0
CKI1_SCHPO F	444852 CI	ATPA1_BOVIN F	5957 Aden	2V7Q	1CSN	1COW		0.91	Sc=6.42412, min distance = 1.623009
CKI1_SCHPO F	447840 CI	PHKG1_RABIT F	5957 Aden	1QL6	1CSN	1PHK			Sc=5.96842, min distance = 2.484066
CKI1_SCHPO F	447916 ac	RIO1_ARCFU Fu	5957 Aden	1ZP9	1CSN	1ZTF		0.94	Sc=6.09769, min distance = 3.222141
CKI1_SCHPO F	448043 2g	KAPCA_BOVIN F	5957 Aden	1Q24	1CSN	1Q8U			Sc=5.97728, min distance = 2.291332
CKI1_SCHPO F	449240 1y	KAPCA_BOVIN F	5957 Aden	1Q24	1CSN	1YDR			Sc=5.90944, min distance = 2.213515
CKI1_SCHPO F	5287969 E	CDK9_HUMAN Fu	5957 Aden	3BLQ	1CSN	3BLR			Sc=6.36579, min distance = 2.173030
CKI1_SCHPO F	6022 Aden	ACT1_DROME Fu	5957 Aden	2HF4	1CSN	2HF3		0.99	Sc=6.35552, min distance = 2.160582
CKI1_SCHPO F	6022 Aden	ARSA1_ECOLX F	5957 Aden	1II0	1CSN	1IHU		0.99	Sc=6.36101, min distance = 2.259845
CKI1_SCHPO F	6022 Aden	BIOD_ECOLI Fu	5957 Aden	1A82	1CSN	1DAD		0.99	Sc=6.34503, min distance = 2.116595
CKI1_SCHPO F	6022 Aden	CLCN5_HUMAN F	5957 Aden	2J9L	1CSN	2JA3		0.99	Sc=6.35256, min distance = 2.664727
CKI1_SCHPO F	6022 Aden	DDL_THET8 Ful	5957 Aden	2ZDQ	1CSN	2ZDH		0.99	Sc=5.93169, min distance = 2.039768
CKI1_SCHPO F	6022 Aden	FAK1_HUMAN Fu	5957 Aden	2IJM	1CSN	1MP8		0.99	Sc=5.90958, min distance = 2.130334
CKI1_SCHPO F	6022 Aden	HSP7F_YEAST F	5957 Aden	3D2F	1CSN	3C7N		0.99	Sc=6.33916, min distance = 2.145406
CKI1_SCHPO F	6022 Aden	MUTS_ECOLI Fu	5957 Aden	1W7A	1CSN	1OH7		0.99	Sc=6.35458, min distance = 2.407821
CKI1_SCHPO F	6022 Aden	PURK_ECOLI Fu	5957 Aden	3ETH	1CSN	3ETJ		0.99	Sc=6.3416, min distance = 2.3639940
CKI1_SCHPO F	6022 Aden	Q72H90_THET2 I	5957 Aden	2BEK	1CSN	2BEJ		0.99	Sc=6.34028, min distance = 1.981186
CKI1_SCHPO F	6022 Aden	RK_BOVIN Full-	5957 Aden	3C4W	1CSN	3C4Z		0.99	Sc=5.91261, min distance = 2.513681
CKI1_SCHPO F	6083 aden	AAKG1_RAT Ful	5957 Aden	2V92	1CSN	2V8Q		0.98	Sc=6.26529, min distance = 2.282788
CKI1_SCHPO F	6083 aden	ASSY_THET8 Fu	5957 Aden	1J1Z	1CSN	1J20		0.98	Sc=6.34029, min distance = 1.159248
CKI1_SCHPO F	6083 aden	PURP_PYRFU Fu	5957 Aden	2R86	1CSN	2R85		0.98	Sc=6.27849, min distance = 2.667036
CKI1_SCHPO F	6133 uric	Q381M1_9TRYP I	5957 Aden	2Q0D	1CSN	2IKF			Sc=5.85102, min distance = 2.549730
CKI1_SCHPO F	6176 Cyti	Q381M1_9TRYP I	5957 Aden	2Q0D	1CSN	2Q0C			Sc=5.84754, min distance = 2.558195
CKI1_SCHPO F	6852207 M	KAPCA_BOVIN F	5957 Aden	1Q24	1CSN	2GNI			Sc=5.90171, min distance = 2.346868
CKI1_SCHPO F	91532 AMP	INSR_HUMAN Fu	5957 Aden	3BU5	1CSN	1I44		0.99	Sc=5.99509, min distance = 1.996288
CKI1_SCHPO F	91532 AMP	PURT_ECOLI Fu	5957 Aden	1KJ8	1CSN	1KJI		0.99	Sc=6.00287, min distance = 2.585928
CLAT_HUMAN F	444493 ac	CACP_MOUSE Fu	87642 coe	1T7Q	2FY4	2H3P	44.69	0.98	Sc=6.04151, min distance = 2.154871
CLC4K_HUMAN I	151504 D-	CVN_NOSEL Ful	185698 al	2RDK	3BC7	2PYS			Sc=5.78908, min distance = 2.458272
CLC4M_HUMAN I	445948 D-	Q9HYN5_PSEAE I	439353 be	1W8F	1SL6	1OXC			Sc=5.67529, min distance = 2.051822
CLC7A_MOUSE I	5326754 S	PHSM_ECOLI Fu	64689 bet	2ASV	2CL8	1QM5		0.88	Sc=5.76476, min distance = 0.416118
CLC7A_MOUSE I	5327083 C	GUN2_THEFU Fu	64689 bet	2BOF	2CL8	2BOD			Sc=5.673, min distance = 1.0591959
CLCN5_HUMAN I	11268094	CDK2_HUMAN Fu	6022 Aden	1GY3	2JA3	2VTQ			Sc=6.49324, min distance = 0.519986
CLCN5_HUMAN I	11696113	KAPCA_BOVIN F	5957 Aden	1Q24	2J9L	2VO3			Sc=6.31323, min distance = 1.696508
CLCN5_HUMAN I	11957417	CDK2_HUMAN Fu	6022 Aden	1GY3	2JA3	2I40			Sc=6.35684, min distance = 1.152299
CLCN5_HUMAN I	16046126	CDK2_HUMAN Fu	6022 Aden	1GY3	2JA3	2DS1			Sc=6.51804, min distance = 1.264550
CLCN5_HUMAN I	16122635	KAPCA_BOVIN F	5957 Aden	1Q24	2J9L	2UW8			Sc=5.60666, min distance = 2.271720
CLCN5_HUMAN I	16214825	CDK2_HUMAN Fu	6022 Aden	1GY3	2JA3	2UZE			Sc=6.38405, min distance = 0.871585

CLCN5_HUMAN I	164628 de	PMS2_HUMAN Fu	6022	Aden	1EA6	2JA3	1H7U			Sc=6.42637, min distance = 1.45735
CLCN5_HUMAN I	17751819	KAPCA_BOVIN Fu	5957	Aden	1Q24	2J9L	2VO6			Sc=6.28649, min distance = 2.27782
CLCN5_HUMAN I	23653515	CDK2_HUMAN Fu	6022	Aden	1GY3	2JA3	2R3F			Sc=6.20012, min distance = 1.67870
CLCN5_HUMAN I	23653518	CDK2_HUMAN Fu	6022	Aden	1GY3	2JA3	2R3J			Sc=6.26, min distance = 1.32347572
CLCN5_HUMAN I	23653519	CDK2_HUMAN Fu	6022	Aden	1GY3	2JA3	2R3K			Sc=6.35673, min distance = 1.76977
CLCN5_HUMAN I	23653521	CDK2_HUMAN Fu	6022	Aden	1GY3	2JA3	2R3M			Sc=6.53395, min distance = 1.06354
CLCN5_HUMAN I	23653522	CDK2_HUMAN Fu	6022	Aden	1GY3	2JA3	2R3N			Sc=6.33074, min distance = 1.28303
CLCN5_HUMAN I	24762195	KAPCA_BOVIN Fu	5957	Aden	1Q24	2J9L	2VO0			Sc=6.29032, min distance = 2.05922
CLCN5_HUMAN I	24832037	PGK1_HUMAN Fu	6022	Aden	2ZGV	2JA3	3C3C			Sc=5.7908, min distance = 2.016215
CLCN5_HUMAN I	24864077	CDK2_HUMAN Fu	6022	Aden	1GY3	2JA3	2VTN			Sc=6.35131, min distance = 0.44149
CLCN5_HUMAN I	24864079	CDK2_HUMAN Fu	6022	Aden	1GY3	2JA3	2VTP			Sc=6.43168, min distance = 2.07629
CLCN5_HUMAN I	24864080	CDK2_HUMAN Fu	6022	Aden	1GY3	2JA3	2VTR			Sc=6.10137, min distance = 1.81760
CLCN5_HUMAN I	24864082	CDK2_HUMAN Fu	6022	Aden	1GY3	2JA3	2VTT			Sc=6.48584, min distance = 0.95476
CLCN5_HUMAN I	3540 1yds	IPKA_RAT Full=	6022	Aden	1L3R	2JA3	1YDS			Sc=5.91126, min distance = 2.01666
CLCN5_HUMAN I	3540 1yds	KAPCA_BOVIN Fu	5957	Aden	1Q24	2J9L	1YDS			Sc=5.93846, min distance = 1.88453
CLCN5_HUMAN I	4369136 C	CDK2_HUMAN Fu	6022	Aden	1GY3	2JA3	1DM2			Sc=6.32029, min distance = 2.18631
CLCN5_HUMAN I	440317 AT	BCKD_RAT Full=	6022	Aden	1GKZ	2JA3	1GJV		0.99	Sc=5.95417, min distance = 1.85508
CLCN5_HUMAN I	440317 AT	FTSK_PSEAE Fu	6022	Aden	2IUU	2JA3	2IUT		0.99	Sc=6.44157, min distance = 1.35700
CLCN5_HUMAN I	444503 FU	NDKC_DICDI Fu	6022	Aden	1KDN	2JA3	1B99			Sc=6.24872, min distance = 2.07862
CLCN5_HUMAN I	444564 AD	BIOD_ECOLI Fu	5957	Aden	1A82	2J9L	1BS1		0.91	Sc=6.40508, min distance = 1.97620
CLCN5_HUMAN I	444564 AD	BIOD_ECOLI Fu	6022	Aden	1DAD	2JA3	1BS1		0.91	Sc=6.34194, min distance = 2.16502
CLCN5_HUMAN I	444564 AD	MYS2_DICDI Fu	5957	Aden	1FMW	2J9L	1W9I		0.91	Sc=5.91025, min distance = 0
CLCN5_HUMAN I	444564 AD	MYS2_DICDI Fu	6022	Aden	1VOM	2JA3	1W9I		0.91	Sc=6.40128, min distance = 0.96122
CLCN5_HUMAN I	444842 CI	CDK2_HUMAN Fu	6022	Aden	1GY3	2JA3	1CKP			Sc=5.62455, min distance = 2.42366
CLCN5_HUMAN I	445840 di	CDK2_HUMAN Fu	6022	Aden	1GY3	2JA3	1GII			Sc=6.39293, min distance = 1.13257
CLCN5_HUMAN I	445966 O6	CDK2_HUMAN Fu	6022	Aden	1GY3	2JA3	1H0V			Sc=6.27543, min distance = 2.22578
CLCN5_HUMAN I	447048 2b	Y1521_ARCFU Fu	6022	Aden	2BFR	2JA3	2BFQ		0.95	Sc=6.05427, min distance = 2.10266
CLCN5_HUMAN I	447656 1c	CDK2_HUMAN Fu	6022	Aden	1GY3	2JA3	1OIY			Sc=6.57578, min distance = 0.75298
CLCN5_HUMAN I	447766 1p	CDK2_HUMAN Fu	6022	Aden	1GY3	2JA3	1P2A			Sc=6.3996, min distance = 1.661141
CLCN5_HUMAN I	447967 CI	CDK2_HUMAN Fu	6022	Aden	1GY3	2JA3	1PYE			Sc=6.41439, min distance = 1.89892
CLCN5_HUMAN I	448005 CI	GSK3B_HUMAN Fu	6022	Aden	1J1C	2JA3	1Q3W			Sc=6.36436, min distance = 0.63774
CLCN5_HUMAN I	448014 1c	GSK3B_HUMAN Fu	6022	Aden	1J1C	2JA3	1Q5K			Sc=6.37616, min distance = 1.39590
CLCN5_HUMAN I	448042 2e	KAPCA_BOVIN Fu	5957	Aden	1Q24	2J9L	1Q8T			Sc=6.16445, min distance = 2.01302
CLCN5_HUMAN I	448043 2g	KAPCA_BOVIN Fu	5957	Aden	1Q24	2J9L	1Q8U			Sc=6.04484, min distance = 2.00044
CLCN5_HUMAN I	449088 1v	CDK2_HUMAN Fu	6022	Aden	1GY3	2JA3	1VYZ			Sc=6.15391, min distance = 1.90918
CLCN5_HUMAN I	449240 1y	KAPCA_BOVIN Fu	5957	Aden	1Q24	2J9L	1YDR			Sc=5.99625, min distance = 1.93814
CLCN5_HUMAN I	4564 1e1v	CDK2_HUMAN Fu	6022	Aden	1GY3	2JA3	1E1V			Sc=6.22689, min distance = 2.27063

CLCN5_HUMAN I	4565 1h1r	CDK2_HUMAN Fu	6022 Aden 1GY3	2JA3	1H1R		Sc=6.39063, min distance = 0.917148
CLCN5_HUMAN I	5288641 i	CDK2_HUMAN Fu	6022 Aden 1GY3	2JA3	1E9H		Sc=6.34855, min distance = 0.996004
CLCN5_HUMAN I	5289411 1	CDK2_HUMAN Fu	6022 Aden 1GY3	2JA3	1OGU		Sc=6.5839, min distance = 1.3723651
CLCN5_HUMAN I	5327124	CDK2_HUMAN Fu	6022 Aden 1GY3	2JA3	2C4G		Sc=6.36362, min distance = 1.683861
CLCN5_HUMAN I	5331010 c	CDK2_HUMAN Fu	6022 Aden 1GY3	2JA3	2BKZ		Sc=6.32036, min distance = 0.802591
CLCN5_HUMAN I	6031 Uric	DCK_HUMAN Ful	6022 Aden 1P5Z	2JA3	2ZIA		Sc=6.20544, min distance = 1.814010
CLCN5_HUMAN I	6031 Uric	RIR1_YEAST Fu	6022 Aden 2CVX	2JA3	2CVV		Sc=5.8138, min distance = 1.7880732
CLCN5_HUMAN I	6083 aden	AAKG1_RAT Ful	5957 Aden 2V92	2J9L	2V8Q	0.98	Sc=6.42271, min distance = 0.950917
CLCN5_HUMAN I	6083 aden	PURP_METJA Fu	5957 Aden 2R7L	2J9L	2R7M	0.98	Sc=6.35674, min distance = 1.897548
CLCN5_HUMAN I	6083 aden	PURP_METJA Fu	6022 Aden 2R7N	2JA3	2R7M	0.99	Sc=6.35386, min distance = 2.361850
CLCN5_HUMAN I	6083 aden	PURP_PYRFU Fu	5957 Aden 2R86	2J9L	2R85	0.98	Sc=6.37563, min distance = 2.077726
CLCN5_HUMAN I	60961 ade	IPKA_RABIT Fu	6022 Aden 1JBP	2JA3	1FMO	0.95	Sc=6.26369, min distance = 1.987767
CLCN5_HUMAN I	6132 Cyti	RIR1_YEAST Fu	6022 Aden 2CVX	2JA3	2CVU		Sc=6.26962, min distance = 1.657096
CLCN5_HUMAN I	6420138 2	CDK2_HUMAN Fu	6022 Aden 1GY3	2JA3	2UUE		Sc=6.47162, min distance = 1.248911
CLCN5_HUMAN I	6420139 C	CDK2_HUMAN Fu	6022 Aden 1GY3	2JA3	2C5V		Sc=6.35233, min distance = 0.886585
CLCN5_HUMAN I	65059 Dec	RNAS1_BOVIN Fu	6022 Aden 1O0H	2JA3	2QCA	0.77	Sc=6.25468, min distance = 1.766096
CLCN5_HUMAN I	6804 guar	NDK_PYRHO Ful	6022 Aden 2DYA	2JA3	2DXF	0.8	Sc=6.37695, min distance = 2.181076
CLCN5_HUMAN I	6830 guar	Q381M1_9TRYP I	5957 Aden 2Q0D	2J9L	2Q0E	0.8	Sc=5.9906, min distance = 2.2592503
CLCN5_HUMAN I	6852207 M	KAPCA_BOVIN Fu	5957 Aden 1Q24	2J9L	2GNI		Sc=6.01391, min distance = 1.894894
CLCN5_HUMAN I	8977 1dar	NDK_PYRHO Ful	6022 Aden 2DYA	2JA3	2DXE	0.8	Sc=6.42316, min distance = 2.219009
CLCN5_HUMAN I	91532 AMP	NIFH1_AZOVI Fu	5957 Aden 2C8V	2J9L	2AFK	0.99	Sc=5.73659, min distance = 1.261467
CLCN5_HUMAN I	9547944 2	RNAS1_BOVIN Fu	6022 Aden 1O0H	2JA3	2G8R		Sc=6.49215, min distance = 0.518475
CLCN5_HUMAN I	9817550 V	CDK2_HUMAN Fu	6022 Aden 1GY3	2JA3	3BHV		Sc=6.37079, min distance = 1.903372
CLCN5_HUMAN I	9991833 S	CDK2_HUMAN Fu	6022 Aden 1GY3	2JA3	2R3H		Sc=6.24246, min distance = 0.583581
CLP1_YEAST Fu	11608401	KAPCA_BOVIN Fu	5957 Aden 1Q24	2NPI	2UVY		Sc=6.23363, min distance = 1.926430
CLP1_YEAST Fu	3540 1yds	KAPCA_BOVIN Fu	5957 Aden 1Q24	2NPI	1YDS		Sc=5.9272, min distance = 2.1794334
CLP1_YEAST Fu	3547 Fasu	KAPCA_BOVIN Fu	5957 Aden 1Q24	2NPI	1Q8W		Sc=5.97951, min distance = 2.248740
CLP1_YEAST Fu	444564 AD	BIOD_ECOLI Fu	5957 Aden 1A82	2NPI	1BS1	0.91	Sc=6.00521, min distance = 2.163331
CLP1_YEAST Fu	444564 AD	MYS2_DICDI Fu	5957 Aden 1FMW	2NPI	1W9I	0.91	Sc=6.0094, min distance = 0
CLP1_YEAST Fu	448042 2e	KAPCA_BOVIN Fu	5957 Aden 1Q24	2NPI	1Q8T		Sc=6.14112, min distance = 2.493846
CLP1_YEAST Fu	448043 2g	KAPCA_BOVIN Fu	5957 Aden 1Q24	2NPI	1Q8U		Sc=6.05675, min distance = 2.011957
CLP1_YEAST Fu	449240 1y	KAPCA_BOVIN Fu	5957 Aden 1Q24	2NPI	1YDR		Sc=5.98692, min distance = 2.297555
CLP1_YEAST Fu	6022 Aden	ARSA1_ECOLX Fu	5957 Aden 1II0	2NPI	1IHU	0.99	Sc=6.01953, min distance = 2.258491
CLP1_YEAST Fu	6022 Aden	CLCN5_HUMAN Fu	5957 Aden 2J9L	2NPI	2JA3	0.99	Sc=5.99926, min distance = 2.435361
CLP1_YEAST Fu	6022 Aden	HSP7F_YEAST Fu	5957 Aden 3D2F	2NPI	3C7N	0.99	Sc=5.98903, min distance = 2.190246
CLP1_YEAST Fu	6022 Aden	KTHY_HUMAN Fu	5957 Aden 1E2Q	2NPI	1NN3	0.99	Sc=6.00521, min distance = 2.054576
CLP1_YEAST Fu	6022 Aden	MUTS_ECOLI Fu	5957 Aden 1W7A	2NPI	1OH7	0.99	Sc=6.01113, min distance = 2.254372

CLP1_YEAST Fı	6022 Ader	MYS2_DICDI Fu:	5957 Aden	1FMW	2NPI	1VOM		0.99	Sc=6.01953, min distance = 0
CLP1_YEAST Fı	6022 Ader	PPCK_ECOLI Fu:	5957 Aden	2OLR	2NPI	1K3C		0.99	Sc=6.0094, min distance = 2.104014(
CLP1_YEAST Fı	6022 Ader	PURK_ECOLI Fu:	5957 Aden	3ETH	2NPI	3ETJ		0.99	Sc=6.45508, min distance = 2.26224(
CLP1_YEAST Fı	6022 Ader	Q72H90_THET2 I	5957 Aden	2BEK	2NPI	2BEJ		0.99	Sc=6.00589, min distance = 2.06551:
CLP1_YEAST Fı	6022 Ader	RK_BOVIN Full:	5957 Aden	3C4W	2NPI	3C4Z		0.99	Sc=6.02484, min distance = 1.82275!
CLP1_YEAST Fı	6083 ader	AAKG1_RAT Ful:	5957 Aden	2V92	2NPI	2V8Q		0.98	Sc=5.92201, min distance = 2.21572‹
CLP1_YEAST Fı	6083 ader	PURP_METJA Fu:	5957 Aden	2R7L	2NPI	2R7M		0.98	Sc=5.92818, min distance = 2.01139:
CLP1_YEAST Fı	6083 ader	PURP_PYRFU Fu:	5957 Aden	2R86	2NPI	2R85		0.98	Sc=5.92437, min distance = 2.04538(
CLP1_YEAST Fı	6083 ader	Y059_METJA Fu:	5957 Aden	2J9C	2NPI	2J9D		0.98	Sc=5.90489, min distance = 2.01851'
CLP1_YEAST Fı	6102784 A	PSPF_ECOLI Fu:	5957 Aden	2C96	2NPI	2C99		0.99	Sc=6.05611, min distance = 2.05066:
CLP1_YEAST Fı	6852207 M	KAPCA_BOVIN Fı	5957 Aden	1Q24	2NPI	2GNI			Sc=5.98583, min distance = 2.29059{
CLP1_YEAST Fı	91532 AMF	BIOD_ECOLI Fu:	5957 Aden	1A82	2NPI	1DAG		0.99	Sc=6.08532, min distance = 1.98794:
CLP1_YEAST Fı	91532 AMF	INSR_HUMAN Fu:	5957 Aden	3BU5	2NPI	1I44		0.99	Sc=6.54607, min distance = 2.05326‹
CLPB_THET8 Fı	16750062	CSK2A_MAIZE Fı	33113 gam	1LP4	1QVR	2OXD			Sc=5.74491, min distance = 1.63283:
CLPB_THET8 Fı	3064778 H	ROCK1_HUMAN Fı	33113 gam	2V55	1QVR	2ETK			Sc=6.13281, min distance = 1.44509‹
CLPB_THET8 Fı	444564 AD	MYS2_DICDI Fu:	33113 gam	1MMN	1QVR	1W9I		0.91	Sc=6.39849, min distance = 1.48275(
CLPB_THET8 Fı	447004 CI	MYS2_DICDI Fu:	33113 gam	1MMN	1QVR	1LVK			Sc=6.15811, min distance = 1.73551!
CLPB_THET8 Fı	448042 2e	ROCK1_HUMAN Fı	33113 gam	2V55	1QVR	2ETR			Sc=6.17987, min distance = 2.02727:
CLPB_THET8 Fı	5149739 2	CSK2A_MAIZE Fı	33113 gam	1LP4	1QVR	2OXY			Sc=5.62544, min distance = 1.58588!
CLPB_THET8 Fı	5326976 1	CSK2A_MAIZE Fı	33113 gam	1LP4	1QVR	1ZOE			Sc=5.78205, min distance = 1.62111!
CLPB_THET8 Fı	5326978 1	CSK2A_MAIZE Fı	33113 gam	1LP4	1QVR	1ZOH			Sc=5.72555, min distance = 1.72051(
CLPB_THET8 Fı	5327148 C	IPKA_RABIT Fu:	33113 gam	1CDK	1QVR	2ERZ			Sc=6.13712, min distance = 2.25784:
CLPB_THET8 Fı	91532 AMF	CHEA_THEMA Fu:	33113 gam	1I59	1QVR	1I58		0.99	Sc=6.47064, min distance = 1.29573!
CLPB_THET8 Fı	91532 AMF	IGF1R_HUMAN Fı	33113 gam	1JQH	1QVR	1K3A		0.99	Sc=6.44154, min distance = 1.74345:
CLPB_THET8 Fı	91532 AMF	PURT_ECOLI Fu:	33113 gam	1EYZ	1QVR	1KJI		0.99	Sc=6.47134, min distance = 1.72185:
CLPP_ECOLI Fı	444211 th	ACES_TORCA Fu:	8172 Trig	2C5G	2FZS	1ACJ			Sc=5.74814, min distance = 2.07373‹
CLPX_HELPY Fı	11957393	FAK1_HUMAN Fu:	6022 Aden	1MP8	1UM8	2ETM			Sc=6.26216, min distance = 1.96244(
CLPX_HELPY Fı	1540 1h1q	CDK2_HUMAN Fu:	6022 Aden	1GY3	1UM8	1H1Q			Sc=6.36793, min distance = 1.73350!
CLPX_HELPY Fı	188966 dA	HSLU_ECOLI Fu:	6022 Aden	1HQY	1UM8	1G4A		0.98	Sc=5.96547, min distance = 2.16239!
CLPX_HELPY Fı	23653515	CDK2_HUMAN Fu:	6022 Aden	1GY3	1UM8	2R3F			Sc=5.96764, min distance = 2.18835(
CLPX_HELPY Fı	23653518	CDK2_HUMAN Fu:	6022 Aden	1GY3	1UM8	2R3J			Sc=6.16345, min distance = 1.76049:
CLPX_HELPY Fı	23653519	CDK2_HUMAN Fu:	6022 Aden	1GY3	1UM8	2R3K			Sc=6.15692, min distance = 1.68437‹
CLPX_HELPY Fı	23653520	CDK2_HUMAN Fu:	6022 Aden	1GY3	1UM8	2R3L			Sc=6.24794, min distance = 1.86741:
CLPX_HELPY Fı	23653526	CDK2_HUMAN Fu:	6022 Aden	1GY3	1UM8	2R3R			Sc=6.25142, min distance = 2.06246!
CLPX_HELPY Fı	2608 1jsv	CDK2_HUMAN Fu:	6022 Aden	1GY3	1UM8	1JSV			Sc=6.04385, min distance = 2.10259(
CLPX_HELPY Fı	398148 1e	CDK2_HUMAN Fu:	6022 Aden	1GY3	1UM8	1E1X			Sc=6.16111, min distance = 1.98776:
CLPX_HELPY Fı	444564 AD	BIOD_ECOLI Fu:	6022 Aden	1DAD	1UM8	1BS1		0.91	Sc=5.67541, min distance = 2.07661!

CLPX_HELPY Fı	445966 O6	CDK2_HUMAN Fu	6022 Aden	1GY3	1UM8	1H0V			Sc=6.06749, min distance = 2.309791
CLPX_HELPY Fı	447649 1c	CDK2_HUMAN Fu	6022 Aden	1GY3	1UM8	1OI9			Sc=6.4305, min distance = 1.7365039
CLPX_HELPY Fı	447656 1c	CDK2_HUMAN Fu	6022 Aden	1GY3	1UM8	1OIY			Sc=6.5088, min distance = 1.3009246
CLPX_HELPY Fı	4564 1e1v	CDK2_HUMAN Fu	6022 Aden	1GY3	1UM8	1E1V			Sc=6.1216, min distance = 2.2335337
CLPX_HELPY Fı	4565 1h1r	CDK2_HUMAN Fu	6022 Aden	1GY3	1UM8	1H1R			Sc=6.36505, min distance = 1.959976
CLPX_HELPY Fı	9547890 I	CDK2_HUMAN Fu	6022 Aden	1GY3	1UM8	1W8C			Sc=6.22897, min distance = 2.342639
CLPX_HELPY Fı	9991833 S	CDK2_HUMAN Fu	6022 Aden	1GY3	1UM8	2R3H			Sc=6.04877, min distance = 2.103769
CLPX_HELPY Fı	9994066 4	CDK2_HUMAN Fu	6022 Aden	1GY3	1UM8	2VTJ			Sc=5.80074, min distance = 2.243463
CM4T_STRPE Fı	188380 Ac	MCES_ENCCU Fu	439155 Ad	1RI1	1TW3	1Z3C		0.91	Sc=6.55295, min distance = 2.067794
CM4T_STRPE Fı	446535 CI	HNMT_HUMAN Fu	439155 Ad	2AOT	1TW3	1JQE		0.95	Sc=6.52, min distance = 2.19045817
CM4T_STRPE Fı	60961 ade	PIMT_PYRFU Fu	439155 Ad	1JG1	1TW3	1JG2		0.84	Sc=6.2668, min distance = 2.3815299
CMA1_HUMAN Fı	8200 TETF	LMBL1_HUMAN Fı	78165 MES	1OZ2	1T31	2RHI			Sc=5.89283, min distance = 0.897247
CMTD1_HUMAN I	439176 Me	MTR1_RHOSH Fu	34756 Acy	1NW5	2AVD	1EG2		0.91	Sc=6.29327, min distance = 2.006590
CMTD1_HUMAN I	445971 CI	COMT_RAT Full	34756 Acy	2CL5	2AVD	1H1D		0.92	Sc=6.5628, min distance = 2.3978951
CNGK1_RHILO I	155448 B-	GLPG_ECOLI Fu	444279 1s	2IRV	3BEH	3B44			Sc=6.29259, min distance = 1.237537
CNGK1_RHILO I	656964 1y	PDE4D_HUMAN Fı	6076 Cycl	2PW3	1VP6	1Y2C			Sc=5.97637, min distance = 2.628012
CNGK1_RHILO I	656966 1y	PDE4D_HUMAN Fı	6076 Cycl	2PW3	1VP6	1Y2E			Sc=6.10705, min distance = 2.585125
CNGK1_RHILO I	6858240 C	KAP0_BOVIN Fu	24316 cyc	1RL3	3CL1	1NE6	31.37	0.79	Sc=6.30399, min distance = 1.979080
CNGK1_RHILO I	6858240 C	KAP0_BOVIN Fu	6076 Cycl	1RGS	1VP6	1NE6	31.37	0.98	Sc=6.28665, min distance = 1.960377
COAA_ECOLI Fı	11957393	FAK1_HUMAN Fu	6022 Aden	1MP8	1SQ5	2ETM			Sc=6.34563, min distance = 2.420500
COAA_ECOLI Fı	16750062	CSK2A_MAIZE Fı	33113 gan	1LP4	1ESN	2OXD			Sc=5.75732, min distance = 2.178017
COAA_ECOLI Fı	23653515	CDK2_HUMAN Fu	6022 Aden	1GY3	1SQ5	2R3F			Sc=6.08092, min distance = 1.997691
COAA_ECOLI Fı	23653516	CDK2_HUMAN Fu	6022 Aden	1GY3	1SQ5	2R3G			Sc=6.2963, min distance = 1.6213802
COAA_ECOLI Fı	23653518	CDK2_HUMAN Fu	6022 Aden	1GY3	1SQ5	2R3J			Sc=6.26075, min distance = 1.994912
COAA_ECOLI Fı	23653519	CDK2_HUMAN Fu	6022 Aden	1GY3	1SQ5	2R3K			Sc=6.26593, min distance = 1.916844
COAA_ECOLI Fı	23653521	CDK2_HUMAN Fu	6022 Aden	1GY3	1SQ5	2R3M			Sc=6.44509, min distance = 1.573659
COAA_ECOLI Fı	23653524	CDK2_HUMAN Fu	6022 Aden	1GY3	1SQ5	2R3P			Sc=6.34295, min distance = 1.489680
COAA_ECOLI Fı	24779678	CSK2A_MAIZE Fı	33113 gan	1LP4	1ESN	2PVM			Sc=6.49548, min distance = 2.148893
COAA_ECOLI Fı	2608 1jsv	CDK2_HUMAN Fu	6022 Aden	1GY3	1SQ5	1JSV			Sc=6.0378, min distance = 1.8834107
COAA_ECOLI Fı	444564 AI	BIOD_ECOLI Fu	6022 Aden	1DAD	1SQ5	1BS1		0.91	Sc=6.45576, min distance = 2.359163
COAA_ECOLI Fı	444564 AI	MYS2_DICDI Fu	33113 gan	1MMN	1ESN	1W9I		0.91	Sc=6.43359, min distance = 2.584163
COAA_ECOLI Fı	444842 CI	CDK2_HUMAN Fu	6022 Aden	1GY3	1SQ5	1CKP			Sc=5.60314, min distance = 2.099134
COAA_ECOLI Fı	445940 O6	CDK2_HUMAN Fu	6022 Aden	1GY3	1SQ5	1GZ8			Sc=6.2103, min distance = 1.4020716
COAA_ECOLI Fı	447004 CI	MYS2_DICDI Fu	33113 gan	1MMN	1ESN	1LVK			Sc=6.17503, min distance = 2.668502
COAA_ECOLI Fı	447052 CI	CSK2A_MAIZE Fı	33113 gan	1LP4	1ESN	1M2R			Sc=6.34402, min distance = 2.233442
COAA_ECOLI Fı	447649 1c	CDK2_HUMAN Fu	6022 Aden	1GY3	1SQ5	1OI9			Sc=6.45113, min distance = 2.246120
COAA_ECOLI Fı	447656 1c	CDK2_HUMAN Fu	6022 Aden	1GY3	1SQ5	1OIY			Sc=6.52095, min distance = 2.525345

COAA_ECOLI F	447955 1p	CDK2_HUMAN Fu	6022 Aden	1GY3	1SQ5	1PXI			Sc=5.76353, min distance = 2.09198
COAA_ECOLI F	449087 1v	CDK2_HUMAN Fu	6022 Aden	1GY3	1SQ5	1VYW			Sc=6.25497, min distance = 1.79320
COAA_ECOLI F	4565 1h1r	CDK2_HUMAN Fu	6022 Aden	1GY3	1SQ5	1H1R			Sc=6.38271, min distance = 2.30001
COAA_ECOLI F	5149739 2	CSK2A_MAIZE F	33113 gam	1LP4	1ESN	2OXY			Sc=5.62853, min distance = 2.25881
COAA_ECOLI F	5326976 1	CSK2A_MAIZE F	33113 gam	1LP4	1ESN	1ZOE			Sc=5.79075, min distance = 2.39826
COAA_ECOLI F	5326978 1	CSK2A_MAIZE F	33113 gam	1LP4	1ESN	1ZOH			Sc=5.69243, min distance = 2.13406
COAA_ECOLI F	5327087 3	CDK2_HUMAN Fu	6022 Aden	1GY3	1SQ5	2BPM			Sc=6.40287, min distance = 1.42877
COAA_ECOLI F	5327130 C	CDK2_HUMAN Fu	6022 Aden	1GY3	1SQ5	2C68			Sc=6.25618, min distance = 1.81156
COAA_ECOLI F	5327133 C	CDK2_HUMAN Fu	6022 Aden	1GY3	1SQ5	2C6K			Sc=6.36662, min distance = 1.81929
COAA_ECOLI F	5327135 D	CDK2_HUMAN Fu	6022 Aden	1GY3	1SQ5	2C6M			Sc=6.28322, min distance = 1.65738
COAA_ECOLI F	5957 Aden	BIOD_ECOLI Fu	6022 Aden	1DAD	1SQ5	1A82		0.99	Sc=6.50178, min distance = 2.34776
COAA_ECOLI F	5957 Aden	HSLU_ECOLI Fu	6022 Aden	1HQY	1SQ5	1DO0		0.99	Sc=6.5136, min distance = 1.285381
COAA_ECOLI F	5957 Aden	MALK_ECOLI Fu	6022 Aden	2AWN	1SQ5	1Q12		0.99	Sc=6.01753, min distance = 1.97108
COAA_ECOLI F	5957 Aden	RK_BOVIN Full	6022 Aden	3C4Z	1SQ5	3C4W		0.99	Sc=6.49041, min distance = 2.04447
COAA_ECOLI F	6031 Uric	DCK_HUMAN Ful	6022 Aden	1P5Z	1SQ5	2ZIA			Sc=5.90774, min distance = 2.03654
COAA_ECOLI F	60961 ade	SKY1_YEAST Fu	33113 gam	1Q99	1ESN	1Q97		0.93	Sc=5.84541, min distance = 2.31562
COAA_ECOLI F	60961 ade	SKY1_YEAST Fu	6022 Aden	1Q8Y	1SQ5	1Q97		0.95	Sc=6.28168, min distance = 2.23039
COAA_ECOLI F	6132 Cyti	ECX2_PYRAB Fu	6022 Aden	2PO0	1SQ5	2PO2			Sc=5.83741, min distance = 2.48972
COAA_ECOLI F	6338561 C	MYS2_DICDI Fu	33113 gam	1MMN	1ESN	1D0X			Sc=6.15819, min distance = 2.36102
COAA_ECOLI F	6338562 C	MYS2_DICDI Fu	33113 gam	1MMN	1ESN	1D0Y			Sc=6.22685, min distance = 2.20755
COAA_ECOLI F	6420139 C	CDK2_HUMAN Fu	6022 Aden	1GY3	1SQ5	2C5V			Sc=6.30344, min distance = 1.19197
COAA_ECOLI F	6540255 C	DAPK1_HUMAN F	33113 gam	1JKL	1ESN	1WVX			Sc=6.28733, min distance = 2.55136
COAA_ECOLI F	656971 CI	CDK2_HUMAN Fu	6022 Aden	1GY3	1SQ5	1Y8Y			Sc=6.01199, min distance = 1.65311
COAA_ECOLI F	91532 AMP	BIOD_ECOLI Fu	6022 Aden	1DAD	1SQ5	1DAG		0.99	Sc=6.52118, min distance = 2.14600
COAA_ECOLI F	91532 AMP	KIF1A_MOUSE F	33113 gam	1VFV	1ESN	1I6I		0.99	Sc=6.49881, min distance = 2.17674
COAA_ECOLI F	91532 AMP	PURT_ECOLI Fu	33113 gam	1EYZ	1ESN	1KJI		0.99	Sc=6.49881, min distance = 2.03394
COABC_ECOLI	101609 Ur	RNAS1_BOVIN F	6131 cyti	1RNN	1U80	1O0M		0.83	Sc=6.2947, min distance = 1.814749
COABC_ECOLI	656946 CI	RNAS1_BOVIN F	6131 cyti	1RNN	1U80	1W4Q			Sc=6.2737, min distance = 1.765557
COAE_ECOLI F	445966 O6	CDK2_HUMAN Fu	6022 Aden	1GY3	1VHL	1H0V			Sc=5.92645, min distance = 2.32387
COAE_ECOLI F	447955 1p	CDK2_HUMAN Fu	6022 Aden	1GY3	1VHL	1PXI			Sc=5.67031, min distance = 1.96904
COAE_ECOLI F	6031 Uric	DCK_HUMAN Ful	6022 Aden	1P5Z	1VHL	2ZIA			Sc=5.68955, min distance = 2.24181
COAE_ECOLI F	60961 ade	STK6_HUMAN Fu	6022 Aden	1MQ4	1VHL	1MUO		0.95	Sc=6.14726, min distance = 2.07111
COAE_HAEIN F	444852 CI	ATPA1_BOVIN F	5957 Aden	2V7Q	1JJV	1COW		0.91	Sc=6.0312, min distance = 2.270152
COAE_HAEIN F	447004 CI	MYS2_DICDI Fu	5957 Aden	1FMW	1JJV	1LVK			Sc=5.75019, min distance = 0
COAE_HAEIN F	6338561 C	MYS2_DICDI Fu	5957 Aden	1FMW	1JJV	1D0X			Sc=6.15821, min distance = 0
COAE_HAEIN F	6338562 C	MYS2_DICDI Fu	5957 Aden	1FMW	1JJV	1D0Y			Sc=6.15821, min distance = 0
COAE_THEMA F	4564 1e1v	CDK2_HUMAN Fu	6022 Aden	1GY3	2GRJ	1E1V			Sc=5.94644, min distance = 1.76815

COAE_THEMA F	91532 AMP	NIFH1_AZOVI F	6022 Aden	1FP6	2GRJ	2AFK		0.99	Sc=6.04695, min distance = 2.084485
COAE_THET8 F	6022 Ader	Q72H90_THET2 I	5957 Aden	2BEK	1UF9	2BEJ		0.99	Sc=5.97148, min distance = 2.302679
COAX_THEMA F	1540 1h1q	CDK2_HUMAN Fu	6022 Aden	1GY3	3BF1	1H1Q			Sc=5.94973, min distance = 2.235757
COAX_THEMA F	23653516	CDK2_HUMAN Fu	6022 Aden	1GY3	3BF1	2R3G			Sc=6.02089, min distance = 2.029637
COAX_THEMA F	24864081	CDK2_HUMAN Fu	6022 Aden	1GY3	3BF1	2VTS			Sc=6.06378, min distance = 2.249189
COAX_THEMA F	2608 1jsv	CDK2_HUMAN Fu	6022 Aden	1GY3	3BF1	1JSV			Sc=5.62052, min distance = 1.962514
COAX_THEMA F	445966 O6	CDK2_HUMAN Fu	6022 Aden	1GY3	3BF1	1H0V			Sc=5.88645, min distance = 1.785734
COAX_THEMA F	447649 1c	CDK2_HUMAN Fu	6022 Aden	1GY3	3BF1	1OI9			Sc=6.06255, min distance = 1.809736
COAX_THEMA F	447654 CI	CDK2_HUMAN Fu	6022 Aden	1GY3	3BF1	1OIT			Sc=5.81219, min distance = 2.034802
COAX_THEMA F	447655 1c	CDK2_HUMAN Fu	6022 Aden	1GY3	3BF1	1OIU			Sc=5.9516, min distance = 2.0788087
COAX_THEMA F	448043 2g	IPKA_RAT Full	6022 Aden	1L3R	3BF1	1Q8U			Sc=5.68228, min distance = 2.388344
COAX_THEMA F	4564 1e1v	CDK2_HUMAN Fu	6022 Aden	1GY3	3BF1	1E1V			Sc=5.75009, min distance = 1.631650
COAX_THEMA F	4565 1h1r	CDK2_HUMAN Fu	6022 Aden	1GY3	3BF1	1H1R			Sc=5.94448, min distance = 2.070423
COAX_THEMA F	5327134 C	CDK2_HUMAN Fu	6022 Aden	1GY3	3BF1	2C6L			Sc=6.13359, min distance = 1.482359
COAX_THEMA F	5957 Ader	AROK_MYCTU Fu	6022 Aden	2IYV	3BF1	2IYW		0.99	Sc=5.9266, min distance = 2.2962730
COAX_THEMA F	5957 Ader	KTHY_HUMAN Fu	6022 Aden	1NN3	3BF1	1E2Q		0.99	Sc=5.93769, min distance = 2.450888
COAX_THEMA F	5957 Ader	MUTS_ECOLI Fu	6022 Aden	1OH7	3BF1	1W7A		0.99	Sc=6.3787, min distance = 2.1504801
COAX_THEMA F	6914614 p	IPKA_RABIT Fu	6022 Aden	1JBP	3BF1	2F7Z			Sc=6.43226, min distance = 2.186252
COAX_THEMA F	91532 AMP	KIF1A_MOUSE F	6022 Aden	1I5S	3BF1	1I6I		0.99	Sc=5.93527, min distance = 1.989056
COAX_THEMA F	9547890 I	CDK2_HUMAN Fu	6022 Aden	1GY3	3BF1	1W8C			Sc=5.74912, min distance = 1.932933
COBU_SALTY F	444699 1b	HPRT_HUMAN Fu	6804 guan	1HMP	1C9K	1BZY			Sc=5.94886, min distance = 2.086701
COBU_SALTY F	73323 Xar	HGXR_TOXGO Fu	6804 guan	1QK3	1C9K	1QK5		0.95	Sc=5.62803, min distance = 1.615291
COFD_METMA F	188347 pp	ARL5A_HUMAN F	8977 1dar	2H17	3C3E	1ZJ6		0.99	Sc=6.54615, min distance = 2.032168
COFD_METMA F	24978488	TBB2B_BOVIN F	8977 1dar	1Z2B	3C3E	3DU7			Sc=6.50825, min distance = 2.068590
COFD_METMA F	37792 gam	GNAT1_BOVIN F	8977 1dar	1TAD	3C3E	1TND		0.99	Sc=6.53076, min distance = 0.722302
COFD_METMA F	37792 gam	RHOA_HUMAN Fu	8977 1dar	1TX4	3C3E	1CXZ		0.99	Sc=6.52496, min distance = 1.612414
COFD_METMA F	444148 1a	RHA1_ARATH Fu	8977 1dar	2EFC	3C3E	2EFE		0.99	Sc=6.48902, min distance = 1.886463
COFD_METMA F	444845 1c	RASH_HUMAN Fu	8977 1dar	2CE2	3C3E	1CLU		0.97	Sc=6.12624, min distance = 2.131379
COFD_METMA F	445463 B-	TGM3_HUMAN Fu	8977 1dar	1VJJ	3C3E	1L9N			Sc=6.25749, min distance = 1.460632
COFD_METMA F	447979 5G	KGUA_ECOLI Fu	8977 1dar	2AN9	3C3E	2ANB			Sc=6.45361, min distance = 2.073152
COFD_METMA F	448405 CI	ARF1_RAT Full	8977 1dar	1RRG	3C3E	1RRF			Sc=6.50154, min distance = 1.674505
COFD_METMA F	6022 Ader	PARM_ECOLX Fu	8977 1dar	2ZGY	3C3E	1MWM		0.8	Sc=6.49017, min distance = 1.732940
COFD_METMA F	6804 guar	EF1A_YEAST Fu	8977 1dar	2B7B	3C3E	1G7C		0.99	Sc=6.41728, min distance = 2.356667
COFD_METMA F	6804 guar	TGM3_HUMAN Fu	8977 1dar	1VJJ	3C3E	1SGX		0.99	Sc=6.46187, min distance = 2.195112
COFD_METMA F	8582 Inos	PURA1_MOUSE F	8977 1dar	1LON	3C3E	1IWE		0.99	Sc=6.40916, min distance = 2.093174
COFD_METMA F	93082 Gab	CDC42_HUMAN F	8977 1dar	2NGR	3C3E	2ODB		0.99	Sc=6.56476, min distance = 1.310429
COFD_METMA F	93082 Gab	RAC3_HUMAN Fu	8977 1dar	2C2H	3C3E	2QME		0.99	Sc=6.11448, min distance = 1.086407

COFD_METMA F	93082 Gab	RASH_HUMAN Fu	8977 1dar	2CE2	3C3E	121P		0.99	Sc=6.11714, min distance = 1.41427
COFE_ARCFU F	24978488	TBB2B_BOVIN F	8977 1dar	1Z2B	2PHN	3DU7			Sc=6.50046, min distance = 1.39878
COFE_ARCFU F	444148 1a	RHA1_ARATH Fu	8977 1dar	2EFC	2PHN	2EFE		0.99	Sc=6.48679, min distance = 2.41878
COFE_ARCFU F	447979 5G	KGUA_ECOLI Fu	8977 1dar	2AN9	2PHN	2ANB			Sc=6.43349, min distance = 1.90021
COFE_ARCFU F	448405 CI	ARF1_RAT Full	8977 1dar	1RRG	2PHN	1RRF			Sc=6.49936, min distance = 2.06214
COFE_ARCFU F	6022 Aden	O33839_THEMA	8977 1dar	1XJE	2PHN	1XJK		0.8	Sc=6.48738, min distance = 1.69924
COFE_ARCFU F	6022 Aden	PARM_ECOLX Fu	8977 1dar	2ZGY	2PHN	1MWM		0.8	Sc=6.47927, min distance = 1.93936
COFE_ARCFU F	8582 Inos	PURA1_MOUSE F	8977 1dar	1LON	2PHN	1IWE		0.99	Sc=6.39681, min distance = 2.06105
COFI_SCHPO F	7054 Isat	AOFB_HUMAN Fu	444279 1s	1OJD	2I2Q	1OJA			Sc=5.68288, min distance = 2.07774
COL12_MOUSE	24808489	IGF1R_HUMAN F	439554 fu	1IGR	2OX9	2ZM3			Sc=6.23967, min distance = 5.07006
COL12_MOUSE	446996 GA	Q9HYN5_PSEAE	439353 be	1W8F	2OX9	1W8H		0.89	Sc=5.77993, min distance = 1.45651
COMB_THET8 F	23831 HEE	DEF_LEPIN Ful	78165 MES	1VEV	2Z0J	1VEY		0.81	Sc=5.60285, min distance = 1.34906
COMT1_MEDSA	70164 Met	XYNY_CLOTM Fu	445858 fe	1GKL	1KYZ	1WB5		0.84	Sc=5.69787, min distance = 2.09193
COMT_HUMAN F	4369234 C	COMT_RAT Full	34756 Acy	2CL5	3BWY	1JR4			Sc=6.72202, min distance = 2.54167
COMT_HUMAN F	445971 CI	COMT_RAT Full	34756 Acy	2CL5	3BWY	1H1D		0.92	Sc=6.60062, min distance = 2.39795
COMT_RAT Ful	439176 Me	MTR1_RHOSH Fu	34756 Acy	1NW5	2CL5	1EG2		0.91	Sc=6.33651, min distance = 2.12395
CONA_CANEN F	126154 Cy	Q54276_SERMA	24139 ace	1UR9	1TEI	1W1P			Sc=5.7972, min distance = 0.654319
CONA_CANEN F	16741013	CARP_YEAST Fu	185698 al	1DPJ	1CVN	1FQ6			Sc=5.64084, min distance = 1.85514
CONA_CANEN F	439554 fu	Q9HYN5_PSEAE	101798 Me	2JDN	5CNA	1UZV		0.96	Sc=5.673, min distance = 2.4990098
CONA_CANEN F	446972 Mu	LECB_LATOC Fu	64947 alp	1LOA	1GIC	1LOD	44.78		Sc=6.10978, min distance = 2.25881
CONA_CANEN F	6102754 2	Q9HYN5_PSEAE	101798 Me	2JDN	5CNA	2BOJ		0.94	Sc=5.60997, min distance = 1.99825
CONA_CANEN F	656981 CU	LYSC_CHICK Fu	24139 ace	1LZB	1TEI	1YIL			Sc=6.10236, min distance = 0.49500
CONA_CANEN F	84265 1ax	LECA_ARTIN Fu	101798 Me	1WS5	5CNA	1M26			Sc=5.99738, min distance = 2.08959
CONA_CANGL F	446578 1r	Q9HYN5_PSEAE	185698 al	1OVS	2OVU	2JDP		0.94	Sc=5.64961, min distance = 1.89250
CONA_CANGL F	446972 Mu	LEC1_LATOC Fu	101798 Me	1LOB	2OVU	1LOD	45.45		Sc=6.15741, min distance = 1.98675
CONA_CANGL F	84265 1ax	LECA_ARTIN Fu	101798 Me	1WS5	2OVU	1M26			Sc=6.01715, min distance = 2.06333
CONA_CANMR F	151504 D-	CONA_CANEN Fu	101798 Me	5CNA	2OW4	1I3H			Sc=5.77106, min distance = 2.12799
CONA_CANMR F	445948 D-	Q9HYN5_PSEAE	101798 Me	2JDN	2OW4	1OXC			Sc=5.67761, min distance = 2.44911
CONA_CANMR F	446578 1r	Q9HYN5_PSEAE	185698 al	1OVS	2OW4	2JDP		0.94	Sc=5.64961, min distance = 1.88211
CONA_CANMR F	6102754 2	Q9HYN5_PSEAE	101798 Me	2JDN	2OW4	2BOJ		0.94	Sc=5.6204, min distance = 2.010971
CONA_CANMR F	84265 1ax	LECA_ARTIN Fu	101798 Me	1WS5	2OW4	1M26			Sc=6.01715, min distance = 2.07409
COTI_BACSU F	10041129	SAHH_MYCTU Fu	60961 ade	3CE6	2Q83	3DHY		0.89	Sc=6.00907, min distance = 2.32177
COX1B_PARDE	155448 B-	GLPG_ECOLI Fu	444279 1s	2IRV	1AR1	3B44			Sc=6.26739, min distance = 0.89631
COX1B_PARDE	445070 fa	AOFB_HUMAN Fu	444279 1s	1OJD	1AR1	2BK3			Sc=5.6917, min distance = 1.878173
COX1_THET8 F	11957363	HBB_HUMAN Ful	444124 HE	1NQP	1EHK	1RQA		0.81	Sc=6.81963, min distance = 2.12835
COX1_THET8 F	11957364	HMOX1_HUMAN F	444124 HE	1OZW	1EHK	1S13		0.86	Sc=6.73944, min distance = 1.94501
COX1_THET8 F	11957370	HMOX1_HUMAN F	444124 HE	1OZW	1EHK	1TWN			Sc=6.68596, min distance = 1.02916

COX1_THET8 F	11957373	CATE_ECOLI Fu	444124 HE	1IPH	1EHK	1P81		0.79	Sc=6.81575, min distance = 1.94642
COX1_THET8 F	11957385	MYG_PHYCA Ful	444098 HE	1A6M	2QPD	2CMM			Sc=6.21777, min distance = 2.06682
COX1_THET8 F	11957385	MYG_PHYCA Ful	444207 He	1VXG	2QPE	2CMM			Sc=6.34959, min distance = 2.10582
COX1_THET8 F	11970219	CCPR_YEAST Fu	444098 HE	2EUT	2QPD	1BEM		0.83	Sc=6.80041, min distance = 2.03741
COX1_THET8 F	16214774	CP51_MYCTU Fu	444124 HE	2CIB	1EHK	2CI0		0.95	Sc=6.69063, min distance = 2.21872
COX1_THET8 F	16741061	MYG_PHYCA Ful	444098 HE	1A6M	2QPD	1MBC		0.83	Sc=6.71505, min distance = 2.03317
COX1_THET8 F	16741183	CPXA_PSEPU Fu	444207 He	1PHC	2QPE	2ZAW		0.93	Sc=6.681, min distance = 2.0993801
COX1_THET8 F	4369122 C	GLB1_LUCPE Fu	444098 HE	1FLP	2QPD	1B0B		0.96	Sc=6.76178, min distance = 2.01523
COX1_THET8 F	4369136 C	GLB_APLLI Ful	444207 He	3MBA	2QPE	1DM1		0.89	Sc=6.80596, min distance = 2.03944
COX1_THET8 F	444097 He	MYG_PHYCA Ful	444207 He	1VXG	2QPE	2EVK		0.96	Sc=6.81164, min distance = 2.08708
COX1_THET8 F	444279 1s	GLPG_ECOLI Fu	155448 B-	3B44	1EHK	2IRV			Sc=5.83138, min distance = 1.25750
COX1_THET8 F	444522 HE	HBA_HORSE Ful	444207 He	2ZLW	2QPE	1Y8K	1		Sc=6.81831, min distance = 2.06123
COX1_THET8 F	444522 HE	MYG_PHYCA Ful	444207 He	1VXG	2QPE	1VXD	1		Sc=6.78388, min distance = 2.04024
COX1_THET8 F	444668 CI	MYG_PHYCA Ful	444098 HE	1A6M	2QPD	1BVD		0.8	Sc=6.35309, min distance = 2.19694
COX1_THET8 F	444668 CI	MYG_PHYCA Ful	444124 HE	1U7R	1EHK	1BVD		0.84	Sc=6.84259, min distance = 2.23712
COX1_THET8 F	444668 CI	MYG_PHYCA Ful	444207 He	1VXG	2QPE	1BVD		0.84	Sc=6.0885, min distance = 2.2650328
COX1_THET8 F	446189 CI	HBG1_HUMAN Fu	444124 HE	1I3E	1EHK	1I3D		0.89	Sc=6.82337, min distance = 2.12135
COX1_THETH F	11957353	HBA_HORSE Ful	444098 HE	2D5X	1XME	1IWH		0.83	Sc=6.81831, min distance = 2.11743
COX1_THETH F	11957363	HBB_HUMAN Ful	444098 HE	1J40	1XME	1RQA		0.77	Sc=6.82312, min distance = 1.91985
COX1_THETH F	11957385	MYG_PHYCA Ful	444098 HE	1A6M	1XME	2CMM			Sc=6.35124, min distance = 2.19521
COX1_THETH F	16741183	CPXA_PSEPU Fu	444098 HE	1YRC	1XME	2ZAW		0.97	Sc=6.68337, min distance = 2.06927
COX1_THETH F	23624229	COX1_THET8 Fu	444098 HE	2QPD	1XME	2QPE			Sc=6.89809, min distance = 2.06833
COX1_THETH F	23624229	COX1_THET8 Fu	445348 HA	1EHK	1XME	2QPE		0.84	Sc=6.89178, min distance = 2.26367
COX1_THETH F	4369122 C	GLB1_LUCPE Fu	444098 HE	1FLP	1XME	1B0B		0.96	Sc=6.81438, min distance = 2.10396
COX2_PARDE F	155448 B-	GLPG_ECOLI Fu	444279 1s	2IRV	1AR1	3B44			Sc=5.85005, min distance = 1.62059
CP119_SULAC	11957370	HMOX1_HUMAN F	444124 HE	1OZW	1IO7	1TWN			Sc=6.66617, min distance = 2.08595
CP119_SULAC	11957371	HMOX1_HUMAN F	444124 HE	1OZW	1IO7	1TWR			Sc=6.67921, min distance = 2.15540
CP119_SULAC	11957385	MYG_PHYCA Ful	444124 HE	1U7R	1IO7	2CMM			Sc=6.31899, min distance = 2.27059
CP119_SULAC	444207 He	CP119_SULTO F	444124 HE	3B4X	1IO7	1UE8		0.99	Sc=6.80596, min distance = 2.37602
CP119_SULAC	444522 HE	NOS3_BOVIN Fu	444124 HE	1ZZS	1IO7	2HX2		0.99	Sc=6.76983, min distance = 2.47860
CP119_SULAC	444522 HE	NOSO_BACSU Fu	444124 HE	2AN0	1IO7	2FC1		0.99	Sc=6.76456, min distance = 2.01274
CP119_SULAC	446406 CZ	MYG_PHYCA Ful	444124 HE	1U7R	1IO7	1J3F			Sc=6.38773, min distance = 2.10092
CP119_SULAC	5326592 C	HMOX1_RAT Ful	444124 HE	1J02	1IO7	1J2C		0.84	Sc=6.75192, min distance = 2.03850
CP119_SULTO	11957370	HMOX1_HUMAN F	444124 HE	1OZW	3B4X	1TWN			Sc=6.64877, min distance = 2.18513
CP119_SULTO	11957371	HMOX1_HUMAN F	444124 HE	1OZW	3B4X	1TWR			Sc=6.6712, min distance = 1.8733387
CP119_SULTO	11957385	MYG_PHYCA Ful	444124 HE	1U7R	3B4X	2CMM			Sc=6.28942, min distance = 2.46115
CP119_SULTO	11957385	MYG_PHYCA Ful	444207 He	1VXG	1UE8	2CMM			Sc=6.32264, min distance = 2.02278

CP119_SULTO I	16214774	CP51_MYCTU Ful	444124 HE	2CIB	3B4X	2CI0	0.95	Sc=6.67065, min distance = 2.37961
CP119_SULTO I	444522 HE	CP2C9_HUMAN Fu	444124 HE	1OG2	3B4X	1R9O	0.99	Sc=6.78803, min distance = 2.25074
CP119_SULTO I	444522 HE	CPXA_PSEPU Ful	444207 He	1PHC	1UE8	1QMQ	1	Sc=6.78763, min distance = 2.30526
CP119_SULTO I	444522 HE	HBA_HORSE Full	444207 He	2ZLW	1UE8	1Y8K	1	Sc=6.81771, min distance = 1.65034
CP119_SULTO I	444522 HE	NOS3_BOVIN Ful	444124 HE	1ZZS	3B4X	2HX2	0.99	Sc=6.74967, min distance = 2.29784
CP119_SULTO I	446406 CZ	MYG_PHYCA Full	444124 HE	1U7R	3B4X	1J3F		Sc=6.39617, min distance = 2.17573
CP120_SYNY3 I	11957353	HBB_HORSE Full	444098 HE	2D5X	2VE3	1IWH	0.83	Sc=6.79297, min distance = 2.10612
CP120_SYNY3 I	11957360	CY1_BOVIN Full	444098 HE	1L0N	2VE3	1NTM		Sc=6.80469, min distance = 2.17934
CP120_SYNY3 I	11957363	HBB_HUMAN Full	444098 HE	1J40	2VE3	1RQA	0.77	Sc=6.79473, min distance = 2.25915
CP120_SYNY3 I	11957385	MYG_PHYCA Full	444098 HE	1A6M	2VE3	2CMM		Sc=6.29556, min distance = 2.71760
CP130_MYCTU I	11957353	HBB_HORSE Full	444124 HE	1Y8I	2UVN	1IWH	0.87	Sc=6.76792, min distance = 2.57707
CP130_MYCTU I	11957355	CY1_BOVIN Full	444098 HE	1L0N	2UUQ	1L0L	0.77	Sc=6.76582, min distance = 2.19148
CP130_MYCTU I	11957360	CY1_BOVIN Full	444098 HE	1L0N	2UUQ	1NTM		Sc=6.80337, min distance = 2.19908
CP130_MYCTU I	11957361	CY1_BOVIN Full	444098 HE	1L0N	2UUQ	1NTZ	0.8	Sc=6.7649, min distance = 2.355903
CP130_MYCTU I	11957370	HMOX1_HUMAN Fu	444124 HE	1OZW	2UVN	1TWN		Sc=6.6173, min distance = 2.301620
CP130_MYCTU I	11957371	HMOX1_HUMAN Fu	444124 HE	1OZW	2UVN	1TWR		Sc=6.66089, min distance = 2.09369
CP130_MYCTU I	11957385	MYG_PHYCA Full	444098 HE	1A6M	2UUQ	2CMM		Sc=6.30099, min distance = 2.17922
CP130_MYCTU I	11957385	MYG_PHYCA Full	444124 HE	1U7R	2UVN	2CMM		Sc=6.16592, min distance = 2.72964
CP130_MYCTU I	16214774	CP51_MYCTU Ful	444124 HE	2CIB	2UVN	2CI0	0.95	Sc=6.65227, min distance = 2.69458
CP130_MYCTU I	16214831	CP3A4_HUMAN Fu	444098 HE	1W0F	2UUQ	2V0M		Sc=5.94252, min distance = 1.77432
CP130_MYCTU I	444207 He	CP119_SULTO Fu	444124 HE	3B4X	2UVN	1UE8	0.99	Sc=6.77121, min distance = 2.50079
CP130_MYCTU I	444522 HE	HBA_HORSE Full	444124 HE	1Y8I	2UVN	1Y8K	0.99	Sc=6.78275, min distance = 2.42176
CP130_MYCTU I	444522 HE	HBA_PAGBE Full	444124 HE	1S5X	2UVN	1PBX	0.99	Sc=6.77026, min distance = 2.00927
CP130_MYCTU I	444522 HE	NOS3_BOVIN Ful	444124 HE	1ZZS	2UVN	2HX2	0.99	Sc=6.73414, min distance = 2.40649
CP130_MYCTU I	5326592 C	HMOX1_RAT Full	444124 HE	1J02	2UVN	1J2C	0.84	Sc=6.71992, min distance = 2.16258
CP1A2_HUMAN I	11957330	CCPR_YEAST Ful	444522 HE	3E2O	2HI4	1BEQ	0.84	Sc=6.79908, min distance = 2.08358
CP1A2_HUMAN I	11957355	CYB_BOVIN Full	444522 HE	1BGY	2HI4	1L0L	0.81	Sc=6.79036, min distance = 2.20273
CP1A2_HUMAN I	11957363	HBA_HUMAN Full	444522 HE	2DN1	2HI4	1RQA	0.81	Sc=6.80971, min distance = 2.43126
CP1A2_HUMAN I	11957385	MYG_PHYCA Full	444522 HE	1VXD	2HI4	2CMM		Sc=6.29896, min distance = 2.36903
CP1A2_HUMAN I	11970220	CCPR_YEAST Ful	444522 HE	3E2O	2HI4	1BEP	0.83	Sc=6.80658, min distance = 2.13499
CP1A2_HUMAN I	11970221	CCPR_YEAST Ful	444522 HE	3E2O	2HI4	1BES	0.87	Sc=6.7636, min distance = 2.048187
CP1A2_HUMAN I	11970222	CCPR_YEAST Ful	444522 HE	3E2O	2HI4	1BJ9	0.81	Sc=6.85121, min distance = 1.99929
CP1A2_HUMAN I	11970242	CCPR_YEAST Ful	444522 HE	3E2O	2HI4	1CPE	0.87	Sc=6.80007, min distance = 2.11583
CP1A2_HUMAN I	4369228 C	PER_COPCI Full	444522 HE	1H3J	2HI4	1LYC	0.96	Sc=6.8053, min distance = 2.113296
CP1A2_HUMAN I	444124 HE	PER_COPCI Full	444522 HE	1H3J	2HI4	1LY9	0.99	Sc=6.80363, min distance = 2.12879
CP1A2_HUMAN I	444124 HE	Q83WG3_9ACTO I	444522 HE	2Z3T	2HI4	2Z3U	0.99	Sc=6.80825, min distance = 2.06753
CP1A2_HUMAN I	444207 He	PER_COPCI Full	444522 HE	1H3J	2HI4	1LY8	1	Sc=6.80337, min distance = 2.05065

CP1A2_HUMAN I	446409 HN	HBA_HUMAN Ful	444522 HE	2DN1	2HI4	1J40	0.98	Sc=6.81003, min distance = 1.909594
CP2A6_HUMAN I	11957370	HMOX1_HUMAN Fu	444124 HE	1OZW	1Z10	1TWN		Sc=6.64839, min distance = 2.082930
CP2A6_HUMAN I	11957385	MYG_PHYCA Ful	444124 HE	1U7R	1Z10	2CMM		Sc=6.28364, min distance = 2.407094
CP2A6_HUMAN I	11957385	MYG_PHYCA Ful	444522 HE	1VXD	2FDV	2CMM		Sc=6.30925, min distance = 2.002167
CP2A6_HUMAN I	122783 8-	Q88NF7_PSEPK I	323 couma	2H90	1Z10	2H8Z	0.86	Sc=5.78959, min distance = 0.831570
CP2A6_HUMAN I	444207 He	COX1_THET8 Fu	444124 HE	1EHK	1Z10	2QPE	0.99	Sc=6.81798, min distance = 2.040070
CP2A6_HUMAN I	444207 He	CP119_SULTO Fu	444124 HE	3B4X	1Z10	1UE8	0.99	Sc=6.81363, min distance = 2.079454
CP2A6_HUMAN I	444207 He	HBB_HORSE Ful	444124 HE	1Y8I	1Z10	2ZLW	0.99	Sc=6.81798, min distance = 1.815271
CP2A6_HUMAN I	446406 CZ	MYG_PHYCA Ful	444124 HE	1U7R	1Z10	1J3F		Sc=6.37091, min distance = 2.176016
CP2A6_HUMAN I	446409 HN	HBA_HUMAN Ful	444522 HE	2DN1	2FDV	1J40	0.98	Sc=6.80791, min distance = 1.922807
CP2A6_HUMAN I	5326592 C	HMOX1_RAT Ful	444124 HE	1J02	1Z10	1J2C	0.84	Sc=6.73699, min distance = 2.365232
CP2AD_HUMAN I	11957370	HMOX1_HUMAN Fu	444124 HE	1OZW	2PG5	1TWN		Sc=6.64215, min distance = 1.902441
CP2AD_HUMAN I	11957371	HMOX1_HUMAN Fu	444124 HE	1OZW	2PG5	1TWR		Sc=6.66899, min distance = 1.751575
CP2AD_HUMAN I	444207 He	COX1_THET8 Fu	444124 HE	1EHK	2PG5	2QPE	0.99	Sc=6.80855, min distance = 2.032402
CP2AD_HUMAN I	444207 He	HBB_HORSE Ful	444124 HE	1Y8I	2PG5	2ZLW	0.99	Sc=6.81333, min distance = 1.786022
CP2AD_HUMAN I	444522 HE	HBA_PAGBE Ful	444124 HE	1S5X	2PG5	1PBX	0.99	Sc=6.80259, min distance = 2.073778
CP2AD_HUMAN I	444522 HE	NOS3_BOVIN Fu	444124 HE	1ZZS	2PG5	2HX2	0.99	Sc=6.79512, min distance = 2.165484
CP2AD_HUMAN I	446406 CZ	MYG_PHYCA Ful	444124 HE	1U7R	2PG5	1J3F		Sc=6.36861, min distance = 2.021663
CP2AD_HUMAN I	5326592 C	HMOX1_RAT Ful	444124 HE	1J02	2PG5	1J2C	0.84	Sc=6.73038, min distance = 2.500734
CP2C8_HUMAN I	11957370	HMOX1_HUMAN Fu	444124 HE	1OZW	1PQ2	1TWN		Sc=6.65305, min distance = 2.036096
CP2C8_HUMAN I	11957371	HMOX1_HUMAN Fu	444124 HE	1OZW	1PQ2	1TWR		Sc=6.67546, min distance = 1.627422
CP2C8_HUMAN I	11957385	MYG_PHYCA Ful	444124 HE	1U7R	1PQ2	2CMM		Sc=6.29556, min distance = 2.081750
CP2C8_HUMAN I	14253 Cet	FABP4_MOUSE Fu	985 palmi	1LIE	1PQ2	1LIC		Sc=5.67935, min distance = 2.093497
CP2C8_HUMAN I	4369228 C	PER_COPCI Ful	444124 HE	1LY9	1PQ2	1LYC	0.95	Sc=6.80662, min distance = 1.967553
CP2C8_HUMAN I	444207 He	PER_COPCI Ful	444124 HE	1LY9	1PQ2	1LY8	0.99	Sc=6.80436, min distance = 1.686255
CP2C8_HUMAN I	444522 HE	HBA_PAGBE Ful	444124 HE	1S5X	1PQ2	1PBX	0.99	Sc=6.79214, min distance = 2.015645
CP2C8_HUMAN I	446406 CZ	MYG_PHYCA Ful	444124 HE	1U7R	1PQ2	1J3F		Sc=6.38415, min distance = 2.145497
CP2C8_HUMAN I	447878 J8	POLG_POL1M Fu	985 palmi	1HXS	1PQ2	1PO1		Sc=6.28557, min distance = 1.572670
CP2C9_HUMAN I	11957330	CCPR_YEAST Fu	444098 HE	2EUT	1OG5	1BEQ	0.81	Sc=6.79908, min distance = 2.116653
CP2C9_HUMAN I	11957353	HBA_HORSE Ful	444124 HE	1Y8I	1OG2	1IWH	0.87	Sc=6.81934, min distance = 1.944997
CP2C9_HUMAN I	11957353	HBB_HORSE Ful	444124 HE	1Y8I	1OG2	1IWH	0.87	Sc=6.81263, min distance = 1.258400
CP2C9_HUMAN I	11957355	CYB_BOVIN Ful	444522 HE	1BGY	1R9O	1L0L	0.81	Sc=6.80099, min distance = 1.999657
CP2C9_HUMAN I	11957360	CYB_BOVIN Ful	444522 HE	1BGY	1R9O	1NTM	0.78	Sc=6.79626, min distance = 1.022521
CP2C9_HUMAN I	11957363	HBA_HUMAN Ful	444522 HE	2DN1	1R9O	1RQA	0.81	Sc=6.80662, min distance = 2.088492
CP2C9_HUMAN I	11957363	HBB_HUMAN Ful	444124 HE	1NQP	1OG2	1RQA	0.81	Sc=6.81196, min distance = 1.881849
CP2C9_HUMAN I	11957370	HMOX1_HUMAN Fu	444124 HE	1OZW	1OG2	1TWN		Sc=6.6616, min distance = 2.1899232
CP2C9_HUMAN I	11957385	MYG_PHYCA Ful	444124 HE	1U7R	1OG2	2CMM		Sc=6.2999, min distance = 2.4192358

CP2C9_HUMAN I	11957385	MYG_PHYCA Ful	444522 HE	1VXD	1R9O	2CMM		Sc=6.31222, min distance = 2.09427
CP2C9_HUMAN I	11970219	CCPR_YEAST Fu	444098 HE	2EUT	1OG5	1BEM	0.83	Sc=6.79973, min distance = 2.35828
CP2C9_HUMAN I	11970219	CCPR_YEAST Fu	444522 HE	3E2O	1R9O	1BEM	0.87	Sc=6.79973, min distance = 2.27765
CP2C9_HUMAN I	11970220	CCPR_YEAST Fu	444098 HE	2EUT	1OG5	1BEP	0.8	Sc=6.80658, min distance = 2.42869
CP2C9_HUMAN I	11970221	CCPR_YEAST Fu	444098 HE	2EUT	1OG5	1BES	0.83	Sc=6.76289, min distance = 2.45855
CP2C9_HUMAN I	11970221	CCPR_YEAST Fu	444522 HE	3E2O	1R9O	1BES	0.87	Sc=6.76289, min distance = 2.29256
CP2C9_HUMAN I	11970222	CCPR_YEAST Fu	444098 HE	2EUT	1OG5	1BJ9	0.77	Sc=6.84196, min distance = 2.16628
CP2C9_HUMAN I	11970222	CCPR_YEAST Fu	444522 HE	3E2O	1R9O	1BJ9	0.81	Sc=6.84508, min distance = 2.37141
CP2C9_HUMAN I	11970242	CCPR_YEAST Fu	444098 HE	2EUT	1OG5	1CPE	0.83	Sc=6.79877, min distance = 2.27175
CP2C9_HUMAN I	11970242	CCPR_YEAST Fu	444522 HE	3E2O	1R9O	1CPE	0.87	Sc=6.80041, min distance = 2.38856
CP2C9_HUMAN I	126994 CI	CCPR_YEAST Fu	444098 HE	2EUT	1OG5	1Z53	0.77	Sc=6.7959, min distance = 2.497944
CP2C9_HUMAN I	126994 CI	CCPR_YEAST Fu	444522 HE	3E2O	1R9O	1Z53	0.81	Sc=6.79973, min distance = 2.22314
CP2C9_HUMAN I	23722944	TTHY_HUMAN Fu	72099 flu	1DVT	1R9O	2QGE		Sc=5.74734, min distance = 2.07943
CP2C9_HUMAN I	25011739	TTHY_HUMAN Fu	72099 flu	1DVT	1R9O	3CN0		Sc=5.74851, min distance = 1.98575
CP2C9_HUMAN I	4369228 C	PER_COPCI Ful	444124 HE	1LY9	1OG2	1LYC	0.95	Sc=6.80662, min distance = 2.33514
CP2C9_HUMAN I	4369228 C	PER_COPCI Ful	444522 HE	1H3J	1R9O	1LYC	0.96	Sc=6.80825, min distance = 2.14603
CP2C9_HUMAN I	444207 He	PER_COPCI Ful	444124 HE	1LY9	1OG2	1LY8	0.99	Sc=6.80662, min distance = 2.31884
CP2C9_HUMAN I	444207 He	PER_COPCI Ful	444522 HE	1H3J	1R9O	1LY8	1	Sc=6.80625, min distance = 2.37307
CP2C9_HUMAN I	445049 pr	PGH2_MOUSE Fu	72099 flu	3PGH	1R9O	1DDX		Sc=6.17186, min distance = 2.47556
CP2C9_HUMAN I	446332 CI	MYG_PHYCA Ful	444124 HE	1U7R	1OG2	1IOP	0.8	Sc=6.69996, min distance = 2.35389
CP2C9_HUMAN I	446406 CZ	MYG_PHYCA Ful	444124 HE	1U7R	1OG2	1J3F		Sc=6.39285, min distance = 2.13823
CP2C9_HUMAN I	446409 HN	HBA_HUMAN Ful	444522 HE	2DN1	1R9O	1J40	0.98	Sc=6.81438, min distance = 1.71690
CP2C9_HUMAN I	447168 ZE	CCPR_YEAST Fu	444098 HE	2EUT	1OG5	1ML2	0.82	Sc=6.82933, min distance = 2.11334
CP2C9_HUMAN I	447168 ZE	CCPR_YEAST Fu	444522 HE	3E2O	1R9O	1ML2	0.86	Sc=6.82758, min distance = 2.22323
CP2C9_HUMAN I	5326592 C	HMOX1_RAT Ful	444098 HE	1DVG	1OG5	1J2C	0.79	Sc=6.73916, min distance = 1.96583
CP2C9_HUMAN I	5326592 C	HMOX1_RAT Ful	444124 HE	1J02	1OG2	1J2C	0.84	Sc=6.74866, min distance = 2.28368
CP2C9_HUMAN I	5327160 2	TTHY_HUMAN Fu	72099 flu	1DVT	1R9O	2F8I		Sc=6.04704, min distance = 2.00705
CP2D6_HUMAN I	11957370	HMOX1_HUMAN Fu	444124 HE	1OZW	2F9Q	1TWN		Sc=6.66617, min distance = 2.26572
CP2D6_HUMAN I	11957373	CATE_ECOLI Fu	444124 HE	1IPH	2F9Q	1P81	0.79	Sc=6.81905, min distance = 2.00101
CP2D6_HUMAN I	11957385	MYG_PHYCA Ful	444124 HE	1U7R	2F9Q	2CMM		Sc=6.30818, min distance = 2.32063
CP2D6_HUMAN I	16214774	CP51_MYCTU Fu	444124 HE	2CIB	2F9Q	2CI0	0.95	Sc=6.68528, min distance = 2.12523
CP2D6_HUMAN I	444207 He	CP119_SULTO Fu	444124 HE	3B4X	2F9Q	1UE8	0.99	Sc=6.81505, min distance = 2.37027
CP2D6_HUMAN I	444207 He	PRXC_CALFU Fu	444124 HE	2CPO	2F9Q	1CPO	0.99	Sc=6.81196, min distance = 1.89719
CP2D6_HUMAN I	444522 HE	CP2C9_HUMAN Fu	444124 HE	1OG2	2F9Q	1R9O	0.99	Sc=6.80697, min distance = 2.48437
CP2D6_HUMAN I	444522 HE	GLB1_GLYDI Fu	444124 HE	1JF3	2F9Q	1HBG	0.99	Sc=6.805, min distance = 2.3171978
CP2D6_HUMAN I	444522 HE	HBA_HORSE Ful	444124 HE	1Y8I	2F9Q	1Y8K	0.99	Sc=6.81741, min distance = 2.12031
CP2D6_HUMAN I	444522 HE	HBA_HUMAN Ful	444124 HE	1NQP	2F9Q	2DN1	0.99	Sc=6.81963, min distance = 2.00276

CP2D6_HUMAN I	446406 CZ	MYG_PHYCA Ful	444124 HE	1U7R	2F9Q	1J3F		Sc=6.3804, min distance = 2.290092
CP2R1_HUMAN I	11957330	CCPR_YEAST Fu	444098 HE	2EUT	3CZH	1BEQ	0.81	Sc=6.79877, min distance = 2.343245
CP2R1_HUMAN I	11957353	HBA_HORSE Ful	444098 HE	2D5X	3CZH	1IWH	0.83	Sc=6.81934, min distance = 2.163514
CP2R1_HUMAN I	11957353	HBB_HORSE Ful	444098 HE	2D5X	3CZH	1IWH	0.83	Sc=6.80971, min distance = 2.420578
CP2R1_HUMAN I	11957360	CY1_BOVIN Ful	444098 HE	1L0N	3CZH	1NTM		Sc=6.82275, min distance = 2.146845
CP2R1_HUMAN I	11957363	HBB_HUMAN Ful	444098 HE	1J40	3CZH	1RQA	0.77	Sc=6.81233, min distance = 2.131292
CP2R1_HUMAN I	11957385	MYG_PHYCA Ful	444098 HE	1A6M	3CZH	2CMM		Sc=6.30925, min distance = 2.423475
CP2R1_HUMAN I	11970219	CCPR_YEAST Fu	444098 HE	2EUT	3CZH	1BEM	0.83	Sc=6.79842, min distance = 2.228812
CP2R1_HUMAN I	11970220	CCPR_YEAST Fu	444098 HE	2EUT	3CZH	1BEP	0.8	Sc=6.80185, min distance = 2.492447
CP2R1_HUMAN I	11970221	CCPR_YEAST Fu	444098 HE	2EUT	3CZH	1BES	0.83	Sc=6.76626, min distance = 2.343052
CP2R1_HUMAN I	11970222	CCPR_YEAST Fu	444098 HE	2EUT	3CZH	1BJ9	0.77	Sc=6.84574, min distance = 2.250851
CP2R1_HUMAN I	11970242	CCPR_YEAST Fu	444098 HE	2EUT	3CZH	1CPE	0.83	Sc=6.80186, min distance = 2.309492
CP2R1_HUMAN I	126994 CI	CCPR_YEAST Fu	444098 HE	2EUT	3CZH	1Z53	0.77	Sc=6.80562, min distance = 2.200201
CP2R1_HUMAN I	446406 CZ	MYG_PHYCA Ful	444098 HE	1A6M	3CZH	1J3F		Sc=6.39685, min distance = 2.155865
CP2R1_HUMAN I	447168 ZE	CCPR_YEAST Fu	444098 HE	2EUT	3CZH	1ML2	0.82	Sc=6.83036, min distance = 2.038014
CP2R1_HUMAN I	5326592 C	HMOX1_RAT Ful	444098 HE	1DVG	3CZH	1J2C	0.79	Sc=6.74364, min distance = 2.002476
CP3A4_HUMAN I	11957353	HBB_HORSE Ful	444124 HE	1Y8I	1TQN	1IWH	0.87	Sc=6.79727, min distance = 2.330891
CP3A4_HUMAN I	11957360	CY1_BOVIN Ful	444098 HE	1L0N	1W0F	1NTM		Sc=6.79473, min distance = 2.242516
CP3A4_HUMAN I	11957370	HMOX1_HUMAN Fu	444124 HE	1OZW	1TQN	1TWN		Sc=6.64428, min distance = 2.159728
CP3A4_HUMAN I	11957371	HMOX1_HUMAN Fu	444124 HE	1OZW	1TQN	1TWR		Sc=6.67642, min distance = 2.025567
CP3A4_HUMAN I	11957385	MYG_PHYCA Ful	444098 HE	1A6M	1W0F	2CMM		Sc=6.27899, min distance = 2.223861
CP3A4_HUMAN I	11957385	MYG_PHYCA Ful	444124 HE	1U7R	1TQN	2CMM		Sc=6.27347, min distance = 2.392201
CP3A4_HUMAN I	16741183	CPXA_PSEPU Fu	444098 HE	1YRC	1W0F	2ZAW	0.97	Sc=6.63511, min distance = 2.110242
CP3A4_HUMAN I	444522 HE	NOS3_BOVIN Fu	444124 HE	1ZZS	1TQN	2HX2	0.99	Sc=6.79113, min distance = 2.113256
CP3A4_HUMAN I	446332 CI	MYG_PHYCA Ful	444124 HE	1U7R	1TQN	1IOP	0.8	Sc=6.66589, min distance = 2.226168
CP3A4_HUMAN I	446406 CZ	MYG_PHYCA Ful	444098 HE	1A6M	1W0F	1J3F		Sc=6.35923, min distance = 2.108467
CP3A4_HUMAN I	446406 CZ	MYG_PHYCA Ful	444124 HE	1U7R	1TQN	1J3F		Sc=6.37409, min distance = 2.273399
CP3A4_HUMAN I	5326592 C	HMOX1_RAT Ful	444124 HE	1J02	1TQN	1J2C	0.84	Sc=6.74038, min distance = 2.026331
CP46A_HUMAN I	11957370	HMOX1_HUMAN Fu	444124 HE	1OZW	2Q9F	1TWN		Sc=6.65018, min distance = 2.125599
CP46A_HUMAN I	11957385	MYG_PHYCA Ful	444124 HE	1U7R	2Q9F	2CMM		Sc=6.18658, min distance = 2.470358
CP46A_HUMAN I	16214774	CP51_MYCTU Fu	444124 HE	2CIB	2Q9F	2CI0	0.95	Sc=6.66871, min distance = 2.373540
CP46A_HUMAN I	444095 HE	RCEL_RHOVI Fu	444124 HE	3D38	2Q9F	1DXR	0.91	Sc=6.79695, min distance = 2.148199
CP46A_HUMAN I	444207 He	CP119_SULTO Fu	444124 HE	3B4X	2Q9F	1UE8	0.99	Sc=6.78719, min distance = 2.560358
CP46A_HUMAN I	444522 HE	CP2C9_HUMAN Fu	444124 HE	1OG2	2Q9F	1R9O	0.99	Sc=6.75557, min distance = 2.125070
CP46A_HUMAN I	446406 CZ	MYG_PHYCA Ful	444124 HE	1U7R	2Q9F	1J3F		Sc=6.38334, min distance = 2.186510
CP51_MYCTU Fu	11957385	MYG_PHYCA Ful	444124 HE	1U7R	2CIB	2CMM		Sc=6.12855, min distance = 2.676615
CP51_MYCTU Fu	444095 HE	RCEL_RHOVI Fu	444124 HE	3D38	2CIB	1DXR	0.91	Sc=6.73812, min distance = 2.517399

CP51_MYCTU Fu	444125 HE	RCEL_RHOVI Ful	444124 HE	3D38	2CIB	2I5N		0.91	Sc=6.37397, min distance = 2.32450
CP51_MYCTU Fu	444207 He	CP119_SULTO Fu	444124 HE	3B4X	2CIB	1UE8		0.99	Sc=6.4335, min distance = 2.732493
CP51_MYCTU Fu	444207 He	HBA_HORSE Ful	444124 HE	1Y8I	2CIB	2ZLW		0.99	Sc=6.43255, min distance = 2.13946
CP51_MYCTU Fu	444207 He	PRXC_CALFU Ful	444124 HE	2CPO	2CIB	1CPO		0.99	Sc=6.43029, min distance = 2.27648
CP51_MYCTU Fu	5326592 C	HMOX1_RAT Ful	444124 HE	1J02	2CIB	1J2C		0.84	Sc=6.32976, min distance = 2.32490
CP51_MYCTU Fu	6420167 M	CPXA_PSEPU Ful	444124 HE	1RE9	2CIB	2FE6		0.89	Sc=6.41044, min distance = 2.20431
CP74A_ARATH	11957363	HBB_HUMAN Ful	444098 HE	1J40	3DSK	1RQA		0.77	Sc=6.78484, min distance = 1.07679
CP74A_ARATH	11957385	MYG_PHYCA Ful	444098 HE	1A6M	3DSK	2CMM			Sc=6.29237, min distance = 2.33869
CP74A_ARATH	4369122 C	GLB1_LUCPE Ful	444098 HE	1FLP	3DSK	1B0B		0.96	Sc=6.7807, min distance = 2.033228
CP74A_ARATH	446406 CZ	MYG_PHYCA Ful	444098 HE	1A6M	3DSK	1J3F			Sc=6.35834, min distance = 2.28153
CP7A1_HUMAN	11957385	MYG_PHYCA Ful	444098 HE	1A6M	3DAX	2CMM			Sc=6.30602, min distance = 2.28991
CP7A1_HUMAN	11970220	CCPR_YEAST Ful	444098 HE	2EUT	3DAX	1BEP		0.8	Sc=6.30441, min distance = 2.70358
CP7A1_HUMAN	447168 ZE	CCPR_YEAST Ful	444098 HE	2EUT	3DAX	1ML2		0.82	Sc=6.3276, min distance = 2.287834
CPKA_PYRFU Fu	447955 1p	CDK2_HUMAN Ful	6022 Aden	1GY3	1E19	1PXI			Sc=5.80053, min distance = 2.21674
CPKA_PYRFU Fu	72194 2-C	ENPL_CANFA Ful	6022 Aden	1TC6	1E19	1QYE		0.89	Sc=6.19888, min distance = 1.91528
CPXA_PSEPU Fu	11957360	CY1_BOVIN Ful	444098 HE	1L0N	1YRC	1NTM			Sc=6.79727, min distance = 2.38095
CPXA_PSEPU Fu	11957361	CY1_BOVIN Ful	444098 HE	1L0N	1YRC	1NTZ		0.8	Sc=6.79473, min distance = 2.29325
CPXA_PSEPU Fu	11957363	HBA_HUMAN Ful	444124 HE	1NQP	1RE9	1RQA		0.81	Sc=6.79512, min distance = 2.17040
CPXA_PSEPU Fu	11957363	HBA_HUMAN Ful	444522 HE	2DN1	1QMQ	1RQA		0.81	Sc=6.809, min distance = 2.2989949
CPXA_PSEPU Fu	11957370	HMOX1_HUMAN Fu	444124 HE	1OZW	1RE9	1TWN			Sc=6.64024, min distance = 1.90748
CPXA_PSEPU Fu	11957371	HMOX1_HUMAN Fu	444124 HE	1OZW	1RE9	1TWR			Sc=6.66899, min distance = 1.86902
CPXA_PSEPU Fu	11957385	MYG_PHYCA Ful	16741185	2EKU	2Z97	2CMM			Sc=6.30925, min distance = 2.21419
CPXA_PSEPU Fu	11957385	MYG_PHYCA Ful	444098 HE	1A6M	1YRC	2CMM			Sc=6.27226, min distance = 2.52872
CPXA_PSEPU Fu	11957385	MYG_PHYCA Ful	444124 HE	1U7R	1RE9	2CMM			Sc=6.17857, min distance = 2.62485
CPXA_PSEPU Fu	11957385	MYG_PHYCA Ful	444207 He	1VXG	1PHC	2CMM			Sc=6.2639, min distance = 2.342600
CPXA_PSEPU Fu	11957385	MYG_PHYCA Ful	444522 HE	1VXD	1QMQ	2CMM			Sc=6.30305, min distance = 2.12902
CPXA_PSEPU Fu	444125 HE	RCEL_RHOVI Ful	444124 HE	3D38	1RE9	2I5N		0.91	Sc=6.78243, min distance = 2.28413
CPXA_PSEPU Fu	446406 CZ	MYG_PHYCA Ful	444124 HE	1U7R	1RE9	1J3F			Sc=6.36452, min distance = 2.38114
CPXB_BACME Fu	11957370	HMOX1_HUMAN Fu	444124 HE	1OZW	1ZO4	1TWN			Sc=6.66485, min distance = 2.10646
CPXB_BACME Fu	11957385	MYG_PHYCA Ful	444098 HE	1A6M	2IJ2	2CMM			Sc=6.32423, min distance = 2.28226
CPXB_BACME Fu	11957385	MYG_PHYCA Ful	444124 HE	1U7R	1ZO4	2CMM			Sc=6.33071, min distance = 2.44104
CPXB_BACME Fu	14253 Cet	FABP4_MOUSE Fu	985 palmi	1LIE	1FAG	1LIC			Sc=5.66377, min distance = 1.84641
CPXB_BACME Fu	16741044	PYRDA_LACLC Fu	444243 FA	1JUB	1BVY	1JRB			Sc=6.24459, min distance = 1.98913
CPXB_BACME Fu	175468 12	LACB_BOVIN Ful	985 palmi	1GXA	1FAG	1BSO		0.83	Sc=5.81392, min distance = 1.79281
CPXB_BACME Fu	444207 He	CP119_SULTO Fu	444124 HE	3B4X	1ZO4	1UE8		0.99	Sc=6.80259, min distance = 2.55332
CPXB_BACME Fu	444522 HE	BFR_ECOLI Ful	444124 HE	1BFR	1ZO4	1BCF		0.99	Sc=6.81505, min distance = 1.83914
CPXB_BACME Fu	446406 CZ	MYG_PHYCA Ful	444098 HE	1A6M	2IJ2	1J3F			Sc=6.39759, min distance = 2.10728

CPXB_BACME Fı	446406 CZ	MYG_PHYCA Ful	444124 HE	1U7R	1ZO4	1J3F			Sc=6.39617, min distance = 2.17050
CPXB_BACME Fı	449551 FM	FLAV_DESVH Fu	444243 FA	1F4P	1BVY	5FX2		0.77	Sc=6.23278, min distance = 2.08185
CPXE_STRGO Fı	11957385	MYG_PHYCA Ful	444098 HE	1A6M	2ZBX	2CMM			Sc=6.31703, min distance = 2.15500
CPXE_STRGO Fı	5326592 C	HMOX1_RAT Ful	444098 HE	1DVG	2ZBX	1J2C		0.79	Sc=6.75011, min distance = 2.25507
CPXJ_SACEN Fı	11957360	CY1_BOVIN Ful	444098 HE	1L0N	1Z8O	1NTM			Sc=6.74927, min distance = 2.10203
CPXJ_SACEN Fı	11957370	HMOX1_HUMAN Fı	444124 HE	1OZW	1JIP	1TWN			Sc=6.60414, min distance = 1.87236
CPXJ_SACEN Fı	11957385	MYG_PHYCA Ful	444098 HE	1A6M	1Z8O	2CMM			Sc=6.16592, min distance = 2.33885
CPXJ_SACEN Fı	11957385	MYG_PHYCA Ful	444124 HE	1U7R	1JIP	2CMM			Sc=6.21728, min distance = 2.22537
CPXJ_SACEN Fı	444095 HE	RCEL_RHOVI Fu	444124 HE	3D38	1JIP	1DXR		0.91	Sc=6.73183, min distance = 2.04501
CPXJ_SACEN Fı	444095 HE	RCEM_RHOVI Fu	444124 HE	3D38	1JIP	1DXR		0.91	Sc=6.72444, min distance = 2.23708
CPXJ_SACEN Fı	444125 HE	RCEL_RHOVI Fu	444124 HE	3D38	1JIP	2I5N		0.91	Sc=6.74499, min distance = 2.33660
CPXJ_SACEN Fı	444522 HE	NOS3_BOVIN Fu	444124 HE	1ZZS	1JIP	2HX2		0.99	Sc=6.6715, min distance = 2.294017
CPXJ_SACEN Fı	5326592 C	HMOX1_RAT Ful	444098 HE	1DVG	1Z8O	1J2C		0.79	Sc=6.6858, min distance = 2.260802
CPXJ_SACEN Fı	5326592 C	HMOX1_RAT Ful	444124 HE	1J02	1JIP	1J2C		0.84	Sc=6.66795, min distance = 2.13125
CPXJ_SACEN Fı	6420167 M	CPXA_PSEPU Fu	444098 HE	1YRC	1Z8O	2FE6		0.86	Sc=6.75861, min distance = 2.00849
CPXL_PSESP Fı	11957370	HMOX1_HUMAN Fı	444124 HE	1OZW	1CPT	1TWN			Sc=6.66898, min distance = 1.76399
CPXL_PSESP Fı	11957371	HMOX1_HUMAN Fı	444124 HE	1OZW	1CPT	1TWR			Sc=6.69199, min distance = 1.76575
CPXL_PSESP Fı	11957385	MYG_PHYCA Ful	444124 HE	1U7R	1CPT	2CMM			Sc=6.31993, min distance = 2.01316
CPXL_PSESP Fı	13472 Bol	CPXJ_SACEN Fu	444124 HE	1JIP	1CPT	1EUP			Sc=6.08308, min distance = 1.33885
CPXL_PSESP Fı	444207 He	CP119_SULTO Fı	444124 HE	3B4X	1CPT	1UE8		0.99	Sc=6.81395, min distance = 2.11661
CPXL_PSESP Fı	444522 HE	NOS3_BOVIN Fu	444124 HE	1ZZS	1CPT	2HX2		0.99	Sc=6.81575, min distance = 2.21854
CRCA_ECOLI Fı	11987742	LHA4_RHOAC Fu	444279 1s	2FKW	1THQ	1NKZ			Sc=6.30243, min distance = 1.15772
CRCA_ECOLI Fı	5326780 C	AOFB_HUMAN Fu	444279 1s	1OJD	1THQ	1S2Y			Sc=5.6574, min distance = 1.901778
CRCA_ECOLI Fı	66182 1,4	AOFB_HUMAN Fu	444279 1s	1OJD	1THQ	1OJ9			Sc=5.63551, min distance = 2.73435
CRP_ECOLI Fu	24316 cyc	KAP0_BOVIN Fu	6076 Cycl	1RGS	1HW5	1RL3	26.32	0.81	Sc=6.36711, min distance = 2.58060
CRP_ECOLI Fu	656964 1y	PDE4D_HUMAN Fı	6076 Cycl	2PW3	1HW5	1Y2C			Sc=5.94217, min distance = 2.29955
CRP_ECOLI Fu	656966 1y	PDE4D_HUMAN Fı	6076 Cycl	2PW3	1HW5	1Y2E			Sc=6.08061, min distance = 1.19463
CRP_ECOLI Fu	6858240 C	KAP0_BOVIN Fu	6076 Cycl	1RGS	1HW5	1NE6	26.32	0.98	Sc=6.26983, min distance = 2.49157
CRTM_STAAU Fı	4395717 b	GGPPS_YEAST Fı	16122552	2E8T	2ZCP	2Z52			Sc=6.30813, min distance = 1.94697
CRTM_STAAU Fı	448400 IS	GGPPS_YEAST Fı	16122552	2E8T	2ZCP	2E8U			Sc=5.80334, min distance = 1.06208
CRTM_STAAU Fı	448987 FE	GGPPS_YEAST Fı	16122552	2E8T	2ZCP	###			Sc=6.30452, min distance = 2.16630
CRTM_STAAU Fı	5276507 b	GGPPS_YEAST Fı	16122552	2E8T	2ZCP	2Z4X			Sc=6.14999, min distance = 1.97832
CRTM_STAAU Fı	5276520 b	GGPPS_YEAST Fı	16122552	2E8T	2ZCP	2Z50			Sc=5.99765, min distance = 2.10222
CRTM_STAAU Fı	6102720 C	GGPPS_YEAST Fı	16122552	2E8T	2ZCP	2E8X			Sc=6.13893, min distance = 1.84586
CRY1_ARATH Fı	1400 nche	HCK_HUMAN Ful	33113 gam	1AD5	1U3D	1QCF			Sc=6.19336, min distance = 2.66478
CRY1_ARATH Fı	16040273	IRAK4_HUMAN Fı	33113 gam	2OID	1U3D	2OIC			Sc=6.29987, min distance = 1.70561
CRY1_ARATH Fı	16750062	CSK2A_MAIZE Fı	33113 gam	1LP4	1U3D	2OXD			Sc=5.61217, min distance = 3.05463

CRY1_ARATH F	24779674	CSK2A_MAIZE F	33113 gar	1LP4	1U3D	2PVH			Sc=6.24576, min distance = 2.09658
CRY1_ARATH F	24963048	AKT2_HUMAN Fu	33113 gar	1O6L	1U3D	3E87			Sc=6.35198, min distance = 2.49602
CRY1_ARATH F	3547 Fasu	ROCK1_HUMAN F	33113 gar	2V55	1U3D	2ESM			Sc=5.89401, min distance = 2.17277
CRY1_ARATH F	444502 CI	PHR_SYNP6 Ful	444188 CI	1TEZ	1U3D	1OWM		0.98	Sc=6.42638, min distance = 2.34511
CRY1_ARATH F	444564 AD	MYS2_DICDI Fu	33113 gar	1MMN	1U3D	1W9I		0.91	Sc=5.81014, min distance = 2.71709
CRY1_ARATH F	444564 AD	PDK2_RAT Full	33113 gar	3CRL	1U3D	1JM6		0.91	Sc=6.2817, min distance = 2.526054
CRY1_ARATH F	5327148 C	IPKA_RABIT Fu	33113 gar	1CDK	1U3D	2ERZ			Sc=6.11282, min distance = 2.22879
CRY1_ARATH F	6083 ader	PIM1_HUMAN Fu	33113 gar	1XR1	1U3D	1YXU		0.97	Sc=6.22859, min distance = 2.47999
CRY1_ARATH F	60961 ade	PIM1_HUMAN Fu	33113 gar	1XR1	1U3D	1YI4		0.93	Sc=6.2074, min distance = 2.101319
CRY1_ARATH F	60961 ade	SKY1_YEAST Fu	33113 gar	1Q99	1U3D	1Q97		0.93	Sc=6.21059, min distance = 2.01252
CRY1_ARATH F	65103 dGT	RIR1_YEAST Fu	33113 gar	2EUD	1U3D	2CVX		0.78	Sc=5.86949, min distance = 2.21287
CRY1_ARATH F	91532 AME	PURT_ECOLI Fu	33113 gar	1EYZ	1U3D	1KJI		0.99	Sc=5.94069, min distance = 2.26438
CRYD_ARATH F	444502 CI	PHR_SYNP6 Ful	444188 CI	1TEZ	2J4D	1OWM		0.98	Sc=6.15297, min distance = 2.38585
CSK21_HUMAN I	1400 nche	HCK_HUMAN Ful	33113 gar	1AD5	2PVR	1QCF	21.67		Sc=6.08873, min distance = 2.08154
CSK21_HUMAN I	16750062	CSK2A_MAIZE F	445479 CI	1F0Q	3BQC	2OXD	77.57		Sc=5.60323, min distance = 2.40133
CSK21_HUMAN I	23656870	CSK2A_MAIZE F	33113 gar	1LP4	2PVR	2PVN	77.57		Sc=6.42814, min distance = 2.10756
CSK21_HUMAN I	24756824	CSK2A_MAIZE F	33113 gar	1LP4	2PVR	3BE9	77.57		Sc=6.3487, min distance = 2.337159
CSK21_HUMAN I	24779678	CSK2A_MAIZE F	33113 gar	1LP4	2PVR	2PVM	77.57		Sc=6.34623, min distance = 2.39308
CSK21_HUMAN I	444345 1c	GSK3B_HUMAN F	33113 gar	1J1B	2PVR	1Q3D	35.32		Sc=6.25463, min distance = 2.13812
CSK21_HUMAN I	5326739 i	GSK3B_HUMAN F	33113 gar	1J1B	2PVR	1Q41	35.32		Sc=6.12131, min distance = 2.82141
CSK21_HUMAN I	5326976 1	CSK2A_MAIZE F	445479 CI	1F0Q	3BQC	1ZOE	77.57		Sc=5.60104, min distance = 2.31452
CSK21_HUMAN I	5327121 I	PIM1_HUMAN Fu	33113 gar	1XR1	2PVR	2C3I	31.73		Sc=6.02719, min distance = 2.26836
CSK21_HUMAN I	5327148 C	IPKA_RABIT Fu	33113 gar	1CDK	2PVR	2ERZ			Sc=5.83903, min distance = 2.44476
CSK21_HUMAN I	5795340 M	CSK2A_MAIZE F	445479 CI	1F0Q	3BQC	2QC6	77.57		Sc=5.72494, min distance = 2.59190
CSK2A_MAIZE I	16758212	GSK3B_HUMAN F	33113 gar	1J1B	1LP4	2O5K	36.84		Sc=6.40334, min distance = 1.69179
CSK2A_MAIZE I	188966 dA	HSLU_ECOLI Fu	33113 gar	1E94	1LP4	1G4A		0.97	Sc=6.3486, min distance = 1.784185
CSK2A_MAIZE I	24871491	INSR_HUMAN Fu	33113 gar	1IR3	1LP4	2Z8C			Sc=6.40559, min distance = 2.58697
CSK2A_MAIZE I	3064778 H	ROCK1_HUMAN F	33113 gar	2V55	1LP4	2ETK	27.91		Sc=6.08885, min distance = 2.04740
CSK2A_MAIZE I	3547 Fasu	ROCK1_HUMAN F	33113 gar	2V55	1LP4	2ESM	27.91		Sc=5.92108, min distance = 2.55901
CSK2A_MAIZE I	444345 1c	GSK3B_HUMAN F	33113 gar	1J1B	1LP4	1Q3D	36.84		Sc=6.3897, min distance = 1.640851
CSK2A_MAIZE I	445432 CI	SYQ_ECOLI Ful	83862 ade	1O0C	1DS5	1QTQ		0.84	Sc=6.49685, min distance = 1.62319
CSK2A_MAIZE I	447956 1p	CDK2_HUMAN Fu	1694 TBBt	1P5E	1J91	2C5O	33.2		Sc=5.8354, min distance = 1.801336
CSK2A_MAIZE I	448005 CI	GSK3B_HUMAN F	33113 gar	1J1B	1LP4	1Q3W	36.84		Sc=6.26713, min distance = 2.32209
CSK2A_MAIZE I	5281680 Q	PIM1_HUMAN Fu	33113 gar	1XR1	1LP4	2O64	29.2		Sc=6.43045, min distance = 2.11121
CSK2A_MAIZE I	5287844 i	GSK3B_HUMAN F	33113 gar	1J1B	1LP4	1UV5	36.84		Sc=6.32019, min distance = 2.56840
CSK2A_MAIZE I	5326739 i	GSK3B_HUMAN F	33113 gar	1J1B	1LP4	1Q41	36.84		Sc=6.29151, min distance = 2.34940
CSK2A_MAIZE I	6022 Ader	Q04230_ECOLX I	36735 Gpp	1GL6	1DAY	1GKI		0.79	Sc=6.41448, min distance = 1.65292

CSK2A_MAIZE	60961 ade	PIM1_HUMAN Fu	33113 gam	1XR1	1LP4	1YI4	29.2	0.93	Sc=6.175, min distance = 2.1954657
CSK2A_MAIZE	60961 ade	SKY1_YEAST Fu	33113 gam	1Q99	1LP4	1Q97	27.91	0.93	Sc=6.15526, min distance = 1.702746
CSK2A_MAIZE	611002 Op	PIM1_HUMAN Fu	33113 gam	1XR1	1LP4	1YXX	29.2		Sc=6.05908, min distance = 2.420122
CSK2A_MAIZE	6918710 5	CDK2_HUMAN Fu	1694 TBBt	1P5E	1J91	3EJ1	33.2		Sc=6.14555, min distance = 2.249483
CSK2A_MAIZE	87031 ZIN	CDK2_HUMAN Fu	1694 TBBt	1P5E	1J91	2VTH	33.2		Sc=5.63197, min distance = 1.963574
CSK2A_MAIZE	91532 AMP	PURT_ECOLI Fu	33113 gam	1EYZ	1LP4	1KJI		0.99	Sc=6.01318, min distance = 2.635305
CSK2A_MAIZE	9547921 G	SYQ_ECOLI Ful	83862 ade	1O0C	1DS5	2RE8		0.82	Sc=6.49998, min distance = 1.386766
CSK2A_MAIZE	9817550 V	CDK2_HUMAN Fu	1694 TBBt	1P5E	1J91	3BHV	33.2		Sc=6.37682, min distance = 2.368157
CSKP_HUMAN F	10109823	PIM1_HUMAN Fu	6083 aden	1YXU	3C0H	3CY3			Sc=6.36703, min distance = 1.951460
CSKP_HUMAN F	16040294	SRC_CHICK Ful	6083 aden	3DQX	3C0H	3F6X			Sc=6.4189, min distance = 2.5381743
CSKP_HUMAN F	24851689	PIM1_HUMAN Fu	6083 aden	1YXU	3C0H	3CY2			Sc=6.13386, min distance = 1.654171
CSKP_HUMAN F	24905143	SRC_CHICK Ful	6083 aden	3DQX	3C0H	3EN7			Sc=6.09374, min distance = 1.943000
CSKP_HUMAN F	3973 nche	PIM1_HUMAN Fu	6083 aden	1YXU	3C0H	1YI3			Sc=5.89372, min distance = 1.605488
CSKP_HUMAN F	440317 AT	SRC_CHICK Ful	6083 aden	3DQX	3C0H	3DQW		0.97	Sc=5.86365, min distance = 2.192549
CSKP_HUMAN F	447832 AD	ACSA_SALTY Fu	6083 aden	2P2F	3C0H	1PG4		0.99	Sc=6.3044, min distance = 2.5770940
CSKP_HUMAN F	448895 AD	UMPK_YEAST Fu	6083 aden	1UKZ	3C0H	1UKY		0.99	Sc=5.81077, min distance = 2.515759
CSKP_HUMAN F	5327121 I	PIM1_HUMAN Fu	6083 aden	1YXU	3C0H	2C3I			Sc=6.06255, min distance = 2.289695
CSKP_HUMAN F	6022 Aden	PURP_METJA Fu	6083 aden	2R7M	3C0H	2R7N		0.99	Sc=6.26604, min distance = 2.498929
CSKP_HUMAN F	6419789 C	HASP_HUMAN Fu	6083 aden	3DLZ	3C0H	3E7V			Sc=5.99053, min distance = 2.945257
CSKP_HUMAN F	656964 1y	PDE4D_HUMAN F	6083 aden	1TB7	3C0H	1Y2C			Sc=5.72713, min distance = 2.443888
CSKP_HUMAN F	656966 1y	PDE4D_HUMAN F	6083 aden	1TB7	3C0H	1Y2E			Sc=5.91095, min distance = 2.268706
CSKP_HUMAN F	8582 Inos	PYGM_RABIT Fu	6083 aden	8GPB	3C0H	2QN7		0.79	Sc=6.18128, min distance = 2.737035
CSKP_HUMAN F	91532 AMP	PURP_METJA Fu	6083 aden	2R7M	3C0H	2R7K		0.97	Sc=5.61273, min distance = 2.708328
CSK_HUMAN Fu	10224714	CDK2_HUMAN Fu	444345 1c	1AQ1	1BYG	3DDP			Sc=6.35867, min distance = 2.420887
CSK_HUMAN Fu	10389239	MAPK2_HUMAN F	444345 1c	2PZY	1BYG	2JBP			Sc=6.23362, min distance = 2.190535
CSK_HUMAN Fu	11348631	CHK1_HUMAN Fu	444345 1c	1NVR	1BYG	2E9U			Sc=6.19086, min distance = 2.736867
CSK_HUMAN Fu	11553058	LCK_HUMAN Ful	444345 1c	1QPD	1BYG	2OF2			Sc=6.13335, min distance = 2.146776
CSK_HUMAN Fu	11992146	MAPK2_HUMAN F	444345 1c	2PZY	1BYG	2P3G			Sc=6.14868, min distance = 2.142227
CSK_HUMAN Fu	153999 Ru	PDPK1_HUMAN F	444345 1c	1OKY	1BYG	1UU3		0.77	Sc=6.29828, min distance = 2.310082
CSK_HUMAN Fu	1540 1h1q	CDK2_HUMAN Fu	444345 1c	1AQ1	1BYG	1H1Q			Sc=6.21045, min distance = 2.661379
CSK_HUMAN Fu	15602982	KAPCA_BOVIN F	444345 1c	1STC	1BYG	2UW6			Sc=6.01906, min distance = 2.529867
CSK_HUMAN Fu	15991572	LCK_HUMAN Ful	444345 1c	1QPD	1BYG	2OF4			Sc=6.22411, min distance = 2.557618
CSK_HUMAN Fu	160355 ro	CDK2_HUMAN Fu	444345 1c	1AQ1	1BYG	3DDQ			Sc=6.11985, min distance = 2.513565
CSK_HUMAN Fu	16122608	PDPK1_HUMAN F	444345 1c	1OKY	1BYG	2PE1			Sc=6.22587, min distance = 2.740595
CSK_HUMAN Fu	16122643	CHK1_HUMAN Fu	444345 1c	1NVR	1BYG	2YWP			Sc=6.22653, min distance = 2.625712
CSK_HUMAN Fu	16214825	CDK2_HUMAN Fu	444345 1c	1AQ1	1BYG	2UZE			Sc=6.14419, min distance = 2.305101
CSK_HUMAN Fu	16758227	CHK1_HUMAN Fu	444345 1c	1NVR	1BYG	2R0U			Sc=6.51196, min distance = 2.508131

CSK_HUMAN Fu	1707 1fvt	CDK2_HUMAN Fu	444345 1c	1AQ1	1BYG	1FVT	Sc=6.02751, min distance = 2.32299
CSK_HUMAN Fu	1907917 2	CHK1_HUMAN Fu	444345 1c	1NVR	1BYG	2CGW	Sc=6.03709, min distance = 2.96481
CSK_HUMAN Fu	23653515	CDK2_HUMAN Fu	444345 1c	1AQ1	1BYG	2R3F	Sc=5.88629, min distance = 2.65189
CSK_HUMAN Fu	23653516	CDK2_HUMAN Fu	444345 1c	1AQ1	1BYG	2R3G	Sc=6.16893, min distance = 2.63492
CSK_HUMAN Fu	23653518	CDK2_HUMAN Fu	444345 1c	1AQ1	1BYG	2R3J	Sc=6.07163, min distance = 2.58783
CSK_HUMAN Fu	23653521	CDK2_HUMAN Fu	444345 1c	1AQ1	1BYG	2R3M	Sc=6.23784, min distance = 2.53904
CSK_HUMAN Fu	23657800	CHK1_HUMAN Fu	444345 1c	1NVR	1BYG	2E9P	Sc=6.24325, min distance = 2.58953
CSK_HUMAN Fu	24864081	CDK2_HUMAN Fu	444345 1c	1AQ1	1BYG	2VTS	Sc=6.21474, min distance = 2.25851
CSK_HUMAN Fu	24905144	PK3CG_HUMAN Fu	444345 1c	1E8Z	1BYG	3ENE	Sc=5.99337, min distance = 2.78844
CSK_HUMAN Fu	24916751	CDK2_HUMAN Fu	444345 1c	1AQ1	1BYG	3DOG	Sc=6.11559, min distance = 2.43191
CSK_HUMAN Fu	24963038	LCK_HUMAN Ful	444345 1c	1QPD	1BYG	2ZM1	Sc=6.03409, min distance = 2.20781
CSK_HUMAN Fu	24963039	LCK_HUMAN Ful	444345 1c	1QPD	1BYG	2ZM4	Sc=6.22936, min distance = 2.20399
CSK_HUMAN Fu	25021197	CDK2_HUMAN Fu	444345 1c	1AQ1	1BYG	3EOC	Sc=6.07265, min distance = 2.78831
CSK_HUMAN Fu	33113 gam	IRAK4_HUMAN Fu	444345 1c	2NRY	1BYG	2OID	Sc=5.78497, min distance = 1.85452
CSK_HUMAN Fu	33113 gam	PIM1_HUMAN Fu	444345 1c	1YHS	1BYG	1XR1	Sc=5.60912, min distance = 2.41259
CSK_HUMAN Fu	3543 1g5s	CDK2_HUMAN Fu	444345 1c	1AQ1	1BYG	1G5S	Sc=6.30314, min distance = 2.56693
CSK_HUMAN Fu	446704 LS	CDK2_HUMAN Fu	444345 1c	1AQ1	1BYG	1KE6	Sc=6.08465, min distance = 2.07053
CSK_HUMAN Fu	447649 1c	CDK2_HUMAN Fu	444345 1c	1AQ1	1BYG	1OI9	Sc=6.28409, min distance = 2.68291
CSK_HUMAN Fu	447654 CI	CDK2_HUMAN Fu	444345 1c	1AQ1	1BYG	1OIT	Sc=5.98191, min distance = 2.81170
CSK_HUMAN Fu	447962 1p	CDK2_HUMAN Fu	444345 1c	1AQ1	1BYG	2C5N	Sc=6.0364, min distance = 3.131193
CSK_HUMAN Fu	447967 CI	CDK2_HUMAN Fu	444345 1c	1AQ1	1BYG	1PYE	Sc=6.24732, min distance = 3.01734
CSK_HUMAN Fu	448043 2g	KAPCA_BOVIN Fu	444345 1c	1STC	1BYG	1Q8U	Sc=5.94252, min distance = 2.52005
CSK_HUMAN Fu	448171 CI	LCK_HUMAN Ful	444345 1c	1QPD	1BYG	1QPE	Sc=6.07096, min distance = 2.78737
CSK_HUMAN Fu	448238 CI	GSK3B_HUMAN Fu	444345 1c	1Q3D	1BYG	1R0E	Sc=6.50349, min distance = 2.03094
CSK_HUMAN Fu	448293 CI	CDK2_HUMAN Fu	444345 1c	1AQ1	1BYG	1R78	Sc=6.36355, min distance = 2.29888
CSK_HUMAN Fu	456214 pu	KS6A1_HUMAN Fu	444345 1c	2Z7R	1BYG	2Z7S	Sc=6.26308, min distance = 2.02047
CSK_HUMAN Fu	4565 1h1r	CDK2_HUMAN Fu	444345 1c	1AQ1	1BYG	1H1R	Sc=5.87321, min distance = 2.82384
CSK_HUMAN Fu	5288708 1	CDK2_HUMAN Fu	444345 1c	1AQ1	1BYG	1KE5	Sc=6.04661, min distance = 2.52485
CSK_HUMAN Fu	5288710 C	CDK2_HUMAN Fu	444345 1c	1AQ1	1BYG	1KE7	Sc=6.24846, min distance = 2.51132
CSK_HUMAN Fu	5288712 1	CDK2_HUMAN Fu	444345 1c	1AQ1	1BYG	1KE9	Sc=6.13713, min distance = 2.78635
CSK_HUMAN Fu	5327123 I	CHK1_HUMAN Fu	444345 1c	1NVR	1BYG	2C3L	Sc=6.12677, min distance = 2.47103
CSK_HUMAN Fu	5327134 C	CDK2_HUMAN Fu	444345 1c	1AQ1	1BYG	2C6L	Sc=6.19653, min distance = 2.07086
CSK_HUMAN Fu	5331010 c	CDK2_HUMAN Fu	444345 1c	1AQ1	1BYG	2BKZ	Sc=6.14826, min distance = 2.50840
CSK_HUMAN Fu	5494414 C	CDK2_HUMAN Fu	444345 1c	1AQ1	1BYG	2A0C	Sc=6.37042, min distance = 2.62746
CSK_HUMAN Fu	5957 Ader	PDPK1_HUMAN Fu	444345 1c	1OKY	1BYG	2BIY	Sc=6.41928, min distance = 2.21797
CSK_HUMAN Fu	6022 Ader	CDK2_HUMAN Fu	444345 1c	1AQ1	1BYG	1GY3	Sc=6.28577, min distance = 2.14113
CSK_HUMAN Fu	6022 Ader	GSK3B_HUMAN Fu	444345 1c	1Q3D	1BYG	1J1C	Sc=5.87734, min distance = 2.07139

CSK_HUMAN Fu	6102670	CDK2_HUMAN Fu	444345 1c	1AQ1	1BYG	1YKR		Sc=6.17735, min distance = 2.74323
CSK_HUMAN Fu	6420139 C	CDK2_HUMAN Fu	444345 1c	1AQ1	1BYG	2C5V		Sc=6.04325, min distance = 2.78983
CSK_HUMAN Fu	6539118 g	CDK2_HUMAN Fu	444345 1c	1AQ1	1BYG	1FVV		Sc=6.35923, min distance = 2.66761
CSK_HUMAN Fu	6540273 2	CHK1_HUMAN Fu	444345 1c	1NVR	1BYG	2CGV		Sc=6.1216, min distance = 2.543075
CSK_HUMAN Fu	656971 CI	CDK2_HUMAN Fu	444345 1c	1AQ1	1BYG	1Y8Y		Sc=5.85128, min distance = 2.61188
CSK_HUMAN Fu	72271 7-H	CHK1_HUMAN Fu	444345 1c	1NVR	1BYG	1NVQ	0.91	Sc=6.39193, min distance = 2.77457
CSLA_PEDHE Fu	448431 CI	P84141_ARTAU	24139 ace	1RWC	1HM3	1RWH		Sc=5.72577, min distance = 2.32634
CSLB_PEDHE Fu	6927060 Z	HYSA_STRPN Fu	446101 AC	1OJO	1OFL	1F9G		Sc=5.63309, min distance = 2.37091
CTBP1_HUMAN	11987786	LDH_PLAFD Ful	5893 nadi	1T2D	1MX3	1T2E	0.77	Sc=6.76878, min distance = 2.67174
CTBP1_HUMAN	16129587	G3PA_SPIOL Fu	5893 nadi	1NBO	1MX3	2PKR		Sc=6.36006, min distance = 1.89587
CTBP1_HUMAN	4369002 C	LDHA_PIG Full=	5893 nadi	9LDB	1MX3	9LDT	0.77	Sc=6.76739, min distance = 2.31619
CTBP1_HUMAN	439153 Di	ADH1B_HUMAN Fu	5893 nadi	1U3U	1MX3	1DEH	0.79	Sc=6.77154, min distance = 1.91401
CTBP1_HUMAN	439153 Di	CTBP1_RAT Ful	5893 nadi	1HKU	1MX3	1HL3	0.79	Sc=6.76564, min distance = 2.53513
CTBP1_HUMAN	440516 CI	MDH_THETH Ful	5893 nadi	1BMD	1MX3	1BDM	0.79	Sc=6.33239, min distance = 2.61345
CTBP1_HUMAN	445794 AD	ADHX_HUMAN Fu	5893 nadi	2FZW	1MX3	2FZE		Sc=6.61414, min distance = 1.57999
CTBP1_HUMAN	6022 Ader	UGDH_HUMAN Fu	5893 nadi	2Q3E	1MX3	2QG4		Sc=6.77086, min distance = 1.93172
CTBP1_HUMAN	6102710 A	Q4PRK9_PLAVI	5893 nadi	2A92	1MX3	2AA3	0.95	Sc=6.77909, min distance = 2.75628
CTBP1_RAT Fu	11987786	LDH_PLAFD Ful	5893 nadi	1T2D	1HKU	1T2E	0.77	Sc=6.76564, min distance = 2.49790
CTBP1_RAT Fu	123927 A3	Q9GT92_CRYPV	5893 nadi	2FM3	1HKU	2EWD	0.97	Sc=6.76818, min distance = 2.37081
CTBP1_RAT Fu	440516 CI	MDH_THETH Ful	5893 nadi	1BMD	1HKU	1BDM	0.79	Sc=6.3314, min distance = 2.634357
CTBP1_RAT Fu	445794 AD	ADHX_HUMAN Fu	5893 nadi	2FZW	1HKU	2FZE		Sc=6.6105, min distance = 2.602595
CTBP1_RAT Fu	446288 NA	G3P_PALVE Ful	5893 nadi	1DSS	1HKU	1IHX		Sc=6.75726, min distance = 2.18158
CTBP2_HUMAN	11987786	LDH_PLAFD Ful	5893 nadi	1T2D	2OME	1T2E	0.77	Sc=6.78241, min distance = 2.66366
CTBP2_HUMAN	123927 A3	Q9GT92_CRYPV	5893 nadi	2FM3	2OME	2EWD	0.97	Sc=6.77941, min distance = 2.19204
CTBP2_HUMAN	439153 Di	ADH1E_HORSE Fu	5893 nadi	1MGO	2OME	2JHF	0.79	Sc=6.77476, min distance = 2.23438
CTBP2_HUMAN	439153 Di	CTBP1_RAT Ful	5893 nadi	1HKU	2OME	1HL3	0.79	Sc=6.78013, min distance = 2.61554
CUEO_ECOLI Fu	5354052 p	MIF_HUMAN Ful	311 citri	1GD0	2FQE	2OOZ		Sc=5.83481, min distance = 0.61845
CUEO_ECOLI Fu	8778 Laur	CTXA3_NAJAT Fu	311 citri	1XT3	2FQE	1H0J		Sc=5.75463, min distance = 0.80248
CUTA_PYRHO Fu	1881 GW34	DYR_CANAL Ful	78165 MES	1M79	1V99	1AOE		Sc=5.75796, min distance = 1.50069
CVN_NOSEL Fu	101798 Me	LEC1_LATOC Fu	185698 al	1LOG	2RDK	1LOB	0.94	Sc=5.81357, min distance = 2.48140
CVN_NOSEL Fu	101798 Me	LECB_LATOC Fu	185698 al	1LOG	2RDK	1LOB	0.94	Sc=5.80653, min distance = 2.49896
CVN_NOSEL Fu	101798 Me	LEC_GALNI Ful	185698 al	1JPC	2RDK	1MSA	0.94	Sc=5.83642, min distance = 2.18492
CVN_NOSEL Fu	16741013	CARP_YEAST Fu	151504 D-	1FQ5	2PYS	1FQ6		Sc=5.63307, min distance = 2.49356
CVN_NOSEL Fu	16741013	CARP_YEAST Fu	185698 al	1DPJ	2RDK	1FQ6		Sc=5.7989, min distance = 0.906157
CVN_NOSEL Fu	445184 1,	Q6P5R5_HUMAN	185698 al	1OP3	2RDK	1ZLW	0.89	Sc=5.70923, min distance = 2.62397
CVN_NOSEL Fu	445184 1,	Q6PYX1_HUMAN	185698 al	1ZLS	2RDK	1ZLW	0.89	Sc=5.83319, min distance = 1.68984
CVN_NOSEL Fu	445948 D-	Q9HYN5_PSEAE	185698 al	1OVS	2RDK	1OXC		Sc=5.79104, min distance = 1.95815

CVN_NOSEL Fu	449023 N2	CONA_CANEN Fu	151504 D-	1I3H	2PYS	1VAM			Sc=6.26117, min distance = 0.61924
CVN_NOSEL Fu	64947 alp	CONA_CANEN Fu	151504 D-	1I3H	2PYS	1GIC			Sc=5.73529, min distance = 2.23312
CVN_NOSEL Fu	64947 alp	LEC1_LATOC Fu	185698 al	1LOG	2RDK	1LOA		0.94	Sc=5.84586, min distance = 1.76905
CVN_NOSEL Fu	64947 alp	LEC_PEA Full=	185698 al	1RIN	2RDK	1HKD		0.94	Sc=5.81357, min distance = 2.34549
CVN_NOSEL Fu	87330 M97	CONA_CANEN Fu	151504 D-	1I3H	2PYS	1CJP			Sc=6.27338, min distance = 2.02221
CY1_BOVIN Fu	11957385	MYG_PHYCA Ful	444098 HE	1A6M	1L0N	2CMM			Sc=6.08163, min distance = 2.35629
CY1_BOVIN Fu	444279 1s	OMPG_ECOLI Fu	62852 B-C	2IWW	1BCC	2IWV			Sc=5.85757, min distance = 1.23502
CY552_THIFE	11957385	MYG_PHYCA Ful	444124 HE	1U7R	1H1O	2CMM			Sc=6.26401, min distance = 2.08570
CY552_THIFE	16214774	CP51_MYCTU Fu	444124 HE	2CIB	1H1O	2CI0		0.95	Sc=6.64391, min distance = 2.02472
CY552_THIFE	444207 He	CP119_SULTO F	444124 HE	3B4X	1H1O	1UE8		0.99	Sc=6.74046, min distance = 1.99471
CYAA_BACAN F	5957 Ader	PANC_MYCTU Fu	91557 1tr	1N2E	1S26	2A84		0.99	Sc=5.96842, min distance = 1.91074
CYAA_BACAN F	6083 ader	NADE_BACAN Fu	91557 1tr	2PZ8	1S26	2PZA		0.97	Sc=6.30896, min distance = 1.67468
CYB2_YEAST F	11957364	HMOX1_HUMAN F	444124 HE	1OZW	1KBI	1S13		0.86	Sc=6.6716, min distance = 1.709093
CYB2_YEAST F	11957371	HMOX1_HUMAN F	444124 HE	1OZW	1KBI	1TWR			Sc=6.66842, min distance = 1.63633
CYB2_YEAST F	11957385	MYG_PHYCA Ful	444124 HE	1U7R	1KBI	2CMM			Sc=6.23686, min distance = 2.02392
CYB2_YEAST F	16741044	PYRDA_LACLC F	444243 FA	1JUB	1KBI	1JRB			Sc=6.67326, min distance = 2.22272
CYB2_YEAST F	24180714	HAOX1_HUMAN F	444243 FA	2NZL	1KBI	2RDT			Sc=6.15247, min distance = 2.38642
CYB2_YEAST F	444095 HE	RCEM_RHOVI Fu	444124 HE	3D38	1KBI	1DXR		0.91	Sc=6.76919, min distance = 2.02754
CYB2_YEAST F	444207 He	CP119_SULTO F	444124 HE	3B4X	1KBI	1UE8		0.99	Sc=6.7621, min distance = 2.085937
CYB2_YEAST F	444207 He	PER_COPCI Ful	444124 HE	1LY9	1KBI	1LY8		0.99	Sc=6.75995, min distance = 2.02319
CYB5B_RAT Fu	11957385	MYG_PHYCA Ful	444124 HE	1U7R	1AWP	2CMM			Sc=6.22067, min distance = 1.95383
CYB_BOVIN Fu	11957363	HBA_HUMAN Ful	444522 HE	2DN1	1BGY	1RQA		0.81	Sc=6.80625, min distance = 2.02414
CYB_BOVIN Fu	11957385	MYG_PHYCA Ful	444522 HE	1VXD	1BGY	2CMM			Sc=6.37803, min distance = 2.10328
CYB_BOVIN Fu	11970219	CCPR_YEAST Fu	444522 HE	3E2O	1BGY	1BEM		0.87	Sc=6.80662, min distance = 1.86233
CYB_BOVIN Fu	11970242	CCPR_YEAST Fu	444522 HE	3E2O	1BGY	1CPE		0.87	Sc=6.80697, min distance = 2.04083
CYB_BOVIN Fu	16741183	MYG_PHYCA Ful	444522 HE	1VXD	1BGY	2EKT		0.93	Sc=6.69694, min distance = 1.99148
CYB_BOVIN Fu	4369228 C	PER_COPCI Ful	444522 HE	1H3J	1BGY	1LYC		0.96	Sc=6.80662, min distance = 1.77818
CYB_BOVIN Fu	444207 He	PER_COPCI Ful	444522 HE	1H3J	1BGY	1LY8	1		Sc=6.809, min distance = 1.989356
CYB_BOVIN Fu	444207 He	PETD_MASLA Fu	444522 HE	2E76	1BGY	1VF5	60.53	1	Sc=6.82764, min distance = 0.69875
CYB_BOVIN Fu	447920 ub	RCEL_RHOVI Fu	447344 CI	1R2C	1NTZ	3D38		0.94	Sc=5.81378, min distance = 2.35862
CYB_RHOSH Fu	130804 NQ	CYB_BOVIN Ful	447884 st	1PP9	2QJY	1NU1	49.09		Sc=6.06528, min distance = 1.40851
CYB_YEAST Fu	130804 NQ	CYB_BOVIN Ful	447884 st	1PP9	3CX5	1NU1	51.78		Sc=6.06325, min distance = 1.30463
CYC1_YEAST F	11957385	MYG_PHYCA Ful	444124 HE	1U7R	1S6V	2CMM			Sc=6.25497, min distance = 2.02087
CYC22_RHOCE	11957385	MYG_PHYCA Ful	444124 HE	1U7R	1JDL	2CMM			Sc=6.12989, min distance = 2.14265
CYC22_RHOCE	444207 He	CP119_SULTO F	444124 HE	3B4X	1JDL	1UE8		0.99	Sc=6.72562, min distance = 2.10161
CYC2_RHOCA F	11957385	MYG_PHYCA Ful	444522 HE	1VXD	1VYD	2CMM			Sc=6.13626, min distance = 2.19561
CYC2_RHOCA F	444124 HE	Q83WG3_9ACTO	444522 HE	2Z3T	1VYD	2Z3U		0.99	Sc=6.7682, min distance = 2.029225

CYC2_RHORU Fu	11957385	MYG_PHYCA Full	444522 HE	1VXD	3C2C	2CMM			Sc=6.26348, min distance = 2.22141
CYC2_RHORU Fu	444124 HE	Q83WG3_9ACTO I	444522 HE	2Z3T	3C2C	2Z3U		0.99	Sc=6.77783, min distance = 2.23668
CYC3_DESVH Fu	11957385	MYG_PHYCA Full	444098 HE	1A6M	2CYM	2CMM			Sc=6.33945, min distance = 1.89470
CYCL_METEX Fu	444207 He	CP119_SULTO Fu	444124 HE	3B4X	2C8S	1UE8		0.99	Sc=6.7807, min distance = 2.019173
CYC_HORSE Ful	11957385	MYG_PHYCA Full	444098 HE	1A6M	2PCB	2CMM			Sc=6.24243, min distance = 2.07115
CYF_CHLRE Ful	11957385	MYG_PHYCA Full	444124 HE	1U7R	1CFM	2CMM			Sc=6.40212, min distance = 2.22108
CYF_CHLRE Ful	444207 He	CP119_SULTO Fu	444124 HE	3B4X	1CFM	1UE8		0.99	Sc=6.83721, min distance = 2.21317
CYF_CHLRE Ful	444522 HE	NOSO_BACSU Ful	444124 HE	2AN0	1CFM	2FC1		0.99	Sc=6.7953, min distance = 2.235596
CYF_MASLA Ful	11957360	CYB_BOVIN Full	444522 HE	1BGY	2E76	1NTM		0.78	Sc=6.83861, min distance = 2.06593
CYGB_HUMAN Fu	11957353	HBA_HORSE Full	444522 HE	1Y8K	1V5H	1IWH	29.73	0.87	Sc=6.70476, min distance = 2.07009
CYGB_HUMAN Fu	11957363	HBB_HUMAN Full	444098 HE	1J40	2DC3	1RQA	29.55	0.77	Sc=6.73025, min distance = 1.87884
CYGB_HUMAN Fu	11957363	HBB_HUMAN Full	444522 HE	2DN1	1V5H	1RQA	29.55	0.81	Sc=6.70476, min distance = 1.91899
CYGB_HUMAN Fu	11957385	MYG_PHYCA Full	444098 HE	1A6M	2DC3	2CMM	32.03		Sc=6.14199, min distance = 2.08637
CYGB_HUMAN Fu	11957385	MYG_PHYCA Full	444522 HE	1VXD	1V5H	2CMM	32.03		Sc=6.16005, min distance = 2.16761
CYGB_HUMAN Fu	11970242	CCPR_YEAST Ful	444522 HE	3E2O	1V5H	1CPE		0.87	Sc=6.69572, min distance = 2.07914
CYGB_HUMAN Fu	126994 CI	CCPR_YEAST Ful	444098 HE	2EUT	2DC3	1Z53		0.77	Sc=6.67592, min distance = 2.18457
CYGB_HUMAN Fu	126994 CI	CCPR_YEAST Ful	444522 HE	3E2O	1V5H	1Z53		0.81	Sc=6.69404, min distance = 2.11185
CYGB_HUMAN Fu	16741061	MYG_PHYCA Full	444098 HE	1A6M	2DC3	1MBC	32.03	0.83	Sc=6.63656, min distance = 2.06268
CYGB_HUMAN Fu	16741061	MYG_PHYCA Full	444522 HE	1VXD	1V5H	1MBC	32.03	0.87	Sc=6.66202, min distance = 2.11480
CYGB_HUMAN Fu	16741183	MYG_PHYCA Full	444522 HE	1VXD	1V5H	2EKT	32.03	0.93	Sc=6.45674, min distance = 2.04868
CYGB_HUMAN Fu	4368974 C	HBA_HUMAN Full	444522 HE	2DN1	1V5H	1FN3	30.15	0.94	Sc=6.75596, min distance = 1.63998
CYGB_HUMAN Fu	444124 HE	HBA_HUMAN Full	444522 HE	2DN1	1V5H	1NQP	30.15	0.99	Sc=6.71145, min distance = 2.03428
CYGB_HUMAN Fu	444124 HE	HBB_HORSE Full	444522 HE	1Y8K	1V5H	1Y8I	31.39	0.99	Sc=6.74507, min distance = 1.94088
CYGB_HUMAN Fu	444124 HE	MYG_HORSE Full	444522 HE	1NZ3	1V5H	1DWT	30.07	0.99	Sc=6.66476, min distance = 2.08715
CYGB_HUMAN Fu	444124 HE	MYG_PIG Full=M	444522 HE	1MNI	1V5H	1MYH	30.07	0.99	Sc=6.76626, min distance = 2.15147
CYGB_HUMAN Fu	444207 He	HBB_HORSE Full	444522 HE	1Y8K	1V5H	2ZLW	31.39	1	Sc=6.71106, min distance = 2.01455
CYGB_HUMAN Fu	446332 CI	MYG_PHYCA Full	444522 HE	1VXD	1V5H	1IOP	32.03	0.79	Sc=6.66428, min distance = 2.04506
CYH2_MOUSE Fu	16058667	AKT1_HUMAN Ful	107758 1b	1UNQ	1U27	2UVM			Sc=5.62425, min distance = 2.52570
CYOB_ECOLI Fu	11957361	CYB_BOVIN Full	444522 HE	1BGY	1FFT	1NTZ		0.84	Sc=6.75085, min distance = 2.03177
CYOB_ECOLI Fu	11957363	HBB_HUMAN Full	444522 HE	2DN1	1FFT	1RQA		0.81	Sc=6.7615, min distance = 2.163820
CYOB_ECOLI Fu	11957385	MYG_PHYCA Full	444522 HE	1VXD	1FFT	2CMM			Sc=6.23061, min distance = 2.12196
CYOB_ECOLI Fu	16741183	MYG_PHYCA Full	444522 HE	1VXD	1FFT	2EKT		0.93	Sc=6.52021, min distance = 2.05505
CYOB_ECOLI Fu	444124 HE	GLB1_GLYDI Ful	444522 HE	1HBG	1FFT	1JF3		0.99	Sc=6.75286, min distance = 2.04785
CYOB_ECOLI Fu	444124 HE	HBB_HUMAN Full	444522 HE	2DN1	1FFT	1NQP		0.99	Sc=6.76073, min distance = 2.18834
CYOB_ECOLI Fu	446409 HN	HBA_HUMAN Full	444522 HE	2DN1	1FFT	1J40		0.98	Sc=6.75934, min distance = 1.87144
CYPC_BACSU Fu	11957363	HBA_HUMAN Full	444522 HE	2DN1	1IZO	1RQA		0.81	Sc=6.80221, min distance = 2.04025
CYPC_BACSU Fu	11957385	MYG_PHYCA Full	444522 HE	1VXD	1IZO	2CMM			Sc=6.29662, min distance = 2.22626

CYPC_BACSU F	11970219	CCPR_YEAST Fu	444522 HE	3E2O	1IZO	1BEM		0.87	Sc=6.79297, min distance = 2.12856
CYPC_BACSU F	11970221	CCPR_YEAST Fu	444522 HE	3E2O	1IZO	1BES		0.87	Sc=6.75325, min distance = 2.18897
CYPC_BACSU F	11970222	CCPR_YEAST Fu	444522 HE	3E2O	1IZO	1BJ9		0.81	Sc=6.3888, min distance = 2.072847
CYPC_BACSU F	11970242	CCPR_YEAST Fu	444522 HE	3E2O	1IZO	1CPE		0.87	Sc=6.79512, min distance = 2.12546
CYPC_BACSU F	126994 CI	CCPR_YEAST Fu	444522 HE	3E2O	1IZO	1Z53		0.81	Sc=6.79941, min distance = 2.09386
CYPC_BACSU F	14368760	LPXC_AQUAE Fu	985 palmi	2GO3	1IZO	2O3Z			Sc=5.9594, min distance = 1.092981
CYPC_BACSU F	185698 al	CD1D1_MOUSE F	985 palmi	2FIK	1IZO	2Q7Y			Sc=5.66077, min distance = 2.15015
CYPC_BACSU F	4369228 C	PER_COPCI Ful	444522 HE	1H3J	1IZO	1LYC		0.96	Sc=6.80337, min distance = 2.16719
CYPC_BACSU F	444124 HE	GLB1_GLYDI Fu	444522 HE	1HBG	1IZO	1JF3		0.99	Sc=6.81062, min distance = 1.73870
CYPC_BACSU F	444124 HE	MYG_HORSE Ful	444522 HE	1NZ3	1IZO	1DWT		0.99	Sc=6.78307, min distance = 1.83830
CYPC_BACSU F	444124 HE	NOS3_BOVIN Fu	444522 HE	2HX2	1IZO	1ZZS		0.99	Sc=6.78275, min distance = 2.15761
CYPC_BACSU F	444124 HE	PER_COPCI Ful	444522 HE	1H3J	1IZO	1LY9		0.99	Sc=6.79877, min distance = 2.20319
CYPC_BACSU F	444207 He	PER_COPCI Ful	444522 HE	1H3J	1IZO	1LY8	1		Sc=6.805, min distance = 2.132644
CYPC_BACSU F	447168 ZE	CCPR_YEAST Fu	444522 HE	3E2O	1IZO	1ML2		0.86	Sc=6.83036, min distance = 2.12001
CYSD_PSESM F	24905142	SRC_CHICK Ful	440317 AT	3DQW	1ZUN	3EN4			Sc=6.39601, min distance = 1.80490
CYSD_PSESM F	25011745	SRC_CHICK Ful	440317 AT	3DQW	1ZUN	3EL8			Sc=6.29179, min distance = 2.03141
CYSD_PSESM F	444564 AD	MYS2_DICDI Fu	440317 AT	1MMG	1ZUN	1W9I		0.91	Sc=5.93109, min distance = 2.45609
CYSD_PSESM F	6022 Aden	FTSK_PSEAE Fu	440317 AT	2IUT	1ZUN	2IUU		0.99	Sc=5.88452, min distance = 2.14091
CYSD_PSESM F	6022 Aden	MYS2_DICDI Fu	440317 AT	1MMG	1ZUN	1VOM		0.99	Sc=5.90688, min distance = 2.01924
CYSD_PSESM F	6083 ader	SRC_CHICK Ful	440317 AT	3DQW	1ZUN	3DQX		0.97	Sc=6.31583, min distance = 2.42269
CYSG_SALTY F	4369099 C	CBIF_BACME Fu	439155 Ad	1CBF	1PJQ	2CBF			Sc=6.49976, min distance = 2.66708
CYSK_MYCTU F	22489152	TRPB_SALTY Fu	445376 LP	2J9X	2Q3D	2J9Y		0.94	Sc=6.41112, min distance = 2.21042
DAAA_BACYM F	445062 CI	HEM1_RHOCA Fu	1051 Code	2BWO	1DAA	2BWP		0.86	Sc=6.37032, min distance = 1.88719
DAPA_ECOLI F	23831 HEP	RBCMT_PEA Ful	5962 L-ly	2H2E	2ATS	1MLV			Sc=5.6016, min distance = 1.150173
DAPA_ECOLI F	6274 hist	ARGT_SALTY Fu	5962 L-ly	1LST	2ATS	1LAG			Sc=5.66873, min distance = 2.66400
DAPB_ECOLI F	11970225	ETR1_CANTR Fu	5886 NADP	1GUF	1DIH	1N9G		0.78	Sc=6.36228, min distance = 0.94403
DAPB_ECOLI F	439153 Di	ADH1B_HUMAN F	5893 nadi	1U3U	1DRU	1DEH		0.79	Sc=6.75868, min distance = 1.53462
DAPB_ECOLI F	439153 Di	ADH1E_HORSE F	5893 nadi	1MGO	1DRU	2JHF		0.79	Sc=6.30052, min distance = 1.93343
DAPB_ECOLI F	446288 NA	G3P_PALVE Ful	5893 nadi	1DSS	1DRU	1IHX			Sc=6.74505, min distance = 2.29538
DAPB_ECOLI F	5326622 C	URIC_ASPFL Fu	10367 Dip	2PES	1ARZ	3BJP			Sc=5.75463, min distance = 2.13968
DAPB_THEMA F	11987786	LDH_PLAFD Ful	5893 nadi	1T2D	1VM6	1T2E		0.77	Sc=6.75137, min distance = 2.31122
DAPB_THEMA F	169266 1,	GALE_HUMAN Fu	5893 nadi	1HZJ	1VM6	1I3K		0.79	Sc=6.76564, min distance = 1.99541
DAPB_THEMA F	445794 AD	ADHX_HUMAN Fu	5893 nadi	2FZW	1VM6	2FZE			Sc=6.27748, min distance = 2.44901
DAPB_THEMA F	6022 Aden	Q5SI02_THET8	5893 nadi	2BJK	1VM6	2BJA			Sc=6.40913, min distance = 1.78225
DAPK1_HUMAN	10109823	PIM1_HUMAN Fu	444345 1c	1YHS	1WVY	3CY3	30.51		Sc=6.35908, min distance = 2.41509
DAPK1_HUMAN	11502647	CHK1_HUMAN Fu	444345 1c	1NVR	1WVY	2GDO	31.42		Sc=6.33465, min distance = 2.29125
DAPK1_HUMAN	153999 Ru	PDPK1_HUMAN F	444345 1c	1OKY	1WVY	1UU3	28.63	0.77	Sc=6.34342, min distance = 2.14183

DAPK1_HUMAN	1540 1h1q	CDK2_HUMAN Fu	444345 1c	1AQ1	1WVY	1H1Q	29.17	Sc=6.19914, min distance = 2.71282
DAPK1_HUMAN	1587957 2	CHK1_HUMAN Fu	444345 1c	1NVR	1WVY	2BRB	31.42	Sc=6.0402, min distance = 2.264129
DAPK1_HUMAN	15991572	LCK_HUMAN Ful	444345 1c	1QPD	1WVY	2OF4	29.1	Sc=6.21975, min distance = 2.36523
DAPK1_HUMAN	160355 r	CDK2_HUMAN Fu	444345 1c	1AQ1	1WVY	3DDQ	29.17	Sc=6.12385, min distance = 2.17791
DAPK1_HUMAN	16122633	KAPCA_BOVIN F	444345 1c	1STC	1WVY	2UW0	29.63	Sc=6.43405, min distance = 2.17404
DAPK1_HUMAN	16122643	CHK1_HUMAN Fu	444345 1c	1NVR	1WVY	2YWP	31.42	Sc=6.23346, min distance = 2.71011
DAPK1_HUMAN	16214823	CDK2_HUMAN Fu	444345 1c	1AQ1	1WVY	2UZB	29.17	Sc=6.04752, min distance = 2.62599
DAPK1_HUMAN	16214826	CDK2_HUMAN Fu	444345 1c	1AQ1	1WVY	2UZL	29.17	Sc=6.07509, min distance = 2.24107
DAPK1_HUMAN	16214827	CDK2_HUMAN Fu	444345 1c	1AQ1	1WVY	2UZN	29.17	Sc=5.99895, min distance = 3.14923
DAPK1_HUMAN	16758227	CHK1_HUMAN Fu	444345 1c	1NVR	1WVY	2R0U	31.42	Sc=6.45766, min distance = 2.27947
DAPK1_HUMAN	1707 1fvt	CDK2_HUMAN Fu	444345 1c	1AQ1	1WVY	1FVT	29.17	Sc=5.87826, min distance = 2.50835
DAPK1_HUMAN	17751819	KAPCA_BOVIN F	444345 1c	1STC	1WVY	2VO6	29.63	Sc=6.25253, min distance = 2.53949
DAPK1_HUMAN	1907917 2	CHK1_HUMAN Fu	444345 1c	1NVR	1WVY	2CGW	31.42	Sc=6.02656, min distance = 2.45142
DAPK1_HUMAN	23653515	CDK2_HUMAN Fu	444345 1c	1AQ1	1WVY	2R3F	29.17	Sc=5.82426, min distance = 2.13437
DAPK1_HUMAN	23653516	CDK2_HUMAN Fu	444345 1c	1AQ1	1WVY	2R3G	29.17	Sc=6.05196, min distance = 3.03947
DAPK1_HUMAN	23653518	CDK2_HUMAN Fu	444345 1c	1AQ1	1WVY	2R3J	29.17	Sc=6.03521, min distance = 2.19124
DAPK1_HUMAN	23653520	CDK2_HUMAN Fu	444345 1c	1AQ1	1WVY	2R3L	29.17	Sc=6.03521, min distance = 2.14118
DAPK1_HUMAN	23653522	CDK2_HUMAN Fu	444345 1c	1AQ1	1WVY	2R3N	29.17	Sc=6.09126, min distance = 2.17479
DAPK1_HUMAN	23653523	CDK2_HUMAN Fu	444345 1c	1AQ1	1WVY	2R3O	29.17	Sc=6.16874, min distance = 2.50077
DAPK1_HUMAN	23653524	CDK2_HUMAN Fu	444345 1c	1AQ1	1WVY	2R3P	29.17	Sc=6.15824, min distance = 2.04438
DAPK1_HUMAN	23657800	CHK1_HUMAN Fu	444345 1c	1NVR	1WVY	2E9P	31.42	Sc=6.2161, min distance = 2.250047
DAPK1_HUMAN	23727982	CDK2_HUMAN Fu	444345 1c	1AQ1	1WVY	3BHU	29.17	Sc=6.23346, min distance = 2.66286
DAPK1_HUMAN	24762195	KAPCA_BOVIN F	444345 1c	1STC	1WVY	2VO0	29.63	Sc=6.27341, min distance = 2.35014
DAPK1_HUMAN	24851689	PIM1_HUMAN Fu	444345 1c	1YHS	1WVY	3CY2	30.51	Sc=6.15314, min distance = 2.50408
DAPK1_HUMAN	24864081	CDK2_HUMAN Fu	444345 1c	1AQ1	1WVY	2VTS	29.17	Sc=6.21287, min distance = 2.29345
DAPK1_HUMAN	24905143	PK3CG_HUMAN F	444345 1c	1E8Z	1WVY	2V4L		Sc=5.95282, min distance = 2.77692
DAPK1_HUMAN	24916751	CDK2_HUMAN Fu	444345 1c	1AQ1	1WVY	3DOG	29.17	Sc=6.12114, min distance = 1.99672
DAPK1_HUMAN	24963033	CDK2_HUMAN Fu	444345 1c	1AQ1	1WVY	2W05	29.17	Sc=6.1103, min distance = 3.241916
DAPK1_HUMAN	3064778 H	ROCK1_HUMAN F	33113 gam	2V55	1JKL	2ETK	27.8	Sc=6.11002, min distance = 1.90536
DAPK1_HUMAN	3543 1g5s	CDK2_HUMAN Fu	444345 1c	1AQ1	1WVY	1G5S	29.17	Sc=6.39669, min distance = 2.23244
DAPK1_HUMAN	3547 Fasu	KAPCA_BOVIN F	444345 1c	1STC	1WVY	1Q8W	29.63	Sc=5.86942, min distance = 2.36203
DAPK1_HUMAN	3547 Fasu	ROCK1_HUMAN F	33113 gam	2V55	1JKL	2ESM	27.8	Sc=5.99815, min distance = 2.34403
DAPK1_HUMAN	3973 nche	PIM1_HUMAN Fu	444345 1c	1YHS	1WVY	1YI3	30.51	Sc=5.86387, min distance = 2.88165
DAPK1_HUMAN	4369136 C	CDK2_HUMAN Fu	444345 1c	1AQ1	1WVY	1DM2	29.17	Sc=6.15759, min distance = 2.89846
DAPK1_HUMAN	4369433 2	CHK1_HUMAN Fu	444345 1c	1NVR	1WVY	2BRM	31.42	Sc=6.15333, min distance = 2.26971
DAPK1_HUMAN	4369434 2	CHK1_HUMAN Fu	444345 1c	1NVR	1WVY	2BRN	31.42	Sc=6.29433, min distance = 2.40897
DAPK1_HUMAN	445966 O	CDK2_HUMAN Fu	444345 1c	1AQ1	1WVY	1H0V	29.17	Sc=6.05663, min distance = 3.40996

DAPK1_HUMAN I	447654 CI	CDK2_HUMAN Ful	444345 1c	1AQ1	1WVY	1OIT	29.17		Sc=6.03283, min distance = 2.863786
DAPK1_HUMAN I	447655 1o	CDK2_HUMAN Ful	444345 1c	1AQ1	1WVY	1OIU	29.17		Sc=6.20648, min distance = 2.756785
DAPK1_HUMAN I	447766 1p	CDK2_HUMAN Ful	444345 1c	1AQ1	1WVY	1P2A	29.17		Sc=6.2363, min distance = 2.5137645
DAPK1_HUMAN I	448043 2q	KAPCA_BOVIN Fu	444345 1c	1STC	1WVY	1Q8U	29.63		Sc=5.95463, min distance = 2.547332
DAPK1_HUMAN I	448043 2q	ROCK1_HUMAN Fu	33113 gam	2V55	1JKL	3D9V	27.8		Sc=6.04643, min distance = 1.915842
DAPK1_HUMAN I	449240 1y	KAPCA_BOVIN Fu	444345 1c	1STC	1WVY	1YDR	29.63		Sc=5.87641, min distance = 2.452882
DAPK1_HUMAN I	4565 1h1r	CDK2_HUMAN Ful	444345 1c	1AQ1	1WVY	1H1R	29.17		Sc=6.20093, min distance = 2.898452
DAPK1_HUMAN I	4566 1h1s	CDK2_HUMAN Ful	444345 1c	1AQ1	1WVY	1H1S	29.17		Sc=6.19165, min distance = 2.836603
DAPK1_HUMAN I	5288711 1	CDK2_HUMAN Ful	444345 1c	1AQ1	1WVY	1KE8	29.17		Sc=6.18248, min distance = 2.531599
DAPK1_HUMAN I	5288712 1	CDK2_HUMAN Ful	444345 1c	1AQ1	1WVY	1KE9	29.17		Sc=6.19182, min distance = 2.645687
DAPK1_HUMAN I	5326637 1	CSK2A_MAIZE Fu	33113 gam	1LP4	1JKL	1M2Q	25.58		Sc=6.23825, min distance = 2.263647
DAPK1_HUMAN I	5327121 I	PIM1_HUMAN Ful	444345 1c	1YHS	1WVY	2C3I	30.51		Sc=6.11773, min distance = 2.344271
DAPK1_HUMAN I	5327123 I	CHK1_HUMAN Ful	444345 1c	1NVR	1WVY	2C3L	31.42		Sc=6.03968, min distance = 2.760464
DAPK1_HUMAN I	5327133 C	CDK2_HUMAN Ful	444345 1c	1AQ1	1WVY	2C6K	29.17		Sc=6.01718, min distance = 2.552163
DAPK1_HUMAN I	5494414 C	CDK2_HUMAN Ful	444345 1c	1AQ1	1WVY	2A0C	29.17		Sc=6.2204, min distance = 2.2389903
DAPK1_HUMAN I	5687 1di8	CDK2_HUMAN Ful	444345 1c	1AQ1	1WVY	1DI8	29.17		Sc=6.17196, min distance = 2.409816
DAPK1_HUMAN I	6022 Aden	CDK2_HUMAN Ful	444345 1c	1AQ1	1WVY	1GY3	29.17		Sc=5.85576, min distance = 2.365169
DAPK1_HUMAN I	6022 Aden	GSK3B_HUMAN Fu	444345 1c	1Q3D	1WVY	1J1C	27.71		Sc=5.90816, min distance = 1.853919
DAPK1_HUMAN I	6083 aden	PIM1_HUMAN Ful	33113 gam	1XR1	1JKL	1YXU	30.51	0.97	Sc=6.40987, min distance = 2.071248
DAPK1_HUMAN I	6102670	CDK2_HUMAN Ful	444345 1c	1AQ1	1WVY	1YKR	29.17		Sc=6.18658, min distance = 2.137185
DAPK1_HUMAN I	6420138 2	CDK2_HUMAN Ful	444345 1c	1AQ1	1WVY	2UUE	29.17		Sc=6.21699, min distance = 2.242782
DAPK1_HUMAN I	6420139 C	CDK2_HUMAN Ful	444345 1c	1AQ1	1WVY	2C5V	29.17		Sc=6.04155, min distance = 2.653323
DAPK1_HUMAN I	6539118 g	CDK2_HUMAN Ful	444345 1c	1AQ1	1WVY	1FVV	29.17		Sc=6.31009, min distance = 2.425366
DAPK1_HUMAN I	6852207 M	KAPCA_BOVIN Fu	444345 1c	1STC	1WVY	2GNI	29.63		Sc=5.85688, min distance = 2.569826
DAPK1_HUMAN I	72271 7-H	CDK2_HUMAN Ful	444345 1c	1AQ1	1WVY	1PKD	29.17	0.91	Sc=6.437, min distance = 2.3670675
DAPK1_HUMAN I	91532 AMP	KIF1A_MOUSE Fu	33113 gam	1VFV	1JKL	1I6I		0.99	Sc=6.05094, min distance = 2.111004
DAPK1_HUMAN I	9547890 I	CDK2_HUMAN Ful	444345 1c	1AQ1	1WVY	1W8C	29.17		Sc=6.13389, min distance = 2.379721
DAPK1_HUMAN I	9817550 V	CDK2_HUMAN Ful	444345 1c	1AQ1	1WVY	3BHV	29.17		Sc=6.35382, min distance = 2.209645
DCDA_ECOLI Fu	6274 hist	ARGT_SALTY Ful	5962 L-ly	1LST	1KO0	1LAG			Sc=5.7261, min distance = 2.5160526
DCDA_METJA Fu	6274 hist	ARGT_SALTY Ful	5962 L-ly	1LST	1TWI	1LAG			Sc=5.73348, min distance = 2.438442
DCDA_METJA Fu	6322 L-ar	ARGT_SALTY Ful	5962 L-ly	1LST	1TWI	1LAF		0.79	Sc=5.83287, min distance = 2.414796
DCD_ECOLI Ful	16741197	DUT_ECOLI Full	65070 dUT	1SYL	1XS1	2HRM		0.98	Sc=5.91989, min distance = 2.270480
DCHM_BPT4 Ful	454194 Tr	DCK_HUMAN Full	13945 Dec	2QRN	1B5E	2NO9			Sc=5.88917, min distance = 2.302176
DCHM_BPT4 Ful	60825 lam	DCK_HUMAN Full	13945 Dec	2QRN	1B5E	2NOA			Sc=5.87663, min distance = 2.384236
DCHM_BPT4 Ful	60877 Emt	DCK_HUMAN Full	13945 Dec	2QRN	1B5E	2NO6			Sc=5.91376, min distance = 2.286926
DCIP_ENTCL Fu	124687 1r	ODP1_ECOLI Ful	1132 thia	1L8A	1OVM	1RP7		0.79	Sc=6.01185, min distance = 2.331350
DCIP_ENTCL Fu	13294447	DCIP_AZOBR Ful	1132 thia	2NXW	1OVM	2Q5Q			Sc=6.07906, min distance = 0.852237

DCIP_ENTCL Fu	444421 CI	MDLC_PSEPU Fu	1132 thia	1BFD	1OVM	2FWN		0.82	Sc=6.40659, min distance = 2.29105(
DCIP_ENTCL Fu	448671 2-	ILVB_YEAST Fu	1132 thia	1N0H	1OVM	1T9B			Sc=5.83321, min distance = 2.80874!
DCIP_ENTCL Fu	448673 PE	ILVB_YEAST Fu	1132 thia	1N0H	1OVM	1T9B			Sc=5.82406, min distance = 2.42794(
DCIP_ENTCL Fu	448721 N3	TKT1_YEAST Fu	1132 thia	1TRK	1OVM	1TKA			Sc=6.43887, min distance = 1.489098
DCIP_ENTCL Fu	448722 1t	TKT1_YEAST Fu	1132 thia	1TRK	1OVM	1TKB		0.78	Sc=6.44149, min distance = 2.22240!
DCIP_ENTCL Fu	6102647 C	ODBA_HUMAN Fu	1132 thia	2BFD	1OVM	1WCI		0.9	Sc=6.12169, min distance = 2.08829:
DCIP_ENTCL Fu	6518182 C	POXB_LACPL Fu	1132 thia	2EZ4	1OVM	2EZ9		0.94	Sc=5.7924, min distance = 2.460527:
DCK_HUMAN Fu	1540 1h1q	CDK2_HUMAN Fu	6022 Aden	1GY3	1P5Z	1H1Q			Sc=6.32438, min distance = 2.18200(
DCK_HUMAN Fu	1707 1fvt	CDK2_HUMAN Fu	6022 Aden	1GY3	1P5Z	1FVT			Sc=6.05533, min distance = 1.97399:
DCK_HUMAN Fu	171548 bi	AVID_CHICK Fu	187790 de	2A8G	2ZI7	2JGS			Sc=6.29605, min distance = 1.01088!
DCK_HUMAN Fu	23653515	CDK2_HUMAN Fu	6022 Aden	1GY3	1P5Z	2R3F			Sc=5.82684, min distance = 1.894618
DCK_HUMAN Fu	23653518	CDK2_HUMAN Fu	6022 Aden	1GY3	1P5Z	2R3J			Sc=6.06255, min distance = 2.03818
DCK_HUMAN Fu	23653522	CDK2_HUMAN Fu	6022 Aden	1GY3	1P5Z	2R3N			Sc=6.25558, min distance = 2.17355!
DCK_HUMAN Fu	23653523	CDK2_HUMAN Fu	6022 Aden	1GY3	1P5Z	2R3O			Sc=6.30983, min distance = 2.41949(
DCK_HUMAN Fu	23653526	CDK2_HUMAN Fu	6022 Aden	1GY3	1P5Z	2R3R			Sc=6.08616, min distance = 2.30511:
DCK_HUMAN Fu	24066 zal	DNK_DROME Ful	13711 dec	2VP5	2NO1	2VP9	37.36	0.96	Sc=6.04399, min distance = 1.38127
DCK_HUMAN Fu	24864080	CDK2_HUMAN Fu	6022 Aden	1GY3	1P5Z	2VTR			Sc=5.93544, min distance = 2.40533!
DCK_HUMAN Fu	2608 1jsv	CDK2_HUMAN Fu	6022 Aden	1GY3	1P5Z	1JSV			Sc=5.88645, min distance = 2.75241:
DCK_HUMAN Fu	444564 AD	BIOD_ECOLI Fu	6022 Aden	1DAD	1P5Z	1BS1		0.91	Sc=5.97221, min distance = 2.26912:
DCK_HUMAN Fu	444564 AD	MYS2_DICDI Fu	6022 Aden	1VOM	1P5Z	1W9I		0.91	Sc=5.65072, min distance = 2.32764!
DCK_HUMAN Fu	445840 di	CDK2_HUMAN Fu	6022 Aden	1GY3	1P5Z	1GII			Sc=6.21249, min distance = 1.708748
DCK_HUMAN Fu	445841 py	CDK2_HUMAN Fu	6022 Aden	1GY3	1P5Z	1GIJ			Sc=6.24644, min distance = 2.18163
DCK_HUMAN Fu	446704 LS	CDK2_HUMAN Fu	6022 Aden	1GY3	1P5Z	1KE6			Sc=6.19776, min distance = 2.02451(
DCK_HUMAN Fu	446903 NC	AVID_CHICK Fu	187790 de	2A8G	2ZI7	1LDO			Sc=6.06612, min distance = 2.39094
DCK_HUMAN Fu	446904 HC	AVID_CHICK Fu	187790 de	2A8G	2ZI7	1LDQ			Sc=6.21774, min distance = 2.30561:
DCK_HUMAN Fu	447655 1c	CDK2_HUMAN Fu	6022 Aden	1GY3	1P5Z	1OIU			Sc=6.34032, min distance = 2.63896
DCK_HUMAN Fu	447766 1p	CDK2_HUMAN Fu	6022 Aden	1GY3	1P5Z	1P2A			Sc=6.28348, min distance = 1.60880!
DCK_HUMAN Fu	447821 CI	CDK2_HUMAN Fu	6022 Aden	1GY3	1P5Z	1PF8			Sc=5.97115, min distance = 2.02198!
DCK_HUMAN Fu	448378 al	APHA_ECOLI Fu	13711 dec	1RMY	2NO1	1RMT			Sc=6.32735, min distance = 1.43808
DCK_HUMAN Fu	4565 1h1r	CDK2_HUMAN Fu	6022 Aden	1GY3	1P5Z	1H1R			Sc=6.32903, min distance = 2.62490:
DCK_HUMAN Fu	4566 1h1s	CDK2_HUMAN Fu	6022 Aden	1GY3	1P5Z	1H1S			Sc=6.36597, min distance = 2.11414
DCK_HUMAN Fu	5287830 6	Q8RLY5_LACHE I	13730 dec	1S2G	2ZI3	1S2I			Sc=5.67266, min distance = 1.97104:
DCK_HUMAN Fu	5288016 6	CDK2_HUMAN Fu	6022 Aden	1GY3	1P5Z	2B53			Sc=6.23658, min distance = 1.94536(
DCK_HUMAN Fu	5288708 1	CDK2_HUMAN Fu	6022 Aden	1GY3	1P5Z	1KE5			Sc=6.07939, min distance = 1.90471:
DCK_HUMAN Fu	5326739 i	GSK3B_HUMAN Fu	6022 Aden	1J1C	1P5Z	1Q41			Sc=6.26018, min distance = 2.21463(
DCK_HUMAN Fu	5327097 2	CDK2_HUMAN Fu	6022 Aden	1GY3	1P5Z	2BTS			Sc=5.81956, min distance = 2.59982
DCK_HUMAN Fu	5327132 C	CDK2_HUMAN Fu	6022 Aden	1GY3	1P5Z	2C6I			Sc=6.18711, min distance = 2.34305!

DCK_HUMAN Fu	5327134 C	CDK2_HUMAN Fu	6022 Aden	1GY3	1P5Z	2C6L			Sc=6.29633, min distance = 2.48969
DCK_HUMAN Fu	5789 thym	DNK_DROME Ful	13711 dec	2VP5	2NO1	1OT3	37.36		Sc=6.21831, min distance = 1.57692
DCK_HUMAN Fu	5790 flox	DNK_DROME Ful	13711 dec	2VP5	2NO1	2VP6	37.36	0.75	Sc=6.15924, min distance = 1.61559
DCK_HUMAN Fu	5957 Aden	BIOD_ECOLI Fu	6022 Aden	1DAD	1P5Z	1A82		0.99	Sc=6.04117, min distance = 2.15778
DCK_HUMAN Fu	5957 Aden	MUTS_ECOLI Fu	6022 Aden	1OH7	1P5Z	1W7A		0.99	Sc=6.02091, min distance = 2.62100
DCK_HUMAN Fu	6083 aden	HSP71_HUMAN F	6022 Aden	1S3X	1P5Z	1XQS		0.99	Sc=5.8835, min distance = 2.182493
DCK_HUMAN Fu	656971 CI	CDK2_HUMAN Fu	6022 Aden	1GY3	1P5Z	1Y8Y			Sc=5.89885, min distance = 2.28115
DCK_HUMAN Fu	72194 2-C	ENPL_CANFA Fu	6022 Aden	1TC6	1P5Z	1QYE		0.89	Sc=6.12943, min distance = 1.99412
DCK_HUMAN Fu	73318 8-C	AVID_CHICK Fu	187790 de	2A8G	2ZI7	2A5B			Sc=6.3457, min distance = 2.063385
DCK_HUMAN Fu	9547890 I	CDK2_HUMAN Fu	6022 Aden	1GY3	1P5Z	1W8C			Sc=6.07058, min distance = 2.06093
DCK_HUMAN Fu	9991833 S	CDK2_HUMAN Fu	6022 Aden	1GY3	1P5Z	2R3H			Sc=5.76266, min distance = 2.40489
DCK_HUMAN Fu	9994066 4	CDK2_HUMAN Fu	6022 Aden	1GY3	1P5Z	2VTJ			Sc=5.81594, min distance = 2.30036
DCP_ECOLI Fu	199528 D-	SYW2_DEIRA Fu	6305 L-tr	1YI8	1Y79	1YIA		0.89	Sc=6.14068, min distance = 2.59740
DCP_ECOLI Fu	3081936 7	P95480_PSEFL	6305 L-tr	2AQJ	1Y79	2AR8		0.9	Sc=6.07591, min distance = 2.02118
DCP_ECOLI Fu	5962 L-ly	THER_BACTH Fu	6305 L-tr	1THL	1Y79	1KEI			Sc=5.67966, min distance = 2.24891
DCP_ECOLI Fu	60961 ade	Q8KHZ8_NOCAE	6305 L-tr	2E4G	1Y79	2OA1			Sc=5.86865, min distance = 2.46956
DCP_ECOLI Fu	6140 L-ph	THER_BACTH Fu	6305 L-tr	1THL	1Y79	1OS0			Sc=5.76419, min distance = 2.12773
DCP_ECOLI Fu	71567 D-p	APX_STRGR Ful	6305 L-tr	1TF8	1Y79	1TKH			Sc=5.81517, min distance = 2.17236
DCXR_HUMAN F	15942680	6PGD_LACLM Fu	5886 NADP	2IZ0	1PR9	2IZ1			Sc=6.26814, min distance = 1.93346
DCXR_HUMAN F	440141 9i	DHB1_HUMAN Fu	5886 NADP	1QYV	1PR9	1QYW			Sc=6.42696, min distance = 2.30816
DDH_CORGL Fu	16741136	DYR_ECOLI Ful	5886 NADP	1RA2	1DAP	1RX9	1		Sc=6.3484, min distance = 2.163850
DDLB_ECOLI F	11957393	FAK1_HUMAN Fu	6022 Aden	1MP8	1IOW	2ETM			Sc=6.38733, min distance = 2.47766
DDLB_ECOLI F	24864080	CDK2_HUMAN Fu	6022 Aden	1GY3	1IOW	2VTR			Sc=6.04652, min distance = 1.97786
DDLB_ECOLI F	24864081	CDK2_HUMAN Fu	6022 Aden	1GY3	1IOW	2VTS			Sc=6.38524, min distance = 1.92471
DDLB_ECOLI F	2608 1jsv	CDK2_HUMAN Fu	6022 Aden	1GY3	1IOW	1JSV			Sc=6.06998, min distance = 2.21981
DDLB_ECOLI F	444564 AD	BIOD_ECOLI Fu	6022 Aden	1DAD	1IOW	1BS1		0.91	Sc=6.46042, min distance = 2.19307
DDLB_ECOLI F	445940 O6	CDK2_HUMAN Fu	6022 Aden	1GY3	1IOW	1GZ8			Sc=6.17124, min distance = 1.88936
DDLB_ECOLI F	447654 CI	CDK2_HUMAN Fu	6022 Aden	1GY3	1IOW	1OIT			Sc=6.19842, min distance = 2.56985
DDLB_ECOLI F	447916 ac	RIO1_ARCFU Fu	6022 Aden	1ZTH	1IOW	1ZTF		0.95	Sc=6.26369, min distance = 2.67301
DDLB_ECOLI F	448222 NE	ENPL_CANFA Fu	6022 Aden	1TC6	1IOW	1QY5		0.81	Sc=6.40328, min distance = 1.87741
DDLB_ECOLI F	5327134 C	CDK2_HUMAN Fu	6022 Aden	1GY3	1IOW	2C6L			Sc=6.38626, min distance = 2.19626
DDLB_ECOLI F	5957 Aden	DDL_THET8 Ful	6022 Aden	2ZDH	1IOW	2ZDQ	36.94	0.99	Sc=6.4993, min distance = 2.247018
DDLB_ECOLI F	5957 Aden	MALK_ECOLI Fu	6022 Aden	2AWN	1IOW	1Q12		0.99	Sc=6.4879, min distance = 1.343595
DDLB_ECOLI F	6420140 2	CDK2_HUMAN Fu	6022 Aden	1GY3	1IOW	2C5Y			Sc=6.54774, min distance = 2.01989
DDLB_ECOLI F	656971 CI	CDK2_HUMAN Fu	6022 Aden	1GY3	1IOW	1Y8Y			Sc=6.06471, min distance = 1.94432
DDLB_ECOLI F	6804 guar	NDK_PYRHO Ful	6022 Aden	2DYA	1IOW	2DXF		0.8	Sc=6.40672, min distance = 2.20111
DDLB_ECOLI F	91532 AMP	MTNK_BACSU Fu	6022 Aden	2OLC	1IOW	2PUL		0.99	Sc=6.09754, min distance = 2.02165

DDLB_ECOLI Fu	9543433 T	NDKC_DICDI Fu	6022 Aden	1KDN	1IOW	1F6T			Sc=6.36947, min distance = 1.85027
DDLB_ECOLI Fu	9994066 4	CDK2_HUMAN Fu	6022 Aden	1GY3	1IOW	2VTJ			Sc=5.9851, min distance = 2.164820
DDL_LEUMM Fu	188966 dA	HSLU_ECOLI Fu	6022 Aden	1HQY	1EHI	1G4A		0.98	Sc=5.96375, min distance = 1.87607
DDL_LEUMM Fu	23653515	CDK2_HUMAN Fu	6022 Aden	1GY3	1EHI	2R3F			Sc=6.13765, min distance = 2.07769
DDL_LEUMM Fu	23653523	CDK2_HUMAN Fu	6022 Aden	1GY3	1EHI	2R3O			Sc=6.44493, min distance = 2.02765
DDL_LEUMM Fu	24864080	CDK2_HUMAN Fu	6022 Aden	1GY3	1EHI	2VTR			Sc=6.01468, min distance = 1.97495
DDL_LEUMM Fu	4369136 C	CDK2_HUMAN Fu	6022 Aden	1GY3	1EHI	1DM2			Sc=6.25549, min distance = 1.79740
DDL_LEUMM Fu	447916 ad	RIO1_ARCFU Fu	6022 Aden	1ZTH	1EHI	1ZTF		0.95	Sc=6.19256, min distance = 2.76314
DDL_LEUMM Fu	447955 1p	CDK2_HUMAN Fu	6022 Aden	1GY3	1EHI	1PXI			Sc=5.75952, min distance = 2.17307
DDL_LEUMM Fu	447967 CI	CDK2_HUMAN Fu	6022 Aden	1GY3	1EHI	1PYE			Sc=6.36335, min distance = 2.18420
DDL_LEUMM Fu	5327148 C	IPKA_RABIT Fu	6022 Aden	1JBP	1EHI	2ERZ			Sc=6.15834, min distance = 2.06935
DDL_LEUMM Fu	5687 1di8	CDK2_HUMAN Fu	6022 Aden	1GY3	1EHI	1DI8			Sc=6.24985, min distance = 2.27437
DDL_LEUMM Fu	6420140 2	CDK2_HUMAN Fu	6022 Aden	1GY3	1EHI	2C5Y			Sc=6.51886, min distance = 2.00210
DDL_LEUMM Fu	9543438 C	DDLB_ECOLI Fu	6022 Aden	1IOW	1EHI	1IOV			Sc=5.99842, min distance = 2.05234
DDL_LEUMM Fu	9543438 C	DDLB_ECOLI Fu	9543432 1	2DLN	1EHI	1IOV			Sc=5.98647, min distance = 2.76231
DDL_LEUMM Fu	9547890 I	CDK2_HUMAN Fu	6022 Aden	1GY3	1EHI	1W8C			Sc=6.27374, min distance = 2.32812
DDL_STAAC Fu	24864080	CDK2_HUMAN Fu	6022 Aden	1GY3	2I8C	2VTR			Sc=5.98094, min distance = 1.98903
DDL_STAAC Fu	3454 gand	KITH_HHV11 Fu	6022 Aden	2VTK	2I8C	1KI2			Sc=6.13456, min distance = 2.67702
DDL_STAAC Fu	4369136 C	CDK2_HUMAN Fu	6022 Aden	1GY3	2I8C	1DM2			Sc=6.25818, min distance = 1.88445
DDL_STAAC Fu	444564 AD	BIOD_ECOLI Fu	6022 Aden	1DAD	2I8C	1BS1		0.91	Sc=6.00576, min distance = 2.18710
DDL_STAAC Fu	447821 CI	CDK2_HUMAN Fu	6022 Aden	1GY3	2I8C	1PF8			Sc=6.12077, min distance = 1.93029
DDL_STAAC Fu	447916 ad	RIO1_ARCFU Fu	6022 Aden	1ZTH	2I8C	1ZTF		0.95	Sc=6.16533, min distance = 2.75333
DDL_STAAC Fu	448222 NE	ENPL_CANFA Fu	6022 Aden	1TC6	2I8C	1QY5		0.81	Sc=5.93094, min distance = 2.50523
DDL_STAAC Fu	448310 CI	KIPN_BPT4 Ful	6022 Aden	1LTQ	2I8C	1RC8			Sc=5.77261, min distance = 2.37671
DDL_STAAC Fu	4725 penc	KITH_HHV11 Fu	6022 Aden	2VTK	2I8C	1KI3			Sc=6.17584, min distance = 2.50599
DDL_STAAC Fu	5327104 2	HS90A_HUMAN Fu	6022 Aden	1BYQ	2I8C	2BYI			Sc=6.44651, min distance = 2.11468
DDL_STAAC Fu	5327148 C	IPKA_RABIT Fu	6022 Aden	1JBP	2I8C	2ERZ			Sc=6.0683, min distance = 2.190253
DDL_STAAC Fu	5957 Aden	RK_BOVIN Full	6022 Aden	3C4Z	2I8C	3C4W		0.99	Sc=5.93778, min distance = 2.12697
DDL_STAAC Fu	5957 Aden	Y059_METJA Fu	6022 Aden	2J9D	2I8C	2J9C		0.99	Sc=5.93058, min distance = 2.03446
DDL_STAAC Fu	6102787 2	HS90A_HUMAN Fu	6022 Aden	1BYQ	2I8C	2CCS			Sc=6.28349, min distance = 1.78512
DDL_STAAC Fu	6804 guar	NDK_PYRHO Ful	6022 Aden	2DYA	2I8C	2DXF		0.8	Sc=6.29104, min distance = 2.24319
DDL_THET8 Fu	11608401	KAPCA_BOVIN Fu	5957 Aden	1Q24	2ZDQ	2UVY			Sc=6.28807, min distance = 2.25244
DDL_THET8 Fu	11708454	KAPCA_BOVIN Fu	5957 Aden	1Q24	2ZDQ	2VNW			Sc=6.19686, min distance = 2.36543
DDL_THET8 Fu	11957393	FAK1_HUMAN Fu	6022 Aden	1MP8	2ZDH	2ETM			Sc=6.41812, min distance = 1.87328
DDL_THET8 Fu	16058649	IGF1R_HUMAN Fu	33113 gam	1JQH	2YZN	2OJ9			Sc=6.5498, min distance = 1.648805
DDL_THET8 Fu	16122607	PDPK1_HUMAN Fu	5957 Aden	2BIY	2ZDQ	2PE0			Sc=6.22671, min distance = 2.20003
DDL_THET8 Fu	16750062	CSK2A_MAIZE Fu	33113 gam	1LP4	2YZN	2OXD			Sc=5.7126, min distance = 2.123743

DDL_THET8 Fu	188966 dA	HSLU_ECOLI Fu	33113 gam	1E94	2YZN	1G4A		0.97	Sc=6.01488, min distance = 2.13493
DDL_THET8 Fu	23653515	CDK2_HUMAN Fu	6022 Aden	1GY3	2ZDH	2R3F			Sc=6.16736, min distance = 1.68981
DDL_THET8 Fu	24779674	CSK2A_MAIZE F	33113 gam	1LP4	2YZN	2PVH			Sc=6.14399, min distance = 2.78058
DDL_THET8 Fu	24864080	CDK2_HUMAN Fu	6022 Aden	1GY3	2ZDH	2VTR			Sc=6.10555, min distance = 1.92165
DDL_THET8 Fu	2608 1jsv	CDK2_HUMAN Fu	6022 Aden	1GY3	2ZDH	1JSV			Sc=6.13078, min distance = 1.48238
DDL_THET8 Fu	3547 Fasu	KAPCA_BOVIN F	5957 Aden	1Q24	2ZDQ	1Q8W			Sc=6.00767, min distance = 2.31621
DDL_THET8 Fu	3547 Fasu	ROCK1_HUMAN F	33113 gam	2V55	2YZN	2ESM			Sc=5.95705, min distance = 2.03300
DDL_THET8 Fu	36735 Gpp	Q9RVK2_DEIRA	5957 Aden	1SU2	2ZDQ	1SZ3		0.8	Sc=6.51394, min distance = 1.88197
DDL_THET8 Fu	440317 AT	PURT_ECOLI Fu	5957 Aden	1KJ8	2ZDQ	1KJJ		0.99	Sc=6.5107, min distance = 1.997358
DDL_THET8 Fu	440317 AT	PURT_ECOLI Fu	6022 Aden	1KJQ	2ZDH	1KJJ		0.99	Sc=6.52324, min distance = 1.85390
DDL_THET8 Fu	444564 AD	MYS2_DICDI Fu	33113 gam	1MMN	2YZN	1W9I		0.91	Sc=6.0266, min distance = 2.130198
DDL_THET8 Fu	444674 CI	ACCC_ECOLI Fu	6022 Aden	2J9G	2ZDH	2VR1	27.78	0.96	Sc=5.84635, min distance = 1.60404
DDL_THET8 Fu	444842 CI	CDK2_HUMAN Fu	6022 Aden	1GY3	2ZDH	1CKP			Sc=5.6386, min distance = 2.214573
DDL_THET8 Fu	447840 CI	PHKG1_RABIT F	5957 Aden	1QL6	2ZDQ	1PHK			Sc=6.0712, min distance = 2.476023
DDL_THET8 Fu	447916 ac	RIO1_ARCFU Fu	6022 Aden	1ZTH	2ZDH	1ZTF		0.95	Sc=6.30124, min distance = 2.37541
DDL_THET8 Fu	447955 1p	CDK2_HUMAN Fu	6022 Aden	1GY3	2ZDH	1PXI			Sc=5.84554, min distance = 2.05463
DDL_THET8 Fu	448042 2e	ROCK1_HUMAN F	33113 gam	2V55	2YZN	2ETR			Sc=6.16445, min distance = 1.46407
DDL_THET8 Fu	448043 2g	KAPCA_BOVIN F	5957 Aden	1Q24	2ZDQ	1Q8U			Sc=6.07427, min distance = 2.07758
DDL_THET8 Fu	448043 2g	ROCK1_HUMAN F	33113 gam	2V55	2YZN	3D9V			Sc=6.01162, min distance = 1.91287
DDL_THET8 Fu	448222 NE	ENPL_CANFA Fu	6022 Aden	1TC6	2ZDH	1QY5		0.81	Sc=6.44225, min distance = 1.50602
DDL_THET8 Fu	449240 1y	KAPCA_BOVIN F	5957 Aden	1Q24	2ZDQ	1YDR			Sc=6.01664, min distance = 2.17014
DDL_THET8 Fu	5326739 i	GSK3B_HUMAN F	33113 gam	1J1B	2YZN	1Q41			Sc=6.29151, min distance = 1.59768
DDL_THET8 Fu	5326976 1	CSK2A_MAIZE F	33113 gam	1LP4	2YZN	1ZOE			Sc=5.74162, min distance = 2.04880
DDL_THET8 Fu	5326978 1	CSK2A_MAIZE F	33113 gam	1LP4	2YZN	1ZOH			Sc=5.6724, min distance = 2.026166
DDL_THET8 Fu	5327097 2	CDK2_HUMAN Fu	6022 Aden	1GY3	2ZDH	2BTS			Sc=5.99134, min distance = 0.91912
DDL_THET8 Fu	5327148 C	IPKA_RABIT Fu	6022 Aden	1JBP	2ZDH	2ERZ			Sc=6.20425, min distance = 2.35714
DDL_THET8 Fu	6083 aden	AAKG1_RAT Ful	5957 Aden	2V92	2ZDQ	2V8Q		0.98	Sc=6.39746, min distance = 1.95422
DDL_THET8 Fu	6083 aden	ASSY_THET8 Fu	33113 gam	1KOR	2YZN	1J20		0.97	Sc=6.31583, min distance = 0.45448
DDL_THET8 Fu	6083 aden	ENTP2_RAT Ful	33113 gam	3CJA	2YZN	3CJ7		0.97	Sc=6.26529, min distance = 1.96874
DDL_THET8 Fu	6083 aden	PSPF_ECOLI Fu	6022 Aden	2C98	2ZDH	2VII		0.99	Sc=6.38367, min distance = 1.84634
DDL_THET8 Fu	6083 aden	PURP_METJA Fu	6022 Aden	2R7N	2ZDH	2R7M		0.99	Sc=6.41112, min distance = 1.80022
DDL_THET8 Fu	6083 aden	PURP_PYRFU Fu	5957 Aden	2R86	2ZDQ	2R85		0.98	Sc=6.39382, min distance = 2.59080
DDL_THET8 Fu	6083 aden	Q6L8F0_THETH	33113 gam	1V25	2YZN	1V26		0.97	Sc=6.29641, min distance = 1.85745
DDL_THET8 Fu	60961 ade	IPKA_RABIT Fu	6022 Aden	1JBP	2ZDH	1FMO		0.95	Sc=6.28066, min distance = 1.96923
DDL_THET8 Fu	656971 CI	CDK2_HUMAN Fu	6022 Aden	1GY3	2ZDH	1Y8Y			Sc=6.13046, min distance = 1.22825
DDL_THET8 Fu	91532 AMP	MTNK_BACSU Fu	6022 Aden	2OLC	2ZDH	2PUL		0.99	Sc=6.55253, min distance = 1.70274
DDL_THET8 Fu	9543432 1	DDLB_ECOLI Fu	6022 Aden	1IOW	2ZDH	2DLN	36.94		Sc=5.97755, min distance = 2.34698

DDL_THET8 Fu	9543438 C	DDLB_ECOLI Fu	6022 Aden	1IOW	2ZDH	1IOV	36.94		Sc=6.00663, min distance = 2.34629
DDL_THET8 Fu	9547890 I	CDK2_HUMAN Fu	6022 Aden	1GY3	2ZDH	1W8C			Sc=6.33008, min distance = 1.96852
DDX10_HUMAN I	4369136 C	CDK2_HUMAN Fu	6022 Aden	1GY3	2PL3	1DM2			Sc=6.24474, min distance = 1.69134
DDX10_HUMAN I	447004 CI	MYS2_DICDI Fu	6022 Aden	1VOM	2PL3	1LVK			Sc=5.74414, min distance = 2.33640
DDX20_HUMAN I	24779674	CSK2A_MAIZE Fu	33113 gam	1LP4	3B7G	2PVH			Sc=6.2186, min distance = 1.942543
DDX20_HUMAN I	24779676	CSK2A_MAIZE Fu	33113 gam	1LP4	3B7G	2PVK			Sc=6.3533, min distance = 2.148365
DDX20_HUMAN I	3064778 H	ROCK1_HUMAN Fu	33113 gam	2V55	3B7G	2ETK			Sc=6.08409, min distance = 2.24643
DDX20_HUMAN I	3547 Fasu	ROCK1_HUMAN Fu	33113 gam	2V55	3B7G	2ESM			Sc=5.71039, min distance = 1.94789
DDX20_HUMAN I	4369136 C	CDK2_HUMAN Fu	6022 Aden	1GY3	2OXC	1DM2			Sc=6.28626, min distance = 1.73218
DDX20_HUMAN I	448042 2e	ROCK1_HUMAN Fu	33113 gam	2V55	3B7G	2ETR			Sc=6.16079, min distance = 2.01821
DDX20_HUMAN I	6083 aden	Q6L8F0_THETH I	33113 gam	1V25	3B7G	1V26		0.97	Sc=5.79819, min distance = 2.66671
DDX3X_HUMAN I	15342951	PYGM_RABIT Fu	6083 aden	8GPB	2I4I	3BCR			Sc=5.98279, min distance = 2.01585
DDX47_HUMAN I	15342951	PYGM_RABIT Fu	6083 aden	8GPB	3BER	3BCR			Sc=6.10029, min distance = 2.13812
DDX47_HUMAN I	5957 Aden	PURP_PYRFU Fu	6083 aden	2R85	3BER	2R86		0.98	Sc=6.0217, min distance = 2.282852
DDX47_HUMAN I	656966 1y	PDE4D_HUMAN Fu	6083 aden	1TB7	3BER	1Y2E			Sc=5.94848, min distance = 2.01848
DDX47_HUMAN I	656969 1y	PDE4B_HUMAN Fu	6083 aden	1ROR	3BER	1Y2J			Sc=6.08885, min distance = 1.63264
DDX47_HUMAN I	657135 Op	PIM1_HUMAN Fu	6083 aden	1YXU	3BER	1YXV			Sc=5.85344, min distance = 1.88728
DDX47_HUMAN I	8582 Inos	PYGM_RABIT Fu	6083 aden	8GPB	3BER	2QN7		0.79	Sc=5.89926, min distance = 2.41126
DDX52_HUMAN I	2608 1jsv	CDK2_HUMAN Fu	6022 Aden	1GY3	3DKP	1JSV			Sc=5.91939, min distance = 2.16228
DEF1_BACCR Fu	159596 1g	DEF_ECOLI Ful	443600 ac	1LRU	1WS1	1G27			Sc=6.35812, min distance = 2.33454
DEF1_BACCR Fu	448475 1s	DEF_PSEAE Ful	443600 ac	1LRY	1WS1	1S17			Sc=6.18656, min distance = 2.16617
DEF2_BACCR Fu	448475 1s	DEF_PSEAE Ful	445753 1g	1IX1	2OKL	1S17			Sc=6.17344, min distance = 2.56546
DEF2_BACST Fu	448475 1s	DEF_PSEAE Ful	445753 1g	1IX1	1LQY	1S17			Sc=6.15756, min distance = 2.74800
DEF_ECOLI Fu	448475 1s	DEF_PSEAE Ful	443600 ac	1LRY	1LRU	1S17	57.76		Sc=6.19578, min distance = 2.62584
DEF_ECOLI Fu	448475 1s	DEF_PSEAE Ful	445753 1g	1IX1	1G2A	1S17	57.76		Sc=6.18118, min distance = 2.56327
DEF_ENTFA Fu	159596 1g	DEF_ECOLI Ful	445753 1g	1G2A	2OS1	1G27		0.79	Sc=6.3161, min distance = 2.365864
DEF_ENTFA Fu	448475 1s	DEF_PSEAE Ful	445753 1g	1IX1	2OS1	1S17			Sc=6.16304, min distance = 2.53892
DEF_LEPIN Fu	159596 1g	DEF_ECOLI Ful	445753 1g	1G2A	1SZZ	1G27	37.65	0.79	Sc=6.362, min distance = 1.436302
DEF_LEPIN Fu	16131873	DEF_ECOLI Ful	445753 1g	1G2A	1SZZ	1BSJ	37.65		Sc=6.50438, min distance = 2.05876
DEF_LEPIN Fu	164795 N(	RBCMT_PEA Ful	23831 HEP	1MLV	1VEY	2H2J			Sc=5.86523, min distance = 2.08979
DEF_LEPIN Fu	443600 ac	DEF_ECOLI Ful	445753 1g	1G2A	1SZZ	1LRU	37.65	0.87	Sc=6.50484, min distance = 2.43183
DEF_LEPIN Fu	443600 ac	DEF_PSEAE Ful	445753 1g	1IX1	1SZZ	1LRY	42.24	0.87	Sc=6.50956, min distance = 2.50931
DEF_LEPIN Fu	447768 CI	PURR_BACSU Fu	23831 HEP	1O57	1VEY	1P4A			Sc=5.78098, min distance = 1.54749
DEF_LEPIN Fu	448475 1s	DEF_PSEAE Ful	445753 1g	1IX1	1SZZ	1S17	42.24		Sc=6.14629, min distance = 2.51642
DEF_LEPIN Fu	5962 L-ly	RBCMT_PEA Ful	23831 HEP	1MLV	1VEY	2H2E			Sc=5.76044, min distance = 2.18983
DEF_LEPIN Fu	854023 Ep	Q8WSF8_APLCA I	23831 HEP	2BR7	1VEY	2BYQ			Sc=5.88845, min distance = 1.58877
DEF_PSEAE Fu	159596 1g	DEF_ECOLI Ful	443600 ac	1LRU	1LRY	1G27	57.76		Sc=6.32842, min distance = 2.27217

DEF_PSEAE Fu	159596 1g	DEF_ECOLI Ful	445753 1g	1G2A	1IX1	1G27	57.76	0.79	Sc=6.38236, min distance = 1.75880
DEF_STAAU Fu	159596 1g	DEF_ECOLI Ful	445753 1g	1G2A	1Q1Y	1G27		0.79	Sc=6.31704, min distance = 2.17678
DEF_STAAU Fu	16131873	DEF_ECOLI Ful	445753 1g	1G2A	1Q1Y	1BSJ			Sc=6.5215, min distance = 2.033355
DEF_STAAU Fu	448475 1s	DEF_PSEAE Ful	445753 1g	1IX1	1Q1Y	1S17			Sc=6.12146, min distance = 2.18801
DEF_STRP1 Fu	448475 1s	DEF_PSEAE Ful	445753 1g	1IX1	2OS3	1S17			Sc=6.15872, min distance = 2.69399
DEF_STRR6 Fu	159596 1g	DEF_ECOLI Ful	5327016 S	2AI8	2AI7	1G27	35		Sc=6.40254, min distance = 0.83004
DEOD_BACAN F	10041129	SAHH_MYCTU Fu	60961 ade	3CE6	2AC7	3DHY		0.89	Sc=6.30081, min distance = 2.27233
DEOD_BACAN F	24180721	PIM1_HUMAN Fu	60961 ade	1YI4	2AC7	3C4E			Sc=5.99648, min distance = 1.86557
DEOD_BACAN F	439176 Me	MTAP_SULSO Fu	60961 ade	1JDV	2AC7	1JDT		0.91	Sc=6.29237, min distance = 1.95133
DEOD_ECOLI F	10041129	SAHH_MYCTU Fu	60961 ade	3CE6	1PK7	3DHY		0.89	Sc=6.30511, min distance = 1.96419
DEOD_ECOLI F	190 adeni	PURR_ECOLI Fu	5287547 6	2PUA	1OTY	2PUB		0.9	Sc=5.67557, min distance = 2.99611
DEOD_ECOLI F	24180721	PIM1_HUMAN Fu	60961 ade	1YI4	1PK7	3C4E			Sc=6.00434, min distance = 1.78792
DEOD_ECOLI F	439176 Me	MTAP_SULSO Fu	60961 ade	1JDV	1PK7	1JDT	33.82	0.91	Sc=6.28867, min distance = 2.15141
DEOD_ECOLI F	444498 in	PNPH_BOVIN Fu	6021 inos	1A9S	1PR0	1B8N			Sc=5.92344, min distance = 2.12933
DEOD_ECOLI F	446470 SA	PIMT_PYRFU Fu	60961 ade	1JG2	1PK7	1JG4		0.84	Sc=6.44479, min distance = 1.16851
DEOD_ECOLI F	447992 1c	Q8I3X4_PLAF7	6021 inos	2BSX	1PR0	1Q1G			Sc=6.36194, min distance = 2.08309
DEOD_ECOLI F	5078682 C	PNPH_BOVIN Fu	6021 inos	1A9S	1PR0	2QPL			Sc=5.85754, min distance = 2.32418
DEOD_ECOLI F	764 guani	PNPH_HUMAN Fu	6021 inos	1RCT	1PR0	1V2H			Sc=5.78152, min distance = 2.72938
DEOD_ECOLI F	8646 Guar	PNPH_HUMAN Fu	6021 inos	1RCT	1PR0	1V41			Sc=5.75767, min distance = 2.53081
DEXB_STRMU F	193758 Va	CDGT2_BACCI F	79025 alp	1D3C	2ZID	1DTU			Sc=5.87191, min distance = 1.88111
DEXB_STRMU F	444809 Th	AMY1_HORVU Fu	79025 alp	1RP8	2ZID	1P6W		0.78	Sc=5.87794, min distance = 0.73660
DEXB_STRMU F	444915 Gl	CDGT2_BACCI F	79025 alp	1D3C	2ZID	1CXL		0.85	Sc=5.71804, min distance = 0.11066
DEXB_STRMU F	445237 1,	AMY1_HORVU Fu	79025 alp	1RP8	2ZID	1P6W		0.77	Sc=5.83448, min distance = 2.05364
DEXB_STRMU F	446000 CI	AMYP_PIG Full	79025 alp	1HX0	2ZID	1JFH		0.83	Sc=5.81666, min distance = 1.87656
DEXB_STRMU F	7027 gluc	Q9C171_PIREQ	79025 alp	1GWM	2ZID	1W8T			Sc=5.91325, min distance = 2.16809
DEXT_PENMI F	445564 4-	GUNG_CLOCE Fu	64689 bet	1GA2	1OGO	1KFG		0.88	Sc=5.80114, min distance = 0.22484
DFPA_LOLVU F	439353 be	O96048_LUMTE	8172 Trig	2DRY	1PJX	2DS0			Sc=5.71337, min distance = 0.96989
DFRA_VITVI F	24180721	PIM1_HUMAN Fu	5281672 m	2O63	2IOD	3C4E			Sc=5.87603, min distance = 2.63945
DFRA_VITVI F	440141 9i	G6PD_LEUME Fu	5886 NADF	1H9A	2IOD	1E7Y			Sc=5.69269, min distance = 1.95297
DFRA_VITVI F	657135 Op	PIM1_HUMAN Fu	5281672 m	2O63	2IOD	1YXV			Sc=5.69232, min distance = 3.00714
DGAL_ECOLI F	15942683	FCN3_HUMAN Fu	439353 be	2J5Z	1GLG	2J60		0.77	Sc=5.68725, min distance = 1.29323
DGAL_ECOLI F	439764 be	ARAF_ECOLI Fu	439353 be	8ABP	1GLG	6ABP		0.93	Sc=5.67623, min distance = 2.55406
DGAL_ECOLI F	6027 xylo	Q9ZB17_9LACT	439353 be	1NSX	1GLG	1MN0		0.93	Sc=5.69659, min distance = 1.99804
DGAL_ECOLI F	7027 gluc	Q72KX2_THET2	439353 be	2B3F	1GLG	2B3B			Sc=5.92229, min distance = 2.38870
DGDA_BURCE F	152217 2,	DYR_CANAL Ful	78165 MES	1M79	1ZOD	1M78			Sc=5.7618, min distance = 2.009304
DGDA_BURCE F	444773 LE	ALR_BACST Ful	445719 EF	1FTX	1M0Q	1L6F		0.92	Sc=6.3693, min distance = 2.458075
DGDA_BURCE F	446862 2k	ALR_BACST Ful	445719 EF	1FTX	1M0Q	1L6G		0.92	Sc=6.35849, min distance = 2.45849

DGUOK_HUMAN I	64968 dTT	DPO42_SULSO Fu	15993 dAT	2AGQ	2OCP	1S0O			Sc=6.4564, min distance = 1.755770
DGUOK_HUMAN I	65091 dCT	DPO42_SULSO Fu	15993 dAT	2AGQ	2OCP	2ASD			Sc=6.38327, min distance = 2.25910
DGUOK_HUMAN I	65103 dGT	DPO42_SULSO Fu	15993 dAT	2AGQ	2OCP	2JEJ		0.8	Sc=6.4897, min distance = 2.078101
DHAK_CITFR Fu	6083 ader	PIM1_HUMAN Ful	33113 gam	1XR1	1UN9	1YXU		0.97	Sc=5.79536, min distance = 2.24209
DHAK_CITFR Fu	64968 dTT	RIR1_YEAST Ful	33113 gam	2EUD	1UN9	2CVW			Sc=5.92023, min distance = 2.09867
DHAL_ECOLI Fu	446090 1h	NDKC_DICDI Ful	6022 Aden	1KDN	2BTD	1HIY		0.97	Sc=6.42021, min distance = 2.19716
DHAL_ECOLI Fu	447017 CI	NDKC_DICDI Ful	6022 Aden	1KDN	2BTD	1LWX			Sc=5.96234, min distance = 1.55929
DHAL_ECOLI Fu	447955 1p	CDK2_HUMAN Ful	6022 Aden	1GY3	2BTD	1PXI			Sc=5.74284, min distance = 2.41347
DHAL_ECOLI Fu	4565 1h1r	CDK2_HUMAN Ful	6022 Aden	1GY3	2BTD	1H1R			Sc=6.01314, min distance = 2.73160
DHAL_ECOLI Fu	5327148 C	IPKA_RABIT Ful	6022 Aden	1JBP	2BTD	2ERZ			Sc=6.17438, min distance = 2.14145
DHAL_ECOLI Fu	60961 ade	IPKA_RABIT Ful	6022 Aden	1JBP	2BTD	1FMO		0.95	Sc=6.21668, min distance = 2.35778
DHAL_ECOLI Fu	6132 Cyti	O33839_THEMA I	6022 Aden	1XJK	2BTD	1XJN			Sc=6.34222, min distance = 2.02474
DHAL_ECOLI Fu	8977 1dar	O33839_THEMA I	6022 Aden	1XJK	2BTD	1XJE		0.8	Sc=6.44913, min distance = 1.86626
DHAL_ECOLI Fu	9817550 V	CDK2_HUMAN Ful	6022 Aden	1GY3	2BTD	3BHV			Sc=6.37079, min distance = 2.12438
DHAL_LACLA Fu	23727981	CDK2_HUMAN Ful	6022 Aden	1GY3	3CR3	3BHT			Sc=6.12331, min distance = 2.29835
DHAL_LACLA Fu	23727982	CDK2_HUMAN Ful	6022 Aden	1GY3	3CR3	3BHU			Sc=6.18043, min distance = 2.21151
DHAL_LACLA Fu	447916 ad	RIO1_ARCFU Ful	6022 Aden	1ZTH	3CR3	1ZTF		0.95	Sc=6.19342, min distance = 2.25695
DHAL_LACLA Fu	447955 1p	CDK2_HUMAN Ful	6022 Aden	1GY3	3CR3	1PXI			Sc=5.70476, min distance = 2.38665
DHAL_LACLA Fu	4565 1h1r	CDK2_HUMAN Ful	6022 Aden	1GY3	3CR3	1H1R			Sc=6.3641, min distance = 1.512169
DHAL_LACLA Fu	5327148 C	IPKA_RABIT Ful	6022 Aden	1JBP	3CR3	2ERZ			Sc=6.14509, min distance = 2.08191
DHAL_LACLA Fu	6083 ader	PSPF_ECOLI Ful	6022 Aden	2C98	3CR3	2VII		0.99	Sc=6.3253, min distance = 2.396207
DHAL_LACLA Fu	60961 ade	IPKA_RABIT Ful	6022 Aden	1JBP	3CR3	1FMO		0.95	Sc=6.16533, min distance = 2.21000
DHAL_LACLA Fu	6918710 5	CDK2_HUMAN Ful	6022 Aden	1GY3	3CR3	3EJ1			Sc=6.14453, min distance = 1.62000
DHA_MYCTU Ful	123927 A3	DAPB_ECOLI Ful	5893 nadi	1DRU	2VHW	1DRV		0.97	Sc=6.77867, min distance = 2.12040
DHA_MYCTU Ful	165230 1a	G6PD_LEUME Ful	5893 nadi	1H94	2VHW	2DPG			Sc=6.46129, min distance = 1.54302
DHA_MYCTU Ful	439153 Di	ADH1E_HORSE Fu	5893 nadi	1MGO	2VHW	2JHF		0.79	Sc=6.7737, min distance = 2.537591
DHA_MYCTU Ful	6083 ader	NADE_ECOLI Ful	5893 nadi	1WXH	2VHW	1WXI			Sc=6.36755, min distance = 2.08362
DHA_MYCTU Ful	6102710 A	Q4PRK9_PLAVI I	5893 nadi	2A92	2VHW	2AA3		0.95	Sc=6.78689, min distance = 2.23944
DHB1_HUMAN Fu	165230 1a	G6PD_LEUME Ful	5886 NADP	1H9A	1QYV	2DPG			Sc=5.79095, min distance = 2.54543
DHB1_HUMAN Fu	444865 Eq	SDIS_COMTE Ful	222865 An	1OHS	1QYW	1OGZ			Sc=5.96669, min distance = 2.56895
DHB4_HUMAN Fu	11987635	ADH_DROLE Ful	5893 nadi	1SBY	1ZBQ	1B2L	23.32	0.76	Sc=6.84927, min distance = 2.03700
DHB4_HUMAN Fu	15942690	INHA_MYCTU Ful	5893 nadi	2H7I	1ZBQ	2NTJ			Sc=6.42983, min distance = 1.60392
DHB4_HUMAN Fu	444427 CI	ADH_DROLE Ful	5893 nadi	1SBY	1ZBQ	1B15	23.32	0.76	Sc=6.82047, min distance = 1.92568
DHB4_HUMAN Fu	449263 IN	INHA_MYCTU Ful	5893 nadi	2H7I	1ZBQ	2NV6		0.93	Sc=6.41062, min distance = 2.21869
DHB4_RAT Ful	15942665	INHA_MYCTU Ful	5893 nadi	2H7I	1GZ6	2H9I		0.88	Sc=6.12323, min distance = 1.97972
DHB4_RAT Ful	15942690	INHA_MYCTU Ful	5893 nadi	2H7I	1GZ6	2NTJ			Sc=6.13749, min distance = 2.27175
DHB4_RAT Ful	169266 1,	GALE_HUMAN Ful	5893 nadi	1HZJ	1GZ6	1I3K		0.79	Sc=6.75075, min distance = 2.31697

DHB4_RAT Ful:	439153 Di	GALE_ECOLI Fu:	5893 nadi	1UDC	1GZ6	1UDB	0.79	Sc=6.31911, min distance = 2.51243
DHB4_RAT Ful:	439153 Di	Q9BJJ9_PLAFA I	5893 nadi	1UH5	1GZ6	1V35	0.79	Sc=6.73996, min distance = 2.07000
DHB4_RAT Ful:	449263 IN	INHA_MYCTU Fu:	5893 nadi	2H7I	1GZ6	2NV6	0.93	Sc=6.10946, min distance = 2.13097
DHB4_RAT Ful:	5957 Ader	PPNK_ARCFU Fu:	5893 nadi	1Z0Z	1GZ6	1Z0S		Sc=6.50735, min distance = 1.19071
DHB8_HUMAN Fu	15942665	INHA_MYCTU Fu:	5893 nadi	2H7I	2PD6	2H9I	0.88	Sc=6.87165, min distance = 2.03928
DHB8_HUMAN Fu	439153 Di	GALE_ECOLI Fu:	5893 nadi	1UDC	2PD6	1UDB	0.79	Sc=6.78116, min distance = 2.38612
DHB8_HUMAN Fu	444427 CI	ADH_DROLE Ful:	5893 nadi	1SBY	2PD6	1B15	0.76	Sc=6.82528, min distance = 2.01676
DHB8_HUMAN Fu	449263 IN	INHA_MYCTU Fu:	5893 nadi	2H7I	2PD6	2NV6	0.93	Sc=6.85612, min distance = 2.05374
DHBE_BACSU Fu	24316 cyc	PDE10_HUMAN Fu	6083 aden	2OUN	1MDB	2OUU	0.81	Sc=6.38954, min distance = 2.02893
DHBE_BACSU Fu	447832 AD	ACSA_SALTY Fu:	6083 aden	2P2F	1MDB	1PG4	0.99	Sc=6.49251, min distance = 2.10065
DHBE_BACSU Fu	5281672 m	PIM1_HUMAN Fu:	6083 aden	1YXU	1MDB	2O63		Sc=6.5107, min distance = 1.533224
DHBE_BACSU Fu	5327121 I	PIM1_HUMAN Fu:	6083 aden	1YXU	1MDB	2C3I		Sc=6.33343, min distance = 0.76969
DHBE_BACSU Fu	6076 Cycl	PDE10_HUMAN Fu	6083 aden	2OUN	1MDB	2OUR	0.99	Sc=6.33036, min distance = 2.31207
DHBE_BACSU Fu	611002 Op	PIM1_HUMAN Fu:	6083 aden	1YXU	1MDB	1YXX		Sc=6.24015, min distance = 1.19525
DHBE_BACSU Fu	656966 1y	PDE4D_HUMAN Fu	6083 aden	1TB7	1MDB	1Y2E		Sc=6.12745, min distance = 2.04381
DHBE_BACSU Fu	657135 Op	PIM1_HUMAN Fu:	6083 aden	1YXU	1MDB	1YXV		Sc=5.97072, min distance = 2.12108
DHD2_LACPA Fu	123927 A3	Q9GT92_CRYPV I	5893 nadi	2FM3	1DXY	2EWD	0.97	Sc=6.33263, min distance = 1.96972
DHD2_LACPA Fu	439153 Di	ADH1B_HUMAN Fu	5893 nadi	1U3U	1DXY	1DEH	0.79	Sc=6.76878, min distance = 2.23923
DHD2_LACPA Fu	439153 Di	ADH1E_HORSE Fu	5893 nadi	1MGO	1DXY	2JHF	0.79	Sc=6.31872, min distance = 2.00577
DHD2_LACPA Fu	440516 CI	MDH_THETH Ful:	5893 nadi	1BMD	1DXY	1BDM	0.79	Sc=6.32334, min distance = 2.00955
DHD2_LACPA Fu	445794 AD	ADHX_HUMAN Fu:	5893 nadi	2FZW	1DXY	2FZE		Sc=6.16312, min distance = 2.08916
DHD2_LACPA Fu	6102710 A	Q4PRK9_PLAVI I	5893 nadi	2A92	1DXY	2AA3	0.95	Sc=6.33483, min distance = 1.80497
DHE2_CLOSY Fu	40539 Qui	GRIK2_RAT Ful:	33032 L-g	1S50	1BGV	1S9T		Sc=5.80344, min distance = 2.22882
DHE2_CLOSY Fu	449178 gl	GLMS_ECOLI Fu:	33032 L-g	1XFF	1BGV	1XFG	0.81	Sc=5.74433, min distance = 2.03667
DHE2_CLOSY Fu	5310984 2	GRM3_RAT Full=	33032 L-g	2E4U	1BGV	2E4Y		Sc=5.86263, min distance = 2.42201
DHE3_BOVIN Fu	3080614 C	HIF1A_HUMAN Fu	51 2-Oxop	1H2L	1HWY	1H2K		Sc=5.70915, min distance = 2.75635
DHE3_BOVIN Fu	3080614 C	HIF1N_HUMAN Fu	51 2-Oxop	1H2L	1HWY	1H2K		Sc=5.70487, min distance = 2.74654
DHE3_BOVIN Fu	440141 9i	G6PD_LEUME Fu:	5886 NADP	1H9A	1HWZ	1E7Y		Sc=6.35352, min distance = 2.07373
DHE3_BOVIN Fu	449178 gl	GLMS_ECOLI Fu:	33032 L-g	1XFF	1HWX	1XFG	0.81	Sc=5.81408, min distance = 2.11634
DHE3_BOVIN Fu	5310984 2	GRM3_RAT Full=	33032 L-g	2E4U	1HWX	2E4Y		Sc=5.91081, min distance = 2.23396
DHE3_BOVIN Fu	5318532 i	IDHP_YEAST Fu:	51 2-Oxop	2QFY	1HWY	2QFW		Sc=6.05394, min distance = 1.11819
DHE3_BOVIN Fu	6083 aden	HSP71_HUMAN Fu	6022 Aden	1S3X	1NQT	1XQS	0.99	Sc=5.85293, min distance = 1.60667
DHE3_BOVIN Fu	6338561 C	MYS2_DICDI Fu:	6022 Aden	1VOM	1NQT	1D0X		Sc=5.74301, min distance = 1.91104
DHGB_ACICA Fu	11987682	DHM1_METME Fu:	5326460 F	2AD8	1CQ1	2AD6	0.83	Sc=6.47921, min distance = 2.42887
DHG_BACME Fu:	169266 1,	GALE_HUMAN Fu:	5893 nadi	1HZJ	1GEE	1I3K	0.79	Sc=6.43706, min distance = 2.50343
DHG_BACME Fu:	440141 9i	G6PD_LEUME Fu:	5893 nadi	1H94	1GEE	1E7Y		Sc=5.9406, min distance = 2.017295
DHG_BACME Fu:	445794 AD	FUCO_ECOLI Fu:	5893 nadi	2BI4	1GEE	1RRM		Sc=6.16037, min distance = 2.05337

DHG_BACME Fu	449263 IN	INHA_MYCTU Fu	5893 nadi	2H7I	1GEE	2NV6		0.93	Sc=6.84866, min distance = 2.10146
DHI1_CAVPO F	15942680	6PGD_LACLM Fu	5886 NADF	2IZ0	1XSE	2IZ1			Sc=6.23723, min distance = 2.14907
DHI1_CAVPO F	165230 1a	FABG_ECOLI Fu	5886 NADF	1Q7B	1XSE	1Q7C			Sc=6.46205, min distance = 1.87597
DHI1_CAVPO F	440141 9i	DHB1_HUMAN Fu	5886 NADF	1QYV	1XSE	1QYW			Sc=5.96857, min distance = 2.01219
DHI1_HUMAN F	440141 9i	DHB1_HUMAN Fu	5886 NADF	1QYV	1XU9	1QYW	30		Sc=6.42391, min distance = 1.90025
DHI1_HUMAN F	446201 1i	DHB1_HUMAN Fu	5886 NADF	1QYV	1XU9	1I5R	30		Sc=6.33477, min distance = 2.03504
DHL2_LACCO F	11987786	LDH_PLAFD Ful	5893 nadi	1T2D	1HYH	1T2E		0.77	Sc=6.7781, min distance = 2.255423
DHL2_LACCO F	123927 A3	DAPB_ECOLI Fu	5893 nadi	1DRU	1HYH	1DRV		0.97	Sc=6.77304, min distance = 1.97738
DHL2_LACCO F	123927 A3	Q9GT92_CRYPV I	5893 nadi	2FM3	1HYH	2EWD		0.97	Sc=6.76557, min distance = 2.41146
DHL2_LACCO F	169266 1,	GALE_HUMAN Fu	5893 nadi	1HZJ	1HYH	1I3K		0.79	Sc=6.78618, min distance = 1.89596
DHL2_LACCO F	439153 Di	ADH1B_HUMAN F	5893 nadi	1U3U	1HYH	1DEH		0.79	Sc=6.77014, min distance = 2.34203
DHL2_LACCO F	439153 Di	ADH1E_HORSE F	5893 nadi	1MGO	1HYH	2JHF		0.79	Sc=6.76919, min distance = 2.41052
DHL2_LACCO F	439153 Di	CTBP1_RAT Ful	5893 nadi	1HKU	1HYH	1HL3		0.79	Sc=6.78175, min distance = 2.15649
DHL2_LACCO F	439153 Di	GALE_ECOLI Fu	5893 nadi	1UDC	1HYH	1UDB		0.79	Sc=6.78663, min distance = 2.13561
DHL2_LACCO F	439153 Di	Q9BJJ9_PLAFA I	5893 nadi	1UH5	1HYH	1V35		0.79	Sc=6.7755, min distance = 1.786546
DHL2_LACCO F	440516 CI	MDH_THETH Ful	5893 nadi	1BMD	1HYH	1BDM		0.79	Sc=6.77835, min distance = 2.33304
DHL2_LACCO F	445794 AI	ADHX_HUMAN Fu	5893 nadi	2FZW	1HYH	2FZE			Sc=6.63114, min distance = 2.73218
DHL2_LACCO F	6102710 A	Q4PRK9_PLAVI I	5893 nadi	2A92	1HYH	2AA3		0.95	Sc=6.77664, min distance = 2.53344
DHL2_LACCO F	9547960 A	NPD_THEMA Ful	5893 nadi	2H4H	1HYH	2H59			Sc=6.63624, min distance = 1.73045
DHM1_METEX F	11987682	DHM1_METME Fu	5326460 F	2AD8	1W6S	2AD6		0.83	Sc=6.5333, min distance = 2.645871
DHOM_YEAST F	444564 AI	ARO1_EMENI Fu	5893 nadi	1SG6	1EBF	1NVA			Sc=6.38579, min distance = 1.92143
DHOM_YEAST F	447048 2k	NPD1_ARCFU Fu	5893 nadi	1ICI	1EBF	1M2K			Sc=6.12412, min distance = 1.74663
DHOM_YEAST F	6022 Ader	ALDH2_HUMAN F	5893 nadi	1O04	1EBF	1NZX			Sc=5.91099, min distance = 2.28889
DHOM_YEAST F	6022 Ader	Q5SI02_THET8 I	5893 nadi	2BJK	1EBF	2BJA			Sc=6.18292, min distance = 2.09234
DHPR_RAT Ful	169266 1,	GALE_HUMAN Fu	5893 nadi	1HZJ	1DHR	1I3K		0.79	Sc=6.31435, min distance = 2.40244
DHPR_RAT Ful	439153 Di	Q9BJJ9_PLAFA I	5893 nadi	1UH5	1DHR	1V35		0.79	Sc=6.30091, min distance = 2.26687
DHPS_ECOLI F	79639 9-M	FOLB_STAAU Fu	69736 Ran	2DHN	1AJ0	1RRW			Sc=5.80766, min distance = 2.00465
DHQSD_ARATH I	445904 DH	AROK_MYCTU Fu	8742 shik	2IYS	2GPT	2DFN			Sc=5.85455, min distance = 2.05502
DHR11_HUMAN I	440141 9i	G6PD_LEUME Fu	5886 NADF	1H9A	1XG5	1E7Y			Sc=6.46179, min distance = 2.11079
DHRS4_PIG Fu	440141 9i	G6PD_LEUME Fu	5886 NADF	1H9A	2ZAT	1E7Y			Sc=6.44387, min distance = 1.98516
DHSD_ECOLI F	11957385	MYG_PHYCA Ful	444522 HE	1VXD	1NEK	2CMM			Sc=5.67245, min distance = 2.53641
DHSD_PIG Ful	11970222	CCPR_YEAST Fu	444098 HE	2EUT	1ZOY	1BJ9		0.77	Sc=6.09985, min distance = 2.55310
DHSO_HUMAN F	4369002 C	LDHA_PIG Full	5893 nadi	9LDB	1PL8	9LDT		0.77	Sc=6.73476, min distance = 1.94039
DHSO_HUMAN F	439153 Di	ADH1E_HORSE F	5893 nadi	1MGO	1PL8	2JHF		0.79	Sc=6.73074, min distance = 2.24142
DHSO_HUMAN F	439153 Di	CTBP1_RAT Ful	5893 nadi	1HKU	1PL8	1HL3		0.79	Sc=6.7372, min distance = 2.437795
DHSO_HUMAN F	440516 CI	MDH_THETH Ful	5893 nadi	1BMD	1PL8	1BDM		0.79	Sc=6.31322, min distance = 2.23586
DHSO_HUMAN F	446006 CI	MDH_THETH Ful	5893 nadi	1BMD	1PL8	2CVQ			Sc=5.81431, min distance = 2.51422

DHTM_METME F	444658 2a	RIFK_HUMAN Ful	444243 FA	1P4M	1DJQ	1NB9	0.97	Sc=6.14272, min distance = 2.25068
DHTM_METME F	446995 FM	CYB2_YEAST Ful	444243 FA	1KBI	1DJQ	1LTD	0.77	Sc=6.64987, min distance = 2.04212
DHTM_METME F	448076 CI	CYB2_YEAST Ful	444243 FA	1KBI	1DJQ	1SZG	0.76	Sc=6.24167, min distance = 1.88929
DHYS_HUMAN F	447151 CI	FABI_ECOLI Ful	5893 nadi	1QSG	1RLZ	1MFP		Sc=6.3575, min distance = 2.013419
DIDH_RAT Ful	15942680	6PGD_LACLM Ful	5886 NADP	2IZ0	1AFS	2IZ1		Sc=6.1049, min distance = 2.443659
DIM5_NEUCR F	188380 Ad	RBCMT_PEA Ful	439155 Ad	2H23	1PEG	2H2E	0.91	Sc=6.51645, min distance = 2.01163
DIM5_NEUCR F	21945395	SETD7_HUMAN F	439155 Ad	2F69	1PEG	1XQH	0.88	Sc=6.45579, min distance = 2.53496
DIM5_NEUCR F	4369099 C	CBIF_BACME Ful	439155 Ad	1CBF	1PEG	2CBF		Sc=6.49237, min distance = 1.73404
DIM5_NEUCR F	446535 CI	HNMT_HUMAN Ful	439155 Ad	2AOT	1PEG	1JQE	0.95	Sc=6.52318, min distance = 1.13001
DIM5_NEUCR F	65482 sir	MTR1_RHOSH Ful	439155 Ad	1NW7	1PEG	1NW6	0.88	Sc=6.55049, min distance = 1.87444
DIM5_NEUCR F	65482 sir	MTTA_THEAQ Ful	439155 Ad	1AQI	1PEG	1AQJ	0.88	Sc=6.52913, min distance = 1.86028
DIM5_NEUCR F	65482 sir	SETD7_HUMAN F	439155 Ad	2F69	1PEG	3CBP	0.88	Sc=6.51367, min distance = 2.60892
DIMT1_HUMAN I	16741210	MTTA_THEAQ Ful	34756 Acy	2ADM	1ZQ9	2JG3		Sc=6.45169, min distance = 1.99049
DIMT1_HUMAN I	439182 5'	Q70GK9_STRCT I	34756 Acy	1RQP	1ZQ9	2CC2	0.86	Sc=6.24657, min distance = 1.65285
DIMT1_HUMAN I	445971 CI	COMT_RAT Full	34756 Acy	2CL5	1ZQ9	1H1D	0.92	Sc=6.55352, min distance = 1.86416
DIMT1_HUMAN I	445971 CI	METJ_ECOLI Ful	34756 Acy	1CMC	1ZQ9	1MJL	0.92	Sc=6.53069, min distance = 1.08434
DIMT1_HUMAN I	5327118 5	Q70GK9_STRCT I	34756 Acy	1RQP	1ZQ9	2C2W	0.81	Sc=5.70204, min distance = 2.69377
DIRA1_HUMAN I	37792 gam	GNAI1_RAT Ful	8977 1dar	1SVK	2GF0	1AS0	0.99	Sc=6.50456, min distance = 2.46425
DIRA1_HUMAN I	37792 gam	RAB6B_HUMAN F	8977 1dar	2E9S	2GF0	2FFQ	0.99	Sc=6.5136, min distance = 2.558427
DIRA1_HUMAN I	37792 gam	RB11A_HUMAN F	8977 1dar	1OIX	2GF0	1OIW	0.99	Sc=6.5014, min distance = 2.676729
DIRA1_HUMAN I	37792 gam	RHOA_HUMAN Ful	8977 1dar	1TX4	2GF0	1CXZ	0.99	Sc=6.52415, min distance = 2.37846
DIRA1_HUMAN I	445871 CI	RASH_HUMAN Ful	8977 1dar	2CE2	2GF0	1GNP		Sc=6.21154, min distance = 2.13741
DIRA1_HUMAN I	445873 CA	RASH_HUMAN Ful	8977 1dar	2CE2	2GF0	2CL6		Sc=6.71948, min distance = 2.00922
DIRA1_HUMAN I	446248 CI	RAN_HUMAN Ful	8977 1dar	3CH5	2GF0	1IBR		Sc=6.53361, min distance = 2.23200
DIRA1_HUMAN I	446248 CI	RASH_HUMAN Ful	8977 1dar	2CE2	2GF0	1IAQ		Sc=6.50298, min distance = 2.13391
DIRA1_HUMAN I	5496554 C	RASH_HUMAN Ful	8977 1dar	2CE2	2GF0	1GNQ		Sc=6.25913, min distance = 1.97254
DIRA1_HUMAN I	6804 guar	EF1A_YEAST Ful	8977 1dar	2B7B	2GF0	1G7C	0.99	Sc=6.05167, min distance = 2.76776
DIRA1_HUMAN I	93082 Gab	CDC42_HUMAN F	8977 1dar	2NGR	2GF0	2ODB	0.99	Sc=6.54919, min distance = 2.48794
DIRA1_HUMAN I	93082 Gab	RASH_HUMAN Ful	8977 1dar	2CE2	2GF0	121P	0.99	Sc=6.54412, min distance = 2.46260
DIRA2_HUMAN I	37792 gam	GNAI1_RAT Ful	8977 1dar	1SVK	2ERX	1AS0	0.99	Sc=6.52771, min distance = 2.24855
DIRA2_HUMAN I	37792 gam	RAB6B_HUMAN F	8977 1dar	2E9S	2ERX	2FFQ	0.99	Sc=6.54039, min distance = 1.91315
DIRA2_HUMAN I	37792 gam	RB11A_HUMAN F	8977 1dar	1OIX	2ERX	1OIW	0.99	Sc=6.53212, min distance = 2.23761
DIRA2_HUMAN I	444148 1a	RHA1_ARATH Ful	8977 1dar	2EFC	2ERX	2EFE	0.99	Sc=6.4858, min distance = 2.193526
DIRA2_HUMAN I	448405 CI	ARF1_RAT Full	8977 1dar	1RRG	2ERX	1RRF		Sc=6.47558, min distance = 2.47195
DIRA2_HUMAN I	8582 Inos	PURA1_MOUSE F	8977 1dar	1LON	2ERX	1IWE	0.99	Sc=6.40268, min distance = 2.31998
DIRA2_HUMAN I	93082 Gab	CDC42_HUMAN F	8977 1dar	2NGR	2ERX	2ODB	0.99	Sc=6.57389, min distance = 2.36093
DIRA2_HUMAN I	93082 Gab	RAC3_HUMAN Ful	8977 1dar	2C2H	2ERX	2QME	0.99	Sc=6.5759, min distance = 2.405496

DIRA2_HUMAN	93082 Gab	RASH_HUMAN Fu	8977 1dar	2CE2	2ERX	121P	0.99	Sc=6.57421, min distance = 2.34732
DISA_THEMA F	91557 1tm	CYAA_BACAN Fu	65562 1xf	1K90	3C23	1S26	0.99	Sc=6.44071, min distance = 1.20678
DLDH1_BACST	11987634	GLPD_ECOLI Fu	444188 CI	2QCU	1EBD	2R4E	0.97	Sc=6.42472, min distance = 1.81133
DLDH1_BACST	16740985	FRDA_SHEFR Fu	444188 CI	1M64	1EBD	1E39	0.93	Sc=6.42676, min distance = 2.28003
DLDH1_BACST	444502 CI	FRDA_SHEFR Fu	444188 CI	1M64	1EBD	1QJD	0.98	Sc=6.86578, min distance = 2.42531
DLDH1_BACST	444502 CI	GSHR_HUMAN Fu	444188 CI	3DK9	1EBD	1BWC	0.98	Sc=6.4299, min distance = 2.596646
DLDH1_BACST	444502 CI	TYTR_TRYCR Fu	444188 CI	1BZL	1EBD	1GXF	0.98	Sc=6.8741, min distance = 2.153719
DLDH1_BACST	446013 1,	O28603_ARCFU	444188 CI	1JNR	1EBD	1JNZ	0.94	Sc=6.87441, min distance = 2.03774
DLDH1_BACST	449465 CI	GSHR_HUMAN Fu	444188 CI	3DK9	1EBD	3GRT	0.98	Sc=6.87188, min distance = 2.26416
DLDH1_PSEPU	447048 2b	NPD1_ARCFU Fu	5893 nadi	1ICI	1LVL	1M2K		Sc=6.13404, min distance = 2.27933
DLDH_AZOVI F	16740985	FRDA_SHEFR Fu	444188 CI	1M64	3LAD	1E39	0.93	Sc=6.86696, min distance = 2.34051
DLDH_AZOVI F	23631921	LSD1_HUMAN Fu	444188 CI	2DW4	3LAD	2Z3Y	0.91	Sc=6.49997, min distance = 2.00863
DLDH_AZOVI F	444502 CI	FRDA_SHEFR Fu	444188 CI	1M64	3LAD	1QJD	0.98	Sc=6.87078, min distance = 2.27826
DLDH_AZOVI F	444502 CI	GSHR_HUMAN Fu	444188 CI	3DK9	3LAD	1BWC	0.98	Sc=6.86673, min distance = 2.54687
DLDH_AZOVI F	444502 CI	TYTR_TRYCR Fu	444188 CI	1BZL	3LAD	1GXF	0.98	Sc=6.86598, min distance = 2.22724
DLDH_AZOVI F	448054 CI	FRDA_SHEFR Fu	444188 CI	1M64	3LAD	1Y0P	0.97	Sc=6.86758, min distance = 2.26993
DLDH_HUMAN F	444502 CI	GSHR_HUMAN Fu	444188 CI	3DK9	1ZMD	1BWC	0.98	Sc=6.88997, min distance = 2.55919
DLDH_HUMAN F	446013 1,	O28603_ARCFU	444188 CI	1JNR	1ZMD	1JNZ	0.94	Sc=6.89628, min distance = 1.85903
DLDH_HUMAN F	449465 CI	GSHR_HUMAN Fu	5893 nadi	1GRB	1ZMD	3GRT	0.82	Sc=6.89225, min distance = 1.79648
DLDH_HUMAN F	6022 Ader	Q5SI02_THET8	5893 nadi	2BJK	1ZMD	2BJA		Sc=6.25479, min distance = 2.13607
DLDH_MYCTU F	16740985	FRDA_SHEFR Fu	444188 CI	1M64	2A8X	1E39	0.93	Sc=6.14689, min distance = 2.34025
DLDH_MYCTU F	444502 CI	FRDA_SHEFR Fu	444188 CI	1M64	2A8X	1QJD	0.98	Sc=6.14558, min distance = 2.39978
DLDH_MYCTU F	444502 CI	TYTR_TRYCR Fu	444188 CI	1BZL	2A8X	1GXF	0.98	Sc=6.42984, min distance = 2.21536
DLDH_MYCTU F	448054 CI	FRDA_SHEFR Fu	444188 CI	1M64	2A8X	1Y0P	0.97	Sc=6.1452, min distance = 2.482075
DLDH_PEA Ful	11987634	GLPD_ECOLI Fu	444188 CI	2QCU	1DXL	2R4E	0.97	Sc=6.43723, min distance = 2.02517
DLDH_PEA Ful	11987634	TYTR_TRYCR Fu	444188 CI	1BZL	1DXL	1AOG	0.97	Sc=6.44586, min distance = 1.73189
DLDH_PEA Ful	444502 CI	FRDA_SHEFR Fu	444188 CI	1M64	1DXL	1QJD	0.98	Sc=6.43876, min distance = 1.97113
DLDH_PEA Ful	444502 CI	TYTR_TRYCR Fu	444188 CI	1BZL	1DXL	1GXF	0.98	Sc=6.44402, min distance = 1.41630
DLDH_PSEFL F	16740985	FRDA_SHEFR Fu	444188 CI	1M64	1LPF	1E39	0.93	Sc=6.8686, min distance = 2.178624
DLDH_PSEFL F	444502 CI	FRDA_SHEFR Fu	444188 CI	1M64	1LPF	1QJD	0.98	Sc=6.8656, min distance = 2.209165
DLDH_PSEFL F	444502 CI	GSHR_HUMAN Fu	444188 CI	3DK9	1LPF	1BWC	0.98	Sc=6.87078, min distance = 2.47104
DLDH_PSEFL F	444502 CI	TYTR_TRYCR Fu	444188 CI	1BZL	1LPF	1GXF	0.98	Sc=6.86841, min distance = 2.19545
DLDH_PSEFL F	448054 CI	FRDA_SHEFR Fu	444188 CI	1M64	1LPF	1Y0P	0.97	Sc=6.86696, min distance = 2.14695
DLDH_TRYCR F	16740985	FRDA_SHEFR Fu	444188 CI	1M64	2QAE	1E39	0.93	Sc=6.8717, min distance = 2.236077
DLDH_TRYCR F	444502 CI	FRDA_SHEFR Fu	444188 CI	1M64	2QAE	1QJD	0.98	Sc=6.87315, min distance = 2.23739
DLDH_TRYCR F	445203 NA	ADRO_BOVIN Fu	444188 CI	1CJC	2QAE	1E1M	0.82	Sc=5.83769, min distance = 2.15757
DLDH_TRYCR F	448054 CI	FRDA_SHEFR Fu	444188 CI	1M64	2QAE	1Y0P	0.97	Sc=6.43183, min distance = 2.24690

DLDH_TRYCR Fu	5893 nadi	NAPE_ENTFA Ful	444188 CI	1NHP	2QAE	2NPX	0.82	Sc=5.99214, min distance = 2.09086
DLDH_YEAST Fu	16740985	FRDA_SHEFR Ful	444188 CI	1M64	1V59	1E39	0.93	Sc=6.43663, min distance = 2.34383
DLDH_YEAST Fu	23727981	CDK2_HUMAN Ful	6022 Aden	1GY3	1V59	3BHT		Sc=6.08864, min distance = 2.42169
DLDH_YEAST Fu	5893 nadi	GSHR_ECOLI Ful	444188 CI	1GES	1V59	1GEU	0.82	Sc=6.30679, min distance = 1.57700
DLDH_YEAST Fu	5957 Ader	REX_BACSU Ful	6022 Aden	2VT2	1V59	2VT3	0.99	Sc=5.67194, min distance = 2.00896
DLDH_YEAST Fu	6918710 5	CDK2_HUMAN Ful	6022 Aden	1GY3	1V59	3EJ1		Sc=6.03258, min distance = 2.53173
DLTA_BACCR Fu	166760 Fa	PURP_METJA Ful	6083 aden	2R7M	3DHV	2R7N	0.76	Sc=6.41728, min distance = 2.29832
DLTA_BACCR Fu	24180721	PIM1_HUMAN Ful	6083 aden	1YXU	3DHV	3C4E		Sc=6.06946, min distance = 1.99605
DLTA_BACCR Fu	445708 AM	NADE_ECOLI Ful	6083 aden	1WXI	3DHV	1WXE	0.91	Sc=6.05032, min distance = 2.14925
DLTA_BACCR Fu	447832 AD	ACSA_SALTY Ful	6083 aden	2P2F	3DHV	1PG4	0.99	Sc=6.47264, min distance = 2.31015
DLTA_BACCR Fu	611002 Op	PIM1_HUMAN Ful	6083 aden	1YXU	3DHV	1YXX		Sc=6.24576, min distance = 1.95503
DLTA_BACCR Fu	65110 AIC	PURP_METJA Ful	6083 aden	2R7M	3DHV	2R7K	0.79	Sc=6.31575, min distance = 2.09158
DLTA_BACCR Fu	657135 Op	PIM1_HUMAN Ful	6083 aden	1YXU	3DHV	1YXV		Sc=5.97463, min distance = 2.13875
DLTA_BACSU Fu	101609 Ur	Q9SSV1_NICGU I	6083 aden	1VD1	3E7W	1VD3		Sc=6.30493, min distance = 2.16463
DLTA_BACSU Fu	166760 Fa	PURP_METJA Ful	6083 aden	2R7M	3E7W	2R7N	0.76	Sc=6.42574, min distance = 2.16803
DLTA_BACSU Fu	24180721	PIM1_HUMAN Ful	6083 aden	1YXU	3E7W	3C4E		Sc=6.07377, min distance = 1.51372
DLTA_BACSU Fu	3758 IBMX	PDE4D_HUMAN Fu	6083 aden	1TB7	3E7W	1ZKN		Sc=6.05437, min distance = 1.14882
DLTA_BACSU Fu	440641 1m	O57693_THETE I	6083 aden	1UXU	3E7W	1UXR		Sc=5.99057, min distance = 2.38831
DLTA_BACSU Fu	445708 AM	NADE_ECOLI Ful	6083 aden	1WXI	3E7W	1WXE	0.91	Sc=6.38435, min distance = 2.27853
DLTA_BACSU Fu	447832 AD	ACSA_SALTY Ful	6083 aden	2P2F	3E7W	1PG4	0.99	Sc=6.49603, min distance = 2.49707
DLTA_BACSU Fu	6076 Cycl	PDE10_HUMAN Fu	6083 aden	2OUN	3E7W	2OUR	0.99	Sc=6.33271, min distance = 2.03569
DLTA_BACSU Fu	65110 AIC	PURP_METJA Ful	6083 aden	2R7M	3E7W	2R7K	0.79	Sc=6.34059, min distance = 2.13513
DLTA_BACSU Fu	65533 cor	O57693_THETE I	6083 aden	1UXU	3E7W	1UXT		Sc=6.01157, min distance = 2.42923
DLTA_BACSU Fu	657135 Op	PIM1_HUMAN Ful	6083 aden	1YXU	3E7W	1YXV		Sc=5.96456, min distance = 2.23663
DLTA_BACSU Fu	6804 guar	NDK_PYRHO Ful	6083 aden	2DXD	3E7W	2DXF	0.8	Sc=6.39134, min distance = 2.21486
DLTA_BACSU Fu	8582 Inos	PYGM_RABIT Ful	6083 aden	8GPB	3E7W	2QN7	0.79	Sc=6.4293, min distance = 2.210368
DMA_BPT4 Ful	188380 Ac	MCES_ENCCU Ful	439155 Ad	1RI1	1YF3	1Z3C	0.91	Sc=6.61319, min distance = 2.16083
DMA_BPT4 Ful	446535 CI	HNMT_HUMAN Ful	439155 Ad	2AOT	1YF3	1JQE	0.95	Sc=6.57889, min distance = 2.28736
DMA_BPT4 Ful	60961 ade	PIMT_PYRFU Ful	439155 Ad	1JG1	1YF3	1JG2	0.84	Sc=6.36004, min distance = 2.47910
DMA_BPT4 Ful	65482 sir	ERM_BACSU Ful	439155 Ad	1QAN	1YF3	1QAQ	0.88	Sc=6.60996, min distance = 2.06939
DMA_ECOLI Ful	122068 de	HNMT_HUMAN Ful	439155 Ad	2AOT	2G1P	2AOU		Sc=5.94967, min distance = 1.73953
DMA_ECOLI Ful	188380 Ac	MCES_ENCCU Ful	439155 Ad	1RI1	2G1P	1Z3C	0.91	Sc=6.57146, min distance = 2.11496
DMA_ECOLI Ful	446535 CI	HNMT_HUMAN Ful	439155 Ad	2AOT	2G1P	1JQE	0.95	Sc=6.51708, min distance = 2.58103
DMA_ECOLI Ful	60961 ade	PIMT_PYRFU Ful	439155 Ad	1JG1	2G1P	1JG2	0.84	Sc=6.26156, min distance = 2.79012
DMA_ECOLI Ful	65482 sir	ERM_BACSU Ful	439155 Ad	1QAN	2G1P	1QAQ	0.88	Sc=6.57926, min distance = 2.08441
DMA_ECOLI Ful	65482 sir	MCES_ENCCU Ful	439155 Ad	1RI1	2G1P	2HV9	0.88	Sc=6.56337, min distance = 2.05814
DMA_ECOLI Ful	65482 sir	PRMA_THET8 Ful	439155 Ad	3EGV	2G1P	2ZBR	0.88	Sc=6.57108, min distance = 2.50908

DMSA_RHOSH Fı	104904 Me	IGKC_MOUSE Fu:	23831 HEF	1P7K	1EU1	2AJZ		Sc=5.74148, min distance = 1.93216'
DMSA_RHOSH Fı	8200 TETF	Q8WSF8_APLCA I	23831 HEF	2BR7	1EU1	2BYN		Sc=5.87169, min distance = 1.03039:
DNAA_AQUAE Fı	13373715	HS90A_HUMAN Fı	6022 Aden	1BYQ	1L8Q	2QF6		Sc=6.2497, min distance = 2.366191:
DNAA_AQUAE Fı	23653516	CDK2_HUMAN Fu:	6022 Aden	1GY3	1L8Q	2R3G		Sc=6.25497, min distance = 2.21554'
DNAA_AQUAE Fı	24864078	CDK2_HUMAN Fu:	6022 Aden	1GY3	1L8Q	2VTO		Sc=6.46477, min distance = 1.80851{
DNAA_AQUAE Fı	33113 gam	KIF1A_MOUSE Fı	91532 AMF	1I6I	2HCB	1VFV	0.99	Sc=6.50779, min distance = 2.32351!
DNAA_AQUAE Fı	398148 1e	CDK2_HUMAN Fu:	6022 Aden	1GY3	1L8Q	1E1X		Sc=6.19565, min distance = 2.01397!
DNAA_AQUAE Fı	444564 AD	MYS2_DICDI Fu:	6022 Aden	1VOM	1L8Q	1W9I	0.91	Sc=5.6745, min distance = 2.384060
DNAA_AQUAE Fı	444852 CI	ATPB_BOVIN Fu:	6022 Aden	2CK3	1L8Q	1COW	0.9	Sc=5.68357, min distance = 2.73374:
DNAA_AQUAE Fı	445940 O6	CDK2_HUMAN Fu:	6022 Aden	1GY3	1L8Q	1GZ8		Sc=6.14555, min distance = 1.85833:
DNAA_AQUAE Fı	445966 O6	CDK2_HUMAN Fu:	6022 Aden	1GY3	1L8Q	1H0V		Sc=6.17756, min distance = 2.07849
DNAA_AQUAE Fı	449088 1v	CDK2_HUMAN Fu:	6022 Aden	1GY3	1L8Q	1VYZ		Sc=6.18373, min distance = 2.13939!
DNAA_AQUAE Fı	4564 1e1v	CDK2_HUMAN Fu:	6022 Aden	1GY3	1L8Q	1E1V		Sc=6.14616, min distance = 2.02461'
DNAA_AQUAE Fı	5327148 C	IPKA_RABIT Fu:	6022 Aden	1JBP	1L8Q	2ERZ		Sc=6.14238, min distance = 2.09432:
DNAA_AQUAE Fı	5957 Ader	DDL_THET8 Ful:	6022 Aden	2ZDH	1L8Q	2ZDQ	0.99	Sc=5.73004, min distance = 1.58734(
DNAA_AQUAE Fı	5957 Ader	MUTS_ECOLI Fu:	6022 Aden	1OH7	1L8Q	1W7A	0.99	Sc=5.70239, min distance = 2.49983(
DNAA_AQUAE Fı	6102784 A	PSPF_ECOLI Fu:	6022 Aden	2C98	1L8Q	2C99	0.99	Sc=5.72073, min distance = 2.40527:
DNAA_AQUAE Fı	9547890 I	CDK2_HUMAN Fu:	6022 Aden	1GY3	1L8Q	1W8C		Sc=6.23034, min distance = 2.18893(
DNAA_THEMA Fı	444564 AD	MYS2_DICDI Fu:	6022 Aden	1VOM	2Z4S	1W9I	0.91	Sc=5.70628, min distance = 2.24453'
DNAA_THEMA Fı	444842 CI	CDK2_HUMAN Fu:	6022 Aden	1GY3	2Z4S	1CKP		Sc=5.60314, min distance = 2.33631(
DNAA_THEMA Fı	5289411 1	CDK2_HUMAN Fu:	6022 Aden	1GY3	2Z4S	1OGU		Sc=6.5757, min distance = 1.497375'
DNAA_THEMA Fı	5957 Ader	BIOD_ECOLI Fu:	6022 Aden	1DAD	2Z4S	1A82	0.99	Sc=5.76321, min distance = 2.15802!
DNAA_THEMA Fı	6083 ader	PSPF_ECOLI Fu:	6022 Aden	2C98	2Z4S	2VII	0.99	Sc=5.9157, min distance = 2.338791!
DNAA_THEMA Fı	91532 AMF	AROK_MYCTU Fu:	6022 Aden	2IYV	2Z4S	1ZYU	0.99	Sc=6.07033, min distance = 2.21842:
DNAA_THEMA Fı	91532 AMF	BIOD_ECOLI Fu:	6022 Aden	1DAD	2Z4S	1DAG	0.99	Sc=5.78455, min distance = 2.01125
DNAK_GEOKA Fı	15993 dAT	RECA_MYCS2 Fu:	6022 Aden	2ZRO	2V7Y	2ZR9	0.97	Sc=6.49236, min distance = 2.12614'
DNAK_GEOKA Fı	36735 Gpp	PARM_ECOLX Fu:	6022 Aden	1MWM	2V7Y	2ZGZ	0.79	Sc=6.51394, min distance = 2.19643!
DNAK_GEOKA Fı	445966 O6	CDK2_HUMAN Fu:	6022 Aden	1GY3	2V7Y	1H0V		Sc=6.29417, min distance = 1.94500!
DNAK_GEOKA Fı	447916 ac	RIO1_ARCFU Fu:	6022 Aden	1ZTH	2V7Y	1ZTF	0.95	Sc=6.2765, min distance = 2.160630!
DNAK_GEOKA Fı	447955 1p	CDK2_HUMAN Fu:	6022 Aden	1GY3	2V7Y	1PXI		Sc=5.79169, min distance = 2.27119!
DNAK_GEOKA Fı	5957 Ader	HSP7F_YEAST Fı	6022 Aden	3C7N	2V7Y	3D2F	0.99	Sc=6.50573, min distance = 2.09897!
DNAK_GEOKA Fı	5957 Ader	MUTS_ECOLI Fu:	6022 Aden	1OH7	2V7Y	1W7A	0.99	Sc=6.50339, min distance = 2.21257'
DNAK_GEOKA Fı	6083 ader	PURP_METJA Fu:	6022 Aden	2R7N	2V7Y	2R7M	0.99	Sc=6.39311, min distance = 2.03842
DNAK_GEOKA Fı	6804 guar	NDK_PYRHO Ful:	6022 Aden	2DYA	2V7Y	2DXF	0.8	Sc=6.42792, min distance = 2.18041(
DNAK_GEOKA Fı	8977 1dar	PARM_ECOLX Fu:	6022 Aden	1MWM	2V7Y	2ZGY	0.8	Sc=6.47331, min distance = 2.15229:
DNAK_GEOKA Fı	91532 AMF	PURP_METJA Fu:	6022 Aden	2R7N	2V7Y	2R7K	0.99	Sc=6.5483, min distance = 2.002070
DNK_DROME Fu:	119119 Di	DPOLB_HUMAN Fı	65091 dCT	1MQ3	2VP4	2FMP	0.93	Sc=6.30035, min distance = 1.96375!

DNK_DROME Fu	119182 Cl	DCK_HUMAN Ful	13711 dec	2NO1	2VP5	2A7Q	37.36		Sc=6.27244, min distance = 2.116488
DNK_DROME Fu	13730 dec	DCK_HUMAN Ful	13711 dec	2NO1	2VP5	2ZI3	37.36		Sc=6.28903, min distance = 1.003643
DNK_DROME Fu	13945 Dec	DCK_HUMAN Ful	13711 dec	2NO1	2VP5	2QRN	37.36	0.93	Sc=6.26746, min distance = 1.736016
DNK_DROME Fu	13945 Dec	Q93IG4_MYCMS	65091 dCT	2JAQ	2VP4	2JAT		0.97	Sc=6.22856, min distance = 1.737150
DNK_DROME Fu	15993 dAT	DPO42_SULSO F	65091 dCT	2ASD	2VP4	2AGQ			Sc=6.50321, min distance = 1.879552
DNK_DROME Fu	15993 dAT	DPO42_SULSO F	65103 dGT	2JEJ	2VP2	2AGQ		0.8	Sc=6.50251, min distance = 1.924071
DNK_DROME Fu	1865 6-hy	KITH_HHV11 Fu	5789 thym	1P7C	1OT3	1E2M			Sc=6.0111, min distance = 2.2777223
DNK_DROME Fu	187790 de	DCK_HUMAN Ful	13711 dec	2NO1	2VP5	2ZI7	37.36		Sc=6.35366, min distance = 0.783425
DNK_DROME Fu	20279 Cla	DCK_HUMAN Ful	13711 dec	2NO1	2VP5	2ZIA	37.36		Sc=6.29985, min distance = 1.280005
DNK_DROME Fu	445210 9-	KITH_HHV11 Fu	5789 thym	1P7C	1OT3	1E2I			Sc=6.02767, min distance = 2.083917
DNK_DROME Fu	445211 9-	KITH_HHV11 Fu	5789 thym	1P7C	1OT3	1E2I			Sc=6.02895, min distance = 0
DNK_DROME Fu	446726 AI	KITH_HHV11 Fu	5789 thym	1P7C	1OT3	1KI6		0.77	Sc=6.17997, min distance = 2.378777
DNK_DROME Fu	447628 [(	KITH_HHV11 Fu	5789 thym	1P7C	1OT3	1OF1			Sc=6.2312, min distance = 2.0650714
DNK_DROME Fu	448378 al	APHA_ECOLI Fu	13711 dec	1RMY	2VP5	1RMT			Sc=6.30972, min distance = 1.602213
DNK_DROME Fu	448381 2'	DPOLL_HUMAN F	65091 dCT	2PFP	2VP4	2PFN		0.82	Sc=6.42344, min distance = 1.785894
DNK_DROME Fu	454194 Tr	DCK_HUMAN Ful	13711 dec	2NO1	2VP5	2NO9	37.36		Sc=6.02467, min distance = 0.368849
DNK_DROME Fu	6031 Uric	DCK_HUMAN Ful	13711 dec	2NO1	2VP5	2ZIA	37.36		Sc=6.36127, min distance = 1.783405
DNK_DROME Fu	60750 gem	DCK_HUMAN Ful	13711 dec	2NO1	2VP5	2NO0	37.36	0.95	Sc=6.13005, min distance = 2.077973
DNK_DROME Fu	60825 lam	DCK_HUMAN Ful	13711 dec	2NO1	2VP5	2NOA	37.36		Sc=5.95701, min distance = 2.990575
DNK_DROME Fu	60877 Emt	DCK_HUMAN Ful	13711 dec	2NO1	2VP5	2NO6	37.36		Sc=6.03555, min distance = 0.436518
DNK_DROME Fu	6253 cyta	DCK_HUMAN Ful	13711 dec	2NO1	2VP5	1P5Z	37.36	0.96	Sc=6.16429, min distance = 2.588020
DNLI1_HUMAN I	5957 Ader	PSPF_ECOLI Fu	6083 aden	2VII	1X9N	2C96		0.98	Sc=5.94617, min distance = 2.201997
DNLI1_HUMAN I	6022 Ader	GLNA_SALTY Fu	6083 aden	1LGR	1X9N	1F52		0.99	Sc=5.86442, min distance = 2.482889
DNLI1_HUMAN I	611002 Op	PIM1_HUMAN Fu	6083 aden	1YXU	1X9N	1YXX			Sc=6.14509, min distance = 1.472923
DNLI1_HUMAN I	6804 guar	NDK_PYRHO Ful	6083 aden	2DXD	1X9N	2DXF		0.8	Sc=5.9343, min distance = 1.8339517
DNLI_BPT7 Fu	6022 Ader	ACT1_DROME Fu	5957 Aden	2HF4	1A0I	2HF3		0.99	Sc=5.96519, min distance = 2.081156
DNLI_BPT7 Fu	6022 Ader	HSP7F_YEAST F	5957 Aden	3D2F	1A0I	3C7N		0.99	Sc=5.7474, min distance = 1.9993323
DNLI_BPT7 Fu	6022 Ader	PUR7_METJA Fu	5957 Aden	2Z02	1A0I	2YZL		0.99	Sc=5.93284, min distance = 2.087527
DNLI_PYRFU F	101609 Ur	Q9SSV1_NICGU I	6083 aden	1VD1	2CFM	1VD3			Sc=6.23512, min distance = 2.349697
DNLI_PYRFU F	166760 Fa	PURP_METJA Fu	6083 aden	2R7M	2CFM	2R7N		0.76	Sc=5.93748, min distance = 2.006154
DNLI_PYRFU F	2519 caff	PYGM_RABIT Fu	6083 aden	8GPB	2CFM	1GFZ			Sc=5.82993, min distance = 2.148479
DNLI_PYRFU F	611002 Op	PIM1_HUMAN Fu	6083 aden	1YXU	2CFM	1YXX			Sc=6.17552, min distance = 2.217771
DNLI_PYRFU F	8582 Inos	PURA1_MOUSE F	6083 aden	1MF0	2CFM	1IWE		0.79	Sc=6.3529, min distance = 2.0496843
DNLI_SULSO F	11708454	KAPCA_BOVIN F	5957 Aden	1Q24	2HIX	2VNW			Sc=6.20508, min distance = 2.197238
DNLJ_ECOLI F	5957 Ader	PURP_PYRFU Fu	6083 aden	2R85	2OWO	2R86		0.98	Sc=6.41857, min distance = 1.892697
DNLJ_ECOLI F	6804 guar	PUR1_ECOLI Fu	6083 aden	1ECJ	2OWO	1ECB		0.8	Sc=6.39681, min distance = 2.039907
DNLJ_HAEIN F	10038928	DNLJ_ENTFA Fu	25023705	3BA9	3BAC	3BA8			Sc=6.27537, min distance = 2.406564

DNLJ_HAEIN Fı	25023707	DNLJ_ENTFA Fu	25023705	3BA9	3BAC	3BAB		Sc=6.23915, min distance = 2.80876
DNMK_BPT4 Fu	6802 guar	RNAS1_BOVIN Fı	65059 Dec	2QCA	1DEK	1EOW	0.94	Sc=6.36252, min distance = 2.03496
DOIS_BACCI Fı	169266 1,	GALE_HUMAN Fu	5893 nadi	1HZJ	2GRU	1I3K	0.79	Sc=6.73389, min distance = 1.93913
DOIS_BACCI Fı	4369261 C	ARO1_EMENI Fu	5893 nadi	1SG6	2GRU	1NRX	0.86	Sc=6.26545, min distance = 2.27063
DOIS_BACCI Fı	439153 Di	GALE_ECOLI Fu	5893 nadi	1UDC	2GRU	1UDB	0.79	Sc=6.74328, min distance = 1.66088
DOT1L_HUMAN I	445762 5'	MTTA_THEAQ Fu	34756 Acy	2ADM	1NW3	2IH2	0.93	Sc=6.378, min distance = 2.0260814
DOT1L_HUMAN I	445971 CI	COMT_RAT Full	34756 Acy	2CL5	1NW3	1H1D	0.92	Sc=6.55014, min distance = 2.40875
DOT1_YEAST Fı	188380 Ac	MCES_ENCCU Fu	439155 Ad	1RI1	1U2Z	1Z3C	0.91	Sc=6.58585, min distance = 2.07462
DOT1_YEAST Fı	446535 CI	HNMT_HUMAN Fu	439155 Ad	2AOT	1U2Z	1JQE	0.95	Sc=6.54321, min distance = 2.59214
DOT1_YEAST Fı	60961 ade	PIMT_PYRFU Fu	439155 Ad	1JG1	1U2Z	1JG2	0.84	Sc=6.32735, min distance = 2.68663
DOT1_YEAST Fı	65482 sir	MTR1_RHOSH Fu	439155 Ad	1NW7	1U2Z	1NW6	0.88	Sc=6.56715, min distance = 2.16898
DPHB_PYRHO Fı	4369099 C	CBIF_BACME Fu	439155 Ad	1CBF	2Z6R	2CBF		Sc=6.52318, min distance = 2.30249
DPO1_BACST Fı	119119 Di	DPO42_SULSO Fı	65091 dCT	2ASD	1LV5	1S97	0.93	Sc=5.80227, min distance = 1.56574
DPO1_BACST Fı	15993 dAT	DPO42_SULSO Fı	65091 dCT	2ASD	1LV5	2AGQ		Sc=5.9195, min distance = 2.281167
DPO1_BACST Fı	15993 dAT	DPOL_BPR69 Fu	65091 dCT	1Q9Y	1LV5	2OZS		Sc=5.97258, min distance = 2.31259
DPO1_BACST Fı	16741164	DPOLB_HUMAN Fı	65091 dCT	1MQ3	1LV5	1ZJN		Sc=6.00794, min distance = 2.49449
DPO1_BACST Fı	187790 de	DPOL_BPR69 Fu	65091 dCT	1Q9Y	1LV5	1Q9X		Sc=6.15372, min distance = 2.55927
DPO1_BACST Fı	24139 ace	LYSC_CHICK Fu	5988 sucr	1JJ0	1L3S	1LZB		Sc=5.85483, min distance = 2.60437
DPO1_BACST Fı	448381 2'	DPOLL_HUMAN Fı	65091 dCT	2PFP	1LV5	2PFN	0.82	Sc=5.60043, min distance = 2.43032
DPO1_BACST Fı	64968 dTT	DPOL_BPR69 Fu	65091 dCT	1Q9Y	1LV5	3CQ8	0.75	Sc=5.65334, min distance = 2.29624
DPO1_BACST Fı	65103 dGT	DPO42_SULSO Fı	65091 dCT	2ASD	1LV5	2JEJ		Sc=5.69306, min distance = 2.76440
DPO1_ECOLI Fı	119119 Di	DPOLB_HUMAN Fı	65091 dCT	1MQ3	1KFD	2FMP	0.93	Sc=5.62469, min distance = 2.02722
DPO1_ECOLI Fı	656930 DD	DPO42_SULSO Fı	65091 dCT	2ASD	1KFD	1S9F	0.92	Sc=5.97993, min distance = 1.85399
DPO1_THEAQ Fı	15942679	DPOLB_HUMAN Fı	65091 dCT	1MQ3	5KTQ	2ISP		Sc=5.93303, min distance = 2.44444
DPO1_THEAQ Fı	64968 dTT	POLI_HUMAN Fu	65091 dCT	2DPI	5KTQ	1T3N	0.75	Sc=5.79086, min distance = 2.19601
DPO3X_ECOLI I	1540 1h1c	CDK2_HUMAN Fu	6022 Aden	1GY3	1NJF	1H1Q		Sc=6.41221, min distance = 1.83087
DPO3X_ECOLI I	444564 AD	BIOD_ECOLI Fu	6022 Aden	1DAD	1NJF	1BS1	0.91	Sc=6.44913, min distance = 2.31648
DPO3X_ECOLI I	444564 AD	MYS2_DICDI Fu	440317 AT	1MMG	1NJF	1W9I	0.91	Sc=6.46237, min distance = 2.16439
DPO3X_ECOLI I	4565 1h1r	CDK2_HUMAN Fu	6022 Aden	1GY3	1NJF	1H1R		Sc=6.40382, min distance = 2.08768
DPO3X_ECOLI I	5957 Aden	DDL_THET8 Full	6022 Aden	2ZDH	1NJF	2ZDQ	0.99	Sc=6.47968, min distance = 2.19687
DPO3X_ECOLI I	6031 Uric	DCK_HUMAN Full	6022 Aden	1P5Z	1NJF	2ZIA		Sc=5.90774, min distance = 2.30507
DPO3X_ECOLI I	6031 Uric	ECX1_PYRAB Fu	6022 Aden	2PO0	1NJF	2PNZ		Sc=5.89029, min distance = 2.05687
DPO3X_ECOLI I	6083 ader	SRC_CHICK Full	440317 AT	3DQW	1NJF	3DQX	0.97	Sc=6.40468, min distance = 1.97023
DPO3X_ECOLI I	6132 Cyti	ECX2_PYRAB Fu	6022 Aden	2PO0	1NJF	2PO2		Sc=6.33834, min distance = 2.18414
DPO3X_ECOLI I	6338561 C	MYS2_DICDI Fu	440317 AT	1MMG	1NJF	1D0X		Sc=6.24551, min distance = 2.19395
DPO3X_ECOLI I	6338566 C	MYS2_DICDI Fu	440317 AT	1MMG	1NJF	1D1C		Sc=6.25616, min distance = 2.13107
DPO3X_ECOLI I	91532 AMP	MTNK_BACSU Fu	6022 Aden	2OLC	1NJF	2PUL	0.99	Sc=6.51485, min distance = 2.14040

DPO3X_ECOLI I	9547890 I	CDK2_HUMAN Fu	6022 Aden	1GY3	1NJF	1W8C		Sc=6.28057, min distance = 2.16983
DPO42_SULSO I	15942678	DPOLB_HUMAN Fu	65091 dCT	1MQ3	2ASD	2ISO		Sc=6.01833, min distance = 2.10802
DPO42_SULSO I	15942679	DPOLB_HUMAN Fu	65091 dCT	1MQ3	2ASD	2ISP		Sc=5.99302, min distance = 2.75707
DPO42_SULSO I	187790 de	DPOL_BPR69 Fu	15993 dAT	2OZS	2AGQ	1Q9X		Sc=5.99922, min distance = 2.99669
DPO42_SULSO I	448381 2'	DPOLL_HUMAN Fu	65091 dCT	2PFP	2ASD	2PFN	0.82	Sc=5.89123, min distance = 2.69047
DPO42_SULSO I	5481180 E	POL_HV1B1 Ful	64968 dTT	1RTD	1S0O	1T05		Sc=5.88836, min distance = 1.73281
DPO42_SULSO I	65051 ddT	DPOLL_HUMAN Fu	64968 dTT	2BCV	1S0O	1XSN	0.94	Sc=5.92366, min distance = 2.34405
DPOLB_HUMAN I	15993 dAT	DPO42_SULSO Fu	65091 dCT	2ASD	1MQ3	2AGQ		Sc=5.65545, min distance = 2.51025
DPOLB_HUMAN I	15993 dAT	DPOL_BPR69 Ful	65091 dCT	1Q9Y	1MQ3	2OZS		Sc=5.68751, min distance = 2.36063
DPOLB_HUMAN I	187790 de	DPOL_BPR69 Ful	65091 dCT	1Q9Y	1MQ3	1Q9X		Sc=6.11262, min distance = 2.75868
DPOLB_HUMAN I	64968 dTT	DPO42_SULSO Fu	65091 dCT	2ASD	1MQ3	1S0O	0.75	Sc=5.63713, min distance = 2.63009
DPOLL_HUMAN I	119119 Di	DPO42_SULSO Fu	64968 dTT	1S0O	2BCV	1S97		Sc=5.62371, min distance = 2.44176
DPOLL_HUMAN I	15993 dAT	RIR3_SALTY Ful	64968 dTT	1PEQ	2BCV	1PEU		Sc=5.89398, min distance = 2.01990
DPOLL_HUMAN I	446577 dc	DPO42_SULSO Fu	64968 dTT	1S0O	2BCV	1JXL		Sc=5.62437, min distance = 2.63524
DPOLL_HUMAN I	5481180 E	POL_HV1B1 Ful	64968 dTT	1RTD	2BCV	1T05		Sc=5.84425, min distance = 2.26566
DPOLL_HUMAN I	65304 ddA	DPOL_BPT7 Ful	65051 ddT	1TKD	1XSN	1SL2		Sc=5.91307, min distance = 2.62055
DPOLL_HUMAN I	656930 DD	DPO42_SULSO Fu	64968 dTT	1S0O	2BCV	1S9F		Sc=6.09606, min distance = 2.77302
DPOLM_MOUSE I	119119 Di	DPOL_BPT7 Ful	65051 ddT	1TKD	2IHM	1TK0		Sc=5.8234, min distance = 2.358353
DPOLM_MOUSE I	446090 1h	NDKC_DICDI Fu	65051 ddT	1F3F	2IHM	1HIY		Sc=5.9464, min distance = 0.937613
DPOLM_MOUSE I	446577 dc	DPOL_BPT7 Ful	65051 ddT	1TKD	2IHM	1T7P		Sc=5.94078, min distance = 2.30315
DPOLM_MOUSE I	6022 Aden	NDKC_DICDI Fu	65051 ddT	1F3F	2IHM	1KDN		Sc=5.89525, min distance = 2.11104
DPOLM_MOUSE I	64968 dTT	DPOLL_HUMAN Fu	65051 ddT	1XSN	2IHM	2BCV	0.94	Sc=5.91045, min distance = 2.81504
DPOLM_MOUSE I	65304 ddA	DPOL_BPT7 Ful	65051 ddT	1TKD	2IHM	1SL2		Sc=5.89903, min distance = 2.23285
DPOL_BPPH2 Fu	13711 dec	DNK_DROME Ful	65103 dGT	2VP2	2PYJ	2VP5		Sc=5.80551, min distance = 2.66446
DPOL_BPPH2 Fu	15993 dAT	DPO42_SULSO Fu	65103 dGT	2JEJ	2PYJ	2AGQ	0.8	Sc=5.92436, min distance = 2.33408
DPOL_BPR69 Fu	3758 IBMX	PDE5A_HUMAN Fu	6804 guan	1T9S	1WAJ	1RKP		Sc=6.05182, min distance = 2.08639
DPOL_BPR69 Fu	449109 CI	FTSZ1_METJA Fu	8977 1dar	2VAP	1CLQ	1W5A		Sc=5.65841, min distance = 2.14111
DPOL_BPR69 Fu	5481180 E	POL_HV1B1 Ful	64968 dTT	1RTD	3CQ8	1T05		Sc=5.85201, min distance = 2.29562
DPOL_BPR69 Fu	6022 Aden	NDK_PYRHO Ful	6804 guan	2DXF	1WAJ	2DYA	0.8	Sc=5.63523, min distance = 2.02206
DPOL_BPR69 Fu	6022 Aden	O33839_THEMA I	8977 1dar	1XJE	1CLQ	1XJK	0.8	Sc=5.9388, min distance = 1.787605
DPOL_BPR69 Fu	6030 Uric	RNMC_MOMCH Ful	6804 guan	1J1F	1WAJ	1UCD		Sc=6.21298, min distance = 2.00208
DPOL_BPR69 Fu	6083 ader	NDK_PYRHO Ful	6804 guan	2DXF	1WAJ	2DXD	0.8	Sc=6.25373, min distance = 2.53863
DPOL_BPR69 Fu	65103 dGT	DPO42_SULSO Fu	65091 dCT	2ASD	1Q9Y	2JEJ		Sc=5.60855, min distance = 2.66588
DPOL_BPR69 Fu	73323 Xar	HGXR_TOXGO Ful	6804 guan	1QK3	1WAJ	1QK5	0.95	Sc=5.91746, min distance = 2.45765
DPOL_BPT7 Ful	23624247	WRBA_ECOLI Ful	62551 Pen	3B6K	1TK5	3B6I		Sc=5.80535, min distance = 2.01753
DPP4_HUMAN Fu	227 anthr	OXLA_AGKRH Ful	439554 fu	2IID	1TK3	1F8S		Sc=5.80519, min distance = 0.90001
DPP4_HUMAN Fu	323 couma	TRFL_BOVIN Ful	444205 CI	2G93	3BJM	3CRB		Sc=5.75539, min distance = 1.30946

DPP4_HUMAN Fu	439353 be	TRFL_BOVIN Fu	444205 CI	2G93	3BJM	2DWJ			Sc=5.64849, min distance = 2.04130
DPP6_HUMAN Fu	445686 CI	CARP_YEAST Fu	185698 al	1DPJ	1XFD	1FQ4			Sc=5.83292, min distance = 1.04854
DPS_STRSU Fu	10918 Car	OCTC_MOUSE Fu	23831 HEF	1XL7	2CF7	1XL8			Sc=5.62959, min distance = 1.88835
DPS_STRSU Fu	64689 bet	O33897_RHOMR	23831 HEF	1H0B	2CF7	2BWA			Sc=5.66326, min distance = 1.42734
DPS_STRSU Fu	8172 Tric	THYX_THEMA Fu	23831 HEF	1O28	2CF7	1O26			Sc=5.70525, min distance = 0.40743
DPYD_PIG Ful	11987634	GLPD_ECOLI Fu	444188 CI	2QCU	1GTE	2R4E		0.97	Sc=6.4433, min distance = 1.909735
DPYD_PIG Ful	16740985	FRDA_SHEFR Fu	444188 CI	1M64	1GTE	1E39		0.93	Sc=6.43763, min distance = 2.20957
DPYD_PIG Ful	440141 9i	GSHR_HUMAN Fu	5886 NADF	3DJJ	1GTH	1GRA			Sc=5.92707, min distance = 1.26513
DPYD_PIG Ful	444502 CI	FRDA_SHEFR Fu	444188 CI	1M64	1GTE	1QJD		0.98	Sc=6.43744, min distance = 2.30287
DPYD_PIG Ful	446013 1,	O28603_ARCFU	444188 CI	1JNR	1GTE	1JNZ		0.94	Sc=6.44609, min distance = 2.22779
DPYD_PIG Ful	448076 CI	CYB2_YEAST Fu	444243 FA	1KBI	1GTE	1SZG		0.76	Sc=6.20746, min distance = 2.09297
DPYD_PIG Ful	449077 CI	FMO1_SCHPO Fu	444188 CI	2GV8	1GTE	1VQW		1	Sc=6.43887, min distance = 2.04182
DPYD_PIG Ful	449465 CI	GSHR_HUMAN Fu	444188 CI	3DK9	1GTE	3GRT		0.98	Sc=6.44083, min distance = 2.34371
DRTS_PLAFK Fu	126941 me	DYR_HUMAN Ful	5886 NADF	1KMS	1J3K	1U72	32.63		Sc=6.21484, min distance = 1.44809
DRTS_PLAFK Fu	126941 me	DYR_PNECA Ful	5886 NADF	2FZI	1J3K	3CD2	31.41		Sc=6.19707, min distance = 2.01879
DRTS_PLAFK Fu	130731 PT	DYR_HUMAN Ful	5886 NADF	1KMS	1J3K	1OHJ	32.63		Sc=6.11445, min distance = 1.70407
DRTS_PLAFK Fu	148138 Lc	DYR_ECOLI Ful	5886 NADF	1RA2	1J3K	1DYJ	33.93		Sc=6.11588, min distance = 2.02728
DRTS_PLAFK Fu	158376 Dp	DYR_PNECA Ful	5886 NADF	2FZI	1J3K	1DAJ	31.41		Sc=5.85601, min distance = 2.24605
DRTS_PLAFK Fu	165230 1a	DYR_ECOLI Ful	5886 NADF	1RA2	1J3K	1RA9	33.93		Sc=6.07255, min distance = 2.62506
DRTS_PLAFK Fu	16741136	DYR_ECOLI Ful	5886 NADF	1RA2	1J3K	1RX9	33.93	1	Sc=6.02232, min distance = 2.61584
DRTS_PLAFK Fu	4369003 C	DYR_ECOLI Ful	5886 NADF	1RA2	1J3K	1TDR	33.93		Sc=5.73654, min distance = 2.09865
DRTS_PLAFK Fu	440141 9i	G6PD_LEUME Fu	5886 NADF	1H9A	1J3K	1E7Y			Sc=5.96627, min distance = 2.20486
DRTS_PLAFK Fu	445175 DZ	DYR_HUMAN Ful	5886 NADF	1KMS	1J3K	2DHF	32.63		Sc=6.22071, min distance = 1.74808
DRTS_PLAFK Fu	446006 CI	DYR_ECOLI Ful	5886 NADF	1RA2	1J3K	1RX4	33.93		Sc=6.22443, min distance = 2.30120
DRTS_PLAFK Fu	446246 CI	DYR_CANAL Ful	5886 NADF	1AOE	1J3K	1IA3	33.77		Sc=6.12915, min distance = 1.44861
DRTS_PLAFK Fu	448303 CI	DYR_ECOLI Ful	5886 NADF	1RA2	1J3K	1RA3	33.93		Sc=6.14011, min distance = 2.50690
DRTS_PLAFK Fu	448305 CI	DYR_ECOLI Ful	5886 NADF	1RA2	1J3K	1RB3	33.93		Sc=6.21164, min distance = 2.47066
DRTS_PLAFK Fu	448308 CI	DYR_ECOLI Ful	5886 NADF	1RA2	1J3K	1RC4	33.93		Sc=6.28816, min distance = 2.20756
DRTS_PLAFK Fu	449049 TA	DYR_PNECA Ful	5886 NADF	2FZI	1J3K	1VJ3	31.41		Sc=6.22924, min distance = 2.10461
DRTS_PLAFK Fu	449577 CI	DYR_ECOLI Ful	5886 NADF	1RA2	1J3K	6DFR	33.93		Sc=6.18373, min distance = 2.46636
DRTS_PLAFK Fu	6037 foli	DYR_HUMAN Ful	5886 NADF	1KMS	1J3K	1DRF	32.63		Sc=6.17201, min distance = 2.37624
DRTS_PLAFK Fu	6037 foli	DYR_PNECA Ful	5886 NADF	2FZI	1J3K	2CD2	31.41		Sc=5.72724, min distance = 2.00021
DRTS_PLAFK Fu	6540275 i	DYR_MYCTU Ful	5886 NADF	1DF7	1J3K	2CIG	33.66	0.93	Sc=6.433, min distance = 2.38337428
DRTS_PLAFK Fu	72440 D-A	DYR_ECOLI Ful	5886 NADF	1RA2	1J3K	1DDS	33.93		Sc=6.21171, min distance = 1.96957
DRTS_PLAFK Fu	93114 Opr	DRTS_PLAVI Fu	4993 pyri	2BL9	1J3J	2BLC	69.16	0.92	Sc=6.10404, min distance = 2.27737
DRTS_PLAVI Fu	126941 me	DYR_ECOLI Ful	5886 NADF	1RA2	2BL9	4DFR	29.63		Sc=5.61328, min distance = 2.12119
DRTS_PLAVI Fu	126941 me	DYR_PNECA Ful	5886 NADF	2FZI	2BL9	3CD2	31.94		Sc=5.61909, min distance = 2.79563

DRTS_PLAVI F	130731 PT	DYR_HUMAN Ful	5886 NADP	1KMS	2BL9	1OHJ	32.98		Sc=6.01575, min distance = 2.12384
DRTS_PLAVI F	3080718 C	DYR_CHICK Ful	5886 NADP	8DFR	2BL9	1DR2	31.91	0.9	Sc=6.79144, min distance = 2.43986
DRTS_PLAVI F	440141 9i	G6PD_LEUME Fu	5886 NADP	1H9A	2BL9	1E7Y			Sc=5.9406, min distance = 2.298451
DRTS_PLAVI F	446246 CI	DYR_CANAL Ful	5886 NADP	1AOE	2BL9	1IA3	31.25		Sc=6.13745, min distance = 1.99967
DRTS_PLAVI F	448308 CI	DYR_ECOLI Ful	5886 NADP	1RA2	2BL9	1RC4	29.63		Sc=6.28833, min distance = 2.45563
DRTS_PLAVI F	449049 TA	DYR_PNECA Ful	5886 NADP	2FZI	2BL9	1VJ3	31.94		Sc=6.06177, min distance = 2.42959
DRTS_TRYCR F	126941 me	DYR_ECOLI Ful	5886 NADP	1RA2	2H2Q	4DFR			Sc=5.73974, min distance = 2.02838
DRTS_TRYCR F	126941 me	DYR_PNECA Ful	5886 NADP	2FZI	2H2Q	3CD2			Sc=6.21541, min distance = 1.76300
DRTS_TRYCR F	148138 Lo	DYR_ECOLI Ful	5886 NADP	1RA2	2H2Q	1DYJ			Sc=6.11797, min distance = 2.05836
DRTS_TRYCR F	158376 Dp	DYR_PNECA Ful	5886 NADP	2FZI	2H2Q	1DAJ			Sc=5.87064, min distance = 1.91000
DRTS_TRYCR F	4369030 C	DYR_ECOLI Ful	5886 NADP	1RA2	2H2Q	1RX5			Sc=6.18615, min distance = 2.41570
DRTS_TRYCR F	445175 DZ	DYR_ECOLI Ful	5886 NADP	1RA2	2H2Q	1DYH			Sc=5.89808, min distance = 1.24173
DRTS_TRYCR F	449049 TA	DYR_PNECA Ful	5886 NADP	2FZI	2H2Q	1VJ3			Sc=6.1949, min distance = 2.409558
DRTS_TRYCR F	5288461 A	DYR_PNECA Ful	5886 NADP	2FZI	2H2Q	1E26			Sc=5.89627, min distance = 1.74863
DRTS_TRYCR F	6037 foli	DYR_PNECA Ful	5886 NADP	2FZI	2H2Q	2CD2			Sc=5.74715, min distance = 1.78071
DRTS_TRYCR F	72440 D-A	DYR_ECOLI Ful	5886 NADP	1RA2	2H2Q	1DDS			Sc=6.18364, min distance = 2.02396
DSBA_ECOLI F	444397 He	RCEL_RHOSH Fu	8182 DODE	2JIY	1TI1	1RZH			Sc=5.67964, min distance = 2.31422
DSBA_ECOLI F	444397 He	RCEM_RHOSH Fu	8182 DODE	2JIY	1TI1	1RZH			Sc=5.66724, min distance = 2.34153
DUS18_HUMAN	445939 1g	PPTA_ECOLI Fu	23831 HEP	1GYX	2ESB	1GYY			Sc=5.83709, min distance = 2.17824
DUT_EBV Full	145729 1c	DUT_MYCTU Ful	448381 2'	1SIX	2BT1	1SLH		0.98	Sc=6.27556, min distance = 2.55764
DUT_EBV Full	445527 DU	DUT_MYCTU Ful	448381 2'	1SIX	2BT1	1SM8		0.98	Sc=5.67493, min distance = 2.40596
DXR_ECOLI Fu	440141 9i	DHB1_HUMAN Fu	5886 NADP	1QYV	1Q0Q	1QYW			Sc=6.37786, min distance = 2.28350
DXR_ECOLI Fu	440334 Ni	DAPB_ECOLI Fu	5886 NADP	1DIH	1Q0Q	1DRW		0.91	Sc=6.75955, min distance = 2.17170
DXR_MYCTU Fu	16129587	G3PA_SPIOL Fu	5886 NADP	1RM4	2JD1	2PKR			Sc=6.75765, min distance = 1.99135
DXR_MYCTU Fu	16741136	DYR_ECOLI Ful	5886 NADP	1RA2	2JD1	1RX9		1	Sc=5.92099, min distance = 2.00842
DXR_MYCTU Fu	440141 9i	DHB1_HUMAN Fu	5886 NADP	1QYV	2JD1	1QYW			Sc=6.32378, min distance = 2.14275
DXR_MYCTU Fu	440334 Ni	DAPB_ECOLI Fu	5886 NADP	1DIH	2JD1	1DRW		0.91	Sc=6.00262, min distance = 2.19401
DXR_MYCTU Fu	443201 DC	DXR_ECOLI Ful	572 fosmi	1Q0H	2JCZ	1Q0Q			Sc=5.80589, min distance = 2.50367
DXR_ZYMMO Fu	5893 nadi	G6PD_LEUME Fu	440141 9i	1E7Y	1R0L	1H94			Sc=6.26777, min distance = 1.82827
DXS_DEIRA Fu	124687 1r	ODP1_ECOLI Fu	1132 thia	1L8A	2O1X	1RP7		0.79	Sc=6.03133, min distance = 1.93325
DXS_DEIRA Fu	24836827	BZNB_PSEFL Fu	1132 thia	2UZ1	2O1X	3D7K			Sc=5.99527, min distance = 1.96783
DXS_DEIRA Fu	444421 CI	MDLC_PSEPU Fu	1132 thia	1BFD	2O1X	2FWN		0.82	Sc=6.46346, min distance = 2.39604
DXS_DEIRA Fu	445886 CI	TKT1_YEAST Fu	1132 thia	1TRK	2O1X	1GPU		0.91	Sc=6.54563, min distance = 2.21024
DXS_DEIRA Fu	448671 2-	ILVB_YEAST Fu	1132 thia	1N0H	2O1X	1T9B			Sc=5.86422, min distance = 2.58120
DXS_DEIRA Fu	448673 PE	ILVB_YEAST Fu	1132 thia	1N0H	2O1X	1T9B			Sc=5.81659, min distance = 2.61812
DXS_DEIRA Fu	448721 N3	TKT1_YEAST Fu	1132 thia	1TRK	2O1X	1TKA			Sc=6.45314, min distance = 2.48625
DXS_DEIRA Fu	448722 1t	TKT1_YEAST Fu	1132 thia	1TRK	2O1X	1TKB		0.78	Sc=6.45085, min distance = 2.40056

DXS_DEIRA Fu	448723 1t	TKT1_YEAST Fu	1132 thia	1TRK	2O1X	1TKC		0.77	Sc=6.49251, min distance = 2.54192
DXS_DEIRA Fu	6102750 T	ODBA_HUMAN Fu	1132 thia	2BFD	2O1X	2BEV		0.9	Sc=6.60557, min distance = 2.14138
DXS_DEIRA Fu	6518187 2	POXB_LACPL Fu	1132 thia	2EZ4	2O1X	2EZ8		0.87	Sc=6.14564, min distance = 2.47392
DXS_ECOLI Fu	124687 1r	ODP1_ECOLI Fu	1132 thia	1L8A	2O1S	1RP7		0.79	Sc=6.42467, min distance = 2.18093
DXS_ECOLI Fu	445886 CI	TKT1_YEAST Fu	1132 thia	1TRK	2O1S	1GPU		0.91	Sc=6.0402, min distance = 2.623690
DXS_ECOLI Fu	448671 2-	ILVB_YEAST Fu	1132 thia	1N0H	2O1S	1T9B			Sc=5.81104, min distance = 2.63768
DXS_ECOLI Fu	448673 PE	ILVB_YEAST Fu	1132 thia	1N0H	2O1S	1T9B			Sc=5.67313, min distance = 2.48611
DXS_ECOLI Fu	448721 N3	TKT1_YEAST Fu	1132 thia	1TRK	2O1S	1TKA			Sc=6.37773, min distance = 2.68691
DXS_ECOLI Fu	448722 1t	TKT1_YEAST Fu	1132 thia	1TRK	2O1S	1TKB		0.78	Sc=6.3729, min distance = 2.644657
DXS_ECOLI Fu	448723 1t	TKT1_YEAST Fu	1132 thia	1TRK	2O1S	1TKC		0.77	Sc=6.42189, min distance = 2.47684
DXS_ECOLI Fu	6102647 C	ODBA_HUMAN Fu	1132 thia	2BFD	2O1S	1WCI		0.9	Sc=6.56873, min distance = 2.10682
DXS_ECOLI Fu	6102749 T	ODBA_HUMAN Fu	1132 thia	2BFD	2O1S	2J9F		0.92	Sc=6.53539, min distance = 2.04566
DXS_ECOLI Fu	6102750 T	ODBA_HUMAN Fu	1132 thia	2BFD	2O1S	2BEV		0.9	Sc=6.56359, min distance = 1.99129
DXS_ECOLI Fu	6102751 T	ODBA_HUMAN Fu	1132 thia	2BFD	2O1S	2BEW		0.76	Sc=6.62232, min distance = 2.12060
DYR1A_HUMAN	23624247	WRBA_ECOLI Fu	62551 Pen	3B6K	2VX3	3B6I			Sc=5.72718, min distance = 0
DYR_CANAL Fu	126941 me	DYR_ECOLI Ful	5886 NADP	1RA2	1AOE	4DFR	34.9		Sc=5.62129, min distance = 2.41121
DYR_CANAL Fu	126941 me	DYR_PNECA Ful	5886 NADP	2FZI	1AOE	3CD2	36.1		Sc=5.80952, min distance = 2.77767
DYR_CANAL Fu	130731 PT	DYR_HUMAN Ful	5886 NADP	1KMS	1AOE	1OHJ	31.75		Sc=6.0402, min distance = 2.216676
DYR_CANAL Fu	15942680	6PGD_LACLM Fu	5886 NADP	2IZ0	1AOE	2IZ1			Sc=6.28155, min distance = 2.27852
DYR_CANAL Fu	165230 1a	DYR_ECOLI Ful	5886 NADP	1RA2	1AOE	1RA9	34.9		Sc=6.53133, min distance = 2.10804
DYR_CANAL Fu	16741136	DYR_ECOLI Ful	5886 NADP	1RA2	1AOE	1RX9	34.9	1	Sc=6.4913, min distance = 2.567740
DYR_CANAL Fu	3080718 C	DYR_CHICK Ful	5886 NADP	8DFR	1AOE	1DR2	38.56	0.9	Sc=6.81093, min distance = 2.36487
DYR_CANAL Fu	4369003 C	DYR_ECOLI Ful	5886 NADP	1RA2	1AOE	1TDR	34.9		Sc=5.6452, min distance = 2.015101
DYR_CANAL Fu	440141 9i	G6PD_LEUME Fu	5886 NADP	1H9A	1AOE	1E7Y			Sc=6.47542, min distance = 1.69523
DYR_CANAL Fu	445175 DZ	DYR_HUMAN Ful	5886 NADP	1KMS	1AOE	2DHF	31.75		Sc=5.85742, min distance = 1.58386
DYR_CANAL Fu	446006 CI	DYR_ECOLI Ful	5886 NADP	1RA2	1AOE	1RX4	34.9		Sc=6.34164, min distance = 2.17255
DYR_CANAL Fu	448303 CI	DYR_ECOLI Ful	5886 NADP	1RA2	1AOE	1RA3	34.9		Sc=6.59874, min distance = 1.78094
DYR_CANAL Fu	448305 CI	DYR_ECOLI Ful	5886 NADP	1RA2	1AOE	1RB3	34.9		Sc=6.6481, min distance = 2.263315
DYR_CANAL Fu	449049 TA	DYR_PNECA Ful	5886 NADP	2FZI	1AOE	1VJ3	36.1		Sc=6.09318, min distance = 2.70218
DYR_CANAL Fu	449577 CI	DYR_ECOLI Ful	5886 NADP	1RA2	1AOE	6DFR	34.9		Sc=6.63414, min distance = 2.23562
DYR_CHICK Fu	165230 1a	DYR_ECOLI Ful	5886 NADP	1RA2	8DFR	1RA9	30.77		Sc=5.74588, min distance = 2.24619
DYR_CHICK Fu	292661 2,	PTR1_LEIMA Fu	445040 6-	1E92	1DR1	1W0C			Sc=5.99137, min distance = 2.78571
DYR_CHICK Fu	440141 9i	G6PD_LEUME Fu	5886 NADP	1H9A	8DFR	1E7Y			Sc=6.38, min distance = 2.33904766
DYR_CHICK Fu	449049 TA	DYR_PNECA Ful	5886 NADP	2FZI	8DFR	1VJ3	37.07		Sc=6.13938, min distance = 2.28185
DYR_CHICK Fu	449577 CI	DYR_ECOLI Ful	5886 NADP	1RA2	8DFR	6DFR	30.77		Sc=6.15934, min distance = 2.41804
DYR_CHICK Fu	6540275 i	DYR_MYCTU Ful	5886 NADP	1DF7	8DFR	2CIG	33.57	0.93	Sc=6.13053, min distance = 2.06604
DYR_ECOLI Fu	11987844	DYR_MOUSE Ful	126941 me	1U70	4DFR	2FZJ			Sc=6.4102, min distance = 2.141072

DYR_ECOLI	Fu	130731 PT	DYR_HUMAN	Ful	445175 DZ	2DHF	1DYH	1OHJ	29.41	0.78	Sc=6.47942, min distance = 1.732305
DYR_ECOLI	Fu	130731 PT	DYR_HUMAN	Ful	5886 NADP	1KMS	1RA2	1OHJ	29.41		Sc=6.11514, min distance = 2.098377
DYR_ECOLI	Fu	130731 PT	DYR_HUMAN	Ful	6037 foli	1DRF	1RA2	1OHJ	29.41	0.8	Sc=6.18611, min distance = 2.265156
DYR_ECOLI	Fu	158376 Dp	DYR_HUMAN	Ful	445175 DZ	2DHF	1DYH	1HFP	29.41		Sc=6.60285, min distance = 1.472451
DYR_ECOLI	Fu	158376 Dp	DYR_HUMAN	Ful	6037 foli	1DRF	1RA2	1HFP	29.41		Sc=6.12515, min distance = 2.205401
DYR_ECOLI	Fu	158376 Dp	DYR_PNECA	Ful	5886 NADP	2FZI	1RA2	1DAJ	30.64		Sc=5.81723, min distance = 2.493751
DYR_ECOLI	Fu	158376 Dp	DYR_PNECA	Ful	6037 foli	2CD2	1RA2	1DAJ	30.64		Sc=5.78919, min distance = 2.607329
DYR_ECOLI	Fu	2429 BROM	DYR_MYCTU	Ful	126941 me	1DF7	4DFR	1DG7	41.67		Sc=6.19033, min distance = 2.185007
DYR_ECOLI	Fu	3080718 C	DYR_CHICK	Ful	5886 NADP	8DFR	1RA2	1DR2	30.77	0.9	Sc=6.07158, min distance = 2.298427
DYR_ECOLI	Fu	444617 AI	DYR_HUMAN	Ful	126941 me	1U72	4DFR	1BOZ	29.41		Sc=6.31046, min distance = 1.999848
DYR_ECOLI	Fu	444617 AI	DYR_HUMAN	Ful	445175 DZ	2DHF	1DYH	1BOZ	29.41		Sc=6.28348, min distance = 2.913850
DYR_ECOLI	Fu	444617 AI	DYR_HUMAN	Ful	6037 foli	1DRF	1RA2	1BOZ	29.41		Sc=6.27537, min distance = 2.655616
DYR_ECOLI	Fu	446246 CI	DYR_CANAL	Ful	5886 NADP	1AOE	1RA2	1IA3	34.9		Sc=6.14738, min distance = 1.068877
DYR_ECOLI	Fu	446744 Pt	DYR_PNECA	Ful	126941 me	3CD2	4DFR	1KLK	30.64		Sc=6.40351, min distance = 1.988673
DYR_ECOLI	Fu	446744 Pt	DYR_PNECA	Ful	6037 foli	2CD2	1RA2	1KLK	30.64		Sc=6.40899, min distance = 2.553260
DYR_ECOLI	Fu	446753 1k	DYR_HUMAN	Ful	445175 DZ	2DHF	1DYH	1KMV	29.41		Sc=6.26472, min distance = 2.814748
DYR_ECOLI	Fu	447021 AI	DYR_PNECA	Ful	126941 me	3CD2	4DFR	1LY3	30.64		Sc=6.23906, min distance = 2.330216
DYR_ECOLI	Fu	447022 CI	DYR_PNECA	Ful	126941 me	3CD2	4DFR	1LY4	30.64		Sc=6.28068, min distance = 2.265640
DYR_ECOLI	Fu	447022 CI	DYR_PNECA	Ful	6037 foli	2CD2	1RA2	1LY4	30.64		Sc=6.22198, min distance = 2.984638
DYR_ECOLI	Fu	447229 AI	DYR_HUMAN	Ful	445175 DZ	2DHF	1DYH	1MVT	29.41		Sc=6.25363, min distance = 3.137417
DYR_ECOLI	Fu	448492 TQ	DYR_HUMAN	Ful	445175 DZ	2DHF	1DYH	1S3U	29.41		Sc=6.21911, min distance = 2.519898
DYR_ECOLI	Fu	448493 TQ	DYR_HUMAN	Ful	445175 DZ	2DHF	1DYH	1S3V	29.41		Sc=6.20876, min distance = 2.714083
DYR_ECOLI	Fu	448494 CI	DYR_PNECA	Ful	126941 me	3CD2	4DFR	1S3Y	30.64		Sc=6.22737, min distance = 2.365739
DYR_ECOLI	Fu	490573 Di	DYR_PNECA	Ful	126941 me	3CD2	4DFR	2FZH	30.64		Sc=6.38661, min distance = 2.413084
DYR_ECOLI	Fu	5288461 A	DYR_PNECA	Ful	126941 me	3CD2	4DFR	1E26	30.64	0.83	Sc=5.8313, min distance = 2.1571309
DYR_ECOLI	Fu	54369 Pir	DYR_HUMAN	Ful	126941 me	1U72	4DFR	1U71	29.41		Sc=6.30385, min distance = 1.991359
DYR_ECOLI	Fu	54369 Pir	DYR_HUMAN	Ful	445175 DZ	2DHF	1DYH	1U71	29.41		Sc=6.27537, min distance = 2.716216
DYR_ECOLI	Fu	5578 trim	DYR_MYCTU	Ful	126941 me	1DF7	4DFR	1DG5	41.67		Sc=6.17209, min distance = 2.232476
DYR_ECOLI	Fu	5578 trim	DYR_PNECA	Ful	126941 me	3CD2	4DFR	1DYR	30.64		Sc=6.13712, min distance = 2.501646
DYR_ECOLI	Fu	5578 trim	DYR_PNECA	Ful	6037 foli	2CD2	1RA2	1DYR	30.64		Sc=6.09202, min distance = 3.034685
DYR_HUMAN	Fu	11987843	DYR_PNECA	Ful	126941 me	3CD2	1U72	2FZI	37.44		Sc=6.46111, min distance = 2.385515
DYR_HUMAN	Fu	11987843	DYR_PNECA	Ful	158376 Dp	1DAJ	1HFP	2FZI	37.44		Sc=6.43096, min distance = 2.228068
DYR_HUMAN	Fu	148138 Lo	DYR_ECOLI	Ful	126941 me	4DFR	1U72	1DYJ	29.41		Sc=6.57355, min distance = 2.460794
DYR_HUMAN	Fu	148138 Lo	DYR_ECOLI	Ful	445175 DZ	1DYH	2DHF	1DYJ	29.41		Sc=6.57994, min distance = 2.424063
DYR_HUMAN	Fu	148138 Lo	DYR_ECOLI	Ful	6037 foli	1RA2	1DRF	1DYJ	29.41		Sc=6.57355, min distance = 2.487406
DYR_HUMAN	Fu	15942680	6PGD_LACLM	Fu	5886 NADP	2IZ0	1KMS	2IZ1			Sc=5.74617, min distance = 2.045929
DYR_HUMAN	Fu	2429 BROM	DYR_MYCTU	Ful	126941 me	1DF7	1U72	1DG7	36.36		Sc=6.20928, min distance = 2.316270

DYR_HUMAN Fu	4369003 C	DYR_ECOLI Ful	126941 me	4DFR	1U72	1TDR	29.41		Sc=6.19707, min distance = 2.331714
DYR_HUMAN Fu	4369003 C	DYR_ECOLI Ful	445175 DZ	1DYH	2DHF	1TDR	29.41		Sc=6.19632, min distance = 2.015290
DYR_HUMAN Fu	4369003 C	DYR_ECOLI Ful	6037 foli	1RA2	1DRF	1TDR	29.41		Sc=5.89688, min distance = 2.421638
DYR_HUMAN Fu	4369030 C	DYR_ECOLI Ful	126941 me	4DFR	1U72	1RX5	29.41		Sc=6.63423, min distance = 2.485769
DYR_HUMAN Fu	4369030 C	DYR_ECOLI Ful	445175 DZ	1DYH	2DHF	1RX5	29.41		Sc=6.64582, min distance = 2.440951
DYR_HUMAN Fu	4369030 C	DYR_ECOLI Ful	6037 foli	1RA2	1DRF	1RX5	29.41		Sc=6.65544, min distance = 2.770950
DYR_HUMAN Fu	440141 9i	G6PD_LEUME Fu	5886 NADP	1H9A	1KMS	1E7Y			Sc=5.96389, min distance = 2.360607
DYR_HUMAN Fu	446744 Pt	DYR_PNECA Ful	126941 me	3CD2	1U72	1KLK	37.44		Sc=6.46262, min distance = 2.211287
DYR_HUMAN Fu	446744 Pt	DYR_PNECA Ful	158376 Dp	1DAJ	1HFP	1KLK	37.44		Sc=6.48975, min distance = 2.269948
DYR_HUMAN Fu	446744 Pt	DYR_PNECA Ful	6037 foli	2CD2	1DRF	1KLK	37.44		Sc=6.53426, min distance = 2.378999
DYR_HUMAN Fu	447021 AI	DYR_PNECA Ful	126941 me	3CD2	1U72	1LY3	37.44		Sc=6.29859, min distance = 2.288105
DYR_HUMAN Fu	447021 AI	DYR_PNECA Ful	158376 Dp	1DAJ	1HFP	1LY3	37.44		Sc=6.23132, min distance = 2.694482
DYR_HUMAN Fu	447021 AI	DYR_PNECA Ful	6037 foli	2CD2	1DRF	1LY3	37.44		Sc=6.29859, min distance = 2.373586
DYR_HUMAN Fu	447022 CI	DYR_PNECA Ful	126941 me	3CD2	1U72	1LY4	37.44		Sc=6.33398, min distance = 2.210941
DYR_HUMAN Fu	447022 CI	DYR_PNECA Ful	158376 Dp	1DAJ	1HFP	1LY4	37.44		Sc=6.24911, min distance = 2.817345
DYR_HUMAN Fu	447022 CI	DYR_PNECA Ful	6037 foli	2CD2	1DRF	1LY4	37.44		Sc=6.33807, min distance = 2.328553
DYR_HUMAN Fu	447238 CI	DYR_ECOLI Ful	126941 me	4DFR	1U72	1RF7	29.41	0.86	Sc=6.11936, min distance = 2.500229
DYR_HUMAN Fu	447238 CI	DYR_ECOLI Ful	6037 foli	1RA2	1DRF	1RF7	29.41	0.96	Sc=6.10871, min distance = 2.585213
DYR_HUMAN Fu	448303 CI	DYR_ECOLI Ful	5886 NADP	1RA2	1KMS	1RA3	29.41		Sc=5.83463, min distance = 2.153552
DYR_HUMAN Fu	448307 CI	DYR_ECOLI Ful	126941 me	4DFR	1U72	1RC4	29.41		Sc=6.19608, min distance = 2.556507
DYR_HUMAN Fu	448307 CI	DYR_ECOLI Ful	445175 DZ	1DYH	2DHF	1RC4	29.41		Sc=6.19212, min distance = 2.603069
DYR_HUMAN Fu	448307 CI	DYR_ECOLI Ful	6037 foli	1RA2	1DRF	1RC4	29.41		Sc=6.20416, min distance = 2.553416
DYR_HUMAN Fu	449049 TA	DYR_PNECA Ful	126941 me	3CD2	1U72	1VJ3	37.44		Sc=6.1209, min distance = 2.6020309
DYR_HUMAN Fu	449049 TA	DYR_PNECA Ful	158376 Dp	1DAJ	1HFP	1VJ3	37.44		Sc=6.15853, min distance = 2.419523
DYR_HUMAN Fu	449049 TA	DYR_PNECA Ful	5886 NADP	2FZI	1KMS	1VJ3	37.44		Sc=6.2062, min distance = 2.0940935
DYR_HUMAN Fu	449049 TA	DYR_PNECA Ful	6037 foli	2CD2	1DRF	1VJ3	37.44		Sc=6.19981, min distance = 2.244986
DYR_HUMAN Fu	490573 Di	DYR_PNECA Ful	126941 me	3CD2	1U72	2FZH	37.44		Sc=6.40803, min distance = 2.315474
DYR_HUMAN Fu	490573 Di	DYR_PNECA Ful	158376 Dp	1DAJ	1HFP	2FZH	37.44		Sc=6.38357, min distance = 2.428813
DYR_HUMAN Fu	5288461 A	DYR_PNECA Ful	126941 me	3CD2	1U72	1E26	37.44	0.83	Sc=6.14236, min distance = 2.642343
DYR_HUMAN Fu	5288461 A	DYR_PNECA Ful	158376 Dp	1DAJ	1HFP	1E26	37.44		Sc=6.1444, min distance = 2.5730460
DYR_HUMAN Fu	5578 trim	DYR_MYCTU Ful	126941 me	1DF7	1U72	1DG5	36.36		Sc=6.1937, min distance = 2.5070343
DYR_HUMAN Fu	5578 trim	DYR_PNECA Ful	126941 me	3CD2	1U72	1DYR	37.44		Sc=6.17763, min distance = 2.522643
DYR_HUMAN Fu	5578 trim	DYR_PNECA Ful	158376 Dp	1DAJ	1HFP	1DYR	37.44		Sc=6.12521, min distance = 2.723364
DYR_HUMAN Fu	5578 trim	DYR_PNECA Ful	6037 foli	2CD2	1DRF	1DYR	37.44		Sc=6.18109, min distance = 2.169352
DYR_LACCA Fu	11987843	DYR_PNECA Ful	126941 me	3CD2	3DFR	2FZI			Sc=6.45007, min distance = 2.336578
DYR_LACCA Fu	11987844	DYR_MOUSE Ful	126941 me	1U70	3DFR	2FZJ			Sc=6.44442, min distance = 2.549398
DYR_LACCA Fu	148138 Lo	DYR_ECOLI Ful	126941 me	4DFR	3DFR	1DYJ			Sc=6.11295, min distance = 2.467005

DYR_LACCA Fu: 165230 1a	DYR_ECOLI Ful: 126941 me 4DFR	3DFR	1RA9			Sc=6.50791, min distance = 2.36543(
DYR_LACCA Fu: 16741136	DYR_ECOLI Ful: 126941 me 4DFR	3DFR	1RX9			Sc=6.46641, min distance = 2.44198'
DYR_LACCA Fu: 2429 BROM	DYR_MYCTU Ful: 126941 me 1DF7	3DFR	1DG7			Sc=6.1937, min distance = 2.3116578
DYR_LACCA Fu: 24798731	DYR_MOUSE Ful: 126941 me 1U70	3DFR	3D84			Sc=6.80321, min distance = 2.07878!
DYR_LACCA Fu: 4369003 C	DYR_ECOLI Ful: 126941 me 4DFR	3DFR	1TDR			Sc=5.68822, min distance = 2.72878(
DYR_LACCA Fu: 4369030 C	DYR_ECOLI Ful: 126941 me 4DFR	3DFR	1RX5			Sc=6.1975, min distance = 2.509973:
DYR_LACCA Fu: 444617 AI	DYR_HUMAN Ful: 126941 me 1U72	3DFR	1BOZ			Sc=6.3264, min distance = 2.428131!
DYR_LACCA Fu: 445175 DZ	DYR_ECOLI Ful: 126941 me 4DFR	3DFR	1DYH		0.85	Sc=6.19759, min distance = 2.43043(
DYR_LACCA Fu: 446744 Pt	DYR_PNECA Ful: 126941 me 3CD2	3DFR	1KLK			Sc=6.43941, min distance = 2.77865:
DYR_LACCA Fu: 446753 1k	DYR_HUMAN Ful: 126941 me 1U72	3DFR	1KMV			Sc=6.34032, min distance = 2.58458!
DYR_LACCA Fu: 447021 AI	DYR_PNECA Ful: 126941 me 3CD2	3DFR	1LY3			Sc=6.26689, min distance = 2.61863!
DYR_LACCA Fu: 447022 CI	DYR_PNECA Ful: 126941 me 3CD2	3DFR	1LY4			Sc=6.31163, min distance = 2.47531:
DYR_LACCA Fu: 447229 AI	DYR_HUMAN Ful: 126941 me 1U72	3DFR	1MVT			Sc=6.33836, min distance = 2.45918+
DYR_LACCA Fu: 447238 CI	DYR_ECOLI Ful: 126941 me 4DFR	3DFR	1RF7		0.86	Sc=6.57207, min distance = 2.30337(
DYR_LACCA Fu: 448303 CI	DYR_ECOLI Ful: 126941 me 4DFR	3DFR	1RA3			Sc=6.59019, min distance = 2.28018(
DYR_LACCA Fu: 448305 CI	DYR_ECOLI Ful: 126941 me 4DFR	3DFR	1RB3			Sc=6.6539, min distance = 1.671892(
DYR_LACCA Fu: 448307 CI	DYR_ECOLI Ful: 126941 me 4DFR	3DFR	1RC4			Sc=5.90869, min distance = 2.30966(
DYR_LACCA Fu: 448492 TÇ	DYR_HUMAN Ful: 126941 me 1U72	3DFR	1S3U			Sc=6.28624, min distance = 2.24411(
DYR_LACCA Fu: 448493 TÇ	DYR_HUMAN Ful: 126941 me 1U72	3DFR	1S3V			Sc=6.28624, min distance = 2.44162:
DYR_LACCA Fu: 448494 CI	DYR_PNECA Ful: 126941 me 3CD2	3DFR	1S3Y			Sc=6.23285, min distance = 2.81871(
DYR_LACCA Fu: 449049 TA	DYR_PNECA Ful: 126941 me 3CD2	3DFR	1VJ3			Sc=6.13447, min distance = 2.37079:
DYR_LACCA Fu: 449577 CI	DYR_ECOLI Ful: 126941 me 4DFR	3DFR	6DFR			Sc=6.61848, min distance = 2.33972:
DYR_LACCA Fu: 490573 Di	DYR_PNECA Ful: 126941 me 3CD2	3DFR	2FZH			Sc=6.40312, min distance = 2.62135:
DYR_LACCA Fu: 54369 Pir	DYR_HUMAN Ful: 126941 me 1U72	3DFR	1U71			Sc=6.33492, min distance = 2.34333(
DYR_LACCA Fu: 5578 trim	DYR_MYCTU Ful: 126941 me 1DF7	3DFR	1DG5			Sc=6.18446, min distance = 2.571528
DYR_LACCA Fu: 5578 trim	DYR_PNECA Ful: 126941 me 3CD2	3DFR	1DYR			Sc=6.16581, min distance = 2.75874+
DYR_LACCA Fu: 5886 NADE	DYR_ECOLI Ful: 126941 me 4DFR	3DFR	1RA2			Sc=6.80824, min distance = 2.380558
DYR_LACCA Fu: 5886 NADE	DYR_PNECA Ful: 126941 me 3CD2	3DFR	2FZI			Sc=6.80321, min distance = 2.12065!
DYR_LACCA Fu: 6037 foli	DYR_PNECA Ful: 126941 me 3CD2	3DFR	2CD2		0.88	Sc=6.16331, min distance = 2.23316+
DYR_MOUSE Fu: 2429 BROM	DYR_MYCTU Ful: 126941 me 1DF7	1U70	1DG7			Sc=6.21548, min distance = 2.28356!
DYR_MOUSE Fu: 4369003 C	DYR_ECOLI Ful: 126941 me 4DFR	1U70	1TDR			Sc=6.1945, min distance = 2.291676+
DYR_MOUSE Fu: 445175 DZ	DYR_ECOLI Ful: 126941 me 4DFR	1U70	1DYH		0.85	Sc=6.626, min distance = 2.16999838
DYR_MOUSE Fu: 445175 DZ	DYR_HUMAN Ful: 126941 me 1U72	1U70	2DHF		0.85	Sc=6.16173, min distance = 2.01281!
DYR_MOUSE Fu: 447022 CI	DYR_PNECA Ful: 126941 me 3CD2	1U70	1LY4			Sc=6.34104, min distance = 2.22238:
DYR_MOUSE Fu: 447238 CI	DYR_ECOLI Ful: 126941 me 4DFR	1U70	1RF7		0.86	Sc=6.10827, min distance = 2.35463(
DYR_MOUSE Fu: 448303 CI	DYR_ECOLI Ful: 126941 me 4DFR	1U70	1RA3			Sc=6.12502, min distance = 2.20531:
DYR_MOUSE Fu: 448307 CI	DYR_ECOLI Ful: 126941 me 4DFR	1U70	1RC4			Sc=6.3187, min distance = 2.390741(

DYR_MOUSE Fu	448450 CI	DYR_ECOLI Ful	126941 me	4DFR	1U70	1RX6			Sc=5.85517, min distance = 2.09935
DYR_MOUSE Fu	490573 Di	DYR_PNECA Ful	126941 me	3CD2	1U70	2FZH			Sc=6.4207, min distance = 2.568191
DYR_MOUSE Fu	5288461 A	DYR_PNECA Ful	126941 me	3CD2	1U70	1E26		0.83	Sc=6.15702, min distance = 2.29253
DYR_MOUSE Fu	5578 trim	DYR_PNECA Ful	126941 me	3CD2	1U70	1DYR			Sc=6.18109, min distance = 2.19557
DYR_MOUSE Fu	6037 foli	DYR_PNECA Ful	126941 me	3CD2	1U70	2CD2		0.88	Sc=5.88086, min distance = 2.32733
DYR_MYCTU Fu	11987843	DYR_PNECA Ful	126941 me	3CD2	1DF7	2FZI	32.28		Sc=6.44943, min distance = 2.59839
DYR_MYCTU Fu	11987843	DYR_PNECA Ful	5578 trim	1DYR	1DG5	2FZI	32.28	0.86	Sc=6.39903, min distance = 2.47894
DYR_MYCTU Fu	11987844	DYR_MOUSE Ful	126941 me	1U70	1DF7	2FZJ			Sc=6.45653, min distance = 2.34665
DYR_MYCTU Fu	130731 PT	DYR_HUMAN Ful	126941 me	1U72	1DF7	1OHJ	36.36	0.89	Sc=6.53197, min distance = 2.61891
DYR_MYCTU Fu	148138 Lo	DYR_ECOLI Ful	126941 me	4DFR	1DF7	1DYJ	41.67		Sc=6.64961, min distance = 1.93733
DYR_MYCTU Fu	158376 Dp	DYR_PNECA Ful	5886 NADP	2FZI	1DF7	1DAJ	32.28		Sc=6.15067, min distance = 2.18530
DYR_MYCTU Fu	15942680	6PGD_LACLM Fu	5886 NADP	2IZ0	1DF7	2IZ1			Sc=6.20345, min distance = 2.75646
DYR_MYCTU Fu	165230 1a	DYR_ECOLI Ful	5886 NADP	1RA2	1DF7	1RA9	41.67		Sc=6.50791, min distance = 2.61307
DYR_MYCTU Fu	16741136	DYR_ECOLI Ful	5886 NADP	1RA2	1DF7	1RX9	41.67	1	Sc=6.45591, min distance = 2.70787
DYR_MYCTU Fu	4369003 C	DYR_ECOLI Ful	126941 me	4DFR	1DF7	1TDR	41.67		Sc=5.9025, min distance = 2.341187
DYR_MYCTU Fu	4369030 C	DYR_ECOLI Ful	126941 me	4DFR	1DF7	1RX5	41.67		Sc=6.19532, min distance = 2.25255
DYR_MYCTU Fu	444617 AI	DYR_HUMAN Ful	126941 me	1U72	1DF7	1BOZ	36.36		Sc=6.34104, min distance = 2.21229
DYR_MYCTU Fu	445175 DZ	DYR_ECOLI Ful	126941 me	4DFR	1DF7	1DYH	41.67	0.85	Sc=6.64384, min distance = 1.89821
DYR_MYCTU Fu	445175 DZ	DYR_HUMAN Ful	126941 me	1U72	1DF7	2DHF	36.36	0.85	Sc=6.1915, min distance = 2.030330
DYR_MYCTU Fu	445175 DZ	DYR_HUMAN Ful	5886 NADP	1KMS	1DF7	2DHF	36.36		Sc=6.20899, min distance = 2.14054
DYR_MYCTU Fu	446246 CI	DYR_CANAL Ful	5886 NADP	1AOE	1DF7	1IA3	34		Sc=6.59466, min distance = 1.96037
DYR_MYCTU Fu	446744 Pt	DYR_PNECA Ful	126941 me	3CD2	1DF7	1KLK	32.28		Sc=6.41432, min distance = 2.57497
DYR_MYCTU Fu	447021 AI	DYR_PNECA Ful	126941 me	3CD2	1DF7	1LY3	32.28		Sc=6.26491, min distance = 2.35940
DYR_MYCTU Fu	447238 CI	DYR_ECOLI Ful	126941 me	4DFR	1DF7	1RF7	41.67	0.86	Sc=6.58226, min distance = 2.41892
DYR_MYCTU Fu	448303 CI	DYR_ECOLI Ful	5886 NADP	1RA2	1DF7	1RA3	41.67		Sc=6.58688, min distance = 2.47881
DYR_MYCTU Fu	448305 CI	DYR_ECOLI Ful	5886 NADP	1RA2	1DF7	1RB3	41.67		Sc=6.65479, min distance = 2.33948
DYR_MYCTU Fu	448307 CI	DYR_ECOLI Ful	126941 me	4DFR	1DF7	1RC4	41.67		Sc=6.19608, min distance = 2.37068
DYR_MYCTU Fu	448492 TQ	DYR_HUMAN Ful	126941 me	1U72	1DF7	1S3U	36.36		Sc=6.29343, min distance = 2.24231
DYR_MYCTU Fu	448494 CI	DYR_PNECA Ful	126941 me	3CD2	1DF7	1S3Y	32.28		Sc=6.25297, min distance = 2.65086
DYR_MYCTU Fu	448494 CI	DYR_PNECA Ful	5578 trim	1DYR	1DG5	1S3Y	32.28		Sc=6.20874, min distance = 2.37800
DYR_MYCTU Fu	449049 TA	DYR_PNECA Ful	126941 me	3CD2	1DF7	1VJ3	32.28		Sc=6.1949, min distance = 2.331281
DYR_MYCTU Fu	449049 TA	DYR_PNECA Ful	5578 trim	1DYR	1DG5	1VJ3	32.28		Sc=6.08092, min distance = 2.26495
DYR_MYCTU Fu	449049 TA	DYR_PNECA Ful	5886 NADP	2FZI	1DF7	1VJ3	32.28		Sc=6.12611, min distance = 2.54887
DYR_MYCTU Fu	449577 CI	DYR_ECOLI Ful	5886 NADP	1RA2	1DF7	6DFR	41.67		Sc=6.6142, min distance = 2.580022
DYR_MYCTU Fu	490573 Di	DYR_PNECA Ful	126941 me	3CD2	1DF7	2FZH	32.28		Sc=6.40312, min distance = 2.43195
DYR_MYCTU Fu	490573 Di	DYR_PNECA Ful	5578 trim	1DYR	1DG5	2FZH	32.28	0.8	Sc=6.36111, min distance = 2.51710
DYR_MYCTU Fu	54369 Pir	DYR_HUMAN Ful	126941 me	1U72	1DF7	1U71	36.36		Sc=6.3286, min distance = 2.462241

DYR_MYCTU Fu	6037 foli	DYR_ECOLI Ful	126941 me	4DFR	1DF7	1RA2	41.67	0.88	Sc=6.18129, min distance = 2.46690
DYR_MYCTU Fu	6037 foli	DYR_PNECA Ful	126941 me	3CD2	1DF7	2CD2	32.28	0.88	Sc=6.17415, min distance = 2.33793
DYR_MYCTU Fu	6037 foli	DYR_PNECA Ful	5578 trim	1DYR	1DG5	2CD2	32.28		Sc=5.80835, min distance = 2.23130
DYR_PNECA Fu	11987844	DYR_MOUSE Ful	126941 me	1U70	3CD2	2FZJ			Sc=6.42088, min distance = 2.43387
DYR_PNECA Fu	130731 PT	DYR_HUMAN Ful	5886 NADF	1KMS	2FZI	1OHJ	37.44		Sc=6.10028, min distance = 2.18585
DYR_PNECA Fu	130731 PT	DYR_HUMAN Ful	6037 foli	1DRF	2CD2	1OHJ	37.44	0.8	Sc=6.54866, min distance = 2.07303
DYR_PNECA Fu	148138 Lc	DYR_ECOLI Ful	126941 me	4DFR	3CD2	1DYJ	30.64		Sc=6.13541, min distance = 2.12552
DYR_PNECA Fu	148138 Lc	DYR_ECOLI Ful	6037 foli	1RA2	2CD2	1DYJ	30.64		Sc=6.09007, min distance = 2.03797
DYR_PNECA Fu	15942680	6PGD_LACLM Fu	5886 NADF	2IZ0	2FZI	2IZ1			Sc=5.87896, min distance = 0.72604
DYR_PNECA Fu	165230 1a	DYR_ECOLI Ful	126941 me	4DFR	3CD2	1RA9	30.64		Sc=6.47599, min distance = 2.19485
DYR_PNECA Fu	165230 1a	DYR_ECOLI Ful	5886 NADF	1RA2	2FZI	1RA9	30.64		Sc=6.48384, min distance = 2.31261
DYR_PNECA Fu	16741136	DYR_ECOLI Ful	126941 me	4DFR	3CD2	1RX9	30.64		Sc=6.4265, min distance = 2.129377
DYR_PNECA Fu	16741136	DYR_ECOLI Ful	5886 NADF	1RA2	2FZI	1RX9	30.64	1	Sc=6.43349, min distance = 2.26639
DYR_PNECA Fu	16741136	DYR_ECOLI Ful	6037 foli	1RA2	2CD2	1RX9	30.64		Sc=6.43349, min distance = 2.22064
DYR_PNECA Fu	2429 BROM	DYR_MYCTU Ful	126941 me	1DF7	3CD2	1DG7	32.28		Sc=6.19684, min distance = 2.33110
DYR_PNECA Fu	3080718 C	DYR_CHICK Ful	5886 NADF	8DFR	2FZI	1DR2	37.07	0.9	Sc=6.78984, min distance = 2.56078
DYR_PNECA Fu	4369003 C	DYR_ECOLI Ful	126941 me	4DFR	3CD2	1TDR	30.64		Sc=5.88758, min distance = 2.38548
DYR_PNECA Fu	4369030 C	DYR_ECOLI Ful	126941 me	4DFR	3CD2	1RX5	30.64		Sc=5.89055, min distance = 2.09046
DYR_PNECA Fu	444617 AI	DYR_HUMAN Ful	126941 me	1U72	3CD2	1BOZ	37.44		Sc=6.31866, min distance = 2.31469
DYR_PNECA Fu	444617 AI	DYR_HUMAN Ful	158376 Dp	1HFP	1DAJ	1BOZ	37.44		Sc=6.34918, min distance = 2.05921
DYR_PNECA Fu	445175 DZ	DYR_ECOLI Ful	126941 me	4DFR	3CD2	1DYH	30.64	0.85	Sc=6.20369, min distance = 1.83970
DYR_PNECA Fu	445175 DZ	DYR_ECOLI Ful	6037 foli	1RA2	2CD2	1DYH	30.64	0.94	Sc=5.88414, min distance = 2.03079
DYR_PNECA Fu	445175 DZ	DYR_HUMAN Ful	5886 NADF	1KMS	2FZI	2DHF	37.44		Sc=5.91237, min distance = 1.23020
DYR_PNECA Fu	445175 DZ	DYR_HUMAN Ful	6037 foli	1DRF	2CD2	2DHF	37.44	0.94	Sc=6.20167, min distance = 2.25985
DYR_PNECA Fu	446006 CI	DYR_ECOLI Ful	126941 me	4DFR	3CD2	1RX4	30.64		Sc=6.30591, min distance = 1.79463
DYR_PNECA Fu	446006 CI	DYR_ECOLI Ful	5886 NADF	1RA2	2FZI	1RX4	30.64		Sc=6.62635, min distance = 2.32475
DYR_PNECA Fu	446246 CI	DYR_CANAL Ful	5886 NADF	1AOE	2FZI	1IA3	36.1		Sc=6.55956, min distance = 2.41540
DYR_PNECA Fu	446752 SF	DYR_HUMAN Ful	158376 Dp	1HFP	1DAJ	1KMS	37.44		Sc=6.38975, min distance = 2.38658
DYR_PNECA Fu	446753 1k	DYR_HUMAN Ful	126941 me	1U72	3CD2	1KMV	37.44		Sc=6.2974, min distance = 2.571926
DYR_PNECA Fu	446753 1k	DYR_HUMAN Ful	158376 Dp	1HFP	1DAJ	1KMV	37.44		Sc=6.31284, min distance = 2.35678
DYR_PNECA Fu	447229 AI	DYR_HUMAN Ful	126941 me	1U72	3CD2	1MVT	37.44		Sc=6.3189, min distance = 2.427932
DYR_PNECA Fu	447229 AI	DYR_HUMAN Ful	158376 Dp	1HFP	1DAJ	1MVT	37.44		Sc=6.34884, min distance = 2.14156
DYR_PNECA Fu	447238 CI	DYR_ECOLI Ful	6037 foli	1RA2	2CD2	1RF7	30.64	0.96	Sc=6.09668, min distance = 2.49592
DYR_PNECA Fu	447815 AI	DYR_HUMAN Ful	158376 Dp	1HFP	1DAJ	1PD8	37.44		Sc=6.37599, min distance = 2.21909
DYR_PNECA Fu	448303 CI	DYR_ECOLI Ful	126941 me	4DFR	3CD2	1RA3	30.64		Sc=6.54791, min distance = 2.17069
DYR_PNECA Fu	448303 CI	DYR_ECOLI Ful	5886 NADF	1RA2	2FZI	1RA3	30.64		Sc=6.56669, min distance = 2.30657
DYR_PNECA Fu	448305 CI	DYR_ECOLI Ful	126941 me	4DFR	3CD2	1RB3	30.64		Sc=6.59531, min distance = 2.03180

DYR_PNECA Fu	448305 CI	DYR_ECOLI Ful	5886 NADF	1RA2	2FZI	1RB3	30.64		Sc=6.62734, min distance = 2.08303
DYR_PNECA Fu	448307 CI	DYR_ECOLI Ful	6037 foli	1RA2	2CD2	1RC4	30.64		Sc=5.86508, min distance = 2.28327
DYR_PNECA Fu	448308 CI	DYR_ECOLI Ful	5886 NADF	1RA2	2FZI	1RC4	30.64		Sc=6.73683, min distance = 2.42257
DYR_PNECA Fu	448493 TQ	DYR_HUMAN Ful	126941 me	1U72	3CD2	1S3V	37.44		Sc=6.27762, min distance = 2.38311
DYR_PNECA Fu	448493 TQ	DYR_HUMAN Ful	158376 Dp	1HFP	1DAJ	1S3V	37.44		Sc=6.31406, min distance = 2.13735
DYR_PNECA Fu	449577 CI	DYR_ECOLI Ful	126941 me	4DFR	3CD2	6DFR	30.64		Sc=6.59238, min distance = 2.11965
DYR_PNECA Fu	449577 CI	DYR_ECOLI Ful	5886 NADF	1RA2	2FZI	6DFR	30.64		Sc=6.5969, min distance = 2.447733
DYR_PNECA Fu	54369 Pir	DYR_HUMAN Ful	126941 me	1U72	3CD2	1U71	37.44		Sc=6.31987, min distance = 2.12426
DYR_PNECA Fu	54369 Pir	DYR_HUMAN Ful	158376 Dp	1HFP	1DAJ	1U71	37.44		Sc=6.35205, min distance = 2.01969
DYR_PNECA Fu	6540275 i	DYR_MYCTU Ful	5886 NADF	1DF7	2FZI	2CIG	32.28	0.93	Sc=6.42862, min distance = 2.19840
DYR_PNECA Fu	72440 D-A	DYR_ECOLI Ful	6037 foli	1RA2	2CD2	1DDS	30.64	0.88	Sc=5.89884, min distance = 2.46680
DYR_THEMA Fu	11987843	DYR_PNECA Ful	126941 me	3CD2	1D1G	2FZI			Sc=6.44206, min distance = 2.34480
DYR_THEMA Fu	11987844	DYR_MOUSE Ful	126941 me	1U70	1D1G	2FZJ			Sc=6.44442, min distance = 1.95143
DYR_THEMA Fu	130731 PT	DYR_HUMAN Ful	5886 NADF	1KMS	1D1G	1OHJ			Sc=6.2281, min distance = 2.145674
DYR_THEMA Fu	148138 Lo	DYR_ECOLI Ful	5886 NADF	1RA2	1D1G	1DYJ			Sc=6.55161, min distance = 2.18753
DYR_THEMA Fu	15942680	6PGD_LACLM Fu	5886 NADF	2IZ0	1D1G	2IZ1			Sc=5.70705, min distance = 2.36890
DYR_THEMA Fu	2429 BROM	DYR_MYCTU Ful	126941 me	1DF7	1D1G	1DG7			Sc=6.18225, min distance = 2.53584
DYR_THEMA Fu	4369003 C	DYR_ECOLI Ful	126941 me	4DFR	1D1G	1TDR			Sc=5.69755, min distance = 2.39273
DYR_THEMA Fu	4369003 C	DYR_ECOLI Ful	5886 NADF	1RA2	1D1G	1TDR			Sc=5.68504, min distance = 2.55193
DYR_THEMA Fu	4369030 C	DYR_ECOLI Ful	126941 me	4DFR	1D1G	1RX5			Sc=6.33251, min distance = 2.57075
DYR_THEMA Fu	4369030 C	DYR_ECOLI Ful	5886 NADF	1RA2	1D1G	1RX5			Sc=6.29016, min distance = 2.14330
DYR_THEMA Fu	440141 9i	G6PD_LEUME Fu	5886 NADF	1H9A	1D1G	1E7Y			Sc=6.38772, min distance = 2.19168
DYR_THEMA Fu	444617 AI	DYR_HUMAN Ful	126941 me	1U72	1D1G	1BOZ			Sc=6.33398, min distance = 2.40674
DYR_THEMA Fu	445175 DZ	DYR_ECOLI Ful	5886 NADF	1RA2	1D1G	1DYH			Sc=6.60928, min distance = 2.13728
DYR_THEMA Fu	445175 DZ	DYR_HUMAN Ful	126941 me	1U72	1D1G	2DHF		0.85	Sc=6.61246, min distance = 2.17838
DYR_THEMA Fu	445175 DZ	DYR_HUMAN Ful	5886 NADF	1KMS	1D1G	2DHF			Sc=6.63522, min distance = 1.86099
DYR_THEMA Fu	446753 1k	DYR_HUMAN Ful	126941 me	1U72	1D1G	1KMV			Sc=6.32331, min distance = 2.00689
DYR_THEMA Fu	447021 AI	DYR_PNECA Ful	126941 me	3CD2	1D1G	1LY3			Sc=6.25142, min distance = 2.51711
DYR_THEMA Fu	447229 AI	DYR_HUMAN Ful	126941 me	1U72	1D1G	1MVT			Sc=6.32241, min distance = 2.42354
DYR_THEMA Fu	447238 CI	DYR_ECOLI Ful	126941 me	4DFR	1D1G	1RF7		0.86	Sc=6.55761, min distance = 2.27336
DYR_THEMA Fu	447238 CI	DYR_ECOLI Ful	5886 NADF	1RA2	1D1G	1RF7			Sc=6.5547, min distance = 1.902019
DYR_THEMA Fu	448307 CI	DYR_ECOLI Ful	126941 me	4DFR	1D1G	1RC4			Sc=6.23541, min distance = 2.55577
DYR_THEMA Fu	448307 CI	DYR_ECOLI Ful	5886 NADF	1RA2	1D1G	1RC4			Sc=6.19516, min distance = 2.20069
DYR_THEMA Fu	448308 CI	DYR_ECOLI Ful	126941 me	4DFR	1D1G	1RC4			Sc=5.73164, min distance = 1.28578
DYR_THEMA Fu	448308 CI	DYR_ECOLI Ful	5886 NADF	1RA2	1D1G	1RC4			Sc=5.83289, min distance = 1.95990
DYR_THEMA Fu	448492 TQ	DYR_HUMAN Ful	126941 me	1U72	1D1G	1S3U			Sc=6.28314, min distance = 2.34979
DYR_THEMA Fu	448493 TQ	DYR_HUMAN Ful	126941 me	1U72	1D1G	1S3V			Sc=6.27663, min distance = 2.46192

DYR_THEMA Fu	448494 CI	DYR_PNECA Ful	126941 me	3CD2	1D1G	1S3Y			Sc=6.23402, min distance = 2.67372
DYR_THEMA Fu	490573 Di	DYR_PNECA Ful	126941 me	3CD2	1D1G	2FZH			Sc=6.4061, min distance = 2.465544
DYR_THEMA Fu	54369 Pir	DYR_HUMAN Ful	126941 me	1U72	1D1G	1U71			Sc=6.31987, min distance = 2.121416
DYR_THEMA Fu	5578 trim	DYR_MYCTU Ful	126941 me	1DF7	1D1G	1DG5			Sc=6.16195, min distance = 2.501588
DYR_THEMA Fu	5578 trim	DYR_PNECA Ful	126941 me	3CD2	1D1G	1DYR			Sc=6.14361, min distance = 2.143510
DYR_THEMA Fu	6037 foli	DYR_HUMAN Ful	5886 NADF	1KMS	1D1G	1DRF			Sc=6.64921, min distance = 1.92466
DYR_THEMA Fu	72440 D-A	DYR_ECOLI Ful	5886 NADF	1RA2	1D1G	1DDS			Sc=6.28807, min distance = 2.68502
E2AK2_HUMAN I	1400 nche	HCK_HUMAN Ful	33113 gam	1AD5	2A19	1QCF			Sc=6.0941, min distance = 1.936758
E2AK2_HUMAN I	15942671	FAK1_CHICK Fu	33113 gam	2J0L	2A19	2J0J			Sc=6.33135, min distance = 2.03637
E2AK2_HUMAN I	176870 Er	EGFR_HUMAN Fu	33113 gam	2ITN	2A19	1M17			Sc=6.18833, min distance = 1.96770
E2AK2_HUMAN I	3547 Fasu	ROCK1_HUMAN Fu	33113 gam	2V55	2A19	2ESM			Sc=5.90321, min distance = 1.97935
E2AK2_HUMAN I	3973 nche	PIM1_HUMAN Fu	33113 gam	1XR1	2A19	1YI3			Sc=5.87993, min distance = 2.06656
E2AK2_HUMAN I	446721 CI	ASSY_THET8 Fu	33113 gam	1KOR	2A19	1KH2		0.93	Sc=5.9208, min distance = 2.160195
E2AK2_HUMAN I	448043 2g	ROCK1_HUMAN Fu	33113 gam	2V55	2A19	3D9V			Sc=5.97219, min distance = 1.61280
E2AK2_HUMAN I	5326739 i	GSK3B_HUMAN Fu	33113 gam	1J1B	2A19	1Q41			Sc=6.18799, min distance = 2.11803
E2AK2_HUMAN I	5327121 I	PIM1_HUMAN Fu	33113 gam	1XR1	2A19	2C3I			Sc=6.13725, min distance = 2.52316
E2AK2_HUMAN I	611002 Op	PIM1_HUMAN Fu	33113 gam	1XR1	2A19	1YXX			Sc=6.04966, min distance = 1.70336
EBA3_FLAME Fu	445184 1,	NRAM_I56A2 Fu	439680 be	1V0Z	1EOM	1W1X		0.89	Sc=5.63826, min distance = 2.51818
EBA3_FLAME Fu	64947 alp	LEC1_LATOC Fu	439680 be	1LOG	1EOM	1LOA		0.94	Sc=5.64425, min distance = 2.53769
ECOT_ECOLI Fu	444279 1s	RCEM_RHOSH Fu	445318 n-	4RCR	1ECZ	1RZH			Sc=5.64806, min distance = 1.46334
ECOT_ECOLI Fu	447607 1d	PYGM_RABIT Fu	79025 alp	1H5U	1ECY	2G9V			Sc=5.60731, min distance = 2.20017
ECOT_ECOLI Fu	64960 Pol	XYLA_STRRU Fu	79025 alp	1XIF	1ECY	1XIE		0.89	Sc=5.60731, min distance = 2.23608
ECX1_PYRAB Fu	445940 O6	CDK2_HUMAN Fu	6022 Aden	1GY3	2PO0	1GZ8			Sc=6.09058, min distance = 1.91747
ECX1_PYRAB Fu	446090 1h	NDKC_DICDI Fu	6022 Aden	1KDN	2PO0	1HIY		0.97	Sc=6.29618, min distance = 2.04620
ECX1_PYRAB Fu	4565 1h1r	CDK2_HUMAN Fu	6022 Aden	1GY3	2PO0	1H1R			Sc=5.97183, min distance = 1.49204
ECX1_PYRAB Fu	6083 ader	PURP_METJA Fu	6022 Aden	2R7N	2PO0	2R7M		0.99	Sc=5.74313, min distance = 2.46522
ECX1_PYRAB Fu	60961 ade	IPKA_RABIT Fu	6022 Aden	1JBP	2PO0	1FMO		0.95	Sc=6.1235, min distance = 2.488195
ECX1_PYRAB Fu	9994066 4	CDK2_HUMAN Fu	6022 Aden	1GY3	2PO0	2VTJ			Sc=5.85135, min distance = 2.03375
ECX2_SULSO Fu	24180721	PIM1_HUMAN Fu	6083 aden	1YXU	2C38	3C4E			Sc=5.86621, min distance = 2.39381
ECX2_SULSO Fu	449193 Ro	PDE4B_HUMAN Fu	6083 aden	1ROR	2C38	1XMU			Sc=5.88601, min distance = 2.23877
ECX2_SULSO Fu	656966 1y	PDE4D_HUMAN Fu	6083 aden	1TB7	2C38	1Y2E			Sc=5.6944, min distance = 2.547363
EF1A_YEAST Fu	445463 B-	TGM3_HUMAN Fu	6804 guan	1SGX	1G7C	1L9N			Sc=6.17322, min distance = 2.41328
EF1A_YEAST Fu	6022 Aden	NDK_PYRHO Ful	6804 guan	2DXF	1G7C	2DYA		0.8	Sc=5.74013, min distance = 2.26427
EF2_YEAST Fu	6804 guan	EF1A_YEAST Fu	8977 1dar	2B7B	1U2R	1G7C		0.99	Sc=5.90674, min distance = 2.54633
EFTU1_THET8 I	37792 gam	RAB6B_HUMAN Fu	8977 1dar	2E9S	1HA3	2FFQ		0.99	Sc=5.79076, min distance = 2.44896
EFTU1_THET8 I	37792 gam	RB11A_HUMAN Fu	8977 1dar	1OIX	1HA3	1OIW		0.99	Sc=6.07603, min distance = 2.46121
EFTU1_THET8 I	445871 CI	RASH_HUMAN Fu	36735 Gpp	1CTQ	2C78	1GNP			Sc=5.6807, min distance = 2.393470

EFTU1_THET8 I	446248 CI	RASH_HUMAN Ful	8977 1dar	2CE2	1HA3	1IAQ			Sc=5.7798, min distance = 2.086408
EFTU1_THET8 I	6804 guar	EF1A_YEAST Ful	8977 1dar	2B7B	1HA3	1G7C	33.86	0.99	Sc=5.97526, min distance = 2.522716
EFTU1_THET8 I	93082 Gab	EFG_THETH Full	36735 Gpp	2BV3	2C78	2J7K	33.65	0.99	Sc=5.81882, min distance = 2.283371
EFTU1_THETH I	445871 CI	RASH_HUMAN Ful	36735 Gpp	1CTQ	1EXM	1GNP			Sc=5.78582, min distance = 2.397046
EFTU2_THET8 I	445871 CI	RASH_HUMAN Ful	36735 Gpp	1CTQ	2C77	1GNP			Sc=5.68541, min distance = 2.413738
EFTU_BOVIN Fu	444825 1d	PURA_ECOLI Ful	8977 1dar	1QF5	1D2E	1CH8		0.95	Sc=5.83165, min distance = 2.236782
EFTU_BOVIN Fu	445871 CI	RASH_HUMAN Ful	8977 1dar	2CE2	1D2E	1GNP			Sc=5.67794, min distance = 2.133487
EFTU_BOVIN Fu	6804 guar	EF1A_YEAST Ful	8977 1dar	2B7B	1D2E	1G7C		0.99	Sc=5.97101, min distance = 2.533823
EFTU_ECOLI Fu	37792 gam	RB11A_HUMAN Fu	8977 1dar	1OIX	1EFC	1OIW		0.99	Sc=6.15299, min distance = 2.475037
EFTU_THEAQ Fu	37792 gam	ARF6_HUMAN Ful	8977 1dar	1E0S	1TUI	2J5X		0.99	Sc=6.06199, min distance = 2.138791
EFTU_THEAQ Fu	37792 gam	RAB6B_HUMAN Fu	8977 1dar	2E9S	1TUI	2FFQ		0.99	Sc=6.05887, min distance = 2.133186
EFTU_THEAQ Fu	37792 gam	RB11A_HUMAN Fu	8977 1dar	1OIX	1TUI	1OIW		0.99	Sc=6.06686, min distance = 2.409256
EFTU_THEAQ Fu	444825 1d	PURA_ECOLI Ful	8977 1dar	1QF5	1TUI	1CH8		0.95	Sc=5.70687, min distance = 2.427535
EFTU_THEAQ Fu	445871 CI	RASH_HUMAN Ful	8977 1dar	2CE2	1TUI	1GNP			Sc=5.83833, min distance = 2.161801
EFTU_THEAQ Fu	446248 CI	RAN_HUMAN Full	8977 1dar	3CH5	1TUI	1IBR			Sc=5.64453, min distance = 2.037455
EFTU_THEAQ Fu	446248 CI	RASH_HUMAN Ful	8977 1dar	2CE2	1TUI	1IAQ			Sc=5.77545, min distance = 2.223324
EFTU_THEAQ Fu	6804 guar	EF1A_YEAST Ful	8977 1dar	2B7B	1TUI	1G7C	33.86	0.99	Sc=5.96717, min distance = 2.503981
EFTU_THEAQ Fu	93082 Gab	EFG_THETH Full	8977 1dar	2BM0	1TUI	2J7K	31.5	0.99	Sc=5.80149, min distance = 2.426508
EFTU_THEAQ Fu	93082 Gab	RAC3_HUMAN Ful	8977 1dar	2C2H	1TUI	2QME		0.99	Sc=5.81193, min distance = 2.342334
EGFR_HUMAN Fu	1400 nche	HCK_HUMAN Full	33113 gam	1AD5	2ITN	1QCF	38.15		Sc=6.14736, min distance = 2.049559
EGFR_HUMAN Fu	16040273	IRAK4_HUMAN Fu	33113 gam	2OID	2ITN	2OIC	35.56		Sc=6.51147, min distance = 0.908019
EGFR_HUMAN Fu	2396 bisi	PIM1_HUMAN Ful	33113 gam	1XR1	2ITN	1XWS	25		Sc=6.33178, min distance = 2.047601
EGFR_HUMAN Fu	3547 Fasu	ROCK1_HUMAN Fu	33113 gam	2V55	2ITN	2ESM	21.5		Sc=5.91243, min distance = 2.381231
EGFR_HUMAN Fu	444345 1d	IRAK4_HUMAN Fu	33113 gam	2OID	2ITN	2NRY	35.56		Sc=6.31814, min distance = 1.903280
EGFR_HUMAN Fu	5281701 T	PIM1_HUMAN Ful	33113 gam	1XR1	2ITN	2O65	25		Sc=6.27629, min distance = 2.176950
EGFR_HUMAN Fu	5287844 i	GSK3B_HUMAN Fu	33113 gam	1J1B	2ITN	1UV5	29.56		Sc=6.1865, min distance = 2.071063
EGFR_HUMAN Fu	611002 Op	PIM1_HUMAN Ful	33113 gam	1XR1	2ITN	1YXX	25		Sc=6.00869, min distance = 2.078165
EHD2_MOUSE Fu	444852 CI	ATPB_BOVIN Ful	33113 gam	2JDI	2QPT	1COW		0.91	Sc=6.08078, min distance = 2.309790
EHD2_MOUSE Fu	6083 ader	HSP71_HUMAN Fu	33113 gam	2E8A	2QPT	1XQS		0.97	Sc=5.9266, min distance = 2.185174
EHMT1_HUMAN I	65482 sir	SETD7_HUMAN Fu	439155 Ad	2F69	2RFI	3CBP		0.88	Sc=5.77962, min distance = 2.680202
EHMT2_HUMAN I	21945395	SETD7_HUMAN Fu	439155 Ad	2F69	2O8J	1XQH		0.88	Sc=6.02944, min distance = 2.632567
EHMT2_HUMAN I	65482 sir	SETD7_HUMAN Fu	439155 Ad	2F69	2O8J	3CBP		0.88	Sc=5.78364, min distance = 2.652345
ELBH_ECOLX Fu	1983 acet	TRFL_BOVIN Ful	439353 be	2DWJ	2O2L	2OCU			Sc=5.65234, min distance = 2.248880
ELBH_ECOLX Fu	657081 CI	LEG3_HUMAN Ful	439353 be	1KJL	2O2L	1KJR			Sc=5.64926, min distance = 1.340920
ELBH_ECOLX Fu	657081 CI	LEG3_HUMAN Ful	64689 bet	2NMO	2O2L	1KJR			Sc=5.64347, min distance = 1.432665
ELBP_ECOLX Fu	24139 ace	LYSC_HUMAN Ful	439353 be	1REZ	1LT3	1LZR			Sc=5.99738, min distance = 1.618830
ELBP_ECOLX Fu	440552 1b	LECA_ARTIN Ful	439353 be	1UGX	1LT3	1UH1			Sc=5.93422, min distance = 2.507943

ELBP_ECOLX F	444635 O3	MBL1_RAT Full	439353 be	2KMB	1LT3	3KMB		0.9	Sc=5.79943, min distance = 1.30800
ELBP_ECOLX F	449462 2-	LEG7_HUMAN Fu	439353 be	4GAL	1LT3	3GAL			Sc=5.85029, min distance = 2.38400
ELIB_PHYCR F	5326970 E	KES1_YEAST Fu	5997 chol	1ZHY	1LRI	1ZHZ		0.97	Sc=5.91272, min distance = 2.31699
ELNE_HUMAN F	446578 1r	MBL1_RAT Full	439554 fu	3KMB	2Z7F	1KWW		0.96	Sc=5.63603, min distance = 2.50581
ELNE_HUMAN F	94214 Met	MBL1_RAT Full	439554 fu	3KMB	2Z7F	1AFA		0.96	Sc=5.71639, min distance = 2.41247
ENGA_THEMA F	6804 guar	EF1A_YEAST Fu	8977 1dar	2B7B	1MKY	1G7C		0.99	Sc=6.04688, min distance = 2.55491
ENGB_BACSU F	60961 ade	NUD16_XENLA F	6830 guan	2A8S	1SVW	2A8T			Sc=5.8357, min distance = 2.521698
ENGB_BACSU F	93082 Gab	RASH_HUMAN Fu	6830 guan	2CL7	1SVW	121P		0.99	Sc=6.55422, min distance = 1.88563
ENPL_CANFA F	10403821	HS90A_HUMAN F	6022 Aden	1BYQ	1TC6	3BM9	53.62		Sc=6.12731, min distance = 2.71324
ENPL_CANFA F	11373270	HSP82_YEAST F	6323491 M	1BGQ	1QY8	2FXS	52.88		Sc=6.48002, min distance = 2.09098
ENPL_CANFA F	11963551	HSP82_YEAST F	6022 Aden	1AMW	1TC6	2IWS	52.88		Sc=6.19577, min distance = 1.65589
ENPL_CANFA F	11963551	HSP82_YEAST F	6323491 M	1BGQ	1QY8	2IWS	52.88	0.96	Sc=6.14422, min distance = 2.67440
ENPL_CANFA F	11963552	HSP82_YEAST F	6022 Aden	1AMW	1TC6	2IWU	52.88		Sc=6.19822, min distance = 2.50981
ENPL_CANFA F	11963552	HSP82_YEAST F	6323491 M	1BGQ	1QY8	2IWU	52.88	0.96	Sc=6.15068, min distance = 2.54423
ENPL_CANFA F	11987828	HSP82_YEAST F	6022 Aden	1AMW	1TC6	2CGF	52.88		Sc=6.2375, min distance = 1.895855
ENPL_CANFA F	11987828	HSP82_YEAST F	6323491 M	1BGQ	1QY8	2CGF	52.88	0.96	Sc=6.20045, min distance = 2.46751
ENPL_CANFA F	13373715	HS90A_HUMAN F	6022 Aden	1BYQ	1TC6	2QF6	53.62		Sc=6.12077, min distance = 1.35903
ENPL_CANFA F	16741208	HSP82_YEAST F	6323491 M	1BGQ	1QY8	2IWX	52.88	0.96	Sc=6.23002, min distance = 2.52495
ENPL_CANFA F	188966 dA	HSLU_ECOLI Fu	33113 gam	1E94	2O1U	1G4A		0.97	Sc=5.84091, min distance = 2.28111
ENPL_CANFA F	24836816	HS90A_HUMAN F	6022 Aden	1BYQ	1TC6	3BMY	53.62		Sc=6.26534, min distance = 2.48216
ENPL_CANFA F	398148 1e	CDK2_HUMAN Fu	6022 Aden	1GY3	1TC6	1E1X			Sc=6.14888, min distance = 1.78823
ENPL_CANFA F	445940 O6	CDK2_HUMAN Fu	6022 Aden	1GY3	1TC6	1GZ8			Sc=6.04323, min distance = 2.09384
ENPL_CANFA F	445966 O6	CDK2_HUMAN Fu	6022 Aden	1GY3	1TC6	1H0V			Sc=6.13394, min distance = 2.48080
ENPL_CANFA F	447955 1p	CDK2_HUMAN Fu	6022 Aden	1GY3	1TC6	1PXI			Sc=5.63155, min distance = 2.67424
ENPL_CANFA F	5326935 1	HS90A_HUMAN F	6022 Aden	1BYQ	1TC6	1YC4	53.62		Sc=6.30222, min distance = 2.56086
ENPL_CANFA F	5326978 1	CSK2A_MAIZE F	33113 gam	1LP4	2O1U	1ZOH			Sc=5.60518, min distance = 1.79833
ENPL_CANFA F	5327104 2	HS90A_HUMAN F	6022 Aden	1BYQ	1TC6	2BYI	53.62		Sc=6.38555, min distance = 2.62725
ENPL_CANFA F	6082103 2	HS90A_HUMAN F	6022 Aden	1BYQ	1TC6	2BT0	53.62		Sc=6.28835, min distance = 2.14712
ENPL_CANFA F	6082103 2	HSP82_YEAST F	6022 Aden	1AMW	1TC6	2BRC	52.88		Sc=6.30818, min distance = 2.38826
ENPL_CANFA F	6102787 2	HS90A_HUMAN F	6022 Aden	1BYQ	1TC6	2CCS	53.62		Sc=6.18277, min distance = 2.66315
ENPL_CANFA F	6102788 2	HS90A_HUMAN F	6022 Aden	1BYQ	1TC6	2CCT	53.62		Sc=6.39323, min distance = 2.50251
ENPL_CANFA F	6804 guar	NDK_PYRHO Ful	6022 Aden	2DYA	1TC6	2DXF		0.8	Sc=5.85094, min distance = 2.07357
ENTP2_RAT Fu	16750062	CSK2A_MAIZE F	33113 gam	1LP4	3CJA	2OXD			Sc=5.65416, min distance = 2.20689
ENTP2_RAT Fu	24905142	SRC_CHICK Ful	6083 aden	3DQX	3CJ7	3EN4			Sc=6.39601, min distance = 1.63695
ENTP2_RAT Fu	448042 2e	ROCK1_HUMAN F	33113 gam	2V55	3CJA	2ETR			Sc=6.15325, min distance = 1.96445
ENTP2_RAT Fu	448043 2g	ROCK1_HUMAN F	33113 gam	2V55	3CJA	3D9V			Sc=6.00452, min distance = 2.02015
ENTP2_RAT Fu	5326976 1	CSK2A_MAIZE F	33113 gam	1LP4	3CJA	1ZOE			Sc=5.6971, min distance = 2.396365

ENTP2_RAT Fu	5327121 I	PIM1_HUMAN Fu	33113 gam	1XR1	3CJA	2C3I		Sc=6.24359, min distance = 2.28961
ENTP2_RAT Fu	5327121 I	PIM1_HUMAN Fu	6083 aden	1YXU	3CJ7	2C3I		Sc=6.27123, min distance = 2.34805
ENTP2_RAT Fu	656964 1y	PDE4D_HUMAN F	6083 aden	1TB7	3CJ7	1Y2C		Sc=5.91374, min distance = 2.52431
ENTP2_RAT Fu	656966 1y	PDE4D_HUMAN F	6083 aden	1TB7	3CJ7	1Y2E		Sc=6.05659, min distance = 2.23365
ENTP2_RAT Fu	656969 1y	PDE4D_HUMAN F	6083 aden	1TB7	3CJ7	1Y2K		Sc=6.1891, min distance = 2.021088
ENTP2_RAT Fu	657135 Op	PIM1_HUMAN Fu	6083 aden	1YXU	3CJ7	1YXV		Sc=5.89949, min distance = 1.57671
ENTP2_RAT Fu	6804 guar	PDE10_HUMAN F	6083 aden	2OUN	3CJ7	2OUQ	0.8	Sc=6.40518, min distance = 0
ENV_SIVMK Fu	444211 th	ACES_TORCA Fu	185698 al	1U65	2BF1	1ACJ		Sc=5.60509, min distance = 2.62442
EPHA2_HUMAN	1400 nche	HCK_HUMAN Ful	33113 gam	1AD5	1MQB	1QCF		Sc=6.25175, min distance = 1.68663
EPHA2_HUMAN	15942671	FAK1_CHICK Fu	33113 gam	2J0L	1MQB	2J0J		Sc=6.32987, min distance = 2.36521
EPHA2_HUMAN	16058647	PIM1_HUMAN Fu	33113 gam	1XR1	1MQB	2OI4		Sc=6.49593, min distance = 1.96033
EPHA2_HUMAN	16750062	CSK2A_MAIZE F	33113 gam	1LP4	1MQB	2OXD		Sc=5.64728, min distance = 2.45193
EPHA2_HUMAN	23656870	CSK2A_MAIZE F	33113 gam	1LP4	1MQB	2PVN		Sc=6.53437, min distance = 0.65653
EPHA2_HUMAN	24756824	CSK2A_MAIZE F	33113 gam	1LP4	1MQB	3BE9		Sc=6.40313, min distance = 2.39434
EPHA2_HUMAN	24779675	CSK2A_MAIZE F	33113 gam	1LP4	1MQB	2PVJ		Sc=6.46191, min distance = 0.50407
EPHA2_HUMAN	24779676	CSK2A_MAIZE F	33113 gam	1LP4	1MQB	2PVK		Sc=6.3198, min distance = 2.442753
EPHA2_HUMAN	24779677	CSK2A_MAIZE F	33113 gam	1LP4	1MQB	2PVL		Sc=6.39947, min distance = 0.53316
EPHA2_HUMAN	24779678	CSK2A_MAIZE F	33113 gam	1LP4	1MQB	2PVM		Sc=6.55984, min distance = 1.04700
EPHA2_HUMAN	3064778 H	ROCK1_HUMAN F	33113 gam	2V55	1MQB	2ETK		Sc=6.04704, min distance = 2.14777
EPHA2_HUMAN	3547 Fasu	ROCK1_HUMAN F	33113 gam	2V55	1MQB	2ESM		Sc=5.92897, min distance = 2.27490
EPHA2_HUMAN	36735 Gpp	CSK2A_MAIZE F	33113 gam	1LP4	1MQB	1DAY	0.8	Sc=5.67479, min distance = 2.34252
EPHA2_HUMAN	3973 nche	PIM1_HUMAN Fu	33113 gam	1XR1	1MQB	1YI3		Sc=5.88487, min distance = 2.13881
EPHA2_HUMAN	444345 1c	PIM1_HUMAN Fu	33113 gam	1XR1	1MQB	1YHS		Sc=6.36357, min distance = 2.01829
EPHA2_HUMAN	444367 CI	CSK2A_MAIZE F	33113 gam	1LP4	1MQB	1OM1		Sc=6.14179, min distance = 1.90309
EPHA2_HUMAN	445479 CI	CSK21_HUMAN F	33113 gam	2PVR	1MQB	3BQC		Sc=6.23323, min distance = 2.04134
EPHA2_HUMAN	446721 CI	ASSY_THET8 Fu	33113 gam	1KOR	1MQB	1KH2	0.93	Sc=5.97716, min distance = 1.61311
EPHA2_HUMAN	448008 CI	GSK3B_HUMAN F	33113 gam	1J1B	1MQB	1Q4L		Sc=6.34382, min distance = 2.59631
EPHA2_HUMAN	448014 1c	GSK3B_HUMAN F	33113 gam	1J1B	1MQB	1Q5K		Sc=6.29268, min distance = 2.26598
EPHA2_HUMAN	448043 2c	ROCK1_HUMAN F	33113 gam	2V55	1MQB	3D9V		Sc=5.99103, min distance = 2.15330
EPHA2_HUMAN	5281680 Q	PIM1_HUMAN Fu	33113 gam	1XR1	1MQB	2O64		Sc=6.43109, min distance = 2.03351
EPHA2_HUMAN	5287844 i	GSK3B_HUMAN F	33113 gam	1J1B	1MQB	1UV5		Sc=6.33027, min distance = 1.99683
EPHA2_HUMAN	5326976 1	CSK2A_MAIZE F	33113 gam	1LP4	1MQB	1ZOE		Sc=5.69895, min distance = 2.49960
EPHA2_HUMAN	5326977 1	CSK2A_MAIZE F	33113 gam	1LP4	1MQB	1ZOG		Sc=5.62063, min distance = 2.42657
EPHA2_HUMAN	5326978 1	CSK2A_MAIZE F	33113 gam	1LP4	1MQB	1ZOH		Sc=5.64781, min distance = 1.68706
EPHA2_HUMAN	5327148 C	IPKA_RABIT Fu	33113 gam	1CDK	1MQB	2ERZ		Sc=6.13286, min distance = 1.52692
EPHA2_HUMAN	60961 ade	SKY1_YEAST Fu	33113 gam	1Q99	1MQB	1Q97	0.93	Sc=6.19342, min distance = 2.28434
EPHA2_HUMAN	91532 AMP	PURT_ECOLI Fu	33113 gam	1EYZ	1MQB	1KJI	0.99	Sc=5.9692, min distance = 2.095540

EPHA3_HUMAN I	11986115	PIM1_HUMAN Fu	33113 gam	1XR1	2QO9	2BZJ			Sc=6.03591, min distance = 2.09871
EPHA3_HUMAN I	15942671	FAK1_CHICK Fu	33113 gam	2J0L	2QO9	2J0J			Sc=6.2898, min distance = 2.134157
EPHA3_HUMAN I	16040273	IRAK4_HUMAN Fu	33113 gam	2OID	2QO9	2OIC			Sc=6.31814, min distance = 1.60223
EPHA3_HUMAN I	3547 Fasu	ROCK1_HUMAN Fu	33113 gam	2V55	2QO9	2ESM			Sc=5.89549, min distance = 2.10966
EPHA3_HUMAN I	3973 nche	PIM1_HUMAN Fu	33113 gam	1XR1	2QO9	1YI3			Sc=5.84143, min distance = 2.80867
EPHA3_HUMAN I	444345 1c	IRAK4_HUMAN Fu	33113 gam	2OID	2QO9	2NRY			Sc=6.31176, min distance = 1.80515
EPHA3_HUMAN I	444852 CI	ATPB_BOVIN Fu	33113 gam	2JDI	2QO9	1COW		0.91	Sc=5.60347, min distance = 1.93865
EPHA3_HUMAN I	445479 CI	CSK21_HUMAN Fu	33113 gam	2PVR	2QO9	3BQC			Sc=6.1503, min distance = 2.547927
EPHA3_HUMAN I	446721 CI	ASSY_THET8 Fu	33113 gam	1KOR	2QO9	1KH2		0.93	Sc=5.64295, min distance = 1.73868
EPHA3_HUMAN I	448005 CI	GSK3B_HUMAN Fu	33113 gam	1J1B	2QO9	1Q3W			Sc=6.18403, min distance = 2.53818
EPHA3_HUMAN I	448043 2q	ROCK1_HUMAN Fu	33113 gam	2V55	2QO9	3D9V			Sc=5.96403, min distance = 2.37968
EPHA3_HUMAN I	456214 pu	SRC_HUMAN Ful	33113 gam	2SRC	2QO9	1YOM			Sc=6.2118, min distance = 2.027726
EPHA3_HUMAN I	5281680 Q	PIM1_HUMAN Fu	33113 gam	1XR1	2QO9	2O64			Sc=6.37232, min distance = 2.33230
EPHA3_HUMAN I	5327148 C	IPKA_RABIT Fu	33113 gam	1CDK	2QO9	2ERZ			Sc=6.10086, min distance = 2.20293
EPHA3_HUMAN I	6083 aden	PIM1_HUMAN Fu	33113 gam	1XR1	2QO9	1YXU		0.97	Sc=6.18468, min distance = 2.10257
EPHA3_HUMAN I	611002 Op	PIM1_HUMAN Fu	33113 gam	1XR1	2QO9	1YXX			Sc=5.97089, min distance = 2.22940
EPHA3_HUMAN I	9547983 1	HCK_HUMAN Ful	33113 gam	1AD5	2QO9	2HK5			Sc=6.4295, min distance = 1.609258
EPHA3_HUMAN I	9934643 2	SRC_HUMAN Ful	33113 gam	2SRC	2QO9	2BDJ			Sc=6.52479, min distance = 2.44462
EPHB2_MOUSE I	159354 To	DCK_HUMAN Ful	6022 Aden	1P5Z	2HEN	2NO7			Sc=5.9181, min distance = 1.955582
EPHB2_MOUSE I	15942671	ABL1_HUMAN Fu	6022 Aden	2G2I	2HEN	2HZ4			Sc=6.32771, min distance = 2.14033
EPHB2_MOUSE I	16046126	CDK2_HUMAN Fu	6022 Aden	1GY3	2HEN	2DS1			Sc=6.3011, min distance = 2.106836
EPHB2_MOUSE I	16214828	CDK2_HUMAN Fu	6022 Aden	1GY3	2HEN	2UZO			Sc=5.96303, min distance = 2.73383
EPHB2_MOUSE I	1707 1fvt	CDK2_HUMAN Fu	6022 Aden	1GY3	2HEN	1FVT			Sc=6.10795, min distance = 2.29582
EPHB2_MOUSE I	23653515	CDK2_HUMAN Fu	6022 Aden	1GY3	2HEN	2R3F			Sc=6.0421, min distance = 2.201520
EPHB2_MOUSE I	23653516	CDK2_HUMAN Fu	6022 Aden	1GY3	2HEN	2R3G			Sc=6.21316, min distance = 2.32883
EPHB2_MOUSE I	23653518	CDK2_HUMAN Fu	6022 Aden	1GY3	2HEN	2R3J			Sc=6.20519, min distance = 1.83461
EPHB2_MOUSE I	23653519	CDK2_HUMAN Fu	6022 Aden	1GY3	2HEN	2R3K			Sc=6.22757, min distance = 1.96096
EPHB2_MOUSE I	23653520	CDK2_HUMAN Fu	6022 Aden	1GY3	2HEN	2R3L			Sc=6.21168, min distance = 2.32814
EPHB2_MOUSE I	23653521	CDK2_HUMAN Fu	6022 Aden	1GY3	2HEN	2R3M			Sc=6.40827, min distance = 2.08313
EPHB2_MOUSE I	23653522	CDK2_HUMAN Fu	6022 Aden	1GY3	2HEN	2R3N			Sc=6.2728, min distance = 2.055200
EPHB2_MOUSE I	23653524	CDK2_HUMAN Fu	6022 Aden	1GY3	2HEN	2R3P			Sc=6.23746, min distance = 2.38750
EPHB2_MOUSE I	23653526	CDK2_HUMAN Fu	6022 Aden	1GY3	2HEN	2R3R			Sc=6.22132, min distance = 2.01422
EPHB2_MOUSE I	23727982	CDK2_HUMAN Fu	6022 Aden	1GY3	2HEN	3BHU			Sc=6.18721, min distance = 2.17279
EPHB2_MOUSE I	24864077	CDK2_HUMAN Fu	6022 Aden	1GY3	2HEN	2VTN			Sc=6.1428, min distance = 2.081398
EPHB2_MOUSE I	24864080	CDK2_HUMAN Fu	6022 Aden	1GY3	2HEN	2VTR			Sc=5.85555, min distance = 2.04533
EPHB2_MOUSE I	24864081	CDK2_HUMAN Fu	6022 Aden	1GY3	2HEN	2VTS			Sc=6.22348, min distance = 1.94411
EPHB2_MOUSE I	24901723	CDK2_HUMAN Fu	6022 Aden	1GY3	2HEN	2W06			Sc=6.21571, min distance = 2.40992

EPHB2_MOUSE I	24963033	CDK2_HUMAN Fu	6022 Aden	1GY3	2HEN	2W05		Sc=6.26255, min distance = 2.311940
EPHB2_MOUSE I	2608 1jsv	CDK2_HUMAN Fu	6022 Aden	1GY3	2HEN	1JSV		Sc=5.86383, min distance = 2.774270
EPHB2_MOUSE I	4369136 C	CDK2_HUMAN Fu	6022 Aden	1GY3	2HEN	1DM2		Sc=6.16824, min distance = 1.652030
EPHB2_MOUSE I	444503 FU	NDKC_DICDI Fu	6022 Aden	1KDN	2HEN	1B99		Sc=6.15398, min distance = 1.977976
EPHB2_MOUSE I	444564 AD	MYS2_DICDI Fu	6022 Aden	1VOM	2HEN	1W9I	0.91	Sc=5.8988, min distance = 2.0478640
EPHB2_MOUSE I	445966 O6	CDK2_HUMAN Fu	6022 Aden	1GY3	2HEN	1H0V		Sc=6.08739, min distance = 1.633045
EPHB2_MOUSE I	446090 1h	NDKC_DICDI Fu	6022 Aden	1KDN	2HEN	1HIY	0.97	Sc=5.87114, min distance = 2.050952
EPHB2_MOUSE I	447654 CI	CDK2_HUMAN Fu	6022 Aden	1GY3	2HEN	1OIT		Sc=6.19127, min distance = 2.760608
EPHB2_MOUSE I	447766 1p	CDK2_HUMAN Fu	6022 Aden	1GY3	2HEN	1P2A		Sc=6.18092, min distance = 2.440086
EPHB2_MOUSE I	447821 CI	CDK2_HUMAN Fu	6022 Aden	1GY3	2HEN	1PF8		Sc=6.10197, min distance = 2.280562
EPHB2_MOUSE I	447916 ac	RIO1_ARCFU Fu	6022 Aden	1ZTH	2HEN	1ZTF	0.95	Sc=6.06591, min distance = 2.964099
EPHB2_MOUSE I	447962 1p	CDK2_HUMAN Fu	6022 Aden	1GY3	2HEN	2C5N		Sc=6.18092, min distance = 2.242229
EPHB2_MOUSE I	448293 CI	CDK2_HUMAN Fu	6022 Aden	1GY3	2HEN	1R78		Sc=6.40605, min distance = 2.096120
EPHB2_MOUSE I	449088 1v	CDK2_HUMAN Fu	6022 Aden	1GY3	2HEN	1VYZ		Sc=6.06692, min distance = 2.432431
EPHB2_MOUSE I	4564 1e1v	CDK2_HUMAN Fu	6022 Aden	1GY3	2HEN	1E1V		Sc=6.03866, min distance = 2.100501
EPHB2_MOUSE I	4566 1h1s	CDK2_HUMAN Fu	6022 Aden	1GY3	2HEN	1H1S		Sc=6.33126, min distance = 2.112308
EPHB2_MOUSE I	5287830 6	Q8RLY5_LACHE I	190 adeni	1S2D	1JPA	1S2I	0.93	Sc=5.61618, min distance = 1.800381
EPHB2_MOUSE I	5288641 i	CDK2_HUMAN Fu	6022 Aden	1GY3	2HEN	1E9H		Sc=6.21179, min distance = 2.340961
EPHB2_MOUSE I	5288711 1	CDK2_HUMAN Fu	6022 Aden	1GY3	2HEN	1KE8		Sc=6.22516, min distance = 1.657614
EPHB2_MOUSE I	5288712 1	CDK2_HUMAN Fu	6022 Aden	1GY3	2HEN	1KE9		Sc=6.24811, min distance = 2.303972
EPHB2_MOUSE I	5289411 1	CDK2_HUMAN Fu	6022 Aden	1GY3	2HEN	1OGU		Sc=6.03881, min distance = 2.085995
EPHB2_MOUSE I	5327096 2	CDK2_HUMAN Fu	6022 Aden	1GY3	2HEN	2BTR		Sc=6.09434, min distance = 2.290231
EPHB2_MOUSE I	5327097 2	CDK2_HUMAN Fu	6022 Aden	1GY3	2HEN	2BTS		Sc=5.83184, min distance = 1.917818
EPHB2_MOUSE I	5327124	CDK2_HUMAN Fu	6022 Aden	1GY3	2HEN	2C4G		Sc=6.18556, min distance = 2.322285
EPHB2_MOUSE I	5327130 C	CDK2_HUMAN Fu	6022 Aden	1GY3	2HEN	2C68		Sc=6.22266, min distance = 1.844500
EPHB2_MOUSE I	5327131 T	CDK2_HUMAN Fu	6022 Aden	1GY3	2HEN	2C69		Sc=6.27537, min distance = 2.019082
EPHB2_MOUSE I	5327132 C	CDK2_HUMAN Fu	6022 Aden	1GY3	2HEN	2C6I		Sc=6.08476, min distance = 2.281167
EPHB2_MOUSE I	5327133 C	CDK2_HUMAN Fu	6022 Aden	1GY3	2HEN	2C6K		Sc=6.18092, min distance = 2.246834
EPHB2_MOUSE I	5327134 C	CDK2_HUMAN Fu	6022 Aden	1GY3	2HEN	2C6L		Sc=6.16039, min distance = 2.407110
EPHB2_MOUSE I	5327148 C	IPKA_RABIT Fu	6022 Aden	1JBP	2HEN	2ERZ		Sc=6.0683, min distance = 2.6576617
EPHB2_MOUSE I	6338561 C	MYS2_DICDI Fu	6022 Aden	1VOM	2HEN	1D0X		Sc=6.08405, min distance = 2.110005
EPHB2_MOUSE I	6420138 2	CDK2_HUMAN Fu	6022 Aden	1GY3	2HEN	2UUE		Sc=6.33478, min distance = 1.697310
EPHB2_MOUSE I	6420139 C	CDK2_HUMAN Fu	6022 Aden	1GY3	2HEN	2C5V		Sc=6.18519, min distance = 1.842625
EPHB2_MOUSE I	6420140 2	CDK2_HUMAN Fu	6022 Aden	1GY3	2HEN	2C5Y		Sc=6.44354, min distance = 2.189784
EPHB2_MOUSE I	6539118 g	CDK2_HUMAN Fu	6022 Aden	1GY3	2HEN	1FVV		Sc=6.38577, min distance = 1.966851
EPHB2_MOUSE I	657072 RY	CDK2_HUMAN Fu	6022 Aden	1GY3	2HEN	2BHH		Sc=6.39568, min distance = 2.277631
EPHB2_MOUSE I	764 guani	PURR_ECOLI Fu	190 adeni	2PUB	1JPA	1WET		Sc=5.64043, min distance = 2.266214

EPHB2_MOUSE I	91532 AMP	MTNK_BACSU Fu	6022 Aden	2OLC	2HEN	2PUL		0.99	Sc=5.67244, min distance = 1.741822
EPHB2_MOUSE I	91532 AMP	PURT_ECOLI Fu	6022 Aden	1KJQ	2HEN	1KJI		0.99	Sc=5.67467, min distance = 2.158160
EPHB2_MOUSE I	9547890 I	CDK2_HUMAN Fu	6022 Aden	1GY3	2HEN	1W8C			Sc=6.11936, min distance = 2.459156
EPHB2_MOUSE I	9817550 V	CDK2_HUMAN Fu	6022 Aden	1GY3	2HEN	3BHV			Sc=6.33294, min distance = 2.102347
EPHB2_MOUSE I	9991833 S	CDK2_HUMAN Fu	6022 Aden	1GY3	2HEN	2R3H			Sc=6.0928, min distance = 2.2497771
EPHB2_MOUSE I	9994066 4	CDK2_HUMAN Fu	6022 Aden	1GY3	2HEN	2VTJ			Sc=5.70428, min distance = 2.541610
ERI1_HUMAN Fu	1400 nche	RET_HUMAN Full	6083 aden	2IVT	1W0H	2IVV			Sc=5.76812, min distance = 2.475420
ERI1_HUMAN Fu	5327121 I	PIM1_HUMAN Fu	6083 aden	1YXU	1W0H	2C3I			Sc=6.18402, min distance = 1.803539
ERI1_HUMAN Fu	611002 Op	PIM1_HUMAN Fu	6083 aden	1YXU	1W0H	1YXX			Sc=6.13286, min distance = 1.363946
ERI1_HUMAN Fu	6419789 C	HASP_HUMAN Fu	6083 aden	3DLZ	1W0H	3E7V			Sc=5.91303, min distance = 2.732887
ERI1_HUMAN Fu	656968 1y	PDE4B_HUMAN Fu	6083 aden	1ROR	1W0H	1Y2H			Sc=5.83686, min distance = 2.484468
ERI1_HUMAN Fu	657135 Op	PIM1_HUMAN Fu	6083 aden	1YXU	1W0H	1YXV			Sc=5.74641, min distance = 2.806240
ERM_BACSU Fu	16741210	MTTA_THEAQ Fu	34756 Acy	2ADM	1QAO	2JG3			Sc=6.49587, min distance = 1.642240
ERM_BACSU Fu	188380 Ac	MCES_ENCCU Fu	439155 Ad	1RI1	1QAN	1Z3C		0.91	Sc=6.55102, min distance = 2.110971
ERM_BACSU Fu	439182 5'	Q70GK9_STRCT I	34756 Acy	1RQP	1QAO	2CC2		0.86	Sc=6.23494, min distance = 1.805068
ERM_BACSU Fu	445762 5'	MTTA_THEAQ Fu	34756 Acy	2ADM	1QAO	2IH2		0.93	Sc=6.30607, min distance = 2.214602
ERM_BACSU Fu	445971 CI	COMT_RAT Full=	34756 Acy	2CL5	1QAO	1H1D		0.92	Sc=6.53614, min distance = 2.274928
ERM_BACSU Fu	446535 CI	HNMT_HUMAN Fu	439155 Ad	2AOT	1QAN	1JQE		0.95	Sc=6.49948, min distance = 2.559672
ERM_BACSU Fu	5327118 5	Q70GK9_STRCT I	34756 Acy	1RQP	1QAO	2C2W		0.81	Sc=5.66217, min distance = 2.591574
ERM_BACSU Fu	60961 ade	PIMT_PYRFU Fu	439155 Ad	1JG1	1QAN	1JG2		0.84	Sc=6.2204, min distance = 2.6732695
ERM_BACSU Fu	6852187 2	Q70GK9_STRCT I	34756 Acy	1RQP	1QAO	2CBX		0.84	Sc=6.1497, min distance = 1.8012878
ERR3_HUMAN Fu	10019418	ESR1_HUMAN Fu	448538 CI	2BJ4	1S9Q	2G5O	38.77		Sc=6.21421, min distance = 0.731885
ERR3_HUMAN Fu	10019418	ESR1_HUMAN Fu	449459 4-	3ERT	2GPU	2G5O	38.77		Sc=6.16237, min distance = 1.835914
ERR3_HUMAN Fu	10286159	ESR2_HUMAN Fu	448538 CI	2FSZ	1S9Q	2I0G	35.5	0.77	Sc=6.08705, min distance = 1.436201
ERR3_HUMAN Fu	104946 Li	ESR1_HUMAN Fu	448538 CI	2BJ4	1S9Q	2R6Y	38.77		Sc=6.35114, min distance = 1.872202
ERR3_HUMAN Fu	11149479	ESR1_HUMAN Fu	448538 CI	2BJ4	1S9Q	2QAB	38.77		Sc=6.15862, min distance = 0.805844
ERR3_HUMAN Fu	11149479	ESR1_HUMAN Fu	449459 4-	3ERT	2GPU	2QAB	38.77		Sc=6.05636, min distance = 2.490732
ERR3_HUMAN Fu	11197931	ESR2_HUMAN Fu	448538 CI	2FSZ	1S9Q	2Z4B	35.5		Sc=6.11481, min distance = 1.085899
ERR3_HUMAN Fu	11368987	ESR1_HUMAN Fu	448538 CI	2BJ4	1S9Q	2QGW	38.77		Sc=6.12266, min distance = 1.125527
ERR3_HUMAN Fu	11368987	ESR1_HUMAN Fu	449459 4-	3ERT	2GPU	2QGW	38.77		Sc=6.0102, min distance = 2.3804984
ERR3_HUMAN Fu	14086997	ESR1_HUMAN Fu	449459 4-	3ERT	2GPU	2QA6	38.77		Sc=6.19838, min distance = 2.596419
ERR3_HUMAN Fu	1493 2,4-	TTHY_HUMAN Fu	448537 di	1TT6	1S9P	2B15			Sc=5.78908, min distance = 2.065856
ERR3_HUMAN Fu	1530 PhIP	ESR1_HUMAN Fu	449459 4-	3ERT	2GPU	2QXM	38.77		Sc=5.87066, min distance = 2.323937
ERR3_HUMAN Fu	15897928	ESR1_HUMAN Fu	449459 4-	3ERT	2GPU	2QSE	38.77		Sc=6.02025, min distance = 2.724692
ERR3_HUMAN Fu	15942662	ESR1_HUMAN Fu	448537 di	3ERD	1S9P	2G44	38.77	0.8	Sc=5.94513, min distance = 1.051954
ERR3_HUMAN Fu	15942662	ESR1_HUMAN Fu	448538 CI	2BJ4	1S9Q	2G44	38.77	0.78	Sc=5.93895, min distance = 1.343915
ERR3_HUMAN Fu	16750074	ESR1_HUMAN Fu	448537 di	3ERD	1S9P	2POG	38.77		Sc=6.14474, min distance = 0.210226

ERR3_HUMAN F	16750074	ESR1_HUMAN Fu	448538 CI	2BJ4	1S9Q	2POG	38.77	0.76	Sc=6.10823, min distance = 0.500136
ERR3_HUMAN F	16750074	ESR1_HUMAN Fu	449459 4-	3ERT	2GPU	2POG	38.77	0.75	Sc=6.01519, min distance = 2.497501
ERR3_HUMAN F	16758210	ESR2_HUMAN Fu	448538 CI	2FSZ	1S9Q	2NV7	35.5		Sc=6.17894, min distance = 1.401814
ERR3_HUMAN F	177880 2'	TTHY_HUMAN Fu	448537 di	1TT6	1S9P	2G9K			Sc=5.76183, min distance = 2.190114
ERR3_HUMAN F	20284644	TTHY_HUMAN Fu	448537 di	1TT6	1S9P	2QGC			Sc=5.96196, min distance = 1.721911
ERR3_HUMAN F	216416 La	ESR1_HUMAN Fu	448537 di	3ERD	1S9P	2OUZ	38.77		Sc=6.10598, min distance = 2.190856
ERR3_HUMAN F	216416 La	ESR1_HUMAN Fu	448538 CI	2BJ4	1S9Q	2OUZ	38.77	0.88	Sc=6.04859, min distance = 2.677416
ERR3_HUMAN F	216416 La	ESR1_HUMAN Fu	449459 4-	3ERT	2GPU	2OUZ	38.77	0.86	Sc=6.09712, min distance = 2.008592
ERR3_HUMAN F	23722943	TTHY_HUMAN Fu	448537 di	1TT6	1S9P	2QGD			Sc=5.93976, min distance = 2.043412
ERR3_HUMAN F	23722944	TTHY_HUMAN Fu	448537 di	1TT6	1S9P	2QGE			Sc=5.8264, min distance = 2.3944571
ERR3_HUMAN F	23728517	ESR1_HUMAN Fu	448537 di	3ERD	1S9P	2QGT	38.77		Sc=6.13391, min distance = 0.742305
ERR3_HUMAN F	23728517	ESR1_HUMAN Fu	449459 4-	3ERT	2GPU	2QGT	38.77		Sc=6.07625, min distance = 2.364920
ERR3_HUMAN F	243556 NS	TTHY_HUMAN Fu	448537 di	1TT6	1S9P	3CN2		0.78	Sc=5.68075, min distance = 2.382079
ERR3_HUMAN F	24892830	ESR1_HUMAN Fu	448538 CI	2BJ4	1S9Q	3DT3	38.77		Sc=6.28472, min distance = 2.383537
ERR3_HUMAN F	4369551 1	ESR2_HUMAN Fu	448538 CI	2FSZ	1S9Q	1U3S	35.5		Sc=6.1912, min distance = 1.4527274
ERR3_HUMAN F	4369563 I	TTHY_HUMAN Fu	448537 di	1TT6	1S9P	1Y1D			Sc=6.09057, min distance = 1.894331
ERR3_HUMAN F	4369568 C	ESR1_HUMAN Fu	448537 di	3ERD	1S9P	1YIM	38.77		Sc=6.2743, min distance = 1.9941040
ERR3_HUMAN F	4369569 C	ESR1_HUMAN Fu	448538 CI	2BJ4	1S9Q	1YIN	38.77	0.82	Sc=6.2365, min distance = 2.0630048
ERR3_HUMAN F	445155 O-	TTHY_HUMAN Fu	448537 di	1TT6	1S9P	1DVZ			Sc=6.10062, min distance = 2.340954
ERR3_HUMAN F	445920 RA	ESR1_HUMAN Fu	448537 di	3ERD	1S9P	1GWQ	38.77		Sc=5.95111, min distance = 0.878665
ERR3_HUMAN F	446849 Lo	ESR1_HUMAN Fu	449459 4-	3ERT	2GPU	1L2I	38.77		Sc=6.14335, min distance = 2.284979
ERR3_HUMAN F	448577 1s	ESR1_HUMAN Fu	448538 CI	2BJ4	1S9Q	1SJ0	38.77		Sc=6.21758, min distance = 1.913711
ERR3_HUMAN F	448708 CI	TTHY_HUMAN Fu	448537 di	1TT6	1S9P	1THC			Sc=6.34583, min distance = 1.928346
ERR3_HUMAN F	448734 CI	TTHY_HUMAN Fu	448537 di	1TT6	1S9P	1TLM			Sc=6.07799, min distance = 1.754574
ERR3_HUMAN F	448915 1u	ESR1_HUMAN Fu	449459 4-	3ERT	2GPU	1UOM	38.77		Sc=6.10968, min distance = 2.104882
ERR3_HUMAN F	449207 1x	ESR1_HUMAN Fu	448538 CI	2BJ4	1S9Q	1XP6	38.77	0.77	Sc=6.22486, min distance = 2.524795
ERR3_HUMAN F	449208 Co	ESR1_HUMAN Fu	448538 CI	2BJ4	1S9Q	1XP9	38.77		Sc=6.21497, min distance = 2.098519
ERR3_HUMAN F	449212 CI	ESR1_HUMAN Fu	448538 CI	2BJ4	1S9Q	1XQC	38.77		Sc=6.1162, min distance = 2.2117588
ERR3_HUMAN F	5035 ralo	ESR1_HUMAN Fu	448538 CI	2BJ4	1S9Q	2QXS	38.77		Sc=6.35367, min distance = 1.560848
ERR3_HUMAN F	5280961 g	ESR1_HUMAN Fu	449459 4-	3ERT	2GPU	2QA8	38.77		Sc=6.178, min distance = 2.11628589
ERR3_HUMAN F	5288193 2	TTHY_HUMAN Fu	448537 di	1TT6	1S9P	2B9A			Sc=5.91431, min distance = 2.099000
ERR3_HUMAN F	5326827 1	ESR2_HUMAN Fu	448538 CI	2FSZ	1S9Q	1U3R	35.5		Sc=6.17128, min distance = 1.231522
ERR3_HUMAN F	5326893 E	ESR2_HUMAN Fu	448538 CI	2FSZ	1S9Q	1X7B	35.5		Sc=6.12959, min distance = 1.050065
ERR3_HUMAN F	5327159 2	TTHY_HUMAN Fu	448537 di	1TT6	1S9P	2F7I			Sc=5.88464, min distance = 2.590317
ERR3_HUMAN F	5327160 2	TTHY_HUMAN Fu	448537 di	1TT6	1S9P	2F8I			Sc=5.9783, min distance = 2.2164681
ERR3_HUMAN F	5478840 C	ESR1_HUMAN Fu	448537 di	3ERD	1S9P	1R5K	38.77		Sc=6.23758, min distance = 0.636057
ERR3_HUMAN F	6102690 1	ESR2_HUMAN Fu	448538 CI	2FSZ	1S9Q	1YY4	35.5		Sc=6.00705, min distance = 1.627714

ERR3_HUMAN F	6542593 1	ESR1_HUMAN Fu	448537 di	3ERD	1S9P	1ZKY	38.77	0.78	Sc=6.09345, min distance = 1.62963
ERR3_HUMAN F	656936 1u	ESR2_HUMAN Fu	448538 CI	2FSZ	1S9Q	1U9E	35.5		Sc=5.93159, min distance = 1.48861
ERR3_HUMAN F	656952 1x	ESR2_HUMAN Fu	448538 CI	2FSZ	1S9Q	1X76	35.5		Sc=6.10482, min distance = 0.65594
ERR3_HUMAN F	656953 1x	ESR1_HUMAN Fu	448537 di	3ERD	1S9P	1X7E	38.77		Sc=6.17187, min distance = 0.52995
ERR3_HUMAN F	656953 1x	ESR1_HUMAN Fu	448538 CI	2BJ4	1S9Q	1X7E	38.77		Sc=6.1908, min distance = 1.516756
ERR3_HUMAN F	656953 1x	ESR2_HUMAN Fu	448538 CI	2FSZ	1S9Q	1X78	35.5		Sc=6.1534, min distance = 1.094519
ERR3_HUMAN F	6852154 2	ESR1_HUMAN Fu	448537 di	3ERD	1S9P	2B1V	38.77	0.79	Sc=6.00229, min distance = 0.74353
ERR3_HUMAN F	6852186 2	ESR1_HUMAN Fu	448537 di	3ERD	1S9P	2FAI	38.77	0.79	Sc=6.03564, min distance = 0.89481
ERR3_HUMAN F	97032 1g3	TTHY_HUMAN Fu	448537 di	1TT6	1S9P	2G5U		0.75	Sc=5.90743, min distance = 2.42807
ERR3_HUMAN F	9927355 2	ESR1_HUMAN Fu	448537 di	3ERD	1S9P	2QE4	38.77		Sc=6.13074, min distance = 1.88758
ERR3_HUMAN F	9927355 2	ESR1_HUMAN Fu	448538 CI	2BJ4	1S9Q	2QE4	38.77		Sc=6.07727, min distance = 2.20168
ERR3_HUMAN F	9927355 2	ESR2_HUMAN Fu	448538 CI	2FSZ	1S9Q	2JJ3	35.5		Sc=6.14199, min distance = 0.89467
ESR1_HUMAN F	10286462	ESR2_HUMAN Fu	448538 CI	2FSZ	2BJ4	1ZAF	61.84		Sc=6.03519, min distance = 2.48198
ESR1_HUMAN F	10286462	ESR2_HUMAN Fu	5280961 g	1QKM	2QA8	1ZAF	61.84		Sc=6.08003, min distance = 2.68583
ESR1_HUMAN F	10286462	ESR2_HUMAN Fu	656953 1x	1X78	1X7E	1ZAF	61.84		Sc=6.06947, min distance = 2.00504
ESR1_HUMAN F	10635 And	SHBG_HUMAN Fu	5757 estr	1LHU	1QKT	1D2S			Sc=6.00035, min distance = 2.18416
ESR1_HUMAN F	11987846	ESR2_HUMAN Fu	11197931	2Z4B	2Q70	2GIU	61.84		Sc=5.93419, min distance = 2.26073
ESR1_HUMAN F	11987846	ESR2_HUMAN Fu	5280961 g	1QKM	2QA8	2GIU	61.84		Sc=6.01004, min distance = 2.16360
ESR1_HUMAN F	11987846	ESR2_HUMAN Fu	9927355 2	2JJ3	2QE4	2GIU	61.84		Sc=5.90099, min distance = 2.22849
ESR1_HUMAN F	16758210	ESR2_HUMAN Fu	448538 CI	2FSZ	2BJ4	2NV7	61.84		Sc=6.11568, min distance = 1.20212
ESR1_HUMAN F	16758210	ESR2_HUMAN Fu	5280961 g	1QKM	2QA8	2NV7	61.84		Sc=6.13252, min distance = 2.38778
ESR1_HUMAN F	16758210	ESR2_HUMAN Fu	656953 1x	1X78	1X7E	2NV7	61.84		Sc=6.16023, min distance = 1.74231
ESR1_HUMAN F	16758210	ESR2_HUMAN Fu	9927355 2	2JJ3	2QE4	2NV7	61.84		Sc=6.08596, min distance = 2.48271
ESR1_HUMAN F	177880 2'	TTHY_HUMAN Fu	448537 di	1TT6	3ERD	2G9K			Sc=5.73945, min distance = 2.19084
ESR1_HUMAN F	20284644	TTHY_HUMAN Fu	448537 di	1TT6	3ERD	2QGC			Sc=5.94355, min distance = 1.70359
ESR1_HUMAN F	222865 Ar	DHB1_HUMAN Fu	5757 estr	1FDS	1QKT	1QYW			Sc=6.03675, min distance = 2.11231
ESR1_HUMAN F	23722943	TTHY_HUMAN Fu	448537 di	1TT6	3ERD	2QGD			Sc=5.89947, min distance = 1.99414
ESR1_HUMAN F	23722944	TTHY_HUMAN Fu	448537 di	1TT6	3ERD	2QGE			Sc=5.76854, min distance = 2.53442
ESR1_HUMAN F	242332 3b	SHBG_HUMAN Fu	5757 estr	1LHU	1QKT	1LHO			Sc=6.02051, min distance = 2.17003
ESR1_HUMAN F	243556 NS	TTHY_HUMAN Fu	448537 di	1TT6	3ERD	3CN2		0.78	Sc=5.63429, min distance = 2.58993
ESR1_HUMAN F	4369551 1	ESR2_HUMAN Fu	10286159	2I0G	2I0J	1U3S	61.84		Sc=6.17518, min distance = 0.82204
ESR1_HUMAN F	4369551 1	ESR2_HUMAN Fu	11197931	2Z4B	2Q70	1U3S	61.84		Sc=6.13041, min distance = 1.85166
ESR1_HUMAN F	4369551 1	ESR2_HUMAN Fu	448538 CI	2FSZ	2BJ4	1U3S	61.84		Sc=6.01669, min distance = 2.14228
ESR1_HUMAN F	4369551 1	ESR2_HUMAN Fu	5280961 g	1QKM	2QA8	1U3S	61.84		Sc=6.05424, min distance = 2.56478
ESR1_HUMAN F	4369551 1	ESR2_HUMAN Fu	656953 1x	1X78	1X7E	1U3S	61.84		Sc=6.03564, min distance = 2.24596
ESR1_HUMAN F	4369551 1	ESR2_HUMAN Fu	9927355 2	2JJ3	2QE4	1U3S	61.84		Sc=6.19842, min distance = 0.87285
ESR1_HUMAN F	4369563 I	TTHY_HUMAN Fu	448537 di	1TT6	3ERD	1Y1D			Sc=6.06823, min distance = 1.91403

ESR1_HUMAN F	445071 D	DHB1_HUMAN Fu	5757 estr	1FDS	1QKT	1DHT			Sc=6.0123, min distance = 2.434783
ESR1_HUMAN F	445155 O-	TTHY_HUMAN Fu	448537 di	1TT6	3ERD	1DVZ			Sc=6.06918, min distance = 2.331223
ESR1_HUMAN F	449455 C	DHB1_HUMAN Fu	5757 estr	1FDS	1QKT	3DHE			Sc=6.02661, min distance = 2.60609
ESR1_HUMAN F	5288193 2	TTHY_HUMAN Fu	448537 di	1TT6	3ERD	2B9A			Sc=5.8801, min distance = 2.325715
ESR1_HUMAN F	5326826 E	ESR2_HUMAN Fu	11197931	2Z4B	2Q70	1U3Q	61.84		Sc=6.1036, min distance = 1.386243
ESR1_HUMAN F	5326826 E	ESR2_HUMAN Fu	9927355 2	2JJ3	2QE4	1U3Q	61.84		Sc=6.12731, min distance = 1.51808
ESR1_HUMAN F	5326827 1	ESR2_HUMAN Fu	448538 CI	2FSZ	2BJ4	1U3R	61.84		Sc=6.16943, min distance = 1.15649
ESR1_HUMAN F	5326827 1	ESR2_HUMAN Fu	5280961 g	1QKM	2QA8	1U3R	61.84		Sc=6.0461, min distance = 2.482799
ESR1_HUMAN F	5326827 1	ESR2_HUMAN Fu	656953 1x	1X78	1X7E	1U3R	61.84		Sc=6.02843, min distance = 1.455611
ESR1_HUMAN F	5326827 1	ESR2_HUMAN Fu	9927355 2	2JJ3	2QE4	1U3R	61.84		Sc=6.17894, min distance = 0.63830
ESR1_HUMAN F	5326893 E	ESR2_HUMAN Fu	10286159	2I0G	2I0J	1X7B	61.84		Sc=6.16041, min distance = 0.59057
ESR1_HUMAN F	5326893 E	ESR2_HUMAN Fu	446849 Lc	1L2J	1L2I	1X7B	61.84		Sc=6.03206, min distance = 1.74057
ESR1_HUMAN F	5326893 E	ESR2_HUMAN Fu	448538 CI	2FSZ	2BJ4	1X7B	61.84		Sc=6.08768, min distance = 1.31691
ESR1_HUMAN F	5326893 E	ESR2_HUMAN Fu	5280961 g	1QKM	2QA8	1X7B	61.84		Sc=6.10614, min distance = 2.56361
ESR1_HUMAN F	5326893 E	ESR2_HUMAN Fu	656953 1x	1X78	1X7E	1X7B	61.84		Sc=6.11294, min distance = 1.55381
ESR1_HUMAN F	5326893 E	ESR2_HUMAN Fu	9927355 2	2JJ3	2QE4	1X7B	61.84		Sc=6.16813, min distance = 1.20470
ESR1_HUMAN F	5327159 2	TTHY_HUMAN Fu	448537 di	1TT6	3ERD	2F7I			Sc=5.84077, min distance = 2.59328
ESR1_HUMAN F	5327160 2	TTHY_HUMAN Fu	448537 di	1TT6	3ERD	2F8I			Sc=5.95111, min distance = 2.34903
ESR1_HUMAN F	6102690 1	ESR2_HUMAN Fu	10286159	2I0G	2I0J	1YY4	61.84		Sc=6.04643, min distance = 1.59244
ESR1_HUMAN F	6102690 1	ESR2_HUMAN Fu	11197931	2Z4B	2Q70	1YY4	61.84		Sc=6.02009, min distance = 1.15300
ESR1_HUMAN F	6102690 1	ESR2_HUMAN Fu	448538 CI	2FSZ	2BJ4	1YY4	61.84		Sc=6.08081, min distance = 0.92481
ESR1_HUMAN F	6102690 1	ESR2_HUMAN Fu	5280961 g	1QKM	2QA8	1YY4	61.84		Sc=5.99881, min distance = 2.55389
ESR1_HUMAN F	6102690 1	ESR2_HUMAN Fu	656953 1x	1X78	1X7E	1YY4	61.84		Sc=5.98219, min distance = 1.76355
ESR1_HUMAN F	6102690 1	ESR2_HUMAN Fu	9927355 2	2JJ3	2QE4	1YY4	61.84		Sc=6.03736, min distance = 0.77375
ESR1_HUMAN F	6102691 1	ESR2_HUMAN Fu	10286159	2I0G	2I0J	1YYE	61.84		Sc=6.11814, min distance = 1.77454
ESR1_HUMAN F	6102691 1	ESR2_HUMAN Fu	11197931	2Z4B	2Q70	1YYE	61.84		Sc=5.98705, min distance = 2.06041
ESR1_HUMAN F	6102691 1	ESR2_HUMAN Fu	448538 CI	2FSZ	2BJ4	1YYE	61.84		Sc=6.14953, min distance = 1.05506
ESR1_HUMAN F	6102691 1	ESR2_HUMAN Fu	5280961 g	1QKM	2QA8	1YYE	61.84		Sc=6.15805, min distance = 2.56968
ESR1_HUMAN F	6102691 1	ESR2_HUMAN Fu	656953 1x	1X78	1X7E	1YYE	61.84		Sc=6.18975, min distance = 1.72103
ESR1_HUMAN F	6102691 1	ESR2_HUMAN Fu	9927355 2	2JJ3	2QE4	1YYE	61.84		Sc=6.11439, min distance = 2.39558
ESR1_HUMAN F	656936 1u	ESR2_HUMAN Fu	448538 CI	2FSZ	2BJ4	1U9E	61.84		Sc=6.00204, min distance = 1.07907
ESR1_HUMAN F	656936 1u	ESR2_HUMAN Fu	5280961 g	1QKM	2QA8	1U9E	61.84	0.79	Sc=6.05515, min distance = 0.53932
ESR1_HUMAN F	656936 1u	ESR2_HUMAN Fu	656953 1x	1X78	1X7E	1U9E	61.84	0.93	Sc=5.89549, min distance = 1.72201
ESR1_HUMAN F	656936 1u	ESR2_HUMAN Fu	9927355 2	2JJ3	2QE4	1U9E	61.84		Sc=5.9594, min distance = 0.340324
ESR1_HUMAN F	656952 1x	ESR2_HUMAN Fu	10286159	2I0G	2I0J	1X76	61.84		Sc=6.13282, min distance = 0.75974
ESR1_HUMAN F	656952 1x	ESR2_HUMAN Fu	11197931	2Z4B	2Q70	1X76	61.84		Sc=6.1171, min distance = 1.061243
ESR1_HUMAN F	656952 1x	ESR2_HUMAN Fu	448538 CI	2FSZ	2BJ4	1X76	61.84		Sc=6.15131, min distance = 1.15128

ESR1_HUMAN F	656952 1x	ESR2_HUMAN Fu	5280961 g	1QKM	2QA8	1X76	61.84		Sc=6.08888, min distance = 2.389965
ESR1_HUMAN F	656952 1x	ESR2_HUMAN Fu	656953 1x	1X78	1X7E	1X76	61.84	0.99	Sc=6.08119, min distance = 1.575648
ESR1_HUMAN F	656952 1x	ESR2_HUMAN Fu	9927355 2	2JJ3	2QE4	1X76	61.84		Sc=6.13766, min distance = 0.929959
ESR1_HUMAN F	6623 Bisp	ERR3_HUMAN Fu	448538 CI	1S9Q	2BJ4	2E2R	38.77	0.75	Sc=5.91712, min distance = 2.179749
ESR1_HUMAN F	6852176 2	ERR3_HUMAN Fu	448538 CI	1S9Q	2BJ4	2EWP	38.77	0.98	Sc=6.2138, min distance = 1.7920013
ESR1_HUMAN F	72099 flu	TTHY_HUMAN Fu	448537 di	1TT6	3ERD	1DVT			Sc=5.92475, min distance = 2.324851
ESR1_HUMAN F	97032 1g3	TTHY_HUMAN Fu	448537 di	1TT6	3ERD	2G5U		0.75	Sc=5.86683, min distance = 2.569408
ESR2_HUMAN F	10019418	ESR1_HUMAN Fu	446849 Lc	1L2I	1L2J	2G5O	61.84	0.79	Sc=6.10267, min distance = 2.461905
ESR2_HUMAN F	104946 Li	ESR1_HUMAN Fu	448538 CI	2BJ4	2FSZ	2R6Y	61.84		Sc=6.32184, min distance = 2.270131
ESR2_HUMAN F	11149479	ESR1_HUMAN Fu	11197931	2Q70	2Z4B	2QAB	61.84		Sc=6.06688, min distance = 2.462420
ESR2_HUMAN F	11149479	ESR1_HUMAN Fu	446849 Lc	1L2I	1L2J	2QAB	61.84		Sc=5.85325, min distance = 2.511264
ESR2_HUMAN F	11149479	ESR1_HUMAN Fu	448538 CI	2BJ4	2FSZ	2QAB	61.84		Sc=6.08003, min distance = 1.147228
ESR2_HUMAN F	11149479	ESR1_HUMAN Fu	5280961 g	2QA8	1QKM	2QAB	61.84		Sc=5.98675, min distance = 1.996245
ESR2_HUMAN F	11149479	ESR1_HUMAN Fu	656953 1x	1X7E	1X78	2QAB	61.84		Sc=6.18708, min distance = 1.221596
ESR2_HUMAN F	11149479	ESR1_HUMAN Fu	9927355 2	2QE4	2JJ3	2QAB	61.84		Sc=6.06947, min distance = 2.633925
ESR2_HUMAN F	11368987	ESR1_HUMAN Fu	10286159	2I0J	2I0G	2QGW	61.84		Sc=6.0011, min distance = 2.2135729
ESR2_HUMAN F	11368987	ESR1_HUMAN Fu	11197931	2Q70	2Z4B	2QGW	61.84		Sc=6.0102, min distance = 2.3588329
ESR2_HUMAN F	11368987	ESR1_HUMAN Fu	446849 Lc	1L2I	1L2J	2QGW	61.84		Sc=5.78977, min distance = 2.388735
ESR2_HUMAN F	11368987	ESR1_HUMAN Fu	448538 CI	2BJ4	2FSZ	2QGW	61.84		Sc=5.94664, min distance = 1.083404
ESR2_HUMAN F	11368987	ESR1_HUMAN Fu	5280961 g	2QA8	1QKM	2QGW	61.84		Sc=5.9307, min distance = 2.5857372
ESR2_HUMAN F	11368987	ESR1_HUMAN Fu	656953 1x	1X7E	1X78	2QGW	61.84		Sc=6.14697, min distance = 1.520085
ESR2_HUMAN F	14086997	ESR1_HUMAN Fu	10286159	2I0J	2I0G	2QA6	61.84		Sc=6.08293, min distance = 2.132882
ESR2_HUMAN F	14086997	ESR1_HUMAN Fu	11197931	2Q70	2Z4B	2QA6	61.84		Sc=6.08121, min distance = 2.176203
ESR2_HUMAN F	14086997	ESR1_HUMAN Fu	448538 CI	2BJ4	2FSZ	2QA6	61.84		Sc=6.25916, min distance = 1.282304
ESR2_HUMAN F	14086997	ESR1_HUMAN Fu	5280961 g	2QA8	1QKM	2QA6	61.84		Sc=6.24633, min distance = 1.722499
ESR2_HUMAN F	14086997	ESR1_HUMAN Fu	656953 1x	1X7E	1X78	2QA6	61.84		Sc=6.32655, min distance = 1.477892
ESR2_HUMAN F	14086997	ESR1_HUMAN Fu	9927355 2	2QE4	2JJ3	2QA6	61.84		Sc=6.11818, min distance = 1.829446
ESR2_HUMAN F	1530 PhIE	ESR1_HUMAN Fu	10286159	2I0J	2I0G	2QXM	61.84		Sc=5.99354, min distance = 2.012501
ESR2_HUMAN F	1530 PhIE	ESR1_HUMAN Fu	11197931	2Q70	2Z4B	2QXM	61.84		Sc=5.85665, min distance = 1.916175
ESR2_HUMAN F	1530 PhIE	ESR1_HUMAN Fu	448538 CI	2BJ4	2FSZ	2QXM	61.84		Sc=6.04238, min distance = 1.366935
ESR2_HUMAN F	1530 PhIE	ESR1_HUMAN Fu	5280961 g	2QA8	1QKM	2QXM	61.84		Sc=6.02151, min distance = 2.811380
ESR2_HUMAN F	1530 PhIE	ESR1_HUMAN Fu	656953 1x	1X7E	1X78	2QXM	61.84		Sc=6.12725, min distance = 0.705969
ESR2_HUMAN F	1530 PhIE	ESR1_HUMAN Fu	9927355 2	2QE4	2JJ3	2QXM	61.84		Sc=5.85843, min distance = 2.234564
ESR2_HUMAN F	15897928	ESR1_HUMAN Fu	10286159	2I0J	2I0G	2QSE	61.84		Sc=6.12077, min distance = 2.148321
ESR2_HUMAN F	15897928	ESR1_HUMAN Fu	11197931	2Q70	2Z4B	2QSE	61.84		Sc=6.01199, min distance = 1.959265
ESR2_HUMAN F	15897928	ESR1_HUMAN Fu	446849 Lc	1L2I	1L2J	2QSE	61.84		Sc=5.80263, min distance = 2.883023
ESR2_HUMAN F	15897928	ESR1_HUMAN Fu	448538 CI	2BJ4	2FSZ	2QSE	61.84		Sc=6.18767, min distance = 1.078577

ESR2_HUMAN Fı	15897928	ESR1_HUMAN Fu:	5280961 g 2QA8	1QKM	2QSE	61.84		Sc=6.17523, min distance = 1.749241
ESR2_HUMAN Fı	15897928	ESR1_HUMAN Fu:	656953 1x 1X7E	1X78	2QSE	61.84		Sc=6.25718, min distance = 0.858991
ESR2_HUMAN Fı	15897928	ESR1_HUMAN Fu:	9927355 2 2QE4	2JJ3	2QSE	61.84		Sc=5.99508, min distance = 2.529901
ESR2_HUMAN Fı	15942662	ESR1_HUMAN Fu:	10286159 2I0J	2I0G	2G44	61.84	0.8	Sc=5.81821, min distance = 2.936545
ESR2_HUMAN Fı	15942662	ESR1_HUMAN Fu:	11197931 2Q70	2Z4B	2G44	61.84	0.78	Sc=5.826, min distance = 2.3383894(
ESR2_HUMAN Fı	15942662	ESR1_HUMAN Fu:	446849 Lc 1L2I	1L2J	2G44	61.84	0.79	Sc=5.70697, min distance = 2.551255
ESR2_HUMAN Fı	15942662	ESR1_HUMAN Fu:	448538 CI 2BJ4	2FSZ	2G44	61.84	0.78	Sc=5.85574, min distance = 1.751953
ESR2_HUMAN Fı	15942662	ESR1_HUMAN Fu:	5280961 g 2QA8	1QKM	2G44	61.84		Sc=5.87959, min distance = 2.162841
ESR2_HUMAN Fı	15942662	ESR1_HUMAN Fu:	656953 1x 1X7E	1X78	2G44	61.84		Sc=5.96092, min distance = 1.599585
ESR2_HUMAN Fı	15942662	ESR1_HUMAN Fu:	9927355 2 2QE4	2JJ3	2G44	61.84	0.78	Sc=5.85574, min distance = 2.322448
ESR2_HUMAN Fı	16214804	ESR1_HUMAN Fu:	448538 CI 2BJ4	2FSZ	2Q6J	61.84		Sc=6.11096, min distance = 0.903949
ESR2_HUMAN Fı	16750074	ESR1_HUMAN Fu:	11197931 2Q70	2Z4B	2POG	61.84	0.89	Sc=6.02396, min distance = 2.725941
ESR2_HUMAN Fı	16750074	ESR1_HUMAN Fu:	448538 CI 2BJ4	2FSZ	2POG	61.84	0.76	Sc=6.06325, min distance = 1.619902
ESR2_HUMAN Fı	16750074	ESR1_HUMAN Fu:	5280961 g 2QA8	1QKM	2POG	61.84	0.76	Sc=6.06109, min distance = 2.277969
ESR2_HUMAN Fı	16750074	ESR1_HUMAN Fu:	656953 1x 1X7E	1X78	2POG	61.84		Sc=6.14901, min distance = 0.701429
ESR2_HUMAN Fı	16750074	ESR1_HUMAN Fu:	9927355 2 2QE4	2JJ3	2POG	61.84	0.9	Sc=6.03061, min distance = 2.157112
ESR2_HUMAN Fı	216416 La	ESR1_HUMAN Fu:	448538 CI 2BJ4	2FSZ	2OUZ	61.84	0.88	Sc=6.03215, min distance = 2.746496
ESR2_HUMAN Fı	23728517	ESR1_HUMAN Fu:	10286159 2I0J	2I0G	2QGT	61.84	0.77	Sc=6.08475, min distance = 1.869813
ESR2_HUMAN Fı	23728517	ESR1_HUMAN Fu:	11197931 2Q70	2Z4B	2QGT	61.84	0.75	Sc=6.08667, min distance = 2.245698
ESR2_HUMAN Fı	23728517	ESR1_HUMAN Fu:	448538 CI 2BJ4	2FSZ	2QGT	61.84		Sc=6.11572, min distance = 1.789504
ESR2_HUMAN Fı	23728517	ESR1_HUMAN Fu:	5280961 g 2QA8	1QKM	2QGT	61.84		Sc=6.13074, min distance = 2.039812
ESR2_HUMAN Fı	23728517	ESR1_HUMAN Fu:	656953 1x 1X7E	1X78	2QGT	61.84		Sc=6.19824, min distance = 0.713148
ESR2_HUMAN Fı	23728517	ESR1_HUMAN Fu:	9927355 2 2QE4	2JJ3	2QGT	61.84	0.76	Sc=6.07929, min distance = 2.379346
ESR2_HUMAN Fı	24892830	ESR1_HUMAN Fu:	448538 CI 2BJ4	2FSZ	3DT3	61.84		Sc=6.30024, min distance = 1.522701
ESR2_HUMAN Fı	24892830	ESR1_HUMAN Fu:	656953 1x 1X7E	1X78	3DT3	61.84		Sc=6.39193, min distance = 0.812849
ESR2_HUMAN Fı	24892830	ESR1_HUMAN Fu:	9927355 2 2QE4	2JJ3	3DT3	61.84	0.83	Sc=6.29541, min distance = 1.874095
ESR2_HUMAN Fı	4369568 C	ESR1_HUMAN Fu:	448538 CI 2BJ4	2FSZ	1YIM	61.84	0.85	Sc=6.21409, min distance = 2.440952
ESR2_HUMAN Fı	4369569 C	ESR1_HUMAN Fu:	448538 CI 2BJ4	2FSZ	1YIN	61.84	0.82	Sc=6.23896, min distance = 2.048410
ESR2_HUMAN Fı	445920 RA	ESR1_HUMAN Fu:	10286159 2I0J	2I0G	1GWQ	61.84		Sc=5.92505, min distance = 1.980294
ESR2_HUMAN Fı	445920 RA	ESR1_HUMAN Fu:	11197931 2Q70	2Z4B	1GWQ	61.84		Sc=5.91553, min distance = 1.896642
ESR2_HUMAN Fı	445920 RA	ESR1_HUMAN Fu:	446849 Lc 1L2I	1L2J	1GWQ	61.84		Sc=5.82424, min distance = 2.400120
ESR2_HUMAN Fı	445920 RA	ESR1_HUMAN Fu:	448538 CI 2BJ4	2FSZ	1GWQ	61.84		Sc=5.96492, min distance = 1.888894
ESR2_HUMAN Fı	445920 RA	ESR1_HUMAN Fu:	5280961 g 2QA8	1QKM	1GWQ	61.84		Sc=5.9594, min distance = 1.4871748
ESR2_HUMAN Fı	445920 RA	ESR1_HUMAN Fu:	656953 1x 1X7E	1X78	1GWQ	61.84		Sc=6.04603, min distance = 1.083961
ESR2_HUMAN Fı	445920 RA	ESR1_HUMAN Fu:	9927355 2 2QE4	2JJ3	1GWQ	61.84		Sc=5.92246, min distance = 2.671797
ESR2_HUMAN Fı	448537 di	ESR1_HUMAN Fu:	446849 Lc 1L2I	1L2J	3ERD	61.84	0.86	Sc=5.93225, min distance = 2.711498
ESR2_HUMAN Fı	448537 di	ESR1_HUMAN Fu:	448538 CI 2BJ4	2FSZ	3ERD	61.84		Sc=6.05608, min distance = 1.382852

ESR2_HUMAN Fı	448537 di	ESR1_HUMAN Fu	5280961 g	2QA8	1QKM	3ERD	61.84		Sc=6.06325, min distance = 1.773521
ESR2_HUMAN Fı	448537 di	ESR1_HUMAN Fu	656953 1x	1X7E	1X78	3ERD	61.84		Sc=6.13675, min distance = 1.241835
ESR2_HUMAN Fı	448537 di	ESR1_HUMAN Fu	9927355 2	2QE4	2JJ3	3ERD	61.84		Sc=6.03984, min distance = 1.964032
ESR2_HUMAN Fı	448577 1s	ESR1_HUMAN Fu	448538 CI	2BJ4	2FSZ	1SJ0	61.84		Sc=6.21561, min distance = 1.971277
ESR2_HUMAN Fı	448915 1u	ESR1_HUMAN Fu	448538 CI	2BJ4	2FSZ	1UOM	61.84		Sc=6.0477, min distance = 2.5714235
ESR2_HUMAN Fı	449207 1x	ESR1_HUMAN Fu	448538 CI	2BJ4	2FSZ	1XP6	61.84	0.77	Sc=6.22084, min distance = 2.410697
ESR2_HUMAN Fı	449208 Cc	ESR1_HUMAN Fu	448538 CI	2BJ4	2FSZ	1XP9	61.84		Sc=6.21102, min distance = 2.108752
ESR2_HUMAN Fı	449212 CI	ESR1_HUMAN Fu	448538 CI	2BJ4	2FSZ	1XQC	61.84		Sc=6.09895, min distance = 2.418544
ESR2_HUMAN Fı	449459 4-	ERR3_HUMAN Fu	448538 CI	1S9Q	2FSZ	2GPU	35.5	0.98	Sc=6.02458, min distance = 2.104546
ESR2_HUMAN Fı	449459 4-	ESR1_HUMAN Fu	448538 CI	2BJ4	2FSZ	3ERT	61.84	0.98	Sc=5.99654, min distance = 2.348097
ESR2_HUMAN Fı	5035 ralc	ESR1_HUMAN Fu	448538 CI	2BJ4	2FSZ	2QXS	61.84		Sc=6.33292, min distance = 1.136034
ESR2_HUMAN Fı	5757 estr	ESR1_HUMAN Fu	10286159	2I0J	2I0G	1QKT	61.84		Sc=6.00533, min distance = 1.884311
ESR2_HUMAN Fı	5757 estr	ESR1_HUMAN Fu	11197931	2Q70	2Z4B	1QKT	61.84		Sc=6.01024, min distance = 2.487096
ESR2_HUMAN Fı	5757 estr	ESR1_HUMAN Fu	448538 CI	2BJ4	2FSZ	1QKT	61.84		Sc=6.05608, min distance = 1.679564
ESR2_HUMAN Fı	5757 estr	ESR1_HUMAN Fu	5280961 g	2QA8	1QKM	1QKT	61.84		Sc=6.05189, min distance = 2.472995
ESR2_HUMAN Fı	5757 estr	ESR1_HUMAN Fu	656953 1x	1X7E	1X78	1QKT	61.84		Sc=6.14072, min distance = 1.521819
ESR2_HUMAN Fı	5757 estr	ESR1_HUMAN Fu	9927355 2	2QE4	2JJ3	1QKT	61.84		Sc=6.02747, min distance = 2.691951
ESR2_HUMAN Fı	6542593 1	ESR1_HUMAN Fu	10286159	2I0J	2I0G	1ZKY	61.84	0.78	Sc=6.02038, min distance = 1.613774
ESR2_HUMAN Fı	6542593 1	ESR1_HUMAN Fu	11197931	2Q70	2Z4B	1ZKY	61.84	0.76	Sc=6.02173, min distance = 2.005259
ESR2_HUMAN Fı	6542593 1	ESR1_HUMAN Fu	446849 Lc	1L2I	1L2J	1ZKY	61.84	0.77	Sc=5.93045, min distance = 2.169425
ESR2_HUMAN Fı	6542593 1	ESR1_HUMAN Fu	448538 CI	2BJ4	2FSZ	1ZKY	61.84	0.76	Sc=6.05608, min distance = 1.455681
ESR2_HUMAN Fı	6542593 1	ESR1_HUMAN Fu	5280961 g	2QA8	1QKM	1ZKY	61.84		Sc=6.03183, min distance = 2.351991
ESR2_HUMAN Fı	6542593 1	ESR1_HUMAN Fu	656953 1x	1X7E	1X78	1ZKY	61.84		Sc=6.14417, min distance = 0.995339
ESR2_HUMAN Fı	6623 Bisp	ERR3_HUMAN Fu	448538 CI	1S9Q	2FSZ	2E2R	35.5	0.75	Sc=6.04234, min distance = 1.099624
ESR2_HUMAN Fı	6852154 2	ESR1_HUMAN Fu	10286159	2I0J	2I0G	2B1V	61.84	0.77	Sc=5.9518, min distance = 2.1823402
ESR2_HUMAN Fı	6852154 2	ESR1_HUMAN Fu	11197931	2Q70	2Z4B	2B1V	61.84		Sc=5.95606, min distance = 2.178084
ESR2_HUMAN Fı	6852154 2	ESR1_HUMAN Fu	446849 Lc	1L2I	1L2J	2B1V	61.84	0.76	Sc=5.82286, min distance = 2.623838
ESR2_HUMAN Fı	6852154 2	ESR1_HUMAN Fu	448538 CI	2BJ4	2FSZ	2B1V	61.84		Sc=5.98089, min distance = 1.952631
ESR2_HUMAN Fı	6852154 2	ESR1_HUMAN Fu	5280961 g	2QA8	1QKM	2B1V	61.84		Sc=5.98738, min distance = 1.798204
ESR2_HUMAN Fı	6852154 2	ESR1_HUMAN Fu	656953 1x	1X7E	1X78	2B1V	61.84		Sc=6.09074, min distance = 1.402280
ESR2_HUMAN Fı	6852154 2	ESR1_HUMAN Fu	9927355 2	2QE4	2JJ3	2B1V	61.84	0.76	Sc=5.95606, min distance = 2.466771
ESR2_HUMAN Fı	6852176 2	ERR3_HUMAN Fu	448538 CI	1S9Q	2FSZ	2EWP	35.5	0.98	Sc=6.20658, min distance = 1.643947
ESR2_HUMAN Fı	6852186 2	ESR1_HUMAN Fu	10286159	2I0J	2I0G	2FAI	61.84	0.78	Sc=5.98705, min distance = 2.447664
ESR2_HUMAN Fı	6852186 2	ESR1_HUMAN Fu	11197931	2Q70	2Z4B	2FAI	61.84	0.75	Sc=5.98574, min distance = 2.546960
ESR2_HUMAN Fı	6852186 2	ESR1_HUMAN Fu	446849 Lc	1L2I	1L2J	2FAI	61.84	0.76	Sc=5.85654, min distance = 2.711071
ESR2_HUMAN Fı	6852186 2	ESR1_HUMAN Fu	448538 CI	2BJ4	2FSZ	2FAI	61.84	0.75	Sc=6.03245, min distance = 1.709122
ESR2_HUMAN Fı	6852186 2	ESR1_HUMAN Fu	5280961 g	2QA8	1QKM	2FAI	61.84		Sc=6.03797, min distance = 2.118932

ESR2_HUMAN Fu	6852186 2	ESR1_HUMAN Fu	656953 1x	1X7E	1X78	2FAI	61.84		Sc=6.11304, min distance = 1.56726
ESR2_HUMAN Fu	6852186 2	ESR1_HUMAN Fu	9927355 2	2QE4	2JJ3	2FAI	61.84	0.77	Sc=5.99007, min distance = 2.44137
ESR2_HUMAN Fu	9549180 C	ESR1_HUMAN Fu	10286159	2I0J	2I0G	2B1Z	61.84		Sc=6.04575, min distance = 2.61840
ESR2_HUMAN Fu	9549180 C	ESR1_HUMAN Fu	11197931	2Q70	2Z4B	2B1Z	61.84		Sc=6.04801, min distance = 2.14009
ESR2_HUMAN Fu	9549180 C	ESR1_HUMAN Fu	9927355 2	2QE4	2JJ3	2B1Z	61.84		Sc=6.0593, min distance = 2.513664
ESR2_RAT Ful	10286159	ESR1_HUMAN Fu	5035 ralc	2QXS	1QKN	2I0J	61.57		Sc=5.98915, min distance = 1.89773
ESR2_RAT Ful	10635 And	SHBG_HUMAN Fu	5757 estr	1LHU	2J7X	1D2S			Sc=6.02051, min distance = 2.32068
ESR2_RAT Ful	11149479	ESR1_HUMAN Fu	5757 estr	1QKT	2J7X	2QAB	61.57		Sc=5.92475, min distance = 2.34072
ESR2_RAT Ful	11368987	ESR1_HUMAN Fu	5757 estr	1QKT	2J7X	2QGW	61.57		Sc=5.87388, min distance = 2.36289
ESR2_RAT Ful	14086997	ESR1_HUMAN Fu	5757 estr	1QKT	2J7X	2QA6	61.57		Sc=6.09743, min distance = 2.68239
ESR2_RAT Ful	1530 PhIE	ESR1_HUMAN Fu	5757 estr	1QKT	2J7X	2QXM	61.57		Sc=5.94743, min distance = 2.30652
ESR2_RAT Ful	15897928	ESR1_HUMAN Fu	5035 ralc	2QXS	1QKN	2QSE	61.57		Sc=5.99817, min distance = 1.67601
ESR2_RAT Ful	15897928	ESR1_HUMAN Fu	5757 estr	1QKT	2J7X	2QSE	61.57		Sc=6.09434, min distance = 2.54846
ESR2_RAT Ful	15942662	ESR1_HUMAN Fu	5035 ralc	2QXS	1QKN	2G44	61.57		Sc=5.7971, min distance = 2.495717
ESR2_RAT Ful	15942662	ESR1_HUMAN Fu	5757 estr	1QKT	2J7X	2G44	61.57	0.75	Sc=5.79523, min distance = 2.33187
ESR2_RAT Ful	16750074	ESR1_HUMAN Fu	5035 ralc	2QXS	1QKN	2POG	61.57		Sc=5.99838, min distance = 2.38759
ESR2_RAT Ful	16750074	ESR1_HUMAN Fu	5757 estr	1QKT	2J7X	2POG	61.57		Sc=6.01269, min distance = 2.26570
ESR2_RAT Ful	216416 La	ESR1_HUMAN Fu	5035 ralc	2QXS	1QKN	2OUZ	61.57		Sc=6.04358, min distance = 2.50919
ESR2_RAT Ful	222865 Ar	DHB1_HUMAN Fu	5757 estr	1FDS	2J7X	1QYW			Sc=6.04912, min distance = 2.22161
ESR2_RAT Ful	242332 3b	SHBG_HUMAN Fu	5757 estr	1LHU	2J7X	1LHO			Sc=6.02791, min distance = 2.32054
ESR2_RAT Ful	245468 6-	DHB1_HUMAN Fu	5757 estr	1FDS	2J7X	1FDU		0.97	Sc=6.01519, min distance = 1.82593
ESR2_RAT Ful	24892830	ESR1_HUMAN Fu	5035 ralc	2QXS	1QKN	3DT3	61.57		Sc=6.27055, min distance = 2.30209
ESR2_RAT Ful	4369568 C	ESR1_HUMAN Fu	5035 ralc	2QXS	1QKN	1YIM	61.57		Sc=6.21689, min distance = 2.51576
ESR2_RAT Ful	4369569 C	ESR1_HUMAN Fu	5035 ralc	2QXS	1QKN	1YIN	61.57		Sc=6.23287, min distance = 2.21973
ESR2_RAT Ful	445071 DH	DHB1_HUMAN Fu	5757 estr	1FDS	2J7X	1DHT			Sc=6.04139, min distance = 2.55168
ESR2_RAT Ful	445410 EQ	DHB1_HUMAN Fu	5757 estr	1FDS	2J7X	1EQU		0.86	Sc=6.00097, min distance = 2.61381
ESR2_RAT Ful	445920 RA	ESR1_HUMAN Fu	5757 estr	1QKT	2J7X	1GWQ	61.57		Sc=5.89401, min distance = 2.31443
ESR2_RAT Ful	446849 Lo	ESR1_HUMAN Fu	5035 ralc	2QXS	1QKN	1L2I	61.57		Sc=6.12252, min distance = 1.63853
ESR2_RAT Ful	446849 Lo	ESR1_HUMAN Fu	5757 estr	1QKT	2J7X	1L2I	61.57	0.83	Sc=6.12343, min distance = 2.36954
ESR2_RAT Ful	448538 CI	ESR1_HUMAN Fu	5035 ralc	2QXS	1QKN	2BJ4	61.57		Sc=6.02273, min distance = 2.48423
ESR2_RAT Ful	448577 1s	ESR1_HUMAN Fu	5035 ralc	2QXS	1QKN	1SJ0	61.57		Sc=6.20961, min distance = 2.05411
ESR2_RAT Ful	448915 1u	ESR1_HUMAN Fu	5035 ralc	2QXS	1QKN	1UOM	61.57		Sc=6.03215, min distance = 2.41504
ESR2_RAT Ful	449207 1x	ESR1_HUMAN Fu	5035 ralc	2QXS	1QKN	1XP6	61.57		Sc=6.21838, min distance = 2.62045
ESR2_RAT Ful	449208 Co	ESR1_HUMAN Fu	5035 ralc	2QXS	1QKN	1XP9	61.57		Sc=6.20658, min distance = 2.23763
ESR2_RAT Ful	449212 CI	ESR1_HUMAN Fu	5035 ralc	2QXS	1QKN	1XQC	61.57		Sc=6.07592, min distance = 2.21960
ESR2_RAT Ful	449455 CI	DHB1_HUMAN Fu	5757 estr	1FDS	2J7X	3DHE			Sc=6.05376, min distance = 2.41202
ESR2_RAT Ful	449459 4-	ESR1_HUMAN Fu	5035 ralc	2QXS	1QKN	3ERT	61.57		Sc=6.0009, min distance = 2.335132

ESR2_RAT Ful	6128 andr	DHB1_HUMAN Fu	5757 estr	1FDS	2J7X	1QYX			Sc=6.04139, min distance = 2.08280(
ESR2_RAT Ful	6542593 1	ESR1_HUMAN Fu	5757 estr	1QKT	2J7X	1ZKY	61.57	0.78	Sc=5.97271, min distance = 2.36222
ESR2_RAT Ful	656953 1x	ESR1_HUMAN Fu	5757 estr	1QKT	2J7X	1X7E	61.57		Sc=6.09896, min distance = 2.34312
ESR2_RAT Ful	6852154 2	ESR1_HUMAN Fu	5035 ralc	2QXS	1QKN	2B1V	61.57		Sc=5.93028, min distance = 1.66423
ESR2_RAT Ful	6852154 2	ESR1_HUMAN Fu	5757 estr	1QKT	2J7X	2B1V	61.57	0.76	Sc=5.9198, min distance = 2.249775
ESR2_RAT Ful	6852186 2	ESR1_HUMAN Fu	5757 estr	1QKT	2J7X	2FAI	61.57	0.77	Sc=5.94964, min distance = 2.40111
EST1_HUMAN Fu	11006 HEX	CD1D1_MOUSE Fu	985 palmi	2FIK	2DQY	1Z5L			Sc=5.70165, min distance = 0.70015
EST1_HUMAN Fu	14253 Cet	FABP4_MOUSE Fu	985 palmi	1LIE	2DQY	1LIC			Sc=5.67679, min distance = 2.42609
EST1_HUMAN Fu	14368760	LPXC_AQUAE Fu	985 palmi	2GO3	2DQY	2O3Z			Sc=5.98255, min distance = 0.76305
EST1_HUMAN Fu	175468 12	LACB_BOVIN Fu	985 palmi	1GXA	2DQY	1BSO		0.83	Sc=5.82313, min distance = 2.31969
EST1_HUMAN Fu	439353 be	TETX_CLOTE Fu	444885 O-	1FV3	2H7C	1DIW			Sc=5.66873, min distance = 2.09068
EST1_HUMAN Fu	439353 be	TRFL_BOVIN Fu	444205 CI	2G93	1MX9	2DWJ			Sc=5.91793, min distance = 0.51758(
EST1_HUMAN Fu	439554 fu	TRFL_BOVIN Fu	444205 CI	2G93	1MX9	2DWA			Sc=5.63561, min distance = 2.29535
EST1_HUMAN Fu	446367 BA	NRAM_I67A0 Fu	444885 O-	2BAT	2H7C	1IVE			Sc=6.00168, min distance = 2.40391
EST1_HUMAN Fu	5280934 l	NLTP_MAIZE Fu	985 palmi	1MZM	2DQY	1FK6		0.75	Sc=6.04235, min distance = 1.33593
EST1_HUMAN Fu	64689 bet	P74325_SYNY3	5988 sucr	1TJ5	2HRQ	1U2S		0.87	Sc=5.74332, min distance = 2.29259(
EST_BACST Fu	445939 1g	PPTA_ECOLI Fu	23831 HEF	1GYX	1R1D	1GYY			Sc=5.77347, min distance = 1.97655
EST_BACST Fu	5962 L-ly	RBCMT_PEA Ful	23831 HEF	1MLV	1R1D	2H2E			Sc=5.68442, min distance = 2.20671
EST_BACST Fu	62551 Per	Q8WSF8_APLCA	23831 HEF	2BR7	1R1D	2BYN			Sc=5.73537, min distance = 2.04307
ETFB_HUMAN Fu	24905143	SRC_CHICK Ful	6083 aden	3DQX	1EFV	3EN7			Sc=6.31699, min distance = 2.09383
ETFB_HUMAN Fu	2519 caff	PYGM_RABIT Fu	6083 aden	8GPB	1EFV	1GFZ			Sc=5.83602, min distance = 2.07229
ETFB_HUMAN Fu	5327121 I	PIM1_HUMAN Fu	6083 aden	1YXU	1EFV	2C3I			Sc=6.34738, min distance = 1.57793
ETFB_HUMAN Fu	6419789 C	HASP_HUMAN Fu	6083 aden	3DLZ	1EFV	3E7V			Sc=6.34552, min distance = 2.44980
ETFB_HUMAN Fu	65533 cor	O57693_THETE	6083 aden	1UXU	1EFV	1UXT			Sc=6.06626, min distance = 2.01851
ETFB_HUMAN Fu	656968 1y	PDE4B_HUMAN Fu	6083 aden	1ROR	1EFV	1Y2H			Sc=6.01664, min distance = 2.15374
ETFB_HUMAN Fu	657135 Op	PIM1_HUMAN Fu	6083 aden	1YXU	1EFV	1YXV			Sc=5.91707, min distance = 2.36764
ETFB_HUMAN Fu	8582 Inos	PURA1_MOUSE Fu	6083 aden	1MF0	1EFV	1IWE		0.79	Sc=6.42061, min distance = 2.10397
ETFB_HUMAN Fu	8582 Inos	PYGM_RABIT Fu	6083 aden	8GPB	1EFV	2QN7		0.79	Sc=6.43925, min distance = 2.27909
ETFB_METME Fu	1400 nche	RET_HUMAN Ful	6083 aden	2IVT	1O97	2IVV			Sc=6.27898, min distance = 2.57405
ETFB_METME Fu	24905143	SRC_CHICK Ful	6083 aden	3DQX	1O97	3EN7			Sc=6.31464, min distance = 2.37089
ETFB_METME Fu	24905152	SRC_CHICK Ful	6083 aden	3DQX	1O97	3EN6			Sc=6.39829, min distance = 2.34026
ETFB_METME Fu	25011744	SRC_CHICK Ful	6083 aden	3DQX	1O97	3EL7			Sc=6.6568, min distance = 1.863333(
ETFB_METME Fu	25011745	SRC_CHICK Ful	6083 aden	3DQX	1O97	3EL8			Sc=6.59741, min distance = 1.76297
ETFB_METME Fu	447832 AD	ACSA_SALTY Fu	6083 aden	2P2F	1O97	1PG4		0.99	Sc=6.52986, min distance = 2.01033
ETFB_METME Fu	5327121 I	PIM1_HUMAN Fu	6083 aden	1YXU	1O97	2C3I			Sc=6.33791, min distance = 2.17657
ETFB_METME Fu	6030 Uric	ECX1_SULSO Fu	6083 aden	2C38	1O97	2C37			Sc=6.34561, min distance = 2.34869
ETFB_METME Fu	6076 Cycl	PDE10_HUMAN Fu	6083 aden	2OUN	1O97	2OUR		0.99	Sc=6.34436, min distance = 2.01554

ETFB_METME F	6419789 C	HASP_HUMAN Fu	6083 aden	3DLZ	1O97	3E7V		Sc=6.33275, min distance = 2.32197
ETFB_METME F	656968 1y	PDE4B_HUMAN F	6083 aden	1ROR	1O97	1Y2H		Sc=6.03552, min distance = 2.03064
ETFB_METME F	657135 Op	PIM1_HUMAN Fu	6083 aden	1YXU	1O97	1YXV		Sc=5.94459, min distance = 2.13935
ETFB_PARDE F	24180721	PIM1_HUMAN Fu	6083 aden	1YXU	1EFP	3C4E		Sc=6.08028, min distance = 2.42618
ETFB_PARDE F	2519 caff	PYGM_RABIT Fu	6083 aden	8GPB	1EFP	1GFZ		Sc=5.86511, min distance = 1.98106
ETFB_PARDE F	445708 AM	NADE_ECOLI Fu	6083 aden	1WXI	1EFP	1WXE	0.91	Sc=6.48562, min distance = 0.18607
ETFB_PARDE F	656964 1y	PDE4D_HUMAN F	6083 aden	1TB7	1EFP	1Y2C		Sc=6.00296, min distance = 1.70504
ETFB_PARDE F	656968 1y	PDE4B_HUMAN F	6083 aden	1ROR	1EFP	1Y2H		Sc=6.02538, min distance = 1.94868
ETFB_PARDE F	657135 Op	PIM1_HUMAN Fu	6083 aden	1YXU	1EFP	1YXV		Sc=5.91029, min distance = 2.41231
ETFB_PARDE F	8582 Inos	PURA1_MOUSE F	6083 aden	1MF0	1EFP	1IWE	0.79	Sc=6.47253, min distance = 0.86746
ETFB_PARDE F	8582 Inos	PYGM_RABIT Fu	6083 aden	8GPB	1EFP	2QN7	0.79	Sc=6.43876, min distance = 2.39970
ETFD_PIG Ful	11987634	GLPD_ECOLI Fu	444188 CI	2QCU	2GMH	2R4E	0.97	Sc=6.44263, min distance = 1.86886
ETFD_PIG Ful	16740985	FRDA_SHEFR Fu	444188 CI	1M64	2GMH	1E93	0.93	Sc=6.43459, min distance = 2.20955
ETFD_PIG Ful	16741253	LSD1_HUMAN Fu	444188 CI	2DW4	2GMH	2UXN	0.95	Sc=6.44112, min distance = 2.32548
ETFD_PIG Ful	444502 CI	FRDA_SHEFR Fu	444188 CI	1M64	2GMH	1QJD	0.98	Sc=6.43576, min distance = 2.19517
ETFD_PIG Ful	444502 CI	TYTR_TRYCR Fu	444188 CI	1BZL	2GMH	1GXF	0.98	Sc=6.44504, min distance = 2.57505
ETFD_PIG Ful	446013 1,	O28603_ARCFU I	444188 CI	1JNR	2GMH	1JNZ	0.94	Sc=6.44388, min distance = 2.03955
ETFD_PIG Ful	446013 1,	O28604_ARCFU I	444188 CI	1JNR	2GMH	1JNZ	0.94	Sc=6.44388, min distance = 2.04891
ETR1_CANTR F	440141 9i	G6PD_LEUME Fu	5886 NADP	1H9A	1GUF	1E7Y		Sc=5.82314, min distance = 2.24900
ETR1_CANTR F	440334 Ni	DAPB_ECOLI Fu	5886 NADP	1DIH	1GUF	1DRW	0.91	Sc=5.98885, min distance = 2.40533
EXG_CANAL Fu	124434 Ca	BGLA_THEMA Fu	54445 cas	2CBU	1EQC	2CBV	0.79	Sc=5.92801, min distance = 2.53164
EXG_CANAL Fu	16214802	Q2PS28_9PSED I	54445 cas	2PWG	1EQC	2PWE		Sc=6.44176, min distance = 2.18867
EXG_CANAL Fu	16741013	CARP_YEAST Fu	64689 bet	1FQ4	2PC8	1FQ6		Sc=5.71966, min distance = 2.20604
EXG_CANAL Fu	16741014	CARP_YEAST Fu	64689 bet	1FQ4	2PC8	1FQ7		Sc=5.73598, min distance = 2.35080
EXG_CANAL Fu	24963032	BGLA_THEMA Fu	54445 cas	2CBU	1EQC	2VRJ		Sc=6.13104, min distance = 2.45227
EXG_CANAL Fu	444809 Th	GUX2_TRIRE Fu	64689 bet	1QK0	2PC8	1QJW	0.78	Sc=5.70694, min distance = 2.75113
EXG_CANAL Fu	444809 Th	Q79G13_MYCTU I	64689 bet	1UP0	2PC8	1UOZ	0.78	Sc=5.80345, min distance = 1.46581
EXG_CANAL Fu	445999 CI	GUN5_BACAG Fu	64689 bet	1W3L	2PC8	1H5V	0.77	Sc=5.62862, min distance = 2.17223
EXG_CANAL Fu	447600 CI	GUX6_HUMIN Fu	64689 bet	1OCN	2PC8	1OCB	0.83	Sc=5.68985, min distance = 2.37059
EXG_CANAL Fu	447601 CI	GUX6_HUMIN Fu	64689 bet	1OCN	2PC8	1OCB		Sc=5.74009, min distance = 2.22223
EXG_CANAL Fu	447607 1c	BGLA_THEMA Fu	54445 cas	2CBU	1EQC	1OIF		Sc=5.74975, min distance = 2.53003
EXG_CANAL Fu	447607 1c	Q79G13_MYCTU I	64689 bet	1UP0	2PC8	1UP2		Sc=5.89688, min distance = 0.75562
EXG_CANAL Fu	447651 CI	BGLA_THEMA Fu	54445 cas	2CBU	1EQC	2J77		Sc=5.85925, min distance = 2.44192
EXG_CANAL Fu	5327083 C	GUN2_THEFU Fu	64689 bet	2BOF	2PC8	2BOD		Sc=5.88823, min distance = 0.76325
EXTL2_MOUSE I	17473 UDP	B3GA3_HUMAN F	6031 Urid	3CU0	1ON8	1KWS	0.78	Sc=6.62468, min distance = 2.30597
EXTL2_MOUSE I	6030 Urid	PYRH_ECOLI Fu	6031 Urid	2BND	1ON8	2BNE	0.99	Sc=6.28339, min distance = 2.14252
EXTL2_MOUSE I	6132 Cyti	ECX1_PYRAB Fu	6031 Urid	2PNZ	1ON8	2PO2	0.83	Sc=5.87838, min distance = 2.01017

EXTL2_MOUSE	8629 UDP-	MGAT1_RABIT F	6031 Urid	2AM5	1ON8	2AM3		0.89	Sc=6.17064, min distance = 2.01733
EXTL2_MOUSE	8629 UDP-	OTSA_ECOLI Fu	6031 Urid	1GZ5	1ON8	1UQU		0.89	Sc=6.62135, min distance = 1.67937
EXTL2_MOUSE	8977 1dar	RIR1_YEAST Fu	6031 Urid	2CVV	1ON8	2CVW			Sc=6.02881, min distance = 2.25807
F16P1_PIG Fu	1400 nche	RET_HUMAN Full	6083 aden	2IVT	2F3D	2IVV			Sc=6.27823, min distance = 2.07426
F16P1_PIG Fu	3758 IBMX	PDE4D_HUMAN F	6083 aden	1TB7	2F3D	1ZKN			Sc=5.87651, min distance = 2.30035
F16P1_PIG Fu	439427 Ro	CGGR_BACSU Fu	440641 1m	3BXH	1NUW	3BXG		0.89	Sc=5.98919, min distance = 2.48598
F16P1_PIG Fu	449190 1x	PDE4B_HUMAN F	6083 aden	1ROR	2F3D	1XM6			Sc=5.94462, min distance = 2.24948
F16P1_PIG Fu	611002 Op	PIM1_HUMAN Fu	6083 aden	1YXU	2F3D	1YXX			Sc=6.15834, min distance = 1.49501
F16P1_PIG Fu	656968 1y	PDE4B_HUMAN F	6083 aden	1ROR	2F3D	1Y2H			Sc=5.79232, min distance = 1.67228
F16P1_PIG Fu	657135 Op	PIM1_HUMAN Fu	6083 aden	1YXU	2F3D	1YXV			Sc=5.78719, min distance = 2.58786
F16PA_ECOLI	107737 ir	SUHB_ARCFU Fu	10267 Har	1LBZ	2Q8M	1LBX		0.75	Sc=6.0693, min distance = 1.703903
F16PA_ECOLI	439162 gl	F261_RAT Full	440641 1m	1TIP	2OWZ	1C7Z			Sc=5.70473, min distance = 0.94958
F16PA_ECOLI	439427 Ro	CGGR_BACSU Fu	440641 1m	3BXH	2OWZ	3BXG		0.89	Sc=5.96982, min distance = 2.40036
F16PA_ECOLI	444848 F6	F16P1_PIG Full	105021 Fr	2QVU	2QVR	1CNQ		0.98	Sc=5.93931, min distance = 2.40531
F16PA_ECOLI	6083 aden	O57693_THETE	440641 1m	1UXR	2OWZ	1UXU			Sc=6.34658, min distance = 2.08964
F17AG_ECOLX	185698 al	MBL2_RAT Full	24139 ace	1KZD	2BSC	1RDL			Sc=5.82145, min distance = 2.35152
F17AG_ECOLX	444205 CI	DPP4_HUMAN Fu	24139 ace	2RGU	2BSC	3BJM		0.91	Sc=6.05676, min distance = 2.25814
F17AG_ECOLX	444863 is	MBL2_RAT Full	24139 ace	1KZD	2BSC	1RDJ			Sc=5.70694, min distance = 2.33460
F17AG_ECOLX	656981 CU	LYSC_CHICK Fu	24139 ace	1LZB	2BSC	1YIL			Sc=5.76685, min distance = 2.76571
F17BG_ECOLX	444205 CI	DPP4_HUMAN Fu	24139 ace	2RGU	2BS8	3BJM		0.91	Sc=6.05676, min distance = 1.76526
F17BG_ECOLX	444863 is	MBL2_RAT Full	24139 ace	1KZD	2BS8	1RDJ			Sc=5.72788, min distance = 2.45047
F17EG_ECOLX	185698 al	MBL2_RAT Full	24139 ace	1KZD	2BSB	1RDL			Sc=5.83634, min distance = 2.35387
F17EG_ECOLX	4369268 C	Q309D1_9AGAR	24139 ace	2C4D	2BSB	2BWM		0.88	Sc=6.06486, min distance = 1.66582
F17EG_ECOLX	444205 CI	DPP4_HUMAN Fu	24139 ace	2RGU	2BSB	3BJM		0.91	Sc=6.05873, min distance = 2.20744
F17EG_ECOLX	444863 is	MBL2_RAT Full	24139 ace	1KZD	2BSB	1RDJ			Sc=5.71966, min distance = 2.37944
F17EG_ECOLX	656981 CU	LYSC_CHICK Fu	24139 ace	1LZB	2BSB	1YIL			Sc=5.80183, min distance = 2.77812
F17FG_ECOLX	185698 al	MBL2_RAT Full	24139 ace	1KZD	1ZK5	1RDL			Sc=5.82145, min distance = 2.31225
F17FG_ECOLX	4369268 C	Q309D1_9AGAR	24139 ace	2C4D	1ZK5	2BWM		0.88	Sc=6.0535, min distance = 1.927748
F17FG_ECOLX	444205 CI	DPP4_HUMAN Fu	24139 ace	2RGU	1ZK5	3BJM		0.91	Sc=6.04584, min distance = 2.17623
F17FG_ECOLX	444863 is	MBL2_RAT Full	24139 ace	1KZD	1ZK5	1RDJ			Sc=5.69804, min distance = 2.30263
F17FG_ECOLX	656981 CU	LYSC_CHICK Fu	24139 ace	1LZB	1ZK5	1YIL			Sc=5.89381, min distance = 2.63488
F17GG_ECOLX	16058615	LYSC_CHICK Fu	24139 ace	1LZB	1OIO	2H9J			Sc=5.93784, min distance = 2.42217
F17GG_ECOLX	185698 al	MBL2_RAT Full	24139 ace	1KZD	1OIO	1RDL			Sc=5.84504, min distance = 2.16779
F17GG_ECOLX	439353 be	MBL2_RAT Full	24139 ace	1KZD	1OIO	1RDK			Sc=5.84332, min distance = 2.30559
F17GG_ECOLX	444205 CI	DPP4_HUMAN Fu	24139 ace	2RGU	1OIO	3BJM		0.91	Sc=6.06561, min distance = 1.87466
F17GG_ECOLX	444863 is	MBL2_RAT Full	24139 ace	1KZD	1OIO	1RDJ			Sc=5.72788, min distance = 2.24571
F17GG_ECOLX	64689 bet	LEG3_HUMAN Fu	24139 ace	1KJL	1OIO	2NMO			Sc=5.8196, min distance = 2.508706

F17GG_ECOLX I	656981 CU	LYSC_CHICK Fu	24139 ace	1LZB	1OIO	1YIL			Sc=5.92645, min distance = 2.34779
F261_RAT Ful	439427 Ro	CGGR_BACSU Fu	440641 1m	3BXH	1TIP	3BXG		0.89	Sc=5.90916, min distance = 2.03219
F261_RAT Ful	443201 DC	PDXJ_ECOLI Fu	439162 gl	1IXO	1C7Z	1M5W			Sc=5.77551, min distance = 1.37689
F261_RAT Ful	446381 1u	PDXJ_ECOLI Fu	439162 gl	1IXO	1C7Z	1IXN			Sc=5.63725, min distance = 1.29514
F261_RAT Ful	6083 ader	O57693_THETE I	440641 1m	1UXR	1TIP	1UXU			Sc=5.89119, min distance = 2.42731
F261_RAT Ful	7478 p-Ar	PA28_DABRR Fu	439162 gl	1Y38	1C7Z	1SV3			Sc=5.63106, min distance = 1.02002
F263_HUMAN Fu	1540 1h1q	CDK2_HUMAN Fu	6022 Aden	1GY3	2AXN	1H1Q			Sc=6.37965, min distance = 1.75319
F263_HUMAN Fu	23653518	CDK2_HUMAN Fu	6022 Aden	1GY3	2AXN	2R3J			Sc=6.27123, min distance = 2.00687
F263_HUMAN Fu	447649 1o	CDK2_HUMAN Fu	6022 Aden	1GY3	2AXN	1OI9			Sc=6.44418, min distance = 1.70158
F263_HUMAN Fu	447655 1o	CDK2_HUMAN Fu	6022 Aden	1GY3	2AXN	1OIU			Sc=6.40253, min distance = 1.98454
F263_HUMAN Fu	447656 1o	CDK2_HUMAN Fu	6022 Aden	1GY3	2AXN	1OIY			Sc=6.51722, min distance = 1.55381
F263_HUMAN Fu	4565 1h1r	CDK2_HUMAN Fu	6022 Aden	1GY3	2AXN	1H1R			Sc=6.38349, min distance = 1.77838
F263_HUMAN Fu	5289411 1	CDK2_HUMAN Fu	6022 Aden	1GY3	2AXN	1OGU			Sc=6.53636, min distance = 2.12250
F263_HUMAN Fu	656971 CI	CDK2_HUMAN Fu	6022 Aden	1GY3	2AXN	1Y8Y			Sc=6.03372, min distance = 2.03149
F263_HUMAN Fu	9547890 I	CDK2_HUMAN Fu	6022 Aden	1GY3	2AXN	1W8C			Sc=6.28422, min distance = 1.60161
F263_HUMAN Fu	9994066 4	CDK2_HUMAN Fu	6022 Aden	1GY3	2AXN	2VTJ			Sc=5.90833, min distance = 1.95618
F264_RAT Ful	15993 dAT	PRIM_BPT7 Ful	33113 gam	1E0J	2BIF	1CR2		0.97	Sc=6.48097, min distance = 1.50642
F264_RAT Ful	188966 dA	HSLU_ECOLI Fu	33113 gam	1E94	2BIF	1G4A		0.97	Sc=6.41112, min distance = 2.13135
F264_RAT Ful	30951 alc	PGH1_SHEEP Fu	62852 B-C	1Q4G	3BIF	1HT8			Sc=5.95942, min distance = 0.79800
F264_RAT Ful	439427 Ro	CGGR_BACSU Fu	440641 1m	3BXH	2BIF	3BXG		0.89	Sc=5.97275, min distance = 1.81751
F264_RAT Ful	444279 1s	OMPG_ECOLI Fu	62852 B-C	2IWW	3BIF	2IWV			Sc=5.93207, min distance = 1.07611
F264_RAT Ful	446721 CI	ASSY_THET8 Fu	33113 gam	1KOR	2BIF	1KH2		0.93	Sc=6.48483, min distance = 1.44787
FA10_HUMAN Fu	183797 Me	TRY1_BOVIN Fu	445756 1g	1G36	1G2M	1K1P	55.9		Sc=6.65174, min distance = 2.37929
FA10_HUMAN Fu	183797 Me	TRY1_BOVIN Fu	9600423 1	1OYQ	1G2L	1K1P	55.9		Sc=6.66527, min distance = 2.27517
FA10_HUMAN Fu	2386 CID2	TRY1_BOVIN Fu	445756 1g	1G36	1G2M	1C1T	55.9	0.91	Sc=6.58247, min distance = 2.25462
FA10_HUMAN Fu	2386 CID2	TRY1_BOVIN Fu	9600423 1	1OYQ	1G2L	1C1T	55.9		Sc=6.59235, min distance = 2.13457
FA10_HUMAN Fu	24963036	TRY1_BOVIN Fu	9600423 1	1OYQ	1G2L	2ZDM	55.9		Sc=6.52426, min distance = 2.13132
FA10_HUMAN Fu	445296 BE	TRY1_BOVIN Fu	445756 1g	1G36	1G2M	1EB2	55.9		Sc=6.62737, min distance = 2.36037
FA10_HUMAN Fu	445296 BE	TRY1_BOVIN Fu	5497056 1	1F0U	1EZQ	1EB2	55.9	0.81	Sc=6.63988, min distance = 2.12958
FA10_HUMAN Fu	5326542 A	TRY1_BOVIN Fu	445756 1g	1G36	1G2M	1GHZ	55.9		Sc=6.27537, min distance = 2.14945
FA10_HUMAN Fu	5326542 A	TRY1_BOVIN Fu	9600423 1	1OYQ	1G2L	1GHZ	55.9		Sc=6.29466, min distance = 2.17735
FA10_HUMAN Fu	5326544 C	TRY1_BOVIN Fu	9600423 1	1OYQ	1G2L	1GI4	55.9		Sc=6.30968, min distance = 2.01537
FA10_HUMAN Fu	5326923 1	TRY1_BOVIN Fu	445756 1g	1G36	1G2M	1Y3Y	55.9		Sc=6.19842, min distance = 2.34794
FA10_HUMAN Fu	5326923 1	TRY1_BOVIN Fu	9600423 1	1OYQ	1G2L	1Y3Y	55.9		Sc=6.22206, min distance = 2.26321
FA10_HUMAN Fu	5326925 D	TRY1_BOVIN Fu	445756 1g	1G36	1G2M	1Y5A	55.9		Sc=6.40391, min distance = 2.38760
FA10_HUMAN Fu	5494448 1	TRY1_BOVIN Fu	9600423 1	1OYQ	1G2L	1YP9	55.9		Sc=6.39216, min distance = 2.24922
FA10_HUMAN Fu	5496535 2	TRY1_BOVIN Fu	445756 1g	1G36	1G2M	1G3B	55.9		Sc=6.22932, min distance = 2.50974

FA10_HUMAN Fu	9547939 2	TRY1_BOVIN Ful	9600423 1	1OYQ	1G2L	2G5N	55.9		Sc=6.13816, min distance = 2.00338(
FA10_HUMAN Fu	9547940 C	TRY1_BOVIN Ful	5497056 1	1F0U	1EZQ	2G5V	55.9		Sc=6.12036, min distance = 2.110032
FA10_HUMAN Fu	9547940 C	TRY1_BOVIN Ful	9600423 1	1OYQ	1G2L	2G5V	55.9		Sc=6.11464, min distance = 2.20967
FABG1_BRANA I	15942680	6PGD_LACLM Ful	5886 NADF	2IZ0	1EDO	2IZ1			Sc=6.21784, min distance = 2.12713(
FABG1_BRANA I	440141 9i	DHB1_HUMAN Ful	5886 NADF	1QYV	1EDO	1QYW			Sc=6.39666, min distance = 2.05052
FABG_ECOLI Fu	15942680	6PGD_LACLM Ful	5886 NADF	2IZ0	1Q7B	2IZ1			Sc=5.70939, min distance = 2.54352
FABG_ECOLI Fu	440141 9i	DHB1_HUMAN Ful	5886 NADF	1QYV	1Q7B	1QYW			Sc=5.85476, min distance = 2.70266
FABG_ECOLI Fu	440141 9i	G6PD_LEUME Ful	165230 1a	2DPG	1Q7C	1E7Y		0.99	Sc=5.88603, min distance = 2.02790
FABG_ECOLI Fu	440141 9i	G6PD_LEUME Ful	5886 NADF	1H9A	1Q7B	1E7Y			Sc=5.90326, min distance = 2.25001(
FABI_BRANA Fu	446185 Di	FABI_ECOLI Ful	5564 tric	1QSG	1D7O	1I2Z			Sc=5.72476, min distance = 2.17039
FABI_ECOLI Fu	169266 1,	GALE_HUMAN Ful	5893 nadi	1HZJ	1QSG	1I3K		0.79	Sc=6.74234, min distance = 2.47530(
FABI_ECOLI Fu	439153 Di	Q9BJJ9_PLAFA I	5893 nadi	1UH5	1QSG	1V35		0.79	Sc=6.74044, min distance = 2.48897
FABI_ECOLI Fu	445794 AD	ADHX_HUMAN Ful	5893 nadi	2FZW	1QSG	2FZE			Sc=5.88916, min distance = 1.28739
FABI_HELPY Fu	11957405	INHA_MYCTU Ful	5564 tric	2B35	2PD3	2H7P	35.27		Sc=6.15327, min distance = 2.18999
FABI_HELPY Fu	2266114 C	INHA_MYCTU Ful	5564 tric	2B35	2PD3	2H7I	35.27		Sc=6.24608, min distance = 0.75708
FABI_HELPY Fu	439153 Di	Q9BJJ9_PLAFA I	5893 nadi	1UH5	2PD4	1V35	34.25	0.79	Sc=6.30628, min distance = 1.99842(
FABI_HELPY Fu	446288 NA	G3P_PALVE Ful	5893 nadi	1DSS	2PD4	1IHX			Sc=6.78861, min distance = 1.31107
FABI_HELPY Fu	447370 CI	Q9BH77_PLAFA I	5564 tric	2O2Y	2PD3	1NHW	36.51		Sc=5.87731, min distance = 1.45061
FABI_HELPY Fu	447409 CI	Q9BH77_PLAFA I	5564 tric	2O2Y	2PD3	1NNU	36.51	0.76	Sc=6.05832, min distance = 1.34105
FABI_HELPY Fu	447767 pi	INHA_MYCTU Ful	5893 nadi	2H7I	2PD4	1P44	35.27		Sc=6.2398, min distance = 0.817489
FABI_HELPY Fu	6852148 2	Q9BH77_PLAFA I	5564 tric	2O2Y	2PD3	1ZW1	36.51	0.79	Sc=5.96752, min distance = 0.71443
FABP1_ECHGR I	12389 Tet	LTC4S_HUMAN Fu	985 palmi	2UUH	1O8V	2UUI			Sc=5.95001, min distance = 2.01191
FABP1_ECHGR I	14253 Cet	FABP4_MOUSE Fu	985 palmi	1LIE	1O8V	1LIC			Sc=5.69027, min distance = 2.02685
FABP1_ECHGR I	16741231	LTC4S_HUMAN Fu	985 palmi	2UUH	1O8V	2PNO			Sc=6.19301, min distance = 0.79567
FABP2_MANSE I	14253 Cet	FABP4_MOUSE Fu	985 palmi	1LIE	1MDC	1LIC			Sc=5.68198, min distance = 2.16802
FABP2_MANSE I	14368760	LPXC_AQUAE Ful	985 palmi	2GO3	1MDC	2O3Z			Sc=5.85497, min distance = 2.27838
FABP2_MANSE I	175468 12	LACB_BOVIN Ful	985 palmi	1GXA	1MDC	1BSO		0.83	Sc=5.74093, min distance = 2.07142
FABP4_MOUSE I	151008 N-	CPXB_BACME Ful	985 palmi	1FAG	1LIE	1JPZ			Sc=6.23388, min distance = 2.31840
FABP4_MOUSE I	16741231	LTC4S_HUMAN Fu	985 palmi	2UUH	1LIE	2PNO			Sc=5.88518, min distance = 0.79682
FABP4_MOUSE I	447234 Ho	EST1_HUMAN Ful	985 palmi	2DQY	1LIE	1MX5			Sc=5.98219, min distance = 0.80002
FABP4_MOUSE I	448769 1t	FABP4_HUMAN Fu	985 palmi	2HNX	1LIE	1TOW			Sc=5.81825, min distance = 2.77851(
FABPH_HUMAN I	449171 Al	RXRA_MOUSE Ful	5281 stea	1DKF	1HMR	1XDK			Sc=6.01653, min distance = 1.97614
FABPI_RAT Ful	12389 Tet	LTC4S_HUMAN Fu	985 palmi	2UUH	2IFB	2UUI			Sc=5.95001, min distance = 1.06569
FABPI_RAT Ful	14253 Cet	FABP4_MOUSE Fu	985 palmi	1LIE	2IFB	1LIC			Sc=5.67679, min distance = 2.75661
FABPI_RAT Ful	14368760	LPXC_AQUAE Ful	11005 Tet	1P42	1ICM	2O3Z			Sc=5.94101, min distance = 1.51631(
FABPI_RAT Ful	14368760	LPXC_AQUAE Ful	985 palmi	2GO3	2IFB	2O3Z			Sc=5.97731, min distance = 1.79632
FABPI_RAT Ful	8209 Myri	ABL1_MOUSE Ful	11005 Tet	1OPK	1ICM	1OPJ			Sc=5.70849, min distance = 2.26407

FABPL_DANRE I	4369567 M	EST1_HUMAN Fu	221493 ch	2DQY	2QO4	1YA8		Sc=6.13326, min distance = 1.45774
FABP_SCHMA Fu	10467 Ara	ALBU_HUMAN Fu	445639 ol	1GNI	1VYF	1GNJ	0.77	Sc=6.06851, min distance = 2.28346
FABP_SCHMA Fu	12530 n-T	ALBU_HUMAN Fu	445639 ol	1GNI	1VYF	1HK4		Sc=5.92379, min distance = 0.54511
FABP_SCHMA Fu	2969 Deca	ALBU_HUMAN Fu	445639 ol	1GNI	1VYF	2VDB		Sc=5.69787, min distance = 2.10628
FABP_SCHMA Fu	3893 laur	ALBU_HUMAN Fu	445639 ol	1GNI	1VYF	1E7G		Sc=5.91243, min distance = 0.86820
FABP_SCHMA Fu	4943 prop	ALBU_HUMAN Fu	445639 ol	1GNI	1VYF	1E7A		Sc=5.70899, min distance = 2.08104
FABP_SCHMA Fu	8158 Pela	ALBU_HUMAN Fu	445639 ol	1GNI	1VYF	1E7E		Sc=5.61837, min distance = 2.31840
FADB_PSEFR Fu	16129587	G3PA_SPIOL Fu	5893 nadi	1NBO	1WDK	2PKR		Sc=6.021, min distance = 2.2549760
FADB_PSEFR Fu	169266 1,	GALE_HUMAN Fu	5893 nadi	1HZJ	1WDK	1I3K	0.79	Sc=6.70433, min distance = 2.02892
FADB_PSEFR Fu	4369002 C	LDHA_PIG Full	5893 nadi	9LDB	1WDK	9LDT	0.77	Sc=6.29069, min distance = 1.93470
FADB_PSEFR Fu	439153 Di	ADH1B_HUMAN Fu	5893 nadi	1U3U	1WDK	1DEH	0.79	Sc=5.99444, min distance = 1.71969
FADB_PSEFR Fu	439153 Di	GALE_ECOLI Fu	5893 nadi	1UDC	1WDK	1UDB	0.79	Sc=6.2748, min distance = 2.202539
FADB_PSEFR Fu	445794 AD	ADHX_HUMAN Fu	5893 nadi	2FZW	1WDK	2FZE		Sc=5.80777, min distance = 2.38148
FADB_PSEFR Fu	446050 CI	G3P_HUMAN Ful	5893 nadi	1U8F	1WDK	3GPD	0.95	Sc=6.28714, min distance = 1.66415
FADB_PSEFR Fu	446288 NA	G3P_PALVE Ful	5893 nadi	1DSS	1WDK	1IHX		Sc=6.79273, min distance = 0.78332
FADB_PSEFR Fu	6022 Ader	Q5SI02_THET8 I	5893 nadi	2BJK	1WDK	2BJA		Sc=6.19685, min distance = 2.16330
FADB_PSEFR Fu	6102710 A	Q4PRK9_PLAVI I	5893 nadi	2A92	1WDK	2AA3	0.95	Sc=5.99708, min distance = 1.71924
FADB_PSEFR Fu	9547960 A	NPD_THEMA Ful	5893 nadi	2H4H	1WDK	2H59		Sc=5.66375, min distance = 1.99050
FADH_ECOLI Fu	11987634	GLPD_ECOLI Fu	444188 CI	2QCU	1PS9	2R4E	0.97	Sc=6.39982, min distance = 2.31862
FADH_ECOLI Fu	11987634	TYTR_TRYCR Fu	444188 CI	1BZL	1PS9	1AOG	0.97	Sc=6.41314, min distance = 2.22690
FADH_ECOLI Fu	15942680	6PGD_LACLM Fu	5886 NADP	2IZ0	1PS9	2IZ1		Sc=6.31047, min distance = 0.84566
FADH_ECOLI Fu	16740985	FRDA_SHEFR Fu	444188 CI	1M64	1PS9	1E39	0.93	Sc=6.4018, min distance = 2.577268
FADH_ECOLI Fu	444502 CI	GSHR_HUMAN Fu	444188 CI	3DK9	1PS9	1BWC	0.98	Sc=6.4018, min distance = 2.355128
FADH_ECOLI Fu	446995 FM	CYB2_YEAST Fu	444243 FA	1KBI	1PS9	1LTD	0.77	Sc=6.15119, min distance = 2.04826
FADH_ECOLI Fu	448054 CI	FRDA_SHEFR Fu	444188 CI	1M64	1PS9	1Y0P	0.97	Sc=6.39874, min distance = 2.56201
FADH_PSEPU Fu	11987786	LDH_PLAFD Ful	5893 nadi	1T2D	1KOL	1T2E	0.77	Sc=6.78198, min distance = 2.51965
FADH_PSEPU Fu	439153 Di	ADH1B_HUMAN Fu	5893 nadi	1U3U	1KOL	1DEH	0.79	Sc=6.7933, min distance = 2.025108
FADH_PSEPU Fu	439153 Di	CTBP1_RAT Ful	5893 nadi	1HKU	1KOL	1HL3	0.79	Sc=6.78309, min distance = 2.08900
FADH_PSEPU Fu	440141 9i	G6PD_LEUME Fu	5893 nadi	1H94	1KOL	1E7Y		Sc=6.41376, min distance = 2.47258
FADH_PSEPU Fu	440516 CI	MDH_THETH Ful	5893 nadi	1BMD	1KOL	1BDM	0.79	Sc=6.79232, min distance = 2.11535
FADH_PSEPU Fu	445794 AD	ADHX_HUMAN Fu	5893 nadi	2FZW	1KOL	2FZE		Sc=6.63717, min distance = 2.12311
FADH_PSEPU Fu	6420113 C	MDH_THETH Ful	5893 nadi	1BMD	1KOL	1WZI	0.77	Sc=6.65767, min distance = 2.20353
FADL_ECOLI Fu	350021 Op	AOFB_HUMAN Fu	444279 1s	1OJD	1T16	1OJC		Sc=5.87515, min distance = 1.23119
FADL_ECOLI Fu	445070 fa	AOFB_HUMAN Fu	444279 1s	1OJD	1T16	2BK3		Sc=5.82254, min distance = 1.66582
FADL_ECOLI Fu	447661 2-	AOFB_HUMAN Fu	444279 1s	1OJD	1T16	1OJB		Sc=5.61343, min distance = 0.92801
FAEA_ASPNG Fu	19844 Met	XYNY_CLOTM Fu	445858 fe	1GKL	1UWC	1WB6	0.88	Sc=5.72254, min distance = 2.66778
FAEA_ASPNG Fu	70164 Met	XYNY_CLOTM Fu	445858 fe	1GKL	1UWC	1WB5	0.84	Sc=5.84265, min distance = 2.35396

FAK1_CHICK Fı	16040273	IRAK4_HUMAN Fı	33113 gar	2OID	2J0L	2OIC	30.77		Sc=6.43279, min distance = 1.35414(
FAK1_CHICK Fı	16058649	IGF1R_HUMAN Fı	33113 gar	1JQH	2J0L	2OJ9	41.7		Sc=6.46229, min distance = 2.15210]
FAK1_CHICK Fı	188966 dA	HSLU_ECOLI Fu]	33113 gar	1E94	2J0L	1G4A		0.97	Sc=5.85032, min distance = 1.74065
FAK1_CHICK Fı	24779674	CSK2A_MAIZE Fı	33113 gar	1LP4	2J0L	2PVH	23.79		Sc=5.99391, min distance = 2.34264
FAK1_CHICK Fı	3064778 H	ROCK1_HUMAN Fı	33113 gar	2V55	2J0L	2ETK	25.81		Sc=6.01669, min distance = 2.20905
FAK1_CHICK Fı	3547 Fasu	ROCK1_HUMAN Fı	33113 gar	2V55	2J0L	2ESM	25.81		Sc=5.88721, min distance = 2.66944
FAK1_CHICK Fı	36735 Gpp	CSK2A_MAIZE Fı	33113 gar	1LP4	2J0L	1DAY	23.79	0.8	Sc=5.97957, min distance = 2.05339
FAK1_CHICK Fı	444345 1c	IRAK4_HUMAN Fı	33113 gar	2OID	2J0L	2NRY	30.77		Sc=6.33838, min distance = 2.04670
FAK1_CHICK Fı	444367 CI	CSK2A_MAIZE Fı	33113 gar	1LP4	2J0L	1OM1	23.79		Sc=6.09998, min distance = 2.09121
FAK1_CHICK Fı	448043 2c	ROCK1_HUMAN Fı	33113 gar	2V55	2J0L	3D9V	25.81		Sc=5.90045, min distance = 2.58281
FAK1_CHICK Fı	5281701 T	PIM1_HUMAN Fu]	33113 gar	1XR1	2J0L	2O65	26.67		Sc=6.25732, min distance = 2.38326
FAK1_CHICK Fı	5287844 i	GSK3B_HUMAN Fı	33113 gar	1J1B	2J0L	1UV5	28.87		Sc=6.19336, min distance = 2.30488
FAK1_CHICK Fı	5326739 i	GSK3B_HUMAN Fı	33113 gar	1J1B	2J0L	1Q41	28.87		Sc=6.16603, min distance = 2.34918
FAK1_CHICK Fı	5327148 C	IPKA_RABIT Fu]	33113 gar	1CDK	2J0L	2ERZ			Sc=6.08504, min distance = 2.11533
FAK1_CHICK Fı	6083 aden	PIM1_HUMAN Fu]	33113 gar	1XR1	2J0L	1YXU	26.67	0.97	Sc=6.25878, min distance = 2.35637
FAK1_CHICK Fı	60961 ade	SKY1_YEAST Fu]	33113 gar	1Q99	2J0L	1Q97		0.93	Sc=6.15212, min distance = 2.25278
FAK1_CHICK Fı	60961 ade	STK6_HUMAN Fu]	33113 gar	2DWB	2J0L	1MUO	31.17	0.93	Sc=6.2204, min distance = 2.257281
FAK1_CHICK Fı	611002 Op	PIM1_HUMAN Fu]	33113 gar	1XR1	2J0L	1YXX	26.67		Sc=6.00356, min distance = 2.99194
FAK1_CHICK Fı	91532 AME	PURT_ECOLI Fu]	33113 gar	1EYZ	2J0L	1KJI		0.99	Sc=5.98685, min distance = 2.76846
FAK1_HUMAN Fı	11708454	KAPCA_BOVIN Fı	5957 Aden	1Q24	2IJM	2VNW	26.64		Sc=6.04274, min distance = 2.05823
FAK1_HUMAN Fı	1540 1h1c	CDK2_HUMAN Fu]	6022 Aden	1GY3	1MP8	1H1Q	31.79		Sc=6.41748, min distance = 1.41276
FAK1_HUMAN Fı	15602982	KAPCA_BOVIN Fı	5957 Aden	1Q24	2IJM	2UW6	26.64		Sc=6.03283, min distance = 2.03317
FAK1_HUMAN Fı	16122607	PDPK1_HUMAN Fı	5957 Aden	2BIY	2IJM	2PE0	23.95		Sc=6.15459, min distance = 2.21621
FAK1_HUMAN Fı	1707 1fvt	CDK2_HUMAN Fu]	6022 Aden	1GY3	1MP8	1FVT	31.79		Sc=6.20316, min distance = 2.63208
FAK1_HUMAN Fı	17754396	PDPK1_HUMAN Fı	5957 Aden	2BIY	2IJM	2R7B	23.95		Sc=6.29557, min distance = 2.25198
FAK1_HUMAN Fı	2398 bisi	PDPK1_HUMAN Fı	5957 Aden	2BIY	2IJM	1UU9	23.95		Sc=6.45307, min distance = 2.29605
FAK1_HUMAN Fı	2403 1uvr	PDPK1_HUMAN Fı	5957 Aden	2BIY	2IJM	1UVR	23.95		Sc=6.38962, min distance = 2.31914
FAK1_HUMAN Fı	2608 1jsv	CDK2_HUMAN Fu]	6022 Aden	1GY3	1MP8	1JSV	31.79		Sc=5.9417, min distance = 2.677947
FAK1_HUMAN Fı	36735 Gpp	Q9RVK2_DEIRA]	5957 Aden	1SU2	2IJM	1SZ3		0.8	Sc=6.05376, min distance = 1.65691
FAK1_HUMAN Fı	444852 CI	ATPB_BOVIN Fu]	5957 Aden	2V7Q	2IJM	1COW		0.91	Sc=6.39176, min distance = 2.13587
FAK1_HUMAN Fı	445840 di	CDK2_HUMAN Fu]	6022 Aden	1GY3	1MP8	1GII	31.79		Sc=6.28761, min distance = 2.26835
FAK1_HUMAN Fı	445841 py	CDK2_HUMAN Fu]	6022 Aden	1GY3	1MP8	1GIJ	31.79		Sc=6.45278, min distance = 2.45971
FAK1_HUMAN Fı	446704 LS	CDK2_HUMAN Fu]	6022 Aden	1GY3	1MP8	1KE6	31.79		Sc=6.32546, min distance = 2.68914
FAK1_HUMAN Fı	447649 1c	CDK2_HUMAN Fu]	6022 Aden	1GY3	1MP8	1OI9	31.79		Sc=6.43846, min distance = 1.89679
FAK1_HUMAN Fı	447652 In	CDK2_HUMAN Fu]	6022 Aden	1GY3	1MP8	1OIQ	31.79		Sc=6.19202, min distance = 2.32359
FAK1_HUMAN Fı	447654 CI	CDK2_HUMAN Fu]	6022 Aden	1GY3	1MP8	1OIT	31.79		Sc=6.26343, min distance = 2.77897
FAK1_HUMAN Fı	447656 1c	CDK2_HUMAN Fu]	6022 Aden	1GY3	1MP8	1OIY	31.79		Sc=6.54371, min distance = 1.51000

FAK1_HUMAN Fu	447766 1p	CDK2_HUMAN Ful	6022 Aden	1GY3	1MP8	1P2A	31.79		Sc=6.30498, min distance = 2.057945
FAK1_HUMAN Fu	447821 CI	CDK2_HUMAN Ful	6022 Aden	1GY3	1MP8	1PF8	31.79		Sc=6.13298, min distance = 2.231989
FAK1_HUMAN Fu	447962 1p	CDK2_HUMAN Ful	6022 Aden	1GY3	1MP8	2C5N	31.79		Sc=6.28728, min distance = 0.791696
FAK1_HUMAN Fu	448005 CI	GSK3B_HUMAN Fu	6022 Aden	1J1C	1MP8	1Q3W	29.89		Sc=6.28104, min distance = 2.146452
FAK1_HUMAN Fu	448310 CI	KIPN_BPT4 Ful	6022 Aden	1LTQ	1MP8	1RC8			Sc=5.74234, min distance = 2.258595
FAK1_HUMAN Fu	4565 1h1r	CDK2_HUMAN Ful	6022 Aden	1GY3	1MP8	1H1R	31.79		Sc=6.3641, min distance = 1.9827236
FAK1_HUMAN Fu	4566 1h1s	CDK2_HUMAN Ful	6022 Aden	1GY3	1MP8	1H1S	31.79		Sc=6.39063, min distance = 1.911822
FAK1_HUMAN Fu	5287844 i	GSK3B_HUMAN Fu	6022 Aden	1J1C	1MP8	1UV5	29.89		Sc=6.34059, min distance = 2.127553
FAK1_HUMAN Fu	5288708 1	CDK2_HUMAN Ful	6022 Aden	1GY3	1MP8	1KE5	31.79		Sc=6.12862, min distance = 2.803480
FAK1_HUMAN Fu	5288711 1	CDK2_HUMAN Ful	6022 Aden	1GY3	1MP8	1KE8	31.79		Sc=6.30438, min distance = 2.690109
FAK1_HUMAN Fu	5288712 1	CDK2_HUMAN Ful	6022 Aden	1GY3	1MP8	1KE9	31.79		Sc=6.32316, min distance = 2.599791
FAK1_HUMAN Fu	5326739 i	GSK3B_HUMAN Fu	6022 Aden	1J1C	1MP8	1Q41	29.89		Sc=6.30951, min distance = 2.333242
FAK1_HUMAN Fu	5327148 C	IPKA_RABIT Ful	6022 Aden	1JBP	1MP8	2ERZ			Sc=6.11441, min distance = 2.404428
FAK1_HUMAN Fu	6083 ader	ASSY_THET8 Ful	5957 Aden	1J1Z	2IJM	1J20		0.98	Sc=6.33257, min distance = 2.039299
FAK1_HUMAN Fu	6083 ader	PURP_PYRFU Ful	5957 Aden	2R86	2IJM	2R85		0.98	Sc=6.2626, min distance = 2.3375955
FAK1_HUMAN Fu	6420138 2	CDK2_HUMAN Ful	6022 Aden	1GY3	1MP8	2UUE	31.79		Sc=6.3641, min distance = 1.9937467
FAK1_HUMAN Fu	6539118 g	CDK2_HUMAN Ful	6022 Aden	1GY3	1MP8	1FVV	31.79		Sc=6.49098, min distance = 2.154814
FAK1_HUMAN Fu	6830 guar	Q381M1_9TRYP I	5957 Aden	2Q0D	2IJM	2Q0E		0.8	Sc=5.67569, min distance = 2.058687
FAK1_HUMAN Fu	72271 7-H	PDPK1_HUMAN Fu	5957 Aden	2BIY	2IJM	1OKZ	23.95		Sc=6.46449, min distance = 2.205874
FAK1_HUMAN Fu	91532 AME	BIOD_ECOLI Ful	5957 Aden	1A82	2IJM	1DAG		0.99	Sc=5.7014, min distance = 2.0224900
FAS1_YEAST Fu	16741044	PYRDA_LACLC Fu	444243 FA	1JUB	2UV8	1JRB			Sc=6.15622, min distance = 2.582937
FAS1_YEAST Fu	446995 FM	CYB2_YEAST Ful	444243 FA	1KBI	2UV8	1LTD		0.77	Sc=6.15567, min distance = 2.771641
FBPA_SERMA Fu	75791 Phe	SRC_HUMAN Full	311 citri	1O4L	1XVY	1O4P			Sc=5.71866, min distance = 2.772104
FBRL_HUMAN Fu	1103 sper	SPSY_HUMAN Ful	439176 Me	3C6K	2IPX	3C6M			Sc=5.91065, min distance = 2.014938
FBRL_HUMAN Fu	65482 sir	MTR1_RHOSH Ful	439176 Me	1EG2	2IPX	1NW6		0.8	Sc=5.76912, min distance = 2.324771
FBX2_MOUSE Fu	445948 D-	Q9HYN5_PSEAE I	185698 al	1OVS	2E33	1OXC			Sc=5.63307, min distance = 2.100765
FCGRN_RAT Ful	2249 ater	TRFL_BOVIN Ful	439353 be	2DWJ	3FRU	2NUV			Sc=5.93894, min distance = 2.563757
FCN2_HUMAN Fu	20544 ACE	ACES_MOUSE Ful	187 acety	2HA4	2J0H	2HA5			Sc=5.72978, min distance = 0.877706
FCN2_HUMAN Fu	79025 alp	MALE_ECOLI Ful	64960 Pol	1LAX	2J0Y	1ANF		0.89	Sc=5.70531, min distance = 1.949630
FCTA_OXAFO Fu	445939 1g	PPTA_ECOLI Ful	23831 HEP	1GYX	2VJQ	1GYY			Sc=5.67799, min distance = 2.116894
FCTA_OXAFO Fu	8200 TETR	Q8WSF8_APLCA I	23831 HEP	2BR7	2VJQ	2BYN			Sc=5.76955, min distance = 0.827580
FDHB_DESGI Fu	854023 Ep	Q8WSF8_APLCA I	23831 HEP	2BR7	1H0H	2BYQ			Sc=5.77975, min distance = 1.442371
FDHF_ECOLI Fu	445112 CI	DMSA_RHOCA Ful	4369050 P	1H5N	2IV2	1DMS	25.8		Sc=6.80852, min distance = 2.269767
FDHF_ECOLI Fu	445198 CI	DMSA_RHOCA Ful	4369050 P	1H5N	2IV2	1E61	25.8	0.98	Sc=6.80979, min distance = 2.185231
FDHF_ECOLI Fu	5326486 C	DMSA_RHOCA Ful	4369050 P	1H5N	2IV2	1DMR	25.8	0.98	Sc=6.80788, min distance = 2.346581
FDH_PSESR Ful	11987786	LDH_PLAFD Full	5893 nadi	1T2D	2NAD	1T2E		0.77	Sc=6.78354, min distance = 2.079092
FDH_PSESR Ful	439153 Di	ADH1B_HUMAN Fu	5893 nadi	1U3U	2NAD	1DEH		0.79	Sc=6.78586, min distance = 1.918817

FDH_PSESR Fu:	439153 Di	ADH1E_HORSE Fu	5893 nadi	1MGO	2NAD	2JHF	0.79	Sc=6.78376, min distance = 2.33318
FDH_PSESR Fu:	440516 CI	MDH_THETH Ful:	5893 nadi	1BMD	2NAD	1BDM	0.79	Sc=6.79448, min distance = 2.11813
FDH_PSESR Fu:	6102710 A	Q4PRK9_PLAVI I	5893 nadi	2A92	2NAD	2AA3	0.95	Sc=6.79345, min distance = 2.35229
FDNI_ECOLI Fu	11957353	HBB_HORSE Ful:	444098 HE	2D5X	1KQF	1IWH	0.83	Sc=6.67401, min distance = 2.17099
FDNI_ECOLI Fu	11957355	CY1_BOVIN Ful:	444098 HE	1L0N	1KQF	1L0L	0.77	Sc=6.67298, min distance = 2.13758
FDNI_ECOLI Fu	11957361	CY1_BOVIN Ful:	444098 HE	1L0N	1KQF	1NTZ	0.8	Sc=6.67038, min distance = 2.00703
FDNI_ECOLI Fu	11957363	HBB_HUMAN Ful:	444098 HE	1J40	1KQF	1RQA	0.77	Sc=6.72105, min distance = 2.00488
FDNI_ECOLI Fu	11957385	MYG_PHYCA Ful:	444098 HE	1A6M	1KQF	2CMM		Sc=6.34098, min distance = 2.11425
FDNI_ECOLI Fu	126994 CI	CCPR_YEAST Fu:	444098 HE	2EUT	1KQF	1Z53	0.77	Sc=6.66531, min distance = 2.29762
FDNI_ECOLI Fu	16741061	MYG_PHYCA Ful:	444098 HE	1A6M	1KQF	1MBC	0.83	Sc=6.66708, min distance = 2.04455
FDNI_ECOLI Fu	24883662	MYG_HORSE Ful:	444098 HE	2FRF	1KQF	1YMC	0.82	Sc=6.68176, min distance = 2.03848
FDNI_ECOLI Fu	5326621 C	FRDD_ECOLI Fu:	1561 HQNC	1KF6	1KQG	1L0V		Sc=6.09262, min distance = 1.82888
FECA_ECOLI Fu	11957399	PYRB_ECOLI Fu:	311 citri	1R0B	1PO0	2H3E		Sc=6.05137, min distance = 1.70162
FECA_ECOLI Fu	11987742	LHA4_RHOAC Fu:	444279 1s	2FKW	1KMO	1NKZ		Sc=6.38863, min distance = 0.77795
FECA_ECOLI Fu	12136 Ber	POL_HV1N5 Ful:	311 citri	2GON	1PO0	9HVP		Sc=5.66676, min distance = 0.79025
FECA_ECOLI Fu	12793 Phe	SRC_HUMAN Ful:	311 citri	1O4L	1PO0	1O4O		Sc=5.70866, min distance = 0.89791
FECA_ECOLI Fu	16750041	PYRB_ECOLI Fu:	311 citri	1R0B	1PO0	2IPO		Sc=6.04197, min distance = 1.70746
FECA_ECOLI Fu	439183 1i	TPIS_TRYBB Fu:	311 citri	2VEK	1PO0	1IIH		Sc=5.61752, min distance = 1.91776
FECA_ECOLI Fu	447530	SRC_HUMAN Ful:	311 citri	1O4L	1PO0	1O4F		Sc=5.92764, min distance = 0.59672
FECA_ECOLI Fu	447550 ph	TPIS_TRYBB Fu:	311 citri	2VEK	1PO0	4TIM		Sc=5.61286, min distance = 1.81720
FECA_ECOLI Fu	51 2-Oxop	SERA_ECOLI Fu:	311 citri	2P9E	1PO0	1YBA	0.76	Sc=5.61763, min distance = 2.09313
FECA_ECOLI Fu	5354052 p	MIF_HUMAN Ful:	311 citri	1GD0	1PO0	2OOZ		Sc=5.8421, min distance = 0.700626
FECA_ECOLI Fu	8778 Laur	CTXA3_NAJAT Fu	311 citri	1XT3	1PO0	1H0J		Sc=5.75354, min distance = 0.57487
FENR1_PEA Fu:	165230 1a	NCPR_RAT Full=	444188 CI	1JA1	1QFZ	1AMO		Sc=6.49156, min distance = 1.86919
FENR_THET8 Fu	16740985	FRDA_SHEFR Fu:	444188 CI	1M64	2ZBW	1E39	0.93	Sc=6.87944, min distance = 1.92090
FENR_THET8 Fu	444502 CI	FRDA_SHEFR Fu:	444188 CI	1M64	2ZBW	1QJD	0.98	Sc=6.87944, min distance = 1.98584
FENR_THET8 Fu	446013 1,	O28603_ARCFU I	444188 CI	1JNR	2ZBW	1JNZ	0.94	Sc=6.87927, min distance = 1.80928
FES_HUMAN Fu:	10224714	CDK2_HUMAN Fu:	444345 1c	1AQ1	3CBL	3DDP		Sc=6.44119, min distance = 2.14838
FES_HUMAN Fu:	11348631	CHK1_HUMAN Fu:	444345 1c	1NVR	3CBL	2E9U		Sc=6.28597, min distance = 2.14294
FES_HUMAN Fu:	11553058	LCK_HUMAN Ful:	444345 1c	1QPD	3CBL	2OF2		Sc=6.29144, min distance = 2.37716
FES_HUMAN Fu:	11696113	KAPCA_BOVIN Fu	444345 1c	1STC	3CBL	2VO3		Sc=6.30948, min distance = 2.37813
FES_HUMAN Fu:	11992146	MAPK2_HUMAN Fu	444345 1c	2PZY	3CBL	2P3G		Sc=6.29397, min distance = 2.40932
FES_HUMAN Fu:	15602982	KAPCA_BOVIN Fu	444345 1c	1STC	3CBL	2UW6		Sc=6.04691, min distance = 2.52917
FES_HUMAN Fu:	15991572	LCK_HUMAN Ful:	444345 1c	1QPD	3CBL	2OF4		Sc=6.25941, min distance = 2.65928
FES_HUMAN Fu:	160355 rc	CDK2_HUMAN Fu:	444345 1c	1AQ1	3CBL	3DDQ		Sc=6.25962, min distance = 2.46356
FES_HUMAN Fu:	16058647	PIM1_HUMAN Fu:	444345 1c	1YHS	3CBL	2OI4	0.75	Sc=6.43611, min distance = 2.37436
FES_HUMAN Fu:	16122608	PDPK1_HUMAN Fu	444345 1c	1OKY	3CBL	2PE1		Sc=6.32207, min distance = 2.68072

FES_HUMAN Fu 16122643	CHK1_HUMAN Fu 444345 1c 1NVR	3CBL	2YWP			Sc=6.32069, min distance = 2.12873
FES_HUMAN Fu 16129574	CHK1_HUMAN Fu 444345 1c 1NVR	3CBL	2HXL			Sc=6.1103, min distance = 2.175774
FES_HUMAN Fu 16214823	CDK2_HUMAN Fu 444345 1c 1AQ1	3CBL	2UZB			Sc=6.08119, min distance = 2.37221
FES_HUMAN Fu 16214825	CDK2_HUMAN Fu 444345 1c 1AQ1	3CBL	2UZE			Sc=6.25806, min distance = 2.04437
FES_HUMAN Fu 16214826	CDK2_HUMAN Fu 444345 1c 1AQ1	3CBL	2UZL			Sc=6.12146, min distance = 2.32266
FES_HUMAN Fu 16214827	CDK2_HUMAN Fu 444345 1c 1AQ1	3CBL	2UZN			Sc=6.07249, min distance = 2.19371
FES_HUMAN Fu 16214828	CDK2_HUMAN Fu 444345 1c 1AQ1	3CBL	2UZO			Sc=6.08183, min distance = 2.20185
FES_HUMAN Fu 16758227	CHK1_HUMAN Fu 444345 1c 1NVR	3CBL	2R0U			Sc=6.54469, min distance = 2.14484
FES_HUMAN Fu 1707 1fvt	CDK2_HUMAN Fu 444345 1c 1AQ1	3CBL	1FVT			Sc=6.09637, min distance = 2.36594
FES_HUMAN Fu 17754396	PDPK1_HUMAN Fu 444345 1c 1OKY	3CBL	2R7B			Sc=6.27663, min distance = 2.69084
FES_HUMAN Fu 1907917 2	CHK1_HUMAN Fu 444345 1c 1NVR	3CBL	2CGW			Sc=6.1515, min distance = 2.711310
FES_HUMAN Fu 23653515	CDK2_HUMAN Fu 444345 1c 1AQ1	3CBL	2R3F			Sc=5.99449, min distance = 2.67345
FES_HUMAN Fu 23653516	CDK2_HUMAN Fu 444345 1c 1AQ1	3CBL	2R3G			Sc=6.19328, min distance = 2.61857
FES_HUMAN Fu 23653519	CDK2_HUMAN Fu 444345 1c 1AQ1	3CBL	2R3K			Sc=6.18861, min distance = 2.51924
FES_HUMAN Fu 23653521	CDK2_HUMAN Fu 444345 1c 1AQ1	3CBL	2R3M			Sc=6.34295, min distance = 2.50156
FES_HUMAN Fu 23653524	CDK2_HUMAN Fu 444345 1c 1AQ1	3CBL	2R3P			Sc=6.2656, min distance = 2.024239
FES_HUMAN Fu 2398 bisi	PDPK1_HUMAN Fu 444345 1c 1OKY	3CBL	1UU9			Sc=6.44163, min distance = 2.07043
FES_HUMAN Fu 2403 1uvr	PDPK1_HUMAN Fu 444345 1c 1OKY	3CBL	1UVR			Sc=6.37778, min distance = 2.12271
FES_HUMAN Fu 24752838	CHK1_HUMAN Fu 444345 1c 1NVR	3CBL	2QHM			Sc=6.05296, min distance = 2.95609
FES_HUMAN Fu 24901723	CDK2_HUMAN Fu 444345 1c 1AQ1	3CBL	2W06			Sc=6.27438, min distance = 2.30785
FES_HUMAN Fu 24905143	PK3CG_HUMAN Fu 444345 1c 1E8Z	3CBL	2V4L			Sc=6.10282, min distance = 2.49856
FES_HUMAN Fu 24905144	PK3CG_HUMAN Fu 444345 1c 1E8Z	3CBL	3ENE			Sc=6.09286, min distance = 2.34697
FES_HUMAN Fu 24916751	CDK2_HUMAN Fu 444345 1c 1AQ1	3CBL	3DOG			Sc=6.25043, min distance = 2.39368
FES_HUMAN Fu 25021197	CDK2_HUMAN Fu 444345 1c 1AQ1	3CBL	3EOC			Sc=6.20553, min distance = 2.71323
FES_HUMAN Fu 33113 gam	IRAK4_HUMAN Fu 444345 1c 2NRY	3CBL	2OID			Sc=5.67546, min distance = 2.20414
FES_HUMAN Fu 4369136 C	CDK2_HUMAN Fu 444345 1c 1AQ1	3CBL	1DM2			Sc=6.23092, min distance = 2.96402
FES_HUMAN Fu 4369435 2	CHK1_HUMAN Fu 444345 1c 1NVR	3CBL	2BRO			Sc=6.41049, min distance = 2.03283
FES_HUMAN Fu 446704 LS	CDK2_HUMAN Fu 444345 1c 1AQ1	3CBL	1KE6			Sc=6.25949, min distance = 2.35560
FES_HUMAN Fu 447654 CI	CDK2_HUMAN Fu 444345 1c 1AQ1	3CBL	1OIT			Sc=6.24576, min distance = 2.19395
FES_HUMAN Fu 447655 1c	CDK2_HUMAN Fu 444345 1c 1AQ1	3CBL	1OIU			Sc=6.25032, min distance = 2.57237
FES_HUMAN Fu 447962 1p	CDK2_HUMAN Fu 444345 1c 1AQ1	3CBL	2C5N			Sc=6.21, min distance = 2.65868632
FES_HUMAN Fu 447967 CI	CDK2_HUMAN Fu 444345 1c 1AQ1	3CBL	1PYE			Sc=6.34036, min distance = 2.37790
FES_HUMAN Fu 448043 2g	KAPCA_BOVIN Fu 444345 1c 1STC	3CBL	1Q8U			Sc=5.94415, min distance = 2.71952
FES_HUMAN Fu 448293 CI	CDK2_HUMAN Fu 444345 1c 1AQ1	3CBL	1R78			Sc=6.43466, min distance = 2.56952
FES_HUMAN Fu 449240 1y	KAPCA_BOVIN Fu 444345 1c 1STC	3CBL	1YDR			Sc=5.88721, min distance = 2.54385
FES_HUMAN Fu 456214 pu	KS6A1_HUMAN Fu 444345 1c 2Z7R	3CBL	2Z7S			Sc=6.3465, min distance = 2.003562
FES_HUMAN Fu 4565 1h1r	CDK2_HUMAN Fu 444345 1c 1AQ1	3CBL	1H1R			Sc=6.22266, min distance = 2.60603

FES_HUMAN Fu	4566 1h1s	CDK2_HUMAN Fu	444345 1c	1AQ1	3CBL	1H1S		Sc=6.27232, min distance = 2.37119
FES_HUMAN Fu	5288016 6	CDK2_HUMAN Fu	444345 1c	1AQ1	3CBL	2B53		Sc=6.01358, min distance = 2.01470
FES_HUMAN Fu	5288708 1	CDK2_HUMAN Fu	444345 1c	1AQ1	3CBL	1KE5		Sc=6.11139, min distance = 2.50830
FES_HUMAN Fu	5288710 C	CDK2_HUMAN Fu	444345 1c	1AQ1	3CBL	1KE7		Sc=6.31516, min distance = 2.53897
FES_HUMAN Fu	5327121 I	PIM1_HUMAN Fu	444345 1c	1YHS	3CBL	2C3I		Sc=6.20648, min distance = 2.51010
FES_HUMAN Fu	5327123 I	CHK1_HUMAN Fu	444345 1c	1NVR	3CBL	2C3L		Sc=6.11735, min distance = 2.50807
FES_HUMAN Fu	5331010 c	CDK2_HUMAN Fu	444345 1c	1AQ1	3CBL	2BKZ		Sc=6.28057, min distance = 2.48256
FES_HUMAN Fu	5687 1di8	CDK2_HUMAN Fu	444345 1c	1AQ1	3CBL	1DI8		Sc=6.23509, min distance = 2.29113
FES_HUMAN Fu	5957 Aden	PDPK1_HUMAN F	444345 1c	1OKY	3CBL	2BIY		Sc=5.99503, min distance = 2.10172
FES_HUMAN Fu	60961 ade	PIM1_HUMAN Fu	444345 1c	1YHS	3CBL	1YI4		Sc=6.15397, min distance = 2.98096
FES_HUMAN Fu	6102670	CDK2_HUMAN Fu	444345 1c	1AQ1	3CBL	1YKR		Sc=6.26803, min distance = 2.20535
FES_HUMAN Fu	6420139 C	CDK2_HUMAN Fu	444345 1c	1AQ1	3CBL	2C5V		Sc=6.20726, min distance = 2.33539
FES_HUMAN Fu	6539118 g	CDK2_HUMAN Fu	444345 1c	1AQ1	3CBL	1FVV		Sc=6.42757, min distance = 2.21991
FES_HUMAN Fu	6540255 C	DAPK1_HUMAN F	444345 1c	1WVY	3CBL	1WVX	0.78	Sc=6.27569, min distance = 1.75401
FES_HUMAN Fu	6540273 2	CHK1_HUMAN Fu	444345 1c	1NVR	3CBL	2CGV		Sc=6.13938, min distance = 2.44612
FES_HUMAN Fu	656971 CI	CDK2_HUMAN Fu	444345 1c	1AQ1	3CBL	1Y8Y		Sc=6.0102, min distance = 2.344734
FES_HUMAN Fu	6852207 M	KAPCA_BOVIN F	444345 1c	1STC	3CBL	2GNI		Sc=5.90002, min distance = 2.44671
FES_HUMAN Fu	72271 7-H	CDK2_HUMAN Fu	444345 1c	1AQ1	3CBL	1PKD	0.91	Sc=6.44412, min distance = 2.63020
FES_HUMAN Fu	9817550 V	CDK2_HUMAN Fu	444345 1c	1AQ1	3CBL	3BHV		Sc=6.35818, min distance = 2.42750
FET3_YEAST F	101798 Me	LEC1_LATOC Fu	185698 al	1LOG	1ZPU	1LOB	0.94	Sc=5.7646, min distance = 2.636934
FET3_YEAST F	15133 m-X	CARP_YEAST Fu	439680 be	1DPJ	1ZPU	1FQ5		Sc=5.81494, min distance = 0.95148
FET3_YEAST F	64947 alp	LEC1_LATOC Fu	185698 al	1LOG	1ZPU	1LOA	0.94	Sc=5.77694, min distance = 2.41102
FGFR2_HUMAN	33113 gam	KIF1A_MOUSE F	91532 AMP	1I6I	2PVF	1VFV	0.99	Sc=5.63686, min distance = 2.36270
FGFR2_HUMAN	444564 AD	BIOD_ECOLI Fu	91532 AMP	1DAG	2PVF	1BS1	0.91	Sc=6.3438, min distance = 2.345711
FGFR2_HUMAN	5957 Aden	BIOD_ECOLI Fu	91532 AMP	1DAG	2PVF	1A82	0.99	Sc=5.64738, min distance = 2.35371
FGFR2_HUMAN	6022 Aden	BIOD_ECOLI Fu	91532 AMP	1DAG	2PVF	1DAD	0.99	Sc=6.35458, min distance = 2.54551
FIBB_PETMA F	446578 1r	Q9HYN5_PSEAE	82313 1rd	1W8H	1LWU	2JDP		Sc=5.60974, min distance = 1.99880
FIBG_HUMAN F	115260 S-	GSTM2_HUMAN F	124886 gl	1XW5	1DUG	2AB6	0.94	Sc=5.85654, min distance = 2.24085
FIBG_HUMAN F	449471 GG	GSTT2_HUMAN F	124886 gl	1LJR	1DUG	3LJR		Sc=6.15115, min distance = 2.21347
FIBG_HUMAN F	5288475 C	GSTM2_HUMAN F	124886 gl	1XW5	1DUG	2C4J		Sc=5.75299, min distance = 2.42175
FIBG_HUMAN F	97536 Hex	GSTP_ONCVO Fu	124886 gl	1TU7	1DUG	1TU8	0.9	Sc=6.45919, min distance = 2.41102
FIMH_ECOLI F	151504 D-	CONA_CANEN Fu	101798 Me	5CNA	1KIU	1I3H		Sc=5.77455, min distance = 2.28078
FIMH_ECOLI F	151504 D-	CONA_CANEN Fu	185698 al	1CVN	2VCO	1I3H		Sc=5.90421, min distance = 0.38280
FIMH_ECOLI F	151504 D-	CVN_NOSEL Ful	185698 al	2RDK	2VCO	2PYS		Sc=5.78164, min distance = 2.48377
FIMH_ECOLI F	16740986	MYRA_SINAL Fu	185698 al	1E4M	2VCO	1E70		Sc=5.76928, min distance = 2.36054
FIMH_ECOLI F	16741034	Q9XEI3_HORVD	185698 al	1X38	2VCO	1IEV	0.82	Sc=5.71966, min distance = 2.05756
FIMH_ECOLI F	439554 fu	Q9HYN5_PSEAE	101798 Me	2JDN	1KIU	1UZV	0.96	Sc=5.91235, min distance = 0.91268

FIMH_ECOLI Fu	439680 be	Q9HYN5_PSEAE I	101798 Me	2JDN	1KIU	1OUR		0.94	Sc=5.89111, min distance = 0.91590
FIMH_ECOLI Fu	445184 1,	Q6P5R5_HUMAN I	185698 al	1OP3	2VCO	1ZLW		0.89	Sc=5.7552, min distance = 2.331204
FIMH_ECOLI Fu	445184 1,	Q6PYX1_HUMAN I	185698 al	1ZLS	2VCO	1ZLW		0.89	Sc=5.76928, min distance = 2.08560
FIMH_ECOLI Fu	447053 Cl	Q9HYN5_PSEAE I	101798 Me	2JDN	1KIU	1W8H		0.81	Sc=5.80266, min distance = 0.94220
FIMH_ECOLI Fu	447147 Ab	TSPE_BPP22 Fu	185698 al	1TYU	2VCO	1TYX		0.98	Sc=5.60659, min distance = 1.24489
FIMH_ECOLI Fu	64947 alp	LEC1_LATOC Fu	185698 al	1LOG	2VCO	1LOA		0.94	Sc=5.82768, min distance = 2.30679
FIMH_ECOLI Fu	82313 1ro	MBL1_RAT Full-	101798 Me	1KWU	1KIU	1KWV			Sc=6.13925, min distance = 1.04135
FIMH_ECOLI Fu	87330 M97	CONA_CANEN Fu	101798 Me	5CNA	1KIU	1CJP			Sc=6.29946, min distance = 1.71023
FIMH_ECOLI Fu	92969 PNE	CONA_CANEN Fu	185698 al	1CVN	2VCO	1VAL			Sc=6.28523, min distance = 2.39319
FIXL_BRAJA Fu	11957330	CCPR_YEAST Fu	444522 HE	3E2O	1XJ3	1BEQ		0.84	Sc=6.64276, min distance = 2.22468
FIXL_BRAJA Fu	11957353	HBA_HORSE Full	444098 HE	2D5X	2VV6	1IWH		0.83	Sc=6.6579, min distance = 2.065792
FIXL_BRAJA Fu	11957353	HBA_HORSE Full	444124 HE	1Y8I	1XJ4	1IWH		0.87	Sc=6.63995, min distance = 2.00632
FIXL_BRAJA Fu	11957353	HBA_HORSE Full	444522 HE	1Y8K	1XJ3	1IWH		0.87	Sc=6.64842, min distance = 2.11156
FIXL_BRAJA Fu	11957353	HBB_HORSE Full	444124 HE	1Y8I	1XJ4	1IWH		0.87	Sc=6.63561, min distance = 2.06038
FIXL_BRAJA Fu	11957355	CYB_BOVIN Full	444522 HE	1BGY	1XJ3	1L0L		0.81	Sc=6.63466, min distance = 2.14844
FIXL_BRAJA Fu	11957361	CY1_BOVIN Full	444098 HE	1L0N	2VV6	1NTZ		0.8	Sc=6.64734, min distance = 2.29589
FIXL_BRAJA Fu	11957361	CYB_BOVIN Full	444522 HE	1BGY	1XJ3	1NTZ		0.84	Sc=6.63703, min distance = 1.27705
FIXL_BRAJA Fu	11957363	HBA_HUMAN Full	444522 HE	2DN1	1XJ3	1RQA		0.81	Sc=6.63278, min distance = 2.09560
FIXL_BRAJA Fu	11957363	HBB_HUMAN Full	444098 HE	1J40	2VV6	1RQA		0.77	Sc=6.67097, min distance = 1.94179
FIXL_BRAJA Fu	11957363	HBB_HUMAN Full	444124 HE	1NQP	1XJ4	1RQA		0.81	Sc=6.6404, min distance = 1.928217
FIXL_BRAJA Fu	11957363	HBB_HUMAN Full	444522 HE	2DN1	1XJ3	1RQA		0.81	Sc=6.65415, min distance = 2.05903
FIXL_BRAJA Fu	11957364	HMOX1_HUMAN Fu	444124 HE	1OZW	1XJ4	1S13		0.86	Sc=6.4716, min distance = 1.791957
FIXL_BRAJA Fu	11957370	HMOX1_HUMAN Fu	444124 HE	1OZW	1XJ4	1TWN			Sc=6.38489, min distance = 1.73234
FIXL_BRAJA Fu	11957371	HMOX1_HUMAN Fu	444124 HE	1OZW	1XJ4	1TWR			Sc=6.39611, min distance = 1.36689
FIXL_BRAJA Fu	11957385	MYG_PHYCA Full	444098 HE	1A6M	2VV6	2CMM			Sc=5.93347, min distance = 2.57261
FIXL_BRAJA Fu	11957385	MYG_PHYCA Full	444124 HE	1U7R	1XJ4	2CMM			Sc=5.86711, min distance = 2.44060
FIXL_BRAJA Fu	11957385	MYG_PHYCA Full	444522 HE	1VXD	1XJ3	2CMM			Sc=5.89468, min distance = 2.36734
FIXL_BRAJA Fu	11970219	CCPR_YEAST Fu	444522 HE	3E2O	1XJ3	1BEM		0.87	Sc=6.6404, min distance = 2.446878
FIXL_BRAJA Fu	11970220	CCPR_YEAST Fu	444522 HE	3E2O	1XJ3	1BEP		0.83	Sc=6.68592, min distance = 2.33469
FIXL_BRAJA Fu	11970221	CCPR_YEAST Fu	444522 HE	3E2O	1XJ3	1BES		0.87	Sc=6.64365, min distance = 2.38745
FIXL_BRAJA Fu	11970242	CCPR_YEAST Fu	444522 HE	3E2O	1XJ3	1CPE		0.87	Sc=6.6395, min distance = 2.232820
FIXL_BRAJA Fu	16741062	MYG_PHYCA Full	444098 HE	1A6M	2VV6	1MBN		0.84	Sc=6.66008, min distance = 1.93208
FIXL_BRAJA Fu	4369228 C	PER_COPCI Full	444124 HE	1LY9	1XJ4	1LYC		0.95	Sc=6.64075, min distance = 1.94841
FIXL_BRAJA Fu	444207 He	PETD_MASLA Fu	444522 HE2E76		1XJ3	1VF5	1		Sc=6.64605, min distance = 1.57254
FIXL_BRAJA Fu	446332 Cl	MYG_PHYCA Full	444098 HE	1A6M	2VV6	1IOP		0.82	Sc=6.57357, min distance = 1.65089
FIXL_BRAJA Fu	446409 HN	HBA_HUMAN Full	444522 HE	2DN1	1XJ3	1J40		0.98	Sc=6.64701, min distance = 2.10071
FIXL_BRAJA Fu	6102730 C	PGH1_SHEEP Fu	444522 HE	1CQE	1XJ3	2AYL		0.98	Sc=6.64219, min distance = 2.06304

FIXL_RHIME F	11957353	HBB_HORSE Ful	444124 HE	1Y8I	1D06	1IWH		0.87	Sc=6.58469, min distance = 2.136648
FIXL_RHIME F	11957364	HMOX1_HUMAN F	444124 HE	1OZW	1D06	1S13		0.86	Sc=6.48542, min distance = 1.638334
FIXL_RHIME F	11957371	HMOX1_HUMAN F	444124 HE	1OZW	1D06	1TWR			Sc=6.42581, min distance = 2.180931
FIXL_RHIME F	11957385	MYG_PHYCA Ful	444124 HE	1U7R	1D06	2CMM			Sc=5.90847, min distance = 2.745711
FIXL_RHIME F	16214774	CP51_MYCTU Fu	444124 HE	2CIB	1D06	2CI0		0.95	Sc=6.31549, min distance = 2.445757
FIXL_RHIME F	16741183	CPXA_PSEPU Fu	444124 HE	1RE9	1D06	2ZAW		0.92	Sc=6.29341, min distance = 2.491082
FIXL_RHIME F	444207 He	PER_COPCI Ful	444124 HE	1LY9	1D06	1LY8		0.99	Sc=6.65986, min distance = 2.010677
FIXL_RHIME F	444522 HE	HBA_PAGBE Ful	444124 HE	1S5X	1D06	1PBX		0.99	Sc=6.663, min distance = 2.4688092
FIXL_RHIME F	446189 CI	HBG1_HUMAN Fu	444124 HE	1I3E	1D06	1I3D		0.89	Sc=6.66508, min distance = 2.182707
FLAV_ANASO F	449551 FM	FLAV_DESVH Fu	444243 FA	1F4P	1OBO	5FX2		0.77	Sc=6.36226, min distance = 2.201300
FLAV_AZOVI F	449551 FM	FLAV_DESVH Fu	444243 FA	1F4P	1YOB	5FX2		0.77	Sc=6.35943, min distance = 2.201316
FLAV_CHOCR F	449551 FM	FLAV_DESVH Fu	444243 FA	1F4P	2FCR	5FX2		0.77	Sc=6.21676, min distance = 2.445021
FLAV_DESVH F	5326566 I	Q9HPW4_HALSA I	444658 2a	2CCB	1BU5	2CC6		0.85	Sc=6.3109, min distance = 2.2145801
FLAV_DESVH F	6323394 C	RIB4_SCHPO Fu	444658 2a	1KYV	1BU5	2A59			Sc=5.90484, min distance = 1.979456
FLAV_ECOLI F	449551 FM	FLAV_DESVH Fu	444243 FA	1F4P	1AG9	5FX2		0.77	Sc=6.69342, min distance = 2.428454
FLAV_HELPJ F	5326566 I	BLVRB_HUMAN F	444243 FA	1HE4	1FUE	1HE5		0.82	Sc=6.28716, min distance = 2.101260
FLAV_SYNE7 F	449551 FM	FLAV_DESVH Fu	444243 FA	1F4P	1CZN	5FX2		0.77	Sc=6.68297, min distance = 2.370881
FLHF_BACSU F	444845 1d	RASH_HUMAN Fu	36735 Gpp	1CTQ	2PX0	1CLU		0.99	Sc=6.18246, min distance = 2.357096
FLHF_BACSU F	444845 1d	RASH_HUMAN Fu	6830 guan	2CL7	2PX3	1CLU		0.98	Sc=6.08694, min distance = 2.175405
FLHF_BACSU F	446248 CI	RASH_HUMAN Fu	36735 Gpp	1CTQ	2PX0	1IAQ			Sc=6.00313, min distance = 2.071266
FLHF_BACSU F	60961 ade	NUD16_XENLA F	6830 guan	2A8S	2PX3	2A8T			Sc=5.90268, min distance = 2.361401
FLHF_BACSU F	93082 Gab	FTSY_THEAQ Fu	36735 Gpp	2J7P	2PX0	1RJ9		0.99	Sc=6.21264, min distance = 2.354384
FLHF_BACSU F	93082 Gab	RASH_HUMAN Fu	36735 Gpp	1CTQ	2PX0	121P		0.99	Sc=6.1855, min distance = 2.1728474
FLHF_BACSU F	93082 Gab	RASH_HUMAN Fu	6830 guan	2CL7	2PX3	121P		0.99	Sc=6.08303, min distance = 2.241663
FLII_SALTY F	440317 AT	FTSK_PSEAE Fu	6022 Aden	2IUU	2DPY	2IUT		0.99	Sc=5.80022, min distance = 2.037336
FLII_SALTY F	5957 Aden	MUTS_ECOLI Fu	6022 Aden	1OH7	2DPY	1W7A		0.99	Sc=5.83407, min distance = 2.152695
FLPA_ARCFU F	16741210	MTTA_THEAQ Fu	34756 Acy	2ADM	1NT2	2JG3			Sc=5.94219, min distance = 2.149685
FLPA_ARCFU F	445762 5'	MTTA_THEAQ Fu	34756 Acy	2ADM	1NT2	2IH2		0.93	Sc=6.32974, min distance = 2.795806
FLPA_ARCFU F	445971 CI	COMT_RAT Full	34756 Acy	2CL5	1NT2	1H1D		0.92	Sc=6.51645, min distance = 2.609280
FMNB_DESVM F	5326566 I	BLVRB_HUMAN F	444243 FA	1HE4	1FLM	1HE5		0.82	Sc=6.24987, min distance = 2.031874
FMO1_SCHPO F	11987634	GLPD_ECOLI Fu	444188 CI	2QCU	2GV8	2R4E		0.97	Sc=5.96555, min distance = 1.979487
FMO1_SCHPO F	16741253	LSD1_HUMAN Fu	444188 CI	2DW4	2GV8	2UXN		0.95	Sc=5.97846, min distance = 2.217892
FMO1_SCHPO F	444502 CI	GSHR_HUMAN Fu	444188 CI	3DK9	2GV8	1BWC		0.98	Sc=5.96357, min distance = 2.188595
FMO1_SCHPO F	444502 CI	TYTR_TRYCR Fu	444188 CI	1BZL	2GV8	1GXF		0.98	Sc=5.9745, min distance = 2.2756998
FMS1_YEAST F	16741253	LSD1_HUMAN Fu	444188 CI	2DW4	1RSG	2UXN	42.68	0.95	Sc=6.88783, min distance = 2.477751
FMS1_YEAST F	444502 CI	GSHR_HUMAN Fu	444188 CI	3DK9	1RSG	1BWC		0.98	Sc=6.44287, min distance = 2.401178
FMS1_YEAST F	444502 CI	TYTR_TRYCR Fu	444188 CI	1BZL	1RSG	1GXF		0.98	Sc=6.44226, min distance = 2.157060

FMS1_YEAST F	446013 1,	O28603_ARCFU	444188 CI	1JNR	1RSG	1JNZ		0.94	Sc=6.44848, min distance = 2.22457
FMS1_YEAST F	449465 CI	GSHR_HUMAN Fu	444188 CI	3DK9	1RSG	3GRT		0.98	Sc=6.44053, min distance = 2.06291
FMS1_YEAST F	6420174 C	O28603_ARCFU	444188 CI	1JNR	1RSG	2FJB		0.92	Sc=6.45303, min distance = 1.96921
FOLB1_ARATH	446357 7-	TGT_ZYMMO Ful	764 guani	2PWU	1SQL	1P0B			Sc=5.83103, min distance = 2.80715
FOLB1_ARATH	447262 CI	TGT_ZYMMO Ful	764 guani	2PWU	1SQL	2Z1W		0.76	Sc=6.01933, min distance = 2.24850
FOLB1_ARATH	8646 Guar	PNPH_HUMAN Fu	764 guani	1V2H	1SQL	1V41			Sc=5.65876, min distance = 2.65078
FOLB_MYCTU F	448839 ne	FOLB_STAAU Fu	69736 Ran	2DHN	1NBU	2NM2		0.83	Sc=6.1836, min distance = 1.591
FOLB_MYCTU F	95034 8-A	FOLB_STAAU Fu	69736 Ran	2DHN	1NBU	1RS2			Sc=5.79879, min distance = 2.71755
FOLC_ECOLI F	446090 1h	NDKC_DICDI Fu	6022 Aden	1KDN	1W78	1HIY		0.97	Sc=6.46179, min distance = 1.88864
FOLC_ECOLI F	447916 ac	RIO1_ARCFU Fu	6022 Aden	1ZTH	1W78	1ZTF		0.95	Sc=6.22742, min distance = 2.19732
FOLC_ECOLI F	447955 1p	CDK2_HUMAN Fu	6022 Aden	1GY3	1W78	1PXI			Sc=5.81757, min distance = 2.45871
FOLC_ECOLI F	6031 Uric	O33839_THEMA	6022 Aden	1XJK	1W78	1XJG			Sc=5.934, min distance = 2.0456778
FOLC_ECOLI F	6083 ader	HSP71_HUMAN F	6022 Aden	1S3X	1W78	1XQS		0.99	Sc=6.40119, min distance = 2.50525
FOLC_ECOLI F	6083 ader	PSPF_ECOLI Fu	6022 Aden	2C98	1W78	2VII		0.99	Sc=6.38744, min distance = 2.17188
FOLC_ECOLI F	6852187 2	O57883_PYRHO	6022 Aden	1WNL	1W78	2DTH		0.89	Sc=6.16206, min distance = 1.47510
FOLC_ECOLI F	8977 1dar	O33839_THEMA	6022 Aden	1XJK	1W78	1XJE		0.8	Sc=6.49503, min distance = 2.21209
FOLH1_HUMAN	33032 L-g	GRIA2_RAT Ful	40539 Qui	1P1O	2OR4	2GFE			Sc=5.69379, min distance = 2.50185
FOLH1_HUMAN	33032 L-g	GRIK2_RAT Ful	40539 Qui	1S9T	2OR4	1S50			Sc=5.68485, min distance = 2.54280
FOLH1_HUMAN	440053 Wi	GRIA2_RAT Ful	40539 Qui	1P1O	2OR4	1MQJ			Sc=6.02521, min distance = 2.38773
FOLH1_HUMAN	447195 1m	GRIA2_RAT Ful	40539 Qui	1P1O	2OR4	1MQD			Sc=5.88971, min distance = 2.25424
FOLH1_HUMAN	95883 4-M	GRIK2_RAT Ful	40539 Qui	1S9T	2OR4	1SD3			Sc=5.82736, min distance = 2.60201
FOSX_LISMO F	23831 HEF	GSK3B_HUMAN F	311 citri	1R0E	2P7K	1H8F			Sc=5.62214, min distance = 0.20211
FPPS_CHICK F	448876 CI	UPPS_ECOLI Fu	445713 fa	1V7U	1UBX	1UEH			Sc=5.9582, min distance = 2.098548
FPPS_HUMAN F	406401 2i	Q86C09_9TRYP	130956 Mi	2EWG	3B7L	2I19	37.65		Sc=6.08269, min distance = 2.26995
FPPS_HUMAN F	448399 DS	ISPA_ECOLI Fu	1195 isop	1RQJ	1ZW5	1RQI			Sc=5.70837, min distance = 2.27930
FPPS_HUMAN F	448400 IS	GGPPS_YEAST F	130956 Mi	2E92	3B7L	2E8U	30.89		Sc=5.72655, min distance = 1.67125
FPPS_HUMAN F	448400 IS	GGPPS_YEAST F	68740 Zol	2E91	2F9K	2E8U	30.89		Sc=5.745, min distance = 2.6540928
FPPS_HUMAN F	448400 IS	ISPA_ECOLI Fu	1195 isop	1RQJ	1ZW5	1RQI			Sc=5.70837, min distance = 2.59283
FPPS_HUMAN F	448400 IS	Q95WL3_TRYCR	1195 isop	1YHM	1ZW5	1YHL	39.07		Sc=5.79937, min distance = 2.02833
FPPS_HUMAN F	6102720 C	GGPPS_YEAST F	68740 Zol	2E91	2F9K	2E8X	30.89		Sc=6.19065, min distance = 2.39478
FPRA_METTM F	16741044	PYRDA_LACLC F	444243 FA	1JUB	2OHH	1JRB			Sc=6.22295, min distance = 2.21399
FPRA_MOOTA F	449551 FM	FLAV_DESVH Fu	444243 FA	1F4P	1YCG	5FX2		0.77	Sc=6.20638, min distance = 2.08742
FPTA_PSEAE F	11987742	LHA4_RHOAC Fu	444279 1s	2FKW	1XKW	1NKZ			Sc=6.38729, min distance = 0.78458
FPTA_PSEAE F	7054 Isat	AOFB_HUMAN Fu	444279 1s	1OJD	1XKW	1OJA			Sc=5.60593, min distance = 2.38173
FRD2_SHEFN F	11987634	TYTR_TRYCR Fu	444188 CI	1BZL	1QO8	1AOG		0.97	Sc=6.45368, min distance = 2.05434
FRD2_SHEFN F	16740985	FRDA_SHEFR Fu	444188 CI	1M64	1QO8	1E39		0.93	Sc=6.45115, min distance = 2.21138
FRD2_SHEFN F	444502 CI	FRDA_SHEFR Fu	444188 CI	1M64	1QO8	1QJD		0.98	Sc=6.45368, min distance = 2.18806

FRD2_SHEFN F	446013 1,	O28604_ARCFU	444188 CI	1JNR	1QO8	1JNZ	0.94	Sc=6.45248, min distance = 2.07014
FRD2_SHEFN F	448054 CI	FRDA_SHEFR Fu	444188 CI	1M64	1QO8	1Y0P	0.97	Sc=6.45208, min distance = 2.21605
FRD2_SHEFN F	6420174	O28603_ARCFU	444188 CI	1JNR	1QO8	2FJB	0.92	Sc=6.45574, min distance = 1.85093
FRD2_SHEFN F	6420174	O28604_ARCFU	444188 CI	1JNR	1QO8	2FJB	0.92	Sc=6.45766, min distance = 2.03383
FRDA_ECOLI F	445905 1	AROQ_HELPY Fu	311 citri	2C4V	2B76	2C57		Sc=5.88049, min distance = 1.20611
FRDA_ECOLI F	51 2-Oxo	SERA_ECOLI Fu	311 citri	2P9E	2B76	1YBA	0.76	Sc=5.70837, min distance = 0.91518
FRDA_ECOLI F	5281 stea	Y1264_MYCTU F	62551 Pen	1Y10	1KF6	2EV1		Sc=6.0894, min distance = 1.421154
FRDA_ECOLI F	6022 Ade	ENPL_CANFA Fu	62551 Pen	2EXL	1KF6	1TC6		Sc=5.92617, min distance = 2.10050
FRDA_ECOLI F	854023 E	Q8WSF8_APLCA	62551 Pen	2BYN	1KF6	2BYQ		Sc=5.6564, min distance = 2.043477
FRDA_SHEFR F	11987634	TYTR_TRYCR Fu	444502 CI	1GXF	1QJD	1AOG	0.95	Sc=6.45686, min distance = 2.07508
FRDA_SHEFR F	449465 CI	GSHR_HUMAN Fu	444188 CI	3DK9	1M64	3GRT	0.98	Sc=6.44304, min distance = 2.13723
FRDC_WOLSU F	11957364	HMOX1_HUMAN F	444124 HE	1OZW	2BS2	1S13	0.86	Sc=6.71584, min distance = 1.91320
FRDC_WOLSU F	446189 CI	HBG1_HUMAN Fu	444124 HE	1I3E	2BS2	1I3D	0.89	Sc=6.7724, min distance = 1.705151
FTSH_HELPY F	440317 AT	MYS2_DICDI Fu	6022 Aden	1VOM	2R65	1MMG	0.99	Sc=5.72497, min distance = 1.86483
FTSH_HELPY F	444564 AD	MYS2_DICDI Fu	6022 Aden	1VOM	2R65	1W9I	0.91	Sc=5.69787, min distance = 2.27780
FTSH_HELPY F	445940 O	CDK2_HUMAN Fu	6022 Aden	1GY3	2R65	1GZ8		Sc=6.17038, min distance = 1.75020
FTSH_HELPY F	447004 CI	MYS2_DICDI Fu	6022 Aden	1VOM	2R65	1LVK		Sc=6.1713, min distance = 2.098350
FTSH_HELPY F	447955 1	CDK2_HUMAN Fu	6022 Aden	1GY3	2R65	1PXI		Sc=5.7387, min distance = 1.989801
FTSH_HELPY F	4565 1h1	CDK2_HUMAN Fu	6022 Aden	1GY3	2R65	1H1R		Sc=6.10404, min distance = 1.90239
FTSH_HELPY F	5327148	IPKA_RABIT Fu	6022 Aden	1JBP	2R65	2ERZ		Sc=6.18109, min distance = 2.06830
FTSH_HELPY F	5957 Ade	AROK_MYCTU Fu	6022 Aden	2IYV	2R65	2IYW	0.99	Sc=6.00406, min distance = 2.05439
FTSH_HELPY F	5957 Ade	BIOD_ECOLI Fu	6022 Aden	1DAD	2R65	1A82	0.99	Sc=5.73597, min distance = 1.52744
FTSH_HELPY F	5957 Ade	KTHY_HUMAN Fu	6022 Aden	1NN3	2R65	1E2Q	0.99	Sc=5.71659, min distance = 2.37627
FTSH_HELPY F	5957 Ade	PSPF_ECOLI Fu	6022 Aden	2C98	2R65	2C96	0.99	Sc=6.02748, min distance = 2.34621
FTSH_HELPY F	6102787 2	HS90A_HUMAN F	6022 Aden	1BYQ	2R65	2CCS		Sc=6.29303, min distance = 1.30602
FTSH_HELPY F	91532 AM	BIOD_ECOLI Fu	6022 Aden	1DAD	2R65	1DAG	0.99	Sc=5.75952, min distance = 1.88729
FTSH_HELPY F	91532 AM	KIF1A_MOUSE F	6022 Aden	1I5S	2R65	1I6I	0.99	Sc=5.76547, min distance = 0.90024
FTSK_PSEAE F	160355 r	CDK2_HUMAN Fu	6022 Aden	1GY3	2IUU	3DDQ		Sc=6.28314, min distance = 1.96896
FTSK_PSEAE F	16214827	CDK2_HUMAN Fu	6022 Aden	1GY3	2IUU	2UZN		Sc=6.09023, min distance = 1.56306
FTSK_PSEAE F	1707 1fvt	CDK2_HUMAN Fu	6022 Aden	1GY3	2IUU	1FVT		Sc=6.09107, min distance = 2.08128
FTSK_PSEAE F	24864078	CDK2_HUMAN Fu	6022 Aden	1GY3	2IUU	2VTO		Sc=6.32962, min distance = 1.82721
FTSK_PSEAE F	2608 1jsv	CDK2_HUMAN Fu	6022 Aden	1GY3	2IUU	1JSV		Sc=5.84301, min distance = 1.92262
FTSK_PSEAE F	4369136	CDK2_HUMAN Fu	6022 Aden	1GY3	2IUU	1DM2		Sc=6.22598, min distance = 1.62870
FTSK_PSEAE F	444564 AD	MYS2_DICDI Fu	440317 AT	1MMG	2IUT	1W9I	0.91	Sc=5.63763, min distance = 2.80723
FTSK_PSEAE F	444842 CI	CDK2_HUMAN Fu	6022 Aden	1GY3	2IUU	1CKP		Sc=5.60768, min distance = 2.74669
FTSK_PSEAE F	445840 di	CDK2_HUMAN Fu	6022 Aden	1GY3	2IUU	1GII		Sc=6.23973, min distance = 2.28713
FTSK_PSEAE F	447004 CI	MYS2_DICDI Fu	440317 AT	1MMG	2IUT	1LVK		Sc=6.03607, min distance = 2.67218

FTSK_PSEAE F	447655 1c	CDK2_HUMAN Fu	6022 Aden	1GY3	2IUU	1OIU		Sc=6.39457, min distance = 2.090952
FTSK_PSEAE F	449088 1v	CDK2_HUMAN Fu	6022 Aden	1GY3	2IUU	1VYZ		Sc=5.97115, min distance = 1.784144
FTSK_PSEAE F	4565 1h1r	CDK2_HUMAN Fu	6022 Aden	1GY3	2IUU	1H1R		Sc=6.25497, min distance = 2.217509
FTSK_PSEAE F	5957 Aden	RK_BOVIN Full	6022 Aden	3C4Z	2IUU	3C4W	0.99	Sc=5.71475, min distance = 2.297040
FTSK_PSEAE F	6338561 C	MYS2_DICDI Fu	440317 AT	1MMG	2IUT	1D0X		Sc=5.76038, min distance = 2.405405
FTSK_PSEAE F	6338562 C	MYS2_DICDI Fu	440317 AT	1MMG	2IUT	1D0Y		Sc=6.15624, min distance = 2.506780
FTSK_PSEAE F	6338562 C	MYS2_DICDI Fu	6022 Aden	1VOM	2IUU	1D0Y		Sc=6.1193, min distance = 2.0781631
FTSK_PSEAE F	72194 2-C	ENPL_CANFA Fu	6022 Aden	1TC6	2IUU	1QYE	0.89	Sc=6.16543, min distance = 1.998004
FTSK_PSEAE F	91532 AMP	MTNK_BACSU Fu	6022 Aden	2OLC	2IUU	2PUL	0.99	Sc=5.73798, min distance = 2.046173
FTSK_PSEAE F	9991833 S	CDK2_HUMAN Fu	6022 Aden	1GY3	2IUU	2R3H		Sc=5.82936, min distance = 2.121120
FTSK_PSEAE F	9994066 4	CDK2_HUMAN Fu	6022 Aden	1GY3	2IUU	2VTJ		Sc=5.85344, min distance = 2.758479
FTSY_THEAQ F	37792 gam	GNAI1_RAT Ful	8977 1dar	1SVK	2IYL	1AS0	0.99	Sc=6.13298, min distance = 2.070236
FTSY_THEAQ F	37792 gam	GNAT1_BOVIN F	8977 1dar	1TAD	2IYL	1TND	0.99	Sc=6.14965, min distance = 2.150345
FTSY_THEAQ F	37792 gam	RAB6B_HUMAN F	8977 1dar	2E9S	2IYL	2FFQ	0.99	Sc=6.13534, min distance = 2.244895
FTSY_THEAQ F	37792 gam	RHOA_HUMAN Fu	8977 1dar	1TX4	2IYL	1CXZ	0.99	Sc=6.14903, min distance = 2.109813
FTSY_THEAQ F	444845 1c	RASH_HUMAN Fu	36735 Gpp	1CTQ	2J7P	1CLU	0.99	Sc=6.54418, min distance = 2.326967
FTSY_THEAQ F	445873 CA	RASH_HUMAN Fu	36735 Gpp	1CTQ	2J7P	2CL6		Sc=6.39857, min distance = 2.268471
FTSY_THEAQ F	446248 CI	RAN_HUMAN Ful	36735 Gpp	1K5D	2J7P	1IBR		Sc=6.52883, min distance = 2.208205
FTSY_THEAQ F	446248 CI	RAN_HUMAN Ful	8977 1dar	3CH5	2IYL	1IBR		Sc=6.51826, min distance = 2.080639
FTSY_THEAQ F	446248 CI	RASH_HUMAN Fu	36735 Gpp	1CTQ	2J7P	1IAQ		Sc=6.14243, min distance = 2.432661
FTSY_THEAQ F	446248 CI	RASH_HUMAN Fu	8977 1dar	2CE2	2IYL	1IAQ		Sc=6.01075, min distance = 2.287707
FTSY_THEAQ F	446248 CI	RASH_HUMAN Fu	93082 Gab	121P	1RJ9	1IAQ		Sc=6.01794, min distance = 2.285961
FTSY_THEAQ F	8582 Inos	PURA1_MOUSE F	8977 1dar	1LON	2IYL	1IWE	0.99	Sc=5.91401, min distance = 2.705149
FTSZ1_METJA	24856355	FTSZ_AQUAE Fu	8977 1dar	2R6R	2VAP	2R75	0.95	Sc=6.59022, min distance = 2.098284
FTSZ1_METJA	24978488	TBB2B_BOVIN F	8977 1dar	1Z2B	2VAP	3DU7		Sc=6.41218, min distance = 2.368077
FTSZ1_METJA	37792 gam	TGM3_HUMAN Fu	8977 1dar	1VJJ	2VAP	1RLE	0.99	Sc=6.43482, min distance = 2.163154
FTSZ1_METJA	447979 5G	KGUA_ECOLI Fu	8977 1dar	2AN9	2VAP	2ANB		Sc=6.39894, min distance = 1.926262
FTSZ1_METJA	5957 Aden	Q381M1_9TRYP	6830 guan	2Q0E	1W5B	2Q0D	0.8	Sc=6.44548, min distance = 1.983917
FTSZ1_METJA	60961 ade	NUD16_XENLA F	6830 guan	2A8S	1W5B	2A8T		Sc=6.19342, min distance = 2.202966
FTSZ1_METJA	6804 guar	DPOL_BPR69 Fu	8977 1dar	1CLQ	2VAP	1WAJ	0.99	Sc=6.32622, min distance = 2.073792
FTSZ1_METJA	6804 guar	EF1A_YEAST Fu	8977 1dar	2B7B	2VAP	1G7C	0.99	Sc=6.34281, min distance = 2.242532
FTSZ_AQUAE F	24978488	TBB2B_BOVIN F	8977 1dar	1Z2B	2R6R	3DU7		Sc=6.00149, min distance = 2.415142
FTSZ_AQUAE F	37792 gam	FTSZ_MYCTU Fu	8977 1dar	1RQ7	2R6R	1RLU	0.99	Sc=5.7589, min distance = 2.3731991
FTSZ_AQUAE F	37792 gam	TBG1_HUMAN Fu	8977 1dar	3CB2	2R6R	1Z5V	0.99	Sc=6.06654, min distance = 1.926079
FTSZ_AQUAE F	448405 CI	ARF1_RAT Full	8977 1dar	1RRG	2R6R	1RRF		Sc=6.46438, min distance = 2.173876
FTSZ_AQUAE F	449108 CI	FTSZ1_METJA F	8977 1dar	2VAP	2R6R	1W58	0.99	Sc=6.08802, min distance = 2.148894
FTSZ_AQUAE F	6804 guar	EF1A_YEAST Fu	8977 1dar	2B7B	2R6R	1G7C	0.99	Sc=6.37109, min distance = 2.369108

FTSZ_AQUAE Fu	6804 guar	TGM3_HUMAN Fu	8977 1dar	1VJJ	2R6R	1SGX	0.99	Sc=6.41155, min distance = 1.906791
FTSZ_AQUAE Fu	8582 Inos	PURA1_MOUSE Fu	8977 1dar	1LON	2R6R	1IWE	0.99	Sc=6.35183, min distance = 2.039856
FTSZ_BACSU Fu	16131862	PAP2_VACCW Fu	8977 1dar	1JTF	2RHL	1P39	0.97	Sc=6.0569, min distance = 2.0680031
FTSZ_BACSU Fu	24978488	TBB2B_BOVIN Fu	8977 1dar	1Z2B	2RHL	3DU7		Sc=6.44684, min distance = 2.221995
FTSZ_BACSU Fu	3713 Indo	TRPA_THET2 Fu	311 citri	1UJP	2VXY	1WXJ		Sc=6.09674, min distance = 1.994410
FTSZ_BACSU Fu	444148 1a	RHA1_ARATH Fu	8977 1dar	2EFC	2RHL	2EFE	0.99	Sc=6.43964, min distance = 1.885076
FTSZ_BACSU Fu	444335 GI	PAP2_VACCW Fu	8977 1dar	1JTF	2RHL	1V39	0.99	Sc=6.47193, min distance = 2.082687
FTSZ_BACSU Fu	446506 DE	ACES_TORCA Fu	8200 TETR	1DX6	2RHJ	1JJB	0.82	Sc=5.85137, min distance = 2.041891
FTSZ_BACSU Fu	447979 5G	KGUA_ECOLI Fu	8977 1dar	2AN9	2RHL	2ANB		Sc=6.40603, min distance = 1.718136
FTSZ_BACSU Fu	449108 CI	FTSZ1_METJA Fu	8977 1dar	2VAP	2RHL	1W58	0.99	Sc=6.08686, min distance = 2.190642
FTSZ_BACSU Fu	449109 CI	FTSZ1_METJA Fu	8977 1dar	2VAP	2RHL	1W5A		Sc=6.51016, min distance = 2.141420
FTSZ_BACSU Fu	5326975 5	KGUA_MYCTU Fu	8977 1dar	1ZNY	2RHL	1ZNX	0.99	Sc=6.41448, min distance = 1.766864
FTSZ_BACSU Fu	6804 guar	TGM3_HUMAN Fu	37792 gam	1RLE	2RHO	1SGX	0.98	Sc=6.40849, min distance = 2.066052
FTSZ_BACSU Fu	6804 guar	TGM3_HUMAN Fu	8977 1dar	1VJJ	2RHL	1SGX	0.99	Sc=6.41073, min distance = 2.082244
FTSZ_BACSU Fu	8582 Inos	PURA1_MOUSE Fu	8977 1dar	1LON	2RHL	1IWE	0.99	Sc=6.3529, min distance = 2.0380098
FTSZ_MYCTU Fu	445463 B-	TGM3_HUMAN Fu	8977 1dar	1VJJ	1RQ7	1L9N		Sc=6.23052, min distance = 2.062530
FTSZ_MYCTU Fu	449109 CI	FTSZ1_METJA Fu	8977 1dar	2VAP	1RQ7	1W5A		Sc=6.49656, min distance = 2.029735
FTSZ_MYCTU Fu	51 2-Oxop	SERA_ECOLI Fu	311 citri	2P9E	1RQ2	1YBA	0.76	Sc=5.66422, min distance = 1.431540
FTSZ_MYCTU Fu	6022 Ader	PARM_ECOLX Fu	8977 1dar	2ZGY	1RQ7	1MWM	0.8	Sc=6.44387, min distance = 1.856402
FTSZ_MYCTU Fu	6804 guar	TGM3_HUMAN Fu	8977 1dar	1VJJ	1RQ7	1SGX	0.99	Sc=6.40849, min distance = 2.195659
FTSZ_PSEAE Fu	24856355	FTSZ_AQUAE Fu	8977 1dar	2R6R	1OFU	2R75	0.95	Sc=6.61764, min distance = 2.149348
FTSZ_PSEAE Fu	24978488	TBB2B_BOVIN Fu	8977 1dar	1Z2B	1OFU	3DU7		Sc=6.45568, min distance = 2.345261
FTSZ_PSEAE Fu	37792 gam	FTSZ_MYCTU Fu	8977 1dar	1RQ7	1OFU	1RLU	0.99	Sc=6.50079, min distance = 2.199339
FTSZ_PSEAE Fu	37792 gam	TGM3_HUMAN Fu	8977 1dar	1VJJ	1OFU	1RLE	0.99	Sc=6.02751, min distance = 1.992784
FTSZ_PSEAE Fu	445404 7-	PAP2_VACCW Fu	8977 1dar	1JTF	1OFU	4DCG	0.93	Sc=6.36638, min distance = 2.116191
FTSZ_PSEAE Fu	445463 B-	TGM3_HUMAN Fu	8977 1dar	1VJJ	1OFU	1L9N		Sc=6.22749, min distance = 2.040211
FTSZ_PSEAE Fu	447979 5G	KGUA_ECOLI Fu	8977 1dar	2AN9	1OFU	2ANB		Sc=5.96585, min distance = 2.017298
FTSZ_PSEAE Fu	449109 CI	FTSZ1_METJA Fu	8977 1dar	2VAP	1OFU	1W5A		Sc=6.52163, min distance = 2.010554
FTSZ_PSEAE Fu	6022 Ader	PARM_ECOLX Fu	8977 1dar	2ZGY	1OFU	1MWM	0.8	Sc=6.45105, min distance = 2.108786
FTSZ_PSEAE Fu	6804 guar	EF1A_YEAST Fu	8977 1dar	2B7B	1OFU	1G7C	0.99	Sc=6.38522, min distance = 2.351301
FTSZ_PSEAE Fu	6804 guar	TGM3_HUMAN Fu	8977 1dar	1VJJ	1OFU	1SGX	0.99	Sc=6.41587, min distance = 1.727288
FTSZ_PSEAE Fu	8582 Inos	PURA1_MOUSE Fu	8977 1dar	1LON	1OFU	1IWE	0.99	Sc=5.90214, min distance = 2.088469
FTSZ_THEMA Fu	449109 CI	FTSZ1_METJA Fu	449108 CI	1W58	1W5F	1W5A		Sc=6.0745, min distance = 2.4365910
FTSZ_THEMA Fu	6830 guar	FTSZ1_METJA Fu	449108 CI	1W58	1W5F	1W5B	0.99	Sc=6.0745, min distance = 2.4002562
FTSZ_THEMA Fu	8977 1dar	FTSZ1_METJA Fu	449108 CI	1W58	1W5F	2VAP	0.99	Sc=6.02422, min distance = 2.635396
FUCA_ECOLI Fu	439276 3-	TPIS_TRYBB Fu	4797 1tpw	1TTJ	4FUA	6TIM		Sc=5.62129, min distance = 2.178901
FUCO_ECOLI Fu	169266 1,	AROB_STAAR Fu	5893 nadi	1XAH	2BI4	1XAG	0.79	Sc=6.30091, min distance = 2.135251

FUCO_ECOLI Fu	6022 Aden	YMX7_YEAST Ful	445794 AD	1TXZ	1RRM	1TY8	0.95	Sc=6.36326, min distance = 1.98648
FUS3_YEAST Fu	11957417	CDK2_HUMAN Ful	6022 Aden	1GY3	2B9H	2I40		Sc=6.32224, min distance = 2.10839
FUS3_YEAST Fu	15942652	CDK2_HUMAN Ful	6022 Aden	1GY3	2B9H	2DUV		Sc=6.35183, min distance = 2.36017
FUS3_YEAST Fu	160355 ro	CDK2_HUMAN Ful	6022 Aden	1GY3	2B9H	3DDQ		Sc=6.47271, min distance = 1.90600
FUS3_YEAST Fu	23653523	CDK2_HUMAN Ful	6022 Aden	1GY3	2B9H	2R3O		Sc=6.32395, min distance = 1.62707
FUS3_YEAST Fu	23727981	CDK2_HUMAN Ful	6022 Aden	1GY3	2B9H	3BHT		Sc=6.22346, min distance = 1.47439
FUS3_YEAST Fu	24864080	CDK2_HUMAN Ful	6022 Aden	1GY3	2B9H	2VTR		Sc=5.95377, min distance = 2.29701
FUS3_YEAST Fu	4369136 C	CDK2_HUMAN Ful	6022 Aden	1GY3	2B9H	1DM2		Sc=6.28626, min distance = 2.26995
FUS3_YEAST Fu	444345 1c	CDK2_HUMAN Ful	6022 Aden	1GY3	2B9H	1AQ1		Sc=6.39832, min distance = 2.10609
FUS3_YEAST Fu	444564 AD	BIOD_ECOLI Ful	6022 Aden	1DAD	2B9H	1BS1	0.91	Sc=6.39627, min distance = 2.26060
FUS3_YEAST Fu	445840 di	CDK2_HUMAN Ful	6022 Aden	1GY3	2B9H	1GII		Sc=6.36489, min distance = 2.12371
FUS3_YEAST Fu	447652 Im	CDK2_HUMAN Ful	6022 Aden	1GY3	2B9H	1OIQ		Sc=6.24477, min distance = 2.30258
FUS3_YEAST Fu	447766 1p	CDK2_HUMAN Ful	6022 Aden	1GY3	2B9H	1P2A		Sc=6.35676, min distance = 2.16729
FUS3_YEAST Fu	447821 CI	CDK2_HUMAN Ful	6022 Aden	1GY3	2B9H	1PF8		Sc=6.17721, min distance = 1.84361
FUS3_YEAST Fu	447916 ac	RIO1_ARCFU Ful	6022 Aden	1ZTH	2B9H	1ZTF	0.95	Sc=6.17941, min distance = 2.76788
FUS3_YEAST Fu	447955 1p	CDK2_HUMAN Ful	6022 Aden	1GY3	2B9H	1PXI		Sc=5.70913, min distance = 2.45796
FUS3_YEAST Fu	447967 CI	CDK2_HUMAN Ful	6022 Aden	1GY3	2B9H	1PYE		Sc=6.38163, min distance = 2.04831
FUS3_YEAST Fu	448008 CI	GSK3B_HUMAN Fu	6022 Aden	1J1C	2B9H	1Q4L		Sc=6.4188, min distance = 1.623481
FUS3_YEAST Fu	4564 1e1v	CDK2_HUMAN Ful	6022 Aden	1GY3	2B9H	1E1V		Sc=6.1556, min distance = 2.077437
FUS3_YEAST Fu	5957 Aden	ARP2_BOVIN Ful	6022 Aden	2P9I	2B9H	1TYQ	0.99	Sc=6.42412, min distance = 2.05115
FUS3_YEAST Fu	5957 Aden	DDL_THET8 Ful	6022 Aden	2ZDH	2B9H	2ZDQ	0.99	Sc=6.4648, min distance = 1.443826
FUS3_YEAST Fu	5957 Aden	MUTS_ECOLI Ful	6022 Aden	1OH7	2B9H	1W7A	0.99	Sc=6.46543, min distance = 1.86115
FUS3_YEAST Fu	5957 Aden	PSPF_ECOLI Ful	6022 Aden	2C98	2B9H	2C96	0.99	Sc=5.99806, min distance = 2.02961
FUS3_YEAST Fu	6083 aden	PURP_METJA Ful	6022 Aden	2R7N	2B9H	2R7M	0.99	Sc=5.8653, min distance = 2.562595
FUS3_YEAST Fu	6420140 2	CDK2_HUMAN Ful	6022 Aden	1GY3	2B9H	2C5Y		Sc=6.52166, min distance = 1.76724
FUS3_YEAST Fu	6852187 2	O57883_PYRHO F	6022 Aden	1WNL	2B9H	2DTH	0.89	Sc=6.06215, min distance = 1.69285
FUS3_YEAST Fu	91532 AMP	MTNK_BACSU Ful	6022 Aden	2OLC	2B9H	2PUL	0.99	Sc=6.4648, min distance = 1.811035
FUS3_YEAST Fu	91532 AMP	PURT_ECOLI Ful	6022 Aden	1KJQ	2B9H	1KJI	0.99	Sc=6.47283, min distance = 1.87556
FUS3_YEAST Fu	9817550 V	CDK2_HUMAN Ful	6022 Aden	1GY3	2B9H	3BHV		Sc=6.37824, min distance = 1.63805
FYN_HUMAN Ful	10224714	CDK2_HUMAN Ful	444345 1c	1AQ1	2DQ7	3DDP		Sc=6.36728, min distance = 2.16448
FYN_HUMAN Ful	11348631	CHK1_HUMAN Ful	444345 1c	1NVR	2DQ7	2E9U		Sc=6.213, min distance = 2.866018
FYN_HUMAN Ful	11553058	LCK_HUMAN Ful	444345 1c	1QPD	2DQ7	2OF2		Sc=6.17573, min distance = 2.34525
FYN_HUMAN Ful	11857236	KAPCA_BOVIN Fu	444345 1c	1STC	2DQ7	3E8C		Sc=6.57843, min distance = 1.92508
FYN_HUMAN Ful	11957417	CDK2_HUMAN Ful	444345 1c	1AQ1	2DQ7	2I40		Sc=6.27483, min distance = 2.46250
FYN_HUMAN Ful	11992146	MAPK2_HUMAN Fu	444345 1c	2PZY	2DQ7	2P3G		Sc=6.192, min distance = 2.248361
FYN_HUMAN Ful	15991572	LCK_HUMAN Ful	444345 1c	1QPD	2DQ7	2OF4		Sc=6.26707, min distance = 2.43971
FYN_HUMAN Ful	160355 ro	CDK2_HUMAN Ful	444345 1c	1AQ1	2DQ7	3DDQ		Sc=6.15595, min distance = 2.26373

FYN_HUMAN Fu	16122608	PDPK1_HUMAN Fu	444345 1c 1OKY	2DQ7	2PE1	Sc=6.32546, min distance = 2.77475
FYN_HUMAN Fu	16122643	CHK1_HUMAN Fu	444345 1c 1NVR	2DQ7	2YWP	Sc=6.25434, min distance = 2.83797
FYN_HUMAN Fu	16214823	CDK2_HUMAN Fu	444345 1c 1AQ1	2DQ7	2UZB	Sc=6.06554, min distance = 2.50210
FYN_HUMAN Fu	16214825	CDK2_HUMAN Fu	444345 1c 1AQ1	2DQ7	2UZE	Sc=6.22897, min distance = 2.82148
FYN_HUMAN Fu	16214826	CDK2_HUMAN Fu	444345 1c 1AQ1	2DQ7	2UZL	Sc=6.11085, min distance = 2.54209
FYN_HUMAN Fu	16214828	CDK2_HUMAN Fu	444345 1c 1AQ1	2DQ7	2UZO	Sc=6.06024, min distance = 2.32403
FYN_HUMAN Fu	16758227	CHK1_HUMAN Fu	444345 1c 1NVR	2DQ7	2R0U	Sc=6.5396, min distance = 2.318315
FYN_HUMAN Fu	1707 1fvt	CDK2_HUMAN Fu	444345 1c 1AQ1	2DQ7	1FVT	Sc=6.07038, min distance = 2.52978
FYN_HUMAN Fu	1907917 2	CHK1_HUMAN Fu	444345 1c 1NVR	2DQ7	2CGW	Sc=6.10392, min distance = 3.02027
FYN_HUMAN Fu	23536770	LCK_HUMAN Ful	444345 1c 1QPD	2DQ7	3BYM	Sc=6.32712, min distance = 2.77682
FYN_HUMAN Fu	23653515	CDK2_HUMAN Fu	444345 1c 1AQ1	2DQ7	2R3F	Sc=5.87941, min distance = 2.65790
FYN_HUMAN Fu	23657800	CHK1_HUMAN Fu	444345 1c 1NVR	2DQ7	2E9P	Sc=6.2584, min distance = 2.732063
FYN_HUMAN Fu	23727982	CDK2_HUMAN Fu	444345 1c 1AQ1	2DQ7	3BHU	Sc=6.21873, min distance = 2.43503
FYN_HUMAN Fu	2396 bisi	PDPK1_HUMAN Fu	444345 1c 1OKY	2DQ7	1UU8	Sc=6.38077, min distance = 2.26368
FYN_HUMAN Fu	2398 bisi	PDPK1_HUMAN Fu	444345 1c 1OKY	2DQ7	1UU9	Sc=6.43336, min distance = 2.44767
FYN_HUMAN Fu	2403 1uvr	PDPK1_HUMAN Fu	444345 1c 1OKY	2DQ7	1UVR	Sc=6.37213, min distance = 2.47633
FYN_HUMAN Fu	24901723	CDK2_HUMAN Fu	444345 1c 1AQ1	2DQ7	2W06	Sc=6.16565, min distance = 2.44003
FYN_HUMAN Fu	24905143	PK3CG_HUMAN Fu	444345 1c 1E8Z	2DQ7	2V4L	Sc=6.09202, min distance = 2.93388
FYN_HUMAN Fu	24905144	PK3CG_HUMAN Fu	444345 1c 1E8Z	2DQ7	3ENE	Sc=6.07542, min distance = 2.40883
FYN_HUMAN Fu	24916751	CDK2_HUMAN Fu	444345 1c 1AQ1	2DQ7	3DOG	Sc=6.14107, min distance = 2.25702
FYN_HUMAN Fu	24963038	LCK_HUMAN Ful	444345 1c 1QPD	2DQ7	2ZM1	Sc=6.04956, min distance = 2.55058
FYN_HUMAN Fu	24963039	LCK_HUMAN Ful	444345 1c 1QPD	2DQ7	2ZM4	Sc=6.24736, min distance = 2.61797
FYN_HUMAN Fu	24963049	KAPCA_BOVIN Fu	444345 1c 1STC	2DQ7	3E8E	Sc=6.5628, min distance = 2.112020
FYN_HUMAN Fu	25021197	CDK2_HUMAN Fu	444345 1c 1AQ1	2DQ7	3EOC	Sc=6.19283, min distance = 2.57125
FYN_HUMAN Fu	33113 gam	IRAK4_HUMAN Fu	444345 1c 2NRY	2DQ7	2OID	Sc=5.96429, min distance = 2.14257
FYN_HUMAN Fu	4369136 C	CDK2_HUMAN Fu	444345 1c 1AQ1	2DQ7	1DM2	Sc=6.20562, min distance = 2.71628
FYN_HUMAN Fu	446704 LS	CDK2_HUMAN Fu	444345 1c 1AQ1	2DQ7	1KE6	Sc=6.23524, min distance = 2.05100
FYN_HUMAN Fu	447649 1c	CDK2_HUMAN Fu	444345 1c 1AQ1	2DQ7	1OI9	Sc=6.2946, min distance = 2.302493
FYN_HUMAN Fu	447652 In	CDK2_HUMAN Fu	444345 1c 1AQ1	2DQ7	1OIQ	Sc=6.09022, min distance = 2.82699
FYN_HUMAN Fu	447654 CI	CDK2_HUMAN Fu	444345 1c 1AQ1	2DQ7	1OIT	Sc=6.13824, min distance = 2.31891
FYN_HUMAN Fu	447655 1c	CDK2_HUMAN Fu	444345 1c 1AQ1	2DQ7	1OIU	Sc=6.24245, min distance = 2.38662
FYN_HUMAN Fu	447962 1p	CDK2_HUMAN Fu	444345 1c 1AQ1	2DQ7	2C5N	Sc=6.09201, min distance = 2.94446
FYN_HUMAN Fu	448043 2g	KAPCA_BOVIN Fu	444345 1c 1STC	2DQ7	1Q8U	Sc=5.94415, min distance = 2.81429
FYN_HUMAN Fu	448171 CI	LCK_HUMAN Ful	444345 1c 1QPD	2DQ7	1QPE	Sc=6.09476, min distance = 2.60330
FYN_HUMAN Fu	456214 pu	KS6A1_HUMAN Fu	444345 1c 2Z7R	2DQ7	2Z7S	Sc=6.27762, min distance = 2.30900
FYN_HUMAN Fu	4565 1h1r	CDK2_HUMAN Fu	444345 1c 1AQ1	2DQ7	1H1R	Sc=6.21437, min distance = 2.38374
FYN_HUMAN Fu	4566 1h1s	CDK2_HUMAN Fu	444345 1c 1AQ1	2DQ7	1H1S	Sc=6.24916, min distance = 2.22647

FYN_HUMAN Fu	5288016 6	CDK2_HUMAN Fu	444345 1c	1AQ1	2DQ7	2B53			Sc=5.86387, min distance = 2.82514
FYN_HUMAN Fu	5288708 1	CDK2_HUMAN Fu	444345 1c	1AQ1	2DQ7	1KE5			Sc=6.0941, min distance = 2.635414
FYN_HUMAN Fu	5288711 1	CDK2_HUMAN Fu	444345 1c	1AQ1	2DQ7	1KE8			Sc=6.25032, min distance = 2.62979
FYN_HUMAN Fu	5288712 1	CDK2_HUMAN Fu	444345 1c	1AQ1	2DQ7	1KE9			Sc=6.18257, min distance = 2.70309
FYN_HUMAN Fu	5327121 I	PIM1_HUMAN Fu	444345 1c	1YHS	2DQ7	2C3I			Sc=6.09239, min distance = 2.74128
FYN_HUMAN Fu	5327122 C	CHK1_HUMAN Fu	444345 1c	1NVR	2DQ7	2C3K			Sc=6.38992, min distance = 2.04009
FYN_HUMAN Fu	5327123 I	CHK1_HUMAN Fu	444345 1c	1NVR	2DQ7	2C3L			Sc=6.13423, min distance = 2.58518
FYN_HUMAN Fu	5331010 c	CDK2_HUMAN Fu	444345 1c	1AQ1	2DQ7	2BKZ			Sc=6.19288, min distance = 2.44133
FYN_HUMAN Fu	5494414 C	CDK2_HUMAN Fu	444345 1c	1AQ1	2DQ7	2A0C			Sc=6.39009, min distance = 2.61655
FYN_HUMAN Fu	6102670	CDK2_HUMAN Fu	444345 1c	1AQ1	2DQ7	1YKR			Sc=6.25497, min distance = 2.35133
FYN_HUMAN Fu	6420138 2	CDK2_HUMAN Fu	444345 1c	1AQ1	2DQ7	2UUE			Sc=6.22886, min distance = 2.61199
FYN_HUMAN Fu	6420139 C	CDK2_HUMAN Fu	444345 1c	1AQ1	2DQ7	2C5V			Sc=6.08593, min distance = 2.76977
FYN_HUMAN Fu	6539118 g	CDK2_HUMAN Fu	444345 1c	1AQ1	2DQ7	1FVV			Sc=6.3457, min distance = 2.366881
FYN_HUMAN Fu	656971 CI	CDK2_HUMAN Fu	444345 1c	1AQ1	2DQ7	1Y8Y			Sc=5.8816, min distance = 2.296026
FYN_HUMAN Fu	6852207 M	KAPCA_BOVIN Fu	444345 1c	1STC	2DQ7	2GNI			Sc=5.90321, min distance = 2.15574
FYN_HUMAN Fu	72271 7-H	CDK2_HUMAN Fu	444345 1c	1AQ1	2DQ7	1PKD		0.91	Sc=6.42697, min distance = 2.02028
FYN_HUMAN Fu	9817550 V	CDK2_HUMAN Fu	444345 1c	1AQ1	2DQ7	3BHV			Sc=6.32218, min distance = 2.47527
G3P1_ECOLI Fu	169266 1,	GALE_HUMAN Fu	5893 nadi	1HZJ	1GAD	1I3K		0.79	Sc=6.72797, min distance = 2.04737
G3P1_ECOLI Fu	439153 Di	Q9BJJ9_PLAFA	5893 nadi	1UH5	1GAD	1V35		0.79	Sc=6.72235, min distance = 1.77627
G3P1_ECOLI Fu	440141 9i	G6PD_LEUME Fu	5893 nadi	1H94	1GAD	1E7Y			Sc=5.64007, min distance = 2.00553
G3P1_ECOLI Fu	446050 CI	G3P_HUMAN Ful	5893 nadi	1U8F	1GAD	3GPD	66.06	0.95	Sc=6.8224, min distance = 0.650687
G3P1_ECOLI Fu	6022 Ader	Q5SI02_THET8	5893 nadi	2BJK	1GAD	2BJA			Sc=6.17595, min distance = 2.66135
G3P1_KLULA Fu	79025 alp	MALE_ECOLI Fu	64960 Pol	1LAX	2I5P	1ANF		0.89	Sc=5.86637, min distance = 0.97858
G3P1_KLUMA Fu	79025 alp	MALE_ECOLI Fu	64960 Pol	1LAX	2I5P	1ANF		0.89	Sc=5.86637, min distance = 0.97858
G3PA_SPIOL Fu	11987635	ADH_DROLE Ful	5893 nadi	1SBY	1NBO	1B2L		0.76	Sc=6.35172, min distance = 1.96631
G3PA_SPIOL Fu	439153 Di	Q9BJJ9_PLAFA	5893 nadi	1UH5	1NBO	1V35		0.79	Sc=6.72482, min distance = 1.77063
G3PA_SPIOL Fu	440141 9i	DHB1_HUMAN Fu	5886 NADP	1QYV	1RM4	1QYW			Sc=5.72406, min distance = 2.74656
G3PA_SPIOL Fu	440141 9i	G6PD_LEUME Fu	5886 NADP	1H9A	1RM4	1E7Y			Sc=6.32967, min distance = 1.87795
G3PA_SPIOL Fu	444564 AI	ARO1_EMENI Fu	5893 nadi	1SG6	1NBO	1NVA			Sc=5.68501, min distance = 1.50882
G3PA_SPIOL Fu	445794 AI	ADHX_HUMAN Fu	5893 nadi	2FZW	1NBO	2FZE			Sc=5.87012, min distance = 1.22728
G3PA_SPIOL Fu	446050 CI	G3P_HUMAN Ful	5893 nadi	1U8F	1NBO	3GPD	45.54	0.95	Sc=6.8224, min distance = 0.982130
G3PA_SPIOL Fu	446288 NA	G3P_PALVE Ful	5893 nadi	1DSS	1NBO	1IHX	46.48		Sc=6.34155, min distance = 0.91435
G3PA_SPIOL Fu	6022 Ader	UGDH_HUMAN Fu	5893 nadi	2Q3E	1NBO	2QG4			Sc=6.54147, min distance = 0.70829
G3PG_LEIME Fu	11987635	ADH_DROLE Ful	5893 nadi	1SBY	1A7K	1B2L		0.76	Sc=6.36057, min distance = 2.05669
G3PG_LEIME Fu	15942690	INHA_MYCTU Fu	5893 nadi	2H7I	1A7K	2NTJ			Sc=6.14303, min distance = 1.66722
G3PG_LEIME Fu	439153 Di	ADH1B_HUMAN Fu	5893 nadi	1U3U	1A7K	1DEH		0.79	Sc=6.30018, min distance = 1.64264
G3PG_LEIME Fu	439153 Di	Q9BJJ9_PLAFA	5893 nadi	1UH5	1A7K	1V35		0.79	Sc=6.74139, min distance = 1.80462

G3PG_LEIME Fu	446288 NA	G3P_PALVE Ful	5893 nadi	1DSS	1A7K	1IHX	54.39		Sc=6.36484, min distance = 1.23788
G3PG_LEIME Fu	447049 OA	NPD_THEMA Ful	5893 nadi	2H4H	1A7K	2H4J			Sc=5.86024, min distance = 2.12620
G3PG_LEIME Fu	449263 IN	INHA_MYCTU Fu	5893 nadi	2H7I	1A7K	2NV6		0.93	Sc=6.83388, min distance = 2.11545
G3PG_LEIME Fu	6022 Aden	Q5SI02_THET8	5893 nadi	2BJK	1A7K	2BJA			Sc=6.24465, min distance = 2.34986
G3PG_TRYCR Fu	439153 Di	Q9BJJ9_PLAFA	5893 nadi	1UH5	3DMT	1V35		0.79	Sc=6.7575, min distance = 1.804949
G3PG_TRYCR Fu	444427 CI	ADH_DROLE Ful	5893 nadi	1SBY	3DMT	1B15		0.76	Sc=6.07254, min distance = 1.74079
G3PG_TRYCR Fu	444564 AD	ARO1_EMENI Fu	5893 nadi	1SG6	3DMT	1NVA			Sc=5.99064, min distance = 1.81770
G3PG_TRYCR Fu	446050 CI	G3P_HUMAN Ful	5893 nadi	1U8F	3DMT	3GPD	51.42	0.95	Sc=6.82297, min distance = 0.85693
G3PG_TRYCR Fu	6022 Aden	Q5SI02_THET8	5893 nadi	2BJK	3DMT	2BJA			Sc=6.27055, min distance = 2.11727
G3P_AQUAE Fu	11987635	ADH_DROLE Ful	5893 nadi	1SBY	2EP7	1B2L		0.76	Sc=6.82117, min distance = 2.00123
G3P_AQUAE Fu	169266 1,	GALE_HUMAN Fu	5893 nadi	1HZJ	2EP7	1I3K		0.79	Sc=6.75794, min distance = 1.99088
G3P_AQUAE Fu	439153 Di	ADH1B_HUMAN F	5893 nadi	1U3U	2EP7	1DEH		0.79	Sc=6.75279, min distance = 1.87244
G3P_AQUAE Fu	439153 Di	ADH1E_HORSE F	5893 nadi	1MGO	2EP7	2JHF		0.79	Sc=6.42284, min distance = 2.00746
G3P_AQUAE Fu	439153 Di	GALE_ECOLI Fu	5893 nadi	1UDC	2EP7	1UDB		0.79	Sc=6.74618, min distance = 2.05435
G3P_AQUAE Fu	439153 Di	Q9BJJ9_PLAFA	5893 nadi	1UH5	2EP7	1V35		0.79	Sc=6.75137, min distance = 1.78516
G3P_AQUAE Fu	440516 CI	MDH_THETH Ful	5893 nadi	1BMD	2EP7	1BDM		0.79	Sc=6.31088, min distance = 1.99602
G3P_AQUAE Fu	444427 CI	ADH_DROLE Ful	5893 nadi	1SBY	2EP7	1B15		0.76	Sc=6.35233, min distance = 2.13376
G3P_AQUAE Fu	444428 CI	ADH_DROLE Ful	5893 nadi	1SBY	2EP7	1B16		0.77	Sc=6.37353, min distance = 2.16163
G3P_AQUAE Fu	445794 AD	ADHX_HUMAN Fu	5893 nadi	2FZW	2EP7	2FZE			Sc=6.57197, min distance = 2.05182
G3P_AQUAE Fu	449263 IN	INHA_MYCTU Fu	5893 nadi	2H7I	2EP7	2NV6		0.93	Sc=6.83523, min distance = 2.06396
G3P_AQUAE Fu	5957 Aden	MAOM_HUMAN Fu	5893 nadi	1PJ3	2EP7	1GZ4			Sc=6.50178, min distance = 1.12138
G3P_AQUAE Fu	6022 Aden	Q5SI02_THET8	5893 nadi	2BJK	2EP7	2BJA			Sc=6.26336, min distance = 2.26733
G3P_BACST Fu	16741136	DYR_ECOLI Ful	5886 NADP	1RA2	2DBV	1RX9	1		Sc=5.63618, min distance = 2.02436
G3P_HUMAN Fu	11987635	ADH_DROLE Ful	5893 nadi	1SBY	1U8F	1B2L		0.76	Sc=6.35889, min distance = 2.04262
G3P_HUMAN Fu	439153 Di	GALE_ECOLI Fu	5893 nadi	1UDC	1U8F	1UDB		0.79	Sc=6.7298, min distance = 2.024719
G3P_HUMAN Fu	445794 AD	G3P_PALVE Ful	5893 nadi	1DSS	1U8F	1IHY	74.31		Sc=6.66314, min distance = 1.28175
G3P_HUMAN Fu	446288 NA	G3P_PALVE Ful	5893 nadi	1DSS	1U8F	1IHX	74.31		Sc=6.36728, min distance = 1.17762
G3P_HUMAN Fu	447049 OA	NPD_THEMA Ful	5893 nadi	2H4H	1U8F	2H4J			Sc=5.86153, min distance = 2.17840
G3P_HUMAN Fu	449263 IN	INHA_MYCTU Fu	5893 nadi	2H7I	1U8F	2NV6		0.93	Sc=6.83012, min distance = 1.92485
G3P_METFE Fu	440141 9i	DHB1_HUMAN Fu	5886 NADP	1QYV	1CF2	1QYW			Sc=6.34773, min distance = 2.74417
G3P_METFE Fu	440334 Ni	DAPB_ECOLI Fu	5886 NADP	1DIH	1CF2	1DRW		0.91	Sc=6.29161, min distance = 2.02774
G3P_METJA Fu	440141 9i	DHB1_HUMAN Fu	5886 NADP	1QYV	2YYY	1QYW			Sc=6.38219, min distance = 2.32355
G3P_PALVE Fu	15942665	INHA_MYCTU Fu	5893 nadi	2H7I	1DSS	2H9I		0.88	Sc=6.54762, min distance = 2.07806
G3P_PALVE Fu	169266 1,	GALE_HUMAN Fu	5893 nadi	1HZJ	1DSS	1I3K		0.79	Sc=6.42848, min distance = 2.03657
G3P_PALVE Fu	439153 Di	Q9BJJ9_PLAFA	5893 nadi	1UH5	1DSS	1V35		0.79	Sc=6.73389, min distance = 1.98425
G3P_PALVE Fu	446050 CI	G3P_HUMAN Ful	5893 nadi	1U8F	1DSS	3GPD	74.31	0.95	Sc=6.76878, min distance = 1.66438
G3P_PALVE Fu	449263 IN	INHA_MYCTU Fu	5893 nadi	2H7I	1DSS	2NV6		0.93	Sc=6.52297, min distance = 2.12472

G3P_PALVE Fu	6022 Aden	Q5SI02_THET8 I	5893 nadi	2BJK	1DSS	2BJA		Sc=6.26431, min distance = 2.37574
G3P_PYRHO Fu	11987635	ADH_DROLE Ful	5893 nadi	1SBY	2CZC	1B2L	0.76	Sc=6.05616, min distance = 2.16053
G3P_PYRHO Fu	123927 A3	DAPB_ECOLI Fu	5893 nadi	1DRU	2CZC	1DRV	0.97	Sc=5.7938, min distance = 2.155797
G3P_PYRHO Fu	16129587	G3PA_SPIOL Fu	5893 nadi	1NBO	2CZC	2PKR		Sc=6.75198, min distance = 1.58021
G3P_PYRHO Fu	439153 Di	GALE_ECOLI Fu	5893 nadi	1UDC	2CZC	1UDB	0.79	Sc=6.28982, min distance = 1.86168
G3P_PYRHO Fu	446050 CI	G3P_HUMAN Ful	5893 nadi	1U8F	2CZC	3GPD	0.95	Sc=6.28042, min distance = 1.26762
G3P_RABIT Fu	5893 nadi	GALE_ECOLI Fu	439153 Di	1UDB	1J0X	1UDC	0.79	Sc=6.40985, min distance = 1.84050
G3P_THEAQ Fu	11987635	ADH_DROLE Ful	5893 nadi	1SBY	2G82	1B2L	0.76	Sc=6.81718, min distance = 2.02256
G3P_THEAQ Fu	15942665	INHA_MYCTU Fu	5893 nadi	2H7I	2G82	2H9I	0.88	Sc=6.54567, min distance = 2.14025
G3P_THEAQ Fu	439153 Di	CTBP1_RAT Ful	5893 nadi	1HKU	2G82	1HL3	0.79	Sc=6.2853, min distance = 2.020240
G3P_THEAQ Fu	446050 CI	G3P_HUMAN Ful	5893 nadi	1U8F	2G82	3GPD	0.95	Sc=6.80855, min distance = 0.93589
G3P_THEAQ Fu	449263 IN	INHA_MYCTU Fu	5893 nadi	2H7I	2G82	2NV6	0.93	Sc=6.52297, min distance = 2.21297
G3P_THEMA Fu	11987635	ADH_DROLE Ful	5893 nadi	1SBY	1HDG	1B2L	0.76	Sc=6.81284, min distance = 2.13330
G3P_THEMA Fu	15942665	INHA_MYCTU Fu	5893 nadi	2H7I	1HDG	2H9I	0.88	Sc=6.86084, min distance = 1.85079
G3P_THEMA Fu	15942690	INHA_MYCTU Fu	5893 nadi	2H7I	1HDG	2NTJ		Sc=6.14731, min distance = 1.67326
G3P_THEMA Fu	169266 1,	GALE_HUMAN Fu	5893 nadi	1HZJ	1HDG	1I3K	0.79	Sc=6.74797, min distance = 2.10576
G3P_THEMA Fu	439153 Di	Q9BJJ9_PLAFA I	5893 nadi	1UH5	1HDG	1V35	0.79	Sc=6.7372, min distance = 2.012791
G3P_THEMA Fu	445794 AD	ADHX_HUMAN Fu	5893 nadi	2FZW	1HDG	2FZE		Sc=5.8252, min distance = 2.109011
G3P_THEMA Fu	446006 CI	MDH_THETH Ful	5893 nadi	1BMD	1HDG	2CVQ		Sc=6.02906, min distance = 2.02012
G3P_THEMA Fu	446050 CI	G3P_HUMAN Ful	5893 nadi	1U8F	1HDG	3GPD	0.95	Sc=6.71564, min distance = 2.34986
G3P_THEMA Fu	446288 NA	G3P_PALVE Ful	5893 nadi	1DSS	1HDG	1IHX		Sc=6.29376, min distance = 2.40302
G3P_THEMA Fu	449263 IN	INHA_MYCTU Fu	5893 nadi	2H7I	1HDG	2NV6	0.93	Sc=6.83262, min distance = 2.01611
G3P_THEMA Fu	6022 Aden	Q5SI02_THET8 I	5893 nadi	2BJK	1HDG	2BJA		Sc=6.24046, min distance = 2.52283
G6PD_HUMAN F	15942680	6PGD_LACLM Fu	5886 NADP	2IZ0	2BH9	2IZ1		Sc=6.02108, min distance = 2.36763
G6PD_HUMAN F	440141 9i	DHB1_HUMAN Fu	5886 NADP	1QYV	2BH9	1QYW		Sc=5.91218, min distance = 1.04992
G6PD_LEUME F	16741136	DYR_ECOLI Ful	165230 1a	1RA9	2DPG	1RX9		Sc=5.9294, min distance = 2.419314
G6PD_LEUME F	439153 Di	Q9BJJ9_PLAFA I	5893 nadi	1UH5	1H94	1V35	0.79	Sc=6.25197, min distance = 2.16935
G6PI_MOUSE F	191445 1g	G6PI_HUMAN Fu	192838 Er	1IRI	1U0G	1NUH		Sc=5.94313, min distance = 1.94546
G6PI_MOUSE F	439284 Ro	GLMS_ECOLI Fu	152306 Sc	2J6H	2CXQ	1MOR	0.81	Sc=5.97755, min distance = 1.83026
G6PI_MOUSE F	446761 CI	G6PI_RABIT Fu	91493 1pg	1DQR	2CXR	1KOJ		Sc=5.92605, min distance = 1.46999
G6PI_MOUSE F	447096 Ro	G6PI_PYRFU Fu	91493 1pg	1QY4	2CXR	2GC3		Sc=6.02042, min distance = 2.49064
G6PI_MOUSE F	6102755 C	GLMS_ECOLI Fu	152306 Sc	2J6H	2CXQ	2BPL		Sc=6.12866, min distance = 0.92053
G6PI_RABIT F	439427 Ro	CGGR_BACSU Fu	440641 1m	3BXH	1HOX	3BXG	0.89	Sc=5.94452, min distance = 2.12631
G6PI_RABIT F	447096 Ro	G6PI_PYRFU Fu	91493 1pg	1QY4	1DQR	2GC3		Sc=6.0025, min distance = 2.300123
G6PI_RABIT F	448823 CI	G6PI_MOUSE Fu	152306 Sc	2CXQ	1XTB	1U0F		Sc=5.92308, min distance = 2.31695
G6PI_RABIT F	448823 CI	G6PI_MOUSE Fu	91493 1pg	2CXR	1DQR	1U0F		Sc=5.92308, min distance = 2.14130
GAL10_YEAST I	439764 be	ARAF_ECOLI Fu	439353 be	8ABP	1Z45	6ABP	0.93	Sc=5.61842, min distance = 2.53558

GAL10_YEAST I	445675 UD	GALE_HUMAN Fu	169266 1,	1I3K	1Z45	1HZJ			Sc=6.1034, min distance = 1.961365
GAL10_YEAST I	5893 nadi	GALE_HUMAN Fu	169266 1,	1I3K	1Z45	1HZJ		0.79	Sc=6.80041, min distance = 2.45836
GAL10_YEAST I	5893 nadi	GALE_HUMAN Fu	8629 UDP-	1I3K	1Z45	1HZJ			Sc=6.7943, min distance = 2.269382
GAL10_YEAST I	6031 Uric	GSTA_BPT4 Ful	8629 UDP-	1Y6F	1Z45	1XV5		0.89	Sc=5.80136, min distance = 2.44478
GAL10_YEAST I	7027 gluc	Q72KX2_THET2 I	439353 be	2B3F	1Z45	2B3B			Sc=5.84152, min distance = 2.31861
GAL1_PYRFU Fu	444564 AD	MYS2_DICDI Fu	6022 Aden	1VOM	1S4E	1W9I		0.91	Sc=5.99534, min distance = 1.86335
GAL1_PYRFU Fu	445966 O6	CDK2_HUMAN Fu	6022 Aden	1GY3	1S4E	1H0V			Sc=6.14268, min distance = 1.71191
GAL1_PYRFU Fu	448968 1u	HS90A_HUMAN Fu	6022 Aden	1BYQ	1S4E	1UY9			Sc=6.12698, min distance = 2.18483
GAL1_PYRFU Fu	448976 1u	HS90A_HUMAN Fu	6022 Aden	1BYQ	1S4E	1UYK			Sc=6.00781, min distance = 2.29888
GAL1_PYRFU Fu	4565 1h1r	CDK2_HUMAN Fu	6022 Aden	1GY3	1S4E	1H1R			Sc=5.9741, min distance = 1.698518
GAL1_PYRFU Fu	489519 AI	DCK_HUMAN Ful	6022 Aden	1P5Z	1S4E	2ZI4		0.92	Sc=6.25868, min distance = 1.79081
GAL1_PYRFU Fu	5957 Aden	MALK_ECOLI Fu	6022 Aden	2AWN	1S4E	1Q12		0.99	Sc=6.04516, min distance = 0.49787
GAL1_PYRFU Fu	5957 Aden	MUTS_ECOLI Fu	6022 Aden	1OH7	1S4E	1W7A		0.99	Sc=6.03743, min distance = 1.89178
GAL1_PYRFU Fu	91532 AMF	AROK_MYCTU Fu	6022 Aden	2IYV	1S4E	1ZYU		0.99	Sc=5.74217, min distance = 2.16179
GAL1_PYRFU Fu	9549213 2	HS90A_HUMAN Fu	6022 Aden	1BYQ	1S4E	2FWZ			Sc=6.14203, min distance = 2.14789
GAL1_PYRHO Fu	439764 be	ARAF_ECOLI Fu	439357 ga	8ABP	2DEJ	6ABP		0.93	Sc=5.64481, min distance = 2.41376
GAL1_YEAST Fu	1400 nche	HCK_HUMAN Ful	33113 gam	1AD5	2AJ4	1QCF			Sc=6.27493, min distance = 1.95258
GAL1_YEAST Fu	3547 Fasu	ROCK1_HUMAN Fu	33113 gam	2V55	2AJ4	2ESM			Sc=5.98489, min distance = 1.71383
GAL1_YEAST Fu	439731 al	ARAF_ECOLI Fu	439357 ga	8ABP	2AJ4	6ABP		0.93	Sc=5.6567, min distance = 2.369975
GAL1_YEAST Fu	439764 be	ARAF_ECOLI Fu	439357 ga	8ABP	2AJ4	6ABP		0.93	Sc=5.65079, min distance = 2.27649
GAL1_YEAST Fu	444564 AD	MYS2_DICDI Fu	33113 gam	1MMN	2AJ4	1W9I		0.91	Sc=5.99994, min distance = 2.21919
GAL1_YEAST Fu	448043 2g	ROCK1_HUMAN Fu	33113 gam	2V55	2AJ4	3D9V			Sc=6.06165, min distance = 1.68775
GAL1_YEAST Fu	5149739 2	CSK2A_MAIZE Fu	33113 gam	1LP4	2AJ4	2OXY			Sc=5.60686, min distance = 2.01307
GAL1_YEAST Fu	5326976 1	CSK2A_MAIZE Fu	33113 gam	1LP4	2AJ4	1ZOE			Sc=5.77644, min distance = 2.18481
GAL1_YEAST Fu	91532 AMF	PURT_ECOLI Fu	33113 gam	1EYZ	2AJ4	1KJI		0.99	Sc=6.53262, min distance = 1.92422
GAL80_YEAST I	445473 NA	GFO_ZYMMO Ful	5893 nadi	1EVJ	3BTS	1RYD		0.94	Sc=6.78334, min distance = 1.56435
GAL80_YEAST I	6022 Aden	Q5SI02_THET8 I	5893 nadi	2BJK	3BTS	2BJA			Sc=6.16289, min distance = 2.62047
GALE_ECOLI Fu	169266 1,	GALE_HUMAN Fu	5893 nadi	1HZJ	1UDC	1I3K	52.79	0.79	Sc=6.79904, min distance = 2.04554
GALE_ECOLI Fu	445794 AD	ADHX_HUMAN Fu	5893 nadi	2FZW	1UDC	2FZE			Sc=6.66569, min distance = 1.87983
GALE_ECOLI Fu	6030 Uric	O87988_BORBR I	6031 Urid	2PZM	1NAH	2PZL		0.99	Sc=6.27868, min distance = 2.28382
GALE_ECOLI Fu	6030 Uric	PYRH_ECOLI Fu	6031 Urid	2BND	1NAH	2BNE		0.99	Sc=6.26565, min distance = 2.54003
GALE_ECOLI Fu	6133 uric	PYRH_ECOLI Fu	6031 Urid	2BND	1NAH	2BNF	1		Sc=5.64627, min distance = 1.89808
GALE_HUMAN Fu	439153 Di	GALE_ECOLI Fu	5893 nadi	1UDC	1HZJ	1UDB	52.79	0.79	Sc=6.79211, min distance = 2.53830
GALE_HUMAN Fu	445794 AD	ADHX_HUMAN Fu	5893 nadi	2FZW	1HZJ	2FZE			Sc=6.67276, min distance = 0.29443
GALE_HUMAN Fu	449263 IN	INHA_MYCTU Fu	5893 nadi	2H7I	1HZJ	2NV6		0.93	Sc=6.87022, min distance = 2.10700
GALE_HUMAN Fu	6031 Uric	GALE_ECOLI Fu	5893 nadi	1UDC	1HZJ	1NAH	52.79		Sc=6.33527, min distance = 2.52239
GALK1_HUMAN I	16750062	CSK2A_MAIZE Fu	33113 gam	1LP4	1WUU	2OXD			Sc=5.7481, min distance = 2.131429

GALK1_HUMAN I	3064778 H	ROCK1_HUMAN Fu	33113 gam	2V55	1WUU	2ETK		Sc=6.15759, min distance = 1.34259(
GALK1_HUMAN I	3547 Fasu	ROCK1_HUMAN Fu	33113 gam	2V55	1WUU	2ESM		Sc=5.97078, min distance = 2.37281(
GALK1_HUMAN I	439731 al	ARAF_ECOLI Ful	439357 ga	8ABP	1WUU	6ABP	0.93	Sc=5.61175, min distance = 2.38186:
GALK1_HUMAN I	439764 be	ARAF_ECOLI Ful	439357 ga	8ABP	1WUU	6ABP	0.93	Sc=5.61842, min distance = 2.28755(
GALK1_HUMAN I	5149739 2	CSK2A_MAIZE Fu	33113 gam	1LP4	1WUU	2OXY		Sc=5.62231, min distance = 2.36897:
GALK1_HUMAN I	5326976 1	CSK2A_MAIZE Fu	33113 gam	1LP4	1WUU	1ZOE		Sc=5.80553, min distance = 2.41119·
GALK1_HUMAN I	5326977 1	CSK2A_MAIZE Fu	33113 gam	1LP4	1WUU	1ZOG		Sc=5.68791, min distance = 2.02095!
GALK1_HUMAN I	5327148 C	IPKA_RABIT Ful	33113 gam	1CDK	1WUU	2ERZ		Sc=6.21742, min distance = 1.61449(
GALK1_HUMAN I	6083 ader	HSP71_HUMAN Fu	33113 gam	2E8A	1WUU	1XQS	0.97	Sc=5.92099, min distance = 2.19070:
GALK1_HUMAN I	64960 Pol	FCN2_HUMAN Ful	439357 ga	2J3U	1WUU	2J0Y	0.89	Sc=5.89111, min distance = 0.56309·
GALK2_HUMAN I	101798 Me	LECA_ARTIN Ful	84265 lax	1M26	2A2D	1WS5		Sc=5.86105, min distance = 2.26588:
GALK2_HUMAN I	3547 Fasu	ROCK1_HUMAN Fu	33113 gam	2V55	2A2D	2ESM		Sc=5.98898, min distance = 2.44012(
GALK2_HUMAN I	439353 be	LEC_ERYCO Ful	84265 lax	1AX0	2A2D	1AX1		Sc=5.77993, min distance = 2.24936(
GALK2_HUMAN I	444564 AD	MYS2_DICDI Ful	6022 Aden	1VOM	2A2C	1W9I	0.91	Sc=6.45524, min distance = 2.01355:
GALK2_HUMAN I	447916 ad	RIO1_ARCFU Ful	6022 Aden	1ZTH	2A2C	1ZTF	0.95	Sc=6.26717, min distance = 2.13757(
GALK2_HUMAN I	448882 NG	LECA_ARTIN Ful	84265 lax	1M26	2A2D	1UGX	0.96	Sc=6.11407, min distance = 2.46180:
GALK2_HUMAN I	5327148 C	IPKA_RABIT Ful	33113 gam	1CDK	2A2D	2ERZ		Sc=6.25285, min distance = 1.45942:
GALK2_HUMAN I	60961 ade	IPKA_RABIT Ful	6022 Aden	1JBP	2A2C	1FMO	0.95	Sc=6.26369, min distance = 2.11242(
GALK2_HUMAN I	60961 ade	PIM1_HUMAN Ful	33113 gam	1XR1	2A2D	1YI4	0.93	Sc=6.25934, min distance = 2.20965(
GALK2_HUMAN I	76935 alp	LECA_ARTIN Ful	84265 lax	1M26	2A2D	1WS4		Sc=5.7389, min distance = 2.552259:
GALK2_HUMAN I	76935 alp	Q868M7_9ECHN I	84265 lax	2Z48	2A2D	2Z49		Sc=5.88141, min distance = 2.11912:
GALK2_HUMAN I	79025 alp	TRFL_BOVIN Ful	84265 lax	2AYS	2A2D	2DWJ		Sc=5.71748, min distance = 2.27242(
GALK2_HUMAN I	91532 AME	NIFH1_AZOVI Fu	6022 Aden	1FP6	2A2C	2AFK	0.99	Sc=6.09252, min distance = 1.72920·
GALM_HUMAN Fu	6027 xylo	Q9ZB17_9LACT I	439353 be	1NSX	1SO0	1MN0	0.93	Sc=5.62221, min distance = 2.10839·
GALT2_HUMAN I	6030 Urid	O87988_BORBR I	6031 Urid	2PZM	2FFU	2PZL	0.99	Sc=6.30651, min distance = 2.22709(
GALT2_HUMAN I	6030 Urid	PYRH_ECOLI Ful	6031 Urid	2BND	2FFU	2BNE	0.99	Sc=6.32026, min distance = 1.88410(
GALT2_HUMAN I	6133 urid	PYRH_ECOLI Ful	6031 Urid	2BND	2FFU	2BNF	1	Sc=5.99785, min distance = 1.42332:
GAMT_HUMAN Fu	60961 ade	PIMT_PYRFU Ful	439155 Ad	1JG1	1ZX0	1JG2	0.84	Sc=6.34196, min distance = 2.28566:
GAMT_HUMAN Fu	65482 sir	ERM_BACSU Ful	439155 Ad	1QAN	1ZX0	1QAQ	0.88	Sc=6.15177, min distance = 2.16314·
GAMT_HUMAN Fu	65482 sir	MCES_ENCCU Ful	439155 Ad	1RI1	1ZX0	2HV9	0.88	Sc=6.60622, min distance = 1.65155:
GAMT_HUMAN Fu	65482 sir	MTR1_RHOSH Ful	439155 Ad	1NW7	1ZX0	1NW6	0.88	Sc=6.12356, min distance = 1.82587·
GAMT_RAT Ful	188380 Ad	MCES_ENCCU Ful	439155 Ad	1RI1	1XCJ	1Z3C	0.91	Sc=6.60622, min distance = 2.02008:
GAMT_RAT Ful	446535 CI	HNMT_HUMAN Ful	439155 Ad	2AOT	1XCJ	1JQE	0.95	Sc=6.56076, min distance = 2.54141(
GAMT_RAT Ful	60961 ade	PIMT_PYRFU Ful	439155 Ad	1JG1	1XCJ	1JG2	0.84	Sc=6.33194, min distance = 2.65822(
GANA_THIHE Fu	854023 Ep	Q8WSF8_APLCA I	23831 HEF	2BR7	1HJS	2BYQ		Sc=5.75071, min distance = 1.36451'
GATB_STAAM Fu	16046126	CDK2_HUMAN Ful	6022 Aden	1GY3	2G5I	2DS1		Sc=6.46315, min distance = 2.14439!
GATB_STAAM Fu	23727981	CDK2_HUMAN Ful	6022 Aden	1GY3	2G5I	3BHT		Sc=6.11627, min distance = 1.92367(

GATB_STAAM F	23727982	CDK2_HUMAN Fu	6022 Aden	1GY3	2G5I	3BHU		Sc=6.14776, min distance = 1.910745
GATB_STAAM F	447652 Im	CDK2_HUMAN Fu	6022 Aden	1GY3	2G5I	1OIQ		Sc=6.09202, min distance = 2.327913
GATB_STAAM F	448005 CI	GSK3B_HUMAN F	6022 Aden	1J1C	2G5I	1Q3W		Sc=6.35883, min distance = 2.071436
GATB_STAAM F	5287844 i	GSK3B_HUMAN F	6022 Aden	1J1C	2G5I	1UV5		Sc=6.38236, min distance = 1.807913
GATB_STAAM F	5288641 i	CDK2_HUMAN Fu	6022 Aden	1GY3	2G5I	1E9H		Sc=6.30034, min distance = 2.086745
GATB_STAAM F	5326739 i	GSK3B_HUMAN F	6022 Aden	1J1C	2G5I	1Q41		Sc=6.37366, min distance = 2.067579
GATB_STAAM F	5327148 C	IPKA_RABIT Fu	6022 Aden	1JBP	2G5I	2ERZ		Sc=6.04966, min distance = 2.392192
GATB_STAAM F	6918710 5	CDK2_HUMAN Fu	6022 Aden	1GY3	2G5I	3EJ1		Sc=6.18428, min distance = 1.674410
GATB_STAAM F	9817550 V	CDK2_HUMAN Fu	6022 Aden	1GY3	2G5I	3BHV		Sc=6.34154, min distance = 2.085157
GBA3_HUMAN F	14368760	LPXC_AQUAE Fu	985 palmi	2GO3	2E9L	2O3Z		Sc=5.95476, min distance = 1.024300
GBA3_HUMAN F	439731 al	ARAF_ECOLI Fu	439353 be	8ABP	2E9M	6ABP	0.93	Sc=5.67963, min distance = 2.412692
GBA3_HUMAN F	445238 O1	GUN2_THEFU Fu	64689 bet	2BOF	2E9L	2BOG	0.94	Sc=5.89398, min distance = 2.274761
GBA3_HUMAN F	445564 4-	GUNG_CLOCE Fu	64689 bet	1GA2	2E9L	1KFG	0.88	Sc=5.83128, min distance = 0.912872
GBA3_HUMAN F	445999 CI	Q79G13_MYCTU	64689 bet	1UP0	2E9L	1UOZ	0.77	Sc=5.77431, min distance = 0.235280
GBA3_HUMAN F	447607 1c	Q79G13_MYCTU	64689 bet	1UP0	2E9L	1UP2		Sc=5.77106, min distance = 2.685558
GBP1_HUMAN F	168120 Po	HINT1_RABIT F	6804 guan	3RHN	2B8W	5RHN	0.78	Sc=6.40049, min distance = 1.997105
GBP1_HUMAN F	188347 pp	ARF1_BOVIN Fu	8977 1dar	1R8S	2B92	1R8Q	0.99	Sc=6.52524, min distance = 1.696706
GBP1_HUMAN F	188347 pp	ARL5A_HUMAN F	8977 1dar	2H17	2B92	1ZJ6	0.99	Sc=6.52393, min distance = 1.934035
GBP1_HUMAN F	37792 gam	GNAT1_BOVIN F	8977 1dar	1TAD	2B92	1TND	0.99	Sc=6.52203, min distance = 1.776504
GBP1_HUMAN F	444335 GT	PAP2_VACCW Fu	8977 1dar	1JTF	2B92	1V39	0.99	Sc=6.49746, min distance = 1.816605
GBP1_HUMAN F	6022 Ader	PARM_ECOLX Fu	8977 1dar	2ZGY	2B92	1MWM	0.8	Sc=6.44981, min distance = 2.180385
GBP1_HUMAN F	6031 Uric	O33839_THEMA	8977 1dar	1XJE	2B92	1XJG		Sc=5.91536, min distance = 2.069647
GBP1_HUMAN F	6802 guar	DPOL_BPR69 Fu	8977 1dar	1CLQ	2B92	1IH7	0.94	Sc=6.26252, min distance = 2.034686
GBP1_HUMAN F	8582 Inos	GMPR2_HUMAN F	6804 guan	2A7R	2B8W	2C6Q	0.99	Sc=6.40532, min distance = 2.436726
GCL_ECOLI Fu	1132 thia	MDLC_PSEPU Fu	444421 CI	2FWN	2PAN	1BFD	0.82	Sc=6.38889, min distance = 2.775450
GCN2_YEAST F	11608401	KAPCA_BOVIN F	5957 Aden	1Q24	1ZYD	2UVY		Sc=6.06514, min distance = 2.586418
GCN2_YEAST F	11708454	KAPCA_BOVIN F	5957 Aden	1Q24	1ZYD	2VNW		Sc=6.10442, min distance = 2.570114
GCN2_YEAST F	129236 2c	CKI1_SCHPO Fu	5957 Aden	1CSN	1ZYD	2CSN		Sc=5.84104, min distance = 2.422225
GCN2_YEAST F	16750062	CSK2A_MAIZE F	33113 gam	1LP4	1ZY5	2OXD		Sc=5.67964, min distance = 2.441861
GCN2_YEAST F	24762195	KAPCA_BOVIN F	5957 Aden	1Q24	1ZYD	2VO0		Sc=6.24359, min distance = 2.304345
GCN2_YEAST F	24779674	CSK2A_MAIZE F	33113 gam	1LP4	1ZY5	2PVH		Sc=6.14859, min distance = 2.442181
GCN2_YEAST F	3064778 H	ROCK1_HUMAN F	33113 gam	2V55	1ZY5	2ETK		Sc=6.07747, min distance = 2.152701
GCN2_YEAST F	3547 Fasu	ROCK1_HUMAN F	33113 gam	2V55	1ZY5	2ESM		Sc=5.9169, min distance = 2.3847444
GCN2_YEAST F	3973 nche	PK3CG_PIG Ful	5957 Aden	1E8X	1ZYD	1E7V		Sc=5.91318, min distance = 2.078716
GCN2_YEAST F	444345 1c	GSK3B_HUMAN F	33113 gam	1J1B	1ZY5	1Q3D		Sc=6.3486, min distance = 2.0453090
GCN2_YEAST F	444564 AD	MYS2_DICDI Fu	5957 Aden	1FMW	1ZYD	1W9I	0.91	Sc=5.71852, min distance = 0
GCN2_YEAST F	446721 CI	ASSY_THET8 Fu	33113 gam	1KOR	1ZY5	1KH2	0.93	Sc=5.97023, min distance = 2.125376

GCN2_YEAST F	448043 2c	ROCK1_HUMAN F	33113 gam	2V55	1ZY5	3D9V		Sc=6.00452, min distance = 2.30272
GCN2_YEAST F	5326976 1	CSK2A_MAIZE F	33113 gam	1LP4	1ZY5	1ZOE		Sc=5.72082, min distance = 1.92571
GCN2_YEAST F	5326978 1	CSK2A_MAIZE F	33113 gam	1LP4	1ZY5	1ZOH		Sc=5.63557, min distance = 2.05416
GCN2_YEAST F	5327121 I	PIM1_HUMAN Fu	33113 gam	1XR1	1ZY5	2C3I		Sc=6.22266, min distance = 2.13827
GCN2_YEAST F	6022 Aden	Y059_METJA Fu	5957 Aden	2J9C	1ZYD	2J9D	0.99	Sc=5.69273, min distance = 1.92426
GCN2_YEAST F	6083 aden	AAKG1_RAT Ful	5957 Aden	2V92	1ZYD	2V8Q	0.98	Sc=5.81653, min distance = 2.09795
GCN2_YEAST F	6083 aden	ENTP2_RAT Ful	33113 gam	3CJA	1ZY5	3CJ7	0.97	Sc=5.88517, min distance = 2.05393
GCNT1_MOUSE	449462 2-	LEG7_HUMAN Fu	439353 be	4GAL	2GAM	3GAL		Sc=5.8196, min distance = 2.028024
GCP_METJA Fu	188966 dA	HSLU_ECOLI Fu	33113 gam	1E94	2VWB	1G4A	0.97	Sc=6.45183, min distance = 1.74889
GCP_METJA Fu	6083 aden	HSP71_HUMAN F	33113 gam	2E8A	2VWB	1XQS	0.97	Sc=6.39065, min distance = 1.73414
GCSPB_THET8	447104 1m	1A1C_MALDO Fu	11987735	1M4N	1WYV	1M7Y		Sc=5.92192, min distance = 2.47718
GELS_HORSE F	11175137	KAPCA_BOVIN F	5957 Aden	1Q24	2FGH	2UW7		Sc=6.15698, min distance = 2.14160
GELS_HORSE F	11608401	KAPCA_BOVIN F	5957 Aden	1Q24	2FGH	2UVY		Sc=6.22444, min distance = 2.00937
GELS_HORSE F	129236 2c	CKI1_SCHPO Fu	5957 Aden	1CSN	2FGH	2CSN		Sc=5.87696, min distance = 2.19938
GELS_HORSE F	15602982	KAPCA_BOVIN F	5957 Aden	1Q24	2FGH	2UW6		Sc=6.11684, min distance = 2.30995
GELS_HORSE F	15602983	KAPCA_BOVIN F	5957 Aden	1Q24	2FGH	2UW5		Sc=6.10814, min distance = 2.23031
GELS_HORSE F	16122635	KAPCA_BOVIN F	5957 Aden	1Q24	2FGH	2UW8		Sc=5.65282, min distance = 2.56734
GELS_HORSE F	3540 1yds	KAPCA_BOVIN F	5957 Aden	1Q24	2FGH	1YDS		Sc=5.88671, min distance = 2.02694
GELS_HORSE F	444564 AD	BIOD_ECOLI Fu	5957 Aden	1A82	2FGH	1BS1	0.91	Sc=6.39627, min distance = 2.18401
GELS_HORSE F	444564 AD	MYS2_DICDI Fu	5957 Aden	1FMW	2FGH	1W9I	0.91	Sc=6.37176, min distance = 0
GELS_HORSE F	447840 CI	PHKG1_RABIT F	5957 Aden	1QL6	2FGH	1PHK		Sc=6.42238, min distance = 2.10617
GELS_HORSE F	449240 1y	KAPCA_BOVIN F	5957 Aden	1Q24	2FGH	1YDR		Sc=5.94462, min distance = 2.09937
GELS_HORSE F	6022 Aden	CLCN5_HUMAN F	5957 Aden	2J9L	2FGH	2JA3	0.99	Sc=6.38281, min distance = 2.42368
GELS_HORSE F	6022 Aden	DDL_THET8 Ful	5957 Aden	2ZDQ	2FGH	2ZDH	0.99	Sc=6.37018, min distance = 2.04857
GELS_HORSE F	6022 Aden	HSP7F_YEAST F	5957 Aden	3D2F	2FGH	3C7N	0.99	Sc=6.41927, min distance = 2.25455
GELS_HORSE F	6022 Aden	MUTS_ECOLI Fu	5957 Aden	1W7A	2FGH	1OH7	0.99	Sc=6.39313, min distance = 2.33644
GELS_HORSE F	6022 Aden	PURK_ECOLI Fu	5957 Aden	3ETH	2FGH	3ETJ	0.99	Sc=6.39465, min distance = 1.96927
GELS_HORSE F	6022 Aden	PURP_METJA Fu	5957 Aden	2R7L	2FGH	2R7N	0.99	Sc=5.94478, min distance = 2.36083
GELS_HORSE F	6022 Aden	Q72H90_THET2	5957 Aden	2BEK	2FGH	2BEJ	0.99	Sc=6.39142, min distance = 2.13833
GELS_HORSE F	6022 Aden	REX_BACSU Ful	5957 Aden	2VT3	2FGH	2VT2	0.99	Sc=6.12062, min distance = 0.52927
GELS_HORSE F	6022 Aden	RK_BOVIN Full	5957 Aden	3C4W	2FGH	3C4Z	0.99	Sc=6.39766, min distance = 2.33352
GELS_HORSE F	6022 Aden	SECA_ECOLI Fu	5957 Aden	2FSG	2FGH	2FSI	0.99	Sc=6.38579, min distance = 2.03990
GELS_HORSE F	6083 aden	AAKG1_RAT Ful	5957 Aden	2V92	2FGH	2V8Q	0.98	Sc=6.33093, min distance = 1.55722
GELS_HORSE F	6083 aden	PURP_METJA Fu	5957 Aden	2R7L	2FGH	2R7M	0.98	Sc=5.88306, min distance = 2.19842
GELS_HORSE F	6083 aden	PURP_PYRFU Fu	5957 Aden	2R86	2FGH	2R85	0.98	Sc=6.31802, min distance = 1.97026
GELS_HORSE F	6083 aden	Y059_METJA Fu	5957 Aden	2J9C	2FGH	2J9D	0.98	Sc=6.38285, min distance = 1.23209
GELS_HORSE F	6133 uric	CCA_ARCFU Ful	5957 Aden	1R8B	2FGH	1R8C		Sc=5.92217, min distance = 0.84450

GFA1_CANAL Fu	439217 1m	GLMS_ECOLI Fu	439284 Rc	1MOR	2POC	1MOQ	41.48		Sc=5.93931, min distance = 2.164549
GFA1_CANAL Fu	6031 Uric	MGAT1_RABIT Fu	445675 UD	1FOA	2POC	2AM5		0.82	Sc=5.88758, min distance = 2.025114
GFO_ZYMMO Fu	439357 ga	FCN2_HUMAN Fu	64960 Pol	2J0Y	1RYD	2J3U		0.89	Sc=5.79727, min distance = 1.069583
GFO_ZYMMO Fu	448155 AD	ILV5_SPIOL Fu	5886 NADP	1YVE	1H6D	1QMG			Sc=6.62203, min distance = 1.867283
GFO_ZYMMO Fu	5957 Aden	MAOM_HUMAN Fu	5893 nadi	1PJ3	1EVJ	1GZ4			Sc=6.02193, min distance = 0.457203
GFO_ZYMMO Fu	79025 alp	MALE_ECOLI Fu	64960 Pol	1LAX	1RYD	1ANF		0.89	Sc=5.71587, min distance = 2.532733
GFRP_RAT Ful	134497 4-	APX_STRGR Ful	6140 L-ph	1F2P	1IS8	1TF9		0.97	Sc=5.65567, min distance = 2.305593
GFRP_RAT Ful	4369560 E	PIP_THEAC Ful	6140 L-ph	1XRM	1IS8	1XQX		0.79	Sc=5.73748, min distance = 1.888663
GFRP_RAT Ful	446237 4-	CAH2_HUMAN Fu	6140 L-ph	2FMG	1IS8	1I9L			Sc=5.86582, min distance = 1.327755
GFRP_RAT Ful	6305 L-tr	APX_STRGR Ful	6140 L-ph	1F2P	1IS8	1TF8			Sc=5.7383, min distance = 1.9836141
GFRP_RAT Ful	6305 L-tr	THER_BACTH Fu	6140 L-ph	1OS0	1IS8	1THL			Sc=5.8888, min distance = 1.9039915
GFRP_RAT Ful	9060 D-Tr	APX_STRGR Ful	6140 L-ph	1F2P	1IS8	1TKF			Sc=5.64745, min distance = 2.822723
GGGPS_ARCFU	24757942	TRPA_SALTY Fu	439276 3-	1K8Y	2F6X	3CEP			Sc=5.62129, min distance = 2.371448
GGGPS_ARCFU	439459 Hc	NIFD_KLEPN Fu	311 citri	1H1L	2F6U	1QGU		0.92	Sc=6.10613, min distance = 1.994832
GGGPS_ARCFU	439459 Hc	NIFK_KLEPN Fu	311 citri	1H1L	2F6U	1QGU		0.92	Sc=6.10379, min distance = 1.980427
GGGPS_ARCFU	439655 d-	MENF_ECOLI Fu	311 citri	3BZM	2F6U	2EUA			Sc=5.74653, min distance = 2.497615
GGGPS_ARCFU	444212 tr	ACON_BOVIN Fu	311 citri	1C96	2F6U	1ACO			Sc=5.93734, min distance = 2.045343
GGGPS_ARCFU	444305 L-	Q3XZW8_ENTFC	311 citri	2JFV	2F6U	2JFW			Sc=5.74653, min distance = 2.130406
GGGPS_ARCFU	447376 2-	ACON_BOVIN Fu	311 citri	1C96	2F6U	1NIS			Sc=5.92218, min distance = 2.161819
GGGPS_ARCFU	447536 1c	SRC_HUMAN Ful	311 citri	1O4L	2F6U	1O4Q			Sc=5.69791, min distance = 2.086879
GGGPS_ARCFU	51 2-Oxop	SERA_ECOLI Fu	311 citri	2P9E	2F6U	1YBA		0.76	Sc=5.80186, min distance = 1.555038
GGGPS_ARCFU	6102712 C	PYRB_ECOLI Fu	311 citri	1R0B	2F6U	2AIR			Sc=5.66249, min distance = 0.455136
GGPPS_YEAST	11266583	FPPS_HUMAN Fu	68740 Zol	2F9K	2E91	2OPM	30.89		Sc=6.01832, min distance = 2.409593
GGPPS_YEAST	1195 isop	ISPA_ECOLI Fu	448400 IS	1RQI	2E8U	1RQJ	26.45		Sc=5.72242, min distance = 2.447963
GGPPS_YEAST	16750060	FPPS_HUMAN Fu	68740 Zol	2F9K	2E91	2OPN	30.89		Sc=5.74109, min distance = 2.305182
GGPPS_YEAST	24764446	FPPS_HUMAN Fu	68740 Zol	2F9K	2E91	3CP6	30.89		Sc=6.06102, min distance = 2.390056
GGPPS_YEAST	445995 Ge	ISPF_ECOLI Fu	6102720 C	2GZL	2E8X	1H47			Sc=6.11541, min distance = 2.214223
GGPPS_YEAST	4674 pami	FPPS_HUMAN Fu	68740 Zol	2F9K	2E91	2F89	30.89		Sc=5.6418, min distance = 2.8590153
GGR_THEAC Fu	11987634	GLPD_ECOLI Fu	444188 CI	2QCU	3CGV	2R4E		0.97	Sc=6.86228, min distance = 2.005336
GGR_THEAC Fu	16740985	FRDA_SHEFR Fu	444188 CI	1M64	3CGV	1E39		0.93	Sc=6.85565, min distance = 2.343564
GGR_THEAC Fu	444502 CI	FRDA_SHEFR Fu	444188 CI	1M64	3CGV	1QJD		0.98	Sc=6.85729, min distance = 2.382066
GGR_THEAC Fu	444502 CI	GSHR_HUMAN Fu	444188 CI	3DK9	3CGV	1BWC		0.98	Sc=6.86228, min distance = 2.524583
GGR_THEAC Fu	444502 CI	TYTR_TRYCR Fu	444188 CI	1BZL	3CGV	1GXF		0.98	Sc=6.86538, min distance = 2.258244
GGR_THEAC Fu	446013 1,	O28603_ARCFU	444188 CI	1JNR	3CGV	1JNZ		0.94	Sc=6.86361, min distance = 2.208462
GGR_THEAC Fu	446013 1,	O28604_ARCFU	444188 CI	1JNR	3CGV	1JNZ		0.94	Sc=6.86336, min distance = 2.212884
GGR_THEAC Fu	448054 CI	FRDA_SHEFR Fu	444188 CI	1M64	3CGV	1Y0P		0.97	Sc=6.85676, min distance = 2.407418
GGR_THEAC Fu	449465 CI	GSHR_HUMAN Fu	444188 CI	3DK9	3CGV	3GRT		0.98	Sc=6.86174, min distance = 2.417268

GGR_THEAC Fu	6420174 (	O28603_ARCFU I	444188 CI	1JNR	3CGV	2FJB	0.92	Sc=6.87563, min distance = 2.21204
GGR_THEAC Fu	6420174 (	O28604_ARCFU I	444188 CI	1JNR	3CGV	2FJB	0.92	Sc=6.87481, min distance = 2.17952
GGTA1_BOVIN I	8172 Trig	O96048_LUMTE I	439353 be	2DS0	1O7Q	2DRY		Sc=5.61831, min distance = 1.09486
GGT_ECOLI Fu	449178 gl	GLMS_ECOLI Fu	33032 L-g	1XFF	2DG5	1XFG	0.81	Sc=5.84745, min distance = 1.93457
GGT_ECOLI Fu	5310984 2	GRM3_RAT Full	33032 L-g	2E4U	2DG5	2E4Y		Sc=5.96577, min distance = 1.94409
GGT_ECOLI Fu	5961 L-gl	SYQ_ECOLI Full	33032 L-g	1O0C	2DG5	1ZJW	0.84	Sc=5.74847, min distance = 2.07878
GH109_FLAME I	123927 A3	DAPB_ECOLI Fu	5893 nadi	1DRU	2IXA	1DRV	0.97	Sc=6.79879, min distance = 2.05294
GH109_FLAME I	439153 Di	ADH1B_HUMAN Fu	5893 nadi	1U3U	2IXA	1DEH	0.79	Sc=6.79666, min distance = 1.81532
GH109_FLAME I	439153 Di	ADH1E_HORSE Fu	5893 nadi	1MGO	2IXA	2JHF	0.79	Sc=6.79494, min distance = 1.99262
GH109_FLAME I	445794 AD	ADHX_HUMAN Fu	5893 nadi	2FZW	2IXA	2FZE		Sc=6.64645, min distance = 2.10249
GH109_FLAME I	446050 CI	G3P_HUMAN Full	5893 nadi	1U8F	2IXA	3GPD	0.95	Sc=6.80375, min distance = 1.54497
GH109_FLAME I	446288 NA	G3P_PALVE Full	5893 nadi	1DSS	2IXA	1IHX		Sc=6.79525, min distance = 2.27340
GH109_FLAME I	5957 Ader	MAOM_HUMAN Fu	5893 nadi	1PJ3	2IXA	1GZ4		Sc=6.5543, min distance = 1.354217
GH109_FLAME I	6022 Ader	Q5SI02_THET8 I	5893 nadi	2BJK	2IXA	2BJA		Sc=6.27712, min distance = 2.06711
GH109_FLAME I	6102710 A	Q4PRK9_PLAVI I	5893 nadi	2A92	2IXA	2AA3	0.95	Sc=6.80368, min distance = 1.87534
GLB1_CHLMO Fu	11957363	HBB_HUMAN Full	444124 HE	1NQP	1DLY	1RQA	0.81	Sc=6.7295, min distance = 2.055071
GLB1_CHLMO Fu	11957370	HMOX1_HUMAN Fu	444124 HE	1OZW	1DLY	1TWN		Sc=6.5774, min distance = 2.245286
GLB1_CHLMO Fu	11957385	MYG_PHYCA Full	444124 HE	1U7R	1DLY	2CMM		Sc=6.25259, min distance = 2.26513
GLB1_CHLMO Fu	444522 HE	HBA_PAGBE Full	444124 HE	1S5X	1DLY	1PBX	0.99	Sc=6.73907, min distance = 2.13102
GLB1_CHLMO Fu	444522 HE	MYG_PIG Full=M	444124 HE	1MYH	1DLY	1MNI	0.99	Sc=6.72536, min distance = 1.74340
GLB1_CHLMO Fu	444522 HE	NOS3_BOVIN Fu	444124 HE	1ZZS	1DLY	2HX2	0.99	Sc=6.4335, min distance = 2.278828
GLB1_GLYDI Fu	11957353	HBA_HORSE Full	444124 HE	1Y8I	1JF3	1IWH	0.87	Sc=6.59553, min distance = 2.80077
GLB1_GLYDI Fu	11957353	HBB_HORSE Full	444098 HE	2D5X	2HBG	1IWH	0.83	Sc=6.5878, min distance = 2.574130
GLB1_GLYDI Fu	11957353	HBB_HORSE Full	444124 HE	1Y8I	1JF3	1IWH	0.87	Sc=6.61151, min distance = 2.42405
GLB1_GLYDI Fu	11957353	HBB_HORSE Full	444522 HE	1Y8K	1HBG	1IWH	0.87	Sc=6.60121, min distance = 2.17605
GLB1_GLYDI Fu	11957363	HBB_HUMAN Full	444098 HE	1J40	2HBG	1RQA	0.77	Sc=6.57834, min distance = 2.41907
GLB1_GLYDI Fu	11957363	HBB_HUMAN Full	444522 HE	2DN1	1HBG	1RQA	0.81	Sc=6.58727, min distance = 2.07566
GLB1_GLYDI Fu	11957371	HMOX1_HUMAN Fu	444124 HE	1OZW	1JF3	1TWR		Sc=6.37141, min distance = 2.43742
GLB1_GLYDI Fu	11957385	MYG_PHYCA Full	444098 HE	1A6M	2HBG	2CMM		Sc=5.90982, min distance = 2.51800
GLB1_GLYDI Fu	11957385	MYG_PHYCA Full	444124 HE	1U7R	1JF3	2CMM		Sc=5.92573, min distance = 2.01032
GLB1_GLYDI Fu	11957385	MYG_PHYCA Full	444522 HE	1VXD	1HBG	2CMM		Sc=5.90727, min distance = 2.29266
GLB1_GLYDI Fu	4369001 (	HBA_HUMAN Full	444124 HE	1NQP	1JF3	4HHB	0.8	Sc=6.40976, min distance = 2.32779
GLB1_LUCPE Fu	11957385	MYG_PHYCA Full	444098 HE	1A6M	1FLP	2CMM		Sc=6.17186, min distance = 2.27120
GLB1_LUCPE Fu	16741062	MYG_PHYCA Full	444098 HE	1A6M	1FLP	1MBN	0.84	Sc=6.36828, min distance = 2.07575
GLB1_SCAIN Fu	11957330	CCPR_YEAST Fu	444044 CI	1DSE	2R4X	1BEQ	0.84	Sc=6.56315, min distance = 2.35804
GLB1_SCAIN Fu	11957353	HBA_HORSE Full	444124 HE	1Y8I	1JZL	1IWH	0.87	Sc=6.60568, min distance = 1.97438
GLB1_SCAIN Fu	11957363	HBA_HUMAN Full	444124 HE	1NQP	1JZL	1RQA	0.81	Sc=6.59134, min distance = 2.12912

GLB1_SCAIN F	11957363	HBB_HUMAN Ful	444098 HE	1J40	2Z8A	1RQA		0.77	Sc=6.65939, min distance = 2.02873
GLB1_SCAIN F	11957363	HBB_HUMAN Ful	444124 HE	1NQP	1JZL	1RQA		0.81	Sc=6.59851, min distance = 2.07904
GLB1_SCAIN F	11957364	HMOX1_HUMAN F	444124 HE	1OZW	1JZL	1S13		0.86	Sc=6.5361, min distance = 1.871897
GLB1_SCAIN F	11957370	HMOX1_HUMAN F	444124 HE	1OZW	1JZL	1TWN			Sc=6.46696, min distance = 2.27331
GLB1_SCAIN F	11957371	HMOX1_HUMAN F	444124 HE	1OZW	1JZL	1TWR			Sc=6.46734, min distance = 2.29294
GLB1_SCAIN F	11957385	MYG_PHYCA Ful	444098 HE	1A6M	2Z8A	2CMM	26.72		Sc=6.08572, min distance = 2.07759
GLB1_SCAIN F	11957385	MYG_PHYCA Ful	444124 HE	1U7R	1JZL	2CMM	26.72		Sc=6.06752, min distance = 2.23643
GLB1_SCAIN F	11970242	CCPR_YEAST Fu	444044 CI	1DSE	2R4X	1CPE		0.87	Sc=6.57035, min distance = 2.11557
GLB1_SCAIN F	126994 CI	CCPR_YEAST Fu	444044 CI	1DSE	2R4X	1Z53		0.8	Sc=6.62866, min distance = 2.19453
GLB1_SCAIN F	16741061	MYG_PHYCA Ful	444098 HE	1A6M	2Z8A	1MBC	26.72	0.83	Sc=6.67764, min distance = 2.11343
GLB1_SCAIN F	16741062	MYG_PHYCA Ful	444124 HE	1U7R	1JZL	1MBN	26.72	0.87	Sc=6.65348, min distance = 2.16996
GLB1_SCAIN F	4369122 C	GLB1_LUCPE Fu	444098 HE	1FLP	2Z8A	1B0B		0.96	Sc=6.68254, min distance = 2.07961
GLB1_SCAIN F	444097 He	HBA_HUMAN Ful	444124 HE	1NQP	1JZL	1RPS		0.95	Sc=6.60224, min distance = 2.39651
GLB1_SCAIN F	444522 HE	BFR_ECOLI Ful	444124 HE	1BFR	1JZL	1BCF		0.99	Sc=6.66648, min distance = 1.69561
GLB1_SCAIN F	444522 HE	MYG_PIG Full=	444124 HE	1MYH	1JZL	1MNI		0.99	Sc=6.66531, min distance = 2.05164
GLB1_SCAIN F	446348 CI	HBA_HUMAN Ful	444124 HE	1NQP	1JZL	1IRD		0.9	Sc=6.5961, min distance = 2.093869
GLB2A_SCAIN	11957353	HBA_HORSE Ful	444124 HE	1Y8I	1SCT	1IWH		0.87	Sc=6.60677, min distance = 2.15447
GLB2A_SCAIN	11957370	HMOX1_HUMAN F	444124 HE	1OZW	1SCT	1TWN			Sc=6.47098, min distance = 2.29460
GLB2A_SCAIN	11957371	HMOX1_HUMAN F	444124 HE	1OZW	1SCT	1TWR			Sc=6.46995, min distance = 2.51109
GLB2A_SCAIN	11957373	CATE_ECOLI Fu	444124 HE	1IPH	1SCT	1P81		0.79	Sc=6.58061, min distance = 2.17705
GLB2A_SCAIN	11957385	MYG_PHYCA Ful	444124 HE	1U7R	1SCT	2CMM			Sc=6.07102, min distance = 2.38303
GLB2A_SCAIN	16741062	MYG_PHYCA Ful	444124 HE	1U7R	1SCT	1MBN		0.87	Sc=6.65779, min distance = 2.26095
GLB2A_SCAIN	444522 HE	CP2C9_HUMAN F	444124 HE	1OG2	1SCT	1R9O		0.99	Sc=6.63618, min distance = 2.00341
GLB2A_SCAIN	444522 HE	GLB1_GLYDI Fu	444124 HE	1JF3	1SCT	1HBG		0.99	Sc=6.62212, min distance = 2.64538
GLB2A_SCAIN	444522 HE	HBA_PAGBE Ful	444124 HE	1S5X	1SCT	1PBX		0.99	Sc=6.60172, min distance = 2.25381
GLB2A_SCAIN	444522 HE	MYG_PIG Full=	444124 HE	1MYH	1SCT	1MNI		0.99	Sc=6.67592, min distance = 2.22437
GLB2B_SCAIN	11957353	HBA_HORSE Ful	444124 HE	1Y8I	1SCT	1IWH		0.87	Sc=6.6039, min distance = 2.185491
GLB2B_SCAIN	11957370	HMOX1_HUMAN F	444124 HE	1OZW	1SCT	1TWN			Sc=6.46492, min distance = 2.38191
GLB2B_SCAIN	11957371	HMOX1_HUMAN F	444124 HE	1OZW	1SCT	1TWR			Sc=6.4608, min distance = 2.319787
GLB2B_SCAIN	11957385	MYG_PHYCA Ful	444124 HE	1U7R	1SCT	2CMM			Sc=6.0605, min distance = 2.343810
GLB2B_SCAIN	16214774	CP51_MYCTU Fu	444124 HE	2CIB	1SCT	2CI0		0.95	Sc=6.35745, min distance = 2.30466
GLB2B_SCAIN	16741062	MYG_PHYCA Ful	444124 HE	1U7R	1SCT	1MBN		0.87	Sc=6.64871, min distance = 2.25549
GLB2B_SCAIN	444207 He	COX1_THET8 Fu	444124 HE	1EHK	1SCT	2QPE		0.99	Sc=6.58829, min distance = 2.21109
GLB2B_SCAIN	444522 HE	GLB1_GLYDI Fu	444124 HE	1JF3	1SCT	1HBG		0.99	Sc=6.63331, min distance = 2.51157
GLB2B_SCAIN	444522 HE	HBA_PAGBE Ful	444124 HE	1S5X	1SCT	1PBX		0.99	Sc=6.59022, min distance = 2.30113
GLB2B_SCAIN	444522 HE	MYG_PIG Full=	444124 HE	1MYH	1SCT	1MNI		0.99	Sc=6.66271, min distance = 2.23677
GLB2_LUCPE F	11957385	MYG_PHYCA Ful	444098 HE	1A6M	2OLP	2CMM			Sc=6.20282, min distance = 2.20032

GLB2_LUMTE F	11957363	HBB_HUMAN Ful	444522 HE	2DN1	1X9F	1RQA	0.81	Sc=6.62445, min distance = 2.33691
GLB2_LUMTE F	11957365	CCPR_YEAST Fu	444522 HE	3E2O	1X9F	1S73	0.83	Sc=6.7088, min distance = 2.587332
GLB2_LUMTE F	11957385	MYG_PHYCA Ful	444522 HE	1VXD	1X9F	2CMM		Sc=6.06387, min distance = 2.203826
GLB2_LUMTE F	11970221	CCPR_YEAST Fu	444522 HE	3E2O	1X9F	1BES	0.87	Sc=6.64631, min distance = 2.524098
GLB2_LUMTE F	126994 CI	CCPR_YEAST Fu	444522 HE	3E2O	1X9F	1Z53	0.81	Sc=6.59264, min distance = 2.546010
GLB2_LUMTE F	16741183	MYG_PHYCA Ful	444522 HE	1VXD	1X9F	2EKT	0.93	Sc=6.3982, min distance = 2.175118
GLB2_LUMTE F	4369228 C	PER_COPCI Ful	444522 HE	1H3J	1X9F	1LYC	0.96	Sc=6.65504, min distance = 2.424186
GLB2_LUMTE F	444097 He	MYG_PHYCA Ful	444522 HE	1VXD	1X9F	2EVK	0.96	Sc=6.62757, min distance = 2.030354
GLB2_LUMTE F	447168 ZE	CCPR_YEAST Fu	444522 HE	3E2O	1X9F	1ML2	0.86	Sc=6.7023, min distance = 2.503924
GLB3_CHITH F	11957385	MYG_PHYCA Ful	444098 HE	1A6M	1ECA	2CMM		Sc=5.92329, min distance = 2.51056
GLB3_CHITH F	16741183	CPXA_PSEPU Fu	444098 HE	1YRC	1ECA	2ZAW	0.97	Sc=6.47493, min distance = 2.57952
GLB4_GLYDI F	11957353	HBB_HORSE Ful	444124 HE	1Y8I	1JF4	1IWH	0.87	Sc=6.60932, min distance = 2.01154
GLB4_GLYDI F	11957364	HMOX1_HUMAN F	444124 HE	1OZW	1JF4	1S13	0.86	Sc=6.4265, min distance = 2.050679
GLB4_GLYDI F	11957370	HMOX1_HUMAN F	444124 HE	1OZW	1JF4	1TWN		Sc=6.36043, min distance = 1.67883
GLB4_GLYDI F	11957371	HMOX1_HUMAN F	444124 HE	1OZW	1JF4	1TWR		Sc=6.36935, min distance = 2.43040
GLB4_GLYDI F	11957385	MYG_PHYCA Ful	444124 HE	1U7R	1JF4	2CMM		Sc=5.92573, min distance = 2.06125
GLB4_GLYDI F	4369001 C	HBA_HUMAN Ful	444124 HE	1NQP	1JF4	4HHB	0.8	Sc=6.41392, min distance = 2.16336
GLB4_GLYDI F	444522 HE	BFR_ECOLI Ful	444124 HE	1BFR	1JF4	1BCF	0.99	Sc=6.59264, min distance = 2.51141
GLB4_LUMTE F	11957360	CYB_BOVIN Ful	444522 HE	1BGY	1X9F	1NTM	0.78	Sc=6.63676, min distance = 1.61540
GLB4_LUMTE F	11957361	CYB_BOVIN Ful	444522 HE	1BGY	1X9F	1NTZ	0.84	Sc=6.6416, min distance = 1.904514
GLB4_LUMTE F	11957363	HBA_HUMAN Ful	444522 HE	2DN1	1X9F	1RQA	0.81	Sc=6.58549, min distance = 2.13363
GLB4_LUMTE F	11957363	HBB_HUMAN Ful	444522 HE	2DN1	1X9F	1RQA	0.81	Sc=6.60282, min distance = 2.14980
GLB4_LUMTE F	11957385	MYG_PHYCA Ful	444522 HE	1VXD	1X9F	2CMM		Sc=5.91556, min distance = 2.02811
GLB4_LUMTE F	11970219	CCPR_YEAST Fu	444522 HE	3E2O	1X9F	1BEM	0.87	Sc=6.57437, min distance = 2.44837
GLB4_LUMTE F	11970221	CCPR_YEAST Fu	444522 HE	3E2O	1X9F	1BES	0.87	Sc=6.62672, min distance = 2.42720
GLB4_LUMTE F	11970242	CCPR_YEAST Fu	444522 HE	3E2O	1X9F	1CPE	0.87	Sc=6.57496, min distance = 2.27875
GLB4_LUMTE F	126994 CI	CCPR_YEAST Fu	444522 HE	3E2O	1X9F	1Z53	0.81	Sc=6.5777, min distance = 2.288778
GLB4_LUMTE F	16741183	MYG_PHYCA Ful	444522 HE	1VXD	1X9F	2EKT	0.93	Sc=6.37326, min distance = 2.00360
GLB4_LUMTE F	447168 ZE	CCPR_YEAST Fu	444522 HE	3E2O	1X9F	1ML2	0.86	Sc=6.63503, min distance = 2.15068
GLBA1_OLIMA	11957330	CCPR_YEAST Fu	444098 HE	2EUT	2ZS0	1BEQ	0.81	Sc=6.64277, min distance = 2.33953
GLBA1_OLIMA	11957355	CY1_BOVIN Ful	444098 HE	1L0N	2ZS0	1L0L	0.77	Sc=6.59485, min distance = 2.04359
GLBA1_OLIMA	11957361	CY1_BOVIN Ful	444098 HE	1L0N	2ZS0	1NTZ	0.8	Sc=6.58727, min distance = 2.18424
GLBA1_OLIMA	11957363	HBB_HUMAN Ful	444098 HE	1J40	2ZS0	1RQA	0.77	Sc=6.60719, min distance = 1.97132
GLBA1_OLIMA	11957385	MYG_PHYCA Ful	444098 HE	1A6M	2ZS0	2CMM		Sc=6.05614, min distance = 2.06599
GLBA1_OLIMA	11970219	CCPR_YEAST Fu	444098 HE	2EUT	2ZS0	1BEM	0.83	Sc=6.58061, min distance = 2.31972
GLBA1_OLIMA	11970221	CCPR_YEAST Fu	444098 HE	2EUT	2ZS0	1BES	0.83	Sc=6.63503, min distance = 2.39435
GLBA1_OLIMA	11970242	CCPR_YEAST Fu	444098 HE	2EUT	2ZS0	1CPE	0.83	Sc=6.58006, min distance = 2.23696

GLBA1_OLIMA I	126994 CI	CCPR_YEAST Fu	444098 HE 2EUT	2ZS0	1Z53		0.77	Sc=6.57834, min distance = 2.26603
GLBA1_OLIMA I	4369122 C	GLB1_LUCPE Fu	444098 HE 1FLP	2ZS0	1B0B		0.96	Sc=6.61486, min distance = 2.06024
GLBA1_OLIMA I	447168 ZE	CCPR_YEAST Fu	444098 HE 2EUT	2ZS0	1ML2		0.82	Sc=6.64046, min distance = 2.34004
GLBA2_OLIMA I	11957330	CCPR_YEAST Fu	444098 HE 2EUT	2ZS0	1BEQ		0.81	Sc=6.64982, min distance = 2.35728
GLBA2_OLIMA I	11957363	HBB_HUMAN Ful	444098 HE 1J40	2ZS0	1RQA		0.77	Sc=6.67654, min distance = 1.95043
GLBA2_OLIMA I	11957385	MYG_PHYCA Ful	444098 HE 1A6M	2ZS0	2CMM			Sc=6.20064, min distance = 2.05755
GLBA2_OLIMA I	11970219	CCPR_YEAST Fu	444098 HE 2EUT	2ZS0	1BEM		0.83	Sc=6.65348, min distance = 2.33734
GLBA2_OLIMA I	11970221	CCPR_YEAST Fu	444098 HE 2EUT	2ZS0	1BES		0.83	Sc=6.65107, min distance = 2.41740
GLBA2_OLIMA I	11970242	CCPR_YEAST Fu	444098 HE 2EUT	2ZS0	1CPE		0.83	Sc=6.65233, min distance = 2.24922
GLBA2_OLIMA I	126994 CI	CCPR_YEAST Fu	444098 HE 2EUT	2ZS0	1Z53		0.77	Sc=6.60007, min distance = 2.72091
GLBA2_OLIMA I	4369122 C	GLB1_LUCPE Fu	444098 HE 1FLP	2ZS0	1B0B		0.96	Sc=6.73133, min distance = 2.07765
GLBA2_OLIMA I	447168 ZE	CCPR_YEAST Fu	444098 HE 2EUT	2ZS0	1ML2		0.82	Sc=6.70783, min distance = 2.35567
GLBB1_OLIMA I	11957363	HBB_HUMAN Ful	444098 HE 1J40	2ZS0	1RQA		0.77	Sc=6.66429, min distance = 1.92169
GLBB1_OLIMA I	4369122 C	GLB1_LUCPE Fu	444098 HE 1FLP	2ZS0	1B0B		0.96	Sc=6.59851, min distance = 2.07089
GLBB2_OLIMA I	11957360	CY1_BOVIN Ful	444098 HE 1L0N	2ZS0	1NTM			Sc=6.62811, min distance = 2.03157
GLBB2_OLIMA I	11957363	HBB_HUMAN Ful	444098 HE 1J40	2ZS0	1RQA		0.77	Sc=6.63561, min distance = 1.94516
GLBB2_OLIMA I	11957385	MYG_PHYCA Ful	444098 HE 1A6M	2ZS0	2CMM			Sc=6.09192, min distance = 2.07251
GLBB2_OLIMA I	16741061	MYG_PHYCA Ful	444098 HE 1A6M	2ZS0	1MBC		0.83	Sc=6.69247, min distance = 2.10762
GLBB2_OLIMA I	4369122 C	GLB1_LUCPE Fu	444098 HE 1FLP	2ZS0	1B0B		0.96	Sc=6.63656, min distance = 2.06307
GLBC_CAUAR Fu	11957353	HBB_HORSE Ful	444124 HE 1Y8I	1HLB	1IWH		0.87	Sc=6.69994, min distance = 1.75770
GLBC_CAUAR Fu	444207 He	CP119_SULTO Fu	444124 HE 3B4X	1HLB	1UE8		0.99	Sc=6.69716, min distance = 1.44069
GLBD_CAUAR Fu	11957353	HBA_HORSE Ful	444124 HE 1Y8I	1HLM	1IWH		0.87	Sc=6.55553, min distance = 2.12197
GLBD_CAUAR Fu	11957363	HBA_HUMAN Ful	444124 HE 1NQP	1HLM	1RQA		0.81	Sc=6.53331, min distance = 2.30862
GLBD_CAUAR Fu	11957363	HBB_HUMAN Ful	444124 HE 1NQP	1HLM	1RQA		0.81	Sc=6.53725, min distance = 1.69794
GLBD_CAUAR Fu	11957364	HMOX1_HUMAN Fu	444124 HE 1OZW	1HLM	1S13		0.86	Sc=6.37566, min distance = 2.16446
GLBD_CAUAR Fu	11957370	HMOX1_HUMAN Fu	444124 HE 1OZW	1HLM	1TWN			Sc=6.30272, min distance = 2.49002
GLBD_CAUAR Fu	11957373	CATE_ECOLI Fu	444124 HE 1IPH	1HLM	1P81		0.79	Sc=6.53648, min distance = 2.01596
GLBD_CAUAR Fu	11957385	MYG_PHYCA Ful	444124 HE 1U7R	1HLM	2CMM			Sc=5.83442, min distance = 2.37377
GLBD_CAUAR Fu	16214774	CP51_MYCTU Fu	444124 HE 2CIB	1HLM	2CI0		0.95	Sc=6.31743, min distance = 2.01892
GLBD_CAUAR Fu	444207 He	HBB_HORSE Ful	444124 HE 1Y8I	1HLM	2ZLW		0.99	Sc=6.5575, min distance = 1.759778
GLBD_CAUAR Fu	444522 HE	HBB_PAGBE Ful	444124 HE 1S5X	1HLM	1PBX		0.99	Sc=6.56581, min distance = 1.99540
GLBF1_EPTBU I	11957385	MYG_PHYCA Ful	444124 HE 1U7R	1IT2	2CMM			Sc=6.09395, min distance = 2.68056
GLBF1_EPTBU I	16741061	MYG_PHYCA Ful	444124 HE 1U7R	1IT2	1MBC		0.87	Sc=6.67401, min distance = 2.21235
GLBF1_EPTBU I	444522 HE	MYG_PIG Full=N	444124 HE 1MYH	1IT2	1MNI		0.99	Sc=6.67592, min distance = 2.05290
GLBN_CERLA Fu	11957363	HBB_HUMAN Ful	444098 HE 1J40	1KR7	1RQA		0.77	Sc=6.70172, min distance = 1.99604
GLBN_CERLA Fu	11957385	MYG_PHYCA Ful	444098 HE 1A6M	1KR7	2CMM			Sc=6.18438, min distance = 2.09709
GLBN_MYCTU Fu	11957330	CCPR_YEAST Fu	444098 HE 2EUT	2GKM	1BEQ		0.81	Sc=6.71541, min distance = 2.38614

GLBN_MYCTU Fu	11957353	HBB_HORSE Ful	444098 HE	2D5X	2GKM	1IWH	0.83	Sc=6.72985, min distance = 2.007005
GLBN_MYCTU Fu	11957360	CY1_BOVIN Ful	444098 HE	1L0N	2GKM	1NTM		Sc=6.67401, min distance = 1.851602
GLBN_MYCTU Fu	11957385	MYG_PHYCA Ful	444098 HE	1A6M	2GKM	2CMM		Sc=6.26518, min distance = 2.038795
GLBN_MYCTU Fu	11970219	CCPR_YEAST Fu	444098 HE	2EUT	2GKM	1BEM	0.83	Sc=6.717, min distance = 2.36431216
GLBN_MYCTU Fu	11970221	CCPR_YEAST Fu	444098 HE	2EUT	2GKM	1BES	0.83	Sc=6.61653, min distance = 2.452664
GLBN_MYCTU Fu	11970242	CCPR_YEAST Fu	444098 HE	2EUT	2GKM	1CPE	0.83	Sc=6.71541, min distance = 2.273615
GLBN_MYCTU Fu	126994 CI	CCPR_YEAST Fu	444098 HE	2EUT	2GKM	1Z53	0.77	Sc=6.71216, min distance = 2.886073
GLBN_MYCTU Fu	4369122 C	GLB1_LUCPE Fu	444098 HE	1FLP	2GKM	1B0B	0.96	Sc=6.73025, min distance = 2.027361
GLBN_SYNY3 Fu	16741061	MYG_PHYCA Ful	444098 HE	1A6M	1S69	1MBC	0.83	Sc=6.76011, min distance = 1.999436
GLBO_MYCTU Fu	11957385	MYG_PHYCA Ful	444098 HE	1A6M	2QRW	2CMM		Sc=6.05493, min distance = 2.220573
GLBO_MYCTU Fu	15342951	ALBU_HUMAN Fu	444098 HE	1N5U	2QRW	3B9L		Sc=6.2103, min distance = 1.8653581
GLBO_MYCTU Fu	16741061	MYG_PHYCA Ful	444098 HE	1A6M	2QRW	1MBC	0.83	Sc=6.69537, min distance = 2.060086
GLBO_MYCTU Fu	4369122 C	GLB1_LUCPE Fu	444098 HE	1FLP	2QRW	1B0B	0.96	Sc=6.692, min distance = 2.06193622
GLB_APLLI Fu	11957385	MYG_PHYCA Ful	444098 HE	1A6M	1MBA	2CMM		Sc=5.93151, min distance = 2.501295
GLB_APLLI Fu	11957385	MYG_PHYCA Ful	444207 He	1VXG	3MBA	2CMM		Sc=6.2472, min distance = 2.0406320
GLB_APLLI Fu	16741183	MYG_PHYCA Ful	444207 He	1VXG	3MBA	2EKT	0.93	Sc=6.60821, min distance = 2.095498
GLB_ASCSU Fu	11957385	MYG_PHYCA Ful	444124 HE	1U7R	1ASH	2CMM		Sc=6.16957, min distance = 2.332681
GLB_LAMFL Fu	11957330	CCPR_YEAST Fu	444098 HE	2EUT	1UC3	1BEQ	0.81	Sc=6.68447, min distance = 2.619446
GLB_LAMFL Fu	11957360	CY1_BOVIN Ful	444098 HE	1L0N	1UC3	1NTM		Sc=6.68732, min distance = 2.214864
GLB_LAMFL Fu	11957365	CCPR_YEAST Fu	444098 HE	2EUT	1UC3	1S73	0.8	Sc=6.74273, min distance = 2.376046
GLB_LAMFL Fu	11957385	MYG_PHYCA Ful	444098 HE	1A6M	1UC3	2CMM		Sc=5.97514, min distance = 2.358279
GLB_LAMFL Fu	11970219	CCPR_YEAST Fu	444098 HE	2EUT	1UC3	1BEM	0.83	Sc=6.68308, min distance = 2.544391
GLB_LAMFL Fu	11970220	CCPR_YEAST Fu	444098 HE	2EUT	1UC3	1BEP	0.8	Sc=6.71952, min distance = 2.630929
GLB_LAMFL Fu	11970221	CCPR_YEAST Fu	444098 HE	2EUT	1UC3	1BES	0.83	Sc=6.68395, min distance = 2.603030
GLB_LAMFL Fu	11970222	CCPR_YEAST Fu	444098 HE	2EUT	1UC3	1BJ9	0.77	Sc=6.77628, min distance = 2.551801
GLB_LAMFL Fu	11970242	CCPR_YEAST Fu	444098 HE	2EUT	1UC3	1CPE	0.83	Sc=6.68732, min distance = 2.483740
GLB_LAMFL Fu	126994 CI	CCPR_YEAST Fu	444098 HE	2EUT	1UC3	1Z53	0.77	Sc=6.68775, min distance = 2.777057
GLB_LAMFL Fu	16741061	MYG_PHYCA Ful	444098 HE	1A6M	1UC3	1MBC	0.83	Sc=6.6966, min distance = 2.0876062
GLB_LAMFL Fu	24883662	MYG_HORSE Ful	444098 HE	2FRF	1UC3	1YMC	0.82	Sc=6.69862, min distance = 1.999295
GLB_LAMFL Fu	4369122 C	GLB1_LUCPE Fu	444098 HE	1FLP	1UC3	1B0B	0.96	Sc=6.70339, min distance = 2.298488
GLB_LAMFL Fu	4369136 C	GLB_APLLI Ful	444098 HE	1MBA	1UC3	1DM1	0.93	Sc=6.6715, min distance = 2.1293419
GLB_LAMFL Fu	447168 ZE	CCPR_YEAST Fu	444098 HE	2EUT	1UC3	1ML2	0.82	Sc=6.74224, min distance = 2.698275
GLB_PARCA Fu	11957353	HBA_HORSE Ful	444124 HE	1Y8I	1UVY	1IWH	0.87	Sc=6.70573, min distance = 2.690911
GLB_PARCA Fu	11957364	HMOX1_HUMAN Fu	444124 HE	1OZW	1UVY	1S13	0.86	Sc=6.6507, min distance = 1.5903031
GLB_PARCA Fu	11957370	HMOX1_HUMAN Fu	444124 HE	1OZW	1UVY	1TWN		Sc=6.59822, min distance = 1.582815
GLB_PARCA Fu	11957371	HMOX1_HUMAN Fu	444124 HE	1OZW	1UVY	1TWR		Sc=6.54896, min distance = 2.120515
GLB_PARCA Fu	11957385	MYG_PHYCA Ful	444098 HE	1A6M	1DLW	2CMM		Sc=6.25508, min distance = 2.420937

GLB_PARCA Fu	11957385	MYG_PHYCA Ful	444124 HE	1U7R	1UVY	2CMM		Sc=6.27112, min distance = 2.29300
GLB_PARCA Fu	4369001 C	HBA_HUMAN Ful	444124 HE	1NQP	1UVY	4HHB	0.8	Sc=6.64454, min distance = 2.62027
GLB_PAREP Fu	11957353	HBB_HORSE Ful	444098 HE	2D5X	1H97	1IWH	0.83	Sc=6.617, min distance = 2.0322996
GLB_PAREP Fu	11957385	MYG_PHYCA Ful	444098 HE	1A6M	1H97	2CMM		Sc=6.05263, min distance = 2.26784
GLCM_HUMAN F	11957431	BGLA_THEMA Fu	447607 1c	1OIF	2NSX	2J7D		Sc=6.24356, min distance = 2.22662
GLCM_HUMAN F	11957432	BGLA_THEMA Fu	447607 1c	1OIF	2NSX	2J7E		Sc=6.27368, min distance = 2.06316
GLCM_HUMAN F	11957433	BGLA_THEMA Fu	447607 1c	1OIF	2NSX	2J7F		Sc=6.22953, min distance = 1.64459
GLCM_HUMAN F	11957434	BGLA_THEMA Fu	447607 1c	1OIF	2NSX	2J7G		Sc=6.30972, min distance = 1.87729
GLCM_HUMAN F	11957435	BGLA_THEMA Fu	447607 1c	1OIF	2NSX	2J7H		Sc=5.72054, min distance = 2.55935
GLCM_HUMAN F	124434 Ca	BGLA_THEMA Fu	447607 1c	1OIF	2NSX	2CBV		Sc=5.9018, min distance = 2.640081
GLCM_HUMAN F	447413 NT	BGLA_THEMA Fu	447607 1c	1OIF	2NSX	2J7B		Sc=6.00663, min distance = 1.95259
GLCM_HUMAN F	447651 CI	BGLA_THEMA Fu	447607 1c	1OIF	2NSX	2J77		Sc=5.84504, min distance = 2.52110
GLCM_HUMAN F	448963 2j	BGLA_THEMA Fu	447607 1c	1OIF	2NSX	2J79		Sc=6.0025, min distance = 1.890661
GLCM_HUMAN F	449101 nc	BGLA_THEMA Fu	447607 1c	1OIF	2NSX	1W3J	0.76	Sc=5.65485, min distance = 2.55036
GLDA_BACST F	4369261 C	ARO1_EMENI Fu	5893 nadi	1SG6	1JQ5	1NRX	0.86	Sc=6.40081, min distance = 2.22664
GLF1_KLEPN F	16740985	FRDA_SHEFR Fu	444188 CI	1M64	2BI7	1E39	0.93	Sc=6.16443, min distance = 2.14653
GLF1_KLEPN F	444502 CI	FRDA_SHEFR Fu	444188 CI	1M64	2BI7	1QJD	0.98	Sc=6.88931, min distance = 2.11941
GLF1_KLEPN F	449465 CI	GSHR_HUMAN Fu	444188 CI	3DK9	2BI7	3GRT	0.98	Sc=6.4433, min distance = 2.037618
GLF_ECOLI Fu	11987634	GLPD_ECOLI Fu	444188 CI	2QCU	1I8T	2R4E	0.97	Sc=6.87563, min distance = 2.06718
GLF_ECOLI Fu	16740985	FRDA_SHEFR Fu	444188 CI	1M64	1I8T	1E39	0.93	Sc=6.88106, min distance = 1.84936
GLF_ECOLI Fu	444502 CI	FRDA_SHEFR Fu	444188 CI	1M64	1I8T	1QJD	0.98	Sc=6.88135, min distance = 1.85504
GLF_ECOLI Fu	446013 1,	O28603_ARCFU	444188 CI	1JNR	1I8T	1JNZ	0.94	Sc=6.88165, min distance = 2.09605
GLF_ECOLI Fu	446013 1,	O28604_ARCFU	444188 CI	1JNR	1I8T	1JNZ	0.94	Sc=6.88241, min distance = 2.15687
GLF_ECOLI Fu	449465 CI	GSHR_HUMAN Fu	444188 CI	3DK9	1I8T	3GRT	0.98	Sc=6.88106, min distance = 2.21388
GLF_ECOLI Fu	6420174 C	O28603_ARCFU	444188 CI	1JNR	1I8T	2FJB	0.92	Sc=6.88631, min distance = 1.74576
GLF_ECOLI Fu	6420174 C	O28604_ARCFU	444188 CI	1JNR	1I8T	2FJB	0.92	Sc=6.88583, min distance = 2.10751
GLGA1_AGRTU	36735 Gpp	PARM_ECOLX Fu	6022 Aden	1MWM	1RZU	2ZGZ	0.79	Sc=6.00979, min distance = 2.02706
GLGA1_AGRTU	4369136 C	CDK2_HUMAN Fu	6022 Aden	1GY3	1RZU	1DM2		Sc=6.29175, min distance = 2.04371
GLGA1_AGRTU	444564 AD	BIOD_ECOLI Fu	6022 Aden	1DAD	1RZU	1BS1	0.91	Sc=5.88226, min distance = 2.71602
GLGA1_AGRTU	445940 O6	CDK2_HUMAN Fu	6022 Aden	1GY3	1RZU	1GZ8		Sc=6.1844, min distance = 2.282408
GLGA1_AGRTU	445966 O6	CDK2_HUMAN Fu	6022 Aden	1GY3	1RZU	1H0V		Sc=6.30512, min distance = 0.86696
GLGA1_AGRTU	447955 1p	CDK2_HUMAN Fu	6022 Aden	1GY3	1RZU	1PXI		Sc=5.77546, min distance = 2.40189
GLGA1_AGRTU	5288641 i	CDK2_HUMAN Fu	6022 Aden	1GY3	1RZU	1E9H		Sc=6.30541, min distance = 1.61733
GLGA1_AGRTU	5327148 C	IPKA_RABIT Fu	6022 Aden	1JBP	1RZU	2ERZ		Sc=6.16716, min distance = 1.98974
GLGA1_AGRTU	5957 Aden	ARSA1_ECOLX F	6022 Aden	1IHU	1RZU	1II0	0.99	Sc=6.40964, min distance = 2.36820
GLGA1_AGRTU	5957 Aden	BIOD_ECOLI Fu	6022 Aden	1DAD	1RZU	1A82	0.99	Sc=6.3782, min distance = 2.735947
GLGA1_AGRTU	5957 Aden	MUTS_ECOLI Fu	6022 Aden	1OH7	1RZU	1W7A	0.99	Sc=6.00225, min distance = 1.79455

GLGA1_AGRTU I	5957 Ader	NIFH1_AZOVI Fu	6022 Aden	1FP6	1RZU	2C8V	0.99	Sc=6.39499, min distance = 2.69975(
GLGA1_AGRTU I	5957 Ader	Q72H90_THET2 I	6022 Aden	2BEJ	1RZU	2BEK	0.99	Sc=6.3978, min distance = 2.8807585
GLGA1_AGRTU I	5957 Ader	Y059_METJA Ful	6022 Aden	2J9D	1RZU	2J9C	0.99	Sc=5.96896, min distance = 1.832151
GLGA1_AGRTU I	6031 Uric	DCK_HUMAN Ful	6022 Aden	1P5Z	1RZU	2ZIA		Sc=6.27083, min distance = 2.249997
GLGA1_AGRTU I	6083 ader	PURP_METJA Ful	6022 Aden	2R7N	1RZU	2R7M	0.99	Sc=5.83918, min distance = 2.450849
GLGA1_AGRTU I	6132 Cyti	RIR1_YEAST Ful	6022 Aden	2CVX	1RZU	2CVU		Sc=5.77898, min distance = 2.706297
GLGA1_AGRTU I	6420138 2	CDK2_HUMAN Ful	6022 Aden	1GY3	1RZU	2UUE		Sc=6.33524, min distance = 2.004548
GLGA1_AGRTU I	6804 guar	NDK_PYRHO Ful	6022 Aden	2DYA	1RZU	2DXF	0.8	Sc=5.86312, min distance = 2.218371
GLGA1_AGRTU I	8977 1dar	O33839_THEMA I	6022 Aden	1XJK	1RZU	1XJE	0.8	Sc=5.95498, min distance = 1.963291
GLGA1_AGRTU I	8977 1dar	PARM_ECOLX Ful	6022 Aden	1MWM	1RZU	2ZGY	0.8	Sc=6.40901, min distance = 2.662376
GLGA1_AGRTU I	91532 AMF	BIOD_ECOLI Ful	6022 Aden	1DAD	1RZU	1DAG	0.99	Sc=5.96777, min distance = 2.729796
GLGA1_AGRTU I	91532 AMF	MTNK_BACSU Ful	6022 Aden	2OLC	1RZU	2PUL	0.99	Sc=6.0178, min distance = 1.7863809
GLGA_ECOLI Fu	124823 6g	PYGM_RABIT Ful	79025 alp	1H5U	2QZS	6GPB	0.78	Sc=6.07943, min distance = 2.236051
GLGA_ECOLI Fu	24864076	PYGM_RABIT Ful	79025 alp	1H5U	2QZS	2QRH		Sc=6.34941, min distance = 2.213599
GLGA_ECOLI Fu	36735 Gpp	PARM_ECOLX Ful	6022 Aden	1MWM	2QZS	2ZGZ	0.79	Sc=6.50408, min distance = 2.302282
GLGA_ECOLI Fu	4369136 C	CDK2_HUMAN Ful	6022 Aden	1GY3	2QZS	1DM2		Sc=6.27881, min distance = 2.221266
GLGA_ECOLI Fu	440317 AT	PURT_ECOLI Ful	6022 Aden	1KJQ	2QZS	1KJJ	0.99	Sc=6.48621, min distance = 2.430261
GLGA_ECOLI Fu	444564 AD	MYS2_DICDI Ful	6022 Aden	1VOM	2QZS	1W9I	0.91	Sc=6.43746, min distance = 2.266445
GLGA_ECOLI Fu	445825 CI	PYGM_RABIT Ful	79025 alp	1H5U	2QZS	1GG8		Sc=6.0057, min distance = 2.4321731
GLGA_ECOLI Fu	445916 be	XYLA_STRRU Ful	79025 alp	1XIF	2QZS	1GW9	0.93	Sc=5.637, min distance = 2.5232512
GLGA_ECOLI Fu	446090 1h	NDKC_DICDI Ful	6022 Aden	1KDN	2QZS	1HIY	0.97	Sc=6.44051, min distance = 2.064436
GLGA_ECOLI Fu	449515 CI	PYGM_RABIT Ful	79025 alp	1H5U	2QZS	4GPB	0.83	Sc=5.96952, min distance = 2.51406(
GLGA_ECOLI Fu	449554 CI	PYGM_RABIT Ful	79025 alp	1H5U	2QZS	5GPB		Sc=6.08378, min distance = 2.204664
GLGA_ECOLI Fu	5327148 C	IPKA_RABIT Ful	6022 Aden	1JBP	2QZS	2ERZ		Sc=6.22823, min distance = 2.335949
GLGA_ECOLI Fu	5957 Ader	AROK_MYCTU Ful	6022 Aden	2IYV	2QZS	2IYW	0.99	Sc=6.03226, min distance = 2.174406
GLGA_ECOLI Fu	5957 Ader	BIOD_ECOLI Ful	6022 Aden	1DAD	2QZS	1A82	0.99	Sc=6.03345, min distance = 2.221141
GLGA_ECOLI Fu	5957 Ader	HSP7F_YEAST Fu	6022 Aden	3C7N	2QZS	3D2F	0.99	Sc=6.48439, min distance = 1.882284
GLGA_ECOLI Fu	5957 Ader	KTHY_HUMAN Ful	6022 Aden	1NN3	2QZS	1E2Q	0.99	Sc=6.02623, min distance = 2.262372
GLGA_ECOLI Fu	5957 Ader	MUTS_ECOLI Ful	6022 Aden	1OH7	2QZS	1W7A	0.99	Sc=6.50958, min distance = 1.826775
GLGA_ECOLI Fu	6027 xylo	XYLA_STRRU Ful	79025 alp	1XIF	2QZS	3XIS	0.93	Sc=5.62032, min distance = 2.283158
GLGA_ECOLI Fu	6083 ader	NDK_PYRHO Ful	6022 Aden	2DYA	2QZS	2DXD	0.99	Sc=5.90214, min distance = 1.202417
GLGA_ECOLI Fu	6083 ader	PSPF_ECOLI Ful	6022 Aden	2C98	2QZS	2VII	0.99	Sc=6.36755, min distance = 2.104194
GLGA_ECOLI Fu	6083 ader	PURP_METJA Ful	6022 Aden	2R7N	2QZS	2R7M	0.99	Sc=6.3787, min distance = 2.4120534
GLGA_ECOLI Fu	6338561 C	MYS2_DICDI Ful	6022 Aden	1VOM	2QZS	1D0X		Sc=6.2608, min distance = 2.0471370
GLGA_ECOLI Fu	64960 Pol	XYLA_STRRU Ful	79025 alp	1XIF	2QZS	1XIE	0.89	Sc=5.78164, min distance = 2.498988
GLGA_ECOLI Fu	6804 guar	NDK_PYRHO Ful	6022 Aden	2DYA	2QZS	2DXF	0.8	Sc=6.41073, min distance = 2.128396
GLGA_ECOLI Fu	8977 1dar	PARM_ECOLX Ful	6022 Aden	1MWM	2QZS	2ZGY	0.8	Sc=6.46037, min distance = 2.331295

GLGA_ECOLI	Fı	91532 AMI	AROK_MYCTU Fu	6022 Aden	2IYV	2QZS	1ZYU		0.99	Sc=6.51596, min distance = 2.05756
GLGA_ECOLI	Fı	91532 AMI	BIOD_ECOLI Fu	6022 Aden	1DAD	2QZS	1DAG		0.99	Sc=6.51068, min distance = 2.06655
GLGA_ECOLI	Fı	91532 AMI	KIF1A_MOUSE Fı	6022 Aden	1I5S	2QZS	1I6I		0.99	Sc=6.07451, min distance = 2.05523
GLGA_ECOLI	Fı	91532 AMI	PURP_METJA Fu	6022 Aden	2R7N	2QZS	2R7K		0.99	Sc=6.52283, min distance = 2.46592
GLGA_ECOLI	Fı	95259 L-x	XYLA_STRRU Fu	79025 alp	1XIF	2QZS	1XIC			Sc=5.73431, min distance = 2.43330
GLGS_SOLTU	Fı	24832037	PGK1_HUMAN Fu	6022 Aden	2ZGV	1YP4	3C3C			Sc=5.62259, min distance = 2.48024
GLGS_SOLTU	Fı	36735 Gpp	PARM_ECOLX Fu	6022 Aden	1MWM	1YP4	2ZGZ		0.79	Sc=6.45369, min distance = 2.16918
GLGS_SOLTU	Fı	447955 1p	CDK2_HUMAN Fu	6022 Aden	1GY3	1YP4	1PXI			Sc=5.76154, min distance = 1.85806
GLGS_SOLTU	Fı	6031 Uric	O33839_THEMA I	6022 Aden	1XJK	1YP4	1XJG			Sc=5.67478, min distance = 2.12738
GLGS_SOLTU	Fı	8977 1dar	PARM_ECOLX Fu	6022 Aden	1MWM	1YP4	2ZGY		0.8	Sc=6.40094, min distance = 2.28744
GLGS_SOLTU	Fı	9817550 V	CDK2_HUMAN Fu	6022 Aden	1GY3	1YP4	3BHV			Sc=6.42521, min distance = 1.32156
GLKA_PYRFU	Fı	439692 D-	XYLA_STRRU Fu	79025 alp	1XIF	1UA4	4XIS			Sc=5.61389, min distance = 2.26711
GLKA_PYRFU	Fı	445825 CI	PYGM_RABIT Fu	79025 alp	1H5U	1UA4	1GG8			Sc=5.98916, min distance = 2.20735
GLKA_PYRFU	Fı	445916 be	XYLA_STRRU Fu	79025 alp	1XIF	1UA4	1GW9		0.93	Sc=5.61175, min distance = 2.60165
GLKA_PYRFU	Fı	449180 XI	XYLA_STRRU Fu	79025 alp	1XIF	1UA4	4XIS			Sc=5.72655, min distance = 2.11762
GLKA_PYRFU	Fı	449225 3-	XYLA_STROL Fu	79025 alp	1S5M	1UA4	1XYC			Sc=5.96277, min distance = 2.13403
GLKA_PYRFU	Fı	449343 2g	XYLA_STROL Fu	79025 alp	1S5M	1UA4	2GYI			Sc=5.72109, min distance = 2.16793
GLKA_PYRFU	Fı	5289590 I	XYLA_STRRU Fu	79025 alp	1XIF	1UA4	1XII			Sc=5.73431, min distance = 2.12551
GLKA_PYRFU	Fı	6027 xylo	Q9ZB17_9LACT I	79025 alp	1NSZ	1UA4	1MN0		0.93	Sc=5.61175, min distance = 2.22141
GLKA_PYRFU	Fı	6027 xylo	XYLA_STRRU Fu	79025 alp	1XIF	1UA4	3XIS		0.93	Sc=5.60036, min distance = 2.41974
GLKA_PYRFU	Fı	64960 Pol	XYLA_STRRU Fu	79025 alp	1XIF	1UA4	1XIE		0.89	Sc=5.73786, min distance = 2.41528
GLKA_PYRFU	Fı	7048614 Z	XYLA_STRRU Fu	79025 alp	1XIF	1UA4	1XID			Sc=5.85216, min distance = 2.68106
GLKA_PYRFU	Fı	95259 L-x	XYLA_STRRU Fu	79025 alp	1XIF	1UA4	1XIC			Sc=5.70837, min distance = 2.41398
GLKA_THELI	Fı	1540 1h1c	CDK2_HUMAN Fu	6022 Aden	1GY3	1GC5	1H1Q			Sc=6.26689, min distance = 1.39897
GLKA_THELI	Fı	16046126	CDK2_HUMAN Fu	6022 Aden	1GY3	1GC5	2DS1			Sc=6.32, min distance = 2.46743794
GLKA_THELI	Fı	23653515	CDK2_HUMAN Fu	6022 Aden	1GY3	1GC5	2R3F			Sc=6.00207, min distance = 2.04650
GLKA_THELI	Fı	23727981	CDK2_HUMAN Fu	6022 Aden	1GY3	1GC5	3BHT			Sc=6.03587, min distance = 2.68786
GLKA_THELI	Fı	23727982	CDK2_HUMAN Fu	6022 Aden	1GY3	1GC5	3BHU			Sc=6.08608, min distance = 2.84807
GLKA_THELI	Fı	24864080	CDK2_HUMAN Fu	6022 Aden	1GY3	1GC5	2VTR			Sc=5.78719, min distance = 2.20529
GLKA_THELI	Fı	4369136 C	CDK2_HUMAN Fu	6022 Aden	1GY3	1GC5	1DM2			Sc=5.9354, min distance = 3.070445
GLKA_THELI	Fı	444564 AI	BIOD_ECOLI Fu	6022 Aden	1DAD	1GC5	1BS1		0.91	Sc=5.65586, min distance = 2.31554
GLKA_THELI	Fı	446090 1h	NDKC_DICDI Fu	6022 Aden	1KDN	1GC5	1HIY		0.97	Sc=5.66722, min distance = 1.99182
GLKA_THELI	Fı	447766 1p	CDK2_HUMAN Fu	6022 Aden	1GY3	1GC5	1P2A			Sc=6.2168, min distance = 2.125461
GLKA_THELI	Fı	5287416 C	ABL1_HUMAN Fu	6022 Aden	2G2I	1GC5	2G1T		0.93	Sc=5.96233, min distance = 2.28082
GLKA_THELI	Fı	5327130 C	CDK2_HUMAN Fu	6022 Aden	1GY3	1GC5	2C68			Sc=6.16565, min distance = 2.02696
GLKA_THELI	Fı	5327131 T	CDK2_HUMAN Fu	6022 Aden	1GY3	1GC5	2C69			Sc=6.27352, min distance = 1.91718
GLKA_THELI	Fı	5327148 C	IPKA_RABIT Fu	6022 Aden	1JBP	1GC5	2ERZ			Sc=6.08504, min distance = 2.13685

GLKA_THELI F	5957 Aden	HSP7F_YEAST F	6022 Aden	3C7N	1GC5	3D2F	0.99	Sc=5.9245, min distance = 2.413113
GLKA_THELI F	5957 Aden	Q53W83_THET8	6022 Aden	1V1A	1GC5	1V1B	0.99	Sc=5.94909, min distance = 2.18715
GLKA_THELI F	6083 aden	PURP_METJA Fu	6022 Aden	2R7N	1GC5	2R7M	0.99	Sc=6.26137, min distance = 2.32827
GLKA_THELI F	6918710 5	CDK2_HUMAN Fu	6022 Aden	1GY3	1GC5	3EJ1		Sc=6.08925, min distance = 2.43261
GLKA_THELI F	8977 1dar	PARM_ECOLX Fu	6022 Aden	1MWM	1GC5	2ZGY	0.8	Sc=5.82935, min distance = 2.94358
GLKA_THELI F	91532 AMP	PURP_METJA Fu	6022 Aden	2R7N	1GC5	2R7K	0.99	Sc=5.94024, min distance = 2.17746
GLK_ECOLI Fu	125409 be	XYNA1_CLOSR F	64689 bet	1OD3	1SZ2	1UY4	0.93	Sc=5.60815, min distance = 1.56824
GLK_ECOLI Fu	24139 ace	LEG3_HUMAN Fu	64689 bet	2NMO	1SZ2	1KJL		Sc=6.08523, min distance = 2.45217
GLK_ECOLI Fu	444809 Th	GUN2_THEFU Fu	64689 bet	2BOF	1SZ2	2BOG	0.78	Sc=5.65103, min distance = 1.49201
GLK_ECOLI Fu	445564 4-	GUNG_CLOCE Fu	64689 bet	1GA2	1SZ2	1KFG	0.88	Sc=5.72073, min distance = 0.17282
GLK_ECOLI Fu	445999 CI	Q79G13_MYCTU	64689 bet	1UP0	1SZ2	1UOZ	0.77	Sc=5.73768, min distance = 0.51938
GLK_ECOLI Fu	447607 1c	Q79G13_MYCTU	64689 bet	1UP0	1SZ2	1UP2		Sc=5.84578, min distance = 0.61251
GLK_ECOLI Fu	6027 xylo	O93715_SULSO	64689 bet	2CDB	1SZ2	2CDC	0.93	Sc=5.65079, min distance = 2.33196
GLMS_ECOLI F	191445 1g	G6PI_RABIT Fu	152306 Sc	1XTB	2J6H	1G98		Sc=5.91025, min distance = 2.17534
GLMS_ECOLI F	24820117	GGT_ECOLI Ful	33032 L-g	2DG5	1XFF	2Z8J		Sc=5.84986, min distance = 1.70471
GLMS_ECOLI F	40539 Qui	GRIA2_RAT Ful	33032 L-g	2GFE	1XFF	1P1O		Sc=5.91638, min distance = 1.95055
GLMS_ECOLI F	440053 Wi	GRIA2_RAT Ful	33032 L-g	2GFE	1XFF	1MQJ		Sc=6.07191, min distance = 1.89646
GLMS_ECOLI F	440641 1m	G6PI_RABIT Fu	152306 Sc	1XTB	2J6H	1HOX	0.84	Sc=5.92093, min distance = 2.23327
GLMS_ECOLI F	446761 CI	G6PI_RABIT Fu	152306 Sc	1XTB	2J6H	1KOJ		Sc=5.97273, min distance = 2.10718
GLMS_ECOLI F	447083 ac	GRIA2_RAT Ful	33032 L-g	2GFE	1XFF	1M5E		Sc=6.1609, min distance = 1.803495
GLMS_ECOLI F	447195 1m	GRIA2_RAT Ful	33032 L-g	2GFE	1XFF	1MQD		Sc=5.90964, min distance = 2.25552
GLMS_ECOLI F	448823 CI	G6PI_MOUSE Fu	152306 Sc	2CXQ	2J6H	1U0F		Sc=5.91908, min distance = 2.37661
GLMS_ECOLI F	69507 Neu	NAGB_ECOLI Fu	446113 AI	1HOR	1MOS	1FQO		Sc=6.03104, min distance = 2.19888
GLMS_ECOLI F	91493 1pg	G6PI_RABIT Fu	152306 Sc	1XTB	2J6H	1DQR		Sc=6.0795, min distance = 2.116077
GLMU_ECOLI F	6031 Urid	EXTL2_MOUSE F	445675 UD	1ON6	1HV9	1ON8	0.82	Sc=5.84985, min distance = 2.41895
GLMU_ECOLI F	6031 Urid	MGAT1_RABIT F	445675 UD	1FOA	1HV9	2AM5	0.82	Sc=5.85994, min distance = 2.05629
GLMU_HAEIN F	6804 guar	PYRR_BACCL Fu	6030 Urid	2IGB	2V0J	1XZ8		Sc=6.2629, min distance = 1.933332
GLMU_STRPN F	6031 Urid	EXTL2_MOUSE F	445675 UD	1ON6	1HM9	1ON8	0.82	Sc=5.84898, min distance = 2.47117
GLMU_STRPN F	6914576 C	MGAT1_RABIT F	445675 UD	1FOA	1HM9	2APC	0.92	Sc=6.67317, min distance = 1.97404
GLNA1_MYCTU	444564 AD	BIOD_ECOLI Fu	6022 Aden	1DAD	2BVC	1BS1	0.91	Sc=6.45105, min distance = 1.86149
GLNA1_MYCTU	447048 2b	Y1521_ARCFU F	6022 Aden	2BFR	2BVC	2BFQ	0.95	Sc=5.89483, min distance = 2.06248
GLNA1_MYCTU	447916 ac	RIO1_ARCFU Fu	6022 Aden	1ZTH	2BVC	1ZTF	0.95	Sc=6.2993, min distance = 1.989807
GLNA1_MYCTU	447955 1p	CDK2_HUMAN Fu	6022 Aden	1GY3	2BVC	1PXI		Sc=5.7969, min distance = 2.108320
GLNA1_MYCTU	5327148 C	IPKA_RABIT Fu	6022 Aden	1JBP	2BVC	2ERZ		Sc=6.25749, min distance = 2.01638
GLNA1_MYCTU	5957 Aden	PURL_THEMA Fu	6022 Aden	2HRU	2BVC	2HS0	0.99	Sc=6.19015, min distance = 2.22265
GLNA1_MYCTU	5957 Aden	Y059_METJA Fu	6022 Aden	2J9D	2BVC	2J9C	0.99	Sc=6.50339, min distance = 1.88439
GLNA1_MYCTU	60961 ade	IPKA_RABIT Fu	6022 Aden	1JBP	2BVC	1FMO	0.95	Sc=6.31434, min distance = 2.18020

GLNA3_MAIZE I	24832037	PGK1_HUMAN Fu	6022 Aden	2ZGV	2D3A	3C3C		Sc=5.86647, min distance = 2.29494(
GLNA3_MAIZE I	444503 FU	NDKC_DICDI Fu	6022 Aden	1KDN	2D3A	1B99		Sc=6.2465, min distance = 2.196318(
GLNA3_MAIZE I	447955 1p	CDK2_HUMAN Fu	6022 Aden	1GY3	2D3A	1PXI		Sc=5.74284, min distance = 1.730540
GLNA3_MAIZE I	5957 Aden	PUR7_METJA Fu	6022 Aden	2YZL	2D3A	2Z02	0.99	Sc=6.02808, min distance = 1.56415
GLNA3_MAIZE I	6031 Uric	DCK_HUMAN Ful	6022 Aden	1P5Z	2D3A	2ZIA		Sc=5.86647, min distance = 2.23445
GLNA3_MAIZE I	72194 2-C	ENPL_CANFA Fu	6022 Aden	1TC6	2D3A	1QYE	0.89	Sc=6.21257, min distance = 2.04794
GLNA3_MAIZE I	91532 AMI	MTNK_BACSU Fu	6022 Aden	2OLC	2D3A	2PUL	0.99	Sc=6.51325, min distance = 1.92842
GLNA_HUMAN Fu	13711 dec	DCK_HUMAN Ful	6022 Aden	1P5Z	2OJW	2NO1		Sc=5.70088, min distance = 2.08327
GLNA_HUMAN Fu	164628 de	PMS2_HUMAN Fu	6022 Aden	1EA6	2OJW	1H7U		Sc=6.38219, min distance = 1.45004
GLNA_HUMAN Fu	24832037	PGK1_HUMAN Fu	6022 Aden	2ZGV	2OJW	3C3C		Sc=6.30251, min distance = 2.39757
GLNA_HUMAN Fu	36735 Gpp	PARM_ECOLX Fu	6022 Aden	1MWM	2OJW	2ZGZ	0.79	Sc=6.49069, min distance = 1.81971(
GLNA_HUMAN Fu	444564 AD	BIOD_ECOLI Fu	6022 Aden	1DAD	2OJW	1BS1	0.91	Sc=6.39101, min distance = 2.15824
GLNA_HUMAN Fu	445940 O6	CDK2_HUMAN Fu	6022 Aden	1GY3	2OJW	1GZ8		Sc=6.1875, min distance = 2.017452
GLNA_HUMAN Fu	4565 1h1r	CDK2_HUMAN Fu	6022 Aden	1GY3	2OJW	1H1R		Sc=6.10256, min distance = 0.87280
GLNA_HUMAN Fu	5957 Aden	BIOD_ECOLI Fu	6022 Aden	1DAD	2OJW	1A82	0.99	Sc=6.43911, min distance = 2.08449
GLNA_HUMAN Fu	5957 Aden	MUTS_ECOLI Fu	6022 Aden	1OH7	2OJW	1W7A	0.99	Sc=6.46904, min distance = 1.97915
GLNA_HUMAN Fu	5957 Aden	RK_BOVIN Full	6022 Aden	3C4Z	2OJW	3C4W	0.99	Sc=6.41296, min distance = 1.88818
GLNA_HUMAN Fu	60750 gem	DCK_HUMAN Ful	6022 Aden	1P5Z	2OJW	2NO0		Sc=5.75079, min distance = 2.18368(
GLNA_HUMAN Fu	60961 ade	IPKA_RABIT Fu	6022 Aden	1JBP	2OJW	1FMO	0.95	Sc=6.22266, min distance = 2.58325
GLNA_HUMAN Fu	91532 AMI	BIOD_ECOLI Fu	6022 Aden	1DAD	2OJW	1DAG	0.99	Sc=6.47856, min distance = 2.35944
GLNA_HUMAN Fu	91532 AMI	PURP_METJA Fu	6022 Aden	2R7N	2OJW	2R7K	0.99	Sc=6.04427, min distance = 1.99931(
GLNA_SALTY Fu	24752834	SYH_ECOLI Ful	6083 aden	1HTT	1LGR	2EL9		Sc=6.5729, min distance = 2.084275
GLNA_SALTY Fu	6131 cyti	RNAS1_BOVIN Fu	6022 Aden	1O0H	1F52	1RNN		Sc=5.6608, min distance = 2.343962(
GLNA_SALTY Fu	65533 cor	O57693_THETE I	6083 aden	1UXU	1LGR	1UXT		Sc=5.88632, min distance = 2.31081
GLNA_SALTY Fu	656969 1y	PDE4B_HUMAN Fu	6083 aden	1ROR	1LGR	1Y2J		Sc=6.18811, min distance = 2.12262
GLNA_SALTY Fu	84265 1ax	GALK2_HUMAN Fu	6022 Aden	2A2C	1F52	2A2D		Sc=6.08392, min distance = 2.16707
GLNA_SALTY Fu	8582 Inos	PYGM_RABIT Fu	6083 aden	8GPB	1LGR	2QN7	0.79	Sc=5.87191, min distance = 2.13768
GLNB_AQUAE Fu	3547 Fasu	KAPCA_BOVIN Fu	5957 Aden	1Q24	2EG2	1Q8W		Sc=5.66414, min distance = 2.18424
GLNB_AQUAE Fu	444852 CI	ATPA1_BOVIN Fu	5957 Aden	2V7Q	2EG2	1COW	0.91	Sc=5.9334, min distance = 1.972160
GLNB_AQUAE Fu	447916 ac	RIO1_ARCFU Fu	5957 Aden	1ZP9	2EG2	1ZTF	0.94	Sc=5.93333, min distance = 2.89441
GLNB_AQUAE Fu	449240 1y	KAPCA_BOVIN Fu	5957 Aden	1Q24	2EG2	1YDR		Sc=5.67534, min distance = 2.01432
GLNB_AQUAE Fu	6022 Aden	CLCN5_HUMAN Fu	5957 Aden	2J9L	2EG2	2JA3	0.99	Sc=6.30809, min distance = 2.16795
GLNB_AQUAE Fu	6022 Aden	Y059_METJA Fu	5957 Aden	2J9C	2EG2	2J9D	0.99	Sc=5.85048, min distance = 2.66839
GLNB_AQUAE Fu	6083 aden	PURP_PYRFU Fu	5957 Aden	2R86	2EG2	2R85	0.98	Sc=6.06938, min distance = 2.10786
GLNK_ECOLI Fu	11957393	FAK1_HUMAN Fu	6022 Aden	1MP8	2NS1	2ETM		Sc=5.96616, min distance = 2.29987
GLNK_ECOLI Fu	3547 Fasu	KAPCA_BOVIN Fu	5957 Aden	1Q24	2GNK	1Q8W		Sc=5.60147, min distance = 3.07703
GLNK_ECOLI Fu	444564 AD	BIOD_ECOLI Fu	6022 Aden	1DAD	2NS1	1BS1	0.91	Sc=6.28224, min distance = 2.06081

GLNK_ECOLI Fu	446090 1h	NDKC_DICDI Ful	6022 Aden	1KDN	2NS1	1HIY	0.97	Sc=6.33043, min distance = 1.664150
GLNK_ECOLI Fu	447916 ad	RIO1_ARCFU Ful	5957 Aden	1ZP9	2GNK	1ZTF	0.94	Sc=5.86074, min distance = 3.110444
GLNK_ECOLI Fu	448505 AD	NDKC_DICDI Ful	6022 Aden	1KDN	2NS1	1S5Z	0.93	Sc=6.33167, min distance = 2.302584
GLNK_ECOLI Fu	6083 ader	AAKG1_RAT Ful	5957 Aden	2V92	2GNK	2V8Q	0.98	Sc=6.05855, min distance = 2.034219
GLNK_ECOLI Fu	60961 ade	SYQ_ECOLI Ful	5957 Aden	1GTR	2GNK	1O0B	0.94	Sc=5.91746, min distance = 2.929229
GLNK_ECOLI Fu	6852187 2	O57883_PYRHO F	5957 Aden	2DTO	2GNK	2DTH		Sc=5.76892, min distance = 2.186962
GLNK_ECOLI Fu	6852207 M	KAPCA_BOVIN Fu	5957 Aden	1Q24	2GNK	2GNI		Sc=5.61943, min distance = 2.826504
GLNK_ECOLI Fu	91532 AMP	F263_HUMAN Ful	6022 Aden	2AXN	2NS1	2DWP	0.99	Sc=6.38767, min distance = 2.039148
GLOX_BACSU Fu	16740985	FRDA_SHEFR Ful	444188 CI	1M64	1RYI	1E39	0.93	Sc=6.43959, min distance = 2.075777
GLOX_BACSU Fu	444502 CI	GSHR_HUMAN Ful	444188 CI	3DK9	1RYI	1BWC	0.98	Sc=6.43876, min distance = 2.014299
GLOX_BACSU Fu	445794 AD	PHHY_PSEFL Ful	444188 CI	1PBE	1RYI	2PHH		Sc=6.64522, min distance = 1.988084
GLOX_BACSU Fu	446013 1,	O28603_ARCFU F	444188 CI	1JNR	1RYI	1JNZ	0.94	Sc=6.4433, min distance = 2.3950183
GLOX_BACSU Fu	446013 1,	O28604_ARCFU F	444188 CI	1JNR	1RYI	1JNZ	0.94	Sc=6.44369, min distance = 2.421787
GLOX_BACSU Fu	449465 CI	GSHR_HUMAN Ful	444188 CI	3DK9	1RYI	3GRT	0.98	Sc=6.44213, min distance = 2.159206
GLOX_BACSU Fu	6420174 C	O28604_ARCFU F	444188 CI	1JNR	1RYI	2FJB	0.92	Sc=6.4522, min distance = 1.9222510
GLPD_ECOLI Fu	444502 CI	TYTR_TRYCR Ful	11987634	1AOG	2R4E	1GXF	0.95	Sc=6.88754, min distance = 1.975112
GLPD_ECOLI Fu	446013 1,	O28603_ARCFU F	444188 CI	1JNR	2QCU	1JNZ	0.94	Sc=6.88783, min distance = 2.029479
GLPD_ECOLI Fu	446013 1,	O28604_ARCFU F	444188 CI	1JNR	2QCU	1JNZ	0.94	Sc=6.88754, min distance = 1.918234
GLPD_ECOLI Fu	449465 CI	GSHR_HUMAN Ful	444188 CI	3DK9	2QCU	3GRT	0.98	Sc=6.88378, min distance = 2.189663
GLPG_ECOLI Fu	11987742	LHA4_RHOAC Ful	444279 1s	2FKW	2IRV	1NKZ		Sc=6.39797, min distance = 0.706745
GLPK_ECOLI Fu	33113 gam	KIF1A_MOUSE Fu	91532 AMP	1I6I	1GLL	1VFV	0.99	Sc=5.96326, min distance = 2.155198
GLPK_ECOLI Fu	33113 gam	PURT_ECOLI Ful	91532 AMP	1KJI	1GLL	1EYZ	0.99	Sc=6.42507, min distance = 1.996792
GLPK_ECOLI Fu	36735 Gpp	PARM_ECOLX Ful	6022 Aden	1MWM	1GLB	2ZGZ	0.79	Sc=6.47425, min distance = 2.156003
GLPK_ECOLI Fu	440317 AT	PKNB_MYCTU Ful	91532 AMP	1O6Y	1GLL	1MRU	0.99	Sc=5.8193, min distance = 2.1898924
GLPK_ECOLI Fu	444564 AD	BIOD_ECOLI Ful	6022 Aden	1DAD	1GLB	1BS1	0.91	Sc=5.9406, min distance = 2.1010314
GLPK_ECOLI Fu	444564 AD	BIOD_ECOLI Ful	91532 AMP	1DAG	1GLL	1BS1	0.91	Sc=6.35803, min distance = 2.297735
GLPK_ECOLI Fu	445940 O6	CDK2_HUMAN Ful	6022 Aden	1GY3	1GLB	1GZ8		Sc=6.20895, min distance = 1.794230
GLPK_ECOLI Fu	445966 O6	CDK2_HUMAN Ful	6022 Aden	1GY3	1GLB	1H0V		Sc=6.25346, min distance = 1.998563
GLPK_ECOLI Fu	446381 1u	PDXJ_ECOLI Ful	439162 gl	1IXO	1GLC	1IXN		Sc=5.70848, min distance = 2.208631
GLPK_ECOLI Fu	5327148 C	IPKA_RABIT Ful	6022 Aden	1JBP	1GLB	2ERZ		Sc=6.16445, min distance = 2.555243
GLPK_ECOLI Fu	5957 Ader	ACT1_DROME Ful	6022 Aden	2HF3	1GLB	2HF4	0.99	Sc=6.46604, min distance = 2.191608
GLPK_ECOLI Fu	5957 Ader	BIOD_ECOLI Ful	91532 AMP	1DAG	1GLL	1A82	0.99	Sc=6.41348, min distance = 2.247515
GLPK_ECOLI Fu	5957 Ader	HSP7F_YEAST Fu	6022 Aden	3C7N	1GLB	3D2F	0.99	Sc=6.4602, min distance = 2.0210544
GLPK_ECOLI Fu	5957 Ader	MUTS_ECOLI Ful	6022 Aden	1OH7	1GLB	1W7A	0.99	Sc=6.45826, min distance = 2.328595
GLPK_ECOLI Fu	5957 Ader	NIFH1_AZOVI Fu	91532 AMP	2AFK	1GLL	2C8V	0.99	Sc=5.98318, min distance = 2.631855
GLPK_ECOLI Fu	5957 Ader	PURK_ECOLI Ful	6022 Aden	3ETJ	1GLB	3ETH	0.99	Sc=6.48195, min distance = 2.049824
GLPK_ECOLI Fu	5957 Ader	PURP_METJA Ful	91532 AMP	2R7K	1GLL	2R7L	0.99	Sc=6.44119, min distance = 2.014569

GLPK_ECOLI F	5962 L-ly	RBCMT_PEA Ful	23831 HEP	1MLV	1BOT	2H2E			Sc=5.78164, min distance = 0.99445
GLPK_ECOLI F	6031 Uric	DCK_HUMAN Ful	6022 Aden	1P5Z	1GLB	2ZIA			Sc=6.27397, min distance = 2.26900
GLPK_ECOLI F	6083 aden	PURP_METJA Fu	91532 AMP	2R7K	1GLL	2R7M		0.97	Sc=5.87191, min distance = 2.62650
GLPK_ECOLI F	6102784 A	PSPF_ECOLI Fu	6022 Aden	2C98	1GLB	2C99		0.99	Sc=6.45826, min distance = 2.00043
GLPK_ECOLI F	668 Glyce	CGGR_BACSU Fu	439162 gl	2OKG	1GLC	3BXF		0.76	Sc=5.69546, min distance = 2.29197
GLPK_ECOLI F	6852187 2	O57883_PYRHO	6022 Aden	1WNL	1GLB	2DTH		0.89	Sc=6.1144, min distance = 1.256132
GLPX_ECOLI F	107737 ir	SUHB_ARCFU Fu	10267 Har	1LBZ	2R8T	1LBX		0.75	Sc=6.05176, min distance = 2.03194
GLRX2_HUMAN	115260 S-	GSTM2_HUMAN F	124886 gl	1XW5	2FLS	2AB6		0.94	Sc=5.86599, min distance = 2.69943
GLRX2_HUMAN	97536 Hex	GSTP_ONCVO Fu	124886 gl	1TU7	2FLS	1TU8		0.9	Sc=6.44208, min distance = 2.29201
GLT10_HUMAN	185698 al	MBL1_RAT Full	440552 1b	1AFB	2D7I	2MSB			Sc=5.63826, min distance = 2.77867
GLT10_HUMAN	444863 is	MBL1_RAT Full	440552 1b	1AFB	2D7I	1KWX			Sc=5.673, min distance = 2.4708213
GLT10_HUMAN	446578 1r	MBL1_RAT Full	440552 1b	1AFB	2D7I	1KWW			Sc=5.68985, min distance = 2.25230
GLT10_HUMAN	94214 Met	MBL1_RAT Full	440552 1b	1AFB	2D7I	1AFA			Sc=5.85925, min distance = 1.27053
GLTP_HUMAN F	7048614 Z	XYLA_STRRU Fu	79025 alp	1XIF	2EVS	1XID			Sc=5.78241, min distance = 1.65025
GLTS_SYNY3 F	16741044	PYRDA_LACLC F	444243 FA	1JUB	1OFD	1JRB			Sc=6.64856, min distance = 2.18175
GLTS_SYNY3 F	446995 FM	CYB2_YEAST Fu	444243 FA	1KBI	1OFD	1LTD		0.77	Sc=6.65136, min distance = 2.25961
GLTS_SYNY3 F	51 2-Oxop	CEFE_STRCL Fu	43 2-hydr	1UOB	1OFD	1RXG		0.78	Sc=5.74847, min distance = 2.40335
GLVA_BACSU F	10267 Har	CGGR_BACSU Fu	439427 Rc	3BXG	1U8X	3BXF		0.87	Sc=6.02532, min distance = 2.26644
GLVA_BACSU F	440641 1m	CGGR_BACSU Fu	439427 Rc	3BXG	1U8X	3BXH		0.89	Sc=5.8705, min distance = 2.512425
GLVA_BACSU F	445794 AD	ADHX_HUMAN Fu	5893 nadi	2FZW	1U8X	2FZE			Sc=6.67517, min distance = 1.81546
GLVA_BACSU F	446288 NA	G3P_PALVE Ful	5893 nadi	1DSS	1U8X	1IHX			Sc=6.79688, min distance = 2.05078
GLYA_ECOLI F	1051 Code	HEM1_RHOCA Fu	445062 CI	2BWP	1DFO	2BWO		0.86	Sc=6.11946, min distance = 2.40393
GLYA_ECOLI F	447013 CI	Q9X266_THEMA	445062 CI	1LW5	1DFO	1LW4		0.94	Sc=5.95977, min distance = 2.55816
GLYG_RABIT F	6132 Cyti	ECX1_PYRAB Fu	6031 Urid	2PNZ	1ZCT	2PO2		0.83	Sc=5.72083, min distance = 1.83965
GM4D_PSEAE F	24978488	TBB2B_BOVIN F	8977 1dar	1Z2B	1RPN	3DU7			Sc=6.46563, min distance = 2.02648
GM4D_PSEAE F	6022 Aden	NDK_PYRHO Ful	8977 1dar	2DXE	1RPN	2DYA		0.8	Sc=6.44771, min distance = 2.05249
GM4D_PSEAE F	6804 guar	DPOL_BPR69 Fu	8977 1dar	1CLQ	1RPN	1WAJ		0.99	Sc=6.42318, min distance = 1.93099
GMD2_ARATH F	165230 1a	G6PD_LEUME Fu	5886 NADF	1H9A	1N7H	2DPG			Sc=5.99503, min distance = 2.30826
GMD2_ARATH F	6022 Aden	NDK_PYRHO Ful	8977 1dar	2DXE	1N7H	2DYA		0.8	Sc=6.42092, min distance = 2.23229
GMD2_ARATH F	6804 guar	DPOL_BPR69 Fu	8977 1dar	1CLQ	1N7H	1WAJ		0.99	Sc=6.39408, min distance = 2.20988
GMD2_ARATH F	6804 guar	GBP1_HUMAN Fu	8977 1dar	2B92	1N7H	2B8W		0.99	Sc=6.38219, min distance = 1.68285
GMD2_ARATH F	8582 Inos	PURA1_MOUSE F	8977 1dar	1LON	1N7H	1IWE		0.99	Sc=6.36522, min distance = 2.38267
GMD2_ARATH F	93082 Gab	RAC3_HUMAN Fu	8977 1dar	2C2H	1N7H	2QME		0.99	Sc=6.52732, min distance = 2.11199
GMDS_HUMAN F	6022 Aden	NDK_PYRHO Ful	8977 1dar	2DXE	1T2A	2DYA		0.8	Sc=6.429, min distance = 2.2256044
GMDS_HUMAN F	6031 Uric	O33839_THEMA	8977 1dar	1XJE	1T2A	1XJG			Sc=6.33191, min distance = 1.72431
GMDS_HUMAN F	6132 Cyti	RIR1_YEAST Fu	8977 1dar	2CVW	1T2A	2CVU			Sc=6.32807, min distance = 2.04654
GMDS_HUMAN F	6804 guar	DPOL_BPR69 Fu	8977 1dar	1CLQ	1T2A	1WAJ		0.99	Sc=6.40359, min distance = 2.24327

GMDS_HUMAN Fu	6804 guar	TGM3_HUMAN Fu	8977 1dar	1VJJ	1T2A	1SGX		0.99	Sc=6.39587, min distance = 1.49216
GMDS_HUMAN Fu	93082 Gab	RAC3_HUMAN Fu	8977 1dar	2C2H	1T2A	2QME		0.99	Sc=6.53236, min distance = 2.17003
GME_ARATH Fu	439153 Di	GALE_ECOLI Fu	5893 nadi	1UDC	2C5A	1UDB	22.55	0.79	Sc=6.80132, min distance = 2.37812
GME_ARATH Fu	439153 Di	Q9BJJ9_PLAFA	5893 nadi	1UH5	2C5A	1V35		0.79	Sc=6.79947, min distance = 2.02301
GMPR1_HUMAN	6030 Uric	PYRR_BACCL Fu	6804 guan	1XZ8	2BLE	2IGB			Sc=6.26874, min distance = 2.04606
GMPR1_HUMAN	6076 Cycl	PDE10_HUMAN Fu	6804 guan	2OUQ	2BLE	2OUR		0.79	Sc=6.32013, min distance = 0
GMPR2_HUMAN	101609 Ur	RNMC_MOMCH Fu	6804 guan	1J1F	2A7R	1UCA			Sc=6.28588, min distance = 2.17820
GMPR2_HUMAN	445708 AM	PURA_ECOLI Fu	8582 Inos	1CIB	2C6Q	1SON		0.81	Sc=6.3315, min distance = 2.013260
GMPR2_HUMAN	6030 Uric	PYRR_BACCL Fu	6804 guan	1XZ8	2A7R	2IGB			Sc=6.27776, min distance = 1.74620
GMPR2_HUMAN	6076 Cycl	PDE10_HUMAN Fu	6804 guan	2OUQ	2A7R	2OUR		0.79	Sc=6.3343, min distance = 0
GMPR2_HUMAN	6083 ader	PYGM_RABIT Fu	8582 Inos	2QN7	2C6Q	8GPB		0.79	Sc=6.33814, min distance = 2.08841
GNA12_MOUSE	37792 gam	GNA13_MOUSE Fu	8977 1dar	1ZCB	1ZCA	3CX8		0.99	Sc=6.54643, min distance = 2.08713
GNA12_MOUSE	37792 gam	GNAI1_RAT Ful	8977 1dar	1SVK	1ZCA	1AS0		0.99	Sc=6.53322, min distance = 2.25079
GNA12_MOUSE	37792 gam	RB11A_HUMAN Fu	8977 1dar	1OIX	1ZCA	1OIW		0.99	Sc=6.53966, min distance = 2.47572
GNA12_MOUSE	37792 gam	RHOA_HUMAN Fu	8977 1dar	1TX4	1ZCA	1CXZ		0.99	Sc=6.54356, min distance = 2.04826
GNA12_MOUSE	444148 1a	RHA1_ARATH Fu	8977 1dar	2EFC	1ZCA	2EFE		0.99	Sc=6.48488, min distance = 2.58892
GNA12_MOUSE	445463 B-	TGM3_HUMAN Fu	8977 1dar	1VJJ	1ZCA	1L9N			Sc=6.22749, min distance = 2.07373
GNA12_MOUSE	446248 CI	RASH_HUMAN Fu	8977 1dar	2CE2	1ZCA	1IAQ			Sc=6.55143, min distance = 2.30302
GNA12_MOUSE	6804 guar	TGM3_HUMAN Fu	8977 1dar	1VJJ	1ZCA	1SGX		0.99	Sc=6.43933, min distance = 1.90612
GNA12_MOUSE	8582 Inos	PURA1_MOUSE Fu	8977 1dar	1LON	1ZCA	1IWE		0.99	Sc=6.41036, min distance = 2.11750
GNA12_MOUSE	93082 Gab	CDC42_HUMAN Fu	8977 1dar	2NGR	1ZCA	2ODB		0.99	Sc=6.58841, min distance = 2.20695
GNA12_MOUSE	93082 Gab	RAC3_HUMAN Fu	8977 1dar	2C2H	1ZCA	2QME		0.99	Sc=6.57759, min distance = 2.57026
GNA12_MOUSE	93082 Gab	RASH_HUMAN Fu	8977 1dar	2CE2	1ZCA	121P		0.99	Sc=6.57575, min distance = 2.06241
GNA13_MOUSE	445463 B-	TGM3_HUMAN Fu	8977 1dar	1VJJ	1ZCB	1L9N			Sc=6.24734, min distance = 1.59832
GNA13_MOUSE	446248 CI	RAN_HUMAN Ful	8977 1dar	3CH5	1ZCB	1IBR			Sc=6.5713, min distance = 2.265306
GNA13_MOUSE	446248 CI	RASH_HUMAN Fu	8977 1dar	2CE2	1ZCB	1IAQ			Sc=6.55735, min distance = 2.20637
GNA13_MOUSE	6804 guar	EF1A_YEAST Fu	8977 1dar	2B7B	1ZCB	1G7C		0.99	Sc=6.44118, min distance = 2.25540
GNA13_MOUSE	93082 Gab	CDC42_HUMAN Fu	8977 1dar	2NGR	1ZCB	2ODB		0.99	Sc=6.59087, min distance = 2.42227
GNA13_MOUSE	93082 Gab	RAC3_HUMAN Fu	8977 1dar	2C2H	1ZCB	2QME		0.99	Sc=6.58488, min distance = 2.19355
GNA1_YEAST Fu	439217 1m	GNA1_HUMAN Fu	439219 NA	2O28	1I1D	3CXQ		0.82	Sc=5.95532, min distance = 0.86981
GNAI1_HUMAN	24978488	TBB2B_BOVIN Fu	8977 1dar	1Z2B	2HLB	3DU7			Sc=6.5181, min distance = 2.011790
GNAI1_HUMAN	37792 gam	RAB6B_HUMAN Fu	8977 1dar	2E9S	2HLB	2FFQ		0.99	Sc=6.54409, min distance = 1.96723
GNAI1_HUMAN	37792 gam	RB11A_HUMAN Fu	8977 1dar	1OIX	2HLB	1OIW		0.99	Sc=6.5367, min distance = 2.109630
GNAI1_HUMAN	445463 B-	TGM3_HUMAN Fu	8977 1dar	1VJJ	2HLB	1L9N			Sc=6.24576, min distance = 1.57823
GNAI1_HUMAN	446248 CI	RAN_HUMAN Ful	8977 1dar	3CH5	2HLB	1IBR			Sc=6.55843, min distance = 2.15330
GNAI1_HUMAN	6804 guar	DPOL_BPR69 Fu	8977 1dar	1CLQ	2HLB	1WAJ		0.99	Sc=6.4487, min distance = 2.216344
GNAI1_HUMAN	6804 guar	EF1A_YEAST Fu	8977 1dar	2B7B	2HLB	1G7C		0.99	Sc=6.44589, min distance = 2.22998

GNAI1_HUMAN I	8582 Inos	PURA1_MOUSE Fu	8977 1dar	1LON	2HLB	1IWE	0.99	Sc=6.41811, min distance = 2.30890
GNAI1_HUMAN I	93082 Gab	RAC3_HUMAN Fu	8977 1dar	2C2H	2HLB	2QME	0.99	Sc=6.5759, min distance = 2.296609
GNAI1_RAT Fu	444845 1d	RASH_HUMAN Fu	36735 Gpp	1CTQ	1SVS	1CLU	0.99	Sc=6.58347, min distance = 1.98248
GNAI1_RAT Fu	445463 B-	TGM3_HUMAN Fu	8977 1dar	1VJJ	1SVK	1L9N		Sc=6.23052, min distance = 2.21315
GNAI1_RAT Fu	446248 CI	RAN_HUMAN Ful	36735 Gpp	1K5D	1SVS	1IBR		Sc=6.55814, min distance = 2.33848
GNAI1_RAT Fu	446248 CI	RAN_HUMAN Ful	8977 1dar	3CH5	1SVK	1IBR		Sc=6.57691, min distance = 2.04312
GNAI1_RAT Fu	446248 CI	RASH_HUMAN Fu	36735 Gpp	1CTQ	1SVS	1IAQ		Sc=6.54289, min distance = 2.49911
GNAI1_RAT Fu	446248 CI	RASH_HUMAN Fu	8977 1dar	2CE2	1SVK	1IAQ		Sc=6.56068, min distance = 2.13662
GNAI1_RAT Fu	51 2-Oxop	SERA_ECOLI Fu	311 citri	2P9E	1AGR	1YBA	0.76	Sc=5.61763, min distance = 2.10801
GNAI1_RAT Fu	6022 Aden	PARM_ECOLX Fu	8977 1dar	2ZGY	1SVK	1MWM	0.8	Sc=6.49399, min distance = 2.00478
GNAI1_RAT Fu	6804 guar	EF1A_YEAST Fu	8977 1dar	2B7B	1SVK	1G7C	0.99	Sc=6.44928, min distance = 2.27556
GNAI1_RAT Fu	6804 guar	GBP1_HUMAN Fu	36735 Gpp	1F5N	1SVS	2B8W	0.98	Sc=6.43401, min distance = 1.92261
GNAI1_RAT Fu	93082 Gab	CDC42_HUMAN Fu	8977 1dar	2NGR	1SVK	2ODB	0.99	Sc=6.59451, min distance = 2.31561
GNAI1_RAT Fu	93082 Gab	RAC3_HUMAN Fu	36735 Gpp	2IC5	1SVS	2QME	0.99	Sc=6.58108, min distance = 2.45168
GNAI1_RAT Fu	93082 Gab	RAC3_HUMAN Fu	8977 1dar	2C2H	1SVK	2QME	0.99	Sc=6.59332, min distance = 2.38555
GNAI1_RAT Fu	93082 Gab	RASH_HUMAN Fu	36735 Gpp	1CTQ	1SVS	121P	0.99	Sc=6.57312, min distance = 2.50486
GNAI1_RAT Fu	93082 Gab	SRP54_THEAQ Fu	36735 Gpp	1JPN	1SVS	1RJ9	0.99	Sc=6.58553, min distance = 2.07908
GNAI3_HUMAN I	24978488	TBB2B_BOVIN Fu	8977 1dar	1Z2B	2ODE	3DU7		Sc=6.5181, min distance = 1.483423
GNAI3_HUMAN I	37792 gam	GNAI1_RAT Ful	8977 1dar	1SVK	2ODE	1AS0	0.99	Sc=6.54914, min distance = 2.53111
GNAI3_HUMAN I	37792 gam	GNAT1_BOVIN Fu	8977 1dar	1TAD	2ODE	1TND	0.99	Sc=6.56061, min distance = 2.27754
GNAI3_HUMAN I	37792 gam	RAB6B_HUMAN Fu	8977 1dar	2E9S	2ODE	2FFQ	0.99	Sc=6.55567, min distance = 2.24414
GNAI3_HUMAN I	37792 gam	RAC1_HUMAN Fu	8977 1dar	1RYF	2ODE	2FJU	0.99	Sc=6.56145, min distance = 2.30894
GNAI3_HUMAN I	37792 gam	RB11A_HUMAN Fu	8977 1dar	1OIX	2ODE	1OIW	0.99	Sc=6.55507, min distance = 2.63969
GNAI3_HUMAN I	37792 gam	RHOA_HUMAN Fu	8977 1dar	1TX4	2ODE	1CXZ	0.99	Sc=6.55881, min distance = 2.14310
GNAI3_HUMAN I	445463 B-	TGM3_HUMAN Fu	8977 1dar	1VJJ	2ODE	1L9N		Sc=6.25133, min distance = 1.70447
GNAI3_HUMAN I	446248 CI	RAN_HUMAN Ful	8977 1dar	3CH5	2ODE	1IBR		Sc=6.56732, min distance = 2.02544
GNAI3_HUMAN I	446248 CI	RASH_HUMAN Fu	8977 1dar	2CE2	2ODE	1IAQ		Sc=6.56251, min distance = 2.23661
GNAI3_HUMAN I	6804 guar	DPOL_BPR69 Fu	8977 1dar	1CLQ	2ODE	1WAJ	0.99	Sc=6.43669, min distance = 2.03242
GNAI3_HUMAN I	6804 guar	EF1A_YEAST Fu	8977 1dar	2B7B	2ODE	1G7C	0.99	Sc=6.4464, min distance = 2.334451
GNAI3_HUMAN I	8582 Inos	PURA1_MOUSE Fu	8977 1dar	1LON	2ODE	1IWE	0.99	Sc=6.41736, min distance = 2.23055
GNAI3_HUMAN I	93082 Gab	CDC42_HUMAN Fu	8977 1dar	2NGR	2ODE	2ODB	0.99	Sc=6.59187, min distance = 2.20284
GNAI3_HUMAN I	93082 Gab	RAC3_HUMAN Fu	8977 1dar	2C2H	2ODE	2QME	0.99	Sc=6.59023, min distance = 2.58707
GNAO_MOUSE Fu	37792 gam	GNA13_MOUSE Fu	8977 1dar	1ZCB	3C7K	3CX8	0.99	Sc=6.5637, min distance = 2.124381
GNAO_MOUSE Fu	37792 gam	GNAI1_RAT Ful	8977 1dar	1SVK	3C7K	1AS0	0.99	Sc=6.56309, min distance = 2.48160
GNAO_MOUSE Fu	37792 gam	GNAT1_BOVIN Fu	8977 1dar	1TAD	3C7K	1TND	0.99	Sc=6.56843, min distance = 2.06435
GNAO_MOUSE Fu	444148 1a	RHA1_ARATH Fu	8977 1dar	2EFC	3C7K	2EFE	0.99	Sc=6.51678, min distance = 1.97989
GNAO_MOUSE Fu	445463 B-	TGM3_HUMAN Fu	8977 1dar	1VJJ	3C7K	1L9N		Sc=6.26536, min distance = 1.84379

GNAO_MOUSE F	446248 CI	RASH_HUMAN Fu	8977 1dar	2CE2	3C7K	1IAQ			Sc=6.57257, min distance = 2.28304
GNAO_MOUSE F	8582 Inos	PURA1_MOUSE F	8977 1dar	1LON	3C7K	1IWE		0.99	Sc=6.44289, min distance = 2.15389
GNAO_MOUSE F	93082 Gab	RAC3_HUMAN Fu	8977 1dar	2C2H	3C7K	2QME		0.99	Sc=6.60001, min distance = 2.14193
GNAO_MOUSE F	93082 Gab	RASH_HUMAN Fu	8977 1dar	2CE2	3C7K	121P		0.99	Sc=6.59971, min distance = 2.28157
GNAQ_MOUSE F	37792 gam	GNAT1_BOVIN F	8977 1dar	1TAD	2BCJ	1TND		0.99	Sc=6.55605, min distance = 2.21877
GNAQ_MOUSE F	37792 gam	RAB6B_HUMAN F	8977 1dar	2E9S	2BCJ	2FFQ		0.99	Sc=6.5543, min distance = 1.951981
GNAQ_MOUSE F	37792 gam	RAC1_HUMAN Fu	8977 1dar	1RYF	2BCJ	2FJU		0.99	Sc=6.55983, min distance = 2.11661
GNAQ_MOUSE F	37792 gam	RB11A_HUMAN F	8977 1dar	1OIX	2BCJ	1OIW		0.99	Sc=6.55392, min distance = 2.45459
GNAQ_MOUSE F	37792 gam	RHOA_HUMAN Fu	8977 1dar	1TX4	2BCJ	1CXZ		0.99	Sc=6.55781, min distance = 2.12134
GNAQ_MOUSE F	445463 B-	TGM3_HUMAN Fu	8977 1dar	1VJJ	2BCJ	1L9N			Sc=6.24196, min distance = 1.70816
GNAQ_MOUSE F	448405 CI	ARF1_RAT Full-	8977 1dar	1RRG	2BCJ	1RRF			Sc=6.50677, min distance = 2.12689
GNAQ_MOUSE F	6804 guar	EF1A_YEAST Fu	8977 1dar	2B7B	2BCJ	1G7C		0.99	Sc=6.44589, min distance = 2.10364
GNAQ_MOUSE F	93082 Gab	CDC42_HUMAN F	8977 1dar	2NGR	2BCJ	2ODB		0.99	Sc=6.59289, min distance = 2.23639
GNAQ_MOUSE F	93082 Gab	RAC3_HUMAN Fu	8977 1dar	2C2H	2BCJ	2QME		0.99	Sc=6.59087, min distance = 2.48896
GNAQ_MOUSE F	93082 Gab	RASH_HUMAN Fu	8977 1dar	2CE2	2BCJ	121P		0.99	Sc=6.58619, min distance = 2.02969
GNAS2_BOVIN	8977 1dar	GNAI1_RAT Ful	37792 gam	1AS0	1AZS	1SVK	43.43	0.99	Sc=6.4878, min distance = 2.536991
GNAS2_BOVIN	8977 1dar	GNAT1_BOVIN F	37792 gam	1TND	1AZS	1TAD		0.99	Sc=6.48483, min distance = 2.44193
GNAS2_BOVIN	8977 1dar	RAB6B_HUMAN F	37792 gam	2FFQ	1AZS	2E9S		0.99	Sc=6.50046, min distance = 2.22091
GNAS2_BOVIN	8977 1dar	RAC1_HUMAN Fu	37792 gam	2FJU	1AZS	1RYF		0.99	Sc=6.47759, min distance = 2.59977
GNAS2_BOVIN	8977 1dar	RB11A_HUMAN F	37792 gam	1OIW	1AZS	1OIX		0.99	Sc=6.49819, min distance = 2.21964
GNAT1_BOVIN	446248 CI	RAN_HUMAN Ful	8977 1dar	3CH5	1TAD	1IBR			Sc=6.56388, min distance = 1.98298
GNAT1_BOVIN	6804 guar	DPOL_BPR69 Fu	8977 1dar	1CLQ	1TAD	1WAJ		0.99	Sc=6.44589, min distance = 1.96057
GNAT1_BOVIN	6804 guar	EF1A_YEAST Fu	8977 1dar	2B7B	1TAD	1G7C		0.99	Sc=6.44404, min distance = 2.37128
GNAT1_BOVIN	93082 Gab	CDC42_HUMAN F	8977 1dar	2NGR	1TAD	2ODB		0.99	Sc=6.58873, min distance = 2.49506
GNAT1_BOVIN	93082 Gab	RAC3_HUMAN Fu	8977 1dar	2C2H	1TAD	2QME		0.99	Sc=6.58553, min distance = 2.49941
GNAT1_BOVIN	93082 Gab	RASH_HUMAN Fu	8977 1dar	2CE2	1TAD	121P		0.99	Sc=6.58328, min distance = 2.27110
GNMT_HUMAN F	439459 Hc	NIFD_KLEPN Fu	311 citri	1H1L	1R74	1QGU		0.92	Sc=5.95599, min distance = 2.14871
GNMT_HUMAN F	439459 Hc	NIFK_KLEPN Fu	311 citri	1H1L	1R74	1QGU		0.92	Sc=5.96229, min distance = 2.14953
GNMT_HUMAN F	447530	SRC_HUMAN Ful	311 citri	1O4L	1R74	1O4F			Sc=5.90032, min distance = 2.17580
GNMT_HUMAN F	447534 IS	SRC_HUMAN Ful	311 citri	1O4L	1R74	1O4J			Sc=5.99014, min distance = 1.04020
GNMT_RAT Ful	16741210	MTTA_THEAQ Fu	439155 Ad	1AQI	1D2H	2JG3			Sc=6.42551, min distance = 0.58410
GNMT_RAT Ful	188380 Ac	MCES_ENCCU Fu	439155 Ad	1RI1	1D2H	1Z3C		0.91	Sc=6.47932, min distance = 2.37173
GNMT_RAT Ful	445762 5'	MTTA_THEAQ Fu	34756 Acy	2ADM	1XVA	2IH2		0.93	Sc=6.28113, min distance = 2.08927
GNMT_RAT Ful	445971 CI	COMT_RAT Full-	34756 Acy	2CL5	1XVA	1H1D		0.92	Sc=6.12441, min distance = 2.20871
GNMT_RAT Ful	446535 CI	HNMT_HUMAN Fu	439155 Ad	2AOT	1D2H	1JQE		0.95	Sc=6.4237, min distance = 2.574163
GNMT_RAT Ful	60961 ade	PIMT_PYRFU Fu	439155 Ad	1JG1	1D2H	1JG2		0.84	Sc=6.21801, min distance = 2.30778
GNMT_RAT Ful	65482 sir	ERM_BACSU Ful	439155 Ad	1QAN	1D2H	1QAQ		0.88	Sc=6.49305, min distance = 2.39777

GNMT_RAT Ful	65482 sir	MCES_ENCCU Fu	439155 Ad	1RI1	1D2H	2HV9		0.88	Sc=6.009, min distance = 1.7745275
GNTK_ECOLI Fu	3540 1yds	KAPCA_BOVIN Fu	5957 Aden	1Q24	1KO5	1YDS			Sc=5.8, min distance = 2.209530719
GNTK_ECOLI Fu	3547 Fasu	KAPCA_BOVIN Fu	5957 Aden	1Q24	1KO5	1Q8W			Sc=5.90171, min distance = 2.38698
GNTK_ECOLI Fu	444564 AD	BIOD_ECOLI Fu	5957 Aden	1A82	1KO5	1BS1		0.91	Sc=5.9382, min distance = 2.472405
GNTK_ECOLI Fu	444564 AD	MYS2_DICDI Fu	5957 Aden	1FMW	1KO5	1W9I		0.91	Sc=5.95006, min distance = 0
GNTK_ECOLI Fu	447004 CI	MYS2_DICDI Fu	5957 Aden	1FMW	1KO5	1LVK			Sc=6.05029, min distance = 0
GNTK_ECOLI Fu	449240 1y	KAPCA_BOVIN Fu	5957 Aden	1Q24	1KO5	1YDR			Sc=5.88721, min distance = 2.37491
GNTK_ECOLI Fu	6022 Ader	BIOD_ECOLI Fu	5957 Aden	1A82	1KO5	1DAD		0.99	Sc=5.93748, min distance = 2.53659
GNTK_ECOLI Fu	6022 Ader	CLCN5_HUMAN Fu	5957 Aden	2J9L	1KO5	2JA3		0.99	Sc=5.93684, min distance = 0.95967
GNTK_ECOLI Fu	6022 Ader	KTHY_HUMAN Fu	5957 Aden	1E2Q	1KO5	1NN3		0.99	Sc=5.95286, min distance = 2.48344
GNTK_ECOLI Fu	6022 Ader	MUTS_ECOLI Fu	5957 Aden	1W7A	1KO5	1OH7		0.99	Sc=5.94478, min distance = 2.35650
GNTK_ECOLI Fu	6022 Ader	NIFH1_AZOVI Fu	5957 Aden	2C8V	1KO5	1FP6		0.99	Sc=5.9447, min distance = 2.282886
GNTK_ECOLI Fu	6022 Ader	Q72H90_THET2 I	5957 Aden	2BEK	1KO5	2BEJ		0.99	Sc=5.63256, min distance = 2.40438
GNTK_ECOLI Fu	6338562 C	MYS2_DICDI Fu	5957 Aden	1FMW	1KO5	1D0Y			Sc=6.18582, min distance = 0
GNTK_ECOLI Fu	91532 AMF	BIOD_ECOLI Fu	5957 Aden	1A82	1KO5	1DAG		0.99	Sc=6.01764, min distance = 2.42320
GOX_ASPNG Fu	11987634	GLPD_ECOLI Fu	444188 CI	2QCU	1CF3	2R4E		0.97	Sc=6.44053, min distance = 1.96877
GOX_ASPNG Fu	11987634	TYTR_TRYCR Fu	444188 CI	1BZL	1CF3	1AOG		0.97	Sc=6.1627, min distance = 2.335460
GOX_ASPNG Fu	16740985	FRDA_SHEFR Fu	444188 CI	1M64	1CF3	1E39		0.93	Sc=6.44165, min distance = 2.11042
GOX_ASPNG Fu	444502 CI	GSHR_HUMAN Fu	444188 CI	3DK9	1CF3	1BWC		0.98	Sc=6.15255, min distance = 2.02323
GOX_ASPNG Fu	444502 CI	TYTR_TRYCR Fu	444188 CI	1BZL	1CF3	1GXF		0.98	Sc=6.43663, min distance = 2.53216
GOX_ASPNG Fu	446013 1,	O28603_ARCFU I	444188 CI	1JNR	1CF3	1JNZ		0.94	Sc=6.16066, min distance = 2.12346
GOX_ASPNG Fu	449465 CI	GSHR_HUMAN Fu	444188 CI	3DK9	1CF3	3GRT		0.98	Sc=6.43959, min distance = 2.39141
GOX_ASPNG Fu	6420174 C	O28603_ARCFU I	444188 CI	1JNR	1CF3	2FJB		0.92	Sc=6.16765, min distance = 2.04343
GOX_ASPNG Fu	6420174 C	O28604_ARCFU I	444188 CI	1JNR	1CF3	2FJB		0.92	Sc=6.16554, min distance = 2.12939
GOX_PENAG Fu	446013 1,	O28603_ARCFU I	444188 CI	1JNR	1GPE	1JNZ		0.94	Sc=6.89821, min distance = 2.24262
GOX_PENAG Fu	446013 1,	O28604_ARCFU I	444188 CI	1JNR	1GPE	1JNZ		0.94	Sc=6.9001, min distance = 2.154548
GOX_PENAG Fu	449465 CI	GSHR_HUMAN Fu	444188 CI	3DK9	1GPE	3GRT		0.98	Sc=6.17728, min distance = 2.17032
GOX_PENAG Fu	6420174 C	O28603_ARCFU I	444188 CI	1JNR	1GPE	2FJB		0.92	Sc=6.9035, min distance = 1.912988
GOX_PENAG Fu	6420174 C	O28604_ARCFU I	444188 CI	1JNR	1GPE	2FJB		0.92	Sc=6.9036, min distance = 1.961548
GOX_SPIOL Fu	445395 Re	PYRDB_LACLM Fu	444301 FM	1EP2	1AL8	1EP3		0.77	Sc=6.65875, min distance = 2.50332
GOX_SPIOL Fu	445395 Re	PYRK_LACLM Fu	444301 FM	1EP2	1AL8	1EP3		0.77	Sc=6.65927, min distance = 2.43351
GOX_SPIOL Fu	446995 FM	CYB2_YEAST Fu	444243 FA	1KBI	1GOX	1LTD	40.97	0.77	Sc=6.2024, min distance = 2.539698
GP1BA_HUMAN I	152217 2,	DYR_CANAL Ful	78165 MES	1M79	1P8V	1M78			Sc=6.01426, min distance = 0.65826
GPD1L_HUMAN I	446050 CI	G3P_HUMAN Ful	5893 nadi	1U8F	2PLA	3GPD		0.95	Sc=6.77809, min distance = 1.51125
GPD1L_HUMAN I	446288 NA	G3P_PALVE Ful	5893 nadi	1DSS	2PLA	1IHX			Sc=6.76834, min distance = 1.89973
GPD1L_HUMAN I	6022 Ader	Q5SI02_THET8 I	5893 nadi	2BJK	2PLA	2BJA			Sc=6.23615, min distance = 2.27851
GPDA_HUMAN Fu	440641 1m	CGGR_BACSU Fu	668 Glyce	3BXF	1WPQ	3BXH			Sc=6.00403, min distance = 0.52905

GPDA_HUMAN Fu	446050 CI	G3P_HUMAN Ful	5893 nadi	1U8F	1WPQ	3GPD		0.95	Sc=6.77481, min distance = 2.29390
GPDA_HUMAN Fu	6022 Ader	Q5SI02_THET8 I	5893 nadi	2BJK	1WPQ	2BJA			Sc=6.09583, min distance = 2.23746
GPDA_LEIME Fu	444564 AD	ARO1_EMENI Ful	5893 nadi	1SG6	1EVZ	1NVA			Sc=6.26369, min distance = 2.17252
GPDA_LEIME Fu	446050 CI	G3P_HUMAN Ful	5893 nadi	1U8F	1EVZ	3GPD		0.95	Sc=5.97181, min distance = 2.19127
GPMI_BACST Fu	1005 phos	KDSA_AQUAE Ful	439278 ph	1PE1	1O98	2NWR			Sc=5.6763, min distance = 2.101825
GPMI_BACST Fu	439168 Tr	TPIS_PLAFA Ful	439183 1i	1M7O	1EJJ	1M7P		0.84	Sc=5.69982, min distance = 1.93403
GPMI_BACST Fu	447550 ph	TPIS_TRYBB Ful	439183 1i	1IIH	1EJJ	4TIM		1	Sc=5.7597, min distance = 2.210761
GRFIN_GRISQ I	101798 Me	LEC_GALNI Ful	185698 al	1JPC	2GUD	1MSA		0.94	Sc=5.7749, min distance = 2.509750
GRFIN_GRISQ I	101798 Me	Q8XUA5_RALSO I	185698 al	2CHH	2GUD	1UQX		0.94	Sc=5.75117, min distance = 2.58674
GRFIN_GRISQ I	151504 D-	CONA_CANEN Ful	185698 al	1CVN	2GUD	1I3H			Sc=5.92902, min distance = 0.55628
GRFIN_GRISQ I	151504 D-	CVN_NOSEL Ful	185698 al	2RDK	2GUD	2PYS			Sc=5.77335, min distance = 2.49261
GRHPR_HUMAN I	440141 9i	DHB1_HUMAN Ful	5886 NADF	1QYV	2GCG	1QYW			Sc=6.37677, min distance = 2.18727
GRHPR_HUMAN I	440334 Ni	DAPB_ECOLI Ful	5886 NADF	1DIH	2GCG	1DRW		0.91	Sc=6.30338, min distance = 2.00992
GRIA2_RAT Ful	24820116	GGT_ECOLI Ful	33032 L-g	2DG5	2GFE	2Z8I			Sc=5.74014, min distance = 2.43156
GRIA2_RAT Ful	24820118	GGT_ECOLI Ful	33032 L-g	2DG5	2GFE	2Z8K			Sc=5.7478, min distance = 2.520347
GRIA2_RAT Ful	449178 gl	GLMS_ECOLI Ful	33032 L-g	1XFF	2GFE	1XFG		0.81	Sc=5.87031, min distance = 1.99719
GRIA2_RAT Ful	449234 CI	GRIK2_RAT Ful	10255 kai	1TT1	1FTK	1YAE	54.94	0.83	Sc=6.45092, min distance = 2.33327
GRIA2_RAT Ful	95883 4-M	GRIK2_RAT Ful	10255 kai	1TT1	1FTK	1SD3	54.94		Sc=5.86725, min distance = 2.24781
GRIA2_RAT Ful	95883 4-M	GRIK2_RAT Ful	40539 Qui	1S9T	1P1O	1SD3	54.94		Sc=5.9108, min distance = 2.284014
GRIK1_RAT Ful	10255 kai	GRIA2_RAT Ful	447249 AT	1N0T	1VSO	1FTK	53.94		Sc=5.96895, min distance = 2.37013
GRIK1_RAT Ful	107883 Di	GRIA2_RAT Ful	447249 AT	1N0T	1VSO	1XHY	53.94		Sc=5.98594, min distance = 2.10994
GRIK1_RAT Ful	126569 5-	GRIA2_RAT Ful	447249 AT	1N0T	1VSO	1MQI	53.94		Sc=5.90952, min distance = 2.29393
GRIK1_RAT Ful	158397 am	GRIA2_RAT Ful	33032 L-g	2GFE	1YCJ	1FTM	53.94		Sc=6.03033, min distance = 0.65848
GRIK1_RAT Ful	158397 am	GRIA2_RAT Ful	447249 AT	1N0T	1VSO	1FTM	53.94		Sc=5.87864, min distance = 1.49133
GRIK1_RAT Ful	167842 1m	GRIA2_RAT Ful	33032 L-g	2GFE	1YCJ	1MY3	53.94		Sc=6.13744, min distance = 1.02032
GRIK1_RAT Ful	167842 1m	GRIA2_RAT Ful	447249 AT	1N0T	1VSO	1MY3	53.94		Sc=5.93546, min distance = 2.24447
GRIK1_RAT Ful	24820116	GGT_ECOLI Ful	33032 L-g	2DG5	1YCJ	2Z8I			Sc=5.72573, min distance = 2.13140
GRIK1_RAT Ful	24820117	GGT_ECOLI Ful	33032 L-g	2DG5	1YCJ	2Z8J			Sc=5.85289, min distance = 2.32980
GRIK1_RAT Ful	24820118	GGT_ECOLI Ful	33032 L-g	2DG5	1YCJ	2Z8K			Sc=5.73773, min distance = 2.23024
GRIK1_RAT Ful	24894162	GRIA2_RAT Ful	447249 AT	1N0T	1VSO	3BKI	53.94		Sc=6.1219, min distance = 1.913752
GRIK1_RAT Ful	3140 dnqx	GRIA2_RAT Ful	447249 AT	1N0T	1VSO	1FTL	53.94		Sc=6.22099, min distance = 2.09948
GRIK1_RAT Ful	40539 Qui	GRIA2_RAT Ful	33032 L-g	2GFE	1YCJ	1P1O	53.94		Sc=5.96134, min distance = 0.79769
GRIK1_RAT Ful	4369370 C	GRIA2_RAT Ful	447249 AT	1N0T	1VSO	1SYI	53.94		Sc=6.16907, min distance = 2.04195
GRIK1_RAT Ful	440053 Wi	GRIA2_RAT Ful	33032 L-g	2GFE	1YCJ	1MQJ	53.94		Sc=6.12408, min distance = 1.03583
GRIK1_RAT Ful	440053 Wi	GRIA2_RAT Ful	447249 AT	1N0T	1VSO	1MQJ	53.94		Sc=5.89494, min distance = 2.20496
GRIK1_RAT Ful	447081 1m	GRIA2_RAT Ful	33032 L-g	2GFE	1YCJ	1M5B	53.94		Sc=6.29213, min distance = 2.11619
GRIK1_RAT Ful	447081 1m	GRIA2_RAT Ful	447249 AT	1N0T	1VSO	1M5B	53.94		Sc=6.21031, min distance = 1.69297

GRIK1_RAT Fu	447082 1m	GRIA2_RAT Ful	33032 L-g	2GFE	1YCJ	1M5C	53.94		Sc=5.9404, min distance = 2.470461
GRIK1_RAT Fu	447082 1m	GRIA2_RAT Ful	447249 AT	1N0T	1VSO	1M5C	53.94		Sc=5.73838, min distance = 2.085298
GRIK1_RAT Fu	447083 ac	GRIA2_RAT Ful	33032 L-g	2GFE	1YCJ	1M5E	53.94		Sc=6.19185, min distance = 0.27258
GRIK1_RAT Fu	447195 1m	GRIA2_RAT Ful	33032 L-g	2GFE	1YCJ	1MQD	53.94		Sc=5.97801, min distance = 0.55121
GRIK1_RAT Fu	447196 5;	GRIA2_RAT Ful	33032 L-g	2GFE	1YCJ	1MY4	53.94		Sc=6.13262, min distance = 1.08123
GRIK1_RAT Fu	447196 5;	GRIA2_RAT Ful	447249 AT	1N0T	1VSO	1MY4	53.94		Sc=5.91977, min distance = 2.07810
GRIK1_RAT Fu	449178 gl	GLMS_ECOLI Fu	33032 L-g	1XFF	1YCJ	1XFG		0.81	Sc=5.8556, min distance = 2.093713
GRIK1_RAT Fu	5289518 T	GRIA2_RAT Ful	447249 AT	1N0T	1VSO	2AIX	53.94		Sc=5.99177, min distance = 2.01823
GRIK1_RAT Fu	657004 1s	GRIA2_RAT Ful	447249 AT	1N0T	1VSO	1SYH	53.94		Sc=6.14726, min distance = 2.03263
GRIK2_RAT Fu	107883 Di	GRIA2_RAT Ful	10255 kai	1FTK	1TT1	1XHY	54.94	0.88	Sc=6.15398, min distance = 2.45808
GRIK2_RAT Fu	107883 Di	GRIA2_RAT Ful	33032 L-g	2GFE	1S50	1XHY	54.94		Sc=6.19685, min distance = 2.07093
GRIK2_RAT Fu	126569 5-	GRIA2_RAT Ful	10255 kai	1FTK	1TT1	1MQI	54.94		Sc=6.04275, min distance = 2.15688
GRIK2_RAT Fu	158397 am	GRIA2_RAT Ful	40539 Qui	1P1O	1S9T	1FTM	54.94		Sc=6.01478, min distance = 2.51303
GRIK2_RAT Fu	167842 1m	GRIA2_RAT Ful	10255 kai	1FTK	1TT1	1MY3	54.94		Sc=6.04275, min distance = 2.11527
GRIK2_RAT Fu	167842 1m	GRIA2_RAT Ful	40539 Qui	1P1O	1S9T	1MY3	54.94		Sc=6.10516, min distance = 2.23060
GRIK2_RAT Fu	24820116	GGT_ECOLI Ful	33032 L-g	2DG5	1S50	2Z8I			Sc=5.69346, min distance = 1.95208
GRIK2_RAT Fu	24820117	GGT_ECOLI Ful	33032 L-g	2DG5	1S50	2Z8J			Sc=5.83588, min distance = 1.92882
GRIK2_RAT Fu	24820118	GGT_ECOLI Ful	33032 L-g	2DG5	1S50	2Z8K			Sc=5.69505, min distance = 2.06661
GRIK2_RAT Fu	4369370 C	GRIA2_RAT Ful	10255 kai	1FTK	1TT1	1SYI	54.94		Sc=6.26754, min distance = 1.91199
GRIK2_RAT Fu	4369370 C	GRIA2_RAT Ful	40539 Qui	1P1O	1S9T	1SYI	54.94		Sc=6.31676, min distance = 2.10760
GRIK2_RAT Fu	440053 Wi	GRIA2_RAT Ful	10255 kai	1FTK	1TT1	1MQJ	54.94		Sc=6.02784, min distance = 2.13171
GRIK2_RAT Fu	440053 Wi	GRIA2_RAT Ful	33032 L-g	2GFE	1S50	1MQJ	54.94		Sc=6.06868, min distance = 2.36788
GRIK2_RAT Fu	440053 Wi	GRIA2_RAT Ful	40539 Qui	1P1O	1S9T	1MQJ	54.94		Sc=6.09253, min distance = 2.13354
GRIK2_RAT Fu	447081 1m	GRIA2_RAT Ful	40539 Qui	1P1O	1S9T	1M5B	54.94		Sc=6.31711, min distance = 2.30556
GRIK2_RAT Fu	447195 1m	GRIA2_RAT Ful	10255 kai	1FTK	1TT1	1MQD	54.94		Sc=5.88106, min distance = 1.93073
GRIK2_RAT Fu	447195 1m	GRIA2_RAT Ful	40539 Qui	1P1O	1S9T	1MQD	54.94		Sc=5.93465, min distance = 2.29152
GRIK2_RAT Fu	447196 5;	GRIA2_RAT Ful	10255 kai	1FTK	1TT1	1MY4	54.94		Sc=6.04165, min distance = 2.06057
GRIK2_RAT Fu	447196 5;	GRIA2_RAT Ful	40539 Qui	1P1O	1S9T	1MY4	54.94		Sc=6.10594, min distance = 2.10787
GRIK2_RAT Fu	447407 1r	GRIA2_RAT Ful	10255 kai	1FTK	1TT1	1NNK	54.94		Sc=6.19275, min distance = 1.98965
GRIK2_RAT Fu	449178 gl	GLMS_ECOLI Fu	33032 L-g	1XFF	1S50	1XFG		0.81	Sc=5.8556, min distance = 2.032667
GRIK2_RAT Fu	5289518 T	GRIA2_RAT Ful	10255 kai	1FTK	1TT1	2AIX	54.94		Sc=6.10137, min distance = 2.25496
GRIK2_RAT Fu	657004 1s	GRIA2_RAT Ful	10255 kai	1FTK	1TT1	1SYH	54.94		Sc=6.25182, min distance = 2.21670
GRIK2_RAT Fu	657004 1s	GRIA2_RAT Ful	40539 Qui	1P1O	1S9T	1SYH	54.94		Sc=6.31616, min distance = 1.96259
GRK6_HUMAN Fu	15993 dAT	PRIM_BPT7 Ful	33113 gam	1E0J	2ACX	1CR2		0.97	Sc=5.94495, min distance = 1.71791
GRK6_HUMAN Fu	23656870	CSK2A_MAIZE Fu	33113 gam	1LP4	2ACX	2PVN			Sc=6.49702, min distance = 1.52491
GRK6_HUMAN Fu	24756824	CSK2A_MAIZE Fu	33113 gam	1LP4	2ACX	3BE9			Sc=6.43199, min distance = 1.81423
GRK6_HUMAN Fu	24779674	CSK2A_MAIZE Fu	33113 gam	1LP4	2ACX	2PVH			Sc=6.21494, min distance = 2.31931

GRK6_HUMAN Fu	24851689	PIM1_HUMAN Fu	33113 gam	1XR1	2ACX	3CY2			Sc=6.09383, min distance = 2.26905
GRK6_HUMAN Fu	3064778 H	ROCK1_HUMAN Fu	33113 gam	2V55	2ACX	2ETK			Sc=6.01515, min distance = 2.18137
GRK6_HUMAN Fu	444564 AD	PDK2_RAT Full-	33113 gam	3CRL	2ACX	1JM6		0.91	Sc=5.92342, min distance = 2.14503
GRK6_HUMAN Fu	445479 CI	CSK21_HUMAN Fu	33113 gam	2PVR	2ACX	3BQC			Sc=6.20406, min distance = 2.50541
GRK6_HUMAN Fu	445479 CI	CSK2A_MAIZE Fu	33113 gam	1LP4	2ACX	1F0Q			Sc=6.18286, min distance = 2.59490
GRK6_HUMAN Fu	448042 2e	ROCK1_HUMAN Fu	33113 gam	2V55	2ACX	2ETR			Sc=5.93628, min distance = 2.58460
GRK6_HUMAN Fu	5287844 i	GSK3B_HUMAN Fu	33113 gam	1J1B	2ACX	1UV5			Sc=6.26713, min distance = 2.04354
GRK6_HUMAN Fu	5326637 1	CSK2A_MAIZE Fu	33113 gam	1LP4	2ACX	1M2Q			Sc=6.13021, min distance = 2.21815
GRK6_HUMAN Fu	5326739 i	GSK3B_HUMAN Fu	33113 gam	1J1B	2ACX	1Q41			Sc=6.22012, min distance = 2.07425
GRK6_HUMAN Fu	5326976 1	CSK2A_MAIZE Fu	33113 gam	1LP4	2ACX	1ZOE			Sc=5.62316, min distance = 2.05706
GRK6_HUMAN Fu	60961 ade	PIM1_HUMAN Fu	33113 gam	1XR1	2ACX	1YI4		0.93	Sc=5.74678, min distance = 2.73638
GRK6_HUMAN Fu	60961 ade	SKY1_YEAST Fu	33113 gam	1Q99	2ACX	1Q97		0.93	Sc=6.08088, min distance = 2.52241
GRLF1_HUMAN I	446248 CI	RASH_HUMAN Fu	36735 Gpp	1CTQ	3C5H	1IAQ			Sc=6.48362, min distance = 2.18706
GRLF1_HUMAN I	93082 Gab	CDC42_HUMAN Fu	36735 Gpp	1NF3	3C5H	2ODB		0.99	Sc=6.52893, min distance = 2.18695
GRLF1_HUMAN I	93082 Gab	FTSY_THEAQ Fu	36735 Gpp	2J7P	3C5H	1RJ9		0.99	Sc=6.53645, min distance = 2.17534
GRLF1_HUMAN I	93082 Gab	RASH_HUMAN Fu	36735 Gpp	1CTQ	3C5H	121P		0.99	Sc=6.52564, min distance = 2.36266
GRM1_RAT Ful	10255 kai	GRIK2_RAT Ful	33032 L-g	1S50	1EWK	1TT1			Sc=6.09058, min distance = 2.14447
GRM1_RAT Ful	104766 tr	GRM3_RAT Full-	33032 L-g	2E4U	1EWK	2E4X	44.1		Sc=5.96888, min distance = 2.54100
GRM1_RAT Ful	24820117	GGT_ECOLI Ful	33032 L-g	2DG5	1EWK	2Z8J			Sc=5.8405, min distance = 1.888625
GRM1_RAT Ful	24820118	GGT_ECOLI Ful	33032 L-g	2DG5	1EWK	2Z8K			Sc=5.71487, min distance = 2.26884
GRM1_RAT Ful	40539 Qui	GRIK2_RAT Ful	33032 L-g	1S50	1EWK	1S9T			Sc=5.90692, min distance = 1.38055
GRM1_RAT Ful	440053 Wi	GRIA2_RAT Ful	33032 L-g	2GFE	1EWK	1MQJ			Sc=6.05624, min distance = 1.71318
GRM1_RAT Ful	445939 1g	PPTA_ECOLI Fu	23831 HEF	1GYX	1EWK	1GYY			Sc=5.77798, min distance = 2.45990
GRM1_RAT Ful	5310979 D	GRM3_RAT Full-	33032 L-g	2E4U	1EWK	2E4V	44.1		Sc=6.10613, min distance = 2.20179
GRM1_RAT Ful	6604704 T	GRM3_RAT Full-	33032 L-g	2E4U	1EWK	2E4W	44.1		Sc=5.98116, min distance = 2.51616
GRM3_RAT Ful	24820116	GGT_ECOLI Ful	33032 L-g	2DG5	2E4U	2Z8I			Sc=5.72302, min distance = 2.24286
GRM7_RAT Ful	8200 TETR	LMBL1_HUMAN Fu	78165 MES	1OZ2	2E4Z	2RHI			Sc=5.65339, min distance = 2.30309
GRSA_ANEMI Fu	166760 Fa	PURP_METJA Fu	6083 aden	2R7M	1AMU	2R7N		0.76	Sc=6.39251, min distance = 2.16898
GRSA_ANEMI Fu	24752834	SYH_ECOLI Ful	6083 aden	1HTT	1AMU	2EL9			Sc=6.56957, min distance = 1.09214
GRSA_ANEMI Fu	2519 caff	PYGM_RABIT Fu	6083 aden	8GPB	1AMU	1GFZ			Sc=5.88874, min distance = 2.10652
GRSA_ANEMI Fu	378 4-nit	APEH_AERPE Fu	6140 L-ph	2HU5	1AMU	1VE7			Sc=5.93576, min distance = 2.29194
GRSA_ANEMI Fu	440641 1m	O57693_THETE I	6083 aden	1UXU	1AMU	1UXR			Sc=5.89046, min distance = 2.51127
GRSA_ANEMI Fu	445708 AM	NADE_ECOLI Fu	6083 aden	1WXI	1AMU	1WXE		0.91	Sc=6.3787, min distance = 2.076937
GRSA_ANEMI Fu	445736 5'	SYS_PYRHO Ful	6083 aden	2ZR2	1AMU	2DQ0		0.86	Sc=6.03032, min distance = 1.88190
GRSA_ANEMI Fu	446757 CI	PPB1_HUMAN Fu	6140 L-ph	1ZEF	1AMU	1ZED			Sc=6.00004, min distance = 1.34733
GRSA_ANEMI Fu	447832 AD	ACSA_SALTY Fu	6083 aden	2P2F	1AMU	1PG4		0.99	Sc=6.46611, min distance = 2.26912
GRSA_ANEMI Fu	5327121 I	PIM1_HUMAN Fu	6083 aden	1YXU	1AMU	2C3I			Sc=6.33275, min distance = 1.46637

GRSA_ANEMI F	5962 L-ly	THER_BACTH Fu	6140 L-ph	1OS0	1AMU	1KEI		Sc=5.72177, min distance = 2.46784
GRSA_ANEMI F	611002 Op	PIM1_HUMAN Fu	6083 aden	1YXU	1AMU	1YXX		Sc=6.21548, min distance = 1.79316
GRSA_ANEMI F	6305 L-tr	APX_STRGR Ful	6140 L-ph	1F2P	1AMU	1TF8		Sc=6.15508, min distance = 0.09155
GRSA_ANEMI F	657135 Op	PIM1_HUMAN Fu	6083 aden	1YXU	1AMU	1YXV		Sc=5.95108, min distance = 2.43831
GSH1_BRAJU F	40539 Qui	GRIA2_RAT Ful	33032 L-g	2GFE	2GWD	1P1O		Sc=5.82917, min distance = 1.98695
GSH1_BRAJU F	449178 gl	GLMS_ECOLI Fu	33032 L-g	1XFF	2GWD	1XFG	0.81	Sc=5.79116, min distance = 2.53172
GSH1_BRAJU F	5310984 2	GRM3_RAT Full	33032 L-g	2E4U	2GWD	2E4Y		Sc=5.90909, min distance = 2.56002
GSH1_ECOLI F	24178104	GLNA_HUMAN Fu	6022 Aden	2OJW	1VA6	2QC8		Sc=5.86907, min distance = 2.29009
GSH1_ECOLI F	36735 Gpp	PARM_ECOLX Fu	6022 Aden	1MWM	1VA6	2ZGZ	0.79	Sc=5.77928, min distance = 2.23651
GSH1_ECOLI F	40539 Qui	GRIK2_RAT Ful	33032 L-g	1S50	2D32	1S9T		Sc=5.83519, min distance = 2.27441
GSH1_ECOLI F	444842 CI	CDK2_HUMAN Fu	6022 Aden	1GY3	1VA6	1CKP		Sc=5.62862, min distance = 1.89710
GSH1_ECOLI F	447916 ac	RIO1_ARCFU Fu	6022 Aden	1ZTH	1VA6	1ZTF	0.95	Sc=6.2993, min distance = 2.236885
GSH1_ECOLI F	6083 aden	HSP71_HUMAN F	33113 gam	2E8A	2D32	1XQS	0.97	Sc=5.93435, min distance = 2.64559
GSH1_ECOLI F	66535 3'-	RNAS1_BOVIN F	6022 Aden	1O0H	1VA6	1RPF		Sc=6.24529, min distance = 2.02841
GSH1_ECOLI F	8977 1dan	PARM_ECOLX Fu	6022 Aden	1MWM	1VA6	2ZGY	0.8	Sc=5.72988, min distance = 2.22794
GSH1_ECOLI F	91532 AMP	BIOD_ECOLI Fu	6022 Aden	1DAD	1VA6	1DAG	0.99	Sc=5.76275, min distance = 2.00377
GSHB_ECOLI F	115260 S-	GSTM2_HUMAN F	124886 gl	1XW5	1GSA	2AB6	0.94	Sc=5.94828, min distance = 2.56757
GSHB_ECOLI F	16214825	CDK2_HUMAN Fu	6022 Aden	1GY3	1GSA	2UZE		Sc=6.36147, min distance = 1.79972
GSHB_ECOLI F	444104 II	GSTP1_MOUSE F	124886 gl	1GSY	1GSA	1GLP	0.9	Sc=5.95563, min distance = 1.98440
GSHB_ECOLI F	444564 AD	BIOD_ECOLI Fu	6022 Aden	1DAD	1GSA	1BS1	0.91	Sc=6.45812, min distance = 2.08067
GSHB_ECOLI F	447916 ac	RIO1_ARCFU Fu	6022 Aden	1ZTH	1GSA	1ZTF	0.95	Sc=6.26145, min distance = 2.34786
GSHB_ECOLI F	447955 1p	CDK2_HUMAN Fu	6022 Aden	1GY3	1GSA	1PXI		Sc=5.80053, min distance = 1.78764
GSHB_ECOLI F	5957 Aden	RK_BOVIN Full	6022 Aden	3C4Z	1GSA	3C4W	0.99	Sc=6.06948, min distance = 1.39955
GSHB_ECOLI F	6083 aden	PURP_METJA Fu	6022 Aden	2R7N	1GSA	2R7M	0.99	Sc=5.9157, min distance = 2.577654
GSHB_ECOLI F	6132 Cyti	O33839_THEMA	6022 Aden	1XJK	1GSA	1XJN		Sc=5.89105, min distance = 2.02026
GSHB_ECOLI F	6804 guan	NDK_PYRHO Ful	6022 Aden	2DYA	1GSA	2DXF	0.8	Sc=6.40603, min distance = 2.22027
GSHB_HUMAN F	115260 S-	GSTM2_HUMAN F	124886 gl	1XW5	2HGS	2AB6	0.94	Sc=6.41392, min distance = 2.15811
GSHB_HUMAN F	1540 1h1q	CDK2_HUMAN Fu	6022 Aden	1GY3	2HGS	1H1Q		Sc=6.39222, min distance = 1.75260
GSHB_HUMAN F	188966 dA	HSLU_ECOLI Fu	6022 Aden	1HQY	2HGS	1G4A	0.98	Sc=6.43349, min distance = 2.18960
GSHB_HUMAN F	23653523	CDK2_HUMAN Fu	6022 Aden	1GY3	2HGS	2R3O		Sc=6.41645, min distance = 2.10869
GSHB_HUMAN F	23653526	CDK2_HUMAN Fu	6022 Aden	1GY3	2HGS	2R3R		Sc=6.31302, min distance = 2.15168
GSHB_HUMAN F	24864081	CDK2_HUMAN Fu	6022 Aden	1GY3	2HGS	2VTS		Sc=6.38029, min distance = 2.17939
GSHB_HUMAN F	444104 II	GSTP1_MOUSE F	124886 gl	1GSY	2HGS	1GLP	0.9	Sc=6.42334, min distance = 1.81744
GSHB_HUMAN F	447916 ac	RIO1_ARCFU Fu	6022 Aden	1ZTH	2HGS	1ZTF	0.95	Sc=6.26828, min distance = 2.81838
GSHB_HUMAN F	5327096 2	CDK2_HUMAN Fu	6022 Aden	1GY3	2HGS	2BTR		Sc=6.15277, min distance = 1.80778
GSHB_HUMAN F	6083 aden	HSP71_HUMAN F	6022 Aden	1S3X	2HGS	1XQS	0.99	Sc=6.37418, min distance = 2.10260
GSHB_HUMAN F	6804 guan	NDK_PYRHO Ful	6022 Aden	2DYA	2HGS	2DXF	0.8	Sc=6.41587, min distance = 2.15763

GSHB_HUMAN F	9547890 I	CDK2_HUMAN Fu	6022 Aden	1GY3	2HGS	1W8C			Sc=6.29075, min distance = 1.80786
GSHB_HUMAN F	9991833 S	CDK2_HUMAN Fu	6022 Aden	1GY3	2HGS	2R3H			Sc=6.14034, min distance = 2.13687
GSHR_ECOLI F	11987634	GLPD_ECOLI Fu	444188 CI	2QCU	1GES	2R4E		0.97	Sc=6.1511, min distance = 2.111034
GSHR_ECOLI F	439153 Di	ADH1E_HORSE F	5893 nadi	1MGO	1GEU	2JHF		0.79	Sc=6.28412, min distance = 1.94296
GSHR_ECOLI F	440141 9i	GSHR_HUMAN Fu	5886 NADP	3DJJ	1GET	1GRA			Sc=5.92366, min distance = 2.71686
GSHR_ECOLI F	449465 CI	GSHR_HUMAN Fu	5886 NADP	3DJJ	1GET	3GRT		0.82	Sc=6.8696, min distance = 2.182931
GSHR_HUMAN F	11987634	TYTR_TRYCR Fu	444188 CI	1BZL	3DK9	1AOG	36.08	0.97	Sc=6.87358, min distance = 2.43930
GSHR_HUMAN F	11987634	TYTR_TRYCR Fu	444502 CI	1GXF	1BWC	1AOG	36.08	0.95	Sc=6.87578, min distance = 2.41721
GSHR_HUMAN F	12389 Tet	LTC4S_HUMAN F	124886 gl	2PNO	3DK4	2UUI			Sc=5.78556, min distance = 1.00951
GSHR_HUMAN F	16740985	FRDA_SHEFR Fu	444188 CI	1M64	3DK9	1E39		0.93	Sc=6.43617, min distance = 1.95815
GSHR_HUMAN F	16740985	FRDA_SHEFR Fu	444502 CI	1QJD	1BWC	1E39		0.92	Sc=6.87057, min distance = 2.08859
GSHR_HUMAN F	2737071 Z	PTGD2_HUMAN F	124886 gl	2CVD	3DK4	2VCZ			Sc=5.76659, min distance = 1.22382
GSHR_HUMAN F	444563 CI	FRDA_SHEFR Fu	444502 CI	1QJD	1BWC	1P2E		0.98	Sc=6.86673, min distance = 2.13065
GSHR_HUMAN F	444969 CI	FRDA_SHEFR Fu	444502 CI	1QJD	1BWC	1JRY		0.98	Sc=6.43135, min distance = 2.14253
GSHR_HUMAN F	446013 1,	O28603_ARCFU	444188 CI	1JNR	3DK9	1JNZ		0.94	Sc=6.44388, min distance = 2.07514
GSHR_HUMAN F	446013 1,	O28604_ARCFU	444188 CI	1JNR	3DK9	1JNZ		0.94	Sc=6.88291, min distance = 2.07324
GSHR_HUMAN F	446546 CI	FRDA_SHEFR Fu	444502 CI	1QJD	1BWC	1KSS		0.98	Sc=6.87246, min distance = 2.14674
GSHR_HUMAN F	448815 NA	TYTR_CRIFA Fu	444188 CI	1FEC	3DK9	1TYP	35.63	0.82	Sc=6.13691, min distance = 1.83048
GSHR_YEAST F	11987634	GLPD_ECOLI Fu	444188 CI	2QCU	2HQM	2R4E		0.97	Sc=6.44042, min distance = 2.00290
GSHR_YEAST F	448815 NA	TYTR_CRIFA Fu	444188 CI	1FEC	2HQM	1TYP		0.82	Sc=6.26513, min distance = 2.19458
GSK3B_HUMAN	10109823	PIM1_HUMAN Fu	444345 1c	1YHS	1Q3D	3CY3	32.04		Sc=6.15903, min distance = 2.93865
GSK3B_HUMAN	10125830	CHK1_HUMAN Fu	444345 1c	1NVR	1Q3D	2AYP	30.65		Sc=6.3425, min distance = 2.162234
GSK3B_HUMAN	10224714	CDK2_HUMAN Fu	444345 1c	1AQ1	1Q3D	3DDP	35.44		Sc=6.32453, min distance = 2.21507
GSK3B_HUMAN	10224714	CDK2_HUMAN Fu	6022 Aden	1GY3	1J1C	3DDP	35.44		Sc=6.48837, min distance = 1.72095
GSK3B_HUMAN	11957417	CDK2_HUMAN Fu	6022 Aden	1GY3	1J1C	2I40	35.44		Sc=6.28325, min distance = 2.45399
GSK3B_HUMAN	11963557	STK6_HUMAN Fu	6022 Aden	1MQ4	1J1C	2J4Z	37.24		Sc=6.45923, min distance = 2.03500
GSK3B_HUMAN	15942671	FAK1_CHICK Fu	33113 gam	2J0L	1J1B	2J0J	28.87		Sc=6.34222, min distance = 2.06918
GSK3B_HUMAN	160355 ro	CDK2_HUMAN Fu	444345 1c	1AQ1	1Q3D	3DDQ	35.44		Sc=6.0801, min distance = 2.331885
GSK3B_HUMAN	160355 ro	CDK2_HUMAN Fu	6022 Aden	1GY3	1J1C	3DDQ	35.44		Sc=6.33171, min distance = 1.54402
GSK3B_HUMAN	16040259	EGFR_HUMAN Fu	33113 gam	2ITN	1J1B	2ITQ	29.56		Sc=6.36357, min distance = 2.07448
GSK3B_HUMAN	16040273	IRAK4_HUMAN F	33113 gam	2OID	1J1B	2OIC	33.68		Sc=6.41256, min distance = 0.95015
GSK3B_HUMAN	16046126	CDK2_HUMAN Fu	6022 Aden	1GY3	1J1C	2DS1	35.44		Sc=6.32971, min distance = 2.63583
GSK3B_HUMAN	16122608	PDPK1_HUMAN F	444345 1c	1OKY	1Q3D	2PE1	29.03		Sc=6.21665, min distance = 2.54780
GSK3B_HUMAN	16214823	CDK2_HUMAN Fu	444345 1c	1AQ1	1Q3D	2UZB	35.44		Sc=5.95249, min distance = 2.97856
GSK3B_HUMAN	16214823	CDK2_HUMAN Fu	6022 Aden	1GY3	1J1C	2UZB	35.44		Sc=6.13953, min distance = 2.03010
GSK3B_HUMAN	16214825	CDK2_HUMAN Fu	6022 Aden	1GY3	1J1C	2UZE	35.44		Sc=6.2772, min distance = 2.152169
GSK3B_HUMAN	16214826	CDK2_HUMAN Fu	6022 Aden	1GY3	1J1C	2UZL	35.44		Sc=6.1579, min distance = 2.195730

GSK3B_HUMAN I	176870 Er	EGFR_HUMAN Fu	33113 gam	2ITN	1J1B	1M17	29.56		Sc=6.03246, min distance = 1.99831
GSK3B_HUMAN I	193758 Va	CDGT2_BACCI Fu	23831 HEP	1OT1	1H8F	1DTU			Sc=5.66724, min distance = 1.52249
GSK3B_HUMAN I	23653515	CDK2_HUMAN Fu	444345 1c	1AQ1	1Q3D	2R3F	35.44		Sc=5.78808, min distance = 3.19975
GSK3B_HUMAN I	23653515	CDK2_HUMAN Fu	6022 Aden	1GY3	1J1C	2R3F	35.44		Sc=5.96764, min distance = 2.65788
GSK3B_HUMAN I	23653516	CDK2_HUMAN Fu	6022 Aden	1GY3	1J1C	2R3G	35.44		Sc=6.13462, min distance = 2.44278
GSK3B_HUMAN I	23653518	CDK2_HUMAN Fu	6022 Aden	1GY3	1J1C	2R3J	35.44		Sc=6.11904, min distance = 2.56212
GSK3B_HUMAN I	23653519	CDK2_HUMAN Fu	6022 Aden	1GY3	1J1C	2R3K	35.44		Sc=6.22132, min distance = 2.56666
GSK3B_HUMAN I	23653520	CDK2_HUMAN Fu	6022 Aden	1GY3	1J1C	2R3L	35.44		Sc=6.23132, min distance = 2.56502
GSK3B_HUMAN I	23653521	CDK2_HUMAN Fu	6022 Aden	1GY3	1J1C	2R3M	35.44		Sc=6.37802, min distance = 2.39578
GSK3B_HUMAN I	23653522	CDK2_HUMAN Fu	6022 Aden	1GY3	1J1C	2R3N	35.44		Sc=6.20681, min distance = 2.05339
GSK3B_HUMAN I	23653524	CDK2_HUMAN Fu	6022 Aden	1GY3	1J1C	2R3P	35.44		Sc=6.19212, min distance = 2.48008
GSK3B_HUMAN I	23727982	CDK2_HUMAN Fu	444345 1c	1AQ1	1Q3D	3BHU	35.44		Sc=6.1766, min distance = 2.614148
GSK3B_HUMAN I	24779675	CSK2A_MAIZE Fu	33113 gam	1LP4	1J1B	2PVJ	33.23		Sc=6.2904, min distance = 2.017783
GSK3B_HUMAN I	24779678	CSK2A_MAIZE Fu	33113 gam	1LP4	1J1B	2PVM	33.23		Sc=6.40702, min distance = 2.27407
GSK3B_HUMAN I	24851689	PIM1_HUMAN Fu	444345 1c	1YHS	1Q3D	3CY2	32.04		Sc=6.11051, min distance = 2.35339
GSK3B_HUMAN I	24864081	CDK2_HUMAN Fu	6022 Aden	1GY3	1J1C	2VTS	35.44		Sc=6.23292, min distance = 2.54022
GSK3B_HUMAN I	24916751	CDK2_HUMAN Fu	444345 1c	1AQ1	1Q3D	3DOG	35.44		Sc=6.07475, min distance = 2.30554
GSK3B_HUMAN I	24963048	AKT2_HUMAN Fu	33113 gam	1O6L	1J1B	3E87	28.62		Sc=6.44914, min distance = 2.06130
GSK3B_HUMAN I	3064778 H	ROCK1_HUMAN Fu	33113 gam	2V55	1J1B	2ETK	30.47		Sc=6.06399, min distance = 2.15261
GSK3B_HUMAN I	3543 1g5s	CDK2_HUMAN Fu	444345 1c	1AQ1	1Q3D	1G5S	35.44		Sc=6.18188, min distance = 2.69675
GSK3B_HUMAN I	3547 Fasu	KAPCA_BOVIN Fu	444345 1c	1STC	1Q3D	1Q8W	31.63		Sc=5.80927, min distance = 2.76722
GSK3B_HUMAN I	3547 Fasu	ROCK1_HUMAN Fu	33113 gam	2V55	1J1B	2ESM	30.47		Sc=5.90464, min distance = 2.26748
GSK3B_HUMAN I	36735 Gpp	PARM_ECOLX Fu	6022 Aden	1MWM	1J1C	2ZGZ		0.79	Sc=5.73554, min distance = 0.94158
GSK3B_HUMAN I	3973 nche	PIM1_HUMAN Fu	444345 1c	1YHS	1Q3D	1YI3	32.04		Sc=5.79238, min distance = 2.38144
GSK3B_HUMAN I	4369136 C	CDK2_HUMAN Fu	444345 1c	1AQ1	1Q3D	1DM2	35.44		Sc=6.10515, min distance = 2.93667
GSK3B_HUMAN I	444367 CI	CSK2A_MAIZE Fu	33113 gam	1LP4	1J1B	1OM1	33.23		Sc=6.12927, min distance = 2.09716
GSK3B_HUMAN I	444564 AI	PDK2_RAT Full=	33113 gam	3CRL	1J1B	1JM6		0.91	Sc=5.93102, min distance = 2.29209
GSK3B_HUMAN I	447656 1c	CDK2_HUMAN Fu	6022 Aden	1GY3	1J1C	1OIY	35.44		Sc=6.2232, min distance = 1.676520
GSK3B_HUMAN I	447962 1p	CDK2_HUMAN Fu	6022 Aden	1GY3	1J1C	2C5N	35.44		Sc=6.27352, min distance = 1.36700
GSK3B_HUMAN I	448043 2g	ROCK1_HUMAN Fu	33113 gam	2V55	1J1B	3D9V	30.47		Sc=5.97728, min distance = 2.12872
GSK3B_HUMAN I	448222 NE	ENPL_CANFA Fu	6022 Aden	1TC6	1J1C	1QY5		0.81	Sc=6.27316, min distance = 2.05901
GSK3B_HUMAN I	448293 CI	CDK2_HUMAN Fu	6022 Aden	1GY3	1J1C	1R78	35.44		Sc=6.42396, min distance = 2.28030
GSK3B_HUMAN I	449087 1v	CDK2_HUMAN Fu	6022 Aden	1GY3	1J1C	1VYW	35.44		Sc=6.28261, min distance = 2.47943
GSK3B_HUMAN I	449240 1y	KAPCA_BOVIN Fu	444345 1c	1STC	1Q3D	1YDR	31.63		Sc=5.81592, min distance = 2.62305
GSK3B_HUMAN I	4565 1h1r	CDK2_HUMAN Fu	444345 1c	1AQ1	1Q3D	1H1R	35.44		Sc=5.8443, min distance = 2.710482
GSK3B_HUMAN I	51 2-Oxop	SERA_ECOLI Fu	311 citri	2P9E	1R0E	1YBA		0.76	Sc=5.76959, min distance = 0.50919
GSK3B_HUMAN I	5288016 6	CDK2_HUMAN Fu	444345 1c	1AQ1	1Q3D	2B53	35.44		Sc=5.69888, min distance = 2.95406

GSK3B_HUMAN I	5288711 1	CDK2_HUMAN Fu	444345 1c	1AQ1	1Q3D	1KE8	35.44		Sc=6.14228, min distance = 2.660776
GSK3B_HUMAN I	5326843 A	CDK5_HUMAN Fu	5326739 i	1UNH	1Q41	1UNG	36.09		Sc=6.06378, min distance = 2.779334
GSK3B_HUMAN I	5327087 3	CDK2_HUMAN Fu	6022 Aden	1GY3	1J1C	2BPM	35.44		Sc=6.42195, min distance = 2.186544
GSK3B_HUMAN I	5327096 2	CDK2_HUMAN Fu	6022 Aden	1GY3	1J1C	2BTR	35.44		Sc=6.11125, min distance = 2.336180
GSK3B_HUMAN I	5327122 C	CHK1_HUMAN Fu	444345 1c	1NVR	1Q3D	2C3K	30.65		Sc=6.44892, min distance = 2.291213
GSK3B_HUMAN I	5327130 C	CDK2_HUMAN Fu	6022 Aden	1GY3	1J1C	2C68	35.44		Sc=6.13326, min distance = 2.591417
GSK3B_HUMAN I	5327131 T	CDK2_HUMAN Fu	6022 Aden	1GY3	1J1C	2C69	35.44		Sc=6.11934, min distance = 2.384451
GSK3B_HUMAN I	5327148 C	IPKA_RABIT Fu	33113 gam	1CDK	1J1B	2ERZ			Sc=6.12677, min distance = 1.988966
GSK3B_HUMAN I	5957 Aden	MALK_ECOLI Fu	6022 Aden	2AWN	1J1C	1Q12		0.99	Sc=5.62864, min distance = 1.507926
GSK3B_HUMAN I	5957 Aden	Q72H90_THET2 I	6022 Aden	2BEJ	1J1C	2BEK		0.99	Sc=5.67253, min distance = 2.098027
GSK3B_HUMAN I	5957 Aden	RK_BOVIN Full	6022 Aden	3C4Z	1J1C	3C4W		0.99	Sc=6.36414, min distance = 2.267462
GSK3B_HUMAN I	5962 L-ly	RBCMT_PEA Full	23831 HEP	1MLV	1H8F	2H2E			Sc=5.61263, min distance = 0.969536
GSK3B_HUMAN I	6083 aden	PURP_METJA Fu	6022 Aden	2R7N	1J1C	2R7M		0.99	Sc=5.90824, min distance = 2.656399
GSK3B_HUMAN I	60961 ade	IPKA_RABIT Fu	6022 Aden	1JBP	1J1C	1FMO		0.95	Sc=6.10946, min distance = 2.418842
GSK3B_HUMAN I	60961 ade	SKY1_YEAST Fu	33113 gam	1Q99	1J1B	1Q97	31.32	0.93	Sc=6.11902, min distance = 2.327055
GSK3B_HUMAN I	60961 ade	SKY1_YEAST Fu	6022 Aden	1Q8Y	1J1C	1Q97	31.32	0.95	Sc=6.09968, min distance = 2.765726
GSK3B_HUMAN I	6420139 C	CDK2_HUMAN Fu	444345 1c	1AQ1	1Q3D	2C5V	35.44		Sc=5.98509, min distance = 2.815229
GSK3B_HUMAN I	6420139 C	CDK2_HUMAN Fu	6022 Aden	1GY3	1J1C	2C5V	35.44		Sc=6.30541, min distance = 1.161260
GSK3B_HUMAN I	657072 RY	CDK2_HUMAN Fu	6022 Aden	1GY3	1J1C	2BHH	35.44		Sc=6.43748, min distance = 2.152520
GSK3B_HUMAN I	6804 guar	NDK_PYRHO Full	6022 Aden	2DYA	1J1C	2DXF		0.8	Sc=5.84279, min distance = 2.070734
GSK3B_HUMAN I	6914614 p	IPKA_RABIT Fu	6022 Aden	1JBP	1J1C	2F7Z			Sc=6.37778, min distance = 2.664278
GSK3B_HUMAN I	6918852 2	CDK2_HUMAN Fu	444345 1c	1AQ1	1Q3D	2FVD	35.44		Sc=6.21168, min distance = 2.256215
GSK3B_HUMAN I	8778 Laur	CTXA3_NAJAT Fu	311 citri	1XT3	1R0E	1H0J			Sc=5.71307, min distance = 1.297820
GSK3B_HUMAN I	8977 1dar	NDK_THET8 Full	6022 Aden	1WKL	1J1C	1WKK		0.8	Sc=5.93592, min distance = 2.145512
GSK3B_HUMAN I	8977 1dar	PARM_ECOLX Fu	6022 Aden	1MWM	1J1C	2ZGY		0.8	Sc=5.62153, min distance = 2.144719
GSK3B_HUMAN I	91532 AMP	NIFH1_AZOVI Fu	6022 Aden	1FP6	1J1C	2AFK		0.99	Sc=6.44506, min distance = 1.642187
GSK3B_HUMAN I	91532 AMP	PURT_ECOLI Fu	33113 gam	1EYZ	1J1B	1KJI		0.99	Sc=5.96474, min distance = 2.260977
GSK3B_HUMAN I	9547890 I	CDK2_HUMAN Fu	444345 1c	1AQ1	1Q3D	1W8C	35.44		Sc=5.88381, min distance = 3.372310
GSK3B_HUMAN I	9817550 V	CDK2_HUMAN Fu	444345 1c	1AQ1	1Q3D	3BHV	35.44		Sc=6.31421, min distance = 2.601326
GSK3B_HUMAN I	9991833 S	CDK2_HUMAN Fu	6022 Aden	1GY3	1J1C	2R3H	35.44		Sc=6.11262, min distance = 1.869238
GSPE_VIBCH Fu	16750062	CSK2A_MAIZE Fu	33113 gam	1LP4	1P9W	2OXD			Sc=5.67353, min distance = 2.424215
GSPE_VIBCH Fu	456214 pu	SRC_HUMAN Full	33113 gam	2SRC	1P9W	1YOM			Sc=6.47354, min distance = 2.262540
GSP_ECOLI Fu	188966 dA	HSLU_ECOLI Fu	33113 gam	1E94	2IO7	1G4A		0.97	Sc=6.45297, min distance = 1.982379
GSP_ECOLI Fu	36735 Gpp	CSK2A_MAIZE Fu	33113 gam	1LP4	2IO7	1DAY		0.8	Sc=6.54685, min distance = 2.178051
GSP_ECOLI Fu	444564 AD	BIOD_ECOLI Fu	6022 Aden	1DAD	2IO8	1BS1		0.91	Sc=6.48196, min distance = 2.280344
GSP_ECOLI Fu	444842 CI	CDK2_HUMAN Fu	6022 Aden	1GY3	2IO8	1CKP			Sc=5.64864, min distance = 2.124308
GSP_ECOLI Fu	447654 CI	CDK2_HUMAN Fu	6022 Aden	1GY3	2IO8	1OIT			Sc=6.40529, min distance = 2.018979

GSP_ECOLI Fu	447916 ac	RIO1_ARCFU Fu	6022 Aden	1ZTH	2IO8	1ZTF		0.95	Sc=6.30512, min distance = 2.30140
GSP_ECOLI Fu	447955 1p	CDK2_HUMAN Fu	6022 Aden	1GY3	2IO8	1PXI			Sc=5.83423, min distance = 2.00918
GSP_ECOLI Fu	448043 2g	ROCK1_HUMAN Fu	33113 gam	2V55	2IO7	3D9V			Sc=6.07344, min distance = 2.01532
GSP_ECOLI Fu	5327148 C	IPKA_RABIT Fu	33113 gam	1CDK	2IO7	2ERZ			Sc=6.25379, min distance = 2.46251
GSP_ECOLI Fu	5327148 C	IPKA_RABIT Fu	6022 Aden	1JBP	2IO8	2ERZ			Sc=6.23397, min distance = 2.51987
GSP_ECOLI Fu	5957 Aden	DDL_THET8 Ful	6022 Aden	2ZDH	2IO8	2ZDQ		0.99	Sc=6.53177, min distance = 2.17557
GSP_ECOLI Fu	6083 aden	PURP_METJA Fu	6022 Aden	2R7N	2IO8	2R7M		0.99	Sc=6.41863, min distance = 2.59459
GSP_ECOLI Fu	60961 ade	PIM1_HUMAN Fu	33113 gam	1XR1	2IO7	1YI4		0.93	Sc=6.3523, min distance = 1.979028
GSP_ECOLI Fu	60961 ade	SKY1_YEAST Fu	33113 gam	1Q99	2IO7	1Q97		0.93	Sc=6.34569, min distance = 2.183988
GSP_ECOLI Fu	6852187 2	O57883_PYRHO	6022 Aden	1WNL	2IO8	2DTH		0.89	Sc=6.18799, min distance = 1.380968
GSP_ECOLI Fu	91532 AMP	PURT_ECOLI Fu	33113 gam	1EYZ	2IO7	1KJI		0.99	Sc=6.55722, min distance = 1.92444
GSP_ECOLI Fu	9547890 I	CDK2_HUMAN Fu	6022 Aden	1GY3	2IO8	1W8C			Sc=6.37599, min distance = 1.92525
GST1D_ANOGA	124886 gl	GSTA3_CHICK Fu	97536 Hex	1VF2	1PN9	1VF1		0.9	Sc=5.82322, min distance = 2.12731
GST1D_ANOGA	445707 s-	GSTA1_HUMAN Fu	97536 Hex	1K3Y	1PN9	1GUH			Sc=6.0748, min distance = 2.197910
GST1D_ANOGA	449366 3g	GSTP1_HUMAN Fu	97536 Hex	1PGT	1PN9	2PGT			Sc=6.18961, min distance = 2.18157
GST26_SCHJA	115260 S-	GSTM2_HUMAN Fu	124886 gl	1XW5	1DUG	2AB6	44.81	0.94	Sc=5.85654, min distance = 2.24085
GST26_SCHJA	444758 CI	GSTM2_CHICK Fu	97536 Hex	1GSU	1M9A	1C72	43.9		Sc=5.74494, min distance = 1.60400
GST26_SCHJA	449471 GG	GSTT2_HUMAN Fu	124886 gl	1LJR	1DUG	3LJR			Sc=6.15115, min distance = 2.213478
GST26_SCHJA	5288475 C	GSTM2_HUMAN Fu	124886 gl	1XW5	1DUG	2C4J	44.81		Sc=5.75299, min distance = 2.42175
GST27_FASHE	443115 S-	GST26_SCHJA Fu	445616 gl	1GNE	1FHE	1U88		0.87	Sc=5.69112, min distance = 2.45749
GST28_SCHBO	124886 gl	GSTA3_CHICK Fu	97536 Hex	1VF2	2C80	1VF1	25	0.9	Sc=6.34357, min distance = 2.028898
GST28_SCHBO	445707 s-	GSTA1_HUMAN Fu	97536 Hex	1K3Y	2C80	1GUH	29.56		Sc=6.56945, min distance = 2.01797
GST28_SCHBO	6128 andr	GSTA2_HUMAN Fu	97536 Hex	1AGS	2C80	2VCT			Sc=5.98987, min distance = 2.38138
GST28_SCHHA	115260 S-	GSTM2_HUMAN Fu	124886 gl	1XW5	1OE8	2AB6		0.94	Sc=6.36853, min distance = 1.93596
GST28_SCHHA	444104 II	GSTP1_MOUSE Fu	124886 gl	1GSY	1OE8	1GLP		0.9	Sc=5.92437, min distance = 2.13418
GST28_SCHHA	449471 GG	GSTT2_HUMAN Fu	124886 gl	1LJR	1OE8	3LJR			Sc=6.20368, min distance = 1.98678
GST28_SCHHA	5288475 C	GSTM2_HUMAN Fu	124886 gl	1XW5	1OE8	2C4J			Sc=6.13873, min distance = 1.73562
GST28_SCHHA	97535 CCR	GSTA3_CHICK Fu	124886 gl	1VF1	1OE8	1VF3			Sc=6.16263, min distance = 2.19858
GST28_SCHHA	97536 Hex	GSTP1_MOUSE Fu	124886 gl	1GSY	1OE8	2GLR		0.9	Sc=6.08447, min distance = 1.97621
GST28_SCHHA	97536 Hex	GSTP_ONCVO Fu	124886 gl	1TU7	1OE8	1TU8		0.9	Sc=6.07016, min distance = 2.38656
GST28_SCHHA	97536 Hex	URE2_YEAST Fu	124886 gl	1K0D	1OE8	1K0A		0.9	Sc=6.47814, min distance = 2.50320
GST5_CAEEL Fu	24764436	PTGD2_HUMAN Fu	124886 gl	2CVD	1ZL9	2VCQ			Sc=6.05532, min distance = 1.46249
GST5_CAEEL Fu	444104 II	GSTP1_MOUSE Fu	124886 gl	1GSY	1ZL9	1GLP		0.9	Sc=5.85636, min distance = 2.03581
GST5_CAEEL Fu	5288478 C	GSTM1_HUMAN Fu	124886 gl	1XW6	1ZL9	2F3M			Sc=6.13594, min distance = 1.49700
GST5_CAEEL Fu	97536 Hex	GSTP1_MOUSE Fu	124886 gl	1GSY	1ZL9	2GLR		0.9	Sc=6.01764, min distance = 1.992498
GST5_CAEEL Fu	97538 s-(	GSTP1_MOUSE Fu	124886 gl	1GSY	1ZL9	1GLQ			Sc=5.77835, min distance = 2.09303
GSTA1_HUMAN	444104 II	GST26_SCHJA Fu	97536 Hex	1M9A	1K3Y	1M99	30.93	0.84	Sc=6.34967, min distance = 2.43307

GSTA1_HUMAN I	445616 gl	GST26_SCHJA Fu	97536 Hex	1M9A	1K3Y	1GNE	30.93	0.9	Sc=6.30011, min distance = 2.17299
GSTA1_HUMAN I	447108 CI	GST26_SCHJA Fu	97536 Hex	1M9A	1K3Y	1M9B	30.93		Sc=6.5325, min distance = 2.2078518
GSTA1_HUMAN I	6128 andr	GSTA2_HUMAN Fu	97536 Hex	1AGS	1K3Y	2VCT			Sc=5.93296, min distance = 2.009956
GSTA1_MOUSE I	115260 S-	GSTM2_HUMAN Fu	124886 gl	1XW5	1F3A	2AB6	30.92	0.94	Sc=6.36108, min distance = 1.878177
GSTA1_MOUSE I	24764436	PTGD2_HUMAN Fu	124886 gl	2CVD	1F3A	2VCQ	26.63		Sc=5.99255, min distance = 1.767612
GSTA1_MOUSE I	2763788 E	PTGD2_HUMAN Fu	124886 gl	2CVD	1F3A	2VCX	26.63		Sc=6.00081, min distance = 1.997939
GSTA1_MOUSE I	444104 II	GST26_SCHJA Fu	124886 gl	1DUG	1F3A	1M99	30.19	0.9	Sc=5.87966, min distance = 2.345585
GSTA1_MOUSE I	444104 II	GSTP1_MOUSE Fu	124886 gl	1GSY	1F3A	1GLP	29.95	0.9	Sc=6.28441, min distance = 2.881657
GSTA1_MOUSE I	449471 GC	GSTT2_HUMAN Fu	124886 gl	1LJR	1F3A	3LJR			Sc=6.14754, min distance = 2.203082
GSTA1_MOUSE I	5288475 C	GSTM2_HUMAN Fu	124886 gl	1XW5	1F3A	2C4J	30.92		Sc=6.09, min distance = 2.525701882
GSTA1_MOUSE I	97536 Hex	GSTP1_HUMAN Fu	445495 CI	3PGT	1F3B	1PGT	31.41		Sc=6.04819, min distance = 1.959765
GSTA1_MOUSE I	97536 Hex	GSTP1_MOUSE Fu	124886 gl	1GSY	1F3A	2GLR	29.95	0.9	Sc=6.46768, min distance = 2.443530
GSTA1_MOUSE I	97538 s-(	GSTP1_MOUSE Fu	124886 gl	1GSY	1F3A	1GLQ	29.95		Sc=6.06512, min distance = 2.652631
GSTA1_RAT Fu	97538 s-(	GSTP1_MOUSE Fu	444104 II	1GLP	1EV4	1GLQ			Sc=6.62399, min distance = 1.546011
GSTA2_MOUSE I	97536 Hex	GSTP1_HUMAN Fu	445495 CI	3PGT	1ML6	1PGT			Sc=6.47727, min distance = 2.087688
GSTA3_CHICK I	443115 S-	GST26_SCHJA Fu	124886 gl	1DUG	1VF1	1U88	31.5	0.87	Sc=6.05672, min distance = 2.020184
GSTA3_CHICK I	444104 II	GST26_SCHJA Fu	124886 gl	1DUG	1VF1	1M99	31.5	0.9	Sc=6.35296, min distance = 0.506202
GSTA3_CHICK I	444104 II	GST26_SCHJA Fu	97536 Hex	1M9A	1VF2	1M99	31.5	0.84	Sc=6.32743, min distance = 1.910252
GSTA3_CHICK I	444104 II	GSTP1_MOUSE Fu	124886 gl	1GSY	1VF1	1GLP	32.2	0.9	Sc=5.84034, min distance = 2.843614
GSTA3_CHICK I	5288475 C	GSTM2_HUMAN Fu	124886 gl	1XW5	1VF1	2C4J	25.6		Sc=6.12968, min distance = 1.196991
GSTA3_HUMAN I	115260 S-	GSTM2_HUMAN Fu	124886 gl	1XW5	1TDI	2AB6		0.94	Sc=6.30717, min distance = 2.507044
GSTA3_HUMAN I	12389 Tet	LTC4S_HUMAN Fu	124886 gl	2PNO	1TDI	2UUI			Sc=6.09142, min distance = 0.812507
GSTA3_HUMAN I	24139 ace	LYSC_HUMAN Fu	124886 gl	1HNL	1TDI	1LZR			Sc=5.95797, min distance = 2.246201
GSTA3_HUMAN I	444104 II	GST26_SCHJA Fu	124886 gl	1DUG	1TDI	1M99		0.9	Sc=6.42203, min distance = 0.495312
GSTA3_HUMAN I	444104 II	GSTP1_MOUSE Fu	124886 gl	1GSY	1TDI	1GLP		0.9	Sc=6.44525, min distance = 0.936465
GSTA3_HUMAN I	5288475 C	GSTM2_HUMAN Fu	124886 gl	1XW5	1TDI	2C4J			Sc=6.07179, min distance = 1.892539
GSTA3_HUMAN I	5757 estr	DHB1_HUMAN Fu	6128 andr	1QYX	2VCV	1FDS			Sc=5.89231, min distance = 2.290715
GSTA3_HUMAN I	97538 s-(	URE2_YEAST Fu	124886 gl	1K0D	1TDI	1K0C			Sc=6.01147, min distance = 2.615711
GSTA4_MOUSE I	2737071 Z	PTGD2_HUMAN Fu	124886 gl	2CVD	1B48	2VCZ			Sc=6.04399, min distance = 2.166784
GSTA4_MOUSE I	444104 II	GSTP1_MOUSE Fu	124886 gl	1GSY	1B48	1GLP		0.9	Sc=5.82976, min distance = 1.948075
GSTA4_MOUSE I	5288475 C	GSTM2_HUMAN Fu	124886 gl	1XW5	1B48	2C4J			Sc=6.16733, min distance = 1.384026
GSTA_BPT4 Fu	6022 Ader	O33839_THEMA I	6031 Urid	1XJG	1XV5	1XJK			Sc=6.47291, min distance = 1.945536
GSTA_BPT4 Fu	6030 Uric	GLMU_HAEIN Fu	6031 Urid	2V0K	1XV5	2V0J		0.99	Sc=6.28244, min distance = 2.027202
GSTA_BPT4 Fu	6030 Uric	PYRH_ECOLI Fu	6031 Urid	2BND	1XV5	2BNE		0.99	Sc=6.30651, min distance = 1.825622
GSTB_BPT4 Fu	6030 Uric	O87988_BORBR I	6031 Urid	2PZM	1JG7	2PZL		0.99	Sc=6.17743, min distance = 2.237419
GSTB_BPT4 Fu	6030 Uric	PYRH_ECOLI Fu	6031 Urid	2BND	1JG7	2BNE		0.99	Sc=6.20731, min distance = 2.268793
GSTB_BPT4 Fu	6131 cyti	KCY_DICDI Ful	6031 Urid	4UKD	1JG7	1QF9		0.82	Sc=6.21298, min distance = 2.199847

GSTB_BPT4 Fu	6132 Cyti	ECX1_PYRAB Fu	6031 Urid	2PNZ	1JG7	2PO2		0.83	Sc=6.27083, min distance = 2.28975
GSTK1_RAT Fu	115260 S-	GSTM2_HUMAN F	124886 gl	1XW5	1R4W	2AB6		0.94	Sc=5.89923, min distance = 2.16340
GSTK1_RAT Fu	444104 II	GSTP1_MOUSE F	124886 gl	1GSY	1R4W	1GLP		0.9	Sc=6.37947, min distance = 0.86859
GSTK1_RAT Fu	97536 Hex	GSTP1_MOUSE F	124886 gl	1GSY	1R4W	2GLR		0.9	Sc=6.52783, min distance = 0.98075
GSTK1_RAT Fu	97536 Hex	URE2_YEAST Fu	124886 gl	1K0D	1R4W	1K0A		0.9	Sc=6.51381, min distance = 2.06129
GSTM1_HUMAN	12389 Tet	LTC4S_HUMAN F	124886 gl	2PNO	1XW6	2UUI			Sc=5.72539, min distance = 1.50686
GSTM1_HUMAN	443115 S-	GST26_SCHJA F	124886 gl	1DUG	1XW6	1U88	42.93	0.87	Sc=6.44696, min distance = 2.02473
GSTM1_HUMAN	444104 II	GST26_SCHJA F	124886 gl	1DUG	1XW6	1M99	42.93	0.9	Sc=5.8653, min distance = 2.202782
GSTM1_HUMAN	447108 CI	GST26_SCHJA F	124886 gl	1DUG	1XW6	1M9B	42.93		Sc=6.06841, min distance = 2.64210
GSTM1_HUMAN	97536 Hex	GSTP_ONCVO Fu	124886 gl	1TU7	1XW6	1TU8		0.9	Sc=6.49111, min distance = 1.86295
GSTM1_RAT Fu	115260 S-	GSTM2_HUMAN F	124886 gl	1XW5	6GST	2AB6	76.96	0.94	Sc=6.40224, min distance = 1.91930
GSTM1_RAT Fu	443115 S-	GST26_SCHJA F	124886 gl	1DUG	6GST	1U88	44.88	0.87	Sc=6.47739, min distance = 2.25069
GSTM1_RAT Fu	444104 II	GST26_SCHJA F	124886 gl	1DUG	6GST	1M99	44.88	0.9	Sc=5.88645, min distance = 2.21483
GSTM1_RAT Fu	444104 II	GSTP1_MOUSE F	124886 gl	1GSY	6GST	1GLP	35	0.9	Sc=6.33093, min distance = 2.20652
GSTM1_RAT Fu	5288475 C	GSTM2_HUMAN F	124886 gl	1XW5	6GST	2C4J	76.96		Sc=6.26121, min distance = 1.75228
GSTM1_RAT Fu	5288478 C	GSTM1_HUMAN F	124886 gl	1XW6	6GST	2F3M	79.72		Sc=6.15114, min distance = 1.74698
GSTM1_RAT Fu	97536 Hex	GSTA3_CHICK F	124886 gl	1VF1	6GST	1VF2		0.9	Sc=5.99849, min distance = 2.17959
GSTM1_RAT Fu	97536 Hex	GSTA3_CHICK F	97535 CCR	1VF3	5GST	1VF2			Sc=6.06042, min distance = 2.12368
GSTM1_RAT Fu	97536 Hex	GSTP1_MOUSE F	124886 gl	1GSY	6GST	2GLR	35	0.9	Sc=6.50752, min distance = 1.87515
GSTM1_RAT Fu	97536 Hex	GSTP_ONCVO Fu	124886 gl	1TU7	6GST	1TU8		0.9	Sc=6.51157, min distance = 1.74735
GSTM2_CHICK	445616 gl	GST28_SCHBO F	97536 Hex	2C80	1GSU	2CAQ	30.18	0.9	Sc=5.81507, min distance = 2.45424
GSTM2_CHICK	6128 andr	GSTA2_HUMAN F	97536 Hex	1AGS	1GSU	2VCT			Sc=6.07501, min distance = 1.34636
GSTM2_HUMAN	12389 Tet	LTC4S_HUMAN F	124886 gl	2PNO	1XW5	2UUI			Sc=5.74711, min distance = 0.19560
GSTM2_HUMAN	443115 S-	GST26_SCHJA F	124886 gl	1DUG	1XW5	1U88	44.81	0.87	Sc=6.16262, min distance = 2.34557
GSTM2_HUMAN	444104 II	GST26_SCHJA F	124886 gl	1DUG	1XW5	1M99	44.81	0.9	Sc=5.89335, min distance = 2.33787
GSTM2_HUMAN	444104 II	GSTP1_MOUSE F	124886 gl	1GSY	1XW5	1GLP	32.7	0.9	Sc=6.28044, min distance = 2.16635
GSTM2_HUMAN	447108 CI	GST26_SCHJA F	124886 gl	1DUG	1XW5	1M9B	44.81		Sc=6.06943, min distance = 2.48496
GSTM2_HUMAN	97535 CCR	GSTA3_CHICK F	124886 gl	1VF1	1XW5	1VF3	25.6		Sc=5.8161, min distance = 2.105905
GSTM2_HUMAN	97536 Hex	GST26_SCHJA F	124886 gl	1DUG	1XW5	1M9A	44.81	0.9	Sc=6.07506, min distance = 2.45168
GSTM2_HUMAN	97536 Hex	GSTP_ONCVO Fu	124886 gl	1TU7	1XW5	1TU8		0.9	Sc=6.52265, min distance = 1.46226
GSTM2_RAT Fu	124886 gl	GSTM1_RAT Ful	444461 2g	2GST	1B4P	6GST			Sc=5.82714, min distance = 2.45452
GSTO1_HUMAN	97536 Hex	GSTP_ONCVO Fu	124886 gl	1TU7	1EEM	1TU8		0.9	Sc=6.0866, min distance = 1.949633
GSTP1_HUMAN	125409 be	XYNA_BACSU Fu	23831 HEP	2B45	1PGT	2QZ3			Sc=5.6567, min distance = 0.392364
GSTP1_HUMAN	5962 L-ly	RBCMT_PEA Ful	23831 HEP	1MLV	1PGT	2H2E			Sc=5.70923, min distance = 1.08000
GSTP1_HUMAN	82313 1rc	LYSC_CHICK Fu	23831 HEP	2HUB	1PGT	1LSZ			Sc=5.99738, min distance = 2.32014
GSTP1_HUMAN	97538 s-(	URE2_YEAST Fu	97536 Hex	1K0A	1PGT	1K0C			Sc=6.17459, min distance = 1.32487
GSTP1_MOUSE	115260 S-	GSTM2_HUMAN F	124886 gl	1XW5	1GSY	2AB6	32.7	0.94	Sc=6.33183, min distance = 2.39650

GSTP1_MOUSE I	444352 GT	GSTM1_RAT Ful	124886 gl	6GST	1GSY	4GST	35		Sc=5.89382, min distance = 2.245562
GSTP1_MOUSE I	449471 GG	GSTT2_HUMAN Fu	124886 gl	1LJR	1GSY	3LJR			Sc=6.29815, min distance = 2.266688
GSTP1_MOUSE I	5288475 C	GSTM2_HUMAN Fu	124886 gl	1XW5	1GSY	2C4J	32.7		Sc=6.12065, min distance = 2.246219
GSTP1_MOUSE I	97535 CCR	GSTM1_RAT Ful	124886 gl	6GST	1GSY	5GST	35		Sc=6.11138, min distance = 2.029971
GSTP_ONCVO Fu	115260 S-	GSTM2_HUMAN Fu	124886 gl	1XW5	1TU7	2AB6		0.94	Sc=6.4546, min distance = 0.766302
GSTP_ONCVO Fu	444104 II	GSTP1_MOUSE Fu	124886 gl	1GSY	1TU7	1GLP		0.9	Sc=5.87117, min distance = 2.401907
GSTP_ONCVO Fu	449471 GG	GSTT2_HUMAN Fu	124886 gl	1LJR	1TU7	3LJR			Sc=6.17791, min distance = 1.956376
GSTP_ONCVO Fu	5288475 C	GSTM2_HUMAN Fu	124886 gl	1XW5	1TU7	2C4J			Sc=6.20525, min distance = 0.744275
GSTP_ONCVO Fu	6128 andr	GSTA2_HUMAN Fu	97536 Hex	1AGS	1TU8	2VCT			Sc=6.023, min distance = 1.69556686
GSTP_ONCVO Fu	97538 s-(	GSTP1_MOUSE Fu	124886 gl	1GSY	1TU7	1GLQ			Sc=6.10061, min distance = 2.587672
GSTT1_HUMAN I	124886 gl	GSTP1_MOUSE Fu	97536 Hex	2GLR	2C3Q	1GSY		0.9	Sc=6.34047, min distance = 2.408147
GSTT2_HUMAN I	115260 S-	GSTM2_HUMAN Fu	124886 gl	1XW5	1LJR	2AB6		0.94	Sc=6.00265, min distance = 2.099066
GSTT2_HUMAN I	444104 II	GSTP1_MOUSE Fu	124886 gl	1GSY	1LJR	1GLP		0.9	Sc=5.93955, min distance = 2.171689
GSTT2_HUMAN I	97536 Hex	GSTP1_MOUSE Fu	124886 gl	1GSY	1LJR	2GLR		0.9	Sc=6.13958, min distance = 2.155357
GSTT2_HUMAN I	97538 s-(	GSTP1_MOUSE Fu	124886 gl	1GSY	1LJR	1GLQ			Sc=6.56273, min distance = 1.738354
GSTU1_ORYSJ I	115260 S-	GSTM2_HUMAN Fu	124886 gl	1XW5	1OYJ	2AB6		0.94	Sc=5.89115, min distance = 2.246412
GSTU1_ORYSJ I	97536 Hex	URE2_YEAST Ful	124886 gl	1K0D	1OYJ	1K0A		0.9	Sc=6.49386, min distance = 2.638231
GST_ECOLI Ful	97538 s-(	GSTP1_MOUSE Fu	444104 II	1GLP	1N2A	1GLQ			Sc=5.80031, min distance = 1.850886
GST_OMMSL Ful	97536 Hex	GSTP1_HUMAN Fu	97535 CCR	18GS	1GSQ	1PGT	34		Sc=6.46672, min distance = 1.720812
GST_PROMI Ful	12389 Tet	LTC4S_HUMAN Fu	124886 gl	2PNO	1PMT	2UUI			Sc=5.62415, min distance = 0.851392
GUAA_HUMAN Fu	100252 Ri	IMDH_TRIFO Ful	73323 Xan	1MEW	2VXO	1ME8			Sc=6.22662, min distance = 2.122505
GUAA_HUMAN Fu	8582 Inos	IMDH_TRIFO Ful	73323 Xan	1MEW	2VXO	1MEH		0.94	Sc=5.90113, min distance = 1.872274
GUAA_THET8 Fu	11963491	PUR9_CHICK Ful	73323 Xan	1M9N	2YWC	2B1G		0.9	Sc=6.01748, min distance = 2.653762
GUAD_HUMAN Fu	764 guani	XAPA_ECOLI Ful	1188 xant	1YR3	2UZ9	1YQQ		0.92	Sc=5.85952, min distance = 2.591195
GUAD_HUMAN Fu	764 guani	XGPT_ECOLI Ful	1188 xant	1A96	2UZ9	1A95		0.92	Sc=5.87031, min distance = 2.515629
GUB_PAEMA Ful	16741014	CARP_YEAST Ful	64689 bet	1FQ4	1U0A	1FQ7			Sc=5.62522, min distance = 2.611485
GUB_PAEMA Ful	445999 CI	GUX6_HUMIN Ful	64689 bet	1OCN	1U0A	1OC7		0.77	Sc=5.74024, min distance = 0.231991
GUB_PAEMA Ful	447601 CI	GUX6_HUMIN Ful	64689 bet	1OCN	1U0A	1OCB			Sc=5.85478, min distance = 1.495181
GUB_PAEMA Ful	447607 1d	Q79G13_MYCTU I	64689 bet	1UP0	1U0A	1UP2			Sc=5.65587, min distance = 2.700491
GUB_PAEMA Ful	82313 1rd	UGL_BACGL Ful	64689 bet	2FV0	1U0A	2FV1			Sc=5.86711, min distance = 3.015575
GUB_PAEMA Ful	854023 Ep	Q8WSF8_APLCA I	23831 HEP	2BR7	1AJK	2BYQ			Sc=5.89068, min distance = 2.113425
GUN1_HUMIN Fu	114718 Be	Q5XKG4_MOUSE I	439353 be	1UZ8	1OJJ	1RIU			Sc=6.14208, min distance = 1.669866
GUN1_HUMIN Fu	29435 1-D	CDGT_BACS0 Ful	64689 bet	1UKS	1OJJ	1I75			Sc=5.88237, min distance = 0.691861
GUN1_HUMIN Fu	439692 D-	XYLA_STRRU Ful	79025 alp	1XIF	1OJJ	4XIS			Sc=5.60269, min distance = 2.410407
GUN1_HUMIN Fu	444635 O3	MBL1_RAT Full	439353 be	2KMB	1OJJ	3KMB		0.9	Sc=5.68884, min distance = 1.588542
GUN1_HUMIN Fu	446578 1r	Q8XXK6_RALSO I	439353 be	2BS6	1OJJ	2BT9		0.94	Sc=5.8588, min distance = 1.3358794
GUN1_HUMIN Fu	6027 xylo	Q9ZB17_9LACT I	79025 alp	1NSZ	1OJJ	1MN0		0.93	Sc=5.63902, min distance = 2.143394

GUN1_HUMIN F	656981 CU	LYSC_CHICK Fu	439353 be	1UC0	1OJJ	1YIL			Sc=5.6124, min distance = 2.490014
GUN1_HUMIN F	7048614 Z	XYLA_STRRU Fu	79025 alp	1XIF	1OJJ	1XID			Sc=5.85754, min distance = 2.339207
GUN1_HUMIN F	892 myo-i	SFTPD_HUMAN F	79025 alp	1PWB	1OJJ	2OS9			Sc=5.85224, min distance = 2.063325
GUN4_THEFU F	23831 HEE	GUB_PAEMA Ful	64689 bet	1U0A	1JS4	1AJK			Sc=5.64237, min distance = 0.781407
GUN4_THEFU F	445564 4-	Q79G13_MYCTU	64689 bet	1UP0	1JS4	1UP3		0.88	Sc=5.61411, min distance = 1.244439
GUN4_THEFU F	445999 CI	Q79G13_MYCTU	64689 bet	1UP0	1JS4	1UOZ		0.77	Sc=5.60314, min distance = 1.262567
GUN4_THEFU F	5326754 S	PHSM_ECOLI Fu	64689 bet	2ASV	1JS4	1QM5		0.88	Sc=5.69497, min distance = 1.779283
GUN5_BACAG F	11957431	BGLA_THEMA Fu	447607 1c	1OIF	1OCQ	2J7D			Sc=6.23813, min distance = 2.348048
GUN5_BACAG F	11957434	BGLA_THEMA Fu	447607 1c	1OIF	1OCQ	2J7G			Sc=6.06735, min distance = 2.430964
GUN5_BACAG F	11957435	BGLA_THEMA Fu	447607 1c	1OIF	1OCQ	2J7H			Sc=5.68936, min distance = 2.567842
GUN5_BACAG F	124434 Ca	BGLA_THEMA Fu	447607 1c	1OIF	1OCQ	2CBV			Sc=5.88758, min distance = 2.665197
GUN5_BACAG F	444393 CI	PYGM_RABIT Fu	447607 1c	2G9V	1OCQ	1AXR			Sc=5.98617, min distance = 2.038330
GUN5_BACAG F	447651 CI	BGLA_THEMA Fu	447607 1c	1OIF	1OCQ	2J77			Sc=5.81359, min distance = 2.534342
GUN5_BACAG F	448963 2j	BGLA_THEMA Fu	447607 1c	1OIF	1OCQ	2J79			Sc=5.98185, min distance = 2.215712
GUN5_BACAG F	448980 nc	BGLA_THEMA Fu	447607 1c	1OIF	1OCQ	1UZ1		0.79	Sc=5.82736, min distance = 2.678181
GUN5_BACAG F	9549193 C	BGLA_THEMA Fu	447607 1c	1OIF	1OCQ	2CES			Sc=6.02115, min distance = 2.140086
GUN5_THEFU F	447607 1c	GUN5_BACAG Fu	64689 bet	1W3L	2CKR	1OCQ			Sc=5.72788, min distance = 2.065517
GUN5_THEFU F	5326754 S	PHSM_ECOLI Fu	64689 bet	2ASV	2CKR	1QM5		0.88	Sc=5.61411, min distance = 0.657721
GUNC_CELFI F	446578 1r	Q8XXK6_RALSO	64689 bet	2BS5	1GU3	2BT9		0.94	Sc=5.62687, min distance = 1.320925
GUNC_CELFI F	447607 1c	Q79G13_MYCTU	64689 bet	1UP0	1GU3	1UP2			Sc=5.69101, min distance = 2.346473
GUNC_CELFI F	8190 Ethy	CLC7A_MOUSE F	64689 bet	2CL8	1GU3	2BPE			Sc=5.6983, min distance = 0.9325781
GUNC_CLOTH F	445999 CI	GUN5_BACAG Fu	64689 bet	1W3L	1CEN	1H5V		0.77	Sc=5.60314, min distance = 2.518181
GUNC_FUSOX F	445999 CI	GUN5_BACAG Fu	10712 cel	2A3H	2OVW	1H5V			Sc=5.6718, min distance = 2.1995331
GUNF_CLOCE F	16741014	CARP_YEAST Fu	64689 bet	1FQ4	1FAE	1FQ7			Sc=5.67966, min distance = 2.337238
GUNF_CLOCE F	29435 1-D	CDGT_BACS0 Fu	64689 bet	1UKS	1FAE	1I75			Sc=5.84332, min distance = 1.047272
GUNF_CLOCE F	446000 CI	GUX6_HUMIN Fu	445238 O1	1OC5	1FCE	1OC7		0.89	Sc=5.70177, min distance = 2.380892
GUNF_CLOCE F	5327083 C	GUN2_THEFU Fu	64689 bet	2BOF	1FAE	2BOD			Sc=5.7479, min distance = 2.4504728
GUNF_CLOCE F	54445 cas	Q2PS28_9PSED	64689 bet	2PWF	1FAE	2PWG			Sc=5.81974, min distance = 2.018452
GUNF_CLOCE F	82313 1rc	Q9HYN5_PSEAE	64689 bet	1W8F	1FAE	1W8H			Sc=5.9442, min distance = 2.0201351
GUNG_CLOCE F	125409 be	XYNA1_CLOSR F	64689 bet	1OD3	1GA2	1UY4		0.93	Sc=5.66363, min distance = 0.983522
GUNG_CLOCE F	16741014	CARP_YEAST Fu	64689 bet	1FQ4	1GA2	1FQ7			Sc=5.66077, min distance = 2.662160
GUNG_CLOCE F	23831 HEE	GUB_PAEMA Ful	64689 bet	1U0A	1GA2	1AJK			Sc=5.63141, min distance = 0.979686
GUNG_CLOCE F	445238 O1	GUNF_CLOCE Fu	445564 4-	1G9J	1KFG	1FCE		0.83	Sc=5.63562, min distance = 3.028910
GUNG_CLOCE F	445999 CI	GUX6_HUMIN Fu	64689 bet	1OCN	1GA2	1OC7		0.77	Sc=5.68067, min distance = 1.273402
GUNG_CLOCE F	447607 1c	PYGM_RABIT Fu	79025 alp	1H5U	1K72	2G9V			Sc=5.60208, min distance = 2.778824
GUNG_CLOCE F	5326754 S	PHSM_ECOLI Fu	64689 bet	2ASV	1GA2	1QM5		0.88	Sc=5.61221, min distance = 1.617123
GUNG_CLOCE F	657081 CI	LEG3_HUMAN Fu	64689 bet	2NMO	1GA2	1KJR			Sc=5.88046, min distance = 0.392968

GUNG_CLOCE Fu	7048614 Z	XYLA_STRRU Fu	79025 alp	1XIF	1K72	1XID			Sc=5.69803, min distance = 2.64394
GUNH_CLOTH Fu	445564 4-	GUNG_CLOCE Fu	64689 bet	1GA2	2V3G	1KFG		0.88	Sc=5.65049, min distance = 2.33333
GUNH_CLOTH Fu	445999 CI	Q79G13_MYCTU I	64689 bet	1UP0	2V3G	1UOZ		0.77	Sc=5.67897, min distance = 0.84737
GUX1_TRIRE Fu	447607 1c	Q79G13_MYCTU I	444809 Th	1UOZ	1Q2E	1UP2			Sc=5.6277, min distance = 1.839107
GUX1_TRIRE Fu	79025 alp	AMY1_HORVU Fu	444809 Th	1P6W	1Q2E	1RP8		0.78	Sc=5.93705, min distance = 0.50690
GUX2_TRIRE Fu	10712 cel	GUN2_BACS4 Fu	444809 Th	1E5J	1QJW	1QI0			Sc=6.38493, min distance = 1.79546
GUX2_TRIRE Fu	151504 D-	CARP_YEAST Fu	64689 bet	1FQ4	1QK0	1FQ5			Sc=5.69804, min distance = 2.71691
GUX2_TRIRE Fu	16741013	CARP_YEAST Fu	64689 bet	1FQ4	1QK0	1FQ6			Sc=5.70694, min distance = 2.80734
GUX2_TRIRE Fu	23831 HEF	GUB_PAEMA Ful	64689 bet	1U0A	1QK0	1AJK			Sc=5.63141, min distance = 1.25247
GUX2_TRIRE Fu	4369530 2	O22321_MUSAC I	64689 bet	2BN0	1QK0	2BMZ		0.85	Sc=6.28939, min distance = 1.88771
GUX2_TRIRE Fu	445999 CI	GUNF_CLOCE Fu	445564 4-	1G9J	1QJW	1F9O		0.88	Sc=5.84043, min distance = 1.27733
GUX2_TRIRE Fu	445999 CI	GUX6_HUMIN Fu	445238 O1	1OC5	1QJW	1OC7	64.27		Sc=5.60314, min distance = 2.38617
GUX2_TRIRE Fu	445999 CI	Q79G13_MYCTU I	445564 4-	1UP3	1QJW	1UOZ	26.8	0.88	Sc=5.7012, min distance = 0.824379
GUX2_TRIRE Fu	446000 CI	GUX6_HUMIN Fu	445238 O1	1OC5	1QJW	1OC7	64.27	0.89	Sc=5.69771, min distance = 2.13550
GUX2_TRIRE Fu	447600 CI	GUX6_HUMIN Fu	445238 O1	1OC5	1QJW	1OCB	64.27	0.89	Sc=5.68594, min distance = 2.77033
GUX2_TRIRE Fu	447607 1c	GUN5_BACAG Fu	444809 Th	1H5V	1QJW	1OCQ			Sc=5.6304, min distance = 2.359946
GUX2_TRIRE Fu	5326754 S	PHSM_ECOLI Fu	64689 bet	2ASV	1QK0	1QM5		0.88	Sc=5.8035, min distance = 0.523116
GUX2_TRIRE Fu	5327083 C	GUN2_THEFU Fu	445564 4-	2BOG	1QJW	2BOD	28.33	0.82	Sc=5.72381, min distance = 2.51617
GUX2_TRIRE Fu	79025 alp	AMY1_HORVU Fu	444809 Th	1P6W	1QJW	1RP8		0.78	Sc=5.75873, min distance = 1.01034
GUX6_HUMIN Fu	125409 be	GUX2_TRIRE Fu	444809 Th	1QJW	1OC7	1QK0	64.54		Sc=5.60042, min distance = 0.81741
GUX6_HUMIN Fu	151504 D-	CARP_YEAST Fu	64689 bet	1FQ4	1OCN	1FQ5			Sc=5.74599, min distance = 2.77612
GUX6_HUMIN Fu	16741013	CARP_YEAST Fu	64689 bet	1FQ4	1OCN	1FQ6			Sc=5.73005, min distance = 2.72838
GUX6_HUMIN Fu	16741014	CARP_YEAST Fu	64689 bet	1FQ4	1OCN	1FQ7			Sc=5.70694, min distance = 2.23023
GUX6_HUMIN Fu	23831 HEF	GUB_PAEMA Ful	64689 bet	1U0A	1OCN	1AJK			Sc=5.65379, min distance = 0.54197
GUX6_HUMIN Fu	445237 1,	AMY1_HORVU Fu	64689 bet	1RP9	1OCN	1P6W		0.77	Sc=5.76391, min distance = 2.31471
GUX6_HUMIN Fu	447607 1c	Q79G13_MYCTU I	445999 CI	1UOZ	1OC7	1UP2	29.21		Sc=5.79727, min distance = 1.35018
GUX6_HUMIN Fu	447607 1c	Q79G13_MYCTU I	64689 bet	1UP0	1OCN	1UP2	29.21		Sc=5.72788, min distance = 0.82720
GUX6_HUMIN Fu	79025 alp	AMY1_HORVU Fu	447600 CI	1P6W	1OCB	1RP8		0.83	Sc=5.7479, min distance = 1.978140
GUX6_HUMIN Fu	82313 1rc	Q9HYN5_PSEAE I	64689 bet	1W8F	1OCN	1W8H			Sc=6.13488, min distance = 1.17255
GUX_CELFI Fu	445615 XY	Q59277_CELFI I	125409 be	1FH9	2XYL	1FHD			Sc=5.86661, min distance = 2.19497
GYAR_PYRHO Fu	15942680	6PGD_LACLM Fu	5886 NADF	2IZ0	2DBQ	2IZ1			Sc=6.21053, min distance = 2.31869
GYAR_PYRHO Fu	440334 Ni	DAPB_ECOLI Fu	5886 NADF	1DIH	2DBQ	1DRW		0.91	Sc=6.32675, min distance = 2.15266
GYRB_ECOLI Fu	16750062	CSK2A_MAIZE Fu	33113 gam	1LP4	1EI1	2OXD			Sc=5.70177, min distance = 2.56702
GYRB_ECOLI Fu	5287844 i	GSK3B_HUMAN Fu	33113 gam	1J1B	1EI1	1UV5			Sc=6.37924, min distance = 1.95466
GYRB_ECOLI Fu	5326976 1	CSK2A_MAIZE Fu	33113 gam	1LP4	1EI1	1ZOE			Sc=5.74321, min distance = 2.34734
GYRB_ECOLI Fu	5326978 1	CSK2A_MAIZE Fu	33113 gam	1LP4	1EI1	1ZOH			Sc=5.67403, min distance = 2.43050
GYRB_ECOLI Fu	5327148 C	IPKA_RABIT Fu	33113 gam	1CDK	1EI1	2ERZ			Sc=6.23052, min distance = 1.24099

H2AY_HUMAN F	23831 HEE	MMP8_HUMAN Fu	78165 MES	1ZS0	1ZR3	1BZS		0.81	Sc=5.63262, min distance = 2.15383
HA1B_MOUSE F	447053 CI	B2MG_HUMAN Fu	444863 is	2H26	1FZK	1ONQ	69.7	0.85	Sc=5.63079, min distance = 1.25532
HA22_MOUSE F	439554 fu	FIBB_HUMAN Fu	444205 CI	1FZF	1FNE	2OYH			Sc=5.69801, min distance = 1.37264
HAL2_YEAST F	5281680 Q	PIM1_HUMAN Fu	6083 aden	1YXU	1QGX	2O64			Sc=6.43497, min distance = 2.26051
HAL3A_ARATH I	5326566 I	Q9WZW1_THEMA I	444243 FA	1T6Y	1MVL	1S4M		0.82	Sc=6.0669, min distance = 2.087300
HAOX1_HUMAN I	16741044	PYRDA_LACLC F	444243 FA	1JUB	2NZL	1JRB			Sc=6.66251, min distance = 2.42492
HAOX1_HUMAN I	446995 FM	CYB2_YEAST Fu	444243 FA	1KBI	2NZL	1LTD		0.77	Sc=6.20746, min distance = 2.08605
HASA_SERMA F	11957353	HBB_HORSE Ful	444124 HE	1Y8I	1DKH	1IWH		0.87	Sc=6.76575, min distance = 2.06325
HASA_SERMA F	11957370	HMOX1_HUMAN F	444124 HE	1OZW	1DKH	1TWN			Sc=6.61785, min distance = 1.99129
HASA_SERMA F	11957371	HMOX1_HUMAN F	444124 HE	1OZW	1DKH	1TWR			Sc=6.63891, min distance = 2.14542
HASA_SERMA F	11957385	MYG_PHYCA Ful	444098 HE	1A6M	1DK0	2CMM			Sc=6.35932, min distance = 2.05938
HASA_SERMA F	11957385	MYG_PHYCA Ful	444124 HE	1U7R	1DKH	2CMM			Sc=6.32345, min distance = 2.33005
HASA_SERMA F	126994 CI	CCPR_YEAST Fu	444098 HE	2EUT	1DK0	1Z53		0.77	Sc=6.77076, min distance = 2.36964
HASA_SERMA F	16741183	CPXA_PSEPU Fu	444124 HE	1RE9	1DKH	2ZAW		0.92	Sc=6.58066, min distance = 2.00856
HASA_SERMA F	4369001 C	HBA_HUMAN Ful	444124 HE	1NQP	1DKH	4HHB		0.8	Sc=6.61486, min distance = 2.04045
HASA_SERMA F	446189 CI	HBG1_HUMAN Fu	444124 HE	1I3E	1DKH	1I3D		0.89	Sc=6.42148, min distance = 2.14056
HASA_SERMA F	446332 CI	MYG_PHYCA Ful	444124 HE	1U7R	1DKH	1IOP		0.8	Sc=6.70179, min distance = 2.32312
HASP_HUMAN F	10109823	PIM1_HUMAN Fu	6083 aden	1YXU	3DLZ	3CY3			Sc=6.52823, min distance = 1.46488
HASP_HUMAN F	16040294	SRC_CHICK Ful	6083 aden	3DQX	3DLZ	3F6X			Sc=6.60389, min distance = 1.33642
HASP_HUMAN F	24180721	PIM1_HUMAN Fu	6083 aden	1YXU	3DLZ	3C4E			Sc=5.94553, min distance = 2.71590
HASP_HUMAN F	5281680 Q	PIM1_HUMAN Fu	6083 aden	1YXU	3DLZ	2O64			Sc=6.45308, min distance = 1.08757
HASP_HUMAN F	5957 Ader	PURP_PYRFU Fu	6083 aden	2R85	3DLZ	2R86		0.98	Sc=6.0282, min distance = 1.591220
HASP_HUMAN F	611002 Op	PIM1_HUMAN Fu	6083 aden	1YXU	3DLZ	1YXX			Sc=6.09899, min distance = 2.70843
HASP_HUMAN F	65110 AIC	PURP_METJA Fu	6083 aden	2R7M	3DLZ	2R7K		0.79	Sc=6.25402, min distance = 2.39873
HASP_HUMAN F	65110 AIC	YL28_SCHPO Fu	6083 aden	2OOX	3DLZ	2QRE		0.79	Sc=6.27293, min distance = 2.08971
HASP_HUMAN F	656964 1y	PDE4D_HUMAN F	6083 aden	1TB7	3DLZ	1Y2C			Sc=5.94217, min distance = 2.20960
HASP_HUMAN F	656966 1y	PDE4D_HUMAN F	6083 aden	1TB7	3DLZ	1Y2E			Sc=6.0852, min distance = 2.140892
HASP_HUMAN F	8582 Inos	PYGM_RABIT Fu	6083 aden	8GPB	3DLZ	2QN7		0.79	Sc=6.34304, min distance = 2.01726
HBA1_ONCMY F	11957385	MYG_PHYCA Ful	444098 HE	1A6M	1OUT	2CMM			Sc=6.08257, min distance = 2.49335
HBA1_TRENE F	11957330	CCPR_YEAST Fu	444098 HE	2EUT	3D1K	1BEQ		0.81	Sc=6.5878, min distance = 2.375255
HBA1_TRENE F	11957353	HBA_HORSE Ful	444098 HE	2D5X	3D1K	1IWH	51.06	0.83	Sc=6.60442, min distance = 2.01253
HBA1_TRENE F	11957370	HMOX1_HUMAN F	444124 HE	1OZW	2AA1	1TWN			Sc=6.45686, min distance = 2.21393
HBA1_TRENE F	11957371	HMOX1_HUMAN F	444124 HE	1OZW	2AA1	1TWR			Sc=6.5496, min distance = 2.416789
HBA1_TRENE F	11957385	MYG_PHYCA Ful	444098 HE	1A6M	3D1K	2CMM	23.29		Sc=5.92694, min distance = 2.11639
HBA1_TRENE F	11957385	MYG_PHYCA Ful	444124 HE	1U7R	2AA1	2CMM	23.29		Sc=5.94514, min distance = 2.62223
HBA1_TRENE F	11970242	CCPR_YEAST Fu	444098 HE	2EUT	3D1K	1CPE		0.83	Sc=6.5889, min distance = 2.312363
HBA1_TRENE F	4369001 C	HBA_HUMAN Ful	444124 HE	1NQP	2AA1	4HHB	48.53	0.8	Sc=6.63791, min distance = 2.03153

HBA1_TRENE Fu	4369100 C	HBB_HUMAN Ful	444124 HE 1NQP	2AA1	1QSH	45.89	0.93	Sc=6.67317, min distance = 2.18858
HBA1_TRENE Fu	444097 He	HBA_HUMAN Ful	444124 HE 1NQP	2AA1	1RPS	48.53	0.95	Sc=6.73133, min distance = 1.53335
HBA1_TRENE Fu	444207 He	HBA_HORSE Ful	444124 HE 1Y8I	2AA1	2ZLW	51.06	0.99	Sc=6.64046, min distance = 1.70143
HBA1_TRENE Fu	446332 CI	MYG_PHYCA Ful	444098 HE 1A6M	3D1K	1IOP	23.29	0.82	Sc=6.54462, min distance = 2.00314
HBA1_TRENE Fu	446409 HN	HBA_HUMAN Ful	444124 HE 1NQP	2AA1	1J40	48.53	0.99	Sc=6.6545, min distance = 2.225894
HBA4_ONCMY Fu	11957385	MYG_PHYCA Ful	444098 HE 1A6M	3BOM	2CMM			Sc=6.07625, min distance = 2.42123
HBAD_ALDEL Fu	11957385	MYG_PHYCA Ful	444522 HE 1VXD	1WMU	2CMM			Sc=6.06752, min distance = 2.38878
HBAD_CHICK Fu	11957364	HMOX1_HUMAN Fu	444124 HE 1OZW	1HBR	1S13		0.86	Sc=6.41945, min distance = 2.60044
HBAD_CHICK Fu	11957385	MYG_PHYCA Ful	444124 HE 1U7R	1HBR	2CMM			Sc=6.05811, min distance = 2.38189
HBAZ_HUMAN Fu	11957385	MYG_PHYCA Ful	444124 HE 1U7R	1JEB	2CMM			Sc=6.06499, min distance = 2.51061
HBA_ANSAN Fu	11957385	MYG_PHYCA Ful	444124 HE 1U7R	1FAW	2CMM			Sc=5.90328, min distance = 2.48764
HBA_ANSIN Fu	11957385	MYG_PHYCA Ful	444098 HE 1A6M	1A4F	2CMM	25.85		Sc=6.06851, min distance = 2.48764
HBA_ANSIN Fu	11957385	MYG_PHYCA Ful	444124 HE 1U7R	1C40	2CMM	25.85		Sc=6.05493, min distance = 2.32737
HBA_ANSIN Fu	444522 HE	HBA_HORSE Ful	444124 HE 1Y8I	1C40	1Y8K	71.63	0.99	Sc=6.58957, min distance = 2.51752
HBA_ANSIN Fu	444522 HE	HBB_HORSE Ful	444124 HE 1Y8I	1C40	1Y8K	39.31	0.99	Sc=6.5878, min distance = 2.130801
HBA_BOVIN Fu	11957385	MYG_PHYCA Ful	444098 HE 1A6M	2QSS	2CMM	26.53		Sc=6.07929, min distance = 2.18687
HBA_BOVIN Fu	11957385	MYG_PHYCA Ful	444124 HE 1U7R	1FSX	2CMM	26.53		Sc=6.07209, min distance = 2.28551
HBA_CHRBR Fu	11957364	HMOX1_HUMAN Fu	444124 HE 1OZW	1FHJ	1S13		0.86	Sc=6.52288, min distance = 2.64739
HBA_CHRBR Fu	11957371	HMOX1_HUMAN Fu	444124 HE 1OZW	1FHJ	1TWR			Sc=6.44098, min distance = 2.31257
HBA_CHRBR Fu	11957385	MYG_PHYCA Ful	444098 HE 1A6M	2B7H	2CMM			Sc=6.10848, min distance = 2.13804
HBA_CHRBR Fu	11957385	MYG_PHYCA Ful	444124 HE 1U7R	1FHJ	2CMM			Sc=6.06276, min distance = 2.47997
HBA_COLLI Fu	11957363	HBB_HUMAN Ful	444098 HE 1J40	2R80	1RQA		0.77	Sc=6.61593, min distance = 2.10170
HBA_COLLI Fu	11957385	MYG_PHYCA Ful	444098 HE 1A6M	2R80	2CMM			Sc=6.08163, min distance = 2.22848
HBA_DASAK Fu	11957353	HBB_HORSE Ful	444124 HE 1Y8I	1CG5	1IWH		0.87	Sc=6.60591, min distance = 2.77446
HBA_DASAK Fu	11957370	HMOX1_HUMAN Fu	444124 HE 1OZW	1CG5	1TWN			Sc=6.41969, min distance = 2.34740
HBA_DASAK Fu	11957371	HMOX1_HUMAN Fu	444124 HE 1OZW	1CG5	1TWR			Sc=6.4309, min distance = 2.551751
HBA_DASAK Fu	11957385	MYG_PHYCA Ful	444124 HE 1U7R	1CG5	2CMM			Sc=5.91092, min distance = 2.72386
HBA_DASAK Fu	16214774	CP51_MYCTU Ful	444124 HE 2CIB	1CG5	2CI0		0.95	Sc=6.45318, min distance = 2.56464
HBA_DASAK Fu	16741183	CPXA_PSEPU Ful	444124 HE 1RE9	1CG5	2ZAW		0.92	Sc=6.43194, min distance = 2.54161
HBA_DASAK Fu	4369001 C	HBA_HUMAN Ful	444124 HE 1NQP	1CG5	4HHB		0.8	Sc=6.48323, min distance = 2.16207
HBA_DASAK Fu	444044 CI	GLB1_SCAIN Ful	444124 HE 1JZL	1CG5	2R4X	1		Sc=6.61513, min distance = 2.16862
HBA_DASAK Fu	444207 He	PRXC_CALFU Ful	444124 HE 2CPO	1CG5	1CPO		0.99	Sc=6.60986, min distance = 2.42421
HBA_DASAK Fu	444522 HE	HBB_PAGBE Ful	444124 HE 1S5X	1CG5	1PBX		0.99	Sc=6.58602, min distance = 2.54142
HBA_DASAK Fu	446189 CI	HBG1_HUMAN Ful	444124 HE 1I3E	1CG5	1I3D		0.89	Sc=6.60869, min distance = 2.60361
HBA_DASAK Fu	446332 CI	MYG_PHYCA Ful	444124 HE 1U7R	1CG5	1IOP		0.8	Sc=6.57831, min distance = 2.17931
HBA_EQUAS Fu	11957385	MYG_PHYCA Ful	444098 HE 1A6M	1S0H	2CMM			Sc=6.07311, min distance = 2.19266
HBA_HORSE Fu	11957385	MYG_PHYCA Ful	444098 HE 1A6M	2D5X	2CMM	26.67		Sc=5.94105, min distance = 2.13612

HBA_HORSE Fu	11957385	MYG_PHYCA Ful	444124 HE	1U7R	1Y8I	2CMM	26.67		Sc=6.0504, min distance = 2.439068
HBA_HORSE Fu	11957385	MYG_PHYCA Ful	444207 He	1VXG	2ZLW	2CMM	26.67		Sc=6.04905, min distance = 2.56961
HBA_HORSE Fu	11957385	MYG_PHYCA Ful	444522 HE	1VXD	1Y8K	2CMM	26.67		Sc=6.07311, min distance = 2.33777
HBA_HORSE Fu	16214774	CP51_MYCTU Fu	444124 HE	2CIB	1Y8I	2CI0		0.95	Sc=6.34788, min distance = 2.10147
HBA_HUMAN Fu	11957385	MYG_PHYCA Ful	444097 He	2EVK	1RPS	2CMM	26.85		Sc=6.02571, min distance = 2.98672
HBA_HUMAN Fu	11957385	MYG_PHYCA Ful	444124 HE	1U7R	1NQP	2CMM	26.85		Sc=6.07311, min distance = 2.44972
HBA_HUMAN Fu	11957385	MYG_PHYCA Ful	444522 HE	1VXD	2DN1	2CMM	26.85		Sc=6.08163, min distance = 2.19880
HBA_MELGA Fu	11957385	MYG_PHYCA Ful	444207 He	1VXG	2QMB	2CMM			Sc=6.07504, min distance = 2.35552
HBA_MUSGR Fu	11957371	HMOX1_HUMAN Fu	444124 HE	1OZW	1GCV	1TWR			Sc=6.4395, min distance = 2.611277
HBA_MUSGR Fu	11957385	MYG_PHYCA Ful	444124 HE	1U7R	1GCV	2CMM			Sc=5.91092, min distance = 2.78456
HBA_MUSGR Fu	444207 He	NOS2_MOUSE Fu	444124 HE	2ORT	1GCV	1NOS		0.99	Sc=6.58602, min distance = 2.51853
HBA_PAGBE Fu	11957353	HBA_HORSE Ful	444522 HE	1Y8K	1PBX	1IWH	52.48	0.87	Sc=6.60282, min distance = 2.63582
HBA_PAGBE Fu	11957363	HBA_HUMAN Ful	444522 HE	2DN1	1PBX	1RQA	50	0.81	Sc=6.58492, min distance = 2.25491
HBA_PAGBE Fu	11957364	HMOX1_HUMAN Fu	444124 HE	1OZW	1S5X	1S13		0.86	Sc=6.5128, min distance = 2.041174
HBA_PAGBE Fu	11957370	HMOX1_HUMAN Fu	444124 HE	1OZW	1S5X	1TWN			Sc=6.45383, min distance = 2.05845
HBA_PAGBE Fu	11957373	CATE_ECOLI Fu	444124 HE	1IPH	1S5X	1P81		0.79	Sc=6.59079, min distance = 2.05775
HBA_PAGBE Fu	11957385	MYG_PHYCA Ful	444098 HE	1A6M	2H8F	2CMM	23.29		Sc=6.08056, min distance = 2.46853
HBA_PAGBE Fu	11957385	MYG_PHYCA Ful	444124 HE	1U7R	1S5X	2CMM	23.29		Sc=6.07209, min distance = 2.39503
HBA_PAGBE Fu	11957385	MYG_PHYCA Ful	444522 HE	1VXD	1PBX	2CMM	23.29		Sc=6.0605, min distance = 2.225665
HBA_PAGBE Fu	16741062	MYG_PHYCA Ful	444098 HE	1A6M	2H8F	1MBN	23.29	0.84	Sc=6.65669, min distance = 2.31753
HBA_PAGBE Fu	16741062	MYG_PHYCA Ful	444124 HE	1U7R	1S5X	1MBN	23.29	0.87	Sc=6.64871, min distance = 2.00642
HBA_PAGBE Fu	4368974 C	HBA_HUMAN Ful	444522 HE	2DN1	1PBX	1FN3	50	0.94	Sc=6.59665, min distance = 2.08860
HBA_PAGBE Fu	4368974 C	HBB_HUMAN Ful	444522 HE	2DN1	1PBX	1FN3	45.89	0.94	Sc=6.59193, min distance = 1.98405
HBA_PAGBE Fu	444097 He	HBA_HUMAN Ful	444522 HE	2DN1	1PBX	1RPS	50	0.96	Sc=6.58957, min distance = 2.37283
HBA_PAGBE Fu	444097 He	HBB_HUMAN Ful	444522 HE	2DN1	1PBX	1RPS	45.89	0.96	Sc=6.60172, min distance = 2.13643
HBA_PAGBE Fu	444207 He	HBA_HORSE Ful	444124 HE	1Y8I	1S5X	2ZLW	52.48	0.99	Sc=6.59736, min distance = 2.17690
HBA_PAGBE Fu	444207 He	HBB_HORSE Ful	444124 HE	1Y8I	1S5X	2ZLW	43.57	0.99	Sc=6.59553, min distance = 2.35198
HBA_PAGBE Fu	444207 He	HBB_HORSE Ful	444522 HE	1Y8K	1PBX	2ZLW	43.57	1	Sc=6.59898, min distance = 2.11775
HBA_PAGBE Fu	446409 HN	HBA_HUMAN Ful	444522 HE	2DN1	1PBX	1J40	50	0.98	Sc=6.59485, min distance = 2.41064
HBA_PIG Full	11957364	HMOX1_HUMAN Fu	444124 HE	1OZW	1QPW	1S13		0.86	Sc=6.41831, min distance = 2.50907
HBA_PIG Full	11957370	HMOX1_HUMAN Fu	444124 HE	1OZW	1QPW	1TWN			Sc=6.3424, min distance = 2.163247
HBA_PIG Full	11957385	MYG_PHYCA Ful	444124 HE	1U7R	1QPW	2CMM			Sc=5.90982, min distance = 2.66652
HBA_PIG Full	11957385	MYG_PHYCA Ful	444522 HE	1VXD	2PGH	2CMM			Sc=6.05263, min distance = 2.67630
HBA_PSIKR Fu	11957385	MYG_PHYCA Ful	444098 HE	1A6M	2ZFB	2CMM			Sc=6.06851, min distance = 2.30817
HBA_RABIT Fu	11957385	MYG_PHYCA Ful	444098 HE	1A6M	2RAO	2CMM			Sc=6.05928, min distance = 2.34087
HBB1_MOUSE Fu	11957385	MYG_PHYCA Ful	444124 HE	1U7R	1JEB	2CMM			Sc=6.06851, min distance = 2.44215
HBB1_ONCMY Fu	11957385	MYG_PHYCA Ful	444098 HE	1A6M	1OUT	2CMM			Sc=6.07625, min distance = 2.48620

HBBC_TRENE Fu	11957385	MYG_PHYCA Full	444124 HE 1U7R	2AA1	2CMM			Sc=5.94724, min distance = 2.61311
HBB_ALDEL Fu	11957385	MYG_PHYCA Full	444522 HE 1VXD	1WMU	2CMM			Sc=6.06974, min distance = 2.30854
HBB_ANSAN Fu	11957385	MYG_PHYCA Full	444124 HE 1U7R	1FAW	2CMM			Sc=6.04785, min distance = 2.50481
HBB_ANSIN Fu	11957385	MYG_PHYCA Full	444098 HE 1A6M	1A4F	2CMM	27.78		Sc=6.06276, min distance = 2.19172
HBB_ANSIN Fu	11957385	MYG_PHYCA Full	444124 HE 1U7R	1C40	2CMM	27.78		Sc=6.05928, min distance = 2.25512
HBB_BOVIN Fu	11957385	MYG_PHYCA Full	444124 HE 1U7R	1FSX	2CMM			Sc=6.07102, min distance = 2.41677
HBB_CHRBR Fu	11957385	MYG_PHYCA Full	444124 HE 1U7R	1FHJ	2CMM			Sc=6.05811, min distance = 2.41873
HBB_COLLI Fu	11957385	MYG_PHYCA Full	444098 HE 1A6M	2R80	2CMM			Sc=6.06631, min distance = 2.09066
HBB_DASAK Fu	11957385	MYG_PHYCA Full	444124 HE 1U7R	1CG5	2CMM			Sc=5.92694, min distance = 2.78982
HBB_DASAK Fu	24883662	MYG_HORSE Full	444124 HE 1DWT	1CG5	1YMC		0.87	Sc=6.65561, min distance = 2.09646
HBB_HORSE Fu	11957385	MYG_PHYCA Full	444098 HE 1A6M	2D5X	2CMM	27.4		Sc=5.93567, min distance = 2.08693
HBB_HORSE Fu	11957385	MYG_PHYCA Full	444124 HE 1U7R	1Y8I	2CMM	27.4		Sc=6.05379, min distance = 2.38363
HBB_HORSE Fu	11957385	MYG_PHYCA Full	444207 He 1VXG	2ZLW	2CMM	27.4		Sc=5.89833, min distance = 2.52416
HBB_HORSE Fu	11957385	MYG_PHYCA Full	444522 HE 1VXD	1Y8K	2CMM	27.4		Sc=6.05928, min distance = 2.15801
HBB_HORSE Fu	16214774	CP51_MYCTU Fu	444124 HE 2CIB	1Y8I	2CI0		0.95	Sc=6.36186, min distance = 2.30487
HBB_HUMAN Fu	11957385	MYG_PHYCA Full	444097 He 2EVK	1RPS	2CMM	26.53		Sc=5.91435, min distance = 2.31236
HBB_HUMAN Fu	11957385	MYG_PHYCA Full	444098 HE 1A6M	1J40	2CMM	26.53		Sc=6.08366, min distance = 2.47269
HBB_HUMAN Fu	11957385	MYG_PHYCA Full	444124 HE 1U7R	1NQP	2CMM	26.53		Sc=6.06499, min distance = 2.35011
HBB_LEIXA Fu	11957385	MYG_PHYCA Full	444124 HE 1U7R	1SPG	2CMM			Sc=5.90727, min distance = 2.24024
HBB_MUSGR Fu	11957364	HMOX1_HUMAN Fu	444124 HE 1OZW	1GCV	1S13		0.86	Sc=6.50799, min distance = 1.45243
HBB_MUSGR Fu	11957370	HMOX1_HUMAN Fu	444124 HE 1OZW	1GCV	1TWN			Sc=6.43956, min distance = 2.19803
HBB_MUSGR Fu	11957371	HMOX1_HUMAN Fu	444124 HE 1OZW	1GCV	1TWR			Sc=6.44737, min distance = 2.40587
HBB_MUSGR Fu	11957385	MYG_PHYCA Full	444124 HE 1U7R	1GCV	2CMM			Sc=5.90464, min distance = 2.78906
HBB_MUSGR Fu	444207 He	NOS2_MOUSE Fu	444124 HE 2ORT	1GCV	1NOS		0.99	Sc=6.63676, min distance = 2.48192
HBB_PAGBE Fu	11957385	MYG_PHYCA Full	444098 HE 1A6M	2H8F	2CMM			Sc=6.08257, min distance = 2.56338
HBB_PAGBE Fu	11957385	MYG_PHYCA Full	444124 HE 1U7R	1S5X	2CMM			Sc=6.12058, min distance = 1.99798
HBB_PAGBE Fu	11957385	MYG_PHYCA Full	444522 HE 1VXD	1PBX	2CMM			Sc=5.92916, min distance = 2.15213
HBB_PANPA Fu	11957385	MYG_PHYCA Full	444124 HE 1U7R	1CH4	2CMM			Sc=6.06631, min distance = 2.21088
HBB_PANTR Fu	11957385	MYG_PHYCA Full	444124 HE 1U7R	1CH4	2CMM			Sc=6.07209, min distance = 2.48676
HBB_PIG Full=	11957385	MYG_PHYCA Full	444124 HE 1U7R	1QPW	2CMM			Sc=6.034, min distance = 2.7990635
HBB_PIG Full=	11957385	MYG_PHYCA Full	444522 HE 1VXD	2PGH	2CMM			Sc=6.05263, min distance = 2.65556
HBB_PSIKR Fu	11957385	MYG_PHYCA Full	444098 HE 1A6M	2ZFB	2CMM			Sc=6.0515, min distance = 2.193549
HBB_RABIT Fu	11957385	MYG_PHYCA Full	444098 HE 1A6M	2RAO	2CMM			Sc=6.05811, min distance = 2.17318
HBB_TRENE Fu	11957385	MYG_PHYCA Full	444098 HE 1A6M	3D1K	2CMM			Sc=5.93446, min distance = 2.22860
HBD_HUMAN Fu	11957385	MYG_PHYCA Full	444124 HE 1U7R	1SHR	2CMM			Sc=5.92441, min distance = 2.36327
HBE_HUMAN Fu	11957353	HBB_HORSE Full	444124 HE 1Y8I	1A9W	1IWH		0.87	Sc=6.5763, min distance = 2.372922
HBE_HUMAN Fu	11957385	MYG_PHYCA Full	444124 HE 1U7R	1A9W	2CMM			Sc=6.04785, min distance = 2.33844

HBF1_URECA Fu	11957385	MYG_PHYCA Ful	444124 HE	1U7R	1ITH	2CMM			Sc=6.04266, min distance = 2.449925
HBG1_HUMAN Fu	11957385	MYG_PHYCA Ful	444124 HE	1U7R	1I3E	2CMM			Sc=6.06499, min distance = 2.355840
HBG2_HUMAN Fu	11957385	MYG_PHYCA Ful	444098 HE	1A6M	1FDH	2CMM			Sc=6.06166, min distance = 2.709970
HBL_HORVU Fu	11957370	HMOX1_HUMAN Fu	444124 HE	1OZW	2OIF	1TWN			Sc=6.47156, min distance = 2.396376
HBL_HORVU Fu	444522 HE	MYG_PIG Full=N	444124 HE	1MYH	2OIF	1MNI		0.99	Sc=6.59318, min distance = 1.856515
HBL_ZEAMP Fu	11957353	HBA_HORSE Ful	444124 HE	1Y8I	2R50	1IWH		0.87	Sc=6.67592, min distance = 1.921979
HBL_ZEAMP Fu	11957385	MYG_PHYCA Ful	444124 HE	1U7R	2R50	2CMM			Sc=6.08976, min distance = 2.222298
HBL_ZEAMP Fu	444522 HE	BFR_ECOLI Ful	444124 HE	1BFR	2R50	1BCF		0.99	Sc=6.67805, min distance = 2.146508
HBL_ZEAMP Fu	444522 HE	CP2C9_HUMAN Fu	444124 HE	1OG2	2R50	1R9O		0.99	Sc=6.66271, min distance = 2.056549
HBL_ZEAMP Fu	444522 HE	GLB1_GLYDI Fu	444124 HE	1JF3	2R50	1HBG		0.99	Sc=6.6709, min distance = 2.0799658
HBL_ZEAMP Fu	444522 HE	HBA_PAGBE Ful	444124 HE	1S5X	2R50	1PBX		0.99	Sc=6.66216, min distance = 2.094679
HBSAG_HBVD3 I	64689 bet	Q9RX51_DEIRA I	6255 malt	2BHZ	1IUD	2BY0		0.87	Sc=5.74332, min distance = 2.314496
HBSAG_HBVD3 I	79025 alp	AMYB_BACCE Fu	6255 malt	1B9Z	1IUD	1VEM		0.87	Sc=5.79566, min distance = 2.223088
HCD2_HUMAN Fu	169266 1,	GALE_HUMAN Fu	5893 nadi	1HZJ	2O23	1I3K		0.79	Sc=6.77738, min distance = 2.312909
HCD2_HUMAN Fu	439153 Di	GALE_ECOLI Fu	5893 nadi	1UDC	2O23	1UDB		0.79	Sc=6.77476, min distance = 2.275555
HCD2_HUMAN Fu	439153 Di	Q9BJJ9_PLAFA I	5893 nadi	1UH5	2O23	1V35		0.79	Sc=6.77154, min distance = 2.348617
HCD2_RAT Ful	169266 1,	GALE_HUMAN Fu	5893 nadi	1HZJ	1E6W	1I3K		0.79	Sc=6.77086, min distance = 2.206131
HCD2_RAT Ful	439153 Di	GALE_ECOLI Fu	5893 nadi	1UDC	1E6W	1UDB		0.79	Sc=6.76808, min distance = 2.141562
HCD2_RAT Ful	439153 Di	Q9BJJ9_PLAFA I	5893 nadi	1UH5	1E6W	1V35		0.79	Sc=6.76564, min distance = 2.220274
HCDH_HUMAN Fu	445794 AD	ADHX_HUMAN Fu	5893 nadi	2FZW	1F0Y	2FZE			Sc=6.63432, min distance = 1.313676
HCDH_HUMAN Fu	6022 Aden	Q5SI02_THET8 I	5893 nadi	2BJK	1F0Y	2BJA			Sc=6.43359, min distance = 1.471753
HCDH_PIG Ful	16129587	G3PA_SPIOL Fu	5893 nadi	1NBO	3HDH	2PKR			Sc=6.03688, min distance = 2.397978
HCDH_PIG Ful	169266 1,	GALE_HUMAN Fu	5893 nadi	1HZJ	3HDH	1I3K		0.79	Sc=6.73639, min distance = 1.782604
HCDH_PIG Ful	445794 AD	ADHX_HUMAN Fu	5893 nadi	2FZW	3HDH	2FZE			Sc=6.62564, min distance = 1.240251
HCDH_PIG Ful	446050 CI	G3P_HUMAN Ful	5893 nadi	1U8F	3HDH	3GPD		0.95	Sc=6.73002, min distance = 1.221828
HCDH_PIG Ful	446288 NA	G3P_PALVE Ful	5893 nadi	1DSS	3HDH	1IHX			Sc=6.71937, min distance = 1.979612
HCDH_PIG Ful	447224 AE	FADB_PSEFR Fu	5893 nadi	1WDK	3HDH	1WDL			Sc=6.60079, min distance = 1.592750
HCDH_PIG Ful	6022 Aden	Q5SI02_THET8 I	5893 nadi	2BJK	3HDH	2BJA			Sc=6.24046, min distance = 2.175723
HCDH_PIG Ful	6083 aden	NADE_ECOLI Fu	5893 nadi	1WXH	3HDH	1WXI			Sc=6.33814, min distance = 1.746754
HCDR_XANP2 Fu	439153 Di	GALE_ECOLI Fu	5893 nadi	1UDC	2CFC	1UDB		0.79	Sc=6.79285, min distance = 2.144392
HCDR_XANP2 Fu	439153 Di	Q9BJJ9_PLAFA I	5893 nadi	1UH5	2CFC	1V35		0.79	Sc=6.79166, min distance = 2.074918
HCDR_XANP2 Fu	6022 Aden	Q5SI02_THET8 I	5893 nadi	2BJK	2CFC	2BJA			Sc=6.46533, min distance = 0.954763
HCK_HUMAN Fu	16058649	IGF1R_HUMAN Fu	33113 gam	1JQH	1AD5	2OJ9	38.32		Sc=6.41342, min distance = 2.266929
HCK_HUMAN Fu	188966 dA	HSLU_ECOLI Fu	33113 gam	1E94	1AD5	1G4A		0.97	Sc=5.84409, min distance = 2.379457
HCK_HUMAN Fu	23656870	CSK2A_MAIZE Fu	33113 gam	1LP4	1AD5	2PVN	22.43		Sc=6.45432, min distance = 2.077895
HCK_HUMAN Fu	2396 bisi	PIM1_HUMAN Fu	33113 gam	1XR1	1AD5	1XWS	29.82		Sc=6.39607, min distance = 2.129139
HCK_HUMAN Fu	3547 Fasu	ROCK1_HUMAN Fu	33113 gam	2V55	1AD5	2ESM	24.88		Sc=5.9594, min distance = 1.7739551

HCK_HUMAN Fu	36735 Gpp	CSK2A_MAIZE F	33113 gam	1LP4	1AD5	1DAY	22.43	0.8	Sc=5.94856, min distance = 1.77033
HCK_HUMAN Fu	444367 CI	CSK2A_MAIZE F	33113 gam	1LP4	1AD5	1OM1	22.43		Sc=6.12205, min distance = 2.07835
HCK_HUMAN Fu	456214 pu	SRC_HUMAN Ful	33113 gam	2SRC	1AD5	1YOM	69.58		Sc=6.22697, min distance = 1.95249
HCK_HUMAN Fu	5281701 T	PIM1_HUMAN Fu	33113 gam	1XR1	1AD5	2O65	29.82		Sc=6.27012, min distance = 2.78580
HCK_HUMAN Fu	5326739 i	GSK3B_HUMAN F	33113 gam	1J1B	1AD5	1Q41	27.6		Sc=6.28232, min distance = 1.75093
HCK_HUMAN Fu	5326978 1	CSK2A_MAIZE F	33113 gam	1LP4	1AD5	1ZOH	22.43		Sc=5.63386, min distance = 1.93703
HCK_HUMAN Fu	5327148 C	IPKA_RABIT Fu	33113 gam	1CDK	1AD5	2ERZ			Sc=6.11595, min distance = 1.70147
HCK_HUMAN Fu	6083 ader	ENTP2_RAT Ful	33113 gam	3CJA	1AD5	3CJ7		0.97	Sc=6.22361, min distance = 2.07137
HCK_HUMAN Fu	60961 ade	PIM1_HUMAN Fu	33113 gam	1XR1	1AD5	1YI4	29.82	0.93	Sc=6.19103, min distance = 1.77896
HCK_HUMAN Fu	91532 AMP	PURT_ECOLI Fu	33113 gam	1EYZ	1AD5	1KJI		0.99	Sc=5.98382, min distance = 1.29390
HCN2_MOUSE F	188955 Cy	CRP_ECOLI Ful	6076 Cycl	1HW5	3BPZ	1J59	35.63	0.95	Sc=6.28202, min distance = 2.56417
HCN2_MOUSE F	656964 1y	PDE4D_HUMAN F	6076 Cycl	2PW3	3BPZ	1Y2C			Sc=5.9783, min distance = 2.441775
HCN2_MOUSE F	656966 1y	PDE4D_HUMAN F	6076 Cycl	2PW3	3BPZ	1Y2E			Sc=6.11325, min distance = 1.44802
HCN2_MOUSE F	6858240 C	KAP0_BOVIN Fu	24316 cyc	1RL3	1Q3E	1NE6		0.79	Sc=6.30338, min distance = 2.13308
HCN2_MOUSE F	6858240 C	KAP0_BOVIN Fu	6076 Cycl	1RGS	3BPZ	1NE6		0.98	Sc=6.29616, min distance = 2.04959
HCRA_THAAR F	10918 Car	OCTC_MOUSE Fu	23831 HEP	1XL7	1RM6	1XL8			Sc=5.72613, min distance = 2.23784
HCRA_THAAR F	448574 CI	MOP_DESGI Ful	4369335 C	1VLB	1RM6	1SIJ		0.95	Sc=6.78031, min distance = 2.32277
HCRA_THAAR F	854023 Ep	Q8WSF8_APLCA	23831 HEP	2BR7	1RM6	2BYQ			Sc=5.8288, min distance = 2.335185
HCRA_THAAR F	9543460 C	MOP_DESGI Ful	4369335 C	1VLB	1RM6	1ZCS		0.92	Sc=6.78355, min distance = 2.11374
HDAC7_HUMAN	92249 Cou	HDAC8_HUMAN F	444732 tr	1T64	3C10	2V5W			Sc=5.85135, min distance = 1.34471
HDAC8_HUMAN	3428304 E	HDAH_ALCSD Fu	5311 Vori	1ZZ1	1T69	1ZZ3			Sc=5.89127, min distance = 1.67506
HDHA_ECOLI F	11987635	ADH_DROLE Ful	5893 nadi	1SBY	1FMC	1B2L		0.76	Sc=6.50193, min distance = 2.26512
HDHA_ECOLI F	15942665	INHA_MYCTU Fu	5893 nadi	2H7I	1FMC	2H9I		0.88	Sc=6.85111, min distance = 2.31493
HDHA_ECOLI F	439153 Di	Q9BJJ9_PLAFA	5893 nadi	1UH5	1FMC	1V35		0.79	Sc=6.73188, min distance = 2.06240
HDHA_ECOLI F	449263 IN	INHA_MYCTU Fu	5893 nadi	2H7I	1FMC	2NV6		0.93	Sc=6.83295, min distance = 2.35002
HDHA_ECOLI F	6022 Ader	Q5SI02_THET8	5893 nadi	2BJK	1FMC	2BJA			Sc=6.25182, min distance = 2.10795
HEM6_HUMAN F	1005 phos	F16PA_ECOLI F	311 citri	2OWZ	2AEX	2OX3			Sc=5.64422, min distance = 2.09623
HEM6_HUMAN F	439351 3-	AROQ_HELPY Fu	311 citri	2C4V	2AEX	1J2Y			Sc=6.04165, min distance = 2.02470
HEM6_HUMAN F	439459 Ho	NIFD_KLEPN Fu	311 citri	1H1L	2AEX	1QGU		0.92	Sc=6.10075, min distance = 2.07531
HEM6_HUMAN F	439459 Ho	NIFK_KLEPN Fu	311 citri	1H1L	2AEX	1QGU		0.92	Sc=6.10613, min distance = 2.10115
HEM6_HUMAN F	439655 d-	MENF_ECOLI Fu	311 citri	3BZM	2AEX	2EUA			Sc=5.79788, min distance = 1.87260
HEM6_HUMAN F	51 2-Oxop	SERA_ECOLI Fu	311 citri	2P9E	2AEX	1YBA		0.76	Sc=5.87464, min distance = 1.06508
HEMAT_BACSU	11957364	HMOX1_HUMAN F	444124 HE	1OZW	1OR4	1S13		0.86	Sc=6.41581, min distance = 2.30078
HEMAT_BACSU	11957370	HMOX1_HUMAN F	444124 HE	1OZW	1OR4	1TWN			Sc=6.43595, min distance = 2.28517
HEMAT_BACSU	11957371	HMOX1_HUMAN F	444124 HE	1OZW	1OR4	1TWR			Sc=6.46472, min distance = 1.99644
HEMAT_BACSU	11957385	MYG_PHYCA Ful	444124 HE	1U7R	1OR4	2CMM			Sc=5.86404, min distance = 2.20163
HEMA_I18A0 F	4369268 C	Q309D1_9AGAR	24139 ace	2C4D	1RUZ	2BWM		0.88	Sc=5.79023, min distance = 2.55314

HEMA_I18A0 Fı	94214 Met	MBL1_RAT Full	82313 1rd	1KWV	1RUZ	1AFA			Sc=5.8196, min distance = 1.711123(
HEMA_I63A3 Fı	448418 DA	Q82500_9INFA I	444885 O-	1RVT	1MQM	1RV0	38.72	0.98	Sc=6.32575, min distance = 2.16871
HEMH_HUMAN Fı	11957355	CY1_BOVIN Full	444098 HE	1L0N	2QD2	1L0L		0.77	Sc=6.60387, min distance = 2.06773
HEMH_HUMAN Fı	11957360	CY1_BOVIN Full	444098 HE	1L0N	2QD2	1NTM			Sc=6.60669, min distance = 2.11626
HEMH_HUMAN Fı	11957385	MYG_PHYCA Full	444098 HE	1A6M	2QD2	2CMM			Sc=5.92098, min distance = 2.41327
HEMK_ECOLI Fı	188380 Ac	MCES_ENCCU Fu	439155 Ad	1RI1	2B3T	1Z3C		0.91	Sc=6.58785, min distance = 2.47335
HEMK_ECOLI Fı	446535 CI	HNMT_HUMAN Fu	439155 Ad	2AOT	2B3T	1JQE		0.95	Sc=6.54865, min distance = 2.54707
HEMK_ECOLI Fı	60961 ade	PIMT_PYRFU Fu	439155 Ad	1JG1	2B3T	1JG2		0.84	Sc=6.34279, min distance = 2.57969
HEMK_ECOLI Fı	65482 sir	ERM_BACSU Full	439155 Ad	1QAN	2B3T	1QAQ		0.88	Sc=6.59715, min distance = 2.59714
HEMO_RABIT Fı	11957385	MYG_PHYCA Full	444098 HE	1A6M	1QHU	2CMM			Sc=6.20064, min distance = 2.13380
HEXA_HUMAN Fı	446429 1j	HEXB_HUMAN Fu	446115 NG	1NP0	2GK1	1NOW			Sc=6.09825, min distance = 2.33275
HEXB_HUMAN Fı	444162 CI	Q54276_SERMA I	87901 EIN	1UR8	1O7A	1UR9		0.78	Sc=6.05212, min distance = 2.65602
HEX_ADE02 Fu	444389 N,	IPSP_HUMAN Fu	311 citri	2HI9	1P2Z	3DY0			Sc=5.68307, min distance = 1.28162
HEX_ADE02 Fu	445905 1g	AROQ_HELPY Fu	311 citri	2C4V	1P2Z	2C57			Sc=5.73555, min distance = 2.66462(
HEX_ADE02 Fu	448573 FI	CASP7_HUMAN Fı	311 citri	2QL9	1P2Z	1SHL			Sc=6.00134, min distance = 0.92230
HEX_ADE02 Fu	51 2-Oxop	SERA_ECOLI Fu	311 citri	2P9E	1P2Z	1YBA		0.76	Sc=5.62282, min distance = 1.88182
HEX_ADE02 Fu	8778 Laur	CTXA3_NAJAT Fı	311 citri	1XT3	1P2Z	1H0J			Sc=5.78575, min distance = 0.198098
HGDC_ACIFE Fı	3454 ganc	KITH_HHV11 Fu	6022 Aden	2VTK	1HUX	1KI2			Sc=6.14376, min distance = 2.02773
HGDC_ACIFE Fı	36735 Gpp	PARM_ECOLX Fu	6022 Aden	1MWM	1HUX	2ZGZ		0.79	Sc=6.47233, min distance = 2.02615
HGDC_ACIFE Fı	398148 1e	CDK2_HUMAN Fu	6022 Aden	1GY3	1HUX	1E1X			Sc=6.18641, min distance = 1.63622
HGDC_ACIFE Fı	445940 O6	CDK2_HUMAN Fu	6022 Aden	1GY3	1HUX	1GZ8			Sc=6.17643, min distance = 2.29023
HGDC_ACIFE Fı	446090 1h	NDKC_DICDI Fu	6022 Aden	1KDN	1HUX	1HIY		0.97	Sc=6.44121, min distance = 1.76509
HGDC_ACIFE Fı	4564 1e1v	CDK2_HUMAN Fu	6022 Aden	1GY3	1HUX	1E1V			Sc=6.15853, min distance = 1.83603(
HGDC_ACIFE Fı	4725 penc	KITH_HHV11 Fu	6022 Aden	2VTK	1HUX	1KI3			Sc=6.18561, min distance = 1.47850
HGDC_ACIFE Fı	5957 Aden	Q53W83_THET8 I	6022 Aden	1V1A	1HUX	1V1B		0.99	Sc=6.46543, min distance = 2.03422
HGDC_ACIFE Fı	6031 Uric	DCK_HUMAN Full	6022 Aden	1P5Z	1HUX	2ZIA			Sc=6.30768, min distance = 2.13669
HGDC_ACIFE Fı	6031 Uric	RIR1_YEAST Fu	6022 Aden	2CVX	1HUX	2CVV			Sc=6.32317, min distance = 2.20365
HGDC_ACIFE Fı	6338566 C	MYS2_DICDI Fu	6022 Aden	1VOM	1HUX	1D1C			Sc=6.24785, min distance = 2.02948
HGDC_ACIFE Fı	8977 1dar	PARM_ECOLX Fu	6022 Aden	1MWM	1HUX	2ZGY		0.8	Sc=6.42476, min distance = 2.16357(
HGDC_ACIFE Fı	8977 1dar	RIR1_YEAST Fu	6022 Aden	2CVX	1HUX	2CVW		0.8	Sc=6.43911, min distance = 2.20521
HGF_HUMAN Fu	164795 N(	RBCMT_PEA Full	23831 HEF	1MLV	1BHT	2H2J			Sc=5.76007, min distance = 1.98403
HGF_HUMAN Fu	165293 De	CTXA3_NAJAT Fı	444389 N,	1XT3	1GMO	2BHI			Sc=5.6666, min distance = 0.743313
HGF_HUMAN Fu	854023 Ep	Q8WSF8_APLCA I	23831 HEF	2BR7	1BHT	2BYQ			Sc=5.90081, min distance = 1.21802
HGXR_TOXGO Fı	11287125	TGT_ZYMMO Full	100684 9-	1Q2R	1FSG	2BBF			Sc=6.21184, min distance = 1.43133
HGXR_TOXGO Fı	168120 Pc	HINT1_RABIT Fı	6804 guan	3RHN	1QK3	5RHN		0.78	Sc=6.40846, min distance = 1.92684
HGXR_TOXGO Fı	171 preQ1	TGT_ZYMMO Full	100684 9-	1Q2R	1FSG	3BLD			Sc=6.00006, min distance = 1.32013
HGXR_TOXGO Fı	190 adeni	RICI_RICCO Fu	100684 9-	1IL4	1FSG	2P8N			Sc=5.76959, min distance = 2.89012

HGXR_TOXGO F	24741806	TGT_ZYMMO Ful	100684 9-	1Q2R	1FSG	2Z7K		Sc=6.27772, min distance = 1.32424
HGXR_TOXGO F	445390 CI	TGT_ZYMMO Ful	100684 9-	1Q2R	1FSG	1ENU		Sc=6.09147, min distance = 1.43867
HGXR_TOXGO F	445497 CI	TGT_ZYMMO Ful	100684 9-	1Q2R	1FSG	1F3E		Sc=6.13286, min distance = 1.89718
HGXR_TOXGO F	446312 1i	RICI_RICCO Fu	100684 9-	1IL4	1FSG	1IL9		Sc=5.92452, min distance = 2.02541
HGXR_TOXGO F	446623 1k	TGT_ZYMMO Ful	100684 9-	1Q2R	1FSG	1K4H		Sc=6.27522, min distance = 0.87541
HGXR_TOXGO F	447262 CI	TGT_ZYMMO Ful	100684 9-	1Q2R	1FSG	2Z1W		Sc=6.15687, min distance = 2.21232
HGXR_TOXGO F	448010 1c	TGT_ZYMMO Ful	100684 9-	1Q2R	1FSG	1R5Y		Sc=6.08118, min distance = 1.32973
HGXR_TOXGO F	448488 1s	TGT_ZYMMO Ful	100684 9-	1Q2R	1FSG	1S38		Sc=6.03424, min distance = 2.19433
HGXR_TOXGO F	6022 Aden	NDK_PYRHO Ful	6804 guan	2DXF	1QK3	2DYA	0.8	Sc=6.47813, min distance = 1.45325
HGXR_TOXGO F	65110 AIC	PURO_METTH Fu	8582 Inos	2NTK	1QK4	2NTL	0.97	Sc=6.30372, min distance = 1.61946
HGXR_TOXGO F	764 guani	TGT_ZYMMO Ful	100684 9-	1Q2R	1FSG	2PWU		Sc=5.87401, min distance = 2.62887
HGXR_TOXGO F	8977 1dar	NDK_PYRHO Ful	6804 guan	2DXF	1QK3	2DXE	0.99	Sc=6.46763, min distance = 2.20178
HGXR_TOXGO F	96253 7-D	RICI_RICCO Fu	100684 9-	1IL4	1FSG	1IL3		Sc=5.90589, min distance = 2.75204
HGXR_TRIFO F	168120 Po	HINT1_RABIT F	6804 guan	3RHN	1HGX	5RHN	0.78	Sc=6.3787, min distance = 2.107770
HGXR_TRIFO F	444699 1b	HPRT_HUMAN Fu	6804 guan	1HMP	1HGX	1BZY		Sc=6.44981, min distance = 2.11816
HINT1_HUMAN	440641 1m	O57693_THETE	6083 aden	1UXU	1KPF	1UXR		Sc=5.98364, min distance = 2.07930
HINT1_HUMAN	445432 CI	SYQ_ECOLI Ful	6083 aden	1ZJW	1KPF	1QTQ	0.84	Sc=6.0658, min distance = 2.171020
HINT1_HUMAN	6804 guar	PDE10_HUMAN F	6083 aden	2OUN	1KPF	2OUQ	0.8	Sc=5.89723, min distance = 0
HINT1_HUMAN	9547921 G	SYQ_ECOLI Ful	6083 aden	1ZJW	1KPF	2RE8	0.82	Sc=6.15865, min distance = 2.20105
HIS1_ECOLI F	2519 caff	PYGM_RABIT Fu	6083 aden	8GPB	1H3D	1GFZ		Sc=5.71163, min distance = 2.65290
HIS1_ECOLI F	403186 CI	PYGM_RABIT Fu	6083 aden	8GPB	1H3D	1Z62		Sc=6.34829, min distance = 2.32232
HIS1_ECOLI F	445736 5'	SYS_THET2 Ful	6083 aden	1SES	1H3D	1SET	0.86	Sc=5.99674, min distance = 2.28898
HIS1_ECOLI F	5281672 m	PIM1_HUMAN Fu	6083 aden	1YXU	1H3D	2O63		Sc=6.35754, min distance = 2.77116
HIS1_ECOLI F	6022 Aden	PSPF_ECOLI Fu	6083 aden	2VII	1H3D	2C98	0.99	Sc=6.07018, min distance = 2.09805
HIS1_ECOLI F	65110 AIC	AAKG1_HUMAN F	6083 aden	2UV4	1H3D	2UV5	0.79	Sc=6.27658, min distance = 2.45114
HIS1_ECOLI F	65110 AIC	YL28_SCHPO Fu	6083 aden	2OOX	1H3D	2QRE	0.79	Sc=5.8945, min distance = 2.198932
HIS1_ECOLI F	65533 cor	O57693_THETE	6083 aden	1UXU	1H3D	1UXT		Sc=5.9636, min distance = 2.103931
HIS1_ECOLI F	6804 guar	NDK_PYRHO Ful	6083 aden	2DXD	1H3D	2DXF	0.8	Sc=5.92734, min distance = 2.68929
HIS1_MYCTU F	656966 1y	PDE4D_HUMAN F	6083 aden	1TB7	1NH8	1Y2E		Sc=5.70849, min distance = 1.91212
HIS4_YEAST F	445496 TE	A2NHM3_MOUSE	311 citri	1ZEA	2AGK	1F3D		Sc=6.1846, min distance = 1.066032
HIS4_YEAST F	51 2-Oxop	SERA_ECOLI Fu	311 citri	2P9E	2AGK	1YBA	0.76	Sc=5.71033, min distance = 1.75522
HIS4_YEAST F	9547936 2	PYRB_ECOLI Fu	311 citri	1R0B	2AGK	2FZC		Sc=5.65846, min distance = 0.77910
HIS8_CORGL F	444286 N-	AAT_ECOLI Ful	1053 pyri	2Q7W	3CQ5	1ASL	0.9	Sc=6.51695, min distance = 2.14956
HIS8_CORGL F	444292 PI	AAT_ECOLI Ful	1053 pyri	2Q7W	3CQ5	1ARG	0.9	Sc=6.48238, min distance = 2.18487
HIS8_CORGL F	444293 PI	HIS8_ECOLI Fu	1053 pyri	1FG7	3CQ5	1GEY	0.89	Sc=6.51038, min distance = 2.14234
HIS8_CORGL F	444362 NE	AAT_ECOLI Ful	1053 pyri	2Q7W	3CQ5	1SPA		Sc=6.131, min distance = 2.1261479
HIS8_CORGL F	845 histi	HIS8_ECOLI Fu	1053 pyri	1FG7	3CQ5	1GEX		Sc=5.88048, min distance = 2.06109

HIS8_ECOLI Fu	3744 Indo	AAT_ECOLI Full	1053 pyri	2Q7W	1FG7	1AHF		Sc=5.87107, min distance = 2.019031
HIS8_ECOLI Fu	4369488 E	AAT_ECOLI Full	1053 pyri	2Q7W	1FG7	1X29	0.89	Sc=6.52676, min distance = 2.221628
HIS8_ECOLI Fu	444286 N-	AAT_ECOLI Full	1053 pyri	2Q7W	1FG7	1ASL	0.9	Sc=6.04762, min distance = 1.955944
HIS8_ECOLI Fu	444292 PI	AAT_ECOLI Full	1053 pyri	2Q7W	1FG7	1ARG	0.9	Sc=6.01746, min distance = 2.135606
HIS8_ECOLI Fu	6140 L-ph	KAT1_HUMAN Fu	1053 pyri	1W7N	1FG7	1W7M		Sc=5.74847, min distance = 2.606767
HIS8_THEMA Fu	1052 pyri	PDXK_SHEEP Fu	95687 Dec	1RFU	1UU2	1RFT	0.92	Sc=5.9307, min distance = 2.1945213
HIS8_THEMA Fu	3744 Indo	AAT_ECOLI Full	1053 pyri	2Q7W	1UU1	1AHF		Sc=5.90383, min distance = 1.242639
HIS8_THEMA Fu	4369488 E	AAT_ECOLI Full	1053 pyri	2Q7W	1UU1	1X29	0.89	Sc=6.07846, min distance = 2.341480
HIS8_THEMA Fu	444286 N-	AAT_ECOLI Full	1053 pyri	2Q7W	1UU1	1ASL	0.9	Sc=6.04762, min distance = 2.124001
HIS8_THEMA Fu	444292 PI	AAT_ECOLI Full	1053 pyri	2Q7W	1UU1	1ARG	0.9	Sc=6.46142, min distance = 1.865931
HIS8_THEMA Fu	444293 PI	HIS8_ECOLI Fu	1053 pyri	1FG7	1UU1	1GEY	0.89	Sc=6.03032, min distance = 2.261465
HIS8_THEMA Fu	444362 NE	AAT_ECOLI Full	1053 pyri	2Q7W	1UU1	1SPA		Sc=6.12908, min distance = 2.121090
HIS8_THEMA Fu	444773 LE	AAT_ECOLI Full	1053 pyri	2Q7W	1UU1	1C9C	0.93	Sc=6.38255, min distance = 2.179422
HIS8_THEMA Fu	444861 CI	AAT_ECOLI Full	1053 pyri	2Q7W	1UU1	1CQ7	0.92	Sc=6.44668, min distance = 2.386168
HIS8_THEMA Fu	444862 CI	AAT_ECOLI Full	1053 pyri	2Q7W	1UU1	1CQ8	0.91	Sc=6.46511, min distance = 2.437150
HIS8_THEMA Fu	6140 L-ph	KAT1_HUMAN Fu	1053 pyri	1W7N	1UU1	1W7M		Sc=5.76659, min distance = 2.198540
HISJ_ECOLI Fu	439767 Ma	HNL_HEVBR Full	6274 hist	1YAS	1HSL	1YB6		Sc=5.69304, min distance = 2.512891
HISJ_ECOLI Fu	5962 L-ly	ARGT_SALTY Fu	6274 hist	1LAG	1HSL	1LST		Sc=5.78164, min distance = 2.736061
HISJ_ECOLI Fu	6322 L-ar	ARGT_SALTY Fu	6274 hist	1LAG	1HSL	1LAF		Sc=5.9839, min distance = 2.7886641
HISP_SALTY Fu	3973 nche	PK3CG_PIG Full	5957 Aden	1E8X	1B0U	1E7V		Sc=5.74613, min distance = 2.189264
HISP_SALTY Fu	6022 Aden	CLCN5_HUMAN Fu	5957 Aden	2J9L	1B0U	2JA3	0.99	Sc=5.92496, min distance = 0.865335
HISP_SALTY Fu	6830 guan	Q381M1_9TRYP I	5957 Aden	2Q0D	1B0U	2Q0E	0.8	Sc=5.66514, min distance = 2.051571
HISX_ECOLI Fu	1986 acet	CAH1_HUMAN Fu	6274 hist	2FW4	1KAH	1AZM		Sc=5.62816, min distance = 0.761788
HISX_ECOLI Fu	445794 AD	G3P_PALVE Full	5893 nadi	1DSS	1KAE	1IHY		Sc=6.12682, min distance = 1.097749
HISX_ECOLI Fu	5962 L-ly	ARGT_SALTY Fu	6274 hist	1LAG	1KAH	1LST		Sc=5.70923, min distance = 2.126015
HISX_ECOLI Fu	6022 Aden	Q5SI02_THET8 I	5893 nadi	2BJK	1KAE	2BJA		Sc=6.01807, min distance = 2.310662
HLDD_ECOLI Fu	11957393	FAK1_HUMAN Fu	6022 Aden	1MP8	1EQ2	2ETM		Sc=6.35053, min distance = 2.049052
HLDD_ECOLI Fu	23653518	CDK2_HUMAN Fu	6022 Aden	1GY3	1EQ2	2R3J		Sc=6.27438, min distance = 1.386322
HLDD_ECOLI Fu	24832037	PGK1_HUMAN Fu	6022 Aden	2ZGV	1EQ2	3C3C		Sc=5.87694, min distance = 1.677156
HLDD_ECOLI Fu	2608 1jsv	CDK2_HUMAN Fu	6022 Aden	1GY3	1EQ2	1JSV		Sc=6.01127, min distance = 2.727756
HLDD_ECOLI Fu	440317 AT	BCKD_RAT Full=	6022 Aden	1GKZ	1EQ2	1GJV	0.99	Sc=5.95772, min distance = 2.536110
HLDD_ECOLI Fu	444564 AD	MYS2_DICDI Fu	6022 Aden	1VOM	1EQ2	1W9I	0.91	Sc=5.95926, min distance = 2.209191
HLDD_ECOLI Fu	447955 1p	CDK2_HUMAN Fu	6022 Aden	1GY3	1EQ2	1PXI		Sc=5.73641, min distance = 2.128340
HLDD_ECOLI Fu	5327131 T	CDK2_HUMAN Fu	6022 Aden	1GY3	1EQ2	2C69		Sc=6.32546, min distance = 2.115816
HLDD_ECOLI Fu	5327133 C	CDK2_HUMAN Fu	6022 Aden	1GY3	1EQ2	2C6K		Sc=6.36957, min distance = 2.090642
HLDD_ECOLI Fu	5327148 C	IPKA_RABIT Fu	6022 Aden	1JBP	1EQ2	2ERZ		Sc=6.15585, min distance = 2.212786
HLDD_ECOLI Fu	5957 Aden	MALK_ECOLI Fu	6022 Aden	2AWN	1EQ2	1Q12	0.99	Sc=6.40253, min distance = 1.316275

HLDD_ECOLI F	6083 aden	PURP_METJA Fu	6022 Aden	2R7N	1EQ2	2R7M	0.99	Sc=6.33353, min distance = 2.38262
HLDD_ECOLI F	60961 ade	IPKA_RABIT Fu	6022 Aden	1JBP	1EQ2	1FMO	0.95	Sc=6.17772, min distance = 2.61945
HLDD_ECOLI F	6132 Cyti	ECX1_PYRAB Fu	6022 Aden	2PO0	1EQ2	2PO2		Sc=5.78566, min distance = 2.09955
HLDD_ECOLI F	6420139 C	CDK2_HUMAN Fu	6022 Aden	1GY3	1EQ2	2C5V		Sc=6.26679, min distance = 2.12392
HLDD_ECOLI F	6804 guar	NDK_PYRHO Ful	6022 Aden	2DYA	1EQ2	2DXF	0.8	Sc=6.36337, min distance = 2.35186
HLDD_ECOLI F	8977 1dar	O33839_THEMA	6022 Aden	1XJK	1EQ2	1XJE	0.8	Sc=5.96313, min distance = 1.90219
HLDD_ECOLI F	9547890 I	CDK2_HUMAN Fu	6022 Aden	1GY3	1EQ2	1W8C		Sc=6.20185, min distance = 2.60254
HLDD_ECOLI F	9994066 4	CDK2_HUMAN Fu	6022 Aden	1GY3	1EQ2	2VTJ		Sc=5.82781, min distance = 1.71328
HLYBP_ECOLX	11608401	KAPCA_BOVIN F	5957 Aden	1Q24	1XEF	2UVY		Sc=5.92263, min distance = 2.17965
HLYBP_ECOLX	16122608	PDPK1_HUMAN F	5957 Aden	2BIY	1XEF	2PE1		Sc=6.12271, min distance = 3.01157
HLYBP_ECOLX	6176 Cyti	Q381M1_9TRYP	5957 Aden	2Q0D	1XEF	2Q0C		Sc=6.32611, min distance = 1.92100
HLYBP_ECOLX	6830 guar	Q381M1_9TRYP	5957 Aden	2Q0D	1XEF	2Q0E	0.8	Sc=5.97799, min distance = 1.84686
HLYBP_ECOLX	91532 AMF	INSR_HUMAN Fu	5957 Aden	3BU5	1XEF	1I44	0.99	Sc=5.97446, min distance = 2.13725
HLYBP_ECOLX	91532 AMF	PURT_ECOLI Fu	5957 Aden	1KJ8	1XEF	1KJI	0.99	Sc=6.00681, min distance = 1.53830
HMDH_HUMAN F	24864080	CDK2_HUMAN Fu	6022 Aden	1GY3	1HW8	2VTR		Sc=6.02114, min distance = 1.38866
HMDH_HUMAN F	4369136 C	CDK2_HUMAN Fu	6022 Aden	1GY3	1HW8	1DM2		Sc=6.24289, min distance = 0.64786
HMDH_HUMAN F	447652 Im	CDK2_HUMAN Fu	6022 Aden	1GY3	1HW8	1OIQ		Sc=6.1787, min distance = 0.992962
HMDH_HUMAN F	6918710 5	CDK2_HUMAN Fu	6022 Aden	1GY3	1HW8	3EJ1		Sc=6.27239, min distance = 0.17323
HMDH_HUMAN F	8977 1dar	NDK_PYRHO Ful	6022 Aden	2DYA	1HW8	2DXE	0.8	Sc=5.65928, min distance = 2.19856
HMOX1_HUMAN	11957385	MYG_PHYCA Ful	444124 HE	1U7R	1OZW	2CMM		Sc=6.01364, min distance = 2.15576
HMOX1_RAT Fu	11957330	CCPR_YEAST Fu	444098 HE	2EUT	1DVG	1BEQ	0.81	Sc=6.40404, min distance = 2.47466
HMOX1_RAT Fu	11957353	HBA_HORSE Ful	444098 HE	2D5X	1DVG	1IWH	0.83	Sc=6.36291, min distance = 2.08673
HMOX1_RAT Fu	11957353	HBA_HORSE Ful	444124 HE	1Y8I	1J02	1IWH	0.87	Sc=6.38348, min distance = 2.01026
HMOX1_RAT Fu	11957353	HBB_HORSE Ful	444098 HE	2D5X	1DVG	1IWH	0.83	Sc=6.36215, min distance = 2.14271
HMOX1_RAT Fu	11957363	HBA_HUMAN Ful	444124 HE	1NQP	1J02	1RQA	0.81	Sc=6.38765, min distance = 2.17103
HMOX1_RAT Fu	11957363	HBB_HUMAN Ful	444098 HE	1J40	1DVG	1RQA	0.77	Sc=6.37093, min distance = 2.01954
HMOX1_RAT Fu	11957363	HBB_HUMAN Ful	444124 HE	1NQP	1J02	1RQA	0.81	Sc=6.68905, min distance = 2.01213
HMOX1_RAT Fu	11957385	MYG_PHYCA Ful	444098 HE	1A6M	1DVG	2CMM		Sc=6.17618, min distance = 2.09644
HMOX1_RAT Fu	11957385	MYG_PHYCA Ful	444124 HE	1U7R	1J02	2CMM		Sc=6.16957, min distance = 2.33076
HMOX1_RAT Fu	11970220	CCPR_YEAST Fu	444098 HE	2EUT	1DVG	1BEP	0.8	Sc=6.42317, min distance = 2.51832
HMOX1_RAT Fu	11970221	CCPR_YEAST Fu	444098 HE	2EUT	1DVG	1BES	0.83	Sc=6.37021, min distance = 2.54722
HMOX1_RAT Fu	11970242	CCPR_YEAST Fu	444098 HE	2EUT	1DVG	1CPE	0.83	Sc=6.40404, min distance = 2.36518
HMOX1_RAT Fu	126994 CI	CCPR_YEAST Fu	444098 HE	2EUT	1DVG	1Z53	0.77	Sc=6.37286, min distance = 2.54901
HMOX1_RAT Fu	444095 HE	RCEL_RHOVI Fu	444124 HE	3D38	1J02	1DXR	0.91	Sc=6.73075, min distance = 2.30780
HMOX1_RAT Fu	444207 He	HBA_HORSE Ful	444124 HE	1Y8I	1J02	2ZLW	0.99	Sc=6.68497, min distance = 2.29485
HMOX1_RAT Fu	444522 HE	Q83WG3_9ACTO	444124 HE	2Z3U	1J02	2Z3T	0.99	Sc=6.33465, min distance = 2.09043
HMOX2_HUMAN	11957330	CCPR_YEAST Fu	444098 HE	2EUT	2QPP	1BEQ	0.81	Sc=6.34281, min distance = 2.29286

HMOX2_HUMAN I	11957353	HBB_HORSE Ful	444124 HE	1Y8I	2RGZ	1IWH		0.87	Sc=6.60986, min distance = 2.16917
HMOX2_HUMAN I	11957363	HBB_HUMAN Ful	444124 HE	1NQP	2RGZ	1RQA		0.81	Sc=6.53189, min distance = 2.02364
HMOX2_HUMAN I	11957364	HMOX1_HUMAN Fu	444124 HE	1OZW	2RGZ	1S13	56.25	0.86	Sc=6.51942, min distance = 2.35896
HMOX2_HUMAN I	11957371	HMOX1_HUMAN Fu	444124 HE	1OZW	2RGZ	1TWR	56.25		Sc=6.50851, min distance = 2.21973
HMOX2_HUMAN I	11957385	MYG_PHYCA Ful	444098 HE	1A6M	2QPP	2CMM			Sc=6.13034, min distance = 1.90753
HMOX2_HUMAN I	11957385	MYG_PHYCA Ful	444124 HE	1U7R	2RGZ	2CMM			Sc=5.98736, min distance = 2.10140
HMOX2_HUMAN I	11970219	CCPR_YEAST Fu	444098 HE	2EUT	2QPP	1BEM		0.83	Sc=6.35011, min distance = 2.26303
HMOX2_HUMAN I	11970242	CCPR_YEAST Fu	444098 HE	2EUT	2QPP	1CPE		0.83	Sc=6.34344, min distance = 2.18553
HMOX2_HUMAN I	126994 CI	CCPR_YEAST Fu	444098 HE	2EUT	2QPP	1Z53		0.77	Sc=6.34634, min distance = 2.53149
HMOX2_HUMAN I	444522 HE	Q83WG3_9ACTO I	444124 HE	2Z3U	2RGZ	2Z3T		0.99	Sc=6.25895, min distance = 2.27439
HMP_ECOLI Fu	11957353	HBB_HORSE Ful	444124 HE	1Y8I	1GVH	1IWH		0.87	Sc=6.76704, min distance = 2.13712
HMP_ECOLI Fu	11957370	HMOX1_HUMAN Fu	444124 HE	1OZW	1GVH	1TWN			Sc=6.5889, min distance = 2.313562
HMP_ECOLI Fu	11957371	HMOX1_HUMAN Fu	444124 HE	1OZW	1GVH	1TWR			Sc=6.60453, min distance = 2.63566
HMP_ECOLI Fu	11957385	MYG_PHYCA Ful	444124 HE	1U7R	1GVH	2CMM			Sc=6.23763, min distance = 2.57966
HMP_ECOLI Fu	16214774	CP51_MYCTU Fu	444124 HE	2CIB	1GVH	2CI0		0.95	Sc=6.54378, min distance = 2.36322
HMP_ECOLI Fu	39042 bez	HBA_HORSE Ful	444124 HE	1Y8I	1GVH	1IWH			Sc=6.2072, min distance = 2.085776
HMP_ECOLI Fu	444207 He	HBB_HORSE Ful	444124 HE	1Y8I	1GVH	2ZLW		0.99	Sc=6.7615, min distance = 2.196728
HMP_ECOLI Fu	444207 He	PER_COPCI Ful	444124 HE	1LY9	1GVH	1LY8		0.99	Sc=6.75738, min distance = 1.99260
HMP_ECOLI Fu	444522 HE	BFR_ECOLI Ful	444124 HE	1BFR	1GVH	1BCF		0.99	Sc=6.77045, min distance = 2.29871
HMP_ECOLI Fu	444522 HE	HBA_PAGBE Ful	444124 HE	1S5X	1GVH	1PBX		0.99	Sc=6.76391, min distance = 2.45354
HMP_ECOLI Fu	446189 CI	HBG1_HUMAN Fu	444124 HE	1I3E	1GVH	1I3D		0.89	Sc=6.76524, min distance = 2.03971
HMP_ECOLI Fu	446332 CI	MYG_PHYCA Ful	444124 HE	1U7R	1GVH	1IOP		0.8	Sc=6.69826, min distance = 2.20102
HMP_RALEH Fu	11957371	HMOX1_HUMAN Fu	444124 HE	1OZW	1CQX	1TWR			Sc=6.32056, min distance = 2.28163
HMUO_CORDI Fu	11957385	MYG_PHYCA Ful	444124 HE	1U7R	1IW1	2CMM			Sc=6.15698, min distance = 2.34410
HMUO_CORDI Fu	444095 HE	RCEL_RHOVI Fu	444124 HE	3D38	1IW1	1DXR		0.91	Sc=6.34763, min distance = 2.15602
HMUO_CORDI Fu	6420167 M	CPXA_PSEPU Fu	444124 HE	1RE9	1IW1	2FE6		0.89	Sc=6.41798, min distance = 2.24556
HNF4G_HUMAN I	14253 Cet	FABP4_MOUSE Fu	985 palmi	1LIE	1LV2	1LIC			Sc=5.66679, min distance = 2.38927
HNF4G_HUMAN I	14368760	LPXC_AQUAE Fu	985 palmi	2GO3	1LV2	2O3Z			Sc=5.95231, min distance = 2.05067
HNL_HEVBR Fu	165271 L-	HISX_ECOLI Fu	6274 hist	1KAH	1YAS	1KAE		0.89	Sc=5.62455, min distance = 1.65118
HNL_HEVBR Fu	6322 L-ar	ARGT_SALTY Fu	6274 hist	1LAG	1YAS	1LAF			Sc=5.73598, min distance = 2.19725
HNMT_HUMAN Fu	445241 CI	ACES_TORCA Fu	444211 th	1ACJ	2AOW	1E66			Sc=6.07897, min distance = 2.03804
HNMT_HUMAN Fu	60961 ade	PIMT_PYRFU Fu	439155 Ad	1JG1	2AOT	1JG2		0.84	Sc=6.24827, min distance = 2.36613
HNMT_HUMAN Fu	65482 sir	MTR1_RHOSH Fu	439155 Ad	1NW7	2AOT	1NW6		0.88	Sc=5.76594, min distance = 2.20990
HN_NDVB Full	449201 1x	NRAM_I75A5 Fu	65309 DAN	2QWC	1USX	1XOG			Sc=6.21184, min distance = 2.43937
HN_NDVB Full	60855 zar	NRAM_I75A5 Fu	65309 DAN	2QWC	1USX	1NNC		0.88	Sc=5.92183, min distance = 2.35533
HN_NDVB Full	60855 zar	Q6WJ03_9PARA I	65309 DAN	1V3D	1USX	1V3E		0.88	Sc=5.61549, min distance = 1.93485
HN_NDVK Full	439353 be	HA33_CLOBO Fu	445063 La	2EHI	1E8U	2EHN			Sc=5.70531, min distance = 2.59741

HN_NDVK Full:	60855 zar	NRAM_I75A5 Fu.	65309 DAN	2QWC	1E8V	1NNC		0.88	Sc=5.6207, min distance = 2.678951
HO2_SYNY3 Fu.	11957385	MYG_PHYCA Full	444124 HE	1U7R	1WOX	2CMM			Sc=6.1963, min distance = 1.999963
HO2_SYNY3 Fu.	444207 He	CP119_SULTO Fu	444124 HE	3B4X	1WOX	1UE8		0.99	Sc=6.72667, min distance = 2.087314
HO2_SYNY3 Fu.	444207 He	HBB_HORSE Full	444124 HE	1Y8I	1WOX	2ZLW		0.99	Sc=6.74553, min distance = 1.640179
HO2_SYNY3 Fu.	444522 HE	NOS3_BOVIN Fu.	444124 HE	1ZZS	1WOX	2HX2		0.99	Sc=6.75325, min distance = 1.504856
HO2_SYNY3 Fu.	444668 CI	MYG_PHYCA Full	444124 HE	1U7R	1WOX	1BVD		0.84	Sc=6.79846, min distance = 1.899725
HO2_SYNY3 Fu.	5326592 C	HMOX1_RAT Full	444124 HE	1J02	1WOX	1J2C	43.66	0.84	Sc=6.68224, min distance = 2.533155
HOME2_MOUSE I	16750041	PYRB_ECOLI Fu.	311 citri	1R0B	1I7A	2IPO			Sc=5.81801, min distance = 2.591696
HOME2_MOUSE I	448573 FI	CASP7_HUMAN Fu	311 citri	2QL9	1I7A	1SHL			Sc=5.95457, min distance = 1.516099
HOME2_MOUSE I	51 2-Oxop	SERA_ECOLI Fu.	311 citri	2P9E	1I7A	1YBA		0.76	Sc=5.62282, min distance = 1.465380
HPPK_ECOLI Fu	447377 1r	SYP_RHOPA Full	5957 Aden	2I4O	1DY3	2I4N		0.84	Sc=6.00103, min distance = 2.108138
HPRT_ECOLI Fu	100252 Ri	IMDH_TRIFO Fu.	8582 Inos	1MEH	1G9S	1ME8			Sc=6.1902, min distance = 2.0596497
HPRT_ECOLI Fu	24316 cyc	PDE10_HUMAN Fu	6804 guan	2OUQ	1G9T	2OUU		0.97	Sc=5.80176, min distance = 0
HPRT_ECOLI Fu	3758 IBMX	PDE5A_HUMAN Fu	6804 guan	1T9S	1G9T	1RKP			Sc=5.97503, min distance = 2.049386
HPRT_ECOLI Fu	444699 1b	HPRT_HUMAN Fu.	6804 guan	1HMP	1G9T	1BZY			Sc=6.43151, min distance = 2.085265
HPRT_ECOLI Fu	6030 Uric	PYRR_BACCL Fu.	6804 guan	1XZ8	1G9T	2IGB			Sc=6.202, min distance = 2.0122688
HPRT_ECOLI Fu	65110 AIC	PURO_METTH Fu.	8582 Inos	2NTK	1G9S	2NTL		0.97	Sc=6.19408, min distance = 1.592382
HPRT_ECOLI Fu	73323 Xar	IMDH_TRIFO Fu.	8582 Inos	1MEH	1G9S	1MEW		0.94	Sc=5.87669, min distance = 2.074130
HPRT_HUMAN Fu	168120 Po	HINT1_RABIT Fu	6804 guan	3RHN	1HMP	5RHN		0.78	Sc=6.36432, min distance = 2.080641
HPRT_HUMAN Fu	6030 Uric	PYRR_BACCL Fu.	6804 guan	1XZ8	1HMP	2IGB			Sc=6.27197, min distance = 2.009540
HPRT_HUMAN Fu	73323 Xar	HGXR_TOXGO Fu.	6804 guan	1QK3	1HMP	1QK5	41.55	0.95	Sc=6.41448, min distance = 1.857951
HPRT_HUMAN Fu	8582 Inos	HGXR_TOXGO Fu.	6804 guan	1QK3	1HMP	1QK4	41.55	0.99	Sc=6.46307, min distance = 0.829865
HPRT_HUMAN Fu	8582 Inos	HGXR_TOXGO Fu.	7339 PRPP	1FSG	1D6N	1QK4	41.55		Sc=6.36522, min distance = 2.085842
HPRT_HUMAN Fu	8582 Inos	HPRT_ECOLI Fu.	6804 guan	1G9T	1HMP	1G9S		0.99	Sc=6.36337, min distance = 2.468887
HPRT_HUMAN Fu	8582 Inos	Q4DRC4_TRYCR I	7339 PRPP	1I0L	1D6N	1P19	32.46		Sc=6.42271, min distance = 1.381111
HPRT_HUMAN Fu	8582 Inos	Q5SLS3_THET8 I	6804 guan	2YWT	1HMP	2YWU	35.48	0.99	Sc=6.35774, min distance = 2.506592
HPR_BACSU Fu.	180621 Me	UROK_HUMAN Fu.	62551 Pen	2O8T	2FXA	2VIN			Sc=5.81001, min distance = 0.781831
HQGT_ARATH Fu	6030 Uric	O87988_BORBR I	6031 Urid	2PZM	2VCH	2PZL		0.99	Sc=6.32307, min distance = 2.365140
HQGT_ARATH Fu	6030 Uric	PYRH_ECOLI Fu.	6031 Urid	2BND	2VCH	2BNE		0.99	Sc=6.32492, min distance = 2.144591
HQGT_ARATH Fu	8629 UDP-	GSTA_BPT4 Full	6031 Urid	1XV5	2VCH	1Y6F		0.89	Sc=6.65859, min distance = 2.260471
HQGT_ARATH Fu	8629 UDP-	Q5IFH7_MEDTR I	6031 Urid	2ACV	2VCH	2ACW		0.89	Sc=6.67116, min distance = 2.198264
HS3S5_HUMAN I	449392 1w	RNAS1_BOVIN Fu	159296 1v	1O0F	3BD9	1W4P			Sc=6.03908, min distance = 2.020783
HS3SA_HUMAN I	15342951	ALBU_HUMAN Fu.	311 citri	1TF0	1T8T	3B9L			Sc=6.07042, min distance = 2.036179
HS3SA_HUMAN I	24180722	FGFR1_HUMAN Fu	445701 CI	1FQ9	1T8U	3C4F			Sc=5.90571, min distance = 1.481223
HS3SB_HUMAN I	15342951	ALBU_HUMAN Fu.	311 citri	1TF0	1T8T	3B9L			Sc=6.07042, min distance = 2.036179
HS3SB_HUMAN I	24180722	FGFR1_HUMAN Fu	445701 CI	1FQ9	1T8U	3C4F			Sc=5.90571, min distance = 1.481223
HS90A_HUMAN I	11963552	HSP82_YEAST Fu	6082103 2	2BRC	2BT0	2IWU	71.36		Sc=6.21962, min distance = 2.314131

HS90A_HUMAN F	398148 1e	CDK2_HUMAN Fu	6022 Aden	1GY3	1BYQ	1E1X			Sc=6.14531, min distance = 2.063196
HS90A_HUMAN F	445966 O6	CDK2_HUMAN Fu	6022 Aden	1GY3	1BYQ	1H0V			Sc=6.1422, min distance = 2.0742258
HS90A_HUMAN F	446090 1h	NDKC_DICDI Fu	6022 Aden	1KDN	1BYQ	1HIY		0.97	Sc=5.94395, min distance = 1.592168
HS90A_HUMAN F	447955 1p	CDK2_HUMAN Fu	6022 Aden	1GY3	1BYQ	1PXI			Sc=5.63155, min distance = 2.329375
HS90A_HUMAN F	6031 Uric	DCK_HUMAN Ful	6022 Aden	1P5Z	1BYQ	2ZIA			Sc=5.76337, min distance = 2.137469
HS90A_HUMAN F	6031 Uric	O33839_THEMA F	6022 Aden	1XJK	1BYQ	1XJG			Sc=5.80502, min distance = 1.699140
HS90A_HUMAN F	6083 ader	PSPF_ECOLI Fu	6022 Aden	2C98	1BYQ	2VII		0.99	Sc=5.88517, min distance = 2.227657
HS90A_HUMAN F	6102757 K	HSP82_YEAST F	6082103 2	2BRC	2BT0	2BRE	71.36	0.82	Sc=6.3641, min distance = 2.5674006
HS90A_HUMAN F	6323491 M	HSP82_YEAST F	6082103 2	2BRC	2BT0	1BGQ	71.36		Sc=6.30745, min distance = 0.924577
HS90A_HUMAN F	6323491 M	PDK3_HUMAN Fu	6022 Aden	1Y8O	1BYQ	2Q8I			Sc=6.29032, min distance = 2.700407
HS90A_HUMAN F	6323491 M	TOP6B_SULSH F	6022 Aden	1Z5B	1BYQ	2HKJ			Sc=6.32186, min distance = 2.177506
HS90A_HUMAN F	6804 guar	NDK_PYRHO Ful	6022 Aden	2DYA	1BYQ	2DXF		0.8	Sc=5.86007, min distance = 2.205148
HS90A_HUMAN F	72194 2-C	ENPL_CANFA Fu	6022 Aden	1TC6	1BYQ	1QYE	53.62	0.89	Sc=5.99526, min distance = 2.842116
HS90B_HUMAN F	10403821	HS90A_HUMAN F	448965 1u	1UY6	1UYM	3BM9			Sc=6.12469, min distance = 2.301627
HS90B_HUMAN F	13373715	HS90A_HUMAN F	448965 1u	1UY6	1UYM	2QF6			Sc=6.09574, min distance = 2.754725
HS90B_HUMAN F	16058668	HS90A_HUMAN F	448965 1u	1UY6	1UYM	2UWD			Sc=6.36276, min distance = 2.413500
HS90B_HUMAN F	24812721	HS90A_HUMAN F	448965 1u	1UY6	1UYM	3D0B			Sc=6.36633, min distance = 2.646635
HS90B_HUMAN F	24832021	HS90A_HUMAN F	448965 1u	1UY6	1UYM	2QG0			Sc=6.34427, min distance = 2.422046
HS90B_HUMAN F	24832022	HS90A_HUMAN F	448965 1u	1UY6	1UYM	2QG2			Sc=6.27257, min distance = 2.584486
HS90B_HUMAN F	24836816	HS90A_HUMAN F	448965 1u	1UY6	1UYM	3BMY			Sc=6.18495, min distance = 2.451581
HS90B_HUMAN F	448968 1u	HS90A_HUMAN F	448965 1u	1UY6	1UYM	1UY9		0.92	Sc=6.24785, min distance = 2.861657
HS90B_HUMAN F	448970 1u	HS90A_HUMAN F	448965 1u	1UY6	1UYM	1UYD		0.91	Sc=6.32623, min distance = 2.600200
HS90B_HUMAN F	448971 1u	HS90A_HUMAN F	448965 1u	1UY6	1UYM	1UYE		0.9	Sc=6.34098, min distance = 2.579032
HS90B_HUMAN F	448972 1u	HS90A_HUMAN F	448965 1u	1UY6	1UYM	1UYF		0.88	Sc=6.35423, min distance = 2.643050
HS90B_HUMAN F	448973 1u	HS90A_HUMAN F	448965 1u	1UY6	1UYM	1UYG		0.81	Sc=6.27225, min distance = 2.617420
HS90B_HUMAN F	5327095 V	HS90A_HUMAN F	448965 1u	1UY6	1UYM	2BSM			Sc=6.41773, min distance = 2.446208
HS90B_HUMAN F	5496922 C	HS90A_HUMAN F	448965 1u	1UY6	1UYM	1UY7		0.89	Sc=6.20874, min distance = 2.805721
HS90B_HUMAN F	5496923 C	HS90A_HUMAN F	448965 1u	1UY6	1UYM	1UY8		0.91	Sc=6.22514, min distance = 2.901521
HS90B_HUMAN F	6082103 2	HS90A_HUMAN F	448965 1u	1UY6	1UYM	2BT0			Sc=6.30515, min distance = 2.409891
HS90B_HUMAN F	6102788 2	HS90A_HUMAN F	448965 1u	1UY6	1UYM	2CCT			Sc=6.40369, min distance = 2.511321
HS90B_HUMAN F	9549213 2	HS90A_HUMAN F	448965 1u	1UY6	1UYM	2FWZ			Sc=6.27648, min distance = 3.060004
HS90B_HUMAN F	9549229 C	HS90A_HUMAN F	448965 1u	1UY6	1UYM	2H55		0.82	Sc=6.30789, min distance = 2.904612
HSD_MYCTU Fu	169266 1,	GALE_HUMAN Fu	5893 nadi	1HZJ	1NFF	1I3K		0.79	Sc=6.79153, min distance = 2.131647
HSD_MYCTU Fu	439153 Di	Q9BJJ9_PLAFA F	5893 nadi	1UH5	1NFF	1V35		0.79	Sc=6.78277, min distance = 2.279374
HSD_STREX Fu	169266 1,	GALE_HUMAN Fu	5893 nadi	1HZJ	2HSD	1I3K		0.79	Sc=6.76226, min distance = 2.683884
HSD_STREX Fu	439153 Di	GALE_ECOLI Fu	5893 nadi	1UDC	2HSD	1UDB		0.79	Sc=6.75642, min distance = 2.551186
HSD_STREX Fu	9547960 A	NPD_THEMA Ful	5893 nadi	2H4H	2HSD	2H59			Sc=6.57657, min distance = 1.978641

HSLU_ECOLI F	16750062	CSK2A_MAIZE F	33113 gam	1LP4	1E94	2OXD			Sc=5.69771, min distance = 1.96382
HSLU_ECOLI F	23653516	CDK2_HUMAN Fu	6022 Aden	1GY3	1HQY	2R3G			Sc=6.27533, min distance = 1.61520
HSLU_ECOLI F	24779675	CSK2A_MAIZE F	33113 gam	1LP4	1E94	2PVJ			Sc=6.38709, min distance = 2.00036
HSLU_ECOLI F	24864080	CDK2_HUMAN Fu	6022 Aden	1GY3	1HQY	2VTR			Sc=6.02268, min distance = 2.03566
HSLU_ECOLI F	3540 1yds	KAPCA_BOVIN F	5957 Aden	1Q24	1DO0	1YDS			Sc=5.87585, min distance = 1.86288
HSLU_ECOLI F	3547 Fasu	KAPCA_BOVIN F	5957 Aden	1Q24	1DO0	1Q8W			Sc=5.96713, min distance = 2.04649
HSLU_ECOLI F	3547 Fasu	ROCK1_HUMAN F	33113 gam	2V55	1E94	2ESM			Sc=5.94346, min distance = 2.16774
HSLU_ECOLI F	444564 AD	MYS2_DICDI Fu	6022 Aden	1VOM	1HQY	1W9I		0.91	Sc=5.97258, min distance = 1.83305
HSLU_ECOLI F	444852 CI	ATPB_BOVIN Fu	6022 Aden	2CK3	1HQY	1COW		0.9	Sc=6.01735, min distance = 1.82858
HSLU_ECOLI F	445966 O6	CDK2_HUMAN Fu	6022 Aden	1GY3	1HQY	1H0V			Sc=6.15372, min distance = 2.30422
HSLU_ECOLI F	449240 1y	KAPCA_BOVIN F	5957 Aden	1Q24	1DO0	1YDR			Sc=5.95366, min distance = 1.89369
HSLU_ECOLI F	4564 1e1v	CDK2_HUMAN Fu	6022 Aden	1GY3	1HQY	1E1V			Sc=6.15251, min distance = 2.55540
HSLU_ECOLI F	4565 1h1r	CDK2_HUMAN Fu	6022 Aden	1GY3	1HQY	1H1R			Sc=6.37881, min distance = 1.66463
HSLU_ECOLI F	5327148 C	IPKA_RABIT Fu	33113 gam	1CDK	1E94	2ERZ			Sc=6.1937, min distance = 2.117664
HSLU_ECOLI F	6031 Uric	DCK_HUMAN Ful	6022 Aden	1P5Z	1HQY	2ZIA			Sc=5.8688, min distance = 2.526039
HSLU_ECOLI F	6083 ader	PSPF_ECOLI Fu	5957 Aden	2C96	1DO0	2VII		0.98	Sc=6.32433, min distance = 2.37804
HSLU_ECOLI F	60961 ade	SKY1_YEAST Fu	33113 gam	1Q99	1E94	1Q97		0.93	Sc=6.16452, min distance = 2.68779
HSLU_ECOLI F	6102784 A	PSPF_ECOLI Fu	6022 Aden	2C98	1HQY	2C99		0.99	Sc=6.0229, min distance = 1.940765
HSLU_ECOLI F	6338561 C	MYS2_DICDI Fu	33113 gam	1MMN	1E94	1D0X			Sc=6.24209, min distance = 2.11588
HSLU_ECOLI F	6338562 C	MYS2_DICDI Fu	5957 Aden	1FMW	1DO0	1D0Y			Sc=6.21966, min distance = 0
HSLU_ECOLI F	6338563 C	MYS2_DICDI Fu	5957 Aden	1FMW	1DO0	1D0Z			Sc=6.25131, min distance = 0
HSLU_ECOLI F	657138 1z	PDPK1_HUMAN F	5957 Aden	2BIY	1DO0	1Z5M			Sc=6.69701, min distance = 1.95933
HSLU_ECOLI F	6852207 M	KAPCA_BOVIN F	5957 Aden	1Q24	1DO0	2GNI			Sc=5.93159, min distance = 1.89387
HSLU_ECOLI F	8582 Inos	RNAS1_BOVIN F	6022 Aden	1O0H	1HQY	1Z6D		0.79	Sc=6.35869, min distance = 2.11067
HSLU_ECOLI F	91532 AMP	BIOD_ECOLI Fu	6022 Aden	1DAD	1HQY	1DAG		0.99	Sc=6.04166, min distance = 1.91407
HSLU_ECOLI F	91532 AMP	F263_HUMAN Fu	6022 Aden	2AXN	1HQY	2DWP		0.99	Sc=5.764, min distance = 2.0893463
HSLU_ECOLI F	9547890 I	CDK2_HUMAN Fu	6022 Aden	1GY3	1HQY	1W8C			Sc=6.17175, min distance = 1.80165
HSLU_HAEIN F	23653515	CDK2_HUMAN Fu	6022 Aden	1GY3	1G41	2R3F			Sc=6.09002, min distance = 1.97539
HSLU_HAEIN F	23653518	CDK2_HUMAN Fu	6022 Aden	1GY3	1G41	2R3J			Sc=6.28446, min distance = 1.92629
HSLU_HAEIN F	23653519	CDK2_HUMAN Fu	6022 Aden	1GY3	1G41	2R3K			Sc=6.16667, min distance = 1.95198
HSLU_HAEIN F	23653520	CDK2_HUMAN Fu	6022 Aden	1GY3	1G41	2R3L			Sc=6.28165, min distance = 2.01549
HSLU_HAEIN F	23653526	CDK2_HUMAN Fu	6022 Aden	1GY3	1G41	2R3R			Sc=6.29397, min distance = 1.80923
HSLU_HAEIN F	24864080	CDK2_HUMAN Fu	6022 Aden	1GY3	1G41	2VTR			Sc=6.01127, min distance = 1.65172
HSLU_HAEIN F	444564 AD	BIOD_ECOLI Fu	6022 Aden	1DAD	1G41	1BS1		0.91	Sc=6.41877, min distance = 2.20850
HSLU_HAEIN F	445940 O6	CDK2_HUMAN Fu	6022 Aden	1GY3	1G41	1GZ8			Sc=6.12546, min distance = 2.10868
HSLU_HAEIN F	4564 1e1v	CDK2_HUMAN Fu	6022 Aden	1GY3	1G41	1E1V			Sc=6.1479, min distance = 2.368990
HSLU_HAEIN F	5327130 C	CDK2_HUMAN Fu	6022 Aden	1GY3	1G41	2C68			Sc=6.17415, min distance = 1.72112

HSLU_HAEIN Fı	5957 Aden	Q72H90_THET2 I	6022 Aden	2BEJ	1G41	2BEK		0.99	Sc=6.0282, min distance = 2.000590(
HSLU_HAEIN Fı	5957 Aden	RK_BOVIN Full·	6022 Aden	3C4Z	1G41	3C4W		0.99	Sc=6.45435, min distance = 1.791198
HSLU_HAEIN Fı	6031 Uric	DCK_HUMAN Full	6022 Aden	1P5Z	1G41	2ZIA			Sc=5.87543, min distance = 2.47098(
HSLU_HAEIN Fı	6852201 2	CDK2_HUMAN Fu	6022 Aden	1GY3	1G41	2G9X			Sc=6.55606, min distance = 2.08726
HSLU_HAEIN Fı	8582 Inos	RNAS1_BOVIN Fı	6022 Aden	1O0H	1G41	1Z6D		0.79	Sc=6.36755, min distance = 2.11236!
HSLU_HAEIN Fı	91532 AMI	BIOD_ECOLI Fu	6022 Aden	1DAD	1G41	1DAG		0.99	Sc=6.03112, min distance = 2.06275
HSLU_HAEIN Fı	9547890 I	CDK2_HUMAN Fu	6022 Aden	1GY3	1G41	1W8C			Sc=6.17435, min distance = 1.82889
HSP71_HUMAN I	15993 dAT	PRIM_BPT7 Full	33113 gam	1E0J	2E8A	1CR2		0.97	Sc=6.51347, min distance = 2.02813
HSP71_HUMAN I	15993 dAT	RECA_MYCS2 Fu	6022 Aden	2ZRO	1S3X	2ZR9		0.97	Sc=6.50935, min distance = 2.22071
HSP71_HUMAN I	24752834	SYH_ECOLI Full	6083 aden	1HTT	1XQS	2EL9			Sc=6.62583, min distance = 1.53613
HSP71_HUMAN I	2519 caff	PYGM_RABIT Fu	6083 aden	8GPB	1XQS	1GFZ			Sc=5.86897, min distance = 2.00854
HSP71_HUMAN I	36735 Gpp	PARM_ECOLX Fu	6022 Aden	1MWM	1S3X	2ZGZ		0.79	Sc=6.52714, min distance = 1.83699
HSP71_HUMAN I	445321 CI	NADE_BACSU Fu	6083 aden	2NSY	1XQS	1EE1		0.85	Sc=5.66972, min distance = 1.91222
HSP71_HUMAN I	445966 O6	CDK2_HUMAN Fu	6022 Aden	1GY3	1S3X	1H0V			Sc=6.31053, min distance = 2.22924!
HSP71_HUMAN I	446090 1h	NDKC_DICDI Fu	6022 Aden	1KDN	1S3X	1HIY		0.97	Sc=6.4803, min distance = 1.310481!
HSP71_HUMAN I	447832 AI	ACSA_SALTY Fu	6083 aden	2P2F	1XQS	1PG4		0.99	Sc=6.44444, min distance = 2.10372(
HSP71_HUMAN I	447955 1p	CDK2_HUMAN Fu	6022 Aden	1GY3	1S3X	1PXI			Sc=5.83292, min distance = 2.19770!
HSP71_HUMAN I	448895 AI	UMPK_YEAST Fu	6083 aden	1UKZ	1XQS	1UKY		0.99	Sc=5.62593, min distance = 2.33597!
HSP71_HUMAN I	6031 Uric	DCK_HUMAN Full	6022 Aden	1P5Z	1S3X	2ZIA			Sc=6.3659, min distance = 1.978775¦
HSP71_HUMAN I	60961 ade	Q84CU3_THETH I	6083 aden	1V8S	1XQS	1V8Y		0.96	Sc=6.31367, min distance = 2.01078
HSP71_HUMAN I	65110 AIC	YL28_SCHPO Fu	6083 aden	2OOX	1XQS	2QRE		0.79	Sc=6.32606, min distance = 2.15138(
HSP71_HUMAN I	656966 1y	PDE4D_HUMAN Fı	6083 aden	1TB7	1XQS	1Y2E			Sc=6.11816, min distance = 1.30076
HSP71_HUMAN I	657135 Op	PIM1_HUMAN Fu	6083 aden	1YXU	1XQS	1YXV			Sc=5.93424, min distance = 2.17370!
HSP71_HUMAN I	6804 guar	NDK_PYRHO Full	6022 Aden	2DYA	1S3X	2DXF		0.8	Sc=6.43599, min distance = 2.12560
HSP71_HUMAN I	6804 guar	Q9SSV1_NICGU I	6083 aden	1VD1	1XQS	1VCZ		0.8	Sc=5.61771, min distance = 2.04412
HSP71_HUMAN I	8582 Inos	PYGM_RABIT Fu	6083 aden	8GPB	1XQS	2QN7		0.79	Sc=5.89808, min distance = 2.12168(
HSP71_HUMAN I	8977 1dar	PARM_ECOLX Fu	6022 Aden	1MWM	1S3X	2ZGY		0.8	Sc=6.49293, min distance = 1.94952
HSP71_HUMAN I	91532 AMI	NIFH1_AZOVI Fı	6022 Aden	1FP6	1S3X	2AFK		0.99	Sc=6.54071, min distance = 1.52452
HSP71_HUMAN I	91532 AMI	PURP_METJA Fu	6022 Aden	2R7N	1S3X	2R7K		0.99	Sc=6.54922, min distance = 2.13137!
HSP71_HUMAN I	91557 1tm	BLS_STRCL Full	6083 aden	1MC1	1XQS	1JGT		0.97	Sc=5.72201, min distance = 1.24737¦
HSP71_HUMAN I	9547921 G	SYQ_ECOLI Full	6083 aden	1ZJW	1XQS	2RE8		0.82	Sc=6.00602, min distance = 2.62329!
HSP7C_BOVIN I	3540 1yds	KAPCA_BOVIN Fı	5957 Aden	1Q24	2BUP	1YDS			Sc=5.94793, min distance = 2.01340
HSP7C_BOVIN I	36735 Gpp	PARM_ECOLX Fu	6022 Aden	1MWM	1BUP	2ZGZ		0.79	Sc=6.54289, min distance = 1.93901(
HSP7C_BOVIN I	444564 AI	BIOD_ECOLI Fu	6022 Aden	1DAD	1BUP	1BS1		0.91	Sc=6.45985, min distance = 2.17181
HSP7C_BOVIN I	444842 CI	CDK2_HUMAN Fu	6022 Aden	1GY3	1BUP	1CKP			Sc=5.62651, min distance = 2.33156
HSP7C_BOVIN I	444852 CI	ATPB_BOVIN Fu	5957 Aden	2V7Q	2BUP	1COW		0.91	Sc=6.52755, min distance = 2.00131!
HSP7C_BOVIN I	445966 O6	CDK2_HUMAN Fu	6022 Aden	1GY3	1BUP	1H0V			Sc=6.32163, min distance = 2.07055

HSP7C_BOVIN	447955 1p	CDK2_HUMAN Fu	6022 Aden	1GY3	1BUP	1PXI			Sc=5.84028, min distance = 2.14110
HSP7C_BOVIN	449240 1y	KAPCA_BOVIN F	5957 Aden	1Q24	2BUP	1YDR			Sc=6.02048, min distance = 2.22637
HSP7C_BOVIN	6031 Uric	DCK_HUMAN Ful	6022 Aden	1P5Z	1BUP	2ZIA			Sc=6.37681, min distance = 2.17133
HSP7C_BOVIN	6083 ader	ENTP2_RAT Ful	33113 gam	3CJA	1NGJ	3CJ7		0.97	Sc=6.41554, min distance = 2.13946
HSP7C_BOVIN	6083 ader	PSPF_ECOLI Fu	5957 Aden	2C96	2BUP	2VII		0.98	Sc=6.39311, min distance = 2.16845
HSP7C_BOVIN	6083 ader	PURP_METJA Fu	5957 Aden	2R7L	2BUP	2R7M		0.98	Sc=6.42465, min distance = 2.01200
HSP7C_BOVIN	6083 ader	PURP_METJA Fu	6022 Aden	2R7N	1BUP	2R7M		0.99	Sc=6.4078, min distance = 2.164928
HSP7C_BOVIN	6804 guar	NDK_PYRHO Ful	6022 Aden	2DYA	1BUP	2DXF		0.8	Sc=6.44071, min distance = 2.17830
HSP7C_BOVIN	6852187 2	O57883_PYRHO	6022 Aden	1WNL	1BUP	2DTH		0.89	Sc=6.19015, min distance = 1.84410
HSP7C_BOVIN	8977 1dar	PARM_ECOLX Fu	6022 Aden	1MWM	1BUP	2ZGY		0.8	Sc=6.50046, min distance = 2.46588
HSP7C_BOVIN	91532 AMP	BIOD_ECOLI Fu	5957 Aden	1A82	2BUP	1DAG		0.99	Sc=6.53652, min distance = 2.18867
HSP7C_BOVIN	91532 AMP	PURP_METJA Fu	6022 Aden	2R7N	1BUP	2R7K		0.99	Sc=6.55619, min distance = 2.13882
HSP7F_YEAST	36735 Gpp	PARM_ECOLX Fu	6022 Aden	1MWM	3C7N	2ZGZ		0.79	Sc=6.52714, min distance = 2.31759
HSP7F_YEAST	445966 O6	CDK2_HUMAN Fu	6022 Aden	1GY3	3C7N	1H0V			Sc=6.31131, min distance = 1.83067
HSP7F_YEAST	447916 ac	RIO1_ARCFU Fu	6022 Aden	1ZTH	3C7N	1ZTF		0.95	Sc=6.2974, min distance = 2.094422
HSP7F_YEAST	447955 1p	CDK2_HUMAN Fu	6022 Aden	1GY3	3C7N	1PXI			Sc=5.8094, min distance = 1.918571
HSP7F_YEAST	449240 1y	KAPCA_BOVIN F	5957 Aden	1Q24	3D2F	1YDR			Sc=6.00204, min distance = 2.06412
HSP7F_YEAST	6031 Uric	DCK_HUMAN Ful	6022 Aden	1P5Z	3C7N	2ZIA			Sc=6.35746, min distance = 2.14879
HSP7F_YEAST	6083 ader	PSPF_ECOLI Fu	6022 Aden	2C98	3C7N	2VII		0.99	Sc=6.404, min distance = 2.078099
HSP7F_YEAST	6083 ader	PURP_METJA Fu	6022 Aden	2R7N	3C7N	2R7M		0.99	Sc=6.39311, min distance = 2.02240
HSP7F_YEAST	60961 ade	IPKA_RABIT Fu	6022 Aden	1JBP	3C7N	1FMO		0.95	Sc=6.29223, min distance = 2.13655
HSP7F_YEAST	6918710 5	CDK2_HUMAN Fu	6022 Aden	1GY3	3C7N	3EJ1			Sc=6.2773, min distance = 1.555471
HSP7F_YEAST	8977 1dar	NDK_PYRHO Ful	6022 Aden	2DYA	3C7N	2DXE		0.8	Sc=6.49442, min distance = 2.13742
HSP82_YEAST	10403821	HS90A_HUMAN F	6022 Aden	1BYQ	1AMW	3BM9	71.36		Sc=6.07738, min distance = 2.43939
HSP82_YEAST	10403821	HS90A_HUMAN F	6082103 2	2BT0	2BRC	3BM9	71.36		Sc=6.09434, min distance = 2.50351
HSP82_YEAST	10403821	HS90A_HUMAN F	6914564 2	2FWY	1ZW9	3BM9	71.36		Sc=6.06692, min distance = 2.42435
HSP82_YEAST	13373715	HS90A_HUMAN F	6082103 2	2BT0	2BRC	2QF6	71.36		Sc=6.1656, min distance = 2.405023
HSP82_YEAST	164628 de	PMS2_HUMAN Fu	6022 Aden	1EA6	1AMW	1H7U			Sc=5.96458, min distance = 1.21015
HSP82_YEAST	24812721	HS90A_HUMAN F	6914564 2	2FWY	1ZW9	3D0B	71.36		Sc=6.22297, min distance = 2.81686
HSP82_YEAST	24832021	HS90A_HUMAN F	6082103 2	2BT0	2BRC	2QG0	71.36		Sc=6.34534, min distance = 2.32271
HSP82_YEAST	24832022	HS90A_HUMAN F	6914564 2	2FWY	1ZW9	2QG2	71.36		Sc=6.2174, min distance = 2.622693
HSP82_YEAST	24836816	HS90A_HUMAN F	6022 Aden	1BYQ	1AMW	3BMY	71.36		Sc=6.24524, min distance = 2.46187
HSP82_YEAST	445966 O6	CDK2_HUMAN Fu	6022 Aden	1GY3	1AMW	1H0V			Sc=6.12761, min distance = 2.39164
HSP82_YEAST	4564 1e1v	CDK2_HUMAN Fu	6022 Aden	1GY3	1AMW	1E1V			Sc=6.09693, min distance = 2.29950
HSP82_YEAST	5326935 1	HS90A_HUMAN F	6082103 2	2BT0	2BRC	1YC4	71.36		Sc=6.30102, min distance = 2.74612
HSP82_YEAST	5327104 2	HS90A_HUMAN F	6082103 2	2BT0	2BRC	2BYI	71.36		Sc=6.40803, min distance = 2.18734
HSP82_YEAST	6102787 2	HS90A_HUMAN F	6082103 2	2BT0	2BRC	2CCS	71.36		Sc=6.0928, min distance = 2.855638

HSP82_YEAST I	6102788 2	HS90A_HUMAN Fu	6022 Aden	1BYQ	1AMW	2CCT	71.36		Sc=6.37917, min distance = 2.07375
HSP82_YEAST I	6102788 2	HS90A_HUMAN Fu	6082103 2	2BT0	2BRC	2CCT	71.36		Sc=6.37531, min distance = 2.79797
HSP82_YEAST I	72194 2-C	ENPL_CANFA Ful	6022 Aden	1TC6	1AMW	1QYE	52.88	0.89	Sc=5.97688, min distance = 2.79749
HTAI2_HUMAN I	16129590	Y5224_ARATH Fu	5886 NADP	1XQ6	2BKA	2Q4B		0.99	Sc=6.72907, min distance = 1.93227
HTPG_ECOLI Fu	16058668	HS90A_HUMAN Fu	6022 Aden	1BYQ	2IOR	2UWD			Sc=6.35948, min distance = 2.37431
HTPG_ECOLI Fu	24832021	HS90A_HUMAN Fu	6022 Aden	1BYQ	2IOR	2QG0			Sc=6.32739, min distance = 2.47589
HTPG_ECOLI Fu	446090 1h	NDKC_DICDI Ful	6022 Aden	1KDN	2IOR	1HIY		0.97	Sc=5.85826, min distance = 1.83455
HTPG_ECOLI Fu	447955 1p	CDK2_HUMAN Ful	6022 Aden	1GY3	2IOR	1PXI			Sc=5.62898, min distance = 2.19477
HTPG_ECOLI Fu	5326935 1	HS90A_HUMAN Fu	6022 Aden	1BYQ	2IOR	1YC4			Sc=6.28022, min distance = 2.72371
HTPG_ECOLI Fu	5327095 V	HS90A_HUMAN Fu	6022 Aden	1BYQ	2IOR	2BSM			Sc=6.40618, min distance = 2.42817
HTPG_ECOLI Fu	5403427 C	PDK4_HUMAN Ful	6022 Aden	2ZKJ	2IOR	2ZDX			Sc=6.23088, min distance = 2.13732
HTPG_ECOLI Fu	6082103 2	HS90A_HUMAN Fu	6022 Aden	1BYQ	2IOR	2BT0			Sc=6.29237, min distance = 2.21028
HTPG_ECOLI Fu	6082103 2	HSP82_YEAST Fu	6022 Aden	1AMW	2IOR	2BRC			Sc=6.31804, min distance = 2.46501
HTPG_ECOLI Fu	6102787 2	HS90A_HUMAN Fu	6022 Aden	1BYQ	2IOR	2CCS			Sc=6.17195, min distance = 2.52465
HTPG_ECOLI Fu	6102788 2	HS90A_HUMAN Fu	6022 Aden	1BYQ	2IOR	2CCT			Sc=6.40497, min distance = 2.37774
HTPG_ECOLI Fu	6323491 M	ENPL_CANFA Ful	6022 Aden	1TC6	2IOR	1QY8			Sc=6.27648, min distance = 2.61076
HTPG_ECOLI Fu	6323491 M	HSP82_YEAST Fu	6022 Aden	1AMW	2IOR	1BGQ			Sc=6.28261, min distance = 2.40641
HTPG_ECOLI Fu	6323491 M	PDK3_HUMAN Ful	6022 Aden	1Y8O	2IOR	2Q8I			Sc=6.27648, min distance = 2.68193
HTPG_ECOLI Fu	6323491 M	TOP6B_SULSH Fu	6022 Aden	1Z5B	2IOR	2HKJ			Sc=6.31696, min distance = 2.17345
HTPG_ECOLI Fu	72194 2-C	ENPL_CANFA Ful	6022 Aden	1TC6	2IOR	1QYE		0.89	Sc=6.05449, min distance = 2.67373
HUCE1_HUMAN I	12771338	MCES_HUMAN Ful	439155 Ad	3BGV	2ZFU	3EPP		0.88	Sc=6.13083, min distance = 2.37958
HUCE1_HUMAN I	188380 Ad	MCES_ENCCU Ful	439155 Ad	1RI1	2ZFU	1Z3C		0.91	Sc=6.11415, min distance = 2.19181
HUCE1_HUMAN I	446535 CI	HNMT_HUMAN Ful	439155 Ad	2AOT	2ZFU	1JQE		0.95	Sc=6.06948, min distance = 2.34649
HUCE1_HUMAN I	60961 ade	PIMT_PYRFU Ful	439155 Ad	1JG1	2ZFU	1JG2		0.84	Sc=6.30124, min distance = 2.41929
HUCE1_HUMAN I	65482 sir	MCES_ENCCU Ful	439155 Ad	1RI1	2ZFU	2HV9		0.88	Sc=6.56768, min distance = 2.17382
HUCE1_HUMAN I	65482 sir	PRMA_THET8 Ful	439155 Ad	3EGV	2ZFU	2ZBR		0.88	Sc=6.12356, min distance = 2.23814
HUGA_APIME Fu	16058615	LYSC_CHICK Ful	24139 ace	1LZB	1FCV	2H9J			Sc=5.82973, min distance = 0.64158
HUTU_BACSU Fu	11987786	LDH_PLAFD Full	5893 nadi	1T2D	2FKN	1T2E		0.77	Sc=6.80592, min distance = 2.60861
HUTU_BACSU Fu	123927 A3	Q9GT92_CRYPV I	5893 nadi	2FM3	2FKN	2EWD		0.97	Sc=6.81065, min distance = 2.50973
HUTU_BACSU Fu	439153 Di	CTBP1_RAT Full	5893 nadi	1HKU	2FKN	1HL3		0.79	Sc=6.81164, min distance = 2.02092
HUTU_BACSU Fu	440516 CI	MDH_THETH Full	5893 nadi	1BMD	2FKN	1BDM		0.79	Sc=6.81505, min distance = 2.49095
HUTU_BACSU Fu	6420113 C	MDH_THETH Full	5893 nadi	1BMD	2FKN	1WZI		0.77	Sc=6.69585, min distance = 2.01422
HUTU_GEOKA Fu	11987786	LDH_PLAFD Full	5893 nadi	1T2D	1X87	1T2E		0.77	Sc=6.37062, min distance = 2.10222
HUTU_GEOKA Fu	5957 Aden	KTRA_BACSU Ful	5893 nadi	1LSU	1X87	2HMW			Sc=5.73277, min distance = 2.19813
HUTU_PSEPU Fu	439153 Di	ADH1B_HUMAN Fu	5893 nadi	1U3U	1UWK	1DEH		0.79	Sc=6.80132, min distance = 1.56301
HUTU_PSEPU Fu	439153 Di	ADH1E_HORSE Fu	5893 nadi	1MGO	1UWK	2JHF		0.79	Sc=6.3669, min distance = 2.121023
HUTU_PSEPU Fu	440516 CI	MDH_THETH Full	5893 nadi	1BMD	1UWK	1BDM		0.79	Sc=6.81581, min distance = 2.01047

HXK1_HUMAN F	11970 2-H	ALDR_HUMAN Fu	439427 Rc	2ACQ	1CZA	2INZ		Sc=5.70476, min distance = 2.11797
HXK1_HUMAN F	124823 6g	PYGM_RABIT Fu	79025 alp	1H5U	1CZA	6GPB	0.78	Sc=6.1951, min distance = 1.971502
HXK1_HUMAN F	16750062	CSK2A_MAIZE F	33113 gam	1LP4	1QHA	2OXD		Sc=5.65191, min distance = 1.44860
HXK1_HUMAN F	439692 D-	XYLA_STRRU Fu	79025 alp	1XIF	1CZA	4XIS		Sc=5.71171, min distance = 2.34672
HXK1_HUMAN F	444393 CI	PYGM_RABIT Fu	79025 alp	1H5U	1CZA	1AXR		Sc=6.0853, min distance = 2.206273
HXK1_HUMAN F	445916 be	XYLA_STRRU Fu	79025 alp	1XIF	1CZA	1GW9	0.93	Sc=5.71905, min distance = 2.20678
HXK1_HUMAN F	445940 O6	CDK2_HUMAN Fu	6022 Aden	1GY3	1CZA	1GZ8		Sc=6.04663, min distance = 2.38751
HXK1_HUMAN F	446090 1h	NDKC_DICDI Fu	6022 Aden	1KDN	1CZA	1HIY	0.97	Sc=5.867, min distance = 2.2268293
HXK1_HUMAN F	447413 NT	PYGM_RABIT Fu	79025 alp	1H5U	1CZA	1NOJ		Sc=6.07827, min distance = 2.21159
HXK1_HUMAN F	5326977 1	CSK2A_MAIZE F	33113 gam	1LP4	1QHA	1ZOG		Sc=5.64926, min distance = 2.18683
HXK1_HUMAN F	6027 xylo	XYLA_STRRU Fu	79025 alp	1XIF	1CZA	3XIS	0.93	Sc=5.70741, min distance = 2.27507
HXK1_HUMAN F	6083 aden	HSP71_HUMAN F	33113 gam	2E8A	1QHA	1XQS	0.97	Sc=5.81796, min distance = 2.43674
HXK1_HUMAN F	6083 aden	HSP71_HUMAN F	6022 Aden	1S3X	1CZA	1XQS	0.99	Sc=5.83793, min distance = 2.63537
HXK1_HUMAN F	64960 Pol	XYLA_STRRU Fu	79025 alp	1XIF	1CZA	1XIE	0.89	Sc=5.83694, min distance = 2.42561
HXK1_HUMAN F	81058 2,6	ALDR_HUMAN Fu	439427 Rc	2ACQ	1CZA	2IS7		Sc=5.62063, min distance = 2.52521
HXK1_HUMAN F	82313 1ro	NAGK_HUMAN Fu	79025 alp	2CH6	1CZA	2CH5		Sc=6.20036, min distance = 1.99282
HXK1_HUMAN F	95259 L-x	XYLA_STRRU Fu	79025 alp	1XIF	1CZA	1XIC		Sc=5.79111, min distance = 2.33299
HXK1_HUMAN F	9543444 A	NDKC_DICDI Fu	6022 Aden	1KDN	1CZA	1MN7		Sc=5.70719, min distance = 2.12925
HXK1_RAT Ful	11970 2-H	ALDR_HUMAN Fu	439427 Rc	2ACQ	1BG3	2INZ		Sc=5.70006, min distance = 1.84804
HXK1_RAT Ful	33113 gam	HXK1_HUMAN Fu	439427 Rc	1CZA	1BG3	1QHA		Sc=5.95254, min distance = 2.40076
HXK1_RAT Ful	81058 2,6	ALDR_HUMAN Fu	439427 Rc	2ACQ	1BG3	2IS7		Sc=5.60241, min distance = 2.64452
HXK2_HUMAN F	10267 Har	CGGR_BACSU Fu	439427 Rc	3BXG	2NZT	3BXF	0.87	Sc=6.07592, min distance = 1.99602
HXK2_HUMAN F	11970 2-H	ALDR_HUMAN Fu	439427 Rc	2ACQ	2NZT	2INZ		Sc=5.75524, min distance = 1.91434
HXK2_HUMAN F	124823 6g	PYGM_RABIT Fu	79025 alp	1H5U	2NZT	6GPB	0.78	Sc=6.20104, min distance = 2.05023
HXK2_HUMAN F	439692 D-	XYLA_STRRU Fu	79025 alp	1XIF	2NZT	4XIS		Sc=5.70585, min distance = 2.30394
HXK2_HUMAN F	439764 be	Q9ZB17_9LACT	79025 alp	1NSZ	2NZT	1MMZ	0.93	Sc=5.73898, min distance = 1.93385
HXK2_HUMAN F	440641 1m	CGGR_BACSU Fu	439427 Rc	3BXG	2NZT	3BXH	0.89	Sc=6.00101, min distance = 2.18458
HXK2_HUMAN F	444185 1a	PYGM_RABIT Fu	79025 alp	1H5U	2NZT	1A8I		Sc=6.2909, min distance = 2.038156
HXK2_HUMAN F	445825 CI	PYGM_RABIT Fu	79025 alp	1H5U	2NZT	1GG8		Sc=6.09426, min distance = 2.06708
HXK2_HUMAN F	445916 be	XYLA_STRRU Fu	79025 alp	1XIF	2NZT	1GW9	0.93	Sc=5.72694, min distance = 2.44466
HXK2_HUMAN F	447413 NT	PYGM_RABIT Fu	79025 alp	1H5U	2NZT	1NOJ		Sc=6.08285, min distance = 2.11410
HXK2_HUMAN F	447607 1c	PYGM_RABIT Fu	79025 alp	1H5U	2NZT	2G9V		Sc=5.8121, min distance = 2.385439
HXK2_HUMAN F	6027 xylo	XYLA_STRRU Fu	79025 alp	1XIF	2NZT	3XIS	0.93	Sc=5.71171, min distance = 2.08553
HXK2_HUMAN F	64960 Pol	XYLA_STRRU Fu	79025 alp	1XIF	2NZT	1XIE	0.89	Sc=5.84333, min distance = 2.41672
HXK2_HUMAN F	82313 1ro	NAGK_HUMAN Fu	79025 alp	2CH6	2NZT	2CH5		Sc=6.20217, min distance = 1.82228
HXK2_HUMAN F	95259 L-x	XYLA_STRRU Fu	79025 alp	1XIF	2NZT	1XIC		Sc=5.78801, min distance = 2.24703
HXK4_HUMAN F	439692 D-	XYLA_STRRU Fu	79025 alp	1XIF	3F9M	4XIS		Sc=5.69799, min distance = 2.29167

HXK4_HUMAN F	444393 CI	PYGM_RABIT Fu	79025 alp	1H5U	3F9M	1AXR			Sc=6.08992, min distance = 2.175456
HXK4_HUMAN F	445916 be	XYLA_STRRU Fu	79025 alp	1XIF	3F9M	1GW9		0.93	Sc=5.72043, min distance = 2.149129
HXK4_HUMAN F	447413 NT	PYGM_RABIT Fu	79025 alp	1H5U	3F9M	1NOJ			Sc=6.07578, min distance = 2.331991
HXK4_HUMAN F	6027 xylo	XYLA_STRRU Fu	79025 alp	1XIF	3F9M	3XIS		0.93	Sc=5.70422, min distance = 2.154545
HXK4_HUMAN F	64960 Pol	XYLA_STRRU Fu	79025 alp	1XIF	3F9M	1XIE		0.89	Sc=5.83694, min distance = 2.287939
HXK4_HUMAN F	95259 L-x	XYLA_STRRU Fu	79025 alp	1XIF	3F9M	1XIC			Sc=5.78653, min distance = 2.269131
HXKA_YEAST F	29435 1-D	Q2PS28_9PSED	64689 bet	2PWF	3B8A	2PWD			Sc=5.88842, min distance = 2.339971
HXKA_YEAST F	445999 CI	Q79G13_MYCTU	64689 bet	1UP0	3B8A	1UOZ		0.77	Sc=5.71804, min distance = 2.262095
HXKA_YEAST F	447607 1c	Q79G13_MYCTU	64689 bet	1UP0	3B8A	1UP2			Sc=5.78503, min distance = 2.173932
HXKA_YEAST F	6102765 C	Q9RX51_DEIRA	64689 bet	2BY0	3B8A	2BY2			Sc=5.60266, min distance = 2.505578
HXK_SCHMA Fu	4369162 1	PYGM_RABIT Fu	79025 alp	1H5U	1BDG	1HLF			Sc=6.25006, min distance = 2.047911
HXK_SCHMA Fu	439764 be	Q9ZB17_9LACT	79025 alp	1NSZ	1BDG	1MMZ		0.93	Sc=5.70741, min distance = 2.329996
HXK_SCHMA Fu	444185 1a	PYGM_RABIT Fu	79025 alp	1H5U	1BDG	1A8I			Sc=6.28121, min distance = 2.119634
HXK_SCHMA Fu	445825 CI	PYGM_RABIT Fu	79025 alp	1H5U	1BDG	1GG8			Sc=6.07969, min distance = 2.029698
HXK_SCHMA Fu	445916 be	XYLA_STRRU Fu	79025 alp	1XIF	1BDG	1GW9		0.93	Sc=5.69505, min distance = 2.338292
HXK_SCHMA Fu	447413 NT	PYGM_RABIT Fu	79025 alp	1H5U	1BDG	1NOJ			Sc=6.07397, min distance = 2.419885
HXK_SCHMA Fu	447607 1c	PYGM_RABIT Fu	79025 alp	1H5U	1BDG	2G9V			Sc=5.78964, min distance = 2.613076
HXK_SCHMA Fu	6027 xylo	XYLA_STRRU Fu	79025 alp	1XIF	1BDG	3XIS		0.93	Sc=5.67438, min distance = 2.261173
HXK_SCHMA Fu	64960 Pol	XYLA_STRRU Fu	79025 alp	1XIF	1BDG	1XIE		0.89	Sc=5.81999, min distance = 2.333457
HXK_SCHMA Fu	892 myo-i	SFTPD_HUMAN F	79025 alp	1PWB	1BDG	2OS9			Sc=5.99317, min distance = 2.056261
HXK_SCHMA Fu	95259 L-x	XYLA_STRRU Fu	79025 alp	1XIF	1BDG	1XIC			Sc=5.75027, min distance = 2.315126
HYES_HUMAN F	4358 1cr6	HYES_MOUSE Fu	4357 1ek1	1EK1	1VJ5	1CR6			Sc=6.15443, min distance = 2.036028
HYP1_TRIRE F	11987742	LHA4_RHOAC Fu	444279 1s	2FKW	2GVM	1NKZ			Sc=6.37032, min distance = 0.374510
HYSA_STRPN F	10367 Dip	LYSC_CHICK Fu	24139 ace	1LZB	1LXK	2PC2			Sc=5.90725, min distance = 1.752477
HYSA_STRPN F	119404 Ma	Q54276_SERMA	24139 ace	1UR9	1LXK	1W1Y			Sc=5.97734, min distance = 2.266091
HYSA_STRPN F	16058615	LYSC_CHICK Fu	24139 ace	1LZB	1LXK	2H9J			Sc=5.95881, min distance = 2.026566
HYSA_STRPN F	444205 CI	DPP4_HUMAN Fu	24139 ace	2RGU	1LXK	3BJM		0.91	Sc=5.8446, min distance = 2.5751852
HYSA_STRPN F	449187 CI	XYLA_ARTS7 Fu	6912 xyli	1XLM	1W3Y	1XLI			Sc=5.84332, min distance = 2.107918
I23O_HUMAN F	11957371	HMOX1_HUMAN F	444124 HE	1OZW	2D0T	1TWR			Sc=6.43886, min distance = 1.946504
I23O_HUMAN F	11957385	MYG_PHYCA Ful	444124 HE	1U7R	2D0T	2CMM			Sc=5.84165, min distance = 2.301388
IAAS_HORVU F	11957399	PYRB_ECOLI Fu	311 citri	1R0B	2IWT	2H3E			Sc=6.0474, min distance = 1.5536608
IAAS_HORVU F	12136 Ber	POL_HV1N5 Ful	311 citri	2GON	2IWT	9HVP			Sc=5.64333, min distance = 0.652619
IAAS_HORVU F	126892 1t	TPIS_TRYBB Fu	311 citri	2VEK	2IWT	1TSI			Sc=5.66352, min distance = 2.119371
IAAS_HORVU F	448032 He	RECA_MYCS2 Fu	311 citri	2G88	2IWT	2ZRJ			Sc=5.7721, min distance = 2.8409653
IDGF2_DROME	444885 O-	NRAM_I56A2 Fu	185698 al	1V0Z	1JND	1W1X			Sc=6.45797, min distance = 1.149963
IDH1_YEAST F	11957399	PYRB_ECOLI Fu	311 citri	1R0B	3BLV	2H3E			Sc=6.08963, min distance = 1.093146
IDH1_YEAST F	166760 Fa	PURP_METJA Fu	6083 aden	2R7M	3BLW	2R7N		0.76	Sc=5.89408, min distance = 2.383855

IDH1_YEAST F	39981 Spa	PYRB_ECOLI Fu	311 citri	1R0B	3BLV	1EKX			Sc=6.11772, min distance = 1.01589
IDH1_YEAST F	447376 2-	ACON_BOVIN Fu	311 citri	1C96	3BLV	1NIS			Sc=5.80152, min distance = 2.30939
IDH1_YEAST F	447550 ph	TPIS_TRYBB Fu	311 citri	2VEK	3BLV	4TIM			Sc=5.67626, min distance = 1.66140
IDH1_YEAST F	448895 AD	UMPK_YEAST Fu	6083 aden	1UKZ	3BLW	1UKY		0.99	Sc=5.96531, min distance = 2.38934
IDH1_YEAST F	51 2-Oxop	SERA_ECOLI Fu	311 citri	2P9E	3BLV	1YBA		0.76	Sc=5.8227, min distance = 0.897250
IDH1_YEAST F	6022 Ader	PURP_METJA Fu	6083 aden	2R7M	3BLW	2R7N		0.99	Sc=5.90765, min distance = 2.35324
IDH1_YEAST F	65110 AIC	PURP_METJA Fu	6083 aden	2R7M	3BLW	2R7K		0.79	Sc=5.79137, min distance = 2.31687
IDH1_YEAST F	6804 guar	PDE10_HUMAN F	6083 aden	2OUN	3BLW	2OUQ		0.8	Sc=5.82171, min distance = 0
IDH1_YEAST F	8582 Inos	PYGM_RABIT Fu	6083 aden	8GPB	3BLW	2QN7		0.79	Sc=6.23669, min distance = 2.21286
IDH1_YEAST F	8778 Laur	CTXA3_NAJAT F	311 citri	1XT3	3BLV	1H0J			Sc=5.74637, min distance = 0.32280
IDHC_MOUSE F	11957399	PYRB_ECOLI Fu	311 citri	1R0B	2CMV	2H3E			Sc=5.98234, min distance = 1.73596
IDHC_MOUSE F	12136 Ber	POL_HV1N5 Ful	311 citri	2GON	2CMV	9HVP			Sc=5.67436, min distance = 1.01939
IDHC_MOUSE F	12793 Phe	SRC_HUMAN Ful	311 citri	1O4L	2CMV	1O4O			Sc=5.70866, min distance = 1.22840
IDHC_MOUSE F	445607 AT	ACON_BOVIN Fu	311 citri	1C96	2CMV	1FGH			Sc=5.86151, min distance = 1.80890
IDHC_MOUSE F	447376 2-	ACON_BOVIN Fu	311 citri	1C96	2CMV	1NIS			Sc=5.79098, min distance = 2.11425
IDHC_MOUSE F	447530	SRC_HUMAN Ful	311 citri	1O4L	2CMV	1O4F			Sc=5.82831, min distance = 2.03546
IDHC_MOUSE F	449604 NI	ACON_BOVIN Fu	311 citri	1C96	2CMV	8ACN			Sc=5.75704, min distance = 2.20914
IDHP_PIG Ful	51 2-Oxop	IDHP_YEAST Fu	5318532 i	2QFW	1LWD	2QFY			Sc=5.82669, min distance = 1.19494
IDHP_YEAST F	444212 tr	ACON_BOVIN Fu	5318532 i	1C97	2QFW	1ACO			Sc=5.75051, min distance = 2.25819
IDHP_YEAST F	447376 2-	ACON_BOVIN Fu	5318532 i	1C97	2QFW	1NIS			Sc=5.71965, min distance = 1.95737
IDHP_YEAST F	449604 NI	ACON_BOVIN Fu	5318532 i	1C97	2QFW	8ACN			Sc=5.73745, min distance = 2.32688
IDH_AZOVI Fu	444591 CI	IDH_ECOLI Ful	5318532 i	1P8F	1ITW	1BL5			Sc=5.60978, min distance = 2.20086
IDH_AZOVI Fu	51 2-Oxop	IDHP_YEAST Fu	5318532 i	2QFW	1ITW	2QFY			Sc=5.82406, min distance = 0.90012
IDH_BACSU Fu	11957399	PYRB_ECOLI Fu	311 citri	1R0B	1HQS	2H3E			Sc=6.04051, min distance = 1.46020
IDH_BACSU Fu	12136 Ber	POL_HV1N5 Ful	311 citri	2GON	1HQS	9HVP			Sc=5.6677, min distance = 38.37279
IDH_BACSU Fu	16750041	PYRB_ECOLI Fu	311 citri	1R0B	1HQS	2IPO			Sc=6.04989, min distance = 0.99529
IDH_BACSU Fu	39981 Spa	PYRB_ECOLI Fu	311 citri	1R0B	1HQS	1EKX			Sc=6.07727, min distance = 1.47766
IDH_BACSU Fu	444212 tr	ACON_BOVIN Fu	311 citri	1C96	1HQS	1ACO			Sc=5.74521, min distance = 2.00945
IDH_BACSU Fu	447530	SRC_HUMAN Ful	311 citri	1O4L	1HQS	1O4F			Sc=5.79766, min distance = 2.28673
IDH_BACSU Fu	51 2-Oxop	SERA_ECOLI Fu	311 citri	2P9E	1HQS	1YBA		0.76	Sc=5.83588, min distance = 0.80506
IDH_BACSU Fu	9547937 2	PYRB_ECOLI Fu	311 citri	1R0B	1HQS	2FZG			Sc=6.41003, min distance = 0.92641
IDH_ECOLI Fu	3080614 C	JHD3A_HUMAN F	51 2-Oxop	2Q8C	1CW4	2OX0			Sc=5.62257, min distance = 2.32717
IDH_THET8 Fu	3758 IBMX	PDE5A_HUMAN F	311 citri	1T9R	2D1C	1RKP			Sc=6.04785, min distance = 2.02654
IDI2_BACSU F	5287459 A	PYRD_HUMAN Fu	444243 FA	1D3G	1P0N	2B0M			Sc=6.13452, min distance = 2.17523
IF4A3_HUMAN	3547 Fasu	ROCK1_HUMAN F	33113 gam	2V55	2J0S	2ESM			Sc=6.00397, min distance = 1.93368
IF4A3_HUMAN	448042 2e	ROCK1_HUMAN F	33113 gam	2V55	2J0S	2ETR			Sc=6.2355, min distance = 1.816986
IF4A3_HUMAN	6083 ader	ENTP2_RAT Ful	33113 gam	3CJA	2J0S	3CJ7		0.97	Sc=6.33538, min distance = 2.40298

IF4A3_HUMAN I	6083 aden	HSP71_HUMAN Fu	33113 gam	2E8A	2J0S	1XQS		0.97	Sc=6.37139, min distance = 2.15990
IF4A3_HUMAN I	6083 aden	Q6L8F0_THETH I	33113 gam	1V25	2J0S	1V26		0.97	Sc=6.38285, min distance = 2.172085
IF4A3_HUMAN I	6338561 C	MYS2_DICDI Fu	33113 gam	1MMN	2J0S	1D0X			Sc=6.25006, min distance = 2.258479
IF4A3_HUMAN I	6338562 C	MYS2_DICDI Fu	33113 gam	1MMN	2J0S	1D0Y			Sc=6.24668, min distance = 2.252447
IF4A3_HUMAN I	6338566 C	MYS2_DICDI Fu	33113 gam	1MMN	2J0S	1D1C			Sc=6.25616, min distance = 2.259672
IF4A3_HUMAN I	64968 dTI	RIR1_YEAST Fu	33113 gam	2EUD	2J0S	2CVW			Sc=6.02717, min distance = 2.102703
IF4A3_HUMAN I	91532 AMF	KIF1A_MOUSE Fu	33113 gam	1VFV	2J0S	1I6I		0.99	Sc=6.11077, min distance = 2.373572
IF4A_YEAST Fu	656966 1y	PDE4D_HUMAN Fu	6083 aden	1TB7	2VSO	1Y2E			Sc=5.88549, min distance = 1.992556
IF4A_YEAST Fu	95054 Pte	RICI_RICCO Fu	6083 aden	1OBT	2VSO	1BR6			Sc=6.3599, min distance = 2.0173889
IGF1R_HUMAN I	188966 dA	HSLU_ECOLI Fu	33113 gam	1E94	1JQH	1G4A		0.97	Sc=5.61812, min distance = 2.593537
IGF1R_HUMAN I	23656870	CSK2A_MAIZE Fu	33113 gam	1LP4	1JQH	2PVN	23.45		Sc=6.48741, min distance = 2.003853
IGF1R_HUMAN I	444564 AD	PDK2_RAT Full=	33113 gam	3CRL	1JQH	1JM6		0.91	Sc=5.9352, min distance = 2.3940977
IGF1R_HUMAN I	5281672 m	PIM1_HUMAN Fu	33113 gam	1XR1	1JQH	2O63	27.24		Sc=6.40703, min distance = 1.859376
IGF1R_HUMAN I	5326739 i	GSK3B_HUMAN Fu	33113 gam	1J1B	1JQH	1Q41	28.71		Sc=6.251, min distance = 2.3122588
IGF1R_HUMAN I	5326978 1	CSK2A_MAIZE Fu	33113 gam	1LP4	1JQH	1ZOH	23.45		Sc=5.62112, min distance = 2.298513
IGF1R_HUMAN I	6022 Ader	GLPK_ECOLI Fu	91532 AMF	1GLL	1K3A	1GLB		0.99	Sc=6.30362, min distance = 1.507164
IGF1R_HUMAN I	60961 ade	SKY1_YEAST Fu	33113 gam	1Q99	1JQH	1Q97		0.93	Sc=6.10571, min distance = 2.621525
IGHA1_HUMAN I	30951 alc	PGH1_SHEEP Fu	444205 CI	1EQG	1OW0	1HT8			Sc=5.75714, min distance = 2.238899
IGHE_HUMAN Fu	70815 CAE	Q6PYX1_HUMAN I	185698 al	1ZLS	1O0V	1N0X	32.99		Sc=5.78713, min distance = 0.641732
IGHG1_HUMAN I	446996 GA	Q9HYN5_PSEAE I	439353 be	1W8F	1L6X	1W8H		0.89	Sc=5.69574, min distance = 2.303557
IGHG3_MOUSE I	101798 Me	Q9HYN5_PSEAE I	439353 be	1W8F	1CLZ	2JDN		0.94	Sc=5.709, min distance = 2.0787104
IGHG3_MOUSE I	445948 D-	Q9HYN5_PSEAE I	439353 be	1W8F	1CLZ	1OXC			Sc=5.67529, min distance = 2.292241
IGHG3_MOUSE I	446220 cc	Q5XKG4_MOUSE I	439353 be	1UZ8	1CLZ	1Q72			Sc=5.95123, min distance = 2.207004
IGHG3_MOUSE I	446578 1r	MBL1_RAT Full=	439554 fu	3KMB	1CLZ	1KWW		0.96	Sc=5.60236, min distance = 2.178789
IGHG3_MOUSE I	446578 1r	Q9HYN5_PSEAE I	439353 be	1W8F	1CLZ	2JDP		0.94	Sc=5.6049, min distance = 2.2251231
IGHG3_MOUSE I	94214 Met	MBL1_RAT Full=	439353 be	2KMB	1CLZ	1AFA		0.94	Sc=5.6862, min distance = 3.0655089
IGKC_MOUSE Fu	23624247	WRBA_ECOLI Fu	62551 Pen	3B6K	1P7K	3B6I			Sc=5.81461, min distance = 2.326865
IIGP1_MOUSE I	444845 1c	RASH_HUMAN Fu	36735 Gpp	1CTQ	1TQ2	1CLU		0.99	Sc=5.80195, min distance = 2.356506
IIGP1_MOUSE I	445871 CI	RASH_HUMAN Fu	36735 Gpp	1CTQ	1TQ2	1GNP			Sc=6.10566, min distance = 2.345828
IIGP1_MOUSE I	445871 CI	RASH_HUMAN Fu	8977 1dar	2CE2	1TQ4	1GNP			Sc=5.83632, min distance = 2.466594
IIGP1_MOUSE I	446248 CI	RAN_HUMAN Full	8977 1dar	3CH5	1TQ4	1IBR			Sc=5.80217, min distance = 2.247470
IIGP1_MOUSE I	446248 CI	RASH_HUMAN Fu	36735 Gpp	1CTQ	1TQ2	1IAQ			Sc=5.7603, min distance = 2.0606736
IIGP1_MOUSE I	6804 guar	DPOL_BPR69 Fu	8977 1dar	1CLQ	1TQ4	1WAJ		0.99	Sc=5.94541, min distance = 2.049837
IIGP1_MOUSE I	8200 TETF	LMBL1_HUMAN Fu	78165 MES	1OZ2	1TQD	2RHI			Sc=5.73273, min distance = 0.841853
IIGP1_MOUSE I	93082 Gak	CDC42_HUMAN Fu	36735 Gpp	1NF3	1TQ2	2ODB		0.99	Sc=5.79931, min distance = 2.395527
IIGP1_MOUSE I	93082 Gak	FTSY_THEAQ Fu	36735 Gpp	2J7P	1TQ2	1RJ9		0.99	Sc=5.81193, min distance = 2.177762
IIGP1_MOUSE I	93082 Gak	RAC3_HUMAN Fu	36735 Gpp	2IC5	1TQ2	2QME		0.99	Sc=5.80803, min distance = 2.425771

IIGP1_MOUSE I	93082 Gab	RASH_HUMAN Fu	36735 Gpp	1CTQ	1TQ2	121P		0.99	Sc=5.79605, min distance = 2.43183
ILV5_SPIOL F	16741136	DYR_ECOLI Ful	5886 NADF	1RA2	1YVE	1RX9	1		Sc=6.39173, min distance = 1.54284
ILV5_SPIOL F	440141 9i	G6PD_LEUME Fu	5886 NADF	1H9A	1YVE	1E7Y			Sc=6.40995, min distance = 1.53506
ILVB_ARATH F	24836827	BZNB_PSEFL Fu	1132 thia	2UZ1	1Z8N	3D7K			Sc=5.92306, min distance = 2.02988
ILVB_ARATH F	444421 CI	MDLC_PSEPU Fu	1132 thia	1BFD	1Z8N	2FWN	23.77	0.82	Sc=6.41013, min distance = 2.48071
ILVB_ARATH F	445886 CI	TKT1_YEAST Fu	1132 thia	1TRK	1Z8N	1GPU		0.91	Sc=6.06071, min distance = 1.96962
ILVB_ARATH F	447248 AY	ILVB_YEAST Fu	153909 Ex	1T9A	1YI1	1N0H	43.68		Sc=6.4239, min distance = 2.144457
ILVB_ARATH F	447248 AY	ILVB_YEAST Fu	47491 CHI	1T9B	1YHZ	1N0H	43.68		Sc=6.36567, min distance = 2.13465
ILVB_ARATH F	447248 AY	ILVB_YEAST Fu	52997 OUS	1T9C	1YI0	1N0H	43.68		Sc=6.40963, min distance = 2.49850
ILVB_ARATH F	448670 PF	ILVB_YEAST Fu	448674 ET	1T9B	1YBH	1T9C	43.68	0.96	Sc=5.71845, min distance = 2.69703
ILVB_ARATH F	448671 2-	ILVB_YEAST Fu	1132 thia	1N0H	1Z8N	1T9B	43.68		Sc=5.93815, min distance = 1.84059
ILVB_ARATH F	448671 2-	ILVB_YEAST Fu	153909 Ex	1T9A	1YI1	1T9B	43.68		Sc=5.97589, min distance = 0.85946
ILVB_ARATH F	448671 2-	ILVB_YEAST Fu	52997 OUS	1T9C	1YI0	1T9B	43.68		Sc=5.85005, min distance = 1.53944
ILVB_ARATH F	448672 DE	ILVB_YEAST Fu	52997 OUS	1T9C	1YI0	1T9A	43.68		Sc=5.65567, min distance = 2.78459
ILVB_ARATH F	448673 PE	ILVB_YEAST Fu	1132 thia	1N0H	1Z8N	1T9B	43.68		Sc=5.86753, min distance = 2.62082
ILVB_ARATH F	448673 PE	ILVB_YEAST Fu	153909 Ex	1T9A	1YI1	1T9B	43.68		Sc=5.85509, min distance = 2.37817
ILVB_ARATH F	448673 PE	ILVB_YEAST Fu	52997 OUS	1T9C	1YI0	1T9B	43.68		Sc=5.8625, min distance = 2.366114
ILVB_ARATH F	448721 N3	TKT1_YEAST Fu	1132 thia	1TRK	1Z8N	1TKA			Sc=6.38419, min distance = 2.08938
ILVB_ARATH F	448722 1t	TKT1_YEAST Fu	1132 thia	1TRK	1Z8N	1TKB		0.78	Sc=6.4317, min distance = 2.071985
ILVB_ARATH F	448723 1t	TKT1_YEAST Fu	1132 thia	1TRK	1Z8N	1TKC		0.77	Sc=6.11332, min distance = 2.17721
ILVB_ARATH F	6518182 C	POXB_LACPL Fu	1132 thia	2EZ4	1Z8N	2EZ9	26.68	0.94	Sc=5.78413, min distance = 2.61786
ILVB_ARATH F	6518187 2	POXB_LACPL Fu	1132 thia	2EZ4	1Z8N	2EZ8	26.68	0.87	Sc=6.07701, min distance = 2.50367
ILVB_YEAST F	124687 1r	ODP1_ECOLI Fu	1132 thia	1L8A	1N0H	1RP7		0.79	Sc=6.02706, min distance = 2.11968
ILVB_YEAST F	13294447	DCIP_AZOBR Fu	1132 thia	2NXW	1N0H	2Q5Q	23.09		Sc=6.04697, min distance = 0.64823
ILVB_YEAST F	448721 N3	TKT1_YEAST Fu	1132 thia	1TRK	1N0H	1TKA			Sc=6.51928, min distance = 1.13990
ILVB_YEAST F	448722 1t	TKT1_YEAST Fu	1132 thia	1TRK	1N0H	1TKB		0.78	Sc=6.51928, min distance = 0.98272
ILVB_YEAST F	448723 1t	TKT1_YEAST Fu	1132 thia	1TRK	1N0H	1TKC		0.77	Sc=6.55152, min distance = 0.89462
ILVB_YEAST F	52999 Met	ILVB_ARATH Fu	1132 thia	1Z8N	1N0H	1YHY	43.68		Sc=6.14764, min distance = 2.44849
ILVB_YEAST F	52999 Met	ILVB_ARATH Fu	52997 OUS	1YI0	1T9C	1YHY	43.68	0.81	Sc=6.10282, min distance = 2.60120
ILVB_YEAST F	52999 Met	ILVB_ARATH Fu	56160 Chl	1YBH	1N0H	1YHY	43.68	0.77	Sc=6.05838, min distance = 2.76214
ILVE_THEMA F	445062 CI	HEM1_RHOCA Fu	1051 Code	2BWO	3CSW	2BWP		0.86	Sc=6.37796, min distance = 1.53429
ILVE_THEMA F	445496 TF	A2NHM3_MOUSE I	311 citri	1ZEA	3CSW	1F3D			Sc=5.67274, min distance = 2.62691
IMDH2_CRIGR I	100252 Ri	IMDH_TRIFO Fu	446541 my	1MEH	1JR1	1ME8			Sc=6.29479, min distance = 1.93601
IMDH2_CRIGR I	447942 MZ	IMDH_TRIFO Fu	446541 my	1MEH	1JR1	1PVN			Sc=6.33557, min distance = 2.13914
IMDH2_HUMAN I	447942 MZ	IMDH_TRIFO Fu	100252 Ri	1ME8	1NF7	1PVN	42.33		Sc=5.85574, min distance = 2.29012
IMDH_PYRHO F	100252 Ri	IMDH_TRIFO Fu	73323 Xan	1MEW	2CU0	1ME8			Sc=6.24035, min distance = 2.33023
IMDH_PYRHO F	446541 my	IMDH_TRIFO Fu	73323 Xan	1MEW	2CU0	1MEH			Sc=6.22853, min distance = 2.07489

IMDH_PYRHO Fu	8582 Inos	IMDH_TRIFO Ful	73323 Xan	1MEW	2CU0	1MEH		0.94	Sc=6.34967, min distance = 2.24702
IMDH_STRPY Fu	447942 MZ	IMDH_TRIFO Ful	8582 Inos	1MEH	1ZFJ	1PVN			Sc=5.76136, min distance = 2.15648
IMDH_STRPY Fu	6083 aden	PYGM_RABIT Ful	8582 Inos	2QN7	1ZFJ	8GPB		0.79	Sc=6.28615, min distance = 1.98794
IMDH_STRPY Fu	65110 AIC	PURO_METTH Ful	8582 Inos	2NTK	1ZFJ	2NTL		0.97	Sc=6.26318, min distance = 1.95896
IMDH_STRPY Fu	6804 guar	GMPR2_HUMAN Fu	8582 Inos	2C6Q	1ZFJ	2A7R		0.99	Sc=6.28562, min distance = 1.83847
IMDH_STRPY Fu	6804 guar	HGXR_TOXGO Ful	8582 Inos	1QK4	1ZFJ	1QK3		0.99	Sc=6.30257, min distance = 2.05920
IMDH_STRPY Fu	73323 Xar	IMDH_TRIFO Ful	8582 Inos	1MEH	1ZFJ	1MEW		0.94	Sc=6.32908, min distance = 2.13636
IMDH_TRIFO Fu	5414 Octy	OMPG_ECOLI Ful	62852 B-C	2IWW	1LRT	2F1C			Sc=5.82197, min distance = 2.06344
IMDH_TRIFO Fu	6083 aden	PYGM_RABIT Ful	8582 Inos	2QN7	1MEH	8GPB		0.79	Sc=6.33495, min distance = 2.20619
IMPA1_HUMAN I	21120523	SFTPD_HUMAN Fu	107737 in	2ORK	1IMA	2RIA			Sc=5.95677, min distance = 1.47537
IMPA1_HUMAN I	440115 N-	SFTPD_HUMAN Fu	107737 in	2ORK	1IMA	2ORJ			Sc=6.01294, min distance = 2.13012
IMPA1_HUMAN I	79025 alp	SFTPD_HUMAN Fu	107737 in	2ORK	1IMA	1PWB			Sc=5.76862, min distance = 2.36986
IMPA1_HUMAN I	892 myo-i	SFTPD_HUMAN Fu	107737 in	2ORK	1IMA	2OS9		0.85	Sc=5.84784, min distance = 2.33510
INHA_MYCTU Fu	16129587	G3PA_SPIOL Ful	5893 nadi	1NBO	2H7I	2PKR			Sc=5.87116, min distance = 1.46518
INHA_MYCTU Fu	169266 1,	GALE_HUMAN Ful	5893 nadi	1HZJ	2H7I	1I3K		0.79	Sc=6.30588, min distance = 2.42556
INHA_MYCTU Fu	439153 Di	Q9BJJ9_PLAFA I	5893 nadi	1UH5	2H7I	1V35	32.62	0.79	Sc=6.74653, min distance = 2.63610
INHA_MYCTU Fu	446288 NA	G3P_PALVE Ful	5893 nadi	1DSS	2H7I	1IHX			Sc=6.34436, min distance = 1.11784
INHA_MYCTU Fu	447151 CI	FABI_ECOLI Ful	5893 nadi	1QSG	2H7I	1MFP			Sc=6.39285, min distance = 1.99789
INMT_HUMAN Fu	188380 Ad	MCES_ENCCU Ful	439155 Ad	1RI1	2A14	1Z3C		0.91	Sc=6.59511, min distance = 2.21461
INMT_HUMAN Fu	446535 CI	HNMT_HUMAN Ful	439155 Ad	2AOT	2A14	1JQE		0.95	Sc=6.55258, min distance = 2.56066
INMT_HUMAN Fu	60961 ade	PIMT_PYRFU Ful	439155 Ad	1JG1	2A14	1JG2		0.84	Sc=6.30787, min distance = 2.56044
INMT_HUMAN Fu	65482 sir	MCES_ENCCU Ful	439155 Ad	1RI1	2A14	2HV9		0.88	Sc=6.13293, min distance = 2.37878
INO1_YEAST Fu	11987635	ADH_DROLE Ful	5893 nadi	1SBY	1P1J	1B2L		0.76	Sc=6.87264, min distance = 1.82945
INO1_YEAST Fu	445794 AD	ADHX_HUMAN Ful	5893 nadi	2FZW	1P1J	2FZE			Sc=6.66643, min distance = 2.16570
INSR_HUMAN Fu	11175137	KAPCA_BOVIN Fu	5957 Aden	1Q24	3BU5	2UW7	26.03		Sc=6.1346, min distance = 2.159375
INSR_HUMAN Fu	11696113	KAPCA_BOVIN Fu	5957 Aden	1Q24	3BU5	2VO3	26.03		Sc=6.27467, min distance = 2.22685
INSR_HUMAN Fu	11708454	KAPCA_BOVIN Fu	5957 Aden	1Q24	3BU5	2VNW	26.03		Sc=6.04274, min distance = 2.23798
INSR_HUMAN Fu	11957393	FAK1_HUMAN Ful	5957 Aden	2IJM	3BU5	2ETM	38.67		Sc=6.33455, min distance = 2.01667
INSR_HUMAN Fu	15602983	KAPCA_BOVIN Fu	5957 Aden	1Q24	3BU5	2UW5	26.03		Sc=6.06623, min distance = 2.20157
INSR_HUMAN Fu	16040273	IRAK4_HUMAN Fu	33113 gam	2OID	1IR3	2OIC	34.55		Sc=6.41081, min distance = 1.16792
INSR_HUMAN Fu	16058649	IGF1R_HUMAN Fu	33113 gam	1JQH	1IR3	2OJ9	79.73		Sc=6.45883, min distance = 1.89159
INSR_HUMAN Fu	16122632	KAPCA_BOVIN Fu	5957 Aden	1Q24	3BU5	2UVZ	26.03		Sc=6.21979, min distance = 2.17137
INSR_HUMAN Fu	16725726	AKT2_HUMAN Ful	33113 gam	1O6L	1IR3	3D0E	30.21		Sc=6.47453, min distance = 2.23796
INSR_HUMAN Fu	17751819	KAPCA_BOVIN Fu	5957 Aden	1Q24	3BU5	2VO6	26.03		Sc=6.24245, min distance = 2.22340
INSR_HUMAN Fu	17754396	PDPK1_HUMAN Fu	5957 Aden	2BIY	3BU5	2R7B	24.38		Sc=6.29653, min distance = 2.09268
INSR_HUMAN Fu	24851689	PIM1_HUMAN Ful	33113 gam	1XR1	1IR3	3CY2	25.17		Sc=6.15501, min distance = 2.19509
INSR_HUMAN Fu	3064778 H	ROCK1_HUMAN Fu	33113 gam	2V55	1IR3	2ETK	25.15		Sc=6.02271, min distance = 2.50379

INSR_HUMAN F	3540 1yds	KAPCA_BOVIN F	5957 Aden	1Q24	3BU5	1YDS	26.03		Sc=5.83184, min distance = 2.37418
INSR_HUMAN F	3547 Fasu	KAPCA_BOVIN F	5957 Aden	1Q24	3BU5	1Q8W	26.03		Sc=5.88166, min distance = 2.07696
INSR_HUMAN F	3547 Fasu	ROCK1_HUMAN F	33113 gam	2V55	1IR3	2ESM	25.15		Sc=5.95366, min distance = 2.34658
INSR_HUMAN F	444345 1c	IRAK4_HUMAN F	33113 gam	2OID	1IR3	2NRY	34.55		Sc=6.35577, min distance = 1.07104
INSR_HUMAN F	444852 CI	ATPB_BOVIN Fu	33113 gam	2JDI	1IR3	1COW		0.91	Sc=5.65803, min distance = 2.01505
INSR_HUMAN F	447916 ac	RIO1_ARCFU Fu	5957 Aden	1ZP9	3BU5	1ZTF		0.94	Sc=6.07709, min distance = 2.91036
INSR_HUMAN F	448043 2c	ROCK1_HUMAN F	33113 gam	2V55	1IR3	3D9V	25.15		Sc=5.97603, min distance = 1.78862
INSR_HUMAN F	449240 1y	KAPCA_BOVIN F	5957 Aden	1Q24	3BU5	1YDR	26.03		Sc=5.88353, min distance = 2.02351
INSR_HUMAN F	456214 pu	KS6A1_HUMAN F	91532 AMP	2Z7Q	1I44	2Z7S			Sc=6.25751, min distance = 2.39135
INSR_HUMAN F	5281680 C	PIM1_HUMAN Fu	33113 gam	1XR1	1IR3	2O64	25.17		Sc=6.40703, min distance = 2.03395
INSR_HUMAN F	5326637 1	CSK2A_MAIZE F	33113 gam	1LP4	1IR3	1M2Q	21.9		Sc=6.07332, min distance = 2.10924
INSR_HUMAN F	5326977 1	CSK2A_MAIZE F	33113 gam	1LP4	1IR3	1ZOG	21.9		Sc=5.61169, min distance = 2.18881
INSR_HUMAN F	5326978 1	CSK2A_MAIZE F	33113 gam	1LP4	1IR3	1ZOH	21.9		Sc=5.65164, min distance = 2.41164
INSR_HUMAN F	5327148 C	IPKA_RABIT Fu	33113 gam	1CDK	1IR3	2ERZ			Sc=6.13841, min distance = 2.37750
INSR_HUMAN F	6022 Aden	GLPK_ECOLI Fu	91532 AMP	1GLL	1I44	1GLB		0.99	Sc=5.84631, min distance = 2.28274
INSR_HUMAN F	6022 Aden	PURP_METJA Fu	91532 AMP	2R7K	1I44	2R7N		0.99	Sc=5.82945, min distance = 2.39998
INSR_HUMAN F	60961 ade	SKY1_YEAST Fu	33113 gam	1Q99	1IR3	1Q97		0.93	Sc=6.17643, min distance = 2.08534
INSR_HUMAN F	9934347 E	FAK1_CHICK Fu	33113 gam	2J0L	1IR3	2JKK	38.82		Sc=6.49513, min distance = 2.03507
INVA_YERPS F	445905 1g	AROQ_HELPY Fu	311 citri	2C4V	1CWV	2C57			Sc=5.73025, min distance = 1.93290
INVA_YERPS F	446127 3-	RENI_HUMAN Fu	311 citri	2IL2	1CWV	1HRN			Sc=5.6304, min distance = 2.000003
INVA_YERPS F	51 2-Oxop	SERA_ECOLI Fu	311 citri	2P9E	1CWV	1YBA		0.76	Sc=5.61763, min distance = 1.39612
INVA_YERPS F	75791 Phe	SRC_HUMAN Ful	311 citri	1O4L	1CWV	1O4P			Sc=5.9065, min distance = 2.068272
IOLG_SALTY F	123927 A3	Q9GT92_CRYPV	5893 nadi	2FM3	3EC7	2EWD		0.97	Sc=6.717, min distance = 1.968984
IOLG_SALTY F	125409 be	XYNA_BACSU Fu	23831 HEP	2B45	3EC7	2QZ3			Sc=5.81049, min distance = 0.62396
IOLG_SALTY F	439153 Di	ADH1B_HUMAN F	5893 nadi	1U3U	3EC7	1DEH		0.79	Sc=6.71778, min distance = 1.78660
IOLG_SALTY F	446050 CI	G3P_HUMAN Ful	5893 nadi	1U8F	3EC7	3GPD		0.95	Sc=6.29213, min distance = 1.07191
IOLG_SALTY F	446288 NA	G3P_PALVE Ful	5893 nadi	1DSS	3EC7	1IHX			Sc=6.60069, min distance = 1.55837
IOLG_SALTY F	5957 Aden	PPNK_ARCFU Fu	5893 nadi	1Z0Z	3EC7	1Z0S			Sc=5.8354, min distance = 1.570559
IOLG_SALTY F	6022 Aden	Q5SI02_THET8	5893 nadi	2BJK	3EC7	2BJA			Sc=6.22929, min distance = 1.99804
IOLG_SALTY F	9547960 A	NPD_THEMA Ful	5893 nadi	2H4H	3EC7	2H59			Sc=5.64251, min distance = 2.49563
IOLG_SALTY F	9547961 3	NPD_THEMA Ful	5893 nadi	2H4H	3EC7	2H59			Sc=5.70077, min distance = 2.14616
IOLS_BACSU F	6022 Aden	HMDH_HUMAN Fu	5886 NADP	1DQA	1PZ0	1HW8			Sc=5.84355, min distance = 2.05669
IP3KA_HUMAN	16046126	CDK2_HUMAN Fu	6022 Aden	1GY3	1W2D	2DS1			Sc=6.23546, min distance = 2.00260
IP3KA_HUMAN	16214825	CDK2_HUMAN Fu	6022 Aden	1GY3	1W2D	2UZE			Sc=6.28879, min distance = 2.16702
IP3KA_HUMAN	23727981	CDK2_HUMAN Fu	6022 Aden	1GY3	1W2D	3BHT			Sc=6.1949, min distance = 1.598547
IP3KA_HUMAN	23727982	CDK2_HUMAN Fu	6022 Aden	1GY3	1W2D	3BHU			Sc=6.25434, min distance = 1.65967
IP3KA_HUMAN	3547 Fasu	ROCK1_HUMAN F	33113 gam	2V55	1W2C	2ESM			Sc=5.90171, min distance = 1.74248

IP3KA_HUMAN I	3973 nche	PIM1_HUMAN Fu	33113 gam	1XR1	1W2C	1YI3			Sc=5.85977, min distance = 2.29940
IP3KA_HUMAN I	4369136 C	CDK2_HUMAN Fu	6022 Aden	1GY3	1W2D	1DM2			Sc=6.18472, min distance = 2.29877
IP3KA_HUMAN I	445479 CI	CSK21_HUMAN Fu	33113 gam	2PVR	1W2C	3BQC			Sc=6.23849, min distance = 2.46948
IP3KA_HUMAN I	445840 di	CDK2_HUMAN Fu	6022 Aden	1GY3	1W2D	1GII			Sc=6.14179, min distance = 2.09098
IP3KA_HUMAN I	445940 O6	CDK2_HUMAN Fu	6022 Aden	1GY3	1W2D	1GZ8			Sc=5.90604, min distance = 2.46530
IP3KA_HUMAN I	4565 1h1r	CDK2_HUMAN Fu	6022 Aden	1GY3	1W2D	1H1R			Sc=6.09988, min distance = 1.23920
IP3KA_HUMAN I	5327121 I	PIM1_HUMAN Fu	33113 gam	1XR1	1W2C	2C3I			Sc=6.1259, min distance = 2.650834
IP3KA_HUMAN I	5331010 c	CDK2_HUMAN Fu	6022 Aden	1GY3	1W2D	2BKZ			Sc=6.20883, min distance = 1.91864
IP3KA_HUMAN I	611002 Op	PIM1_HUMAN Fu	33113 gam	1XR1	1W2C	1YXX			Sc=6.04838, min distance = 2.59165
IP3KA_HUMAN I	6338561 C	MYS2_DICDI Fu	6022 Aden	1VOM	1W2D	1D0X			Sc=6.09392, min distance = 2.22508
IP3KA_HUMAN I	6918710 5	CDK2_HUMAN Fu	6022 Aden	1GY3	1W2D	3EJ1			Sc=6.11551, min distance = 2.33110
IP3KA_HUMAN I	9817550 V	CDK2_HUMAN Fu	6022 Aden	1GY3	1W2D	3BHV			Sc=6.22933, min distance = 2.33550
IP3KA_RAT Fu	16214823	CDK2_HUMAN Fu	6022 Aden	1GY3	1TZD	2UZB			Sc=6.10735, min distance = 1.66891
IP3KA_RAT Fu	23727982	CDK2_HUMAN Fu	6022 Aden	1GY3	1TZD	3BHU			Sc=6.2133, min distance = 2.278528
IP3KA_RAT Fu	398148 1e	CDK2_HUMAN Fu	6022 Aden	1GY3	1TZD	1E1X			Sc=6.05837, min distance = 2.02349
IP3KA_RAT Fu	4369136 C	CDK2_HUMAN Fu	6022 Aden	1GY3	1TZD	1DM2			Sc=6.20041, min distance = 2.30635
IP3KA_RAT Fu	445840 di	CDK2_HUMAN Fu	6022 Aden	1GY3	1TZD	1GII			Sc=6.11198, min distance = 2.52918
IP3KA_RAT Fu	447766 1p	CDK2_HUMAN Fu	6022 Aden	1GY3	1TZD	1P2A			Sc=6.27288, min distance = 2.19568
IP3KA_RAT Fu	447962 1p	CDK2_HUMAN Fu	6022 Aden	1GY3	1TZD	2C5N			Sc=6.10253, min distance = 2.35730
IP3KA_RAT Fu	448005 CI	GSK3B_HUMAN Fu	6022 Aden	1J1C	1TZD	1Q3W			Sc=6.24811, min distance = 2.15441
IP3KA_RAT Fu	5288016 6	CDK2_HUMAN Fu	6022 Aden	1GY3	1TZD	2B53			Sc=6.12903, min distance = 2.18604
IP3KA_RAT Fu	5326739 i	GSK3B_HUMAN Fu	6022 Aden	1J1C	1TZD	1Q41			Sc=6.17865, min distance = 1.89574
IP3KA_RAT Fu	6420139 C	CDK2_HUMAN Fu	6022 Aden	1GY3	1TZD	2C5V			Sc=6.1091, min distance = 1.641487
IP3KA_RAT Fu	6918710 5	CDK2_HUMAN Fu	6022 Aden	1GY3	1TZD	3EJ1			Sc=6.06692, min distance = 2.29728
IP3KB_RAT Fu	10172943	KAPCA_BOVIN Fu	5957 Aden	1Q24	2AQX	2JDS			Sc=6.53435, min distance = 2.07638
IP3KB_RAT Fu	11608401	KAPCA_BOVIN Fu	5957 Aden	1Q24	2AQX	2UVY			Sc=6.18565, min distance = 2.03923
IP3KB_RAT Fu	11708454	KAPCA_BOVIN Fu	5957 Aden	1Q24	2AQX	2VNW			Sc=6.08502, min distance = 2.04293
IP3KB_RAT Fu	15602983	KAPCA_BOVIN Fu	5957 Aden	1Q24	2AQX	2UW5			Sc=6.18541, min distance = 2.06875
IP3KB_RAT Fu	16122635	KAPCA_BOVIN Fu	5957 Aden	1Q24	2AQX	2UW8			Sc=5.80534, min distance = 2.07193
IP3KB_RAT Fu	17751819	KAPCA_BOVIN Fu	5957 Aden	1Q24	2AQX	2VO6			Sc=6.32621, min distance = 2.16819
IP3KB_RAT Fu	24762195	KAPCA_BOVIN Fu	5957 Aden	1Q24	2AQX	2VO0			Sc=6.33407, min distance = 2.22554
IP3KB_RAT Fu	3547 Fasu	KAPCA_BOVIN Fu	5957 Aden	1Q24	2AQX	1Q8W			Sc=5.99721, min distance = 2.11825
IP3KB_RAT Fu	36735 Gpp	Q9RVK2_DEIRA I	5957 Aden	1SU2	2AQX	1SZ3		0.8	Sc=6.42539, min distance = 2.07298
IP3KB_RAT Fu	440317 AT	PURT_ECOLI Fu	5957 Aden	1KJ8	2AQX	1KJJ		0.99	Sc=5.99517, min distance = 2.29733
IP3KB_RAT Fu	447840 CI	PHKG1_RABIT Fu	5957 Aden	1QL6	2AQX	1PHK			Sc=6.01621, min distance = 2.09479
IP3KB_RAT Fu	447916 ac	RIO1_ARCFU Fu	5957 Aden	1ZP9	2AQX	1ZTF		0.94	Sc=6.22742, min distance = 2.19605
IP3KB_RAT Fu	449240 1y	KAPCA_BOVIN Fu	5957 Aden	1Q24	2AQX	1YDR			Sc=6.01898, min distance = 2.03820

IP3KB_RAT Fu	449241 1y	KAPCA_BOVIN F	5957 Aden	1Q24	2AQX	1YDT			Sc=6.17707, min distance = 1.97851
IP3KB_RAT Fu	5280343 c	PK3CG_PIG Ful	5957 Aden	1E8X	2AQX	1E8W			Sc=6.41005, min distance = 1.63740
IP3KB_RAT Fu	6022 Aden	CLCN5_HUMAN F	5957 Aden	2J9L	2AQX	2JA3		0.99	Sc=6.44264, min distance = 1.84062
IP3KB_RAT Fu	6022 Aden	DDL_THET8 Ful	5957 Aden	2ZDQ	2AQX	2ZDH		0.99	Sc=5.68792, min distance = 1.81168
IP3KB_RAT Fu	6022 Aden	FAK1_HUMAN Fu	5957 Aden	2IJM	2AQX	1MP8		0.99	Sc=5.96524, min distance = 1.90328
IP3KB_RAT Fu	6022 Aden	PDK2_HUMAN Fu	5957 Aden	2BU2	2AQX	2BU8		0.99	Sc=5.96283, min distance = 2.22298
IP3KB_RAT Fu	6022 Aden	RK_BOVIN Full	5957 Aden	3C4W	2AQX	3C4Z		0.99	Sc=5.97663, min distance = 2.43756
IP3KB_RAT Fu	6022 Aden	Y059_METJA Fu	5957 Aden	2J9C	2AQX	2J9D		0.99	Sc=6.4207, min distance = 2.386132
IP3KB_RAT Fu	6083 aden	PURP_PYRFU Fu	5957 Aden	2R86	2AQX	2R85		0.98	Sc=6.37349, min distance = 2.52541
IP3KB_RAT Fu	6083 aden	Y059_METJA Fu	5957 Aden	2J9C	2AQX	2J9D		0.98	Sc=6.36475, min distance = 1.84263
IP3KB_RAT Fu	6830 guar	Q381M1_9TRYP	5957 Aden	2Q0D	2AQX	2Q0E		0.8	Sc=5.7442, min distance = 2.097472
IP3KB_RAT Fu	6852207 M	KAPCA_BOVIN F	5957 Aden	1Q24	2AQX	2GNI			Sc=6.00397, min distance = 1.99798
IPMK_YEAST F	15942652	CDK2_HUMAN Fu	6022 Aden	1GY3	2IF8	2DUV			Sc=6.27883, min distance = 2.26412
IPMK_YEAST F	23727982	CDK2_HUMAN Fu	6022 Aden	1GY3	2IF8	3BHU			Sc=6.24086, min distance = 2.14090
IPMK_YEAST F	4369136 C	CDK2_HUMAN Fu	6022 Aden	1GY3	2IF8	1DM2			Sc=6.21983, min distance = 2.50286
IPMK_YEAST F	445840 di	CDK2_HUMAN Fu	6022 Aden	1GY3	2IF8	1GII			Sc=6.10512, min distance = 2.22259
IPMK_YEAST F	447766 1p	CDK2_HUMAN Fu	6022 Aden	1GY3	2IF8	1P2A			Sc=6.27685, min distance = 2.15226
IPMK_YEAST F	447962 1p	CDK2_HUMAN Fu	6022 Aden	1GY3	2IF8	2C5N			Sc=6.11568, min distance = 2.09023
IPMK_YEAST F	448005 CI	GSK3B_HUMAN F	6022 Aden	1J1C	2IF8	1Q3W			Sc=6.251, min distance = 1.9009339
IPMK_YEAST F	6420138 2	CDK2_HUMAN Fu	6022 Aden	1GY3	2IF8	2UUE			Sc=6.2448, min distance = 2.552049
IPTZ_AGRT5 F	24905143	SRC_CHICK Ful	6083 aden	3DQX	2ZE6	3EN7			Sc=6.30668, min distance = 2.40089
IPTZ_AGRT5 F	2519 caff	PYGM_RABIT Fu	6083 aden	8GPB	2ZE6	1GFZ			Sc=5.78631, min distance = 2.02775
IPTZ_AGRT5 F	444818 6-	PURA1_MOUSE F	6083 aden	1MF0	2ZE6	1LON		0.92	Sc=5.93109, min distance = 2.46702
IPTZ_AGRT5 F	445708 AM	NADE_ECOLI Fu	6083 aden	1WXI	2ZE6	1WXE		0.91	Sc=6.34563, min distance = 2.29572
IPTZ_AGRT5 F	5327121 I	PIM1_HUMAN Fu	6083 aden	1YXU	2ZE6	2C3I			Sc=6.29931, min distance = 1.60469
IPTZ_AGRT5 F	65110 AIC	PURP_METJA Fu	6083 aden	2R7M	2ZE6	2R7K		0.79	Sc=6.24971, min distance = 2.15569
IPTZ_AGRT5 F	8582 Inos	PURA1_MOUSE F	6083 aden	1MF0	2ZE6	1IWE		0.79	Sc=5.848, min distance = 2.5944483
IPTZ_AGRT5 F	9549204 C	PURA1_MOUSE F	6083 aden	1MF0	2ZE6	2DGN		0.91	Sc=6.35895, min distance = 2.43803
IPUA_ASPNG F	444139 CI	AMYP_PIG Full	79025 alp	1HX0	2Z8G	1PIG			Sc=5.67002, min distance = 0
IPUA_ASPNG F	444139 CI	CDGT2_BACCI F	79025 alp	1D3C	2Z8G	1DTU			Sc=5.76133, min distance = 0
IPUA_ASPNG F	444809 Th	AMY1_HORVU Fu	79025 alp	1RP8	2Z8G	1P6W		0.78	Sc=5.82954, min distance = 0
IPUA_ASPNG F	446685 Va	CDGT2_BACCI F	79025 alp	1D3C	2Z8G	1KCK			Sc=5.83136, min distance = 0
IPUA_ASPNG F	7027 gluc	Q9C171_PIREQ	79025 alp	1GWM	2Z8G	1W8T			Sc=5.73348, min distance = 0
IPUA_ASPNG F	892 myo-i	SFTPD_HUMAN F	79025 alp	1PWB	2Z8G	2OS9			Sc=5.78361, min distance = 0
IRAK4_HUMAN	10109823	PIM1_HUMAN Fu	444345 1c	1YHS	2NRY	3CY3	26.02		Sc=6.43563, min distance = 2.76491
IRAK4_HUMAN	10138993	CDK2_HUMAN Fu	444345 1c	1AQ1	2NRY	2R64	35.75		Sc=6.38883, min distance = 2.31570
IRAK4_HUMAN	10224714	CDK2_HUMAN Fu	444345 1c	1AQ1	2NRY	3DDP	35.75		Sc=6.40415, min distance = 1.90218

IRAK4_HUMAN I	11348631	CHK1_HUMAN Fu	444345 1c	1NVR	2NRY	2E9U	37.04		Sc=6.213, min distance = 2.7884985
IRAK4_HUMAN I	11502647	CHK1_HUMAN Fu	444345 1c	1NVR	2NRY	2GDO	37.04		Sc=6.37053, min distance = 2.50717
IRAK4_HUMAN I	11553058	LCK_HUMAN Full	444345 1c	1QPD	2NRY	2OF2	41.28		Sc=6.17416, min distance = 2.29474
IRAK4_HUMAN I	11690363	CHK1_HUMAN Fu	444345 1c	1NVR	2NRY	2HY0	37.04		Sc=6.50334, min distance = 2.05960
IRAK4_HUMAN I	11986113	PIM1_HUMAN Fu	444345 1c	1YHS	2NRY	2BZH	26.02	0.76	Sc=6.49921, min distance = 2.39539
IRAK4_HUMAN I	11986114	PIM1_HUMAN Fu	444345 1c	1YHS	2NRY	2BZI	26.02		Sc=6.51103, min distance = 2.23792
IRAK4_HUMAN I	1400 nche	HCK_HUMAN Full	33113 gam	1AD5	2OID	1QCF	30.71		Sc=6.20471, min distance = 1.33889
IRAK4_HUMAN I	1540 1h1q	CDK2_HUMAN Fu	444345 1c	1AQ1	2NRY	1H1Q	35.75		Sc=6.25142, min distance = 2.56572
IRAK4_HUMAN I	15602982	KAPCA_BOVIN Fu	444345 1c	1STC	2NRY	2UW6	35		Sc=6.09998, min distance = 2.12427
IRAK4_HUMAN I	15942652	CDK2_HUMAN Fu	444345 1c	1AQ1	2NRY	2DUV	35.75		Sc=6.31512, min distance = 2.20258
IRAK4_HUMAN I	15942671	FAK1_CHICK Fu	33113 gam	2J0L	2OID	2J0J	33.75		Sc=6.34152, min distance = 1.30029
IRAK4_HUMAN I	15991572	LCK_HUMAN Full	444345 1c	1QPD	2NRY	2OF4	41.28		Sc=6.15318, min distance = 2.87703
IRAK4_HUMAN I	160355 rc	CDK2_HUMAN Fu	444345 1c	1AQ1	2NRY	3DDQ	35.75		Sc=6.26534, min distance = 2.16170
IRAK4_HUMAN I	16058617	CHK1_HUMAN Fu	444345 1c	1NVR	2NRY	2HOG	37.04		Sc=6.31278, min distance = 2.61558
IRAK4_HUMAN I	16058649	IGF1R_HUMAN Fu	33113 gam	1JQH	2OID	2OJ9	36.49		Sc=6.56614, min distance = 1.60817
IRAK4_HUMAN I	16122608	PDPK1_HUMAN Fu	444345 1c	1OKY	2NRY	2PE1	39.02		Sc=6.33095, min distance = 2.89586
IRAK4_HUMAN I	16122643	CHK1_HUMAN Fu	444345 1c	1NVR	2NRY	2YWP	37.04		Sc=6.27445, min distance = 2.74391
IRAK4_HUMAN I	16750062	CSK2A_MAIZE Fu	33113 gam	1LP4	2OID	2OXD	26.76		Sc=5.61713, min distance = 2.60726
IRAK4_HUMAN I	17754396	PDPK1_HUMAN Fu	444345 1c	1OKY	2NRY	2R7B	39.02		Sc=6.28314, min distance = 2.73102
IRAK4_HUMAN I	188966 dA	HSLU_ECOLI Fu	33113 gam	1E94	2OID	1G4A		0.97	Sc=6.29104, min distance = 2.18933
IRAK4_HUMAN I	1907917 2	CHK1_HUMAN Fu	444345 1c	1NVR	2NRY	2CGW	37.04		Sc=6.1238, min distance = 2.960064
IRAK4_HUMAN I	23653515	CDK2_HUMAN Fu	444345 1c	1AQ1	2NRY	2R3F	35.75		Sc=5.98555, min distance = 2.60252
IRAK4_HUMAN I	23653516	CDK2_HUMAN Fu	444345 1c	1AQ1	2NRY	2R3G	35.75		Sc=6.10593, min distance = 2.66967
IRAK4_HUMAN I	23653518	CDK2_HUMAN Fu	444345 1c	1AQ1	2NRY	2R3J	35.75		Sc=6.20519, min distance = 2.70848
IRAK4_HUMAN I	23653519	CDK2_HUMAN Fu	444345 1c	1AQ1	2NRY	2R3K	35.75		Sc=6.21168, min distance = 2.48323
IRAK4_HUMAN I	23653522	CDK2_HUMAN Fu	444345 1c	1AQ1	2NRY	2R3N	35.75		Sc=6.27155, min distance = 2.27995
IRAK4_HUMAN I	23653524	CDK2_HUMAN Fu	444345 1c	1AQ1	2NRY	2R3P	35.75		Sc=6.29497, min distance = 2.05177
IRAK4_HUMAN I	23653526	CDK2_HUMAN Fu	444345 1c	1AQ1	2NRY	2R3R	35.75		Sc=6.19165, min distance = 2.62348
IRAK4_HUMAN I	23657799	CHK1_HUMAN Fu	444345 1c	1NVR	2NRY	2E9O	37.04		Sc=6.48093, min distance = 2.65138
IRAK4_HUMAN I	23657800	CHK1_HUMAN Fu	444345 1c	1NVR	2NRY	2E9P	37.04		Sc=6.27157, min distance = 2.74986
IRAK4_HUMAN I	23727982	CDK2_HUMAN Fu	444345 1c	1AQ1	2NRY	3BHU	35.75		Sc=6.26096, min distance = 2.22974
IRAK4_HUMAN I	2396 bisi	PIM1_HUMAN Fu	33113 gam	1XR1	2OID	1XWS	26.02		Sc=6.33062, min distance = 2.11581
IRAK4_HUMAN I	24752838	CHK1_HUMAN Fu	444345 1c	1NVR	2NRY	2QHM	37.04		Sc=6.05296, min distance = 3.00624
IRAK4_HUMAN I	24779674	CSK2A_MAIZE Fu	33113 gam	1LP4	2OID	2PVH	26.76		Sc=5.99877, min distance = 2.49930
IRAK4_HUMAN I	24779677	CSK2A_MAIZE Fu	33113 gam	1LP4	2OID	2PVL	26.76		Sc=6.26491, min distance = 2.45925
IRAK4_HUMAN I	24779678	CSK2A_MAIZE Fu	33113 gam	1LP4	2OID	2PVM	26.76		Sc=6.38989, min distance = 1.29085
IRAK4_HUMAN I	24820122	PIM1_HUMAN Fu	444345 1c	1YHS	2NRY	3BWF	26.02		Sc=6.51656, min distance = 2.27421

IRAK4_HUMAN	24851689	PIM1_HUMAN Fu	33113 gar	1XR1	2OID	3CY2	26.02		Sc=6.18243, min distance = 2.07218
IRAK4_HUMAN	24864081	CDK2_HUMAN Fu	444345 1c	1AQ1	2NRY	2VTS	35.75		Sc=6.28487, min distance = 2.13873
IRAK4_HUMAN	24901723	CDK2_HUMAN Fu	444345 1c	1AQ1	2NRY	2W06	35.75		Sc=6.19381, min distance = 1.77390
IRAK4_HUMAN	24905143	PK3CG_HUMAN Fu	444345 1c	1E8Z	2NRY	2V4L			Sc=5.99448, min distance = 2.99346
IRAK4_HUMAN	24905144	PK3CG_HUMAN Fu	444345 1c	1E8Z	2NRY	3ENE			Sc=6.09651, min distance = 2.55310
IRAK4_HUMAN	24916751	CDK2_HUMAN Fu	444345 1c	1AQ1	2NRY	3DOG	35.75		Sc=6.26308, min distance = 2.10557
IRAK4_HUMAN	24963033	CDK2_HUMAN Fu	444345 1c	1AQ1	2NRY	2W05	35.75		Sc=6.21777, min distance = 2.33100
IRAK4_HUMAN	3064778 H	ROCK1_HUMAN Fu	33113 gar	2V55	2OID	2ETK	33.59		Sc=6.05783, min distance = 2.41567
IRAK4_HUMAN	3543 1g5s	CDK2_HUMAN Fu	444345 1c	1AQ1	2NRY	1G5S	35.75		Sc=6.36836, min distance = 2.43712
IRAK4_HUMAN	3547 Fasu	KAPCA_BOVIN Fu	444345 1c	1STC	2NRY	1Q8W	35		Sc=5.91979, min distance = 2.73667
IRAK4_HUMAN	36735 Gpp	CSK2A_MAIZE Fu	33113 gar	1LP4	2OID	1DAY	26.76	0.8	Sc=5.98597, min distance = 2.49724
IRAK4_HUMAN	4369136 C	CDK2_HUMAN Fu	444345 1c	1AQ1	2NRY	1DM2	35.75		Sc=6.24611, min distance = 2.94143
IRAK4_HUMAN	444367 CI	CSK2A_MAIZE Fu	33113 gar	1LP4	2OID	1OM1	26.76		Sc=6.12811, min distance = 1.39738
IRAK4_HUMAN	445966 O6	CDK2_HUMAN Fu	444345 1c	1AQ1	2NRY	1H0V	35.75		Sc=6.07485, min distance = 2.88060
IRAK4_HUMAN	447649 1c	CDK2_HUMAN Fu	444345 1c	1AQ1	2NRY	1OI9	35.75		Sc=6.34252, min distance = 2.54736
IRAK4_HUMAN	447653 CI	CDK2_HUMAN Fu	444345 1c	1AQ1	2NRY	1OIR	35.75		Sc=6.33232, min distance = 3.13391
IRAK4_HUMAN	447654 CI	CDK2_HUMAN Fu	444345 1c	1AQ1	2NRY	1OIT	35.75		Sc=6.15371, min distance = 1.73879
IRAK4_HUMAN	447655 1c	CDK2_HUMAN Fu	444345 1c	1AQ1	2NRY	1OIU	35.75		Sc=6.18239, min distance = 2.56707
IRAK4_HUMAN	447766 1p	CDK2_HUMAN Fu	444345 1c	1AQ1	2NRY	1P2A	35.75		Sc=6.30385, min distance = 2.19460
IRAK4_HUMAN	447962 1p	CDK2_HUMAN Fu	444345 1c	1AQ1	2NRY	2C5N	35.75		Sc=6.11439, min distance = 2.57190
IRAK4_HUMAN	448005 CI	GSK3B_HUMAN Fu	33113 gar	1J1B	2OID	1Q3W	35.29		Sc=6.26018, min distance = 1.86404
IRAK4_HUMAN	448008 CI	GSK3B_HUMAN Fu	33113 gar	1J1B	2OID	1Q4L	35.29		Sc=6.36346, min distance = 1.62578
IRAK4_HUMAN	448008 CI	GSK3B_HUMAN Fu	444345 1c	1Q3D	2NRY	1Q4L	35.29		Sc=6.35401, min distance = 2.52666
IRAK4_HUMAN	448014 1c	GSK3B_HUMAN Fu	33113 gar	1J1B	2OID	1Q5K	35.29		Sc=6.2226, min distance = 2.237104
IRAK4_HUMAN	448042 2e	ROCK1_HUMAN Fu	33113 gar	2V55	2OID	2ETR	33.59		Sc=6.02432, min distance = 2.23646
IRAK4_HUMAN	448043 2g	KAPCA_BOVIN Fu	444345 1c	1STC	2NRY	1Q8U	35		Sc=5.97973, min distance = 2.63467
IRAK4_HUMAN	448043 2g	ROCK1_HUMAN Fu	33113 gar	2V55	2OID	3D9V	33.59		Sc=5.97844, min distance = 2.58429
IRAK4_HUMAN	448293 CI	CDK2_HUMAN Fu	444345 1c	1AQ1	2NRY	1R78	35.75		Sc=6.45251, min distance = 1.57625
IRAK4_HUMAN	449240 1y	KAPCA_BOVIN Fu	444345 1c	1STC	2NRY	1YDR	35		Sc=5.92379, min distance = 2.55884
IRAK4_HUMAN	456214 pu	KS6A1_HUMAN Fu	444345 1c	2Z7R	2NRY	2Z7S			Sc=6.27985, min distance = 2.07706
IRAK4_HUMAN	456214 pu	SRC_HUMAN Ful	33113 gar	2SRC	2OID	1YOM	40		Sc=6.32395, min distance = 1.42197
IRAK4_HUMAN	4566 1h1s	CDK2_HUMAN Fu	444345 1c	1AQ1	2NRY	1H1S	35.75		Sc=6.30357, min distance = 1.29843
IRAK4_HUMAN	5281672 m	PIM1_HUMAN Fu	444345 1c	1YHS	2NRY	2O63	26.02		Sc=6.4081, min distance = 2.030271
IRAK4_HUMAN	5281680 Q	PIM1_HUMAN Fu	33113 gar	1XR1	2OID	2O64	26.02		Sc=6.42683, min distance = 1.84415
IRAK4_HUMAN	5287844 i	GSK3B_HUMAN Fu	33113 gar	1J1B	2OID	1UV5	35.29		Sc=6.30549, min distance = 2.12663
IRAK4_HUMAN	5326739 i	GSK3B_HUMAN Fu	33113 gar	1J1B	2OID	1Q41	35.29		Sc=6.24957, min distance = 2.46086
IRAK4_HUMAN	5326976 1	CSK2A_MAIZE Fu	33113 gar	1LP4	2OID	1ZOE	26.76		Sc=5.61588, min distance = 3.02517

IRAK4_HUMAN I	5327122 C	CHK1_HUMAN Fu	444345 1c	1NVR	2NRY	2C3K	37.04		Sc=6.48266, min distance = 1.26567
IRAK4_HUMAN I	5327123 I	CHK1_HUMAN Fu	444345 1c	1NVR	2NRY	2C3L	37.04		Sc=6.11735, min distance = 2.68057
IRAK4_HUMAN I	5327130 C	CDK2_HUMAN Fu	444345 1c	1AQ1	2NRY	2C68	35.75		Sc=6.21699, min distance = 1.92849
IRAK4_HUMAN I	5327131 T	CDK2_HUMAN Fu	444345 1c	1AQ1	2NRY	2C69	35.75		Sc=6.19527, min distance = 1.91606
IRAK4_HUMAN I	5331010 c	CDK2_HUMAN Fu	444345 1c	1AQ1	2NRY	2BKZ	35.75		Sc=6.21838, min distance = 2.21961
IRAK4_HUMAN I	5494414 C	CDK2_HUMAN Fu	444345 1c	1AQ1	2NRY	2A0C	35.75		Sc=6.41339, min distance = 2.35515
IRAK4_HUMAN I	6083 aden	HSP71_HUMAN Fu	33113 gam	2E8A	2OID	1XQS		0.97	Sc=6.23953, min distance = 2.08624
IRAK4_HUMAN I	6083 aden	PIM1_HUMAN Fu	444345 1c	1YHS	2NRY	1YXU	26.02		Sc=6.33641, min distance = 2.30378
IRAK4_HUMAN I	6083 aden	Q6L8F0_THETH I	33113 gam	1V25	2OID	1V26		0.97	Sc=6.2676, min distance = 2.375834
IRAK4_HUMAN I	60961 ade	PIM1_HUMAN Fu	33113 gam	1XR1	2OID	1YI4	26.02	0.93	Sc=6.15985, min distance = 1.71125
IRAK4_HUMAN I	60961 ade	PIM1_HUMAN Fu	444345 1c	1YHS	2NRY	1YI4	26.02		Sc=6.1349, min distance = 2.961697
IRAK4_HUMAN I	60961 ade	SKY1_YEAST Fu	33113 gam	1Q99	2OID	1Q97		0.93	Sc=6.15803, min distance = 2.91631
IRAK4_HUMAN I	611002 Op	PIM1_HUMAN Fu	33113 gam	1XR1	2OID	1YXX	26.02		Sc=6.04186, min distance = 2.60668
IRAK4_HUMAN I	6420139 C	CDK2_HUMAN Fu	444345 1c	1AQ1	2NRY	2C5V	35.75		Sc=6.11139, min distance = 2.33698
IRAK4_HUMAN I	6420140 2	CDK2_HUMAN Fu	444345 1c	1AQ1	2NRY	2C5Y	35.75		Sc=6.43862, min distance = 2.61698
IRAK4_HUMAN I	6540272 C	CHK1_HUMAN Fu	444345 1c	1NVR	2NRY	2CGU	37.04		Sc=6.37484, min distance = 2.59319
IRAK4_HUMAN I	6540273 2	CHK1_HUMAN Fu	444345 1c	1NVR	2NRY	2CGV	37.04		Sc=6.13393, min distance = 2.32329
IRAK4_HUMAN I	657072 RY	CDK2_HUMAN Fu	444345 1c	1AQ1	2NRY	2BHH	35.75		Sc=6.454, min distance = 2.751771
IRAK4_HUMAN I	657138 1z	PDPK1_HUMAN Fu	444345 1c	1OKY	2NRY	1Z5M	39.02		Sc=6.66265, min distance = 2.07727
IRAK4_HUMAN I	6852201 2	CDK2_HUMAN Fu	444345 1c	1AQ1	2NRY	2G9X	35.75		Sc=6.45765, min distance = 2.11453
IRAK4_HUMAN I	6852207 M	KAPCA_BOVIN Fu	444345 1c	1STC	2NRY	2GNI	35		Sc=5.91374, min distance = 2.46264
IRAK4_HUMAN I	6914568 C	CHK1_HUMAN Fu	444345 1c	1NVR	2NRY	1ZYS	37.04		Sc=6.48043, min distance = 2.20633
IRAK4_HUMAN I	72271 7-H	CDK2_HUMAN Fu	444345 1c	1AQ1	2NRY	1PKD	35.75	0.91	Sc=6.47702, min distance = 2.15044
IRAK4_HUMAN I	91532 AMP	IGF1R_HUMAN Fu	33113 gam	1JQH	2OID	1K3A	36.49	0.99	Sc=6.41949, min distance = 2.09255
IRAK4_HUMAN I	91532 AMP	KIF1A_MOUSE Fu	33113 gam	1VFV	2OID	1I6I		0.99	Sc=5.99986, min distance = 2.19120
IRAK4_HUMAN I	91532 AMP	PURT_ECOLI Fu	33113 gam	1EYZ	2OID	1KJI		0.99	Sc=6.44013, min distance = 1.82542
IRAK4_HUMAN I	9547890 I	CDK2_HUMAN Fu	444345 1c	1AQ1	2NRY	1W8C	35.75		Sc=6.20235, min distance = 2.35894
IRE1_YEAST Fu	11957417	CDK2_HUMAN Fu	6022 Aden	1GY3	2RIO	2I40	30.3		Sc=6.31112, min distance = 1.90196
IRE1_YEAST Fu	1540 1h1q	CDK2_HUMAN Fu	6022 Aden	1GY3	2RIO	1H1Q	30.3		Sc=6.40622, min distance = 1.47537
IRE1_YEAST Fu	15942652	CDK2_HUMAN Fu	6022 Aden	1GY3	2RIO	2DUV	30.3		Sc=6.32552, min distance = 2.38206
IRE1_YEAST Fu	160355 ro	CDK2_HUMAN Fu	6022 Aden	1GY3	2RIO	3DDQ	30.3		Sc=6.41938, min distance = 0.72714
IRE1_YEAST Fu	16214823	CDK2_HUMAN Fu	6022 Aden	1GY3	2RIO	2UZB	30.3		Sc=6.16348, min distance = 1.59661
IRE1_YEAST Fu	16214825	CDK2_HUMAN Fu	6022 Aden	1GY3	2RIO	2UZE	30.3		Sc=6.29726, min distance = 2.74733
IRE1_YEAST Fu	23653515	CDK2_HUMAN Fu	6022 Aden	1GY3	2RIO	2R3F	30.3		Sc=6.0749, min distance = 2.222575
IRE1_YEAST Fu	23653518	CDK2_HUMAN Fu	6022 Aden	1GY3	2RIO	2R3J	30.3		Sc=6.25253, min distance = 2.24899
IRE1_YEAST Fu	23653519	CDK2_HUMAN Fu	6022 Aden	1GY3	2RIO	2R3K	30.3		Sc=6.26271, min distance = 2.14339
IRE1_YEAST Fu	23653520	CDK2_HUMAN Fu	6022 Aden	1GY3	2RIO	2R3L	30.3		Sc=6.25365, min distance = 2.21591

IRE1_YEAST Fu	23653521	CDK2_HUMAN Ful	6022 Aden	1GY3	2RIO	2R3M	30.3		Sc=6.45007, min distance = 2.158075
IRE1_YEAST Fu	23653522	CDK2_HUMAN Ful	6022 Aden	1GY3	2RIO	2R3N	30.3		Sc=6.32697, min distance = 2.518542
IRE1_YEAST Fu	23653524	CDK2_HUMAN Ful	6022 Aden	1GY3	2RIO	2R3P	30.3		Sc=6.31072, min distance = 2.280267
IRE1_YEAST Fu	23653526	CDK2_HUMAN Ful	6022 Aden	1GY3	2RIO	2R3R	30.3		Sc=6.26593, min distance = 2.262867
IRE1_YEAST Fu	24864078	CDK2_HUMAN Ful	6022 Aden	1GY3	2RIO	2VTO	30.3		Sc=6.40312, min distance = 2.307281
IRE1_YEAST Fu	3543 1g5s	CDK2_HUMAN Ful	6022 Aden	1GY3	2RIO	1G5S	30.3		Sc=6.46313, min distance = 2.284892
IRE1_YEAST Fu	398148 1e	CDK2_HUMAN Ful	6022 Aden	1GY3	2RIO	1E1X	30.3		Sc=6.17532, min distance = 1.701568
IRE1_YEAST Fu	4369136 C	CDK2_HUMAN Ful	6022 Aden	1GY3	2RIO	1DM2	30.3		Sc=6.23385, min distance = 2.383195
IRE1_YEAST Fu	444345 1d	CDK2_HUMAN Ful	6022 Aden	1GY3	2RIO	1AQ1	30.3		Sc=6.3596, min distance = 2.0985506
IRE1_YEAST Fu	444564 AD	MYS2_DICDI Ful	6022 Aden	1VOM	2RIO	1W9I		0.91	Sc=6.39587, min distance = 2.238275
IRE1_YEAST Fu	445840 di	CDK2_HUMAN Ful	6022 Aden	1GY3	2RIO	1GII	30.3		Sc=6.23524, min distance = 2.247115
IRE1_YEAST Fu	445966 O6	CDK2_HUMAN Ful	6022 Aden	1GY3	2RIO	1H0V	30.3		Sc=6.17941, min distance = 2.072969
IRE1_YEAST Fu	447652 Im	CDK2_HUMAN Ful	6022 Aden	1GY3	2RIO	1OIQ	30.3		Sc=6.13286, min distance = 2.306813
IRE1_YEAST Fu	447655 1o	CDK2_HUMAN Ful	6022 Aden	1GY3	2RIO	1OIU	30.3		Sc=6.3977, min distance = 1.1830646
IRE1_YEAST Fu	447766 1p	CDK2_HUMAN Ful	6022 Aden	1GY3	2RIO	1P2A	30.3		Sc=6.31734, min distance = 2.345911
IRE1_YEAST Fu	447821 CI	CDK2_HUMAN Ful	6022 Aden	1GY3	2RIO	1PF8	30.3		Sc=6.12862, min distance = 2.207747
IRE1_YEAST Fu	447916 ac	RIO1_ARCFU Ful	6022 Aden	1ZTH	2RIO	1ZTF		0.95	Sc=6.17756, min distance = 2.796612
IRE1_YEAST Fu	447962 1p	CDK2_HUMAN Ful	6022 Aden	1GY3	2RIO	2C5N	30.3		Sc=6.27915, min distance = 1.158385
IRE1_YEAST Fu	447967 CI	CDK2_HUMAN Ful	6022 Aden	1GY3	2RIO	1PYE	30.3		Sc=6.35941, min distance = 2.272829
IRE1_YEAST Fu	449087 1v	CDK2_HUMAN Ful	6022 Aden	1GY3	2RIO	1VYW	30.3		Sc=6.29126, min distance = 2.097918
IRE1_YEAST Fu	449088 1v	CDK2_HUMAN Ful	6022 Aden	1GY3	2RIO	1VYZ	30.3		Sc=6.13164, min distance = 2.425621
IRE1_YEAST Fu	4564 1e1v	CDK2_HUMAN Ful	6022 Aden	1GY3	2RIO	1E1V	30.3		Sc=6.1411, min distance = 2.0391454
IRE1_YEAST Fu	4565 1h1r	CDK2_HUMAN Ful	6022 Aden	1GY3	2RIO	1H1R	30.3		Sc=6.36022, min distance = 1.7323466
IRE1_YEAST Fu	5287845 N	CDK2_HUMAN Ful	6022 Aden	1GY3	2RIO	2BHE	30.3		Sc=6.22185, min distance = 2.316096
IRE1_YEAST Fu	5288641 i	CDK2_HUMAN Ful	6022 Aden	1GY3	2RIO	1E9H	30.3		Sc=6.27113, min distance = 2.585430
IRE1_YEAST Fu	5327124	CDK2_HUMAN Ful	6022 Aden	1GY3	2RIO	2C4G	30.3		Sc=6.28487, min distance = 2.106187
IRE1_YEAST Fu	5327148 C	IPKA_RABIT Ful	6022 Aden	1JBP	2RIO	2ERZ			Sc=6.12828, min distance = 2.413642
IRE1_YEAST Fu	5494414 C	CDK2_HUMAN Ful	6022 Aden	1GY3	2RIO	2A0C	30.3		Sc=6.4896, min distance = 2.1444535
IRE1_YEAST Fu	5957 Aden	HSLU_ECOLI Ful	6022 Aden	1HQY	2RIO	1DO0		0.99	Sc=6.41442, min distance = 1.544215
IRE1_YEAST Fu	5957 Aden	MALK_ECOLI Ful	6022 Aden	2AWN	2RIO	1Q12		0.99	Sc=6.42875, min distance = 2.195448
IRE1_YEAST Fu	5957 Aden	RK_BOVIN Full=	6022 Aden	3C4Z	2RIO	3C4W		0.99	Sc=5.98265, min distance = 2.610759
IRE1_YEAST Fu	6083 aden	PURP_METJA Ful	6022 Aden	2R7N	2RIO	2R7M		0.99	Sc=5.86642, min distance = 2.569624
IRE1_YEAST Fu	656972 CT	CDK2_HUMAN Ful	6022 Aden	1GY3	2RIO	1Y91	30.3		Sc=6.41549, min distance = 1.931149
IRE1_YEAST Fu	72194 2-C	ENPL_CANFA Ful	6022 Aden	1TC6	2RIO	1QYE		0.89	Sc=6.07254, min distance = 2.321857
IRE1_YEAST Fu	91532 AMP	MTNK_BACSU Ful	6022 Aden	2OLC	2RIO	2PUL		0.99	Sc=6.46427, min distance = 2.249761
IRE1_YEAST Fu	9991833 S	CDK2_HUMAN Ful	6022 Aden	1GY3	2RIO	2R3H	30.3		Sc=6.11262, min distance = 2.708568
ISDC_STAA8 Fu	11957385	MYG_PHYCA Ful	444098 HE	1A6M	2O6P	2CMM			Sc=6.12595, min distance = 1.901075

ISDG_STAAN Fu	11957355	CY1_BOVIN Full	444098 HE	1L0N	2ZDO	1L0L		0.77	Sc=6.63037, min distance = 1.27973
ISDG_STAAN Fu	11957360	CY1_BOVIN Full	444098 HE	1L0N	2ZDO	1NTM			Sc=6.61541, min distance = 1.58082
ISDG_STAAN Fu	11957385	MYG_PHYCA Full	444098 HE	1A6M	2ZDO	2CMM			Sc=6.19212, min distance = 2.20337
ISDG_STAAN Fu	11970242	CCPR_YEAST Fu	444098 HE	2EUT	2ZDO	1CPE		0.83	Sc=6.692, min distance = 2.0858823
ISDH_STAAB Fu	11957385	MYG_PHYCA Full	444098 HE	1A6M	2Z6F	2CMM			Sc=6.08257, min distance = 2.47821
ISPA_ECOLI Fu	11266583	FPPS_HUMAN Fu	5245 Rise	2QIS	1RQJ	2OPM		0.84	Sc=6.05283, min distance = 2.24101
ISPA_ECOLI Fu	130956 Mi	FPPS_HUMAN Fu	1195 isop	1ZW5	1RQJ	3B7L			Sc=6.2709, min distance = 2.319880
ISPA_ECOLI Fu	16750060	FPPS_HUMAN Fu	1195 isop	1ZW5	1RQJ	2OPN			Sc=5.7696, min distance = 2.195965
ISPA_ECOLI Fu	16750107	FPPS_HUMAN Fu	1195 isop	1ZW5	1RQJ	2RAH			Sc=6.23391, min distance = 2.24934
ISPA_ECOLI Fu	4674 pami	FPPS_HUMAN Fu	1195 isop	1ZW5	1RQJ	2F89			Sc=5.70193, min distance = 2.48119
ISPA_ECOLI Fu	4674 pami	FPPS_HUMAN Fu	5245 Rise	2QIS	1RQJ	2F89			Sc=5.67424, min distance = 2.37540
ISPD_ECOLI Fu	6131 cyti	KPSU5_ECOLX Fu	6176 Cyti	1H7G	1I52	1H7F		0.98	Sc=6.27868, min distance = 2.11375
ISPD_ECOLI Fu	6132 Cyti	KPSU5_ECOLX Fu	6176 Cyti	1H7G	1I52	1H7H		0.99	Sc=6.34612, min distance = 2.04004
ISPD_THEMA Fu	6131 cyti	KPSU5_ECOLX Fu	6176 Cyti	1H7G	1VPA	1H7F		0.98	Sc=6.29152, min distance = 1.67304
ISPD_THEMA Fu	6132 Cyti	KPSU5_ECOLX Fu	6176 Cyti	1H7G	1VPA	1H7H		0.99	Sc=6.36324, min distance = 2.08626
ISPE_AQUAE Fu	440317 AT	VPS4_YEAST Fu	6022 Aden	2QPA	2V8P	3EIH		0.99	Sc=6.00956, min distance = 1.62151
ISPE_AQUAE Fu	446090 1h	NDKC_DICDI Fu	6022 Aden	1KDN	2V8P	1HIY		0.97	Sc=5.94326, min distance = 2.16333
ISPE_AQUAE Fu	447017 CI	NDKC_DICDI Fu	6022 Aden	1KDN	2V8P	1LWX			Sc=5.87822, min distance = 2.02237
ISPE_AQUAE Fu	6133 urid	UCK2_HUMAN Fu	6175 cyti	1UEJ	2V34	1UEI		0.75	Sc=5.63022, min distance = 1.82198
ISPE_AQUAE Fu	6176 Cyti	UCK2_HUMAN Fu	6175 cyti	1UEJ	2V34	1UDW		0.9	Sc=5.6104, min distance = 2.182017
ITK_HUMAN Fu	10125830	CHK1_HUMAN Fu	444345 1c	1NVR	1SM2	2AYP			Sc=6.38334, min distance = 2.63818
ITK_HUMAN Fu	10224714	CDK2_HUMAN Fu	444345 1c	1AQ1	1SM2	3DDP			Sc=6.47674, min distance = 2.30505
ITK_HUMAN Fu	11175137	KAPCA_BOVIN Fu	444345 1c	1STC	1SM2	2UW7			Sc=6.1997, min distance = 1.958679
ITK_HUMAN Fu	11348631	CHK1_HUMAN Fu	444345 1c	1NVR	1SM2	2E9U			Sc=6.23509, min distance = 2.63326
ITK_HUMAN Fu	11502647	CHK1_HUMAN Fu	444345 1c	1NVR	1SM2	2GDO			Sc=6.37621, min distance = 2.30407
ITK_HUMAN Fu	11553058	LCK_HUMAN Full	444345 1c	1QPD	1SM2	2OF2			Sc=6.30229, min distance = 2.00942
ITK_HUMAN Fu	11696113	KAPCA_BOVIN Fu	444345 1c	1STC	1SM2	2VO3			Sc=6.32395, min distance = 1.56994
ITK_HUMAN Fu	11992146	MAPK2_HUMAN Fu	444345 1c	2PZY	1SM2	2P3G			Sc=6.29792, min distance = 2.25147
ITK_HUMAN Fu	1540 1h1c	CDK2_HUMAN Fu	444345 1c	1AQ1	1SM2	1H1Q			Sc=6.26593, min distance = 1.72261
ITK_HUMAN Fu	15602982	KAPCA_BOVIN Fu	444345 1c	1STC	1SM2	2UW6			Sc=6.08317, min distance = 2.45627
ITK_HUMAN Fu	15602983	KAPCA_BOVIN Fu	444345 1c	1STC	1SM2	2UW5			Sc=6.12205, min distance = 1.49081
ITK_HUMAN Fu	15942652	CDK2_HUMAN Fu	444345 1c	1AQ1	1SM2	2DUV			Sc=6.31323, min distance = 2.77847
ITK_HUMAN Fu	15991572	LCK_HUMAN Full	444345 1c	1QPD	1SM2	2OF4			Sc=6.27354, min distance = 1.49970
ITK_HUMAN Fu	160355 rc	CDK2_HUMAN Fu	444345 1c	1AQ1	1SM2	3DDQ			Sc=6.35644, min distance = 1.88366
ITK_HUMAN Fu	16040274	KAPCA_BOVIN Fu	444345 1c	1STC	1SM2	2OJF			Sc=6.52543, min distance = 2.23857
ITK_HUMAN Fu	16122608	PDPK1_HUMAN Fu	444345 1c	1OKY	1SM2	2PE1			Sc=6.34671, min distance = 2.44010
ITK_HUMAN Fu	16122633	KAPCA_BOVIN Fu	444345 1c	1STC	1SM2	2UW0			Sc=6.45246, min distance = 1.74951

ITK_HUMAN Fu	16122642	KAPCA_BOVIN Fu	444345 1c	1STC	1SM2	2UZW		Sc=6.37409, min distance = 2.35389
ITK_HUMAN Fu	16122643	CHK1_HUMAN Fu	444345 1c	1NVR	1SM2	2YWP		Sc=6.28077, min distance = 2.58653
ITK_HUMAN Fu	16214823	CDK2_HUMAN Fu	444345 1c	1AQ1	1SM2	2UZB		Sc=6.09911, min distance = 2.28421
ITK_HUMAN Fu	16214825	CDK2_HUMAN Fu	444345 1c	1AQ1	1SM2	2UZE		Sc=6.2671, min distance = 2.310441
ITK_HUMAN Fu	16214827	CDK2_HUMAN Fu	444345 1c	1AQ1	1SM2	2UZN		Sc=6.0864, min distance = 2.561346
ITK_HUMAN Fu	16214828	CDK2_HUMAN Fu	444345 1c	1AQ1	1SM2	2UZO		Sc=6.08061, min distance = 2.31105
ITK_HUMAN Fu	16758227	CHK1_HUMAN Fu	444345 1c	1NVR	1SM2	2R0U		Sc=6.55315, min distance = 2.09826
ITK_HUMAN Fu	1707 1fvt	CDK2_HUMAN Fu	444345 1c	1AQ1	1SM2	1FVT		Sc=6.11218, min distance = 2.15407
ITK_HUMAN Fu	1907917 2	CHK1_HUMAN Fu	444345 1c	1NVR	1SM2	2CGW		Sc=6.16831, min distance = 2.72144
ITK_HUMAN Fu	23653515	CDK2_HUMAN Fu	444345 1c	1AQ1	1SM2	2R3F		Sc=6.03316, min distance = 2.48829
ITK_HUMAN Fu	23653516	CDK2_HUMAN Fu	444345 1c	1AQ1	1SM2	2R3G		Sc=6.20786, min distance = 2.69433
ITK_HUMAN Fu	23653518	CDK2_HUMAN Fu	444345 1c	1AQ1	1SM2	2R3J		Sc=6.21437, min distance = 2.45348
ITK_HUMAN Fu	23653519	CDK2_HUMAN Fu	444345 1c	1AQ1	1SM2	2R3K		Sc=6.10993, min distance = 2.29193
ITK_HUMAN Fu	23653520	CDK2_HUMAN Fu	444345 1c	1AQ1	1SM2	2R3L		Sc=6.20648, min distance = 2.44322
ITK_HUMAN Fu	23653521	CDK2_HUMAN Fu	444345 1c	1AQ1	1SM2	2R3M		Sc=6.37599, min distance = 2.33427
ITK_HUMAN Fu	23657800	CHK1_HUMAN Fu	444345 1c	1NVR	1SM2	2E9P		Sc=6.27734, min distance = 2.38488
ITK_HUMAN Fu	23727982	CDK2_HUMAN Fu	444345 1c	1AQ1	1SM2	3BHU		Sc=6.2587, min distance = 2.481677
ITK_HUMAN Fu	2403 1uvr	PDPK1_HUMAN Fu	444345 1c	1OKY	1SM2	1UVR		Sc=6.3943, min distance = 2.145851
ITK_HUMAN Fu	24752838	CHK1_HUMAN Fu	444345 1c	1NVR	1SM2	2QHM		Sc=6.08062, min distance = 2.82585
ITK_HUMAN Fu	24762195	KAPCA_BOVIN Fu	444345 1c	1STC	1SM2	2VO0		Sc=6.29397, min distance = 2.49072
ITK_HUMAN Fu	24820122	PIM1_HUMAN Fu	444345 1c	1YHS	1SM2	3BWF		Sc=6.25659, min distance = 2.05248
ITK_HUMAN Fu	24901723	CDK2_HUMAN Fu	444345 1c	1AQ1	1SM2	2W06		Sc=6.1891, min distance = 2.421725
ITK_HUMAN Fu	24905143	PK3CG_HUMAN Fu	444345 1c	1E8Z	1SM2	2V4L		Sc=6.12521, min distance = 2.57597
ITK_HUMAN Fu	24916751	CDK2_HUMAN Fu	444345 1c	1AQ1	1SM2	3DOG		Sc=6.3533, min distance = 1.884511
ITK_HUMAN Fu	25021197	CDK2_HUMAN Fu	444345 1c	1AQ1	1SM2	3EOC		Sc=6.23262, min distance = 2.47993
ITK_HUMAN Fu	3543 1g5s	CDK2_HUMAN Fu	444345 1c	1AQ1	1SM2	1G5S		Sc=6.36557, min distance = 2.00840
ITK_HUMAN Fu	3547 Fasu	KAPCA_BOVIN Fu	444345 1c	1STC	1SM2	1Q8W		Sc=5.91095, min distance = 2.62379
ITK_HUMAN Fu	4369136 C	CDK2_HUMAN Fu	444345 1c	1AQ1	1SM2	1DM2		Sc=6.25952, min distance = 2.71977
ITK_HUMAN Fu	447649 1c	CDK2_HUMAN Fu	444345 1c	1AQ1	1SM2	1OI9		Sc=6.34884, min distance = 1.80133
ITK_HUMAN Fu	447652 Im	CDK2_HUMAN Fu	444345 1c	1AQ1	1SM2	1OIQ		Sc=6.13569, min distance = 1.99913
ITK_HUMAN Fu	447654 CI	CDK2_HUMAN Fu	444345 1c	1AQ1	1SM2	1OIT		Sc=6.15275, min distance = 2.40535
ITK_HUMAN Fu	447655 1c	CDK2_HUMAN Fu	444345 1c	1AQ1	1SM2	1OIU		Sc=6.27842, min distance = 1.64699
ITK_HUMAN Fu	447962 1p	CDK2_HUMAN Fu	444345 1c	1AQ1	1SM2	2C5N		Sc=6.13598, min distance = 2.78989
ITK_HUMAN Fu	448043 2g	KAPCA_BOVIN Fu	444345 1c	1STC	1SM2	1Q8U		Sc=5.98089, min distance = 2.62426
ITK_HUMAN Fu	448170 CI	LCK_HUMAN Ful	444345 1c	1QPD	1SM2	1QPC		Sc=6.36251, min distance = 2.12313
ITK_HUMAN Fu	448293 CI	CDK2_HUMAN Fu	444345 1c	1AQ1	1SM2	1R78		Sc=6.44774, min distance = 2.18145
ITK_HUMAN Fu	449087 1v	CDK2_HUMAN Fu	444345 1c	1AQ1	1SM2	1VYW		Sc=6.27029, min distance = 2.43398

ITK_HUMAN Fu	449240 1y	KAPCA_BOVIN F	444345 1c	1STC	1SM2	1YDR		Sc=5.92505, min distance = 2.55280
ITK_HUMAN Fu	4565 1h1r	CDK2_HUMAN Fu	444345 1c	1AQ1	1SM2	1H1R		Sc=6.24794, min distance = 2.78721
ITK_HUMAN Fu	4566 1h1s	CDK2_HUMAN Fu	444345 1c	1AQ1	1SM2	1H1S		Sc=6.29859, min distance = 2.54779
ITK_HUMAN Fu	5281680 Q	PIM1_HUMAN Fu	444345 1c	1YHS	1SM2	2O64		Sc=6.42308, min distance = 2.58771
ITK_HUMAN Fu	5288016 6	CDK2_HUMAN Fu	444345 1c	1AQ1	1SM2	2B53		Sc=6.16143, min distance = 2.38304
ITK_HUMAN Fu	5288708 1	CDK2_HUMAN Fu	444345 1c	1AQ1	1SM2	1KE5		Sc=6.12862, min distance = 2.56889
ITK_HUMAN Fu	5288711 1	CDK2_HUMAN Fu	444345 1c	1AQ1	1SM2	1KE8		Sc=6.27842, min distance = 2.52449
ITK_HUMAN Fu	5288712 1	CDK2_HUMAN Fu	444345 1c	1AQ1	1SM2	1KE9		Sc=6.22185, min distance = 2.48655
ITK_HUMAN Fu	5327109 2	KAPCA_BOVIN F	444345 1c	1STC	1SM2	2C1A		Sc=6.14277, min distance = 2.20320
ITK_HUMAN Fu	5327121 I	PIM1_HUMAN Fu	444345 1c	1YHS	1SM2	2C3I		Sc=6.23523, min distance = 2.48721
ITK_HUMAN Fu	5327122 C	CHK1_HUMAN Fu	444345 1c	1NVR	1SM2	2C3K		Sc=6.47609, min distance = 2.08104
ITK_HUMAN Fu	5327123 I	CHK1_HUMAN Fu	444345 1c	1NVR	1SM2	2C3L		Sc=6.14892, min distance = 2.54311
ITK_HUMAN Fu	5331010 c	CDK2_HUMAN Fu	444345 1c	1AQ1	1SM2	2BKZ		Sc=6.30646, min distance = 2.34918
ITK_HUMAN Fu	5494414 C	CDK2_HUMAN Fu	444345 1c	1AQ1	1SM2	2A0C		Sc=6.47159, min distance = 2.36367
ITK_HUMAN Fu	5687 1di8	CDK2_HUMAN Fu	444345 1c	1AQ1	1SM2	1DI8		Sc=6.24353, min distance = 2.12716
ITK_HUMAN Fu	5957 Aden	PDPK1_HUMAN F	444345 1c	1OKY	1SM2	2BIY		Sc=5.97606, min distance = 2.25013
ITK_HUMAN Fu	6083 aden	PIM1_HUMAN Fu	444345 1c	1YHS	1SM2	1YXU		Sc=6.33538, min distance = 2.32913
ITK_HUMAN Fu	6420138 2	CDK2_HUMAN Fu	444345 1c	1AQ1	1SM2	2UUE		Sc=6.27029, min distance = 2.28116
ITK_HUMAN Fu	6539118 g	CDK2_HUMAN Fu	444345 1c	1AQ1	1SM2	1FVV		Sc=6.36896, min distance = 2.28115
ITK_HUMAN Fu	6540255 C	DAPK1_HUMAN F	444345 1c	1WVY	1SM2	1WVX	0.78	Sc=6.31323, min distance = 1.82201
ITK_HUMAN Fu	656971 CI	CDK2_HUMAN Fu	444345 1c	1AQ1	1SM2	1Y8Y		Sc=6.00741, min distance = 2.28410
ITK_HUMAN Fu	6852207 M	KAPCA_BOVIN F	444345 1c	1STC	1SM2	2GNI		Sc=5.92776, min distance = 2.59321
ITK_HUMAN Fu	6914611 p	KAPCA_BOVIN F	444345 1c	1STC	1SM2	2F7E		Sc=6.42757, min distance = 2.09829
ITK_HUMAN Fu	6914614 p	KAPCA_BOVIN F	444345 1c	1STC	1SM2	2F7Z		Sc=6.48589, min distance = 1.96144
ITK_HUMAN Fu	72271 7-H	CDK2_HUMAN Fu	444345 1c	1AQ1	1SM2	1PKD	0.91	Sc=6.45765, min distance = 2.33119
ITK_HUMAN Fu	72271 7-H	CHK1_HUMAN Fu	444345 1c	1NVR	1SM2	1NVQ	0.91	Sc=6.45363, min distance = 2.46132
ITK_HUMAN Fu	9817550 V	CDK2_HUMAN Fu	444345 1c	1AQ1	1SM2	3BHV		Sc=6.37591, min distance = 2.24064
ITPA_HUMAN F	445708 AM	PURA_ECOLI Fu	8582 Inos	1CIB	2J4E	1SON	0.81	Sc=6.41736, min distance = 2.06713
ITPA_HUMAN F	6083 aden	PURA1_MOUSE F	8582 Inos	1IWE	2J4E	1MF0	0.79	Sc=5.94271, min distance = 1.04309
ITPA_HUMAN F	65110 AIC	PURO_METTH Fu	8582 Inos	2NTK	2J4E	2NTL	0.97	Sc=5.88808, min distance = 2.07331
ITPA_HUMAN F	73323 Xan	HGXR_TOXGO Fu	8582 Inos	1QK4	2J4E	1QK5	0.94	Sc=6.46063, min distance = 2.11250
ITPK1_ENTHI	33113 gam	PURT_ECOLI Fu	91532 AMP	1KJI	1Z2P	1EYZ	0.99	Sc=6.47984, min distance = 1.95179
ITPK1_ENTHI	445940 O6	CDK2_HUMAN Fu	6022 Aden	1GY3	1Z2N	1GZ8		Sc=6.19765, min distance = 2.06555
ITPK1_ENTHI	447916 ac	RIO1_ARCFU Fu	6022 Aden	1ZTH	1Z2N	1ZTF	0.95	Sc=6.23707, min distance = 2.63476
ITPK1_ENTHI	447955 1p	CDK2_HUMAN Fu	6022 Aden	1GY3	1Z2N	1PXI		Sc=5.75124, min distance = 2.13914
ITPK1_ENTHI	5957 Aden	PURK_ECOLI Fu	6022 Aden	3ETJ	1Z2N	3ETH	0.99	Sc=6.48498, min distance = 2.15724
ITPK1_ENTHI	5957 Aden	RK_BOVIN Full	6022 Aden	3C4Z	1Z2N	3C4W	0.99	Sc=6.02028, min distance = 2.01519

ITPK1_ENTHI I	6083 ader	HSP71_HUMAN Fu	6022 Aden	1S3X	1Z2N	1XQS		0.99	Sc=5.91291, min distance = 1.83749
ITPK1_ENTHI I	6083 ader	PURP_METJA Ful	6022 Aden	2R7N	1Z2N	2R7M		0.99	Sc=6.36157, min distance = 2.02331
ITPK1_ENTHI I	6083 ader	PURP_METJA Ful	91532 AMP	2R7K	1Z2P	2R7M		0.97	Sc=6.36603, min distance = 2.15231
ITPK1_ENTHI I	6852187 2	O57883_PYRHO I	6022 Aden	1WNL	1Z2N	2DTH		0.89	Sc=6.14715, min distance = 2.03296
ITPK1_HUMAN I	11987876	GSP_ECOLI Ful	6022 Aden	2IO8	2QB5	2IOA			Sc=6.07033, min distance = 1.08491
ITPK1_HUMAN I	16750063	CSK2A_MAIZE Fu	33113 gam	1LP4	2Q7D	2OXX			Sc=5.65713, min distance = 2.12914
ITPK1_HUMAN I	3454 ganc	KITH_HHV11 Ful	6022 Aden	2VTK	2QB5	1KI2			Sc=6.16831, min distance = 0.59735
ITPK1_HUMAN I	444842 CI	CDK2_HUMAN Ful	6022 Aden	1GY3	2QB5	1CKP			Sc=5.64083, min distance = 2.03717
ITPK1_HUMAN I	445940 O6	CDK2_HUMAN Ful	6022 Aden	1GY3	2QB5	1GZ8			Sc=6.25289, min distance = 1.97154
ITPK1_HUMAN I	447916 ac	RIO1_ARCFU Ful	6022 Aden	1ZTH	2QB5	1ZTF		0.95	Sc=6.28168, min distance = 2.76378
ITPK1_HUMAN I	448043 2c	ROCK1_HUMAN Fu	33113 gam	2V55	2Q7D	3D9V			Sc=6.08362, min distance = 1.41170
ITPK1_HUMAN I	5327148 C	IPKA_RABIT Ful	33113 gam	1CDK	2Q7D	2ERZ			Sc=6.23397, min distance = 2.12249
ITPK1_HUMAN I	5957 Ader	RK_BOVIN Full	6022 Aden	3C4Z	2QB5	3C4W		0.99	Sc=6.06004, min distance = 2.01989
ITPK1_HUMAN I	6083 ader	PSPF_ECOLI Ful	6022 Aden	2C98	2QB5	2VII		0.99	Sc=5.92973, min distance = 2.05918
ITPK1_HUMAN I	91532 AMP	IGF1R_HUMAN Fu	33113 gam	1JQH	2Q7D	1K3A		0.99	Sc=5.77394, min distance = 2.03768
ITPK1_HUMAN I	91532 AMP	MTNK_BACSU Ful	6022 Aden	2OLC	2QB5	2PUL		0.99	Sc=6.09926, min distance = 2.02095
ITPK1_HUMAN I	9543438 C	DDLB_ECOLI Ful	6022 Aden	1IOW	2QB5	1IOV			Sc=5.92872, min distance = 2.35528
JHD3A_HUMAN I	444591 CI	IDH_ECOLI Ful	51 2-Oxop	1CW4	2Q8C	1BL5			Sc=5.70422, min distance = 2.20878
JHD3A_HUMAN I	5288998 1	HIF1N_HUMAN Fu	3080614 C	1H2K	2OX0	1YCI			Sc=6.21246, min distance = 2.29367
JHD3A_HUMAN I	5496500 I	IDH_ECOLI Ful	51 2-Oxop	1CW4	2Q8C	1AI2			Sc=6.00403, min distance = 1.45254
JIP1_RAT Ful	2386 CID2	TRY1_BOVIN Fu	17472 Hex	2G81	2FPE	1C1T			Sc=5.61096, min distance = 2.51362
K6PF1_ECOLI I	23727982	CDK2_HUMAN Ful	6022 Aden	1GY3	1PFK	3BHU			Sc=6.23595, min distance = 1.33307
K6PF1_ECOLI I	2608 1jsv	CDK2_HUMAN Ful	6022 Aden	1GY3	1PFK	1JSV			Sc=6.19467, min distance = 1.04544
K6PF1_ECOLI I	398148 1e	CDK2_HUMAN Ful	6022 Aden	1GY3	1PFK	1E1X			Sc=6.31008, min distance = 1.70455
K6PF1_ECOLI I	444564 AD	BIOD_ECOLI Ful	6022 Aden	1DAD	1PFK	1BS1		0.91	Sc=6.43746, min distance = 2.24654
K6PF1_ECOLI I	445966 O6	CDK2_HUMAN Ful	6022 Aden	1GY3	1PFK	1H0V			Sc=6.30319, min distance = 1.58736
K6PF1_ECOLI I	447048 2k	Y1521_ARCFU Fu	6022 Aden	2BFR	1PFK	2BFQ		0.95	Sc=6.63477, min distance = 1.69265
K6PF1_ECOLI I	447654 CI	CDK2_HUMAN Ful	6022 Aden	1GY3	1PFK	1OIT			Sc=6.3773, min distance = 0.653894
K6PF1_ECOLI I	447955 1p	CDK2_HUMAN Ful	6022 Aden	1GY3	1PFK	1PXI			Sc=5.77922, min distance = 1.84419
K6PF1_ECOLI I	447962 1p	CDK2_HUMAN Ful	6022 Aden	1GY3	1PFK	2C5N			Sc=5.9116, min distance = 2.355135
K6PF1_ECOLI I	4565 1h1r	CDK2_HUMAN Ful	6022 Aden	1GY3	1PFK	1H1R			Sc=6.08723, min distance = 2.07364
K6PF1_ECOLI I	5327133 C	CDK2_HUMAN Ful	6022 Aden	1GY3	1PFK	2C6K			Sc=5.94925, min distance = 2.05166
K6PF1_ECOLI I	5327135 D	CDK2_HUMAN Ful	6022 Aden	1GY3	1PFK	2C6M			Sc=6.40087, min distance = 0.97105
K6PF1_ECOLI I	5687 1di8	CDK2_HUMAN Ful	6022 Aden	1GY3	1PFK	1DI8			Sc=5.76117, min distance = 1.40309
K6PF1_ECOLI I	6031 Uric	DCK_HUMAN Ful	6022 Aden	1P5Z	1PFK	2ZIA			Sc=6.34222, min distance = 2.36608
K6PF1_ECOLI I	60961 ade	IPKA_RABIT Ful	6022 Aden	1JBP	1PFK	1FMO		0.95	Sc=5.87388, min distance = 2.00886
K6PF1_ECOLI I	6132 Cyti	ECX1_PYRAB Ful	6022 Aden	2PO0	1PFK	2PO2			Sc=6.32, min distance = 2.02771349

K6PF1_ECOLI I	6132 Cyti	ECX2_PYRAB Fu	6022 Aden	2PO0	1PFK	2PO2		Sc=6.32807, min distance = 2.11168
K6PF1_ECOLI I	6420138 2	CDK2_HUMAN Fu	6022 Aden	1GY3	1PFK	2UUE		Sc=6.4863, min distance = 0.752011
K6PF1_ECOLI I	656971 CI	CDK2_HUMAN Fu	6022 Aden	1GY3	1PFK	1Y8Y		Sc=6.18506, min distance = 1.16307
K6PF1_ECOLI I	72194 2-C	ENPL_CANFA Fu	6022 Aden	1TC6	1PFK	1QYE	0.89	Sc=6.21486, min distance = 1.16079
K6PF1_ECOLI I	8977 1dar	NDK_PYRHO Full	6022 Aden	2DYA	1PFK	2DXE	0.8	Sc=6.46298, min distance = 1.23870
K6PF1_ECOLI I	9817550 V	CDK2_HUMAN Fu	6022 Aden	1GY3	1PFK	3BHV		Sc=6.41259, min distance = 1.10548
K6PF1_ECOLI I	9991833 S	CDK2_HUMAN Fu	6022 Aden	1GY3	1PFK	2R3H		Sc=5.75819, min distance = 2.54008
K6PF1_ECOLI I	9994066 4	CDK2_HUMAN Fu	6022 Aden	1GY3	1PFK	2VTJ		Sc=5.71327, min distance = 2.08278
K6PF2_ECOLI I	11314340	KAPCA_BOVIN Fu	5957 Aden	1Q24	3CQD	2UZT		Sc=6.4215, min distance = 2.129312
K6PF2_ECOLI I	11708454	KAPCA_BOVIN Fu	5957 Aden	1Q24	3CQD	2VNW		Sc=6.19465, min distance = 2.35015
K6PF2_ECOLI I	3540 1yds	KAPCA_BOVIN Fu	5957 Aden	1Q24	3CQD	1YDS		Sc=5.95413, min distance = 2.28119
K6PF2_ECOLI I	3547 Fasu	KAPCA_BOVIN Fu	5957 Aden	1Q24	3CQD	1Q8W		Sc=6.0197, min distance = 2.294479
K6PF2_ECOLI I	440317 AT	PURT_ECOLI Fu	5957 Aden	1KJ8	3CQD	1KJJ	0.99	Sc=6.5042, min distance = 1.350138
K6PF2_ECOLI I	444564 AD	BIOD_ECOLI Fu	5957 Aden	1A82	3CQD	1BS1	0.91	Sc=6.44573, min distance = 2.34350
K6PF2_ECOLI I	449240 1y	KAPCA_BOVIN Fu	5957 Aden	1Q24	3CQD	1YDR		Sc=6.03239, min distance = 2.13139
K6PF2_ECOLI I	6022 Aden	ARSA1_ECOLX Fu	5957 Aden	1II0	3CQD	1IHU	0.99	Sc=6.44121, min distance = 2.01504
K6PF2_ECOLI I	6022 Aden	BIOD_ECOLI Fu	5957 Aden	1A82	3CQD	1DAD	0.99	Sc=6.45175, min distance = 2.25962
K6PF2_ECOLI I	6022 Aden	CLCN5_HUMAN Fu	5957 Aden	2J9L	3CQD	2JA3	0.99	Sc=6.48088, min distance = 1.58298
K6PF2_ECOLI I	6022 Aden	DDL_THET8 Full	5957 Aden	2ZDQ	3CQD	2ZDH	0.99	Sc=6.46586, min distance = 1.69251
K6PF2_ECOLI I	6022 Aden	KTHY_HUMAN Fu	5957 Aden	1E2Q	3CQD	1NN3	0.99	Sc=6.44446, min distance = 2.35007
K6PF2_ECOLI I	6022 Aden	MUTS_ECOLI Fu	5957 Aden	1W7A	3CQD	1OH7	0.99	Sc=6.4349, min distance = 2.123091
K6PF2_ECOLI I	6022 Aden	Q72H90_THET2 I	5957 Aden	2BEK	3CQD	2BEJ	0.99	Sc=6.45761, min distance = 2.19682
K6PF2_ECOLI I	6022 Aden	RK_BOVIN Full=	5957 Aden	3C4W	3CQD	3C4Z	0.99	Sc=6.44521, min distance = 2.36812
K6PF2_ECOLI I	6022 Aden	Y059_METJA Fu	5957 Aden	2J9C	3CQD	2J9D	0.99	Sc=6.46635, min distance = 1.73761
K6PF2_ECOLI I	6083 aden	PURP_PYRFU Fu	5957 Aden	2R86	3CQD	2R85	0.98	Sc=6.40339, min distance = 2.32731
K6PF2_ECOLI I	91532 AME	AROK_MYCTU Fu	5957 Aden	2IYW	3CQD	1ZYU	0.99	Sc=5.76083, min distance = 2.23052
K6PF2_ECOLI I	91532 AME	NIFH1_AZOVI Fu	5957 Aden	2C8V	3CQD	2AFK	0.99	Sc=6.08494, min distance = 1.91455
K6PF2_ECOLI I	91532 AME	PDXK_SHEEP Fu	5957 Aden	1LHR	3CQD	1RFT	0.99	Sc=6.52283, min distance = 2.22984
K6PF_BACST Fu	11957393	FAK1_HUMAN Fu	6022 Aden	1MP8	4PFK	2ETM		Sc=6.26773, min distance = 2.15109
K6PF_BACST Fu	1540 1h1q	CDK2_HUMAN Fu	6022 Aden	1GY3	4PFK	1H1Q		Sc=6.17431, min distance = 1.90545
K6PF_BACST Fu	1694 TBBt	CDK2_HUMAN Fu	6022 Aden	1GY3	4PFK	1P5E		Sc=5.68067, min distance = 1.78883
K6PF_BACST Fu	23653515	CDK2_HUMAN Fu	6022 Aden	1GY3	4PFK	2R3F		Sc=5.72005, min distance = 2.29375
K6PF_BACST Fu	23653516	CDK2_HUMAN Fu	6022 Aden	1GY3	4PFK	2R3G		Sc=5.88185, min distance = 1.99656
K6PF_BACST Fu	23653522	CDK2_HUMAN Fu	6022 Aden	1GY3	4PFK	2R3N		Sc=6.11377, min distance = 2.40393
K6PF_BACST Fu	23653526	CDK2_HUMAN Fu	6022 Aden	1GY3	4PFK	2R3R		Sc=6.06967, min distance = 2.18374
K6PF_BACST Fu	23727981	CDK2_HUMAN Fu	6022 Aden	1GY3	4PFK	3BHT		Sc=6.19365, min distance = 1.83261
K6PF_BACST Fu	24864077	CDK2_HUMAN Fu	6022 Aden	1GY3	4PFK	2VTN		Sc=5.96753, min distance = 2.35287

K6PF_BACST F	2608 1jsv	CDK2_HUMAN Fu	6022 Aden	1GY3	4PFK	1JSV		Sc=5.75136, min distance = 2.509128
K6PF_BACST F	444503 FU	NDKC_DICDI Fu	6022 Aden	1KDN	4PFK	1B99		Sc=6.30334, min distance = 2.000140
K6PF_BACST F	444564 AD	BIOD_ECOLI Fu	6022 Aden	1DAD	4PFK	1BS1	0.91	Sc=6.42696, min distance = 2.052545
K6PF_BACST F	445966 O6	CDK2_HUMAN Fu	6022 Aden	1GY3	4PFK	1H0V		Sc=6.31367, min distance = 0.951490
K6PF_BACST F	446090 1h	NDKC_DICDI Fu	6022 Aden	1KDN	4PFK	1HIY	0.97	Sc=6.4311, min distance = 1.3859718
K6PF_BACST F	447017 CI	NDKC_DICDI Fu	6022 Aden	1KDN	4PFK	1LWX		Sc=6.42384, min distance = 1.655492
K6PF_BACST F	447654 CI	CDK2_HUMAN Fu	6022 Aden	1GY3	4PFK	1OIT		Sc=6.04123, min distance = 2.805055
K6PF_BACST F	447655 1c	CDK2_HUMAN Fu	6022 Aden	1GY3	4PFK	1OIU		Sc=6.19635, min distance = 1.838208
K6PF_BACST F	4564 1e1v	CDK2_HUMAN Fu	6022 Aden	1GY3	4PFK	1E1V		Sc=6.26708, min distance = 0.586876
K6PF_BACST F	4565 1h1r	CDK2_HUMAN Fu	6022 Aden	1GY3	4PFK	1H1R		Sc=6.14778, min distance = 2.054368
K6PF_BACST F	5287844 i	GSK3B_HUMAN F	6022 Aden	1J1C	4PFK	1UV5		Sc=6.11078, min distance = 2.121764
K6PF_BACST F	5327097 2	CDK2_HUMAN Fu	6022 Aden	1GY3	4PFK	2BTS		Sc=5.68835, min distance = 2.140137
K6PF_BACST F	5957 Aden	BIOD_ECOLI Fu	6022 Aden	1DAD	4PFK	1A82	0.99	Sc=6.47426, min distance = 2.129771
K6PF_BACST F	5957 Aden	PDXK_SHEEP Fu	6022 Aden	1RFU	4PFK	1LHR	0.99	Sc=6.01311, min distance = 1.953331
K6PF_BACST F	6083 aden	PSPF_ECOLI Fu	6022 Aden	2C98	4PFK	2VII	0.99	Sc=6.38049, min distance = 2.105648
K6PF_BACST F	6420139 C	CDK2_HUMAN Fu	6022 Aden	1GY3	4PFK	2C5V		Sc=6.11778, min distance = 1.852411
K6PF_BACST F	72194 2-C	ENPL_CANFA Fu	6022 Aden	1TC6	4PFK	1QYE	0.89	Sc=6.21183, min distance = 0.959165
K6PF_BACST F	8977 1dar	NDK_PYRHO Ful	6022 Aden	2DYA	4PFK	2DXE	0.8	Sc=6.00149, min distance = 1.357478
K6PF_BACST F	8977 1dar	O33839_THEMA	6022 Aden	1XJK	4PFK	1XJE	0.8	Sc=5.70617, min distance = 1.180265
K6PF_BACST F	9926933 i	CDK2_HUMAN Fu	6022 Aden	1GY3	4PFK	2VTI		Sc=5.80064, min distance = 2.438890
K6PF_BACST F	9991833 S	CDK2_HUMAN Fu	6022 Aden	1GY3	4PFK	2R3H		Sc=5.91201, min distance = 2.345648
KAD3_BOVIN F	166760 Fa	PURP_METJA Fu	6083 aden	2R7M	2AK3	2R7N	0.76	Sc=5.88563, min distance = 1.608148
KAD3_BOVIN F	33113 gam	ASSY_THET8 Fu	6083 aden	1J20	2AK3	1KOR	0.97	Sc=5.99705, min distance = 1.931938
KAD3_BOVIN F	440317 AT	SRC_CHICK Ful	6083 aden	3DQX	2AK3	3DQW	0.97	Sc=5.90149, min distance = 2.825870
KAD3_BOVIN F	445708 AM	NADE_ECOLI Fu	6083 aden	1WXI	2AK3	1WXE	0.91	Sc=5.88243, min distance = 2.489085
KAD3_BOVIN F	447832 AD	ACSA_SALTY Fu	6083 aden	2P2F	2AK3	1PG4	0.99	Sc=6.41655, min distance = 2.006901
KAD3_BOVIN F	448895 AD	UMPK_YEAST Fu	6083 aden	1UKZ	2AK3	1UKY	0.99	Sc=5.60274, min distance = 2.572539
KAD3_BOVIN F	5957 Aden	AAKG1_RAT Ful	6083 aden	2V8Q	2AK3	2V92	0.98	Sc=5.93252, min distance = 1.989467
KAD3_BOVIN F	5957 Aden	PURP_PYRFU Fu	6083 aden	2R85	2AK3	2R86	0.98	Sc=5.76043, min distance = 2.090618
KAD3_BOVIN F	6022 Aden	HSP71_HUMAN F	6083 aden	1XQS	2AK3	1S3X	0.99	Sc=5.98415, min distance = 2.114141
KAD3_BOVIN F	6076 Cycl	PDE10_HUMAN F	6083 aden	2OUN	2AK3	2OUR	0.99	Sc=6.30607, min distance = 2.125357
KAD3_BOVIN F	65110 AIC	YL28_SCHPO Fu	6083 aden	2OOX	2AK3	2QRE	0.79	Sc=6.27987, min distance = 2.033391
KAD3_BOVIN F	657135 Op	PIM1_HUMAN Fu	6083 aden	1YXU	2AK3	1YXV		Sc=5.77725, min distance = 2.657571
KAD3_BOVIN F	8582 Inos	PURA1_MOUSE F	6083 aden	1MF0	2AK3	1IWE	0.79	Sc=5.93126, min distance = 2.022937
KAD3_BOVIN F	8582 Inos	PYGM_RABIT Fu	6083 aden	8GPB	2AK3	2QN7	0.79	Sc=6.29641, min distance = 2.406890
KADA_METVO F	445708 AM	NADE_ECOLI Fu	6083 aden	1WXI	1KHT	1WXE	0.91	Sc=6.35674, min distance = 1.997587
KADA_METVO F	448895 AD	UMPK_YEAST Fu	6083 aden	1UKZ	1KHT	1UKY	0.99	Sc=6.4092, min distance = 2.3502044

KADA_METVO F	5281680 Q	PIM1_HUMAN Fu	6083 aden	1YXU	1KHT	2O64		Sc=6.40487, min distance = 2.05127
KADA_METVO F	5957 Aden	AAKG1_RAT Ful	6083 aden	2V8Q	1KHT	2V92	0.98	Sc=6.00512, min distance = 2.15395
KADA_METVO F	5957 Aden	PURP_PYRFU Fu	6083 aden	2R85	1KHT	2R86	0.98	Sc=6.45568, min distance = 1.06464
KADA_METVO F	6022 Aden	PURP_METJA Fu	6083 aden	2R7M	1KHT	2R7N	0.99	Sc=6.49545, min distance = 0.74574
KADA_METVO F	6076 Cycl	PDE10_HUMAN F	6083 aden	2OUN	1KHT	2OUR	0.99	Sc=6.31053, min distance = 2.07236
KADA_METVO F	65110 AIC	AAKG1_HUMAN F	6083 aden	2UV4	1KHT	2UV5	0.79	Sc=5.78605, min distance = 2.17691
KADA_METVO F	65110 AIC	YL28_SCHPO Fu	6083 aden	2OOX	1KHT	2QRE	0.79	Sc=6.28713, min distance = 2.06530
KADA_METVO F	656964 1y	PDE4D_HUMAN F	6083 aden	1TB7	1KHT	1Y2C		Sc=5.93976, min distance = 2.13613
KADA_METVO F	656966 1y	PDE4D_HUMAN F	6083 aden	1TB7	1KHT	1Y2E		Sc=6.07837, min distance = 2.02300
KADA_METVO F	656968 1y	PDE4B_HUMAN F	6083 aden	1ROR	1KHT	1Y2H		Sc=5.95589, min distance = 2.03726
KADA_METVO F	657135 Op	PIM1_HUMAN Fu	6083 aden	1YXU	1KHT	1YXV		Sc=5.83858, min distance = 2.85453
KADA_SULAC F	1540 1h1q	CDK2_HUMAN Fu	6022 Aden	1GY3	1NKS	1H1Q		Sc=6.35409, min distance = 2.03263
KADA_SULAC F	16214827	CDK2_HUMAN Fu	6022 Aden	1GY3	1NKS	2UZN		Sc=6.08768, min distance = 1.68724
KADA_SULAC F	1707 1fvt	CDK2_HUMAN Fu	6022 Aden	1GY3	1NKS	1FVT		Sc=6.18225, min distance = 1.88159
KADA_SULAC F	23653516	CDK2_HUMAN Fu	6022 Aden	1GY3	1NKS	2R3G		Sc=6.22641, min distance = 2.37828
KADA_SULAC F	23653518	CDK2_HUMAN Fu	6022 Aden	1GY3	1NKS	2R3J		Sc=6.11186, min distance = 2.51360
KADA_SULAC F	23653526	CDK2_HUMAN Fu	6022 Aden	1GY3	1NKS	2R3R		Sc=6.18248, min distance = 2.34877
KADA_SULAC F	24864077	CDK2_HUMAN Fu	6022 Aden	1GY3	1NKS	2VTN		Sc=6.23783, min distance = 2.09923
KADA_SULAC F	24901723	CDK2_HUMAN Fu	6022 Aden	1GY3	1NKS	2W06		Sc=6.22516, min distance = 1.70000
KADA_SULAC F	2608 1jsv	CDK2_HUMAN Fu	6022 Aden	1GY3	1NKS	1JSV		Sc=5.90109, min distance = 3.15828
KADA_SULAC F	398148 1e	CDK2_HUMAN Fu	6022 Aden	1GY3	1NKS	1E1X		Sc=6.16111, min distance = 2.29440
KADA_SULAC F	445708 AM	NADE_ECOLI Fu	6083 aden	1WXI	1NKS	1WXE	0.91	Sc=6.35106, min distance = 2.34817
KADA_SULAC F	445966 O6	CDK2_HUMAN Fu	6022 Aden	1GY3	1NKS	1H0V		Sc=6.19342, min distance = 2.43317
KADA_SULAC F	446704 LS	CDK2_HUMAN Fu	6022 Aden	1GY3	1NKS	1KE6		Sc=6.30849, min distance = 2.09098
KADA_SULAC F	447004 CI	MYS2_DICDI Fu	6022 Aden	1VOM	1NKS	1LVK		Sc=5.72823, min distance = 2.03656
KADA_SULAC F	447649 1o	CDK2_HUMAN Fu	6022 Aden	1GY3	1NKS	1OI9		Sc=6.41423, min distance = 2.06172
KADA_SULAC F	447654 CI	CDK2_HUMAN Fu	6022 Aden	1GY3	1NKS	1OIT		Sc=6.20874, min distance = 2.51746
KADA_SULAC F	447655 1o	CDK2_HUMAN Fu	6022 Aden	1GY3	1NKS	1OIU		Sc=6.36505, min distance = 2.02053
KADA_SULAC F	447656 1o	CDK2_HUMAN Fu	6022 Aden	1GY3	1NKS	1OIY		Sc=6.47845, min distance = 2.03090
KADA_SULAC F	447766 1p	CDK2_HUMAN Fu	6022 Aden	1GY3	1NKS	1P2A		Sc=6.26283, min distance = 2.11391
KADA_SULAC F	448895 AD	UMPK_YEAST Fu	6083 aden	1UKZ	1NKS	1UKY	0.99	Sc=6.41155, min distance = 2.26845
KADA_SULAC F	449088 1v	CDK2_HUMAN Fu	6022 Aden	1GY3	1NKS	1VYZ		Sc=6.04854, min distance = 2.30762
KADA_SULAC F	4564 1e1v	CDK2_HUMAN Fu	6022 Aden	1GY3	1NKS	1E1V		Sc=6.13393, min distance = 2.79178
KADA_SULAC F	4565 1h1r	CDK2_HUMAN Fu	6022 Aden	1GY3	1NKS	1H1R		Sc=6.33938, min distance = 2.10884
KADA_SULAC F	5288016 6	CDK2_HUMAN Fu	6022 Aden	1GY3	1NKS	2B53		Sc=6.25298, min distance = 1.99173
KADA_SULAC F	5288018 C	CDK2_HUMAN Fu	6022 Aden	1GY3	1NKS	2B52		Sc=6.5214, min distance = 1.985304
KADA_SULAC F	5288708 1	CDK2_HUMAN Fu	6022 Aden	1GY3	1NKS	1KE5		Sc=6.10026, min distance = 1.77337

KADA_SULAC Fu	5288710 C	CDK2_HUMAN Fu	6022 Aden	1GY3	1NKS	1KE7		Sc=6.30925, min distance = 1.983758
KADA_SULAC Fu	5288712 1	CDK2_HUMAN Fu	6022 Aden	1GY3	1NKS	1KE9		Sc=6.27969, min distance = 1.967459
KADA_SULAC Fu	5327096 2	CDK2_HUMAN Fu	6022 Aden	1GY3	1NKS	2BTR		Sc=6.06861, min distance = 2.493275
KADA_SULAC Fu	5327097 2	CDK2_HUMAN Fu	6022 Aden	1GY3	1NKS	2BTS		Sc=5.85603, min distance = 2.291035
KADA_SULAC Fu	5327133 C	CDK2_HUMAN Fu	6022 Aden	1GY3	1NKS	2C6K		Sc=6.20438, min distance = 2.777619
KADA_SULAC Fu	5327135 D	CDK2_HUMAN Fu	6022 Aden	1GY3	1NKS	2C6M		Sc=6.15501, min distance = 2.345205
KADA_SULAC Fu	5957 Aden	ARP3_BOVIN Fu	6022 Aden	2P9I	1NKS	1TYQ	0.99	Sc=5.70929, min distance = 2.067393
KADA_SULAC Fu	5957 Aden	BIOD_ECOLI Fu	6022 Aden	1DAD	1NKS	1A82	0.99	Sc=6.02751, min distance = 2.730552
KADA_SULAC Fu	5957 Aden	MALK_ECOLI Fu	6022 Aden	2AWN	1NKS	1Q12	0.99	Sc=5.97214, min distance = 2.236485
KADA_SULAC Fu	5957 Aden	MUTS_ECOLI Fu	6022 Aden	1OH7	1NKS	1W7A	0.99	Sc=5.99438, min distance = 2.156822
KADA_SULAC Fu	6076 Cycl	PDE10_HUMAN Fu	6083 aden	2OUN	1NKS	2OUR	0.99	Sc=6.31779, min distance = 2.134005
KADA_SULAC Fu	611002 Op	PIM1_HUMAN Fu	6083 aden	1YXU	1NKS	1YXX		Sc=6.21251, min distance = 0.818039
KADA_SULAC Fu	65110 AIC	AAKG1_HUMAN Fu	6083 aden	2UV4	1NKS	2UV5	0.79	Sc=5.82655, min distance = 2.062825
KADA_SULAC Fu	65110 AIC	YL28_SCHPO Fu	6083 aden	2OOX	1NKS	2QRE	0.79	Sc=6.28598, min distance = 2.296605
KADA_SULAC Fu	656966 1y	PDE4D_HUMAN Fu	6083 aden	1TB7	1NKS	1Y2E		Sc=6.07944, min distance = 2.271606
KADA_SULAC Fu	656968 1y	PDE4B_HUMAN Fu	6083 aden	1ROR	1NKS	1Y2H		Sc=5.96376, min distance = 2.213979
KADA_SULAC Fu	656971 CI	CDK2_HUMAN Fu	6022 Aden	1GY3	1NKS	1Y8Y		Sc=5.88986, min distance = 2.811022
KADA_SULAC Fu	6804 guar	PDE10_HUMAN Fu	6083 aden	2OUN	1NKS	2OUQ	0.8	Sc=5.6518, min distance = 0
KADA_SULAC Fu	91532 AMP	BIOD_ECOLI Fu	6022 Aden	1DAD	1NKS	1DAG	0.99	Sc=6.05178, min distance = 2.554138
KADA_SULAC Fu	91532 AMP	KIF1A_MOUSE Fu	6022 Aden	1I5S	1NKS	1I6I	0.99	Sc=6.05039, min distance = 2.411211
KADA_SULAC Fu	91532 AMP	PURP_METJA Fu	6083 aden	2R7M	1NKS	2R7K	0.97	Sc=6.47158, min distance = 2.045759
KADA_SULAC Fu	9547890 I	CDK2_HUMAN Fu	6022 Aden	1GY3	1NKS	1W8C		Sc=6.17582, min distance = 2.944853
KADA_SULAC Fu	9926933 i	CDK2_HUMAN Fu	6022 Aden	1GY3	1NKS	2VTI		Sc=6.13107, min distance = 2.544743
KADA_SULAC Fu	9991833 S	CDK2_HUMAN Fu	6022 Aden	1GY3	1NKS	2R3H		Sc=6.01996, min distance = 2.577198
KAD_MYCTU Fu	440317 AT	BCKD_RAT Full=	6022 Aden	1GKZ	2CDN	1GJV	0.99	Sc=6.4895, min distance = 2.0207496
KAD_MYCTU Fu	444503 FU	NDKC_DICDI Fu	6022 Aden	1KDN	2CDN	1B99		Sc=6.30334, min distance = 2.254096
KAD_MYCTU Fu	444842 CI	CDK2_HUMAN Fu	6022 Aden	1GY3	2CDN	1CKP		Sc=5.65414, min distance = 1.589180
KAD_MYCTU Fu	447955 1p	CDK2_HUMAN Fu	6022 Aden	1GY3	2CDN	1PXI		Sc=5.7882, min distance = 2.3867976
KAD_MYCTU Fu	4565 1h1r	CDK2_HUMAN Fu	6022 Aden	1GY3	2CDN	1H1R		Sc=6.16526, min distance = 0.990246
KAD_MYCTU Fu	5957 Aden	MALK_ECOLI Fu	6022 Aden	2AWN	2CDN	1Q12	0.99	Sc=6.52159, min distance = 1.140400
KAD_MYCTU Fu	5957 Aden	PDK2_HUMAN Fu	6022 Aden	2BU8	2CDN	2BU2	0.99	Sc=6.49477, min distance = 2.151181
KAD_MYCTU Fu	5957 Aden	Y059_METJA Fu	6022 Aden	2J9D	2CDN	2J9C	0.99	Sc=6.50237, min distance = 1.819827
KAD_MYCTU Fu	6031 Uric	DCK_HUMAN Full	6022 Aden	1P5Z	2CDN	2ZIA		Sc=6.33113, min distance = 2.014031
KAD_MYCTU Fu	6083 aden	GLNA1_MYCTU Fu	6022 Aden	2BVC	2CDN	1HTO	0.99	Sc=6.41554, min distance = 0.907507
KAD_MYCTU Fu	6083 aden	PURP_METJA Fu	6022 Aden	2R7N	2CDN	2R7M	0.99	Sc=6.3898, min distance = 2.4703864
KAD_MYCTU Fu	60961 ade	IPKA_RABIT Fu	6022 Aden	1JBP	2CDN	1FMO	0.95	Sc=6.2694, min distance = 2.2320730
KAD_MYCTU Fu	6804 guar	NDK_PYRHO Full	6022 Aden	2DYA	2CDN	2DXF	0.8	Sc=6.42574, min distance = 2.346505

KAD_MYCTU Fu	6852187 2	O57883_PYRHO	6022 Aden	1WNL	2CDN	2DTH		0.89	Sc=6.18296, min distance = 1.61218
KAD_MYCTU Fu	72194 2-C	ENPL_CANFA Fu	6022 Aden	1TC6	2CDN	1QYE		0.89	Sc=6.19362, min distance = 1.64383
KAD_MYCTU Fu	8977 1dar	NDK_PYRHO Ful	6022 Aden	2DYA	2CDN	2DXE		0.8	Sc=6.47747, min distance = 2.17462
KAD_MYCTU Fu	91532 AMF	MTNK_BACSU Fu	6022 Aden	2OLC	2CDN	2PUL		0.99	Sc=6.52885, min distance = 1.05068
KAD_MYCTU Fu	91532 AMF	NIFH1_AZOVI Fu	6022 Aden	1FP6	2CDN	2AFK		0.99	Sc=6.52232, min distance = 0.79955
KAMA_CLOSU Fu	6852187 2	MOAA_STAA8 Fu	34756 Acy	2FB2	2A5H	2FB3		0.84	Sc=6.21357, min distance = 2.41031
KAP3_RAT Ful	24316 cyc	CNGK1_RHILO Fu	6076 Cycl	1VP6	1CX4	3CL1		0.81	Sc=6.34436, min distance = 2.28095
KAP3_RAT Ful	24316 cyc	KAP0_BOVIN Fu	6076 Cycl	1RGS	1CX4	1RL3		0.81	Sc=6.35166, min distance = 2.35648
KAP3_RAT Ful	656964 1y	PDE4D_HUMAN Fu	6076 Cycl	2PW3	1CX4	1Y2C			Sc=5.973, min distance = 1.2130403
KAP3_RAT Ful	656966 1y	PDE4D_HUMAN Fu	6076 Cycl	2PW3	1CX4	1Y2E			Sc=6.10189, min distance = 1.20191
KAP3_RAT Ful	656969 1y	PDE4D_HUMAN Fu	6076 Cycl	2PW3	1CX4	1Y2K			Sc=6.24576, min distance = 1.33978
KAP3_RAT Ful	6858240 C	KAP0_BOVIN Fu	6076 Cycl	1RGS	1CX4	1NE6		0.98	Sc=6.30779, min distance = 2.41611
KAPCA_BOVIN	11270500	CDK2_HUMAN Fu	444345 1c	1AQ1	1STC	2VV9	34.78		Sc=6.52211, min distance = 2.08793
KAPCA_BOVIN	11986115	PIM1_HUMAN Fu	444345 1c	1YHS	1STC	2BZJ	30.06		Sc=6.31688, min distance = 2.16175
KAPCA_BOVIN	15991572	LCK_HUMAN Ful	444345 1c	1QPD	1STC	2OF4	24.59		Sc=6.38424, min distance = 2.19250
KAPCA_BOVIN	16122608	PDPK1_HUMAN Fu	444345 1c	1OKY	1STC	2PE1	39.18		Sc=6.36747, min distance = 2.57895
KAPCA_BOVIN	16725726	AKT2_HUMAN Fu	11857236	3E88	3E8C	3D0E	44.86	0.85	Sc=6.51759, min distance = 2.61004
KAPCA_BOVIN	1907917 2	CHK1_HUMAN Fu	444345 1c	1NVR	1STC	2CGW	33.01		Sc=6.10978, min distance = 2.58224
KAPCA_BOVIN	23653515	CDK2_HUMAN Fu	444345 1c	1AQ1	1STC	2R3F	34.78		Sc=6.05097, min distance = 2.62327
KAPCA_BOVIN	23653516	CDK2_HUMAN Fu	444345 1c	1AQ1	1STC	2R3G	34.78		Sc=6.1409, min distance = 2.487025
KAPCA_BOVIN	23653524	CDK2_HUMAN Fu	444345 1c	1AQ1	1STC	2R3P	34.78		Sc=6.2656, min distance = 2.443569
KAPCA_BOVIN	23727982	CDK2_HUMAN Fu	444345 1c	1AQ1	1STC	3BHU	34.78		Sc=6.27324, min distance = 1.58771
KAPCA_BOVIN	2403 1uvr	PDPK1_HUMAN Fu	444345 1c	1OKY	1STC	1UVR	39.18		Sc=6.39117, min distance = 2.00144
KAPCA_BOVIN	24752838	CHK1_HUMAN Fu	444345 1c	1NVR	1STC	2QHM	33.01		Sc=6.036, min distance = 2.8977789
KAPCA_BOVIN	24851689	PIM1_HUMAN Fu	444345 1c	1YHS	1STC	3CY2	30.06		Sc=6.05771, min distance = 2.44382
KAPCA_BOVIN	24901723	CDK2_HUMAN Fu	444345 1c	1AQ1	1STC	2W06	34.78		Sc=6.08059, min distance = 2.42376
KAPCA_BOVIN	24905143	PK3CG_HUMAN Fu	444345 1c	1E8Z	1STC	2V4L			Sc=6.1096, min distance = 2.760260
KAPCA_BOVIN	24905144	PK3CG_HUMAN Fu	444345 1c	1E8Z	1STC	3ENE			Sc=6.10026, min distance = 2.28507
KAPCA_BOVIN	24963048	AKT2_HUMAN Fu	11175137	2UW9	2UW7	3E87	44.86		Sc=6.43552, min distance = 2.58238
KAPCA_BOVIN	24963048	AKT2_HUMAN Fu	11857236	3E88	3E8C	3E87	44.86		Sc=6.40642, min distance = 2.39776
KAPCA_BOVIN	24963048	AKT2_HUMAN Fu	24963049	3E8D	3E8E	3E87	44.86		Sc=6.42637, min distance = 2.64133
KAPCA_BOVIN	3064778 H	ROCK1_HUMAN Fu	448042 2e	2ETR	1Q8T	2ETK	37.61		Sc=6.12393, min distance = 1.94426
KAPCA_BOVIN	3064778 H	ROCK1_HUMAN Fu	448043 2g	3D9V	1Q8U	2ETK	37.61		Sc=6.06153, min distance = 2.25798
KAPCA_BOVIN	33113 gam	IRAK4_HUMAN Fu	444345 1c	2NRY	1STC	2OID	35		Sc=5.68188, min distance = 1.66555
KAPCA_BOVIN	3973 nche	PIM1_HUMAN Fu	444345 1c	1YHS	1STC	1YI3	30.06		Sc=5.88117, min distance = 2.36758
KAPCA_BOVIN	4369136 C	CDK2_HUMAN Fu	444345 1c	1AQ1	1STC	1DM2	34.78		Sc=6.19844, min distance = 2.58460
KAPCA_BOVIN	445840 di	CDK2_HUMAN Fu	444345 1c	1AQ1	1STC	1GII	34.78		Sc=6.11323, min distance = 2.18276

KAPCA_BOVIN I	445966 O6	CDK2_HUMAN Fu	444345 1c	1AQ1	1STC	1H0V	34.78		Sc=6.08739, min distance = 2.552043
KAPCA_BOVIN I	447649 1c	CDK2_HUMAN Fu	444345 1c	1AQ1	1STC	1OI9	34.78		Sc=6.34252, min distance = 2.553108
KAPCA_BOVIN I	447654 CI	CDK2_HUMAN Fu	444345 1c	1AQ1	1STC	1OIT	34.78		Sc=6.15711, min distance = 2.362562
KAPCA_BOVIN I	447916 ac	RIO1_ARCFU Fu	5957 Aden	1ZP9	1Q24	1ZTF		0.94	Sc=6.12413, min distance = 2.282780
KAPCA_BOVIN I	447962 1p	CDK2_HUMAN Fu	444345 1c	1AQ1	1STC	2C5N	34.78		Sc=6.24105, min distance = 2.178337
KAPCA_BOVIN I	448293 CI	CDK2_HUMAN Fu	444345 1c	1AQ1	1STC	1R78	34.78		Sc=6.42616, min distance = 2.086084
KAPCA_BOVIN I	4565 1h1r	CDK2_HUMAN Fu	444345 1c	1AQ1	1STC	1H1R	34.78		Sc=6.24359, min distance = 2.659440
KAPCA_BOVIN I	4566 1h1s	CDK2_HUMAN Fu	444345 1c	1AQ1	1STC	1H1S	34.78		Sc=6.30204, min distance = 2.496024
KAPCA_BOVIN I	5288710 C	CDK2_HUMAN Fu	444345 1c	1AQ1	1STC	1KE7	34.78		Sc=6.35124, min distance = 2.043372
KAPCA_BOVIN I	5289411 1	CDK2_HUMAN Fu	444345 1c	1AQ1	1STC	1OGU	34.78		Sc=6.44738, min distance = 2.239998
KAPCA_BOVIN I	5326844 C	CDK2_HUMAN Fu	444345 1c	1AQ1	1STC	1URW	34.78		Sc=6.42039, min distance = 2.056800
KAPCA_BOVIN I	5327121 I	PIM1_HUMAN Fu	444345 1c	1YHS	1STC	2C3I	30.06		Sc=6.00097, min distance = 2.516966
KAPCA_BOVIN I	5327132 C	CDK2_HUMAN Fu	444345 1c	1AQ1	1STC	2C6I	34.78		Sc=6.27951, min distance = 2.373484
KAPCA_BOVIN I	5331010 c	CDK2_HUMAN Fu	444345 1c	1AQ1	1STC	2BKZ	34.78		Sc=6.2435, min distance = 2.3631927
KAPCA_BOVIN I	6022 Aden	CLCN5_HUMAN F	5957 Aden	2J9L	1Q24	2JA3		0.99	Sc=6.41728, min distance = 2.312820
KAPCA_BOVIN I	6022 Aden	DDL_THET8 Full	5957 Aden	2ZDQ	1Q24	2ZDH		0.99	Sc=6.43556, min distance = 2.453731
KAPCA_BOVIN I	6022 Aden	FAK1_HUMAN Fu	5957 Aden	2IJM	1Q24	1MP8	26.64	0.99	Sc=6.40755, min distance = 2.071376
KAPCA_BOVIN I	6022 Aden	GSK3B_HUMAN F	444345 1c	1Q3D	1STC	1J1C	31.63		Sc=6.41531, min distance = 1.364264
KAPCA_BOVIN I	6022 Aden	PDK2_HUMAN Fu	5957 Aden	2BU2	1Q24	2BU8		0.99	Sc=6.41775, min distance = 2.371290
KAPCA_BOVIN I	6022 Aden	PURK_ECOLI Fu	5957 Aden	3ETH	1Q24	3ETJ		0.99	Sc=6.40672, min distance = 1.807541
KAPCA_BOVIN I	6022 Aden	PURP_METJA Fu	5957 Aden	2R7L	1Q24	2R7N		0.99	Sc=6.39408, min distance = 2.003959
KAPCA_BOVIN I	6022 Aden	REX_BACSU Full	5957 Aden	2VT3	1Q24	2VT2		0.99	Sc=6.45105, min distance = 1.998296
KAPCA_BOVIN I	6022 Aden	RK_BOVIN Full=	5957 Aden	3C4W	1Q24	3C4Z		0.99	Sc=6.42254, min distance = 2.507509
KAPCA_BOVIN I	6022 Aden	SECA_ECOLI Fu	5957 Aden	2FSG	1Q24	2FSI		0.99	Sc=5.98903, min distance = 2.081901
KAPCA_BOVIN I	6083 aden	PURP_METJA Fu	5957 Aden	2R7L	1Q24	2R7M		0.98	Sc=6.3274, min distance = 2.4145444
KAPCA_BOVIN I	6083 aden	PURP_PYRFU Fu	5957 Aden	2R86	1Q24	2R85		0.98	Sc=6.35386, min distance = 2.701177
KAPCA_BOVIN I	6083 aden	Y059_METJA Fu	5957 Aden	2J9C	1Q24	2J9D		0.98	Sc=6.31685, min distance = 1.880619
KAPCA_BOVIN I	60961 ade	PIM1_HUMAN Fu	444345 1c	1YHS	1STC	1YI4	30.06		Sc=6.17235, min distance = 2.605825
KAPCA_BOVIN I	6102670	CDK2_HUMAN Fu	444345 1c	1AQ1	1STC	1YKR	34.78		Sc=6.27679, min distance = 2.006777
KAPCA_BOVIN I	6420138 2	CDK2_HUMAN Fu	444345 1c	1AQ1	1STC	2UUE	34.78		Sc=6.36207, min distance = 1.892479
KAPCA_BOVIN I	6420139 C	CDK2_HUMAN Fu	444345 1c	1AQ1	1STC	2C5V	34.78		Sc=6.23091, min distance = 2.201892
KAPCA_BOVIN I	6540273 2	CHK1_HUMAN Fu	444345 1c	1NVR	1STC	2CGV	33.01		Sc=6.14938, min distance = 2.704509
KAPCA_BOVIN I	6540279 C	ROCK2_BOVIN F	448042 2e	2H9V	1Q8T	2F2U			Sc=5.98801, min distance = 2.924928
KAPCA_BOVIN I	656971 CI	CDK2_HUMAN Fu	444345 1c	1AQ1	1STC	1Y8Y	34.78		Sc=6.03747, min distance = 2.451156
KAPCA_BOVIN I	6918852 2	CDK2_HUMAN Fu	444345 1c	1AQ1	1STC	2FVD	34.78		Sc=6.34353, min distance = 2.009106
KAPCA_BOVIN I	9549254 2	CDK2_HUMAN Fu	444345 1c	1AQ1	1STC	2IW6	34.78		Sc=6.46961, min distance = 2.065552
KAPCA_MOUSE I	6021 inos	DEOD_ECOLI Fu	60961 ade	1PK7	1FMO	1PR0		0.79	Sc=6.26369, min distance = 2.056225

KAPCA_MOUSE	6021 inos	TMPC_TREPA Fu	60961 ade	2FQY	1FMO	2FQW		0.79	Sc=6.27193, min distance = 2.48154
KAPCA_MOUSE	6802 gua	A5KE01_PLAVI	60961 ade	2PGF	1FMO	2QVN		0.79	Sc=6.31685, min distance = 2.44861
KAPCA_MOUSE	6802 gua	TMPC_TREPA Fu	60961 ade	2FQY	1FMO	2FQX		0.79	Sc=6.33741, min distance = 2.50546
KAPCA_MOUSE	72443 for	DEOD_ECOLI Fu	60961 ade	1PK7	1FMO	1A69			Sc=6.29221, min distance = 2.12151
KAPCA_MOUSE	72443 for	MTAP_SULSO Fu	60961 ade	1JDV	1FMO	1JDZ			Sc=6.25934, min distance = 1.79278
KAPCA_MOUSE	97184 6-	DEOD_ECOLI Fu	60961 ade	1PK7	1FMO	1OVG		0.9	Sc=6.21122, min distance = 1.25459
KAPCA_PIG Fu	16750062	CSK2A_MAIZE F	33113 ga	1LP4	1CDK	2OXD	26.83		Sc=5.69986, min distance = 2.11579
KAPCA_PIG Fu	1694 TBB	CSK2A_MAIZE F	33113 ga	1LP4	1CDK	1J91	26.83		Sc=5.60997, min distance = 2.66734
KAPCA_PIG Fu	3064778	ROCK1_HUMAN F	33113 ga	2V55	1CDK	2ETK	36.39		Sc=6.11325, min distance = 2.01245
KAPCA_PIG Fu	3547 Fas	ROCK1_HUMAN F	33113 ga	2V55	1CDK	2ESM	36.39		Sc=5.99721, min distance = 2.05088
KAPCA_PIG Fu	439353 be	BACR_HALSA Fu	15600 DEC	1XJI	1CTP	1IW6			Sc=5.71966, min distance = 2.03220
KAPCA_PIG Fu	448043 2	ROCK1_HUMAN F	33113 ga	2V55	1CDK	3D9V	36.39		Sc=6.04484, min distance = 1.89472
KAPCA_PIG Fu	5326976	CSK2A_MAIZE F	33113 ga	1LP4	1CDK	1ZOE	26.83		Sc=5.71195, min distance = 2.05802
KAPCA_PIG Fu	91532 AM	PURT_ECOLI Fu	33113 ga	1EYZ	1CDK	1KJI		0.99	Sc=6.51027, min distance = 2.43333
KAPS_PENCH F	10214 PA	PAPS1_HUMAN F	10238 Ade	1XNJ	1M7G	2OFX	58.99	0.99	Sc=6.04092, min distance = 1.18955
KAPS_PENCH F	12876354	PAPS1_HUMAN F	10238 Ade	1XNJ	1M7G	2PEZ	58.99	0.94	Sc=6.39828, min distance = 1.49373
KAPS_PENCH F	444564 A	BIOD_ECOLI Fu	6022 Aden	1DAD	1M7G	1BS1		0.91	Sc=5.65072, min distance = 2.37892
KAPS_PENCH F	445940 O	CDK2_HUMAN Fu	6022 Aden	1GY3	1M7G	1GZ8			Sc=6.00406, min distance = 2.09074
KAPS_PENCH F	4565 1h1r	CDK2_HUMAN Fu	6022 Aden	1GY3	1M7G	1H1R			Sc=6.01103, min distance = 2.66637
KAPS_PENCH F	5957 Ade	SECA_ECOLI Fu	6022 Aden	2FSI	1M7G	2FSG		0.99	Sc=5.68188, min distance = 2.29891
KAR3_YEAST F	23653515	CDK2_HUMAN Fu	6022 Aden	1GY3	1F9V	2R3F			Sc=6.21274, min distance = 0.82901
KAR3_YEAST F	25021197	CDK2_HUMAN Fu	6022 Aden	1GY3	1F9V	3EOC			Sc=6.22054, min distance = 2.21597
KAR3_YEAST F	2608 1js	CDK2_HUMAN Fu	6022 Aden	1GY3	1F9V	1JSV			Sc=6.19898, min distance = 1.07126
KAR3_YEAST F	444842 C	CDK2_HUMAN Fu	6022 Aden	1GY3	1F9V	1CKP			Sc=5.6348, min distance = 2.10223
KAR3_YEAST F	446090 1	NDKC_DICDI Fu	6022 Aden	1KDN	1F9V	1HIY		0.97	Sc=5.94868, min distance = 1.29418
KAR3_YEAST F	4565 1h1r	CDK2_HUMAN Fu	6022 Aden	1GY3	1F9V	1H1R			Sc=6.11788, min distance = 1.45814
KAR3_YEAST F	4566 1h1s	CDK2_HUMAN Fu	6022 Aden	1GY3	1F9V	1H1S			Sc=6.21316, min distance = 2.57499
KAR3_YEAST F	6083 ade	PURP_METJA Fu	6022 Aden	2R7N	1F9V	2R7M		0.99	Sc=5.87191, min distance = 2.31661
KAR3_YEAST F	6918710	CDK2_HUMAN Fu	6022 Aden	1GY3	1F9V	3EJ1			Sc=6.28423, min distance = 1.09326
KARG_LIMPO F	122108 Ri	NDKC_DICDI Fu	6022 Aden	1KDN	1M15	1MN9			Sc=5.94032, min distance = 1.54329
KARG_LIMPO F	123831 N,	NOS3_BOVIN Fu	6322 L-a	4NSE	1M15	7NSE		0.89	Sc=6.00361, min distance = 1.70173
KARG_LIMPO F	123895 1	NOS3_BOVIN Fu	6322 L-a	4NSE	1M15	5NSE		0.96	Sc=5.94637, min distance = 2.07708
KARG_LIMPO F	181426 2-	NOS3_BOVIN Fu	6322 L-a	4NSE	1M15	1DM6			Sc=5.75117, min distance = 1.57707
KARG_LIMPO F	347590 S-	NOS3_BOVIN Fu	6322 L-a	4NSE	1M15	1D1V			Sc=5.66434, min distance = 2.01137
KARG_LIMPO F	439202 L-	NOS3_BOVIN Fu	6322 L-a	4NSE	1M15	6NSE			Sc=5.78152, min distance = 2.42343
KARG_LIMPO F	445940 O	CDK2_HUMAN Fu	6022 Aden	1GY3	1M15	1GZ8			Sc=6.25479, min distance = 2.04857
KARG_LIMPO F	445966 O	CDK2_HUMAN Fu	6022 Aden	1GY3	1M15	1H0V			Sc=6.32499, min distance = 2.33819

KARG_LIMPO Fu	446090 1h	NDKC_DICDI Ful	6022 Aden	1KDN	1M15	1HIY	0.97	Sc=6.48153, min distance = 1.67370
KARG_LIMPO Fu	446122 S2	ARGI1_RAT Ful	6322 L-ar	1T5G	1M15	3E9B		Sc=5.60659, min distance = 2.25439
KARG_LIMPO Fu	447017 CI	NDKC_DICDI Ful	6022 Aden	1KDN	1M15	1LWX		Sc=6.46119, min distance = 1.43471
KARG_LIMPO Fu	447955 1p	CDK2_HUMAN Ful	6022 Aden	1GY3	1M15	1PXI		Sc=5.84946, min distance = 1.98946
KARG_LIMPO Fu	4564 1e1v	CDK2_HUMAN Ful	6022 Aden	1GY3	1M15	1E1V		Sc=6.25975, min distance = 1.63596
KARG_LIMPO Fu	5327148 C	IPKA_RABIT Ful	6022 Aden	1JBP	1M15	2ERZ		Sc=6.22749, min distance = 2.11643
KARG_LIMPO Fu	5957 Aden	PUR7_METJA Ful	6022 Aden	2YZL	1M15	2Z02	0.99	Sc=6.49312, min distance = 2.21710
KARG_LIMPO Fu	5957 Aden	PURP_METJA Ful	6022 Aden	2R7N	1M15	2R7L	0.99	Sc=6.06121, min distance = 2.06055
KARG_LIMPO Fu	6031 Uric	DCK_HUMAN Ful	6022 Aden	1P5Z	1M15	2ZIA		Sc=6.36127, min distance = 1.96753
KARG_LIMPO Fu	6083 aden	PURP_METJA Ful	6022 Aden	2R7N	1M15	2R7M	0.99	Sc=6.41112, min distance = 2.10108
KARG_LIMPO Fu	60961 ade	IPKA_RABIT Ful	6022 Aden	1JBP	1M15	1FMO	0.95	Sc=6.30124, min distance = 2.07505
KARG_LIMPO Fu	6132 Cyti	O33839_THEMA I	6022 Aden	1XJK	1M15	1XJN		Sc=6.38695, min distance = 1.74157
KARG_LIMPO Fu	6274 hist	ARGT_SALTY Ful	6322 L-ar	1LAF	1M15	1LAG		Sc=5.79287, min distance = 2.31392
KARG_LIMPO Fu	65051 ddT	NDKC_DICDI Ful	6022 Aden	1KDN	1M15	1F3F		Sc=5.99654, min distance = 2.03386
KARG_LIMPO Fu	657087 CI	ARGI1_RAT Ful	6322 L-ar	1T5G	1M15	1T4T	0.76	Sc=5.75366, min distance = 2.37983
KARG_LIMPO Fu	6804 guar	NDK_PYRHO Ful	6022 Aden	2DYA	1M15	2DXF	0.8	Sc=6.44695, min distance = 1.97423
KARG_LIMPO Fu	8977 1dar	PARM_ECOLX Ful	6022 Aden	1MWM	1M15	2ZGY	0.8	Sc=6.49764, min distance = 2.14379
KARG_LIMPO Fu	9085 homc	NOS3_BOVIN Ful	6322 L-ar	4NSE	1M15	1DM7	0.9	Sc=5.94444, min distance = 2.22982
KARG_LIMPO Fu	91532 AMI	KIF1A_MOUSE Fu	6022 Aden	1I5S	1M15	1I6I	0.99	Sc=6.55296, min distance = 2.07429
KAT1_HUMAN Fu	378 4-nit	APEH_AERPE Ful	6140 L-ph	2HU5	1W7M	1VE7		Sc=5.77335, min distance = 2.18103
KAT1_HUMAN Fu	444293 PI	HIS8_ECOLI Ful	1053 pyri	1FG7	1W7N	1GEY	0.89	Sc=6.04178, min distance = 2.40314
KAT1_HUMAN Fu	6305 L-tr	THER_BACTH Ful	6140 L-ph	1OS0	1W7M	1THL		Sc=5.88361, min distance = 2.04519
KATG2_HALMA I	11957363	HBA_HUMAN Ful	444124 HE	1NQP	1ITK	1RQA	0.81	Sc=6.77045, min distance = 2.13000
KATG2_HALMA I	11957370	HMOX1_HUMAN Fu	444124 HE	1OZW	1ITK	1TWN		Sc=6.60698, min distance = 2.09998
KATG2_HALMA I	11957371	HMOX1_HUMAN Fu	444124 HE	1OZW	1ITK	1TWR		Sc=6.60998, min distance = 2.34560
KATG2_HALMA I	11957385	MYG_PHYCA Ful	444124 HE	1U7R	1ITK	2CMM		Sc=5.95027, min distance = 2.43871
KATG2_HALMA I	444207 He	NOS2_MOUSE Ful	444124 HE	2ORT	1ITK	1NOS	0.99	Sc=6.64778, min distance = 2.09032
KATG2_HALMA I	444522 HE	BFR_ECOLI Ful	444124 HE	1BFR	1ITK	1BCF	0.99	Sc=6.6739, min distance = 2.122831
KATG2_HALMA I	444522 HE	HBA_PAGBE Ful	444124 HE	1S5X	1ITK	1PBX	0.99	Sc=6.66634, min distance = 2.33278
KATG2_HALMA I	444522 HE	HBB_PAGBE Ful	444124 HE	1S5X	1ITK	1PBX	0.99	Sc=6.66634, min distance = 2.11196
KATG2_HALMA I	444522 HE	NOSO_BACSU Ful	444124 HE	2AN0	1ITK	2FC1	0.99	Sc=6.7649, min distance = 2.283172
KATG2_HALMA I	446348 CI	HBA_HUMAN Ful	444124 HE	1NQP	1ITK	1IRD	0.9	Sc=6.72433, min distance = 2.03196
KATG_BURPS Fu	11957330	CCPR_YEAST Ful	444098 HE	2EUT	2DV1	1BEQ	0.81	Sc=6.72175, min distance = 2.22773
KATG_BURPS Fu	11957385	MYG_PHYCA Ful	444098 HE	1A6M	2DV1	2CMM		Sc=6.08257, min distance = 2.63362
KATG_BURPS Fu	11970219	CCPR_YEAST Ful	444098 HE	2EUT	2DV1	1BEM	0.83	Sc=6.66756, min distance = 2.27986
KATG_BURPS Fu	11970220	CCPR_YEAST Ful	444098 HE	2EUT	2DV1	1BEP	0.8	Sc=6.75607, min distance = 2.20102
KATG_BURPS Fu	11970221	CCPR_YEAST Ful	444098 HE	2EUT	2DV1	1BES	0.83	Sc=6.72337, min distance = 2.20502

KATG_BURPS F	11970242	CCPR_YEAST Fu	444098 HE	2EUT	2DV1	1CPE	0.83	Sc=6.66634, min distance = 2.35208
KATG_BURPS F	126994 CI	CCPR_YEAST Fu	444098 HE	2EUT	2DV1	1Z53	0.77	Sc=6.66542, min distance = 2.66071
KATG_BURPS F	15342951	ALBU_HUMAN Fu	444098 HE	1N5U	2DV1	3B9L		Sc=6.17643, min distance = 2.08211
KATG_BURPS F	446332 CI	MYG_PHYCA Ful	444098 HE	1A6M	2DV1	1IOP	0.82	Sc=6.56808, min distance = 2.43826
KATG_BURPS F	447168 ZE	CCPR_YEAST Fu	444098 HE	2EUT	2DV1	1ML2	0.82	Sc=6.72657, min distance = 2.24856
KATG_MYCTU F	11957330	CCPR_YEAST Fu	444098 HE	2EUT	2CCA	1BEQ	0.81	Sc=6.73035, min distance = 2.23679
KATG_MYCTU F	11957353	HBA_HORSE Ful	444098 HE	2D5X	2CCA	1IWH	0.83	Sc=6.66377, min distance = 2.08828
KATG_MYCTU F	11970219	CCPR_YEAST Fu	444098 HE	2EUT	2CCA	1BEM	0.83	Sc=6.73094, min distance = 2.26787
KATG_MYCTU F	11970220	CCPR_YEAST Fu	444098 HE	2EUT	2CCA	1BEP	0.8	Sc=6.80119, min distance = 2.18595
KATG_MYCTU F	11970221	CCPR_YEAST Fu	444098 HE	2EUT	2CCA	1BES	0.83	Sc=6.72789, min distance = 2.18848
KATG_MYCTU F	11970242	CCPR_YEAST Fu	444098 HE	2EUT	2CCA	1CPE	0.83	Sc=6.67435, min distance = 2.33085
KATG_MYCTU F	126994 CI	CCPR_YEAST Fu	444098 HE	2EUT	2CCA	1Z53	0.77	Sc=6.72594, min distance = 2.73284
KATG_SYNE7 F	11957353	HBB_HORSE Ful	444124 HE	1Y8I	1UB2	1IWH	0.87	Sc=6.72887, min distance = 2.05349
KATG_SYNE7 F	11957370	HMOX1_HUMAN F	444124 HE	1OZW	1UB2	1TWN		Sc=6.60786, min distance = 1.92545
KATG_SYNE7 F	11957371	HMOX1_HUMAN F	444124 HE	1OZW	1UB2	1TWR		Sc=6.52315, min distance = 2.23628
KATG_SYNE7 F	11957373	CATE_ECOLI Fu	444124 HE	1IPH	1UB2	1P81	0.79	Sc=6.76919, min distance = 1.74955
KATG_SYNE7 F	11957385	MYG_PHYCA Ful	444124 HE	1U7R	1UB2	2CMM		Sc=6.09878, min distance = 2.26072
KATG_SYNE7 F	444207 He	PER_COPCI Ful	444124 HE	1LY9	1UB2	1LY8	0.99	Sc=6.77568, min distance = 2.06802
KATG_SYNE7 F	444522 HE	HBB_PAGBE Ful	444124 HE	1S5X	1UB2	1PBX	0.99	Sc=6.72562, min distance = 2.11526
KATG_SYNE7 F	444522 HE	NOSO_BACSU Fu	444124 HE	2AN0	1UB2	2FC1	0.99	Sc=6.76424, min distance = 2.10471
KATG_SYNE7 F	446189 CI	HBG1_HUMAN Fu	444124 HE	1I3E	1UB2	1I3D	0.89	Sc=6.6684, min distance = 1.234342
KBAY_ECOLI F	439183 1i	TPIS_TRYBB Fu	4797 1tpw	1TTJ	1GVF	1IIH		Sc=5.7893, min distance = 2.005964
KBAY_ECOLI F	445225 CI	FUCA_ECOLI Fu	4797 1tpw	4FUA	1GVF	1E47		Sc=5.73556, min distance = 2.06918
KBAY_ECOLI F	754 3-Gly	FUCA_ECOLI Fu	4797 1tpw	4FUA	1GVF	1E48		Sc=5.74309, min distance = 2.01147
KBL_ECOLI Fu	445062 CI	GLYA_ECOLI Fu	1053 pyri	1EQB	1FC4	1DFO	0.95	Sc=6.31363, min distance = 2.51574
KCMB2_HUMAN	446115 NG	HEXB_HUMAN Fu	446429 1j	1NOW	1JAK	1NP0		Sc=6.02913, min distance = 2.42588
KCRM_RABIT F	188966 dA	PAPS1_HUMAN F	6022 Aden	1X6V	1U6R	2PEZ	0.98	Sc=6.45656, min distance = 2.37626
KCRM_RABIT F	444503 FU	NDKC_DICDI Fu	6022 Aden	1KDN	1U6R	1B99		Sc=6.32492, min distance = 1.58774
KCRM_RABIT F	444564 AD	BIOD_ECOLI Fu	6022 Aden	1DAD	1U6R	1BS1	0.91	Sc=6.38384, min distance = 2.03117
KCRM_RABIT F	444842 CI	CDK2_HUMAN Fu	6022 Aden	1GY3	1U6R	1CKP		Sc=5.65049, min distance = 2.30974
KCRM_RABIT F	444852 CI	ATPB_BOVIN Fu	6022 Aden	2CK3	1U6R	1COW	0.9	Sc=6.06422, min distance = 2.01859
KCRM_RABIT F	445940 O6	CDK2_HUMAN Fu	6022 Aden	1GY3	1U6R	1GZ8		Sc=6.24966, min distance = 2.50062
KCRM_RABIT F	445966 O6	CDK2_HUMAN Fu	6022 Aden	1GY3	1U6R	1H0V		Sc=6.31779, min distance = 2.30110
KCRM_RABIT F	446090 1h	NDKC_DICDI Fu	6022 Aden	1KDN	1U6R	1HIY	0.97	Sc=6.4718, min distance = 1.883767
KCRM_RABIT F	447916 ad	RIO1_ARCFU Fu	6022 Aden	1ZTH	1U6R	1ZTF	0.95	Sc=6.28168, min distance = 2.47746
KCRM_RABIT F	447955 1p	CDK2_HUMAN Fu	6022 Aden	1GY3	1U6R	1PXI		Sc=5.84157, min distance = 1.90140
KCRM_RABIT F	4564 1e1v	CDK2_HUMAN Fu	6022 Aden	1GY3	1U6R	1E1V		Sc=6.26061, min distance = 1.99841

KCRM_RABIT F	4565 1h1r	CDK2_HUMAN Fu	6022 Aden	1GY3	1U6R	1H1R		Sc=6.16117, min distance = 2.56389
KCRM_RABIT F	5327148 C	IPKA_RABIT Fu	6022 Aden	1JBP	1U6R	2ERZ		Sc=6.21937, min distance = 2.14124
KCRM_RABIT F	5957 Aden	BIOD_ECOLI Fu	6022 Aden	1DAD	1U6R	1A82	0.99	Sc=6.50279, min distance = 1.78667
KCRM_RABIT F	5957 Aden	MUTS_ECOLI Fu	6022 Aden	1OH7	1U6R	1W7A	0.99	Sc=6.51159, min distance = 2.09412
KCRM_RABIT F	5957 Aden	PURL_THEMA Fu	6022 Aden	2HRU	1U6R	2HS0	0.99	Sc=6.18712, min distance = 2.05744
KCRM_RABIT F	5957 Aden	RK_BOVIN Full=	6022 Aden	3C4Z	1U6R	3C4W	0.99	Sc=6.04117, min distance = 2.00773
KCRM_RABIT F	6031 Uric	O33839_THEMA I	6022 Aden	1XJK	1U6R	1XJG		Sc=6.37155, min distance = 2.10216
KCRM_RABIT F	6031 Uric	RIR1_YEAST Fu	6022 Aden	2CVX	1U6R	2CVV		Sc=6.37474, min distance = 2.05167
KCRM_RABIT F	6083 aden	NDK_PYRHO Full	6022 Aden	2DYA	1U6R	2DXD	0.99	Sc=6.39465, min distance = 1.79175
KCRM_RABIT F	6083 aden	PSPF_ECOLI Fu	6022 Aden	2C98	1U6R	2VII	0.99	Sc=6.38285, min distance = 2.34721
KCRM_RABIT F	6102784 A	PSPF_ECOLI Fu	6022 Aden	2C98	1U6R	2C99	0.99	Sc=6.51073, min distance = 2.21295
KCRM_RABIT F	6338562 C	MYS2_DICDI Fu	6022 Aden	1VOM	1U6R	1D0Y		Sc=6.09546, min distance = 2.24103
KCRM_RABIT F	6338566 C	MYS2_DICDI Fu	6022 Aden	1VOM	1U6R	1D1C		Sc=6.26737, min distance = 0.71857
KCRM_RABIT F	8977 1dar	NDK_PYRHO Full	6022 Aden	2DYA	1U6R	2DXE	0.8	Sc=6.47986, min distance = 1.92435
KCRM_RABIT F	8977 1dar	O33839_THEMA I	6022 Aden	1XJK	1U6R	1XJE	0.8	Sc=6.496, min distance = 2.3176270
KCRM_RABIT F	8977 1dar	RIR1_YEAST Fu	6022 Aden	2CVX	1U6R	2CVW	0.8	Sc=6.48789, min distance = 2.11883
KCRM_RABIT F	91532 AMP	BIOD_ECOLI Fu	6022 Aden	1DAD	1U6R	1DAG	0.99	Sc=6.53489, min distance = 2.00422
KCRM_RABIT F	91532 AMP	KIF1A_MOUSE F	6022 Aden	1I5S	1U6R	1I6I	0.99	Sc=6.54166, min distance = 2.06184
KCRM_RABIT F	91532 AMP	NIFH1_AZOVI F	6022 Aden	1FP6	1U6R	2AFK	0.99	Sc=6.59342, min distance = 1.01914
KCRM_RABIT F	91557 1tm	Q94M05_9VIRU I	6022 Aden	1W44	1U6R	1W48	0.99	Sc=6.5029, min distance = 2.1739001
KCRM_TORCA F	101543 3'	RNAS1_BOVIN F	6022 Aden	1O0H	1VRP	1O0N		Sc=6.02817, min distance = 2.13702
KCRM_TORCA F	188966 dA	PAPS1_HUMAN F	6022 Aden	1X6V	1VRP	2PEZ	0.98	Sc=6.4413, min distance = 2.326718
KCRM_TORCA F	444842 CI	CDK2_HUMAN Fu	6022 Aden	1GY3	1VRP	1CKP		Sc=5.63079, min distance = 2.35161
KCRM_TORCA F	445940 O6	CDK2_HUMAN Fu	6022 Aden	1GY3	1VRP	1GZ8		Sc=6.23395, min distance = 1.97575
KCRM_TORCA F	447916 ac	RIO1_ARCFU Fu	6022 Aden	1ZTH	1VRP	1ZTF	0.95	Sc=6.27543, min distance = 2.57558
KCRM_TORCA F	447955 1p	CDK2_HUMAN Fu	6022 Aden	1GY3	1VRP	1PXI		Sc=5.82837, min distance = 2.03188
KCRM_TORCA F	4564 1e1v	CDK2_HUMAN Fu	6022 Aden	1GY3	1VRP	1E1V		Sc=6.23538, min distance = 2.02262
KCRM_TORCA F	4565 1h1r	CDK2_HUMAN Fu	6022 Aden	1GY3	1VRP	1H1R		Sc=6.15579, min distance = 2.75861
KCRM_TORCA F	5327148 C	IPKA_RABIT Fu	6022 Aden	1JBP	1VRP	2ERZ		Sc=6.20928, min distance = 2.15553
KCRM_TORCA F	5957 Aden	MUTS_ECOLI Fu	6022 Aden	1OH7	1VRP	1W7A	0.99	Sc=6.4993, min distance = 2.003354
KCRM_TORCA F	5957 Aden	NIFH1_AZOVI F	6022 Aden	1FP6	1VRP	2C8V	0.99	Sc=6.51057, min distance = 1.70388
KCRM_TORCA F	5957 Aden	PDK3_HUMAN Fu	6022 Aden	1Y8O	1VRP	1Y8P	0.99	Sc=5.98891, min distance = 2.02416
KCRM_TORCA F	5957 Aden	PUR7_METJA Fu	6022 Aden	2YZL	1VRP	2Z02	0.99	Sc=6.48032, min distance = 2.19562
KCRM_TORCA F	5957 Aden	PURL_THEMA Fu	6022 Aden	2HRU	1VRP	2HS0	0.99	Sc=6.17139, min distance = 2.06745
KCRM_TORCA F	6031 Uric	O33839_THEMA I	6022 Aden	1XJK	1VRP	1XJG		Sc=6.36324, min distance = 2.26894
KCRM_TORCA F	6031 Uric	RIR1_YEAST Fu	6022 Aden	2CVX	1VRP	2CVV		Sc=6.36324, min distance = 2.11423
KCRM_TORCA F	6083 aden	HSP71_HUMAN F	6022 Aden	1S3X	1VRP	1XQS	0.99	Sc=6.40716, min distance = 2.20994

KCRM_TORCA Fu	6083 aden	NDK_PYRHO Full	6022 Aden	2DYA	1VRP	2DXD	0.99	Sc=6.37245, min distance = 1.64965
KCRM_TORCA Fu	6083 aden	PSPF_ECOLI Fu	6022 Aden	2C98	1VRP	2VII	0.99	Sc=6.36917, min distance = 2.372648
KCRM_TORCA Fu	6083 aden	PURP_METJA Fu	6022 Aden	2R7N	1VRP	2R7M	0.99	Sc=6.39465, min distance = 1.61075
KCRM_TORCA Fu	60961 ade	IPKA_RABIT Fu	6022 Aden	1JBP	1VRP	1FMO	0.95	Sc=6.28168, min distance = 2.36580
KCRM_TORCA Fu	65051 ddT	NDKC_DICDI Fu	6022 Aden	1KDN	1VRP	1F3F		Sc=5.96834, min distance = 2.10522
KCRM_TORCA Fu	8977 1dar	NDK_PYRHO Full	6022 Aden	2DYA	1VRP	2DXE	0.8	Sc=6.4716, min distance = 2.359331
KCRM_TORCA Fu	8977 1dar	PARM_ECOLX Fu	6022 Aden	1MWM	1VRP	2ZGY	0.8	Sc=6.46789, min distance = 2.08986
KCRM_TORCA Fu	8977 1dar	RIR1_YEAST Fu	6022 Aden	2CVX	1VRP	2CVW	0.8	Sc=6.47558, min distance = 2.22557
KCRM_TORCA Fu	91532 AMP	BIOD_ECOLI Fu	6022 Aden	1DAD	1VRP	1DAG	0.99	Sc=6.51962, min distance = 2.101118
KCRM_TORCA Fu	91557 1tm	Q94M05_9VIRU	6022 Aden	1W44	1VRP	1W48	0.99	Sc=6.05614, min distance = 2.14059
KCRS_HUMAN Fu	440317 AT	BCKD_RAT Full	6022 Aden	1GKZ	2GL6	1GJV	0.99	Sc=6.49293, min distance = 1.82275
KCRS_HUMAN Fu	444503 FU	NDKC_DICDI Fu	6022 Aden	1KDN	2GL6	1B99		Sc=6.33161, min distance = 1.94979
KCRS_HUMAN Fu	445966 O6	CDK2_HUMAN Fu	6022 Aden	1GY3	2GL6	1H0V		Sc=6.33354, min distance = 1.71037
KCRS_HUMAN Fu	447916 ac	RIO1_ARCFU Fu	6022 Aden	1ZTH	2GL6	1ZTF	0.95	Sc=6.30124, min distance = 2.50910
KCRS_HUMAN Fu	447955 1p	CDK2_HUMAN Fu	6022 Aden	1GY3	2GL6	1PXI		Sc=5.86266, min distance = 2.22998
KCRS_HUMAN Fu	448505 AD	NDKC_DICDI Fu	6022 Aden	1KDN	2GL6	1S5Z	0.93	Sc=6.50229, min distance = 1.44110
KCRS_HUMAN Fu	4564 1e1v	CDK2_HUMAN Fu	6022 Aden	1GY3	2GL6	1E1V		Sc=6.27773, min distance = 2.01640
KCRS_HUMAN Fu	5327148 C	IPKA_RABIT Fu	6022 Aden	1JBP	2GL6	2ERZ		Sc=6.22907, min distance = 1.89354
KCRS_HUMAN Fu	5957 Aden	MALK_ECOLI Fu	6022 Aden	2AWN	2GL6	1Q12	0.99	Sc=6.03875, min distance = 1.16354
KCRS_HUMAN Fu	5957 Aden	MUTS_ECOLI Fu	6022 Aden	1OH7	2GL6	1W7A	0.99	Sc=6.51851, min distance = 1.57633
KCRS_HUMAN Fu	5957 Aden	PUR7_METJA Fu	6022 Aden	2YZL	2GL6	2Z02	0.99	Sc=6.05421, min distance = 1.72550
KCRS_HUMAN Fu	5957 Aden	RK_BOVIN Full	6022 Aden	3C4Z	2GL6	3C4W	0.99	Sc=6.05135, min distance = 1.89620
KCRS_HUMAN Fu	6031 Uric	DCK_HUMAN Full	6022 Aden	1P5Z	2GL6	2ZIA		Sc=6.37474, min distance = 1.89069
KCRS_HUMAN Fu	6031 Uric	RIR1_YEAST Fu	6022 Aden	2CVX	2GL6	2CVV		Sc=6.38344, min distance = 1.85780
KCRS_HUMAN Fu	6083 aden	PURP_METJA Fu	6022 Aden	2R7N	2GL6	2R7M	0.99	Sc=6.40846, min distance = 2.07466
KCRS_HUMAN Fu	60961 ade	IPKA_RABIT Fu	6022 Aden	1JBP	2GL6	1FMO	0.95	Sc=6.31205, min distance = 1.99304
KCRS_HUMAN Fu	6852187 2	O57883_PYRHO	6022 Aden	1WNL	2GL6	2DTH	0.89	Sc=6.21357, min distance = 1.21489
KCRS_HUMAN Fu	8977 1dar	RIR1_YEAST Fu	6022 Aden	2CVX	2GL6	2CVW	0.8	Sc=6.50261, min distance = 1.866498
KCRS_HUMAN Fu	91532 AMP	BIOD_ECOLI Fu	6022 Aden	1DAD	2GL6	1DAG	0.99	Sc=6.52617, min distance = 2.01488
KCRS_HUMAN Fu	9543444 A	NDKC_DICDI Fu	6022 Aden	1KDN	2GL6	1MN7		Sc=6.02012, min distance = 1.33811
KCY_DICDI Fu	101544 2'	RNAS1_BOVIN Fu	6131 cyti	1RNN	1QF9	1ROB	1	Sc=6.29854, min distance = 2.13710
KCY_DICDI Fu	101609 Ur	RNAS1_BOVIN Fu	6131 cyti	1RNN	1QF9	1O0M	0.83	Sc=6.29542, min distance = 2.39881
KCY_DICDI Fu	24864080	CDK2_HUMAN Fu	6022 Aden	1GY3	1QF9	2VTR		Sc=5.7797, min distance = 2.962643
KCY_DICDI Fu	398148 1e	CDK2_HUMAN Fu	6022 Aden	1GY3	1QF9	1E1X		Sc=5.99458, min distance = 2.05165
KCY_DICDI Fu	445940 O6	CDK2_HUMAN Fu	6022 Aden	1GY3	1QF9	1GZ8		Sc=6.00759, min distance = 2.18810
KCY_DICDI Fu	4565 1h1r	CDK2_HUMAN Fu	6022 Aden	1GY3	1QF9	1H1R		Sc=6.09318, min distance = 2.26412
KCY_DICDI Fu	5326739 i	GSK3B_HUMAN Fu	6022 Aden	1J1C	1QF9	1Q41		Sc=6.12302, min distance = 2.18593

KCY_DICDI Fu	5327087 3	CDK2_HUMAN Fu	6022 Aden	1GY3	1QF9	2BPM		Sc=6.26472, min distance = 2.08704
KCY_DICDI Fu	6030 Urid	GLMU_HAEIN Fu	6031 Urid	2V0K	4UKD	2V0J	0.99	Sc=6.25523, min distance = 2.89046
KCY_DICDI Fu	6030 Urid	O87988_BORBR	6031 Urid	2PZM	4UKD	2PZL	0.99	Sc=6.22803, min distance = 2.48249
KCY_DICDI Fu	6132 Cyti	ECX1_PYRAB Fu	6031 Urid	2PNZ	4UKD	2PO2	0.83	Sc=6.33527, min distance = 2.02493
KCY_DICDI Fu	656946 CI	RNAS1_BOVIN Fu	6131 cyti	1RNN	1QF9	1W4Q		Sc=6.28136, min distance = 2.10142
KCY_DICDI Fu	72194 2-C	ENPL_CANFA Fu	6022 Aden	1TC6	1QF9	1QYE	0.89	Sc=6.10338, min distance = 2.02413
KDSA_AQUAE Fu	12598269	RPIB_MYCTU Fu	77982 Rib	2VVP	1ZHA	2VVO		Sc=6.10126, min distance = 0.69980
KDSA_AQUAE Fu	448888 ri	RPIA_THET2 Fu	188324 Ri	1UJ6	1FXQ	1UJ5		Sc=5.85332, min distance = 2.61474
KES1_YEAST Fu	444679 EF	ELIB_PHYCR Fu	5997 chol	1LRI	1ZHY	1BXM	0.96	Sc=5.98217, min distance = 2.98595
KEX2_YEAST Fu	51634 Mic	GLCM_HUMAN Fu	81462 Bis	2V3F	1R64	2V3D		Sc=5.8963, min distance = 2.529794
KGUA_ECOLI Fu	188347 pp	ARF1_BOVIN Fu	8977 1dar	1R8S	2AN9	1R8Q	0.99	Sc=6.07756, min distance = 2.17317
KGUA_ECOLI Fu	188347 pp	ARL5A_HUMAN Fu	8977 1dar	2H17	2AN9	1ZJ6	0.99	Sc=6.50482, min distance = 1.37385
KGUA_ECOLI Fu	444148 1a	RHA1_ARATH Fu	8977 1dar	2EFC	2AN9	2EFE	0.99	Sc=6.42759, min distance = 1.69068
KGUA_ECOLI Fu	446248 CI	RASH_HUMAN Fu	8977 1dar	2CE2	2AN9	1IAQ		Sc=6.04363, min distance = 2.27048
KGUA_ECOLI Fu	448405 CI	ARF1_RAT Full	8977 1dar	1RRG	2AN9	1RRF		Sc=6.43401, min distance = 1.66190
KGUA_ECOLI Fu	5496554 C	RASH_HUMAN Fu	8977 1dar	2CE2	2AN9	1GNQ		Sc=5.9626, min distance = 1.688978
KGUA_ECOLI Fu	6022 Aden	NDK_PYRHO Ful	8977 1dar	2DXE	2AN9	2DYA	0.8	Sc=6.41614, min distance = 2.41262
KGUA_ECOLI Fu	6022 Aden	O33839_THEMA	8977 1dar	1XJE	2AN9	1XJK	0.8	Sc=6.45701, min distance = 2.12701
KGUA_ECOLI Fu	6031 Urid	O33839_THEMA	8977 1dar	1XJE	2AN9	1XJG		Sc=6.35095, min distance = 2.12899
KGUA_ECOLI Fu	6083 aden	NDK_PYRHO Ful	8977 1dar	2DXE	2AN9	2DXD	0.79	Sc=5.8895, min distance = 2.003826
KGUA_ECOLI Fu	6132 Cyti	O33839_THEMA	8977 1dar	1XJE	2AN9	1XJN		Sc=6.35668, min distance = 1.94366
KGUA_ECOLI Fu	8582 Inos	PURA1_MOUSE Fu	8977 1dar	1LON	2AN9	1IWE	0.99	Sc=6.36603, min distance = 2.22003
KGUA_ECOLI Fu	93082 Gab	EFG_THETH Ful	8977 1dar	2BM0	2AN9	2J7K	0.99	Sc=6.06357, min distance = 2.00646
KGUA_MOUSE Fu	24864080	CDK2_HUMAN Fu	6022 Aden	1GY3	1LVG	2VTR		Sc=5.88645, min distance = 2.26640
KGUA_MOUSE Fu	445940 O6	CDK2_HUMAN Fu	6022 Aden	1GY3	1LVG	1GZ8		Sc=6.00047, min distance = 2.19306
KGUA_MOUSE Fu	4565 1h1r	CDK2_HUMAN Fu	6022 Aden	1GY3	1LVG	1H1R		Sc=6.08723, min distance = 1.54095
KGUA_MOUSE Fu	5288710 C	CDK2_HUMAN Fu	6022 Aden	1GY3	1LVG	1KE7		Sc=6.1464, min distance = 2.281412
KGUA_MOUSE Fu	5327134 C	CDK2_HUMAN Fu	6022 Aden	1GY3	1LVG	2C6L		Sc=6.06903, min distance = 2.30753
KGUA_MOUSE Fu	5957 Ader	BIOD_ECOLI Fu	6022 Aden	1DAD	1LVG	1A82	0.99	Sc=6.03769, min distance = 2.48713
KGUA_MOUSE Fu	8582 Inos	HGXR_TOXGO Fu	6804 guan	1QK3	1LVG	1QK4	0.99	Sc=6.44581, min distance = 1.50859
KGUA_MOUSE Fu	8582 Inos	HPRT_ECOLI Fu	6804 guan	1G9T	1LVG	1G9S	0.99	Sc=6.43469, min distance = 2.31053
KGUA_MOUSE Fu	91532 AMF	NIFH1_AZOVI Fu	6022 Aden	1FP6	1LVG	2AFK	0.99	Sc=6.06663, min distance = 2.37604
KGUA_MOUSE Fu	9926933 i	CDK2_HUMAN Fu	6022 Aden	1GY3	1LVG	2VTI		Sc=5.77469, min distance = 2.32100
KGUA_STAAC Fu	101543 3'	RNMC_MOMCH Fu	6804 guan	1J1F	2J41	1UCC		Sc=6.26373, min distance = 2.47988
KGUA_STAAC Fu	65059 Dec	RNAS1_BOVIN Fu	6804 guan	1RNC	2J41	2QCA	0.97	Sc=5.9228, min distance = 2.304474
KGUA_STAAC Fu	8582 Inos	HPRT_ECOLI Fu	6804 guan	1G9T	2J41	1G9S	0.99	Sc=6.3991, min distance = 2.137644
KGUA_YEAST Fu	6030 Urid	PYRR_BACCL Fu	6804 guan	1XZ8	1EX7	2IGB		Sc=6.26565, min distance = 1.91239

KGUA_YEAST F	65059 Dec	RNAS1_BOVIN F	6804 guan	1RNC	1EX7	2QCA	0.97	Sc=6.32041, min distance = 2.47921
KGUA_YEAST F	6802 guar	RNAS1_BOVIN F	6804 guan	1RNC	1EX7	1EOW	0.95	Sc=5.96026, min distance = 2.50788
KGUA_YEAST F	73323 Xar	HGXR_TOXGO Fu	6804 guan	1QK3	1EX7	1QK5	0.95	Sc=6.41938, min distance = 1.93554
KGUA_YEAST F	8582 Inos	HPRT_ECOLI Fu	6804 guan	1G9T	1EX7	1G9S	0.99	Sc=5.90214, min distance = 2.23904
KGUA_YEAST F	8582 Inos	RNAS1_BOVIN F	6804 guan	1RNC	1EX7	1Z6D	0.99	Sc=6.35571, min distance = 2.23572
KGUA_YEAST F	8977 1dar	GBP1_HUMAN Fu	6804 guan	2B8W	1EX7	2B92	0.99	Sc=6.45967, min distance = 1.59332
KHK_HUMAN Fu	188966 dA	HSLU_ECOLI Fu	33113 gar	1E94	2HW1	1G4A	0.97	Sc=6.37418, min distance = 2.34233
KHK_HUMAN Fu	3064778 H	ROCK1_HUMAN F	33113 gar	2V55	2HW1	2ETK		Sc=6.06024, min distance = 2.22947
KHK_HUMAN Fu	3547 Fasu	ROCK1_HUMAN F	33113 gar	2V55	2HW1	2ESM		Sc=5.97078, min distance = 2.70562
KHK_HUMAN Fu	444367 CI	CSK2A_MAIZE F	33113 gar	1LP4	2HW1	1OM1		Sc=6.24226, min distance = 2.20757
KHK_HUMAN Fu	444564 AI	MYS2_DICDI Fu	33113 gar	1MMN	2HW1	1W9I	0.91	Sc=6.39549, min distance = 2.41799
KHK_HUMAN Fu	445479 CI	CSK21_HUMAN F	33113 gar	2PVR	2HW1	3BQC		Sc=6.17217, min distance = 2.34283
KHK_HUMAN Fu	445479 CI	CSK2A_MAIZE F	33113 gar	1LP4	2HW1	1F0Q		Sc=6.22288, min distance = 2.10624
KHK_HUMAN Fu	448042 2e	ROCK1_HUMAN F	33113 gar	2V55	2HW1	2ETR		Sc=6.09202, min distance = 2.31960
KHK_HUMAN Fu	6083 ader	ENTP2_RAT Ful	33113 gar	3CJA	2HW1	3CJ7	0.97	Sc=5.8787, min distance = 2.4064282
KHK_HUMAN Fu	6083 ader	HSP71_HUMAN F	33113 gar	2E8A	2HW1	1XQS	0.97	Sc=6.31605, min distance = 2.01745
KHK_HUMAN Fu	60961 ade	PIM1_HUMAN Fu	33113 gar	1XR1	2HW1	1YI4	0.93	Sc=6.28264, min distance = 2.03620
KHK_HUMAN Fu	60961 ade	SKY1_YEAST Fu	33113 gar	1Q99	2HW1	1Q97	0.93	Sc=6.26252, min distance = 1.87031
KHK_HUMAN Fu	6338561 C	MYS2_DICDI Fu	33113 gar	1MMN	2HW1	1D0X		Sc=6.21075, min distance = 2.10062
KHK_HUMAN Fu	91532 AMI	IGF1R_HUMAN F	33113 gar	1JQH	2HW1	1K3A	0.99	Sc=6.45064, min distance = 2.21392
KHK_HUMAN Fu	91532 AMI	PURT_ECOLI Fu	33113 gar	1EYZ	2HW1	1KJI	0.99	Sc=6.4648, min distance = 2.560514
KHSE_METJA F	6031 Uric	DCK_HUMAN Ful	6022 Aden	1P5Z	1H74	2ZIA		Sc=6.26254, min distance = 2.21317
KHSE_METJA F	91532 AMI	F263_HUMAN Fu	6022 Aden	2AXN	1H74	2DWP	0.99	Sc=6.46207, min distance = 2.12537
KIF11_HUMAN	128882 1a	OXC_ECOLI Ful	6022 Aden	2Q28	1X88	2Q29	0.99	Sc=5.83562, min distance = 2.53970
KIF11_HUMAN	24916751	CDK2_HUMAN Fu	6022 Aden	1GY3	1X88	3DOG		Sc=6.28081, min distance = 2.35527
KIF11_HUMAN	444564 AI	BIOD_ECOLI Fu	6022 Aden	1DAD	1X88	1BS1	0.91	Sc=6.05413, min distance = 2.15632
KIF11_HUMAN	444564 AI	MYS2_DICDI Fu	6022 Aden	1VOM	1X88	1W9I	0.91	Sc=5.83233, min distance = 2.50406
KIF11_HUMAN	444852 CI	ATPB_BOVIN Fu	6022 Aden	2CK3	1X88	1COW	0.9	Sc=5.93159, min distance = 2.15757
KIF11_HUMAN	5957 Ader	RK_BOVIN Full	6022 Aden	3C4Z	1X88	3C4W	0.99	Sc=6.08916, min distance = 1.82133
KIF1A_MOUSE	16750062	CSK2A_MAIZE F	33113 gar	1LP4	1VFV	2OXD		Sc=5.65627, min distance = 2.18286
KIF1A_MOUSE	447004 CI	MYS2_DICDI Fu	33113 gar	1MMN	1VFV	1LVK		Sc=6.0519, min distance = 2.354159
KIF1A_MOUSE	448042 2e	ROCK1_HUMAN F	33113 gar	2V55	1VFV	2ETR		Sc=6.08885, min distance = 1.86930
KIF1A_MOUSE	448043 2g	ROCK1_HUMAN F	33113 gar	2V55	1VFV	3D9V		Sc=6.04119, min distance = 2.14031
KIF1A_MOUSE	5326976 1	CSK2A_MAIZE F	33113 gar	1LP4	1VFV	1ZOE		Sc=5.73334, min distance = 2.28246
KIF22_HUMAN	160355 rc	CDK2_HUMAN Fu	6022 Aden	1GY3	3BFN	3DDQ		Sc=6.43005, min distance = 2.60633
KIF22_HUMAN	23653515	CDK2_HUMAN Fu	6022 Aden	1GY3	3BFN	2R3F		Sc=5.95585, min distance = 2.18957
KIF22_HUMAN	24864077	CDK2_HUMAN Fu	6022 Aden	1GY3	3BFN	2VTN		Sc=6.18355, min distance = 2.02879

KIF22_HUMAN I	2608 1jsv	CDK2_HUMAN Fu	6022 Aden	1GY3	3BFN	1JSV		Sc=5.78579, min distance = 2.08938
KIF22_HUMAN I	447004 CI	MYS2_DICDI Fu	6022 Aden	1VOM	3BFN	1LVK		Sc=5.67413, min distance = 2.46179
KIF22_HUMAN I	4566 1h1s	CDK2_HUMAN Fu	6022 Aden	1GY3	3BFN	1H1S		Sc=6.22886, min distance = 2.64531
KIF22_HUMAN I	6338562 C	MYS2_DICDI Fu	6022 Aden	1VOM	3BFN	1D0Y		Sc=5.75645, min distance = 2.41580
KIF22_HUMAN I	91557 1tm	Q94M05_9VIRU I	6022 Aden	1W44	3BFN	1W48	0.99	Sc=6.00198, min distance = 1.61430
KIF22_HUMAN I	9991833 S	CDK2_HUMAN Fu	6022 Aden	1GY3	3BFN	2R3H		Sc=5.89401, min distance = 2.60988
KIF2A_HUMAN I	440317 AT	FTSK_PSEAE Fu	6022 Aden	2IUU	2GRY	2IUT	0.99	Sc=6.00361, min distance = 2.28826
KIF2C_MOUSE I	188966 dA	HSLU_ECOLI Fu	33113 gam	1E94	1V8K	1G4A	0.97	Sc=6.36398, min distance = 2.19091
KIF2C_MOUSE I	447004 CI	MYS2_DICDI Fu	6022 Aden	1VOM	1V8J	1LVK		Sc=5.84931, min distance = 2.09523
KIF2C_MOUSE I	448042 2e	ROCK1_HUMAN Fu	33113 gam	2V55	1V8K	2ETR		Sc=6.0679, min distance = 1.997920
KIF2C_MOUSE I	448043 2g	ROCK1_HUMAN Fu	33113 gam	2V55	1V8K	3D9V		Sc=5.85388, min distance = 2.14048
KIF2C_MOUSE I	5326976 1	CSK2A_MAIZE Fu	33113 gam	1LP4	1V8K	1ZOE		Sc=5.68516, min distance = 2.50227
KIF3C_HUMAN I	444564 AD	BIOD_ECOLI Fu	6022 Aden	1DAD	3B6V	1BS1	0.91	Sc=5.66741, min distance = 2.39079
KIF3C_HUMAN I	444564 AD	MYS2_DICDI Fu	6022 Aden	1VOM	3B6V	1W9I	0.91	Sc=5.61167, min distance = 2.50471
KIF3C_HUMAN I	448965 1u	HS90A_HUMAN Fu	6022 Aden	1BYQ	3B6V	1UY6		Sc=6.3183, min distance = 2.068850
KIF3C_HUMAN I	91557 1tm	Q94M05_9VIRU I	6022 Aden	1W44	3B6V	1W48	0.99	Sc=5.9975, min distance = 1.680058
KIF5C_MOUSE I	160355 rc	CDK2_HUMAN Fu	6022 Aden	1GY3	1VFZ	3DDQ		Sc=6.2701, min distance = 2.598450
KIF5C_MOUSE I	16750062	CSK2A_MAIZE Fu	33113 gam	1LP4	1VFV	2OXD		Sc=5.65627, min distance = 2.18286
KIF5C_MOUSE I	24779678	CSK2A_MAIZE Fu	33113 gam	1LP4	1VFV	2PVM		Sc=6.07084, min distance = 2.04705
KIF5C_MOUSE I	447004 CI	MYS2_DICDI Fu	33113 gam	1MMN	1VFV	1LVK		Sc=6.0519, min distance = 2.354159
KIF5C_MOUSE I	448042 2e	ROCK1_HUMAN Fu	33113 gam	2V55	1VFV	2ETR		Sc=6.08885, min distance = 1.86930
KIF5C_MOUSE I	448043 2g	ROCK1_HUMAN Fu	33113 gam	2V55	1VFV	3D9V		Sc=6.04119, min distance = 2.14031
KIF5C_MOUSE I	5326976 1	CSK2A_MAIZE Fu	33113 gam	1LP4	1VFV	1ZOE		Sc=5.73334, min distance = 2.28246
KIF5C_RAT Fu	1694 TBBt	CDK2_HUMAN Fu	6022 Aden	1GY3	2KIN	1P5E		Sc=5.65947, min distance = 1.79540
KIF9_HUMAN Fu	444564 AD	MYS2_DICDI Fu	6022 Aden	1VOM	2NR8	1W9I	0.91	Sc=5.7048, min distance = 2.654494
KIFC1_HUMAN I	11957393	FAK1_HUMAN Fu	6022 Aden	1MP8	2REP	2ETM		Sc=6.05263, min distance = 2.74630
KIFC1_HUMAN I	1694 TBBt	CDK2_HUMAN Fu	6022 Aden	1GY3	2REP	1P5E		Sc=5.71012, min distance = 1.65263
KIFC1_HUMAN I	23653526	CDK2_HUMAN Fu	6022 Aden	1GY3	2REP	2R3R		Sc=5.7952, min distance = 2.201765
KIFC1_HUMAN I	5289411 1	CDK2_HUMAN Fu	6022 Aden	1GY3	2REP	1OGU		Sc=6.40053, min distance = 2.11234
KIFC1_HUMAN I	5957 Ader	BIOD_ECOLI Fu	6022 Aden	1DAD	2REP	1A82	0.99	Sc=5.73824, min distance = 2.02381
KIFC1_HUMAN I	91557 1tm	Q94M05_9VIRU I	6022 Aden	1W44	2REP	1W48	0.99	Sc=5.99808, min distance = 1.69425
KIFC1_HUMAN I	9991833 S	CDK2_HUMAN Fu	6022 Aden	1GY3	2REP	2R3H		Sc=5.68878, min distance = 2.14490
KIFC3_HUMAN I	160355 rc	CDK2_HUMAN Fu	6022 Aden	1GY3	2H58	3DDQ		Sc=6.17957, min distance = 2.43194
KIFC3_HUMAN I	23653515	CDK2_HUMAN Fu	6022 Aden	1GY3	2H58	2R3F		Sc=5.93867, min distance = 2.07293
KIFC3_HUMAN I	25021197	CDK2_HUMAN Fu	6022 Aden	1GY3	2H58	3EOC		Sc=6.10554, min distance = 2.70057
KIFC3_HUMAN I	440317 AT	FTSK_PSEAE Fu	6022 Aden	2IUU	2H58	2IUT	0.99	Sc=5.71388, min distance = 2.15811
KIFC3_HUMAN I	447655 1c	CDK2_HUMAN Fu	6022 Aden	1GY3	2H58	1OIU		Sc=6.21316, min distance = 2.39656

KIFC3_HUMAN I	4565 1h1r	CDK2_HUMAN Ful	6022 Aden	1GY3	2H58	1H1R			Sc=6.19635, min distance = 2.409955
KIFC3_HUMAN I	4566 1h1s	CDK2_HUMAN Ful	6022 Aden	1GY3	2H58	1H1S			Sc=6.22132, min distance = 2.468565
KIFC3_HUMAN I	5289411 1	CDK2_HUMAN Ful	6022 Aden	1GY3	2H58	1OGU			Sc=6.37503, min distance = 2.416335
KIFC3_HUMAN I	91557 1tm	Q94M05_9VIRU I	6022 Aden	1W44	2H58	1W48		0.99	Sc=5.71078, min distance = 2.022552
KIME_RAT Ful	11608401	KAPCA_BOVIN Fu	5957 Aden	1Q24	1KVK	2UVY			Sc=6.0826, min distance = 2.2818689
KIME_RAT Ful	3540 1yds	KAPCA_BOVIN Fu	5957 Aden	1Q24	1KVK	1YDS			Sc=5.80802, min distance = 2.405304
KIME_RAT Ful	3547 Fasu	KAPCA_BOVIN Fu	5957 Aden	1Q24	1KVK	1Q8W			Sc=5.74491, min distance = 2.305350
KIME_RAT Ful	6022 Aden	BIOD_ECOLI Ful	5957 Aden	1A82	1KVK	1DAD		0.99	Sc=6.3797, min distance = 2.1318935
KIME_RAT Ful	6022 Aden	FAK1_HUMAN Ful	5957 Aden	2IJM	1KVK	1MP8		0.99	Sc=6.38772, min distance = 2.140741
KIME_RAT Ful	6022 Aden	KTHY_HUMAN Ful	5957 Aden	1E2Q	1KVK	1NN3		0.99	Sc=5.91368, min distance = 2.063030
KIME_RAT Ful	6022 Aden	MUTS_ECOLI Ful	5957 Aden	1W7A	1KVK	1OH7		0.99	Sc=6.38867, min distance = 1.757116
KIME_RAT Ful	6022 Aden	PDXK_SHEEP Ful	5957 Aden	1LHR	1KVK	1RFU		0.99	Sc=6.37677, min distance = 2.012126
KIME_RAT Ful	6022 Aden	PURK_ECOLI Ful	5957 Aden	3ETH	1KVK	3ETJ		0.99	Sc=5.93109, min distance = 1.788016
KIME_RAT Ful	6022 Aden	Q72H90_THET2 I	5957 Aden	2BEK	1KVK	2BEJ		0.99	Sc=5.90651, min distance = 2.006988
KIME_RAT Ful	6022 Aden	Y059_METJA Ful	5957 Aden	2J9C	1KVK	2J9D		0.99	Sc=6.37067, min distance = 2.203224
KIME_RAT Ful	6083 ader	PSPF_ECOLI Ful	5957 Aden	2C96	1KVK	2VII		0.98	Sc=6.2626, min distance = 2.2351890
KIME_RAT Ful	6830 guar	Q381M1_9TRYP I	5957 Aden	2Q0D	1KVK	2Q0E		0.8	Sc=5.98822, min distance = 2.220672
KIME_RAT Ful	6852207 M	KAPCA_BOVIN Fu	5957 Aden	1Q24	1KVK	2GNI			Sc=5.86061, min distance = 2.307344
KIME_RAT Ful	91532 AMP	BIOD_ECOLI Ful	5957 Aden	1A82	1KVK	1DAG		0.99	Sc=5.69716, min distance = 2.093138
KIME_RAT Ful	91532 AMP	Q5SJV7_THET8 I	5957 Aden	2Z08	1KVK	2Z09		0.99	Sc=6.476, min distance = 2.11429751
KINH_NEUCR Fu	447004 CI	MYS2_DICDI Ful	6022 Aden	1VOM	1GOJ	1LVK			Sc=5.86839, min distance = 2.107599
KIPN_BPT4 Fu	12599 dAM	DCK_HUMAN Ful	6022 Aden	1P5Z	1LTQ	2QRO		0.97	Sc=5.91635, min distance = 1.831807
KIPN_BPT4 Fu	13945 Dec	DCK_HUMAN Ful	6022 Aden	1P5Z	1LTQ	2QRN			Sc=6.15891, min distance = 2.366616
KIPN_BPT4 Fu	159354 To	DCK_HUMAN Ful	6022 Aden	1P5Z	1LTQ	2NO7			Sc=5.90835, min distance = 2.138794
KIPN_BPT4 Fu	24178104	GLNA_HUMAN Fu	6022 Aden	2OJW	1LTQ	2QC8			Sc=5.67797, min distance = 1.760107
KIPN_BPT4 Fu	445940 O6	CDK2_HUMAN Fu	6022 Aden	1GY3	1LTQ	1GZ8			Sc=5.93835, min distance = 2.261338
KIPN_BPT4 Fu	60825 lam	DCK_HUMAN Ful	6022 Aden	1P5Z	1LTQ	2NOA			Sc=5.77976, min distance = 2.579415
KITH_BACAN Fu	13711 dec	DNK_DROME Ful	5789 thym	1OT3	2J9R	2VP5			Sc=6.11838, min distance = 2.344464
KITH_BACAN Fu	445211 9-	KITH_HHV11 Fu	5789 thym	1P7C	2J9R	1E2I			Sc=6.06449, min distance = 1.882152
KITH_BACAN Fu	445212 1e	KITH_HHV11 Fu	5789 thym	1P7C	2J9R	1E2K			Sc=6.25881, min distance = 2.068398
KITH_BACAN Fu	446725 BV	KITH_HHV11 Fu	5789 thym	1P7C	2J9R	1KI4			Sc=6.35843, min distance = 1.969078
KITH_BACAN Fu	446726 AI	KITH_HHV11 Fu	5789 thym	1P7C	2J9R	1KI6		0.77	Sc=6.21419, min distance = 2.405587
KITH_BACAN Fu	447628 [(	KITH_HHV11 Fu	5789 thym	1P7C	2J9R	1OF1			Sc=6.26863, min distance = 2.071661
KITH_BACAN Fu	64968 dTI	KITH_UREPA Fu	5789 thym	2B8T	2J9R	2UZ3		0.9	Sc=6.03654, min distance = 2.191300
KITH_BACCR Fu	65070 dUI	DCD_ECOLI Ful	64968 dTI	2J4Q	2JA1	1XS1		0.9	Sc=6.40526, min distance = 2.205961
KITH_BACCR Fu	65091 dCI	DCD_ECOLI Ful	64968 dTI	2J4Q	2JA1	1XS4		0.75	Sc=6.40526, min distance = 2.078827
KITH_CLOAB Fu	447955 1p	CDK2_HUMAN Fu	6022 Aden	1GY3	1XX6	1PXI			Sc=5.69024, min distance = 2.112964

KITH_CLOAB F	4565 1h1r	CDK2_HUMAN Fu	6022 Aden	1GY3	1XX6	1H1R			Sc=6.1234, min distance = 2.1254528
KITH_EHV4 Fu	160355 ro	CDK2_HUMAN Fu	6022 Aden	1GY3	1P72	3DDQ			Sc=6.34185, min distance = 2.353584
KITH_EHV4 Fu	16046126	CDK2_HUMAN Fu	6022 Aden	1GY3	1P72	2DS1			Sc=6.45577, min distance = 2.001510
KITH_EHV4 Fu	24864080	CDK2_HUMAN Fu	6022 Aden	1GY3	1P72	2VTR			Sc=5.84834, min distance = 2.067929
KITH_EHV4 Fu	25021197	CDK2_HUMAN Fu	6022 Aden	1GY3	1P72	3EOC			Sc=6.36494, min distance = 1.709218
KITH_EHV4 Fu	445210 9-	KITH_HHV11 Fu	5789 thym	1P7C	1P6X	1E2I	38.72		Sc=6.09745, min distance = 1.631278
KITH_EHV4 Fu	445211 9-	KITH_HHV11 Fu	5789 thym	1P7C	1P6X	1E2I	38.72		Sc=6.10032, min distance = 1.674802
KITH_EHV4 Fu	445213 HY	KITH_HHV11 Fu	5789 thym	1P7C	1P6X	1E2N	38.72		Sc=6.38338, min distance = 2.539746
KITH_EHV4 Fu	445940 O6	CDK2_HUMAN Fu	6022 Aden	1GY3	1P72	1GZ8			Sc=6.18954, min distance = 1.601234
KITH_EHV4 Fu	446725 BV	KITH_HHV11 Fu	5789 thym	1P7C	1P6X	1KI4	38.72		Sc=6.3426, min distance = 2.5127620
KITH_EHV4 Fu	446726 AI	KITH_HHV11 Fu	5789 thym	1P7C	1P6X	1KI6	38.72	0.77	Sc=6.20176, min distance = 2.184967
KITH_EHV4 Fu	446727 Br	KITH_HHV11 Fu	5789 thym	1P7C	1P6X	1KI8	38.72	0.89	Sc=6.25436, min distance = 2.627418
KITH_EHV4 Fu	447004 CI	MYS2_DICDI Fu	6022 Aden	1VOM	1P72	1LVK			Sc=5.79776, min distance = 1.969026
KITH_EHV4 Fu	449088 1v	CDK2_HUMAN Fu	6022 Aden	1GY3	1P72	1VYZ			Sc=6.00274, min distance = 2.356401
KITH_EHV4 Fu	449489 Io	KITH_HHV11 Fu	5789 thym	1P7C	1P6X	3VTK	38.72	0.84	Sc=6.27855, min distance = 2.084592
KITH_EHV4 Fu	4564 1e1v	CDK2_HUMAN Fu	6022 Aden	1GY3	1P72	1E1V			Sc=6.10296, min distance = 2.417448
KITH_EHV4 Fu	4565 1h1r	CDK2_HUMAN Fu	6022 Aden	1GY3	1P72	1H1R			Sc=6.34131, min distance = 1.997681
KITH_EHV4 Fu	5288016 6	CDK2_HUMAN Fu	6022 Aden	1GY3	1P72	2B53			Sc=6.26534, min distance = 2.055652
KITH_EHV4 Fu	5288017 I	CDK2_HUMAN Fu	6022 Aden	1GY3	1P72	2B55			Sc=6.48247, min distance = 2.070375
KITH_EHV4 Fu	5326739 i	GSK3B_HUMAN F	6022 Aden	1J1C	1P72	1Q41			Sc=6.17468, min distance = 2.122162
KITH_EHV4 Fu	5327087 3	CDK2_HUMAN Fu	6022 Aden	1GY3	1P72	2BPM			Sc=6.33379, min distance = 2.317580
KITH_EHV4 Fu	5327135 D	CDK2_HUMAN Fu	6022 Aden	1GY3	1P72	2C6M			Sc=6.07084, min distance = 2.025622
KITH_EHV4 Fu	5905 idox	KITH_HHV11 Fu	5789 thym	1P7C	1P6X	1KI7	38.72	0.91	Sc=6.1269, min distance = 2.5619424
KITH_EHV4 Fu	5957 Aden	BIOD_ECOLI Fu	6022 Aden	1DAD	1P72	1A82		0.99	Sc=6.05887, min distance = 2.337231
KITH_EHV4 Fu	6031 Urid	DCK_HUMAN Ful	6022 Aden	1P5Z	1P72	2ZIA			Sc=5.88368, min distance = 2.427857
KITH_EHV4 Fu	6031 Urid	ECX1_PYRAB Fu	6022 Aden	2PO0	1P72	2PNZ			Sc=5.96088, min distance = 2.367645
KITH_EHV4 Fu	6083 aden	PSPF_ECOLI Fu	6022 Aden	2C98	1P72	2VII		0.99	Sc=6.02127, min distance = 2.285017
KITH_EHV4 Fu	6132 Cyti	ECX1_PYRAB Fu	6022 Aden	2PO0	1P72	2PO2			Sc=5.96504, min distance = 2.517979
KITH_EHV4 Fu	6132 Cyti	ECX2_PYRAB Fu	6022 Aden	2PO0	1P72	2PO2			Sc=5.95449, min distance = 2.298050
KITH_EHV4 Fu	656971 CI	CDK2_HUMAN Fu	6022 Aden	1GY3	1P72	1Y8Y			Sc=5.68406, min distance = 2.064658
KITH_EHV4 Fu	9700 thym	KITH_HHV11 Fu	5789 thym	1P7C	1P6X	1VTK	38.72	0.92	Sc=6.34067, min distance = 2.109947
KITH_EHV4 Fu	9994066 4	CDK2_HUMAN Fu	6022 Aden	1GY3	1P72	2VTJ			Sc=5.76418, min distance = 2.063852
KITH_HHV11 F	13712 dec	TYSY_ECOLI Fu	9700 thym	1TYS	1VTK	1BDU		0.83	Sc=6.07266, min distance = 2.590527
KITH_HHV11 F	398148 1e	CDK2_HUMAN Fu	6022 Aden	1GY3	2VTK	1E1X			Sc=6.14343, min distance = 2.155228
KITH_HHV11 F	444320 2'	TYSY_ECOLI Fu	9700 thym	1TYS	1VTK	1AOB		0.81	Sc=5.98592, min distance = 2.277422
KITH_HHV11 F	445269 CI	KTHY_MYCTU Fu	9700 thym	1GTV	1VTK	1W2H		0.85	Sc=6.3232, min distance = 2.1898038
KITH_HHV11 F	5288016 6	CDK2_HUMAN Fu	6022 Aden	1GY3	2VTK	2B53			Sc=6.26308, min distance = 2.048512

KITH_HHV11 Fu	5289411 1	CDK2_HUMAN Ful	6022 Aden	1GY3	2VTK	1OGU		Sc=6.19584, min distance = 2.269867
KITH_HHV11 Fu	5790 flox	DNK_DROME Full	5789 thym	1OT3	1P7C	2VP6	0.92	Sc=6.11642, min distance = 2.224190
KITH_HHV11 Fu	6030 Uric	PYR5_HUMAN Ful	449489 Id	2QCH	3VTK	2V30	0.9	Sc=6.25, min distance = 2.43568327
KITH_HHV11 Fu	65059 Dec	TYSY_ECOLI Ful	9700 thym	1TYS	1VTK	1TLC		Sc=6.38157, min distance = 2.212155
KITH_HHV11 Fu	656971 CI	CDK2_HUMAN Ful	6022 Aden	1GY3	2VTK	1Y8Y		Sc=5.66383, min distance = 2.091129
KITH_HHV11 Fu	72194 2-C	ENPL_CANFA Ful	6022 Aden	1TC6	2VTK	1QYE	0.89	Sc=6.12672, min distance = 1.901301
KITH_HHV11 Fu	9994066 4	CDK2_HUMAN Ful	6022 Aden	1GY3	2VTK	2VTJ		Sc=5.75665, min distance = 2.319696
KITH_HUMAN Fu	65051 ddT	DPOLL_HUMAN Fu	64968 dTT	2BCV	1W4R	1XSN	0.94	Sc=6.48386, min distance = 1.908024
KITH_HUMAN Fu	65091 dCT	DPOLL_HUMAN Fu	64968 dTT	2BCV	1W4R	2PFP	0.75	Sc=6.43446, min distance = 1.951530
KITH_THEMA Fu	1855 CID1	KITH_HHV11 Ful	9700 thym	1VTK	2QQ0	1E2P		Sc=6.15858, min distance = 2.261367
KITH_THEMA Fu	1865 6-hy	KITH_HHV11 Ful	5789 thym	1P7C	2QQ0	1E2M		Sc=6.02767, min distance = 2.318658
KITH_THEMA Fu	440141 9i	RNAS1_BOVIN Fu	6022 Aden	1O0H	2QQ0	1O0O	1	Sc=6.03544, min distance = 2.126729
KITH_THEMA Fu	445211 9-	KITH_HHV11 Ful	5789 thym	1P7C	2QQ0	1E2I		Sc=6.06561, min distance = 2.285027
KITH_THEMA Fu	445269 CI	KTHY_HUMAN Ful	9700 thym	1E2F	2QQ0	1E9B	0.85	Sc=6.39703, min distance = 2.581264
KITH_THEMA Fu	445269 CI	KTHY_YEAST Ful	9700 thym	1TMK	2QQ0	2TMK	0.85	Sc=6.38435, min distance = 2.460204
KITH_THEMA Fu	445269 CI	NT5M_HUMAN Ful	9700 thym	1Z4L	2QQ0	2JAU	0.85	Sc=6.3983, min distance = 2.3693351
KITH_THEMA Fu	446725 BV	KITH_HHV11 Ful	5789 thym	1P7C	2QQ0	1KI4		Sc=6.35, min distance = 2.148002560
KITH_THEMA Fu	446726 AI	KITH_HHV11 Ful	5789 thym	1P7C	2QQ0	1KI6	0.77	Sc=6.21183, min distance = 2.028042
KITH_THEMA Fu	447628 [(	KITH_HHV11 Ful	5789 thym	1P7C	2QQ0	1OF1		Sc=6.27053, min distance = 1.981857
KITH_THEMA Fu	447628 [(	KITH_HHV11 Ful	9700 thym	1VTK	2QQ0	1OF1		Sc=6.25014, min distance = 2.290042
KITH_THEMA Fu	449489 Id	KITH_HHV11 Ful	9700 thym	1VTK	2QQ0	3VTK	0.91	Sc=6.2737, min distance = 2.0377212
KITH_THEMA Fu	5790 flox	DNK_DROME Full	5789 thym	1OT3	2QQ0	2VP6	0.92	Sc=6.14964, min distance = 2.134398
KITH_THEMA Fu	6083 aden	RNAS1_BOVIN Fu	6022 Aden	1O0H	2QQ0	1Z6S	0.99	Sc=5.81284, min distance = 2.029630
KITH_UREPA Fu	13711 deo	DNK_DROME Full	5789 thym	1OT3	2B8T	2VP5		Sc=6.11407, min distance = 2.576652
KITH_UREPA Fu	13711 deo	DNK_DROME Full	64968 dTT	1ZM7	2UZ3	2VP5		Sc=6.10709, min distance = 2.175671
KITH_UREPA Fu	15993 dAT	DPO42_SULSO Fu	64968 dTT	1S0O	2UZ3	2AGQ		Sc=6.51347, min distance = 1.852746
KITH_UREPA Fu	15993 dAT	PRIM_BPT7 Full	64968 dTT	1CR1	2UZ3	1CR2		Sc=6.51745, min distance = 1.720478
KITH_UREPA Fu	1865 6-hy	KITH_HHV11 Ful	5789 thym	1P7C	2B8T	1E2M		Sc=6.03462, min distance = 2.376467
KITH_UREPA Fu	445210 9-	KITH_HHV11 Ful	5789 thym	1P7C	2B8T	1E2I		Sc=6.06338, min distance = 2.223506
KITH_UREPA Fu	445211 9-	KITH_HHV11 Ful	5789 thym	1P7C	2B8T	1E2I		Sc=6.06561, min distance = 2.176779
KITH_UREPA Fu	446726 AI	KITH_HHV11 Ful	5789 thym	1P7C	2B8T	1KI6	0.77	Sc=6.21831, min distance = 2.316079
KITH_UREPA Fu	447628 [(	KITH_HHV11 Ful	5789 thym	1P7C	2B8T	1OF1		Sc=6.26563, min distance = 2.230783
KITH_UREPA Fu	65091 dCT	DPOLL_HUMAN Fu	64968 dTT	2BCV	2UZ3	2PFP	0.75	Sc=6.41538, min distance = 2.149147
KITH_VACCA Fu	15342951	DNK_DROME Full	64968 dTT	1ZM7	2J87	2JJ8	0.81	Sc=6.27562, min distance = 1.443737
KITH_VACCA Fu	5789 thym	DNK_DROME Full	64968 dTT	1ZM7	2J87	1OT3	0.9	Sc=6.15278, min distance = 2.224858
KITH_VACCA Fu	65051 ddT	DPOLL_HUMAN Fu	64968 dTT	2BCV	2J87	1XSN	0.94	Sc=6.45183, min distance = 2.012675
KITH_VACCA Fu	65091 dCT	DPO42_SULSO Fu	64968 dTT	1S0O	2J87	2ASD	0.75	Sc=6.4004, min distance = 2.0453823

KITH_VACCA Fu	65091 dCT	DPOLL_HUMAN Fu	64968 dTT	2BCV	2J87	2PFP		0.75	Sc=5.9482, min distance = 2.0907924
KIT_HUMAN Fu	11387605	ABL1_HUMAN Fu	5291 Imat	2HYY	1T46	2E2B	38.78	0.9	Sc=6.69165, min distance = 2.389041
KIT_HUMAN Fu	11957417	CDK2_HUMAN Fu	6022 Aden	1GY3	1PKG	2I40	31.44		Sc=6.35268, min distance = 2.010031
KIT_HUMAN Fu	15942672	ABL1_MOUSE Fu	5291 Imat	1OPJ	1T46	2HZN	40		Sc=6.36187, min distance = 2.118035
KIT_HUMAN Fu	160355 rc	CDK2_HUMAN Fu	6022 Aden	1GY3	1PKG	3DDQ	31.44		Sc=6.41645, min distance = 1.480872
KIT_HUMAN Fu	16758212	GSK3B_HUMAN Fu	6022 Aden	1J1C	1PKG	2O5K	33.59		Sc=6.50706, min distance = 2.020412
KIT_HUMAN Fu	1694 TBBt	CDK2_HUMAN Fu	6022 Aden	1GY3	1PKG	1P5E	31.44		Sc=5.60554, min distance = 2.301741
KIT_HUMAN Fu	1707 1fvt	CDK2_HUMAN Fu	6022 Aden	1GY3	1PKG	1FVT	31.44		Sc=6.24889, min distance = 2.005138
KIT_HUMAN Fu	24860537	ABL1_MOUSE Fu	5291 Imat	1OPJ	1T46	3DK7	40	0.75	Sc=6.49564, min distance = 1.911291
KIT_HUMAN Fu	24905152	SRC_CHICK Full	5291 Imat	2OIQ	1T46	3EN6	39		Sc=6.37293, min distance = 2.363278
KIT_HUMAN Fu	24963033	CDK2_HUMAN Fu	6022 Aden	1GY3	1PKG	2W05	31.44		Sc=6.29343, min distance = 1.484395
KIT_HUMAN Fu	24963038	LCK_HUMAN Full	5291 Imat	2PL0	1T46	2ZM1	40.53		Sc=6.10607, min distance = 2.219315
KIT_HUMAN Fu	24978497	CDK2_HUMAN Fu	6022 Aden	1GY3	1PKG	3EID	31.44		Sc=6.61583, min distance = 1.184840
KIT_HUMAN Fu	25011744	SRC_CHICK Full	5291 Imat	2OIQ	1T46	3EL7	39		Sc=6.63538, min distance = 2.127962
KIT_HUMAN Fu	25011745	SRC_CHICK Full	5291 Imat	2OIQ	1T46	3EL8	39		Sc=6.58512, min distance = 2.231391
KIT_HUMAN Fu	25021197	CDK2_HUMAN Fu	6022 Aden	1GY3	1PKG	3EOC	31.44		Sc=6.3843, min distance = 2.3132111
KIT_HUMAN Fu	3543 1g5s	CDK2_HUMAN Fu	6022 Aden	1GY3	1PKG	1G5S	31.44		Sc=6.50877, min distance = 2.392451
KIT_HUMAN Fu	4327 CID4	ABL1_MOUSE Fu	5291 Imat	1OPJ	1T46	1FPU	40	0.93	Sc=6.51586, min distance = 2.076531
KIT_HUMAN Fu	4369136 C	CDK2_HUMAN Fu	6022 Aden	1GY3	1PKG	1DM2	31.44		Sc=6.28397, min distance = 2.052585
KIT_HUMAN Fu	444345 1c	CDK2_HUMAN Fu	6022 Aden	1GY3	1PKG	1AQ1	31.44		Sc=6.40847, min distance = 1.947182
KIT_HUMAN Fu	444640 CI	BIOD_ECOLI Fu	6022 Aden	1DAD	1PKG	1DAG			Sc=6.12953, min distance = 2.073581
KIT_HUMAN Fu	447077 PD	ABL1_MOUSE Fu	5291 Imat	1OPJ	1T46	1M52	40		Sc=6.32349, min distance = 2.343719
KIT_HUMAN Fu	447700 NS	ABL1_HUMAN Fu	5291 Imat	2HYY	1T46	2G2H	38.78		Sc=6.49635, min distance = 2.472315
KIT_HUMAN Fu	447700 NS	ABL1_MOUSE Fu	5291 Imat	1OPJ	1T46	1OPK	40		Sc=6.41689, min distance = 2.592111
KIT_HUMAN Fu	448014 1c	GSK3B_HUMAN Fu	6022 Aden	1J1C	1PKG	1Q5K	33.59		Sc=6.27599, min distance = 1.680068
KIT_HUMAN Fu	448171 CI	LCK_HUMAN Full	5291 Imat	2PL0	1T46	1QPE	40.53		Sc=6.14514, min distance = 2.037485
KIT_HUMAN Fu	4564 1e1v	CDK2_HUMAN Fu	6022 Aden	1GY3	1PKG	1E1V	31.44		Sc=6.11604, min distance = 1.894160
KIT_HUMAN Fu	5287844 i	GSK3B_HUMAN Fu	6022 Aden	1J1C	1PKG	1UV5	33.59		Sc=6.35505, min distance = 2.161235
KIT_HUMAN Fu	5288018 C	CDK2_HUMAN Fu	6022 Aden	1GY3	1PKG	2B52	31.44		Sc=6.60491, min distance = 1.980671
KIT_HUMAN Fu	5289411 1	CDK2_HUMAN Fu	6022 Aden	1GY3	1PKG	1OGU	31.44		Sc=6.55655, min distance = 1.519011
KIT_HUMAN Fu	5311104 2	ABL1_HUMAN Fu	5291 Imat	2HYY	1T46	2HZI	38.78	0.76	Sc=6.31574, min distance = 2.492876
KIT_HUMAN Fu	5326739 i	GSK3B_HUMAN Fu	6022 Aden	1J1C	1PKG	1Q41	33.59		Sc=6.33978, min distance = 1.548579
KIT_HUMAN Fu	5327148 C	IPKA_RABIT Fu	6022 Aden	1JBP	1PKG	2ERZ			Sc=6.1937, min distance = 2.2288304
KIT_HUMAN Fu	5331010 c	CDK2_HUMAN Fu	6022 Aden	1GY3	1PKG	2BKZ	31.44		Sc=6.345, min distance = 1.7103131
KIT_HUMAN Fu	5957 Aden	RK_BOVIN Full	6022 Aden	3C4Z	1PKG	3C4W		0.99	Sc=6.49995, min distance = 1.913826
KIT_HUMAN Fu	6083 aden	HSP71_HUMAN Fu	6022 Aden	1S3X	1PKG	1XQS		0.99	Sc=6.3548, min distance = 2.2810745
KIT_HUMAN Fu	6083 aden	PURP_METJA Fu	6022 Aden	2R7N	1PKG	2R7M		0.99	Sc=6.34951, min distance = 2.371309

KIT_HUMAN Fu	60961 ade	SKY1_YEAST Fu	6022 Aden	1Q8Y	1PKG	1Q97		0.95	Sc=6.22156, min distance = 1.80708
KIT_HUMAN Fu	6420138 2	CDK2_HUMAN Fu	6022 Aden	1GY3	1PKG	2UUE	31.44		Sc=6.3527, min distance = 2.085213
KIT_HUMAN Fu	6420139 C	CDK2_HUMAN Fu	6022 Aden	1GY3	1PKG	2C5V	31.44		Sc=6.31036, min distance = 1.76312
KIT_HUMAN Fu	644241 Ni	ABL1_HUMAN Fu	5291 Imat	2HYY	1T46	3CS9	38.78	0.9	Sc=6.66364, min distance = 2.58275
KIT_HUMAN Fu	6539118 g	CDK2_HUMAN Fu	6022 Aden	1GY3	1PKG	1FVV	31.44		Sc=6.49689, min distance = 1.44562
KIT_HUMAN Fu	657072 RY	CDK2_HUMAN Fu	6022 Aden	1GY3	1PKG	2BHH	31.44		Sc=6.50972, min distance = 1.92888
KIT_HUMAN Fu	6852187 2	O57883_PYRHO	6022 Aden	1WNL	1PKG	2DTH		0.89	Sc=6.15309, min distance = 1.82779
KIT_HUMAN Fu	72194 2-C	ENPL_CANFA Fu	6022 Aden	1TC6	1PKG	1QYE		0.89	Sc=6.18549, min distance = 1.97870
KIT_HUMAN Fu	9543438 C	DDLB_ECOLI Fu	6022 Aden	1IOW	1PKG	1IOV			Sc=5.97132, min distance = 2.05431
KIT_HUMAN Fu	9547982 7	ABL1_HUMAN Fu	5291 Imat	2HYY	1T46	2HIW	38.78		Sc=6.59355, min distance = 2.58962
KIT_HUMAN Fu	9549303 2	STK6_HUMAN Fu	6022 Aden	1MQ4	1PKG	2NP8	28.48		Sc=6.51582, min distance = 1.66029
KKA3_ENTFA F	10224714	CDK2_HUMAN Fu	6022 Aden	1GY3	2BKK	3DDP			Sc=6.5061, min distance = 2.079622
KKA3_ENTFA F	11957393	FAK1_HUMAN Fu	6022 Aden	1MP8	2BKK	2ETM			Sc=6.23234, min distance = 2.47650
KKA3_ENTFA F	11957417	CDK2_HUMAN Fu	6022 Aden	1GY3	2BKK	2I40			Sc=6.32167, min distance = 1.83723
KKA3_ENTFA F	1540 1h1c	CDK2_HUMAN Fu	6022 Aden	1GY3	2BKK	1H1Q			Sc=6.39853, min distance = 1.70113
KKA3_ENTFA F	16046126	CDK2_HUMAN Fu	6022 Aden	1GY3	2BKK	2DS1			Sc=6.37249, min distance = 1.89685
KKA3_ENTFA F	16214823	CDK2_HUMAN Fu	6022 Aden	1GY3	2BKK	2UZB			Sc=6.13563, min distance = 1.61700
KKA3_ENTFA F	16214825	CDK2_HUMAN Fu	6022 Aden	1GY3	2BKK	2UZE			Sc=6.31741, min distance = 2.38283
KKA3_ENTFA F	16214826	CDK2_HUMAN Fu	6022 Aden	1GY3	2BKK	2UZL			Sc=6.165, min distance = 1.9789365
KKA3_ENTFA F	16758212	GSK3B_HUMAN F	33113 gam	1J1B	1J7U	2O5K			Sc=6.47447, min distance = 1.98474
KKA3_ENTFA F	16758212	GSK3B_HUMAN F	6022 Aden	1J1C	2BKK	2O5K			Sc=6.45382, min distance = 2.23692
KKA3_ENTFA F	1707 1fvt	CDK2_HUMAN Fu	6022 Aden	1GY3	2BKK	1FVT			Sc=6.21251, min distance = 0.93992
KKA3_ENTFA F	23653515	CDK2_HUMAN Fu	6022 Aden	1GY3	2BKK	2R3F			Sc=5.98023, min distance = 2.37602
KKA3_ENTFA F	23653516	CDK2_HUMAN Fu	6022 Aden	1GY3	2BKK	2R3G			Sc=6.14433, min distance = 2.15295
KKA3_ENTFA F	23653518	CDK2_HUMAN Fu	6022 Aden	1GY3	2BKK	2R3J			Sc=6.14751, min distance = 2.27231
KKA3_ENTFA F	23653519	CDK2_HUMAN Fu	6022 Aden	1GY3	2BKK	2R3K			Sc=6.24359, min distance = 2.28590
KKA3_ENTFA F	23653522	CDK2_HUMAN Fu	6022 Aden	1GY3	2BKK	2R3N			Sc=6.23068, min distance = 2.12440
KKA3_ENTFA F	23653526	CDK2_HUMAN Fu	6022 Aden	1GY3	2BKK	2R3R			Sc=6.15327, min distance = 1.89719
KKA3_ENTFA F	24779674	CSK2A_MAIZE F	33113 gam	1LP4	1J7U	2PVH			Sc=6.15711, min distance = 2.02080
KKA3_ENTFA F	24779677	CSK2A_MAIZE F	33113 gam	1LP4	1J7U	2PVL			Sc=6.21535, min distance = 1.67858
KKA3_ENTFA F	24864077	CDK2_HUMAN Fu	6022 Aden	1GY3	2BKK	2VTN			Sc=6.21991, min distance = 2.70291
KKA3_ENTFA F	24864080	CDK2_HUMAN Fu	6022 Aden	1GY3	2BKK	2VTR			Sc=5.92944, min distance = 2.57256
KKA3_ENTFA F	24963033	CDK2_HUMAN Fu	6022 Aden	1GY3	2BKK	2W05			Sc=6.32987, min distance = 2.15152
KKA3_ENTFA F	2608 1jsv	CDK2_HUMAN Fu	6022 Aden	1GY3	2BKK	1JSV			Sc=5.82363, min distance = 1.60635
KKA3_ENTFA F	3064778 H	ROCK1_HUMAN F	33113 gam	2V55	1J7U	2ETK			Sc=6.10296, min distance = 1.96341
KKA3_ENTFA F	36735 Gpp	CSK2A_MAIZE F	33113 gam	1LP4	1J7U	1DAY		0.8	Sc=6.00943, min distance = 2.19404
KKA3_ENTFA F	3973 nche	PIM1_HUMAN Fu	33113 gam	1XR1	1J7U	1YI3			Sc=5.92194, min distance = 2.11825

KKA3_ENTFA Fu	398148 1e	CDK2_HUMAN Fu	6022 Aden	1GY3	2BKK	1E1X			Sc=6.15601, min distance = 2.02687
KKA3_ENTFA Fu	4369136 C	CDK2_HUMAN Fu	6022 Aden	1GY3	2BKK	1DM2			Sc=6.29523, min distance = 2.04389
KKA3_ENTFA Fu	444345 1c	CDK2_HUMAN Fu	6022 Aden	1GY3	2BKK	1AQ1			Sc=6.35169, min distance = 1.76928
KKA3_ENTFA Fu	444564 AD	BIOD_ECOLI Fu	6022 Aden	1DAD	2BKK	1BS1		0.91	Sc=6.38203, min distance = 2.27669
KKA3_ENTFA Fu	444564 AD	MYS2_DICDI Fu	6022 Aden	1VOM	2BKK	1W9I		0.91	Sc=6.40124, min distance = 2.26182
KKA3_ENTFA Fu	444852 CI	ATPB_BOVIN Fu	6022 Aden	2CK3	2BKK	1COW		0.9	Sc=6.45126, min distance = 1.85464
KKA3_ENTFA Fu	445840 di	CDK2_HUMAN Fu	6022 Aden	1GY3	2BKK	1GII			Sc=6.24576, min distance = 2.22421
KKA3_ENTFA Fu	447649 1c	CDK2_HUMAN Fu	6022 Aden	1GY3	2BKK	1OI9			Sc=6.40599, min distance = 2.61625
KKA3_ENTFA Fu	447652 Im	CDK2_HUMAN Fu	6022 Aden	1GY3	2BKK	1OIQ			Sc=6.16079, min distance = 2.30693
KKA3_ENTFA Fu	447654 CI	CDK2_HUMAN Fu	6022 Aden	1GY3	2BKK	1OIT			Sc=6.25949, min distance = 1.47011
KKA3_ENTFA Fu	447655 1c	CDK2_HUMAN Fu	6022 Aden	1GY3	2BKK	1OIU			Sc=6.28355, min distance = 2.03637
KKA3_ENTFA Fu	447766 1p	CDK2_HUMAN Fu	6022 Aden	1GY3	2BKK	1P2A			Sc=6.32962, min distance = 1.93615
KKA3_ENTFA Fu	447962 1p	CDK2_HUMAN Fu	6022 Aden	1GY3	2BKK	2C5N			Sc=6.25637, min distance = 2.67170
KKA3_ENTFA Fu	448005 CI	GSK3B_HUMAN Fu	6022 Aden	1J1C	2BKK	1Q3W			Sc=6.30298, min distance = 2.27151
KKA3_ENTFA Fu	448222 NE	ENPL_CANFA Fu	6022 Aden	1TC6	2BKK	1QY5		0.81	Sc=6.33288, min distance = 2.07990
KKA3_ENTFA Fu	448238 CI	GSK3B_HUMAN Fu	33113 gam	1J1B	1J7U	1R0E			Sc=6.52875, min distance = 2.09820
KKA3_ENTFA Fu	448238 CI	GSK3B_HUMAN Fu	6022 Aden	1J1C	2BKK	1R0E			Sc=6.5098, min distance = 1.885577
KKA3_ENTFA Fu	448293 CI	CDK2_HUMAN Fu	6022 Aden	1GY3	2BKK	1R78			Sc=6.46563, min distance = 2.15624
KKA3_ENTFA Fu	4564 1e1v	CDK2_HUMAN Fu	6022 Aden	1GY3	2BKK	1E1V			Sc=6.10007, min distance = 2.54433
KKA3_ENTFA Fu	4565 1h1r	CDK2_HUMAN Fu	6022 Aden	1GY3	2BKK	1H1R			Sc=6.27533, min distance = 2.42035
KKA3_ENTFA Fu	5288712 1	CDK2_HUMAN Fu	6022 Aden	1GY3	2BKK	1KE9			Sc=6.23413, min distance = 1.98917
KKA3_ENTFA Fu	5326739 i	GSK3B_HUMAN Fu	33113 gam	1J1B	1J7U	1Q41			Sc=6.30298, min distance = 2.54257
KKA3_ENTFA Fu	5326739 i	GSK3B_HUMAN Fu	6022 Aden	1J1C	2BKK	1Q41			Sc=6.23849, min distance = 2.41012
KKA3_ENTFA Fu	5326977 1	CSK2A_MAIZE Fu	33113 gam	1LP4	1J7U	1ZOG			Sc=5.68246, min distance = 2.42420
KKA3_ENTFA Fu	5326978 1	CSK2A_MAIZE Fu	33113 gam	1LP4	1J7U	1ZOH			Sc=5.66617, min distance = 2.54284
KKA3_ENTFA Fu	5327132 C	CDK2_HUMAN Fu	6022 Aden	1GY3	2BKK	2C6I			Sc=6.15791, min distance = 2.31530
KKA3_ENTFA Fu	5327133 C	CDK2_HUMAN Fu	6022 Aden	1GY3	2BKK	2C6K			Sc=6.23861, min distance = 0.87165
KKA3_ENTFA Fu	5327135 D	CDK2_HUMAN Fu	6022 Aden	1GY3	2BKK	2C6M			Sc=6.16023, min distance = 1.31348
KKA3_ENTFA Fu	5331010 c	CDK2_HUMAN Fu	6022 Aden	1GY3	2BKK	2BKZ			Sc=6.23398, min distance = 1.62935
KKA3_ENTFA Fu	5494449 T	STK6_HUMAN Fu	33113 gam	2DWB	1J7U	3E5A			Sc=6.27238, min distance = 2.10836
KKA3_ENTFA Fu	5687 1di8	CDK2_HUMAN Fu	6022 Aden	1GY3	2BKK	1DI8			Sc=6.17332, min distance = 1.96252
KKA3_ENTFA Fu	5957 Aden	MUTS_ECOLI Fu	6022 Aden	1OH7	2BKK	1W7A		0.99	Sc=6.00815, min distance = 0.89629
KKA3_ENTFA Fu	5957 Aden	Q72H90_THET2 I	6022 Aden	2BEJ	2BKK	2BEK		0.99	Sc=6.02426, min distance = 1.96837
KKA3_ENTFA Fu	5957 Aden	RK_BOVIN Full=	6022 Aden	3C4Z	2BKK	3C4W		0.99	Sc=5.99201, min distance = 2.19107
KKA3_ENTFA Fu	5957 Aden	SECA_ECOLI Fu	6022 Aden	2FSI	2BKK	2FSG		0.99	Sc=6.461, min distance = 1.7557909
KKA3_ENTFA Fu	60961 ade	IPKA_RABIT Fu	6022 Aden	1JBP	2BKK	1FMO		0.95	Sc=6.09568, min distance = 2.11503
KKA3_ENTFA Fu	6132 Cyti	O33839_THEMA I	6022 Aden	1XJK	2BKK	1XJN			Sc=6.24971, min distance = 1.92112

KKA3_ENTFA F	6338563 C	MYS2_DICDI Fu	6022 Aden	1VOM	2BKK	1D0Z			Sc=6.12974, min distance = 2.32638
KKA3_ENTFA F	6420138 2	CDK2_HUMAN Fu	6022 Aden	1GY3	2BKK	2UUE			Sc=6.29556, min distance = 1.83826
KKA3_ENTFA F	6420139 C	CDK2_HUMAN Fu	6022 Aden	1GY3	2BKK	2C5V			Sc=6.15059, min distance = 1.35128
KKA3_ENTFA F	657072 RY	CDK2_HUMAN Fu	6022 Aden	1GY3	2BKK	2BHH			Sc=6.46012, min distance = 2.36230
KKA3_ENTFA F	6918710 5	CDK2_HUMAN Fu	6022 Aden	1GY3	2BKK	3EJ1			Sc=6.23709, min distance = 2.36742
KKA3_ENTFA F	8582 Inos	RNAS1_BOVIN F	6022 Aden	1O0H	2BKK	1Z6D		0.79	Sc=6.32378, min distance = 1.65642
KKA3_ENTFA F	8977 1dar	NDK_PYRHO Ful	6022 Aden	2DYA	2BKK	2DXE		0.8	Sc=5.96924, min distance = 1.94128
KKA3_ENTFA F	91532 AMP	MTNK_BACSU Fu	6022 Aden	2OLC	2BKK	2PUL		0.99	Sc=6.47719, min distance = 2.29488
KKA3_ENTFA F	91532 AMP	PURP_METJA Fu	6022 Aden	2R7N	2BKK	2R7K		0.99	Sc=6.48055, min distance = 2.04559
KKA3_ENTFA F	9547890 I	CDK2_HUMAN Fu	6022 Aden	1GY3	2BKK	1W8C			Sc=6.09597, min distance = 2.56170
KKA3_ENTFA F	9817550 V	CDK2_HUMAN Fu	6022 Aden	1GY3	2BKK	3BHV			Sc=6.35758, min distance = 2.43695
KKA3_ENTFA F	9934347 E	FAK1_CHICK Fu	33113 gam	2J0L	1J7U	2JKK			Sc=6.49574, min distance = 1.39287
KLK3_HUMAN F	439353 be	IGHG1_HUMAN F	444205 CI	1HZH	2ZCL	1L6X			Sc=5.68635, min distance = 2.19088
KPCI_HUMAN F	10109823	PIM1_HUMAN Fu	2396 bisi	1XWS	1ZRZ	3CY3			Sc=6.27172, min distance = 2.44931
KPCI_HUMAN F	2398 bisi	PDPK1_HUMAN F	2396 bisi	1UU8	1ZRZ	1UU9		0.99	Sc=6.36696, min distance = 2.71460
KPCI_HUMAN F	24180721	PIM1_HUMAN Fu	2396 bisi	1XWS	1ZRZ	3C4E			Sc=5.98708, min distance = 1.88085
KPCI_HUMAN F	5327121 I	PIM1_HUMAN Fu	2396 bisi	1XWS	1ZRZ	2C3I			Sc=6.17261, min distance = 2.07265
KPCI_HUMAN F	60961 ade	PIM1_HUMAN Fu	2396 bisi	1XWS	1ZRZ	1YI4			Sc=6.17235, min distance = 2.18681
KPCI_HUMAN F	611002 Op	PIM1_HUMAN Fu	2396 bisi	1XWS	1ZRZ	1YXX			Sc=6.11441, min distance = 2.07622
KPCT_HUMAN F	10109823	PIM1_HUMAN Fu	444345 1c	1YHS	1XJD	3CY3	36.94		Sc=6.36805, min distance = 2.79556
KPCT_HUMAN F	10224714	CDK2_HUMAN Fu	444345 1c	1AQ1	1XJD	3DDP	30.4		Sc=6.35867, min distance = 2.15640
KPCT_HUMAN F	11314340	KAPCA_BOVIN F	444345 1c	1STC	1XJD	2UZT	38.91		Sc=6.34567, min distance = 2.42901
KPCT_HUMAN F	11348631	CHK1_HUMAN Fu	444345 1c	1NVR	1XJD	2E9U	28.89		Sc=6.21179, min distance = 2.73761
KPCT_HUMAN F	11553058	LCK_HUMAN Ful	444345 1c	1QPD	1XJD	2OF2	27.83		Sc=6.17086, min distance = 2.43418
KPCT_HUMAN F	11957417	CDK2_HUMAN Fu	444345 1c	1AQ1	1XJD	2I40	30.4		Sc=6.29759, min distance = 2.31609
KPCT_HUMAN F	11986113	PIM1_HUMAN Fu	444345 1c	1YHS	1XJD	2BZH	36.94	0.76	Sc=6.4275, min distance = 2.277287
KPCT_HUMAN F	11986115	PIM1_HUMAN Fu	444345 1c	1YHS	1XJD	2BZJ	36.94		Sc=6.60352, min distance = 2.03465
KPCT_HUMAN F	1540 1h1q	CDK2_HUMAN Fu	444345 1c	1AQ1	1XJD	1H1Q	30.4		Sc=6.25365, min distance = 2.81455
KPCT_HUMAN F	15942652	CDK2_HUMAN Fu	444345 1c	1AQ1	1XJD	2DUV	30.4		Sc=6.30424, min distance = 2.26184
KPCT_HUMAN F	15991572	LCK_HUMAN Ful	444345 1c	1QPD	1XJD	2OF4	27.83		Sc=6.15244, min distance = 2.88582
KPCT_HUMAN F	160355 ro	CDK2_HUMAN Fu	444345 1c	1AQ1	1XJD	3DDQ	30.4		Sc=6.1626, min distance = 2.462893
KPCT_HUMAN F	16058647	PIM1_HUMAN Fu	444345 1c	1YHS	1XJD	2OI4	36.94	0.75	Sc=6.45307, min distance = 2.20131
KPCT_HUMAN F	16113377	CDK2_HUMAN Fu	444345 1c	1AQ1	1XJD	2W17	30.4		Sc=6.39678, min distance = 2.12831
KPCT_HUMAN F	16122608	PDPK1_HUMAN F	444345 1c	1OKY	1XJD	2PE1	37.87		Sc=6.31952, min distance = 2.70641
KPCT_HUMAN F	16122643	CHK1_HUMAN Fu	444345 1c	1NVR	1XJD	2YWP	28.89		Sc=6.25313, min distance = 2.69966
KPCT_HUMAN F	16214825	CDK2_HUMAN Fu	444345 1c	1AQ1	1XJD	2UZE	30.4		Sc=6.25806, min distance = 2.47619
KPCT_HUMAN F	16214826	CDK2_HUMAN Fu	444345 1c	1AQ1	1XJD	2UZL	30.4		Sc=6.12458, min distance = 2.08058

KPCT_HUMAN F	1707 1fvt	CDK2_HUMAN Fu	444345 1c 1AQ1	1XJD	1FVT	30.4	Sc=6.0986, min distance = 2.035205
KPCT_HUMAN F	17754396	PDPK1_HUMAN F	444345 1c 1OKY	1XJD	2R7B	37.87	Sc=6.18059, min distance = 2.58942
KPCT_HUMAN F	1907917 2	CHK1_HUMAN Fu	444345 1c 1NVR	1XJD	2CGW	28.89	Sc=6.12501, min distance = 2.87907
KPCT_HUMAN F	23536770	LCK_HUMAN Ful	444345 1c 1QPD	1XJD	3BYM	27.83	Sc=6.43675, min distance = 2.17516
KPCT_HUMAN F	23653515	CDK2_HUMAN Fu	444345 1c 1AQ1	1XJD	2R3F	30.4	Sc=5.87941, min distance = 2.66268
KPCT_HUMAN F	23653518	CDK2_HUMAN Fu	444345 1c 1AQ1	1XJD	2R3J	30.4	Sc=6.08616, min distance = 2.67748
KPCT_HUMAN F	23653519	CDK2_HUMAN Fu	444345 1c 1AQ1	1XJD	2R3K	30.4	Sc=6.08476, min distance = 2.51682
KPCT_HUMAN F	23653520	CDK2_HUMAN Fu	444345 1c 1AQ1	1XJD	2R3L	30.4	Sc=6.07676, min distance = 2.63901
KPCT_HUMAN F	23653521	CDK2_HUMAN Fu	444345 1c 1AQ1	1XJD	2R3M	30.4	Sc=6.17541, min distance = 2.49150
KPCT_HUMAN F	23653524	CDK2_HUMAN Fu	444345 1c 1AQ1	1XJD	2R3P	30.4	Sc=6.1865, min distance = 2.021111
KPCT_HUMAN F	23653526	CDK2_HUMAN Fu	444345 1c 1AQ1	1XJD	2R3R	30.4	Sc=5.94973, min distance = 2.87623
KPCT_HUMAN F	23657800	CHK1_HUMAN Fu	444345 1c 1NVR	1XJD	2E9P	28.89	Sc=6.24785, min distance = 2.53606
KPCT_HUMAN F	23727982	CDK2_HUMAN Fu	444345 1c 1AQ1	1XJD	3BHU	30.4	Sc=6.24206, min distance = 2.51558
KPCT_HUMAN F	2403 1uv	PDPK1_HUMAN F	444345 1c 1OKY	1XJD	1UVR	37.87	Sc=6.37931, min distance = 2.09566
KPCT_HUMAN F	24752838	CHK1_HUMAN Fu	444345 1c 1NVR	1XJD	2QHM	28.89	Sc=6.04835, min distance = 2.49392
KPCT_HUMAN F	24820122	PIM1_HUMAN Fu	444345 1c 1YHS	1XJD	3BWF	36.94	Sc=6.51741, min distance = 2.11972
KPCT_HUMAN F	24864081	CDK2_HUMAN Fu	444345 1c 1AQ1	1XJD	2VTS	30.4	Sc=6.27338, min distance = 2.32563
KPCT_HUMAN F	24905143	PK3CG_HUMAN F	444345 1c 1E8Z	1XJD	2V4L		Sc=5.99973, min distance = 2.85487
KPCT_HUMAN F	24905144	PK3CG_HUMAN F	444345 1c 1E8Z	1XJD	3ENE		Sc=6.08873, min distance = 2.56226
KPCT_HUMAN F	24916751	CDK2_HUMAN Fu	444345 1c 1AQ1	1XJD	3DOG	30.4	Sc=6.15482, min distance = 2.38943
KPCT_HUMAN F	33113 gam	IRAK4_HUMAN F	444345 1c 2NRY	1XJD	2OID	37.62	Sc=6.45294, min distance = 2.21159
KPCT_HUMAN F	3543 1g5s	CDK2_HUMAN Fu	444345 1c 1AQ1	1XJD	1G5S	30.4	Sc=6.33338, min distance = 2.58871
KPCT_HUMAN F	3547 Fasu	KAPCA_BOVIN F	444345 1c 1STC	1XJD	1Q8W	38.91	Sc=5.92897, min distance = 2.59407
KPCT_HUMAN F	3973 nche	PIM1_HUMAN Fu	444345 1c 1YHS	1XJD	1YI3	36.94	Sc=5.88242, min distance = 2.36862
KPCT_HUMAN F	4369136 C	CDK2_HUMAN Fu	444345 1c 1AQ1	1XJD	1DM2	30.4	Sc=6.17871, min distance = 2.93235
KPCT_HUMAN F	446704 LS	CDK2_HUMAN Fu	444345 1c 1AQ1	1XJD	1KE6	30.4	Sc=6.26963, min distance = 2.15675
KPCT_HUMAN F	447649 1c	CDK2_HUMAN Fu	444345 1c 1AQ1	1XJD	1OI9	30.4	Sc=6.34252, min distance = 2.71826
KPCT_HUMAN F	447652 Im	CDK2_HUMAN Fu	444345 1c 1AQ1	1XJD	1OIQ	30.4	Sc=6.08853, min distance = 2.57386
KPCT_HUMAN F	447766 1p	CDK2_HUMAN Fu	444345 1c 1AQ1	1XJD	1P2A	30.4	Sc=6.27806, min distance = 2.25689
KPCT_HUMAN F	447962 1p	CDK2_HUMAN Fu	444345 1c 1AQ1	1XJD	2C5N	30.4	Sc=6.09998, min distance = 2.36811
KPCT_HUMAN F	447967 CI	CDK2_HUMAN Fu	444345 1c 1AQ1	1XJD	1PYE	30.4	Sc=6.35312, min distance = 2.50559
KPCT_HUMAN F	448043 2c	KAPCA_BOVIN F	444345 1c 1STC	1XJD	1Q8U	38.91	Sc=5.98475, min distance = 2.42429
KPCT_HUMAN F	448293 CI	CDK2_HUMAN Fu	444345 1c 1AQ1	1XJD	1R78	30.4	Sc=6.43154, min distance = 2.53076
KPCT_HUMAN F	449240 1y	KAPCA_BOVIN F	444345 1c 1STC	1XJD	1YDR	38.91	Sc=5.92108, min distance = 2.47502
KPCT_HUMAN F	456214 pu	KS6A1_HUMAN F	444345 1c 2Z7R	1XJD	2Z7S		Sc=6.27109, min distance = 2.16900
KPCT_HUMAN F	4565 1h1r	CDK2_HUMAN Fu	444345 1c 1AQ1	1XJD	1H1R	30.4	Sc=6.25618, min distance = 2.63014
KPCT_HUMAN F	4566 1h1s	CDK2_HUMAN Fu	444345 1c 1AQ1	1XJD	1H1S	30.4	Sc=6.30357, min distance = 1.84769

KPCT_HUMAN F	5288708 1	CDK2_HUMAN Fu	444345 1c	1AQ1	1XJD	1KE5	30.4		Sc=6.11374, min distance = 2.19051
KPCT_HUMAN F	5288710 C	CDK2_HUMAN Fu	444345 1c	1AQ1	1XJD	1KE7	30.4		Sc=6.34098, min distance = 2.00570
KPCT_HUMAN F	5327121 I	PIM1_HUMAN Fu	444345 1c	1YHS	1XJD	2C3I	36.94		Sc=6.12206, min distance = 2.71521
KPCT_HUMAN F	5327123 I	CHK1_HUMAN Fu	444345 1c	1NVR	1XJD	2C3L	28.89		Sc=6.12054, min distance = 2.42896
KPCT_HUMAN F	5327130 C	CDK2_HUMAN Fu	444345 1c	1AQ1	1XJD	2C68	30.4		Sc=6.09558, min distance = 2.51581
KPCT_HUMAN F	5327131 T	CDK2_HUMAN Fu	444345 1c	1AQ1	1XJD	2C69	30.4		Sc=6.17469, min distance = 2.68550
KPCT_HUMAN F	5327133 C	CDK2_HUMAN Fu	444345 1c	1AQ1	1XJD	2C6K	30.4		Sc=6.14179, min distance = 2.37692
KPCT_HUMAN F	5327134 C	CDK2_HUMAN Fu	444345 1c	1AQ1	1XJD	2C6L	30.4		Sc=6.28086, min distance = 2.30448
KPCT_HUMAN F	5687 1di8	CDK2_HUMAN Fu	444345 1c	1AQ1	1XJD	1DI8	30.4		Sc=6.13165, min distance = 2.56325
KPCT_HUMAN F	60961 ade	PIM1_HUMAN Fu	444345 1c	1YHS	1XJD	1YI4	36.94		Sc=6.14213, min distance = 2.63585
KPCT_HUMAN F	6102670	CDK2_HUMAN Fu	444345 1c	1AQ1	1XJD	1YKR	30.4		Sc=6.27227, min distance = 2.39461
KPCT_HUMAN F	6420138 2	CDK2_HUMAN Fu	444345 1c	1AQ1	1XJD	2UUE	30.4		Sc=6.25253, min distance = 2.09600
KPCT_HUMAN F	6420139 C	CDK2_HUMAN Fu	444345 1c	1AQ1	1XJD	2C5V	30.4		Sc=6.10146, min distance = 2.17297
KPCT_HUMAN F	6540273 2	CHK1_HUMAN Fu	444345 1c	1NVR	1XJD	2CGV	28.89		Sc=6.1556, min distance = 2.584151
KPCT_HUMAN F	656971 CI	CDK2_HUMAN Fu	444345 1c	1AQ1	1XJD	1Y8Y	30.4		Sc=5.9087, min distance = 2.529309
KPCT_HUMAN F	657072 RY	CDK2_HUMAN Fu	444345 1c	1AQ1	1XJD	2BHH	30.4		Sc=6.44315, min distance = 2.56158
KPCT_HUMAN F	657138 1z	PDPK1_HUMAN F	444345 1c	1OKY	1XJD	1Z5M	37.87		Sc=6.65331, min distance = 2.29803
KPCT_HUMAN F	6852207 M	KAPCA_BOVIN F	444345 1c	1STC	1XJD	2GNI	38.91		Sc=5.91836, min distance = 2.23768
KPCT_HUMAN F	6918852 2	CDK2_HUMAN Fu	444345 1c	1AQ1	1XJD	2FVD	30.4		Sc=6.3346, min distance = 2.483312
KPCT_HUMAN F	72271 7-H	CDK2_HUMAN Fu	444345 1c	1AQ1	1XJD	1PKD	30.4	0.91	Sc=6.46334, min distance = 2.46454
KPCT_HUMAN F	72271 7-H	CHK1_HUMAN Fu	444345 1c	1NVR	1XJD	1NVQ	28.89	0.91	Sc=6.44909, min distance = 2.69780
KPCT_HUMAN F	72271 7-H	PDPK1_HUMAN F	444345 1c	1OKY	1XJD	1OKZ	37.87	0.91	Sc=6.44573, min distance = 2.63251
KPCT_HUMAN F	848641 2b	CHK1_HUMAN Fu	444345 1c	1NVR	1XJD	2BRH	28.89		Sc=6.16329, min distance = 2.00999
KPCT_HUMAN F	9817550 V	CDK2_HUMAN Fu	444345 1c	1AQ1	1XJD	3BHV	30.4		Sc=6.35261, min distance = 2.17126
KPRS_BACSU F	60961 ade	USHA_ECOLI Fu	92199 AMP	1HPU	1DKU	1HO5		0.93	Sc=6.09716, min distance = 1.48881
KPYM_RABIT F	11608401	KAPCA_BOVIN F	5957 Aden	1Q24	1A49	2UVY			Sc=6.2023, min distance = 2.129239
KPYM_RABIT F	16122607	PDPK1_HUMAN F	5957 Aden	2BIY	1A49	2PE0			Sc=6.17099, min distance = 1.99540
KPYM_RABIT F	3973 nche	PK3CG_PIG Ful	5957 Aden	1E8X	1A49	1E7V			Sc=5.93443, min distance = 1.91582
KPYM_RABIT F	440317 AT	PURT_ECOLI Fu	5957 Aden	1KJ8	1A49	1KJJ		0.99	Sc=6.43691, min distance = 1.37122
KPYM_RABIT F	6022 Aden	DDL_THET8 Ful	5957 Aden	2ZDQ	1A49	2ZDH		0.99	Sc=6.39627, min distance = 1.84226
KPYM_RABIT F	6022 Aden	Y059_METJA Fu	5957 Aden	2J9C	1A49	2J9D		0.99	Sc=5.9388, min distance = 1.903851
KPYM_RABIT F	6083 aden	PURP_METJA Fu	5957 Aden	2R7L	1A49	2R7M		0.98	Sc=6.32157, min distance = 2.12640
KPYM_RABIT F	6176 Cyti	CCA_ARCFU Ful	5957 Aden	1R8B	1A49	1R89			Sc=6.36577, min distance = 1.53723
KPYM_RABIT F	91532 AMP	PURT_ECOLI Fu	5957 Aden	1KJ8	1A49	1KJI		0.99	Sc=6.47483, min distance = 1.75998
KRE2_YEAST F	37792 gam	GNAI1_RAT Ful	8977 1dar	1SVK	1S4O	1AS0		0.99	Sc=5.75524, min distance = 2.28996
KRE2_YEAST F	37792 gam	GNAT1_BOVIN F	8977 1dar	1TAD	1S4O	1TND		0.99	Sc=5.76386, min distance = 2.24406
KRE2_YEAST F	37792 gam	RAB6B_HUMAN F	8977 1dar	2E9S	1S4O	2FFQ		0.99	Sc=5.76046, min distance = 2.10655

KRE2_YEAST F	37792 gam	RB11A_HUMAN F	8977 1dar	1OIX	1S4O	1OIW	0.99	Sc=5.75524, min distance = 2.01542
KRE2_YEAST F	445939 1g	PPTA_ECOLI Ful	23831 HE	1GYX	1S4O	1GYY		Sc=5.78465, min distance = 2.01537
KRE2_YEAST F	446248 CI	RASH_HUMAN Ful	8977 1dar	2CE2	1S4O	1IAQ		Sc=5.62365, min distance = 1.99510
KRE2_YEAST F	447979 5G	KGUA_ECOLI Ful	8977 1dar	2AN9	1S4O	2ANB		Sc=5.97365, min distance = 2.38355
KRE2_YEAST F	5326975 5	KGUA_MYCTU Ful	8977 1dar	1ZNY	1S4O	1ZNX	0.99	Sc=6.41524, min distance = 2.46700
KRE2_YEAST F	5962 L-ly	RBCMT_PEA Ful	23831 HE	1MLV	1S4O	2H2E		Sc=5.67059, min distance = 1.80131
KRE2_YEAST F	6022 Ader	NDK_PYRHO Ful	8977 1dar	2DXE	1S4O	2DYA	0.8	Sc=5.68882, min distance = 2.12967
KRE2_YEAST F	6022 Ader	PARM_ECOLX Ful	8977 1dar	2ZGY	1S4O	1MWM	0.8	Sc=5.99459, min distance = 1.71856
KRE2_YEAST F	6802 guar	DPOL_BPR69 Ful	8977 1dar	1CLQ	1S4O	1IH7	0.94	Sc=6.32827, min distance = 2.04355
KRE2_YEAST F	6804 guar	DPOL_BPR69 Ful	8977 1dar	1CLQ	1S4O	1WAJ	0.99	Sc=5.92436, min distance = 2.22264
KRE2_YEAST F	6804 guar	EF1A_YEAST Ful	8977 1dar	2B7B	1S4O	1G7C	0.99	Sc=5.93241, min distance = 2.24560
KRE2_YEAST F	6804 guar	TGM3_HUMAN Ful	8977 1dar	1VJJ	1S4O	1SGX	0.99	Sc=6.42454, min distance = 2.16391
KRE2_YEAST F	93082 Gab	RAC3_HUMAN Ful	8977 1dar	2C2H	1S4O	2QME	0.99	Sc=5.6201, min distance = 1.973203
KRE2_YEAST F	93082 Gab	RASH_HUMAN Ful	8977 1dar	2CE2	1S4O	121P	0.99	Sc=5.62667, min distance = 2.04275
KS6A1_HUMAN	10224714	CDK2_HUMAN Ful	444345 1c	1AQ1	2Z7R	3DDP		Sc=6.49423, min distance = 2.47763
KS6A1_HUMAN	11957417	CDK2_HUMAN Ful	444345 1c	1AQ1	2Z7R	2I40		Sc=6.28196, min distance = 2.24440
KS6A1_HUMAN	11986115	PIM1_HUMAN Ful	444345 1c	1YHS	2Z7R	2BZJ		Sc=6.27067, min distance = 2.08297
KS6A1_HUMAN	153999 Ru	PDPK1_HUMAN F	444345 1c	1OKY	2Z7R	1UU3	0.77	Sc=6.35226, min distance = 2.19503
KS6A1_HUMAN	15602982	KAPCA_BOVIN F	444345 1c	1STC	2Z7R	2UW6		Sc=6.05048, min distance = 2.35697
KS6A1_HUMAN	15991572	LCK_HUMAN Ful	444345 1c	1QPD	2Z7R	2OF4		Sc=6.34653, min distance = 2.52498
KS6A1_HUMAN	160355 rc	CDK2_HUMAN Ful	444345 1c	1AQ1	2Z7R	3DDQ		Sc=6.24644, min distance = 2.51423
KS6A1_HUMAN	16122608	PDPK1_HUMAN F	444345 1c	1OKY	2Z7R	2PE1		Sc=6.33798, min distance = 2.62101
KS6A1_HUMAN	16122643	CHK1_HUMAN Ful	444345 1c	1NVR	2Z7R	2YWP		Sc=6.26572, min distance = 2.22447
KS6A1_HUMAN	16214823	CDK2_HUMAN Ful	444345 1c	1AQ1	2Z7R	2UZB		Sc=6.05636, min distance = 2.58677
KS6A1_HUMAN	16214826	CDK2_HUMAN Ful	444345 1c	1AQ1	2Z7R	2UZL		Sc=6.1037, min distance = 2.553302
KS6A1_HUMAN	16214827	CDK2_HUMAN Ful	444345 1c	1AQ1	2Z7R	2UZN		Sc=6.04121, min distance = 2.80258
KS6A1_HUMAN	16214828	CDK2_HUMAN Ful	444345 1c	1AQ1	2Z7R	2UZO		Sc=6.05533, min distance = 2.90615
KS6A1_HUMAN	16758227	CHK1_HUMAN Ful	444345 1c	1NVR	2Z7R	2R0U		Sc=6.53496, min distance = 2.41381
KS6A1_HUMAN	1707 1fvt	CDK2_HUMAN Ful	444345 1c	1AQ1	2Z7R	1FVT		Sc=6.07396, min distance = 2.20395
KS6A1_HUMAN	17754396	PDPK1_HUMAN F	444345 1c	1OKY	2Z7R	2R7B		Sc=6.28624, min distance = 2.63558
KS6A1_HUMAN	1907917 2	CHK1_HUMAN Ful	444345 1c	1NVR	2Z7R	2CGW		Sc=6.14999, min distance = 2.74388
KS6A1_HUMAN	23653515	CDK2_HUMAN Ful	444345 1c	1AQ1	2Z7R	2R3F		Sc=5.99006, min distance = 2.55972
KS6A1_HUMAN	23653516	CDK2_HUMAN Ful	444345 1c	1AQ1	2Z7R	2R3G		Sc=6.19484, min distance = 2.78608
KS6A1_HUMAN	23657800	CHK1_HUMAN Ful	444345 1c	1NVR	2Z7R	2E9P		Sc=6.33058, min distance = 2.18248
KS6A1_HUMAN	23727982	CDK2_HUMAN Ful	444345 1c	1AQ1	2Z7R	3BHU		Sc=6.22391, min distance = 2.58730
KS6A1_HUMAN	2398 bisi	PDPK1_HUMAN F	444345 1c	1OKY	2Z7R	1UU9		Sc=6.44318, min distance = 2.01282
KS6A1_HUMAN	24752838	CHK1_HUMAN Ful	444345 1c	1NVR	2Z7R	2QHM		Sc=6.036, min distance = 2.8102587

KS6A1_HUMAN I	24808489	IGF1R_HUMAN Fu	91532 AMP	1K3A	2Z7Q	2ZM3		Sc=6.25807, min distance = 2.54311
KS6A1_HUMAN I	24864081	CDK2_HUMAN Ful	444345 1c	1AQ1	2Z7R	2VTS		Sc=6.28326, min distance = 2.47118
KS6A1_HUMAN I	24905143	PK3CG_HUMAN Fu	444345 1c	1E8Z	2Z7R	2V4L		Sc=5.99096, min distance = 2.38187
KS6A1_HUMAN I	24905144	PK3CG_HUMAN Fu	444345 1c	1E8Z	2Z7R	3ENE		Sc=6.06987, min distance = 2.13830
KS6A1_HUMAN I	24916751	CDK2_HUMAN Ful	444345 1c	1AQ1	2Z7R	3DOG		Sc=6.23222, min distance = 2.40242
KS6A1_HUMAN I	33113 gam	IRAK4_HUMAN Fu	444345 1c	2NRY	2Z7R	2OID		Sc=5.95874, min distance = 2.29995
KS6A1_HUMAN I	33113 gam	PIM1_HUMAN Ful	444345 1c	1YHS	2Z7R	1XR1		Sc=6.45126, min distance = 2.02324
KS6A1_HUMAN I	3543 1g5s	CDK2_HUMAN Ful	444345 1c	1AQ1	2Z7R	1G5S		Sc=6.46292, min distance = 2.03222
KS6A1_HUMAN I	3973 nche	PIM1_HUMAN Ful	444345 1c	1YHS	2Z7R	1YI3		Sc=5.85831, min distance = 2.55506
KS6A1_HUMAN I	4369136 C	CDK2_HUMAN Ful	444345 1c	1AQ1	2Z7R	1DM2		Sc=6.21087, min distance = 2.82798
KS6A1_HUMAN I	440317 AT	PKNB_MYCTU Ful	91532 AMP	1O6Y	2Z7Q	1MRU	0.99	Sc=5.98969, min distance = 2.16688
KS6A1_HUMAN I	443200 C1	ACK1_HUMAN Ful	91532 AMP	1U54	2Z7Q	1U4D		Sc=6.22629, min distance = 1.44653
KS6A1_HUMAN I	444564 AD	BIOD_ECOLI Ful	91532 AMP	1DAG	2Z7Q	1BS1	0.91	Sc=5.95057, min distance = 2.48265
KS6A1_HUMAN I	446048 AM	PGK1_PIG Full=	91532 AMP	1KF0	2Z7Q	1HDI	0.97	Sc=6.16697, min distance = 2.09089
KS6A1_HUMAN I	446704 LS	CDK2_HUMAN Ful	444345 1c	1AQ1	2Z7R	1KE6		Sc=6.22259, min distance = 2.27031
KS6A1_HUMAN I	446795 CI	SRC_HUMAN Full	456214 pu	1YOM	2Z7S	1KSW	0.75	Sc=5.80688, min distance = 2.27120
KS6A1_HUMAN I	447649 1c	CDK2_HUMAN Ful	444345 1c	1AQ1	2Z7R	1OI9		Sc=6.31777, min distance = 2.69702
KS6A1_HUMAN I	447655 1c	CDK2_HUMAN Ful	444345 1c	1AQ1	2Z7R	1OIU		Sc=6.2457, min distance = 2.764709
KS6A1_HUMAN I	447962 1p	CDK2_HUMAN Ful	444345 1c	1AQ1	2Z7R	2C5N		Sc=6.18984, min distance = 2.65375
KS6A1_HUMAN I	448043 2g	KAPCA_BOVIN Fu	444345 1c	1STC	2Z7R	1Q8U		Sc=5.95463, min distance = 2.43450
KS6A1_HUMAN I	448293 CI	CDK2_HUMAN Ful	444345 1c	1AQ1	2Z7R	1R78		Sc=6.40695, min distance = 2.45954
KS6A1_HUMAN I	449240 1y	KAPCA_BOVIN Fu	444345 1c	1STC	2Z7R	1YDR		Sc=5.90464, min distance = 2.30392
KS6A1_HUMAN I	4565 1h1r	CDK2_HUMAN Ful	444345 1c	1AQ1	2Z7R	1H1R		Sc=6.23002, min distance = 2.79718
KS6A1_HUMAN I	4566 1h1s	CDK2_HUMAN Ful	444345 1c	1AQ1	2Z7R	1H1S		Sc=6.24245, min distance = 2.79831
KS6A1_HUMAN I	5288708 1	CDK2_HUMAN Ful	444345 1c	1AQ1	2Z7R	1KE5		Sc=6.08331, min distance = 2.22124
KS6A1_HUMAN I	5288710 C	CDK2_HUMAN Ful	444345 1c	1AQ1	2Z7R	1KE7		Sc=6.29789, min distance = 2.48412
KS6A1_HUMAN I	5327121 I	PIM1_HUMAN Ful	444345 1c	1YHS	2Z7R	2C3I		Sc=6.20376, min distance = 2.46324
KS6A1_HUMAN I	5331010 c	CDK2_HUMAN Ful	444345 1c	1AQ1	2Z7R	2BKZ		Sc=6.19523, min distance = 2.30102
KS6A1_HUMAN I	5687 1di8	CDK2_HUMAN Ful	444345 1c	1AQ1	2Z7R	1DI8		Sc=6.21962, min distance = 2.24613
KS6A1_HUMAN I	5957 Aden	NIFH1_AZOVI Fu	91532 AMP	2AFK	2Z7Q	2C8V	0.99	Sc=5.91362, min distance = 2.06551
KS6A1_HUMAN I	6022 Aden	BIOD_ECOLI Ful	91532 AMP	1DAG	2Z7Q	1DAD	0.99	Sc=5.83817, min distance = 2.34355
KS6A1_HUMAN I	6022 Aden	CDK2_HUMAN Ful	444345 1c	1AQ1	2Z7R	1GY3		Sc=6.38594, min distance = 2.03503
KS6A1_HUMAN I	60961 ade	PIM1_HUMAN Ful	444345 1c	1YHS	2Z7R	1YI4		Sc=6.16886, min distance = 2.28556
KS6A1_HUMAN I	6420139 C	CDK2_HUMAN Ful	444345 1c	1AQ1	2Z7R	2C5V		Sc=6.08331, min distance = 2.72987
KS6A1_HUMAN I	6539118 g	CDK2_HUMAN Ful	444345 1c	1AQ1	2Z7R	1FVV		Sc=6.39741, min distance = 2.18908
KS6A1_HUMAN I	6540272 C	CHK1_HUMAN Ful	444345 1c	1NVR	2Z7R	2CGU		Sc=6.38236, min distance = 2.55783
KS6A1_HUMAN I	6540273 2	CHK1_HUMAN Ful	444345 1c	1NVR	2Z7R	2CGV		Sc=6.12509, min distance = 2.23157

KS6A1_HUMAN I	656971 CI	CDK2_HUMAN Fu	444345 1c	1AQ1	2Z7R	1Y8Y			Sc=5.97999, min distance = 2.96064
KS6A1_HUMAN I	6852207 M	KAPCA_BOVIN Fu	444345 1c	1STC	2Z7R	2GNI			Sc=5.9169, min distance = 2.313321
KS6A1_HUMAN I	848641 2b	CHK1_HUMAN Fu	444345 1c	1NVR	2Z7R	2BRH			Sc=6.17973, min distance = 2.14655
KS6A1_HUMAN I	9548003 C	KC1G3_HUMAN Fu	456214 pu	2IZU	2Z7S	2IZT		0.9	Sc=5.86582, min distance = 2.79270
KS6A1_HUMAN I	9817550 V	CDK2_HUMAN Fu	444345 1c	1AQ1	2Z7R	3BHV			Sc=6.35579, min distance = 2.37880
KSYK_HUMAN Fu	11348631	CHK1_HUMAN Fu	444345 1c	1NVR	1XBC	2E9U			Sc=6.32019, min distance = 2.22158
KSYK_HUMAN Fu	11553058	LCK_HUMAN Ful	444345 1c	1QPD	1XBC	2OF2			Sc=6.29916, min distance = 2.45666
KSYK_HUMAN Fu	11957417	CDK2_HUMAN Fu	444345 1c	1AQ1	1XBC	2I40			Sc=6.30057, min distance = 2.40849
KSYK_HUMAN Fu	11986115	PIM1_HUMAN Fu	444345 1c	1YHS	1XBC	2BZJ			Sc=6.27557, min distance = 2.35937
KSYK_HUMAN Fu	11992146	MAPK2_HUMAN Fu	444345 1c	2PZY	1XBC	2P3G			Sc=6.30438, min distance = 2.07236
KSYK_HUMAN Fu	1540 1h1q	CDK2_HUMAN Fu	444345 1c	1AQ1	1XBC	1H1Q			Sc=6.24016, min distance = 2.62481
KSYK_HUMAN Fu	15942652	CDK2_HUMAN Fu	444345 1c	1AQ1	1XBC	2DUV			Sc=6.30424, min distance = 2.26460
KSYK_HUMAN Fu	15991572	LCK_HUMAN Ful	444345 1c	1QPD	1XBC	2OF4			Sc=6.26802, min distance = 2.66020
KSYK_HUMAN Fu	16122608	PDPK1_HUMAN Fu	444345 1c	1OKY	1XBC	2PE1			Sc=6.34141, min distance = 2.72482
KSYK_HUMAN Fu	16122643	CHK1_HUMAN Fu	444345 1c	1NVR	1XBC	2YWP			Sc=6.33889, min distance = 2.18105
KSYK_HUMAN Fu	16214825	CDK2_HUMAN Fu	444345 1c	1AQ1	1XBC	2UZE			Sc=6.24634, min distance = 2.41404
KSYK_HUMAN Fu	16214827	CDK2_HUMAN Fu	444345 1c	1AQ1	1XBC	2UZN			Sc=6.08505, min distance = 2.11301
KSYK_HUMAN Fu	1907917 2	CHK1_HUMAN Fu	444345 1c	1NVR	1XBC	2CGW			Sc=6.13917, min distance = 3.00948
KSYK_HUMAN Fu	23536770	LCK_HUMAN Ful	444345 1c	1QPD	1XBC	3BYM			Sc=6.52309, min distance = 2.06837
KSYK_HUMAN Fu	23653515	CDK2_HUMAN Fu	444345 1c	1AQ1	1XBC	2R3F			Sc=6.00907, min distance = 2.57320
KSYK_HUMAN Fu	23657800	CHK1_HUMAN Fu	444345 1c	1NVR	1XBC	2E9P			Sc=6.33601, min distance = 2.02803
KSYK_HUMAN Fu	23727982	CDK2_HUMAN Fu	444345 1c	1AQ1	1XBC	3BHU			Sc=6.2322, min distance = 2.397987
KSYK_HUMAN Fu	2396 bisi	PDPK1_HUMAN Fu	444345 1c	1OKY	1XBC	1UU8			Sc=6.39224, min distance = 2.00487
KSYK_HUMAN Fu	2398 bisi	PDPK1_HUMAN Fu	444345 1c	1OKY	1XBC	1UU9			Sc=6.44814, min distance = 2.22388
KSYK_HUMAN Fu	2403 1uvr	PDPK1_HUMAN Fu	444345 1c	1OKY	1XBC	1UVR			Sc=6.39117, min distance = 2.24926
KSYK_HUMAN Fu	24752838	CHK1_HUMAN Fu	444345 1c	1NVR	1XBC	2QHM			Sc=6.04983, min distance = 3.07184
KSYK_HUMAN Fu	24762195	KAPCA_BOVIN Fu	444345 1c	1STC	1XBC	2VO0			Sc=6.27341, min distance = 2.22416
KSYK_HUMAN Fu	24905143	PK3CG_HUMAN Fu	444345 1c	1E8Z	1XBC	2V4L			Sc=6.09899, min distance = 2.46089
KSYK_HUMAN Fu	24905144	PK3CG_HUMAN Fu	444345 1c	1E8Z	1XBC	3ENE			Sc=6.08724, min distance = 2.48901
KSYK_HUMAN Fu	24916751	CDK2_HUMAN Fu	444345 1c	1AQ1	1XBC	3DOG			Sc=6.25872, min distance = 2.01403
KSYK_HUMAN Fu	25021197	CDK2_HUMAN Fu	444345 1c	1AQ1	1XBC	3EOC			Sc=6.21508, min distance = 2.46777
KSYK_HUMAN Fu	4369136 C	CDK2_HUMAN Fu	444345 1c	1AQ1	1XBC	1DM2			Sc=6.23385, min distance = 2.96925
KSYK_HUMAN Fu	446704 LS	CDK2_HUMAN Fu	444345 1c	1AQ1	1XBC	1KE6			Sc=6.25949, min distance = 2.41752
KSYK_HUMAN Fu	447962 1p	CDK2_HUMAN Fu	444345 1c	1AQ1	1XBC	2C5N			Sc=6.22259, min distance = 2.16192
KSYK_HUMAN Fu	447967 CI	CDK2_HUMAN Fu	444345 1c	1AQ1	1XBC	1PYE			Sc=6.34996, min distance = 2.37446
KSYK_HUMAN Fu	448043 2g	KAPCA_BOVIN Fu	444345 1c	1STC	1XBC	1Q8U			Sc=5.95735, min distance = 2.84610
KSYK_HUMAN Fu	448293 CI	CDK2_HUMAN Fu	444345 1c	1AQ1	1XBC	1R78			Sc=6.44386, min distance = 2.45603

KSYK_HUMAN Fu	456214 pu	KS6A1_HUMAN Fu	444345 1c	2Z7R	1XBC	2Z7S			Sc=6.37104, min distance = 2.06131
KSYK_HUMAN Fu	4565 1h1r	CDK2_HUMAN Fu	444345 1c	1AQ1	1XBC	1H1R			Sc=6.2448, min distance = 2.836374
KSYK_HUMAN Fu	5327123 I	CHK1_HUMAN Fu	444345 1c	1NVR	1XBC	2C3L			Sc=6.14509, min distance = 2.44311
KSYK_HUMAN Fu	5331010 d	CDK2_HUMAN Fu	444345 1c	1AQ1	1XBC	2BKZ			Sc=6.21011, min distance = 2.13923
KSYK_HUMAN Fu	5687 1di8	CDK2_HUMAN Fu	444345 1c	1AQ1	1XBC	1DI8			Sc=6.23849, min distance = 2.59407
KSYK_HUMAN Fu	6420138 2	CDK2_HUMAN Fu	444345 1c	1AQ1	1XBC	2UUE			Sc=6.33258, min distance = 1.85673
KSYK_HUMAN Fu	6420139 C	CDK2_HUMAN Fu	444345 1c	1AQ1	1XBC	2C5V			Sc=6.20072, min distance = 2.46885
KSYK_HUMAN Fu	6540273 2	CHK1_HUMAN Fu	444345 1c	1NVR	1XBC	2CGV			Sc=6.17225, min distance = 2.24532
KSYK_HUMAN Fu	6852201 2	CDK2_HUMAN Fu	444345 1c	1AQ1	1XBC	2G9X			Sc=6.49108, min distance = 1.78695
KSYK_HUMAN Fu	6852207 M	KAPCA_BOVIN Fu	444345 1c	1STC	1XBC	2GNI			Sc=5.90625, min distance = 2.57085
KSYK_HUMAN Fu	72271 7-H	CDK2_HUMAN Fu	444345 1c	1AQ1	1XBC	1PKD		0.91	Sc=6.44344, min distance = 2.42380
KSYK_HUMAN Fu	9817550 V	CDK2_HUMAN Fu	444345 1c	1AQ1	1XBC	3BHV			Sc=6.33168, min distance = 2.14916
KTHY2_SULTO I	10918 Car	OCTC_MOUSE Fu	23831 HEP	1XL7	2PLR	1XL8			Sc=5.80476, min distance = 0.87951
KTHY_HUMAN Fu	1855 CID1	KITH_HHV11 Fu	6022 Aden	2VTK	1NN3	1E2P			Sc=6.10978, min distance = 2.15041
KTHY_HUMAN Fu	1865 6-hy	KITH_HHV11 Fu	9700 thym	1VTK	1E2F	1E2M			Sc=6.01982, min distance = 2.65394
KTHY_HUMAN Fu	3547 Fasu	KAPCA_BOVIN Fu	5957 Aden	1Q24	1E2Q	1Q8W			Sc=5.87289, min distance = 2.09517
KTHY_HUMAN Fu	444320 2'	TYSY_ECOLI Fu	9700 thym	1TYS	1E2F	1AOB		0.81	Sc=5.99243, min distance = 2.29087
KTHY_HUMAN Fu	444564 AD	MYS2_DICDI Fu	6022 Aden	1VOM	1NN3	1W9I		0.91	Sc=5.77812, min distance = 2.38053
KTHY_HUMAN Fu	444842 CI	CDK2_HUMAN Fu	6022 Aden	1GY3	1NN3	1CKP			Sc=5.60554, min distance = 2.80618
KTHY_HUMAN Fu	445154 re	ATPA1_BOVIN Fu	6022 Aden	2CK3	1NN3	2JIZ			Sc=5.88046, min distance = 2.60878
KTHY_HUMAN Fu	445210 9-	KITH_HHV11 Fu	9700 thym	1VTK	1E2F	1E2I			Sc=6.03026, min distance = 2.33238
KTHY_HUMAN Fu	445213 HY	KITH_HHV11 Fu	9700 thym	1VTK	1E2F	1E2N			Sc=6.43425, min distance = 2.18696
KTHY_HUMAN Fu	446725 BV	KITH_HHV11 Fu	6022 Aden	2VTK	1NN3	1KI4			Sc=6.30572, min distance = 2.06076
KTHY_HUMAN Fu	446727 Br	KITH_HHV11 Fu	6022 Aden	2VTK	1NN3	1KI8			Sc=6.20803, min distance = 2.13835
KTHY_HUMAN Fu	447004 CI	MYS2_DICDI Fu	33113 gam	1MMN	1NN5	1LVK			Sc=5.8328, min distance = 2.224634
KTHY_HUMAN Fu	447004 CI	MYS2_DICDI Fu	6022 Aden	1VOM	1NN3	1LVK			Sc=5.73324, min distance = 2.25282
KTHY_HUMAN Fu	447206 1m	KTHY_MYCTU Fu	445269 CI	1W2H	1E9B	1MRS	32.24	0.81	Sc=6.37713, min distance = 2.32228
KTHY_HUMAN Fu	447206 1m	KTHY_MYCTU Fu	9700 thym	1GTV	1E2F	1MRS	32.24	0.95	Sc=6.3457, min distance = 2.021391
KTHY_HUMAN Fu	447628 [(	KITH_HHV11 Fu	9700 thym	1VTK	1E2F	1OF1			Sc=6.24382, min distance = 2.43795
KTHY_HUMAN Fu	448042 2e	ROCK1_HUMAN Fu	33113 gam	2V55	1NN5	2ETR			Sc=6.14509, min distance = 2.46529
KTHY_HUMAN Fu	448043 2g	ROCK1_HUMAN Fu	33113 gam	2V55	1NN5	3D9V			Sc=6.00229, min distance = 1.88411
KTHY_HUMAN Fu	449489 Io	KITH_HHV11 Fu	9700 thym	1VTK	1E2F	3VTK		0.91	Sc=6.23776, min distance = 2.37114
KTHY_HUMAN Fu	5789 thym	KTHY_MYCTU Fu	164628 de	1GTV	1E2G	1W2G	32.24	0.9	Sc=6.18118, min distance = 2.44071
KTHY_HUMAN Fu	5789 thym	KTHY_MYCTU Fu	445269 CI	1W2H	1E9B	1W2G	32.24	0.8	Sc=6.1822, min distance = 2.449760
KTHY_HUMAN Fu	5789 thym	KTHY_MYCTU Fu	9700 thym	1GTV	1E2F	1W2G	32.24	0.92	Sc=6.16543, min distance = 2.73811
KTHY_HUMAN Fu	5789 thym	Q9HU22_PSEAE I	9700 thym	1FXO	1E2F	1G0R		0.92	Sc=6.18422, min distance = 2.51616
KTHY_HUMAN Fu	5789 thym	RMLA1_ECOLI Fu	164628 de	1H5T	1E2G	1H5R		0.9	Sc=6.24331, min distance = 1.45111

KTHY_HUMAN F	5905 idox	KITH_HHV11 Fu	6022 Aden	2VTK	1NN3	1KI7			Sc=6.09473, min distance = 2.09126
KTHY_HUMAN F	6031 Uric	ECX1_PYRAB Fu	6022 Aden	2PO0	1NN3	2PNZ			Sc=5.91313, min distance = 2.24246
KTHY_MYCTU F	13711 dec	DNK_DROME Ful	5789 thym	1OT3	1W2G	2VP5			Sc=6.12596, min distance = 2.70268
KTHY_MYCTU F	13712 dec	TYSY_ECOLI Fu	9700 thym	1TYS	1GTV	1BDU		0.83	Sc=6.11535, min distance = 2.31022
KTHY_MYCTU F	16122607	PDPK1_HUMAN F	5957 Aden	2BIY	1N5I	2PE0			Sc=5.94918, min distance = 2.12516
KTHY_MYCTU F	16122608	PDPK1_HUMAN F	5957 Aden	2BIY	1N5I	2PE1			Sc=6.14065, min distance = 2.11249
KTHY_MYCTU F	162501 Am	KTHY_HUMAN Fu	164628 de	1E2G	1GTV	1NMZ	32.24	0.86	Sc=6.34436, min distance = 2.29038
KTHY_MYCTU F	165151 do	KTHY_HUMAN Fu	164628 de	1E2G	1GTV	1NN5	32.24	0.9	Sc=6.28507, min distance = 2.06808
KTHY_MYCTU F	17754396	PDPK1_HUMAN F	5957 Aden	2BIY	1N5I	2R7B			Sc=6.13618, min distance = 2.01532
KTHY_MYCTU F	1855 CID1	KITH_HHV11 Fu	5789 thym	1P7C	1W2G	1E2P			Sc=6.18405, min distance = 2.42992
KTHY_MYCTU F	1855 CID1	KITH_HHV11 Fu	9700 thym	1VTK	1GTV	1E2P			Sc=6.19122, min distance = 2.17503
KTHY_MYCTU F	1865 6-hy	KITH_HHV11 Fu	5789 thym	1P7C	1W2G	1E2M			Sc=6.04967, min distance = 2.64873
KTHY_MYCTU F	1865 6-hy	KITH_HHV11 Fu	9700 thym	1VTK	1GTV	1E2M			Sc=6.06449, min distance = 2.56944
KTHY_MYCTU F	3454 gano	KITH_HHV11 Fu	5789 thym	1P7C	1W2G	1KI2			Sc=6.2737, min distance = 1.748570
KTHY_MYCTU F	444320 2'	TYSY_ECOLI Fu	9700 thym	1TYS	1GTV	1AOB		0.81	Sc=6.03303, min distance = 2.27236
KTHY_MYCTU F	445210 9-	KITH_HHV11 Fu	5789 thym	1P7C	1W2G	1E2I			Sc=6.07467, min distance = 2.06122
KTHY_MYCTU F	445211 9-	KITH_HHV11 Fu	5789 thym	1P7C	1W2G	1E2I			Sc=6.07373, min distance = 2.00656
KTHY_MYCTU F	445213 HY	KITH_HHV11 Fu	5789 thym	1P7C	1W2G	1E2N			Sc=5.98278, min distance = 2.29790
KTHY_MYCTU F	445213 HY	KITH_HHV11 Fu	9700 thym	1VTK	1GTV	1E2N			Sc=6.45341, min distance = 2.02276
KTHY_MYCTU F	446726 AI	KITH_HHV11 Fu	5789 thym	1P7C	1W2G	1KI6		0.77	Sc=6.2192, min distance = 2.679887
KTHY_MYCTU F	447628 [(	KITH_HHV11 Fu	5789 thym	1P7C	1W2G	1OF1			Sc=6.27206, min distance = 2.74988
KTHY_MYCTU F	447628 [(	KITH_HHV11 Fu	9700 thym	1VTK	1GTV	1OF1			Sc=6.26863, min distance = 2.46741
KTHY_MYCTU F	448782 CI	TYSY_ECOLI Fu	9700 thym	1TYS	1GTV	1TSN		0.79	Sc=6.27134, min distance = 2.68953
KTHY_MYCTU F	449489 Id	KITH_HHV11 Fu	5789 thym	1P7C	1W2G	3VTK		0.84	Sc=6.26337, min distance = 2.24243
KTHY_MYCTU F	449489 Id	KITH_HHV11 Fu	9700 thym	1VTK	1GTV	3VTK		0.91	Sc=6.26337, min distance = 2.73918
KTHY_MYCTU F	514815 BE	DNK_DROME Ful	5789 thym	1OT3	1W2G	2VQS		0.9	Sc=6.27218, min distance = 2.16533
KTHY_MYCTU F	51 2-Oxop	SERA_ECOLI Fu	311 citri	2P9E	1N5I	1YBA		0.76	Sc=5.67557, min distance = 1.54327
KTHY_MYCTU F	65059 Dec	TYSY_ECOLI Fu	9700 thym	1TYS	1GTV	1TLC			Sc=6.38744, min distance = 1.81315
KTHY_STAAM F	1855 CID1	KITH_HHV11 Fu	9700 thym	1VTK	2CCJ	1E2P			Sc=6.06022, min distance = 2.28746
KTHY_STAAM F	445210 9-	KITH_HHV11 Fu	9700 thym	1VTK	2CCJ	1E2I			Sc=5.99137, min distance = 0
KTHY_STAAM F	445213 HY	KITH_HHV11 Fu	9700 thym	1VTK	2CCJ	1E2N			Sc=6.01654, min distance = 2.04577
KTHY_STAAM F	445269 CI	KTHY_YEAST Fu	9700 thym	1TMK	2CCJ	2TMK		0.85	Sc=5.90304, min distance = 2.37237
KTHY_STAAM F	448782 CI	TYSY_ECOLI Fu	9700 thym	1TYS	2CCJ	1TSN		0.79	Sc=6.16952, min distance = 2.18284
KTHY_STAAM F	449489 Id	KITH_HHV11 Fu	9700 thym	1VTK	2CCJ	3VTK		0.91	Sc=5.8031, min distance = 2.741799
KTHY_STAAM F	5789 thym	RMLA1_ECOLI F	9700 thym	1H5S	2CCJ	1H5R		0.92	Sc=6.15533, min distance = 1.39110
KTHY_YEAST F	12599 dAM	YFBR_ECOLI Fu	9700 thym	2PAR	1TMK	2PAU			Sc=6.40693, min distance = 2.35699
KTHY_YEAST F	13712 dec	TYSY_ECOLI Fu	9700 thym	1TYS	1TMK	1BDU		0.83	Sc=6.12799, min distance = 2.01567

KTHY_YEAST Fı	164628 de	KTHY_MYCTU Fu	445269 CI	1W2H	2TMK	1GTV		0.83	Sc=6.40987, min distance = 2.24102
KTHY_YEAST Fı	1855 CID1	KITH_HHV11 Fu	9700 thym	1VTK	1TMK	1E2P			Sc=6.19713, min distance = 2.06936
KTHY_YEAST Fı	1865 6-hy	KITH_HHV11 Fu	9700 thym	1VTK	1TMK	1E2M			Sc=6.05766, min distance = 2.22968
KTHY_YEAST Fı	445211 9-	KITH_HHV11 Fu	9700 thym	1VTK	1TMK	1E2I			Sc=6.06814, min distance = 0
KTHY_YEAST Fı	445213 HY	KITH_HHV11 Fu	9700 thym	1VTK	1TMK	1E2N			Sc=6.47353, min distance = 1.87998
KTHY_YEAST Fı	446727 Br	KITH_HHV11 Fu	9700 thym	1VTK	1TMK	1KI8		0.83	Sc=6.28088, min distance = 2.06178
KTHY_YEAST Fı	447628 [(	KITH_HHV11 Fu	9700 thym	1VTK	1TMK	1OF1			Sc=6.27852, min distance = 2.46067
KTHY_YEAST Fı	448782 CI	TYSY_ECOLI Fu	9700 thym	1TYS	1TMK	1TSN		0.79	Sc=6.28658, min distance = 2.39892
KTHY_YEAST Fı	449489 Ic	KITH_HHV11 Fu	9700 thym	1VTK	1TMK	3VTK		0.91	Sc=6.28439, min distance = 2.57111
KTHY_YEAST Fı	5789 thym	KTHY_MYCTU Fu	445269 CI	1W2H	2TMK	1W2G		0.8	Sc=6.20371, min distance = 2.38192
KTHY_YEAST Fı	5789 thym	KTHY_MYCTU Fu	9700 thym	1GTV	1TMK	1W2G		0.92	Sc=6.20659, min distance = 2.67308
KTRA_BACSU Fı	11608401	KAPCA_BOVIN Fı	5957 Aden	1Q24	2HMW	2UVY			Sc=6.25284, min distance = 2.18969
KTRA_BACSU Fı	11708454	KAPCA_BOVIN Fı	5957 Aden	1Q24	2HMW	2VNW			Sc=6.14216, min distance = 2.46115
KTRA_BACSU Fı	16122632	KAPCA_BOVIN Fı	5957 Aden	1Q24	2HMW	2UVZ			Sc=6.37158, min distance = 1.94796
KTRA_BACSU Fı	16122635	KAPCA_BOVIN Fı	5957 Aden	1Q24	2HMW	2UW8			Sc=5.80043, min distance = 2.10369
KTRA_BACSU Fı	24762195	KAPCA_BOVIN Fı	5957 Aden	1Q24	2HMW	2VO0			Sc=6.3051, min distance = 2.440312
KTRA_BACSU Fı	3540 1yds	KAPCA_BOVIN Fı	5957 Aden	1Q24	2HMW	1YDS			Sc=5.93725, min distance = 2.20824
KTRA_BACSU Fı	3547 Fasu	KAPCA_BOVIN Fı	5957 Aden	1Q24	2HMW	1Q8W			Sc=5.98692, min distance = 2.35939
KTRA_BACSU Fı	6022 Aden	CLCN5_HUMAN Fı	5957 Aden	2J9L	2HMW	2JA3		0.99	Sc=6.43822, min distance = 1.21650
KTRA_BACSU Fı	6022 Aden	MUTS_ECOLI Fu	5957 Aden	1W7A	2HMW	1OH7		0.99	Sc=6.37067, min distance = 2.14645
KTRA_BACSU Fı	6022 Aden	PUR7_METJA Fu	5957 Aden	2Z02	2HMW	2YZL		0.99	Sc=5.95402, min distance = 2.05496
KTRA_BACSU Fı	6022 Aden	REX_BACSU Ful	5957 Aden	2VT3	2HMW	2VT2		0.99	Sc=5.97404, min distance = 1.76618
LAC1_MELAO Fı	24139 ace	Q9HYN5_PSEAE I	439680 be	1OUR	2Q9O	1W8F			Sc=5.9442, min distance = 2.056002
LACB_BOVIN Fı	14253 Cet	FABP4_MOUSE Fı	985 palmi	1LIE	1GXA	1LIC			Sc=5.61978, min distance = 2.04939
LACB_BOVIN Fı	5280934 l	NLTP_MAIZE Fu	985 palmi	1MZM	1GXA	1FK6		0.75	Sc=5.91714, min distance = 2.49892
LACC_ENTFA Fı	11608401	KAPCA_BOVIN Fı	5957 Aden	1Q24	2F02	2UVY			Sc=6.19023, min distance = 2.43228
LACC_ENTFA Fı	11708454	KAPCA_BOVIN Fı	5957 Aden	1Q24	2F02	2VNW			Sc=6.08661, min distance = 2.16007
LACC_ENTFA Fı	129236 2c	CKI1_SCHPO Fu	5957 Aden	1CSN	2F02	2CSN			Sc=5.78163, min distance = 2.38852
LACC_ENTFA Fı	16122607	PDPK1_HUMAN Fı	5957 Aden	2BIY	2F02	2PE0			Sc=6.12197, min distance = 1.84288
LACC_ENTFA Fı	16122608	PDPK1_HUMAN Fı	5957 Aden	2BIY	2F02	2PE1			Sc=6.20571, min distance = 2.18731
LACC_ENTFA Fı	3540 1yds	KAPCA_BOVIN Fı	5957 Aden	1Q24	2F02	1YDS			Sc=5.86216, min distance = 2.34443
LACC_ENTFA Fı	3547 Fasu	KAPCA_BOVIN Fı	5957 Aden	1Q24	2F02	1Q8W			Sc=5.93439, min distance = 2.25403
LACC_ENTFA Fı	440317 AT	PURT_ECOLI Fu	5957 Aden	1KJ8	2F02	1KJJ		0.99	Sc=6.42789, min distance = 2.38780
LACC_ENTFA Fı	444564 AD	BIOD_ECOLI Fu	5957 Aden	1A82	2F02	1BS1		0.91	Sc=6.37677, min distance = 2.06417
LACC_ENTFA Fı	444852 CI	ATPA1_BOVIN Fı	5957 Aden	2V7Q	2F02	1COW		0.91	Sc=6.44038, min distance = 2.26535
LACC_ENTFA Fı	447004 CI	MYS2_DICDI Fu	5957 Aden	1FMW	2F02	1LVK			Sc=6.45752, min distance = 0
LACC_ENTFA Fı	447840 CI	PHKG1_RABIT Fı	5957 Aden	1QL6	2F02	1PHK			Sc=5.97251, min distance = 1.98329

LACC_ENTFA Fu	447916 ac	RIO1_ARCFU Ful	5957 Aden	1ZP9	2F02	1ZTF	0.94	Sc=6.19177, min distance = 2.66537
LACC_ENTFA Fu	448042 2e	KAPCA_BOVIN Fu	5957 Aden	1Q24	2F02	1Q8T		Sc=6.14361, min distance = 2.135526
LACC_ENTFA Fu	448043 2g	KAPCA_BOVIN Fu	5957 Aden	1Q24	2F02	1Q8U		Sc=6.0168, min distance = 2.083004
LACC_ENTFA Fu	449240 1y	KAPCA_BOVIN Fu	5957 Aden	1Q24	2F02	1YDR		Sc=5.92108, min distance = 2.00675
LACC_ENTFA Fu	6022 Aden	ACT1_DROME Ful	5957 Aden	2HF4	2F02	2HF3	0.99	Sc=5.92216, min distance = 2.34603
LACC_ENTFA Fu	6022 Aden	ARSA1_ECOLX Fu	5957 Aden	1II0	2F02	1IHU	0.99	Sc=6.34631, min distance = 2.09155
LACC_ENTFA Fu	6022 Aden	BIOD_ECOLI Ful	5957 Aden	1A82	2F02	1DAD	0.99	Sc=6.36505, min distance = 1.98718
LACC_ENTFA Fu	6022 Aden	CLCN5_HUMAN Fu	5957 Aden	2J9L	2F02	2JA3	0.99	Sc=6.39849, min distance = 1.342466
LACC_ENTFA Fu	6022 Aden	DDL_THET8 Ful	5957 Aden	2ZDQ	2F02	2ZDH	0.99	Sc=6.40442, min distance = 2.08462
LACC_ENTFA Fu	6022 Aden	FAK1_HUMAN Ful	5957 Aden	2IJM	2F02	1MP8	0.99	Sc=6.39251, min distance = 1.70325
LACC_ENTFA Fu	6022 Aden	HSP7F_YEAST Fu	5957 Aden	3D2F	2F02	3C7N	0.99	Sc=6.38281, min distance = 1.91556
LACC_ENTFA Fu	6022 Aden	KTHY_HUMAN Ful	5957 Aden	1E2Q	2F02	1NN3	0.99	Sc=6.35803, min distance = 2.24771
LACC_ENTFA Fu	6022 Aden	MUTS_ECOLI Ful	5957 Aden	1W7A	2F02	1OH7	0.99	Sc=6.35154, min distance = 2.49064
LACC_ENTFA Fu	6022 Aden	PPCK_ECOLI Ful	5957 Aden	2OLR	2F02	1K3C	0.99	Sc=6.34405, min distance = 2.26395
LACC_ENTFA Fu	6022 Aden	Q72H90_THET2	5957 Aden	2BEK	2F02	2BEJ	0.99	Sc=6.36326, min distance = 2.32740
LACC_ENTFA Fu	6022 Aden	REX_BACSU Ful	5957 Aden	2VT3	2F02	2VT2	0.99	Sc=6.39142, min distance = 0.96729
LACC_ENTFA Fu	6022 Aden	RK_BOVIN Full=	5957 Aden	3C4W	2F02	3C4Z	0.99	Sc=6.37067, min distance = 2.44113
LACC_ENTFA Fu	6022 Aden	Y059_METJA Ful	5957 Aden	2J9C	2F02	2J9D	0.99	Sc=6.38867, min distance = 1.89575
LACC_ENTFA Fu	6083 aden	AAKG1_RAT Ful	5957 Aden	2V92	2F02	2V8Q	0.98	Sc=6.32842, min distance = 2.09602
LACC_ENTFA Fu	6083 aden	PURP_PYRFU Ful	5957 Aden	2R86	2F02	2R85	0.98	Sc=6.32743, min distance = 2.31070
LACC_ENTFA Fu	6083 aden	Y059_METJA Ful	5957 Aden	2J9C	2F02	2J9D	0.98	Sc=6.31254, min distance = 2.06844
LACC_ENTFA Fu	60961 ade	SYQ_ECOLI Ful	5957 Aden	1GTR	2F02	1O0B	0.94	Sc=6.15065, min distance = 2.40779
LACC_ENTFA Fu	6830 guar	Q381M1_9TRYP	5957 Aden	2Q0D	2F02	2Q0E	0.8	Sc=5.98423, min distance = 1.71419
LACC_ENTFA Fu	91532 AMP	AROK_MYCTU Ful	5957 Aden	2IYW	2F02	1ZYU	0.99	Sc=5.96474, min distance = 2.43589
LACC_ENTFA Fu	91532 AMP	PDXK_SHEEP Ful	5957 Aden	1LHR	2F02	1RFT	0.99	Sc=6.43796, min distance = 2.67056
LACC_ENTFA Fu	91532 AMP	PURP_METJA Ful	5957 Aden	2R7L	2F02	2R7K	0.99	Sc=6.02685, min distance = 1.93543
LACC_STAA8 Fu	1540 1h1q	CDK2_HUMAN Ful	6022 Aden	1GY3	2JGV	1H1Q		Sc=6.27029, min distance = 1.36585
LACC_STAA8 Fu	15993 dAT	PRIM_BPT7 Ful	33113 gam	1E0J	2JG1	1CR2	0.97	Sc=6.44104, min distance = 2.42955
LACC_STAA8 Fu	160355 rc	CDK2_HUMAN Ful	6022 Aden	1GY3	2JGV	3DDQ		Sc=6.45395, min distance = 1.42400
LACC_STAA8 Fu	16750062	CSK2A_MAIZE Fu	33113 gam	1LP4	2JG1	2OXD		Sc=5.67768, min distance = 2.28194
LACC_STAA8 Fu	1694 TBBt	CSK2A_MAIZE Fu	33113 gam	1LP4	2JG1	1J91		Sc=5.6348, min distance = 2.0600548
LACC_STAA8 Fu	188966 dA	HSLU_ECOLI Ful	33113 gam	1E94	2JG1	1G4A	0.97	Sc=6.41367, min distance = 2.11453
LACC_STAA8 Fu	23653522	CDK2_HUMAN Ful	6022 Aden	1GY3	2JGV	2R3N		Sc=6.37424, min distance = 2.02557
LACC_STAA8 Fu	23727981	CDK2_HUMAN Ful	6022 Aden	1GY3	2JGV	3BHT		Sc=5.94603, min distance = 2.80384
LACC_STAA8 Fu	24756824	CSK2A_MAIZE Fu	33113 gam	1LP4	2JG1	3BE9		Sc=6.5088, min distance = 1.719114
LACC_STAA8 Fu	24779674	CSK2A_MAIZE Fu	33113 gam	1LP4	2JG1	2PVH		Sc=6.363, min distance = 1.7849865
LACC_STAA8 Fu	24779677	CSK2A_MAIZE Fu	33113 gam	1LP4	2JG1	2PVL		Sc=6.41285, min distance = 2.10109

LACC_STAA8 Fu	24779678	CSK2A_MAIZE Fu	33113 gam	1LP4	2JG1	2PVM			Sc=6.50809, min distance = 1.86067
LACC_STAA8 Fu	24832037	PGK1_HUMAN Fu	6022 Aden	2ZGV	2JGV	3C3C			Sc=5.78687, min distance = 2.06130
LACC_STAA8 Fu	24864080	CDK2_HUMAN Fu	6022 Aden	1GY3	2JGV	2VTR			Sc=5.9709, min distance = 1.345693
LACC_STAA8 Fu	25021197	CDK2_HUMAN Fu	6022 Aden	1GY3	2JGV	3EOC			Sc=6.38992, min distance = 1.97165
LACC_STAA8 Fu	3064778 H	ROCK1_HUMAN Fu	33113 gam	2V55	2JG1	2ETK			Sc=6.08885, min distance = 1.89892
LACC_STAA8 Fu	3547 Fasu	ROCK1_HUMAN Fu	33113 gam	2V55	2JG1	2ESM			Sc=5.98053, min distance = 2.47990
LACC_STAA8 Fu	36735 Gpp	CSK2A_MAIZE Fu	33113 gam	1LP4	2JG1	1DAY		0.8	Sc=6.05364, min distance = 2.16514
LACC_STAA8 Fu	398148 1e	CDK2_HUMAN Fu	6022 Aden	1GY3	2JGV	1E1X			Sc=6.23701, min distance = 1.48707
LACC_STAA8 Fu	4369136 C	CDK2_HUMAN Fu	6022 Aden	1GY3	2JGV	1DM2			Sc=6.12575, min distance = 2.44364
LACC_STAA8 Fu	4369271 C	CSK2A_MAIZE Fu	33113 gam	1LP4	2JG1	1M2P			Sc=6.32291, min distance = 2.13477
LACC_STAA8 Fu	444345 1c	CDK2_HUMAN Fu	6022 Aden	1GY3	2JGV	1AQ1			Sc=6.36104, min distance = 2.24539
LACC_STAA8 Fu	444564 AD	BIOD_ECOLI Fu	6022 Aden	1DAD	2JGV	1BS1		0.91	Sc=6.33616, min distance = 2.05353
LACC_STAA8 Fu	444564 AD	MYS2_DICDI Fu	33113 gam	1MMN	2JG1	1W9I		0.91	Sc=6.39535, min distance = 2.56818
LACC_STAA8 Fu	444564 AD	MYS2_DICDI Fu	6022 Aden	1VOM	2JGV	1W9I		0.91	Sc=6.39232, min distance = 2.26126
LACC_STAA8 Fu	444852 CI	ATPB_BOVIN Fu	6022 Aden	2CK3	2JGV	1COW		0.9	Sc=6.41135, min distance = 2.23745
LACC_STAA8 Fu	445840 di	CDK2_HUMAN Fu	6022 Aden	1GY3	2JGV	1GII			Sc=6.31622, min distance = 1.19693
LACC_STAA8 Fu	445966 O6	CDK2_HUMAN Fu	6022 Aden	1GY3	2JGV	1H0V			Sc=6.19103, min distance = 1.98002
LACC_STAA8 Fu	447004 CI	MYS2_DICDI Fu	33113 gam	1MMN	2JG1	1LVK			Sc=6.51284, min distance = 2.06477
LACC_STAA8 Fu	447649 1c	CDK2_HUMAN Fu	6022 Aden	1GY3	2JGV	1OI9			Sc=6.3631, min distance = 1.552372
LACC_STAA8 Fu	447655 1c	CDK2_HUMAN Fu	6022 Aden	1GY3	2JGV	1OIU			Sc=6.23795, min distance = 1.65847
LACC_STAA8 Fu	447766 1p	CDK2_HUMAN Fu	6022 Aden	1GY3	2JGV	1P2A			Sc=6.28199, min distance = 2.14883
LACC_STAA8 Fu	448005 CI	GSK3B_HUMAN Fu	6022 Aden	1J1C	2JGV	1Q3W			Sc=6.27833, min distance = 2.04421
LACC_STAA8 Fu	448042 2e	ROCK1_HUMAN Fu	33113 gam	2V55	2JG1	2ETR			Sc=6.15459, min distance = 1.90591
LACC_STAA8 Fu	448310 CI	KIPN_BPT4 Full	6022 Aden	1LTQ	2JGV	1RC8			Sc=5.86055, min distance = 2.07450
LACC_STAA8 Fu	4565 1h1r	CDK2_HUMAN Fu	6022 Aden	1GY3	2JGV	1H1R			Sc=6.27341, min distance = 1.86514
LACC_STAA8 Fu	5281672 m	PIM1_HUMAN Fu	33113 gam	1XR1	2JG1	2O63			Sc=6.41013, min distance = 1.90226
LACC_STAA8 Fu	5287844 i	GSK3B_HUMAN Fu	6022 Aden	1J1C	2JGV	1UV5			Sc=6.26713, min distance = 2.33894
LACC_STAA8 Fu	5288641 i	CDK2_HUMAN Fu	6022 Aden	1GY3	2JGV	1E9H			Sc=6.10665, min distance = 1.59981
LACC_STAA8 Fu	5326739 i	GSK3B_HUMAN Fu	6022 Aden	1J1C	2JGV	1Q41			Sc=6.28232, min distance = 1.31499
LACC_STAA8 Fu	5326976 1	CSK2A_MAIZE Fu	33113 gam	1LP4	2JG1	1ZOE			Sc=5.74475, min distance = 2.06035
LACC_STAA8 Fu	5326977 1	CSK2A_MAIZE Fu	33113 gam	1LP4	2JG1	1ZOG			Sc=5.66824, min distance = 2.04958
LACC_STAA8 Fu	5326978 1	CSK2A_MAIZE Fu	33113 gam	1LP4	2JG1	1ZOH			Sc=5.6912, min distance = 2.062247
LACC_STAA8 Fu	5327148 C	IPKA_RABIT Fu	6022 Aden	1JBP	2JGV	2ERZ			Sc=6.13569, min distance = 1.90947
LACC_STAA8 Fu	5957 Aden	BIOD_ECOLI Fu	6022 Aden	1DAD	2JGV	1A82		0.99	Sc=5.95286, min distance = 2.51635
LACC_STAA8 Fu	5957 Aden	DDL_THET8 Full	6022 Aden	2ZDH	2JGV	2ZDQ		0.99	Sc=6.42637, min distance = 2.13754
LACC_STAA8 Fu	5957 Aden	PSPF_ECOLI Fu	6022 Aden	2C98	2JGV	2C96		0.99	Sc=6.42158, min distance = 2.47842
LACC_STAA8 Fu	5957 Aden	RK_BOVIN Full=	6022 Aden	3C4Z	2JGV	3C4W		0.99	Sc=6.40327, min distance = 2.27407

LACC_STAA8 F	6031 Uric	DCK_HUMAN Ful	6022 Aden	1P5Z	2JGV	2ZIA			Sc=6.26962, min distance = 2.10065
LACC_STAA8 F	6082103 2	HSP82_YEAST F	6022 Aden	1AMW	2JGV	2BRC			Sc=6.33224, min distance = 2.04215
LACC_STAA8 F	6083 ader	ENTP2_RAT Ful	33113 gam	3CJA	2JG1	3CJ7		0.97	Sc=6.35183, min distance = 2.50779
LACC_STAA8 F	6083 ader	PIM1_HUMAN Fu	33113 gam	1XR1	2JG1	1YXU		0.97	Sc=6.3137, min distance = 2.016156
LACC_STAA8 F	60961 ade	SKY1_YEAST Fu	33113 gam	1Q99	2JG1	1Q97		0.93	Sc=6.23717, min distance = 1.80149
LACC_STAA8 F	6102784 A	PSPF_ECOLI Fu	6022 Aden	2C98	2JGV	2C99		0.99	Sc=6.43217, min distance = 2.23324
LACC_STAA8 F	6338561 C	MYS2_DICDI Fu	33113 gam	1MMN	2JG1	1D0X			Sc=6.1998, min distance = 2.216731
LACC_STAA8 F	6338561 C	MYS2_DICDI Fu	6022 Aden	1VOM	2JGV	1D0X			Sc=6.16509, min distance = 2.14571
LACC_STAA8 F	6338563 C	MYS2_DICDI Fu	6022 Aden	1VOM	2JGV	1D0Z			Sc=6.18879, min distance = 2.25259
LACC_STAA8 F	6338564 C	MYS2_DICDI Fu	33113 gam	1MMN	2JG1	1D1A			Sc=5.84253, min distance = 2.12116
LACC_STAA8 F	6338564 C	MYS2_DICDI Fu	6022 Aden	1VOM	2JGV	1D1A			Sc=6.27367, min distance = 1.54209
LACC_STAA8 F	6338565 D	MYS2_DICDI Fu	33113 gam	1MMN	2JG1	1D1B			Sc=5.90426, min distance = 2.06060
LACC_STAA8 F	6338566 C	MYS2_DICDI Fu	33113 gam	1MMN	2JG1	1D1C			Sc=6.23073, min distance = 2.06531
LACC_STAA8 F	6420140 2	CDK2_HUMAN Fu	6022 Aden	1GY3	2JGV	2C5Y			Sc=6.49774, min distance = 2.03766
LACC_STAA8 F	6540255 C	DAPK1_HUMAN F	33113 gam	1JKL	2JG1	1WVX			Sc=6.32694, min distance = 2.05196
LACC_STAA8 F	657072 RY	CDK2_HUMAN Fu	6022 Aden	1GY3	2JGV	2BHH			Sc=6.46666, min distance = 1.71258
LACC_STAA8 F	6804 guar	NDK_PYRHO Ful	6022 Aden	2DYA	2JGV	2DXF		0.8	Sc=5.86994, min distance = 2.20971
LACC_STAA8 F	91532 AMP	BIOD_ECOLI Fu	6022 Aden	1DAD	2JGV	1DAG		0.99	Sc=5.99702, min distance = 2.36931
LACC_STAA8 F	91532 AMP	F263_HUMAN Fu	6022 Aden	2AXN	2JGV	2DWP		0.99	Sc=6.42852, min distance = 2.00842
LACC_STAA8 F	91532 AMP	PURP_METJA Fu	6022 Aden	2R7N	2JGV	2R7K		0.99	Sc=5.99986, min distance = 1.33548
LACC_STAA8 F	91532 AMP	PURT_ECOLI Fu	33113 gam	1EYZ	2JG1	1KJI		0.99	Sc=6.49905, min distance = 1.65280
LACC_STAA8 F	9547890 I	CDK2_HUMAN Fu	6022 Aden	1GY3	2JGV	1W8C			Sc=6.26575, min distance = 2.15141
LACC_STAA8 F	9817550 V	CDK2_HUMAN Fu	6022 Aden	1GY3	2JGV	3BHV			Sc=6.27099, min distance = 1.73861
LACC_STAA8 F	9991833 S	CDK2_HUMAN Fu	6022 Aden	1GY3	2JGV	2R3H			Sc=6.15826, min distance = 1.14834
LAMB_ECOLI F	446000 CI	AMYP_PIG Full	79025 alp	1HX0	1AF6	1JFH		0.83	Sc=5.66724, min distance = 2.28421
LAMB_ECOLI F	446465 CI	AMYP_PIG Full	79025 alp	1HX0	1AF6	1JFH			Sc=5.7321, min distance = 2.215476
LAMB_SALTY F	16741013	CARP_YEAST Fu	64689 bet	1FQ4	2MPR	1FQ6			Sc=5.68635, min distance = 2.53626
LAMB_SALTY F	444809 Th	Q79G13_MYCTU	64689 bet	1UP0	2MPR	1UOZ		0.78	Sc=5.71526, min distance = 2.41833
LAMB_SALTY F	445037 D-	UGL_BACGL Ful	64689 bet	2FV0	2MPR	2FV1			Sc=5.70029, min distance = 1.84741
LAMB_SALTY F	445237 1,	AMY1_HORVU Fu	64689 bet	1RP9	2MPR	1P6W		0.77	Sc=5.71966, min distance = 2.11340
LAT_MYCTU Fu	3080614 C	JHD3A_HUMAN F	51 2-Oxop	2Q8C	2CJH	2OX0			Sc=5.64481, min distance = 2.33232
LCK_HUMAN Fu	11175137	KAPCA_BOVIN F	444345 1c	1STC	1QPD	2UW7	24.59		Sc=6.1003, min distance = 2.515886
LCK_HUMAN Fu	11387605	ABL1_HUMAN Fu	5291 Imat	2HYY	2PL0	2E2B	48.48	0.9	Sc=6.70216, min distance = 1.83387
LCK_HUMAN Fu	11992146	MAPK2_HUMAN F	444345 1c	2PZY	1QPD	2P3G	26.63		Sc=6.16042, min distance = 2.16957
LCK_HUMAN Fu	15942672	ABL1_MOUSE Fu	5291 Imat	1OPJ	2PL0	2HZN	48.3		Sc=6.34013, min distance = 2.15156
LCK_HUMAN Fu	16122608	PDPK1_HUMAN F	444345 1c	1OKY	1QPD	2PE1	26.62		Sc=6.2569, min distance = 2.620049
LCK_HUMAN Fu	16122643	CHK1_HUMAN Fu	444345 1c	1NVR	1QPD	2YWP	30.2		Sc=6.26572, min distance = 2.35525

LCK_HUMAN Fu 16214823	CDK2_HUMAN Fu 444345 1c 1AQ1	1QPD	2UZB	28.39		Sc=6.03659, min distance = 2.99934
LCK_HUMAN Fu 16214825	CDK2_HUMAN Fu 444345 1c 1AQ1	1QPD	2UZE	28.39		Sc=6.21515, min distance = 3.34761
LCK_HUMAN Fu 16214826	CDK2_HUMAN Fu 444345 1c 1AQ1	1QPD	2UZL	28.39		Sc=6.10259, min distance = 2.76578
LCK_HUMAN Fu 16214827	CDK2_HUMAN Fu 444345 1c 1AQ1	1QPD	2UZN	28.39		Sc=6.02884, min distance = 2.80111
LCK_HUMAN Fu 1707 1fvt	CDK2_HUMAN Fu 444345 1c 1AQ1	1QPD	1FVT	28.39		Sc=6.04966, min distance = 2.75641
LCK_HUMAN Fu 23653515	CDK2_HUMAN Fu 444345 1c 1AQ1	1QPD	2R3F	28.39		Sc=5.90981, min distance = 2.08252
LCK_HUMAN Fu 23727982	CDK2_HUMAN Fu 444345 1c 1AQ1	1QPD	3BHU	28.39		Sc=6.17847, min distance = 2.49079
LCK_HUMAN Fu 24851689	PIM1_HUMAN Fu 444345 1c 1YHS	1QPD	3CY2	34.33		Sc=5.99868, min distance = 2.60640
LCK_HUMAN Fu 24901723	CDK2_HUMAN Fu 444345 1c 1AQ1	1QPD	2W06	28.39		Sc=6.13076, min distance = 2.53321
LCK_HUMAN Fu 24905143	PK3CG_HUMAN Fu 444345 1c 1E8Z	1QPD	2V4L			Sc=6.14591, min distance = 2.78399
LCK_HUMAN Fu 24905144	PK3CG_HUMAN Fu 444345 1c 1E8Z	1QPD	3ENE			Sc=6.14761, min distance = 2.68929
LCK_HUMAN Fu 24905153	SRC_CHICK Full 5291 Imat 2OIQ	2PL0	3EN5	67.17		Sc=6.15371, min distance = 2.16532
LCK_HUMAN Fu 24916751	CDK2_HUMAN Fu 444345 1c 1AQ1	1QPD	3DOG	28.39		Sc=6.29939, min distance = 2.25005
LCK_HUMAN Fu 25011744	SRC_CHICK Full 5291 Imat 2OIQ	2PL0	3EL7	67.17		Sc=6.58102, min distance = 2.19412
LCK_HUMAN Fu 25011745	SRC_CHICK Full 5291 Imat 2OIQ	2PL0	3EL8	67.17		Sc=6.53966, min distance = 2.50120
LCK_HUMAN Fu 25021197	CDK2_HUMAN Fu 444345 1c 1AQ1	1QPD	3EOC	28.39		Sc=6.17965, min distance = 2.69058
LCK_HUMAN Fu 3973 nche	PIM1_HUMAN Fu 444345 1c 1YHS	1QPD	1YI3	34.33		Sc=5.77616, min distance = 2.56162
LCK_HUMAN Fu 4327 CID4	ABL1_MOUSE Fu 5291 Imat 1OPJ	2PL0	1FPU	48.3	0.93	Sc=6.53574, min distance = 2.34773
LCK_HUMAN Fu 4369136 C	CDK2_HUMAN Fu 444345 1c 1AQ1	1QPD	1DM2	28.39		Sc=6.14531, min distance = 2.97528
LCK_HUMAN Fu 445966 O6	CDK2_HUMAN Fu 444345 1c 1AQ1	1QPD	1H0V	28.39		Sc=6.05197, min distance = 2.77012
LCK_HUMAN Fu 446704 LS	CDK2_HUMAN Fu 444345 1c 1AQ1	1QPD	1KE6	28.39		Sc=6.27685, min distance = 2.51661
LCK_HUMAN Fu 447654 CI	CDK2_HUMAN Fu 444345 1c 1AQ1	1QPD	1OIT	28.39		Sc=6.10119, min distance = 2.62495
LCK_HUMAN Fu 447967 CI	CDK2_HUMAN Fu 444345 1c 1AQ1	1QPD	1PYE	28.39		Sc=6.29892, min distance = 2.89432
LCK_HUMAN Fu 448043 2g	KAPCA_BOVIN Fu 444345 1c 1STC	1QPD	1Q8U	24.59		Sc=5.94415, min distance = 2.70329
LCK_HUMAN Fu 449087 1v	CDK2_HUMAN Fu 444345 1c 1AQ1	1QPD	1VYW	28.39		Sc=6.2448, min distance = 2.171908
LCK_HUMAN Fu 456214 pu	KS6A1_HUMAN Fu 444345 1c 2Z7R	1QPD	2Z7S			Sc=6.36686, min distance = 2.06808
LCK_HUMAN Fu 5281680 Q	PIM1_HUMAN Fu 444345 1c 1YHS	1QPD	2O64	34.33		Sc=6.36523, min distance = 2.79849
LCK_HUMAN Fu 5287845 N	CDK2_HUMAN Fu 444345 1c 1AQ1	1QPD	2BHE	28.39		Sc=6.13713, min distance = 2.48210
LCK_HUMAN Fu 5288641 i	CDK2_HUMAN Fu 444345 1c 1AQ1	1QPD	1E9H	28.39		Sc=6.20471, min distance = 2.60007
LCK_HUMAN Fu 5288708 1	CDK2_HUMAN Fu 444345 1c 1AQ1	1QPD	1KE5	28.39		Sc=6.06808, min distance = 2.62102
LCK_HUMAN Fu 5288710 C	CDK2_HUMAN Fu 444345 1c 1AQ1	1QPD	1KE7	28.39		Sc=6.28584, min distance = 2.57209
LCK_HUMAN Fu 5288712 1	CDK2_HUMAN Fu 444345 1c 1AQ1	1QPD	1KE9	28.39		Sc=6.24811, min distance = 2.78499
LCK_HUMAN Fu 5311104 2	ABL1_HUMAN Fu 5291 Imat 2HYY	2PL0	2HZI	48.48	0.76	Sc=6.26096, min distance = 2.00194
LCK_HUMAN Fu 5331010 c	CDK2_HUMAN Fu 444345 1c 1AQ1	1QPD	2BKZ	28.39		Sc=6.17309, min distance = 2.55985
LCK_HUMAN Fu 5687 1di8	CDK2_HUMAN Fu 444345 1c 1AQ1	1QPD	1DI8	28.39		Sc=6.11253, min distance = 2.66550
LCK_HUMAN Fu 60961 ade	PIM1_HUMAN Fu 444345 1c 1YHS	1QPD	1YI4	34.33		Sc=6.12246, min distance = 2.54764
LCK_HUMAN Fu 6102670	CDK2_HUMAN Fu 444345 1c 1AQ1	1QPD	1YKR	28.39		Sc=6.21387, min distance = 2.23052

LCK_HUMAN Fu	6420138 2	CDK2_HUMAN Fu	444345 1c	1AQ1	1QPD	2UUE	28.39		Sc=6.20376, min distance = 2.71409
LCK_HUMAN Fu	6420139 C	CDK2_HUMAN Fu	444345 1c	1AQ1	1QPD	2C5V	28.39		Sc=6.07124, min distance = 2.65310
LCK_HUMAN Fu	644241 Ni	ABL1_HUMAN Fu	5291 Imat	2HYY	2PL0	3CS9	48.48	0.9	Sc=6.68482, min distance = 2.39179
LCK_HUMAN Fu	6539118 g	CDK2_HUMAN Fu	444345 1c	1AQ1	1QPD	1FVV	28.39		Sc=6.39429, min distance = 2.72835
LCK_HUMAN Fu	656971 CI	CDK2_HUMAN Fu	444345 1c	1AQ1	1QPD	1Y8Y	28.39		Sc=5.87547, min distance = 2.40314
LCK_HUMAN Fu	6852207 M	KAPCA_BOVIN Fu	444345 1c	1STC	1QPD	2GNI	24.59		Sc=5.91836, min distance = 2.44589
LCK_HUMAN Fu	9817550 V	CDK2_HUMAN Fu	444345 1c	1AQ1	1QPD	3BHV	28.39		Sc=6.22995, min distance = 2.29169
LCMT1_YEAST I	16741210	MTTA_THEAQ Fu	439155 Ad	1AQI	2OB2	2JG3			Sc=6.49547, min distance = 1.56361
LCMT1_YEAST I	188380 Ac	MCES_ENCCU Fu	439155 Ad	1RI1	2OB2	1Z3C		0.91	Sc=6.59563, min distance = 2.39239
LCMT1_YEAST I	445762 5'	MTTA_THEAQ Fu	34756 Acy	2ADM	1RJD	2IH2		0.93	Sc=6.40321, min distance = 1.96405
LCMT1_YEAST I	445971 CI	COMT_RAT Full=	34756 Acy	2CL5	1RJD	1H1D		0.92	Sc=6.57247, min distance = 2.35278
LCMT1_YEAST I	446535 CI	HNMT_HUMAN Fu	439155 Ad	2AOT	2OB2	1JQE		0.95	Sc=6.5443, min distance = 2.492757
LCMT1_YEAST I	60961 ade	PIMT_PYRFU Fu	439155 Ad	1JG1	2OB2	1JG2		0.84	Sc=6.32806, min distance = 2.49841
LCMT1_YEAST I	65482 sir	ERM_BACSU Ful	439155 Ad	1QAN	2OB2	1QAQ		0.88	Sc=6.57983, min distance = 2.38142
LDH2_BIFLO Fu	11987786	LDH_PLAFD Ful	5893 nadi	1T2D	1LLD	1T2E		0.77	Sc=6.27681, min distance = 2.37900
LDH2_BIFLO Fu	16129587	G3PA_SPIOL Fu	5893 nadi	1NBO	1LLD	2PKR			Sc=6.77501, min distance = 0.79462
LDH2_BIFLO Fu	165230 1a	G6PD_LEUME Fu	5893 nadi	1H94	1LLD	2DPG			Sc=5.97148, min distance = 1.78384
LDH2_BIFLO Fu	439153 Di	ADH1B_HUMAN Fu	5893 nadi	1U3U	1LLD	1DEH		0.79	Sc=6.72624, min distance = 2.29882
LDH2_BIFLO Fu	439153 Di	CTBP1_RAT Ful	5893 nadi	1HKU	1LLD	1HL3		0.79	Sc=6.73313, min distance = 2.58236
LDH2_BIFLO Fu	440141 9i	G6PD_LEUME Fu	5893 nadi	1H94	1LLD	1E7Y			Sc=6.3806, min distance = 2.225962
LDH2_BIFLO Fu	440516 CI	MDH_THETH Ful	5893 nadi	1BMD	1LLD	1BDM		0.79	Sc=6.7371, min distance = 2.389540
LDH2_BIFLO Fu	445794 AD	ADHX_HUMAN Fu	5893 nadi	2FZW	1LLD	2FZE			Sc=6.57547, min distance = 2.48741
LDH2_BIFLO Fu	446288 NA	G3P_PALVE Ful	5893 nadi	1DSS	1LLD	1IHX			Sc=6.79354, min distance = 0.91006
LDH2_BIFLO Fu	6083 ader	NADE_ECOLI Fu	5893 nadi	1WXH	1LLD	1WXI			Sc=6.31141, min distance = 1.20695
LDHA_HUMAN Fu	11987786	LDH_PLAFD Ful	5893 nadi	1T2D	1I10	1T2E		0.77	Sc=6.31435, min distance = 2.53212
LDHA_HUMAN Fu	439153 Di	ADH1E_HORSE Fu	5893 nadi	1MGO	1I10	2JHF		0.79	Sc=6.76564, min distance = 2.45741
LDHA_HUMAN Fu	439153 Di	CTBP1_RAT Ful	5893 nadi	1HKU	1I10	1HL3		0.79	Sc=6.77299, min distance = 1.97608
LDHA_HUMAN Fu	445794 AD	ADHX_HUMAN Fu	5893 nadi	2FZW	1I10	2FZE			Sc=6.62023, min distance = 2.53947
LDHA_HUMAN Fu	445794 AD	G3P_PALVE Ful	5893 nadi	1DSS	1I10	1IHY			Sc=6.6649, min distance = 1.160934
LDHA_HUMAN Fu	5957 Ader	MAOM_HUMAN Fu	5893 nadi	1PJ3	1I10	1GZ4			Sc=6.45189, min distance = 1.87995
LDHA_PIG Ful	11987786	LDH_PLAFD Ful	5893 nadi	1T2D	9LDB	1T2E	32.47	0.77	Sc=6.75916, min distance = 2.50723
LDHA_PIG Ful	123927 A3	Q9GT92_CRYPV I	5893 nadi	2FM3	9LDB	2EWD	31.25	0.97	Sc=6.77011, min distance = 2.49472
LDHA_PIG Ful	439153 Di	ADH1B_HUMAN Fu	5893 nadi	1U3U	9LDB	1DEH		0.79	Sc=6.76491, min distance = 2.44808
LDHA_PIG Ful	439153 Di	ADH1E_HORSE Fu	5893 nadi	1MGO	9LDB	2JHF		0.79	Sc=6.75642, min distance = 2.63699
LDHA_PIG Ful	439153 Di	GALE_ECOLI Fu	5893 nadi	1UDC	9LDB	1UDB		0.79	Sc=6.76739, min distance = 1.98580
LDHA_PIG Ful	446288 NA	G3P_PALVE Ful	5893 nadi	1DSS	9LDB	1IHX			Sc=6.75528, min distance = 1.56845
LDHA_PIG Ful	6102709 A	LDH_PLAFD Ful	5893 nadi	1T2D	9LDB	2A94	32.47	0.88	Sc=6.75628, min distance = 2.41203

LDHA_PIG Ful:	6102710 A	Q4PRK9_PLAVI I	5893 nadi	2A92	9LDB	2AA3	0.95	Sc=6.76901, min distance = 2.40627
LDHA_SQUAC Fu	123927 A3	DAPB_ECOLI Fu:	5893 nadi	1DRU	1LDM	1DRV	0.97	Sc=6.76003, min distance = 2.30257
LDHA_SQUAC Fu	123927 A3	Q9GT92_CRYPV I	5893 nadi	2FM3	1LDM	2EWD	0.97	Sc=6.75911, min distance = 2.72747
LDHA_SQUAC Fu	439153 Di	ADH1B_HUMAN Fu	5893 nadi	1U3U	1LDM	1DEH	0.79	Sc=6.75682, min distance = 2.72618
LDHA_SQUAC Fu	439153 Di	ADH1E_HORSE Fu	5893 nadi	1MGO	1LDM	2JHF	0.79	Sc=6.75388, min distance = 2.62696
LDHA_SQUAC Fu	439153 Di	GALE_ECOLI Fu:	5893 nadi	1UDC	1LDM	1UDB	0.79	Sc=6.76187, min distance = 2.04198
LDHA_SQUAC Fu	440516 CI	MDH_THETH Ful:	5893 nadi	1BMD	1LDM	1BDM	0.79	Sc=6.76946, min distance = 2.51632
LDHA_SQUAC Fu	445794 AD	ADHX_HUMAN Fu:	5893 nadi	2FZW	1LDM	2FZE		Sc=6.60546, min distance = 2.48022
LDHA_SQUAC Fu	448573 FI	CASP7_HUMAN Fu	311 citri	2QL9	8LDH	1SHL		Sc=5.92755, min distance = 0.23559
LDHA_SQUAC Fu	51 2-Oxop	SERA_ECOLI Fu:	311 citri	2P9E	8LDH	1YBA	0.76	Sc=5.86551, min distance = 1.00069
LDHA_SQUAC Fu	6102710 A	Q4PRK9_PLAVI I	5893 nadi	2A92	1LDM	2AA3	0.95	Sc=6.76678, min distance = 2.44582
LDHB_HUMAN Fu	11987786	LDH_PLAFD Ful:	5893 nadi	1T2D	1I0Z	1T2E	0.77	Sc=6.32424, min distance = 2.41601
LDHB_HUMAN Fu	123927 A3	DAPB_ECOLI Fu:	5893 nadi	1DRU	1I0Z	1DRV	0.97	Sc=6.75037, min distance = 2.23318
LDHB_HUMAN Fu	439153 Di	ADH1E_HORSE Fu	5893 nadi	1MGO	1I0Z	2JHF	0.79	Sc=6.75388, min distance = 2.52771
LDHB_HUMAN Fu	439153 Di	CTBP1_RAT Ful:	5893 nadi	1HKU	1I0Z	1HL3	0.79	Sc=6.76165, min distance = 2.11209
LDHB_HUMAN Fu	440516 CI	MDH_THETH Ful:	5893 nadi	1BMD	1I0Z	1BDM	0.79	Sc=6.77407, min distance = 2.59638
LDHB_HUMAN Fu	445794 AD	ADHX_HUMAN Fu:	5893 nadi	2FZW	1I0Z	2FZE		Sc=6.61107, min distance = 2.49011
LDHB_HUMAN Fu	5957 Aden	MAOM_HUMAN Fu:	5893 nadi	1PJ3	1I0Z	1GZ4		Sc=6.43911, min distance = 2.08475
LDHB_HUMAN Fu	6083 aden	NADE_ECOLI Fu:	5893 nadi	1WXH	1I0Z	1WXI		Sc=6.29257, min distance = 2.06229
LDHD_LACDA Fu	11987786	LDH_PLAFD Ful:	5893 nadi	1T2D	1J49	1T2E	0.77	Sc=6.3231, min distance = 2.320828
LDHD_LACDA Fu	439153 Di	ADH1B_HUMAN Fu	5893 nadi	1U3U	1J49	1DEH	0.79	Sc=6.33161, min distance = 2.02904
LDHD_LACDA Fu	439153 Di	ADH1E_HORSE Fu	5893 nadi	1MGO	1J49	2JHF	0.79	Sc=6.32493, min distance = 1.94251
LDHD_LACDA Fu	439153 Di	CTBP1_RAT Ful:	5893 nadi	1HKU	1J49	1HL3	0.79	Sc=6.31911, min distance = 2.03832
LDHD_LACDA Fu	445794 AD	ADHX_HUMAN Fu:	5893 nadi	2FZW	1J49	2FZE		Sc=6.17004, min distance = 0.97633
LDH_LACPE Fu:	11987786	LDH_PLAFD Ful:	5893 nadi	1T2D	1EZ4	1T2E	0.77	Sc=6.30426, min distance = 2.12947
LDH_LACPE Fu:	123927 A3	DAPB_ECOLI Fu:	5893 nadi	1DRU	1EZ4	1DRV	0.97	Sc=6.31176, min distance = 2.11905
LDH_LACPE Fu:	4369002 C	LDHA_PIG Full=	5893 nadi	9LDB	1EZ4	9LDT	0.77	Sc=6.75715, min distance = 1.82297
LDH_LACPE Fu:	5957 Aden	MAOM_HUMAN Fu:	5893 nadi	1PJ3	1EZ4	1GZ4		Sc=6.47558, min distance = 1.83047
LDH_LACPE Fu:	6022 Aden	UGDH_HUMAN Fu:	5893 nadi	2Q3E	1EZ4	2QG4		Sc=6.76665, min distance = 1.32483
LDH_LACPE Fu:	6083 aden	NADE_ECOLI Fu:	5893 nadi	1WXH	1EZ4	1WXI		Sc=6.29766, min distance = 2.10162
LDH_PLABA Fu:	11987786	LDH_PLAFD Ful:	5893 nadi	1T2D	1OC4	1T2E	0.77	Sc=6.78446, min distance = 2.07316
LDH_PLABA Fu:	4369002 C	LDHA_PIG Full=	5893 nadi	9LDB	1OC4	9LDT	0.77	Sc=6.78198, min distance = 2.02457
LDH_PLABA Fu:	439153 Di	ADH1E_HORSE Fu	5893 nadi	1MGO	1OC4	2JHF	0.79	Sc=6.78013, min distance = 2.20500
LDH_PLABA Fu:	440516 CI	MDH_THETH Ful:	5893 nadi	1BMD	1OC4	1BDM	0.79	Sc=6.78508, min distance = 2.28820
LDH_PLABA Fu:	445794 AD	ADHX_HUMAN Fu:	5893 nadi	2FZW	1OC4	2FZE		Sc=6.63161, min distance = 1.86554
LDH_PLABA Fu:	6083 aden	NADE_ECOLI Fu:	5893 nadi	1WXH	1OC4	1WXI		Sc=6.36251, min distance = 2.18171
LDH_PLABA Fu:	6102710 A	Q4PRK9_PLAVI I	5893 nadi	2A92	1OC4	2AA3	0.95	Sc=6.77941, min distance = 2.17248

LDH_PLAFD Fu	123927 A3	Q9GT92_CRYPV I	5893 nadi	2FM3	1T2D	2EWD	44.04	0.97	Sc=6.79644, min distance = 1.96789
LDH_PLAFD Fu	16129587	G3PA_SPIOL Fu	5893 nadi	1NBO	1T2D	2PKR			Sc=6.08884, min distance = 1.838098
LDH_PLAFD Fu	4369002 C	LDHA_PIG Full	5893 nadi	9LDB	1T2D	9LDT	32.47	0.77	Sc=6.79107, min distance = 2.110175
LDH_PLAFD Fu	439153 Di	ADH1B_HUMAN Fu	5893 nadi	1U3U	1T2D	1DEH		0.79	Sc=6.79107, min distance = 2.095962
LDH_PLAFD Fu	440516 CI	MDH_THETH Ful	5893 nadi	1BMD	1T2D	1BDM		0.79	Sc=6.79937, min distance = 2.398913
LDH_PLAFD Fu	445794 AD	ADHX_HUMAN Fu	5893 nadi	2FZW	1T2D	2FZE			Sc=6.64138, min distance = 2.395065
LDH_PLAFD Fu	6102710 A	Q4PRK9_PLAVI I	5893 nadi	2A92	1T2D	2AA3		0.95	Sc=6.79776, min distance = 2.655656
LDH_THET8 Fu	123927 A3	DAPB_ECOLI Fu	5893 nadi	1DRU	2V7P	1DRV		0.97	Sc=6.32603, min distance = 2.008254
LDH_THET8 Fu	439153 Di	ADH1B_HUMAN Fu	5893 nadi	1U3U	2V7P	1DEH		0.79	Sc=6.77054, min distance = 2.111386
LDH_THET8 Fu	439153 Di	ADH1E_HORSE Fu	5893 nadi	1MGO	2V7P	2JHF		0.79	Sc=6.76107, min distance = 2.548358
LDH_THET8 Fu	439153 Di	GALE_ECOLI Fu	5893 nadi	1UDC	2V7P	1UDB		0.79	Sc=6.76878, min distance = 1.924980
LDH_THET8 Fu	444959 CI	STP22_YEAST Fu	78165 MES	1UZX	2V6M	2F6M			Sc=5.63429, min distance = 40.76224
LDH_THET8 Fu	446288 NA	G3P_PALVE Ful	5893 nadi	1DSS	2V7P	1IHX			Sc=6.76477, min distance = 2.014659
LDH_THET8 Fu	6083 ader	NADE_ECOLI Fu	5893 nadi	1WXH	2V7P	1WXI			Sc=6.3249, min distance = 1.8615181
LDH_TOXGO Fu	11987786	LDH_PLAFD Ful	5893 nadi	1T2D	1SOW	1T2E		0.77	Sc=6.33328, min distance = 2.752138
LDH_TOXGO Fu	16129587	G3PA_SPIOL Fu	5893 nadi	1NBO	1SOW	2PKR			Sc=5.91173, min distance = 1.921212
LDH_TOXGO Fu	4369002 C	LDHA_PIG Full	5893 nadi	9LDB	1SOW	9LDT		0.77	Sc=6.77212, min distance = 2.395030
LDH_TOXGO Fu	439153 Di	CTBP1_RAT Ful	5893 nadi	1HKU	1SOW	1HL3		0.79	Sc=6.77775, min distance = 2.106525
LDH_TOXGO Fu	446288 NA	G3P_PALVE Ful	5893 nadi	1DSS	1SOW	1IHX			Sc=6.77275, min distance = 1.966227
LDH_TOXGO Fu	6083 ader	NADE_ECOLI Fu	5893 nadi	1WXH	1SOW	1WXI			Sc=5.85406, min distance = 1.933922
LDOX_ARATH Fu	152217 2,	DYR_CANAL Ful	78165 MES	1M79	1GP6	1M78			Sc=5.71122, min distance = 2.481339
LDOX_ARATH Fu	23831 HEE	GSTP1_HUMAN Fu	78165 MES	2A2R	1GP6	1PGT		0.81	Sc=5.61773, min distance = 1.319853
LDOX_ARATH Fu	5318532 i	IDHP_YEAST Fu	51 2-Oxop	2QFY	1GP4	2QFW			Sc=5.97755, min distance = 0.913676
LEC1_CRAMO Fu	151504 D-	CONA_CANEN Fu	101798 Me	5CNA	1MVQ	1I3H			Sc=5.73207, min distance = 2.244599
LEC1_CRAMO Fu	185698 al	LEC1_LATOC Fu	101798 Me	1LOB	1MVQ	1LOG		0.94	Sc=5.71748, min distance = 2.725344
LEC1_CRAMO Fu	185698 al	LECB_LATOC Fu	101798 Me	1LOB	1MVQ	1LOG		0.94	Sc=5.71337, min distance = 2.726028
LEC1_CRAMO Fu	185698 al	Q9HYN5_PSEAE I	101798 Me	2JDN	1MVQ	1OVS		0.94	Sc=5.75684, min distance = 2.035972
LEC1_CRAMO Fu	439554 fu	Q9HYN5_PSEAE I	101798 Me	2JDN	1MVQ	1UZV		0.96	Sc=5.72788, min distance = 1.841022
LEC1_CRAMO Fu	445948 D-	Q9HYN5_PSEAE I	101798 Me	2JDN	1MVQ	1OXC			Sc=5.74199, min distance = 1.890217
LEC1_ULEEU Fu	24310 fru	Q9HYN5_PSEAE I	446578 1r	2JDP	1JXN	1OVP		0.9	Sc=5.79509, min distance = 2.570981
LEC1_ULEEU Fu	445948 D-	Q9HYN5_PSEAE I	446578 1r	2JDP	1JXN	1OXC			Sc=5.73598, min distance = 2.098484
LEC1_ULEEU Fu	447053 CI	Q9HYN5_PSEAE I	446578 1r	2JDP	1JXN	1W8H		0.81	Sc=5.62651, min distance = 2.444835
LEC2_LATOC Fu	13455857	IGKC_MOUSE Fu	24139 ace	3BZ4	1LGC	3C6S			Sc=5.6348, min distance = 1.2957353
LECA_ARTIN Fu	151504 D-	CONA_CANEN Fu	101798 Me	5CNA	1WS5	1I3H			Sc=5.74009, min distance = 2.399981
LECA_ARTIN Fu	151504 D-	CONA_CANEN Fu	64947 alp	1GIC	1WS4	1I3H			Sc=5.75684, min distance = 2.547428
LECA_ARTIN Fu	185698 al	LECB_LATOC Fu	101798 Me	1LOB	1WS5	1LOG		0.94	Sc=5.74199, min distance = 2.391612
LECA_DIOGR Fu	151504 D-	CVN_NOSEL Ful	185698 al	2RDK	1DGL	2PYS			Sc=5.7552, min distance = 2.5703252

LECA_DIOGR Fu	445184 1,	Q6PYX1_HUMAN I	185698 al	1ZLS	1DGL	1ZLW		0.89	Sc=5.74599, min distance = 2.40696(
LECA_MACPO Fu	446578 1r	Q9HYN5_PSEAE I	439353 be	1W8F	1JOT	2JDP		0.94	Sc=5.63829, min distance = 1.56310
LECB_LATOC Fu	70815 CAE	Q6PYX1_HUMAN I	439554 fu	1S3K	1LGC	1N0X			Sc=5.60726, min distance = 2.131938
LECF_ALEAU Fu	446807 D-	MBL1_RAT Full=	444863 is	1KWX	1OFZ	4KMB		0.88	Sc=5.76476, min distance = 1.25452
LECG_ARAHY Fu	16741014	CARP_YEAST Fu	82313 1rd	1FMU	1CIW	1FQ7			Sc=5.77268, min distance = 0.88026
LECG_ARAHY Fu	441478 gl	CSLB_PEDHE Fu	440552 1b	1DBO	2TEP	1OFM			Sc=5.79938, min distance = 2.02144
LECG_ARAHY Fu	444863 is	MBL1_RAT Full=	94214 Met	1AFA	1QF3	1KWX		0.96	Sc=5.72381, min distance = 2.07022
LECG_ARAHY Fu	445037 D-	CSLB_PEDHE Fu	440552 1b	1DBO	2TEP	1OFL			Sc=5.71147, min distance = 1.76603
LECG_ARAHY Fu	446996 GA	Q9HYN5_PSEAE I	439353 be	1W8F	1V6I	1W8H		0.89	Sc=5.77651, min distance = 1.92189(
LECG_CROAT Fu	446996 GA	Q9HYN5_PSEAE I	439353 be	1W8F	1JZN	1W8H		0.89	Sc=5.80476, min distance = 1.65919
LEC_BOWMI Fu	101798 Me	LEC1_LATOC Fu	185698 al	1LOG	2FMD	1LOB		0.94	Sc=5.82241, min distance = 2.25963(
LEC_BOWMI Fu	101798 Me	LEC_GALNI Full	185698 al	1JPC	2FMD	1MSA		0.94	Sc=5.97106, min distance = 0.90768
LEC_BOWMI Fu	151504 D-	CVN_NOSEL Full	185698 al	2RDK	2FMD	2PYS			Sc=5.76391, min distance = 2.357418
LEC_BOWMI Fu	16740986	MYRA_SINAL Fu	185698 al	1E4M	2FMD	1E70			Sc=5.73005, min distance = 2.32039
LEC_BOWMI Fu	16741013	CARP_YEAST Fu	185698 al	1DPJ	2FMD	1FQ6			Sc=5.63826, min distance = 2.15201
LEC_BOWMI Fu	445184 1,	Q6PYX1_HUMAN I	185698 al	1ZLS	2FMD	1ZLW		0.89	Sc=5.75163, min distance = 1.99830
LEC_BOWMI Fu	446972 Mu	LEC1_LATOC Fu	185698 al	1LOG	2FMD	1LOD			Sc=6.12109, min distance = 1.42905
LEC_BOWMI Fu	446972 Mu	LECB_LATOC Fu	185698 al	1LOG	2FMD	1LOD			Sc=6.12109, min distance = 1.45720
LEC_BOWMI Fu	5289587 D	LECA_DIOGR Fu	185698 al	1DGL	2FMD	2JEC			Sc=6.24206, min distance = 2.08309
LEC_ERYCG Fu	446578 1r	Q8XXK6_RALSO I	439554 fu	2BS6	1GZ9	2BT9		0.96	Sc=5.7213, min distance = 1.734688
LEC_ERYCO Fu	446996 GA	Q9HYN5_PSEAE I	439353 be	1W8F	1AX1	1W8H		0.89	Sc=5.79423, min distance = 1.47660
LEC_ERYCO Fu	448882 NC	LECA_ARTIN Fu	84265 1ax	1M26	1AX0	1UGX		0.96	Sc=6.00168, min distance = 2.64665
LEC_GALNI Fu	444885 O-	NRAM_I56A2 Fu	185698 al	1V0Z	1JPC	1W1X			Sc=5.79912, min distance = 2.37265
LEC_GALNI Fu	82313 1rd	MBL1_RAT Full=	101798 Me	1KWU	1MSA	1KWV			Sc=5.87924, min distance = 1.69575
LEC_LENCU Fu	193758 Va	AMY1_HUMAN Fu	79025 alp	3DHP	1LES	1MFU			Sc=5.74414, min distance = 2.01754
LEC_SOYBN Fu	18950 D-m	TRFL_BOVIN Fu	444205 CI	2G93	1SBD	2B65			Sc=5.8821, min distance = 0.72690
LEC_SOYBN Fu	24139 ace	LYSC_HUMAN Fu	439353 be	1REZ	1SBD	1LZR			Sc=5.91557, min distance = 2.10574
LEC_SOYBN Fu	446996 GA	Q9HYN5_PSEAE I	439353 be	1W8F	1SBD	1W8H		0.89	Sc=5.76391, min distance = 1.33884
LEC_SOYBN Fu	449462 2-	LEG7_HUMAN Fu	439353 be	4GAL	1SBD	3GAL			Sc=5.77565, min distance = 2.44988
LED2_PHYAM Fu	656941 1u	FIMH_ECOLI Fu	24139 ace	2VCO	1ULM	1UWF			Sc=5.97903, min distance = 1.57453
LEG1_BOVIN Fu	448896 NC	CGL2_COPCI Fu	439353 be	1ULD	1SLT	1ULG			Sc=5.94347, min distance = 1.57280
LEG1_CONMY Fu	24139 ace	LYSC_HUMAN Fu	439353 be	1REZ	1C1L	1LZR			Sc=5.91971, min distance = 2.50828
LEG1_CONMY Fu	440552 1b	LECA_ARTIN Fu	439353 be	1UGX	1C1L	1UH1			Sc=5.91971, min distance = 1.52692
LEG1_CONMY Fu	444635 O3	ATLE_AGRAE Fu	439353 be	1WW6	1C1L	1WW5		0.9	Sc=5.66296, min distance = 2.43405(
LEG1_CONMY Fu	444635 O3	MBL1_RAT Full=	439353 be	2KMB	1C1L	3KMB		0.9	Sc=5.71681, min distance = 2.00221
LEG1_CONMY Fu	446996 GA	Q9HYN5_PSEAE I	439353 be	1W8F	1C1L	1W8H		0.89	Sc=5.74199, min distance = 2.28726
LEG1_CONMY Fu	449462 2-	LEG7_HUMAN Fu	439353 be	4GAL	1C1L	3GAL			Sc=5.81167, min distance = 2.70426

LEG1_CONMY F	657081 CI	LEG3_HUMAN Fu	439353 be	1KJL	1C1L	1KJR		Sc=5.61169, min distance = 2.11891
LEU3_THET8 F	6102710 A	Q4PRK9_PLAVI	5893 nadi	2A92	1HEX	2AA3	0.95	Sc=5.90077, min distance = 2.07918
LGB2_LUPLU F	11957363	HBB_HUMAN Ful	444098 HE	1J40	1GDJ	1RQA	0.77	Sc=6.74445, min distance = 2.04512
LGB2_LUPLU F	11957385	MYG_PHYCA Ful	444098 HE	1A6M	1GDJ	2CMM		Sc=6.25033, min distance = 2.24671
LGB2_LUPLU F	11957385	MYG_PHYCA Ful	444522 HE	1VXD	2GDM	2CMM		Sc=6.07625, min distance = 2.01809
LGB2_LUPLU F	11970222	CCPR_YEAST Fu	444098 HE	2EUT	1GDJ	1BJ9	0.77	Sc=6.32548, min distance = 2.36140
LHA4_RHOAC F	155448 B-	GLPG_ECOLI Fu	444279 1s	2IRV	2FKW	3B44		Sc=5.7078, min distance = 1.190351
LHA4_RHOAC F	7054 Isat	AOFB_HUMAN Fu	444279 1s	1OJD	2FKW	1OJA		Sc=5.63155, min distance = 2.27899
LIG2_PHACH F	11957385	MYG_PHYCA Ful	444124 HE	1U7R	1LLP	2CMM		Sc=6.06631, min distance = 2.49960
LIG4_PHACH F	11957330	CCPR_YEAST Fu	444098 HE	2EUT	1QPA	1BEQ	0.81	Sc=6.69862, min distance = 2.28582
LIG4_PHACH F	11957353	HBA_HORSE Ful	444098 HE	2D5X	1QPA	1IWH	0.83	Sc=6.69153, min distance = 2.13972
LIG4_PHACH F	11957365	CCPR_YEAST Fu	444098 HE	2EUT	1QPA	1S73	0.8	Sc=6.7512, min distance = 2.075053
LIG4_PHACH F	11957385	MYG_PHYCA Ful	444098 HE	1A6M	1QPA	2CMM		Sc=6.18658, min distance = 2.50624
LIG4_PHACH F	11970219	CCPR_YEAST Fu	444098 HE	2EUT	1QPA	1BEM	0.83	Sc=6.69905, min distance = 2.31299
LIG4_PHACH F	11970242	CCPR_YEAST Fu	444098 HE	2EUT	1QPA	1CPE	0.83	Sc=6.69822, min distance = 2.38795
LIG4_PHACH F	126994 CI	CCPR_YEAST Fu	444098 HE	2EUT	1QPA	1Z53	0.77	Sc=6.69572, min distance = 2.87623
LIG4_PHACH F	447168 ZE	CCPR_YEAST Fu	444098 HE	2EUT	1QPA	1ML2	0.82	Sc=6.74602, min distance = 2.28419
LIGO1_HUMAN	445692 CI	CARP_YEAST Fu	439680 be	1DPJ	2ID5	1FQ5		Sc=5.62898, min distance = 1.42646
LIPG_CANFA F	446816 1k	MK14_HUMAN Fu	62852 B-C	2FST	1K8Q	1KV1		Sc=6.19837, min distance = 1.94730
LIPG_CANFA F	656949 Ur	MK14_HUMAN Fu	62852 B-C	2FST	1K8Q	1W82		Sc=6.34328, min distance = 2.05915
LIVG_METJA F	11957393	FAK1_HUMAN Fu	6022 Aden	1MP8	1G6H	2ETM		Sc=6.01653, min distance = 2.42502
LIVG_METJA F	11957417	CDK2_HUMAN Fu	6022 Aden	1GY3	1G6H	2I40		Sc=6.13375, min distance = 1.91279
LIVG_METJA F	1540 1h1c	CDK2_HUMAN Fu	6022 Aden	1GY3	1G6H	1H1Q		Sc=5.78622, min distance = 2.24015
LIVG_METJA F	16214823	CDK2_HUMAN Fu	6022 Aden	1GY3	1G6H	2UZB		Sc=5.9953, min distance = 2.926552
LIVG_METJA F	16214826	CDK2_HUMAN Fu	6022 Aden	1GY3	1G6H	2UZL		Sc=6.04282, min distance = 2.03354
LIVG_METJA F	23653515	CDK2_HUMAN Fu	6022 Aden	1GY3	1G6H	2R3F		Sc=5.69563, min distance = 2.23544
LIVG_METJA F	23653516	CDK2_HUMAN Fu	6022 Aden	1GY3	1G6H	2R3G		Sc=5.85005, min distance = 2.49442
LIVG_METJA F	23653518	CDK2_HUMAN Fu	6022 Aden	1GY3	1G6H	2R3J		Sc=5.89231, min distance = 2.08911
LIVG_METJA F	23653519	CDK2_HUMAN Fu	6022 Aden	1GY3	1G6H	2R3K		Sc=5.87945, min distance = 2.32462
LIVG_METJA F	23653520	CDK2_HUMAN Fu	6022 Aden	1GY3	1G6H	2R3L		Sc=5.88628, min distance = 2.16606
LIVG_METJA F	23653524	CDK2_HUMAN Fu	6022 Aden	1GY3	1G6H	2R3P		Sc=5.99539, min distance = 2.48230
LIVG_METJA F	23653526	CDK2_HUMAN Fu	6022 Aden	1GY3	1G6H	2R3R		Sc=5.91111, min distance = 2.07065
LIVG_METJA F	23727981	CDK2_HUMAN Fu	6022 Aden	1GY3	1G6H	3BHT		Sc=5.89294, min distance = 2.40474
LIVG_METJA F	23727982	CDK2_HUMAN Fu	6022 Aden	1GY3	1G6H	3BHU		Sc=5.94231, min distance = 2.39038
LIVG_METJA F	24864080	CDK2_HUMAN Fu	6022 Aden	1GY3	1G6H	2VTR		Sc=5.69541, min distance = 3.25674
LIVG_METJA F	25021197	CDK2_HUMAN Fu	6022 Aden	1GY3	1G6H	3EOC		Sc=6.08623, min distance = 1.77961
LIVG_METJA F	445940 O6	CDK2_HUMAN Fu	6022 Aden	1GY3	1G6H	1GZ8		Sc=5.79455, min distance = 2.23651

LIVG_METJA Fı	447656 1c	CDK2_HUMAN Fu:	6022 Aden	1GY3	1G6H	1OIY		Sc=5.97907, min distance = 2.33262(
LIVG_METJA Fı	447962 1p	CDK2_HUMAN Fu:	6022 Aden	1GY3	1G6H	2C5N		Sc=5.92357, min distance = 3.11458(
LIVG_METJA Fı	4566 1h1s	CDK2_HUMAN Fu:	6022 Aden	1GY3	1G6H	1H1S		Sc=5.83213, min distance = 2.08981
LIVG_METJA Fı	5288016 6	CDK2_HUMAN Fu:	6022 Aden	1GY3	1G6H	2B53		Sc=5.87954, min distance = 2.24733
LIVG_METJA Fı	5327130 C	CDK2_HUMAN Fu:	6022 Aden	1GY3	1G6H	2C68		Sc=5.86147, min distance = 2.18785
LIVG_METJA Fı	5327131 T	CDK2_HUMAN Fu:	6022 Aden	1GY3	1G6H	2C69		Sc=5.87236, min distance = 2.15568(
LIVG_METJA Fı	5331010 c	CDK2_HUMAN Fu:	6022 Aden	1GY3	1G6H	2BKZ		Sc=6.06433, min distance = 2.16555
LIVG_METJA Fı	6420139 C	CDK2_HUMAN Fu:	6022 Aden	1GY3	1G6H	2C5V		Sc=5.89124, min distance = 2.96197
LIVG_METJA Fı	6918710 5	CDK2_HUMAN Fu:	6022 Aden	1GY3	1G6H	3EJ1		Sc=5.93549, min distance = 2.59944
LIVG_METJA Fı	9991833 S	CDK2_HUMAN Fu:	6022 Aden	1GY3	1G6H	2R3H		Sc=5.60266, min distance = 2.05894
LMAN2_CANFA I	101798 Me	LEC1_LATOC Fu:	185698 al	1LOG	2E6V	1LOB	0.94	Sc=5.7665, min distance = 2.4280527
LMAN2_CANFA I	151504 D-	CARP_YEAST Fu:	185698 al	1DPJ	2E6V	1FQ5		Sc=5.63561, min distance = 1.904625
LMAN2_CANFA I	151504 D-	CONA_CANEN Fu:	185698 al	1CVN	2E6V	1I3H		Sc=5.92103, min distance = 0.60444
LMAN2_CANFA I	16741013	CARP_YEAST Fu:	185698 al	1DPJ	2E6V	1FQ6		Sc=5.62522, min distance = 1.69549(
LMAN2_CANFA I	446578 1r	Q9HYN5_PSEAE I	185698 al	1OVS	2E6V	2JDP	0.94	Sc=5.61217, min distance = 2.07198(
LMAN2_CANFA I	64947 alp	LEC1_LATOC Fu:	185698 al	1LOG	2E6V	1LOA	0.94	Sc=5.7646, min distance = 2.413372
LMBL1_HUMAN I	6914582 C	ACES_TORCA Fu:	8200 TETR	1DX6	2RHI	2C5F		Sc=5.6138, min distance = 2.126588
LMRA_LACLA Fı	101544 2'	RNAS1_BOVIN Fı	6022 Aden	1O0H	1MV5	1ROB		Sc=6.09928, min distance = 2.08994
LMRA_LACLA Fı	11608401	KAPCA_BOVIN Fı	5957 Aden	1Q24	1MV5	2UVY		Sc=6.03334, min distance = 2.65488(
LMRA_LACLA Fı	24864080	CDK2_HUMAN Fu:	6022 Aden	1GY3	1MV5	2VTR		Sc=5.62052, min distance = 2.84604
LMRA_LACLA Fı	440317 AT	BCKD_RAT Full	6022 Aden	1GKZ	1MV5	1GJV	0.99	Sc=6.05783, min distance = 1.51477
LMRA_LACLA Fı	444564 AD	MYS2_DICDI Fu:	6022 Aden	1VOM	1MV5	1W9I	0.91	Sc=6.33233, min distance = 1.18502
LMRA_LACLA Fı	445940 O6	CDK2_HUMAN Fu:	6022 Aden	1GY3	1MV5	1GZ8		Sc=5.9127, min distance = 2.173184
LMRA_LACLA Fı	4565 1h1r	CDK2_HUMAN Fu:	6022 Aden	1GY3	1MV5	1H1R		Sc=6.1139, min distance = 1.795307
LMRA_LACLA Fı	5687 1di8	CDK2_HUMAN Fu:	6022 Aden	1GY3	1MV5	1DI8		Sc=5.82054, min distance = 2.46586
LMRA_LACLA Fı	6083 aden	AAKG1_RAT Ful:	5957 Aden	2V92	1MV5	2V8Q	0.98	Sc=6.24257, min distance = 2.58869
LMRA_LACLA Fı	6083 aden	PURP_METJA Fu:	6022 Aden	2R7N	1MV5	2R7M	0.99	Sc=6.2262, min distance = 2.503989(
LMRA_LACLA Fı	6083 aden	PURP_PYRFU Fu:	5957 Aden	2R86	1MV5	2R85	0.98	Sc=6.21636, min distance = 2.77912(
LMRA_LACLA Fı	60961 ade	IPKA_RABIT Fu:	6022 Aden	1JBP	1MV5	1FMO	0.95	Sc=5.99922, min distance = 2.96178
LMRA_LACLA Fı	60961 ade	SKY1_YEAST Fu:	6022 Aden	1Q8Y	1MV5	1Q97	0.95	Sc=6.17772, min distance = 2.01833
LMRA_LACLA Fı	60961 ade	SYQ_ECOLI Ful:	5957 Aden	1GTR	1MV5	1O0B	0.94	Sc=6.01545, min distance = 2.35026(
LMRA_LACLA Fı	6804 guar	NDK_PYRHO Ful:	6022 Aden	2DYA	1MV5	2DXF	0.8	Sc=6.31363, min distance = 2.23849(
LOT6_YEAST Fı	16741044	PYRDA_LACLC Fı	444243 FA	1JUB	1T0I	1JRB		Sc=5.83443, min distance = 2.19086(
LOT6_YEAST Fı	446995 FM	CYB2_YEAST Fu:	444243 FA	1KBI	1T0I	1LTD	0.77	Sc=5.84711, min distance = 2.19731
LOT6_YEAST Fı	448076 CI	CYB2_YEAST Fu:	444243 FA	1KBI	1T0I	1SZG	0.76	Sc=5.84011, min distance = 2.11511(
LPXC_AQUAE Fı	445638 pa	NLTP_MAIZE Fu:	11005 Tet	1FK2	1P42	1FK3	0.75	Sc=5.895, min distance = 2.2553150
LPXC_AQUAE Fı	445639 ol	ALBU_HUMAN Fu:	11005 Tet	1N5U	1P42	1GNI		Sc=5.97751, min distance = 2.38175

LPXC_AQUAE Fu	445641 Ri	NLTP_MAIZE Ful	11005 Tet	1FK2	1P42	1FK7			Sc=6.14868, min distance = 2.10692
LPXD_CHLTR Fu	445639 ol	NLTP_MAIZE Ful	985 palmi	1MZM	2IU8	1FK5		0.77	Sc=5.75954, min distance = 3.26420
LSD1_HUMAN Fu	11963950	FMS1_YEAST Ful	444188 CI	1RSG	2DW4	3BI4	42.68	0.99	Sc=6.88875, min distance = 2.26062
LSD1_HUMAN Fu	16740985	FRDA_SHEFR Ful	444188 CI	1M64	2DW4	1E39		0.93	Sc=6.88736, min distance = 2.04214
LSD1_HUMAN Fu	444502 CI	GSHR_HUMAN Ful	444188 CI	3DK9	2DW4	1BWC		0.98	Sc=6.88668, min distance = 2.18325
LSD1_HUMAN Fu	444502 CI	TYTR_TRYCR Ful	444188 CI	1BZL	2DW4	1GXF		0.98	Sc=6.8906, min distance = 2.148501
LSD1_HUMAN Fu	446013 1,	O28603_ARCFU I	444188 CI	1JNR	2DW4	1JNZ		0.94	Sc=6.892, min distance = 2.1198259
LSD1_HUMAN Fu	446013 1,	O28604_ARCFU I	444188 CI	1JNR	2DW4	1JNZ		0.94	Sc=6.8929, min distance = 2.132510
LSD1_HUMAN Fu	6420174 C	O28603_ARCFU I	444188 CI	1JNR	2DW4	2FJB		0.92	Sc=6.89532, min distance = 2.02610
LSD1_HUMAN Fu	6420174 C	O28604_ARCFU I	444188 CI	1JNR	2DW4	2FJB		0.92	Sc=6.89608, min distance = 2.03100
LT_SV40 Full	444564 AD	BIOD_ECOLI Ful	6022 Aden	1DAD	1SVL	1BS1		0.91	Sc=5.69269, min distance = 2.11463
LT_SV40 Full	444842 CI	CDK2_HUMAN Ful	6022 Aden	1GY3	1SVL	1CKP			Sc=5.60997, min distance = 1.98248
LT_SV40 Full	445940 O6	CDK2_HUMAN Ful	6022 Aden	1GY3	1SVL	1GZ8			Sc=6.16907, min distance = 2.15103
LT_SV40 Full	447955 1p	CDK2_HUMAN Ful	6022 Aden	1GY3	1SVL	1PXI			Sc=5.74695, min distance = 2.20433
LT_SV40 Full	448222 NE	ENPL_CANFA Ful	6022 Aden	1TC6	1SVL	1QY5		0.81	Sc=6.22806, min distance = 2.28199
LT_SV40 Full	4725 penc	KITH_HHV11 Ful	6022 Aden	2VTK	1SVL	1KI3			Sc=6.03133, min distance = 1.94077
LT_SV40 Full	5327148 C	IPKA_RABIT Ful	6022 Aden	1JBP	1SVL	2ERZ			Sc=6.1869, min distance = 2.229148
LT_SV40 Full	72194 2-C	ENPL_CANFA Ful	6022 Aden	1TC6	1SVL	1QYE		0.89	Sc=6.17245, min distance = 1.02890
LT_SV40 Full	8977 1dar	O33839_THEMA I	6022 Aden	1XJK	1SVL	1XJE		0.8	Sc=5.74131, min distance = 2.05482
LT_SV40 Full	91532 AME	F263_HUMAN Ful	6022 Aden	2AXN	1SVL	2DWP		0.99	Sc=5.76275, min distance = 2.14337
LUCI_LUCCR Fu	24316 cyc	PDE10_HUMAN Fu	6083 aden	2OUN	2D1R	2OUU		0.81	Sc=6.34279, min distance = 2.12543
LUCI_LUCCR Fu	445708 AM	NADE_ECOLI Ful	6083 aden	1WXI	2D1R	1WXE		0.91	Sc=6.39065, min distance = 2.18561
LUCI_LUCCR Fu	446916 1l	F16P1_PIG Full	6083 aden	2F3D	2D1R	1LEV			Sc=6.26156, min distance = 1.64121
LUCI_LUCCR Fu	447832 AD	ACSA_SALTY Ful	6083 aden	2P2F	2D1R	1PG4		0.99	Sc=6.48892, min distance = 2.29470
LUCI_LUCCR Fu	5327121 I	PIM1_HUMAN Ful	6083 aden	1YXU	2D1R	2C3I			Sc=6.3241, min distance = 1.456934
LUCI_LUCCR Fu	6076 Cycl	PDE10_HUMAN Fu	6083 aden	2OUN	2D1R	2OUR		0.99	Sc=6.32806, min distance = 2.11119
LUCI_LUCCR Fu	611002 Op	PIM1_HUMAN Ful	6083 aden	1YXU	2D1R	1YXX			Sc=6.22671, min distance = 1.68042
LUCI_LUCCR Fu	6419789 C	HASP_HUMAN Ful	6083 aden	3DLZ	2D1R	3E7V			Sc=6.33862, min distance = 2.11986
LUCI_LUCCR Fu	65533 cor	O57693_THETE I	6083 aden	1UXU	2D1R	1UXT			Sc=5.96519, min distance = 2.15242
LUCI_LUCCR Fu	657135 Op	PIM1_HUMAN Ful	6083 aden	1YXU	2D1R	1YXV			Sc=5.9577, min distance = 2.108398
LUCI_LUCCR Fu	8582 Inos	PYGM_RABIT Ful	6083 aden	8GPB	2D1R	2QN7		0.79	Sc=6.4078, min distance = 2.041328
LUXF_PHOLE Fu	5326566 l	BLVRB_HUMAN Fu	444243 FA	1HE4	1NFP	1HE5		0.82	Sc=6.2714, min distance = 2.636868
LUXF_PHOLE Fu	5326566 l	Q9WZW1_THEMA I	444243 FA	1T6Y	1NFP	1S4M		0.82	Sc=6.27488, min distance = 2.08781
LUXP_PHOLE Fu	440869 Ph	RIB4_SCHPO Ful	444658 2a	1KYV	3DDY	2A57			Sc=6.4574, min distance = 2.264807
LUXP_PHOLE Fu	444243 FA	Q9WZW1_THEMA I	444658 2a	1T6Z	3DDY	1T6Y		0.97	Sc=6.61682, min distance = 2.01581
LUXP_PHOLE Fu	446835 1t	RIB4_SCHPO Ful	444658 2a	1KYV	3DDY	1KYY			Sc=6.34068, min distance = 2.18318
LUXP_PHOLE Fu	449551 FM	FLAV_DESVH Ful	444658 2a	1BU5	3DDY	5FX2			Sc=6.10945, min distance = 2.17760

LUXP_PHOLE F	5326566 l	Q9HPW4_HALSA I	444658 2a	2CCB	3DDY	2CC6		0.85	Sc=6.18175, min distance = 2.25419
LUXP_PHOLE F	5326566 l	Q9WZW1_THEMA I	444658 2a	1T6Z	3DDY	1S4M		0.85	Sc=6.24612, min distance = 2.51627
LY96_HUMAN F	8209 Myri	ABL1_MOUSE Fu	11005 Tet	1OPK	2E56	1OPJ			Sc=5.6414, min distance = 2.637997
LYAM3_HUMAN I	185698 al	MBL1_RAT Full	444885 O-	2KMB	1G1S	2MSB	46.51		Sc=5.80476, min distance = 1.66840
LYAM3_HUMAN I	446578 1r	MBL1_RAT Full	439554 fu	3KMB	1G1S	1KWW	46.51	0.96	Sc=5.75863, min distance = 1.27435
LYAM3_HUMAN I	6914591 E	A2NHM3_MOUSE I	439554 fu	1CLZ	1G1S	2CJU			Sc=6.24206, min distance = 2.18944
LYG_ANSAN Fu	185698 al	Q9HYN5_PSEAE I	24139 ace	1W8F	154L	1OVS			Sc=5.77268, min distance = 1.13094
LYG_ANSAN Fu	449023 N2	CONA_CANEN Fu	24139 ace	1TEI	154L	1VAM			Sc=6.24738, min distance = 1.08143
LYG_ANSAN Fu	449094 Cy	Q54276_SERMA I	24139 ace	1UR9	154L	1W1T			Sc=5.99458, min distance = 2.37541
LYG_ANSAN Fu	64947 alp	CONA_CANEN Fu	24139 ace	1TEI	154L	1GIC			Sc=5.6966, min distance = 2.464700
LYG_ANSAN Fu	656981 CU	LYSC_CHICK Fu	24139 ace	1LZB	154L	1YIL			Sc=5.83255, min distance = 2.35133
LYS1_YEAST F	5957 Ader	AAKG1_RAT Full	6083 aden	2V8Q	2QRK	2V92		0.98	Sc=5.66103, min distance = 2.17735
LYS1_YEAST F	5957 Ader	PURP_PYRFU Fu	6083 aden	2R85	2QRK	2R86		0.98	Sc=5.64755, min distance = 2.05530
LYS9_MAGGR F	440141 9i	DHB1_HUMAN Fu	5886 NADF	1QYV	1E5Q	1QYW			Sc=5.95593, min distance = 2.21214
LYSC2_ONCMY I	185698 al	Q9HYN5_PSEAE I	82313 1rd	1W8H	1LMQ	1OVS			Sc=5.6153, min distance = 2.120251
LYSC2_ONCMY I	439680 be	SPIKE_CVHSA F	82313 1rd	3D0G	1LMQ	2AJF			Sc=5.65103, min distance = 2.00444
LYSC2_ONCMY I	439710 rh	PGRP_CAMDR Fu	82313 1rd	3C93	1LMQ	3CG9			Sc=5.77268, min distance = 0.97205
LYSC2_ONCMY I	444305 L-	PGRP_CAMDR Fu	82313 1rd	3C93	1LMQ	3C2X			Sc=5.63497, min distance = 2.03722
LYSC2_ONCMY I	444762 CI	HYSA_STRPN Fu	24139 ace	1LXK	1LMQ	1C82			Sc=5.7881, min distance = 1.020174
LYSC2_ONCMY I	446996 GA	Q9HYN5_PSEAE I	24139 ace	1W8F	1LMQ	1W8H			Sc=5.673, min distance = 1.8093305
LYSC2_ONCMY I	64689 bet	Q9HYN5_PSEAE I	82313 1rd	1W8H	1LMQ	1W8F			Sc=5.72788, min distance = 1.77567
LYSC2_ONCMY I	656981 CU	LYSC_CHICK Fu	24139 ace	1LZB	1LMQ	1YIL	62.99		Sc=5.68362, min distance = 3.03392
LYSC_CHICK F	165293 De	CTXA3_NAJAT F	8778 Laur	1H0J	3B6L	2BHI			Sc=5.89158, min distance = 0.70335
LYSC_CHICK F	2153 thec	Q873X9_ASPFU I	24139 ace	1WNO	1LZB	2A3A			Sc=5.78719, min distance = 1.90454
LYSC_CHICK F	439554 fu	LECB_LATOC Fu	24139 ace	1LOG	1LZB	1LGC			Sc=5.62651, min distance = 2.03015
LYSC_CHICK F	445948 D-	Q9HYN5_PSEAE I	439353 be	1W8F	1UC0	1OXC			Sc=5.8121, min distance = 1.843212
LYSC_CHICK F	445948 D-	Q9HYN5_PSEAE I	82313 1rd	1W8H	1LSZ	1OXC			Sc=5.66805, min distance = 2.19806
LYSC_CHICK F	446101 AC	P84141_ARTAU I	24139 ace	1RWC	1LZB	1RWH		0.94	Sc=5.71498, min distance = 2.73395
LYSC_CHICK F	446476 Bi	A0A5D7_MOUSE I	24139 ace	1M7I	1LZB	1JGU			Sc=5.9784, min distance = 2.113047
LYSC_CHICK F	446578 1r	Q9HYN5_PSEAE I	439353 be	1W8F	1UC0	2JDP		0.94	Sc=5.77779, min distance = 1.74874
LYSC_CHICK F	446578 1r	Q9HYN5_PSEAE I	82313 1rd	1W8H	1LSZ	2JDP			Sc=5.64022, min distance = 2.25102
LYSC_CHICK F	6102754 2	Q9HYN5_PSEAE I	439353 be	1W8F	1UC0	2BOJ		0.88	Sc=5.69964, min distance = 1.73538
LYSC_CHICK F	64689 bet	P74325_SYNY3 I	5988 sucr	1TJ5	1JJ0	1U2S		0.87	Sc=5.78001, min distance = 2.09076
LYSC_CHICK F	64689 bet	Q9HYN5_PSEAE I	82313 1rd	1W8H	1LSZ	1W8F			Sc=5.63561, min distance = 1.99385
LYSC_HUMAN F	119138 Th	LEG1_BUFAR Fu	439353 be	1GAN	1REZ	1A78			Sc=6.01279, min distance = 2.13208
LYSC_HUMAN F	16058615	LYSC_CHICK Fu	24139 ace	1LZB	1LZR	2H9J	63.28		Sc=5.98094, min distance = 1.84192
LYSC_HUMAN F	185698 al	CONA_CANEN Fu	24139 ace	1TEI	1LZR	1CVN			Sc=5.96906, min distance = 1.02813

LYSC_HUMAN Fu	439554 fu	LECB_LATOC Ful	24139 ace	1LOG	1LZR	1LGC		Sc=5.61221, min distance = 1.119384
LYSC_HUMAN Fu	445256 NG	Q54276_SERMA I	24139 ace	1UR9	1LZR	1E6Z	0.82	Sc=5.77722, min distance = 2.103441
LYSC_HUMAN Fu	445750 Al	LYAM3_HUMAN Fu	439353 be	1G1S	1REZ	1G1R		Sc=6.10599, min distance = 1.304113
LYSC_HUMAN Fu	446996 GA	Q9HYN5_PSEAE I	24139 ace	1W8F	1LZR	1W8H		Sc=5.64592, min distance = 1.253239
LYSC_HUMAN Fu	656981 CU	LYSC_CHICK Ful	24139 ace	1LZB	1LZR	1YIL	63.28	Sc=5.95481, min distance = 2.090998
LYSC_MELGA Fu	101798 Me	MBL1_RAT Full=	82313 1rd	1KWV	1LZY	1KWU		Sc=5.86661, min distance = 1.479678
LYSC_MELGA Fu	10367 Dip	LYSC_CHICK Ful	24139 ace	1LZB	1LZY	2PC2		Sc=5.71883, min distance = 2.396469
LYSC_MELGA Fu	185698 al	MBL2_RAT Full=	82313 1rd	1RDN	1LZY	1RDL		Sc=5.77694, min distance = 1.722162
LYSC_MELGA Fu	439353 be	AGI3_WHEAT Ful	24139 ace	1K7U	1LZY	1K7V		Sc=5.70694, min distance = 2.033328
LYSC_MELGA Fu	439353 be	CLC4M_HUMAN Fu	24139 ace	1K9J	1LZY	1SL6		Sc=5.90354, min distance = 1.066607
LYSC_MELGA Fu	444863 is	MBL1_RAT Full=	82313 1rd	1KWV	1LZY	1KWX		Sc=5.77455, min distance = 1.180178
LYSC_MELGA Fu	446578 1r	MBL2_RAT Full=	82313 1rd	1RDN	1LZY	1RDI		Sc=5.64728, min distance = 2.266169
LYSC_MELGA Fu	6134 lact	LECG_ARAHY Ful	82313 1rd	1CIW	1LZY	2PEL		Sc=6.30962, min distance = 2.116565
LYSC_MELGA Fu	64689 bet	UGL_BACGL Full	82313 1rd	2FV1	1LZY	2FV0		Sc=5.69574, min distance = 1.276665
LYSC_MELGA Fu	79025 alp	LECG_ARAHY Ful	82313 1rd	1CIW	1LZY	1V6I		Sc=5.71322, min distance = 1.883546
LYSC_MELGA Fu	79025 alp	LEC_ERYCO Full	82313 1rd	1AX2	1LZY	1FYU		Sc=5.70048, min distance = 2.892745
LYSC_MELGA Fu	84571 lac	LECG_ARAHY Ful	82313 1rd	1CIW	1LZY	2PEL		Sc=6.31482, min distance = 2.173007
LYS_BPCP1 Ful	656981 CU	LYSC_CHICK Ful	24139 ace	1LZB	2J8G	1YIL		Sc=6.02577, min distance = 1.355656
LYS_BPP1 Full	51 2-Oxop	SERA_ECOLI Ful	311 citri	2P9E	1XJT	1YBA	0.76	Sc=5.60978, min distance = 2.306343
LYS_BPT4 Full	79025 alp	AMYB_BACCE Ful	6255 malt	1B9Z	2RH1	1VEM	0.87	Sc=5.72108, min distance = 0.762391
LYS_LAMBD Ful	126154 Cy	Q54276_SERMA I	24139 ace	1UR9	1D9U	1W1P		Sc=5.66913, min distance = 1.765548
LYS_LAMBD Ful	16058615	LYSC_CHICK Ful	24139 ace	1LZB	1D9U	2H9J		Sc=5.93998, min distance = 1.090898
LYS_LAMBD Ful	185698 al	CONA_CANEN Ful	24139 ace	1TEI	1D9U	1CVN		Sc=5.93, min distance = 1.018164031
LYS_LAMBD Ful	439353 be	LEG1_BOVIN Ful	24139 ace	1SLC	1D9U	1SLT		Sc=5.78164, min distance = 1.658292
LYS_LAMBD Ful	439353 be	LYSC_CHICK Ful	24139 ace	1LZB	1D9U	1UC0		Sc=5.7024, min distance = 1.3773877
LYS_LAMBD Ful	444762 CI	HYSA_STRPN Ful	24139 ace	1LXK	1D9U	1C82		Sc=5.77455, min distance = 1.208621
LYS_LAMBD Ful	445247 CI	Q54276_SERMA I	24139 ace	1UR9	1D9U	1E6R		Sc=5.62287, min distance = 2.721083
LYS_LAMBD Ful	445595 CI	CHIA_SERMA Ful	24139 ace	1K9T	1D9U	1FFQ		Sc=5.9065, min distance = 2.4302172
LYS_LAMBD Ful	445750 Al	HVM32_MOUSE Fu	24139 ace	1M7I	1D9U	1M7D	0.96	Sc=5.99204, min distance = 2.115881
LYS_LAMBD Ful	446101 AC	HYSA_STRPN Ful	24139 ace	1LXK	1D9U	1OJO	0.94	Sc=6.00979, min distance = 2.453900
LYS_LAMBD Ful	6927060 Z	HYSA_STRPN Ful	24139 ace	1LXK	1D9U	1F9G		Sc=5.66303, min distance = 2.196644
M3K5_HUMAN Fu	10109823	PIM1_HUMAN Ful	444345 1c	1YHS	2CLQ	3CY3		Sc=6.20298, min distance = 2.934884
M3K5_HUMAN Fu	11348631	CHK1_HUMAN Ful	444345 1c	1NVR	2CLQ	2E9U		Sc=6.20726, min distance = 2.396549
M3K5_HUMAN Fu	11553058	LCK_HUMAN Full	444345 1c	1QPD	2CLQ	2OF2		Sc=6.26278, min distance = 2.150051
M3K5_HUMAN Fu	11957417	CDK2_HUMAN Ful	444345 1c	1AQ1	2CLQ	2I40		Sc=6.27165, min distance = 2.241215
M3K5_HUMAN Fu	11992146	MAPK2_HUMAN Fu	444345 1c	2PZY	2CLQ	2P3G		Sc=6.26689, min distance = 2.187559
M3K5_HUMAN Fu	1540 1h1q	CDK2_HUMAN Ful	444345 1c	1AQ1	2CLQ	1H1Q		Sc=6.23906, min distance = 2.670077

M3K5_HUMAN F	15602982	KAPCA_BOVIN F	444345 1c 1STC	2CLQ	2UW6		Sc=6.04164, min distance = 2.67353
M3K5_HUMAN F	15991572	LCK_HUMAN Ful	444345 1c 1QPD	2CLQ	2OF4		Sc=6.23672, min distance = 2.41843
M3K5_HUMAN F	16058647	PIM1_HUMAN Fu	444345 1c 1YHS	2CLQ	2OI4	0.75	Sc=6.41333, min distance = 2.12311
M3K5_HUMAN F	16113377	CDK2_HUMAN Fu	444345 1c 1AQ1	2CLQ	2W17		Sc=6.37859, min distance = 1.73714
M3K5_HUMAN F	16122608	PDPK1_HUMAN F	444345 1c 1OKY	2CLQ	2PE1		Sc=6.29469, min distance = 2.55713
M3K5_HUMAN F	16214823	CDK2_HUMAN Fu	444345 1c 1AQ1	2CLQ	2UZB		Sc=6.04282, min distance = 2.56456
M3K5_HUMAN F	16214825	CDK2_HUMAN Fu	444345 1c 1AQ1	2CLQ	2UZE		Sc=6.22132, min distance = 2.22640
M3K5_HUMAN F	16214826	CDK2_HUMAN Fu	444345 1c 1AQ1	2CLQ	2UZL		Sc=6.09246, min distance = 2.13204
M3K5_HUMAN F	16758227	CHK1_HUMAN Fu	444345 1c 1NVR	2CLQ	2R0U		Sc=6.53023, min distance = 2.01616
M3K5_HUMAN F	1907917 2	CHK1_HUMAN Fu	444345 1c 1NVR	2CLQ	2CGW		Sc=6.08153, min distance = 2.87902
M3K5_HUMAN F	23536770	LCK_HUMAN Ful	444345 1c 1QPD	2CLQ	3BYM		Sc=6.43613, min distance = 2.27595
M3K5_HUMAN F	23653515	CDK2_HUMAN Fu	444345 1c 1AQ1	2CLQ	2R3F		Sc=5.96968, min distance = 2.60642
M3K5_HUMAN F	23653516	CDK2_HUMAN Fu	444345 1c 1AQ1	2CLQ	2R3G		Sc=6.10304, min distance = 2.66879
M3K5_HUMAN F	23653518	CDK2_HUMAN Fu	444345 1c 1AQ1	2CLQ	2R3J		Sc=6.14413, min distance = 2.42817
M3K5_HUMAN F	23653519	CDK2_HUMAN Fu	444345 1c 1AQ1	2CLQ	2R3K		Sc=6.17261, min distance = 2.42024
M3K5_HUMAN F	23653520	CDK2_HUMAN Fu	444345 1c 1AQ1	2CLQ	2R3L		Sc=6.14228, min distance = 2.51521
M3K5_HUMAN F	23653521	CDK2_HUMAN Fu	444345 1c 1AQ1	2CLQ	2R3M		Sc=6.33169, min distance = 2.38501
M3K5_HUMAN F	23653522	CDK2_HUMAN Fu	444345 1c 1AQ1	2CLQ	2R3N		Sc=6.219, min distance = 2.6234441
M3K5_HUMAN F	23653524	CDK2_HUMAN Fu	444345 1c 1AQ1	2CLQ	2R3P		Sc=6.15068, min distance = 2.56884
M3K5_HUMAN F	23657800	CHK1_HUMAN Fu	444345 1c 1NVR	2CLQ	2E9P		Sc=6.23402, min distance = 2.34049
M3K5_HUMAN F	23727982	CDK2_HUMAN Fu	444345 1c 1AQ1	2CLQ	3BHU		Sc=6.14238, min distance = 2.19884
M3K5_HUMAN F	24752838	CHK1_HUMAN Fu	444345 1c 1NVR	2CLQ	2QHM		Sc=6.02903, min distance = 2.69274
M3K5_HUMAN F	24851689	PIM1_HUMAN Fu	444345 1c 1YHS	2CLQ	3CY2		Sc=6.16549, min distance = 2.29346
M3K5_HUMAN F	24864081	CDK2_HUMAN Fu	444345 1c 1AQ1	2CLQ	2VTS		Sc=6.22348, min distance = 2.66700
M3K5_HUMAN F	24901723	CDK2_HUMAN Fu	444345 1c 1AQ1	2CLQ	2W06		Sc=6.15216, min distance = 2.08868
M3K5_HUMAN F	24905144	PK3CG_HUMAN F	444345 1c 1E8Z	2CLQ	3ENE		Sc=6.16148, min distance = 2.84429
M3K5_HUMAN F	24916751	CDK2_HUMAN Fu	444345 1c 1AQ1	2CLQ	3DOG		Sc=6.2256, min distance = 2.509214
M3K5_HUMAN F	25021197	CDK2_HUMAN Fu	444345 1c 1AQ1	2CLQ	3EOC		Sc=6.20681, min distance = 2.43312
M3K5_HUMAN F	33113 gam	ZAP70_HUMAN F	444345 1c 1U59	2CLQ	2OZO		Sc=5.67376, min distance = 2.15459
M3K5_HUMAN F	3543 1g5s	CDK2_HUMAN Fu	444345 1c 1AQ1	2CLQ	1G5S		Sc=6.45641, min distance = 2.62958
M3K5_HUMAN F	4369136 C	CDK2_HUMAN Fu	444345 1c 1AQ1	2CLQ	1DM2		Sc=6.11749, min distance = 2.93760
M3K5_HUMAN F	445840 di	CDK2_HUMAN Fu	444345 1c 1AQ1	2CLQ	1GII		Sc=6.18522, min distance = 2.63244
M3K5_HUMAN F	445966 O6	CDK2_HUMAN Fu	444345 1c 1AQ1	2CLQ	1H0V		Sc=6.05004, min distance = 2.91361
M3K5_HUMAN F	447446 1r	CHK1_HUMAN Fu	444345 1c 1NVR	2CLQ	1NVS	0.84	Sc=6.24088, min distance = 2.80410
M3K5_HUMAN F	447649 1c	CDK2_HUMAN Fu	444345 1c 1AQ1	2CLQ	1OI9		Sc=6.32241, min distance = 2.76902
M3K5_HUMAN F	447652 Im	CDK2_HUMAN Fu	444345 1c 1AQ1	2CLQ	1OIQ		Sc=6.1214, min distance = 2.613624
M3K5_HUMAN F	447654 CI	CDK2_HUMAN Fu	444345 1c 1AQ1	2CLQ	1OIT		Sc=6.12698, min distance = 1.98907

M3K5_HUMAN Fı 447655 1c	CDK2_HUMAN Fu 444345 1c 1AQ1	2CLQ	1OIU			Sc=6.24794, min distance = 2.65439
M3K5_HUMAN Fı 447962 1p	CDK2_HUMAN Fu 444345 1c 1AQ1	2CLQ	2C5N			Sc=6.08166, min distance = 2.38125
M3K5_HUMAN Fı 447967 CI	CDK2_HUMAN Fu 444345 1c 1AQ1	2CLQ	1PYE			Sc=6.29781, min distance = 2.25426
M3K5_HUMAN Fı 448043 2g	KAPCA_BOVIN Fı 444345 1c 1STC	2CLQ	1Q8U			Sc=5.96524, min distance = 2.66325
M3K5_HUMAN Fı 449240 1y	KAPCA_BOVIN Fı 444345 1c 1STC	2CLQ	1YDR			Sc=5.89708, min distance = 2.45246
M3K5_HUMAN Fı 4565 1h1r	CDK2_HUMAN Fu 444345 1c 1AQ1	2CLQ	1H1R			Sc=6.23651, min distance = 2.87957
M3K5_HUMAN Fı 4566 1h1s	CDK2_HUMAN Fu 444345 1c 1AQ1	2CLQ	1H1S			Sc=6.27533, min distance = 2.39642
M3K5_HUMAN Fı 5281672 m	PIM1_HUMAN Fu 444345 1c 1YHS	2CLQ	2O63			Sc=6.36523, min distance = 2.59384
M3K5_HUMAN Fı 5288016 6	CDK2_HUMAN Fu 444345 1c 1AQ1	2CLQ	2B53			Sc=6.11559, min distance = 2.34808
M3K5_HUMAN Fı 5288017 I	CDK2_HUMAN Fu 444345 1c 1AQ1	2CLQ	2B55			Sc=6.46044, min distance = 2.81839
M3K5_HUMAN Fı 5288708 1	CDK2_HUMAN Fu 444345 1c 1AQ1	2CLQ	1KE5			Sc=6.08724, min distance = 2.45207
M3K5_HUMAN Fı 5288710 C	CDK2_HUMAN Fu 444345 1c 1AQ1	2CLQ	1KE7			Sc=6.2946, min distance = 2.259588
M3K5_HUMAN Fı 5327121 I	PIM1_HUMAN Fu 444345 1c 1YHS	2CLQ	2C3I			Sc=6.08322, min distance = 2.36796
M3K5_HUMAN Fı 5327123 I	CHK1_HUMAN Fu 444345 1c 1NVR	2CLQ	2C3L			Sc=6.11735, min distance = 2.19436
M3K5_HUMAN Fı 5327134 C	CDK2_HUMAN Fu 444345 1c 1AQ1	2CLQ	2C6L			Sc=6.31163, min distance = 2.34108
M3K5_HUMAN Fı 5331010 c	CDK2_HUMAN Fu 444345 1c 1AQ1	2CLQ	2BKZ			Sc=6.26455, min distance = 2.42191
M3K5_HUMAN Fı 5687 1di8	CDK2_HUMAN Fu 444345 1c 1AQ1	2CLQ	1DI8			Sc=6.18391, min distance = 2.32626
M3K5_HUMAN Fı 6022 Aden	CDK2_HUMAN Fu 444345 1c 1AQ1	2CLQ	1GY3			Sc=5.87245, min distance = 2.02173
M3K5_HUMAN Fı 6022 Aden	GSK3B_HUMAN Fı 444345 1c 1Q3D	2CLQ	1J1C			Sc=5.91537, min distance = 1.81202
M3K5_HUMAN Fı 6102670	CDK2_HUMAN Fu 444345 1c 1AQ1	2CLQ	1YKR			Sc=6.23061, min distance = 2.09949
M3K5_HUMAN Fı 6420139 C	CDK2_HUMAN Fu 444345 1c 1AQ1	2CLQ	2C5V			Sc=6.08465, min distance = 2.28948
M3K5_HUMAN Fı 656972 CT	CDK2_HUMAN Fu 444345 1c 1AQ1	2CLQ	1Y91			Sc=6.43048, min distance = 2.42565
M3K5_HUMAN Fı 6852166	PK3CG_HUMAN Fı 444345 1c 1E8Z	2CLQ	2CHX			Sc=6.18025, min distance = 2.06839
M3K5_HUMAN Fı 6852207 M	KAPCA_BOVIN Fı 444345 1c 1STC	2CLQ	2GNI			Sc=5.91243, min distance = 2.22470
M3K5_HUMAN Fı 72271 7-H	CDK2_HUMAN Fu 444345 1c 1AQ1	2CLQ	1PKD		0.91	Sc=6.43571, min distance = 2.67056
M3K5_HUMAN Fı 72271 7-H	CHK1_HUMAN Fu 444345 1c 1NVR	2CLQ	1NVQ		0.91	Sc=6.43061, min distance = 2.75774
M3K5_HUMAN Fı 9547890 I	CDK2_HUMAN Fu 444345 1c 1AQ1	2CLQ	1W8C			Sc=6.16841, min distance = 2.48553
M3K5_HUMAN Fı 9817550 V	CDK2_HUMAN Fu 444345 1c 1AQ1	2CLQ	3BHV			Sc=6.35382, min distance = 2.19101
M3K7_HUMAN Fı 3973 nche	PIM1_HUMAN Fu 60961 ade 1YI4	2EVA	1YI3			Sc=5.93862, min distance = 2.04404
M3K7_HUMAN Fı 447864 2'	DEOD_ECOLI Fu 60961 ade 1PK7	2EVA	1PKE		0.96	Sc=6.10488, min distance = 2.42174
M3K7_HUMAN Fı 5281672 m	PIM1_HUMAN Fu 60961 ade 1YI4	2EVA	2O63			Sc=6.40594, min distance = 2.15304
M3K7_HUMAN Fı 6021 inos	TMPC_TREPA Fu 60961 ade 2FQY	2EVA	2FQW		0.79	Sc=6.17038, min distance = 2.76253
M3K7_HUMAN Fı 6802 guar	A5KE01_PLAVI I 60961 ade 2PGF	2EVA	2QVN		0.79	Sc=6.23554, min distance = 2.57718
M3K7_HUMAN Fı 6802 guar	TMPC_TREPA Fu 60961 ade 2FQY	2EVA	2FQX		0.79	Sc=6.24216, min distance = 2.56497
M3K7_HUMAN Fı 97184 6-M	DEOD_ECOLI Fu 60961 ade 1PK7	2EVA	1OVG		0.9	Sc=6.14068, min distance = 1.21460
MAAI_HUMAN Fı 115260 S-	GSTM2_HUMAN Fı 124886 gl 1XW5	1FW1	2AB6		0.94	Sc=5.94287, min distance = 2.43118
MAAI_HUMAN Fı 97536 Hex	GSTP1_MOUSE Fı 124886 gl 1GSY	1FW1	2GLR		0.9	Sc=6.56777, min distance = 1.42997

MAAI_HUMAN Fı	97536 Hex	GSTP_ONCVO Fu	124886 gl	1TU7	1FW1	1TU8		0.9	Sc=6.56652, min distance = 2.43796
MAAI_MOUSE Fı	115260 S-	GSTM2_HUMAN Fı	124886 gl	1XW5	2CZ2	2AB6		0.94	Sc=6.41392, min distance = 1.94443
MAAI_MOUSE Fı	444104 II	GSTP1_MOUSE Fı	124886 gl	1GSY	2CZ2	1GLP		0.9	Sc=6.42654, min distance = 1.99272
MAAI_MOUSE Fı	97536 Hex	GSTP1_MOUSE Fı	124886 gl	1GSY	2CZ2	2GLR		0.9	Sc=6.58556, min distance = 1.72354
MALE_ECOLI Fı	15602983	KAPCA_BOVIN Fı	5957 Aden	1Q24	2NVU	2UW5			Sc=6.12698, min distance = 2.38565
MALE_ECOLI Fı	16122632	KAPCA_BOVIN Fı	5957 Aden	1Q24	2NVU	2UVZ			Sc=6.37965, min distance = 2.10244
MALE_ECOLI Fı	17751819	KAPCA_BOVIN Fı	5957 Aden	1Q24	2NVU	2VO6			Sc=6.29212, min distance = 2.36794
MALE_ECOLI Fı	3547 Fasu	KAPCA_BOVIN Fı	5957 Aden	1Q24	2NVU	1Q8W			Sc=5.99195, min distance = 2.24586
MALE_ECOLI Fı	444139 CI	CDGT2_BACCI Fı	79025 alp	1D3C	1ANF	1DTU			Sc=5.68403, min distance = 1.45061
MALE_ECOLI Fı	445916 be	XYLA_STRRU Fu	64960 Pol	1XIE	1LAX	1GW9		0.82	Sc=5.64704, min distance = 2.73322
MALE_ECOLI Fı	446000 CI	AMYP_PIG Full	79025 alp	1HX0	1ANF	1JFH		0.83	Sc=5.7389, min distance = 2.022522
MALE_ECOLI Fı	448042 2e	KAPCA_BOVIN Fı	5957 Aden	1Q24	2NVU	1Q8T			Sc=6.17099, min distance = 2.21573
MALE_ECOLI Fı	449571 CI	CDGT1_BACCI Fı	79025 alp	1CGU	1ANF	6CGT			Sc=5.80997, min distance = 2.30349
MALE_ECOLI Fı	6022 Aden	REX_BACSU Ful	5957 Aden	2VT3	2NVU	2VT2		0.99	Sc=6.4463, min distance = 1.960637
MALE_ECOLI Fı	64689 bet	Q9RX51_DEIRA I	6255 malt	2BHZ	1IUD	2BY0		0.87	Sc=5.74332, min distance = 2.31449
MALE_ECOLI Fı	657035 CI	AMYB_SOYBN Fu	79025 alp	1V3H	1ANF	1WDR		0.87	Sc=6.34855, min distance = 2.47308
MALE_ECOLI Fı	7027 gluc	Q9C171_PIREQ I	79025 alp	1GWM	1ANF	1W8T			Sc=5.86264, min distance = 2.12559
MALE_ECOLI Fı	7048614 Z	XYLA_STRRU Fu	64960 Pol	1XIE	1LAX	1XID			Sc=5.84152, min distance = 2.46328
MALE_ECOLI Fı	7427 treh	CONA_CANLI Fu	657130 CI	2CYF	1YTV	2CY6	1		Sc=6.40245, min distance = 2.52630
MALE_ECOLI Fı	95259 L-x	XYLA_STRRU Fu	64960 Pol	1XIE	1LAX	1XIC			Sc=5.74678, min distance = 2.49027
MALE_PYRFU Fı	193758 Va	CDGT2_BACCI Fı	79025 alp	1D3C	1ELJ	1DTU			Sc=5.86105, min distance = 1.11252
MALE_PYRFU Fı	23831 HEE	CDGT2_BACCI Fı	79025 alp	1D3C	1ELJ	1OT1			Sc=5.70919, min distance = 0.17725
MALE_PYRFU Fı	444139 CI	AMYP_PIG Full	79025 alp	1HX0	1ELJ	1PIG			Sc=5.68067, min distance = 1.57821
MALE_PYRFU Fı	444139 CI	CDGT2_BACCI Fı	79025 alp	1D3C	1ELJ	1DTU			Sc=5.8065, min distance = 0.720762
MALE_PYRFU Fı	444440 AC	AMY1_HORVU Fu	79025 alp	1RP8	1ELJ	1RP9			Sc=6.34963, min distance = 2.60505
MALE_PYRFU Fı	444915 Gl	CDGT2_BACCI Fı	79025 alp	1D3C	1ELJ	1CXL		0.85	Sc=5.66645, min distance = 0.75009
MALE_PYRFU Fı	445237 1,	AMY1_HORVU Fu	79025 alp	1RP8	1ELJ	1P6W		0.77	Sc=5.85815, min distance = 0.82006
MALE_PYRFU Fı	446000 CI	AMYP_PIG Full	79025 alp	1HX0	1ELJ	1JFH		0.83	Sc=5.67768, min distance = 2.57275
MALE_PYRFU Fı	446685 Va	NEPU1_THEVU Fı	79025 alp	1UH4	1ELJ	1UH3			Sc=5.92347, min distance = 1.46574
MALE_PYRFU Fı	447600 CI	AMY1_HORVU Fu	79025 alp	1RP8	1ELJ	1P6W		0.83	Sc=5.76468, min distance = 1.67080
MALE_PYRFU Fı	657034 1w	AMYB_SOYBN Fu	79025 alp	1V3H	1ELJ	1WDQ			Sc=6.38028, min distance = 2.16617
MALK_ECOLI Fı	11957393	FAK1_HUMAN Fu	6022 Aden	1MP8	2AWN	2ETM			Sc=6.02053, min distance = 1.65667
MALK_ECOLI Fı	1540 1h1c	CDK2_HUMAN Fu	6022 Aden	1GY3	2AWN	1H1Q			Sc=5.91491, min distance = 1.92104
MALK_ECOLI Fı	16214823	CDK2_HUMAN Fu	6022 Aden	1GY3	2AWN	2UZB			Sc=5.90142, min distance = 2.22085
MALK_ECOLI Fı	24864081	CDK2_HUMAN Fu	6022 Aden	1GY3	2AWN	2VTS			Sc=6.08224, min distance = 1.88164
MALK_ECOLI Fı	25021197	CDK2_HUMAN Fu	6022 Aden	1GY3	2AWN	3EOC			Sc=6.00673, min distance = 1.76538
MALK_ECOLI Fı	447962 1p	CDK2_HUMAN Fu	6022 Aden	1GY3	2AWN	2C5N			Sc=5.71596, min distance = 2.73017

MALK_ECOLI F	4565 1h1r	CDK2_HUMAN Fu	6022 Aden	1GY3	2AWN	1H1R		Sc=5.93225, min distance = 1.91703
MALK_ECOLI F	4566 1h1s	CDK2_HUMAN Fu	6022 Aden	1GY3	2AWN	1H1S		Sc=5.95698, min distance = 1.48103
MALK_ECOLI F	5288016 6	CDK2_HUMAN Fu	6022 Aden	1GY3	2AWN	2B53		Sc=5.90244, min distance = 1.59264
MALK_ECOLI F	5327133 C	CDK2_HUMAN Fu	6022 Aden	1GY3	2AWN	2C6K		Sc=5.81416, min distance = 2.14726
MALK_ECOLI F	5327135 D	CDK2_HUMAN Fu	6022 Aden	1GY3	2AWN	2C6M		Sc=5.80905, min distance = 2.03699
MALK_ECOLI F	5494449 T	STK6_HUMAN Fu	6022 Aden	1MQ4	2AWN	3E5A		Sc=6.30902, min distance = 2.00962
MALK_ECOLI F	6083 aden	Y059_METJA Fu	5957 Aden	2J9C	1Q12	2J9D	0.98	Sc=5.99332, min distance = 2.44783
MALK_ECOLI F	6852207 M	KAPCA_BOVIN F	5957 Aden	1Q24	1Q12	2GNI		Sc=5.83279, min distance = 1.53866
MALK_ECOLI F	72194 2-C	ENPL_CANFA Fu	6022 Aden	1TC6	2AWN	1QYE	0.89	Sc=5.86383, min distance = 2.19448
MALK_ECOLI F	91532 AMP	INSR_HUMAN Fu	5957 Aden	3BU5	1Q12	1I44	0.99	Sc=6.32933, min distance = 1.64682
MALK_ECOUT F	11708454	KAPCA_BOVIN F	5957 Aden	1Q24	2R6G	2VNW		Sc=6.02728, min distance = 1.66110
MALK_ECOUT F	16122607	PDPK1_HUMAN F	5957 Aden	2BIY	2R6G	2PE0		Sc=5.99436, min distance = 2.34699
MALK_ECOUT F	3540 1yds	KAPCA_BOVIN F	5957 Aden	1Q24	2R6G	1YDS		Sc=5.68323, min distance = 1.54450
MALQ_THELI F	447607 1c	PYGM_RABIT Fu	79025 alp	1H5U	1K1W	2G9V		Sc=5.71337, min distance = 2.10126
MALQ_THELI F	6027 xylo	Q9ZB17_9LACT	79025 alp	1NSZ	1K1W	1MN0	0.93	Sc=5.64481, min distance = 2.39617
MALQ_THELI F	84571 lac	LECG_ARAHY Fu	79025 alp	1V6I	1K1W	2PEL	0.87	Sc=6.4201, min distance = 2.007604
MAN2_DROME F	101798 Me	Q8XUA5_RALSO	185698 al	2CHH	3CZS	1UQX	0.94	Sc=5.88516, min distance = 2.46550
MAN2_DROME F	11957432	BGLA_THEMA Fu	445248 nc	2J78	3D51	2J7E		Sc=6.2956, min distance = 2.098093
MAN2_DROME F	11957432	BGLA_THEMA Fu	9549193 C	2CES	3D4Z	2J7E	0.87	Sc=6.31471, min distance = 1.93026
MAN2_DROME F	11957433	BGLA_THEMA Fu	9549193 C	2CES	3D4Z	2J7F	0.89	Sc=6.22437, min distance = 2.02594
MAN2_DROME F	11957434	BGLA_THEMA Fu	445248 nc	2J78	3D51	2J7G		Sc=6.09696, min distance = 2.22135
MAN2_DROME F	11957434	BGLA_THEMA Fu	9549193 C	2CES	3D4Z	2J7G	0.89	Sc=6.10047, min distance = 2.01023
MAN2_DROME F	11957435	BGLA_THEMA Fu	445248 nc	2J78	3D51	2J7H		Sc=5.76107, min distance = 2.40013
MAN2_DROME F	11957435	BGLA_THEMA Fu	9549193 C	2CES	3D4Z	2J7H		Sc=5.76449, min distance = 2.44469
MAN2_DROME F	151504 D-	CVN_NOSEL Ful	185698 al	2RDK	3CZS	2PYS		Sc=5.90715, min distance = 2.06258
MAN2_DROME F	444885 O-	Q309D1_9AGAR	24139 ace	2C4D	1PS3	2C25	0.79	Sc=6.30307, min distance = 0.53683
MAN2_DROME F	447413 NT	BGLA_THEMA Fu	445248 nc	2J78	3D51	2J7B		Sc=6.02841, min distance = 2.16687
MAN2_DROME F	447413 NT	MYRA_SINAL Fu	445248 nc	1E6S	3D51	1E6Q		Sc=6.02177, min distance = 2.11033
MAN2_DROME F	447651 CI	BGLA_THEMA Fu	9549193 C	2CES	3D4Z	2J77		Sc=5.89491, min distance = 2.01648
MAN2_DROME F	448963 2j	BGLA_THEMA Fu	9549193 C	2CES	3D4Z	2J79		Sc=6.0263, min distance = 2.011839
MAN2_DROME F	449101 nc	BGLA_THEMA Fu	9549193 C	2CES	3D4Z	1W3J		Sc=5.70118, min distance = 2.56113
MAN2_DROME F	64947 alp	LEC_PEA Full=	185698 al	1RIN	3CZS	1HKD	0.94	Sc=5.89127, min distance = 2.05231
MAN2_DROME F	9549194 C	BGAL_SULSO Fu	445248 nc	1UWU	3D51	2CER		Sc=6.11185, min distance = 2.00048
MAOM_ASCSU F	11987786	LDH_PLAFD Ful	5893 nadi	1T2D	1O0S	1T2E	0.77	Sc=6.25291, min distance = 2.07743
MAOM_ASCSU F	439153 Di	ADH1B_HUMAN F	5893 nadi	1U3U	1O0S	1DEH	0.79	Sc=6.26114, min distance = 1.67261
MAOM_ASCSU F	440516 CI	MDH_THETH Ful	5893 nadi	1BMD	1O0S	1BDM	0.79	Sc=5.81134, min distance = 2.00054
MAOM_ASCSU F	446288 NA	G3P_PALVE Ful	5893 nadi	1DSS	1O0S	1IHX		Sc=6.71496, min distance = 2.15293

MAOM_ASCSU Fu	5957 Aden	MAOM_HUMAN Ful	5893 nadi	1PJ3	1O0S	1GZ4		Sc=6.4897, min distance = 0.529400
MAOM_ASCSU Fu	6022 Aden	Q5SI02_THET8 I	5893 nadi	2BJK	1O0S	2BJA		Sc=6.18118, min distance = 1.87033
MAOM_HUMAN Fu	11987786	LDH_PLAFD Ful	5893 nadi	1T2D	1PJ3	1T2E	0.77	Sc=6.77014, min distance = 2.51538
MAOM_HUMAN Fu	123927 A3	DAPB_ECOLI Ful	5893 nadi	1DRU	1PJ3	1DRV	0.97	Sc=6.3253, min distance = 2.136734
MAOM_HUMAN Fu	159296 1v	RNAS2_HUMAN Fu	5957 Aden	2C05	1GZ4	1HI4	0.99	Sc=5.9615, min distance = 1.419890
MAOM_HUMAN Fu	3540 1yds	KAPCA_BOVIN Fu	5957 Aden	1Q24	1GZ4	1YDS		Sc=5.90878, min distance = 2.18697
MAOM_HUMAN Fu	4369002 C	LDHA_PIG Full	5893 nadi	9LDB	1PJ3	9LDT	0.77	Sc=6.7695, min distance = 2.506630
MAOM_HUMAN Fu	439153 Di	ADH1E_HORSE Fu	5893 nadi	1MGO	1PJ3	2JHF	0.79	Sc=6.31926, min distance = 2.43684
MAOM_HUMAN Fu	439153 Di	CTBP1_RAT Ful	5893 nadi	1HKU	1PJ3	1HL3	0.79	Sc=6.32869, min distance = 2.29266
MAOM_HUMAN Fu	440516 CI	MDH_THETH Ful	5893 nadi	1BMD	1PJ3	1BDM	0.79	Sc=6.77334, min distance = 2.41642
MAOM_HUMAN Fu	446288 NA	G3P_PALVE Ful	5893 nadi	1DSS	1PJ3	1IHX		Sc=6.77468, min distance = 2.15810
MAOM_HUMAN Fu	449240 1y	KAPCA_BOVIN Fu	5957 Aden	1Q24	1GZ4	1YDR		Sc=5.97177, min distance = 1.61615
MAOM_HUMAN Fu	6022 Aden	GLGS_SOLTU Ful	5957 Aden	1YP3	1GZ4	1YP4	0.99	Sc=6.40657, min distance = 1.63320
MAOM_HUMAN Fu	6022 Aden	MUTS_ECOLI Ful	5957 Aden	1W7A	1GZ4	1OH7	0.99	Sc=6.40508, min distance = 2.15495
MAOM_HUMAN Fu	6022 Aden	PURK_ECOLI Ful	5957 Aden	3ETH	1GZ4	3ETJ	0.99	Sc=5.94234, min distance = 1.54141
MAOM_HUMAN Fu	6022 Aden	Q72H90_THET2 I	5957 Aden	2BEK	1GZ4	2BEJ	0.99	Sc=6.39485, min distance = 1.94508
MAOM_HUMAN Fu	6083 aden	PURP_PYRFU Ful	5957 Aden	2R86	1GZ4	2R85	0.98	Sc=6.36157, min distance = 1.39757
MAOX_COLLI Fu	440141 9i	DHB1_HUMAN Ful	5886 NADP	1QYV	1GQ2	1QYW		Sc=5.92439, min distance = 2.25825
MAOX_COLLI Fu	440141 9i	G6PD_LEUME Ful	5886 NADP	1H9A	1GQ2	1E7Y		Sc=6.35154, min distance = 1.88998
MAPK2_HUMAN I	10109823	PIM1_HUMAN Ful	444345 1c	1YHS	2PZY	3CY3	36.72	Sc=6.41041, min distance = 2.57831
MAPK2_HUMAN I	10224714	CDK2_HUMAN Ful	444345 1c	1AQ1	2PZY	3DDP	35.5	Sc=6.49515, min distance = 2.00459
MAPK2_HUMAN I	11314340	KAPCA_BOVIN Fu	444345 1c	1STC	2PZY	2UZT	35.94	Sc=6.33163, min distance = 2.42624
MAPK2_HUMAN I	11348631	CHK1_HUMAN Ful	444345 1c	1NVR	2PZY	2E9U	33.67	Sc=6.18391, min distance = 2.83617
MAPK2_HUMAN I	11502647	CHK1_HUMAN Ful	444345 1c	1NVR	2PZY	2GDO	33.67	Sc=6.33232, min distance = 2.20256
MAPK2_HUMAN I	11553058	LCK_HUMAN Ful	444345 1c	1QPD	2PZY	2OF2	26.63	Sc=6.27252, min distance = 2.26375
MAPK2_HUMAN I	11957417	CDK2_HUMAN Ful	444345 1c	1AQ1	2PZY	2I40	35.5	Sc=6.31233, min distance = 2.09338
MAPK2_HUMAN I	1540 1h1q	CDK2_HUMAN Ful	444345 1c	1AQ1	2PZY	1H1Q	35.5	Sc=6.20919, min distance = 2.64983
MAPK2_HUMAN I	15602982	KAPCA_BOVIN Fu	444345 1c	1STC	2PZY	2UW6	35.94	Sc=6.04691, min distance = 2.25547
MAPK2_HUMAN I	15942652	CDK2_HUMAN Ful	444345 1c	1AQ1	2PZY	2DUV	35.5	Sc=6.28733, min distance = 2.13622
MAPK2_HUMAN I	160355 rc	CDK2_HUMAN Ful	444345 1c	1AQ1	2PZY	3DDQ	35.5	Sc=6.3191, min distance = 2.256827
MAPK2_HUMAN I	16122608	PDPK1_HUMAN Fu	444345 1c	1OKY	2PZY	2PE1	30.68	Sc=6.2882, min distance = 2.917034
MAPK2_HUMAN I	16122643	CHK1_HUMAN Ful	444345 1c	1NVR	2PZY	2YWP	33.67	Sc=6.22784, min distance = 2.72859
MAPK2_HUMAN I	16214823	CDK2_HUMAN Ful	444345 1c	1AQ1	2PZY	2UZB	35.5	Sc=6.03978, min distance = 2.18008
MAPK2_HUMAN I	16214828	CDK2_HUMAN Ful	444345 1c	1AQ1	2PZY	2UZO	35.5	Sc=5.95912, min distance = 2.68606
MAPK2_HUMAN I	16758227	CHK1_HUMAN Ful	444345 1c	1NVR	2PZY	2R0U	33.67	Sc=6.51608, min distance = 2.49777
MAPK2_HUMAN I	17754396	PDPK1_HUMAN Fu	444345 1c	1OKY	2PZY	2R7B	30.68	Sc=6.25043, min distance = 2.73733
MAPK2_HUMAN I	1907917 2	CHK1_HUMAN Ful	444345 1c	1NVR	2PZY	2CGW	33.67	Sc=6.16831, min distance = 2.46465

MAPK2_HUMAN	23653515	CDK2_HUMAN Fu	444345 1c 1AQ1	2PZY	2R3F	35.5	Sc=5.91833, min distance = 2.80851
MAPK2_HUMAN	23653518	CDK2_HUMAN Fu	444345 1c 1AQ1	2PZY	2R3J	35.5	Sc=6.14228, min distance = 2.71199
MAPK2_HUMAN	23653519	CDK2_HUMAN Fu	444345 1c 1AQ1	2PZY	2R3K	35.5	Sc=6.04487, min distance = 2.60144
MAPK2_HUMAN	23653520	CDK2_HUMAN Fu	444345 1c 1AQ1	2PZY	2R3L	35.5	Sc=6.023, min distance = 2.7559646
MAPK2_HUMAN	23653521	CDK2_HUMAN Fu	444345 1c 1AQ1	2PZY	2R3M	35.5	Sc=6.30724, min distance = 2.16516
MAPK2_HUMAN	23653524	CDK2_HUMAN Fu	444345 1c 1AQ1	2PZY	2R3P	35.5	Sc=6.24674, min distance = 2.07865
MAPK2_HUMAN	23653526	CDK2_HUMAN Fu	444345 1c 1AQ1	2PZY	2R3R	35.5	Sc=6.11186, min distance = 2.68762
MAPK2_HUMAN	23657800	CHK1_HUMAN Fu	444345 1c 1NVR	2PZY	2E9P	33.67	Sc=6.22853, min distance = 2.44648
MAPK2_HUMAN	23727982	CDK2_HUMAN Fu	444345 1c 1AQ1	2PZY	3BHU	35.5	Sc=6.24477, min distance = 1.93989
MAPK2_HUMAN	24752838	CHK1_HUMAN Fu	444345 1c 1NVR	2PZY	2QHM	33.67	Sc=6.05142, min distance = 2.85425
MAPK2_HUMAN	24864081	CDK2_HUMAN Fu	444345 1c 1AQ1	2PZY	2VTS	35.5	Sc=6.25367, min distance = 2.26018
MAPK2_HUMAN	24905144	PK3CG_HUMAN Fu	444345 1c 1E8Z	2PZY	3ENE		Sc=6.16603, min distance = 2.79631
MAPK2_HUMAN	24916751	CDK2_HUMAN Fu	444345 1c 1AQ1	2PZY	3DOG	35.5	Sc=6.23524, min distance = 2.08422
MAPK2_HUMAN	33113 gam	IRAK4_HUMAN Fu	444345 1c 2NRY	2PZY	2OID	34.62	Sc=5.99139, min distance = 1.99989
MAPK2_HUMAN	3543 1g5s	CDK2_HUMAN Fu	444345 1c 1AQ1	2PZY	1G5S	35.5	Sc=6.40897, min distance = 2.13174
MAPK2_HUMAN	3547 Fasu	KAPCA_BOVIN Fu	444345 1c 1STC	2PZY	1Q8W	35.94	Sc=5.89062, min distance = 2.72627
MAPK2_HUMAN	3973 nche	PIM1_HUMAN Fu	444345 1c 1YHS	2PZY	1YI3	36.72	Sc=5.8758, min distance = 2.207117
MAPK2_HUMAN	4369136 C	CDK2_HUMAN Fu	444345 1c 1AQ1	2PZY	1DM2	35.5	Sc=6.22322, min distance = 3.05757
MAPK2_HUMAN	445966 O6	CDK2_HUMAN Fu	444345 1c 1AQ1	2PZY	1H0V	35.5	Sc=6.03133, min distance = 2.70626
MAPK2_HUMAN	447649 1c	CDK2_HUMAN Fu	444345 1c 1AQ1	2PZY	1OI9	35.5	Sc=6.2868, min distance = 2.800629
MAPK2_HUMAN	447655 1c	CDK2_HUMAN Fu	444345 1c 1AQ1	2PZY	1OIU	35.5	Sc=6.21979, min distance = 2.80088
MAPK2_HUMAN	447766 1p	CDK2_HUMAN Fu	444345 1c 1AQ1	2PZY	1P2A	35.5	Sc=6.24771, min distance = 2.28037
MAPK2_HUMAN	448043 2g	KAPCA_BOVIN Fu	444345 1c 1STC	2PZY	1Q8U	35.94	Sc=5.95001, min distance = 2.61672
MAPK2_HUMAN	448293 CI	CDK2_HUMAN Fu	444345 1c 1AQ1	2PZY	1R78	35.5	Sc=6.40141, min distance = 2.13060
MAPK2_HUMAN	449240 1y	KAPCA_BOVIN Fu	444345 1c 1STC	2PZY	1YDR	35.94	Sc=5.88549, min distance = 2.60526
MAPK2_HUMAN	4565 1h1r	CDK2_HUMAN Fu	444345 1c 1AQ1	2PZY	1H1R	35.5	Sc=6.21571, min distance = 2.80356
MAPK2_HUMAN	4566 1h1s	CDK2_HUMAN Fu	444345 1c 1AQ1	2PZY	1H1S	35.5	Sc=6.10593, min distance = 2.88551
MAPK2_HUMAN	5288641 i	CDK2_HUMAN Fu	444345 1c 1AQ1	2PZY	1E9H	35.5	Sc=6.20471, min distance = 2.34799
MAPK2_HUMAN	5326739 i	GSK3B_HUMAN Fu	444345 1c 1Q3D	2PZY	1Q41	37.97	Sc=6.32316, min distance = 1.63155
MAPK2_HUMAN	5327121 I	PIM1_HUMAN Fu	444345 1c 1YHS	2PZY	2C3I	36.72	Sc=6.23388, min distance = 2.08574
MAPK2_HUMAN	5327123 I	CHK1_HUMAN Fu	444345 1c 1NVR	2PZY	2C3L	33.67	Sc=6.05208, min distance = 2.64139
MAPK2_HUMAN	5331010 c	CDK2_HUMAN Fu	444345 1c 1AQ1	2PZY	2BKZ	35.5	Sc=6.2569, min distance = 2.289868
MAPK2_HUMAN	5494414 C	CDK2_HUMAN Fu	444345 1c 1AQ1	2PZY	2A0C	35.5	Sc=6.39749, min distance = 2.18647
MAPK2_HUMAN	6083 ader	PIM1_HUMAN Fu	444345 1c 1YHS	2PZY	1YXU	36.72	Sc=6.32215, min distance = 1.79109
MAPK2_HUMAN	6420139 C	CDK2_HUMAN Fu	444345 1c 1AQ1	2PZY	2C5V	35.5	Sc=6.03787, min distance = 2.46460
MAPK2_HUMAN	6852166	PK3CG_HUMAN Fu	444345 1c 1E8Z	2PZY	2CHX		Sc=6.18623, min distance = 2.16220
MAPK2_HUMAN	6852207 M	KAPCA_BOVIN Fu	444345 1c 1STC	2PZY	2GNI	35.94	Sc=5.89062, min distance = 2.03585

MAPK2_HUMAN I	72271 7-H	CHK1_HUMAN Fu.	444345 1c	1NVR	2PZY	1NVQ	33.67	0.91	Sc=6.44148, min distance = 2.653016
MAPK2_HUMAN I	72271 7-H	PDPK1_HUMAN Fu	444345 1c	1OKY	2PZY	1OKZ	30.68	0.91	Sc=6.43142, min distance = 2.605292
MAPK2_HUMAN I	9547890 I	CDK2_HUMAN Fu.	444345 1c	1AQ1	2PZY	1W8C	35.5		Sc=6.16626, min distance = 2.618025
MARR_ECOLI Fu	3059 difl	ALBU_HUMAN Fu.	338 salic	2I2Z	1JGS	2BXE		0.86	Sc=5.99967, min distance = 0.918608
MARR_ECOLI Fu	8631 3,5-	ALBU_HUMAN Fu.	338 salic	2I2Z	1JGS	2BXL			Sc=5.64022, min distance = 2.132409
MASZ_ECOLI Fu	445892 1g	ACES_TORCA Fu.	8200 TETR	1DX6	1P7T	1GQR			Sc=5.61111, min distance = 0.760939
MASZ_MYCTU Fu	5962 L-ly	RBCMT_PEA Ful.	23831 HEP	1MLV	2GQ3	2H2E			Sc=5.60208, min distance = 1.850666
MBL1_RAT Ful.	445948 D-	Q9HYN5_PSEAE I	446578 1r	2JDP	1KWW	1OXC			Sc=5.69341, min distance = 1.993031
MBL1_RAT Ful.	6102754 2	Q9HYN5_PSEAE I	444863 is	1OXC	1KWX	2BOJ		0.9	Sc=5.60554, min distance = 1.768365
MBL2_RAT Ful.	101798 Me	MBL1_RAT Full=	82313 1rd	1KWV	1RDN	1KWU			Sc=5.73074, min distance = 2.050798
MBL2_RAT Ful.	101798 Me	Q9HYN5_PSEAE I	444863 is	1OXC	1RDJ	2JDN		0.96	Sc=5.68353, min distance = 1.901978
MCEL_VACCW Fu	12771338	MCES_HUMAN Fu.	439155 Ad	3BGV	2VDW	3EPP		0.88	Sc=6.58436, min distance = 1.368699
MCEL_VACCW Fu	446535 CI	HNMT_HUMAN Fu.	439155 Ad	2AOT	2VDW	1JQE		0.95	Sc=6.54215, min distance = 1.519334
MCEL_VACCW Fu	65482 sir	ERM_BACSU Ful.	439155 Ad	1QAN	2VDW	1QAQ		0.88	Sc=6.58646, min distance = 1.679811
MCEL_VACCW Fu	65482 sir	MTR1_RHOSH Fu.	439155 Ad	1NW7	2VDW	1NW6		0.88	Sc=6.12309, min distance = 1.912235
MCES_ENCCU Fu	445762 5'	MTTA_THEAQ Fu.	34756 Acy	2ADM	1RI4	2IH2		0.93	Sc=6.31156, min distance = 1.845727
MCES_ENCCU Fu	445971 CI	COMT_RAT Full=	34756 Acy	2CL5	1RI4	1H1D		0.92	Sc=6.49998, min distance = 2.257355
MCES_ENCCU Fu	446535 CI	HNMT_HUMAN Fu.	439155 Ad	2AOT	1RI1	1JQE		0.95	Sc=6.04259, min distance = 2.302631
MCES_ENCCU Fu	5326531 C	CHOMT_MEDSA Fu	34756 Acy	1FPQ	1RI4	1FP1		0.92	Sc=6.50451, min distance = 2.115561
MCES_ENCCU Fu	60961 ade	PIMT_PYRFU Fu.	439155 Ad	1JG1	1RI1	1JG2		0.84	Sc=6.24966, min distance = 2.925409
MCES_HUMAN Fu	188380 Ac	MCES_ENCCU Fu.	439155 Ad	1RI1	3BGV	1Z3C		0.91	Sc=6.49921, min distance = 2.122626
MCES_HUMAN Fu	446535 CI	HNMT_HUMAN Fu.	439155 Ad	2AOT	3BGV	1JQE		0.95	Sc=6.45007, min distance = 2.528201
MCES_HUMAN Fu	60961 ade	PIMT_PYRFU Fu.	439155 Ad	1JG1	3BGV	1JG2		0.84	Sc=6.2103, min distance = 2.7224108
MCES_HUMAN Fu	65482 sir	MCES_ENCCU Fu.	439155 Ad	1RI1	3BGV	2HV9		0.88	Sc=6.04309, min distance = 2.055669
MCES_HUMAN Fu	65482 sir	MTR1_RHOSH Fu.	439155 Ad	1NW7	3BGV	1NW6		0.88	Sc=5.77058, min distance = 2.178157
MCES_HUMAN Fu	65482 sir	PRMA_THET8 Fu.	439155 Ad	3EGV	3BGV	2ZBR		0.88	Sc=6.07411, min distance = 2.750596
MCE_PBCV1 Fu.	440641 1m	F261_RAT Full=	6830 guan	1C80	1CKM	1TIP			Sc=5.88189, min distance = 1.332871
MCR_HUMAN Fu.	11987686	P71278_ENTCL I	5994 prog	2ABA	2AA6	1H61			Sc=6.44096, min distance = 2.305238
MCR_HUMAN Fu.	11987785	PRGR_HUMAN Fu.	5994 prog	1A28	2AA6	1SQN	56.85	0.78	Sc=6.08782, min distance = 1.781169
MCR_HUMAN Fu.	13472 Bol	P71278_ENTCL I	5994 prog	2ABA	2AA6	1H62		0.96	Sc=6.02791, min distance = 2.595878
MCR_HUMAN Fu.	24860529	AK1D1_HUMAN Fu	5865 pred	3CMF	2AAX	3CAV			Sc=6.09595, min distance = 1.779432
MCR_HUMAN Fu.	261000 Me	PRGR_HUMAN Fu.	5994 prog	1A28	2AA6	1E3K	56.85		Sc=6.0593, min distance = 1.6429681
MCR_HUMAN Fu.	4369524 T	PRGR_HUMAN Fu.	5994 prog	1A28	2AA6	1ZUC	56.85		Sc=6.01578, min distance = 1.460965
MCR_HUMAN Fu.	445033 CI	P71278_ENTCL I	5994 prog	2ABA	2AA6	1H60		0.8	Sc=6.09701, min distance = 2.769351
MDHC_PIG Ful.	11987786	LDH_PLAFD Ful.	5893 nadi	1T2D	5MDH	1T2E		0.77	Sc=6.3076, min distance = 2.3823192
MDHC_PIG Ful.	4369002 C	LDHA_PIG Full=	5893 nadi	9LDB	5MDH	9LDT		0.77	Sc=6.30671, min distance = 2.217818
MDHC_PIG Ful.	439153 Di	ADH1B_HUMAN Fu	5893 nadi	1U3U	5MDH	1DEH		0.79	Sc=6.75142, min distance = 2.040943

MDHC_PIG Ful	439153 Di	CTBP1_RAT Ful	5893 nadi 1HKU	5MDH	1HL3	0.79	Sc=6.75057, min distance = 1.60128
MDHC_PIG Ful	440141 9i	G6PD_LEUME Fu	5893 nadi 1H94	5MDH	1E7Y		Sc=5.64484, min distance = 2.00740
MDHC_PIG Ful	440516 CI	MDH_THETH Ful	5893 nadi 1BMD	5MDH	1BDM	0.79	Sc=6.75794, min distance = 2.43499
MDHC_PIG Ful	445794 AI	ADHX_HUMAN Fu	5893 nadi 2FZW	5MDH	2FZE		Sc=6.59616, min distance = 2.47458
MDHC_PIG Ful	6083 ader	NADE_ECOLI Fu	5893 nadi 1WXH	5MDH	1WXI		Sc=6.29104, min distance = 2.22047
MDHG_CITLA Fu	51 2-Oxop	SERA_ECOLI Fu	311 citri 2P9E	1SMK	1YBA	0.76	Sc=5.71244, min distance = 1.22170
MDHM_HUMAN Fu	11987786	LDH_PLAFD Ful	5893 nadi 1T2D	2DFD	1T2E	0.77	Sc=6.33328, min distance = 2.45858
MDHM_HUMAN Fu	123927 A3	DAPB_ECOLI Fu	5893 nadi 1DRU	2DFD	1DRV	0.97	Sc=6.76809, min distance = 2.14279
MDHM_HUMAN Fu	440516 CI	MDH_THETH Ful	5893 nadi 1BMD	2DFD	1BDM	0.79	Sc=6.78437, min distance = 2.64774
MDHM_HUMAN Fu	445794 AI	ADHX_HUMAN Fu	5893 nadi 2FZW	2DFD	2FZE		Sc=6.62283, min distance = 2.60129
MDHM_HUMAN Fu	6102709 A	LDH_PLAFD Ful	5893 nadi 1T2D	2DFD	2A94	0.88	Sc=6.76479, min distance = 2.54885
MDHM_HUMAN Fu	6420113 C	MDH_THETH Ful	5893 nadi 1BMD	2DFD	1WZI	0.77	Sc=6.2151, min distance = 1.9098518
MDHM_PIG Ful	12136 Ber	POL_HV1N5 Ful	311 citri 2GON	1MLD	9HVP		Sc=5.65082, min distance = 1.45324
MDHM_PIG Ful	439183 1i	TPIS_TRYBB Fu	311 citri 2VEK	1MLD	1IIH		Sc=5.63798, min distance = 2.30622
MDHM_PIG Ful	445607 AT	ACON_BOVIN Fu	311 citri 1C96	1MLD	1FGH		Sc=5.93582, min distance = 1.55827
MDHM_PIG Ful	447376 2-	ACON_BOVIN Fu	311 citri 1C96	1MLD	1NIS		Sc=5.83924, min distance = 1.45664
MDHM_PIG Ful	51 2-Oxop	SERA_ECOLI Fu	311 citri 2P9E	1MLD	1YBA	0.76	Sc=5.68485, min distance = 2.81278
MDH_AQUAR Fu	439153 Di	ADH1B_HUMAN Fu	5893 nadi 1U3U	1B8V	1DEH	0.79	Sc=6.30496, min distance = 2.37432
MDH_AQUAR Fu	445794 AI	ADHX_HUMAN Fu	5893 nadi 2FZW	1B8V	2FZE		Sc=6.12919, min distance = 2.60476
MDH_CHLAA Fu	439153 Di	ADH1B_HUMAN Fu	5893 nadi 1U3U	1UR5	1DEH	0.79	Sc=6.30727, min distance = 2.26443
MDH_CHLAA Fu	440516 CI	MDH_THETH Ful	5893 nadi 1BMD	1UR5	1BDM	0.79	Sc=6.32415, min distance = 2.48969
MDH_CHLAA Fu	446288 NA	G3P_PALVE Ful	5893 nadi 1DSS	1UR5	1IHX		Sc=6.79525, min distance = 0.67201
MDH_CHLTE Fu	169266 1,	GALE_HUMAN Fu	5893 nadi 1HZJ	1GUZ	1I3K	0.79	Sc=6.75955, min distance = 1.69111
MDH_CHLTE Fu	439153 Di	ADH1E_HORSE Fu	5893 nadi 1MGO	1GUZ	2JHF	0.79	Sc=6.74086, min distance = 2.66177
MDH_CHLTE Fu	439153 Di	CTBP1_RAT Ful	5893 nadi 1HKU	1GUZ	1HL3	0.79	Sc=6.75501, min distance = 2.42122
MDH_CHLTE Fu	440141 9i	G6PD_LEUME Fu	5893 nadi 1H94	1GUZ	1E7Y		Sc=5.64194, min distance = 1.88462
MDH_CHLTE Fu	440516 CI	MDH_THETH Ful	5893 nadi 1BMD	1GUZ	1BDM	0.79	Sc=6.32037, min distance = 2.56159
MDH_CHLTE Fu	445794 AI	ADHX_HUMAN Fu	5893 nadi 2FZW	1GUZ	2FZE		Sc=6.60035, min distance = 2.62755
MDH_CHLTE Fu	446288 NA	G3P_PALVE Ful	5893 nadi 1DSS	1GUZ	1IHX		Sc=6.75542, min distance = 1.97829
MDH_CHLVI Fu	123927 A3	DAPB_ECOLI Fu	5893 nadi 1DRU	1GUZ	1DRV	0.97	Sc=6.00753, min distance = 2.10094
MDH_CHLVI Fu	169266 1,	GALE_HUMAN Fu	5893 nadi 1HZJ	1GUZ	1I3K	0.79	Sc=6.75955, min distance = 1.69111
MDH_CHLVI Fu	439153 Di	ADH1E_HORSE Fu	5893 nadi 1MGO	1GUZ	2JHF	0.79	Sc=6.74086, min distance = 2.66177
MDH_CHLVI Fu	439153 Di	CTBP1_RAT Ful	5893 nadi 1HKU	1GUZ	1HL3	0.79	Sc=6.75501, min distance = 2.42122
MDH_CHLVI Fu	440516 CI	MDH_THETH Ful	5893 nadi 1BMD	1GUZ	1BDM	0.79	Sc=6.32037, min distance = 2.56159
MDH_CHLVI Fu	445794 AI	ADHX_HUMAN Fu	5893 nadi 2FZW	1GUZ	2FZE		Sc=6.60035, min distance = 2.62755
MDH_CHLVI Fu	446288 NA	G3P_PALVE Ful	5893 nadi 1DSS	1GUZ	1IHX		Sc=6.75542, min distance = 1.97829
MDH_ECOLI Fu	11987786	LDH_PLAFD Ful	5893 nadi 1T2D	1EMD	1T2E	0.77	Sc=6.33828, min distance = 2.48660

MDH_ECOLI Fu	439153 Di	ADH1B_HUMAN F	5893 nadi	1U3U	1EMD	1DEH		0.79	Sc=6.7781, min distance = 2.357037
MDH_ECOLI Fu	439153 Di	ADH1E_HORSE F	5893 nadi	1MGO	1EMD	2JHF		0.79	Sc=6.76491, min distance = 2.524282
MDH_ECOLI Fu	439153 Di	CTBP1_RAT Ful	5893 nadi	1HKU	1EMD	1HL3		0.79	Sc=6.77913, min distance = 2.253656
MDH_ECOLI Fu	440516 CI	MDH_THETH Ful	5893 nadi	1BMD	1EMD	1BDM		0.79	Sc=6.78767, min distance = 2.106737
MDH_ECOLI Fu	445794 AD	ADHX_HUMAN Fu	5893 nadi	2FZW	1EMD	2FZE			Sc=6.63239, min distance = 2.589513
MDH_HALMA Fu	11987786	LDH_PLAFD Ful	5893 nadi	1T2D	1O6Z	1T2E		0.77	Sc=6.29237, min distance = 2.325256
MDH_HALMA Fu	123927 A3	DAPB_ECOLI Fu	5893 nadi	1DRU	1O6Z	1DRV		0.97	Sc=6.72286, min distance = 1.986530
MDH_HALMA Fu	439153 Di	ADH1E_HORSE F	5893 nadi	1MGO	1O6Z	2JHF		0.79	Sc=6.73389, min distance = 2.572035
MDH_HALMA Fu	439153 Di	CTBP1_RAT Ful	5893 nadi	1HKU	1O6Z	1HL3		0.79	Sc=6.74943, min distance = 1.639442
MDH_HALMA Fu	439153 Di	Q9BJJ9_PLAFA	5893 nadi	1UH5	1O6Z	1V35		0.79	Sc=6.74888, min distance = 1.977115
MDH_HALMA Fu	440516 CI	MDH_THETH Ful	5893 nadi	1BMD	1O6Z	1BDM		0.79	Sc=6.30671, min distance = 2.479224
MDH_HALMA Fu	445794 AD	G3P_PALVE Ful	5893 nadi	1DSS	1O6Z	1IHY			Sc=6.65359, min distance = 1.463862
MDH_HALMA Fu	446288 NA	G3P_PALVE Ful	5893 nadi	1DSS	1O6Z	1IHX			Sc=6.7282, min distance = 2.1418302
MDH_HALMA Fu	6102710 A	Q4PRK9_PLAVI	5893 nadi	2A92	1O6Z	2AA3		0.95	Sc=6.74182, min distance = 2.586528
MDH_METJA Fu	440141 9i	DHB1_HUMAN Fu	5886 NADP	1QYV	1HYG	1QYW			Sc=5.90466, min distance = 2.095132
MDH_METJA Fu	440141 9i	G6PD_LEUME Fu	5886 NADP	1H9A	1HYG	1E7Y			Sc=6.34315, min distance = 2.247424
MDH_METJA Fu	440141 9i	GSHR_HUMAN Fu	5886 NADP	3DJJ	1HYG	1GRA			Sc=5.99431, min distance = 2.260409
MDH_METJA Fu	445473 NA	GFO_ZYMMO Ful	5886 NADP	1H6D	1HYG	1RYD		0.95	Sc=6.40624, min distance = 2.032984
MDH_THETH Fu	11987786	LDH_PLAFD Ful	5893 nadi	1T2D	1BMD	1T2E		0.77	Sc=6.73957, min distance = 2.680741
MDH_THETH Fu	439153 Di	ADH1B_HUMAN F	5893 nadi	1U3U	1BMD	1DEH		0.79	Sc=6.75183, min distance = 2.397756
MDLB_PSEPU F	444302 4-	GOX_SPIOL Ful	444243 FA	1GOX	1P4C	1AL7	41.33		Sc=6.20737, min distance = 2.273168
MDLB_PSEPU F	446995 FM	CYB2_YEAST Fu	444243 FA	1KBI	1P4C	1LTD	32.32	0.77	Sc=6.21601, min distance = 2.134492
MDLC_PSEPU F	10364977	OXC_OXAFO Ful	124687 1r	2C31	1Q6Z	2JI6	23.09		Sc=6.46474, min distance = 2.552184
MDLC_PSEPU F	13294447	DCIP_AZOBR Fu	1132 thia	2NXW	1BFD	2Q5Q	24.76		Sc=5.74726, min distance = 2.396727
MDLC_PSEPU F	445886 CI	TKT1_YEAST Fu	1132 thia	1TRK	1BFD	1GPU		0.91	Sc=5.77523, min distance = 2.169371
MDLC_PSEPU F	448671 2-	ILVB_YEAST Fu	1132 thia	1N0H	1BFD	1T9B	27.04		Sc=5.61588, min distance = 2.900874
MDLC_PSEPU F	448673 PE	ILVB_YEAST Fu	1132 thia	1N0H	1BFD	1T9B	27.04		Sc=5.8115, min distance = 2.3296106
MDLC_PSEPU F	448721 N3	TKT1_YEAST Fu	1132 thia	1TRK	1BFD	1TKA			Sc=6.43887, min distance = 2.181331
MDLC_PSEPU F	448723 1t	TKT1_YEAST Fu	1132 thia	1TRK	1BFD	1TKC		0.77	Sc=6.46245, min distance = 2.352222
MDLC_PSEPU F	6102647 C	ODBA_HUMAN Fu	1132 thia	2BFD	1BFD	1WCI		0.9	Sc=6.12023, min distance = 2.162836
MDLC_PSEPU F	6102751 T	ODBA_HUMAN Fu	124687 1r	2BFF	1Q6Z	2BEW			Sc=6.15437, min distance = 2.344750
MDLC_PSEPU F	6518182 C	POXB_LACPL Fu	1132 thia	2EZ4	1BFD	2EZ9	24.86	0.94	Sc=5.81146, min distance = 2.258992
MDLC_PSEPU F	6518187 2	POXB_LACPL Fu	1132 thia	2EZ4	1BFD	2EZ8	24.86	0.87	Sc=5.81027, min distance = 2.226748
ME11A_HUMAN	12771338	MCES_HUMAN Fu	439155 Ad	3BGV	2EX4	3EPP		0.88	Sc=6.59138, min distance = 2.282235
ME11A_HUMAN	188380 Ad	MCES_ENCCU Fu	439155 Ad	1RI1	2EX4	1Z3C		0.91	Sc=6.59297, min distance = 2.152831
ME11A_HUMAN	446535 CI	HNMT_HUMAN Fu	439155 Ad	2AOT	2EX4	1JQE		0.95	Sc=6.5443, min distance = 2.1474231
ME11A_HUMAN	60961 ade	PIMT_PYRFU Fu	439155 Ad	1JG1	2EX4	1JG2		0.84	Sc=6.33515, min distance = 2.663663

ME11A_HUMAN I	6420137 C	O85726_STRCL I	439155 Ad	2BR5	2EX4	2BR4			Sc=6.05952, min distance = 0.891305
ME11A_HUMAN I	65482 sir	ERM_BACSU Ful	439155 Ad	1QAN	2EX4	1QAQ		0.88	Sc=6.58253, min distance = 2.262545
ME11A_HUMAN I	65482 sir	MCES_ENCCU Fu	439155 Ad	1RI1	2EX4	2HV9		0.88	Sc=6.58743, min distance = 2.161335
ME11A_HUMAN I	65482 sir	MTR1_RHOSH Fu	439155 Ad	1NW7	2EX4	1NW6		0.88	Sc=6.59189, min distance = 2.018050
ME11A_HUMAN I	65482 sir	PRMA_THET8 Fu	439155 Ad	3EGV	2EX4	2ZBR		0.88	Sc=6.58845, min distance = 2.493331
MENC_ECOLI Fu	448580 N-	Q44244_9PSEU I	955 2-Suc	1SJB	1FHV	1SJA			Sc=5.78318, min distance = 1.685214
MENC_ECOLI Fu	448581 N-	Q44244_9PSEU I	955 2-Suc	1SJB	1FHV	1SJC			Sc=6.17032, min distance = 2.017516
MENF_ECOLI Fu	51 2-Oxop	SERA_ECOLI Fu	311 citri	2P9E	3BZM	1YBA		0.76	Sc=5.70422, min distance = 1.679720
MERA_PSEAE Fu	16740985	FRDA_SHEFR Fu	444188 CI	1M64	1ZK7	1E39		0.93	Sc=6.43069, min distance = 2.294470
MERA_PSEAE Fu	444502 CI	FRDA_SHEFR Fu	444188 CI	1M64	1ZK7	1QJD		0.98	Sc=6.43135, min distance = 2.341371
MERA_PSEAE Fu	444502 CI	TYTR_TRYCR Fu	444188 CI	1BZL	1ZK7	1GXF		0.98	Sc=6.43156, min distance = 2.615191
MERA_PSEAE Fu	446013 1,	O28603_ARCFU I	444188 CI	1JNR	1ZK7	1JNZ		0.94	Sc=6.15605, min distance = 2.034084
MERA_PSEAE Fu	449465 CI	GSHR_HUMAN Fu	444188 CI	3DK9	1ZK7	3GRT		0.98	Sc=6.43519, min distance = 2.302737
MERA_PSEAE Fu	6420174 C	O28603_ARCFU I	444188 CI	1JNR	1ZK7	2FJB		0.92	Sc=6.44586, min distance = 1.963121
MERA_PSEAE Fu	6420174 C	O28604_ARCFU I	444188 CI	1JNR	1ZK7	2FJB		0.92	Sc=6.16443, min distance = 1.977784
MET10_HUMAN I	122068 de	HNMT_HUMAN Fu	439155 Ad	2AOT	2H00	2AOU			Sc=6.20726, min distance = 1.073553
MET10_HUMAN I	188380 Ad	MCES_ENCCU Fu	439155 Ad	1RI1	2H00	1Z3C		0.91	Sc=6.55872, min distance = 2.134959
MET10_HUMAN I	446535 CI	HNMT_HUMAN Fu	439155 Ad	2AOT	2H00	1JQE		0.95	Sc=6.51063, min distance = 2.441258
MET10_HUMAN I	60961 ade	PIMT_PYRFU Fu	439155 Ad	1JG1	2H00	1JG2		0.84	Sc=6.28604, min distance = 2.565984
MET10_HUMAN I	65482 sir	ERM_BACSU Ful	439155 Ad	1QAN	2H00	1QAQ		0.88	Sc=6.57185, min distance = 2.063105
MET10_HUMAN I	65482 sir	MCES_ENCCU Fu	439155 Ad	1RI1	2H00	2HV9		0.88	Sc=6.55932, min distance = 2.087595
MET16_YEAST I	8582 Inos	RNAS1_BOVIN Fu	159296 1v	1O0F	2OQ2	1Z6D		0.79	Sc=6.40585, min distance = 1.228977
MET3_PENCH Fu	12876354	PAPS1_HUMAN Fu	10238 Ade	1XNJ	1I2D	2PEZ	41.24	0.94	Sc=6.41892, min distance = 1.312974
METK1_HUMAN I	439182 5'	Q70GK9_STRCT I	34756 Acy	1RQP	2OBV	2CC2		0.86	Sc=6.18561, min distance = 1.682776
METK1_HUMAN I	6852187 2	Q70GK9_STRCT I	34756 Acy	1RQP	2OBV	2CBX		0.84	Sc=6.14196, min distance = 1.337890
METK1_RAT Ful	4565 1h1r	CDK2_HUMAN Fu	6022 Aden	1GY3	1O92	1H1R			Sc=5.73222, min distance = 2.461997
METK1_RAT Ful	6852187 2	O57883_PYRHO I	5957 Aden	2DTO	1O9T	2DTH			Sc=6.041, min distance = 2.16568880
METK2_HUMAN I	439182 5'	Q70GK9_STRCT I	34756 Acy	1RQP	2P02	2CC2		0.86	Sc=6.1964, min distance = 1.6322662
METK2_HUMAN I	6852187 2	Q70GK9_STRCT I	34756 Acy	1RQP	2P02	2CBX		0.84	Sc=6.14715, min distance = 1.318534
MFNA_METJA Fu	445062 CI	HEM1_RHOCA Fu	1051 Code	2BWO	3F9T	2BWP		0.86	Sc=6.33353, min distance = 1.985496
MGSA_ECOLI Fu	311 citri	TPIS_TRYBB Fu	4797 1tpw	1TTJ	1IK4	2VEK			Sc=6.0496, min distance = 2.0015726
MGSA_ECOLI Fu	439183 1i	TPIS_TRYBB Fu	4797 1tpw	1TTJ	1IK4	1IIH			Sc=5.77693, min distance = 2.148712
MIOX_MOUSE Fu	440115 N-	SFTPD_HUMAN Fu	892 myo-i	2OS9	2HUO	2ORJ			Sc=6.15342, min distance = 2.039981
MIOX_MOUSE Fu	441481 ri	TRFL_BOVIN Fu	892 myo-i	2R71	2HUO	2PX1			Sc=5.67266, min distance = 2.318404
MIOX_MOUSE Fu	79025 alp	SFTPD_HUMAN Fu	892 myo-i	2OS9	2HUO	1PWB			Sc=5.96872, min distance = 0.900968
MK01_RAT Ful	27934 May	MK14_HUMAN Fu	5164 3erk	1BL7	3ERK	1W84			Sc=5.9354, min distance = 2.7718401
MK01_RAT Ful	5326871 1	MK14_HUMAN Fu	5164 3erk	1BL7	3ERK	1WBW			Sc=5.97603, min distance = 2.083679

MK12_HUMAN Fu	3064778 H	ROCK1_HUMAN Fu	33113 gam	2V55	1CM8	2ETK			Sc=6.07396, min distance = 1.99603
MK12_HUMAN Fu	3547 Fasu	ROCK1_HUMAN Fu	33113 gam	2V55	1CM8	2ESM			Sc=5.94101, min distance = 2.05232
MK12_HUMAN Fu	448005 CI	GSK3B_HUMAN Fu	33113 gam	1J1B	1CM8	1Q3W			Sc=6.33613, min distance = 2.11936
MK12_HUMAN Fu	5327121 I	PIM1_HUMAN Ful	33113 gam	1XR1	1CM8	2C3I			Sc=6.16042, min distance = 2.13550
MK12_HUMAN Fu	91532 AMP	PURT_ECOLI Ful	33113 gam	1EYZ	1CM8	1KJI		0.99	Sc=5.68057, min distance = 2.24673
MK14_HUMAN Fu	445463 B-	OPSD_BOVIN Ful	62852 B-C	3DQB	2FST	3CAP		0.98	Sc=6.16844, min distance = 0.96748
MKNK2_HUMAN	10109823	PIM1_HUMAN Ful	444345 1c	1YHS	2HW7	3CY3			Sc=6.31796, min distance = 2.79539
MKNK2_HUMAN	10126464	KAPCA_BOVIN Fu	444345 1c	1STC	2HW7	2OH0			Sc=6.41536, min distance = 2.11960
MKNK2_HUMAN	10172943	KAPCA_BOVIN Fu	444345 1c	1STC	2HW7	2JDS			Sc=6.4283, min distance = 2.338988
MKNK2_HUMAN	11175137	KAPCA_BOVIN Fu	444345 1c	1STC	2HW7	2UW7			Sc=6.16193, min distance = 2.44913
MKNK2_HUMAN	11348631	CHK1_HUMAN Ful	444345 1c	1NVR	2HW7	2E9U			Sc=6.12036, min distance = 2.21590
MKNK2_HUMAN	11502647	CHK1_HUMAN Ful	444345 1c	1NVR	2HW7	2GDO			Sc=6.38322, min distance = 2.16120
MKNK2_HUMAN	11696113	KAPCA_BOVIN Fu	444345 1c	1STC	2HW7	2VO3			Sc=6.27109, min distance = 2.30646
MKNK2_HUMAN	1540 1h1q	CDK2_HUMAN Ful	444345 1c	1AQ1	2HW7	1H1Q			Sc=6.24129, min distance = 2.16086
MKNK2_HUMAN	15602983	KAPCA_BOVIN Fu	444345 1c	1STC	2HW7	2UW5			Sc=6.04691, min distance = 2.39746
MKNK2_HUMAN	15942652	CDK2_HUMAN Ful	444345 1c	1AQ1	2HW7	2DUV			Sc=6.28314, min distance = 2.42553
MKNK2_HUMAN	15991572	LCK_HUMAN Full	444345 1c	1QPD	2HW7	2OF4			Sc=6.22992, min distance = 2.29779
MKNK2_HUMAN	16113377	CDK2_HUMAN Ful	444345 1c	1AQ1	2HW7	2W17			Sc=6.3607, min distance = 2.447609
MKNK2_HUMAN	16122643	CHK1_HUMAN Ful	444345 1c	1NVR	2HW7	2YWP			Sc=6.18225, min distance = 2.59961
MKNK2_HUMAN	16214825	CDK2_HUMAN Ful	444345 1c	1AQ1	2HW7	2UZE			Sc=6.22897, min distance = 2.70319
MKNK2_HUMAN	16758227	CHK1_HUMAN Ful	444345 1c	1NVR	2HW7	2R0U			Sc=6.5359, min distance = 2.096980
MKNK2_HUMAN	1707 1fvt	CDK2_HUMAN Ful	444345 1c	1AQ1	2HW7	1FVT			Sc=6.06399, min distance = 2.20670
MKNK2_HUMAN	17754396	PDPK1_HUMAN Fu	444345 1c	1OKY	2HW7	2R7B			Sc=6.17434, min distance = 2.62378
MKNK2_HUMAN	1907917 2	CHK1_HUMAN Ful	444345 1c	1NVR	2HW7	2CGW			Sc=6.00345, min distance = 2.55162
MKNK2_HUMAN	23653515	CDK2_HUMAN Ful	444345 1c	1AQ1	2HW7	2R3F			Sc=5.90348, min distance = 2.33482
MKNK2_HUMAN	23653518	CDK2_HUMAN Ful	444345 1c	1AQ1	2HW7	2R3J			Sc=6.08163, min distance = 2.24046
MKNK2_HUMAN	23653519	CDK2_HUMAN Ful	444345 1c	1AQ1	2HW7	2R3K			Sc=6.08785, min distance = 2.22273
MKNK2_HUMAN	23653520	CDK2_HUMAN Ful	444345 1c	1AQ1	2HW7	2R3L			Sc=6.18861, min distance = 2.17285
MKNK2_HUMAN	23653521	CDK2_HUMAN Ful	444345 1c	1AQ1	2HW7	2R3M			Sc=6.33512, min distance = 2.20739
MKNK2_HUMAN	23653524	CDK2_HUMAN Ful	444345 1c	1AQ1	2HW7	2R3P			Sc=6.0768, min distance = 2.158351
MKNK2_HUMAN	23657800	CHK1_HUMAN Ful	444345 1c	1NVR	2HW7	2E9P			Sc=6.14522, min distance = 2.67980
MKNK2_HUMAN	23727982	CDK2_HUMAN Ful	444345 1c	1AQ1	2HW7	3BHU			Sc=6.12976, min distance = 2.75462
MKNK2_HUMAN	24851689	PIM1_HUMAN Ful	444345 1c	1YHS	2HW7	3CY2			Sc=6.12055, min distance = 2.17869
MKNK2_HUMAN	24901723	CDK2_HUMAN Ful	444345 1c	1AQ1	2HW7	2W06			Sc=6.1296, min distance = 2.465252
MKNK2_HUMAN	24905143	PK3CG_HUMAN Fu	444345 1c	1E8Z	2HW7	2V4L			Sc=5.96904, min distance = 2.97523
MKNK2_HUMAN	24905144	PK3CG_HUMAN Fu	444345 1c	1E8Z	2HW7	3ENE			Sc=6.15222, min distance = 2.78648
MKNK2_HUMAN	24916751	CDK2_HUMAN Ful	444345 1c	1AQ1	2HW7	3DOG			Sc=6.15117, min distance = 2.30549

MKNK2_HUMAN I	24963033	CDK2_HUMAN Fu	444345 1c 1AQ1	2HW7	2W05			Sc=6.17857, min distance = 2.60625
MKNK2_HUMAN I	24978497	CDK2_HUMAN Fu	444345 1c 1AQ1	2HW7	3EID			Sc=6.35584, min distance = 2.42156
MKNK2_HUMAN I	25021197	CDK2_HUMAN Fu	444345 1c 1AQ1	2HW7	3EOC			Sc=6.08874, min distance = 2.62303
MKNK2_HUMAN I	3543 1g5s	CDK2_HUMAN Fu	444345 1c 1AQ1	2HW7	1G5S			Sc=6.42039, min distance = 2.24433
MKNK2_HUMAN I	3547 Fasu	KAPCA_BOVIN Fu	444345 1c 1STC	2HW7	1Q8W			Sc=5.88549, min distance = 2.57036
MKNK2_HUMAN I	4369136 C	CDK2_HUMAN Fu	444345 1c 1AQ1	2HW7	1DM2			Sc=6.14708, min distance = 2.92404
MKNK2_HUMAN I	445966 O6	CDK2_HUMAN Fu	444345 1c 1AQ1	2HW7	1H0V			Sc=6.02212, min distance = 2.40303
MKNK2_HUMAN I	446704 LS	CDK2_HUMAN Fu	444345 1c 1AQ1	2HW7	1KE6			Sc=6.22259, min distance = 2.47165
MKNK2_HUMAN I	447654 CI	CDK2_HUMAN Fu	444345 1c 1AQ1	2HW7	1OIT			Sc=6.10381, min distance = 2.42104
MKNK2_HUMAN I	447766 1p	CDK2_HUMAN Fu	444345 1c 1AQ1	2HW7	1P2A			Sc=6.16375, min distance = 2.39268
MKNK2_HUMAN I	447962 1p	CDK2_HUMAN Fu	444345 1c 1AQ1	2HW7	2C5N			Sc=6.08166, min distance = 2.89609
MKNK2_HUMAN I	449240 1y	KAPCA_BOVIN Fu	444345 1c 1STC	2HW7	1YDR			Sc=5.90321, min distance = 2.42308
MKNK2_HUMAN I	449241 1y	KAPCA_BOVIN Fu	444345 1c 1STC	2HW7	1YDT			Sc=6.08782, min distance = 2.07474
MKNK2_HUMAN I	4565 1h1r	CDK2_HUMAN Fu	444345 1c 1AQ1	2HW7	1H1R			Sc=5.91177, min distance = 2.37145
MKNK2_HUMAN I	5281680 Q	PIM1_HUMAN Fu	444345 1c 1YHS	2HW7	2O64			Sc=6.39781, min distance = 2.27298
MKNK2_HUMAN I	5288708 1	CDK2_HUMAN Fu	444345 1c 1AQ1	2HW7	1KE5			Sc=6.0821, min distance = 2.300517
MKNK2_HUMAN I	5288710 C	CDK2_HUMAN Fu	444345 1c 1AQ1	2HW7	1KE7			Sc=6.29556, min distance = 2.29908
MKNK2_HUMAN I	5288712 1	CDK2_HUMAN Fu	444345 1c 1AQ1	2HW7	1KE9			Sc=6.18519, min distance = 2.32617
MKNK2_HUMAN I	5327110 i	KAPCA_BOVIN Fu	444345 1c 1STC	2HW7	2C1B			Sc=6.09935, min distance = 2.05684
MKNK2_HUMAN I	5327121 I	PIM1_HUMAN Fu	444345 1c 1YHS	2HW7	2C3I			Sc=5.97271, min distance = 2.40341
MKNK2_HUMAN I	5327123 I	CHK1_HUMAN Fu	444345 1c 1NVR	2HW7	2C3L			Sc=6.12054, min distance = 2.44568
MKNK2_HUMAN I	5327134 C	CDK2_HUMAN Fu	444345 1c 1AQ1	2HW7	2C6L			Sc=6.11568, min distance = 2.35736
MKNK2_HUMAN I	5494414 C	CDK2_HUMAN Fu	444345 1c 1AQ1	2HW7	2A0C			Sc=6.39566, min distance = 2.13324
MKNK2_HUMAN I	5687 1di8	CDK2_HUMAN Fu	444345 1c 1AQ1	2HW7	1DI8			Sc=6.10026, min distance = 2.71640
MKNK2_HUMAN I	6102670	CDK2_HUMAN Fu	444345 1c 1AQ1	2HW7	1YKR			Sc=6.21878, min distance = 2.28705
MKNK2_HUMAN I	6420138 2	CDK2_HUMAN Fu	444345 1c 1AQ1	2HW7	2UUE			Sc=6.23651, min distance = 2.63052
MKNK2_HUMAN I	6420139 C	CDK2_HUMAN Fu	444345 1c 1AQ1	2HW7	2C5V			Sc=6.0821, min distance = 2.531478
MKNK2_HUMAN I	6539118 g	CDK2_HUMAN Fu	444345 1c 1AQ1	2HW7	1FVV			Sc=6.41956, min distance = 2.41516
MKNK2_HUMAN I	656971 CI	CDK2_HUMAN Fu	444345 1c 1AQ1	2HW7	1Y8Y			Sc=5.8673, min distance = 2.286958
MKNK2_HUMAN I	6852167 2	PK3CG_HUMAN Fu	444345 1c 1E8Z	2HW7	2CHZ			Sc=6.15834, min distance = 2.40358
MKNK2_HUMAN I	6852207 M	KAPCA_BOVIN Fu	444345 1c 1STC	2HW7	2GNI			Sc=5.90171, min distance = 2.41762
MKNK2_HUMAN I	6914611 p	KAPCA_BOVIN Fu	444345 1c 1STC	2HW7	2F7E			Sc=6.34323, min distance = 2.25324
MKNK2_HUMAN I	72271 7-H	CDK2_HUMAN Fu	444345 1c 1AQ1	2HW7	1PKD		0.91	Sc=6.43632, min distance = 2.09531
MKNK2_HUMAN I	72271 7-H	CHK1_HUMAN Fu	444345 1c 1NVR	2HW7	1NVQ		0.91	Sc=6.42614, min distance = 2.20939
MKNK2_HUMAN I	9817550 V	CDK2_HUMAN Fu	444345 1c 1AQ1	2HW7	3BHV			Sc=6.30357, min distance = 2.16618
ML1_VISAL Fu	323 couma	TRFL_BOVIN Fu	444205 CI 2G93	1M2T	3CRB			Sc=5.61929, min distance = 1.37424
ML1_VISAL Fu	446280 CI	RICI_RICCO Fu	190 adeni 2P8N	1M2T	1IFU	40.56		Sc=6.25229, min distance = 2.13598

ML1_VISAL Fu	5287547 6	PURR_ECOLI Fu	190 adeni	2PUB	1M2T	2PUA		0.9	Sc=5.82541, min distance = 2.523356
ML1_VISAL Fu	5287830 6	Q8RLY5_LACHE I	190 adeni	1S2D	1M2T	1S2I		0.93	Sc=5.75386, min distance = 1.849003
ML1_VISAL Fu	764 guani	PURR_ECOLI Fu	190 adeni	2PUB	1M2T	1WET			Sc=5.90012, min distance = 2.249436
ML1_VISAL Fu	790 hypox	PURR_ECOLI Fu	190 adeni	2PUB	1M2T	1JFT			Sc=5.80589, min distance = 2.678329
MLTA_ECOLI Fu	104904 Me	IGKC_MOUSE Fu	24139 ace	3BZ4	2PI8	2AJZ			Sc=5.81298, min distance = 1.586619
MLTA_ECOLI Fu	439353 be	AGI3_WHEAT Fu	24139 ace	1K7U	2PI8	1K7V			Sc=5.7479, min distance = 1.9610135
MLTA_ECOLI Fu	439353 be	LYSC_CHICK Fu	24139 ace	1LZB	2PI8	1UC0			Sc=5.69574, min distance = 1.659008
MLTA_ECOLI Fu	444305 L-	PGRP_CAMDR Fu	24139 ace	2R2K	2PI8	3C2X			Sc=5.84189, min distance = 1.118414
MLTA_ECOLI Fu	444863 is	Q97N96_STRPN I	24139 ace	2J1V	2PI8	2J1S			Sc=5.71147, min distance = 1.212195
MLTA_ECOLI Fu	445595 CI	CHIA_SERMA Fu	24139 ace	1K9T	2PI8	1FFQ			Sc=6.03171, min distance = 1.248306
MLTA_ECOLI Fu	445750 Al	A0A5D7_MOUSE I	24139 ace	1M7I	2PI8	1M7D		0.96	Sc=5.94579, min distance = 2.290960
MLTA_ECOLI Fu	446101 AC	HYSA_STRPN Fu	24139 ace	1LXK	2PI8	1OJO		0.94	Sc=6.06814, min distance = 0.655674
MLTA_ECOLI Fu	448431 CI	P84141_ARTAU I	24139 ace	1RWC	2PI8	1RWH			Sc=5.65587, min distance = 1.745814
MLTA_ECOLI Fu	6540269 2	HYSA_STRPN Fu	24139 ace	1LXK	2PI8	2BRP			Sc=5.94712, min distance = 1.916536
MLTA_ECOLI Fu	656981 CU	LYSC_CHICK Fu	24139 ace	1LZB	2PI8	1YIL			Sc=6.17141, min distance = 0.971483
MLTB_ECOLI Fu	185698 al	Q9HYN5_PSEAE I	444205 CI	2JDH	1D0K	1OVS			Sc=5.7534, min distance = 1.3888459
MLTB_ECOLI Fu	94214 Met	MBL1_RAT Full=	82313 1rd	1KWV	1D0M	1AFA			Sc=5.61167, min distance = 2.188932
MMAB_HUMAN Fu	129236 2c	CKI1_SCHPO Fu	5957 Aden	1CSN	2IDX	2CSN			Sc=5.70207, min distance = 2.293774
MMAB_HUMAN Fu	6022 Aden	ACT1_DROME Fu	5957 Aden	2HF4	2IDX	2HF3		0.99	Sc=5.8988, min distance = 2.0817497
MMAB_HUMAN Fu	6022 Aden	HSP7F_YEAST Fu	5957 Aden	3D2F	2IDX	3C7N		0.99	Sc=5.90816, min distance = 2.137557
MMP10_HUMAN I	25049754	MMP12_HUMAN Fu	448002 1r	1RMZ	1Q3A	3F18			Sc=6.07995, min distance = 2.120185
MMP10_HUMAN I	25049755	MMP12_HUMAN Fu	448002 1r	1RMZ	1Q3A	3F19			Sc=5.85775, min distance = 2.200353
MMP10_HUMAN I	25049756	MMP12_HUMAN Fu	448002 1r	1RMZ	1Q3A	3F1A			Sc=5.84946, min distance = 2.094790
MMP10_HUMAN I	5362422 b	MMP12_HUMAN Fu	448002 1r	1RMZ	1Q3A	1JK3			Sc=6.3339, min distance = 2.4995797
MMP13_HUMAN I	1836 1g4k	MMP3_HUMAN Fu	6914601 2	2D1O	2D1N	1G4K	66.23		Sc=6.4124, min distance = 2.0078620
MMP13_HUMAN I	1948 1b8y	MMP3_HUMAN Fu	6914601 2	2D1O	2D1N	1B8Y	66.23		Sc=6.15067, min distance = 1.986504
MMP13_HUMAN I	4218 mmp;	MMP3_HUMAN Fu	6914601 2	2D1O	2D1N	1BQO	66.23		Sc=6.31513, min distance = 2.311167
MMP13_HUMAN I	4369141 1	MMP3_HUMAN Fu	6914601 2	2D1O	2D1N	1C3I	66.23		Sc=6.54895, min distance = 2.703637
MMP13_HUMAN I	444789 1c	MMP3_HUMAN Fu	6914601 2	2D1O	2D1N	1CAQ	66.23		Sc=6.42105, min distance = 2.069331
MMP13_HUMAN I	444831 CI	MMP3_HUMAN Fu	6914601 2	2D1O	2D1N	1CIZ	66.23		Sc=6.40804, min distance = 2.024786
MMP13_HUMAN I	445010 CI	MMP3_HUMAN Fu	6914601 2	2D1O	2D1N	1D7X	66.23		Sc=6.20064, min distance = 2.138807
MMP13_HUMAN I	446165 1h	MMP3_HUMAN Fu	6914601 2	2D1O	2D1N	1HY7	66.23		Sc=6.17341, min distance = 2.027212
MMP13_HUMAN I	5287415 1	MMP3_HUMAN Fu	6914601 2	2D1O	2D1N	1G49	66.23		Sc=6.18125, min distance = 1.770045
MMP13_HUMAN I	5288814 1	MMP3_HUMAN Fu	6914601 2	2D1O	2D1N	1D5J	66.23		Sc=6.14424, min distance = 2.352692
MMP1_HUMAN Fu	446675 CI	MMP8_HUMAN Fu	194777 ME	1MNC	1HFC	1KBC	66.67		Sc=5.96318, min distance = 2.664317
MMP3_HUMAN Fu	657061 1y	MMP13_HUMAN Fu	6914601 2	2D1N	2D1O	1YOU	66.23		Sc=6.43622, min distance = 1.983279
MMP8_HUMAN Fu	164795 N(	RBCMT_PEA Ful	23831 HEF	1MLV	1BZS	2H2J			Sc=5.63262, min distance = 2.270602

MNMA_ECOLI F	445796 AN	ECX1_SULSO Fu	6083 aden	2C38	2DEU	2C37			Sc=5.66898, min distance = 2.297148
MNMA_STRPN F	439182 5'	Q70GK9_STRCT	34756 Acy	1RQP	2HMA	2CC2		0.86	Sc=6.18169, min distance = 1.844170
MNMA_STRPN F	6852187 2	Q70GK9_STRCT	34756 Acy	1RQP	2HMA	2CBX		0.84	Sc=6.08495, min distance = 2.097708
MNME_ECOLI F	37792 gam	GNAI1_RAT Ful	8977 1dar	1SVK	2GJ8	1AS0		0.99	Sc=6.09072, min distance = 2.646686
MNME_ECOLI F	37792 gam	RAB6B_HUMAN F	8977 1dar	2E9S	2GJ8	2FFQ		0.99	Sc=6.1023, min distance = 2.4020168
MNME_ECOLI F	37792 gam	RAC1_HUMAN Fu	8977 1dar	1RYF	2GJ8	2FJU		0.99	Sc=6.10766, min distance = 2.404566
MNME_ECOLI F	37792 gam	RB11A_HUMAN F	8977 1dar	1OIX	2GJ8	1OIW		0.99	Sc=6.54147, min distance = 2.592464
MNME_ECOLI F	37792 gam	RHOA_HUMAN Fu	8977 1dar	1TX4	2GJ8	1CXZ		0.99	Sc=6.10268, min distance = 2.436956
MNME_ECOLI F	444825 1c	PURA_ECOLI Fu	8977 1dar	1QF5	2GJ8	1CH8		0.95	Sc=6.49642, min distance = 1.957151
MNME_ECOLI F	446248 CI	RAN_HUMAN Ful	8977 1dar	3CH5	2GJ8	1IBR			Sc=6.12367, min distance = 2.212366
MNME_ECOLI F	446248 CI	RASH_HUMAN Fu	8977 1dar	2CE2	2GJ8	1IAQ			Sc=6.1003, min distance = 2.5687851
MNME_ECOLI F	6804 guar	EF1A_YEAST Fu	8977 1dar	2B7B	2GJ8	1G7C		0.99	Sc=5.99287, min distance = 2.681449
MNME_ECOLI F	8582 Inos	PURA1_MOUSE F	8977 1dar	1LON	2GJ8	1IWE		0.99	Sc=5.95264, min distance = 2.809250
MNME_ECOLI F	93082 Gab	CDC42_HUMAN F	8977 1dar	2NGR	2GJ8	2ODB		0.99	Sc=6.13315, min distance = 2.322727
MNME_ECOLI F	93082 Gab	RAC3_HUMAN Fu	8977 1dar	2C2H	2GJ8	2QME		0.99	Sc=6.13984, min distance = 2.256258
MOBA_ECOLI F	51 2-Oxop	SERA_ECOLI Fu	311 citri	2P9E	1E5K	1YBA		0.76	Sc=5.84986, min distance = 1.400601
MOEB_ECOLI F	15602983	KAPCA_BOVIN F	5957 Aden	1Q24	1JWA	2UW5			Sc=5.98705, min distance = 2.040198
MOEB_ECOLI F	2519 caff	PYGM_RABIT Fu	6083 aden	8GPB	1JWB	1GFZ			Sc=5.84677, min distance = 2.057514
MOEB_ECOLI F	448042 2e	KAPCA_BOVIN F	5957 Aden	1Q24	1JWA	1Q8T			Sc=6.1463, min distance = 2.3767332
MOEB_ECOLI F	5893 nadi	KTRA_BACSU Fu	5957 Aden	2HMW	1JWA	1LSU			Sc=6.30231, min distance = 1.359742
MOEB_ECOLI F	6022 Ader	REX_BACSU Ful	5957 Aden	2VT3	1JWA	2VT2		0.99	Sc=6.43155, min distance = 1.939432
MOEB_ECOLI F	6030 Uric	ECX1_SULSO Fu	6083 aden	2C38	1JWB	2C37			Sc=6.33461, min distance = 0.371269
MOEB_ECOLI F	656968 1y	PDE4B_HUMAN F	6083 aden	1ROR	1JWB	1Y2H			Sc=6.00938, min distance = 2.025910
MOEB_ECOLI F	6804 guar	PDE10_HUMAN F	6083 aden	2OUN	1JWB	2OUQ		0.8	Sc=6.43215, min distance = 0
MOSA_AZOVI F	6022 Ader	DDL_THET8 Ful	5957 Aden	2ZDQ	2OGX	2ZDH		0.99	Sc=6.44188, min distance = 1.858161
MOXC_BACSU F	5326566 l	BLVRB_HUMAN F	444243 FA	1HE4	1YW1	1HE5		0.82	Sc=5.89257, min distance = 2.536219
MOXC_BACSU F	5326566 l	Q9WZW1_THEMA	444243 FA	1T6Y	1YW1	1S4M		0.82	Sc=5.87931, min distance = 2.381988
MP2K1_HUMAN	11175137	KAPCA_BOVIN F	5957 Aden	1Q24	1S9J	2UW7	29.44		Sc=6.21777, min distance = 2.281154
MP2K1_HUMAN	11608401	KAPCA_BOVIN F	5957 Aden	1Q24	1S9J	2UVY	29.44		Sc=6.12603, min distance = 2.697657
MP2K1_HUMAN	11708454	KAPCA_BOVIN F	5957 Aden	1Q24	1S9J	2VNW	29.44		Sc=6.03565, min distance = 2.327832
MP2K1_HUMAN	15602983	KAPCA_BOVIN F	5957 Aden	1Q24	1S9J	2UW5	29.44		Sc=6.12453, min distance = 2.591276
MP2K1_HUMAN	16122607	PDPK1_HUMAN F	5957 Aden	2BIY	1S9J	2PE0	28.77		Sc=6.1787, min distance = 2.7491180
MP2K1_HUMAN	16122633	KAPCA_BOVIN F	5957 Aden	1Q24	1S9J	2UW0	29.44		Sc=6.3797, min distance = 2.3164468
MP2K1_HUMAN	17751819	KAPCA_BOVIN F	5957 Aden	1Q24	1S9J	2VO6	29.44		Sc=6.28836, min distance = 2.327325
MP2K1_HUMAN	3540 1yds	KAPCA_BOVIN F	5957 Aden	1Q24	1S9J	1YDS	29.44		Sc=5.88003, min distance = 2.684329
MP2K1_HUMAN	3547 Fasu	KAPCA_BOVIN F	5957 Aden	1Q24	1S9J	1Q8W	29.44		Sc=5.92379, min distance = 2.158655
MP2K1_HUMAN	444564 AD	BIOD_ECOLI Fu	5957 Aden	1A82	1S9J	1BS1		0.91	Sc=6.37695, min distance = 2.306845

MP2K1_HUMAN I	444564 AD	MYS2_DICDI Fu	5957 Aden	1FMW	1S9J	1W9I		0.91	Sc=5.60858, min distance = 0
MP2K1_HUMAN I	447916 ac	RIO1_ARCFU Fu	5957 Aden	1ZP9	1S9J	1ZTF		0.94	Sc=6.07287, min distance = 2.52715
MP2K1_HUMAN I	448043 2g	KAPCA_BOVIN Fu	5957 Aden	1Q24	1S9J	1Q8U	29.44		Sc=6.00119, min distance = 2.66667
MP2K1_HUMAN I	449240 1y	KAPCA_BOVIN Fu	5957 Aden	1Q24	1S9J	1YDR	29.44		Sc=5.94101, min distance = 2.33202
MP2K1_HUMAN I	6022 Aden	BIOD_ECOLI Fu	5957 Aden	1A82	1S9J	1DAD		0.99	Sc=6.37875, min distance = 2.61329
MP2K1_HUMAN I	6022 Aden	CLCN5_HUMAN Fu	5957 Aden	2J9L	1S9J	2JA3		0.99	Sc=6.37789, min distance = 1.70286
MP2K1_HUMAN I	6022 Aden	GLGS_SOLTU Fu	5957 Aden	1YP3	1S9J	1YP4		0.99	Sc=6.37499, min distance = 1.46544
MP2K1_HUMAN I	6022 Aden	KTHY_HUMAN Fu	5957 Aden	1E2Q	1S9J	1NN3		0.99	Sc=6.34154, min distance = 2.19731
MP2K1_HUMAN I	6022 Aden	MUTS_ECOLI Fu	5957 Aden	1W7A	1S9J	1OH7		0.99	Sc=6.36101, min distance = 2.12114
MP2K1_HUMAN I	6022 Aden	PDXK_SHEEP Fu	5957 Aden	1LHR	1S9J	1RFU		0.99	Sc=6.37312, min distance = 2.03741
MP2K1_HUMAN I	6022 Aden	PUR7_METJA Fu	5957 Aden	2Z02	1S9J	2YZL		0.99	Sc=6.37312, min distance = 2.10399
MP2K1_HUMAN I	6022 Aden	PURT_ECOLI Fu	5957 Aden	1KJ8	1S9J	1KJQ		0.99	Sc=6.36004, min distance = 2.18096
MP2K1_HUMAN I	6022 Aden	Q72H90_THET2 I	5957 Aden	2BEK	1S9J	2BEJ		0.99	Sc=6.35076, min distance = 2.32716
MP2K1_HUMAN I	6022 Aden	RIO1_ARCFU Fu	5957 Aden	1ZP9	1S9J	1ZTH		0.99	Sc=6.37789, min distance = 1.53522
MP2K1_HUMAN I	6083 aden	AAKG1_RAT Ful	5957 Aden	2V92	1S9J	2V8Q		0.98	Sc=6.24461, min distance = 2.46976
MP2K1_HUMAN I	91532 AMP	Q5SJV7_THET8 I	5957 Aden	2Z08	1S9J	2Z09		0.99	Sc=5.70212, min distance = 2.05715
MP2K2_HUMAN I	11175137	KAPCA_BOVIN Fu	5957 Aden	1Q24	1S9I	2UW7			Sc=6.21296, min distance = 2.09745
MP2K2_HUMAN I	11608401	KAPCA_BOVIN Fu	5957 Aden	1Q24	1S9I	2UVY			Sc=6.13424, min distance = 2.50081
MP2K2_HUMAN I	11708454	KAPCA_BOVIN Fu	5957 Aden	1Q24	1S9I	2VNW			Sc=6.07458, min distance = 1.77973
MP2K2_HUMAN I	15602983	KAPCA_BOVIN Fu	5957 Aden	1Q24	1S9I	2UW5			Sc=6.15275, min distance = 2.20138
MP2K2_HUMAN I	16122607	PDPK1_HUMAN Fu	5957 Aden	2BIY	1S9I	2PE0			Sc=6.18811, min distance = 2.45680
MP2K2_HUMAN I	3540 1yds	KAPCA_BOVIN Fu	5957 Aden	1Q24	1S9I	1YDS			Sc=5.87874, min distance = 2.29722
MP2K2_HUMAN I	3547 Fasu	KAPCA_BOVIN Fu	5957 Aden	1Q24	1S9I	1Q8W			Sc=5.94101, min distance = 1.85219
MP2K2_HUMAN I	36735 Gpp	Q9RVK2_DEIRA I	5957 Aden	1SU2	1S9I	1SZ3		0.8	Sc=6.477, min distance = 1.24034269
MP2K2_HUMAN I	444564 AD	MYS2_DICDI Fu	5957 Aden	1FMW	1S9I	1W9I		0.91	Sc=6.33579, min distance = 0
MP2K2_HUMAN I	444852 CI	ATPB_BOVIN Fu	5957 Aden	2V7Q	1S9I	1COW		0.91	Sc=6.46629, min distance = 1.76091
MP2K2_HUMAN I	446202 TF	SYW_BACST Ful	5957 Aden	1MAU	1S9I	1I6K			Sc=5.83844, min distance = 2.19348
MP2K2_HUMAN I	447004 CI	MYS2_DICDI Fu	5957 Aden	1FMW	1S9I	1LVK			Sc=6.01636, min distance = 0
MP2K2_HUMAN I	447916 ac	RIO1_ARCFU Fu	5957 Aden	1ZP9	1S9I	1ZTF		0.94	Sc=6.13668, min distance = 2.65954
MP2K2_HUMAN I	448043 2g	KAPCA_BOVIN Fu	5957 Aden	1Q24	1S9I	1Q8U			Sc=6.00705, min distance = 2.35025
MP2K2_HUMAN I	449240 1y	KAPCA_BOVIN Fu	5957 Aden	1Q24	1S9I	1YDR			Sc=5.94848, min distance = 2.00294
MP2K2_HUMAN I	5287416 C	INSR_HUMAN Fu	5957 Aden	3BU5	1S9I	1RQQ		0.94	Sc=6.06709, min distance = 1.98060
MP2K2_HUMAN I	6022 Aden	BIOD_ECOLI Fu	5957 Aden	1A82	1S9I	1DAD		0.99	Sc=6.39485, min distance = 2.25567
MP2K2_HUMAN I	6022 Aden	CLCN5_HUMAN Fu	5957 Aden	2J9L	1S9I	2JA3		0.99	Sc=6.37789, min distance = 2.12550
MP2K2_HUMAN I	6022 Aden	FAK1_HUMAN Fu	5957 Aden	2IJM	1S9I	1MP8		0.99	Sc=6.39694, min distance = 1.91700
MP2K2_HUMAN I	6022 Aden	MUTS_ECOLI Fu	5957 Aden	1W7A	1S9I	1OH7		0.99	Sc=6.37056, min distance = 1.83084
MP2K2_HUMAN I	6022 Aden	PDXK_SHEEP Fu	5957 Aden	1LHR	1S9I	1RFU		0.99	Sc=6.39391, min distance = 2.40144

MP2K2_HUMAN I	6022 Ader	PURK_ECOLI Fu	5957 Aden	3ETH	1S9I	3ETJ		0.99	Sc=6.3806, min distance = 1.775070
MP2K2_HUMAN I	6022 Ader	PURP_METJA Fu	5957 Aden	2R7L	1S9I	2R7N		0.99	Sc=5.92146, min distance = 2.38174
MP2K2_HUMAN I	6022 Ader	PURT_ECOLI Fu	5957 Aden	1KJ8	1S9I	1KJQ		0.99	Sc=6.38377, min distance = 1.95401
MP2K2_HUMAN I	6022 Ader	REX_BACSU Full	5957 Aden	2VT3	1S9I	2VT2		0.99	Sc=6.43215, min distance = 1.24703
MP2K2_HUMAN I	6022 Ader	RIO1_ARCFU Fu	5957 Aden	1ZP9	1S9I	1ZTH		0.99	Sc=6.38522, min distance = 1.72302
MP2K2_HUMAN I	6022 Ader	RK_BOVIN Full=	5957 Aden	3C4W	1S9I	3C4Z		0.99	Sc=6.39965, min distance = 2.12006
MP2K2_HUMAN I	6083 ader	AAKG1_RAT Ful	5957 Aden	2V92	1S9I	2V8Q		0.98	Sc=6.29517, min distance = 2.69421
MP2K2_HUMAN I	6083 ader	PURP_METJA Fu	5957 Aden	2R7L	1S9I	2R7M		0.98	Sc=6.30932, min distance = 2.28967
MP2K2_HUMAN I	6830 guar	Q381M1_9TRYP I	5957 Aden	2Q0D	1S9I	2Q0E		0.8	Sc=5.99503, min distance = 1.92131
MP2K2_HUMAN I	91532 AMF	Q5SJV7_THET8 I	5957 Aden	2Z08	1S9I	2Z09		0.99	Sc=6.46846, min distance = 2.23889
MP2K2_HUMAN I	9547921 C	SYQ_ECOLI Ful	5957 Aden	1GTR	1S9I	2RE8		0.83	Sc=6.17514, min distance = 2.03621
MRAW_THEMA Fu	188380 Ac	MCES_ENCCU Fu	439155 Ad	1RI1	1M6Y	1Z3C		0.91	Sc=6.59352, min distance = 2.00931
MRAW_THEMA Fu	445762 5'	MTTA_THEAQ Fu	34756 Acy	2ADM	1N2X	2IH2		0.93	Sc=6.33091, min distance = 2.22639
MRAW_THEMA Fu	445971 CI	COMT_RAT Full=	34756 Acy	2CL5	1N2X	1H1D		0.92	Sc=6.57247, min distance = 2.62539
MRAW_THEMA Fu	446535 CI	HNMT_HUMAN Fu	439155 Ad	2AOT	1M6Y	1JQE		0.95	Sc=6.53313, min distance = 1.70872
MRAW_THEMA Fu	60961 ade	PIMT_PYRFU Fu	439155 Ad	1JG1	1M6Y	1JG2		0.84	Sc=6.25868, min distance = 2.71981
MRAW_THET8 Fu	445762 5'	MTTA_THEAQ Fu	34756 Acy	2ADM	1WG8	2IH2		0.93	Sc=6.43317, min distance = 2.25146
MRAW_THET8 Fu	445971 CI	COMT_RAT Full=	34756 Acy	2CL5	1WG8	1H1D		0.92	Sc=6.58631, min distance = 2.66247
MRAW_THET8 Fu	5326531 C	CHOMT_MEDSA Fu	34756 Acy	1FPQ	1WG8	1FP1		0.92	Sc=6.57889, min distance = 1.94874
MRP1_HUMAN Fu	11957393	FAK1_HUMAN Fu	5957 Aden	2IJM	2CBZ	2ETM			Sc=5.87995, min distance = 2.35562
MSBA_SALTY Fu	23656870	CSK2A_MAIZE Fu	33113 gam	1LP4	3B60	2PVN			Sc=6.39896, min distance = 2.79875
MSBA_SALTY Fu	5287844 i	GSK3B_HUMAN Fu	33113 gam	1J1B	3B60	1UV5			Sc=5.98545, min distance = 3.32421
MSBA_SALTY Fu	5326739 i	GSK3B_HUMAN Fu	33113 gam	1J1B	3B60	1Q41			Sc=5.96739, min distance = 3.20868
MSBA_SALTY Fu	5326976 1	CSK2A_MAIZE Fu	33113 gam	1LP4	3B60	1ZOE			Sc=5.63272, min distance = 2.89245
MSBA_SALTY Fu	5327148 C	IPKA_RABIT Fu	33113 gam	1CDK	3B60	2ERZ			Sc=5.96303, min distance = 2.20793
MSBA_SALTY Fu	9549303 2	STK6_HUMAN Fu	33113 gam	2DWB	3B60	2NP8			Sc=6.27155, min distance = 2.48636
MSH2_HUMAN Fu	1694 TBBt	CDK2_HUMAN Fu	6022 Aden	1GY3	2O8B	1P5E			Sc=5.7055, min distance = 1.431223
MSH2_HUMAN Fu	398148 1e	CDK2_HUMAN Fu	6022 Aden	1GY3	2O8B	1E1X			Sc=6.09744, min distance = 2.11320
MSH2_HUMAN Fu	444842 CI	CDK2_HUMAN Fu	6022 Aden	1GY3	2O8B	1CKP			Sc=5.61822, min distance = 2.83387
MSH2_HUMAN Fu	5957 Ader	HSLU_ECOLI Fu	6022 Aden	1HQY	2O8B	1DO0		0.99	Sc=6.03147, min distance = 2.05639
MSH2_HUMAN Fu	5957 Ader	RK_BOVIN Full=	6022 Aden	3C4Z	2O8B	3C4W		0.99	Sc=6.02491, min distance = 2.00992
MSH2_HUMAN Fu	6132 Cyti	ECX1_PYRAB Fu	6022 Aden	2PO0	2O8B	2PO2			Sc=5.80934, min distance = 2.60871
MSH2_HUMAN Fu	91532 AMF	AROK_MYCTU Fu	6022 Aden	2IYV	2O8B	1ZYU		0.99	Sc=6.05614, min distance = 2.06030
MSH6_HUMAN Fu	1694 TBBt	CDK2_HUMAN Fu	6022 Aden	1GY3	2O8B	1P5E			Sc=5.7055, min distance = 1.691518
MSH6_HUMAN Fu	24864077	CDK2_HUMAN Fu	6022 Aden	1GY3	2O8B	2VTN			Sc=6.24862, min distance = 2.32684
MSH6_HUMAN Fu	9991833 S	CDK2_HUMAN Fu	6022 Aden	1GY3	2O8B	2R3H			Sc=6.07041, min distance = 2.12509
MTAP_HUMAN Fu	100684 9-	RICI_RICCO Fu	190 adeni	2P8N	1CB0	1IL4			Sc=5.87184, min distance = 2.72044

MTAP_HUMAN F	11979 5-M	COBT_SALTY Fu	190 adeni	1JH8	1CB0	1JHM			Sc=5.68791, min distance = 2.54685
MTAP_HUMAN F	446312 1i	RICI_RICCO Fu	190 adeni	2P8N	1CB0	1IL9			Sc=5.90022, min distance = 2.09188
MTAP_HUMAN F	5287547 6	PURR_ECOLI Fu	190 adeni	2PUB	1CB0	2PUA		0.9	Sc=5.77268, min distance = 2.64424
MTAP_HUMAN F	675 Dimec	COBT_SALTY Fu	190 adeni	1JH8	1CB0	1D0S			Sc=5.75987, min distance = 2.36276
MTAP_HUMAN F	764 guani	PURR_ECOLI Fu	190 adeni	2PUB	1CB0	1WET			Sc=5.83519, min distance = 2.56099
MTAP_HUMAN F	78598 5-M	COBT_SALTY Fu	190 adeni	1JH8	1CB0	1JHP			Sc=5.71186, min distance = 2.52313
MTAP_HUMAN F	790 hypox	PURR_ECOLI Fu	190 adeni	2PUB	1CB0	1JFT			Sc=5.745, min distance = 2.7044247
MTAP_HUMAN F	96253 7-D	RICI_RICCO Fu	190 adeni	2P8N	1CB0	1IL3			Sc=5.87184, min distance = 2.63445
MTAP_SULSO F	24180721	PIM1_HUMAN Fu	60961 ade	1YI4	1JDV	3C4E			Sc=5.99047, min distance = 1.98622
MTAP_SULSO F	446609 1y	MTAP_HUMAN Fu	439176 Me	1CG6	1JDT	1K27			Sc=6.02034, min distance = 2.28842
MTAP_SULSO F	447724 9-	DEOD_ECOLI Fu	60961 ade	1PK7	1JDV	1OV6	33.82	0.9	Sc=6.37813, min distance = 0.74279
MTAP_SULSO F	5327148 C	KAPCA_MOUSE F	60961 ade	1FMO	1JDV	2ERZ			Sc=6.13712, min distance = 1.98169
MTAP_SULSO F	656970 MT	MTNN_ECOLI Fu	439176 Me	1Z5O	1JDT	1Y6Q	28.22		Sc=6.32584, min distance = 0.35382
MTD1_YEAST F	123927 A3	DAPB_ECOLI Fu	5893 nadi	1DRU	1EE9	1DRV		0.97	Sc=5.733, min distance = 2.3727960
MTD1_YEAST F	6022 Aden	Q5SI02_THET8	5893 nadi	2BJK	1EE9	2BJA			Sc=6.18695, min distance = 2.29020
MTD21_STRPN	4369234 C	COMT_RAT Full	34756 Acy	2CL5	2DPM	1JR4			Sc=6.03069, min distance = 2.18615
MTD21_STRPN	445762 5'	MTTA_THEAQ Fu	34756 Acy	2ADM	2DPM	2IH2		0.93	Sc=6.34855, min distance = 2.62220
MTD21_STRPN	445971 CI	COMT_RAT Full	34756 Acy	2CL5	2DPM	1H1D		0.92	Sc=6.55352, min distance = 2.87005
MTDA_METEX F	165230 1a	G6PD_LEUME Fu	5886 NADP	1H9A	1LUA	2DPG			Sc=5.94281, min distance = 2.03768
MTH1_HAEPH F	188380 Ac	MCES_ENCCU Fu	439155 Ad	1RI1	2C7P	1Z3C		0.91	Sc=6.57146, min distance = 2.10594
MTH1_HAEPH F	439176 Me	MTR1_RHOSH Fu	34756 Acy	1NW5	6MHT	1EG2		0.91	Sc=6.21122, min distance = 2.51790
MTH1_HAEPH F	445762 5'	MTTA_THEAQ Fu	34756 Acy	2ADM	6MHT	2IH2		0.93	Sc=6.27964, min distance = 2.71983
MTH1_HAEPH F	445971 CI	COMT_RAT Full	34756 Acy	2CL5	6MHT	1H1D		0.92	Sc=6.49729, min distance = 2.28022
MTH1_HAEPH F	446535 CI	HNMT_HUMAN Fu	439155 Ad	2AOT	2C7P	1JQE		0.95	Sc=6.52614, min distance = 1.88081
MTH1_HAEPH F	51 2-Oxop	SERA_ECOLI Fu	311 citri	2P9E	2C7P	1YBA		0.76	Sc=5.89311, min distance = 0.71698
MTH1_HAEPH F	5327118 5	Q70GK9_STRCT	34756 Acy	1RQP	6MHT	2C2W		0.81	Sc=5.70837, min distance = 1.90070
MTH1_HAEPH F	60961 ade	PIMT_PYRFU Fu	439155 Ad	1JG1	2C7P	1JG2		0.84	Sc=6.27417, min distance = 2.80676
MTH1_HAEPH F	65482 sir	ERM_BACSU Ful	439155 Ad	1QAN	2C7P	1QAQ		0.88	Sc=6.58631, min distance = 2.07376
MTH1_HAEPH F	65482 sir	MCES_ENCCU Fu	439155 Ad	1RI1	2C7P	2HV9		0.88	Sc=6.56779, min distance = 2.17981
MTH1_HAEPH F	65482 sir	MTR1_RHOSH Fu	439155 Ad	1NW7	2C7P	1NW6		0.88	Sc=6.57296, min distance = 2.30150
MTH1_HAEPH F	854023 Ep	Q8WSF8_APLCA	23831 HEP	2BR7	2C7P	2BYQ			Sc=5.6948, min distance = 2.427157
MTHFS_MYCPN	445940 O6	CDK2_HUMAN Fu	6022 Aden	1GY3	1U3F	1GZ8			Sc=6.05449, min distance = 2.64127
MTHFS_MYCPN	446090 1h	NDKC_DICDI Fu	6022 Aden	1KDN	1U3F	1HIY		0.97	Sc=6.00118, min distance = 1.42797
MTHFS_MYCPN	4565 1h1r	CDK2_HUMAN Fu	6022 Aden	1GY3	1U3F	1H1R			Sc=6.11354, min distance = 2.30602
MTHFS_MYCPN	6804 guar	NDK_PYRHO Ful	6022 Aden	2DYA	1U3F	2DXF		0.8	Sc=5.9343, min distance = 2.043049
MTNK_BACSU F	10224714	CDK2_HUMAN Fu	6022 Aden	1GY3	2OLC	3DDP			Sc=6.44489, min distance = 1.83073
MTNK_BACSU F	11957417	CDK2_HUMAN Fu	6022 Aden	1GY3	2OLC	2I40			Sc=6.31681, min distance = 1.65792

MTNK_BACSU F	1540 1h1q	CDK2_HUMAN Fu	6022 Aden	1GY3	2OLC	1H1Q		Sc=6.28549, min distance = 1.51273
MTNK_BACSU F	15942652	CDK2_HUMAN Fu	6022 Aden	1GY3	2OLC	2DUV		Sc=6.34013, min distance = 1.95163
MTNK_BACSU F	160355 rc	CDK2_HUMAN Fu	6022 Aden	1GY3	2OLC	3DDQ		Sc=6.2945, min distance = 1.391634
MTNK_BACSU F	16214823	CDK2_HUMAN Fu	6022 Aden	1GY3	2OLC	2UZB		Sc=6.08768, min distance = 2.87372
MTNK_BACSU F	16214827	CDK2_HUMAN Fu	6022 Aden	1GY3	2OLC	2UZN		Sc=6.0521, min distance = 2.674502
MTNK_BACSU F	1707 1fvt	CDK2_HUMAN Fu	6022 Aden	1GY3	2OLC	1FVT		Sc=6.17438, min distance = 2.54239
MTNK_BACSU F	23653516	CDK2_HUMAN Fu	6022 Aden	1GY3	2OLC	2R3G		Sc=6.04342, min distance = 1.87994
MTNK_BACSU F	23653518	CDK2_HUMAN Fu	6022 Aden	1GY3	2OLC	2R3J		Sc=6.13597, min distance = 1.76000
MTNK_BACSU F	23653519	CDK2_HUMAN Fu	6022 Aden	1GY3	2OLC	2R3K		Sc=6.24245, min distance = 1.97651
MTNK_BACSU F	23653520	CDK2_HUMAN Fu	6022 Aden	1GY3	2OLC	2R3L		Sc=6.23906, min distance = 1.92822
MTNK_BACSU F	23653521	CDK2_HUMAN Fu	6022 Aden	1GY3	2OLC	2R3M		Sc=6.2904, min distance = 1.847091
MTNK_BACSU F	23653524	CDK2_HUMAN Fu	6022 Aden	1GY3	2OLC	2R3P		Sc=6.22317, min distance = 1.76889
MTNK_BACSU F	23727982	CDK2_HUMAN Fu	6022 Aden	1GY3	2OLC	3BHU		Sc=6.27983, min distance = 2.29819
MTNK_BACSU F	24864079	CDK2_HUMAN Fu	6022 Aden	1GY3	2OLC	2VTP		Sc=6.35676, min distance = 1.60648
MTNK_BACSU F	24864080	CDK2_HUMAN Fu	6022 Aden	1GY3	2OLC	2VTR		Sc=5.90646, min distance = 2.18779
MTNK_BACSU F	24864081	CDK2_HUMAN Fu	6022 Aden	1GY3	2OLC	2VTS		Sc=6.29375, min distance = 1.27502
MTNK_BACSU F	24916751	CDK2_HUMAN Fu	6022 Aden	1GY3	2OLC	3DOG		Sc=6.27883, min distance = 1.71843
MTNK_BACSU F	2608 1jsv	CDK2_HUMAN Fu	6022 Aden	1GY3	2OLC	1JSV		Sc=5.77444, min distance = 1.79716
MTNK_BACSU F	3543 1g5s	CDK2_HUMAN Fu	6022 Aden	1GY3	2OLC	1G5S		Sc=6.4649, min distance = 1.238792
MTNK_BACSU F	443200 C1	ACK1_HUMAN Fu	91532 AMP	1U54	2PUL	1U4D		Sc=6.24295, min distance = 2.66731
MTNK_BACSU F	444345 1a	CDK2_HUMAN Fu	6022 Aden	1GY3	2OLC	1AQ1		Sc=6.35042, min distance = 2.03014
MTNK_BACSU F	444564 AD	BIOD_ECOLI Fu	91532 AMP	1DAG	2PUL	1BS1	0.91	Sc=5.62161, min distance = 2.18644
MTNK_BACSU F	444564 AD	MYS2_DICDI Fu	6022 Aden	1VOM	2OLC	1W9I	0.91	Sc=5.63256, min distance = 2.15173
MTNK_BACSU F	445840 di	CDK2_HUMAN Fu	6022 Aden	1GY3	2OLC	1GII		Sc=6.28348, min distance = 2.66283
MTNK_BACSU F	445940 O6	CDK2_HUMAN Fu	6022 Aden	1GY3	2OLC	1GZ8		Sc=6.12943, min distance = 2.09362
MTNK_BACSU F	445966 O6	CDK2_HUMAN Fu	6022 Aden	1GY3	2OLC	1H0V		Sc=6.16128, min distance = 2.47444
MTNK_BACSU F	447652 Im	CDK2_HUMAN Fu	6022 Aden	1GY3	2OLC	1OIQ		Sc=6.14509, min distance = 2.43844
MTNK_BACSU F	447656 1c	CDK2_HUMAN Fu	6022 Aden	1GY3	2OLC	1OIY		Sc=6.45007, min distance = 1.56761
MTNK_BACSU F	447766 1p	CDK2_HUMAN Fu	6022 Aden	1GY3	2OLC	1P2A		Sc=6.2901, min distance = 2.253181
MTNK_BACSU F	447821 CI	CDK2_HUMAN Fu	6022 Aden	1GY3	2OLC	1PF8		Sc=6.15048, min distance = 1.68357
MTNK_BACSU F	447916 ac	RIO1_ARCFU Fu	6022 Aden	1ZTH	2OLC	1ZTF	0.95	Sc=6.15666, min distance = 2.89751
MTNK_BACSU F	447962 1p	CDK2_HUMAN Fu	6022 Aden	1GY3	2OLC	2C5N		Sc=6.13359, min distance = 2.77672
MTNK_BACSU F	448222 NE	ENPL_CANFA Fu	6022 Aden	1TC6	2OLC	1QY5	0.81	Sc=6.34962, min distance = 2.05123
MTNK_BACSU F	448310 CI	KIPN_BPT4 Ful	6022 Aden	1LTQ	2OLC	1RC8		Sc=5.85332, min distance = 2.10751
MTNK_BACSU F	4564 1e1v	CDK2_HUMAN Fu	6022 Aden	1GY3	2OLC	1E1V		Sc=6.10007, min distance = 2.36722
MTNK_BACSU F	5288711 1	CDK2_HUMAN Fu	6022 Aden	1GY3	2OLC	1KE8		Sc=6.12053, min distance = 2.70542
MTNK_BACSU F	5326739 i	GSK3B_HUMAN F	6022 Aden	1J1C	2OLC	1Q41		Sc=6.27713, min distance = 2.33839

MTNK_BACSU F	5327096 2	CDK2_HUMAN Fu	6022 Aden	1GY3	2OLC	2BTR			Sc=6.10516, min distance = 1.576262
MTNK_BACSU F	5327097 2	CDK2_HUMAN Fu	6022 Aden	1GY3	2OLC	2BTS			Sc=5.884, min distance = 1.9140545
MTNK_BACSU F	5327135 D	CDK2_HUMAN Fu	6022 Aden	1GY3	2OLC	2C6M			Sc=6.14179, min distance = 2.305903
MTNK_BACSU F	5331010 c	CDK2_HUMAN Fu	6022 Aden	1GY3	2OLC	2BKZ			Sc=6.19613, min distance = 2.388503
MTNK_BACSU F	5957 Ader	BIOD_ECOLI Fu	91532 AMP	1DAG	2PUL	1A82		0.99	Sc=5.67253, min distance = 2.155125
MTNK_BACSU F	6083 ader	PSPF_ECOLI Fu	6022 Aden	2C98	2OLC	2VII		0.99	Sc=6.30457, min distance = 2.049417
MTNK_BACSU F	60961 ade	IPKA_RABIT Fu	6022 Aden	1JBP	2OLC	1FMO		0.95	Sc=6.1349, min distance = 2.8243806
MTNK_BACSU F	6420139 C	CDK2_HUMAN Fu	6022 Aden	1GY3	2OLC	2C5V			Sc=6.10545, min distance = 2.189625
MTNK_BACSU F	8977 1dar	NDK_PYRHO Full	6022 Aden	2DYA	2OLC	2DXE		0.8	Sc=5.96703, min distance = 2.103694
MTNK_BACSU F	8977 1dar	PARM_ECOLX Fu	6022 Aden	1MWM	2OLC	2ZGY		0.8	Sc=5.97258, min distance = 2.050772
MTNK_BACSU F	8977 1dar	RIR1_YEAST Fu	6022 Aden	2CVX	2OLC	2CVW		0.8	Sc=5.63619, min distance = 2.158916
MTNK_BACSU F	9817550 V	CDK2_HUMAN Fu	6022 Aden	1GY3	2OLC	3BHV			Sc=6.3834, min distance = 2.1410656
MTNK_BACSU F	9991833 S	CDK2_HUMAN Fu	6022 Aden	1GY3	2OLC	2R3H			Sc=6.10815, min distance = 1.450112
MTNK_BACSU F	9994066 4	CDK2_HUMAN Fu	6022 Aden	1GY3	2OLC	2VTJ			Sc=5.7577, min distance = 2.9164226
MTNN_ECOLI F	100684 9-	RICI_RICCO Fu	190 adeni	2P8N	1JYS	1IL4			Sc=5.84867, min distance = 2.585690
MTNN_ECOLI F	11979 5-M	COBT_SALTY Fu	190 adeni	1JH8	1JYS	1JHM			Sc=5.67531, min distance = 1.831088
MTNN_ECOLI F	444632 ne	RICI_RICCO Fu	190 adeni	2P8N	1JYS	1BR5			Sc=5.75593, min distance = 3.130981
MTNN_ECOLI F	446312 1i	RICI_RICCO Fu	190 adeni	2P8N	1JYS	1IL9			Sc=5.87488, min distance = 2.170995
MTNN_ECOLI F	5287547 6	PURR_ECOLI Fu	190 adeni	2PUB	1JYS	2PUA		0.9	Sc=5.74599, min distance = 2.915408
MTNN_ECOLI F	5287830 6	Q8RLY5_LACHE I	190 adeni	1S2D	1JYS	1S2I		0.93	Sc=5.70741, min distance = 1.874838
MTNN_ECOLI F	764 guani	PURR_ECOLI Fu	190 adeni	2PUB	1JYS	1WET			Sc=5.82763, min distance = 2.079610
MTNN_ECOLI F	76635 4,5	COBT_SALTY Fu	190 adeni	1JH8	1JYS	1L4F			Sc=5.67883, min distance = 1.980531
MTNN_ECOLI F	790 hypox	PURR_ECOLI Fu	190 adeni	2PUB	1JYS	1JFT			Sc=5.71862, min distance = 2.946212
MTNN_ECOLI F	96253 7-D	RICI_RICCO Fu	190 adeni	2P8N	1JYS	1IL3			Sc=5.84332, min distance = 2.393461
MTP2_PROVU F	122068 de	HNMT_HUMAN Fu	439155 Ad	2AOT	1BOO	2AOU			Sc=6.27898, min distance = 1.670927
MTP2_PROVU F	188380 Ac	MCES_ENCCU Fu	439155 Ad	1RI1	1BOO	1Z3C		0.91	Sc=6.56715, min distance = 2.143324
MTP2_PROVU F	446535 CI	HNMT_HUMAN Fu	439155 Ad	2AOT	1BOO	1JQE		0.95	Sc=6.53067, min distance = 2.033165
MTP2_PROVU F	60961 ade	PIMT_PYRFU Fu	439155 Ad	1JG1	1BOO	1JG2		0.84	Sc=6.27328, min distance = 2.727729
MTP2_PROVU F	65482 sir	ERM_BACSU Full	439155 Ad	1QAN	1BOO	1QAQ		0.88	Sc=6.58861, min distance = 2.051562
MTP2_PROVU F	65482 sir	MTR1_RHOSH Fu	439155 Ad	1NW7	1BOO	1NW6		0.88	Sc=6.58785, min distance = 2.044457
MTR1_RHOSH F	16741210	MTTA_THEAQ Fu	34756 Acy	2ADM	1NW5	2JG3			Sc=6.331, min distance = 2.0292781
MTR1_RHOSH F	16741210	MTTA_THEAQ Fu	439155 Ad	1AQI	1NW7	2JG3			Sc=5.98589, min distance = 1.904586
MTR1_RHOSH F	4369234 C	COMT_RAT Full	34756 Acy	2CL5	1NW5	1JR4			Sc=5.97772, min distance = 2.341700
MTR1_RHOSH F	445762 5'	MTTA_THEAQ Fu	34756 Acy	2ADM	1NW5	2IH2		0.93	Sc=6.32514, min distance = 2.468583
MTR1_RHOSH F	445971 CI	COMT_RAT Full	34756 Acy	2CL5	1NW5	1H1D		0.92	Sc=6.52246, min distance = 2.579711
MTR1_RHOSH F	5327118 5	Q70GK9_STRCT I	34756 Acy	1RQP	1NW5	2C2W		0.81	Sc=5.73619, min distance = 1.265702
MTTA_THEAQ F	4369533 C	SETD8_HUMAN F	439155 Ad	1ZKK	1AQI	2BQZ		0.95	Sc=6.0753, min distance = 2.0330238

MTTA_THEAQ F	439176 Me	MTR1_RHOSH Ful	65482 sin	1NW6	1AQJ	1EG2	0.8	Sc=6.20401, min distance = 2.30455
MTTA_THEAQ F	445971 CI	COMT_RAT Full=	34756 Acy	2CL5	2ADM	1H1D	0.92	Sc=6.45301, min distance = 2.52416
MTTA_THEAQ F	446535 CI	HNMT_HUMAN Ful	439155 Ad	2AOT	1AQI	1JQE	0.95	Sc=6.12973, min distance = 1.99751
MUP1_MOUSE F	445180 CI	OBP_PIG Full=(	32594 1dz	1DZK	1QY1	1DZJ		Sc=5.75169, min distance = 2.44640
MUP2_MOUSE F	7037 Diph	OBP_PIG Full=(	32594 1dz	1DZK	2NND	1DZP		Sc=5.67886, min distance = 2.06998
MUP2_MOUSE F	8184 Unde	OBP_PIG Full=(	32594 1dz	1DZK	2NND	1E02		Sc=5.62067, min distance = 2.09159
MURA_ECOLI F	448871 CI	GALE_ECOLI Ful	6031 Urid	1NAH	2Z2C	1UDA	0.87	Sc=6.12343, min distance = 2.03879
MURA_ECOLI F	6030 Uric	GLMU_HAEIN Ful	6031 Urid	2V0K	2Z2C	2V0J	0.99	Sc=6.26874, min distance = 2.13026
MURA_ECOLI F	6030 Uric	O87988_BORBR	6031 Urid	2PZM	2Z2C	2PZL	0.99	Sc=6.23512, min distance = 2.04542
MURA_ECOLI F	6132 Cyti	ECX1_PYRAB Ful	6031 Urid	2PNZ	2Z2C	2PO2	0.83	Sc=6.30768, min distance = 2.15345
MURA_ECOLI F	6133 uric	PYRH_ECOLI Ful	6031 Urid	2BND	2Z2C	2BNF	1	Sc=5.89172, min distance = 1.62070
MURA_ECOLI F	8629 UDP-	GALE_ECOLI Ful	6031 Urid	1NAH	2Z2C	1LRL	0.89	Sc=6.57063, min distance = 2.05834
MURA_ECOLI F	8629 UDP-	Q5IFH7_MEDTR	6031 Urid	2ACV	2Z2C	2ACW	0.89	Sc=6.57447, min distance = 1.89963
MURA_HAEIN F	24794398	MURA_ECOLI Ful	445675 UD	1UAE	2RL1	2Z2C		Sc=5.81897, min distance = 2.78257
MURA_HAEIN F	444127 CI	MURA_ECOLI Ful	445675 UD	1UAE	2RL1	1A2N	0.93	Sc=6.28849, min distance = 2.61856
MURC_HAEIN F	5957 Ader	Q5SJV7_THET8	91532 AMP	2Z09	1GQY	2Z08	0.99	Sc=6.48384, min distance = 1.95416
MURC_HAEIN F	6022 Ader	F263_HUMAN Ful	91532 AMP	2DWP	1GQY	2AXN	0.99	Sc=6.45105, min distance = 2.38458
MURC_HAEIN F	6022 Ader	RBSK_ECOLI Ful	91532 AMP	1GQT	1GQY	1RKD	0.99	Sc=6.46408, min distance = 2.23406
MURD_ECOLI F	447916 ac	RIO1_ARCFU Ful	6022 Aden	1ZTH	2JFG	1ZTF	0.95	Sc=6.31434, min distance = 2.05695
MURD_ECOLI F	447955 1p	CDK2_HUMAN Ful	6022 Aden	1GY3	2JFG	1PXI		Sc=5.81598, min distance = 2.35922
MURD_ECOLI F	6031 Uric	RIR1_YEAST Ful	6022 Aden	2CVX	2JFG	2CVV		Sc=6.4031, min distance = 2.0902224
MURD_ECOLI F	6083 ader	HSP71_HUMAN F	6022 Aden	1S3X	2JFG	1XQS	0.99	Sc=6.41996, min distance = 1.72629
MURD_ECOLI F	8977 1dar	NDK_PYRHO Full	6022 Aden	2DYA	2JFG	2DXE	0.8	Sc=6.49764, min distance = 2.04753
MURD_ECOLI F	8977 1dar	O33839_THEMA	6022 Aden	1XJK	2JFG	1XJE	0.8	Sc=6.50154, min distance = 2.22376
MUTB_PROFR F	5327118 5	Q70GK9_STRCT	439182 5'	2CC2	4REQ	2C2W	0.94	Sc=5.90479, min distance = 0.80640
MUTB_PROFR F	6852187 2	Q70GK9_STRCT	439182 5'	2CC2	4REQ	2CBX	0.97	Sc=6.09466, min distance = 1.98554
MUTL_ECO57 F	16058649	IGF1R_HUMAN F	33113 gam	1JQH	1NHI	2OJ9		Sc=6.52031, min distance = 1.67014
MUTL_ECO57 F	16750062	CSK2A_MAIZE F	33113 gam	1LP4	1NHI	2OXD		Sc=5.62446, min distance = 2.46124
MUTL_ECO57 F	188966 dA	HSLU_ECOLI Ful	33113 gam	1E94	1NHI	1G4A	0.97	Sc=6.37875, min distance = 2.04032
MUTL_ECO57 F	5326976 1	CSK2A_MAIZE F	33113 gam	1LP4	1NHI	1ZOE		Sc=5.68718, min distance = 2.65299
MUTL_ECO57 F	5327148 C	IPKA_RABIT Ful	33113 gam	1CDK	1NHI	2ERZ		Sc=6.17763, min distance = 2.05427
MUTL_ECOLI F	11963551	HSP82_YEAST F	6022 Aden	1AMW	1B62	2IWS		Sc=6.22185, min distance = 2.05436
MUTL_ECOLI F	11963552	HSP82_YEAST F	6022 Aden	1AMW	1B62	2IWU		Sc=6.22895, min distance = 2.03702
MUTL_ECOLI F	16750062	CSK2A_MAIZE F	33113 gam	1LP4	1B63	2OXD		Sc=5.63829, min distance = 2.39523
MUTL_ECOLI F	440317 AT	BCKD_RAT Full=	6022 Aden	1GKZ	1B62	1GJV	0.99	Sc=6.01997, min distance = 2.46694
MUTL_ECOLI F	444503 FU	NDKC_DICDI Ful	6022 Aden	1KDN	1B62	1B99		Sc=6.27581, min distance = 2.06999
MUTL_ECOLI F	5326976 1	CSK2A_MAIZE F	33113 gam	1LP4	1B63	1ZOE		Sc=5.69297, min distance = 2.63960

MUTL_ECOLI Fı	5327103 2	HS90A_HUMAN Fı	6022 Aden	1BYQ	1B62	2BYH			Sc=6.48066, min distance = 2.19529
MUTL_ECOLI Fı	5327134 C	CDK2_HUMAN Fu	6022 Aden	1GY3	1B62	2C6L			Sc=6.43562, min distance = 1.968308
MUTL_ECOLI Fı	5327148 C	IPKA_RABIT Fu	33113 gam	1CDK	1B63	2ERZ			Sc=6.18811, min distance = 2.07591
MUTL_ECOLI Fı	60961 ade	STK6_HUMAN Fu	33113 gam	2DWB	1B63	1MUO		0.93	Sc=6.18281, min distance = 2.40317
MUTL_ECOLI Fı	6132 Cyti	O33839_THEMA	6022 Aden	1XJK	1B62	1XJN			Sc=6.3191, min distance = 1.983989
MUTL_ECOLI Fı	6323491 M	TOP6B_SULSH Fı	6022 Aden	1Z5B	1B62	2HKJ			Sc=6.33524, min distance = 2.36454
MUTL_ECOLI Fı	72194 2-C	ENPL_CANFA Fu	6022 Aden	1TC6	1B62	1QYE		0.89	Sc=6.07109, min distance = 2.63905
MUTL_ECOLI Fı	8977 1dar	NDK_PYRHO Full	6022 Aden	2DYA	1B62	2DXE		0.8	Sc=5.99611, min distance = 2.24306
MUTL_ECOLI Fı	8977 1dar	O33839_THEMA	6022 Aden	1XJK	1B62	1XJE		0.8	Sc=6.44157, min distance = 2.04199
MUTS_ECOLI Fı	447004 CI	MYS2_DICDI Fu	5957 Aden	1FMW	1W7A	1LVK			Sc=5.8995, min distance = 0
MUTS_ECOLI Fı	448042 2e	KAPCA_BOVIN Fı	5957 Aden	1Q24	1W7A	1Q8T			Sc=6.1096, min distance = 2.423403
MUTS_ECOLI Fı	6083 ader	PURP_METJA Fu	5957 Aden	2R7L	1W7A	2R7M		0.98	Sc=5.74718, min distance = 2.00377
MUTS_ECOLI Fı	6338561 C	MYS2_DICDI Fu	5957 Aden	1FMW	1W7A	1D0X			Sc=6.19202, min distance = 0
MUTS_THEAQ Fı	24832022	HS90A_HUMAN Fı	6022 Aden	1BYQ	1FW6	2QG2			Sc=6.41578, min distance = 1.17327
MUTS_THEAQ Fı	24864077	CDK2_HUMAN Fu	6022 Aden	1GY3	1FW6	2VTN			Sc=6.27599, min distance = 2.36629
MUTS_THEAQ Fı	2608 1jsv	CDK2_HUMAN Fu	6022 Aden	1GY3	1FW6	1JSV			Sc=6.05437, min distance = 1.17327
MUTS_THEAQ Fı	72194 2-C	ENPL_CANFA Fu	6022 Aden	1TC6	1FW6	1QYE		0.89	Sc=6.15756, min distance = 1.89956
MUTY_ECOLI Fı	100684 9-	RICI_RICCO Fu	190 adeni	2P8N	1WEI	1IL4			Sc=5.6743, min distance = 2.732309
MUTY_ECOLI Fı	444632 ne	RICI_RICCO Fu	190 adeni	2P8N	1WEI	1BR5			Sc=6.13291, min distance = 2.50260
MUTY_ECOLI Fı	764 guani	PURR_ECOLI Fu	190 adeni	2PUB	1WEI	1WET			Sc=5.64684, min distance = 2.99363
MUTY_ECOLI Fı	96253 7-D	RICI_RICCO Fu	190 adeni	2P8N	1WEI	1IL3			Sc=5.60481, min distance = 2.73439
MVAA_PSEMV Fı	5957 Ader	MAOM_HUMAN Fu	5893 nadi	1PJ3	1QAX	1GZ4			Sc=5.99292, min distance = 1.78869
MYG_CARCR Fu	11957385	MYG_PHYCA Full	444098 HE	1A6M	1LHS	2CMM			Sc=6.18781, min distance = 2.48364
MYG_ELEMA Fu	11957385	MYG_PHYCA Full	444098 HE	1A6M	1EMY	2CMM			Sc=6.05379, min distance = 2.31324
MYG_HORSE Fu	11957364	HMOX1_HUMAN Fı	444124 HE	1OZW	1DWT	1S13		0.86	Sc=6.44293, min distance = 1.86131
MYG_HORSE Fu	11957370	HMOX1_HUMAN Fı	444124 HE	1OZW	1DWT	1TWN			Sc=6.35517, min distance = 2.30915
MYG_HUMAN Fu	11957385	MYG_PHYCA Full	444098 HE	1A6M	2MM1	2CMM			Sc=6.06387, min distance = 2.73176
MYG_HUMAN Fu	16741061	MYG_PHYCA Full	444098 HE	1A6M	2MM1	1MBC		0.83	Sc=6.58727, min distance = 2.70069
MYG_HUMAN Fu	4369122 C	GLB1_LUCPE Fu	444098 HE	1FLP	2MM1	1B0B		0.96	Sc=6.58663, min distance = 2.51960
MYG_PHYCA Fu	11957363	HBB_HUMAN Full	4368958 C	1COH	1YOG	1RQA	26.53	0.8	Sc=6.65561, min distance = 2.31443
MYG_PHYCA Fu	11957363	HBB_HUMAN Full	444097 He	1RPS	2EVK	1RQA	26.53	0.77	Sc=6.54215, min distance = 2.91266
MYG_PHYCA Fu	11957363	HBB_HUMAN Full	444098 HE	1J40	1A6M	1RQA	26.53	0.77	Sc=6.66875, min distance = 2.08654
MYG_PHYCA Fu	6420167 M	CPXA_PSEPU Fu	16741185	2Z97	2EKU	2FE6		0.83	Sc=6.60064, min distance = 2.04174
MYG_PIG Full	11957364	HMOX1_HUMAN Fı	444124 HE	1OZW	1MYH	1S13		0.86	Sc=6.4332, min distance = 1.585096
MYG_PIG Full	11957370	HMOX1_HUMAN Fı	444124 HE	1OZW	1MYH	1TWN			Sc=6.36494, min distance = 2.24233
MYG_PIG Full	11957371	HMOX1_HUMAN Fı	444124 HE	1OZW	1MYH	1TWR			Sc=6.36263, min distance = 2.33424
MYG_THUAL Fu	11957385	MYG_PHYCA Full	444098 HE	1A6M	1MYT	2CMM			Sc=5.94417, min distance = 2.38570

MYH11_CHICK I	1540 1h1q	CDK2_HUMAN Ful	6022 Aden	1GY3	1BR2	1H1Q		Sc=6.40317, min distance = 1.839959
MYH11_CHICK I	2608 1jsv	CDK2_HUMAN Ful	6022 Aden	1GY3	1BR2	1JSV		Sc=5.99875, min distance = 2.064709
MYH11_CHICK I	444564 AD	MYS2_DICDI Ful	6022 Aden	1VOM	1BR2	1W9I	0.91	Sc=5.98032, min distance = 2.385097
MYH11_CHICK I	444852 CI	ATPB_BOVIN Ful	6022 Aden	2CK3	1BR2	1COW	0.9	Sc=6.50029, min distance = 2.106442
MYH11_CHICK I	445966 O6	CDK2_HUMAN Ful	6022 Aden	1GY3	1BR2	1H0V		Sc=6.18281, min distance = 1.985335
MYH11_CHICK I	447649 1c	CDK2_HUMAN Ful	6022 Aden	1GY3	1BR2	1OI9		Sc=6.46472, min distance = 1.955091
MYH11_CHICK I	448310 CI	KIPN_BPT4 Ful	6022 Aden	1LTQ	1BR2	1RC8		Sc=5.97321, min distance = 1.994192
MYH11_CHICK I	4564 1e1v	CDK2_HUMAN Ful	6022 Aden	1GY3	1BR2	1E1V		Sc=6.1571, min distance = 2.1655163
MYH11_CHICK I	4566 1h1s	CDK2_HUMAN Ful	6022 Aden	1GY3	1BR2	1H1S		Sc=6.40975, min distance = 2.162221
MYH11_CHICK I	5289411 1	CDK2_HUMAN Ful	6022 Aden	1GY3	1BR2	1OGU		Sc=6.55088, min distance = 1.989650
MYH11_CHICK I	5327133 C	CDK2_HUMAN Ful	6022 Aden	1GY3	1BR2	2C6K		Sc=6.33058, min distance = 2.023439
MYH11_CHICK I	5327135 D	CDK2_HUMAN Ful	6022 Aden	1GY3	1BR2	2C6M		Sc=6.25403, min distance = 1.988613
MYH11_CHICK I	5957 Aden	BIOD_ECOLI Ful	6022 Aden	1DAD	1BR2	1A82	0.99	Sc=6.51159, min distance = 2.647586
MYH11_CHICK I	5957 Aden	RK_BOVIN Full=	6022 Aden	3C4Z	1BR2	3C4W	0.99	Sc=6.50846, min distance = 1.657269
MYH11_CHICK I	6132 Cyti	ECX1_PYRAB Ful	6022 Aden	2PO0	1BR2	2PO2		Sc=5.90525, min distance = 2.051027
MYH11_CHICK I	6132 Cyti	ECX2_PYRAB Ful	6022 Aden	2PO0	1BR2	2PO2		Sc=5.89507, min distance = 2.067023
MYH11_CHICK I	6338562 C	MYS2_DICDI Ful	6022 Aden	1VOM	1BR2	1D0Y		Sc=6.26552, min distance = 2.081163
MYH11_CHICK I	6420139 C	CDK2_HUMAN Ful	6022 Aden	1GY3	1BR2	2C5V		Sc=6.29814, min distance = 1.597128
MYH11_CHICK I	656971 CI	CDK2_HUMAN Ful	6022 Aden	1GY3	1BR2	1Y8Y		Sc=5.99508, min distance = 2.170138
MYH11_CHICK I	91532 AMP	BIOD_ECOLI Ful	6022 Aden	1DAD	1BR2	1DAG	0.99	Sc=6.54015, min distance = 2.204866
MYO5A_CHICK I	11957393	FAK1_HUMAN Ful	6022 Aden	1MP8	1W7J	2ETM		Sc=6.34328, min distance = 2.257468
MYO5A_CHICK I	1540 1h1q	CDK2_HUMAN Ful	6022 Aden	1GY3	1W7J	1H1Q		Sc=6.35926, min distance = 2.038915
MYO5A_CHICK I	2608 1jsv	CDK2_HUMAN Ful	6022 Aden	1GY3	1W7J	1JSV		Sc=5.86166, min distance = 2.330105
MYO5A_CHICK I	447004 CI	MYS2_DICDI Ful	6022 Aden	1VOM	1W7J	1LVK		Sc=5.99783, min distance = 2.033056
MYO5A_CHICK I	447649 1c	CDK2_HUMAN Ful	6022 Aden	1GY3	1W7J	1OI9		Sc=6.41519, min distance = 2.089525
MYO5A_CHICK I	447916 ac	RIO1_ARCFU Ful	6022 Aden	1ZTH	1W7J	1ZTF	0.95	Sc=6.19006, min distance = 2.685175
MYO5A_CHICK I	4565 1h1r	CDK2_HUMAN Ful	6022 Aden	1GY3	1W7J	1H1R		Sc=6.33379, min distance = 1.882605
MYO5A_CHICK I	5327133 C	CDK2_HUMAN Ful	6022 Aden	1GY3	1W7J	2C6K		Sc=6.28068, min distance = 2.110340
MYO5A_CHICK I	5327134 C	CDK2_HUMAN Ful	6022 Aden	1GY3	1W7J	2C6L		Sc=6.35254, min distance = 2.107731
MYO5A_CHICK I	5327135 D	CDK2_HUMAN Ful	6022 Aden	1GY3	1W7J	2C6M		Sc=6.19776, min distance = 2.234481
MYO5A_CHICK I	5957 Aden	BIOD_ECOLI Ful	6022 Aden	1DAD	1W7J	1A82	0.99	Sc=6.05025, min distance = 2.446756
MYO5A_CHICK I	6031 Uric	DCK_HUMAN Ful	6022 Aden	1P5Z	1W7J	2ZIA		Sc=5.86086, min distance = 2.082779
MYO5A_CHICK I	6031 Uric	ECX1_PYRAB Ful	6022 Aden	2PO0	1W7J	2PNZ		Sc=5.86187, min distance = 2.280172
MYO5A_CHICK I	6083 aden	PSPF_ECOLI Ful	6022 Aden	2C98	1W7J	2VII	0.99	Sc=5.92099, min distance = 2.206292
MYO5A_CHICK I	6132 Cyti	ECX1_PYRAB Ful	6022 Aden	2PO0	1W7J	2PO2		Sc=5.86086, min distance = 2.227474
MYO5A_CHICK I	6132 Cyti	ECX2_PYRAB Ful	6022 Aden	2PO0	1W7J	2PO2		Sc=5.84898, min distance = 2.464385
MYO5A_CHICK I	6420139 C	CDK2_HUMAN Ful	6022 Aden	1GY3	1W7J	2C5V		Sc=6.26127, min distance = 1.608741

MYO5A_CHICK I	6852201 2	CDK2_HUMAN Fu	6022 Aden	1GY3	1W7J	2G9X			Sc=6.55948, min distance = 2.12101
MYO5A_CHICK I	91532 AMF	BIOD_ECOLI Fu	6022 Aden	1DAD	1W7J	1DAG		0.99	Sc=6.07269, min distance = 2.04727
MYO5A_CHICK I	91532 AMF	KIF1A_MOUSE Fu	6022 Aden	1I5S	1W7J	1I6I		0.99	Sc=6.06681, min distance = 2.37132
MYO5A_CHICK I	91532 AMF	NIFH1_AZOVI Fu	6022 Aden	1FP6	1W7J	2AFK		0.99	Sc=6.09714, min distance = 1.88216
MYO6_PIG Ful	444564 AD	BIOD_ECOLI Fu	6022 Aden	1DAD	2V26	1BS1		0.91	Sc=6.44771, min distance = 2.36195
MYO6_PIG Ful	445940 O6	CDK2_HUMAN Fu	6022 Aden	1GY3	2V26	1GZ8			Sc=6.18043, min distance = 2.15360
MYO6_PIG Ful	4564 1e1v	CDK2_HUMAN Fu	6022 Aden	1GY3	2V26	1E1V			Sc=6.15391, min distance = 2.30603
MYO6_PIG Ful	4565 1h1r	CDK2_HUMAN Fu	6022 Aden	1GY3	2V26	1H1R			Sc=6.3977, min distance = 1.928311
MYO6_PIG Ful	5957 Aden	BIOD_ECOLI Fu	6022 Aden	1DAD	2V26	1A82		0.99	Sc=6.5136, min distance = 2.419313
MYO6_PIG Ful	60961 ade	IPKA_RABIT Fu	6022 Aden	1JBP	2V26	1FMO		0.95	Sc=6.21668, min distance = 2.42558
MYO6_PIG Ful	6132 Cyti	ECX1_PYRAB Fu	6022 Aden	2PO0	2V26	2PO2			Sc=6.35597, min distance = 2.16168
MYP2_HORSE Fu	164795 N(	RBCMT_PEA Ful	23831 HEF	1MLV	1YIV	2H2J			Sc=5.65521, min distance = 1.64854
MYP2_HORSE Fu	447768 CI	PURR_BACSU Fu	23831 HEF	1O57	1YIV	1P4A			Sc=6.13007, min distance = 1.64153
MYP2_HORSE Fu	7054 Isat	AOFB_HUMAN Fu	444279 1s	1OJD	1YIV	1OJA			Sc=5.68777, min distance = 1.97299
MYP2_HORSE Fu	854023 Ep	Q8WSF8_APLCA I	23831 HEF	2BR7	1YIV	2BYQ			Sc=5.88071, min distance = 0.76355
MYRA_SINAL Fu	11987879	BGLA_THEMA Fu	447413 NT	2J7B	1E6Q	2J75	34.11		Sc=5.84332, min distance = 2.51527
MYRA_SINAL Fu	24963032	BGLA_THEMA Fu	447413 NT	2J7B	1E6Q	2VRJ	34.11		Sc=6.13217, min distance = 1.37696
MYRA_SINAL Fu	447607 1c	BGLA_THEMA Fu	445248 nc	2J78	1E6S	1OIF	34.11		Sc=5.75163, min distance = 0.96131
MYRA_SINAL Fu	447651 CI	BGLA_THEMA Fu	445248 nc	2J78	1E6S	2J77	34.11	0.81	Sc=5.85412, min distance = 2.73171
MYRA_SINAL Fu	448980 nc	BGLA_THEMA Fu	445248 nc	2J78	1E6S	1UZ1	34.11		Sc=5.89109, min distance = 1.79600
MYRA_SINAL Fu	448980 nc	BGLA_THEMA Fu	447413 NT	2J7B	1E6Q	1UZ1	34.11		Sc=5.85754, min distance = 2.52941
MYRA_SINAL Fu	5326892 C	BGLA_THEMA Fu	447413 NT	2J7B	1E6Q	2J7C	34.11		Sc=6.37513, min distance = 0.70133
MYRA_SINAL Fu	79025 alp	Q9C171_PIREQ I	7027 gluc	1W8T	1E6X	1GWM			Sc=5.77106, min distance = 2.15254
MYRA_SINAL Fu	9549193 C	BGLA_THEMA Fu	445248 nc	2J78	1E6S	2CES	34.11		Sc=6.03953, min distance = 2.42524
MYRA_SINAL Fu	9549193 C	BGLA_THEMA Fu	447413 NT	2J7B	1E6Q	2CES	34.11	0.81	Sc=6.03712, min distance = 2.57435
MYS2_DICDI Fu	11608401	KAPCA_BOVIN Fu	5957 Aden	1Q24	1FMW	2UVY			Sc=6.11019, min distance = 0
MYS2_DICDI Fu	11708454	KAPCA_BOVIN Fu	5957 Aden	1Q24	1FMW	2VNW			Sc=6.0847, min distance = 0
MYS2_DICDI Fu	16122607	PDPK1_HUMAN Fu	5957 Aden	2BIY	1FMW	2PE0			Sc=6.12976, min distance = 0
MYS2_DICDI Fu	188966 dA	HSLU_ECOLI Fu	33113 gan	1E94	1MMN	1G4A		0.97	Sc=6.41587, min distance = 1.75265
MYS2_DICDI Fu	2608 1jsv	CDK2_HUMAN Fu	6022 Aden	1GY3	1VOM	1JSV			Sc=5.91746, min distance = 2.00358
MYS2_DICDI Fu	3540 1yds	KAPCA_BOVIN Fu	5957 Aden	1Q24	1FMW	1YDS			Sc=5.87585, min distance = 0
MYS2_DICDI Fu	3547 Fasu	KAPCA_BOVIN Fu	5957 Aden	1Q24	1FMW	1Q8W			Sc=5.93852, min distance = 0
MYS2_DICDI Fu	3547 Fasu	ROCK1_HUMAN Fu	33113 gan	2V55	1MMN	2ESM			Sc=5.94346, min distance = 1.92470
MYS2_DICDI Fu	444852 CI	ATPA1_BOVIN Fu	5957 Aden	2V7Q	1FMW	1COW		0.91	Sc=6.52842, min distance = 0
MYS2_DICDI Fu	445940 O6	CDK2_HUMAN Fu	6022 Aden	1GY3	1VOM	1GZ8			Sc=6.19765, min distance = 2.01690
MYS2_DICDI Fu	445966 O6	CDK2_HUMAN Fu	6022 Aden	1GY3	1VOM	1H0V			Sc=6.1422, min distance = 2.014186
MYS2_DICDI Fu	447649 1c	CDK2_HUMAN Fu	6022 Aden	1GY3	1VOM	1OI9			Sc=6.44799, min distance = 1.77114

MYS2_DICDI F	447656 1c	CDK2_HUMAN Fu	6022 Aden	1GY3	1VOM	1OIY			Sc=6.5088, min distance = 2.012253
MYS2_DICDI F	447962 1p	CDK2_HUMAN Fu	6022 Aden	1GY3	1VOM	2C5N			Sc=6.31822, min distance = 1.69484
MYS2_DICDI F	449240 1y	KAPCA_BOVIN F	5957 Aden	1Q24	1FMW	1YDR			Sc=5.91553, min distance = 0
MYS2_DICDI F	4564 1e1v	CDK2_HUMAN Fu	6022 Aden	1GY3	1VOM	1E1V			Sc=6.1428, min distance = 2.106700
MYS2_DICDI F	4565 1h1r	CDK2_HUMAN Fu	6022 Aden	1GY3	1VOM	1H1R			Sc=6.36116, min distance = 1.98628
MYS2_DICDI F	4566 1h1s	CDK2_HUMAN Fu	6022 Aden	1GY3	1VOM	1H1S			Sc=6.39947, min distance = 1.76583
MYS2_DICDI F	5326739 i	GSK3B_HUMAN F	6022 Aden	1J1C	1VOM	1Q41			Sc=6.27467, min distance = 2.19123
MYS2_DICDI F	5327133 C	CDK2_HUMAN Fu	6022 Aden	1GY3	1VOM	2C6K			Sc=6.32209, min distance = 2.11370
MYS2_DICDI F	5327134 C	CDK2_HUMAN Fu	6022 Aden	1GY3	1VOM	2C6L			Sc=6.39399, min distance = 1.99125
MYS2_DICDI F	5327135 D	CDK2_HUMAN Fu	6022 Aden	1GY3	1VOM	2C6M			Sc=6.2364, min distance = 2.153878
MYS2_DICDI F	6031 Uric	DCK_HUMAN Ful	6022 Aden	1P5Z	1VOM	2ZIA			Sc=6.35668, min distance = 2.11322
MYS2_DICDI F	6083 aden	PSPF_ECOLI Fu	5957 Aden	2C96	1FMW	2VII		0.98	Sc=6.35183, min distance = 0
MYS2_DICDI F	6083 aden	SRC_CHICK Ful	440317 AT	3DQW	1MMG	3DQX		0.97	Sc=5.90021, min distance = 1.80280
MYS2_DICDI F	6132 Cyti	ECX1_PYRAB Fu	6022 Aden	2PO0	1VOM	2PO2			Sc=5.90593, min distance = 2.01767
MYS2_DICDI F	6132 Cyti	ECX2_PYRAB Fu	6022 Aden	2PO0	1VOM	2PO2			Sc=5.90464, min distance = 2.38457
MYS2_DICDI F	6420139 C	CDK2_HUMAN Fu	6022 Aden	1GY3	1VOM	2C5V			Sc=6.29006, min distance = 1.65172
MYS2_DICDI F	6852201 2	CDK2_HUMAN Fu	6022 Aden	1GY3	1VOM	2G9X			Sc=6.28649, min distance = 2.14172
MYS2_DICDI F	91532 AMP	BIOD_ECOLI Fu	444564 AD	1BS1	1W9I	1DAG		0.91	Sc=6.50774, min distance = 2.18602
MYS2_DICDI F	91532 AMP	KIF1A_MOUSE F	33113 gam	1VFV	1MMN	1I6I		0.99	Sc=6.08927, min distance = 2.32144
MYS_AEQIR Fu	16750062	CSK2A_MAIZE F	33113 gam	1LP4	1KQM	2OXD			Sc=5.70362, min distance = 2.40983
MYS_AEQIR Fu	188966 dA	HSLU_ECOLI Fu	33113 gam	1E94	1KQM	1G4A		0.97	Sc=6.41587, min distance = 2.04205
MYS_AEQIR Fu	3547 Fasu	ROCK1_HUMAN F	33113 gam	2V55	1KQM	2ESM			Sc=5.94101, min distance = 2.27442
MYS_AEQIR Fu	444564 AD	BIOD_ECOLI Fu	6022 Aden	1DAD	1KK8	1BS1		0.91	Sc=6.4564, min distance = 2.163399
MYS_AEQIR Fu	444564 AD	MYS2_DICDI Fu	6022 Aden	1VOM	1KK8	1W9I	50	0.91	Sc=5.98503, min distance = 2.08584
MYS_AEQIR Fu	445940 O6	CDK2_HUMAN Fu	6022 Aden	1GY3	1KK8	1GZ8			Sc=6.18169, min distance = 1.59060
MYS_AEQIR Fu	445966 O6	CDK2_HUMAN Fu	6022 Aden	1GY3	1KK8	1H0V			Sc=6.19664, min distance = 2.01736
MYS_AEQIR Fu	447052 CI	CSK2A_MAIZE F	33113 gam	1LP4	1KQM	1M2R			Sc=6.31593, min distance = 2.44073
MYS_AEQIR Fu	448042 2e	ROCK1_HUMAN F	33113 gam	2V55	1KQM	2ETR			Sc=6.22319, min distance = 2.03943
MYS_AEQIR Fu	5326977 1	CSK2A_MAIZE F	33113 gam	1LP4	1KQM	1ZOG			Sc=5.65149, min distance = 2.33713
MYS_AEQIR Fu	5326978 1	CSK2A_MAIZE F	33113 gam	1LP4	1KQM	1ZOH			Sc=5.63735, min distance = 2.60285
MYS_AEQIR Fu	6031 Uric	DCK_HUMAN Ful	6022 Aden	1P5Z	1KK8	2ZIA			Sc=6.35668, min distance = 1.99618
MYS_AEQIR Fu	6031 Uric	ECX1_PYRAB Fu	6022 Aden	2PO0	1KK8	2PNZ			Sc=6.35159, min distance = 2.11449
MYS_AEQIR Fu	60961 ade	PIM1_HUMAN Fu	33113 gam	1XR1	1KQM	1YI4		0.93	Sc=6.12979, min distance = 2.64764
MYS_AEQIR Fu	6132 Cyti	ECX1_PYRAB Fu	6022 Aden	2PO0	1KK8	2PO2			Sc=5.90905, min distance = 2.10554
MYS_AEQIR Fu	656971 CI	CDK2_HUMAN Fu	6022 Aden	1GY3	1KK8	1Y8Y			Sc=6.00741, min distance = 2.19379
MYS_AEQIR Fu	91532 AMP	KIF1A_MOUSE F	6022 Aden	1I5S	1KK8	1I6I		0.99	Sc=6.53262, min distance = 2.29273
MYS_AEQIR Fu	91532 AMP	PURT_ECOLI Fu	33113 gam	1EYZ	1KQM	1KJI		0.99	Sc=6.10247, min distance = 2.00345

NADB_ECOLI F	16740985	FRDA_SHEFR Fu	444188 CI	1M64	1KNR	1E39		0.93	Sc=6.44144, min distance = 2.45207
NADB_ECOLI F	444502 CI	GSHR_HUMAN Fu	444188 CI	3DK9	1KNR	1BWC		0.98	Sc=6.44213, min distance = 2.17992
NADB_ECOLI F	445029 FA	OXDA_PIG Full	444188 CI	1VE9	1KNR	1DAO		0.94	Sc=6.47632, min distance = 2.31769
NADB_ECOLI F	446013 1,	O28603_ARCFU	444188 CI	1JNR	1KNR	1JNZ		0.94	Sc=6.44883, min distance = 2.28292
NADB_ECOLI F	446013 1,	O28604_ARCFU	444188 CI	1JNR	1KNR	1JNZ		0.94	Sc=6.44832, min distance = 2.30401
NADB_ECOLI F	448054 CI	FRDA_SHEFR Fu	444188 CI	1M64	1KNR	1Y0P		0.97	Sc=6.44098, min distance = 2.35554
NADB_ECOLI F	449465 CI	GSHR_HUMAN Fu	444188 CI	3DK9	1KNR	3GRT		0.98	Sc=6.44263, min distance = 2.11629
NADB_ECOLI F	6420174 C	O28603_ARCFU	444188 CI	1JNR	1KNR	2FJB		0.92	Sc=6.45675, min distance = 2.30474
NADB_ECOLI F	6420174 C	O28604_ARCFU	444188 CI	1JNR	1KNR	2FJB		0.92	Sc=6.45706, min distance = 2.32813
NADB_SULTO F	11987634	GLPD_ECOLI Fu	444188 CI	2QCU	2E5V	2R4E		0.97	Sc=6.44648, min distance = 2.09647
NADB_SULTO F	16740985	FRDA_SHEFR Fu	444188 CI	1M64	2E5V	1E39		0.93	Sc=6.44985, min distance = 2.45249
NADB_SULTO F	444502 CI	FRDA_SHEFR Fu	444188 CI	1M64	2E5V	1QJD		0.98	Sc=6.45045, min distance = 2.44429
NADB_SULTO F	444502 CI	TYTR_TRYCR Fu	444188 CI	1BZL	2E5V	1GXF		0.98	Sc=6.44709, min distance = 2.20467
NADB_SULTO F	446013 1,	O28604_ARCFU	444188 CI	1JNR	2E5V	1JNZ		0.94	Sc=6.44739, min distance = 2.00419
NADB_SULTO F	448054 CI	FRDA_SHEFR Fu	444188 CI	1M64	2E5V	1Y0P		0.97	Sc=6.44795, min distance = 2.50312
NADB_SULTO F	6420174 C	O28603_ARCFU	444188 CI	1JNR	2E5V	2FJB		0.92	Sc=6.45293, min distance = 2.13325
NADC_MYCTU F	446857 7-	COBT_SALTY Fu	121992 ni	1L5O	1QPN	1L5L			Sc=6.09567, min distance = 1.68751
NADC_MYCTU F	446858 7-	COBT_SALTY Fu	121992 ni	1L5O	1QPN	1L5M			Sc=6.14611, min distance = 1.72659
NADC_SALTY F	1017 phth	NADC_HELPY Fu	1066 quin	2B7N	1QAP	2B7P			Sc=5.88557, min distance = 2.49788
NADD_ECOLI F	12530 n-T	ALBU_HUMAN Fu	311 citri	1TF0	1K4M	1HK4			Sc=5.85688, min distance = 2.17893
NADD_ECOLI F	12793 Phe	SRC_HUMAN Ful	311 citri	1O4L	1K4M	1O4O			Sc=5.67897, min distance = 2.11327
NADD_ECOLI F	439153 Di	ADH1B_HUMAN F	5893 nadi	1U3U	1K4M	1DEH		0.79	Sc=6.77675, min distance = 1.75591
NADD_ECOLI F	447530	SRC_HUMAN Ful	311 citri	1O4L	1K4M	1O4F			Sc=5.88391, min distance = 2.45740
NADD_ECOLI F	8158 Pela	ALBU_HUMAN Fu	311 citri	1TF0	1K4M	1E7E			Sc=5.88694, min distance = 1.07152
NADD_ECOLI F	91557 1tm	NMNA3_HUMAN F	5893 nadi	1NUU	1K4M	1NUT			Sc=6.52393, min distance = 1.92973
NADD_PSEAE F	12136 Ber	POL_HV1N5 Ful	311 citri	2GON	1YUM	9HVP			Sc=5.69984, min distance = 68.1827
NADD_PSEAE F	12793 Phe	SRC_HUMAN Ful	311 citri	1O4L	1YUM	1O4O			Sc=5.616, min distance = 2.0472813
NADD_PSEAE F	439183 1i	TPIS_TRYBB Fu	311 citri	2VEK	1YUM	1IIH			Sc=5.70644, min distance = 1.43863
NADD_PSEAE F	439459 Ho	NIFD_KLEPN Fu	311 citri	1H1L	1YUM	1QGU		0.92	Sc=5.91238, min distance = 2.35653
NADD_PSEAE F	439459 Ho	NIFK_KLEPN Fu	311 citri	1H1L	1YUM	1QGU		0.92	Sc=5.92123, min distance = 2.27051
NADD_PSEAE F	447550 ph	TPIS_TRYBB Fu	311 citri	2VEK	1YUM	4TIM			Sc=5.70644, min distance = 1.68331
NADD_PSEAE F	448573 FI	CASP7_HUMAN F	311 citri	2QL9	1YUM	1SHL			Sc=5.844, min distance = 2.1154195
NADD_STAAC F	91557 1tm	NMNA3_HUMAN F	165491 de	1NUQ	2H29	1NUT			Sc=6.52283, min distance = 2.22308
NADE_BACAN F	216878 CM	CYAA_BACAN Fu	91557 1tm	1S26	2PZ8	1SK6		0.96	Sc=6.30512, min distance = 1.27181
NADE_BACAN F	21826754	PYGM_RABIT Fu	6083 aden	8GPB	2PZA	3BD6			Sc=5.68689, min distance = 2.26399
NADE_BACAN F	24851689	PIM1_HUMAN Fu	6083 aden	1YXU	2PZA	3CY2			Sc=6.25297, min distance = 1.30887
NADE_BACAN F	445321 CI	NADE_BACSU Fu	6083 aden	2NSY	2PZA	1EE1	77.86	0.85	Sc=6.514, min distance = 1.6910582

NADE_BACAN Fı	445708 AM	NADE_ECOLI Fu:	6083 aden	1WXI	2PZA	1WXE	53.28	0.91	Sc=6.36984, min distance = 2.48261:
NADE_BACAN Fı	448895 AD	UMPK_YEAST Fu:	6083 aden	1UKZ	2PZA	1UKY		0.99	Sc=6.42539, min distance = 2.51588!
NADE_BACAN Fı	5281701 T	PIM1_HUMAN Fu:	6083 aden	1YXU	2PZA	2O65			Sc=6.38594, min distance = 1.37177(
NADE_BACAN Fı	5327121 I	PIM1_HUMAN Fu:	6083 aden	1YXU	2PZA	2C3I			Sc=6.28744, min distance = 2.35054!
NADE_BACAN Fı	5957 Ader	AAKG1_RAT Ful:	6083 aden	2V8Q	2PZA	2V92		0.98	Sc=5.76664, min distance = 1.23917‹
NADE_BACAN Fı	6022 Ader	PSPF_ECOLI Fu:	6083 aden	2VII	2PZA	2C98		0.99	Sc=5.7139, min distance = 2.023536!
NADE_BACAN Fı	6076 Cycl	PDE10_HUMAN Fı	6083 aden	2OUN	2PZA	2OUR		0.99	Sc=6.32013, min distance = 2.30674!
NADE_BACAN Fı	611002 Op	PIM1_HUMAN Fu:	6083 aden	1YXU	2PZA	1YXX			Sc=6.18446, min distance = 2.06973:
NADE_BACAN Fı	657135 Op	PIM1_HUMAN Fu:	6083 aden	1YXU	2PZA	1YXV			Sc=5.87977, min distance = 1.98781:
NADE_BACAN Fı	6802 guar	NADE_BACSU Fu:	6083 aden	2NSY	2PZA	1IH8	77.86	0.76	Sc=6.54319, min distance = 2.16026:
NADE_BACAN Fı	6804 guar	PDE10_HUMAN Fı	6083 aden	2OUN	2PZA	2OUQ		0.8	Sc=5.9565, min distance = 0
NADE_BACSU Fı	21826754	PYGM_RABIT Fu:	6083 aden	8GPB	2NSY	3BD6			Sc=5.685, min distance = 2.3446522:
NADE_BACSU Fı	445708 AM	NADE_ECOLI Fu:	6083 aden	1WXI	2NSY	1WXE	53.33	0.91	Sc=6.46526, min distance = 1.26769‹
NADE_BACSU Fı	447916 ac	RIO1_ARCFU Fu:	5957 Aden	1ZP9	1NSY	1ZTF		0.94	Sc=6.01305, min distance = 2.00735:
NADE_BACSU Fı	448895 AD	UMPK_YEAST Fu:	6083 aden	1UKZ	2NSY	1UKY		0.99	Sc=5.67979, min distance = 2.47425ɛ
NADE_BACSU Fı	5281701 T	PIM1_HUMAN Fu:	6083 aden	1YXU	2NSY	2O65			Sc=6.38015, min distance = 1.36365:
NADE_BACSU Fı	5327121 I	PIM1_HUMAN Fu:	6083 aden	1YXU	2NSY	2C3I			Sc=6.28165, min distance = 2.16707:
NADE_BACSU Fı	6022 Ader	PSPF_ECOLI Fu:	6083 aden	2VII	2NSY	2C98		0.99	Sc=5.71702, min distance = 2.13877:
NADE_BACSU Fı	611002 Op	PIM1_HUMAN Fu:	6083 aden	1YXU	2NSY	1YXX			Sc=6.17438, min distance = 2.14647!
NADE_BACSU Fı	657135 Op	PIM1_HUMAN Fu:	6083 aden	1YXU	2NSY	1YXV			Sc=5.78965, min distance = 2.03228:
NADE_BACSU Fı	6804 guar	PDE10_HUMAN Fı	6083 aden	2OUN	2NSY	2OUQ		0.8	Sc=5.95567, min distance = 0
NADE_BACSU Fı	91557 1tm	BLS_STRCL Ful:	6083 aden	1MC1	2NSY	1JGT		0.97	Sc=6.08101, min distance = 2.12806‹
NADE_ECOLI Fı	24180721	PIM1_HUMAN Fu:	6083 aden	1YXU	1WXI	3C4E			Sc=6.00741, min distance = 1.65827(
NADE_ECOLI Fı	3973 nche	PIM1_HUMAN Fu:	6083 aden	1YXU	1WXI	1YI3			Sc=5.89507, min distance = 2.18974‹
NADE_ECOLI Fı	6076 Cycl	PDE10_HUMAN Fı	6083 aden	2OUN	1WXI	2OUR		0.99	Sc=6.29836, min distance = 2.21733(
NADE_ECOLI Fı	611002 Op	PIM1_HUMAN Fu:	6083 aden	1YXU	1WXI	1YXX			Sc=6.14892, min distance = 2.38596(
NADE_ECOLI Fı	656968 1y	PDE4B_HUMAN Fı	6083 aden	1ROR	1WXI	1Y2H			Sc=5.9783, min distance = 2.0598126
NADE_HELPY Fı	16122635	KAPCA_BOVIN Fı	5957 Aden	1Q24	1XNG	2UW8			Sc=5.66414, min distance = 2.16187:
NADE_HELPY Fı	447916 ac	RIO1_ARCFU Fu:	5957 Aden	1ZP9	1XNG	1ZTF		0.94	Sc=6.26491, min distance = 1.76673:
NADE_HELPY Fı	6022 Ader	DDL_THET8 Ful:	5957 Aden	2ZDQ	1XNG	2ZDH		0.99	Sc=5.97404, min distance = 2.34357:
NADE_HELPY Fı	6022 Ader	FAK1_HUMAN Fu:	5957 Aden	2IJM	1XNG	1MP8		0.99	Sc=5.98372, min distance = 2.11881:
NADE_HELPY Fı	6022 Ader	PURP_METJA Fu:	5957 Aden	2R7L	1XNG	2R7N		0.99	Sc=5.98372, min distance = 2.37904:
NADE_HELPY Fı	6022 Ader	RK_BOVIN Full=	5957 Aden	3C4W	1XNG	3C4Z		0.99	Sc=5.98308, min distance = 2.19218:
NADE_HELPY Fı	6083 ader	PSPF_ECOLI Fu:	5957 Aden	2C96	1XNG	2VII		0.98	Sc=6.35571, min distance = 2.22425:
NADE_HELPY Fı	6083 ader	PURP_METJA Fu:	5957 Aden	2R7L	1XNG	2R7M		0.98	Sc=5.90214, min distance = 2.34869˜
NADE_HELPY Fı	6083 ader	PURP_PYRFU Fu:	5957 Aden	2R86	1XNG	2R85		0.98	Sc=5.89926, min distance = 2.13058(
NADE_HELPY Fı	6914611 p	KAPCA_BOVIN Fı	5957 Aden	1Q24	1XNG	2F7E			Sc=6.45491, min distance = 2.10312:

NADM_METJA F	129236 2c	CKI1_SCHPO Fu	5957 Aden	1CSN	1F9A	2CSN			Sc=5.94336, min distance = 1.93641
NADM_METJA F	16122642	KAPCA_BOVIN F	5957 Aden	1Q24	1F9A	2UZW			Sc=6.4628, min distance = 2.074404
NADM_METJA F	3547 Fasu	KAPCA_BOVIN F	5957 Aden	1Q24	1F9A	1Q8W			Sc=6.00586, min distance = 1.98828
NADM_METJA F	444564 AI	MYS2_DICDI Fu	5957 Aden	1FMW	1F9A	1W9I		0.91	Sc=6.4262, min distance = 0
NADM_METJA F	445432 CI	SYQ_ECOLI Ful	5957 Aden	1GTR	1F9A	1QTQ		0.85	Sc=5.79648, min distance = 1.87980
NADM_METJA F	447004 CI	MYS2_DICDI Fu	5957 Aden	1FMW	1F9A	1LVK			Sc=6.4962, min distance = 0
NADM_METJA F	449240 1y	KAPCA_BOVIN F	5957 Aden	1Q24	1F9A	1YDR			Sc=5.98993, min distance = 1.93389
NADM_METJA F	6022 Aden	BIOD_ECOLI Fu	5957 Aden	1A82	1F9A	1DAD		0.99	Sc=5.96079, min distance = 1.90842
NADM_METJA F	6022 Aden	DDL_THET8 Ful	5957 Aden	2ZDQ	1F9A	2ZDH		0.99	Sc=6.02896, min distance = 1.86631
NADM_METJA F	6022 Aden	FAK1_HUMAN Fu	5957 Aden	2IJM	1F9A	1MP8		0.99	Sc=5.99459, min distance = 2.28420
NADM_METJA F	6022 Aden	MALK_ECOLI Fu	5957 Aden	1Q12	1F9A	2AWN		0.99	Sc=6.48525, min distance = 1.42436
NADM_METJA F	6022 Aden	MUTS_ECOLI Fu	5957 Aden	1W7A	1F9A	1OH7		0.99	Sc=6.0563, min distance = 1.759974
NADM_METJA F	6022 Aden	Q72H90_THET2	5957 Aden	2BEK	1F9A	2BEJ		0.99	Sc=5.94326, min distance = 2.136984
NADM_METJA F	6022 Aden	Y059_METJA Fu	5957 Aden	2J9C	1F9A	2J9D		0.99	Sc=6.41531, min distance = 2.00015
NADM_METJA F	6083 aden	LPLA_THEAC Fu	5957 Aden	2ARU	1F9A	2ART		0.98	Sc=6.32215, min distance = 1.82810
NADM_METJA F	6083 aden	PSPF_ECOLI Fu	5957 Aden	2C96	1F9A	2VII		0.98	Sc=5.87454, min distance = 2.06801
NADM_METJA F	6083 aden	PURP_PYRFU Fu	5957 Aden	2R86	1F9A	2R85		0.98	Sc=6.37635, min distance = 1.96001
NADM_METJA F	6830 guar	Q381M1_9TRYP	5957 Aden	2Q0D	1F9A	2Q0E		0.8	Sc=6.12375, min distance = 1.69996
NADM_METJA F	6914613 p	KAPCA_BOVIN F	5957 Aden	1Q24	1F9A	2F7X			Sc=6.49168, min distance = 2.00365
NADM_METJA F	91532 AMI	Q5SJV7_THET8	5957 Aden	2Z08	1F9A	2Z09		0.99	Sc=6.17298, min distance = 2.14226
NADM_METJA F	91557 1tn	PANC_MYCTU Fu	5957 Aden	2A84	1F9A	1N2E		0.99	Sc=6.06852, min distance = 1.87242
NADM_METJA F	9547921 G	SYQ_ECOLI Ful	5957 Aden	1GTR	1F9A	2RE8		0.83	Sc=5.80307, min distance = 1.58004
NADM_SYNY3 F	91557 1tn	NMNA3_HUMAN F	5893 nadi	1NUU	2QJO	1NUT			Sc=6.52458, min distance = 2.02924
NAGAB_CHICK	439353 be	LEC_ERYCO Ful	84265 1ax	1AX0	1KTC	1AX1			Sc=5.74599, min distance = 2.29965
NAGAB_CHICK	448882 NC	LECA_ARTIN Fu	84265 1ax	1M26	1KTC	1UGX		0.96	Sc=6.11642, min distance = 2.250334
NAGAB_CHICK	76935 alp	LECA_ARTIN Fu	84265 1ax	1M26	1KTC	1WS4			Sc=5.69586, min distance = 2.44106
NAGAB_CHICK	76935 alp	Q868M7_9ECHN	84265 1ax	2Z48	1KTC	2Z49			Sc=5.86794, min distance = 1.87920
NAGAB_CHICK	79025 alp	TRFL_BOVIN Fu	84265 1ax	2AYS	1KTC	2DWJ			Sc=5.74599, min distance = 2.25439
NAGA_BACSU F	439284 Rc	GLMS_ECOLI Fu	439217 1m	1MOQ	2VHL	1MOR			Sc=5.99587, min distance = 2.02329
NAGB_BACSU F	444305 L-	NAGB_ECOLI Fu	69507 Neu	1FQO	2BKX	1FS5			Sc=5.63013, min distance = 2.80521
NAGB_ECOLI F	24139 ace	PGRP_CAMDR Fu	444305 L-	3C2X	1FS5	2R2K			Sc=5.92811, min distance = 1.96166
NAGB_ECOLI F	439710 rh	PGRP_CAMDR Fu	444305 L-	3C2X	1FS5	3CG9			Sc=5.67059, min distance = 2.45805
NAGB_STRMU F	439284 Rc	GLMS_ECOLI Fu	439217 1m	1MOQ	2RI1	1MOR			Sc=5.89235, min distance = 2.26446
NAGB_STRMU F	6102755 C	GLMS_ECOLI Fu	439217 1m	1MOQ	2RI1	2BPL			Sc=6.00499, min distance = 2.12631
NAGK_HUMAN F	101798 Me	MBL1_RAT Full	82313 1rc	1KWV	2CH5	1KWU			Sc=5.85789, min distance = 1.50794
NAGK_HUMAN F	15993 dAT	RECA_MYCTU Fu	6022 Aden	1MO3	2CH6	1MO6		0.97	Sc=6.48042, min distance = 1.412804
NAGK_HUMAN F	185698 al	MBL1_RAT Full	82313 1rc	1KWV	2CH5	2MSB			Sc=5.71748, min distance = 2.48140

NAGK_HUMAN F	185698 al	MBL2_RAT Full	82313 1rd	1RDN	2CH5	1RDL			Sc=5.86394, min distance = 2.33440
NAGK_HUMAN F	193758 Va	AMY1_HUMAN Fu	79025 alp	3DHP	2CH6	1MFU			Sc=5.72118, min distance = 1.43896
NAGK_HUMAN F	25058128	MURD_ECOLI Fu	6022 Aden	2JFG	2CH6	2VTD			Sc=6.48796, min distance = 1.68699
NAGK_HUMAN F	36735 Gpp	Q04230_ECOLX	6022 Aden	1GKI	2CH6	1GL6		0.79	Sc=6.53268, min distance = 1.11493
NAGK_HUMAN F	439692 D-	XYLA_STRRU Fu	79025 alp	1XIF	2CH6	4XIS			Sc=5.65079, min distance = 1.92256
NAGK_HUMAN F	439710 rh	PGRP_CAMDR Fu	82313 1rd	3C93	2CH5	3CG9			Sc=5.7479, min distance = 1.630435
NAGK_HUMAN F	439710 rh	UGL_BACGL Ful	82313 1rd	2FV1	2CH5	2FV0			Sc=5.64864, min distance = 2.26027
NAGK_HUMAN F	439764 be	Q9ZB17_9LACT	79025 alp	1NSZ	2CH6	1MMZ		0.93	Sc=5.63902, min distance = 2.42314
NAGK_HUMAN F	440115 N-	SFTPD_HUMAN F	79025 alp	1PWB	2CH6	2ORJ			Sc=6.10344, min distance = 1.43832
NAGK_HUMAN F	440317 AT	BCKD_RAT Full	6022 Aden	1GKZ	2CH6	1GJV		0.99	Sc=6.04497, min distance = 0.88387
NAGK_HUMAN F	444564 AD	BIOD_ECOLI Fu	6022 Aden	1DAD	2CH6	1BS1		0.91	Sc=6.4311, min distance = 2.064477
NAGK_HUMAN F	444564 AD	MYS2_DICDI Fu	6022 Aden	1VOM	2CH6	1W9I		0.91	Sc=6.44706, min distance = 1.98042
NAGK_HUMAN F	444809 Th	AMY1_HORVU Fu	79025 alp	1RP8	2CH6	1P6W		0.78	Sc=5.82133, min distance = 1.01882
NAGK_HUMAN F	444863 is	MBL1_RAT Full	82313 1rd	1KWV	2CH5	1KWX			Sc=5.7479, min distance = 2.064460
NAGK_HUMAN F	445825 CI	PYGM_RABIT Fu	79025 alp	1H5U	2CH6	1GG8			Sc=6.02904, min distance = 1.91435
NAGK_HUMAN F	445916 be	XYLA_STRRU Fu	79025 alp	1XIF	2CH6	1GW9		0.93	Sc=5.62221, min distance = 2.54005
NAGK_HUMAN F	447004 CI	MYS2_DICDI Fu	6022 Aden	1VOM	2CH6	1LVK			Sc=6.51755, min distance = 2.19010
NAGK_HUMAN F	447017 CI	NDKC_DICDI Fu	6022 Aden	1KDN	2CH6	1LWX			Sc=6.45898, min distance = 0.97885
NAGK_HUMAN F	447413 NT	PYGM_RABIT Fu	79025 alp	1H5U	2CH6	1NOJ			Sc=6.0025, min distance = 2.448421
NAGK_HUMAN F	447607 1c	PYGM_RABIT Fu	79025 alp	1H5U	2CH6	2G9V			Sc=5.71966, min distance = 2.63241
NAGK_HUMAN F	447955 1p	CDK2_HUMAN Fu	6022 Aden	1GY3	2CH6	1PXI			Sc=5.80215, min distance = 1.89308
NAGK_HUMAN F	449180 XI	XYLA_STRRU Fu	79025 alp	1XIF	2CH6	4XIS			Sc=5.67557, min distance = 2.56083
NAGK_HUMAN F	449225 3-	XYLA_STROL Fu	79025 alp	1S5M	2CH6	1XYC			Sc=5.94012, min distance = 2.03294
NAGK_HUMAN F	4564 1e1v	CDK2_HUMAN Fu	6022 Aden	1GY3	2CH6	1E1V			Sc=6.2419, min distance = 2.140152
NAGK_HUMAN F	5289590 D	XYLA_STRRU Fu	79025 alp	1XIF	2CH6	1XII			Sc=5.6803, min distance = 2.453731
NAGK_HUMAN F	5780 D-Sc	XYLA_STRRU Fu	79025 alp	1XIF	2CH6	1XIH		0.78	Sc=5.90364, min distance = 2.27611
NAGK_HUMAN F	5957 Aden	HSP7F_YEAST F	6022 Aden	3C7N	2CH6	3D2F		0.99	Sc=6.49587, min distance = 1.78676
NAGK_HUMAN F	5957 Aden	KTHY_HUMAN Fu	6022 Aden	1NN3	2CH6	1E2Q		0.99	Sc=6.49312, min distance = 1.96724
NAGK_HUMAN F	5957 Aden	MUTS_ECOLI Fu	6022 Aden	1OH7	2CH6	1W7A		0.99	Sc=6.51057, min distance = 1.80722
NAGK_HUMAN F	5957 Aden	PSPF_ECOLI Fu	6022 Aden	2C98	2CH6	2C96		0.99	Sc=6.50024, min distance = 2.04174
NAGK_HUMAN F	5957 Aden	PURL_THEMA Fu	6022 Aden	2HRU	2CH6	2HS0		0.99	Sc=6.14452, min distance = 2.05596
NAGK_HUMAN F	5957 Aden	RIO2_ARCFU Fu	6022 Aden	1ZAR	2CH6	1ZAO		0.99	Sc=6.45125, min distance = 2.05455
NAGK_HUMAN F	5957 Aden	VG17_BPT4 Ful	6022 Aden	2O0J	2CH6	2O0H		0.99	Sc=6.49477, min distance = 1.92374
NAGK_HUMAN F	5957 Aden	Y059_METJA Fu	6022 Aden	2J9D	2CH6	2J9C		0.99	Sc=6.49982, min distance = 1.94585
NAGK_HUMAN F	6027 xylc	XYLA_STRRU Fu	79025 alp	1XIF	2CH6	3XIS		0.93	Sc=5.62032, min distance = 2.18648
NAGK_HUMAN F	6031 Uric	DCK_HUMAN Ful	6022 Aden	1P5Z	2CH6	2ZIA			Sc=6.32462, min distance = 1.62143
NAGK_HUMAN F	6083 ader	PURP_METJA Fu	6022 Aden	2R7N	2CH6	2R7M		0.99	Sc=6.36839, min distance = 2.19377

NAGK_HUMAN F	6132 Cyti	ECX2_PYRAB Fu	6022 Aden	2PO0	2CH6	2PO2			Sc=6.31729, min distance = 1.929568
NAGK_HUMAN F	64960 Pol	XYLA_STRRU Fu	79025 alp	1XIF	2CH6	1XIE		0.89	Sc=5.75873, min distance = 2.378694
NAGK_HUMAN F	6804 guar	NDK_PYRHO Ful	6022 Aden	2DYA	2CH6	2DXF		0.8	Sc=6.41003, min distance = 1.740079
NAGK_HUMAN F	8977 1dar	NDK_PYRHO Ful	6022 Aden	2DYA	2CH6	2DXE		0.8	Sc=6.43482, min distance = 2.074755
NAGK_HUMAN F	91532 AMP	BIOD_ECOLI Fu	6022 Aden	1DAD	2CH6	1DAG		0.99	Sc=6.52984, min distance = 0.991182
NAGK_HUMAN F	91532 AMP	F263_HUMAN Fu	6022 Aden	2AXN	2CH6	2DWP		0.99	Sc=6.48439, min distance = 2.090849
NAGK_HUMAN F	91532 AMP	MTNK_BACSU Fu	6022 Aden	2OLC	2CH6	2PUL		0.99	Sc=6.5483, min distance = 0.9427942
NAGK_HUMAN F	95259 L-x	XYLA_STRRU Fu	79025 alp	1XIF	2CH6	1XIC			Sc=5.71033, min distance = 2.524570
NAGK_HUMAN F	9860102 G	PYGM_RABIT Fu	79025 alp	1H5U	2CH6	2G9R			Sc=6.09246, min distance = 2.065820
NAGZ_VIBCH F	126154 Cy	Q54276_SERMA	87901 EIN	1UR8	1Y65	1W1P			Sc=5.62446, min distance = 2.246881
NAGZ_VIBCH F	445247 CI	Q54276_SERMA	87901 EIN	1UR8	1Y65	1E6R			Sc=5.93288, min distance = 2.390538
NAGZ_VIBCH F	449094 Cy	Q54276_SERMA	87901 EIN	1UR8	1Y65	1W1T			Sc=6.22982, min distance = 0.720748
NAHC_PSEU8 F	178518 1l	BPHC_BURXL Fu	254 3-phe	1KMY	2EI3	1LGT	37.78	0.9	Sc=5.98028, min distance = 2.372336
NAHC_PSEU8 F	446948 CH	BPHC_BURXL Fu	254 3-phe	1KMY	2EI3	1LKD	37.78	0.88	Sc=5.99765, min distance = 2.056177
NAHD_PSEPU F	97536 Hex	URE2_YEAST Fu	124886 gl	1K0D	2IMF	1K0A		0.9	Sc=6.50303, min distance = 1.682151
NAMPT_HUMAN	9543513 F	NAMPT_RAT Ful	14181 nic	2G96	2GVG	2G97			Sc=6.47144, min distance = 1.009041
NAMPT_MOUSE	9543513 F	NAMPT_RAT Ful	14181 nic	2G96	2H3D	2G97			Sc=6.4666, min distance = 1.0966644
NAMPT_RAT Fu	457954 Ri	NRK1_HUMAN Fu	14181 nic	2QG6	2G96	2P0E			Sc=6.06375, min distance = 2.063945
NANA_STRPN F	24820115	NANB_STRPN Fu	65309 DAN	2VW1	2VVZ	2VW2			Sc=5.61415, min distance = 1.437336
NANA_STRPN F	449401 2,	NANL_MACDE Fu	65309 DAN	1SLI	2VVZ	2SLI			Sc=6.26513, min distance = 2.536931
NANB_STRPN F	444885 O-	NRAM_I75A5 Fu	65309 DAN	2QWC	2VW1	1MWE			Sc=5.90453, min distance = 2.284671
NANB_STRPN F	445078 CI	NANH_SALTY Fu	65309 DAN	2SIM	2VW1	1DIM			Sc=6.33302, min distance = 2.287147
NANB_STRPN F	445079 CI	NANH_SALTY Fu	65309 DAN	2SIM	2VW1	1DIL			Sc=6.33396, min distance = 2.247914
NANH_MICVI F	15991558	OGA_CLOPE Ful	439353 be	2J1A	2BZD	2J62	35.34		Sc=6.15333, min distance = 41.79262
NANH_MICVI F	444885 O-	NRAM_I75A5 Fu	65309 DAN	2QWC	1EUS	1MWE			Sc=5.91352, min distance = 2.398108
NANH_MICVI F	445078 CI	NANH_SALTY Fu	65309 DAN	2SIM	1EUS	1DIM	24.48		Sc=5.86193, min distance = 2.494258
NANH_MICVI F	445079 CI	NANH_SALTY Fu	65309 DAN	2SIM	1EUS	1DIL	24.48		Sc=5.86309, min distance = 2.454541
NANH_MICVI F	449381 Os	NRAM_I75A5 Fu	65309 DAN	2QWC	1EUS	2QWH			Sc=6.25083, min distance = 2.396801
NANH_MICVI F	449535 CI	NANL_MACDE Fu	65309 DAN	1SLI	1EUS	4SLI			Sc=6.39451, min distance = 2.474637
NANH_MICVI F	51 2-Oxop	SERA_ECOLI Fu	311 citri	2P9E	1W8O	1YBA		0.76	Sc=5.78653, min distance = 1.109921
NANH_VIBCH F	445078 CI	NANH_SALTY Fu	65309 DAN	2SIM	1W0O	1DIM			Sc=6.30896, min distance = 2.086405
NANH_VIBCH F	445079 CI	NANH_SALTY Fu	65309 DAN	2SIM	1W0O	1DIL			Sc=6.30127, min distance = 2.182926
NANH_VIBCH F	449380 2q	NRAM_I75A5 Fu	65309 DAN	2QWC	1W0O	2QWG		0.76	Sc=6.28306, min distance = 2.321508
NANH_VIBCH F	449401 2,	NANL_MACDE Fu	65309 DAN	1SLI	1W0O	2SLI			Sc=6.25167, min distance = 2.278853
NANH_VIBCH F	449535 CI	NANL_MACDE Fu	65309 DAN	1SLI	1W0O	4SLI			Sc=6.3807, min distance = 2.1938997
NANH_VIBCH F	60855 zar	NRAM_I75A5 Fu	444885 O-	1MWE	1W0P	1NNC			Sc=5.89969, min distance = 2.334137
NANH_VIBCH F	60855 zar	NRAM_I75A5 Fu	65309 DAN	2QWC	1W0O	1NNC		0.88	Sc=6.43515, min distance = 2.050181

NANL_MACDE F	445078 CI	NANH_SALTY Fu	65309 DAN	2SIM	1SLI	1DIM			Sc=5.85183, min distance = 2.49222
NANL_MACDE F	445079 CI	NANH_SALTY Fu	65309 DAN	2SIM	1SLI	1DIL			Sc=5.84034, min distance = 2.47020
NANL_MACDE F	449381 Os	NRAM_I75A5 Fu	65309 DAN	2QWC	1SLI	2QWH			Sc=6.23346, min distance = 2.43293
NAPA_DESDE F	445198 CI	DMSA_RHOCA Fu	4369050 F	1H5N	2NAP	1E61		0.98	Sc=6.8216, min distance = 2.019349
NAPA_DESDE F	5326486 C	DMSA_RHOCA Fu	4369050 F	1H5N	2NAP	1DMR		0.98	Sc=6.81873, min distance = 2.32568
NAPA_ECOLI F	445198 CI	DMSA_RHOCA Fu	4369050 F	1H5N	2NYA	1E61		0.98	Sc=6.81859, min distance = 2.12843
NAPA_ECOLI F	5326486 C	DMSA_RHOCA Fu	4369050 F	1H5N	2NYA	1DMR		0.98	Sc=6.81707, min distance = 2.14267
NCBP1_HUMAN	854023 Ep	Q8WSF8_APLCA	8200 TETR	2BYN	1N52	2BYQ			Sc=5.95863, min distance = 0.75212
NCD_DROME Fu	1540 1h1q	CDK2_HUMAN Fu	6022 Aden	1GY3	1N6M	1H1Q			Sc=6.32793, min distance = 2.32972
NCD_DROME Fu	23653516	CDK2_HUMAN Fu	6022 Aden	1GY3	1N6M	2R3G			Sc=5.95869, min distance = 2.19075
NCD_DROME Fu	24864080	CDK2_HUMAN Fu	6022 Aden	1GY3	1N6M	2VTR			Sc=5.90306, min distance = 2.16956
NCD_DROME Fu	2608 1jsv	CDK2_HUMAN Fu	6022 Aden	1GY3	1N6M	1JSV			Sc=5.96092, min distance = 2.15479
NCD_DROME Fu	444564 AD	BIOD_ECOLI Fu	6022 Aden	1DAD	1N6M	1BS1		0.91	Sc=5.68219, min distance = 2.22707
NCD_DROME Fu	444564 AD	MYS2_DICDI Fu	6022 Aden	1VOM	1N6M	1W9I		0.91	Sc=5.65217, min distance = 2.16835
NCD_DROME Fu	445840 di	CDK2_HUMAN Fu	6022 Aden	1GY3	1N6M	1GII			Sc=6.16417, min distance = 2.09729
NCD_DROME Fu	4564 1e1v	CDK2_HUMAN Fu	6022 Aden	1GY3	1N6M	1E1V			Sc=5.99449, min distance = 2.15786
NCD_DROME Fu	5327133 C	CDK2_HUMAN Fu	6022 Aden	1GY3	1N6M	2C6K			Sc=6.17469, min distance = 2.12794
NCD_DROME Fu	5327135 D	CDK2_HUMAN Fu	6022 Aden	1GY3	1N6M	2C6M			Sc=6.18092, min distance = 2.16322
NCD_DROME Fu	5957 Aden	MUTS_ECOLI Fu	6022 Aden	1OH7	1N6M	1W7A		0.99	Sc=5.71475, min distance = 2.04918
NCD_DROME Fu	6420138 2	CDK2_HUMAN Fu	6022 Aden	1GY3	1N6M	2UUE			Sc=6.40465, min distance = 1.09128
NCD_DROME Fu	656971 CI	CDK2_HUMAN Fu	6022 Aden	1GY3	1N6M	1Y8Y			Sc=5.83712, min distance = 2.26300
NCD_DROME Fu	91532 AMF	BIOD_ECOLI Fu	6022 Aden	1DAD	1N6M	1DAG		0.99	Sc=5.76403, min distance = 2.06122
NCD_DROME Fu	91532 AMF	KIF1A_MOUSE F	6022 Aden	1I5S	1N6M	1I6I		0.99	Sc=5.74665, min distance = 2.48312
NCF2_HUMAN F	51 2-Oxop	SERA_ECOLI Fu	311 citri	2P9E	1HH8	1YBA		0.76	Sc=5.65027, min distance = 1.49109
NCF2_HUMAN F	6102712 C	PYRB_ECOLI Fu	311 citri	1R0B	1HH8	2AIR			Sc=5.65228, min distance = 0.22131
NCF2_HUMAN F	93072 Ure	PYRB_ECOLI Fu	311 citri	1R0B	1HH8	1R0C			Sc=5.82413, min distance = 1.24311
NCPR_HUMAN F	444658 2a	FLAV_DESVH Fu	444429 FM	3FX2	1B1C	1BU5		0.97	Sc=6.6001, min distance = 2.183054
NCPR_HUMAN F	449551 FM	FLAV_DESVH Fu	444429 FM	3FX2	1B1C	5FX2		0.77	Sc=6.6838, min distance = 2.102564
NCPR_RAT Ful	16058622	NOS1_RAT Full	444243 FA	1TLL	1JA1	2HX4	33.86		Sc=6.30634, min distance = 1.42317
NCPR_RAT Ful	444658 2a	FLAV_DESVH Fu	444243 FA	1F4P	1JA1	1BU5	27.27	0.97	Sc=6.56359, min distance = 2.38523
NCPR_RAT Ful	449551 FM	FLAV_DESVH Fu	444243 FA	1F4P	1JA1	5FX2	27.27	0.77	Sc=6.63759, min distance = 1.71878
NCPR_YEAST F	446995 FM	CYB2_YEAST Fu	444243 FA	1KBI	2BPO	1LTD		0.77	Sc=6.24103, min distance = 1.43361
NCPR_YEAST F	449551 FM	FLAV_DESVH Fu	444243 FA	1F4P	2BPO	5FX2		0.77	Sc=6.68119, min distance = 2.22674
NDK2_ARATH F	4290313 E	TPIS_TRYBB Fu	23831 HEF	2V0T	1S57	2VEK			Sc=5.63307, min distance = 2.04303
NDK2_ARATH F	64968 dTT	DPO42_SULSO F	65103 dGT	2JEJ	1S59	1S0O			Sc=5.86312, min distance = 2.36408
NDK3_HUMAN F	11957393	FAK1_HUMAN Fu	6022 Aden	1MP8	1ZS6	2ETM			Sc=6.14594, min distance = 1.87314
NDK3_HUMAN F	188966 dA	PAPS1_HUMAN F	6022 Aden	1X6V	1ZS6	2PEZ		0.98	Sc=6.31781, min distance = 1.87396

NDK3_HUMAN F	23727981	CDK2_HUMAN Fu	6022 Aden	1GY3	1ZS6	3BHT		Sc=5.92198, min distance = 3.18345
NDK3_HUMAN F	23727982	CDK2_HUMAN Fu	6022 Aden	1GY3	1ZS6	3BHU		Sc=6.0016, min distance = 2.751835
NDK3_HUMAN F	3540 1yds	IPKA_RAT Full	6022 Aden	1L3R	1ZS6	1YDS		Sc=5.87874, min distance = 2.08388
NDK3_HUMAN F	3543 1g5s	CDK2_HUMAN Fu	6022 Aden	1GY3	1ZS6	1G5S		Sc=6.53208, min distance = 2.36959
NDK3_HUMAN F	4369136 C	CDK2_HUMAN Fu	6022 Aden	1GY3	1ZS6	1DM2		Sc=6.16474, min distance = 1.58767
NDK3_HUMAN F	447649 1c	CDK2_HUMAN Fu	6022 Aden	1GY3	1ZS6	1OI9		Sc=6.39222, min distance = 1.19484
NDK3_HUMAN F	447655 1c	CDK2_HUMAN Fu	6022 Aden	1GY3	1ZS6	1OIU		Sc=6.33655, min distance = 1.41127
NDK3_HUMAN F	447916 ac	RIO1_ARCFU Fu	6022 Aden	1ZTH	1ZS6	1ZTF	0.95	Sc=6.16128, min distance = 2.22989
NDK3_HUMAN F	447955 1p	CDK2_HUMAN Fu	6022 Aden	1GY3	1ZS6	1PXI		Sc=5.66824, min distance = 1.65556
NDK3_HUMAN F	447962 1p	CDK2_HUMAN Fu	6022 Aden	1GY3	1ZS6	2C5N		Sc=6.15711, min distance = 1.40483
NDK3_HUMAN F	4565 1h1r	CDK2_HUMAN Fu	6022 Aden	1GY3	1ZS6	1H1R		Sc=6.26922, min distance = 1.98523
NDK3_HUMAN F	5327096 2	CDK2_HUMAN Fu	6022 Aden	1GY3	1ZS6	2BTR		Sc=6.05676, min distance = 1.68597
NDK3_HUMAN F	5327124	CDK2_HUMAN Fu	6022 Aden	1GY3	1ZS6	2C4G		Sc=6.18225, min distance = 0.98939
NDK3_HUMAN F	5327148 C	IPKA_RABIT Fu	6022 Aden	1JBP	1ZS6	2ERZ		Sc=6.16581, min distance = 2.21468
NDK3_HUMAN F	5957 Aden	PUR7_METJA Fu	6022 Aden	2YZL	1ZS6	2Z02	0.99	Sc=5.97322, min distance = 2.35619
NDK3_HUMAN F	5957 Aden	PURK_ECOLI Fu	6022 Aden	3ETJ	1ZS6	3ETH	0.99	Sc=5.68127, min distance = 2.02614
NDK3_HUMAN F	5957 Aden	PURL_THEMA Fu	6022 Aden	2HRU	1ZS6	2HS0	0.99	Sc=6.03169, min distance = 2.44975
NDK3_HUMAN F	5957 Aden	Y059_METJA Fu	6022 Aden	2J9D	1ZS6	2J9C	0.99	Sc=6.40273, min distance = 1.76465
NDK3_HUMAN F	6083 aden	PSPF_ECOLI Fu	6022 Aden	2C98	1ZS6	2VII	0.99	Sc=6.23146, min distance = 2.46910
NDK3_HUMAN F	8977 1dar	NDK_PYRHO Full	6022 Aden	2DYA	1ZS6	2DXE	0.8	Sc=5.6228, min distance = 2.509567
NDK3_HUMAN F	91532 AMP	PURP_METJA Fu	6022 Aden	2R7N	1ZS6	2R7K	0.99	Sc=5.70504, min distance = 2.01983
NDK3_HUMAN F	9549303 2	STK6_HUMAN Fu	6022 Aden	1MQ4	1ZS6	2NP8		Sc=6.39838, min distance = 2.55096
NDKA1_BOVIN	37792 gam	RAC1_HUMAN Fu	8977 1dar	1RYF	1BHN	2FJU	0.99	Sc=5.90148, min distance = 1.67534
NDKA1_BOVIN	37792 gam	RHOA_HUMAN Fu	8977 1dar	1TX4	1BHN	1CXZ	0.99	Sc=5.6106, min distance = 1.842966
NDKA_HUMAN F	11957393	FAK1_HUMAN Fu	6022 Aden	1MP8	1UCN	2ETM		Sc=6.13755, min distance = 1.85272
NDKA_HUMAN F	1540 1h1q	CDK2_HUMAN Fu	6022 Aden	1GY3	1UCN	1H1Q		Sc=6.04758, min distance = 2.16520
NDKA_HUMAN F	16214825	CDK2_HUMAN Fu	6022 Aden	1GY3	1UCN	2UZE		Sc=6.2671, min distance = 1.895837
NDKA_HUMAN F	23653515	CDK2_HUMAN Fu	6022 Aden	1GY3	1UCN	2R3F		Sc=6.09181, min distance = 1.46971
NDKA_HUMAN F	23653518	CDK2_HUMAN Fu	6022 Aden	1GY3	1UCN	2R3J		Sc=6.29859, min distance = 1.69572
NDKA_HUMAN F	23653519	CDK2_HUMAN Fu	6022 Aden	1GY3	1UCN	2R3K		Sc=6.1891, min distance = 1.933826
NDKA_HUMAN F	23727981	CDK2_HUMAN Fu	6022 Aden	1GY3	1UCN	3BHT		Sc=5.77033, min distance = 2.92615
NDKA_HUMAN F	24864077	CDK2_HUMAN Fu	6022 Aden	1GY3	1UCN	2VTN		Sc=5.98945, min distance = 1.83457
NDKA_HUMAN F	24864078	CDK2_HUMAN Fu	6022 Aden	1GY3	1UCN	2VTO		Sc=6.13714, min distance = 1.86662
NDKA_HUMAN F	24864080	CDK2_HUMAN Fu	6022 Aden	1GY3	1UCN	2VTR		Sc=5.95881, min distance = 1.44925
NDKA_HUMAN F	3543 1g5s	CDK2_HUMAN Fu	6022 Aden	1GY3	1UCN	1G5S		Sc=6.45923, min distance = 2.23941
NDKA_HUMAN F	447649 1c	CDK2_HUMAN Fu	6022 Aden	1GY3	1UCN	1OI9		Sc=6.18335, min distance = 2.00116
NDKA_HUMAN F	447652 Im	CDK2_HUMAN Fu	6022 Aden	1GY3	1UCN	1OIQ		Sc=6.01515, min distance = 2.03935

NDKA_HUMAN	F	447916 ad	RIO1_ARCFU	Fu	6022 Aden	1ZTH	1UCN	1ZTF	0.95	Sc=6.04242, min distance = 1.970322
NDKA_HUMAN	F	447955 1p	CDK2_HUMAN	Fu	6022 Aden	1GY3	1UCN	1PXI		Sc=5.65739, min distance = 1.967011
NDKA_HUMAN	F	447962 1p	CDK2_HUMAN	Fu	6022 Aden	1GY3	1UCN	2C5N		Sc=6.14399, min distance = 2.605196
NDKA_HUMAN	F	449088 1v	CDK2_HUMAN	Fu	6022 Aden	1GY3	1UCN	1VYZ		Sc=5.98708, min distance = 1.585490
NDKA_HUMAN	F	4565 1h1r	CDK2_HUMAN	Fu	6022 Aden	1GY3	1UCN	1H1R		Sc=6.04758, min distance = 2.794768
NDKA_HUMAN	F	5327096 2	CDK2_HUMAN	Fu	6022 Aden	1GY3	1UCN	2BTR		Sc=6.03085, min distance = 1.874934
NDKA_HUMAN	F	5327130 C	CDK2_HUMAN	Fu	6022 Aden	1GY3	1UCN	2C68		Sc=6.28549, min distance = 1.365908
NDKA_HUMAN	F	6420139 C	CDK2_HUMAN	Fu	6022 Aden	1GY3	1UCN	2C5V		Sc=6.04299, min distance = 1.615427
NDKA_HUMAN	F	65051 ddT	NDKC_DICDI	Fu	6022 Aden	1KDN	1UCN	1F3F		Sc=5.66668, min distance = 1.261482
NDKA_HUMAN	F	6918710 5	CDK2_HUMAN	Fu	6022 Aden	1GY3	1UCN	3EJ1		Sc=5.94142, min distance = 2.260156
NDKA_HUMAN	F	9547890 I	CDK2_HUMAN	Fu	6022 Aden	1GY3	1UCN	1W8C		Sc=6.10149, min distance = 1.617726
NDKA_HUMAN	F	9549303 2	STK6_HUMAN	Fu	6022 Aden	1MQ4	1UCN	2NP8		Sc=6.18484, min distance = 2.865115
NDKA_HUMAN	F	9991833 S	CDK2_HUMAN	Fu	6022 Aden	1GY3	1UCN	2R3H		Sc=6.13011, min distance = 1.885800
NDKC_DICDI	F	11957393	FAK1_HUMAN	Fu	6022 Aden	1MP8	1KDN	2ETM		Sc=6.10612, min distance = 1.974583
NDKC_DICDI	F	11957417	CDK2_HUMAN	Fu	6022 Aden	1GY3	1KDN	2I40		Sc=5.97897, min distance = 2.132939
NDKC_DICDI	F	23653518	CDK2_HUMAN	Fu	6022 Aden	1GY3	1KDN	2R3J		Sc=6.27029, min distance = 1.442268
NDKC_DICDI	F	23653519	CDK2_HUMAN	Fu	6022 Aden	1GY3	1KDN	2R3K		Sc=6.16667, min distance = 1.742787
NDKC_DICDI	F	23653520	CDK2_HUMAN	Fu	6022 Aden	1GY3	1KDN	2R3L		Sc=6.28165, min distance = 1.159833
NDKC_DICDI	F	23727981	CDK2_HUMAN	Fu	6022 Aden	1GY3	1KDN	3BHT		Sc=5.87457, min distance = 3.160443
NDKC_DICDI	F	23727982	CDK2_HUMAN	Fu	6022 Aden	1GY3	1KDN	3BHU		Sc=5.94671, min distance = 3.095645
NDKC_DICDI	F	24864081	CDK2_HUMAN	Fu	6022 Aden	1GY3	1KDN	2VTS		Sc=6.28597, min distance = 2.075175
NDKC_DICDI	F	25021197	CDK2_HUMAN	Fu	6022 Aden	1GY3	1KDN	3EOC		Sc=6.24917, min distance = 2.140043
NDKC_DICDI	F	3543 1g5s	CDK2_HUMAN	Fu	6022 Aden	1GY3	1KDN	1G5S		Sc=6.35669, min distance = 2.405076
NDKC_DICDI	F	447649 1c	CDK2_HUMAN	Fu	6022 Aden	1GY3	1KDN	1OI9		Sc=6.31667, min distance = 1.943432
NDKC_DICDI	F	447652 Im	CDK2_HUMAN	Fu	6022 Aden	1GY3	1KDN	1OIQ		Sc=5.98225, min distance = 2.135822
NDKC_DICDI	F	447655 1c	CDK2_HUMAN	Fu	6022 Aden	1GY3	1KDN	1OIU		Sc=6.27438, min distance = 2.010375
NDKC_DICDI	F	447656 1c	CDK2_HUMAN	Fu	6022 Aden	1GY3	1KDN	1OIY		Sc=6.38945, min distance = 1.379578
NDKC_DICDI	F	447916 ac	RIO1_ARCFU	Fu	6022 Aden	1ZTH	1KDN	1ZTF	0.95	Sc=6.06749, min distance = 2.251845
NDKC_DICDI	F	447962 1p	CDK2_HUMAN	Fu	6022 Aden	1GY3	1KDN	2C5N		Sc=6.10512, min distance = 2.312980
NDKC_DICDI	F	449088 1v	CDK2_HUMAN	Fu	6022 Aden	1GY3	1KDN	1VYZ		Sc=5.81118, min distance = 1.611491
NDKC_DICDI	F	4565 1h1r	CDK2_HUMAN	Fu	6022 Aden	1GY3	1KDN	1H1R		Sc=6.22886, min distance = 2.251100
NDKC_DICDI	F	5327096 2	CDK2_HUMAN	Fu	6022 Aden	1GY3	1KDN	2BTR		Sc=5.98873, min distance = 1.674490
NDKC_DICDI	F	5327130 C	CDK2_HUMAN	Fu	6022 Aden	1GY3	1KDN	2C68		Sc=6.16763, min distance = 2.083162
NDKC_DICDI	F	5327131 T	CDK2_HUMAN	Fu	6022 Aden	1GY3	1KDN	2C69		Sc=6.24785, min distance = 2.130635
NDKC_DICDI	F	5957 Aden	MUTS_ECOLI	Fu	6022 Aden	1OH7	1KDN	1W7A	0.99	Sc=6.38956, min distance = 1.861151
NDKC_DICDI	F	5957 Aden	PURP_METJA	Fu	6022 Aden	2R7N	1KDN	2R7L	0.99	Sc=5.95772, min distance = 1.077620
NDKC_DICDI	F	5957 Aden	Y059_METJA	Fu	6022 Aden	2J9D	1KDN	2J9C	0.99	Sc=6.38484, min distance = 1.757293

NDKC_DICDI Fu	6083 aden	NDK_PYRHO Ful	6022 Aden	2DYA	1KDN	2DXD	56.83	0.99	Sc=6.14017, min distance = 2.85131
NDKC_DICDI Fu	6083 aden	PURP_METJA Fu	6022 Aden	2R7N	1KDN	2R7M		0.99	Sc=6.21358, min distance = 2.64060
NDKC_DICDI Fu	65304 ddA	DPO1_THEAQ Fu	65051 ddT	1QTM	1F3F	1QSY			Sc=6.34806, min distance = 0
NDKC_DICDI Fu	6804 guar	NDK_PYRHO Ful	6022 Aden	2DYA	1KDN	2DXF	56.83	0.8	Sc=6.26137, min distance = 2.28719
NDKC_DICDI Fu	8977 1dar	NDK_PYRHO Ful	6022 Aden	2DYA	1KDN	2DXE	56.83	0.8	Sc=6.31919, min distance = 2.98763
NDKC_DICDI Fu	8977 1dar	NDK_THET8 Ful	6022 Aden	1WKL	1KDN	1WKK		0.8	Sc=5.92286, min distance = 1.76035
NDKC_DICDI Fu	91532 AME	PURP_METJA Fu	6022 Aden	2R7N	1KDN	2R7K		0.99	Sc=5.68674, min distance = 2.49900
NDKC_DICDI Fu	91532 AME	RBSK_ECOLI Fu	6022 Aden	1RKD	1KDN	1GQT		0.99	Sc=5.96591, min distance = 1.80467
NDKC_DICDI Fu	9547890 I	CDK2_HUMAN Fu	6022 Aden	1GY3	1KDN	1W8C			Sc=6.08249, min distance = 1.55566
NDKC_DICDI Fu	9991833 S	CDK2_HUMAN Fu	6022 Aden	1GY3	1KDN	2R3H			Sc=6.07738, min distance = 1.81971
NDK_HALSA Fu	446696 do	KCY_ECOLI Ful	6132 Cyti	2CMK	2AZ3	1KDT		0.88	Sc=5.99204, min distance = 2.91405
NDK_HALSA Fu	6031 Uric	ECX1_PYRAB Fu	6132 Cyti	2PO2	2AZ3	2PNZ		0.83	Sc=5.77733, min distance = 2.48477
NDK_MIMIV Fu	122108 Ri	NDKC_DICDI Fu	164628 de	1NDC	2B8Q	1MN9			Sc=5.89312, min distance = 2.06020
NDK_MIMIV Fu	15993 dAT	PRIM_BPT7 Ful	164628 de	1CR4	2B8Q	1CR2			Sc=5.9447, min distance = 2.057439
NDK_MIMIV Fu	5789 thym	KTHY_MYCTU Fu	164628 de	1GTV	2B8Q	1W2G		0.9	Sc=6.12413, min distance = 2.52175
NDK_MIMIV Fu	6031 Uric	SPSA_BACSU Fu	164628 de	1H7L	2B8Q	1QGQ		0.87	Sc=6.2878, min distance = 1.757625
NDK_MIMIV Fu	65051 ddT	NDKC_DICDI Fu	164628 de	1NDC	2B8Q	1F3F		0.94	Sc=5.96375, min distance = 2.35153
NDK_MIMIV Fu	9543433 T	NDKC_DICDI Fu	164628 de	1NDC	2B8Q	1F6T		0.97	Sc=6.34029, min distance = 2.43582
NDK_MYXXA Fu	6083 aden	PDE10_HUMAN Fu	6076 Cycl	2OUR	1NHK	2OUN		0.99	Sc=6.17217, min distance = 2.46503
NDK_MYXXA Fu	6083 aden	PDE4D_HUMAN Fu	6076 Cycl	2PW3	1NHK	1TB7		0.99	Sc=6.18823, min distance = 2.08922
NDK_MYXXA Fu	6804 guar	PDE10_HUMAN Fu	6076 Cycl	2OUR	1NHK	2OUQ		0.79	Sc=5.82593, min distance = 0
NDK_MYXXA Fu	6858240 C	KAP0_BOVIN Fu	6076 Cycl	1RGS	1NHK	1NE6		0.98	Sc=5.85265, min distance = 2.29927
NDK_PYRHO Fu	10109823	PIM1_HUMAN Fu	6083 aden	1YXU	2DXD	3CY3			Sc=6.06165, min distance = 2.68121
NDK_PYRHO Fu	122108 Ri	NDKC_DICDI Fu	6022 Aden	1KDN	2DYA	1MN9	56.83		Sc=5.88732, min distance = 1.67426
NDK_PYRHO Fu	16131862	PAP2_VACCW Fu	8977 1dar	1JTF	2DXE	1P39		0.97	Sc=6.00918, min distance = 2.02269
NDK_PYRHO Fu	23727981	CDK2_HUMAN Fu	6022 Aden	1GY3	2DYA	3BHT			Sc=5.7897, min distance = 3.031418
NDK_PYRHO Fu	23727982	CDK2_HUMAN Fu	6022 Aden	1GY3	2DYA	3BHU			Sc=5.87469, min distance = 2.69541
NDK_PYRHO Fu	2519 caff	PYGM_RABIT Fu	6083 aden	8GPB	2DXD	1GFZ			Sc=5.64347, min distance = 2.70577
NDK_PYRHO Fu	3758 IBMX	PDE4D_HUMAN Fu	6083 aden	1TB7	2DXD	1ZKN			Sc=5.96332, min distance = 1.71920
NDK_PYRHO Fu	3973 nche	PIM1_HUMAN Fu	6083 aden	1YXU	2DXD	1YI3			Sc=5.84929, min distance = 1.51504
NDK_PYRHO Fu	444345 1c	PIM1_HUMAN Fu	6083 aden	1YXU	2DXD	1YHS			Sc=6.28079, min distance = 2.20650
NDK_PYRHO Fu	444564 AD	BIOD_ECOLI Fu	6022 Aden	1DAD	2DYA	1BS1		0.91	Sc=6.36432, min distance = 2.07013
NDK_PYRHO Fu	445966 O6	CDK2_HUMAN Fu	6022 Aden	1GY3	2DYA	1H0V			Sc=6.19578, min distance = 1.81027
NDK_PYRHO Fu	446090 1h	NDKC_DICDI Fu	6022 Aden	1KDN	2DYA	1HIY	56.83	0.97	Sc=6.3529, min distance = 2.559370
NDK_PYRHO Fu	447832 AD	ACSA_SALTY Fu	6083 aden	2P2F	2DXD	1PG4		0.99	Sc=5.98992, min distance = 2.87514
NDK_PYRHO Fu	447979 5G	KGUA_ECOLI Fu	8977 1dar	2AN9	2DXE	2ANB			Sc=5.88715, min distance = 2.12289
NDK_PYRHO Fu	448466 CI	HINT1_RABIT Fu	6804 guan	3RHN	2DXF	1RZY			Sc=6.2419, min distance = 2.525476

NDK_PYRHO Fu	448505 AD	NDKC_DICDI Fu	6022 Aden	1KDN	2DYA	1S5Z	56.83	0.93	Sc=6.41317, min distance = 2.08737
NDK_PYRHO Fu	4565 1h1r	CDK2_HUMAN Fu	6022 Aden	1GY3	2DYA	1H1R			Sc=6.10677, min distance = 0.67583
NDK_PYRHO Fu	5957 Aden	PSPF_ECOLI Fu	6022 Aden	2C98	2DYA	2C96		0.99	Sc=5.99134, min distance = 2.14580
NDK_PYRHO Fu	5957 Aden	Y059_METJA Fu	6022 Aden	2J9D	2DYA	2J9C		0.99	Sc=6.42759, min distance = 1.84770
NDK_PYRHO Fu	6076 Cycl	PDE10_HUMAN F	6083 aden	2OUN	2DXD	2OUR		0.99	Sc=6.15533, min distance = 2.29264
NDK_PYRHO Fu	6076 Cycl	PDE10_HUMAN F	6804 guan	2OUQ	2DXF	2OUR		0.79	Sc=6.10459, min distance = 0
NDK_PYRHO Fu	6102784 A	PSPF_ECOLI Fu	6022 Aden	2C98	2DYA	2C99		0.99	Sc=6.46037, min distance = 2.03965
NDK_PYRHO Fu	6419789 C	HASP_HUMAN Fu	6083 aden	3DLZ	2DXD	3E7V			Sc=5.94096, min distance = 2.47803
NDK_PYRHO Fu	65051 ddT	NDKC_DICDI Fu	6022 Aden	1KDN	2DYA	1F3F	56.83		Sc=5.9858, min distance = 1.679054
NDK_PYRHO Fu	656964 1y	PDE4D_HUMAN F	6083 aden	1TB7	2DXD	1Y2C			Sc=5.83279, min distance = 2.61196
NDK_PYRHO Fu	656966 1y	PDE4D_HUMAN F	6083 aden	1TB7	2DXD	1Y2E			Sc=5.98749, min distance = 2.61407
NDK_PYRHO Fu	9543433 T	NDKC_DICDI Fu	6022 Aden	1KDN	2DYA	1F6T	56.83		Sc=6.36323, min distance = 1.90261
NDK_THET8 Fu	11608401	KAPCA_BOVIN F	5957 Aden	1Q24	1WKL	2UVY			Sc=5.91467, min distance = 2.36580
NDK_THET8 Fu	11708454	KAPCA_BOVIN F	5957 Aden	1Q24	1WKL	2VNW			Sc=5.82755, min distance = 2.60043
NDK_THET8 Fu	16122632	KAPCA_BOVIN F	5957 Aden	1Q24	1WKL	2UVZ			Sc=6.04487, min distance = 2.58065
NDK_THET8 Fu	17751819	KAPCA_BOVIN F	5957 Aden	1Q24	1WKL	2VO6			Sc=6.03298, min distance = 2.12926
NDK_THET8 Fu	17754396	PDPK1_HUMAN F	5957 Aden	2BIY	1WKL	2R7B			Sc=6.04336, min distance = 2.22610
NDK_THET8 Fu	24978488	TBB2B_BOVIN F	8977 1dar	1Z2B	1WKK	3DU7			Sc=5.83958, min distance = 2.20897
NDK_THET8 Fu	37792 gam	RAB6B_HUMAN F	8977 1dar	2E9S	1WKK	2FFQ		0.99	Sc=5.91339, min distance = 2.02191
NDK_THET8 Fu	446090 1h	NDKC_DICDI Fu	6022 Aden	1KDN	1WKL	1HIY		0.97	Sc=6.3137, min distance = 2.167152
NDK_THET8 Fu	446248 CI	RAN_HUMAN Ful	8977 1dar	3CH5	1WKK	1IBR			Sc=5.95237, min distance = 2.06902
NDK_THET8 Fu	446248 CI	RASH_HUMAN Fu	8977 1dar	2CE2	1WKK	1IAQ			Sc=5.9432, min distance = 2.294922
NDK_THET8 Fu	447916 ad	RIO1_ARCFU Fu	5957 Aden	1ZP9	1WKL	1ZTF		0.94	Sc=5.98467, min distance = 2.39168
NDK_THET8 Fu	448505 AD	NDKC_DICDI Fu	6022 Aden	1KDN	1WKL	1S5Z		0.93	Sc=5.91531, min distance = 2.01578
NDK_THET8 Fu	6176 Cyti	PYRI_ECOLI Fu	5957 Aden	7AT1	1WKL	2FZC			Sc=5.84505, min distance = 2.35318
NDK_THET8 Fu	6914614 p	KAPCA_BOVIN F	5957 Aden	1Q24	1WKL	2F7Z			Sc=6.35096, min distance = 2.20900
NDK_THET8 Fu	6918710 5	CDK2_HUMAN Fu	6022 Aden	1GY3	1WKL	3EJ1			Sc=5.97472, min distance = 2.12599
NDRG2_MOUSE	23624247	WRBA_ECOLI Fu	62551 Pen	3B6K	2QMQ	3B6I			Sc=5.64926, min distance = 2.29894
NEPU1_THEVU	15600 DEC	BACR_HALSA Fu	79025 alp	1DZE	1UH4	1XJI			Sc=5.64868, min distance = 0.77427
NEPU1_THEVU	193758 Va	AMY1_HUMAN Fu	79025 alp	3DHP	1UH4	1MFU			Sc=5.9662, min distance = 0.714258
NEPU1_THEVU	193758 Va	CDGT2_BACCI F	79025 alp	1D3C	1UH4	1DTU			Sc=5.96232, min distance = 1.03687
NEPU1_THEVU	444140 CI	CDGT_THETU Fu	79025 alp	3BMW	1UH4	1A47			Sc=5.76468, min distance = 0.39824
NEPU1_THEVU	446564 LI	BACR_HALSA Fu	79025 alp	1DZE	1UH4	1JV7			Sc=5.69423, min distance = 1.13191
NEPU1_THEVU	448009 TR	AMY1_HUMAN Fu	79025 alp	3DHP	1UH4	1Q4N			Sc=5.60282, min distance = 2.32024
NEPU1_THEVU	448819 ty	TSPE_BPP22 Fu	79025 alp	1TYW	1UH4	1TYU		0.98	Sc=5.68041, min distance = 1.43251
NEPU1_THEVU	449571 CI	CDGT1_BACCI F	79025 alp	1CGU	1UH4	6CGT			Sc=5.7665, min distance = 1.201457
NEPU2_THEVU	4369386 A	AMY1_HUMAN Fu	79025 alp	3DHP	2D2O	1Z32			Sc=5.69347, min distance = 0.83106

NEPU2_THEVU	444139 CI	AMYP_PIG Full	79025 alp	1HX0	2D2O	1PIG		Sc=5.71012, min distance = 0.49467
NEPU2_THEVU	446000 CI	AMYP_PIG Full	79025 alp	1HX0	2D2O	1JFH	0.83	Sc=5.75277, min distance = 2.25447
NEPU2_THEVU	95687 Dec	PHSM_ECOLI Fu	79025 alp	1L5W	2D2O	1QM5		Sc=6.09361, min distance = 1.00523
NEPU_BACST F	13455857	IGKC_MOUSE Fu	79025 alp	3BZ4	1J0I	3C6S	0.83	Sc=5.74407, min distance = 1.54661
NEPU_BACST F	4369386 A	AMY1_HUMAN Fu	79025 alp	3DHP	1J0I	1Z32		Sc=5.73152, min distance = 0.67313
NEPU_BACST F	439692 D-	XYLA_STRRU Fu	79025 alp	1XIF	1J0I	4XIS		Sc=5.62032, min distance = 2.30760
NEPU_BACST F	447607 1d	PYGM_RABIT Fu	79025 alp	1H5U	1J0I	2G9V		Sc=5.76222, min distance = 2.37310
NEPU_BACST F	6027 xylo	XYLA_STRRU Fu	79025 alp	1XIF	1J0I	3XIS	0.93	Sc=5.65294, min distance = 2.67968
NEUR2_HUMAN	164795 N(	RBCMT_PEA Full	23831 HEP	1MLV	2F25	2H2J		Sc=5.6862, min distance = 1.561367
NFNB_ECOLI F	8778 Laur	CTXA3_NAJAT F	311 citri	1XT3	1YKI	1H0J		Sc=5.72741, min distance = 0.59818
NFSA_ECOLI F	5326566 l	Q9WZW1_THEMA	444243 FA	1T6Y	1F5V	1S4M	0.82	Sc=6.2183, min distance = 1.663861
NGB_HUMAN Fu	11957385	MYG_PHYCA Full	444098 HE	1A6M	1OJ6	2CMM		Sc=6.21878, min distance = 2.11447
NGB_MOUSE Fu	11957385	MYG_PHYCA Full	444522 HE	1VXD	1W92	2CMM		Sc=5.90078, min distance = 2.09889
NGB_MOUSE Fu	16741062	MYG_PHYCA Full	444522 HE	1VXD	1W92	1MBN	0.88	Sc=6.58727, min distance = 2.16422
NGB_MOUSE Fu	16741183	MYG_PHYCA Full	444522 HE	1VXD	1W92	2EKT	0.93	Sc=6.37677, min distance = 2.07514
NGB_MOUSE Fu	444207 He	MYG_PHYCA Full	444522 HE	1VXD	1W92	1VXG	1	Sc=6.58727, min distance = 2.21380
NGB_MOUSE Fu	6102730 C	PGH1_SHEEP Fu	444522 HE	1CQE	1W92	2AYL	0.98	Sc=6.59022, min distance = 2.15983
NGLY1_MOUSE	25011741	IPSP_HUMAN Fu	185698 al	1LQ8	2I74	3DY0		Sc=5.72381, min distance = 0.77399
NGLY1_MOUSE	444211 th	ACES_TORCA Fu	185698 al	1U65	2I74	1ACJ		Sc=5.75308, min distance = 1.99031
NIA1_MAIZE F	23727982	CDK2_HUMAN Fu	6022 Aden	1GY3	1CNF	3BHU		Sc=5.82235, min distance = 2.41400
NIA1_MAIZE F	24832021	HS90A_HUMAN F	6022 Aden	1BYQ	1CNF	2QG0		Sc=6.28635, min distance = 2.11400
NIA1_MAIZE F	24864080	CDK2_HUMAN Fu	6022 Aden	1GY3	1CNF	2VTR		Sc=5.68376, min distance = 2.22423
NIA1_MAIZE F	440317 AT	BCKD_RAT Full	6022 Aden	1GKZ	1CNF	1GJV	0.99	Sc=5.8785, min distance = 2.622463
NIA1_MAIZE F	447652 Im	CDK2_HUMAN Fu	6022 Aden	1GY3	1CNF	1OIQ		Sc=5.89031, min distance = 2.57174
NIA1_MAIZE F	4565 1h1r	CDK2_HUMAN Fu	6022 Aden	1GY3	1CNF	1H1R		Sc=6.28165, min distance = 1.51685
NIA1_MAIZE F	6804 guar	NDK_PYRHO Full	6022 Aden	2DYA	1CNF	2DXF	0.8	Sc=5.82196, min distance = 2.16012
NIA1_MAIZE F	6918710 5	CDK2_HUMAN Fu	6022 Aden	1GY3	1CNF	3EJ1		Sc=5.9395, min distance = 2.676520
NIA1_MAIZE F	8977 1dar	NDK_PYRHO Full	6022 Aden	2DYA	1CNF	2DXE	0.8	Sc=5.87734, min distance = 2.24381
NIFD_KLEPN F	12793 Phe	SRC_HUMAN Full	311 citri	1O4L	1H1L	1O4O		Sc=5.86728, min distance = 0.86158
NIFD_KLEPN F	6961 PYRO	FUMC_ECOLI Fu	311 citri	1FUO	1H1L	1FUP		Sc=6.14104, min distance = 2.46102
NIFH1_AZOVI	160355 ro	PDXK_SHEEP Fu	91532 AMP	1RFT	2AFK	1YGK		Sc=6.41572, min distance = 1.65888
NIFH1_AZOVI	1720 4-Am	PARP1_CHICK F	6022 Aden	1A26	1FP6	2PAX		Sc=6.05632, min distance = 1.32957
NIFH1_AZOVI	33113 gam	KIF1A_MOUSE F	91532 AMP	1I6I	2AFK	1VFV	0.99	Sc=6.45945, min distance = 2.41835
NIFH1_AZOVI	3547 Fasu	KAPCA_BOVIN F	5957 Aden	1Q24	2C8V	1Q8W		Sc=5.90171, min distance = 2.16349
NIFH1_AZOVI	444852 CI	ATPA1_BOVIN F	5957 Aden	2V7Q	2C8V	1COW	0.91	Sc=6.48884, min distance = 2.23239
NIFH1_AZOVI	444852 CI	ATPB_BOVIN Fu	5957 Aden	2V7Q	2C8V	1COW	0.91	Sc=6.49123, min distance = 2.29817
NIFH1_AZOVI	444852 CI	ATPB_BOVIN Fu	6022 Aden	2CK3	1FP6	1COW	0.9	Sc=5.96341, min distance = 2.29954

NIFH1_AZOVI	445736 5	SYS2_METBF Fu	5957 Aden	2CJA	2C8V	2CJ9		0.87	Sc=6.13409, min distance = 1.21598
NIFH1_AZOVI	447916 ac	RIO1_ARCFU Fu	5957 Aden	1ZP9	2C8V	1ZTF		0.94	Sc=5.80367, min distance = 2.86095
NIFH1_AZOVI	448042 2e	KAPCA_BOVIN F	5957 Aden	1Q24	2C8V	1Q8T			Sc=6.07421, min distance = 2.58558
NIFH1_AZOVI	6083 ader	PSPF_ECOLI Fu	5957 Aden	2C96	2C8V	2VII		0.98	Sc=5.96483, min distance = 2.01784
NIFH1_AZOVI	60961 ade	IPKA_RABIT Fu	6022 Aden	1JBP	1FP6	1FMO		0.95	Sc=5.79175, min distance = 2.93875
NIFH1_AZOVI	6102784 A	PSPF_ECOLI Fu	5957 Aden	2C96	2C8V	2C99		0.99	Sc=6.10309, min distance = 2.21019
NIFH1_AZOVI	6132 Cyti	ECX1_PYRAB Fu	6022 Aden	2PO0	1FP6	2PO2			Sc=5.76632, min distance = 2.43919
NIFH1_AZOVI	6132 Cyti	ECX2_PYRAB Fu	6022 Aden	2PO0	1FP6	2PO2			Sc=5.92379, min distance = 2.42538
NIFH1_AZOVI	6338561 C	MYS2_DICDI Fu	5957 Aden	1FMW	2C8V	1D0X			Sc=5.79124, min distance = 0
NIFH1_AZOVI	6852207 M	KAPCA_BOVIN F	5957 Aden	1Q24	2C8V	2GNI			Sc=5.90171, min distance = 2.35438
NIFK_AZOVI F	23624247	WRBA_ECOLI Fu	62551 Pen	3B6K	2AFH	3B6I			Sc=5.69537, min distance = 2.41428
NIFK_KLEPN F	62551 Per	NIFK_AZOVI Fu	439459 Hc	1M1N	1QGU	2AFH	67.43		Sc=5.83178, min distance = 2.09618
NIKR_HELPY F	448573 FI	CASP7_HUMAN F	311 citri	2QL9	2CAD	1SHL			Sc=5.75589, min distance = 2.72615
NLGNX_HUMAN	2969 Deca	ALBU_HUMAN Fu	311 citri	1TF0	3BE8	2VDB			Sc=5.75169, min distance = 1.15365
NLGNX_HUMAN	448573 FI	CASP7_HUMAN F	311 citri	2QL9	3BE8	1SHL			Sc=5.89391, min distance = 2.02373
NLGNX_HUMAN	8778 Laur	CTXA3_NAJAT F	311 citri	1XT3	3BE8	1H0J			Sc=5.69225, min distance = 0.75609
NLTP1_ORYSJ	14253 Cet	FABP4_MOUSE F	985 palmi	1LIE	1UVB	1LIC			Sc=5.70387, min distance = 1.29476
NLTP1_ORYSJ	175468 12	LACB_BOVIN Fu	985 palmi	1GXA	1UVB	1BSO		0.83	Sc=5.79174, min distance = 2.60528
NLTP1_ORYSJ	445638 pa	NLTP_MAIZE Fu	5281 stea	1FK4	1UVC	1FK3	78.26	0.75	Sc=5.99764, min distance = 2.53500
NLTP1_ORYSJ	445638 pa	NLTP_MAIZE Fu	985 palmi	1MZM	1UVB	1FK3	78.26	0.75	Sc=5.90209, min distance = 2.81393
NLTP1_ORYSJ	445639 ol	ALBU_HUMAN Fu	11005 Tet	1N5U	1UVA	1GNI			Sc=6.03632, min distance = 1.28714
NLTP1_ORYSJ	445639 ol	NLTP_MAIZE Fu	11005 Tet	1FK2	1UVA	1FK5	78.26		Sc=5.9805, min distance = 2.183838
NLTP1_ORYSJ	445639 ol	NLTP_MAIZE Fu	985 palmi	1MZM	1UVB	1FK5	78.26	0.77	Sc=6.0352, min distance = 2.147327
NLTP1_ORYSJ	445641 Ri	NLTP_MAIZE Fu	5281 stea	1FK4	1UVC	1FK7	78.26		Sc=6.1891, min distance = 2.567388
NLTP1_ORYSJ	447234 Hc	EST1_HUMAN Fu	985 palmi	2DQY	1UVB	1MX5			Sc=6.04643, min distance = 1.01484
NLTP1_ORYSJ	447681 al	S14L2_HUMAN F	985 palmi	1O6U	1UVB	1OLM			Sc=6.39809, min distance = 1.20819
NLTP1_ORYSJ	5280934 l	NLTP_MAIZE Fu	5281 stea	1FK4	1UVC	1FK6	78.26	0.75	Sc=6.06816, min distance = 2.13399
NLTP1_ORYSJ	5496506 s	POLG_POL1M Fu	985 palmi	1HXS	1UVB	1AL2			Sc=6.09988, min distance = 2.34215
NLTP1_ORYSJ	62551 Per	Y1264_MYCTU F	5281 stea	2EV1	1UVC	1Y10			Sc=5.89162, min distance = 1.13800
NLTP1_ORYSJ	6603901 C	PPARD_HUMAN F	5281 stea	2AWH	1UVC	3D5F			Sc=6.43499, min distance = 1.17392
NLTP1_ORYSJ	8209 Myri	ABL1_MOUSE Fu	11005 Tet	1OPK	1UVA	1OPJ			Sc=5.66181, min distance = 2.54533
NLTP1_ORYSJ	91486 Sph	POLG_POL1M Fu	985 palmi	1HXS	1UVB	2PLV			Sc=6.08476, min distance = 2.25506
NLTP_MAIZE F	12530 n-T	ALBU_HUMAN Fu	445639 ol	1GNI	1FK5	1HK4			Sc=5.83075, min distance = 2.48610
NLTP_MAIZE F	175468 12	LACB_BOVIN Fu	985 palmi	1GXA	1MZM	1BSO		0.83	Sc=5.74322, min distance = 2.13298
NLTP_MAIZE F	5496506 s	POLG_POL1M Fu	985 palmi	1HXS	1MZM	1AL2			Sc=6.11904, min distance = 2.15111
NLTP_MAIZE F	8180 UNDE	ALBU_HUMAN Fu	445639 ol	1GNI	1FK5	1E7F			Sc=5.73718, min distance = 2.29937
NLTP_MAIZE F	8209 Myri	ABL1_MOUSE Fu	11005 Tet	1OPK	1FK2	1OPJ			Sc=5.68588, min distance = 2.09305

NMDE1_RAT Fu	10255 kai	GRIA2_RAT Ful	33032 L-g	2GFE	2A5S	1FTK			Sc=6.1875, min distance = 1.928401
NMDE1_RAT Fu	10255 kai	GRIK2_RAT Ful	33032 L-g	1S50	2A5S	1TT1			Sc=6.20073, min distance = 2.01507
NMDE1_RAT Fu	24820118	GGT_ECOLI Ful	33032 L-g	2DG5	2A5S	2Z8K			Sc=5.7104, min distance = 2.240558
NMDE1_RAT Fu	40539 Qui	GRIA2_RAT Ful	33032 L-g	2GFE	2A5S	1P1O			Sc=5.92573, min distance = 2.042458
NMDE1_RAT Fu	447195 1m	GRIA2_RAT Ful	33032 L-g	2GFE	2A5S	1MQD			Sc=5.95352, min distance = 2.091080
NMDE1_RAT Fu	449178 gl	GLMS_ECOLI Fu	33032 L-g	1XFF	2A5S	1XFG		0.81	Sc=5.87401, min distance = 2.24172
NMNA1_HUMAN I	6022 Ader	PNTAA_RHORU Fu	5893 nadi	2FSV	1KQN	2FR8			Sc=5.86442, min distance = 2.09837
NMNA3_HUMAN I	128225 be	NMNA1_HUMAN Fu	5893 nadi	1KQN	1NUU	1KR2			Sc=6.74264, min distance = 2.127528
NMNA3_HUMAN I	5957 Ader	PANC_MYCTU Fu	91557 1tm	1N2E	1NUT	2A84		0.99	Sc=6.43113, min distance = 2.478798
NMNA3_HUMAN I	6022 Ader	Q94M05_9VIRU I	91557 1tm	1W48	1NUT	1W44		0.99	Sc=6.40345, min distance = 2.037646
NMNA3_HUMAN I	6083 ader	BLS_STRCL Ful	91557 1tm	1JGT	1NUT	1MC1		0.97	Sc=6.36681, min distance = 1.312805
NMNA3_HUMAN I	6083 ader	NADE_BACAN Fu	91557 1tm	2PZ8	1NUT	2PZA		0.97	Sc=6.35961, min distance = 2.257528
NMNA3_HUMAN I	6083 ader	PANC_MYCTU Fu	91557 1tm	1N2E	1NUT	2A7X		0.97	Sc=6.32622, min distance = 2.416390
NMRL1_HUMAN I	6022 Ader	HMDH_HUMAN Fu	5886 NADP	1DQA	2EXX	1HW8			Sc=6.41775, min distance = 1.98971
NNMT_HUMAN Fu	188380 Ad	MCES_ENCCU Fu	439155 Ad	1RI1	2IIP	1Z3C		0.91	Sc=6.60806, min distance = 2.045565
NNMT_HUMAN Fu	60961 ade	PIMT_PYRFU Fu	439155 Ad	1JG1	2IIP	1JG2		0.84	Sc=6.32085, min distance = 2.53401
NNMT_HUMAN Fu	65482 sir	MCES_ENCCU Fu	439155 Ad	1RI1	2IIP	2HV9		0.88	Sc=6.59138, min distance = 2.33631
NNMT_MOUSE Fu	188380 Ad	MCES_ENCCU Fu	439155 Ad	1RI1	2I62	1Z3C		0.91	Sc=6.59715, min distance = 2.067885
NNMT_MOUSE Fu	60961 ade	PIMT_PYRFU Fu	439155 Ad	1JG1	2I62	1JG2		0.84	Sc=6.30124, min distance = 2.48937
NNMT_MOUSE Fu	65482 sir	MCES_ENCCU Fu	439155 Ad	1RI1	2I62	2HV9		0.88	Sc=6.13232, min distance = 2.28542
NNTM_HUMAN Fu	15942680	6PGD_LACLM Fu	5886 NADP	2IZ0	1DJL	2IZ1			Sc=6.27132, min distance = 2.49892
NNTM_HUMAN Fu	16040277	PNTB_RHORU Fu	5886 NADP	2FSV	1DJL	2OOR		0.79	Sc=6.82282, min distance = 2.101566
NNTM_HUMAN Fu	440141 9i	DHB1_HUMAN Fu	5886 NADP	1QYV	1DJL	1QYW			Sc=6.4707, min distance = 2.050243
NOR_FUSOX Fu	11957330	CCPR_YEAST Fu	444098 HE	2EUT	1F24	1BEQ		0.81	Sc=6.73855, min distance = 2.23593
NOR_FUSOX Fu	11957353	HBA_HORSE Ful	444098 HE	2D5X	1F24	1IWH		0.83	Sc=6.76788, min distance = 2.079135
NOR_FUSOX Fu	11957353	HBB_HORSE Ful	444098 HE	2D5X	1F24	1IWH		0.83	Sc=6.75816, min distance = 2.12969
NOR_FUSOX Fu	11957353	HBB_HORSE Ful	444124 HE	1Y8I	1GEJ	1IWH		0.87	Sc=6.78803, min distance = 2.36146
NOR_FUSOX Fu	11957360	CY1_BOVIN Ful	444098 HE	1L0N	1F24	1NTM			Sc=6.76669, min distance = 2.190899
NOR_FUSOX Fu	11957361	CY1_BOVIN Ful	444098 HE	1L0N	1F24	1NTZ		0.8	Sc=6.79877, min distance = 2.336575
NOR_FUSOX Fu	11957363	HBA_HUMAN Ful	444124 HE	1NQP	1GEJ	1RQA		0.81	Sc=6.76424, min distance = 2.090249
NOR_FUSOX Fu	11957363	HBB_HUMAN Ful	444098 HE	1J40	1F24	1RQA		0.77	Sc=6.78994, min distance = 1.914058
NOR_FUSOX Fu	11957370	HMOX1_HUMAN Fu	444124 HE	1OZW	1GEJ	1TWN			Sc=6.62344, min distance = 2.15357
NOR_FUSOX Fu	11957371	HMOX1_HUMAN Fu	444124 HE	1OZW	1GEJ	1TWR			Sc=6.59758, min distance = 2.361298
NOR_FUSOX Fu	11957385	MYG_PHYCA Ful	444098 HE	1A6M	1F24	2CMM			Sc=6.17973, min distance = 2.096835
NOR_FUSOX Fu	11957385	MYG_PHYCA Ful	444124 HE	1U7R	1GEJ	2CMM			Sc=6.1707, min distance = 2.280460
NOR_FUSOX Fu	11970219	CCPR_YEAST Fu	444098 HE	2EUT	1F24	1BEM		0.83	Sc=6.74063, min distance = 2.140105
NOR_FUSOX Fu	11970221	CCPR_YEAST Fu	444098 HE	2EUT	1F24	1BES		0.83	Sc=6.69447, min distance = 2.16301

NOR_FUSOX Fu	11970222	CCPR_YEAST Fu	444098 HE	2EUT	1F24	1BJ9		0.77	Sc=6.80995, min distance = 2.26019
NOR_FUSOX Fu	11970242	CCPR_YEAST Fu	444098 HE	2EUT	1F24	1CPE		0.83	Sc=6.74507, min distance = 2.19991
NOR_FUSOX Fu	4369260 C	KATG_BURPS Fu	444098 HE	2DV1	1F24	1MWV		0.93	Sc=6.75085, min distance = 2.09191
NOR_FUSOX Fu	444097 He	HBA_HUMAN Ful	444124 HE	1NQP	1GEJ	1RPS		0.95	Sc=6.7649, min distance = 1.848860
NOR_FUSOX Fu	444207 He	CP119_SULTO Fu	444124 HE	3B4X	1GEJ	1UE8		0.99	Sc=6.75164, min distance = 2.18193
NOR_FUSOX Fu	444522 HE	NOS3_BOVIN Fu	444124 HE	1ZZS	1GEJ	2HX2		0.99	Sc=6.75861, min distance = 2.21863
NOR_FUSOX Fu	446332 CI	MYG_PHYCA Ful	444098 HE	1A6M	1F24	1IOP		0.82	Sc=6.63807, min distance = 2.01109
NOR_FUSOX Fu	446406 CZ	MYG_PHYCA Ful	444098 HE	1A6M	1F24	1J3F			Sc=6.37326, min distance = 2.00156
NOR_FUSOX Fu	446406 CZ	MYG_PHYCA Ful	444124 HE	1U7R	1GEJ	1J3F			Sc=6.37091, min distance = 2.08698
NOR_FUSOX Fu	447168 ZE	CCPR_YEAST Fu	444098 HE	2EUT	1F24	1ML2		0.82	Sc=6.79255, min distance = 2.07502
NOR_FUSOX Fu	5326592 C	HMOX1_RAT Ful	444098 HE	1DVG	1F24	1J2C		0.79	Sc=6.73083, min distance = 2.09698
NOR_FUSOX Fu	5326592 C	HMOX1_RAT Ful	444124 HE	1J02	1GEJ	1J2C		0.84	Sc=6.7396, min distance = 2.139950
NOS1_RAT Ful	107984 L-	NOS3_BOVIN Fu	123895 1h	5NSE	1LZX	1ED6	68.91	0.88	Sc=5.846, min distance = 2.4477559
NOS1_RAT Ful	107984 L-	NOS3_BOVIN Fu	1649 3-br	1D0C	1OM5	1ED6	68.91		Sc=6.08507, min distance = 1.28876
NOS1_RAT Ful	107984 L-	NOS3_BOVIN Fu	347590 S-	1D1V	1K2T	1ED6	68.91		Sc=5.75361, min distance = 2.56737
NOS1_RAT Ful	107984 L-	NOS3_BOVIN Fu	440005 L-	1ED5	1K2R	1ED6	68.91	0.78	Sc=5.85251, min distance = 2.18324
NOS1_RAT Ful	107984 L-	NOS3_BOVIN Fu	444951 CI	1Q2O	1OM4	1ED6	68.91		Sc=5.769, min distance = 2.6994128
NOS1_RAT Ful	107984 L-	NOS3_BOVIN Fu	6322 L-ar	4NSE	1OM4	1ED6	68.91	0.91	Sc=5.846, min distance = 2.2402673
NOS1_RAT Ful	11149707	NOS3_BOVIN Fu	1649 3-br	1D0C	1OM5	3E7S	68.91		Sc=6.09444, min distance = 1.51377
NOS1_RAT Ful	11149707	NOS3_BOVIN Fu	347590 S-	1D1V	1K2T	3E7S	68.91		Sc=5.88782, min distance = 2.01419
NOS1_RAT Ful	11149707	NOS3_BOVIN Fu	656911 HS	1P6M	1P6I	3E7S	68.91		Sc=5.92734, min distance = 2.34087
NOS1_RAT Ful	11149707	NOS3_BOVIN Fu	656914 DF	1RS8	1RS6	3E7S	68.91		Sc=5.91562, min distance = 2.08195
NOS1_RAT Ful	11149707	NOS3_BOVIN Fu	656915 CI	1RS9	1RS7	3E7S	68.91		Sc=6.01356, min distance = 1.35456
NOS1_RAT Ful	11957370	HMOX1_HUMAN Fu	444124 HE	1OZW	1ZVI	1TWN			Sc=6.56359, min distance = 2.44071
NOS1_RAT Ful	11957371	HMOX1_HUMAN Fu	444124 HE	1OZW	1ZVI	1TWR			Sc=6.49486, min distance = 2.01374
NOS1_RAT Ful	11957385	MYG_PHYCA Ful	444098 HE	1A6M	1OM4	2CMM			Sc=6.04136, min distance = 2.26626
NOS1_RAT Ful	11957385	MYG_PHYCA Ful	444124 HE	1U7R	1ZVI	2CMM			Sc=6.04386, min distance = 2.60121
NOS1_RAT Ful	11957385	MYG_PHYCA Ful	444207 He	1VXG	1ZVL	2CMM			Sc=6.0245, min distance = 2.253885
NOS1_RAT Ful	11957385	MYG_PHYCA Ful	444522 HE	1VXD	1VAG	2CMM			Sc=6.03526, min distance = 2.43651
NOS1_RAT Ful	123831 N,	NOS3_BOVIN Fu	123895 1h	5NSE	1LZX	7NSE	68.91	0.86	Sc=5.95974, min distance = 1.929509
NOS1_RAT Ful	123831 N,	NOS3_BOVIN Fu	347590 S-	1D1V	1K2T	7NSE	68.91		Sc=5.886, min distance = 2.8544496
NOS1_RAT Ful	123831 N,	NOS3_BOVIN Fu	444951 CI	1Q2O	1OM4	7NSE	68.91		Sc=5.90438, min distance = 2.55589
NOS1_RAT Ful	123831 N,	NOS3_BOVIN Fu	6322 L-ar	4NSE	1OM4	7NSE	68.91	0.89	Sc=5.95233, min distance = 1.87226
NOS1_RAT Ful	123831 N,	NOS3_BOVIN Fu	656914 DF	1RS8	1RS6	7NSE	68.91		Sc=6.02364, min distance = 1.85940
NOS1_RAT Ful	123831 N,	NOS3_BOVIN Fu	656915 CI	1RS9	1RS7	7NSE	68.91		Sc=5.93639, min distance = 2.22109
NOS1_RAT Ful	1331 Lopa	NOS3_BOVIN Fu	123895 1h	5NSE	1LZX	1D1Y	68.91		Sc=6.09318, min distance = 2.26820
NOS1_RAT Ful	1331 Lopa	NOS3_BOVIN Fu	1649 3-br	1D0C	1OM5	1D1Y	68.91		Sc=6.06519, min distance = 2.18327

NOS1_RAT Ful	1331 Lopa	NOS3_BOVIN Fu	347590 S-	1D1V	1K2T	1D1Y	68.91		Sc=6.00052, min distance = 2.674132
NOS1_RAT Ful	1331 Lopa	NOS3_BOVIN Fu	440005 L-	1ED5	1K2R	1D1Y	68.91		Sc=6.10256, min distance = 2.398011
NOS1_RAT Ful	1331 Lopa	NOS3_BOVIN Fu	444951 CI	1Q2O	1OM4	1D1Y	68.91		Sc=6.04748, min distance = 2.465731
NOS1_RAT Ful	1331 Lopa	NOS3_BOVIN Fu	656914 DF	1RS8	1RS6	1D1Y	68.91		Sc=6.11067, min distance = 1.809879
NOS1_RAT Ful	1331 Lopa	NOS3_BOVIN Fu	656915 CI	1RS9	1RS7	1D1Y	68.91		Sc=6.01932, min distance = 2.306249
NOS1_RAT Ful	133246 di	NOS2_HUMAN Fu	6322 L-ar	1NSI	1OM4	4NOS	65.87		Sc=6.17781, min distance = 2.243289
NOS1_RAT Ful	1337 Lopa	NOS3_BOVIN Fu	123895 1h	5NSE	1LZX	1D1X	68.91		Sc=6.1209, min distance = 1.9388524
NOS1_RAT Ful	1337 Lopa	NOS3_BOVIN Fu	1649 3-br	1D0C	1OM5	1D1X	68.91		Sc=6.11604, min distance = 1.534354
NOS1_RAT Ful	1337 Lopa	NOS3_BOVIN Fu	347590 S-	1D1V	1K2T	1D1X	68.91		Sc=6.03316, min distance = 2.546116
NOS1_RAT Ful	1337 Lopa	NOS3_BOVIN Fu	440005 L-	1ED5	1K2R	1D1X	68.91		Sc=6.13104, min distance = 2.039224
NOS1_RAT Ful	1337 Lopa	NOS3_BOVIN Fu	444951 CI	1Q2O	1OM4	1D1X	68.91		Sc=6.05794, min distance = 2.609670
NOS1_RAT Ful	1337 Lopa	NOS3_BOVIN Fu	6322 L-ar	4NSE	1OM4	1D1X	68.91		Sc=6.12611, min distance = 2.126891
NOS1_RAT Ful	1337 Lopa	NOS3_BOVIN Fu	656914 DF	1RS8	1RS6	1D1X	68.91		Sc=6.13217, min distance = 1.995881
NOS1_RAT Ful	1337 Lopa	NOS3_BOVIN Fu	656915 CI	1RS9	1RS7	1D1X	68.91		Sc=6.0421, min distance = 2.4162379
NOS1_RAT Ful	181426 2-	NOS3_BOVIN Fu	123895 1h	5NSE	1LZX	1DM6	68.91		Sc=5.94812, min distance = 0.424721
NOS1_RAT Ful	181426 2-	NOS3_BOVIN Fu	16058621	2HX2	2HX3	1DM6	68.91		Sc=5.99335, min distance = 0.962229
NOS1_RAT Ful	181426 2-	NOS3_BOVIN Fu	1649 3-br	1D0C	1OM5	1DM6	68.91		Sc=5.9915, min distance = 1.0128598
NOS1_RAT Ful	181426 2-	NOS3_BOVIN Fu	440005 L-	1ED5	1K2R	1DM6	68.91		Sc=5.9423, min distance = 0.5285527
NOS1_RAT Ful	181426 2-	NOS3_BOVIN Fu	444951 CI	1Q2O	1OM4	1DM6	68.91		Sc=5.71884, min distance = 1.909645
NOS1_RAT Ful	181426 2-	NOS3_BOVIN Fu	6322 L-ar	4NSE	1OM4	1DM6	68.91		Sc=5.9124, min distance = 0.3946466
NOS1_RAT Ful	181426 2-	NOS3_BOVIN Fu	656911 HS	1P6M	1P6I	1DM6	68.91		Sc=5.89781, min distance = 0.127769
NOS1_RAT Ful	1893 7-ni	NOS3_BOVIN Fu	123895 1h	5NSE	1LZX	1FOJ	68.91		Sc=5.73529, min distance = 0.714361
NOS1_RAT Ful	1893 7-ni	NOS3_BOVIN Fu	1649 3-br	1D0C	1OM5	1FOJ	68.91	0.89	Sc=5.99737, min distance = 0.595011
NOS1_RAT Ful	1893 7-ni	NOS3_BOVIN Fu	440005 L-	1ED5	1K2R	1FOJ	68.91		Sc=5.73279, min distance = 0.950041
NOS1_RAT Ful	1893 7-ni	NOS3_BOVIN Fu	656911 HS	1P6M	1P6I	1FOJ	68.91		Sc=5.676, min distance = 2.2642089
NOS1_RAT Ful	1894 7-ni	NOS3_BOVIN Fu	123895 1h	5NSE	1LZX	1FOJ	68.91		Sc=6.11869, min distance = 0.915455
NOS1_RAT Ful	1894 7-ni	NOS3_BOVIN Fu	1649 3-br	1D0C	1OM5	1FOJ	68.91		Sc=6.02512, min distance = 0.274069
NOS1_RAT Ful	1894 7-ni	NOS3_BOVIN Fu	347590 S-	1D1V	1K2T	1FOJ	68.91		Sc=5.82513, min distance = 1.344742
NOS1_RAT Ful	1894 7-ni	NOS3_BOVIN Fu	440005 L-	1ED5	1K2R	1FOJ	68.91		Sc=6.09147, min distance = 1.082811
NOS1_RAT Ful	1894 7-ni	NOS3_BOVIN Fu	656914 DF	1RS8	1RS6	1FOJ	68.91		Sc=5.77737, min distance = 2.487937
NOS1_RAT Ful	1894 7-ni	NOS3_BOVIN Fu	656915 CI	1RS9	1RS7	1FOJ	68.91		Sc=5.8615, min distance = 1.4857651
NOS1_RAT Ful	21501 5-N	NOS3_HUMAN Fu	1649 3-br	1M9R	1OM5	1M9Q	68.33	0.82	Sc=6.0067, min distance = 0.6046990
NOS1_RAT Ful	24239 6-N	NOS3_HUMAN Fu	1649 3-br	1M9R	1OM5	1M9M	68.33	0.8	Sc=6.01616, min distance = 0.853199
NOS1_RAT Ful	24941263	NOS3_HUMAN Fu	123895 1h	3NOS	1LZX	3EAH	68.33		Sc=5.91689, min distance = 0.616559
NOS1_RAT Ful	24941263	NOS3_HUMAN Fu	1649 3-br	1M9R	1OM5	3EAH	68.33		Sc=5.92064, min distance = 0.922131
NOS1_RAT Ful	292661 2,	PTR1_LEIMA Fu	444951 CI	2BFP	1OM4	1W0C			Sc=6.11489, min distance = 0.945899
NOS1_RAT Ful	292661 2,	PTR1_LEIMA Fu	445040 6-	1E92	2G6I	1W0C			Sc=6.14987, min distance = 0.648450

NOS1_RAT Ful	439202 L-	NOS3_BOVIN Fu	123895 1h	5NSE	1LZX	6NSE	68.91	0.75	Sc=5.74433, min distance = 1.957145
NOS1_RAT Ful	439202 L-	NOS3_BOVIN Fu	347590 S-	1D1V	1K2T	6NSE	68.91		Sc=5.66723, min distance = 2.688730
NOS1_RAT Ful	439202 L-	NOS3_BOVIN Fu	440005 L-	1ED5	1K2R	6NSE	68.91		Sc=5.75701, min distance = 2.250481
NOS1_RAT Ful	439202 L-	NOS3_BOVIN Fu	6322 L-ar	4NSE	1OM4	6NSE	68.91		Sc=5.74876, min distance = 1.908826
NOS1_RAT Ful	444658 2a	FLAV_DESVH Fu	444243 FA	1F4P	1TLL	1BU5	28.19	0.97	Sc=6.61742, min distance = 2.170556
NOS1_RAT Ful	444965 2(	ARGI1_RAT Ful	123895 1h	1HQF	1LZX	1D3V			Sc=5.60314, min distance = 2.201321
NOS1_RAT Ful	445108 AE	NOS3_BOVIN Fu	123895 1h	5NSE	1LZX	1DMJ	68.91		Sc=6.12311, min distance = 1.215201
NOS1_RAT Ful	445108 AE	NOS3_BOVIN Fu	1649 3-br	1D0C	1OM5	1DMJ	68.91		Sc=6.14954, min distance = 1.533111
NOS1_RAT Ful	445108 AE	NOS3_BOVIN Fu	347590 S-	1D1V	1K2T	1DMJ	68.91		Sc=6.04782, min distance = 2.080732
NOS1_RAT Ful	445108 AE	NOS3_BOVIN Fu	440005 L-	1ED5	1K2R	1DMJ	68.91		Sc=5.96856, min distance = 1.723421
NOS1_RAT Ful	445108 AE	NOS3_BOVIN Fu	6322 L-ar	4NSE	1OM4	1DMJ	68.91		Sc=5.95797, min distance = 2.638062
NOS1_RAT Ful	445108 AE	NOS3_BOVIN Fu	656914 DF	1RS8	1RS6	1DMJ	68.91		Sc=5.99729, min distance = 2.572458
NOS1_RAT Ful	445108 AE	NOS3_BOVIN Fu	656915 CI	1RS9	1RS7	1DMJ	68.91		Sc=6.03586, min distance = 0.328001
NOS1_RAT Ful	445109 2,	NOS3_BOVIN Fu	123895 1h	5NSE	1LZX	1DMK	68.91		Sc=6.1556, min distance = 2.0477932
NOS1_RAT Ful	445109 2,	NOS3_BOVIN Fu	1649 3-br	1D0C	1OM5	1DMK	68.91		Sc=6.3206, min distance = 1.3197969
NOS1_RAT Ful	445109 2,	NOS3_BOVIN Fu	347590 S-	1D1V	1K2T	1DMK	68.91		Sc=6.19241, min distance = 1.504471
NOS1_RAT Ful	445109 2,	NOS3_BOVIN Fu	440005 L-	1ED5	1K2R	1DMK	68.91		Sc=6.35131, min distance = 0.790742
NOS1_RAT Ful	445109 2,	NOS3_BOVIN Fu	444951 CI	1Q2O	1OM4	1DMK	68.91		Sc=6.1411, min distance = 2.5321449
NOS1_RAT Ful	445109 2,	NOS3_BOVIN Fu	6322 L-ar	4NSE	1OM4	1DMK	68.91		Sc=6.19115, min distance = 1.763065
NOS1_RAT Ful	445109 2,	NOS3_BOVIN Fu	656914 DF	1RS8	1RS6	1DMK	68.91		Sc=6.17497, min distance = 1.923561
NOS1_RAT Ful	445109 2,	NOS3_BOVIN Fu	656915 CI	1RS9	1RS7	1DMK	68.91		Sc=5.88156, min distance = 1.544654
NOS1_RAT Ful	445678 CI	NOS3_BOVIN Fu	16058621	2HX2	2HX3	1FOI	68.91		Sc=5.8538, min distance = 1.2930715
NOS1_RAT Ful	445678 CI	NOS3_BOVIN Fu	1649 3-br	1D0C	1OM5	1FOI	68.91		Sc=5.88165, min distance = 0.689914
NOS1_RAT Ful	445678 CI	NOS3_BOVIN Fu	656911 HS	1P6M	1P6I	1FOI	68.91		Sc=5.68516, min distance = 0.794291
NOS1_RAT Ful	449551 FM	FLAV_DESVH Fu	444243 FA	1F4P	1TLL	5FX2	28.19	0.77	Sc=6.68236, min distance = 2.172801
NOS1_RAT Ful	9085 homo	NOS3_BOVIN Fu	123895 1h	5NSE	1LZX	1DM7	68.91	0.87	Sc=5.89265, min distance = 2.264708
NOS1_RAT Ful	9085 homo	NOS3_BOVIN Fu	347590 S-	1D1V	1K2T	1DM7	68.91		Sc=5.83163, min distance = 2.662319
NOS1_RAT Ful	9085 homo	NOS3_BOVIN Fu	440005 L-	1ED5	1K2R	1DM7	68.91	0.76	Sc=5.91359, min distance = 2.153726
NOS1_RAT Ful	9085 homo	NOS3_BOVIN Fu	444951 CI	1Q2O	1OM4	1DM7	68.91		Sc=5.8563, min distance = 2.6939855
NOS1_RAT Ful	9085 homo	NOS3_BOVIN Fu	6322 L-ar	4NSE	1OM4	1DM7	68.91	0.9	Sc=5.89908, min distance = 2.214386
NOS1_RAT Ful	9085 homo	NOS3_BOVIN Fu	656914 DF	1RS8	1RS6	1DM7	68.91		Sc=5.98298, min distance = 2.065039
NOS1_RAT Ful	9547942 C	NOS3_BOVIN Fu	123895 1h	5NSE	1LZX	2G6O	68.91	0.81	Sc=5.86263, min distance = 1.955668
NOS1_RAT Ful	9547942 C	NOS3_BOVIN Fu	1649 3-br	1D0C	1OM5	2G6O	68.91		Sc=6.08966, min distance = 1.534931
NOS1_RAT Ful	9547942 C	NOS3_BOVIN Fu	347590 S-	1D1V	1K2T	2G6O	68.91		Sc=5.80787, min distance = 2.610491
NOS1_RAT Ful	9547942 C	NOS3_BOVIN Fu	6322 L-ar	4NSE	1OM4	2G6O	68.91	0.84	Sc=5.86465, min distance = 1.912358
NOS2_HUMAN Fu	107984 L-	NOS3_BOVIN Fu	444951 CI	1Q2O	1NSI	1ED6	60.34		Sc=5.81516, min distance = 2.393492
NOS2_HUMAN Fu	107984 L-	NOS3_BOVIN Fu	6322 L-ar	4NSE	1NSI	1ED6	60.34	0.91	Sc=5.84822, min distance = 2.424171

NOS2_HUMAN Fı 11957371	HMOX1_HUMAN Fı 444124 HE 1OZW	4NOS	1TWR			Sc=6.579, min distance = 2.4937854
NOS2_HUMAN Fı 11957385	MYG_PHYCA Ful 444098 HE 1A6M	3E7G	2CMM			Sc=6.16329, min distance = 2.252264
NOS2_HUMAN Fı 11957385	MYG_PHYCA Ful 444124 HE 1U7R	4NOS	2CMM			Sc=6.18559, min distance = 2.544543
NOS2_HUMAN Fı 123831 N,	NOS3_BOVIN Fu 444951 CI 1Q2O	1NSI	7NSE	60.34		Sc=5.95424, min distance = 2.376248
NOS2_HUMAN Fı 123831 N,	NOS3_BOVIN Fu 6322 L-ar 4NSE	1NSI	7NSE	60.34	0.89	Sc=5.95609, min distance = 2.568898
NOS2_HUMAN Fı 123895 1h	NOS1_RAT Full 444951 CI 1OM4	1NSI	1LZX	65.87		Sc=5.9536, min distance = 2.2097210
NOS2_HUMAN Fı 123895 1h	NOS1_RAT Full 445040 6- 2G6I	3E7G	1LZX	65.87		Sc=6.09812, min distance = 0.937413
NOS2_HUMAN Fı 123895 1h	NOS1_RAT Full 6322 L-ar 1OM4	1NSI	1LZX	65.87	0.96	Sc=6.06626, min distance = 1.794786
NOS2_HUMAN Fı 123895 1h	NOS3_BOVIN Fu 6322 L-ar 4NSE	1NSI	5NSE	60.34	0.96	Sc=5.92872, min distance = 2.100766
NOS2_HUMAN Fı 126494 N(	NOS1_RAT Full 444951 CI 1OM4	1NSI	1K2S	65.87		Sc=6.13196, min distance = 1.598657
NOS2_HUMAN Fı 126494 N(	NOS1_RAT Full 445040 6- 2G6I	3E7G	1K2S	65.87		Sc=6.24655, min distance = 1.220565
NOS2_HUMAN Fı 126494 N(	NOS1_RAT Full 6322 L-ar 1OM4	1NSI	1K2S	65.87	0.76	Sc=6.22447, min distance = 0.865987
NOS2_HUMAN Fı 1331 Lopa	NOS3_BOVIN Fu 444951 CI 1Q2O	1NSI	1D1Y	60.34		Sc=6.06349, min distance = 2.144940
NOS2_HUMAN Fı 1337 Lopa	NOS3_BOVIN Fu 444951 CI 1Q2O	1NSI	1D1X	60.34		Sc=6.08416, min distance = 1.873670
NOS2_HUMAN Fı 1337 Lopa	NOS3_BOVIN Fu 6322 L-ar 4NSE	1NSI	1D1X	60.34		Sc=6.1234, min distance = 2.0136764
NOS2_HUMAN Fı 1433 Tocr	NOS1_RAT Full 445040 6- 2G6I	3E7G	1QWC	65.87		Sc=5.85955, min distance = 1.446467
NOS2_HUMAN Fı 1433 Tocr	NOS1_RAT Full 6322 L-ar 1OM4	1NSI	1QWC	65.87		Sc=5.87577, min distance = 2.318825
NOS2_HUMAN Fı 16058621	NOS1_RAT Full 6322 L-ar 1OM4	1NSI	2HX3	65.87		Sc=6.2135, min distance = 2.0288999
NOS2_HUMAN Fı 16058621	NOS3_BOVIN Fu 6322 L-ar 4NSE	1NSI	2HX2	60.34		Sc=6.11554, min distance = 2.224253
NOS2_HUMAN Fı 16058622	NOS1_RAT Full 444124 HE 1ZVI	4NOS	2HX4	65.87		Sc=5.79369, min distance = 2.514867
NOS2_HUMAN Fı 1649 3-br	NOS3_BOVIN Fu 444951 CI 1Q2O	1NSI	1D0C	60.34		Sc=5.81884, min distance = 0.403200
NOS2_HUMAN Fı 1649 3-br	NOS3_BOVIN Fu 6322 L-ar 4NSE	1NSI	1D0C	60.34		Sc=5.92245, min distance = 1.119471
NOS2_HUMAN Fı 1649 3-br	NOS3_HUMAN Fu 133246 di 3NOS	4NOS	1M9R	59.85		Sc=5.86794, min distance = 0.667074
NOS2_HUMAN Fı 181426 2-	NOS3_BOVIN Fu 444951 CI 1Q2O	1NSI	1DM6	60.34		Sc=5.67106, min distance = 2.347419
NOS2_HUMAN Fı 181426 2-	NOS3_BOVIN Fu 6322 L-ar 4NSE	1NSI	1DM6	60.34		Sc=5.92468, min distance = 0.815006
NOS2_HUMAN Fı 1893 7-ni	NOS3_BOVIN Fu 6322 L-ar 4NSE	1NSI	1FOJ	60.34		Sc=5.7665, min distance = 0.8722923
NOS2_HUMAN Fı 1894 7-ni	NOS3_BOVIN Fu 444951 CI 1Q2O	1NSI	1FOJ	60.34		Sc=5.75952, min distance = 2.052398
NOS2_HUMAN Fı 24941263	NOS3_HUMAN Fu 133246 di 3NOS	4NOS	3EAH	59.85		Sc=5.77615, min distance = 1.880709
NOS2_HUMAN Fı 292661 2,	PTR1_LEIMA Fu 444951 CI 2BFP	1NSI	1W0C			Sc=6.09254, min distance = 1.066760
NOS2_HUMAN Fı 292661 2,	PTR1_LEIMA Fu 445040 6-1E92	3E7G	1W0C			Sc=6.12975, min distance = 1.351476
NOS2_HUMAN Fı 3311 S-Et	NOS1_RAT Full 444951 CI 1OM4	1NSI	1K2U	65.87		Sc=5.72051, min distance = 1.750578
NOS2_HUMAN Fı 3311 S-Et	NOS1_RAT Full 445040 6- 2G6I	3E7G	1K2U	65.87		Sc=5.85521, min distance = 1.137309
NOS2_HUMAN Fı 3311 S-Et	NOS1_RAT Full 6322 L-ar 1OM4	1NSI	1K2U	65.87		Sc=5.81461, min distance = 1.944450
NOS2_HUMAN Fı 347590 S-	NOS1_RAT Full 444951 CI 1OM4	1NSI	1K2T	65.87		Sc=5.6304, min distance = 1.8181234
NOS2_HUMAN Fı 347590 S-	NOS1_RAT Full 445040 6- 2G6I	3E7G	1K2T	65.87		Sc=5.77389, min distance = 1.092004
NOS2_HUMAN Fı 347590 S-	NOS1_RAT Full 6322 L-ar 1OM4	1NSI	1K2T	65.87		Sc=5.71384, min distance = 1.943924
NOS2_HUMAN Fı 347590 S-	NOS3_BOVIN Fu 11149707 3E7S	3E7G	1D1V	60.34		Sc=5.88629, min distance = 0.555342

NOS2_HUMAN F	347590 S-	NOS3_BOVIN Fu	444951 CI	1Q2O	1NSI	1D1V	60.34		Sc=5.62082, min distance = 1.85563
NOS2_HUMAN F	347590 S-	NOS3_BOVIN Fu	6322 L-ar	4NSE	1NSI	1D1V	60.34		Sc=5.65991, min distance = 2.12413
NOS2_HUMAN F	439202 L-	NOS3_BOVIN Fu	444951 CI	1Q2O	1NSI	6NSE	60.34		Sc=5.7281, min distance = 2.175699
NOS2_HUMAN F	439202 L-	NOS3_BOVIN Fu	6322 L-ar	4NSE	1NSI	6NSE	60.34		Sc=5.74433, min distance = 2.04673
NOS2_HUMAN F	440005 L-	NOS1_RAT Full	444951 CI	1OM4	1NSI	1K2R	65.87		Sc=6.04054, min distance = 2.16168
NOS2_HUMAN F	440005 L-	NOS1_RAT Full	445040 6-	2G6I	3E7G	1K2R	65.87		Sc=6.14128, min distance = 0.96604
NOS2_HUMAN F	440005 L-	NOS1_RAT Full	6322 L-ar	1OM4	1NSI	1K2R	65.87	0.84	Sc=6.13329, min distance = 1.77588
NOS2_HUMAN F	440005 L-	NOS3_BOVIN Fu	444951 CI	1Q2O	1NSI	1ED5	60.34		Sc=6.01116, min distance = 1.82042
NOS2_HUMAN F	440005 L-	NOS3_BOVIN Fu	6322 L-ar	4NSE	1NSI	1ED5	60.34	0.84	Sc=6.02018, min distance = 2.02640
NOS2_HUMAN F	444288 CI	KARG_LIMPO Fu	6322 L-ar	1M15	1NSI	1P52			Sc=5.73102, min distance = 2.10447
NOS2_HUMAN F	445107 CI	NOS3_BOVIN Fu	444951 CI	1Q2O	1NSI	1DMI	60.34	0.81	Sc=6.15745, min distance = 2.47295
NOS2_HUMAN F	445108 AB	NOS3_BOVIN Fu	6322 L-ar	4NSE	1NSI	1DMJ	60.34		Sc=6.13615, min distance = 1.01051
NOS2_HUMAN F	445109 2,	NOS3_BOVIN Fu	444951 CI	1Q2O	1NSI	1DMK	60.34		Sc=6.15853, min distance = 2.39166
NOS2_HUMAN F	445109 2,	NOS3_BOVIN Fu	6322 L-ar	4NSE	1NSI	1DMK	60.34		Sc=6.33349, min distance = 0.87652
NOS2_HUMAN F	447180 To	NOS1_RAT Full	444951 CI	1OM4	1NSI	1MMV	65.87		Sc=6.12763, min distance = 1.76671
NOS2_HUMAN F	447180 To	NOS1_RAT Full	445040 6-	2G6I	3E7G	1MMV	65.87		Sc=6.25051, min distance = 0.88498
NOS2_HUMAN F	447180 To	NOS1_RAT Full	6322 L-ar	1OM4	1NSI	1MMV	65.87	0.83	Sc=6.17241, min distance = 1.45466
NOS2_HUMAN F	447181 VI	NOS1_RAT Full	444951 CI	1OM4	1NSI	1MMW	65.87		Sc=6.0535, min distance = 1.898126
NOS2_HUMAN F	447181 VI	NOS1_RAT Full	445040 6-	2G6I	3E7G	1MMW	65.87		Sc=6.1891, min distance = 1.346166
NOS2_HUMAN F	447181 VI	NOS1_RAT Full	6322 L-ar	1OM4	1NSI	1MMW	65.87	0.78	Sc=6.15687, min distance = 1.78275
NOS2_HUMAN F	449021 CI	NOS1_RAT Full	444124 HE	1ZVI	4NOS	1VAG	65.87		Sc=6.173, min distance = 2.0138304
NOS2_HUMAN F	449021 CI	NOS1_RAT Full	445040 6-	2G6I	3E7G	1VAG	65.87		Sc=6.18438, min distance = 1.59874
NOS2_HUMAN F	6251 D-ma	NOS1_RAT Full	444951 CI	1OM4	1NSI	1P6K	65.87		Sc=5.84181, min distance = 1.85698
NOS2_HUMAN F	6251 D-ma	NOS1_RAT Full	6322 L-ar	1OM4	1NSI	1P6K	65.87		Sc=5.85224, min distance = 1.55705
NOS2_HUMAN F	656910 di	NOS1_RAT Full	444098 HE	1OM4	3E7G	1P6K	65.87		Sc=6.24844, min distance = 2.16974
NOS2_HUMAN F	656910 di	NOS1_RAT Full	444124 HE	1ZVI	4NOS	1P6K	65.87		Sc=6.27437, min distance = 2.06839
NOS2_HUMAN F	656910 di	NOS1_RAT Full	444951 CI	1OM4	1NSI	1P6K	65.87		Sc=6.28377, min distance = 2.30244
NOS2_HUMAN F	656910 di	NOS1_RAT Full	445040 6-	2G6I	3E7G	1P6K	65.87		Sc=6.25924, min distance = 2.08709
NOS2_HUMAN F	656910 di	NOS1_RAT Full	6322 L-ar	1OM4	1NSI	1P6K	65.87		Sc=6.26604, min distance = 2.51220
NOS2_HUMAN F	656911 HS	NOS3_BOVIN Fu	444951 CI	1Q2O	1NSI	1P6M	60.34		Sc=5.91845, min distance = 2.66844
NOS2_HUMAN F	656911 HS	NOS3_BOVIN Fu	6322 L-ar	4NSE	1NSI	1P6M	60.34		Sc=5.99929, min distance = 2.81193
NOS2_HUMAN F	656912 N;	NOS3_BOVIN Fu	444951 CI	1Q2O	1NSI	1ZZS	60.34		Sc=5.94769, min distance = 2.64572
NOS2_HUMAN F	656914 DE	NOS1_RAT Full	444951 CI	1OM4	1NSI	1RS6	65.87		Sc=5.85425, min distance = 1.68079
NOS2_HUMAN F	656914 DE	NOS3_BOVIN Fu	444951 CI	1Q2O	1NSI	1RS8	60.34		Sc=5.85963, min distance = 2.20459
NOS2_HUMAN F	656915 CI	NOS1_RAT Full	444124 HE	1ZVI	4NOS	1RS7	65.87		Sc=5.92368, min distance = 1.99510
NOS2_HUMAN F	657085 DE	ARGI1_RAT Ful	6322 L-ar	1T5G	1NSI	1T4P			Sc=5.62651, min distance = 2.13678
NOS2_HUMAN F	9085 homo	NOS3_BOVIN Fu	444951 CI	1Q2O	1NSI	1DM7	60.34		Sc=5.886, min distance = 2.1146191

NOS2_HUMAN F	9085 hom	NOS3_BOVIN Fu	6322 L-ar	4NSE	1NSI	1DM7	60.34	0.9	Sc=5.89908, min distance = 2.46120
NOS2_HUMAN F	9547941 4	NOS1_RAT Full	444951 CI	1OM4	1NSI	2G6J	65.87		Sc=5.76659, min distance = 2.80529
NOS2_HUMAN F	9547941 4	NOS1_RAT Full	445040 6-	2G6I	3E7G	2G6J	65.87	0.79	Sc=5.77558, min distance = 2.58327
NOS2_HUMAN F	9547941 4	NOS1_RAT Full	6322 L-ar	1OM4	1NSI	2G6J	65.87		Sc=5.87273, min distance = 2.16069
NOS2_HUMAN F	9547942 C	NOS3_BOVIN Fu	444951 CI	1Q2O	1NSI	2G6O	60.34		Sc=5.85006, min distance = 1.97944
NOS2_HUMAN F	9547942 C	NOS3_BOVIN Fu	6322 L-ar	4NSE	1NSI	2G6O	60.34	0.84	Sc=5.85835, min distance = 2.06093
NOS2_MOUSE F	11957363	HBA_HUMAN Ful	444124 HE	1NQP	2ORT	1RQA		0.81	Sc=6.42952, min distance = 2.21030
NOS2_MOUSE F	11957363	HBB_HUMAN Ful	444124 HE	1NQP	2ORT	1RQA		0.81	Sc=6.73604, min distance = 2.20242
NOS2_MOUSE F	11957385	MYG_PHYCA Ful	444098 HE	1A6M	3DWJ	2CMM			Sc=6.14512, min distance = 2.31391
NOS2_MOUSE F	11957385	MYG_PHYCA Ful	444124 HE	1U7R	2ORT	2CMM			Sc=6.23061, min distance = 2.08046
NOS2_MOUSE F	123831 N,	NOS3_BOVIN Fu	444951 CI	1Q2O	1N2N	7NSE	63.46		Sc=5.9404, min distance = 2.642434
NOS2_MOUSE F	123895 1h	NOS1_RAT Full	1433 Tocr	1QWC	1QW5	1LZX	65.48		Sc=5.93229, min distance = 2.17107
NOS2_MOUSE F	123895 1h	NOS1_RAT Full	444951 CI	1OM4	1N2N	1LZX	65.48		Sc=5.93753, min distance = 2.32564
NOS2_MOUSE F	123895 1h	NOS1_RAT Full	445040 6-	2G6I	3E6N	1LZX	65.48		Sc=5.9636, min distance = 2.325029
NOS2_MOUSE F	123895 1h	NOS3_BOVIN Fu	444951 CI	1Q2O	1N2N	5NSE	63.46		Sc=5.90286, min distance = 2.41174
NOS2_MOUSE F	123895 1h	NOSO_BACSU Fu	445040 6-	2FC1	3E6N	2FBZ			Sc=5.64228, min distance = 2.25163
NOS2_MOUSE F	126494 N(	NOS1_RAT Full	1433 Tocr	1QWC	1QW5	1K2S	65.48		Sc=6.1238, min distance = 2.058973
NOS2_MOUSE F	126494 N(	NOS1_RAT Full	444951 CI	1OM4	1N2N	1K2S	65.48		Sc=6.14066, min distance = 2.14115
NOS2_MOUSE F	126494 N(	NOS1_RAT Full	445040 6-	2G6I	3E6N	1K2S	65.48		Sc=6.15309, min distance = 1.93634
NOS2_MOUSE F	1331 Lopa	NOS3_BOVIN Fu	444951 CI	1Q2O	1N2N	1D1Y	63.46		Sc=6.05794, min distance = 2.49468
NOS2_MOUSE F	1337 Lopa	NOS3_BOVIN Fu	444951 CI	1Q2O	1N2N	1D1X	63.46		Sc=6.07799, min distance = 2.35458
NOS2_MOUSE F	16058622	NOS1_RAT Full	445040 6-	2G6I	3E6N	2HX4	65.48		Sc=5.98291, min distance = 2.40347
NOS2_MOUSE F	1649 3-br	NOS3_BOVIN Fu	444951 CI	1Q2O	1N2N	1D0C	63.46		Sc=5.80476, min distance = 0.66522
NOS2_MOUSE F	1649 3-br	NOS3_HUMAN Fu	133246 di	3NOS	3DWJ	1M9R	63.71		Sc=5.80997, min distance = 0.81039
NOS2_MOUSE F	181426 2-	NOS3_BOVIN Fu	444951 CI	1Q2O	1N2N	1DM6	63.46		Sc=5.62959, min distance = 2.46663
NOS2_MOUSE F	24941263	NOS3_HUMAN Fu	133246 di	3NOS	3DWJ	3EAH	63.71		Sc=5.73097, min distance = 2.02202
NOS2_MOUSE F	292661 2,	PTR1_LEIMA Fu	444951 CI	2BFP	1N2N	1W0C			Sc=5.90874, min distance = 2.36125
NOS2_MOUSE F	292661 2,	PTR1_LEIMA Fu	445040 6-1E92		3E6N	1W0C			Sc=5.93211, min distance = 2.12775
NOS2_MOUSE F	3311 S-Et	NOS1_RAT Full	1433 Tocr	1QWC	1QW5	1K2U	65.48		Sc=5.72718, min distance = 2.16351
NOS2_MOUSE F	3311 S-Et	NOS1_RAT Full	444951 CI	1OM4	1N2N	1K2U	65.48		Sc=5.71811, min distance = 2.32541
NOS2_MOUSE F	3311 S-Et	NOS1_RAT Full	445040 6-	2G6I	3E6N	1K2U	65.48		Sc=5.72494, min distance = 1.89689
NOS2_MOUSE F	3311 S-Et	NOS1_RAT Full	447180 Tc	1MMV	1QW4	1K2U	65.48		Sc=5.70428, min distance = 2.57502
NOS2_MOUSE F	347590 S-	NOS1_RAT Full	1433 Tocr	1QWC	1QW5	1K2T	65.48		Sc=5.62082, min distance = 2.20010
NOS2_MOUSE F	347590 S-	NOS1_RAT Full	444951 CI	1OM4	1N2N	1K2T	65.48		Sc=5.6257, min distance = 2.294778
NOS2_MOUSE F	347590 S-	NOS1_RAT Full	445040 6-	2G6I	3E6N	1K2T	65.48		Sc=5.63477, min distance = 1.84386
NOS2_MOUSE F	347590 S-	NOS1_RAT Full	447180 Tc	1MMV	1QW4	1K2T	65.48		Sc=5.60103, min distance = 2.51974
NOS2_MOUSE F	347590 S-	NOS3_BOVIN Fu	444951 CI	1Q2O	1N2N	1D1V	63.46		Sc=5.61098, min distance = 2.29080

NOS2_MOUSE Fu	440005 L-	NOS1_RAT Full	1433 Tocr	1QWC	1QW5	1K2R	65.48		Sc=6.02018, min distance = 1.80348(
NOS2_MOUSE Fu	440005 L-	NOS1_RAT Full	444951 CI	1OM4	1N2N	1K2R	65.48		Sc=6.03618, min distance = 2.22608
NOS2_MOUSE Fu	440005 L-	NOS1_RAT Full	445040 6-	2G6I	3E6N	1K2R	65.48		Sc=6.06147, min distance = 2.19656
NOS2_MOUSE Fu	440005 L-	NOS1_RAT Full	447180 Tc	1MMV	1QW4	1K2R	65.48		Sc=6.02436, min distance = 2.29402
NOS2_MOUSE Fu	440005 L-	NOS3_BOVIN Fu	444951 CI	1Q2O	1N2N	1ED5	63.46		Sc=5.9939, min distance = 2.377140
NOS2_MOUSE Fu	445109 2,	NOS3_BOVIN Fu	444951 CI	1Q2O	1N2N	1DMK	63.46		Sc=6.12509, min distance = 2.38082
NOS2_MOUSE Fu	446189 CI	HBG1_HUMAN Fu	444124 HE	1I3E	2ORT	1I3D		0.89	Sc=6.74356, min distance = 2.28765(
NOS2_MOUSE Fu	447181 VI	NOS1_RAT Full	1433 Tocr	1QWC	1QW5	1MMW	65.48		Sc=6.04895, min distance = 2.12795
NOS2_MOUSE Fu	447181 VI	NOS1_RAT Full	444951 CI	1OM4	1N2N	1MMW	65.48		Sc=6.04895, min distance = 2.24183
NOS2_MOUSE Fu	447181 VI	NOS1_RAT Full	445040 6-	2G6I	3E6N	1MMW	65.48		Sc=6.07266, min distance = 2.27319
NOS2_MOUSE Fu	447181 VI	NOS1_RAT Full	447180 Tc	1MMV	1QW4	1MMW	65.48		Sc=6.04278, min distance = 2.35372(
NOS2_MOUSE Fu	6322 L-ar	NOS1_RAT Full	1433 Tocr	1QWC	1QW5	1OM4	65.48		Sc=5.85639, min distance = 2.14365
NOS2_MOUSE Fu	6322 L-ar	NOS1_RAT Full	445040 6-	2G6I	3E6N	1OM4	65.48		Sc=5.88942, min distance = 2.33605
NOS2_MOUSE Fu	6322 L-ar	NOS3_BOVIN Fu	444951 CI	1Q2O	1N2N	4NSE	63.46		Sc=5.83738, min distance = 2.58082
NOS2_MOUSE Fu	656910 di	NOS1_RAT Full	1433 Tocr	1QWC	1QW5	1P6K	65.48		Sc=5.91475, min distance = 2.15659
NOS2_MOUSE Fu	656910 di	NOS1_RAT Full	444098 HE	1OM4	3DWJ	1P6K	65.48		Sc=6.2947, min distance = 2.129472
NOS2_MOUSE Fu	656910 di	NOS1_RAT Full	444951 CI	1OM4	1N2N	1P6K	65.48		Sc=6.24672, min distance = 2.60336
NOS2_MOUSE Fu	656910 di	NOS1_RAT Full	445040 6-	2G6I	3E6N	1P6K	65.48		Sc=6.27524, min distance = 2.51777
NOS2_MOUSE Fu	656910 di	NOS1_RAT Full	447180 Tc	1MMV	1QW4	1P6K	65.48		Sc=5.85242, min distance = 2.23734
NOS2_MOUSE Fu	656914 DE	NOS1_RAT Full	444207 He	1ZVL	1NOS	1RS6	65.48		Sc=5.618, min distance = 2.4945055
NOS2_MOUSE Fu	656915 CI	NOS1_RAT Full	445040 6-	2G6I	3E6N	1RS7	65.48		Sc=5.93971, min distance = 2.03481
NOS2_MOUSE Fu	656915 CI	NOS1_RAT Full	447180 Tc	1MMV	1QW4	1RS7	65.48		Sc=5.9472, min distance = 2.290289
NOS2_MOUSE Fu	9085 homo	NOS3_BOVIN Fu	444951 CI	1Q2O	1N2N	1DM7	63.46		Sc=5.88145, min distance = 2.56822(
NOS2_MOUSE Fu	9547941 4	NOS1_RAT Full	1433 Tocr	1QWC	1QW5	2G6J	65.48		Sc=5.84969, min distance = 2.00382
NOS2_MOUSE Fu	9547941 4	NOS1_RAT Full	444951 CI	1OM4	1N2N	2G6J	65.48		Sc=5.7576, min distance = 2.774682
NOS2_MOUSE Fu	9547941 4	NOS1_RAT Full	445040 6-	2G6I	3E6N	2G6J	65.48	0.79	Sc=5.73644, min distance = 3.19974
NOS3_BOVIN Fu	11957370	HMOX1_HUMAN Fu	444124 HE	1OZW	1ZZS	1TWN			Sc=6.57891, min distance = 2.44166
NOS3_BOVIN Fu	11957371	HMOX1_HUMAN Fu	444124 HE	1OZW	1ZZS	1TWR			Sc=6.58222, min distance = 2.48503
NOS3_BOVIN Fu	11957385	MYG_PHYCA Full	444098 HE	1A6M	1D0C	2CMM			Sc=6.04905, min distance = 2.40941
NOS3_BOVIN Fu	11957385	MYG_PHYCA Full	444124 HE	1U7R	1ZZS	2CMM			Sc=6.18909, min distance = 2.57852
NOS3_BOVIN Fu	11957385	MYG_PHYCA Full	444522 HE	1VXD	2HX2	2CMM			Sc=6.17511, min distance = 2.27523
NOS3_BOVIN Fu	126494 N(	NOS1_RAT Full	123895 1h	1LZX	5NSE	1K2S	68.91		Sc=6.09925, min distance = 2.37993(
NOS3_BOVIN Fu	126494 N(	NOS1_RAT Full	347590 S-	1K2T	1D1V	1K2S	68.91		Sc=6.11712, min distance = 1.31513
NOS3_BOVIN Fu	126494 N(	NOS1_RAT Full	440005 L-	1K2R	1ED5	1K2S	68.91		Sc=6.06893, min distance = 2.32459
NOS3_BOVIN Fu	126494 N(	NOS1_RAT Full	444951 CI	1OM4	1Q2O	1K2S	68.91		Sc=6.15634, min distance = 1.71750
NOS3_BOVIN Fu	126494 N(	NOS1_RAT Full	6322 L-ar	1OM4	4NSE	1K2S	68.91	0.76	Sc=6.09153, min distance = 2.58321
NOS3_BOVIN Fu	126494 N(	NOS1_RAT Full	656914 DE	1RS6	1RS8	1K2S	68.91		Sc=6.24855, min distance = 1.04288

NOS3_BOVIN F	126494 N	NOS1_RAT Full	656915 CI	1RS7	1RS9	1K2S	68.91		Sc=6.08666, min distance = 2.59261
NOS3_BOVIN F	1433 Tocr	NOS1_RAT Full	123895 1h	1LZX	5NSE	1QWC	68.91		Sc=5.95639, min distance = 1.74702
NOS3_BOVIN F	1433 Tocr	NOS1_RAT Full	1649 3-br	1OM5	1D0C	1QWC	68.91		Sc=5.92188, min distance = 0.95714
NOS3_BOVIN F	1433 Tocr	NOS1_RAT Full	347590 S-	1K2T	1D1V	1QWC	68.91		Sc=5.79455, min distance = 2.05036
NOS3_BOVIN F	1433 Tocr	NOS1_RAT Full	440005 L-	1K2R	1ED5	1QWC	68.91		Sc=6.01588, min distance = 1.13113
NOS3_BOVIN F	1433 Tocr	NOS1_RAT Full	6322 L-ar	1OM4	4NSE	1QWC	68.91		Sc=5.98299, min distance = 1.61478
NOS3_BOVIN F	1433 Tocr	NOS1_RAT Full	656911 HS	1P6I	1P6M	1QWC	68.91		Sc=5.96581, min distance = 0.47629
NOS3_BOVIN F	1433 Tocr	NOS2_MOUSE Fu	444951 CI	1N2N	1Q2O	1QW5	63.46		Sc=5.62567, min distance = 2.47571
NOS3_BOVIN F	16058622	NOS1_RAT Full	16058621	2HX3	2HX2	2HX4	68.91		Sc=5.94886, min distance = 1.55509
NOS3_BOVIN F	16058622	NOS1_RAT Full	440005 L-	1K2R	1ED5	2HX4	68.91		Sc=6.52072, min distance = 0.60254
NOS3_BOVIN F	24850786	NOS1_RAT Full	656914 DF	1RS6	1RS8	3B3N	68.91		Sc=5.95331, min distance = 2.12315
NOS3_BOVIN F	24850913	NOS1_RAT Full	16058621	2HX3	2HX2	3B3M	68.91		Sc=6.15161, min distance = 1.09104
NOS3_BOVIN F	24850913	NOS1_RAT Full	656915 CI	1RS7	1RS9	3B3M	68.91		Sc=5.84628, min distance = 1.93873
NOS3_BOVIN F	292661 2,	PTR1_LEIMA Fu	444951 CI	2BFP	1Q2O	1W0C			Sc=6.13136, min distance = 0.98637
NOS3_BOVIN F	3311 S-Et	NOS1_RAT Full	347590 S-	1K2T	1D1V	1K2U	68.91	0.87	Sc=5.716, min distance = 1.7854708
NOS3_BOVIN F	3311 S-Et	NOS1_RAT Full	440005 L-	1K2R	1ED5	1K2U	68.91		Sc=5.88208, min distance = 0.62532
NOS3_BOVIN F	3311 S-Et	NOS1_RAT Full	444951 CI	1OM4	1Q2O	1K2U	68.91		Sc=5.72718, min distance = 1.82299
NOS3_BOVIN F	3311 S-Et	NOS1_RAT Full	6322 L-ar	1OM4	4NSE	1K2U	68.91		Sc=5.85385, min distance = 0.36715
NOS3_BOVIN F	3311 S-Et	NOS1_RAT Full	656914 DF	1RS6	1RS8	1K2U	68.91		Sc=5.71811, min distance = 2.12650
NOS3_BOVIN F	3311 S-Et	NOS1_RAT Full	656915 CI	1RS7	1RS9	1K2U	68.91		Sc=5.70013, min distance = 2.58193
NOS3_BOVIN F	4369001 C	HBA_HUMAN Ful	444522 HE	2DN1	2HX2	4HHB		0.81	Sc=6.53669, min distance = 2.31444
NOS3_BOVIN F	444288 CI	KARG_LIMPO Fu	6322 L-ar	1M15	4NSE	1P52			Sc=5.72108, min distance = 2.01345
NOS3_BOVIN F	444965 2	ARGI1_RAT Ful	6322 L-ar	1T5G	4NSE	1D3V			Sc=5.62651, min distance = 1.84201
NOS3_BOVIN F	445040 6-	NOS1_RAT Full	656915 CI	1RS7	1RS9	2G6I	68.91		Sc=6.16683, min distance = 1.73950
NOS3_BOVIN F	446406 CZ	MYG_PHYCA Ful	444124 HE	1U7R	1ZZS	1J3F			Sc=6.12834, min distance = 2.62591
NOS3_BOVIN F	447180 T	NOS1_RAT Full	123895 1h	1LZX	5NSE	1MMV	68.91	0.81	Sc=6.08831, min distance = 2.29150
NOS3_BOVIN F	447180 T	NOS1_RAT Full	347590 S-	1K2T	1D1V	1MMV	68.91		Sc=6.10847, min distance = 1.14696
NOS3_BOVIN F	447180 T	NOS1_RAT Full	440005 L-	1K2R	1ED5	1MMV	68.91		Sc=6.27815, min distance = 0.79149
NOS3_BOVIN F	447180 T	NOS1_RAT Full	444951 CI	1OM4	1Q2O	1MMV	68.91		Sc=6.1395, min distance = 1.495451
NOS3_BOVIN F	447180 T	NOS1_RAT Full	6322 L-ar	1OM4	4NSE	1MMV	68.91	0.83	Sc=6.07608, min distance = 2.38764
NOS3_BOVIN F	447180 T	NOS1_RAT Full	656914 DF	1RS6	1RS8	1MMV	68.91		Sc=6.1508, min distance = 1.085962
NOS3_BOVIN F	447180 T	NOS1_RAT Full	656915 CI	1RS7	1RS9	1MMV	68.91		Sc=6.08666, min distance = 2.28016
NOS3_BOVIN F	447180 T	NOS2_MOUSE Fu	444951 CI	1N2N	1Q2O	1QW4	63.46		Sc=6.11157, min distance = 2.47339
NOS3_BOVIN F	447181 VI	NOS1_RAT Full	123895 1h	1LZX	5NSE	1MMW	68.91	0.76	Sc=6.01049, min distance = 2.56053
NOS3_BOVIN F	447181 VI	NOS1_RAT Full	347590 S-	1K2T	1D1V	1MMW	68.91		Sc=6.03656, min distance = 1.05413
NOS3_BOVIN F	447181 VI	NOS1_RAT Full	440005 L-	1K2R	1ED5	1MMW	68.91		Sc=6.21433, min distance = 1.10596
NOS3_BOVIN F	447181 VI	NOS1_RAT Full	444951 CI	1OM4	1Q2O	1MMW	68.91		Sc=6.07959, min distance = 1.70224

NOS3_BOVIN F	447181 VI	NOS1_RAT Full	6322 L-ar	1OM4	4NSE	1MMW	68.91	0.78	Sc=6.17977, min distance = 1.17786
NOS3_BOVIN F	447181 VI	NOS1_RAT Full	656914 DF	1RS6	1RS8	1MMW	68.91		Sc=6.09338, min distance = 1.21986
NOS3_BOVIN F	447181 VI	NOS1_RAT Full	656915 CI	1RS7	1RS9	1MMW	68.91		Sc=6.01396, min distance = 2.48733
NOS3_BOVIN F	449021 CI	NOS1_RAT Full	440005 L-	1K2R	1ED5	1VAG	68.91		Sc=6.20892, min distance = 1.54579
NOS3_BOVIN F	449021 CI	NOS1_RAT Full	444124 HE	1ZVI	1ZZS	1VAG	68.91		Sc=6.16592, min distance = 1.92206
NOS3_BOVIN F	449021 CI	NOS1_RAT Full	444951 CI	1OM4	1Q2O	1VAG	68.91		Sc=6.18438, min distance = 2.14275
NOS3_BOVIN F	449021 CI	NOS1_RAT Full	656914 DF	1RS6	1RS8	1VAG	68.91		Sc=6.18909, min distance = 2.47522
NOS3_BOVIN F	449021 CI	NOS1_RAT Full	656915 CI	1RS7	1RS9	1VAG	68.91		Sc=6.1492, min distance = 2.532117
NOS3_BOVIN F	6251 D-ma	NOS1_RAT Full	347590 S-	1K2T	1D1V	1P6K	68.91		Sc=5.79829, min distance = 2.06298
NOS3_BOVIN F	6251 D-ma	NOS1_RAT Full	444951 CI	1OM4	1Q2O	1P6K	68.91		Sc=5.74254, min distance = 2.86746
NOS3_BOVIN F	6251 D-ma	NOS1_RAT Full	656914 DF	1RS6	1RS8	1P6K	68.91		Sc=5.80558, min distance = 1.89075
NOS3_BOVIN F	6251 D-ma	NOS1_RAT Full	656915 CI	1RS7	1RS9	1P6K	68.91		Sc=5.79088, min distance = 1.63732
NOS3_BOVIN F	657085 DE	ARGI1_RAT Ful	123895 1h	1HQF	5NSE	1T4P			Sc=5.61411, min distance = 2.33773
NOS3_BOVIN F	657087 CI	ARGI1_RAT Ful	123895 1h	1HQF	5NSE	1T4T		0.8	Sc=5.6117, min distance = 2.728539
NOS3_BOVIN F	9547941 4	NOS1_RAT Full	347590 S-	1K2T	1D1V	2G6J	68.91		Sc=5.71402, min distance = 3.18116
NOS3_BOVIN F	9547941 4	NOS1_RAT Full	440005 L-	1K2R	1ED5	2G6J	68.91		Sc=5.93987, min distance = 1.09380
NOS3_BOVIN F	9547941 4	NOS1_RAT Full	444951 CI	1OM4	1Q2O	2G6J	68.91		Sc=5.72905, min distance = 2.87398
NOS3_BOVIN F	9547941 4	NOS1_RAT Full	6322 L-ar	1OM4	4NSE	2G6J	68.91		Sc=5.93815, min distance = 1.34363
NOS3_BOVIN F	9547941 4	NOS1_RAT Full	656914 DF	1RS6	1RS8	2G6J	68.91		Sc=5.69605, min distance = 2.88002
NOS3_HUMAN F	11957363	HBB_HUMAN Ful	444124 HE	1NQP	1M9K	1RQA		0.81	Sc=6.65233, min distance = 2.18249
NOS3_HUMAN F	11957370	HMOX1_HUMAN F	444124 HE	1OZW	1M9K	1TWN			Sc=6.57397, min distance = 2.39736
NOS3_HUMAN F	11957371	HMOX1_HUMAN F	444124 HE	1OZW	1M9K	1TWR			Sc=6.57408, min distance = 1.84208
NOS3_HUMAN F	11957385	MYG_PHYCA Ful	444098 HE	1A6M	1M9J	2CMM			Sc=6.14109, min distance = 2.32639
NOS3_HUMAN F	11957385	MYG_PHYCA Ful	444124 HE	1U7R	1M9K	2CMM			Sc=6.04657, min distance = 2.40782
NOS3_HUMAN F	11957385	MYG_PHYCA Ful	444207 He	1VXG	1M9M	2CMM			Sc=6.16464, min distance = 2.34866
NOS3_HUMAN F	126494 N	NOS1_RAT Full	123895 1h	1LZX	3NOS	1K2S	68.33		Sc=6.0777, min distance = 2.112373
NOS3_HUMAN F	126494 N	NOS1_RAT Full	1649 3-br	1OM5	1M9R	1K2S	68.33		Sc=6.16561, min distance = 1.10196
NOS3_HUMAN F	1433 Tocr	NOS1_RAT Full	1649 3-br	1OM5	1M9R	1QWC	68.33		Sc=5.99305, min distance = 1.70261
NOS3_HUMAN F	1433 Tocr	NOS2_MOUSE Fu	133246 di	3DWJ	3NOS	1QW5	63.71		Sc=5.74908, min distance = 1.99120
NOS3_HUMAN F	16058622	NOS1_RAT Full	444207 He	1ZVL	1M9M	2HX4	68.33		Sc=5.62426, min distance = 2.28457
NOS3_HUMAN F	3311 S-Et	NOS1_RAT Full	1649 3-br	1OM5	1M9R	1K2U	68.33		Sc=5.8763, min distance = 0.978376
NOS3_HUMAN F	440005 L-	NOS1_RAT Full	123895 1h	1LZX	3NOS	1K2R	68.33	0.85	Sc=5.99219, min distance = 2.28904
NOS3_HUMAN F	440005 L-	NOS1_RAT Full	1649 3-br	1OM5	1M9R	1K2R	68.33		Sc=5.94632, min distance = 1.15434
NOS3_HUMAN F	444522 HE	CPXA_PSEPU Fu	444207 He	1PHC	1M9M	1QMQ	1		Sc=6.7235, min distance = 1.971672
NOS3_HUMAN F	447180 Tc	NOS1_RAT Full	123895 1h	1LZX	3NOS	1MMV	68.33	0.81	Sc=6.05842, min distance = 2.36280
NOS3_HUMAN F	447180 Tc	NOS1_RAT Full	1649 3-br	1OM5	1M9R	1MMV	68.33		Sc=6.16206, min distance = 1.03848
NOS3_HUMAN F	447181 VI	NOS1_RAT Full	123895 1h	1LZX	3NOS	1MMW	68.33	0.76	Sc=5.98411, min distance = 2.51439

NOS3_HUMAN Fı	447181 VI	NOS1_RAT Full:	1649 3-br 1OM5	1M9R	1MMW	68.33		Sc=5.96531, min distance = 1.40591
NOS3_HUMAN Fı	6322 L-ar	ARGI1_RAT Ful	123895 1h 1HQF	3NOS	1T5G		0.96	Sc=5.78107, min distance = 2.28289
NOS3_HUMAN Fı	6322 L-ar	NOS1_RAT Full:	1649 3-br 1OM5	1M9R	1OM4	68.33		Sc=5.94196, min distance = 1.26045
NOS3_HUMAN Fı	656910 di	NOS1_RAT Full:	123895 1h 1LZX	3NOS	1P6K	68.33		Sc=5.64792, min distance = 2.24988
NOS3_HUMAN Fı	656910 di	NOS1_RAT Full:	444098 HE 1OM4	1M9J	1P6K	68.33		Sc=5.85872, min distance = 2.26452
NOS3_HUMAN Fı	656910 di	NOS1_RAT Full:	444207 He 1ZVL	1M9M	1P6K	68.33		Sc=6.21106, min distance = 2.59692
NOSO_BACSU Fı	107984 L-	NOS3_BOVIN Fu	123895 1h 5NSE	2FBZ	1ED6		0.88	Sc=5.81516, min distance = 2.42968
NOSO_BACSU Fı	11149707	NOS3_BOVIN Fu	123895 1h 5NSE	2FBZ	3E7S			Sc=5.96111, min distance = 2.22206
NOSO_BACSU Fı	11149707	NOS3_BOVIN Fu	6322 L-ar 4NSE	2FC1	3E7S			Sc=5.96491, min distance = 2.11961
NOSO_BACSU Fı	11957370	HMOX1_HUMAN Fı	444124 HE 1OZW	2AN0	1TWN			Sc=6.57891, min distance = 2.04689
NOSO_BACSU Fı	11957371	HMOX1_HUMAN Fı	444124 HE 1OZW	2AN0	1TWR			Sc=6.58556, min distance = 2.22632
NOSO_BACSU Fı	11957385	MYG_PHYCA Ful	444207 He 1VXG	1M7V	2CMM			Sc=6.17735, min distance = 2.31203
NOSO_BACSU Fı	11957385	MYG_PHYCA Ful	444522 HE 1VXD	2FC1	2CMM			Sc=6.19534, min distance = 2.24948
NOSO_BACSU Fı	11970222	CCPR_YEAST Fu	444522 HE 3E2O	2FC1	1BJ9		0.81	Sc=6.33725, min distance = 2.34046
NOSO_BACSU Fı	123831 N,	NOS3_BOVIN Fu	123895 1h 5NSE	2FBZ	7NSE		0.86	Sc=5.94833, min distance = 2.50380
NOSO_BACSU Fı	123831 N,	NOS3_BOVIN Fu	6322 L-ar 4NSE	2FC1	7NSE		0.89	Sc=5.95797, min distance = 2.63117
NOSO_BACSU Fı	126494 N(	NOS1_RAT Full:	123895 1h 1LZX	2FBZ	1K2S			Sc=6.10847, min distance = 2.37982
NOSO_BACSU Fı	126494 N(	NOS1_RAT Full:	6322 L-ar 1OM4	2FC1	1K2S		0.76	Sc=6.09775, min distance = 2.30918
NOSO_BACSU Fı	1433 Tocı	NOS1_RAT Full:	123895 1h 1LZX	2FBZ	1QWC			Sc=5.6038, min distance = 2.132821
NOSO_BACSU Fı	1433 Tocı	NOS1_RAT Full:	6322 L-ar 1OM4	2FC1	1QWC			Sc=5.61779, min distance = 2.19439
NOSO_BACSU Fı	16058622	NOS1_RAT Full:	123895 1h 1LZX	2FBZ	2HX4			Sc=5.86209, min distance = 2.08564
NOSO_BACSU Fı	16058622	NOS1_RAT Full:	444207 He 1ZVL	1M7V	2HX4			Sc=5.86764, min distance = 2.42142
NOSO_BACSU Fı	16058622	NOS1_RAT Full:	444522 HE 1VAG	2FC1	2HX4			Sc=5.89091, min distance = 2.24466
NOSO_BACSU Fı	1649 3-br	NOS3_BOVIN Fu	6322 L-ar 4NSE	2FC1	1D0C			Sc=5.78318, min distance = 0.66455
NOSO_BACSU Fı	292661 2,	PTR1_LEIMA Fu	445040 6-1E92	2FC1	1W0C			Sc=5.81508, min distance = 2.48236
NOSO_BACSU Fı	3311 S-Et	NOS1_RAT Full:	6322 L-ar 1OM4	2FC1	1K2U			Sc=5.691, min distance = 2.1964717
NOSO_BACSU Fı	4369001 (	HBA_HUMAN Ful	444522 HE 2DN1	2FC1	4HHB		0.81	Sc=6.54958, min distance = 2.27433
NOSO_BACSU Fı	439202 L-	NOS3_BOVIN Fu	123895 1h 5NSE	2FBZ	6NSE		0.75	Sc=5.73294, min distance = 2.49307
NOSO_BACSU Fı	439202 L-	NOS3_BOVIN Fu	6322 L-ar 4NSE	2FC1	6NSE			Sc=5.74647, min distance = 2.57065
NOSO_BACSU Fı	440005 L-	NOS1_RAT Full:	123895 1h 1LZX	2FBZ	1K2R		0.85	Sc=5.98332, min distance = 2.16940
NOSO_BACSU Fı	440005 L-	NOS1_RAT Full:	6322 L-ar 1OM4	2FC1	1K2R		0.84	Sc=5.98332, min distance = 2.14216
NOSO_BACSU Fı	440005 L-	NOS3_BOVIN Fu	123895 1h 5NSE	2FBZ	1ED5		0.85	Sc=5.97448, min distance = 2.11785
NOSO_BACSU Fı	440005 L-	NOS3_BOVIN Fu	6322 L-ar 4NSE	2FC1	1ED5		0.84	Sc=5.99049, min distance = 1.89929
NOSO_BACSU Fı	444965 2(	ARGI1_RAT Ful	123895 1h 1HQF	2FBZ	1D3V			Sc=5.62651, min distance = 2.06861
NOSO_BACSU Fı	446122 S2	ARGI1_RAT Ful	6322 L-ar 1T5G	2FC1	3E9B			Sc=5.61707, min distance = 1.36946
NOSO_BACSU Fı	447180 To	NOS1_RAT Full:	123895 1h 1LZX	2FBZ	1MMV		0.81	Sc=6.09153, min distance = 2.31491
NOSO_BACSU Fı	447180 To	NOS1_RAT Full:	6322 L-ar 1OM4	2FC1	1MMV		0.83	Sc=6.09153, min distance = 2.16757

NOSO_BACSU Fu	447181 VI	NOS1_RAT Full=	123895 1h	1LZX	2FBZ	1MMW		0.76	Sc=6.01933, min distance = 2.407912
NOSO_BACSU Fu	447181 VI	NOS1_RAT Full=	6322 L-ar	1OM4	2FC1	1MMW		0.78	Sc=6.01933, min distance = 2.426218
NOSO_BACSU Fu	449021 CI	NOS1_RAT Full=	444124 HE	1ZVI	2AN0	1VAG			Sc=6.12724, min distance = 2.167989
NOSO_BACSU Fu	449021 CI	NOS1_RAT Full=	444207 He	1ZVL	1M7V	1VAG			Sc=6.16464, min distance = 2.257186
NOSO_BACSU Fu	656912 N;	NOS3_BOVIN Fu	444522 HE	2HX2	2FC1	1ZZS			Sc=6.2802, min distance = 2.4713415
NOSO_BACSU Fu	657085 DE	ARGI1_RAT Full	123895 1h	1HQF	2FBZ	1T4P			Sc=5.64273, min distance = 2.081432
NOSO_BACSU Fu	657087 CI	ARGI1_RAT Full	123895 1h	1HQF	2FBZ	1T4T		0.8	Sc=5.65131, min distance = 2.419050
NOSO_BACSU Fu	9085 homo	NOS3_BOVIN Fu	123895 1h	5NSE	2FBZ	1DM7		0.87	Sc=5.89494, min distance = 2.360623
NOSO_BACSU Fu	9085 homo	NOS3_BOVIN Fu	6322 L-ar	4NSE	2FC1	1DM7		0.9	Sc=5.90537, min distance = 2.343043
NOSO_BACSU Fu	9547942 C	NOS3_BOVIN Fu	123895 1h	5NSE	2FBZ	2G6O		0.81	Sc=5.84595, min distance = 2.317739
NOSO_BACSU Fu	9547942 C	NOS3_BOVIN Fu	6322 L-ar	4NSE	2FC1	2G6O		0.84	Sc=5.86046, min distance = 2.154231
NOSO_STAAU Fu	11957370	HMOX1_HUMAN Fu	444124 HE	1OZW	1MJT	1TWN			Sc=6.58375, min distance = 2.184231
NOSO_STAAU Fu	11957371	HMOX1_HUMAN Fu	444124 HE	1OZW	1MJT	1TWR			Sc=6.58277, min distance = 2.412892
NOSO_STAAU Fu	11957385	MYG_PHYCA Full	444124 HE	1U7R	1MJT	2CMM			Sc=6.17186, min distance = 2.595150
NOSO_STAAU Fu	449021 CI	NOS1_RAT Full=	444124 HE	1ZVI	1MJT	1VAG			Sc=6.12855, min distance = 2.651981
NP2_RHOPR Ful	11957371	HMOX1_HUMAN Fu	444124 HE	1OZW	1EUO	1TWR			Sc=6.48028, min distance = 1.590853
NP2_RHOPR Ful	5354052 p	MIF_HUMAN Full	311 citri	1GD0	2AL0	2OOZ			Sc=5.62687, min distance = 2.041135
NP2_RHOPR Ful	8778 Laur	CTXA3_NAJAT Fu	311 citri	1XT3	2AL0	1H0J			Sc=5.6106, min distance = 0.5907097
NP4_RHOPR Ful	3713 Indo	TRPA_THET2 Ful	311 citri	1UJP	1ERX	1WXJ			Sc=6.05533, min distance = 2.465525
NPD2_ARCFU Fu	2968 deca	ACES_MOUSE Ful	8200 TETR	2H9Y	1S7G	1MAA			Sc=5.90747, min distance = 2.065911
NPD2_ARCFU Fu	439353 be	O96048_LUMTE	8172 Trig	2DRY	1YC2	2DS0			Sc=5.6304, min distance = 2.2760582
NPD2_ARCFU Fu	9547960 A	NPD_THEMA Full	5893 nadi	2H4H	1S7G	2H59			Sc=5.84646, min distance = 2.316371
NPD2_ARCFU Fu	9548000 G	CD38_HUMAN Ful	5893 nadi	2I65	1S7G	2I66			Sc=6.16472, min distance = 1.747870
NQO1_MOUSE Fu	449077 CI	NQO1_HUMAN Ful	444188 CI	1D4A	1DXQ	2F1O		1	Sc=6.86064, min distance = 1.888379
NQO1_THET8 Fu	5326566 l	BLVRB_HUMAN Fu	444243 FA	1HE4	2FUG	1HE5		0.82	Sc=6.28716, min distance = 2.442792
NQO2_HUMAN Fu	449077 CI	NQO1_HUMAN Ful	444188 CI	1D4A	1SG0	2F1O	49.09	1	Sc=6.84126, min distance = 2.465059
NQO2_HUMAN Fu	656982 CI	NFNB_ECOLI Ful	89105 Tre	1IDT	1XI2	1YKI			Sc=5.96822, min distance = 1.456796
NR1D2_HUMAN	11957353	HBA_HORSE Full	444098 HE	2D5X	3CQV	1IWH		0.83	Sc=6.73414, min distance = 2.064017
NR1D2_HUMAN	11957360	CY1_BOVIN Full	444098 HE	1L0N	3CQV	1NTM			Sc=6.72306, min distance = 2.128753
NR1D2_HUMAN	11957361	CY1_BOVIN Full	444098 HE	1L0N	3CQV	1NTZ		0.8	Sc=6.72705, min distance = 2.231404
NR1D2_HUMAN	11957363	HBB_HUMAN Full	444098 HE	1J40	3CQV	1RQA		0.77	Sc=6.77337, min distance = 2.085143
NR1D2_HUMAN	11957385	MYG_PHYCA Full	444098 HE	1A6M	3CQV	2CMM			Sc=6.27783, min distance = 2.173011
NR1D2_HUMAN	16741061	MYG_PHYCA Full	444098 HE	1A6M	3CQV	1MBC		0.83	Sc=6.73226, min distance = 2.044862
NR1D2_HUMAN	16741062	MYG_PHYCA Full	444098 HE	1A6M	3CQV	1MBN		0.84	Sc=6.7754, min distance = 2.1138207
NR1D2_HUMAN	4369122 G	GLB1_LUCPE Ful	444098 HE	1FLP	3CQV	1B0B		0.96	Sc=6.69537, min distance = 2.210282
NR1I2_HUMAN	447911 CI	NR1H2_HUMAN Fu	447912 T2	1UPV	2O9I	1PQ9	31.79	0.83	Sc=5.8025, min distance = 2.0593098
NRAM_I18A0 Fu	445533 2c	NRAM_I75A5 Ful	60855 zan	1NNC	3B7E	1F8C		0.94	Sc=5.99845, min distance = 2.592441

NRAM_I18A0 Fı	445535 1f	NRAM_I75A5 Fu.	60855 zan	1NNC	3B7E	1F8E		0.92	Sc=5.99961, min distance = 2.65371(
NRAM_I18A0 Fı	449201 1x	NRAM_I75A5 Fu.	60855 zan	1NNC	3B7E	1XOG			Sc=6.25748, min distance = 2.32534!
NRAM_I18A0 Fı	65309 DAN	Q6WJ03_9PARA 1	60855 zan	1V3E	3B7E	1V3D		0.88	Sc=6.32468, min distance = 2.45175.
NRAM_I56A2 Fı	15133 m-X	CARP_YEAST Fu.	185698 al	1DPJ	1V0Z	1FQ5			Sc=5.76844, min distance = 1.39040!
NRAM_I56A2 Fı	154234 Pe	NRAM_I75A5 Fu.	444885 O-	1MWE	1W1X	1L7F	69.85		Sc=6.43501, min distance = 2.60930(
NRAM_I56A2 Fı	3364665 E	NRAM_I67A0 Fu.	444885 O-	2BAT	1W1X	1IVD	51.54		Sc=6.24064, min distance = 1.34996.
NRAM_I56A2 Fı	445534 1f	NRAM_I75A5 Fu.	444885 O-	1MWE	1W1X	1F8D	69.85		Sc=6.3003, min distance = 2.658135.
NRAM_I56A2 Fı	445535 1f	NRAM_I75A5 Fu.	444885 O-	1MWE	1W1X	1F8E	69.85		Sc=5.98357, min distance = 2.59546(
NRAM_I56A2 Fı	446367 BA	NRAM_I67A0 Fu.	444885 O-	2BAT	1W1X	1IVE	51.54		Sc=6.17859, min distance = 1.25610!
NRAM_I56A2 Fı	449379 2c	NRAM_I75A5 Fu.	444885 O-	1MWE	1W1X	2QWF	69.85		Sc=6.36685, min distance = 1.71624.
NRAM_I56A2 Fı	449381 Os	NRAM_I75A5 Fu.	444885 O-	1MWE	1W1X	2QWH	69.85		Sc=6.21873, min distance = 2.71452!
NRAM_I56A2 Fı	60855 zar	NRAM_I75A5 Fu.	444885 O-	1MWE	1W1X	1NNC	69.85		Sc=6.41533, min distance = 2.52642{
NRAM_I56A2 Fı	65309 DAN	NRAM_I67A0 Fu.	444885 O-	2BAT	1W1X	1IVF	51.54		Sc=6.02207, min distance = 2.70233.
NRAM_I56A2 Fı	65309 DAN	NRAM_I75A5 Fu.	444885 O-	1MWE	1W1X	2QWC	69.85		Sc=5.96761, min distance = 2.62318.
NRAM_I56A2 Fı	9543480 2	SN_MOUSE Full:	444885 O-	1QFO	1W1X	2BVE			Sc=5.70261, min distance = 2.07138(
NRAM_I63A3 Fı	11963505	NEUR2_HUMAN Fı	154234 Pe	2F10	2HTU	2F11			Sc=6.18679, min distance = 2.49857.
NRAM_I63A3 Fı	11963505	NEUR2_HUMAN Fı	447208 DA	2F25	2HTR	2F11		0.86	Sc=6.19241, min distance = 2.40905.
NRAM_I63A3 Fı	11963506	NEUR2_HUMAN Fı	154234 Pe	2F10	2HTU	2F12			Sc=6.21501, min distance = 2.21562.
NRAM_I63A3 Fı	11963506	NEUR2_HUMAN Fı	447208 DA	2F25	2HTR	2F12		0.88	Sc=6.21501, min distance = 1.79238.
NRAM_I63A3 Fı	11963507	NEUR2_HUMAN Fı	154234 Pe	2F10	2HTU	2F13			Sc=6.26897, min distance = 2.42364!
NRAM_I63A3 Fı	11963507	NEUR2_HUMAN Fı	447208 DA	2F25	2HTR	2F13		0.89	Sc=6.27383, min distance = 1.92314.
NRAM_I63A3 Fı	444885 O-	NRAM_I75A5 Fu.	60855 zan	1NNC	2HTQ	1MWE	43.15		Sc=5.85654, min distance = 2.24520.
NRAM_I63A3 Fı	445533 2c	NRAM_I75A5 Fu.	154234 Pe	1L7F	2HTU	1F8C	43.15		Sc=5.99845, min distance = 2.68829(
NRAM_I63A3 Fı	445533 2c	NRAM_I75A5 Fu.	60855 zan	1NNC	2HTQ	1F8C	43.15	0.94	Sc=6.3029, min distance = 2.212341
NRAM_I63A3 Fı	445534 1f	NRAM_I75A5 Fu.	154234 Pe	1L7F	2HTU	1F8D	43.15		Sc=5.99622, min distance = 2.62686(
NRAM_I63A3 Fı	445534 1f	NRAM_I75A5 Fu.	60855 zan	1NNC	2HTQ	1F8D	43.15	0.86	Sc=6.29597, min distance = 2.25214.
NRAM_I63A3 Fı	445535 1f	NRAM_I75A5 Fu.	154234 Pe	1L7F	2HTU	1F8E	43.15		Sc=5.99732, min distance = 2.64327
NRAM_I63A3 Fı	445535 1f	NRAM_I75A5 Fu.	60855 zan	1NNC	2HTQ	1F8E	43.15	0.92	Sc=6.3003, min distance = 2.279144.
NRAM_I63A3 Fı	449201 1x	NRAM_I75A5 Fu.	154234 Pe	1L7F	2HTU	1XOG	43.15		Sc=6.26319, min distance = 2.48839.
NRAM_I63A3 Fı	449201 1x	NRAM_I75A5 Fu.	60855 zan	1NNC	2HTQ	1XOG	43.15		Sc=6.24086, min distance = 2.24097
NRAM_I63A3 Fı	449379 2c	NRAM_I75A5 Fu.	60855 zan	1NNC	2HTQ	2QWF	43.15	0.83	Sc=6.42774, min distance = 2.34080.
NRAM_I63A3 Fı	449380 2c	NRAM_I75A5 Fu.	154234 Pe	1L7F	2HTU	2QWG	43.15		Sc=6.29084, min distance = 2.71440(
NRAM_I63A3 Fı	449380 2c	NRAM_I75A5 Fu.	60855 zan	1NNC	2HTQ	2QWG	43.15	0.77	Sc=6.2612, min distance = 2.165616.
NRAM_I63A3 Fı	5288452 C	NEUR2_HUMAN Fı	154234 Pe	2F10	2HTU	2F0Z			Sc=6.4463, min distance = 2.340444{
NRAM_I63A3 Fı	65309 DAN	NRAM_I75A5 Fu.	154234 Pe	1L7F	2HTU	2QWC	43.15		Sc=5.98108, min distance = 2.71026.
NRAM_I63A3 Fı	65309 DAN	NRAM_I75A5 Fu.	60855 zan	1NNC	2HTQ	2QWC	43.15	0.88	Sc=6.28546, min distance = 2.17983
NRAM_I63A3 Fı	65309 DAN	Q6WJ03_9PARA 1	60855 zan	1V3E	2HTQ	1V3D		0.88	Sc=6.30164, min distance = 2.23629.

NRAM_I67A0 Fu	16122528	NRAM_I56A2 Ful	444885 O-	1W1X	2BAT	2CML	51.54		Sc=6.46295, min distance = 1.491398
NRAM_I67A0 Fu	24139 ace	Q309D1_9AGAR I	444885 O-	2C25	2BAT	2C4D		0.79	Sc=5.89692, min distance = 2.310928
NRAM_I67A0 Fu	60855 zar	NRAM_I75A5 Ful	444885 O-	1MWE	2BAT	1NNC	48.45		Sc=5.99008, min distance = 2.328575
NRAM_I67A0 Fu	9543480 2	SN_MOUSE Full-	444885 O-	1QFO	2BAT	2BVE			Sc=5.65104, min distance = 2.194498
NRAM_I75A5 Fu	11963505	NEUR2_HUMAN Fu	154234 Pe	2F10	1L7F	2F11			Sc=6.18017, min distance = 2.480119
NRAM_I75A5 Fu	11963506	NEUR2_HUMAN Fu	154234 Pe	2F10	1L7F	2F12			Sc=6.2016, min distance = 2.5080340
NRAM_I75A5 Fu	11963507	NEUR2_HUMAN Fu	154234 Pe	2F10	1L7F	2F13			Sc=6.249, min distance = 2.4636317
NRAM_I75A5 Fu	16122528	NRAM_I56A2 Ful	444885 O-	1W1X	1MWE	2CML	69.85		Sc=6.48821, min distance = 1.239737
NRAM_I75A5 Fu	24139 ace	Q309D1_9AGAR I	444885 O-	2C25	1MWE	2C4D		0.79	Sc=5.94766, min distance = 2.502250
NRAM_I75A5 Fu	3364665 E	NRAM_I67A0 Ful	444885 O-	2BAT	1MWE	1IVD	48.45		Sc=6.11175, min distance = 2.699516
NRAM_I75A5 Fu	446367 BA	NRAM_I67A0 Ful	444885 O-	2BAT	1MWE	1IVE	48.45		Sc=5.96531, min distance = 2.908011
NRAM_I75A5 Fu	447208 DA	NEUR2_HUMAN Fu	154234 Pe	2F10	1L7F	2F25			Sc=6.30784, min distance = 2.509376
NRAM_I75A5 Fu	449401 2,	NANL_MACDE Ful	65309 DAN	1SLI	2QWC	2SLI			Sc=5.8751, min distance = 2.5953876
NRAM_I75A5 Fu	5288452 C	NEUR2_HUMAN Fu	154234 Pe	2F10	1L7F	2F0Z			Sc=6.43097, min distance = 2.536036
NRAM_INBLE Fu	185698 al	PGH1_SHEEP Ful	444205 CI	1EQG	1B9V	1Q4G			Sc=5.79423, min distance = 1.509905
NRDD_BPT4 Ful	33113 gam	PRIM_BPT7 Full	64968 dTT	1CR1	1H79	1E0J			Sc=6.31248, min distance = 2.437753
NRDI_BACSU Fu	16741044	PYRDA_LACLC Fu	444243 FA	1JUB	1RLJ	1JRB			Sc=6.22899, min distance = 2.238552
NRDI_BACSU Fu	449551 FM	FLAV_DESVH Ful	444243 FA	1F4P	1RLJ	5FX2		0.77	Sc=6.22625, min distance = 2.177795
NRDI_BACSU Fu	5326566 1	BLVRB_HUMAN Fu	444243 FA	1HE4	1RLJ	1HE5		0.82	Sc=6.30701, min distance = 2.249556
NRK1_HUMAN Fu	16750062	CSK2A_MAIZE Fu	33113 gam	1LP4	2QT0	2OXD			Sc=5.66724, min distance = 2.034164
NRK1_HUMAN Fu	1694 TBBt	CDK2_HUMAN Ful	6022 Aden	1GY3	2QSY	1P5E			Sc=5.64083, min distance = 1.433700
NRK1_HUMAN Fu	188966 dA	HSLU_ECOLI Ful	33113 gam1E94		2QT0	1G4A		0.97	Sc=5.94388, min distance = 2.198044
NRK1_HUMAN Fu	444564 AD	BIOD_ECOLI Ful	6022 Aden	1DAD	2QSY	1BS1		0.91	Sc=5.93233, min distance = 2.160378
NRK1_HUMAN Fu	445940 O6	CDK2_HUMAN Ful	6022 Aden	1GY3	2QSY	1GZ8			Sc=6.04851, min distance = 2.198308
NRK1_HUMAN Fu	448042 2e	ROCK1_HUMAN Fu	33113 gam	2V55	2QT0	2ETR			Sc=6.14112, min distance = 2.163981
NRK1_HUMAN Fu	448043 2g	ROCK1_HUMAN Fu	33113 gam	2V55	2QT0	3D9V			Sc=6.01882, min distance = 2.147682
NRK1_HUMAN Fu	4565 1h1r	CDK2_HUMAN Ful	6022 Aden	1GY3	2QSY	1H1R			Sc=6.0669, min distance = 2.7313489
NRK1_HUMAN Fu	5289411 1	CDK2_HUMAN Ful	6022 Aden	1GY3	2QSY	1OGU			Sc=5.64205, min distance = 1.981614
NRK1_HUMAN Fu	5326976 1	CSK2A_MAIZE Fu	33113 gam	1LP4	2QT0	1ZOE			Sc=5.75401, min distance = 2.085539
NRK1_HUMAN Fu	5957 Aden	BIOD_ECOLI Ful	6022 Aden	1DAD	2QSY	1A82		0.99	Sc=5.98651, min distance = 2.119031
NRK1_HUMAN Fu	6083 aden	ENTP2_RAT Full	33113 gam	3CJA	2QT0	3CJ7		0.97	Sc=5.92734, min distance = 2.964047
NRK1_HUMAN Fu	6083 aden	HSP71_HUMAN Fu	33113 gam	2E8A	2QT0	1XQS		0.97	Sc=5.97529, min distance = 1.962521
NRK1_HUMAN Fu	91532 AMP	AROK_MYCTU Ful	6022 Aden	2IYV	2QSY	1ZYU		0.99	Sc=6.02455, min distance = 2.309979
NRK1_HUMAN Fu	91532 AMP	BIOD_ECOLI Ful	6022 Aden	1DAD	2QSY	1DAG		0.99	Sc=6.01454, min distance = 2.394465
NSF_CRIGR Ful	188966 dA	HSLU_ECOLI Ful	33113 gam1E94		1D2N	1G4A		0.97	Sc=6.45414, min distance = 1.504363
NSF_CRIGR Ful	444564 AD	MYS2_DICDI Ful	5957 Aden	1FMW	1NSF	1W9I		0.91	Sc=6.47813, min distance = 0
NSF_CRIGR Ful	444852 CI	ATPA1_BOVIN Fu	5957 Aden	2V7Q	1NSF	1COW		0.91	Sc=6.52437, min distance = 1.925983

NSF_CRIGR Fu	444852 CI	ATPB_BOVIN Fu	5957 Aden	2V7Q	1NSF	1COW	0.91	Sc=6.53366, min distance = 1.69772
NSF_CRIGR Fu	447004 CI	MYS2_DICDI Fu	5957 Aden	1FMW	1NSF	1LVK		Sc=6.56245, min distance = 0
NSF_CRIGR Fu	6022 Ader	AROK_MYCTU Fu	5957 Aden	2IYW	1NSF	2IYV	0.99	Sc=6.47469, min distance = 2.23541
NSF_CRIGR Fu	6022 Ader	Q72H90_THET2 I	5957 Aden	2BEK	1NSF	2BEJ	0.99	Sc=6.4787, min distance = 2.224673
NSF_CRIGR Fu	6022 Ader	VPS4B_MOUSE Fu	5957 Aden	2ZAN	1NSF	2ZAO	0.99	Sc=6.47416, min distance = 2.10254
NSF_CRIGR Fu	6022 Ader	Y059_METJA Fu	5957 Aden	2J9C	1NSF	2J9D	0.99	Sc=6.47471, min distance = 2.34461
NSF_CRIGR Fu	6852187 2	O57883_PYRHO I	5957 Aden	2DTO	1NSF	2DTH		Sc=6.20542, min distance = 1.28404
NSF_CRIGR Fu	91532 AME	AROK_MYCTU Fu	5957 Aden	2IYW	1NSF	1ZYU	0.99	Sc=6.08494, min distance = 2.33073
NSF_CRIGR Fu	91532 AME	NIFH1_AZOVI Fu	5957 Aden	2C8V	1NSF	2AFK	0.99	Sc=6.54868, min distance = 1.77332
NSUN5_HUMAN I	439176 Me	MTR1_RHOSH Fu	34756 Acy	1NW5	2B9E	1EG2	0.91	Sc=6.28285, min distance = 2.15485
NSUN5_HUMAN I	445971 CI	COMT_RAT Full=	34756 Acy	2CL5	2B9E	1H1D	0.92	Sc=6.55883, min distance = 2.47799
NT5C_HUMAN Fu	65059 Dec	TYSY_ECOLI Fu	13712 dec	1BDU	2I7D	1TLC		Sc=6.32041, min distance = 2.30871
NT5M_HUMAN Fu	445210 9-	KITH_HHV11 Fu	9700 thym	1VTK	1Z4L	1E2I		Sc=5.93349, min distance = 2.16687
NT5M_HUMAN Fu	445211 9-	KITH_HHV11 Fu	9700 thym	1VTK	1Z4L	1E2I		Sc=5.93528, min distance = 2.16665
NT5M_HUMAN Fu	5789 thym	RMLA1_ECOLI Fu	9700 thym	1H5S	1Z4L	1H5R	0.92	Sc=6.10137, min distance = 1.52201
NTPA_METJA Fu	15993 dAT	PRIM_BPT7 Full	33113 gam	1E0J	2MJP	1CR2	0.97	Sc=5.68751, min distance = 2.08204
NTPA_METJA Fu	446721 CI	ASSY_THET8 Fu	33113 gam	1KOR	2MJP	1KH2	0.93	Sc=6.35571, min distance = 1.82832
NTPA_METJA Fu	6083 ader	HSP71_HUMAN Fu	33113 gam	2E8A	2MJP	1XQS	0.97	Sc=6.26663, min distance = 2.28733
NTPK_ENTHR Fu	445180 CI	OBP_PIG Full=(	8184 Unde1E02		2BL2	1DZJ		Sc=5.71375, min distance = 1.76516
NUD16_XENLA I	444845 1c	RASH_HUMAN Fu	6830 guan	2CL7	2A8S	1CLU	0.98	Sc=5.61423, min distance = 2.01366
NUD16_XENLA I	445871 CI	RASH_HUMAN Fu	6830 guan	2CL7	2A8S	1GNP		Sc=5.71209, min distance = 1.09957
NUDD_ECOLI Fu	447979 5G	KGUA_ECOLI Fu	8977 1dar	2AN9	1RYA	2ANB		Sc=6.3529, min distance = 2.446566
NUDD_ECOLI Fu	6804 guar	NDK_PYRHO Full	8977 1dar	2DXE	1RYA	2DXF	0.99	Sc=6.35183, min distance = 2.26648
NUDD_ECOLI Fu	6804 guar	TGM3_HUMAN Fu	8977 1dar	1VJJ	1RYA	1SGX	0.99	Sc=6.34047, min distance = 2.20950
NUDT5_HUMAN I	10109823	PIM1_HUMAN Fu	6083 aden	1YXU	2DSD	3CY3		Sc=6.2823, min distance = 2.035637
NUDT5_HUMAN I	9578243 E	PDE4B_HUMAN Fu	6083 aden	1ROR	2DSD	1XLZ		Sc=5.76574, min distance = 2.35565
O04941_AEGTA	124886 gl	GST26_SCHJA Fu	97536 Hex	1M9A	1GWC	1DUG	0.9	Sc=6.32965, min distance = 2.22035
O04941_AEGTA	124886 gl	GSTA3_CHICK Fu	97536 Hex	1VF2	1GWC	1VF1	0.9	Sc=5.88199, min distance = 2.07928
O04941_AEGTA	124886 gl	GSTP1_MOUSE Fu	97536 Hex	2GLR	1GWC	1GSY	0.9	Sc=5.86895, min distance = 1.99929
O04941_AEGTA	124886 gl	URE2_YEAST Fu	97536 Hex	1K0A	1GWC	1K0D	0.9	Sc=5.88618, min distance = 1.89375
O04941_AEGTA	444104 II	GST26_SCHJA Fu	97536 Hex	1M9A	1GWC	1M99	0.84	Sc=6.3486, min distance = 2.178609
O04941_AEGTA	445616 gl	GST26_SCHJA Fu	97536 Hex	1M9A	1GWC	1GNE	0.9	Sc=5.84409, min distance = 2.28992
O04941_AEGTA	445707 s-	GSTA1_HUMAN Fu	97536 Hex	1K3Y	1GWC	1GUH		Sc=6.1063, min distance = 2.534283
O04941_AEGTA	447108 CI	GST26_SCHJA Fu	97536 Hex	1M9A	1GWC	1M9B		Sc=6.24358, min distance = 2.28278
O04941_AEGTA	449366 3c	GSTP1_HUMAN Fu	97536 Hex	1PGT	1GWC	2PGT		Sc=6.66657, min distance = 1.82780
O04941_AEGTA	5326751 1	LGUL_HUMAN Fu	97536 Hex	1BH5	1GWC	1QIN		Sc=6.15102, min distance = 1.86241
O06934_MYCTU	16740985	FRDA_SHEFR Fu	444188 CI	1M64	1V0J	1E39	0.93	Sc=6.44709, min distance = 2.02249

O08355_PSEFL	439153 Di	ADH1B_HUMAN Fu	5893 nadi	1U3U	1LJ8	1DEH	0.79	Sc=6.30211, min distance = 1.86315
O08355_PSEFL	445794 AD	ADHX_HUMAN Ful	5893 nadi	2FZW	1LJ8	2FZE		Sc=6.06792, min distance = 2.44657
O08355_PSEFL	446288 NA	G3P_PALVE Ful	5893 nadi	1DSS	1LJ8	1IHX		Sc=6.27521, min distance = 2.04785
O08375_MORSP	123927 A3	Q9GT92_CRYPV	5893 nadi	2FM3	2GSD	2EWD	0.97	Sc=6.78429, min distance = 2.25790
O08375_MORSP	439153 Di	ADH1B_HUMAN Fu	5893 nadi	1U3U	2GSD	1DEH	0.79	Sc=6.77968, min distance = 2.08340
O08375_MORSP	439153 Di	ADH1E_HORSE Fu	5893 nadi	1MGO	2GSD	2JHF	0.79	Sc=6.77904, min distance = 2.30059
O08375_MORSP	6102709 A	LDH_PLAFD Ful	5893 nadi	1T2D	2GSD	2A94	0.88	Sc=6.78361, min distance = 2.15452
O22304_VITVI	6030 Uric	PYRH_ECOLI Fu	6031 Urid	2BND	2C1X	2BNE	0.99	Sc=6.30728, min distance = 1.84097
O22304_VITVI	8629 UDP-	Q5IFH7_MEDTR	6031 Urid	2ACV	2C1X	2ACW	0.89	Sc=6.66053, min distance = 2.07456
O22321_MUSAC	29435 1-D	CDGT_BACS0 Fu	64689 bet	1UKS	2BN0	1I75		Sc=5.82924, min distance = 1.84916
O22321_MUSAC	447607 1c	Q79G13_MYCTU	64689 bet	1UP0	2BN0	1UP2		Sc=5.63826, min distance = 2.87566
O22443_SOYBN	11957353	HBB_HORSE Ful	444124 HE	1Y8I	1FHF	1IWH	0.87	Sc=6.73456, min distance = 1.75886
O22443_SOYBN	11957363	HBA_HUMAN Ful	444124 HE	1NQP	1FHF	1RQA	0.81	Sc=6.68824, min distance = 1.90304
O22443_SOYBN	11957370	HMOX1_HUMAN Fu	444124 HE	1OZW	1FHF	1TWN		Sc=6.56176, min distance = 2.02897
O22443_SOYBN	11957371	HMOX1_HUMAN Fu	444124 HE	1OZW	1FHF	1TWR		Sc=6.49157, min distance = 2.16624
O22443_SOYBN	11957385	MYG_PHYCA Ful	444124 HE	1U7R	1FHF	2CMM		Sc=6.0515, min distance = 2.381756
O22443_SOYBN	444097 He	HBA_HUMAN Ful	444124 HE	1NQP	1FHF	1RPS	0.95	Sc=6.73855, min distance = 2.20640
O22443_SOYBN	444207 He	HBA_HORSE Ful	444124 HE	1Y8I	1FHF	2ZLW	0.99	Sc=6.67457, min distance = 2.15827
O24984_HELPY	13711 dec	DNK_DROME Ful	65103 dGT	2VP2	2ATZ	2VP5		Sc=6.0246, min distance = 2.352649
O24984_HELPY	15993 dAT	DPO42_SULSO Fu	65103 dGT	2JEJ	2ATZ	2AGQ	0.8	Sc=6.00584, min distance = 2.44287
O24984_HELPY	5790 flox	DNK_DROME Ful	65103 dGT	2VP2	2ATZ	2VP6		Sc=6.06612, min distance = 2.30223
O24984_HELPY	64968 dTT	DPO42_SULSO Fu	65103 dGT	2JEJ	2ATZ	1S0O		Sc=6.40759, min distance = 2.13943
O24984_HELPY	65091 dCT	DPO42_SULSO Fu	65103 dGT	2JEJ	2ATZ	2ASD		Sc=6.33789, min distance = 2.51068
O24984_HELPY	656930 DD	DPO42_SULSO Fu	65103 dGT	2JEJ	2ATZ	1S9F		Sc=6.20077, min distance = 2.52485
O25511_HELPY	15942680	6PGD_LACLM Fu	5886 NADP	2IZ0	2GN8	2IZ1		Sc=6.37943, min distance = 0.56399
O25511_HELPY	440141 9i	DHB1_HUMAN Fu	5886 NADP	1QYV	2GN8	1QYW		Sc=6.44573, min distance = 2.35496
O25511_HELPY	6030 Uric	GLMU_HAEIN Fu	6031 Urid	2V0K	2GN8	2V0J	0.99	Sc=6.1836, min distance = 2.479680
O25511_HELPY	6030 Uric	O87988_BORBR	6031 Urid	2PZM	2GN8	2PZL	0.99	Sc=6.2465, min distance = 2.073174
O25511_HELPY	6030 Uric	PYRH_ECOLI Fu	6031 Urid	2BND	2GN8	2BNE	0.99	Sc=6.23826, min distance = 2.21482
O25511_HELPY	6132 Cyti	ECX1_PYRAB Fu	6031 Urid	2PNZ	2GN8	2PO2	0.83	Sc=6.30695, min distance = 2.34252
O25511_HELPY	6133 uric	PYRH_ECOLI Fu	6031 Urid	2BND	2GN8	2BNF	1	Sc=5.91815, min distance = 1.64555
O27819_METTH	445269 CI	KTHY_MYCTU Fu	164628 de	1GTV	1LVW	1W2H	0.83	Sc=6.29662, min distance = 1.14412
O27819_METTH	5789 thym	RMLA1_ECOLI Fu	164628 de	1H5T	1LVW	1H5R	0.9	Sc=6.31693, min distance = 0.72785
O27819_METTH	6031 Uric	SPSA_BACSU Fu	164628 de	1H7L	1LVW	1QGQ	0.87	Sc=5.82503, min distance = 2.06629
O28163_ARCFU	125409 be	Q09LX0_BACST	78165 MES	2EXH	2JHN	2EXJ		Sc=5.78179, min distance = 0.30181
O28442_ARCFU	445040 6-	NOS1_RAT Full	444243 FA	1TLL	2IML	2G6I		Sc=6.23948, min distance = 1.41468
O28442_ARCFU	5326566 1	BLVRB_HUMAN Fu	444243 FA	1HE4	2IML	1HE5	0.82	Sc=6.21674, min distance = 2.35829

O28480_ARCFU	439153 Di	Q9BJJ9_PLAFA I	5893 nadi	1UH5	1U1I	1V35	0.79	Sc=6.7787, min distance = 1.862552
O28480_ARCFU	445794 AD	ADHX_HUMAN Fu	5893 nadi	2FZW	1U1I	2FZE		Sc=6.64354, min distance = 1.38985
O28480_ARCFU	9547960 A	NPD_THEMA Ful	5893 nadi	2H4H	1U1I	2H59		Sc=6.64354, min distance = 2.06130
O28480_ARCFU	9547961 3	NPD_THEMA Ful	5893 nadi	2H4H	1U1I	2H59		Sc=6.70388, min distance = 1.85860
O28603_ARCFU	11987634	GLPD_ECOLI Fu	444188 CI	2QCU	1JNR	2R4E	0.97	Sc=6.45598, min distance = 1.94669
O28603_ARCFU	16750063	CSK2A_MAIZE Fu	83862 ade	1DS5	2FJB	2OXX		Sc=5.80486, min distance = 1.14729
O28603_ARCFU	24779674	CSK2A_MAIZE Fu	83862 ade	1DS5	2FJB	2PVH		Sc=6.31751, min distance = 1.45147
O28603_ARCFU	24779675	CSK2A_MAIZE Fu	83862 ade	1DS5	2FJB	2PVJ		Sc=6.46505, min distance = 1.27848
O28603_ARCFU	449465 CI	GSHR_HUMAN Fu	444188 CI	3DK9	1JNR	3GRT	0.98	Sc=6.44921, min distance = 2.18750
O28603_ARCFU	5326976 1	CSK2A_MAIZE Fu	83862 ade	1DS5	2FJB	1ZOE		Sc=5.71555, min distance = 1.58734
O28603_ARCFU	5326978 1	CSK2A_MAIZE Fu	83862 ade	1DS5	2FJB	1ZOH		Sc=5.71327, min distance = 2.29681
O28608_ARCFU	11987786	LDH_PLAFD Ful	5893 nadi	1T2D	1OMO	1T2E	0.77	Sc=6.28324, min distance = 2.61763
O28608_ARCFU	439153 Di	ADH1B_HUMAN Fu	5893 nadi	1U3U	1OMO	1DEH	0.79	Sc=6.27301, min distance = 2.13702
O28608_ARCFU	440516 CI	MDH_THETH Ful	5893 nadi	1BMD	1OMO	1BDM	0.79	Sc=6.29615, min distance = 2.54232
O29370_ARCFU	15942680	6PGD_LACLM Fu	5886 NADP	2IZ0	1JAY	2IZ1		Sc=6.19826, min distance = 2.10634
O29536_ARCFU	4369099 C	CBIF_BACME Fu	439155 Ad	1CBF	2BB3	2CBF		Sc=6.18706, min distance = 2.64445
O29536_ARCFU	4369533 C	SETD8_HUMAN Fu	439155 Ad	1ZKK	2BB3	2BQZ	0.95	Sc=6.47826, min distance = 1.98194
O29598_ARCFU	16750062	CSK2A_MAIZE Fu	33113 gam	1LP4	2OAP	2OXD		Sc=5.68392, min distance = 2.56301
O29598_ARCFU	188966 dA	HSLU_ECOLI Fu	33113 gam1E94		2OAP	1G4A	0.97	Sc=6.39986, min distance = 2.05891
O29598_ARCFU	24779674	CSK2A_MAIZE Fu	33113 gam	1LP4	2OAP	2PVH		Sc=6.18701, min distance = 2.02015
O29598_ARCFU	3547 Fasu	ROCK1_HUMAN Fu	33113 gam	2V55	2OAP	2ESM		Sc=5.90171, min distance = 1.75455
O29598_ARCFU	447052 CI	CSK2A_MAIZE Fu	33113 gam	1LP4	2OAP	1M2R		Sc=6.27619, min distance = 2.08949
O29598_ARCFU	5326739 i	GSK3B_HUMAN Fu	33113 gam	1J1B	2OAP	1Q41		Sc=6.16913, min distance = 2.41787
O29598_ARCFU	5326976 1	CSK2A_MAIZE Fu	33113 gam	1LP4	2OAP	1ZOE		Sc=5.75077, min distance = 2.27711
O29598_ARCFU	6083 aden	ENTP2_RAT Ful	33113 gam	3CJA	2OAP	3CJ7	0.97	Sc=5.85293, min distance = 2.56130
O29598_ARCFU	6083 aden	HSP71_HUMAN Fu	33113 gam	2E8A	2OAP	1XQS	0.97	Sc=5.87117, min distance = 2.24392
O29598_ARCFU	6083 aden	Q6L8F0_THETH I	33113 gam	1V25	2OAP	1V26	0.97	Sc=6.30776, min distance = 2.49588
O29598_ARCFU	60961 ade	PIM1_HUMAN Fu	33113 gam	1XR1	2OAP	1YI4	0.93	Sc=6.15612, min distance = 2.38657
O29598_ARCFU	6338562 C	MYS2_DICDI Fu	33113 gam	1MMN	2OAP	1D0Y		Sc=6.24287, min distance = 2.28150
O29598_ARCFU	6338566 C	MYS2_DICDI Fu	33113 gam	1MMN	2OAP	1D1C		Sc=6.24207, min distance = 2.26186
O29598_ARCFU	91532 AME	KIF1A_MOUSE Fu	33113 gam	1VFV	2OAP	1I6I	0.99	Sc=6.0424, min distance = 2.190737
O33253_MYCTU	16741210	MTTA_THEAQ Fu	34756 Acy	2ADM	1I9G	2JG3		Sc=6.46595, min distance = 2.42833
O33253_MYCTU	4369234 C	COMT_RAT Full=	34756 Acy	2CL5	1I9G	1JR4		Sc=5.89033, min distance = 2.22482
O33253_MYCTU	445762 5'	MTTA_THEAQ Fu	34756 Acy	2ADM	1I9G	2IH2	0.93	Sc=6.35058, min distance = 2.50507
O33253_MYCTU	445971 CI	COMT_RAT Full=	34756 Acy	2CL5	1I9G	1H1D	0.92	Sc=6.56065, min distance = 2.57110
O33253_MYCTU	5326531 C	CHOMT_MEDSA Fu	34756 Acy	1FPQ	1I9G	1FP1	0.92	Sc=6.55909, min distance = 2.02246
O33253_MYCTU	5327118 5	Q70GK9_STRCT I	34756 Acy	1RQP	1I9G	2C2W	0.81	Sc=5.73796, min distance = 1.66740

O33705_PSEPA	97536 Hex	GSTP1_MOUSE Fu	124886 gl	1GSY	1F2E	2GLR	0.9	Sc=6.55288, min distance = 1.18238
O33838_THEMA	95259 L-x	XYLA_STRRU Fu	79025 alp	1XIF	1GJW	1XIC		Sc=5.6826, min distance = 2.233586
O33839_THEMA	188966 dA	PAPS1_HUMAN Fu	6022 Aden	1X6V	1XJK	2PEZ	0.98	Sc=6.0323, min distance = 1.979612
O33839_THEMA	33113 gam	PRIM_BPT7 Ful	64968 dTT	1CR1	1XJE	1E0J		Sc=5.76812, min distance = 2.00733
O33839_THEMA	444335 GT	PAP2_VACCW Fu	8977 1dar	1JTF	1XJE	1V39	0.99	Sc=6.48507, min distance = 2.21581
O33839_THEMA	444564 AD	BIOD_ECOLI Fu	6022 Aden	1DAD	1XJK	1BS1	0.91	Sc=6.01971, min distance = 1.81410
O33839_THEMA	446696 do	KCY_ECOLI Ful	6132 Cyti	2CMK	1XJN	1KDT	0.88	Sc=6.06186, min distance = 2.17791
O33839_THEMA	447979 5G	KGUA_ECOLI Fu	8977 1dar	2AN9	1XJE	2ANB		Sc=6.38455, min distance = 2.43367
O33839_THEMA	4565 1h1r	CDK2_HUMAN Fu	6022 Aden	1GY3	1XJK	1H1R		Sc=5.79863, min distance = 2.14219
O33839_THEMA	5327148 C	IPKA_RABIT Fu	6022 Aden	1JBP	1XJK	2ERZ		Sc=6.1096, min distance = 1.972971
O33839_THEMA	6030 Urid	O87988_BORBR	6031 Urid	2PZM	1XJG	2PZL	0.99	Sc=5.80407, min distance = 2.22533
O33839_THEMA	6030 Urid	PYRH_ECOLI Fu	6031 Urid	2BND	1XJG	2BNE	0.99	Sc=5.80767, min distance = 2.60724
O33839_THEMA	6083 aden	NDK_PYRHO Ful	8977 1dar	2DXE	1XJE	2DXD	0.79	Sc=6.34967, min distance = 1.85832
O33839_THEMA	6083 aden	PSPF_ECOLI Fu	6022 Aden	2C98	1XJK	2VII	0.99	Sc=5.94333, min distance = 2.26669
O33839_THEMA	6083 aden	PURP_METJA Fu	6022 Aden	2R7N	1XJK	2R7M	0.99	Sc=5.86549, min distance = 1.65241
O33839_THEMA	6131 cyti	KCY_DICDI Ful	6031 Urid	4UKD	1XJG	1QF9	0.82	Sc=5.61005, min distance = 2.92893
O33839_THEMA	65091 dCT	DPO42_SULSO Fu	15993 dAT	2AGQ	1XJN	2ASD		Sc=5.67273, min distance = 2.12176
O33839_THEMA	656930 DD	DPO42_SULSO Fu	64968 dTT	1S0O	1XJE	1S9F		Sc=6.20348, min distance = 2.30206
O33839_THEMA	6804 guan	EF1A_YEAST Fu	8977 1dar	2B7B	1XJE	1G7C	0.99	Sc=6.38867, min distance = 2.46777
O33839_THEMA	6804 guan	GBP1_HUMAN Fu	8977 1dar	2B92	1XJE	2B8W	0.99	Sc=6.37499, min distance = 2.04169
O33839_THEMA	6804 guan	NDK_PYRHO Ful	6022 Aden	2DYA	1XJK	2DXF	0.8	Sc=5.91324, min distance = 1.47685
O33839_THEMA	6804 guan	NDK_PYRHO Ful	8977 1dar	2DXE	1XJE	2DXF	0.99	Sc=6.37789, min distance = 2.22011
O33839_THEMA	6804 guan	TGM3_HUMAN Fu	8977 1dar	1VJJ	1XJE	1SGX	0.99	Sc=6.37312, min distance = 1.84386
O33839_THEMA	6852187 2	O57883_PYRHO	6022 Aden	1WNL	1XJK	2DTH	0.89	Sc=6.08666, min distance = 1.21891
O33897_RHOMR	14626249	ACHP_LYMST Fu	23831 HEP	1UX2	1H0B	2ZJU		Sc=6.03171, min distance = 1.01911
O33897_RHOMR	445999 CI	GUN5_BACAG Fu	64689 bet	1W3L	2BWA	1H5V	0.77	Sc=5.65237, min distance = 1.82569
O33897_RHOMR	445999 CI	Q79G13_MYCTU	64689 bet	1UP0	2BWA	1UOZ	0.77	Sc=5.66645, min distance = 1.65330
O33897_RHOMR	447607 1c	Q79G13_MYCTU	64689 bet	1UP0	2BWA	1UP2		Sc=5.72381, min distance = 2.09225
O44934_LOLPE	1540 1h1q	CDK2_HUMAN Fu	6022 Aden	1GY3	2OY6	1H1Q		Sc=6.35496, min distance = 1.86870
O44934_LOLPE	23653515	CDK2_HUMAN Fu	6022 Aden	1GY3	2OY6	2R3F		Sc=5.9833, min distance = 2.082677
O44934_LOLPE	23653519	CDK2_HUMAN Fu	6022 Aden	1GY3	2OY6	2R3K		Sc=6.20519, min distance = 2.01762
O44934_LOLPE	23653521	CDK2_HUMAN Fu	6022 Aden	1GY3	2OY6	2R3M		Sc=6.35424, min distance = 2.02414
O44934_LOLPE	2608 1jsv	CDK2_HUMAN Fu	6022 Aden	1GY3	2OY6	1JSV		Sc=5.90363, min distance = 2.14113
O44934_LOLPE	445966 O6	CDK2_HUMAN Fu	6022 Aden	1GY3	2OY6	1H0V		Sc=6.1235, min distance = 2.223105
O44934_LOLPE	447004 CI	MYS2_DICDI Fu	6022 Aden	1VOM	2OY6	1LVK		Sc=6.47838, min distance = 2.07647
O44934_LOLPE	447916 ac	RIO1_ARCFU Fu	6022 Aden	1ZTH	2OY6	1ZTF	0.95	Sc=6.21951, min distance = 2.42162
O44934_LOLPE	447955 1p	CDK2_HUMAN Fu	6022 Aden	1GY3	2OY6	1PXI		Sc=5.68534, min distance = 1.70060

O44934_LOLPE	4565 1h1r	CDK2_HUMAN Fu	6022 Aden	1GY3	2OY6	1H1R		Sc=6.34032, min distance = 1.900715
O44934_LOLPE	5957 Aden	BIOD_ECOLI Fu	6022 Aden	1DAD	2OY6	1A82	0.99	Sc=6.50401, min distance = 2.191740
O44934_LOLPE	5957 Aden	HSLU_ECOLI Fu	6022 Aden	1HQY	2OY6	1DO0	0.99	Sc=6.50958, min distance = 2.325332
O44934_LOLPE	6083 aden	PSPF_ECOLI Fu	6022 Aden	2C98	2OY6	2VII	0.99	Sc=6.3787, min distance = 2.137039
O44934_LOLPE	6852187 2	O57883_PYRHO	6022 Aden	1WNL	2OY6	2DTH	0.89	Sc=6.08666, min distance = 1.594485
O44934_LOLPE	6852201 2	CDK2_HUMAN Fu	6022 Aden	1GY3	2OY6	2G9X		Sc=6.25504, min distance = 2.032605
O44934_LOLPE	72194 2-C	ENPL_CANFA Fu	6022 Aden	1TC6	2OY6	1QYE	0.89	Sc=6.10955, min distance = 2.222901
O44934_LOLPE	91532 AMP	BIOD_ECOLI Fu	6022 Aden	1DAD	2OY6	1DAG	0.99	Sc=6.53085, min distance = 2.005901
O49908_TOBAC	501640 N-	GLCM_HUMAN Fu	81462 Bis	2V3F	1RJ4	2V3E		Sc=6.04285, min distance = 1.871108
O50095_PYRHO	11987786	LDH_PLAFD Ful	5893 nadi	1T2D	1WWK	1T2E	0.77	Sc=6.02082, min distance = 2.628470
O50095_PYRHO	123927 A3	DAPB_ECOLI Fu	5893 nadi	1DRU	1WWK	1DRV	0.97	Sc=6.74725, min distance = 1.838461
O50095_PYRHO	4369002 C	LDHA_PIG Full=	5893 nadi	9LDB	1WWK	9LDT	0.77	Sc=6.75075, min distance = 2.517637
O50095_PYRHO	439153 Di	ADH1E_HORSE Fu	5893 nadi	1MGO	1WWK	2JHF	0.79	Sc=6.74573, min distance = 2.460309
O50095_PYRHO	439153 Di	CTBP1_RAT Ful	5893 nadi	1HKU	1WWK	1HL3	0.79	Sc=6.74741, min distance = 2.217711
O50095_PYRHO	445794 AD	ADHX_HUMAN Fu	5893 nadi	2FZW	1WWK	2FZE		Sc=6.5804, min distance = 2.9359751
O50095_PYRHO	446288 NA	G3P_PALVE Ful	5893 nadi	1DSS	1WWK	1IHX		Sc=6.80709, min distance = 1.055985
O50095_PYRHO	6102709 A	LDH_PLAFD Ful	5893 nadi	1T2D	1WWK	2A94	0.88	Sc=6.436, min distance = 2.8080153
O50604_STAAU	23831 HEE	GSTP1_HUMAN Fu	78165 MES	2A2R	1PVL	1PGT	0.81	Sc=5.67436, min distance = 1.574292
O51052_BORBU	439427 Ro	CGGR_BACSU Fu	10267 Har	3BXF	2F48	3BXG	0.87	Sc=5.99708, min distance = 2.008948
O52140_ECOLX	72194 2-C	ENPL_CANFA Fu	6022 Aden	1TC6	2OBM	1QYE	0.89	Sc=6.07456, min distance = 2.586131
O52942_PHOLP	11987786	LDH_PLAFD Ful	5893 nadi	1T2D	1PJC	1T2E	0.77	Sc=6.02738, min distance = 2.347636
O52942_PHOLP	4369002 C	LDHA_PIG Full=	5893 nadi	9LDB	1PJC	9LDT	0.77	Sc=6.30964, min distance = 2.307789
O52942_PHOLP	439153 Di	ADH1B_HUMAN Fu	5893 nadi	1U3U	1PJC	1DEH	0.79	Sc=6.75794, min distance = 2.361614
O52942_PHOLP	439153 Di	ADH1E_HORSE Fu	5893 nadi	1MGO	1PJC	2JHF	0.79	Sc=6.75312, min distance = 2.565673
O52942_PHOLP	439153 Di	CTBP1_RAT Ful	5893 nadi	1HKU	1PJC	1HL3	0.79	Sc=6.75332, min distance = 2.231392
O52942_PHOLP	440516 CI	MDH_THETH Ful	5893 nadi	1BMD	1PJC	1BDM	0.79	Sc=6.03188, min distance = 2.116063
O52942_PHOLP	445794 AD	ADHX_HUMAN Fu	5893 nadi	2FZW	1PJC	2FZE		Sc=5.8744, min distance = 2.5275964
O52942_PHOLP	5957 Aden	MAOM_HUMAN Fu	5893 nadi	1PJ3	1PJC	1GZ4		Sc=6.05135, min distance = 1.564771
O53355_MYCTU	16740985	FRDA_SHEFR Fu	444188 CI	1M64	1XDI	1E39	0.93	Sc=6.87078, min distance = 2.070886
O53355_MYCTU	23631921	LSD1_HUMAN Fu	444188 CI	2DW4	1XDI	2Z3Y	0.91	Sc=6.49732, min distance = 2.186372
O53355_MYCTU	444502 CI	FRDA_SHEFR Fu	444188 CI	1M64	1XDI	1QJD	0.98	Sc=6.87405, min distance = 2.305604
O53355_MYCTU	444502 CI	TYTR_TRYCR Fu	444188 CI	1BZL	1XDI	1GXF	0.98	Sc=6.87274, min distance = 2.376341
O53355_MYCTU	448054 CI	FRDA_SHEFR Fu	444188 CI	1M64	1XDI	1Y0P	0.97	Sc=6.87222, min distance = 2.151271
O53355_MYCTU	449465 CI	GSHR_HUMAN Fu	444188 CI	3DK9	1XDI	3GRT	0.98	Sc=6.87246, min distance = 2.571624
O53512_MYCTU	444348 PE	KDSA_AQUAE Fu	1005 phos	2NWR	2B7O	2NXG		Sc=5.6094, min distance = 2.4846281
O53512_MYCTU	446060 2-	ENO1_YEAST Fu	1005 phos	1ONE	2B7O	2AL1		Sc=5.63198, min distance = 1.573767
O57693_THETE	24180721	PIM1_HUMAN Fu	6083 aden	1YXU	1UXU	3C4E		Sc=5.97999, min distance = 2.113353

O57693_THETE	440141 9i	DHB1_HUMAN Fu]	5886 NADF	1QYV	1UXN	1QYW		Sc=5.91475, min distance = 2.39388
O57758_PYRHO	11608401	KAPCA_BOVIN Fu	5957 Aden	1Q24	1VCI	2UVY		Sc=5.72821, min distance = 2.42760
O57883_PYRHO	444564 AD	MYS2_DICDI Fu]	6022 Aden	1VOM	1WNL	1W9I	0.91	Sc=6.04258, min distance = 2.02204
O57883_PYRHO	446903 NO	AVID_CHICK Fu]	171548 bi	2JGS	2DTO	1LDO	0.99	Sc=6.17576, min distance = 2.10673
O57883_PYRHO	6083 ader	PSPF_ECOLI Fu]	6022 Aden	2C98	1WNL	2VII	0.99	Sc=5.80442, min distance = 2.01249
O57965_PYRHO	439176 Me	MTR1_RHOSH Fu]	34756 Acy	1NW5	1VE3	1EG2	0.91	Sc=6.29644, min distance = 2.12490
O57965_PYRHO	445971 CI	COMT_RAT Full	34756 Acy	2CL5	1VE3	1H1D	0.92	Sc=6.56394, min distance = 1.93047
O57965_PYRHO	5327118 5	Q70GK9_STRCT I	34756 Acy	1RQP	1VE3	2C2W	0.81	Sc=5.69793, min distance = 2.17944
O58346_PYRHO	444564 AD	BIOD_ECOLI Fu]	6022 Aden	1DAD	1ION	1BS1	0.91	Sc=6.02012, min distance = 2.40112
O58346_PYRHO	444842 CI	CDK2_HUMAN Fu]	6022 Aden	1GY3	1ION	1CKP		Sc=5.6204, min distance = 2.051064
O58346_PYRHO	445940 O6	CDK2_HUMAN Fu]	6022 Aden	1GY3	1ION	1GZ8		Sc=6.15372, min distance = 2.81533
O58346_PYRHO	445966 O6	CDK2_HUMAN Fu]	6022 Aden	1GY3	1ION	1H0V		Sc=6.25481, min distance = 2.26714
O58346_PYRHO	4565 1h1r	CDK2_HUMAN Fu]	6022 Aden	1GY3	1ION	1H1R		Sc=6.08092, min distance = 1.82636
O58346_PYRHO	5957 Ader	Q72H90_THET2 I	6022 Aden	2BEJ	1ION	2BEK	0.99	Sc=6.05572, min distance = 2.33190
O58346_PYRHO	6031 Uric	DCK_HUMAN Ful]	6022 Aden	1P5Z	1ION	2ZIA		Sc=5.90393, min distance = 2.34306
O58346_PYRHO	91532 AMF	AROK_MYCTU Fu]	6022 Aden	2IYV	1ION	1ZYU	0.99	Sc=6.07269, min distance = 2.05100
O58346_PYRHO	91532 AMF	NIFH1_AZOVI Fu	6022 Aden	1FP6	1ION	2AFK	0.99	Sc=6.09145, min distance = 2.49575
O58368_PYRHO	292661 2,	PTR1_LEIMA Fu]	445040 6-1E92		2DTT	1W0C		Sc=5.90438, min distance = 1.03398
O58403_PYRHO	1005 phos	F16PA_ECOLI Fu	311 citri	2OWZ	2DX7	2OX3		Sc=5.68502, min distance = 2.29585
O58403_PYRHO	439276 3-	GGGPS_ARCFU Fu	311 citri	2F6U	2DX7	2F6X		Sc=5.70848, min distance = 2.29132
O58403_PYRHO	51 2-Oxop	SERA_ECOLI Fu]	311 citri	2P9E	2DX7	1YBA	0.76	Sc=5.89375, min distance = 1.44673
O59000_PYRHO	188380 Ad	MCES_ENCCU Fu]	439155 Ad	1RI1	1WZN	1Z3C	0.91	Sc=6.58585, min distance = 2.49566
O59000_PYRHO	446535 CI	HNMT_HUMAN Fu]	439155 Ad	2AOT	1WZN	1JQE	0.95	Sc=6.53687, min distance = 2.72093
O59000_PYRHO	60961 ade	PIMT_PYRFU Fu]	439155 Ad	1JG1	1WZN	1JG2	0.84	Sc=6.30701, min distance = 2.61503
O59000_PYRHO	65482 sir	ERM_BACSU Ful]	439155 Ad	1QAN	1WZN	1QAQ	0.88	Sc=6.57421, min distance = 2.34920
O59000_PYRHO	65482 sir	MCES_ENCCU Fu]	439155 Ad	1RI1	1WZN	2HV9	0.88	Sc=6.11651, min distance = 2.43076
O59029_PYRHO	11987786	LDH_PLAFD Ful]	5893 nadi	1T2D	2DVM	1T2E	0.77	Sc=6.72786, min distance = 2.41764
O59029_PYRHO	125409 be	Q09LX0_BACST I	78165 MES	2EXH	2DVM	2EXJ		Sc=5.80469, min distance = 1.25052
O59029_PYRHO	439153 Di	ADH1B_HUMAN Fu	5893 nadi	1U3U	2DVM	1DEH	0.79	Sc=6.73124, min distance = 2.39866
O59029_PYRHO	440516 CI	MDH_THETH Ful]	5893 nadi	1BMD	2DVM	1BDM	0.79	Sc=6.30257, min distance = 2.41413
O59029_PYRHO	445794 AD	ADHX_HUMAN Fu]	5893 nadi	2FZW	2DVM	2FZE		Sc=6.58252, min distance = 2.51397
O59029_PYRHO	446006 CI	MDH_THETH Ful]	5893 nadi	1BMD	2DVM	2CVQ		Sc=6.11971, min distance = 2.45758
O59029_PYRHO	446288 NA	G3P_PALVE Ful]	5893 nadi	1DSS	2DVM	1IHX		Sc=6.80543, min distance = 0.64007
O59029_PYRHO	5957 Ader	KTRA_BACSU Fu]	5893 nadi	1LSU	2DVM	2HMW		Sc=5.9796, min distance = 2.131698
O59029_PYRHO	9547960 A	NPD_THEMA Ful]	5893 nadi	2H4H	2DVM	2H59		Sc=6.54052, min distance = 2.29908
O59416_PYRHO	11349402	PNMT_HUMAN Fu]	34756 Acy	2G72	2YZQ	2G71		Sc=5.72134, min distance = 1.91331
O59416_PYRHO	5326531 C	CHOMT_MEDSA Fu	34756 Acy	1FPQ	2YZQ	1FP1	0.92	Sc=5.9757, min distance = 2.059379

O59416_PYRHO	6852187 2	MOAA_STAA8 Fu	34756 Acy	2FB2	2YZQ	2FB3		0.84	Sc=6.13456, min distance = 2.37840
O59580_PYRHO	6083 aden	PYGM_RABIT Fu	8582 Inos	2QN7	2DVN	8GPB		0.79	Sc=5.96878, min distance = 1.77326
O59611_PYRHO	188380 Ad	MCES_ENCCU Fu	439155 Ad	1RI1	1WY7	1Z3C		0.91	Sc=6.57541, min distance = 2.34224
O59611_PYRHO	60961 ade	PIMT_PYRFU Fu	439155 Ad	1JG1	1WY7	1JG2		0.84	Sc=6.25289, min distance = 2.75964
O59611_PYRHO	65482 sir	MCES_ENCCU Fu	439155 Ad	1RI1	1WY7	2HV9		0.88	Sc=6.56445, min distance = 2.05906
O60990_PLAMA	10713671	POL_HV1A2 Ful	446837 1k	1KZK	2ANL	3BXS			Sc=6.35966, min distance = 2.09379
O60990_PLAMA	24883483	POL_HV1BR Ful	446837 1k	1MSM	2ANL	3BGC			Sc=6.3787, min distance = 2.096311
O60990_PLAMA	444480 1b	POL_HV1A2 Ful	446837 1k	1KZK	2ANL	1B6L			Sc=6.6454, min distance = 2.126316
O60990_PLAMA	466960 1z	POL_HV1A2 Ful	446837 1k	1KZK	2ANL	1Z1H			Sc=6.42178, min distance = 1.97875
O60990_PLAMA	64143 nel	POL_HV1BR Ful	446837 1k	1MSM	2ANL	2PYN		0.88	Sc=6.5878, min distance = 1.560322
O61233_LUMTE	11957355	CYB_BOVIN Ful	444522 HE	1BGY	1X9F	1L0L		0.81	Sc=6.66429, min distance = 2.33774
O61233_LUMTE	11957360	CYB_BOVIN Ful	444522 HE	1BGY	1X9F	1NTM		0.78	Sc=6.66332, min distance = 1.83998
O61233_LUMTE	11957361	CYB_BOVIN Ful	444522 HE	1BGY	1X9F	1NTZ		0.84	Sc=6.67552, min distance = 2.09502
O61233_LUMTE	11957363	HBA_HUMAN Ful	444522 HE	2DN1	1X9F	1RQA		0.81	Sc=6.67401, min distance = 2.03622
O61233_LUMTE	11957363	HBB_HUMAN Ful	444522 HE	2DN1	1X9F	1RQA		0.81	Sc=6.67705, min distance = 2.20577
O61233_LUMTE	11970219	CCPR_YEAST Fu	444522 HE	3E2O	1X9F	1BEM		0.87	Sc=6.65779, min distance = 2.33816
O61233_LUMTE	11970221	CCPR_YEAST Fu	444522 HE	3E2O	1X9F	1BES		0.87	Sc=6.65561, min distance = 2.42292
O61233_LUMTE	11970242	CCPR_YEAST Fu	444522 HE	3E2O	1X9F	1CPE		0.87	Sc=6.65939, min distance = 2.29627
O61233_LUMTE	126994 CI	CCPR_YEAST Fu	444522 HE	3E2O	1X9F	1Z53		0.81	Sc=6.65233, min distance = 2.42571
O61233_LUMTE	16741183	MYG_PHYCA Ful	444522 HE	1VXD	1X9F	2EKT		0.93	Sc=6.51806, min distance = 2.05254
O61233_LUMTE	444097 He	MYG_PHYCA Ful	444522 HE	1VXD	1X9F	2EVK		0.96	Sc=6.69447, min distance = 1.89497
O61233_LUMTE	447168 ZE	CCPR_YEAST Fu	444522 HE	3E2O	1X9F	1ML2		0.86	Sc=6.70783, min distance = 2.39972
O67135_AQUAE	3428304 E	HDAH_ALCSD Fu	5311 Vori	1ZZ1	1C3S	1ZZ3			Sc=5.87415, min distance = 1.02885
O67135_AQUAE	449096 1w	HDAC8_HUMAN Fu	5311 Vori	1T69	1C3S	1W22	35.53		Sc=6.29686, min distance = 2.01202
O67135_AQUAE	92249 Cou	HDAC8_HUMAN Fu	444732 tr	1T64	1C3R	2V5W	35.53		Sc=5.83052, min distance = 1.20041
O67198_AQUAE	11373270	HSP82_YEAST Fu	6022 Aden	1AMW	1NY5	2FXS			Sc=6.47829, min distance = 0.86257
O67198_AQUAE	444564 AI	BIOD_ECOLI Fu	6022 Aden	1DAD	1NY5	1BS1		0.91	Sc=5.99064, min distance = 2.68545
O67198_AQUAE	445940 O6	CDK2_HUMAN Fu	6022 Aden	1GY3	1NY5	1GZ8			Sc=6.19765, min distance = 1.87520
O67198_AQUAE	445966 O6	CDK2_HUMAN Fu	6022 Aden	1GY3	1NY5	1H0V			Sc=6.23274, min distance = 2.17496
O67198_AQUAE	447004 CI	MYS2_DICDI Fu	6022 Aden	1VOM	1NY5	1LVK			Sc=6.49162, min distance = 2.31903
O67198_AQUAE	447955 1p	CDK2_HUMAN Fu	6022 Aden	1GY3	1NY5	1PXI			Sc=5.6138, min distance = 2.052504
O67198_AQUAE	4564 1e1v	CDK2_HUMAN Fu	6022 Aden	1GY3	1NY5	1E1V			Sc=6.10404, min distance = 2.44582
O67198_AQUAE	4565 1h1r	CDK2_HUMAN Fu	6022 Aden	1GY3	1NY5	1H1R			Sc=6.40317, min distance = 1.41668
O67198_AQUAE	5957 Aden	BIOD_ECOLI Fu	6022 Aden	1DAD	1NY5	1A82		0.99	Sc=6.04338, min distance = 2.38469
O67198_AQUAE	5957 Aden	DDL_THET8 Ful	6022 Aden	2ZDH	1NY5	2ZDQ		0.99	Sc=6.0342, min distance = 1.679531
O67198_AQUAE	6083 aden	HSP71_HUMAN Fu	6022 Aden	1S3X	1NY5	1XQS		0.99	Sc=6.30021, min distance = 1.89460
O67198_AQUAE	6083 aden	PSPF_ECOLI Fu	6022 Aden	2C98	1NY5	2VII		0.99	Sc=5.91291, min distance = 2.38091

O67198_AQUAE	6338564 C	MYS2_DICDI Fu	6022 Aden	1VOM	1NY5	1D1A		Sc=5.88191, min distance = 2.10646
O67198_AQUAE	91532 AMI	BIOD_ECOLI Fu	6022 Aden	1DAD	1NY5	1DAG	0.99	Sc=6.05953, min distance = 2.61219
O67636_AQUAE	11987895	CD38_HUMAN Fu	5893 nadi	2I65	2G5C	2O3T		Sc=6.14154, min distance = 2.03034
O67636_AQUAE	169266 1,	GALE_HUMAN Fu	5893 nadi	1HZJ	2G5C	1I3K	0.79	Sc=6.3235, min distance = 2.330727
O67636_AQUAE	439153 Di	GALE_ECOLI Fu	5893 nadi	1UDC	2G5C	1UDB	0.79	Sc=6.31684, min distance = 2.26723
O67636_AQUAE	6022 Aden	Q5SI02_THET8 I	5893 nadi	2BJK	2G5C	2BJA		Sc=6.28284, min distance = 2.38397
O67636_AQUAE	9547960 A	NPD_THEMA Ful	5893 nadi	2H4H	2G5C	2H59		Sc=6.14403, min distance = 2.03642
O68195_KLEOX	440317 AT	FTSK_PSEAE Fu	6022 Aden	2IUU	2D0O	2IUT	0.99	Sc=6.46623, min distance = 2.10487
O68195_KLEOX	444564 AD	BIOD_ECOLI Fu	6022 Aden	1DAD	2D0O	1BS1	0.91	Sc=6.43242, min distance = 2.07189
O68195_KLEOX	446090 1h	NDKC_DICDI Fu	6022 Aden	1KDN	2D0O	1HIY	0.97	Sc=6.45302, min distance = 2.03977
O68195_KLEOX	447955 1p	CDK2_HUMAN Fu	6022 Aden	1GY3	2D0O	1PXI		Sc=5.74284, min distance = 2.15580
O68195_KLEOX	5957 Aden	ACT1_DROME Fu	6022 Aden	2HF3	2D0O	2HF4	0.99	Sc=6.0358, min distance = 2.144834
O68195_KLEOX	5957 Aden	ARP3_BOVIN Fu	6022 Aden	2P9I	2D0O	1TYQ	0.99	Sc=6.03682, min distance = 2.52887
O68195_KLEOX	5957 Aden	HSP7F_YEAST Fu	6022 Aden	3C7N	2D0O	3D2F	0.99	Sc=6.48384, min distance = 2.03831
O68195_KLEOX	5957 Aden	MUTS_ECOLI Fu	6022 Aden	1OH7	2D0O	1W7A	0.99	Sc=6.49236, min distance = 1.63453
O68195_KLEOX	5957 Aden	PPCK_ECOLI Fu	6022 Aden	1K3C	2D0O	2OLR	0.99	Sc=6.03402, min distance = 2.24919
O68195_KLEOX	6031 Urid	DCK_HUMAN Ful	6022 Aden	1P5Z	2D0O	2ZIA		Sc=6.34612, min distance = 2.05991
O68195_KLEOX	6083 aden	PSPF_ECOLI Fu	6022 Aden	2C98	2D0O	2VII	0.99	Sc=6.38203, min distance = 2.02872
O68195_KLEOX	6852187 2	O57883_PYRHO I	6022 Aden	1WNL	2D0O	2DTH	0.89	Sc=6.14196, min distance = 1.88195
O68195_KLEOX	91532 AMI	NIFH1_AZOVI Fu	6022 Aden	1FP6	2D0O	2AFK	0.99	Sc=6.08755, min distance = 2.11095
O69002_PSEAE	446603 CI	HBB_HUMAN Ful	444124 HE	1NQP	1SK7	1K0Y		Sc=6.31928, min distance = 2.03932
O69755_PSEAE	5326566 l	BLVRB_HUMAN Fu	444243 FA	1HE4	1T9M	1HE5	0.82	Sc=6.10886, min distance = 2.43400
O69755_PSEAE	5326566 l	Q9WZW1_THEMA I	444243 FA	1T6Y	1T9M	1S4M	0.82	Sc=6.18017, min distance = 1.90413
O70018_SHIDY	11957353	HBA_HORSE Ful	444098 HE	2D5X	2R7A	1IWH	0.83	Sc=6.71216, min distance = 2.21230
O70018_SHIDY	11957363	HBB_HUMAN Ful	444098 HE	1J40	2R7A	1RQA	0.77	Sc=6.74338, min distance = 2.02482
O70018_SHIDY	11957385	MYG_PHYCA Ful	444098 HE	1A6M	2R7A	2CMM		Sc=6.23243, min distance = 2.22618
O70018_SHIDY	16741062	MYG_PHYCA Ful	444098 HE	1A6M	2R7A	1MBN	0.84	Sc=6.70516, min distance = 2.54730
O70018_SHIDY	24883662	MYG_HORSE Ful	444098 HE	2FRF	2R7A	1YMC	0.82	Sc=6.43533, min distance = 2.17571
O76290_TRYBB	16129590	Y5224_ARATH Fu	5886 NADF	1XQ6	2C7V	2Q4B	0.99	Sc=6.78164, min distance = 1.91174
O76290_TRYBB	440141 9i	DHB1_HUMAN Fu	5886 NADF	1QYV	2C7V	1QYW		Sc=6.39694, min distance = 2.53766
O76290_TRYBB	440141 9i	G6PD_LEUME Fu	5886 NADF	1H9A	2C7V	1E7Y		Sc=5.9567, min distance = 2.387597
O76290_TRYBB	445175 DZ	DYR_HUMAN Ful	126941 me	1U72	2C7V	2DHF	0.85	Sc=5.60541, min distance = 2.31674
O76290_TRYBB	449049 TA	DYR_PNECA Ful	126941 me	3CD2	2C7V	1VJ3		Sc=6.08253, min distance = 2.45451
O76290_TRYBB	490573 Di	DYR_PNECA Ful	126941 me	3CD2	2C7V	2FZH		Sc=6.33998, min distance = 1.85757
O76745_CIMLE	11957385	MYG_PHYCA Ful	444097 He	2EVK	1YJH	2CMM		Sc=6.28707, min distance = 2.33639
O76745_CIMLE	11957385	MYG_PHYCA Ful	444098 HE	1A6M	2IMQ	2CMM		Sc=6.31993, min distance = 2.35396
O76745_CIMLE	11970222	CCPR_YEAST Fu	444098 HE	2EUT	2IMQ	1BJ9	0.77	Sc=6.07162, min distance = 2.20849

O76745_CIMLE	444522 HE	HBA_HUMAN Ful	444097 He	1RPS	1YJH	2DN1	0.96	Sc=6.78637, min distance = 2.33894
O76745_CIMLE	446406 CZ	MYG_PHYCA Ful	444098 HE	1A6M	2IMQ	1J3F		Sc=6.39916, min distance = 2.28480
O76977_STRPU	24316 cyc	CNGK1_RHILO Fu	6076 Cycl	1VP6	2PTM	3CL1	0.81	Sc=6.3485, min distance = 2.216933
O76977_STRPU	24316 cyc	HCN2_MOUSE Fu	6076 Cycl	3BPZ	2PTM	1Q3E	0.81	Sc=6.34279, min distance = 2.24658
O76977_STRPU	24316 cyc	KAP0_BOVIN Fu	6076 Cycl	1RGS	2PTM	1RL3	0.81	Sc=6.37796, min distance = 2.15504
O76977_STRPU	656966 1y	PDE4D_HUMAN Fu	6076 Cycl	2PW3	2PTM	1Y2E		Sc=6.08984, min distance = 2.38344
O76977_STRPU	6858240 C	KAP0_BOVIN Fu	6076 Cycl	1RGS	2PTM	1NE6	0.98	Sc=6.22215, min distance = 2.02101
O85726_STRCL	11349402	PNMT_HUMAN Fu	439155 Ad	2G8N	2BR5	2G71		Sc=5.63121, min distance = 2.31925
O85726_STRCL	11485425	PNMT_HUMAN Fu	439155 Ad	2G8N	2BR5	2G72		Sc=5.61689, min distance = 2.38314
O85726_STRCL	188380 Ad	MCES_ENCCU Fu	439155 Ad	1RI1	2BR5	1Z3C	0.91	Sc=6.54451, min distance = 1.94811
O85726_STRCL	4369234 C	COMT_RAT Full	34756 Acy	2CL5	2BR4	1JR4		Sc=6.20835, min distance = 2.06679
O85726_STRCL	439176 Me	MTR1_RHOSH Fu	34756 Acy	1NW5	2BR4	1EG2	0.91	Sc=6.29861, min distance = 2.47910
O85726_STRCL	445971 CI	COMT_RAT Full	34756 Acy	2CL5	2BR4	1H1D	0.92	Sc=6.54875, min distance = 2.47238
O85726_STRCL	446535 CI	HNMT_HUMAN Fu	439155 Ad	2AOT	2BR5	1JQE	0.95	Sc=6.51919, min distance = 2.28157
O85726_STRCL	60961 ade	PIMT_PYRFU Fu	439155 Ad	1JG1	2BR5	1JG2	0.84	Sc=6.27072, min distance = 2.84076
O85726_STRCL	65482 sir	ERM_BACSU Ful	439155 Ad	1QAN	2BR5	1QAQ	0.88	Sc=6.5636, min distance = 2.156407
O85726_STRCL	65482 sir	MCES_ENCCU Fu	439155 Ad	1RI1	2BR5	2HV9	0.88	Sc=6.56047, min distance = 2.01777
O85726_STRCL	65482 sir	MTR1_RHOSH Fu	439155 Ad	1NW7	2BR5	1NW6	0.88	Sc=6.50428, min distance = 1.93035
O85726_STRCL	65482 sir	MTTA_THEAQ Fu	439155 Ad	1AQI	2BR5	1AQJ	0.88	Sc=6.5636, min distance = 0.499223
O85726_STRCL	65482 sir	SETD7_HUMAN Fu	439155 Ad	2F69	2BR5	3CBP	0.88	Sc=6.55817, min distance = 1.85037
O87605_9ACTO	11957370	HMOX1_HUMAN Fu	444124 HE	1OZW	2C7X	1TWN		Sc=6.66115, min distance = 1.93691
O87605_9ACTO	11957371	HMOX1_HUMAN Fu	444124 HE	1OZW	2C7X	1TWR		Sc=6.68455, min distance = 1.93297
O87605_9ACTO	11957385	MYG_PHYCA Ful	444124 HE	1U7R	2C7X	2CMM		Sc=6.31222, min distance = 2.44938
O87605_9ACTO	16214774	CP51_MYCTU Fu	444124 HE	2CIB	2C7X	2CI0	0.95	Sc=6.68245, min distance = 2.38057
O87605_9ACTO	444095 HE	RCEL_RHOVI Fu	444124 HE	3D38	2C7X	1DXR	0.91	Sc=6.81085, min distance = 2.27624
O87605_9ACTO	444207 He	CP119_SULTO Fu	444124 HE	3B4X	2C7X	1UE8	0.99	Sc=6.805, min distance = 2.6729927
O87605_9ACTO	444522 HE	NOS3_BOVIN Fu	444124 HE	1ZZS	2C7X	2HX2	0.99	Sc=6.80259, min distance = 2.23729
O87988_BORBR	169266 1,	GALE_HUMAN Fu	5893 nadi	1HZJ	2PZM	1I3K	0.79	Sc=6.78884, min distance = 2.47259
O87988_BORBR	439153 Di	GALE_ECOLI Fu	5893 nadi	1UDC	2PZM	1UDB	0.79	Sc=6.77976, min distance = 2.35150
O87988_BORBR	439153 Di	Q9BJJ9_PLAFA	5893 nadi	1UH5	2PZM	1V35	0.79	Sc=6.46465, min distance = 2.08858
O87988_BORBR	448846 CI	HCD2_HUMAN Fu	5893 nadi	2O23	2PZM	1U7T	0.8	Sc=6.56683, min distance = 2.20434
O93715_SULSO	15942680	6PGD_LACLM Fu	5886 NADF	2IZ0	2CDC	2IZ1		Sc=6.27051, min distance = 2.10869
O93715_SULSO	16741014	CARP_YEAST Fu	64689 bet	1FQ4	2CDB	1FQ7		Sc=5.60731, min distance = 2.55850
O93715_SULSO	440141 9i	DHB1_HUMAN Fu	5886 NADF	1QYV	2CDC	1QYW		Sc=6.01996, min distance = 2.05129
O93715_SULSO	444809 Th	GUN5_BACAG Fu	64689 bet	1W3L	2CDB	1H5V	0.78	Sc=5.63307, min distance = 2.04926
O93715_SULSO	444809 Th	Q79G13_MYCTU	64689 bet	1UP0	2CDB	1UOZ	0.78	Sc=5.65587, min distance = 2.26607
O93715_SULSO	445238 O1	GUN2_THEFU Fu	64689 bet	2BOF	2CDB	2BOG	0.94	Sc=5.7139, min distance = 2.129053

O93715_SULSO	445564 4-	GUNG_CLOCE Fu	64689 bet	1GA2	2CDB	1KFG		0.88	Sc=5.80033, min distance = 0.43422
O93715_SULSO	447607 1c	GUN5_BACAG Fu	64689 bet	1W3L	2CDB	1OCQ			Sc=5.7024, min distance = 2.040322
O93968_CANBO	162636 1f	UROK_HUMAN Fu	8200 TETR	2O8U	2J6I	1FV9			Sc=5.61167, min distance = 2.56430
O96048_LUMTE	441481 ri	TRFL_BOVIN Fu	439353 be	2DWJ	2DS0	2PX1		0.93	Sc=5.61175, min distance = 2.28786
OBP_BOVIN Fu	2345 BENZ	OBP_PIG Full=(	445183 1e	1E00	1GT3	1DZM			Sc=5.77778, min distance = 2.22758
OBP_BOVIN Fu	2345 BENZ	OBP_PIG Full=(	7037 Diph	1DZP	1GT5	1DZM			Sc=5.79918, min distance = 2.67135
OBP_BOVIN Fu	8184 Unde	OBP_PIG Full=(	7037 Diph	1DZP	1GT5	1E02			Sc=5.61242, min distance = 2.47581
OBP_PIG Full=	11885 1-A	OBP_BOVIN Ful	445180 CI	1PBO	1DZJ	1GT1			Sc=5.80654, min distance = 2.15076
OCTC_MOUSE F	104904 Me	IGKC_MOUSE Fu	23831 HEP	1P7K	1XL7	2AJZ			Sc=5.79564, min distance = 1.51110
OCTC_MOUSE F	3246939 H	CACP_MOUSE Fu	10918 Car	1T7Q	1XL8	2H3W		0.8	Sc=6.12348, min distance = 2.59112
OCTC_MOUSE F	854023 Ep	Q8WSF8_APLCA	23831 HEP	2BR7	1XL7	2BYQ			Sc=5.87331, min distance = 1.61984
ODB2_BOVIN F	8582 Inos	RNAS1_BOVIN F	128882 1a	1AFK	2II5	1Z6D		0.79	Sc=5.78154, min distance = 1.99589
ODBA_HUMAN F	10364977	OXC_OXAFO Ful	124687 1r	2C31	2BFF	2JI6			Sc=6.4475, min distance = 2.307801
ODBA_HUMAN F	444421 CI	MDLC_PSEPU Fu	1132 thia	1BFD	2BFD	2FWN		0.82	Sc=6.38372, min distance = 2.35486
ODBA_HUMAN F	444421 CI	MDLC_PSEPU Fu	124687 1r	1Q6Z	2BFF	2FWN		0.95	Sc=6.4453, min distance = 2.491519
ODBA_HUMAN F	448671 2-	ILVB_YEAST Fu	1132 thia	1N0H	2BFD	1T9B			Sc=5.641, min distance = 3.0436356
ODBA_HUMAN F	448721 N3	TKT1_YEAST Fu	1132 thia	1TRK	2BFD	1TKA			Sc=6.37953, min distance = 2.10860
ODBA_HUMAN F	448722 1t	TKT1_YEAST Fu	1132 thia	1TRK	2BFD	1TKB		0.78	Sc=6.37485, min distance = 2.30436
ODBA_HUMAN F	448723 1t	TKT1_YEAST Fu	1132 thia	1TRK	2BFD	1TKC		0.77	Sc=6.09587, min distance = 2.44007
ODBA_PSEPU F	124687 1r	ODP1_ECOLI Fu	1132 thia	1L8A	1QS0	1RP7		0.79	Sc=5.95711, min distance = 2.39370
ODBA_PSEPU F	444421 CI	MDLC_PSEPU Fu	1132 thia	1BFD	1QS0	2FWN		0.82	Sc=6.38372, min distance = 2.39603
ODBA_PSEPU F	448671 2-	ILVB_YEAST Fu	1132 thia	1N0H	1QS0	1T9B			Sc=5.62866, min distance = 3.05179
ODBA_PSEPU F	448673 PE	ILVB_YEAST Fu	1132 thia	1N0H	1QS0	1T9B			Sc=5.78801, min distance = 2.35696
ODBA_PSEPU F	448721 N3	TKT1_YEAST Fu	1132 thia	1TRK	1QS0	1TKA			Sc=6.36799, min distance = 2.13269
ODBA_PSEPU F	448722 1t	TKT1_YEAST Fu	1132 thia	1TRK	1QS0	1TKB		0.78	Sc=6.37953, min distance = 2.45850
ODBA_PSEPU F	448723 1t	TKT1_YEAST Fu	1132 thia	1TRK	1QS0	1TKC		0.77	Sc=6.09293, min distance = 2.39940
ODBA_PSEPU F	6102647 C	ODBA_HUMAN Fu	1132 thia	2BFD	1QS0	1WCI		0.9	Sc=6.06876, min distance = 2.30689
ODBA_PSEPU F	6102749 T	ODBA_HUMAN Fu	1132 thia	2BFD	1QS0	2J9F		0.92	Sc=6.04785, min distance = 2.30774
ODBA_THET8 F	124687 1r	ODP1_ECOLI Fu	1132 thia	1L8A	1UMD	1RP7		0.79	Sc=6.43425, min distance = 2.16053
ODBA_THET8 F	444421 CI	MDLC_PSEPU Fu	1132 thia	1BFD	1UMD	2FWN		0.82	Sc=6.39621, min distance = 2.48965
ODBA_THET8 F	448671 2-	ILVB_YEAST Fu	1132 thia	1N0H	1UMD	1T9B			Sc=5.65267, min distance = 2.86140
ODBA_THET8 F	448673 PE	ILVB_YEAST Fu	1132 thia	1N0H	1UMD	1T9B			Sc=5.8115, min distance = 2.212751
ODBA_THET8 F	448722 1t	TKT1_YEAST Fu	1132 thia	1TRK	1UMD	1TKB		0.78	Sc=6.39179, min distance = 2.28051
ODBA_THET8 F	448723 1t	TKT1_YEAST Fu	1132 thia	1TRK	1UMD	1TKC		0.77	Sc=6.10652, min distance = 2.16352
ODBA_THET8 F	6102647 C	ODBA_HUMAN Fu	1132 thia	2BFD	1UMD	1WCI		0.9	Sc=6.09543, min distance = 2.41770
ODBA_THET8 F	6102749 T	ODBA_HUMAN Fu	1132 thia	2BFD	1UMD	2J9F		0.92	Sc=6.0624, min distance = 2.360258
ODBA_THET8 F	6102750 T	ODBA_HUMAN Fu	1132 thia	2BFD	1UMD	2BEV		0.9	Sc=6.09294, min distance = 2.36299

ODO1_ECOLI F	2519 caff	PYGM_RABIT Fu	6083 aden	8GPB	2JGD	1GFZ		Sc=5.87754, min distance = 2.093442
ODO1_ECOLI F	5327121 I	PIM1_HUMAN Fu	6083 aden	1YXU	2JGD	2C3I		Sc=6.27533, min distance = 1.251636
ODO1_ECOLI F	656964 1y	PDE4D_HUMAN F	6083 aden	1TB7	2JGD	1Y2C		Sc=5.96254, min distance = 2.238208
ODO1_ECOLI F	656966 1y	PDE4D_HUMAN F	6083 aden	1TB7	2JGD	1Y2E		Sc=6.10608, min distance = 2.122176
ODO1_ECOLI F	656969 1y	PDE4B_HUMAN F	6083 aden	1ROR	2JGD	1Y2J		Sc=6.19464, min distance = 1.988722
ODO1_ECOLI F	657135 Op	PIM1_HUMAN Fu	6083 aden	1YXU	2JGD	1YXV		Sc=5.7542, min distance = 2.659740
ODO1_ECOLI F	6804 guar	Q9SSV1_NICGU	6083 aden	1VD1	2JGD	1VCZ	0.8	Sc=5.63529, min distance = 1.963396
ODP1_ECOLI F	13294447	DCIP_AZOBR Fu	1132 thia	2NXW	1L8A	2Q5Q		Sc=5.85758, min distance = 1.859297
ODP1_ECOLI F	4369479 2	POXB_LACPL Fu	6518182 C	2EZ9	2QTC	2EZU	0.93	Sc=6.46244, min distance = 2.272127
ODP1_ECOLI F	444421 CI	MDLC_PSEPU Fu	1132 thia	1BFD	1L8A	2FWN	0.82	Sc=6.06055, min distance = 2.611611
ODP1_ECOLI F	444421 CI	MDLC_PSEPU Fu	124687 1r	1Q6Z	1RP7	2FWN	0.95	Sc=6.38479, min distance = 2.387544
ODP1_ECOLI F	448673 PE	ILVB_YEAST Fu	1132 thia	1N0H	1L8A	1T9B		Sc=5.79537, min distance = 2.562424
ODP1_ECOLI F	448722 1t	TKT1_YEAST Fu	1132 thia	1TRK	1L8A	1TKB	0.78	Sc=6.03791, min distance = 2.277984
ODP1_ECOLI F	448723 1t	TKT1_YEAST Fu	1132 thia	1TRK	1L8A	1TKC	0.77	Sc=5.98952, min distance = 1.892726
ODP1_ECOLI F	6518187 2	POXB_LACPL Fu	6518182 C	2EZ9	2QTC	2EZ8	0.91	Sc=6.06954, min distance = 2.245312
ODPA_BACST F	445886 CI	TKT1_YEAST Fu	1132 thia	1TRK	1W85	1GPU	0.91	Sc=5.75866, min distance = 2.077731
ODPA_BACST F	448671 2-	ILVB_YEAST Fu	1132 thia	1N0H	1W85	1T9B		Sc=5.67526, min distance = 2.919200
ODPA_BACST F	448723 1t	TKT1_YEAST Fu	1132 thia	1TRK	1W85	1TKC	0.77	Sc=6.11231, min distance = 2.041347
ODPA_BACST F	6102749 T	ODBA_HUMAN Fu	1132 thia	2BFD	1W85	2J9F	0.92	Sc=6.07374, min distance = 2.083730
ODPA_BACST F	6102750 T	ODBA_HUMAN Fu	1132 thia	2BFD	1W85	2BEV	0.9	Sc=6.0993, min distance = 2.110750
ODPA_BACST F	6102751 T	ODBA_HUMAN Fu	1132 thia	2BFD	1W85	2BEW	0.76	Sc=6.14544, min distance = 2.145643
ODPA_BACST F	6518187 2	POXB_LACPL Fu	1132 thia	2EZ4	1W85	2EZ8	0.87	Sc=5.77695, min distance = 2.096644
ODPA_HUMAN F	124687 1r	ODP1_ECOLI Fu	1132 thia	1L8A	2OZL	1RP7	0.79	Sc=5.95148, min distance = 2.211390
ODPA_HUMAN F	444421 CI	MDLC_PSEPU Fu	1132 thia	1BFD	2OZL	2FWN	0.82	Sc=6.34029, min distance = 2.607460
ODPA_HUMAN F	448671 2-	ILVB_YEAST Fu	1132 thia	1N0H	2OZL	1T9B		Sc=5.64395, min distance = 3.032617
ODPA_HUMAN F	448673 PE	ILVB_YEAST Fu	1132 thia	1N0H	2OZL	1T9B		Sc=5.78502, min distance = 2.379817
ODPA_HUMAN F	448721 N3	TKT1_YEAST Fu	1132 thia	1TRK	2OZL	1TKA		Sc=6.31728, min distance = 2.477555
ODPA_HUMAN F	448722 1t	TKT1_YEAST Fu	1132 thia	1TRK	2OZL	1TKB	0.78	Sc=6.36179, min distance = 2.560856
ODPA_HUMAN F	448723 1t	TKT1_YEAST Fu	1132 thia	1TRK	2OZL	1TKC	0.77	Sc=6.03791, min distance = 2.522050
ODPA_HUMAN F	6518187 2	POXB_LACPL Fu	1132 thia	2EZ4	2OZL	2EZ8	0.87	Sc=5.7398, min distance = 2.335287
OGA_CLOPE Fu	445948 D-	Q9HYN5_PSEAE	82313 1rd	1W8H	2J7M	1OXC		Sc=5.69574, min distance = 1.789319
OGA_CLOPE Fu	446578 1r	Q9HYN5_PSEAE	82313 1rd	1W8H	2J7M	2JDP		Sc=5.68392, min distance = 1.661836
OLED_STRAT F	6030 Uric	O87988_BORBR	6031 Urid	2PZM	2IYF	2PZL	0.99	Sc=5.8633, min distance = 2.162748
OLED_STRAT F	6030 Uric	PYRH_ECOLI Fu	6031 Urid	2BND	2IYF	2BNE	0.99	Sc=5.92723, min distance = 2.201156
OLED_STRAT F	6132 Cyti	ECX1_PYRAB Fu	6031 Urid	2PNZ	2IYF	2PO2	0.83	Sc=6.30554, min distance = 1.980498
OMPF_ECOLI F	447559 1d	SQHC_ALIAC Fu	5414 Octy	2SQC	2ZFG	1O6Q		Sc=5.79727, min distance = 2.027838
OMPG_ECOLI F	11987742	LHA4_RHOAC Fu	444279 1s	2FKW	2IWV	1NKZ		Sc=6.32315, min distance = 0.961420

OMPG_ECOLI Fu	155448 B-	GLPG_ECOLI Ful	444279 1s	2IRV	2IWV	3B44			Sc=6.34766, min distance = 0.578968
OMPG_ECOLI Fu	448339 ZI	RCEH_RHOSH Ful	444279 1s	1RZH	2IWV	1RG5			Sc=5.8749, min distance = 0.8938909
OMPG_ECOLI Fu	448339 ZI	RCEM_RHOSH Ful	444279 1s	1RZH	2IWV	1RG5			Sc=5.86387, min distance = 0.712054
OMPG_ECOLI Fu	657082 GA	LEG3_HUMAN Ful	64689 bet	2NMO	2IWW	1KJR		0.75	Sc=5.71155, min distance = 1.469759
OPR1_ARATH Fu	16741044	PYRDA_LACLC Fu	444243 FA	1JUB	1VJI	1JRB			Sc=5.6813, min distance = 2.5547267
OPR1_ARATH Fu	446995 FM	CYB2_YEAST Ful	444243 FA	1KBI	1VJI	1LTD		0.77	Sc=5.86563, min distance = 2.133939
OPR1_ARATH Fu	448076 CI	CYB2_YEAST Ful	444243 FA	1KBI	1VJI	1SZG		0.76	Sc=5.87264, min distance = 1.761400
OPR1_SOLLC Fu	16741044	PYRDA_LACLC Fu	444243 FA	1JUB	1ICP	1JRB			Sc=5.87681, min distance = 2.586081
OPR1_SOLLC Fu	446995 FM	CYB2_YEAST Ful	444243 FA	1KBI	1ICP	1LTD		0.77	Sc=6.16518, min distance = 2.224179
OPR1_SOLLC Fu	448076 CI	CYB2_YEAST Ful	444243 FA	1KBI	1ICP	1SZG		0.76	Sc=6.17951, min distance = 1.996837
OPR3_ARATH Fu	16741044	PYRDA_LACLC Fu	444243 FA	1JUB	1Q45	1JRB			Sc=5.90516, min distance = 2.481018
OPR3_ARATH Fu	446995 FM	CYB2_YEAST Ful	444243 FA	1KBI	1Q45	1LTD		0.77	Sc=6.19828, min distance = 2.437253
OPR3_ARATH Fu	448076 CI	CYB2_YEAST Ful	444243 FA	1KBI	1Q45	1SZG		0.76	Sc=6.21027, min distance = 1.859185
OPR3_SOLLC Fu	16741044	PYRDA_LACLC Fu	444243 FA	1JUB	2HSA	1JRB			Sc=5.88513, min distance = 2.547486
OPR3_SOLLC Fu	446995 FM	CYB2_YEAST Ful	444243 FA	1KBI	2HSA	1LTD		0.77	Sc=6.17843, min distance = 2.563910
OPSD_BOVIN Fu	79025 alp	BACR2_HALS2 Fu	155448 B-	1VGO	1L9H	2EI4		0.85	Sc=5.89959, min distance = 1.316050
ORN_ECOLI Ful	12136 Ber	POL_HV1N5 Ful	311 citri	2GON	1YTA	9HVP			Sc=5.64876, min distance = 0.378343
ORN_ECOLI Ful	445496 TE	A2NHM3_MOUSE I	311 citri	1ZEA	1YTA	1F3D			Sc=5.90487, min distance = 0.568022
OTSA_ECOLI Fu	439236 ri	ALGC_PSEAE Ful	439284 Rc	1P5G	1GZ5	2H4L		0.87	Sc=5.71244, min distance = 1.962024
OTSA_ECOLI Fu	440102 2h	ALGC_PSEAE Ful	439284 Rc	1P5G	1GZ5	2H5A		0.94	Sc=5.80344, min distance = 2.138997
OTSA_ECOLI Fu	6030 Uric	GLMU_HAEIN Ful	6031 Urid	2V0K	1GZ5	2V0J		0.99	Sc=6.2709, min distance = 1.9040286
OTSA_ECOLI Fu	6030 Uric	O87988_BORBR I	6031 Urid	2PZM	1GZ5	2PZL		0.99	Sc=6.26061, min distance = 1.997166
OTSA_ECOLI Fu	6030 Uric	PYRH_ECOLI Ful	6031 Urid	2BND	1GZ5	2BNE		0.99	Sc=6.25827, min distance = 2.119613
OTSA_ECOLI Fu	6132 Cyti	ECX1_PYRAB Ful	6031 Urid	2PNZ	1GZ5	2PO2		0.83	Sc=6.32896, min distance = 2.151322
OXC_ECOLI Ful	10364977	OXC_OXAFO Ful	1132 thia	2JI8	2Q28	2JI6	54.59	0.76	Sc=6.45782, min distance = 2.530295
OXC_ECOLI Ful	10364977	OXC_OXAFO Ful	6022 Aden	2C31	2Q28	2JI6	54.59		Sc=6.47598, min distance = 2.151390
OXC_ECOLI Ful	11966123	OXC_OXAFO Ful	6022 Aden	2C31	2Q28	2JI6	54.59	0.81	Sc=6.29912, min distance = 1.917232
OXC_ECOLI Ful	13294447	DCIP_AZOBR Ful	1132 thia	2NXW	2Q28	2Q5Q	22.39		Sc=6.10608, min distance = 0.352320
OXC_ECOLI Ful	3082032 f	OXC_OXAFO Ful	6022 Aden	2C31	2Q28	2JI8	54.59	0.82	Sc=6.72992, min distance = 2.429099
OXC_ECOLI Ful	36735 Gpp	PARM_ECOLX Ful	6022 Aden	1MWM	2Q28	2ZGZ		0.79	Sc=6.53076, min distance = 1.939288
OXC_ECOLI Ful	444421 CI	MDLC_PSEPU Ful	1132 thia	1BFD	2Q28	2FWN	23.16	0.82	Sc=6.42205, min distance = 2.537262
OXC_ECOLI Ful	445886 CI	TKT1_YEAST Ful	1132 thia	1TRK	2Q28	1GPU		0.91	Sc=6.5414, min distance = 2.1528306
OXC_ECOLI Ful	447955 1p	CDK2_HUMAN Ful	6022 Aden	1GY3	2Q28	1PXI			Sc=5.8077, min distance = 2.2092408
OXC_ECOLI Ful	448671 2-	ILVB_YEAST Ful	1132 thia	1N0H	2Q28	1T9B	24.91		Sc=5.81328, min distance = 2.410273
OXC_ECOLI Ful	448673 PE	ILVB_YEAST Ful	1132 thia	1N0H	2Q28	1T9B	24.91		Sc=5.87464, min distance = 2.515729
OXC_ECOLI Ful	448721 N3	TKT1_YEAST Ful	1132 thia	1TRK	2Q28	1TKA			Sc=6.43819, min distance = 2.257048
OXC_ECOLI Ful	448723 1t	TKT1_YEAST Ful	1132 thia	1TRK	2Q28	1TKC		0.77	Sc=6.46944, min distance = 2.200702

OXC_ECOLI Fu	6518182 C	ODP1_ECOLI Fu	1132 thia	1L8A	2Q28	2QTC		0.94	Sc=6.26647, min distance = 1.56114(
OXC_ECOLI Fu	8582 Inos	RNAS1_BOVIN Fu	128882 1a	1AFK	2Q29	1Z6D		0.79	Sc=6.30377, min distance = 2.315942
OXC_OXAFO Fu	128882 1a	OXC_ECOLI Ful	1132 thia	2Q28	2JI8	2Q29	54.59		Sc=6.41627, min distance = 2.37671(
OXC_OXAFO Fu	128882 1a	OXC_ECOLI Ful	6022 Aden	2Q28	2C31	2Q29	54.59	0.99	Sc=6.02389, min distance = 2.17340(
OXC_OXAFO Fu	36735 Gpp	PARM_ECOLX Fu	6022 Aden	1MWM	2C31	2ZGZ		0.79	Sc=5.80034, min distance = 2.014722
OXC_OXAFO Fu	444421 CI	MDLC_PSEPU Fu	1132 thia	1BFD	2JI8	2FWN	23.09	0.82	Sc=6.41733, min distance = 2.34649(
OXC_OXAFO Fu	444421 CI	MDLC_PSEPU Fu	124687 1r	1Q6Z	2C31	2FWN	23.09	0.95	Sc=6.41226, min distance = 2.442562
OXC_OXAFO Fu	444564 AD	BIOD_ECOLI Fu	6022 Aden	1DAD	2C31	1BS1		0.91	Sc=6.4803, min distance = 2.2131994
OXC_OXAFO Fu	445886 CI	TKT1_YEAST Fu	1132 thia	1TRK	2JI8	1GPU		0.91	Sc=6.52861, min distance = 2.125439
OXC_OXAFO Fu	447955 1p	CDK2_HUMAN Fu	6022 Aden	1GY3	2C31	1PXI			Sc=5.79518, min distance = 2.148353
OXC_OXAFO Fu	448671 2-	ILVB_YEAST Fu	1132 thia	1N0H	2JI8	1T9B	25.26		Sc=5.80014, min distance = 2.285457
OXC_OXAFO Fu	448673 PE	ILVB_YEAST Fu	1132 thia	1N0H	2JI8	1T9B	25.26		Sc=5.86456, min distance = 2.480023
OXC_OXAFO Fu	448721 N3	TKT1_YEAST Fu	1132 thia	1TRK	2JI8	1TKA			Sc=6.43819, min distance = 2.152646
OXC_OXAFO Fu	448723 1t	TKT1_YEAST Fu	1132 thia	1TRK	2JI8	1TKC		0.77	Sc=6.46544, min distance = 2.244479
OXC_OXAFO Fu	5957 Aden	BIOD_ECOLI Fu	6022 Aden	1DAD	2C31	1A82		0.99	Sc=6.50735, min distance = 2.049907
OXC_OXAFO Fu	6102751 T	ODBA_HUMAN Fu	124687 1r	2BFF	2C31	2BEW			Sc=6.04101, min distance = 2.066987
OXC_OXAFO Fu	8977 1dar	PARM_ECOLX Fu	6022 Aden	1MWM	2C31	2ZGY		0.8	Sc=6.4911, min distance = 1.9087197
OXDA_HUMAN Fu	16740985	FRDA_SHEFR Fu	444188 CI	1M64	3CUK	1E39		0.93	Sc=6.44027, min distance = 1.834626
OXDA_HUMAN Fu	16741253	LSD1_HUMAN Fu	444188 CI	2DW4	3CUK	2UXN		0.95	Sc=6.44067, min distance = 2.360364
OXDA_HUMAN Fu	16750117	LSD1_HUMAN Fu	444188 CI	2DW4	3CUK	2UXX		0.88	Sc=6.94515, min distance = 2.137023
OXDA_HUMAN Fu	444502 CI	FRDA_SHEFR Fu	444188 CI	1M64	3CUK	1QJD		0.98	Sc=6.44098, min distance = 1.95238(
OXDA_HUMAN Fu	444502 CI	TYTR_TRYCR Fu	444188 CI	1BZL	3CUK	1GXF		0.98	Sc=6.44346, min distance = 2.074503
OXDA_HUMAN Fu	445794 AD	PHHY_PSEFL Fu	444188 CI	1PBE	3CUK	2PHH			Sc=6.65859, min distance = 1.972813
OXDA_HUMAN Fu	6420174 C	O28603_ARCFU I	444188 CI	1JNR	3CUK	2FJB		0.92	Sc=6.4506, min distance = 2.1656615
OXDA_HUMAN Fu	6420174 C	O28604_ARCFU I	444188 CI	1JNR	3CUK	2FJB		0.92	Sc=6.45045, min distance = 1.804024
OXDA_PIG Ful	16740985	FRDA_SHEFR Fu	444188 CI	1M64	1VE9	1E39		0.93	Sc=6.44542, min distance = 1.597712
OXDA_PIG Ful	444502 CI	GSHR_HUMAN Fu	444188 CI	3DK9	1VE9	1BWC		0.98	Sc=6.44346, min distance = 1.829082
OXDA_PIG Ful	445794 AD	PHHY_PSEFL Fu	444188 CI	1PBE	1VE9	2PHH			Sc=6.66231, min distance = 1.946764
OXDA_PIG Ful	446013 1,	O28603_ARCFU I	444188 CI	1JNR	1VE9	1JNZ		0.94	Sc=6.44623, min distance = 2.029031
OXDA_PIG Ful	446013 1,	O28604_ARCFU I	444188 CI	1JNR	1VE9	1JNZ		0.94	Sc=6.44623, min distance = 2.103974
OXDA_PIG Ful	448054 CI	FRDA_SHEFR Fu	444188 CI	1M64	1VE9	1Y0P		0.97	Sc=6.44561, min distance = 1.587824
OXDA_PIG Ful	449077 CI	FMO1_SCHPO Fu	444188 CI	2GV8	1VE9	1VQW		1	Sc=6.16103, min distance = 1.97242(
OXDA_PIG Ful	449465 CI	GSHR_HUMAN Fu	444188 CI	3DK9	1VE9	3GRT		0.98	Sc=6.44456, min distance = 1.926325
OXDA_PIG Ful	6420174 C	O28603_ARCFU I	444188 CI	1JNR	1VE9	2FJB		0.92	Sc=6.45368, min distance = 2.137362
OXDA_PIG Ful	6420174 C	O28604_ARCFU I	444188 CI	1JNR	1VE9	2FJB		0.92	Sc=6.45355, min distance = 2.159809
OXLA_AGKHA Fu	11987634	GLPD_ECOLI Fu	444188 CI	2QCU	1TDK	2R4E		0.97	Sc=6.87267, min distance = 2.45415(
OXLA_AGKHA Fu	11987634	TYTR_TRYCR Fu	444188 CI	1BZL	1TDK	1AOG		0.97	Sc=6.87881, min distance = 2.113174

OXLA_AGKHA F	444502 CI	GSHR_HUMAN Ful	444188 CI	3DK9	1TDK	1BWC		0.98	Sc=6.87246, min distance = 2.038418
OXLA_AGKHA F	446013 1,	O28603_ARCFU I	444188 CI	1JNR	1TDK	1JNZ		0.94	Sc=6.88209, min distance = 2.482689
OXLA_AGKHA F	449465 CI	GSHR_HUMAN Ful	444188 CI	3DK9	1TDK	3GRT		0.98	Sc=6.87381, min distance = 2.375825
OXLA_AGKHA F	6420174 C	O28603_ARCFU I	444188 CI	1JNR	1TDK	2FJB		0.92	Sc=6.88531, min distance = 2.308196
OXLA_AGKHA F	6420174 C	O28604_ARCFU I	444188 CI	1JNR	1TDK	2FJB		0.92	Sc=6.88594, min distance = 2.363698
OXLA_AGKHP F	11987634	GLPD_ECOLI Ful	444188 CI	2QCU	1REO	2R4E		0.97	Sc=6.87246, min distance = 2.355107
OXLA_AGKHP F	11987634	TYTR_TRYCR Ful	444188 CI	1BZL	1REO	1AOG		0.97	Sc=6.87481, min distance = 2.044446
OXLA_AGKHP F	444502 CI	GSHR_HUMAN Ful	444188 CI	3DK9	1REO	1BWC		0.98	Sc=6.87315, min distance = 2.089193
OXLA_AGKHP F	446013 1,	O28603_ARCFU I	444188 CI	1JNR	1REO	1JNZ		0.94	Sc=6.87919, min distance = 2.436753
OXLA_AGKHP F	446013 1,	O28604_ARCFU I	444188 CI	1JNR	1REO	1JNZ		0.94	Sc=6.87862, min distance = 2.504714
OXLA_AGKHP F	447550 ph	TPIS_TRYBB Ful	311 citri	2VEK	1REO	4TIM			Sc=5.60412, min distance = 2.103804
OXLA_AGKHP F	449465 CI	GSHR_HUMAN Ful	444188 CI	3DK9	1REO	3GRT		0.98	Sc=6.43393, min distance = 2.309759
OXLA_AGKHP F	6420174 C	O28603_ARCFU I	444188 CI	1JNR	1REO	2FJB		0.92	Sc=6.88404, min distance = 2.507194
OXLA_AGKHP F	6420174 C	O28604_ARCFU I	444188 CI	1JNR	1REO	2FJB		0.92	Sc=6.88426, min distance = 2.584051
OXLA_AGKRH F	11005 Tet	ALBU_HUMAN Ful	311 citri	1TF0	1F8R	1N5U			Sc=5.79114, min distance = 2.315129
OXLA_AGKRH F	11957399	PYRB_ECOLI Ful	311 citri	1R0B	1F8R	2H3E			Sc=6.03735, min distance = 1.140151
OXLA_AGKRH F	11963950	FMS1_YEAST Ful	444188 CI	1RSG	2IID	3BI4	54.76	0.99	Sc=6.88629, min distance = 2.569738
OXLA_AGKRH F	12136 Ber	POL_HV1N5 Ful	311 citri	2GON	1F8R	9HVP			Sc=5.70505, min distance = 0.316015
OXLA_AGKRH F	16741253	LSD1_HUMAN Ful	444188 CI	2DW4	2IID	2UXN	45	0.95	Sc=6.88378, min distance = 2.287850
OXLA_AGKRH F	16750041	PYRB_ECOLI Ful	311 citri	1R0B	1F8R	2IPO			Sc=6.03149, min distance = 2.337883
OXLA_AGKRH F	439459 Ho	NIFD_KLEPN Ful	311 citri	1H1L	1F8R	1QGU		0.92	Sc=5.92596, min distance = 2.703706
OXLA_AGKRH F	439459 Ho	NIFK_KLEPN Ful	311 citri	1H1L	1F8R	1QGU		0.92	Sc=5.92596, min distance = 2.729844
OXLA_AGKRH F	449465 CI	GSHR_HUMAN Ful	444188 CI	3DK9	2IID	3GRT		0.98	Sc=6.88531, min distance = 2.278124
OXLA_AGKRH F	51 2-Oxop	SERA_ECOLI Ful	311 citri	2P9E	1F8R	1YBA		0.76	Sc=5.89311, min distance = 0.844363
OXLA_AGKRH F	5789 thym	Q9HU22_PSEAE I	311 citri	1G1L	1F8R	1G0R			Sc=6.28972, min distance = 0.718068
OXLA_AGKRH F	6420174 C	O28603_ARCFU I	444188 CI	1JNR	2IID	2FJB		0.92	Sc=6.89415, min distance = 2.130664
OXLA_AGKRH F	6420174 C	O28604_ARCFU I	444188 CI	1JNR	2IID	2FJB		0.92	Sc=6.89225, min distance = 2.207416
OXLA_AGKRH F	6961 PYRC	FUMC_ECOLI Ful	311 citri	1FUO	1F8R	1FUP			Sc=6.21101, min distance = 2.514382
OXSR1_HUMAN I	11987888	IRAK4_HUMAN F	33113 gam	2OID	2VWI	2NRU			Sc=6.5706, min distance = 0.8362954
OXSR1_HUMAN I	15993 dAT	PRIM_BPT7 Ful	33113 gam	1E0J	2VWI	1CR2		0.97	Sc=5.66451, min distance = 1.944058
OXSR1_HUMAN I	24779674	CSK2A_MAIZE F	33113 gam	1LP4	2VWI	2PVH			Sc=5.9604, min distance = 2.7975476
OXSR1_HUMAN I	24779675	CSK2A_MAIZE F	33113 gam	1LP4	2VWI	2PVJ			Sc=6.33169, min distance = 2.160731
OXSR1_HUMAN I	24779676	CSK2A_MAIZE F	33113 gam	1LP4	2VWI	2PVK			Sc=6.33512, min distance = 2.153004
OXSR1_HUMAN I	24779677	CSK2A_MAIZE F	33113 gam	1LP4	2VWI	2PVL			Sc=6.22886, min distance = 1.900324
OXSR1_HUMAN I	24779678	CSK2A_MAIZE F	33113 gam	1LP4	2VWI	2PVM			Sc=6.39569, min distance = 2.287113
OXSR1_HUMAN I	3064778 H	ROCK1_HUMAN F	33113 gam	2V55	2VWI	2ETK			Sc=6.00356, min distance = 2.507439
OXSR1_HUMAN I	3547 Fasu	ROCK1_HUMAN F	33113 gam	2V55	2VWI	2ESM			Sc=5.87289, min distance = 1.799009

OXSR1_HUMAN I	3973 nche	PIM1_HUMAN Fu	33113 gam	1XR1	2VWI	1YI3		Sc=5.80379, min distance = 2.253626
OXSR1_HUMAN I	444564 AD	MYS2_DICDI Fu	33113 gam	1MMN	2VWI	1W9I	0.91	Sc=5.81251, min distance = 2.699025
OXSR1_HUMAN I	448042 2e	ROCK1_HUMAN Fu	33113 gam	2V55	2VWI	2ETR		Sc=5.98225, min distance = 2.448016
OXSR1_HUMAN I	5326739 i	GSK3B_HUMAN Fu	33113 gam	1J1B	2VWI	1Q41		Sc=6.14251, min distance = 2.667937
OXSR1_HUMAN I	5327121 I	PIM1_HUMAN Fu	33113 gam	1XR1	2VWI	2C3I		Sc=6.17767, min distance = 2.242012
OXSR1_HUMAN I	60961 ade	PIM1_HUMAN Fu	33113 gam	1XR1	2VWI	1YI4	0.93	Sc=6.1383, min distance = 1.957571
OXSR1_HUMAN I	60961 ade	SKY1_YEAST Fu	33113 gam	1Q99	2VWI	1Q97	0.93	Sc=5.74912, min distance = 2.784441
OXSR1_HUMAN I	611002 Op	PIM1_HUMAN Fu	33113 gam	1XR1	2VWI	1YXX		Sc=6.00869, min distance = 2.172762
OXSR1_HUMAN I	91532 AMP	MURC_HAEIN Fu	33113 gam	1P3D	2VWI	1GQY	0.99	Sc=5.89871, min distance = 1.990895
OYE1_SACPS Fu	16741044	PYRDA_LACLC Fu	444243 FA	1JUB	1OYA	1JRB		Sc=5.87736, min distance = 2.573764
OYE1_SACPS Fu	446995 FM	CYB2_YEAST Fu	444243 FA	1KBI	1OYA	1LTD	0.77	Sc=6.1639, min distance = 2.391056
P152_METTH Fu	5326566 l	Q9WZW1_THEMA I	444243 FA	1T6Y	1EJE	1S4M	0.82	Sc=6.3327, min distance = 2.286553
P1_ARATH Full	440334 Ni	DAPB_ECOLI Fu	5886 NADP	1DIH	2J3I	1DRW	0.91	Sc=5.97233, min distance = 1.870189
P5CR1_HUMAN I	446050 CI	G3P_HUMAN Full	5893 nadi	1U8F	2IZZ	3GPD	0.95	Sc=6.2811, min distance = 1.255340
P5CR1_HUMAN I	6022 Aden	Q5SI02_THET8 I	5893 nadi	2BJK	2IZZ	2BJA		Sc=6.19275, min distance = 2.413917
P71278_ENTCL	23722944	TTHY_HUMAN Fu	1493 2,4-	2B15	1GVO	2QGE		Sc=5.84969, min distance = 2.199946
P71278_ENTCL	4369524 T	PRGR_HUMAN Fu	5994 prog	1A28	2ABA	1ZUC		Sc=5.96255, min distance = 2.478054
P71278_ENTCL	448734 CI	TTHY_HUMAN Fu	1493 2,4-	2B15	1GVO	1TLM		Sc=6.10787, min distance = 2.235068
P71454_LEUME	188966 dA	HSLU_ECOLI Fu	6022 Aden	1HQY	1EHI	1G4A	0.98	Sc=5.96375, min distance = 1.876075
P71454_LEUME	23653515	CDK2_HUMAN Fu	6022 Aden	1GY3	1EHI	2R3F		Sc=6.13765, min distance = 2.077695
P71454_LEUME	23653523	CDK2_HUMAN Fu	6022 Aden	1GY3	1EHI	2R3O		Sc=6.44493, min distance = 2.027656
P71454_LEUME	24864080	CDK2_HUMAN Fu	6022 Aden	1GY3	1EHI	2VTR		Sc=6.01468, min distance = 1.974950
P71454_LEUME	4369136 C	CDK2_HUMAN Fu	6022 Aden	1GY3	1EHI	1DM2		Sc=6.25549, min distance = 1.797409
P71454_LEUME	447916 ad	RIO1_ARCFU Fu	6022 Aden	1ZTH	1EHI	1ZTF	0.95	Sc=6.19256, min distance = 2.763142
P71454_LEUME	447955 1p	CDK2_HUMAN Fu	6022 Aden	1GY3	1EHI	1PXI		Sc=5.75952, min distance = 2.173071
P71454_LEUME	447967 CI	CDK2_HUMAN Fu	6022 Aden	1GY3	1EHI	1PYE		Sc=6.36335, min distance = 2.184209
P71454_LEUME	5327148 C	IPKA_RABIT Fu	6022 Aden	1JBP	1EHI	2ERZ		Sc=6.15834, min distance = 2.069354
P71454_LEUME	5687 1di8	CDK2_HUMAN Fu	6022 Aden	1GY3	1EHI	1DI8		Sc=6.24985, min distance = 2.274376
P71454_LEUME	6420140 2	CDK2_HUMAN Fu	6022 Aden	1GY3	1EHI	2C5Y		Sc=6.51886, min distance = 2.002101
P71454_LEUME	9543438 C	DDLB_ECOLI Fu	6022 Aden	1IOW	1EHI	1IOV		Sc=5.99842, min distance = 2.052342
P71454_LEUME	9543438 C	DDLB_ECOLI Fu	9543432 1	2DLN	1EHI	1IOV		Sc=5.98647, min distance = 2.762311
P71454_LEUME	9547890 I	CDK2_HUMAN Fu	6022 Aden	1GY3	1EHI	1W8C		Sc=6.27374, min distance = 2.328128
P74325_SYNY3	185698 al	LEC_PEA Full=I	5988 sucr	1OFS	1TJ5	1RIN	0.87	Sc=5.77565, min distance = 2.143239
P74325_SYNY3	445238 O1	GUN2_THEFU Fu	64689 bet	2BOF	1U2S	2BOG	0.94	Sc=5.77061, min distance = 2.200392
P74325_SYNY3	447607 1c	Q79G13_MYCTU I	64689 bet	1UP0	1U2S	1UP2		Sc=5.8821, min distance = 0.614292
P83787_MARHY	1005 phos	F16PA_ECOLI Fu	311 citri	2OWZ	1NML	2OX3		Sc=5.62674, min distance = 2.307924
P83787_MARHY	11869260	DGAL_ECOLI Fu	311 citri	2FW0	1NML	2QW1		Sc=5.83642, min distance = 2.473169

P83787_MARHY	12136 Ber	POL_HV1N5 Ful	311 citri	2GON	1NML	9HVP			Sc=5.68082, min distance = 0.804374
P83787_MARHY	126892 1t	TPIS_TRYBB Ful	311 citri	2VEK	1NML	1TSI			Sc=5.83725, min distance = 1.363103
P83787_MARHY	23831 HEE	GSK3B_HUMAN Fu	311 citri	1R0E	1NML	1H8F			Sc=5.66388, min distance = 1.986877
P83787_MARHY	439183 1i	TPIS_TRYBB Ful	311 citri	2VEK	1NML	1IIH			Sc=5.76601, min distance = 1.646751
P83787_MARHY	439276 3-	GGGPS_ARCFU Fu	311 citri	2F6U	1NML	2F6X			Sc=5.60752, min distance = 2.326547
P83787_MARHY	439276 3-	TPIS_TRYBB Ful	311 citri	2VEK	1NML	6TIM			Sc=5.66427, min distance = 1.484481
P83787_MARHY	439351 3-	AROQ_HELPY Ful	311 citri	2C4V	1NML	1J2Y			Sc=5.94793, min distance = 2.139400
P83787_MARHY	439459 Ho	NIFD_KLEPN Ful	311 citri	1H1L	1NML	1QGU		0.92	Sc=5.98895, min distance = 2.684042
P83787_MARHY	439459 Ho	NIFK_KLEPN Ful	311 citri	1H1L	1NML	1QGU		0.92	Sc=5.99413, min distance = 2.693184
P83787_MARHY	444212 tr	ACON_BOVIN Ful	311 citri	1C96	1NML	1ACO			Sc=5.94606, min distance = 2.024405
P83787_MARHY	445607 AT	ACON_BOVIN Ful	311 citri	1C96	1NML	1FGH			Sc=5.9997, min distance = 2.2441671
P83787_MARHY	447376 2-	ACON_BOVIN Ful	311 citri	1C96	1NML	1NIS			Sc=5.94331, min distance = 2.332455
P83787_MARHY	449604 NI	ACON_BOVIN Ful	311 citri	1C96	1NML	8ACN			Sc=5.90282, min distance = 2.395982
P83787_MARHY	4797 1tpw	TPIS_TRYBB Ful	311 citri	2VEK	1NML	1TTJ			Sc=5.65555, min distance = 2.162569
P83812_BACST	12389 Tet	LTC4S_HUMAN Fu	985 palmi	2UUH	1PZX	2UUI			Sc=6.04643, min distance = 1.291068
P83812_BACST	175468 12	LACB_BOVIN Ful	985 palmi	1GXA	1PZX	1BSO		0.83	Sc=5.89996, min distance = 2.468304
P83818_THETH	5326566 1	Q9WZW1_THEMA I	444243 FA	1T6Y	1USC	1S4M		0.82	Sc=6.32205, min distance = 2.575292
P83820_THETH	11957393	FAK1_HUMAN Ful	6022 Aden	1MP8	1V9O	2ETM			Sc=6.05614, min distance = 1.994032
P83820_THETH	1540 1h1q	CDK2_HUMAN Ful	6022 Aden	1GY3	1V9O	1H1Q			Sc=6.0678, min distance = 2.5213198
P83820_THETH	23653518	CDK2_HUMAN Ful	6022 Aden	1GY3	1V9O	2R3J			Sc=5.95869, min distance = 2.175909
P83820_THETH	23653519	CDK2_HUMAN Ful	6022 Aden	1GY3	1V9O	2R3K			Sc=6.08476, min distance = 2.071854
P83820_THETH	24864080	CDK2_HUMAN Ful	6022 Aden	1GY3	1V9O	2VTR			Sc=6.0335, min distance = 0.9099541
P83820_THETH	444564 AD	BIOD_ECOLI Ful	6022 Aden	1DAD	1V9O	1BS1		0.91	Sc=6.34029, min distance = 2.204778
P83820_THETH	447649 1o	CDK2_HUMAN Ful	6022 Aden	1GY3	1V9O	1OI9			Sc=6.1139, min distance = 2.0565466
P83820_THETH	447655 1o	CDK2_HUMAN Ful	6022 Aden	1GY3	1V9O	1OIU			Sc=6.01024, min distance = 2.208924
P83820_THETH	447916 ad	RIO1_ARCFU Ful	6022 Aden	1ZTH	1V9O	1ZTF		0.95	Sc=6.07993, min distance = 2.739871
P83820_THETH	4565 1h1r	CDK2_HUMAN Ful	6022 Aden	1GY3	1V9O	1H1R			Sc=5.96356, min distance = 2.309219
P83820_THETH	5327130 C	CDK2_HUMAN Ful	6022 Aden	1GY3	1V9O	2C68			Sc=5.99186, min distance = 2.242343
P83820_THETH	5957 Aden	Q53W83_THET8 I	6022 Aden	1V1A	1V9O	1V1B		0.99	Sc=5.63529, min distance = 2.662635
P83820_THETH	6031 Urid	RIR1_YEAST Ful	6022 Aden	2CVX	1V9O	2CVV			Sc=5.87264, min distance = 2.015591
P83820_THETH	9543433 T	NDKC_DICDI Ful	6022 Aden	1KDN	1V9O	1F6T			Sc=6.33043, min distance = 2.091771
P84125_THETH	11987635	ADH_DROLE Ful	5893 nadi	1SBY	1VC2	1B2L		0.76	Sc=6.5099, min distance = 1.9369909
P84125_THETH	169266 1,	GALE_HUMAN Ful	5893 nadi	1HZJ	1VC2	1I3K		0.79	Sc=6.75137, min distance = 1.948222
P84125_THETH	439153 Di	GALE_ECOLI Ful	5893 nadi	1UDC	1VC2	1UDB		0.79	Sc=6.29771, min distance = 2.013787
P84125_THETH	439153 Di	Q9BJJ9_PLAFA I	5893 nadi	1UH5	1VC2	1V35		0.79	Sc=6.74618, min distance = 1.967768
P84125_THETH	445794 AD	ADHX_HUMAN Ful	5893 nadi	2FZW	1VC2	2FZE			Sc=5.68509, min distance = 2.218487
P84125_THETH	446006 CI	MDH_THETH Ful	5893 nadi	1BMD	1VC2	2CVQ			Sc=5.95224, min distance = 2.079930

P84125_THETH	446050 CI	G3P_HUMAN Ful	5893 nadi	1U8F	1VC2	3GPD		0.95	Sc=6.71932, min distance = 2.37369
P84125_THETH	446288 NA	G3P_PALVE Ful	5893 nadi	1DSS	1VC2	1IHX			Sc=6.29237, min distance = 2.56184
P84125_THETH	447224 AE	FADB_PSEFR Fu	5893 nadi	1WDK	1VC2	1WDL			Sc=6.33962, min distance = 1.59170
P84125_THETH	6022 Ader	Q5SI02_THET8	5893 nadi	2BJK	1VC2	2BJA			Sc=6.25868, min distance = 2.39760
P84141_ARTAU	6540269 2	HYSA_STRPN Fu	446101 AC	1OJO	1RWH	2BRP			Sc=5.89059, min distance = 2.29451
P90613_TOXGO	11987786	LDH_PLAFD Ful	5893 nadi	1T2D	1PZH	1T2E		0.77	Sc=6.77256, min distance = 2.76181
P90613_TOXGO	4369002 C	LDHA_PIG Full	5893 nadi	9LDB	1PZH	9LDT		0.77	Sc=6.7712, min distance = 2.171367
P90613_TOXGO	439153 Di	ADH1E_HORSE F	5893 nadi	1MGO	1PZH	2JHF		0.79	Sc=6.75332, min distance = 2.27096
P90613_TOXGO	439153 Di	CTBP1_RAT Ful	5893 nadi	1HKU	1PZH	1HL3		0.79	Sc=6.77212, min distance = 2.09484
P90613_TOXGO	440516 CI	MDH_THETH Ful	5893 nadi	1BMD	1PZH	1BDM		0.79	Sc=6.7803, min distance = 2.476916
P90613_TOXGO	445794 AD	ADHX_HUMAN Fu	5893 nadi	2FZW	1PZH	2FZE			Sc=6.6175, min distance = 2.487247
P90613_TOXGO	6083 ader	NADE_ECOLI Fu	5893 nadi	1WXH	1PZH	1WXI			Sc=5.85293, min distance = 2.13466
P93799_VITVI	3973 nche	PIM1_HUMAN Fu	5281672 m	2O63	2IOD	1YI3			Sc=5.86387, min distance = 1.67242
P93799_VITVI	440141 9i	G6PD_LEUME Fu	5886 NADP	1H9A	2IOD	1E7Y			Sc=5.69269, min distance = 1.95297
P93799_VITVI	657135 Op	PIM1_HUMAN Fu	5281672 m	2O63	2IOD	1YXV			Sc=5.69232, min distance = 3.00714
P94182_9NOST	656964 1y	PDE4D_HUMAN F	6076 Cycl	2PW3	1YKD	1Y2C			Sc=5.91374, min distance = 2.27775
P94182_9NOST	656966 1y	PDE4D_HUMAN F	6076 Cycl	2PW3	1YKD	1Y2E			Sc=6.04966, min distance = 2.37572
P94182_9NOST	656969 1y	PDE4D_HUMAN F	6076 Cycl	2PW3	1YKD	1Y2K			Sc=6.19161, min distance = 2.16148
P94182_9NOST	6858240 C	KAP0_BOVIN Fu	6076 Cycl	1RGS	1YKD	1NE6		0.98	Sc=6.23199, min distance = 1.92000
P94692_DESAF	124687 1r	MDLC_PSEPU Fu	444421 CI	2FWN	1B0P	1Q6Z		0.95	Sc=6.51911, min distance = 2.39008
P94692_DESAF	124687 1r	OXC_OXAFO Ful	1132 thia	2JI8	2C42	2C31		0.79	Sc=6.51645, min distance = 2.13435
P94692_DESAF	24836827	BZNB_PSEFL Fu	1132 thia	2UZ1	2C42	3D7K			Sc=6.30289, min distance = 1.63276
P94692_DESAF	448671 2-	ILVB_YEAST Fu	1132 thia	1N0H	2C42	1T9B			Sc=5.98325, min distance = 2.52136
P94692_DESAF	448721 N3	TKT1_YEAST Fu	1132 thia	1TRK	2C42	1TKA			Sc=6.48198, min distance = 2.20107
P94692_DESAF	448723 1t	TKT1_YEAST Fu	1132 thia	1TRK	2C42	1TKC		0.77	Sc=6.52587, min distance = 2.04694
P94692_DESAF	6102647 C	ODBA_HUMAN Fu	1132 thia	2BFD	2C42	1WCI		0.9	Sc=6.63256, min distance = 2.08410
P94692_DESAF	6102749 T	ODBA_HUMAN Fu	1132 thia	2BFD	2C42	2J9F		0.92	Sc=6.5975, min distance = 2.138059
P94692_DESAF	6518182 C	POXB_LACPL Fu	1132 thia	2EZ4	2C42	2EZ9		0.94	Sc=6.20043, min distance = 2.09562
P94692_DESAF	6518187 2	POXB_LACPL Fu	1132 thia	2EZ4	2C42	2EZ8		0.87	Sc=6.18361, min distance = 2.04669
P95480_PSEFL	11987634	GLPD_ECOLI Fu	444188 CI	2QCU	2AQJ	2R4E		0.97	Sc=6.86598, min distance = 2.11508
P95480_PSEFL	446013 1,	O28603_ARCFU	444188 CI	1JNR	2AQJ	1JNZ		0.94	Sc=6.43559, min distance = 2.19482
P95480_PSEFL	446013 1,	O28604_ARCFU	444188 CI	1JNR	2AQJ	1JNZ		0.94	Sc=6.43436, min distance = 2.16920
P95480_PSEFL	5962 L-ly	THER_BACTH Fu	6305 L-tr	1THL	2AQJ	1KEI			Sc=5.74009, min distance = 2.24814
P95480_PSEFL	60961 ade	Q8KHZ8_NOCAE	6305 L-tr	2E4G	2AQJ	2OA1			Sc=6.29013, min distance = 2.51408
P95480_PSEFL	6140 L-ph	APX_STRGR Ful	6305 L-tr	1TF8	2AQJ	1F2P			Sc=5.85821, min distance = 2.05072
P95480_PSEFL	6140 L-ph	THER_BACTH Fu	6305 L-tr	1THL	2AQJ	1OS0			Sc=5.86884, min distance = 2.29969
P95480_PSEFL	71567 D-p	APX_STRGR Ful	6305 L-tr	1TF8	2AQJ	1TKH			Sc=5.89451, min distance = 2.12140

P95480_PSEFL	9060 D-Tr	P95481_PSEFL I	3081936 7	2V7L	2AR8	2V7K		0.9	Sc=6.13361, min distance = 2.42861
P95481_PSEFL	11957385	MYG_PHYCA Ful	444098 HE	1A6M	2V7K	2CMM			Sc=6.03775, min distance = 2.07454
P96896_MYCTU	134497 4-	APX_STRGR Ful	6305 L-tr	1TF8	2VBZ	1TF9			Sc=5.6586, min distance = 2.095563
P96896_MYCTU	6140 L-ph	APX_STRGR Ful	6305 L-tr	1TF8	2VBZ	1F2P			Sc=5.641, min distance = 2.1656823
P96896_MYCTU	71567 D-p	APX_STRGR Ful	6305 L-tr	1TF8	2VBZ	1TKH			Sc=5.77558, min distance = 2.46445
PA1L_PSEAE Fu	6027 xylo	Q9ZB17_9LACT I	439353 be	1NSX	1OKO	1MN0		0.93	Sc=5.60935, min distance = 1.83838
PA1L_PSEAE Fu	657081 CI	LEG3_HUMAN Fu	64689 bet	2NMO	2VXJ	1KJR			Sc=5.62063, min distance = 1.48792
PA25_NAJSG Fu	444205 CI	DPP4_HUMAN Fu	24139 ace	2RGU	1Y75	3BJM		0.91	Sc=5.95424, min distance = 2.31185
PA25_NAJSG Fu	445247 CI	CHLY_HEVBR Fu	24139 ace	1KQY	1Y75	1LLO			Sc=5.72905, min distance = 2.33080
PA28_DABRR Fu	177880 2'	TTHY_HUMAN Fu	3033 dicl	1DVX	1SV9	2G9K			Sc=5.61836, min distance = 2.00714
PA28_DABRR Fu	20284644	TTHY_HUMAN Fu	3033 dicl	1DVX	1SV9	2QGC			Sc=5.67325, min distance = 2.51565
PA28_DABRR Fu	23722944	TTHY_HUMAN Fu	3033 dicl	1DVX	1SV9	2QGE			Sc=5.87607, min distance = 0.81645
PA28_DABRR Fu	25011739	TTHY_HUMAN Fu	3033 dicl	1DVX	1SV9	3CN0			Sc=5.66793, min distance = 2.31171
PA28_DABRR Fu	30951 ald	PGH1_SHEEP Fu	39912 ibu	1EQG	2PWS	1HT8			Sc=5.64328, min distance = 1.95361
PA28_DABRR Fu	445302 AC	PGH1_SHEEP Fu	39912 ibu	1EQG	2PWS	1EBV			Sc=5.74321, min distance = 2.57896
PA28_DABRR Fu	4488 nifl	PA23_NAJSG Fu	2244 aspi	1OXR	1TGM	1TD7	44.78		Sc=5.90573, min distance = 2.48779
PA28_DABRR Fu	5287495 2	TTHY_HUMAN Fu	3033 dicl	1DVX	1SV9	2B77			Sc=5.88986, min distance = 2.30703
PA2_APIME Fu	446400 sE	PA2GA_HUMAN Fu	129148 Di	1POE	1POC	1J1A			Sc=6.51447, min distance = 2.26634
PA2_APIME Fu	446783 6-	PA2GA_HUMAN Fu	129148 Di	1POE	1POC	1KQU			Sc=6.4449, min distance = 2.258813
PA2_NAJNA Fu	444205 CI	DPP4_HUMAN Fu	24139 ace	2RGU	1OWS	3BJM		0.91	Sc=5.94643, min distance = 2.36164
PA2_NAJNA Fu	445247 CI	CHLY_HEVBR Fu	24139 ace	1KQY	1OWS	1LLO			Sc=5.72167, min distance = 2.38092
PA2_NAJNA Fu	446101 AC	CSLA_PEDHE Fu	24139 ace	1HM3	1OWS	1HM2		0.94	Sc=5.8728, min distance = 2.182060
PABB_ECOLI Fu	134497 4-	APX_STRGR Ful	6305 L-tr	1TF8	1K0E	1TF9			Sc=5.90272, min distance = 2.05245
PABB_ECOLI Fu	3081936 7	P95480_PSEFL I	6305 L-tr	2AQJ	1K0E	2AR8		0.9	Sc=6.17596, min distance = 2.27297
PABB_ECOLI Fu	6140 L-ph	APX_STRGR Ful	6305 L-tr	1TF8	1K0E	1F2P			Sc=5.87794, min distance = 1.93552
PABB_ECOLI Fu	71567 D-p	APX_STRGR Ful	6305 L-tr	1TF8	1K0E	1TKH			Sc=5.93597, min distance = 2.26800
PABC_ECOLI Fu	447037 ME	DGDA_BURCE Fu	445005 D-	1D7S	1I2L	1M0O		0.88	Sc=5.98439, min distance = 2.17102
PANC_MYCTU Fu	2519 caff	PYGM_RABIT Fu	6083 aden	8GPB	2A7X	1GFZ			Sc=5.9085, min distance = 2.192403
PANC_MYCTU Fu	440641 1m	O57693_THETE I	6083 aden	1UXU	2A7X	1UXR			Sc=5.96519, min distance = 2.22735
PANC_MYCTU Fu	445736 5'	SYS2_METBF Fu	5957 Aden	2CJA	2A84	2CJ9		0.87	Sc=6.50754, min distance = 1.54480
PANC_MYCTU Fu	447916 ad	RIO1_ARCFU Fu	5957 Aden	1ZP9	2A84	1ZTF		0.94	Sc=6.26491, min distance = 2.55386
PANC_MYCTU Fu	5327121 I	PIM1_HUMAN Fu	6083 aden	1YXU	2A7X	2C3I			Sc=6.31383, min distance = 0.67733
PANC_MYCTU Fu	6022 Aden	DDL_THET8 Ful	5957 Aden	2ZDQ	2A84	2ZDH		0.99	Sc=6.46179, min distance = 1.93677
PANC_MYCTU Fu	6022 Aden	MUTS_ECOLI Fu	5957 Aden	1W7A	2A84	1OH7		0.99	Sc=6.43556, min distance = 2.02432
PANC_MYCTU Fu	6022 Aden	Q94M05_9VIRU I	91557 1tm	1W48	1N2E	1W44		0.99	Sc=6.48902, min distance = 1.72398
PANC_MYCTU Fu	6022 Aden	RIO1_ARCFU Fu	5957 Aden	1ZP9	2A84	1ZTH		0.99	Sc=6.42829, min distance = 1.81711
PANC_MYCTU Fu	611002 Op	PIM1_HUMAN Fu	6083 aden	1YXU	2A7X	1YXX			Sc=6.21358, min distance = 1.57547

PANC_MYCTU Fı	6133 uric	CCA_ARCFU Ful	5957 Aden	1R8B	2A84	1R8C			Sc=6.38853, min distance = 0.79198
PANC_MYCTU Fı	65110 AIC	YL28_SCHPO Fu	6083 aden	2OOX	2A7X	2QRE		0.79	Sc=5.87921, min distance = 1.97791
PANC_MYCTU Fı	65533 cor	O57693_THETE	6083 aden	1UXU	2A7X	1UXT			Sc=5.95873, min distance = 2.13051
PANC_MYCTU Fı	656966 1y	PDE4D_HUMAN Fı	6083 aden	1TB7	2A7X	1Y2E			Sc=6.1427, min distance = 2.005080
PANC_MYCTU Fı	6830 guar	Q381M1_9TRYP	5957 Aden	2Q0D	2A84	2Q0E		0.8	Sc=6.48297, min distance = 2.06947
PANC_MYCTU Fı	91532 AMI	Q5SJV7_THET8	5957 Aden	2Z08	2A84	2Z09		0.99	Sc=6.50172, min distance = 2.10406
PANE_ECOLI Fı	15942680	6PGD_LACLM Fu	5886 NADP	2IZ0	1YJQ	2IZ1			Sc=5.8105, min distance = 2.062162
PAP1_VACCW Fı	444564 AI	MYS2_DICDI Fu	440317 AT	1MMG	2GA9	1W9I		0.91	Sc=5.85224, min distance = 2.35014
PAP1_VACCW Fı	6083 ader	SRC_CHICK Ful	440317 AT	3DQW	2GA9	3DQX		0.97	Sc=6.27355, min distance = 2.07698
PAP2_VACCW Fı	188380 Ac	MCES_ENCCU Fu	439155 Ad	1RI1	1V39	1Z3C		0.91	Sc=6.58684, min distance = 2.27540
PAP2_VACCW Fı	445971 CI	COMT_RAT Full	34756 Acy	2CL5	1VPT	1H1D		0.92	Sc=6.55883, min distance = 2.02724
PAP2_VACCW Fı	446535 CI	HNMT_HUMAN Fu	439155 Ad	2AOT	1V39	1JQE		0.95	Sc=6.54321, min distance = 2.30065
PAP2_VACCW Fı	60961 ade	PIMT_PYRFU Fu	439155 Ad	1JG1	1V39	1JG2		0.84	Sc=6.31289, min distance = 2.54224
PAPC_ECOLX Fı	131682 Sa	AOFB_HUMAN Fu	444279 1s	1OJD	2VQI	2V5Z			Sc=6.01327, min distance = 2.08819
PAPC_ECOLX Fı	8182 DODE	RCEM_RHOSH Fu	444279 1s	1RZH	2VQI	2JIY			Sc=5.696, min distance = 0.672387
PAPS1_HUMAN	445940 O6	CDK2_HUMAN Fu	6022 Aden	1GY3	1X6V	1GZ8			Sc=6.16907, min distance = 2.02946
PAPS1_HUMAN	4565 1h1r	CDK2_HUMAN Fu	6022 Aden	1GY3	1X6V	1H1R			Sc=6.1148, min distance = 2.074793
PAPS1_HUMAN	5957 Aden	BIOD_ECOLI Fu	6022 Aden	1DAD	1X6V	1A82		0.99	Sc=5.75831, min distance = 2.25545
PAPS1_HUMAN	6031 Uric	RIR1_YEAST Fu	6022 Aden	2CVX	1X6V	2CVV			Sc=5.60259, min distance = 2.05314
PAPS1_HUMAN	91532 AMI	KIF1A_MOUSE Fı	6022 Aden	1I5S	1X6V	1I6I		0.99	Sc=5.76906, min distance = 2.40559
PAPS1_HUMAN	91532 AMI	NIFH1_AZOVI Fı	6022 Aden	1FP6	1X6V	2AFK		0.99	Sc=5.79718, min distance = 2.10966
PAPS2_HUMAN	445940 O6	CDK2_HUMAN Fu	6022 Aden	1GY3	2AX4	1GZ8			Sc=6.17038, min distance = 2.42732
PAPS2_HUMAN	447004 CI	MYS2_DICDI Fu	6022 Aden	1VOM	2AX4	1LVK			Sc=6.05461, min distance = 2.40485
PAPS2_HUMAN	4564 1e1v	CDK2_HUMAN Fu	6022 Aden	1GY3	2AX4	1E1V			Sc=6.09693, min distance = 2.28887
PAPS2_HUMAN	4565 1h1r	CDK2_HUMAN Fu	6022 Aden	1GY3	2AX4	1H1R			Sc=6.13882, min distance = 1.76094
PAPS2_HUMAN	91532 AMI	DNAA_AQUAE Fu	6022 Aden	1L8Q	2AX4	2HCB		0.99	Sc=5.71004, min distance = 1.76176
PAP_YEAST Fu	11708454	KAPCA_BOVIN Fı	5957 Aden	1Q24	2Q66	2VNW			Sc=5.8915, min distance = 2.214344
PAP_YEAST Fu	17754396	PDPK1_HUMAN Fı	5957 Aden	2BIY	2Q66	2R7B			Sc=6.27985, min distance = 2.26588
PAP_YEAST Fu	3540 1yds	KAPCA_BOVIN Fı	5957 Aden	1Q24	2Q66	1YDS			Sc=5.66462, min distance = 2.12664
PAP_YEAST Fu	3547 Fasu	KAPCA_BOVIN Fı	5957 Aden	1Q24	2Q66	1Q8W			Sc=5.75747, min distance = 2.12970
PAP_YEAST Fu	444564 AI	BIOD_ECOLI Fu	5957 Aden	1A82	2Q66	1BS1		0.91	Sc=5.84355, min distance = 2.38028
PAP_YEAST Fu	444564 AI	MYS2_DICDI Fu	5957 Aden	1FMW	2Q66	1W9I		0.91	Sc=6.301, min distance = 0
PAP_YEAST Fu	444852 CI	ATPA1_BOVIN Fı	5957 Aden	2V7Q	2Q66	1COW		0.91	Sc=6.40901, min distance = 1.28164
PAP_YEAST Fu	6022 Ader	ARSA1_ECOLX Fı	5957 Aden	1II0	2Q66	1IHU		0.99	Sc=5.83829, min distance = 2.18029
PAP_YEAST Fu	6022 Ader	BIOD_ECOLI Fu	5957 Aden	1A82	2Q66	1DAD		0.99	Sc=6.29708, min distance = 2.01527
PAP_YEAST Fu	6022 Ader	MUTS_ECOLI Fu	5957 Aden	1W7A	2Q66	1OH7		0.99	Sc=6.29979, min distance = 2.14413
PAP_YEAST Fu	6022 Ader	PDK2_HUMAN Fu	5957 Aden	2BU2	2Q66	2BU8		0.99	Sc=5.87081, min distance = 2.45592

PAP_YEAST Fu	6022 Aden	PURK_ECOLI Fu	5957 Aden	3ETH	2Q66	3ETJ		0.99	Sc=6.28756, min distance = 2.21511
PAP_YEAST Fu	6022 Aden	PURP_METJA Fu	5957 Aden	2R7L	2Q66	2R7N		0.99	Sc=5.81251, min distance = 2.81827
PAP_YEAST Fu	6022 Aden	Q72H90_THET2 I	5957 Aden	2BEK	2Q66	2BEJ		0.99	Sc=5.86961, min distance = 2.11376
PAP_YEAST Fu	6022 Aden	Y059_METJA Fu	5957 Aden	2J9C	2Q66	2J9D		0.99	Sc=6.35256, min distance = 2.17650
PAP_YEAST Fu	6083 aden	Y059_METJA Fu	5957 Aden	2J9C	2Q66	2J9D		0.98	Sc=6.24727, min distance = 2.16391
PAP_YEAST Fu	6176 Cyti	Q381M1_9TRYP I	5957 Aden	2Q0D	2Q66	2Q0C			Sc=5.85899, min distance = 2.42657
PAP_YEAST Fu	65070 dUT	Q381M1_9TRYP I	5957 Aden	2Q0D	2Q66	2NOM			Sc=5.8362, min distance = 1.480048
PAP_YEAST Fu	6830 guan	Q381M1_9TRYP I	5957 Aden	2Q0D	2Q66	2Q0E		0.8	Sc=5.92799, min distance = 2.32872
PAP_YEAST Fu	91532 AMP	NIFH1_AZOVI Fu	5957 Aden	2C8V	2Q66	2AFK		0.99	Sc=6.4119, min distance = 2.056666
PAP_YEAST Fu	91532 AMP	Q5SJV7_THET8 I	5957 Aden	2Z08	2Q66	2Z09		0.99	Sc=5.66497, min distance = 2.12677
PAR12_HUMAN I	439183 1i	TPIS_TRYBB Fu	311 citri	2VEK	2PQF	1IIH			Sc=5.64399, min distance = 1.22211
PAR12_HUMAN I	439459 Ho	NIFD_KLEPN Fu	311 citri	1H1L	2PQF	1QGU		0.92	Sc=6.01351, min distance = 1.80836
PAR12_HUMAN I	439459 Ho	NIFK_KLEPN Fu	311 citri	1H1L	2PQF	1QGU		0.92	Sc=6.01351, min distance = 1.74974
PAR12_HUMAN I	444568 HI	RENI_HUMAN Fu	311 citri	2IL2	2PQF	1BIL			Sc=5.86771, min distance = 0.77391
PAR12_HUMAN I	51 2-Oxop	SERA_ECOLI Fu	311 citri	2P9E	2PQF	1YBA		0.76	Sc=5.87955, min distance = 0.53943
PARE_ECOLI Fu	16058649	IGF1R_HUMAN Fu	33113 gam	1JQH	1S16	2OJ9			Sc=6.57326, min distance = 1.96377
PARE_ECOLI Fu	16750062	CSK2A_MAIZE Fu	33113 gam	1LP4	1S16	2OXD			Sc=5.67964, min distance = 2.64984
PARE_ECOLI Fu	5287844 i	GSK3B_HUMAN Fu	33113 gam	1J1B	1S16	1UV5			Sc=6.35883, min distance = 2.05008
PARE_ECOLI Fu	5326976 1	CSK2A_MAIZE Fu	33113 gam	1LP4	1S16	1ZOE			Sc=5.72439, min distance = 2.60059
PARE_ECOLI Fu	5326978 1	CSK2A_MAIZE Fu	33113 gam	1LP4	1S16	1ZOH			Sc=5.64619, min distance = 2.56301
PARE_ECOLI Fu	5327148 C	IPKA_RABIT Fu	33113 gam	1CDK	1S16	2ERZ			Sc=6.21035, min distance = 1.35766
PARE_ECOLI Fu	9549303 2	STK6_HUMAN Fu	33113 gam	2DWB	1S16	2NP8			Sc=6.54531, min distance = 2.01268
PARM_ECOLX Fu	24978488	TBB2B_BOVIN Fu	8977 1dar	1Z2B	2ZGY	3DU7			Sc=6.38579, min distance = 2.28853
PARM_ECOLX Fu	37792 gam	ARF6_HUMAN Fu	8977 1dar	1E0S	2ZGY	2J5X		0.99	Sc=6.44399, min distance = 2.04054
PARM_ECOLX Fu	37792 gam	RHOA_HUMAN Fu	8977 1dar	1TX4	2ZGY	1CXZ		0.99	Sc=6.00025, min distance = 2.06701
PARM_ECOLX Fu	444564 AD	BIOD_ECOLI Fu	6022 Aden	1DAD	1MWM	1BS1		0.91	Sc=6.37018, min distance = 1.73856
PARM_ECOLX Fu	444845 1d	RASH_HUMAN Fu	36735 Gpp	1CTQ	2ZGZ	1CLU		0.99	Sc=6.5017, min distance = 2.195093
PARM_ECOLX Fu	444852 CI	ATPB_BOVIN Fu	6022 Aden	2CK3	1MWM	1COW		0.9	Sc=6.44828, min distance = 2.09063
PARM_ECOLX Fu	445940 O6	CDK2_HUMAN Fu	6022 Aden	1GY3	1MWM	1GZ8			Sc=6.15666, min distance = 2.51629
PARM_ECOLX Fu	445966 O6	CDK2_HUMAN Fu	6022 Aden	1GY3	1MWM	1H0V			Sc=6.15212, min distance = 1.68215
PARM_ECOLX Fu	446248 CI	RAN_HUMAN Full	36735 Gpp	1K5D	2ZGZ	1IBR			Sc=6.00575, min distance = 2.31097
PARM_ECOLX Fu	446248 CI	RASH_HUMAN Fu	36735 Gpp	1CTQ	2ZGZ	1IAQ			Sc=6.44977, min distance = 2.24173
PARM_ECOLX Fu	447955 1p	CDK2_HUMAN Fu	6022 Aden	1GY3	1MWM	1PXI			Sc=5.68777, min distance = 2.67019
PARM_ECOLX Fu	448405 CI	ARF1_RAT Full-	8977 1dar	1RRG	2ZGY	1RRF			Sc=5.9388, min distance = 2.204664
PARM_ECOLX Fu	4565 1h1r	CDK2_HUMAN Fu	6022 Aden	1GY3	1MWM	1H1R			Sc=6.39701, min distance = 1.51273
PARM_ECOLX Fu	5957 Aden	ACTS_RABIT Fu	6022 Aden	1J6Z	1MWM	2FXU		0.99	Sc=6.48042, min distance = 1.88281
PARM_ECOLX Fu	5957 Aden	HSP7C_BOVIN Fu	6022 Aden	1BUP	1MWM	2BUP		0.99	Sc=6.46729, min distance = 1.34367

PARM_ECOLX Fu	5957 Aden	HSP7F_YEAST Fu	6022 Aden	3C7N	1MWM	3D2F	0.99	Sc=6.4769, min distance = 2.156591(
PARM_ECOLX Fu	5957 Aden	LT_SV40 Full=l	6022 Aden	1SVL	1MWM	1SVM	0.99	Sc=6.56537, min distance = 0.52766:
PARM_ECOLX Fu	5957 Aden	MUTS_ECOLI Ful	6022 Aden	1OH7	1MWM	1W7A	0.99	Sc=6.46129, min distance = 1.80546(
PARM_ECOLX Fu	5957 Aden	Q9RVK2_DEIRA l	36735 Gpp	1SZ3	2ZGZ	1SU2	0.8	Sc=6.45321, min distance = 1.65438!
PARM_ECOLX Fu	5957 Aden	Y059_METJA Ful	6022 Aden	2J9D	1MWM	2J9C	0.99	Sc=6.44794, min distance = 1.65553'
PARM_ECOLX Fu	60961 ade	IPKA_RABIT Ful	6022 Aden	1JBP	1MWM	1FMO	0.95	Sc=6.1383, min distance = 1.775393:
PARM_ECOLX Fu	6804 guar	EF1A_YEAST Ful	8977 1dar	2B7B	2ZGY	1G7C	0.99	Sc=5.88104, min distance = 2.17596'
PARM_ECOLX Fu	6804 guar	TGM3_HUMAN Ful	8977 1dar	1VJJ	2ZGY	1SGX	0.99	Sc=6.33143, min distance = 2.30560{
PARM_ECOLX Fu	6852187 2	O57883_PYRHO l	6022 Aden	1WNL	1MWM	2DTH	0.89	Sc=6.03507, min distance = 1.79903:
PARM_ECOLX Fu	91532 AMF	PURP_METJA Ful	6022 Aden	2R7N	1MWM	2R7K	0.99	Sc=6.50713, min distance = 1.52353:
PARM_ECOLX Fu	93082 Gak	CDC42_HUMAN Fu	36735 Gpp	1NF3	2ZGZ	2ODB	0.99	Sc=6.47132, min distance = 2.29550{
PARM_ECOLX Fu	93082 Gak	FTSY_THEAQ Ful	36735 Gpp	2J7P	2ZGZ	1RJ9	0.99	Sc=6.47344, min distance = 2.07287'
PARM_ECOLX Fu	93082 Gak	RASH_HUMAN Ful	36735 Gpp	1CTQ	2ZGZ	121P	0.99	Sc=6.03785, min distance = 2.31548:
PBPA_AQUAE Fu	125409 be	XYNA_BACSU Ful	23831 HEF	2B45	2OQO	2QZ3		Sc=5.8193, min distance = 1.301698{
PBPA_AQUAE Fu	164795 N(	RBCMT_PEA Full	23831 HEF	1MLV	2OQO	2H2J		Sc=5.7749, min distance = 1.703892{
PBPA_AQUAE Fu	447768 CI	PURR_BACSU Ful	23831 HEF	1O57	2OQO	1P4A		Sc=5.67444, min distance = 1.55730·
PBPA_AQUAE Fu	62551 Per	Q8WSF8_APLCA l	23831 HEF	2BR7	2OQO	2BYN		Sc=5.75357, min distance = 2.07088:
PBPA_AQUAE Fu	854023 Ep	Q8WSF8_APLCA l	23831 HEF	2BR7	2OQO	2BYQ		Sc=5.72343, min distance = 1.34256{
PCAF_HUMAN Fu	449233 CI	O53831_MYCTU l	87642 coe	1P0H	1CM0	2C27		Sc=5.95746, min distance = 2.27085!
PCCA_HUMAN Fu	24832019	ACES_TORCA Ful	8200 TETF	1DX6	2JKU	2J3Q		Sc=5.64814, min distance = 2.32483!
PCRA_BACST Fu	11708454	KAPCA_BOVIN Fu	5957 Aden	1Q24	3PJR	2VNW		Sc=6.10093, min distance = 2.43284·
PCRA_BACST Fu	6022 Aden	PURK_ECOLI Ful	5957 Aden	3ETH	3PJR	3ETJ	0.99	Sc=6.4564, min distance = 1.934170(
PCRA_BACST Fu	6022 Aden	Y059_METJA Ful	5957 Aden	2J9C	3PJR	2J9D	0.99	Sc=6.4349, min distance = 1.696301:
PCRA_BACST Fu	6852207 M	KAPCA_BOVIN Fu	5957 Aden	1Q24	3PJR	2GNI		Sc=5.89708, min distance = 2.02186!
PCXB_ACIAD Fu	127 4-hyd	PCXA_PSEPU Ful	72 protoc	1YKN	2BUV	3PCG	0.79	Sc=5.72521, min distance = 2.18716{
PCXB_ACIAD Fu	127 4-hyd	PCXB_PSEPU Ful	72 protoc	1YKN	2BUV	3PCG	0.79	Sc=5.74284, min distance = 2.33650:
PCXB_ACIAD Fu	160456 3p	PCXB_PSEPU Ful	135 p-Hyd	3PCC	2BUR	3PCF	0.97	Sc=5.68392, min distance = 2.33318:
PCY2_HUMAN Fu	6132 Cyti	KPSU5_ECOLX Fu	6131 cyti	1H7F	3ELB	1H7H	0.99	Sc=6.36819, min distance = 1.00951:
PCYB_PSEPA Fu	12122 3-H	PCXB_PSEPU Ful	72 protoc	1YKN	1B4U	3PCE	0.84	Sc=5.71389, min distance = 2.86567'
PCYB_PSEPA Fu	127 4-hyd	PCXA_PSEPU Ful	72 protoc	1YKN	1B4U	3PCG	0.79	Sc=5.77331, min distance = 2.65016'
PCYB_PSEPA Fu	127 4-hyd	PCXB_PSEPU Ful	72 protoc	1YKN	1B4U	3PCG	0.79	Sc=5.77146, min distance = 2.66598·
PCYB_PSEPA Fu	135 p-Hyd	PCXA_PSEPU Ful	72 protoc	1YKN	1B4U	3PCC	0.89	Sc=5.65416, min distance = 2.62244!
PCYB_PSEPA Fu	135 p-Hyd	PHHY_PSEFL Ful	72 protoc	1PHH	1B4U	1PBE	0.89	Sc=5.65867, min distance = 2.66510:
PCYB_PSEPA Fu	160456 3p	PCXA_PSEPU Ful	72 protoc	1YKN	1B4U	3PCF	0.87	Sc=5.70177, min distance = 2.81932!
PCYB_PSEPA Fu	3505109 4	LOX3_SOYBN Ful	72 protoc	1N8Q	1B4U	1NO3		Sc=5.78164, min distance = 2.17791!
PCYB_PSEPA Fu	3505109 4	PCXB_ACIAD Ful	72 protoc	2BUV	1B4U	2BUU		Sc=5.76391, min distance = 2.03538!
PCYB_PSEPA Fu	7420 3-Hy	PCXA_PSEPU Ful	72 protoc	1YKN	1B4U	3PCB	0.89	Sc=5.67564, min distance = 2.31030:

PCYB_PSEPA Fu	7420 3-Hy	PCXB_PSEPU Ful	72 protoc	1YKN	1B4U	3PCB		0.89	Sc=5.77648, min distance = 0.529185
PDC1_YEAST Fu	124687 1r	ODP1_ECOLI Ful	1132 thia	1L8A	1PVD	1RP7		0.79	Sc=6.0009, min distance = 1.9221854
PDC1_YEAST Fu	13294447	DCIP_AZOBR Ful	1132 thia	2NXW	1PVD	2Q5Q			Sc=6.08207, min distance = 1.005624
PDC1_YEAST Fu	444421 CI	MDLC_PSEPU Ful	1132 thia	1BFD	1PVD	2FWN		0.82	Sc=6.43308, min distance = 2.381036
PDC1_YEAST Fu	445886 CI	TKT1_YEAST Ful	1132 thia	1TRK	1PVD	1GPU		0.91	Sc=5.77658, min distance = 1.977424
PDC1_YEAST Fu	448671 2-	ILVB_YEAST Ful	1132 thia	1N0H	1PVD	1T9B			Sc=5.83815, min distance = 2.666225
PDC1_YEAST Fu	448673 PE	ILVB_YEAST Ful	1132 thia	1N0H	1PVD	1T9B			Sc=5.80455, min distance = 2.116964
PDC1_YEAST Fu	448721 N3	TKT1_YEAST Ful	1132 thia	1TRK	1PVD	1TKA			Sc=6.43027, min distance = 2.139874
PDC1_YEAST Fu	448723 1t	TKT1_YEAST Ful	1132 thia	1TRK	1PVD	1TKC		0.77	Sc=6.45782, min distance = 2.375278
PDC1_YEAST Fu	6102647 C	ODBA_HUMAN Ful	1132 thia	2BFD	1PVD	1WCI		0.9	Sc=6.10814, min distance = 2.289337
PDC1_YEAST Fu	6102749 T	ODBA_HUMAN Ful	1132 thia	2BFD	1PVD	2J9F		0.92	Sc=6.07952, min distance = 2.203022
PDC1_YEAST Fu	6102750 T	ODBA_HUMAN Ful	1132 thia	2BFD	1PVD	2BEV		0.9	Sc=6.10705, min distance = 2.002410
PDC1_YEAST Fu	6518187 2	POXB_LACPL Ful	1132 thia	2EZ4	1PVD	2EZ8		0.87	Sc=5.77909, min distance = 2.043065
PDC_ZYMMO Ful	447530	SRC_HUMAN Full	311 citri	1O4L	1ZPD	1O4F			Sc=5.77975, min distance = 2.630084
PDE10_HUMAN F	3758 IBMX	PDE4D_HUMAN Fu	6076 Cycl	2PW3	2OUR	1ZKN			Sc=5.70912, min distance = 2.276946
PDE10_HUMAN F	3758 IBMX	PDE5A_HUMAN Fu	6804 guan	1T9S	2OUQ	1RKP			Sc=6.03031, min distance = 0
PDE10_HUMAN F	6419789 C	HASP_HUMAN Ful	6083 aden	3DLZ	2OUN	3E7V			Sc=6.06816, min distance = 2.010096
PDE10_HUMAN F	656964 1y	PDE4D_HUMAN Fu	6076 Cycl	2PW3	2OUR	1Y2C			Sc=5.70438, min distance = 2.169420
PDE10_HUMAN F	656964 1y	PDE4D_HUMAN Fu	6083 aden	1TB7	2OUN	1Y2C			Sc=5.69628, min distance = 2.923500
PDE10_HUMAN F	656966 1y	PDE4D_HUMAN Fu	6083 aden	1TB7	2OUN	1Y2E			Sc=5.86942, min distance = 2.858697
PDE10_HUMAN F	656969 1y	PDE4D_HUMAN Fu	6076 Cycl	2PW3	2OUR	1Y2K			Sc=6.01978, min distance = 2.113108
PDE10_HUMAN F	656969 1y	PDE4D_HUMAN Fu	6083 aden	1TB7	2OUN	1Y2K			Sc=6.0336, min distance = 2.4498577
PDE4A_HUMAN F	5212 sild	PDE4B_HUMAN Fu	9999276 N	2QYL	2QYK	1XOS			Sc=6.24253, min distance = 2.179035
PDE4A_HUMAN F	656964 1y	PDE4D_HUMAN Fu	9999276 N	2QYN	2QYK	1Y2C			Sc=5.74108, min distance = 2.771878
PDE4A_HUMAN F	656965 1y	PDE4D_HUMAN Fu	9999276 N	2QYN	2QYK	1Y2D			Sc=5.76854, min distance = 2.737717
PDE4A_HUMAN F	656966 1y	PDE4D_HUMAN Fu	9999276 N	2QYN	2QYK	1Y2E			Sc=5.90321, min distance = 2.479066
PDE4A_HUMAN F	656968 1y	PDE4B_HUMAN Fu	9999276 N	2QYL	2QYK	1Y2H			Sc=5.76113, min distance = 2.495722
PDE4A_HUMAN F	656969 1y	PDE4B_HUMAN Fu	9999276 N	2QYL	2QYK	1Y2J			Sc=6.05533, min distance = 2.396435
PDE4A_HUMAN F	656969 1y	PDE4D_HUMAN Fu	9999276 N	2QYN	2QYK	1Y2K			Sc=6.06519, min distance = 2.530304
PDE4B_HUMAN F	6804 guar	PDE10_HUMAN Fu	6083 aden	2OUN	1ROR	2OUQ		0.8	Sc=5.60666, min distance = 0
PDE5A_HUMAN F	9578243 E	PDE4B_HUMAN Fu	5212 sild	1XOS	1TBF	1XLZ	34.97		Sc=6.10149, min distance = 2.052842
PDE6C_CHICK F	6858240 C	KAP0_BOVIN Ful	24316 cyc	1RL3	3DBA	1NE6		0.79	Sc=6.24465, min distance = 2.026436
PDE7A_HUMAN F	215436 1y	PDE4D_HUMAN Fu	3758 IBMX	1ZKN	1ZKL	1Y2B			Sc=5.78991, min distance = 2.197135
PDE7A_HUMAN F	448055 To	PDE4D_HUMAN Fu	3758 IBMX	1ZKN	1ZKL	1Q9M			Sc=6.07096, min distance = 1.682037
PDE7A_HUMAN F	656964 1y	PDE4D_HUMAN Fu	3758 IBMX	1ZKN	1ZKL	1Y2C			Sc=5.86799, min distance = 1.743897
PDE7A_HUMAN F	656965 1y	PDE4D_HUMAN Fu	3758 IBMX	1ZKN	1ZKL	1Y2D			Sc=5.96442, min distance = 1.441188
PDE7A_HUMAN F	656966 1y	PDE4D_HUMAN Fu	3758 IBMX	1ZKN	1ZKL	1Y2E			Sc=6.02133, min distance = 1.578558

PDK2_HUMAN Fı	11608401	KAPCA_BOVIN Fı	5957 Aden	1Q24	2BU2	2UVY			Sc=6.0826, min distance = 2.233719(
PDK2_HUMAN Fı	11708454	KAPCA_BOVIN Fı	5957 Aden	1Q24	2BU2	2VNW			Sc=6.05619, min distance = 2.17974&
PDK2_HUMAN Fı	11963552	HSP82_YEAST Fı	6022 Aden	1AMW	2BU8	2IWU			Sc=6.16455, min distance = 2.85150'
PDK2_HUMAN Fı	15602982	KAPCA_BOVIN Fı	5957 Aden	1Q24	2BU2	2UW6			Sc=5.95649, min distance = 2.44469'
PDK2_HUMAN Fı	24762195	KAPCA_BOVIN Fı	5957 Aden	1Q24	2BU2	2VO0			Sc=6.21316, min distance = 2.18436:
PDK2_HUMAN Fı	24832021	HS90A_HUMAN Fı	6022 Aden	1BYQ	2BU8	2QG0			Sc=6.35581, min distance = 2.21089(
PDK2_HUMAN Fı	440317 AT	BCKD_RAT Full-	6022 Aden	1GKZ	2BU8	1GJV		0.99	Sc=5.65796, min distance = 2.77232'
PDK2_HUMAN Fı	447004 CI	MYS2_DICDI Ful	5957 Aden	1FMW	2BU2	1LVK			Sc=5.97545, min distance = 0
PDK2_HUMAN Fı	447004 CI	MYS2_DICDI Ful	6022 Aden	1VOM	2BU8	1LVK			Sc=6.06208, min distance = 2.25813:
PDK2_HUMAN Fı	447955 1p	CDK2_HUMAN Ful	6022 Aden	1GY3	2BU8	1PXI			Sc=5.62352, min distance = 2.36324:
PDK2_HUMAN Fı	5327135 I	CDK2_HUMAN Ful	6022 Aden	1GY3	2BU8	2C6M			Sc=6.12055, min distance = 1.38255!
PDK2_HUMAN Fı	5327148 C	IPKA_RABIT Ful	6022 Aden	1JBP	2BU8	2ERZ			Sc=6.05012, min distance = 2.08207&
PDK2_HUMAN Fı	6082103 2	HSP82_YEAST Fı	6022 Aden	1AMW	2BU8	2BRC			Sc=6.304, min distance = 2.2651505
PDK2_HUMAN Fı	6102787 2	HS90A_HUMAN Fı	6022 Aden	1BYQ	2BU8	2CCS			Sc=6.15882, min distance = 2.54566
PDK2_HUMAN Fı	6323491 M	PDK3_HUMAN Ful	5957 Aden	1Y8P	2BU2	2Q8I			Sc=6.29126, min distance = 2.64646:
PDK2_HUMAN Fı	6323491 M	PDK3_HUMAN Ful	6022 Aden	1Y8O	2BU8	2Q8I			Sc=6.27438, min distance = 2.70783
PDK2_HUMAN Fı	6323491 M	TOP6B_SULSH Fı	6022 Aden	1Z5B	2BU8	2HKJ			Sc=6.28836, min distance = 2.42417!
PDK2_HUMAN Fı	8977 1dar	NDK_THET8 Full	5957 Aden	1WKL	2BU2	1WKK		0.8	Sc=5.8325, min distance = 2.417707:
PDK2_HUMAN Fı	8977 1dar	O33839_THEMA I	6022 Aden	1XJK	2BU8	1XJE		0.8	Sc=5.62736, min distance = 2.11018:
PDK2_RAT Ful	15993 dAT	PRIM_BPT7 Full	33113 gam	1E0J	3CRL	1CR2		0.97	Sc=5.96944, min distance = 2.02117(
PDK2_RAT Ful	16058649	IGF1R_HUMAN Fı	33113 gam	1JQH	3CRL	2OJ9			Sc=6.4202, min distance = 2.1210348
PDK2_RAT Ful	188966 dA	HSLU_ECOLI Ful	33113 gam	1E94	3CRL	1G4A		0.97	Sc=5.84096, min distance = 1.93616'
PDK2_RAT Ful	24871491	INSR_HUMAN Ful	33113 gam	1IR3	3CRL	2Z8C			Sc=6.09251, min distance = 2.20678:
PDK2_RAT Ful	24963048	AKT2_HUMAN Ful	33113 gam	1O6L	3CRL	3E87			Sc=6.42568, min distance = 1.98960(
PDK2_RAT Ful	3547 Fasu	ROCK1_HUMAN Fı	33113 gam	2V55	3CRL	2ESM			Sc=5.81378, min distance = 2.05456(
PDK2_RAT Ful	448042 2e	ROCK1_HUMAN Fı	33113 gam	2V55	3CRL	2ETR			Sc=6.08504, min distance = 2.03182'
PDK2_RAT Ful	5327148 C	IPKA_RABIT Ful	33113 gam	1CDK	3CRL	2ERZ			Sc=6.07421, min distance = 2.00792
PDK2_RAT Ful	64968 dTI	RIR1_YEAST Ful	33113 gam	2EUD	3CRL	2CVW			Sc=5.63093, min distance = 2.06436:
PDK2_RAT Ful	91532 AME	CHEA_THEMA Ful	33113 gam	1I59	3CRL	1I58		0.99	Sc=5.70447, min distance = 2.26528'
PDK2_RAT Ful	91532 AME	IGF1R_HUMAN Fı	33113 gam	1JQH	3CRL	1K3A		0.99	Sc=5.69229, min distance = 1.97853
PDK3_HUMAN Fı	11175137	KAPCA_BOVIN Fı	5957 Aden	1Q24	1Y8P	2UW7			Sc=6.22872, min distance = 2.00140!
PDK3_HUMAN Fı	11608401	KAPCA_BOVIN Fı	5957 Aden	1Q24	1Y8P	2UVY			Sc=6.17654, min distance = 2.09717!
PDK3_HUMAN Fı	11963552	HSP82_YEAST Fı	6022 Aden	1AMW	1Y8O	2IWU			Sc=6.18136, min distance = 2.06873!
PDK3_HUMAN Fı	15602983	KAPCA_BOVIN Fı	5957 Aden	1Q24	1Y8P	2UW5			Sc=6.12453, min distance = 2.01011(
PDK3_HUMAN Fı	16122635	KAPCA_BOVIN Fı	5957 Aden	1Q24	1Y8P	2UW8			Sc=5.68806, min distance = 2.14095(
PDK3_HUMAN Fı	188966 dA	PAPS1_HUMAN Fı	6022 Aden	1X6V	1Y8O	2PEZ		0.98	Sc=6.29831, min distance = 1.90976:
PDK3_HUMAN Fı	444564 AI	MYS2_DICDI Ful	5957 Aden	1FMW	1Y8P	1W9I		0.91	Sc=5.94862, min distance = 0

PDK3_HUMAN Fu	446090 1h	NDKC_DICDI Fu	6022 Aden	1KDN	1Y8O	1HIY		0.97	Sc=5.91339, min distance = 1.932415
PDK3_HUMAN Fu	448042 2e	KAPCA_BOVIN Fu	5957 Aden	1Q24	1Y8P	1Q8T			Sc=6.01208, min distance = 2.028920
PDK3_HUMAN Fu	6083 aden	AAKG1_RAT Ful	5957 Aden	2V92	1Y8P	2V8Q		0.98	Sc=5.84034, min distance = 2.401521
PDK3_HUMAN Fu	6083 aden	Y059_METJA Fu	5957 Aden	2J9C	1Y8P	2J9D		0.98	Sc=5.85522, min distance = 2.512556
PDK3_HUMAN Fu	656971 CI	CDK2_HUMAN Fu	6022 Aden	1GY3	1Y8O	1Y8Y			Sc=5.91238, min distance = 2.005881
PDK3_HUMAN Fu	72194 2-C	ENPL_CANFA Fu	6022 Aden	1TC6	1Y8O	1QYE		0.89	Sc=5.95481, min distance = 2.298559
PDK3_HUMAN Fu	72194 2-C	ENPL_CANFA Fu	6323491 M	1QY8	2Q8I	1QYE			Sc=6.02402, min distance = 2.843155
PDK3_HUMAN Fu	8977 1dar	NDK_PYRHO Ful	6022 Aden	2DYA	1Y8O	2DXE		0.8	Sc=5.61573, min distance = 2.286347
PDK4_HUMAN Fu	11963551	HSP82_YEAST Fu	6022 Aden	1AMW	2ZKJ	2IWS			Sc=6.18391, min distance = 2.194296
PDK4_HUMAN Fu	11963552	HSP82_YEAST Fu	6022 Aden	1AMW	2ZKJ	2IWU			Sc=6.19952, min distance = 2.346524
PDK4_HUMAN Fu	16058649	IGF1R_HUMAN Fu	33113 gam	1JQH	2E0A	2OJ9			Sc=6.45577, min distance = 2.243407
PDK4_HUMAN Fu	16058668	HS90A_HUMAN Fu	6022 Aden	1BYQ	2ZKJ	2UWD			Sc=6.34406, min distance = 2.281284
PDK4_HUMAN Fu	164795 N(	RBCMT_PEA Ful	23831 HEP	1MLV	2ZDY	2H2J			Sc=5.65794, min distance = 2.211440
PDK4_HUMAN Fu	188966 dA	HSLU_ECOLI Fu	33113 gam	1E94	2E0A	1G4A		0.97	Sc=6.39408, min distance = 2.466319
PDK4_HUMAN Fu	24832021	HS90A_HUMAN Fu	6022 Aden	1BYQ	2ZKJ	2QG0			Sc=6.39776, min distance = 2.040564
PDK4_HUMAN Fu	24836816	HS90A_HUMAN Fu	6022 Aden	1BYQ	2ZKJ	3BMY			Sc=6.29747, min distance = 2.172421
PDK4_HUMAN Fu	3064778 H	ROCK1_HUMAN Fu	33113 gam	2V55	2E0A	2ETK			Sc=6.04583, min distance = 2.113311
PDK4_HUMAN Fu	3547 Fasu	ROCK1_HUMAN Fu	33113 gam	2V55	2E0A	2ESM			Sc=5.94101, min distance = 2.005445
PDK4_HUMAN Fu	440317 AT	BCKD_RAT Full	6022 Aden	1GKZ	2ZKJ	1GJV		0.99	Sc=6.02593, min distance = 2.535604
PDK4_HUMAN Fu	444503 FU	NDKC_DICDI Fu	6022 Aden	1KDN	2ZKJ	1B99			Sc=6.2851, min distance = 1.9119547
PDK4_HUMAN Fu	445940 O6	CDK2_HUMAN Fu	6022 Aden	1GY3	2ZKJ	1GZ8			Sc=6.10631, min distance = 2.004528
PDK4_HUMAN Fu	446721 CI	ASSY_THET8 Fu	33113 gam	1KOR	2E0A	1KH2		0.93	Sc=6.03998, min distance = 2.084181
PDK4_HUMAN Fu	447955 1p	CDK2_HUMAN Fu	6022 Aden	1GY3	2ZKJ	1PXI			Sc=5.72751, min distance = 2.246978
PDK4_HUMAN Fu	448014 1c	GSK3B_HUMAN Fu	33113 gam	1J1B	2E0A	1Q5K			Sc=6.24738, min distance = 1.556961
PDK4_HUMAN Fu	448042 2e	ROCK1_HUMAN Fu	33113 gam	2V55	2E0A	2ETR			Sc=6.09737, min distance = 2.181331
PDK4_HUMAN Fu	448043 2g	ROCK1_HUMAN Fu	33113 gam	2V55	2E0A	3D9V			Sc=5.96917, min distance = 2.068809
PDK4_HUMAN Fu	4565 1h1r	CDK2_HUMAN Fu	6022 Aden	1GY3	2ZKJ	1H1R			Sc=6.26922, min distance = 1.853728
PDK4_HUMAN Fu	5326976 1	CSK2A_MAIZE Fu	33113 gam	1LP4	2E0A	1ZOE			Sc=5.64852, min distance = 2.811681
PDK4_HUMAN Fu	5327095 V	HS90A_HUMAN Fu	6022 Aden	1BYQ	2ZKJ	2BSM			Sc=6.39566, min distance = 2.407401
PDK4_HUMAN Fu	5327103 2	HS90A_HUMAN Fu	6022 Aden	1BYQ	2ZKJ	2BYH			Sc=6.46646, min distance = 2.354200
PDK4_HUMAN Fu	5327148 C	IPKA_RABIT Fu	33113 gam	1CDK	2E0A	2ERZ			Sc=6.15459, min distance = 1.816232
PDK4_HUMAN Fu	5327148 C	IPKA_RABIT Fu	6022 Aden	1JBP	2ZKJ	2ERZ			Sc=6.17987, min distance = 1.921760
PDK4_HUMAN Fu	5957 Aden	PDK2_HUMAN Fu	6022 Aden	2BU8	2ZKJ	2BU2		0.99	Sc=6.04171, min distance = 2.627306
PDK4_HUMAN Fu	5957 Aden	PDK3_HUMAN Fu	6022 Aden	1Y8O	2ZKJ	1Y8P		0.99	Sc=6.03633, min distance = 2.610157
PDK4_HUMAN Fu	5957 Aden	SP2AA_BACST Fu	6022 Aden	1TH8	2ZKJ	1TID		0.99	Sc=6.03455, min distance = 2.482925
PDK4_HUMAN Fu	6082103 2	HSP82_YEAST Fu	6022 Aden	1AMW	2ZKJ	2BRC			Sc=6.32659, min distance = 2.438716
PDK4_HUMAN Fu	6102787 2	HS90A_HUMAN Fu	6022 Aden	1BYQ	2ZKJ	2CCS			Sc=6.19023, min distance = 2.430013

PDK4_HUMAN Fı	6102788 2	HS90A_HUMAN Fı	6022 Aden	1BYQ	2ZKJ	2CCT			Sc=6.37661, min distance = 2.21923
PDK4_HUMAN Fı	6323491 M	ENPL_CANFA Ful	33113 gam	2O1U	2E0A	1QY8			Sc=6.27533, min distance = 2.15831
PDK4_HUMAN Fı	6323491 M	HSP82_YEAST Fı	6022 Aden	1AMW	2ZKJ	1BGQ			Sc=6.28937, min distance = 2.05646
PDK4_HUMAN Fı	6323491 M	PDK3_HUMAN Ful	6022 Aden	1Y8O	2ZKJ	2Q8I			Sc=6.28649, min distance = 2.52891
PDK4_HUMAN Fı	6323491 M	TOP6B_SULSH Fı	6022 Aden	1Z5B	2ZKJ	2HKJ			Sc=6.3116, min distance = 2.119968
PDK4_HUMAN Fı	6804 guar	NDK_PYRHO Ful	6022 Aden	2DYA	2ZKJ	2DXF		0.8	Sc=5.90339, min distance = 2.22524
PDK4_HUMAN Fı	72194 2-C	ENPL_CANFA Ful	6022 Aden	1TC6	2ZKJ	1QYE		0.89	Sc=6.06405, min distance = 2.47172
PDK4_HUMAN Fı	8977 1dar	NDK_PYRHO Ful	6022 Aden	2DYA	2ZKJ	2DXE		0.8	Sc=5.98206, min distance = 2.27884
PDK4_HUMAN Fı	8977 1dar	O33839_THEMA I	6022 Aden	1XJK	2ZKJ	1XJE		0.8	Sc=5.99931, min distance = 2.03212
PDLI1_HUMAN I	91420 5-T	ACES_MOUSE Ful	8200 TETR	2H9Y	2PKT	2HA0			Sc=5.63521, min distance = 1.97001
PDPK1_HUMAN I	10109823	PIM1_HUMAN Ful	2396 bisi	1XWS	1UU8	3CY3	25.47		Sc=6.34836, min distance = 2.44412
PDPK1_HUMAN I	10224714	CDK2_HUMAN Ful	444345 1c	1AQ1	1OKY	3DDP	29.3		Sc=6.49125, min distance = 2.28208
PDPK1_HUMAN I	11175137	KAPCA_BOVIN Fı	444345 1c	1STC	1OKY	2UW7	39.18		Sc=6.1707, min distance = 2.571806
PDPK1_HUMAN I	11314340	KAPCA_BOVIN Fı	444345 1c	1STC	1OKY	2UZT	39.18		Sc=6.32121, min distance = 2.27222
PDPK1_HUMAN I	11314340	KAPCA_BOVIN Fı	5957 Aden	1Q24	2BIY	2UZT	39.18		Sc=6.29536, min distance = 2.15947
PDPK1_HUMAN I	11348631	CHK1_HUMAN Ful	444345 1c	1NVR	1OKY	2E9U	33.65		Sc=6.22185, min distance = 2.27798
PDPK1_HUMAN I	11348631	CHK1_HUMAN Ful	72271 7-H	1NVQ	1OKZ	2E9U	33.65		Sc=6.31376, min distance = 2.47818
PDPK1_HUMAN I	11553058	LCK_HUMAN Full	444345 1c	1QPD	1OKY	2OF2	26.62		Sc=6.16344, min distance = 2.77052
PDPK1_HUMAN I	11608401	KAPCA_BOVIN Fı	5957 Aden	1Q24	2BIY	2UVY	39.18		Sc=6.05633, min distance = 2.44326
PDPK1_HUMAN I	11696113	KAPCA_BOVIN Fı	444345 1c	1STC	1OKY	2VO3	39.18		Sc=6.31184, min distance = 2.39349
PDPK1_HUMAN I	11708454	KAPCA_BOVIN Fı	5957 Aden	1Q24	2BIY	2VNW	39.18		Sc=6.0788, min distance = 2.425810
PDPK1_HUMAN I	11957393	FAK1_HUMAN Ful	5957 Aden	2IJM	2BIY	2ETM	23.95		Sc=6.2159, min distance = 1.914641
PDPK1_HUMAN I	11986115	PIM1_HUMAN Ful	444345 1c	1YHS	1OKY	2BZJ	25.47		Sc=6.28258, min distance = 2.01480
PDPK1_HUMAN I	129236 2c	CKI1_SCHPO Ful	5957 Aden	1CSN	2BIY	2CSN			Sc=5.76343, min distance = 2.65923
PDPK1_HUMAN I	1540 1h1q	CDK2_HUMAN Ful	444345 1c	1AQ1	1OKY	1H1Q	29.3		Sc=6.23388, min distance = 2.92814
PDPK1_HUMAN I	1540 1h1q	CDK2_HUMAN Ful	72271 7-H	1PKD	1OKZ	1H1Q	29.3		Sc=6.33258, min distance = 2.54777
PDPK1_HUMAN I	15602982	KAPCA_BOVIN Fı	444345 1c	1STC	1OKY	2UW6	39.18		Sc=6.02719, min distance = 2.73215
PDPK1_HUMAN I	15602982	KAPCA_BOVIN Fı	5957 Aden	1Q24	2BIY	2UW6	39.18		Sc=6.02504, min distance = 2.72920
PDPK1_HUMAN I	15602983	KAPCA_BOVIN Fı	444345 1c	1STC	1OKY	2UW5	39.18		Sc=6.08775, min distance = 2.68315
PDPK1_HUMAN I	15991572	LCK_HUMAN Full	444345 1c	1QPD	1OKY	2OF4	26.62		Sc=6.12483, min distance = 2.46675
PDPK1_HUMAN I	160355 rc	CDK2_HUMAN Ful	444345 1c	1AQ1	1OKY	3DDQ	29.3		Sc=6.14352, min distance = 2.71788
PDPK1_HUMAN I	160355 rc	CDK2_HUMAN Ful	72271 7-H	1PKD	1OKZ	3DDQ	29.3		Sc=6.26424, min distance = 2.42250
PDPK1_HUMAN I	16113377	CDK2_HUMAN Ful	72271 7-H	1PKD	1OKZ	2W17	29.3		Sc=6.39761, min distance = 1.72505
PDPK1_HUMAN I	16122643	CHK1_HUMAN Ful	444345 1c	1NVR	1OKY	2YWP	33.65		Sc=6.26794, min distance = 2.22864
PDPK1_HUMAN I	16122643	CHK1_HUMAN Ful	72271 7-H	1NVQ	1OKZ	2YWP	33.65		Sc=6.27029, min distance = 2.48540
PDPK1_HUMAN I	16214823	CDK2_HUMAN Ful	444345 1c	1AQ1	1OKY	2UZB	29.3		Sc=6.08768, min distance = 1.86556
PDPK1_HUMAN I	16214823	CDK2_HUMAN Ful	72271 7-H	1PKD	1OKZ	2UZB	29.3		Sc=6.08768, min distance = 2.12490

PDPK1_HUMAN I	16214825	CDK2_HUMAN Fu	444345 1c	1AQ1	1OKY	2UZE	29.3		Sc=6.24503, min distance = 2.769675
PDPK1_HUMAN I	16214826	CDK2_HUMAN Fu	444345 1c	1AQ1	1OKY	2UZL	29.3		Sc=6.12765, min distance = 2.296831
PDPK1_HUMAN I	16214826	CDK2_HUMAN Fu	72271 7-H	1PKD	1OKZ	2UZL	29.3		Sc=6.13282, min distance = 2.340981
PDPK1_HUMAN I	16214827	CDK2_HUMAN Fu	72271 7-H	1PKD	1OKZ	2UZN	29.3		Sc=6.09246, min distance = 2.480520
PDPK1_HUMAN I	16214828	CDK2_HUMAN Fu	444345 1c	1AQ1	1OKY	2UZO	29.3		Sc=6.07173, min distance = 2.192827
PDPK1_HUMAN I	16214828	CDK2_HUMAN Fu	72271 7-H	1PKD	1OKZ	2UZO	29.3		Sc=6.08984, min distance = 2.255668
PDPK1_HUMAN I	1707 1fvt	CDK2_HUMAN Fu	72271 7-H	1PKD	1OKZ	1FVT	29.3		Sc=6.20726, min distance = 1.968156
PDPK1_HUMAN I	1907917 2	CHK1_HUMAN Fu	444345 1c	1NVR	1OKY	2CGW	33.65		Sc=6.0777, min distance = 2.9104147
PDPK1_HUMAN I	23653515	CDK2_HUMAN Fu	444345 1c	1AQ1	1OKY	2R3F	29.3		Sc=5.9833, min distance = 2.5196511
PDPK1_HUMAN I	23653515	CDK2_HUMAN Fu	72271 7-H	1PKD	1OKZ	2R3F	29.3		Sc=5.99449, min distance = 2.381284
PDPK1_HUMAN I	23653516	CDK2_HUMAN Fu	72271 7-H	1PKD	1OKZ	2R3G	29.3		Sc=6.23002, min distance = 1.976414
PDPK1_HUMAN I	23653519	CDK2_HUMAN Fu	72271 7-H	1PKD	1OKZ	2R3K	29.3		Sc=6.19004, min distance = 2.181307
PDPK1_HUMAN I	23653520	CDK2_HUMAN Fu	72271 7-H	1PKD	1OKZ	2R3L	29.3		Sc=6.18711, min distance = 2.044806
PDPK1_HUMAN I	23653521	CDK2_HUMAN Fu	444345 1c	1AQ1	1OKY	2R3M	29.3		Sc=6.38895, min distance = 2.422119
PDPK1_HUMAN I	23653521	CDK2_HUMAN Fu	72271 7-H	1PKD	1OKZ	2R3M	29.3		Sc=6.32676, min distance = 2.320045
PDPK1_HUMAN I	23653522	CDK2_HUMAN Fu	444345 1c	1AQ1	1OKY	2R3N	29.3		Sc=6.14197, min distance = 2.295885
PDPK1_HUMAN I	23653524	CDK2_HUMAN Fu	72271 7-H	1PKD	1OKZ	2R3P	29.3		Sc=6.18257, min distance = 2.050850
PDPK1_HUMAN I	23653526	CDK2_HUMAN Fu	72271 7-H	1PKD	1OKZ	2R3R	29.3		Sc=6.09239, min distance = 1.981391
PDPK1_HUMAN I	23657800	CHK1_HUMAN Fu	444345 1c	1NVR	1OKY	2E9P	33.65		Sc=6.25403, min distance = 2.077250
PDPK1_HUMAN I	23657800	CHK1_HUMAN Fu	72271 7-H	1NVQ	1OKZ	2E9P	33.65		Sc=6.25637, min distance = 2.358841
PDPK1_HUMAN I	23727982	CDK2_HUMAN Fu	444345 1c	1AQ1	1OKY	3BHU	29.3		Sc=6.22933, min distance = 2.611869
PDPK1_HUMAN I	23727982	CDK2_HUMAN Fu	72271 7-H	1PKD	1OKZ	3BHU	29.3		Sc=6.2384, min distance = 1.7751625
PDPK1_HUMAN I	24180721	PIM1_HUMAN Fu	2396 bisi	1XWS	1UU8	3C4E	25.47		Sc=5.97287, min distance = 2.442019
PDPK1_HUMAN I	24752838	CHK1_HUMAN Fu	444345 1c	1NVR	1OKY	2QHM	33.65		Sc=6.03075, min distance = 3.042327
PDPK1_HUMAN I	24752838	CHK1_HUMAN Fu	72271 7-H	1NVQ	1OKZ	2QHM	33.65		Sc=6.04155, min distance = 2.920054
PDPK1_HUMAN I	24901723	CDK2_HUMAN Fu	444345 1c	1AQ1	1OKY	2W06	29.3		Sc=6.16251, min distance = 2.352262
PDPK1_HUMAN I	24901723	CDK2_HUMAN Fu	72271 7-H	1PKD	1OKZ	2W06	29.3		Sc=6.2457, min distance = 2.3025294
PDPK1_HUMAN I	24905143	PK3CG_HUMAN Fu	444345 1c	1E8Z	1OKY	2V4L			Sc=6.08853, min distance = 3.034162
PDPK1_HUMAN I	24905144	PK3CG_HUMAN Fu	444345 1c	1E8Z	1OKY	3ENE			Sc=6.18136, min distance = 2.531017
PDPK1_HUMAN I	24916751	CDK2_HUMAN Fu	444345 1c	1AQ1	1OKY	3DOG	29.3		Sc=6.13732, min distance = 2.901079
PDPK1_HUMAN I	24916751	CDK2_HUMAN Fu	72271 7-H	1PKD	1OKZ	3DOG	29.3		Sc=6.24915, min distance = 2.674092
PDPK1_HUMAN I	24963049	KAPCA_BOVIN Fu	444345 1c	1STC	1OKY	3E8E	39.18		Sc=6.64642, min distance = 1.969231
PDPK1_HUMAN I	25021197	CDK2_HUMAN Fu	444345 1c	1AQ1	1OKY	3EOC	29.3		Sc=6.21402, min distance = 2.473591
PDPK1_HUMAN I	33113 gam	IRAK4_HUMAN Fu	444345 1c	2NRY	1OKY	2OID	38.55		Sc=5.6905, min distance = 2.0949959
PDPK1_HUMAN I	3540 1yds	KAPCA_BOVIN Fu	5957 Aden	1Q24	2BIY	1YDS	39.18		Sc=5.83015, min distance = 2.732625
PDPK1_HUMAN I	3547 Fasu	KAPCA_BOVIN Fu	5957 Aden	1Q24	2BIY	1Q8W	39.18		Sc=5.89549, min distance = 2.630202
PDPK1_HUMAN I	36735 Gpp	Q9RVK2_DEIRA I	5957 Aden	1SU2	2BIY	1SZ3		0.8	Sc=6.43826, min distance = 2.045521

PDPK1_HUMAN I	4369136 C	CDK2_HUMAN Fu	444345 1c	1AQ1	1OKY	1DM2	29.3		Sc=6.16474, min distance = 2.874029
PDPK1_HUMAN I	4369136 C	CDK2_HUMAN Fu	72271 7-H	1PKD	1OKZ	1DM2	29.3		Sc=6.21983, min distance = 2.553463
PDPK1_HUMAN I	444564 AD	BIOD_ECOLI Fu	5957 Aden	1A82	2BIY	1BS1		0.91	Sc=5.86312, min distance = 2.240554
PDPK1_HUMAN I	444852 CI	ATPA1_BOVIN Fu	5957 Aden	2V7Q	2BIY	1COW		0.91	Sc=5.96189, min distance = 1.688765
PDPK1_HUMAN I	444852 CI	ATPB_BOVIN Fu	5957 Aden	2V7Q	2BIY	1COW		0.91	Sc=5.96168, min distance = 1.929569
PDPK1_HUMAN I	445840 di	CDK2_HUMAN Fu	72271 7-H	1PKD	1OKZ	1GII	29.3		Sc=6.29501, min distance = 2.187994
PDPK1_HUMAN I	446704 LS	CDK2_HUMAN Fu	444345 1c	1AQ1	1OKY	1KE6	29.3		Sc=6.22853, min distance = 2.797918
PDPK1_HUMAN I	446704 LS	CDK2_HUMAN Fu	72271 7-H	1PKD	1OKZ	1KE6	29.3		Sc=6.33398, min distance = 2.145707
PDPK1_HUMAN I	447649 1c	CDK2_HUMAN Fu	72271 7-H	1PKD	1OKZ	1OI9	29.3		Sc=6.38755, min distance = 2.455404
PDPK1_HUMAN I	447654 CI	CDK2_HUMAN Fu	444345 1c	1AQ1	1OKY	1OIT	29.3		Sc=6.13824, min distance = 2.278944
PDPK1_HUMAN I	447655 1c	CDK2_HUMAN Fu	444345 1c	1AQ1	1OKY	1OIU	29.3		Sc=6.25142, min distance = 2.919340
PDPK1_HUMAN I	447655 1c	CDK2_HUMAN Fu	72271 7-H	1PKD	1OKZ	1OIU	29.3		Sc=6.26271, min distance = 2.595348
PDPK1_HUMAN I	447656 1c	CDK2_HUMAN Fu	72271 7-H	1PKD	1OKZ	1OIY	29.3		Sc=6.4613, min distance = 2.4035777
PDPK1_HUMAN I	447962 1p	CDK2_HUMAN Fu	444345 1c	1AQ1	1OKY	2C5N	29.3		Sc=6.11074, min distance = 2.470623
PDPK1_HUMAN I	447962 1p	CDK2_HUMAN Fu	72271 7-H	1PKD	1OKZ	2C5N	29.3		Sc=6.10119, min distance = 2.496265
PDPK1_HUMAN I	448043 2g	KAPCA_BOVIN Fu	444345 1c	1STC	1OKY	1Q8U	39.18		Sc=5.9518, min distance = 2.8233069
PDPK1_HUMAN I	448043 2g	KAPCA_BOVIN Fu	5957 Aden	1Q24	2BIY	1Q8U	39.18		Sc=5.95606, min distance = 2.596740
PDPK1_HUMAN I	448170 CI	LCK_HUMAN Ful	444345 1c	1QPD	1OKY	1QPC	26.62		Sc=5.86419, min distance = 2.213155
PDPK1_HUMAN I	449240 1y	KAPCA_BOVIN Fu	5957 Aden	1Q24	2BIY	1YDR	39.18		Sc=5.89549, min distance = 2.704396
PDPK1_HUMAN I	4565 1h1r	CDK2_HUMAN Fu	444345 1c	1AQ1	1OKY	1H1R	29.3		Sc=6.22641, min distance = 2.972956
PDPK1_HUMAN I	4565 1h1r	CDK2_HUMAN Fu	72271 7-H	1PKD	1OKZ	1H1R	29.3		Sc=5.99259, min distance = 2.716207
PDPK1_HUMAN I	4566 1h1s	CDK2_HUMAN Fu	444345 1c	1AQ1	1OKY	1H1S	29.3		Sc=6.26593, min distance = 2.201397
PDPK1_HUMAN I	4566 1h1s	CDK2_HUMAN Fu	72271 7-H	1PKD	1OKZ	1H1S	29.3		Sc=6.27842, min distance = 2.667328
PDPK1_HUMAN I	5281680 Q	PIM1_HUMAN Fu	444345 1c	1YHS	1OKY	2O64	25.47		Sc=6.39441, min distance = 2.558277
PDPK1_HUMAN I	5288016 6	CDK2_HUMAN Fu	444345 1c	1AQ1	1OKY	2B53	29.3		Sc=5.86249, min distance = 2.248249
PDPK1_HUMAN I	5288016 6	CDK2_HUMAN Fu	72271 7-H	1PKD	1OKZ	2B53	29.3		Sc=6.14598, min distance = 2.408363
PDPK1_HUMAN I	5288708 1	CDK2_HUMAN Fu	72271 7-H	1PKD	1OKZ	1KE5	29.3		Sc=6.1181, min distance = 1.9541893
PDPK1_HUMAN I	5288710 C	CDK2_HUMAN Fu	444345 1c	1AQ1	1OKY	1KE7	29.3		Sc=6.31804, min distance = 2.486863
PDPK1_HUMAN I	5288710 C	CDK2_HUMAN Fu	72271 7-H	1PKD	1OKZ	1KE7	29.3		Sc=6.33224, min distance = 2.469485
PDPK1_HUMAN I	5327121 I	PIM1_HUMAN Fu	444345 1c	1YHS	1OKY	2C3I	25.47		Sc=6.10167, min distance = 2.623815
PDPK1_HUMAN I	5327123 I	CHK1_HUMAN Fu	444345 1c	1NVR	1OKY	2C3L	33.65		Sc=6.13569, min distance = 2.503262
PDPK1_HUMAN I	5327130 C	CDK2_HUMAN Fu	72271 7-H	1PKD	1OKZ	2C68	29.3		Sc=6.19484, min distance = 2.043109
PDPK1_HUMAN I	5327131 T	CDK2_HUMAN Fu	72271 7-H	1PKD	1OKZ	2C69	29.3		Sc=6.24458, min distance = 2.127846
PDPK1_HUMAN I	5327133 C	CDK2_HUMAN Fu	72271 7-H	1PKD	1OKZ	2C6K	29.3		Sc=6.31163, min distance = 2.265735
PDPK1_HUMAN I	5327135 D	CDK2_HUMAN Fu	72271 7-H	1PKD	1OKZ	2C6M	29.3		Sc=6.2375, min distance = 2.3246685
PDPK1_HUMAN I	5331010 c	CDK2_HUMAN Fu	444345 1c	1AQ1	1OKY	2BKZ	29.3		Sc=6.19059, min distance = 2.505216
PDPK1_HUMAN I	5331010 c	CDK2_HUMAN Fu	72271 7-H	1PKD	1OKZ	2BKZ	29.3		Sc=6.2054, min distance = 2.5019290

PDPK1_HUMAN I	5494414 C	CDK2_HUMAN Fu	72271 7-H	1PKD	1OKZ	2A0C	29.3		Sc=6.40015, min distance = 2.11228(
PDPK1_HUMAN I	5687 1di8	CDK2_HUMAN Fu	444345 1c	1AQ1	1OKY	1DI8	29.3		Sc=6.22317, min distance = 2.54827
PDPK1_HUMAN I	6022 Ader	BIOD_ECOLI Fu	5957 Aden	1A82	2BIY	1DAD		0.99	Sc=5.85826, min distance = 2.53167
PDPK1_HUMAN I	6022 Ader	CLCN5_HUMAN Fu	5957 Aden	2J9L	2BIY	2JA3		0.99	Sc=6.32679, min distance = 2.16152(
PDPK1_HUMAN I	6022 Ader	HSP7F_YEAST Fu	5957 Aden	3D2F	2BIY	3C7N		0.99	Sc=5.92647, min distance = 2.38831
PDPK1_HUMAN I	6022 Ader	MUTS_ECOLI Fu	5957 Aden	1W7A	2BIY	1OH7		0.99	Sc=5.85951, min distance = 1.94934
PDPK1_HUMAN I	6022 Ader	SKY1_YEAST Fu	5957 Aden	1Q97	2BIY	1Q8Y	27.5	0.99	Sc=5.86285, min distance = 2.24982
PDPK1_HUMAN I	6022 Ader	Y059_METJA Fu	5957 Aden	2J9C	2BIY	2J9D		0.99	Sc=5.86827, min distance = 2.41774
PDPK1_HUMAN I	6083 ader	ASSY_THET8 Fu	5957 Aden	1J1Z	2BIY	1J20		0.98	Sc=6.29882, min distance = 1.52612
PDPK1_HUMAN I	6083 ader	PURP_METJA Fu	5957 Aden	2R7L	2BIY	2R7M		0.98	Sc=6.23033, min distance = 2.73580
PDPK1_HUMAN I	6083 ader	PURP_PYRFU Fu	5957 Aden	2R86	2BIY	2R85		0.98	Sc=5.81796, min distance = 2.57438
PDPK1_HUMAN I	6083 ader	Y059_METJA Fu	5957 Aden	2J9C	2BIY	2J9D		0.98	Sc=5.78538, min distance = 2.46371
PDPK1_HUMAN I	60961 ade	PIM1_HUMAN Fu	444345 1c	1YHS	1OKY	1YI4	25.47		Sc=6.12606, min distance = 2.18611
PDPK1_HUMAN I	6102670	CDK2_HUMAN Fu	444345 1c	1AQ1	1OKY	1YKR	29.3		Sc=6.25726, min distance = 2.17405
PDPK1_HUMAN I	6420138 2	CDK2_HUMAN Fu	444345 1c	1AQ1	1OKY	2UUE	29.3		Sc=6.2584, min distance = 2.057632
PDPK1_HUMAN I	6420138 2	CDK2_HUMAN Fu	72271 7-H	1PKD	1OKZ	2UUE	29.3		Sc=6.26075, min distance = 2.56008
PDPK1_HUMAN I	6420139 C	CDK2_HUMAN Fu	72271 7-H	1PKD	1OKZ	2C5V	29.3		Sc=6.11253, min distance = 2.40466
PDPK1_HUMAN I	6420140 2	CDK2_HUMAN Fu	72271 7-H	1PKD	1OKZ	2C5Y	29.3		Sc=6.45592, min distance = 2.17165
PDPK1_HUMAN I	6539118 g	CDK2_HUMAN Fu	444345 1c	1AQ1	1OKY	1FVV	29.3		Sc=6.33188, min distance = 2.11018
PDPK1_HUMAN I	6540273 2	CHK1_HUMAN Fu	444345 1c	1NVR	1OKY	2CGV	33.65		Sc=6.15251, min distance = 2.70815
PDPK1_HUMAN I	656971 CI	CDK2_HUMAN Fu	444345 1c	1AQ1	1OKY	1Y8Y	29.3		Sc=6.01336, min distance = 2.40060
PDPK1_HUMAN I	656971 CI	CDK2_HUMAN Fu	72271 7-H	1PKD	1OKZ	1Y8Y	29.3		Sc=6.02151, min distance = 2.29947
PDPK1_HUMAN I	657072 RY	CDK2_HUMAN Fu	72271 7-H	1PKD	1OKZ	2BHH	29.3		Sc=6.42603, min distance = 2.48304(
PDPK1_HUMAN I	6830 guar	Q381M1_9TRYP I	5957 Aden	2Q0D	2BIY	2Q0E		0.8	Sc=5.91273, min distance = 2.38519
PDPK1_HUMAN I	6852207 M	KAPCA_BOVIN Fu	444345 1c	1STC	1OKY	2GNI	39.18		Sc=5.91243, min distance = 2.27844
PDPK1_HUMAN I	91532 AMF	INSR_HUMAN Fu	5957 Aden	3BU5	2BIY	1I44	24.38	0.99	Sc=5.95775, min distance = 2.15408
PDPK1_HUMAN I	9549254 2	CDK2_HUMAN Fu	444345 1c	1AQ1	1OKY	2IW6	29.3		Sc=6.44308, min distance = 2.13837
PDPK1_HUMAN I	9817550 V	CDK2_HUMAN Fu	444345 1c	1AQ1	1OKY	3BHV	29.3		Sc=6.34034, min distance = 2.50439
PDPK1_HUMAN I	9817550 V	CDK2_HUMAN Fu	72271 7-H	1PKD	1OKZ	3BHV	29.3		Sc=6.32732, min distance = 1.92670
PDR_BURCE Fu	5326566 I	BLVRB_HUMAN Fu	444243 FA	1HE4	2PIA	1HE5		0.82	Sc=6.33577, min distance = 2.70598
PDX3_YEAST Fu	5326566 I	BLVRB_HUMAN Fu	444243 FA	1HE4	1CI0	1HE5		0.82	Sc=6.05144, min distance = 2.61821
PDX3_YEAST Fu	5326566 I	Q9WZW1_THEMA I	444243 FA	1T6Y	1CI0	1S4M		0.82	Sc=6.15983, min distance = 2.67793
PDXB_PSEAE Fu	11957403	INHA_MYCTU Fu	5893 nadi	2H7I	2O4C	2H7M			Sc=6.02129, min distance = 2.00097
PDXB_PSEAE Fu	11987786	LDH_PLAFD Ful	5893 nadi	1T2D	2O4C	1T2E		0.77	Sc=6.31186, min distance = 2.63333
PDXB_PSEAE Fu	123927 A3	Q9GT92_CRYPV I	5893 nadi	2FM3	2O4C	2EWD		0.97	Sc=6.32316, min distance = 2.45611
PDXB_PSEAE Fu	439153 Di	ADH1E_HORSE Fu	5893 nadi	1MGO	2O4C	2JHF		0.79	Sc=6.30628, min distance = 2.47369
PDXB_PSEAE Fu	439153 Di	CTBP1_RAT Ful	5893 nadi	1HKU	2O4C	1HL3		0.79	Sc=6.75575, min distance = 2.00373(

PDXB_PSEAE Fu	440516 CI	MDH_THETH Ful	5893 nadi	1BMD	2O4C	1BDM	0.79	Sc=6.33674, min distance = 2.57902(
PDXB_PSEAE Fu	445794 AD	ADHX_HUMAN Fu	5893 nadi	2FZW	2O4C	2FZE		Sc=6.14151, min distance = 2.857674
PDXH_ECOLI Fu	5326566 l	BLVRB_HUMAN Fu	444243 FA	1HE4	1DNL	1HE5	0.82	Sc=6.13555, min distance = 2.56533
PDXH_ECOLI Fu	5326566 l	Q9WZW1_THEMA I	444243 FA	1T6Y	1DNL	1S4M	0.82	Sc=6.18679, min distance = 2.022756
PDXJ_ECOLI Fu	1983 acet	PA28_DABRR Ful	439162 gl	1Y38	1IXO	2DPZ		Sc=5.88541, min distance = 0.98630(
PDXJ_ECOLI Fu	332 4-vir	PA28_DABRR Ful	439162 gl	1Y38	1IXO	2QU9		Sc=5.60726, min distance = 2.175422
PDXJ_ECOLI Fu	668 Glyce	CGGR_BACSU Ful	439162 gl	2OKG	1IXO	3BXF	0.76	Sc=5.61353, min distance = 1.88753(
PDXJ_ECOLI Fu	7478 p-Ar	PA28_DABRR Ful	439162 gl	1Y38	1IXO	1SV3		Sc=5.69643, min distance = 1.553997
PDXK_ECOLI Fu	11608401	KAPCA_BOVIN Fu	5957 Aden	1Q24	2DDO	2UVY		Sc=6.20364, min distance = 2.28116(
PDXK_ECOLI Fu	3547 Fasu	KAPCA_BOVIN Fu	5957 Aden	1Q24	2DDO	1Q8W		Sc=5.89708, min distance = 2.34611(
PDXK_ECOLI Fu	448043 2q	KAPCA_BOVIN Fu	5957 Aden	1Q24	2DDO	1Q8U		Sc=6.00229, min distance = 2.006795
PDXK_ECOLI Fu	449240 1y	KAPCA_BOVIN Fu	5957 Aden	1Q24	2DDO	1YDR		Sc=5.92505, min distance = 2.144502
PDXK_ECOLI Fu	6022 Aden	FAK1_HUMAN Ful	5957 Aden	2IJM	2DDO	1MP8	0.99	Sc=5.83958, min distance = 2.541994
PDXK_ECOLI Fu	6022 Aden	Y059_METJA Ful	5957 Aden	2J9C	2DDO	2J9D	0.99	Sc=5.8788, min distance = 2.3167738
PDXK_ECOLI Fu	6852207 M	KAPCA_BOVIN Fu	5957 Aden	1Q24	2DDO	2GNI		Sc=5.92505, min distance = 2.44584
PDXK_ECOLI Fu	91532 AMF	Q5SJV7_THET8 I	5957 Aden	2Z08	2DDO	2Z09	0.99	Sc=5.94191, min distance = 2.287356
PDXK_HUMAN Fu	11608401	KAPCA_BOVIN Fu	5957 Aden	1Q24	2YXU	2UVY		Sc=6.17797, min distance = 2.337129
PDXK_HUMAN Fu	11957393	FAK1_HUMAN Ful	5957 Aden	2IJM	2YXU	2ETM		Sc=6.37359, min distance = 1.873834
PDXK_HUMAN Fu	129236 2c	CKI1_SCHPO Ful	5957 Aden	1CSN	2YXU	2CSN		Sc=5.77165, min distance = 2.166028
PDXK_HUMAN Fu	16040273	IRAK4_HUMAN Fu	33113 gam	2OID	2AJP	2OIC		Sc=6.36357, min distance = 1.161744
PDXK_HUMAN Fu	24779674	CSK2A_MAIZE Fu	33113 gam	1LP4	2AJP	2PVH		Sc=6.143, min distance = 2.13635039
PDXK_HUMAN Fu	24779675	CSK2A_MAIZE Fu	33113 gam	1LP4	2AJP	2PVJ		Sc=6.31512, min distance = 1.872082
PDXK_HUMAN Fu	24779676	CSK2A_MAIZE Fu	33113 gam	1LP4	2AJP	2PVK		Sc=6.22288, min distance = 2.025427
PDXK_HUMAN Fu	24779677	CSK2A_MAIZE Fu	33113 gam	1LP4	2AJP	2PVL		Sc=6.28836, min distance = 1.896475
PDXK_HUMAN Fu	3064778 H	ROCK1_HUMAN Fu	33113 gam	2V55	2AJP	2ETK		Sc=6.01208, min distance = 2.081362
PDXK_HUMAN Fu	3540 1yds	KAPCA_BOVIN Fu	5957 Aden	1Q24	2YXU	1YDS		Sc=5.83721, min distance = 1.93588
PDXK_HUMAN Fu	3547 Fasu	KAPCA_BOVIN Fu	5957 Aden	1Q24	2YXU	1Q8W		Sc=5.91836, min distance = 2.13624
PDXK_HUMAN Fu	3547 Fasu	ROCK1_HUMAN Fu	33113 gam	2V55	2AJP	2ESM		Sc=5.90944, min distance = 2.204825
PDXK_HUMAN Fu	440317 AT	PURT_ECOLI Ful	5957 Aden	1KJ8	2YXU	1KJJ	0.99	Sc=5.98123, min distance = 1.15804
PDXK_HUMAN Fu	444345 1c	IRAK4_HUMAN Fu	33113 gam	2OID	2AJP	2NRY		Sc=6.36297, min distance = 1.20672
PDXK_HUMAN Fu	444564 AD	BIOD_ECOLI Ful	5957 Aden	1A82	2YXU	1BS1	0.91	Sc=5.848, min distance = 2.5513265
PDXK_HUMAN Fu	444852 CI	ATPA1_BOVIN Fu	5957 Aden	2V7Q	2YXU	1COW	0.91	Sc=6.42272, min distance = 1.82643(
PDXK_HUMAN Fu	448005 CI	GSK3B_HUMAN Fu	33113 gam	1J1B	2AJP	1Q3W		Sc=6.20063, min distance = 2.61863
PDXK_HUMAN Fu	448042 2e	KAPCA_BOVIN Fu	5957 Aden	1Q24	2YXU	1Q8T		Sc=6.01356, min distance = 1.948314
PDXK_HUMAN Fu	448042 2e	ROCK1_HUMAN Fu	33113 gam	2V55	2AJP	2ETR		Sc=5.99448, min distance = 2.12913(
PDXK_HUMAN Fu	448043 2q	KAPCA_BOVIN Fu	5957 Aden	1Q24	2YXU	1Q8U		Sc=6.00003, min distance = 2.156365
PDXK_HUMAN Fu	448043 2q	ROCK1_HUMAN Fu	33113 gam	2V55	2AJP	3D9V		Sc=5.96255, min distance = 2.192314

PDXK_HUMAN Fι	449240 1y	KAPCA_BOVIN Fι	5957 Aden	1Q24	2YXU	1YDR			Sc=5.91243, min distance = 2.11813(
PDXK_HUMAN Fι	456214 pu	SRC_HUMAN Ful:	33113 gam	2SRC	2AJP	1YOM			Sc=6.38365, min distance = 1.48957:
PDXK_HUMAN Fι	5287844 i	GSK3B_HUMAN Fι	33113 gam	1J1B	2AJP	1UV5			Sc=6.16734, min distance = 2.27218
PDXK_HUMAN Fι	5326739 i	GSK3B_HUMAN Fι	33113 gam	1J1B	2AJP	1Q41			Sc=6.23585, min distance = 2.46224
PDXK_HUMAN Fι	5327121 I	PIM1_HUMAN Fu:	33113 gam	1XR1	2AJP	2C3I			Sc=6.13822, min distance = 2.55631
PDXK_HUMAN Fι	5327148 C	IPKA_RABIT Fu:	33113 gam	1CDK	2AJP	2ERZ			Sc=6.15325, min distance = 2.16481
PDXK_HUMAN Fι	6022 Aden	ARSA1_ECOLX Fι	5957 Aden	1II0	2YXU	1IHU		0.99	Sc=5.89903, min distance = 2.48160
PDXK_HUMAN Fι	6022 Aden	BIOD_ECOLI Fu:	5957 Aden	1A82	2YXU	1DAD		0.99	Sc=5.85954, min distance = 2.36791
PDXK_HUMAN Fι	6022 Aden	CLCN5_HUMAN Fι	5957 Aden	2J9L	2YXU	2JA3		0.99	Sc=5.95286, min distance = 1.67294
PDXK_HUMAN Fι	6022 Aden	DDL_THET8 Ful:	5957 Aden	2ZDQ	2YXU	2ZDH		0.99	Sc=5.90232, min distance = 2.04008
PDXK_HUMAN Fι	6022 Aden	KTHY_HUMAN Fu:	5957 Aden	1E2Q	2YXU	1NN3		0.99	Sc=5.85406, min distance = 2.02679
PDXK_HUMAN Fι	6022 Aden	MUTS_ECOLI Fu:	5957 Aden	1W7A	2YXU	1OH7		0.99	Sc=5.88807, min distance = 2.55965
PDXK_HUMAN Fι	6022 Aden	PURP_METJA Fu:	5957 Aden	2R7L	2YXU	2R7N		0.99	Sc=5.8653, min distance = 2.195465
PDXK_HUMAN Fι	6022 Aden	Q72H90_THET2 I	5957 Aden	2BEK	2YXU	2BEJ		0.99	Sc=5.87548, min distance = 2.18555
PDXK_HUMAN Fι	6022 Aden	RK_BOVIN Full:	5957 Aden	3C4W	2YXU	3C4Z		0.99	Sc=5.86071, min distance = 2.46455
PDXK_HUMAN Fι	6022 Aden	Y059_METJA Fu:	5957 Aden	2J9C	2YXU	2J9D		0.99	Sc=6.3392, min distance = 2.644000
PDXK_HUMAN Fι	6083 aden	Y059_METJA Fu:	5957 Aden	2J9C	2YXU	2J9D		0.98	Sc=6.23405, min distance = 2.29699
PDXK_HUMAN Fι	6176 Cyti	PYRI_ECOLI Fu:	5957 Aden	7AT1	2YXU	2FZC			Sc=5.92217, min distance = 1.40880(
PDXK_HUMAN Fι	6338562 C	MYS2_DICDI Fu:	5957 Aden	1FMW	2YXU	1D0Y			Sc=6.15714, min distance = 0
PDXK_HUMAN Fι	6338563 C	MYS2_DICDI Fu:	33113 gam	1MMN	2AJP	1D0Z			Sc=6.18879, min distance = 2.49626
PDXK_HUMAN Fι	6830 guar	Q381M1_9TRYP I	5957 Aden	2Q0D	2YXU	2Q0E		0.8	Sc=5.98112, min distance = 2.04844
PDXK_HUMAN Fι	91532 AME	KIF1A_MOUSE Fι	33113 gam	1VFV	2AJP	1I6I		0.99	Sc=6.0153, min distance = 1.985071
PDXK_HUMAN Fι	91532 AME	NIFH1_AZOVI Fι	5957 Aden	2C8V	2YXU	2AFK		0.99	Sc=6.01611, min distance = 1.99242
PDXK_HUMAN Fι	9549303 2	STK6_HUMAN Fu:	33113 gam	2DWB	2AJP	2NP8			Sc=6.51111, min distance = 1.79504
PDXK_SHEEP Fι	11708454	KAPCA_BOVIN Fι	5957 Aden	1Q24	1LHR	2VNW			Sc=6.0788, min distance = 2.021045
PDXK_SHEEP Fι	129236 2c	CKI1_SCHPO Fu:	5957 Aden	1CSN	1LHR	2CSN			Sc=5.78761, min distance = 2.07399
PDXK_SHEEP Fι	1540 1h1q	CDK2_HUMAN Fu:	160355 rc	3DDQ	1YGK	1H1Q		0.76	Sc=6.32677, min distance = 2.09418
PDXK_SHEEP Fι	16046126	CDK2_HUMAN Fu:	160355 rc	3DDQ	1YGK	2DS1			Sc=6.32223, min distance = 2.03352
PDXK_SHEEP Fι	16214823	CDK2_HUMAN Fu:	160355 rc	3DDQ	1YGK	2UZB			Sc=6.06947, min distance = 2.29070
PDXK_SHEEP Fι	16214825	CDK2_HUMAN Fu:	160355 rc	3DDQ	1YGK	2UZE			Sc=6.24634, min distance = 2.39325
PDXK_SHEEP Fι	16214826	CDK2_HUMAN Fu:	160355 rc	3DDQ	1YGK	2UZL			Sc=6.12458, min distance = 2.11513
PDXK_SHEEP Fι	16214827	CDK2_HUMAN Fu:	160355 rc	3DDQ	1YGK	2UZN			Sc=5.95702, min distance = 2.40337
PDXK_SHEEP Fι	16214830	CDK2_HUMAN Fu:	160355 rc	3DDQ	1YGK	2V0D			Sc=5.86101, min distance = 2.53954
PDXK_SHEEP Fι	1707 1fvt	CDK2_HUMAN Fu:	160355 rc	3DDQ	1YGK	1FVT			Sc=6.06269, min distance = 2.14851
PDXK_SHEEP Fι	23653516	CDK2_HUMAN Fu:	160355 rc	3DDQ	1YGK	2R3G			Sc=6.23771, min distance = 2.09511
PDXK_SHEEP Fι	23727981	CDK2_HUMAN Fu:	160355 rc	3DDQ	1YGK	3BHT			Sc=6.17768, min distance = 2.28973
PDXK_SHEEP Fι	23727982	CDK2_HUMAN Fu:	160355 rc	3DDQ	1YGK	3BHU			Sc=6.22933, min distance = 2.24502

PDXK_SHEEP F	24864077	CDK2_HUMAN Fu	160355 rc	3DDQ	1YGK	2VTN		Sc=6.2226, min distance = 2.309122
PDXK_SHEEP F	24864078	CDK2_HUMAN Fu	160355 rc	3DDQ	1YGK	2VTO		Sc=6.30614, min distance = 2.455570
PDXK_SHEEP F	24864080	CDK2_HUMAN Fu	160355 rc	3DDQ	1YGK	2VTR		Sc=5.87387, min distance = 2.54400
PDXK_SHEEP F	24916751	CDK2_HUMAN Fu	160355 rc	3DDQ	1YGK	3DOG	0.8	Sc=6.36182, min distance = 2.20165
PDXK_SHEEP F	25021197	CDK2_HUMAN Fu	160355 rc	3DDQ	1YGK	3EOC		Sc=6.34759, min distance = 2.07646
PDXK_SHEEP F	2608 1jsv	CDK2_HUMAN Fu	160355 rc	3DDQ	1YGK	1JSV		Sc=5.88718, min distance = 1.60005
PDXK_SHEEP F	33113 gam	IGF1R_HUMAN F	91532 AMP	1K3A	1RFT	1JQH	0.99	Sc=5.9636, min distance = 2.121218
PDXK_SHEEP F	33113 gam	MURC_HAEIN Fu	91532 AMP	1GQY	1RFT	1P3D	0.99	Sc=5.90466, min distance = 2.10829
PDXK_SHEEP F	33113 gam	PURT_ECOLI Fu	91532 AMP	1KJI	1RFT	1EYZ	0.99	Sc=5.95143, min distance = 2.33143
PDXK_SHEEP F	3540 1yds	KAPCA_BOVIN F	5957 Aden	1Q24	1LHR	1YDS		Sc=5.81956, min distance = 2.18875
PDXK_SHEEP F	3547 Fasu	KAPCA_BOVIN F	5957 Aden	1Q24	1LHR	1Q8W		Sc=5.89549, min distance = 2.30966
PDXK_SHEEP F	443200 C1	ACK1_HUMAN Fu	91532 AMP	1U54	1RFT	1U4D		Sc=5.73924, min distance = 2.06390
PDXK_SHEEP F	444564 AD	BIOD_ECOLI Fu	5957 Aden	1A82	1LHR	1BS1	0.91	Sc=5.85289, min distance = 2.12208
PDXK_SHEEP F	444564 AD	BIOD_ECOLI Fu	91532 AMP	1DAG	1RFT	1BS1	0.91	Sc=5.85224, min distance = 2.14937
PDXK_SHEEP F	444564 AD	MYS2_DICDI Fu	5957 Aden	1FMW	1LHR	1W9I	0.91	Sc=5.84665, min distance = 0
PDXK_SHEEP F	445840 di	CDK2_HUMAN Fu	160355 rc	3DDQ	1YGK	1GII		Sc=6.27806, min distance = 2.02704
PDXK_SHEEP F	445840 di	CDK2_HUMAN Fu	6022 Aden	1GY3	1RFU	1GII		Sc=6.31734, min distance = 1.94481
PDXK_SHEEP F	445940 O6	CDK2_HUMAN Fu	6022 Aden	1GY3	1RFU	1GZ8		Sc=6.10488, min distance = 2.08113
PDXK_SHEEP F	445966 O6	CDK2_HUMAN Fu	160355 rc	3DDQ	1YGK	1H0V		Sc=6.10946, min distance = 2.12152
PDXK_SHEEP F	446704 LS	CDK2_HUMAN Fu	160355 rc	3DDQ	1YGK	1KE6		Sc=6.23088, min distance = 2.23106
PDXK_SHEEP F	447649 1o	CDK2_HUMAN Fu	160355 rc	3DDQ	1YGK	1OI9		Sc=6.32903, min distance = 2.07289
PDXK_SHEEP F	447652 Im	CDK2_HUMAN Fu	160355 rc	3DDQ	1YGK	1OIQ	0.77	Sc=6.16783, min distance = 1.91120
PDXK_SHEEP F	447654 CI	CDK2_HUMAN Fu	160355 rc	3DDQ	1YGK	1OIT		Sc=6.2186, min distance = 1.632256
PDXK_SHEEP F	447840 CI	PHKG1_RABIT F	5957 Aden	1QL6	1LHR	1PHK		Sc=5.93013, min distance = 2.33817
PDXK_SHEEP F	447956 1p	CDK2_HUMAN Fu	160355 rc	3DDQ	1YGK	2C5O		Sc=5.87048, min distance = 2.00380
PDXK_SHEEP F	447962 1p	CDK2_HUMAN Fu	160355 rc	3DDQ	1YGK	2C5N		Sc=6.0933, min distance = 2.272699
PDXK_SHEEP F	447967 CI	CDK2_HUMAN Fu	6022 Aden	1GY3	1RFU	1PYE		Sc=6.35384, min distance = 1.72831
PDXK_SHEEP F	448005 CI	GSK3B_HUMAN F	6022 Aden	1J1C	1RFU	1Q3W		Sc=6.28487, min distance = 2.11011
PDXK_SHEEP F	448042 2e	KAPCA_BOVIN F	5957 Aden	1Q24	1LHR	1Q8T		Sc=5.9768, min distance = 2.017238
PDXK_SHEEP F	448043 2g	KAPCA_BOVIN F	5957 Aden	1Q24	1LHR	1Q8U		Sc=5.98219, min distance = 2.20794
PDXK_SHEEP F	449088 1v	CDK2_HUMAN Fu	160355 rc	3DDQ	1YGK	1VYZ		Sc=6.06514, min distance = 2.01314
PDXK_SHEEP F	449240 1y	KAPCA_BOVIN F	5957 Aden	1Q24	1LHR	1YDR		Sc=5.89401, min distance = 1.98059
PDXK_SHEEP F	4564 1e1v	CDK2_HUMAN Fu	160355 rc	3DDQ	1YGK	1E1V		Sc=6.13938, min distance = 2.13532
PDXK_SHEEP F	4565 1h1r	CDK2_HUMAN Fu	160355 rc	3DDQ	1YGK	1H1R		Sc=6.3212, min distance = 2.125276
PDXK_SHEEP F	4565 1h1r	CDK2_HUMAN Fu	6022 Aden	1GY3	1RFU	1H1R		Sc=6.39144, min distance = 2.53148
PDXK_SHEEP F	4566 1h1s	CDK2_HUMAN Fu	160355 rc	3DDQ	1YGK	1H1S		Sc=6.36505, min distance = 1.82360
PDXK_SHEEP F	5287845 N	CDK2_HUMAN Fu	160355 rc	3DDQ	1YGK	2BHE		Sc=6.18391, min distance = 2.37173

PDXK_SHEEP F	5288017 I	CDK2_HUMAN Fu	160355 rc	3DDQ	1YGK	2B55		Sc=6.42305, min distance = 2.14440
PDXK_SHEEP F	5288017 I	CDK2_HUMAN Fu	6022 Aden	1GY3	1RFU	2B55		Sc=6.56087, min distance = 2.23134
PDXK_SHEEP F	5288708 1	CDK2_HUMAN Fu	160355 rc	3DDQ	1YGK	1KE5		Sc=6.0978, min distance = 2.222884
PDXK_SHEEP F	5288710 C	CDK2_HUMAN Fu	160355 rc	3DDQ	1YGK	1KE7		Sc=6.29044, min distance = 1.93906
PDXK_SHEEP F	5326843 A	CDK5_HUMAN Fu	160355 rc	1UNL	1YGK	1UNG		Sc=6.17606, min distance = 2.15044
PDXK_SHEEP F	5326844 C	CDK2_HUMAN Fu	160355 rc	3DDQ	1YGK	1URW		Sc=6.42988, min distance = 2.23798
PDXK_SHEEP F	5327097 2	CDK2_HUMAN Fu	160355 rc	3DDQ	1YGK	2BTS		Sc=5.86216, min distance = 1.85280
PDXK_SHEEP F	5327124	CDK2_HUMAN Fu	160355 rc	3DDQ	1YGK	2C4G		Sc=6.2322, min distance = 2.191873
PDXK_SHEEP F	5327131 T	CDK2_HUMAN Fu	160355 rc	3DDQ	1YGK	2C69		Sc=6.14047, min distance = 2.10357
PDXK_SHEEP F	5327134 C	CDK2_HUMAN Fu	160355 rc	3DDQ	1YGK	2C6L		Sc=6.32739, min distance = 2.17895
PDXK_SHEEP F	5331010 c	CDK2_HUMAN Fu	160355 rc	3DDQ	1YGK	2BKZ		Sc=6.28521, min distance = 2.14785
PDXK_SHEEP F	5687 1di8	CDK2_HUMAN Fu	160355 rc	3DDQ	1YGK	1DI8		Sc=6.09896, min distance = 2.06525
PDXK_SHEEP F	6338561 C	MYS2_DICDI Fu	6022 Aden	1VOM	1RFU	1D0X		Sc=6.22685, min distance = 2.28242
PDXK_SHEEP F	6338564 C	MYS2_DICDI Fu	6022 Aden	1VOM	1RFU	1D1A		Sc=6.30402, min distance = 2.17157
PDXK_SHEEP F	6420138 2	CDK2_HUMAN Fu	160355 rc	3DDQ	1YGK	2UUE		Sc=6.27341, min distance = 2.05680
PDXK_SHEEP F	6420139 C	CDK2_HUMAN Fu	160355 rc	3DDQ	1YGK	2C5V		Sc=6.09286, min distance = 2.25398
PDXK_SHEEP F	656971 CI	CDK2_HUMAN Fu	160355 rc	3DDQ	1YGK	1Y8Y		Sc=5.90344, min distance = 2.13166
PDXK_SHEEP F	680935 py	CDK2_HUMAN Fu	160355 rc	3DDQ	1YGK	2VTL		Sc=5.95481, min distance = 2.10349
PDXK_SHEEP F	6830 guan	Q381M1_9TRYP	5957 Aden	2Q0D	1LHR	2Q0E	0.8	Sc=5.95327, min distance = 2.11569
PDXK_SHEEP F	6852207 M	KAPCA_BOVIN F	5957 Aden	1Q24	1LHR	2GNI		Sc=5.88721, min distance = 2.24866
PDXK_SHEEP F	6918710 5	CDK2_HUMAN Fu	160355 rc	3DDQ	1YGK	3EJ1		Sc=6.09574, min distance = 2.27703
PDXK_SHEEP F	9547890 I	CDK2_HUMAN Fu	160355 rc	3DDQ	1YGK	1W8C		Sc=6.22288, min distance = 2.48124
PDXK_SHEEP F	9547890 I	CDK2_HUMAN Fu	6022 Aden	1GY3	1RFU	1W8C		Sc=6.13953, min distance = 1.78623
PDXK_SHEEP F	9926933 i	CDK2_HUMAN Fu	160355 rc	3DDQ	1YGK	2VTI		Sc=6.1463, min distance = 2.149913
PDXK_SHEEP F	9994066 4	CDK2_HUMAN Fu	160355 rc	3DDQ	1YGK	2VTJ		Sc=5.89422, min distance = 2.34671
PDXT_BACSU F	33032 L-g	SYQ_ECOLI Ful	5961 L-gl	1ZJW	2NV2	1O0C	0.84	Sc=5.745, min distance = 1.8540307
PECR_HUMAN F	100684 9-	RICI_RICCO Fu	190 adeni	2P8N	1YXM	1IL4		Sc=5.84332, min distance = 2.60715
PECR_HUMAN F	11979 5-M	COBT_SALTY Fu	190 adeni	1JH8	1YXM	1JHM		Sc=5.65339, min distance = 2.06619
PECR_HUMAN F	446312 1i	RICI_RICCO Fu	190 adeni	2P8N	1YXM	1IL9		Sc=5.87327, min distance = 2.63521
PECR_HUMAN F	5287547 6	PURR_ECOLI Fu	190 adeni	2PUB	1YXM	2PUA	0.9	Sc=5.7552, min distance = 2.840332
PECR_HUMAN F	5287830 6	Q8RLY5_LACHE	190 adeni	1S2D	1YXM	1S2I	0.93	Sc=5.71776, min distance = 1.65162
PECR_HUMAN F	764 guani	PURR_ECOLI Fu	190 adeni	2PUB	1YXM	1WET		Sc=5.81408, min distance = 2.34762
PECR_HUMAN F	76635 4,5	COBT_SALTY Fu	190 adeni	1JH8	1YXM	1L4F		Sc=5.64738, min distance = 2.08092
PECR_HUMAN F	96253 7-D	RICI_RICCO Fu	190 adeni	2P8N	1YXM	1IL3		Sc=5.83634, min distance = 2.54513
PEM1_PHACH F	11957363	HBA_HUMAN Ful	444124 HE	1NQP	1YYD	1RQA	0.81	Sc=6.81473, min distance = 2.17108
PEM1_PHACH F	11957363	HBB_HUMAN Ful	444124 HE	1NQP	1YYD	1RQA	0.81	Sc=6.81934, min distance = 2.11062
PEM1_PHACH F	11957370	HMOX1_HUMAN F	444124 HE	1OZW	1YYD	1TWN		Sc=6.65823, min distance = 2.01711

PEM1_PHACH Fu	11957371	HMOX1_HUMAN Fu	444124 HE	1OZW	1YYD	1TWR			Sc=6.67228, min distance = 1.94381
PEM1_PHACH Fu	11957385	MYG_PHYCA Ful	444124 HE	1U7R	1YYD	2CMM			Sc=6.23298, min distance = 2.41739
PEM1_PHACH Fu	4369001 C	HBA_HUMAN Ful	444124 HE	1NQP	1YYD	4HHB		0.8	Sc=6.73703, min distance = 2.01743
PEM1_PHACH Fu	444207 He	HBB_HORSE Ful	444124 HE	1Y8I	1YYD	2ZLW		0.99	Sc=6.81333, min distance = 1.52228
PEM1_PHACH Fu	444207 He	PER_COPCI Ful	444124 HE	1LY9	1YYD	1LY8		0.99	Sc=6.78352, min distance = 2.17504
PEM1_PHACH Fu	444522 HE	BFR_ECOLI Ful	444124 HE	1BFR	1YYD	1BCF		0.99	Sc=6.81771, min distance = 2.03678
PEM1_PHACH Fu	444522 HE	HBA_PAGBE Ful	444124 HE	1S5X	1YYD	1PBX		0.99	Sc=6.77454, min distance = 2.52388
PEM1_PHACH Fu	446189 CI	HBG1_HUMAN Fu	444124 HE	1I3E	1YYD	1I3D		0.89	Sc=6.81395, min distance = 2.13437
PER1A_ARMRU I	11957330	CCPR_YEAST Fu	444098 HE	2EUT	7ATJ	1BEQ		0.81	Sc=6.76289, min distance = 2.03512
PER1A_ARMRU I	11957363	HBA_HUMAN Ful	444124 HE	1NQP	1W4W	1RQA		0.81	Sc=6.76846, min distance = 2.02708
PER1A_ARMRU I	11957363	HBB_HUMAN Ful	444098 HE	1J40	7ATJ	1RQA		0.77	Sc=6.72039, min distance = 1.88044
PER1A_ARMRU I	11957363	HBB_HUMAN Ful	444124 HE	1NQP	1W4W	1RQA		0.81	Sc=6.72501, min distance = 2.31610
PER1A_ARMRU I	11957370	HMOX1_HUMAN Fu	444124 HE	1OZW	1W4W	1TWN			Sc=6.66445, min distance = 1.92223
PER1A_ARMRU I	11957371	HMOX1_HUMAN Fu	444124 HE	1OZW	1W4W	1TWR			Sc=6.66002, min distance = 2.35940
PER1A_ARMRU I	11957385	MYG_PHYCA Ful	444098 HE	1A6M	7ATJ	2CMM			Sc=6.2149, min distance = 2.433458
PER1A_ARMRU I	11957385	MYG_PHYCA Ful	444124 HE	1U7R	1W4W	2CMM			Sc=6.0997, min distance = 2.277465
PER1A_ARMRU I	11970219	CCPR_YEAST Fu	444098 HE	2EUT	7ATJ	1BEM		0.83	Sc=6.72103, min distance = 2.06071
PER1A_ARMRU I	11970242	CCPR_YEAST Fu	444098 HE	2EUT	7ATJ	1CPE		0.83	Sc=6.71759, min distance = 2.14031
PER1A_ARMRU I	126994 CI	CCPR_YEAST Fu	444098 HE	2EUT	7ATJ	1Z53		0.77	Sc=6.76109, min distance = 2.64178
PER1A_ARMRU I	4369228 C	PER_COPCI Ful	444124 HE	1LY9	1W4W	1LYC		0.95	Sc=6.81233, min distance = 1.82653
PER1A_ARMRU I	444097 He	HBA_HUMAN Ful	444124 HE	1NQP	1W4W	1RPS		0.95	Sc=6.76983, min distance = 2.10224
PER1A_ARMRU I	444207 He	PER_COPCI Ful	444124 HE	1LY9	1W4W	1LY8		0.99	Sc=6.77383, min distance = 2.02993
PER1A_ARMRU I	446348 CI	HBA_HUMAN Ful	444124 HE	1NQP	1W4W	1IRD		0.9	Sc=6.76919, min distance = 1.93932
PER1A_ARMRU I	447168 ZE	CCPR_YEAST Fu	444098 HE	2EUT	7ATJ	1ML2		0.82	Sc=6.76704, min distance = 2.03511
PER1A_ARMRU I	6420167 M	CPXA_PSEPU Fu	444124 HE	1RE9	1W4W	2FE6		0.89	Sc=6.76704, min distance = 2.01093
PER1_ARAHY Fu	11957353	HBA_HORSE Ful	444124 HE	1Y8I	1SCH	1IWH		0.87	Sc=6.74967, min distance = 2.03341
PER1_ARAHY Fu	11957353	HBB_HORSE Ful	444124 HE	1Y8I	1SCH	1IWH		0.87	Sc=6.76325, min distance = 1.94545
PER1_ARAHY Fu	11957370	HMOX1_HUMAN Fu	444124 HE	1OZW	1SCH	1TWN			Sc=6.5966, min distance = 1.936437
PER1_ARAHY Fu	11957371	HMOX1_HUMAN Fu	444124 HE	1OZW	1SCH	1TWR			Sc=6.60687, min distance = 2.07552
PER1_ARAHY Fu	11957373	CATE_ECOLI Fu	444124 HE	1IPH	1SCH	1P81		0.79	Sc=6.75246, min distance = 1.96467
PER1_ARAHY Fu	11957385	MYG_PHYCA Ful	444124 HE	1U7R	1SCH	2CMM			Sc=6.07823, min distance = 2.28817
PER1_ARAHY Fu	444207 He	COX1_THET8 Fu	444124 HE	1EHK	1SCH	2QPE		0.99	Sc=6.75596, min distance = 2.11775
PER1_ARAHY Fu	444522 HE	GLB1_GLYDI Fu	444124 HE	1JF3	1SCH	1HBG		0.99	Sc=6.73855, min distance = 2.06252
PER1_ARAHY Fu	444522 HE	HBA_PAGBE Ful	444124 HE	1S5X	1SCH	1PBX		0.99	Sc=6.71185, min distance = 2.18876
PER53_ARATH I	11957353	HBB_HORSE Ful	444124 HE	1Y8I	1QO4	1IWH		0.87	Sc=6.76554, min distance = 1.90705
PER53_ARATH I	11957363	HBB_HUMAN Ful	444124 HE	1NQP	1QO4	1RQA		0.81	Sc=6.71622, min distance = 1.65151
PER53_ARATH I	11957365	CCPR_YEAST Fu	444098 HE	2EUT	1PA2	1S73		0.8	Sc=6.80259, min distance = 2.00209

PER53_ARATH	11957370	HMOX1_HUMAN F	444124	HE	1OZW	1QO4	1TWN			Sc=6.59477, min distance = 2.00715
PER53_ARATH	11957371	HMOX1_HUMAN F	444124	HE	1OZW	1QO4	1TWR			Sc=6.60737, min distance = 2.17328
PER53_ARATH	11957373	CATE_ECOLI Fu	444124	HE	1IPH	1QO4	1P81		0.79	Sc=6.7621, min distance = 2.114881
PER53_ARATH	11957385	MYG_PHYCA Ful	444098	HE	1A6M	1PA2	2CMM			Sc=6.10166, min distance = 2.45649
PER53_ARATH	11957385	MYG_PHYCA Ful	444124	HE	1U7R	1QO4	2CMM			Sc=6.09395, min distance = 2.34803
PER53_ARATH	11970219	CCPR_YEAST Fu	444098	HE	2EUT	1PA2	1BEM		0.83	Sc=6.72131, min distance = 2.07262
PER53_ARATH	11970222	CCPR_YEAST Fu	444098	HE	2EUT	1PA2	1BJ9		0.77	Sc=6.83004, min distance = 2.00574
PER53_ARATH	11970242	CCPR_YEAST Fu	444098	HE	2EUT	1PA2	1CPE		0.83	Sc=6.76109, min distance = 2.14714
PER53_ARATH	126994 CI	CCPR_YEAST Fu	444098	HE	2EUT	1PA2	1Z53		0.77	Sc=6.7578, min distance = 2.698170
PER53_ARATH	444522 HE	GLB1_GLYDI Fu	444124	HE	1JF3	1QO4	1HBG		0.99	Sc=6.75286, min distance = 2.02065
PER53_ARATH	444522 HE	HBB_PAGBE Ful	444124	HE	1S5X	1QO4	1PBX		0.99	Sc=6.76456, min distance = 2.10953
PER53_ARATH	446189 CI	HBG1_HUMAN Fu	444124	HE	1I3E	1QO4	1I3D		0.89	Sc=6.76035, min distance = 1.37858
PER53_ARATH	447168 ZE	CCPR_YEAST Fu	444098	HE	2EUT	1PA2	1ML2		0.82	Sc=6.79842, min distance = 2.04388
PER59_ARATH	11957353	HBB_HORSE Ful	444124	HE	1Y8I	1QGJ	1IWH		0.87	Sc=6.7578, min distance = 2.105445
PER59_ARATH	11957364	HMOX1_HUMAN F	444124	HE	1OZW	1QGJ	1S13		0.86	Sc=6.65936, min distance = 1.90687
PER59_ARATH	11957371	HMOX1_HUMAN F	444124	HE	1OZW	1QGJ	1TWR			Sc=6.60268, min distance = 2.21974
PER59_ARATH	11957385	MYG_PHYCA Ful	444124	HE	1U7R	1QGJ	2CMM			Sc=6.07504, min distance = 2.43337
PER59_ARATH	4369228 C	PER_COPCI Ful	444124	HE	1LY9	1QGJ	1LYC		0.95	Sc=6.76109, min distance = 1.96801
PER59_ARATH	444207 He	PER_COPCI Ful	444124	HE	1LY9	1QGJ	1LY8		0.99	Sc=6.75363, min distance = 2.22949
PER59_ARATH	444522 HE	HBA_PAGBE Ful	444124	HE	1S5X	1QGJ	1PBX		0.99	Sc=6.74967, min distance = 2.13047
PER59_ARATH	444522 HE	HBB_PAGBE Ful	444124	HE	1S5X	1QGJ	1PBX		0.99	Sc=6.75697, min distance = 1.97870
PER59_ARATH	444522 HE	NOSO_BACSU Fu	444124	HE	2AN0	1QGJ	2FC1		0.99	Sc=6.7479, min distance = 2.259306
PER59_ARATH	446189 CI	HBG1_HUMAN Fu	444124	HE	1I3E	1QGJ	1I3D		0.89	Sc=6.75246, min distance = 1.55653
PERL_BOVIN F	11957353	HBA_HORSE Ful	444098	HE	2D5X	2NQX	1IWH		0.83	Sc=6.75956, min distance = 2.05087
PERL_BOVIN F	16741061	MYG_PHYCA Ful	444207	He	1VXG	2QRB	1MBC		0.87	Sc=6.47831, min distance = 0
PERL_BOVIN F	16741062	MYG_PHYCA Ful	444098	HE	1A6M	2NQX	1MBN		0.84	Sc=6.44201, min distance = 2.04894
PERL_BOVIN F	16741062	MYG_PHYCA Ful	444207	He	1VXG	2QRB	1MBN		0.88	Sc=6.43667, min distance = 0
PERL_BOVIN F	4369122 C	GLB1_LUCPE Fu	444098	HE	1FLP	2NQX	1B0B		0.96	Sc=6.44689, min distance = 2.21305
PERL_BOVIN F	4369136 C	GLB_APLLI Ful	444207	He	3MBA	2QRB	1DM1		0.89	Sc=6.75934, min distance = 0
PERL_BOVIN F	444124 HE	HBB_HORSE Ful	444207	He	2ZLW	2QRB	1Y8I		0.99	Sc=6.43263, min distance = 0
PERL_BOVIN F	444522 HE	HBB_HORSE Ful	444207	He	2ZLW	2QRB	1Y8K	1		Sc=6.43002, min distance = 0
PERL_BOVIN F	444522 HE	MYG_PHYCA Ful	444207	He	1VXG	2QRB	1VXD	1		Sc=6.4441, min distance = 0
PERL_BOVIN F	444668 CI	MYG_PHYCA Ful	444207	He	1VXG	2QRB	1BVD		0.84	Sc=6.49609, min distance = 0
PERM_HUMAN F	11957385	MYG_PHYCA Ful	444098	HE	1A6M	1MYP	2CMM			Sc=6.2296, min distance = 2.084480
PER_COPCI Fu	11957330	CCPR_YEAST Fu	444207	He	2CCP	1LY8	1BEQ	25.37	0.84	Sc=6.63327, min distance = 2.31436
PER_COPCI Fu	11957360	CYB_BOVIN Ful	444522	HE	1BGY	1H3J	1NTM		0.78	Sc=6.60932, min distance = 2.29607
PER_COPCI Fu	11957361	CYB_BOVIN Ful	444522	HE	1BGY	1H3J	1NTZ		0.84	Sc=6.52521, min distance = 2.11340

PER_COPCI Fu	11957363	HBA_HUMAN Ful	444522 HE	2DN1	1H3J	1RQA		0.81	Sc=6.67764, min distance = 2.250762
PER_COPCI Fu	11957363	HBB_HUMAN Ful	444124 HE	1NQP	1LY9	1RQA		0.81	Sc=6.74445, min distance = 2.013631
PER_COPCI Fu	11957363	HBB_HUMAN Ful	444522 HE	2DN1	1H3J	1RQA		0.81	Sc=6.68534, min distance = 1.457490
PER_COPCI Fu	11957365	CCPR_YEAST Fu	444207 He	2CCP	1LY8	1S73	25.37	0.83	Sc=6.6966, min distance = 2.2732087
PER_COPCI Fu	11957365	CCPR_YEAST Fu	444522 HE	3E2O	1H3J	1S73	25.37	0.83	Sc=6.62679, min distance = 2.224165
PER_COPCI Fu	11957370	HMOX1_HUMAN Fu	444124 HE	1OZW	1LY9	1TWN			Sc=6.5024, min distance = 2.2575041
PER_COPCI Fu	11957371	HMOX1_HUMAN Fu	444124 HE	1OZW	1LY9	1TWR			Sc=6.50339, min distance = 2.628601
PER_COPCI Fu	11957385	MYG_PHYCA Ful	444124 HE	1U7R	1LY9	2CMM			Sc=6.06276, min distance = 2.419469
PER_COPCI Fu	11957385	MYG_PHYCA Ful	444207 He	1VXG	1LY8	2CMM			Sc=6.02833, min distance = 2.717125
PER_COPCI Fu	11957385	MYG_PHYCA Ful	444522 HE	1VXD	1H3J	2CMM			Sc=5.82905, min distance = 2.717870
PER_COPCI Fu	11970219	CCPR_YEAST Fu	444522 HE	3E2O	1H3J	1BEM	25.37	0.87	Sc=6.5404, min distance = 2.5722336
PER_COPCI Fu	11970221	CCPR_YEAST Fu	444207 He	2CCP	1LY8	1BES	25.37	0.87	Sc=6.63278, min distance = 2.255522
PER_COPCI Fu	11970221	CCPR_YEAST Fu	444522 HE	3E2O	1H3J	1BES	25.37	0.87	Sc=6.53669, min distance = 2.439582
PER_COPCI Fu	11970242	CCPR_YEAST Fu	444207 He	2CCP	1LY8	1CPE	25.37	0.87	Sc=6.6311, min distance = 2.4238917
PER_COPCI Fu	11987718	CCPR_YEAST Fu	444522 HE	3E2O	1H3J	1KOK	25.37	0.86	Sc=6.62445, min distance = 2.477952
PER_COPCI Fu	126994 CI	CCPR_YEAST Fu	444522 HE	3E2O	1H3J	1Z53	25.37	0.81	Sc=6.61752, min distance = 2.828693
PER_COPCI Fu	16741062	MYG_PHYCA Ful	444207 He	1VXG	1LY8	1MBN		0.88	Sc=6.62009, min distance = 1.988551
PER_COPCI Fu	16741062	MYG_PHYCA Ful	444522 HE	1VXD	1H3J	1MBN		0.88	Sc=6.66174, min distance = 2.144668
PER_COPCI Fu	16741183	MYG_PHYCA Ful	444207 He	1VXG	1LY8	2EKT		0.93	Sc=6.48383, min distance = 2.492684
PER_COPCI Fu	16741183	MYG_PHYCA Ful	444522 HE	1VXD	1H3J	2EKT		0.93	Sc=6.35596, min distance = 2.543901
PER_COPCI Fu	4369001 C	HBA_HUMAN Ful	444522 HE	2DN1	1H3J	4HHB		0.81	Sc=6.68043, min distance = 2.070931
PER_COPCI Fu	444044 CI	CCPR_YEAST Fu	444207 He	2CCP	1LY8	1DSE	25.37	0.99	Sc=6.6311, min distance = 2.8102875
PER_COPCI Fu	446332 CI	MYG_PHYCA Ful	444124 HE	1U7R	1LY9	1IOP		0.8	Sc=6.55168, min distance = 2.179301
PER_COPCI Fu	446348 CI	HBA_HUMAN Ful	444124 HE	1NQP	1LY9	1IRD		0.9	Sc=6.74602, min distance = 2.141596
PER_COPCI Fu	447168 ZE	CCPR_YEAST Fu	444522 HE	3E2O	1H3J	1ML2	25.37	0.86	Sc=6.68085, min distance = 2.668796
PER_COPCI Fu	448845 CI	CCPR_YEAST Fu	444207 He	2CCP	1LY8	2B11	25.37	0.99	Sc=6.63508, min distance = 2.201697
PFLB_ECOLI Fu	24832019	ACES_TORCA Fu	8200 TETR	1DX6	1H16	2J3Q			Sc=5.7563, min distance = 2.4578767
PFLB_ECOLI Fu	854023 Ep	Q8WSF8_APLCA I	8200 TETR	2BYN	1H16	2BYQ			Sc=6.03257, min distance = 1.140907
PGAM1_HUMAN I	11957399	PYRB_ECOLI Fu	311 citri	1R0B	1YFK	2H3E			Sc=6.11556, min distance = 1.257402
PGAM1_HUMAN I	39981 Spa	PYRB_ECOLI Fu	311 citri	1R0B	1YFK	1EKX			Sc=6.12267, min distance = 1.174884
PGAM1_HUMAN I	439459 Ho	NIFD_KLEPN Fu	311 citri	1H1L	1YFK	1QGU		0.92	Sc=5.97406, min distance = 2.517999
PGAM1_HUMAN I	439459 Ho	NIFK_KLEPN Fu	311 citri	1H1L	1YFK	1QGU		0.92	Sc=5.97221, min distance = 2.504521
PGAM1_HUMAN I	445905 1g	AROQ_HELPY Fu	311 citri	2C4V	1YFK	2C57			Sc=5.82648, min distance = 1.972940
PGAM1_HUMAN I	51 2-Oxop	SERA_ECOLI Fu	311 citri	2P9E	1YFK	1YBA		0.76	Sc=5.75384, min distance = 0.998682
PGDH_HUMAN Fu	15942690	INHA_MYCTU Fu	5893 nadi	2H7I	2GDZ	2NTJ			Sc=6.45724, min distance = 2.087621
PGDH_HUMAN Fu	16741204	INHA_MYCTU Fu	5893 nadi	2H7I	2GDZ	2IDZ		0.91	Sc=6.43294, min distance = 2.235871
PGDH_HUMAN Fu	169266 1,	GALE_HUMAN Fu	5893 nadi	1HZJ	2GDZ	1I3K		0.79	Sc=6.79971, min distance = 2.103295

PGDH_HUMAN F	439153 Di	GALE_ECOLI Fu	5893 nadi	1UDC	2GDZ	1UDB		0.79	Sc=6.79849, min distance = 2.08021
PGDH_HUMAN F	439153 Di	Q9BJJ9_PLAFA	5893 nadi	1UH5	2GDZ	1V35		0.79	Sc=6.79349, min distance = 2.09984
PGDH_HUMAN F	449263 IN	INHA_MYCTU Fu	5893 nadi	2H7I	2GDZ	2NV6		0.93	Sc=6.87572, min distance = 2.14960
PGH1_SHEEP F	11957385	MYG_PHYCA Ful	444098 HE	1A6M	2OYU	2CMM			Sc=6.304, min distance = 2.8406819
PGH1_SHEEP F	11957385	MYG_PHYCA Ful	444522 HE	1VXD	1CQE	2CMM			Sc=6.32581, min distance = 2.02147
PGH1_SHEEP F	445463 B-	OPSD_BOVIN Fu	62852 B-C	3DQB	1Q4G	3CAP		0.98	Sc=6.20222, min distance = 0.98239
PGH1_SHEEP F	445999 CI	Q79G13_MYCTU	64689 bet	1UP0	2OYU	1UOZ		0.77	Sc=5.7012, min distance = 2.015198
PGH1_SHEEP F	446406 CZ	MYG_PHYCA Ful	444098 HE	1A6M	2OYU	1J3F			Sc=6.36932, min distance = 2.45159
PGH1_SHEEP F	8158 Pela	ALBU_HUMAN Fu	338 salic	2I2Z	1PTH	1E7E			Sc=5.69297, min distance = 0.67238
PGH1_SHEEP F	8631 3,5-	ALBU_HUMAN Fu	39912 ibu	2BXG	1EQG	2BXL			Sc=5.6049, min distance = 2.513690
PGH2_MOUSE F	119346 3,	TTHY_HUMAN Fu	72099 flu	1DVT	3PGH	2GAB			Sc=5.75276, min distance = 1.91247
PGH2_MOUSE F	11957385	MYG_PHYCA Ful	444124 HE	1U7R	3PGH	2CMM			Sc=6.18194, min distance = 2.45407
PGH2_MOUSE F	16741062	MYG_PHYCA Ful	444124 HE	1U7R	3PGH	1MBN		0.87	Sc=6.7039, min distance = 2.469506
PGH2_MOUSE F	20284644	TTHY_HUMAN Fu	3033 dicl	1DVX	1PXX	2QGC			Sc=5.98563, min distance = 1.59093
PGH2_MOUSE F	23722944	TTHY_HUMAN Fu	3033 dicl	1DVX	1PXX	2QGE			Sc=5.82384, min distance = 2.15764
PGH2_MOUSE F	23722944	TTHY_HUMAN Fu	72099 flu	1DVT	3PGH	2QGE			Sc=5.83929, min distance = 1.89235
PGH2_MOUSE F	25011739	TTHY_HUMAN Fu	72099 flu	1DVT	3PGH	3CN0			Sc=5.84031, min distance = 1.88541
PGH2_MOUSE F	39912 ibu	ALBU_HUMAN Fu	3715 indc	2BXK	4COX	2BXG			Sc=6.02823, min distance = 1.00258
PGH2_MOUSE F	4369228 C	PER_COPCI Ful	444124 HE	1LY9	3PGH	1LYC		0.95	Sc=6.72306, min distance = 1.85891
PGH2_MOUSE F	4369477 D	TTHY_HUMAN Fu	3033 dicl	1DVX	1PXX	1U21		0.78	Sc=6.08948, min distance = 1.08217
PGH2_MOUSE F	4369477 D	TTHY_HUMAN Fu	72099 flu	1DVT	3PGH	1U21			Sc=6.11544, min distance = 1.19200
PGH2_MOUSE F	444044 CI	GLB1_SCAIN Fu	444124 HE	1JZL	3PGH	2R4X	1		Sc=6.66216, min distance = 2.43854
PGH2_MOUSE F	444207 He	PER_COPCI Ful	444124 HE	1LY9	3PGH	1LY8		0.99	Sc=6.72156, min distance = 2.30820
PGH2_MOUSE F	444207 He	PRXC_CALFU Fu	444124 HE	2CPO	3PGH	1CPO		0.99	Sc=6.7023, min distance = 2.142658
PGH2_MOUSE F	444522 HE	MYG_PIG Full=	444124 HE	1MYH	3PGH	1MNI		0.99	Sc=6.66429, min distance = 2.29109
PGH2_MOUSE F	444637 DE	BACR_HALSA Fu	185698 al	1IW6	1CVU	1BRR			Sc=6.00667, min distance = 0.60447
PGH2_MOUSE F	446189 CI	HBG1_HUMAN Fu	444124 HE	1I3E	3PGH	1I3D		0.89	Sc=6.7235, min distance = 2.038153
PGH2_MOUSE F	446406 CZ	MYG_PHYCA Ful	444124 HE	1U7R	3PGH	1J3F			Sc=6.23655, min distance = 2.46663
PGH2_MOUSE F	5287495 2	TTHY_HUMAN Fu	72099 flu	1DVT	3PGH	2B77			Sc=6.06823, min distance = 2.48094
PGH2_MOUSE F	5288193 2	TTHY_HUMAN Fu	72099 flu	1DVT	3PGH	2B9A		0.87	Sc=5.93976, min distance = 1.82512
PGH2_MOUSE F	5327159 2	TTHY_HUMAN Fu	72099 flu	1DVT	3PGH	2F7I		0.87	Sc=5.9194, min distance = 2.277257
PGIP2_PHAVU	10450114	DPP4_HUMAN Fu	444205 CI	3BJM	1OGQ	2IIV			Sc=6.09465, min distance = 2.13066
PGIP2_PHAVU	11987831	TRFL_BOVIN Fu	444205 CI	2G93	1OGQ	2DWJ			Sc=5.67712, min distance = 2.25891
PGK1_HUMAN F	446090 1h	NDKC_DICDI Fu	6022 Aden	1KDN	2ZGV	1HIY		0.97	Sc=5.94779, min distance = 1.64671
PGK1_HUMAN F	447550 ph	TPIS_TRYBB Fu	439183 1i	1IIH	3C39	4TIM	1		Sc=5.63988, min distance = 2.17991
PGK1_HUMAN F	448222 NE	ENPL_CANFA Fu	6022 Aden	1TC6	2ZGV	1QY5		0.81	Sc=6.28929, min distance = 2.01437
PGK1_HUMAN F	449139 CI	TPIS_PLAFA Fu	439183 1i	1M7O	3C39	1WOA		0.86	Sc=5.63198, min distance = 1.60579

PGK1_HUMAN Fu	8582 Inos	RNAS1_BOVIN Fu	6022 Aden	1O0H	2ZGV	1Z6D	0.79	Sc=6.34466, min distance = 1.81907
PGK1_HUMAN Fu	8977 1dar	PARM_ECOLX Ful	6022 Aden	1MWM	2ZGV	2ZGY	0.8	Sc=5.82078, min distance = 1.78842
PGK1_PIG Ful	11175137	KAPCA_BOVIN Fu	5957 Aden	1Q24	1VJD	2UW7		Sc=6.19736, min distance = 2.49337
PGK1_PIG Ful	15602983	KAPCA_BOVIN Fu	5957 Aden	1Q24	1VJD	2UW5		Sc=6.14859, min distance = 2.51461
PGK1_PIG Ful	16122635	KAPCA_BOVIN Fu	5957 Aden	1Q24	1VJD	2UW8		Sc=5.64806, min distance = 2.43843
PGK1_PIG Ful	439278 ph	GPMI_BACST Ful	439183 1i	1EJJ	1HDI	1O98	1	Sc=5.64399, min distance = 2.28742
PGK1_PIG Ful	444564 AD	BIOD_ECOLI Ful	5957 Aden	1A82	1VJD	1BS1	0.91	Sc=5.98372, min distance = 2.07555
PGK1_PIG Ful	447550 ph	TPIS_TRYBB Ful	439183 1i	1IIH	1HDI	4TIM	1	Sc=5.60617, min distance = 2.23879
PGK1_PIG Ful	447916 ad	RIO1_ARCFU Ful	5957 Aden	1ZP9	1VJD	1ZTF	0.94	Sc=6.20256, min distance = 2.60814
PGK1_PIG Ful	6022 Ader	HSP7F_YEAST Fu	5957 Aden	3D2F	1VJD	3C7N	0.99	Sc=6.45463, min distance = 2.11891
PGK1_PIG Ful	6022 Ader	Y059_METJA Ful	5957 Aden	2J9C	1VJD	2J9D	0.99	Sc=6.44051, min distance = 1.57210
PGK1_PIG Ful	6083 ader	PSPF_ECOLI Ful	5957 Aden	2C96	1VJD	2VII	0.98	Sc=6.34124, min distance = 2.57060
PGK1_PIG Ful	6083 ader	Y059_METJA Ful	5957 Aden	2J9C	1VJD	2J9D	0.98	Sc=6.35183, min distance = 2.30693
PGK2_MOUSE Fu	439278 ph	GPMI_BACST Ful	439183 1i	1EJJ	2P9T	1O98	1	Sc=5.64399, min distance = 2.30070
PGK2_MOUSE Fu	444564 AD	BIOD_ECOLI Ful	5957 Aden	1A82	2PAA	1BS1	0.91	Sc=5.92436, min distance = 2.17428
PGK2_MOUSE Fu	444564 AD	MYS2_DICDI Ful	5957 Aden	1FMW	2PAA	1W9I	0.91	Sc=6.37789, min distance = 0
PGK2_MOUSE Fu	444852 CI	ATPB_BOVIN Ful	5957 Aden	2V7Q	2PAA	1COW	0.91	Sc=6.02986, min distance = 2.05138
PGK2_MOUSE Fu	447550 ph	TPIS_TRYBB Ful	439183 1i	1IIH	2P9T	4TIM	1	Sc=5.63131, min distance = 2.25140
PGK2_MOUSE Fu	447916 ad	RIO1_ARCFU Ful	5957 Aden	1ZP9	2PAA	1ZTF	0.94	Sc=6.18169, min distance = 2.30632
PGK2_MOUSE Fu	6022 Ader	ARSA1_ECOLX Fu	5957 Aden	1II0	2PAA	1IHU	0.99	Sc=6.38867, min distance = 1.97708
PGK2_MOUSE Fu	6022 Ader	BIOD_ECOLI Ful	5957 Aden	1A82	2PAA	1DAD	0.99	Sc=5.92707, min distance = 2.23876
PGK2_MOUSE Fu	6022 Ader	HSP7F_YEAST Fu	5957 Aden	3D2F	2PAA	3C7N	0.99	Sc=6.40442, min distance = 2.27161
PGK2_MOUSE Fu	6022 Ader	MUTS_ECOLI Ful	5957 Aden	1W7A	2PAA	1OH7	0.99	Sc=6.38961, min distance = 2.07147
PGK2_MOUSE Fu	6022 Ader	PUR7_METJA Ful	5957 Aden	2Z02	2PAA	2YZL	0.99	Sc=5.95521, min distance = 1.99561
PGK2_MOUSE Fu	6022 Ader	SECA_ECOLI Ful	5957 Aden	2FSG	2PAA	2FSI	0.99	Sc=5.63804, min distance = 2.12642
PGK2_MOUSE Fu	6022 Ader	Y059_METJA Ful	5957 Aden	2J9C	2PAA	2J9D	0.99	Sc=6.39232, min distance = 1.93121
PGK2_MOUSE Fu	6083 ader	AAKG1_RAT Ful	5957 Aden	2V92	2PAA	2V8Q	0.98	Sc=6.3071, min distance = 2.250661
PGK2_MOUSE Fu	6133 uric	CCA_ARCFU Ful	5957 Aden	1R8B	2PAA	1R8C		Sc=5.93367, min distance = 1.12196
PGKT_THEMA Fu	447550 ph	TPIS_TRYBB Ful	439183 1i	1IIH	1VPE	4TIM	1	Sc=5.64399, min distance = 2.18665
PGKT_THEMA Fu	6083 ader	ENTP2_RAT Ful	33113 gam	3CJA	1VPE	3CJ7	0.97	Sc=6.30411, min distance = 2.23411
PGK_BACST Ful	444842 CI	CDK2_HUMAN Ful	6022 Aden	1GY3	1PHP	1CKP		Sc=5.6204, min distance = 2.021305
PGK_BACST Ful	445940 O6	CDK2_HUMAN Ful	6022 Aden	1GY3	1PHP	1GZ8		Sc=6.16759, min distance = 2.08799
PGK_BACST Ful	5957 Ader	ARP3_BOVIN Ful	6022 Aden	2P9I	1PHP	1TYQ	0.99	Sc=6.01304, min distance = 2.16506
PGK_BACST Ful	5957 Ader	HSP7F_YEAST Fu	6022 Aden	3C7N	1PHP	3D2F	0.99	Sc=6.03998, min distance = 2.38225
PGK_BACST Ful	60961 ade	IPKA_RABIT Ful	6022 Aden	1JBP	1PHP	1FMO	0.95	Sc=6.19881, min distance = 2.17566
PGK_BACST Ful	60961 ade	STK6_HUMAN Ful	6022 Aden	1MQ4	1PHP	1MUO	0.95	Sc=6.13801, min distance = 2.14722
PGK_BACST Ful	8977 1dar	PARM_ECOLX Ful	6022 Aden	1MWM	1PHP	2ZGY	0.8	Sc=6.48483, min distance = 2.02934

PGK_PLAF7 Fu	5281701 T	PIM1_HUMAN Fu	6083 aden	1YXU	1LTK	2O65			Sc=6.38594, min distance = 1.85946
PGK_PLAF7 Fu	6022 Aden	PSPF_ECOLI Fu	6083 aden	2VII	1LTK	2C98		0.99	Sc=5.6666, min distance = 2.068004
PGK_PLAF7 Fu	65110 AIC	AAKG1_HUMAN F	6083 aden	2UV4	1LTK	2UV5		0.79	Sc=6.24317, min distance = 2.35346
PGK_PLAF7 Fu	92199 AMF	HINT1_HUMAN F	6083 aden	1KPF	1LTK	1AV5		0.97	Sc=5.6282, min distance = 2.165966
PGMI_PYRAE F	311 citri	G6PI_TRYBB Fu	439958 gl	2O2C	1X9I	2O2D			Sc=5.96038, min distance = 1.67681
PGRP_CAMDR F	439680 be	HEMA_I63A3 Fu	82313 1rd	1MQL	3C93	1MQM			Sc=5.81878, min distance = 0.96290
PH2M_TRICU F	449465 CI	GSHR_HUMAN Fu	444188 CI	3DK9	1PN0	3GRT		0.98	Sc=6.88883, min distance = 2.19664
PH4H_CHRVO F	133246 di	NOS2_MOUSE Fu	445040 6-	3E6N	1LTZ	3DWJ			Sc=6.1656, min distance = 3.032361
PH4H_CHRVO F	444951 CI	PTR1_LEIMA Fu	445040 6-	1E92	1LTZ	2BFP			Sc=6.18156, min distance = 2.50477
PH4H_CHRVO F	9547941 4	NOS1_RAT Full	445040 6-	2G6I	1LTZ	2G6J		0.79	Sc=6.10847, min distance = 3.13530
PH4H_HUMAN F	1649 3-br	NOS3_BOVIN Fu	444951 CI	1Q2O	1MMK	1D0C			Sc=5.83477, min distance = 2.82232
PH4H_HUMAN F	1649 3-br	NOS3_HUMAN Fu	133246 di	3NOS	1J8U	1M9R			Sc=5.75342, min distance = 2.14604
PH4H_HUMAN F	181426 2-	NOS3_BOVIN Fu	444951 CI	1Q2O	1MMK	1DM6			Sc=5.80288, min distance = 2.39055
PH4H_HUMAN F	445109 2,	NOS3_BOVIN Fu	444951 CI	1Q2O	1MMK	1DMK			Sc=6.31378, min distance = 2.43226
PH4H_HUMAN F	5202 serc	Q9BIH3_ANOGA	5814 nore	2QEO	4PAH	2QEH			Sc=5.69537, min distance = 2.29150
PH4H_HUMAN F	9547941 4	NOS1_RAT Full	444951 CI	1OM4	1MMK	2G6J			Sc=6.26061, min distance = 1.95460
PH4H_HUMAN F	9547941 4	NOS1_RAT Full	445040 6-	2G6I	1DMW	2G6J		0.79	Sc=6.15298, min distance = 2.54277
PHEA_ECOLI F	444881 L-	CHMU_YEAST Fu	445314 1f	4CSM	1ECM	1CSM			Sc=5.89671, min distance = 1.56090
PHHY_PSEAE F	11987634	GLPD_ECOLI Fu	444188 CI	2QCU	1K0I	2R4E		0.97	Sc=6.52288, min distance = 1.89597
PHHY_PSEAE F	16740985	FRDA_SHEFR Fu	444188 CI	1M64	1K0I	1E39		0.93	Sc=6.81755, min distance = 2.15044
PHHY_PSEAE F	3505109 4	PCXA_ACIAD Fu	135 p-Hyd	2BUR	1K0I	2BUU			Sc=5.77455, min distance = 1.83400
PHHY_PSEAE F	3505109 4	PCXB_ACIAD Fu	135 p-Hyd	2BUR	1K0I	2BUU			Sc=5.76571, min distance = 1.81255
PHHY_PSEAE F	444502 CI	GSHR_HUMAN Fu	444188 CI	3DK9	1K0I	1BWC		0.98	Sc=6.39948, min distance = 2.26970
PHHY_PSEAE F	446013 1,	O28603_ARCFU	444188 CI	1JNR	1K0I	1JNZ		0.94	Sc=6.83329, min distance = 2.07628
PHHY_PSEAE F	446013 1,	O28604_ARCFU	444188 CI	1JNR	1K0I	1JNZ		0.94	Sc=6.83689, min distance = 2.07546
PHHY_PSEAE F	449465 CI	GSHR_HUMAN Fu	444188 CI	3DK9	1K0I	3GRT		0.98	Sc=6.83213, min distance = 2.15084
PHHY_PSEAE F	72 protoc	PCXB_PSEPU Fu	135 p-Hyd	3PCC	1K0I	1YKN		0.89	Sc=5.89526, min distance = 1.55927
PHHY_PSEFL F	12122 3-H	PCXA_PSEPU Fu	72 protoc	1YKN	1PHH	3PCE		0.84	Sc=5.79518, min distance = 1.85924
PHHY_PSEFL F	12122 3-H	PCXB_PSEPU Fu	72 protoc	1YKN	1PHH	3PCE		0.84	Sc=5.79518, min distance = 1.97014
PHHY_PSEFL F	127 4-hyd	PCXA_PSEPU Fu	72 protoc	1YKN	1PHH	3PCG		0.79	Sc=5.80215, min distance = 2.04822
PHHY_PSEFL F	127 4-hyd	PCXB_PSEPU Fu	72 protoc	1YKN	1PHH	3PCG		0.79	Sc=5.80053, min distance = 2.15637
PHHY_PSEFL F	160456 3p	PCXB_PSEPU Fu	135 p-Hyd	3PCC	1PBE	3PCF		0.97	Sc=5.8718, min distance = 0.982691
PHHY_PSEFL F	19860 3-C	PCXA_PSEPU Fu	72 protoc	1YKN	1PHH	3PCH		0.79	Sc=5.70727, min distance = 2.12403
PHHY_PSEFL F	3505109 4	LOX3_SOYBN Fu	72 protoc	1N8Q	1PHH	1NO3			Sc=5.74599, min distance = 2.47621
PHHY_PSEFL F	446013 1,	O28603_ARCFU	444188 CI	1JNR	1PBE	1JNZ		0.94	Sc=6.44226, min distance = 2.01329
PHHY_PSEFL F	446013 1,	O28604_ARCFU	444188 CI	1JNR	1PBE	1JNZ		0.94	Sc=6.44144, min distance = 1.96367
PHHY_PSEFL F	449465 CI	GSHR_HUMAN Fu	444188 CI	3DK9	1PBE	3GRT		0.98	Sc=6.87798, min distance = 2.12050

PHHY_PSEFL Fu	449473 Am	PCXA_PSEPU Fu	72 protoc	1YKN	1PHH	3PCI		0.87	Sc=5.7213, min distance = 1.908921
PHHY_PSEFL Fu	7420 3-Hy	PCXA_PSEPU Fu	72 protoc	1YKN	1PHH	3PCB		0.89	Sc=5.67564, min distance = 2.12076
PHHY_PSEFL Fu	967 Oroti	PYRDA_LACLC Fu	72 protoc	2BSL	1PHH	1JQX			Sc=5.78552, min distance = 2.54495
PHI_METJA Fu	11957399	PYRB_ECOLI Fu	311 citri	1R0B	1JEO	2H3E			Sc=5.97045, min distance = 2.07229
PHI_METJA Fu	16750041	PYRB_ECOLI Fu	311 citri	1R0B	1JEO	2IPO			Sc=5.96047, min distance = 2.21383
PHI_METJA Fu	39981 Spa	PYRB_ECOLI Fu	311 citri	1R0B	1JEO	1EKX			Sc=6.05651, min distance = 1.64745
PHI_METJA Fu	439459 Ho	NIFD_KLEPN Fu	311 citri	1H1L	1JEO	1QGU		0.92	Sc=5.93262, min distance = 2.27237
PHI_METJA Fu	439459 Ho	NIFK_KLEPN Fu	311 citri	1H1L	1JEO	1QGU		0.92	Sc=5.93051, min distance = 2.27308
PHI_METJA Fu	447376 2-	ACON_BOVIN Fu	311 citri	1C96	1JEO	1NIS			Sc=5.68885, min distance = 2.08038
PHI_METJA Fu	447534 IS	SRC_HUMAN Ful	311 citri	1O4L	1JEO	1O4J			Sc=5.84674, min distance = 2.04022
PHL2_BACCE Fu	8200 TETR	LMBL1_HUMAN Fu	78165 MES	1OZ2	2DDT	2RHI			Sc=5.70104, min distance = 2.41463
PHOQ_ECOLI Fu	16058649	IGF1R_HUMAN Fu	33113 gam	1JQH	1ID0	2OJ9			Sc=6.59683, min distance = 1.43859
PHOQ_ECOLI Fu	16750062	CSK2A_MAIZE Fu	33113 gam	1LP4	1ID0	2OXD			Sc=5.65867, min distance = 2.15573
PHOQ_ECOLI Fu	188966 dA	HSLU_ECOLI Fu	33113 gam	1E94	1ID0	1G4A		0.97	Sc=5.88452, min distance = 2.13941
PHOQ_ECOLI Fu	24851689	PIM1_HUMAN Fu	33113 gam	1XR1	1ID0	3CY2			Sc=6.2186, min distance = 2.333594
PHOQ_ECOLI Fu	446721 CI	ASSY_THET8 Fu	33113 gam	1KOR	1ID0	1KH2		0.93	Sc=5.96842, min distance = 2.28752
PHOQ_SALTY Fu	72194 2-C	ENPL_CANFA Fu	6323491 M	1QY8	3CGY	1QYE			Sc=5.90109, min distance = 2.12586
PHOT_BACSU Fu	444658 2a	Q9WZW1_THEMA F	444243 FA	1T6Y	2PR5	1T6Z		0.97	Sc=6.13012, min distance = 2.03381
PHOT_BACSU Fu	446995 FM	CYB2_YEAST Fu	444243 FA	1KBI	2PR5	1LTD		0.77	Sc=6.15191, min distance = 2.10288
PHR_THET8 Fu	444502 CI	PHR_SYNP6 Ful	444188 CI	1TEZ	2J07	1OWM	36.61	0.98	Sc=6.15275, min distance = 2.25762
PHR_THET8 Fu	447737 CI	PHR_SYNP6 Ful	444188 CI	1TEZ	2J07	1OWL	36.61	0.98	Sc=6.1511, min distance = 2.183890
PHR_THET8 Fu	5326566 I	Q9WZW1_THEMA F	444243 FA	1T6Y	2J09	1S4M		0.82	Sc=6.28617, min distance = 1.65571
PHSM_ECOLI Fu	1052 pyri	PDXK_SHEEP Fu	95687 Dec	1RFU	1QM5	1RFT		0.92	Sc=5.89264, min distance = 1.56255
PHSM_ECOLI Fu	124823 6g	PYGM_RABIT Fu	65533 cor	3GPB	1L5V	6GPB		0.91	Sc=6.0749, min distance = 2.425589
PHSM_ECOLI Fu	14257 Unc	BACR_HALSA Fu	79025 alp	1DZE	1L5W	1CWQ			Sc=5.67613, min distance = 1.07499
PHSM_ECOLI Fu	151504 D-	CARP_YEAST Fu	64689 bet	1FQ4	2ASV	1FQ5			Sc=5.68216, min distance = 2.41913
PHSM_ECOLI Fu	16741013	CARP_YEAST Fu	64689 bet	1FQ4	2ASV	1FQ6			Sc=5.72577, min distance = 2.19232
PHSM_ECOLI Fu	21678292	PYGM_RABIT Fu	65533 cor	3GPB	1L5V	3BD8			Sc=6.27415, min distance = 2.47635
PHSM_ECOLI Fu	24856350	PYGM_RABIT Fu	65533 cor	3GPB	1L5V	2QN7			Sc=6.48461, min distance = 2.16215
PHSM_ECOLI Fu	24856351	PYGM_RABIT Fu	65533 cor	3GPB	1L5V	2QN8			Sc=6.51204, min distance = 2.13772
PHSM_ECOLI Fu	25049743	PYGM_RABIT Fu	65533 cor	3GPB	1L5V	3BDA			Sc=6.27987, min distance = 2.18091
PHSM_ECOLI Fu	2519 caff	PYGM_RABIT Fu	65533 cor	3GPB	1L5V	1GFZ			Sc=5.69024, min distance = 2.77822
PHSM_ECOLI Fu	4369162 1	PYGM_RABIT Fu	65533 cor	3GPB	1L5V	1HLF			Sc=6.14146, min distance = 2.42674
PHSM_ECOLI Fu	439692 D-	XYLA_STRRU Fu	64960 Pol	1XIE	2ASV	4XIS			Sc=5.60036, min distance = 2.72004
PHSM_ECOLI Fu	444139 CI	CDGT2_BACCI Fu	79025 alp	1D3C	1L5W	1DTU			Sc=5.79943, min distance = 1.03490
PHSM_ECOLI Fu	444139 CI	NEPU1_THEVU Fu	64689 bet	1UH4	2ASV	1UH3			Sc=5.67478, min distance = 0.77502
PHSM_ECOLI Fu	444140 CI	CDGT_THETU Fu	79025 alp	3BMW	1L5W	1A47			Sc=5.7338, min distance = 1.192128

PHSM_ECOLI Fι	444185 1a	PYGM_RABIT Fu:	65533	cor	3GPB	1L5V	1A8I			Sc=6.16927, min distance = 2.37911:
PHSM_ECOLI Fι	444393 CI	PYGM_RABIT Fu:	65533	cor	3GPB	1L5V	1AXR			Sc=6.01112, min distance = 2.416345
PHSM_ECOLI Fι	444440 AC	AMY1_HORVU Fu:	79025	alp	1RP8	1L5W	1RP9			Sc=6.39486, min distance = 1.458387
PHSM_ECOLI Fι	444809 Th	GUN2_THEFU Fu:	64689	bet	2BOF	2ASV	2BOG		0.78	Sc=5.80938, min distance = 0.881034
PHSM_ECOLI Fι	444809 Th	GUN5_BACAG Fu:	64689	bet	1W3L	2ASV	1H5V		0.78	Sc=5.76571, min distance = 0.802542
PHSM_ECOLI Fι	444809 Th	GUX2_TRIRE Fu:	64689	bet	1QK0	2ASV	1QJW		0.78	Sc=5.69804, min distance = 2.586043
PHSM_ECOLI Fι	444809 Th	Q79G13_MYCTU]	64689	bet	1UP0	2ASV	1UOZ		0.78	Sc=5.72788, min distance = 2.505494
PHSM_ECOLI Fι	445237 1,	AMY1_HORVU Fu:	64689	bet	1RP9	2ASV	1P6W		0.77	Sc=5.75684, min distance = 1.733368
PHSM_ECOLI Fι	445382 2p	PYGM_RABIT Fu:	65533	cor	3GPB	1L5V	1WW2			Sc=6.0813, min distance = 2.3612109
PHSM_ECOLI Fι	445723 1-	PYGM_RABIT Fu:	65533	cor	3GPB	1L5V	1P4H			Sc=6.23709, min distance = 2.564685
PHSM_ECOLI Fι	445825 CI	PYGM_RABIT Fu:	65533	cor	3GPB	1L5V	1GG8			Sc=5.98461, min distance = 2.602475
PHSM_ECOLI Fι	445916 be	XYLA_STRRU Fu:	64960	Pol	1XIE	2ASV	1GW9		0.82	Sc=5.63483, min distance = 2.711264
PHSM_ECOLI Fι	445999 CI	GUX6_HUMIN Fu:	64689	bet	1OCN	2ASV	1OC7		0.77	Sc=5.60314, min distance = 1.755010
PHSM_ECOLI Fι	446000 CI	AMYP_PIG Full=	79025	alp	1HX0	1L5W	1JFH		0.83	Sc=5.69173, min distance = 2.353928
PHSM_ECOLI Fι	446600 1k	PYGM_RABIT Fu:	65533	cor	3GPB	1L5V	1K06			Sc=6.45343, min distance = 2.148514
PHSM_ECOLI Fι	446685 Va	NEPU1_THEVU Fι	79025	alp	1UH4	1L5W	1UH3			Sc=5.95768, min distance = 1.111833
PHSM_ECOLI Fι	446801 1k	PYGM_RABIT Fu:	65533	cor	3GPB	1L5V	1KTI			Sc=6.23277, min distance = 2.135731
PHSM_ECOLI Fι	446996 GA	Q9HYN5_PSEAE]	64689	bet	1W8F	2ASV	1W8H		0.89	Sc=5.80046, min distance = 0.975837
PHSM_ECOLI Fι	447413 NT	PYGM_RABIT Fu:	65533	cor	3GPB	1L5V	1NOJ			Sc=5.9919, min distance = 2.4589560
PHSM_ECOLI Fι	447601 CI	GUX6_HUMIN Fu:	64689	bet	1OCN	2ASV	1OCB			Sc=5.67761, min distance = 2.646187
PHSM_ECOLI Fι	447770 CC	PYGM_RABIT Fu:	65533	cor	3GPB	1L5V	1P4G			Sc=6.11778, min distance = 2.568166
PHSM_ECOLI Fι	448009 TF	AMY1_HUMAN Fu:	79025	alp	3DHP	1L5W	1Q4N			Sc=5.71884, min distance = 1.138239
PHSM_ECOLI Fι	449180 XI	XYLA_STRRU Fu:	64960	Pol	1XIE	2ASV	4XIS		0.8	Sc=5.74678, min distance = 2.535118
PHSM_ECOLI Fι	449554 CI	PYGM_RABIT Fu:	65533	cor	3GPB	1L5V	5GPB		0.82	Sc=6.08064, min distance = 2.243685
PHSM_ECOLI Fι	449571 CI	CDGT1_BACCI Fι	79025	alp	1CGU	1L5W	6CGT			Sc=5.61476, min distance = 2.660864
PHSM_ECOLI Fι	6134 lact	Q6YD91_ERYCG]	64689	bet	1UZY	2ASV	1V00		0.87	Sc=6.42935, min distance = 1.930821
PHSM_ECOLI Fι	657042 CH	PYGM_RABIT Fu:	65533	cor	3GPB	1L5V	1XKX			Sc=6.30979, min distance = 2.030420
PHSM_ECOLI Fι	6852183 N	PYGM_RABIT Fu:	65533	cor	3GPB	1L5V	2F3U			Sc=6.35633, min distance = 2.005442
PHSM_ECOLI Fι	84265 1ax	TRFL_BOVIN Fu:	79025	alp	2DWJ	1L5W	2AYS			Sc=6.09334, min distance = 1.690274
PHSM_ECOLI Fι	95259 L-x	XYLA_STRRU Fu:	64960	Pol	1XIE	2ASV	1XIC			Sc=5.73619, min distance = 2.284510
PHS_RAT Full=	292661 2,	PTR1_LEIMA Fu:	445040	6-1E92		1DCP	1W0C			Sc=6.01426, min distance = 2.457152
PHS_RAT Full=	444951 CI	PTR1_LEIMA Fu:	445040	6-1E92		1DCP	2BFP			Sc=5.86453, min distance = 2.170302
PHS_RAT Full=	9547941 4	NOS1_RAT Full=	445040	6-	2G6I	1DCP	2G6J		0.79	Sc=5.81294, min distance = 2.345679
PHZF_PSEFL Fι	114947 4-	3HAO_RALME Fu:	86	3-Hydr	1YFY	1U1W	1YFW		0.87	Sc=5.89646, min distance = 2.229183
PHZG_PSEFL Fι	5326566]	BLVRB_HUMAN Fι	444243	FA	1HE4	1TY9	1HE5		0.82	Sc=6.10486, min distance = 2.613192
PIM1_HUMAN Fι	10224714	CDK2_HUMAN Fu:	444345	1c	1AQ1	1YHS	3DDP	31.71		Sc=6.23463, min distance = 2.391868
PIM1_HUMAN Fι	11314340	KAPCA_BOVIN Fι	444345	1c	1STC	1YHS	2UZT	30.06		Sc=6.33688, min distance = 2.402375

PIM1_HUMAN F	11348631	CHK1_HUMAN Ful	444345 1c	1NVR	1YHS	2E9U	34.71		Sc=6.08593, min distance = 2.07668
PIM1_HUMAN F	11502647	CHK1_HUMAN Ful	444345 1c	1NVR	1YHS	2GDO	34.71		Sc=6.25829, min distance = 2.72056
PIM1_HUMAN F	1400 nche	RET_HUMAN Ful	6083 aden	2IVT	1YXU	2IVV	27.45		Sc=5.88909, min distance = 2.32683
PIM1_HUMAN F	1540 1h1q	CDK2_HUMAN Ful	444345 1c	1AQ1	1YHS	1H1Q	31.71		Sc=6.01153, min distance = 2.89138
PIM1_HUMAN F	15942652	CDK2_HUMAN Ful	444345 1c	1AQ1	1YHS	2DUV	31.71		Sc=6.30136, min distance = 2.81981
PIM1_HUMAN F	15991572	LCK_HUMAN Ful	444345 1c	1QPD	1YHS	2OF4	34.33		Sc=6.11865, min distance = 2.33935
PIM1_HUMAN F	160355 rc	CDK2_HUMAN Ful	444345 1c	1AQ1	1YHS	3DDQ	31.71		Sc=6.03675, min distance = 2.52536
PIM1_HUMAN F	16058649	IGF1R_HUMAN F	33113 gam	1JQH	1XR1	2OJ9			Sc=6.31598, min distance = 1.76795
PIM1_HUMAN F	16122608	PDPK1_HUMAN F	2396 bisi	1UU8	1XWS	2PE1	25.47	0.79	Sc=6.27629, min distance = 2.13917
PIM1_HUMAN F	16122608	PDPK1_HUMAN F	444345 1c	1OKY	1YHS	2PE1	25.47		Sc=6.25561, min distance = 2.84494
PIM1_HUMAN F	16758227	CHK1_HUMAN Ful	444345 1c	1NVR	1YHS	2R0U	34.71		Sc=6.52635, min distance = 2.30086
PIM1_HUMAN F	17754396	PDPK1_HUMAN F	2396 bisi	1UU8	1XWS	2R7B	25.47		Sc=6.13445, min distance = 2.00315
PIM1_HUMAN F	1907917 2	CHK1_HUMAN Ful	444345 1c	1NVR	1YHS	2CGW	34.71		Sc=5.96971, min distance = 3.11082
PIM1_HUMAN F	23536770	LCK_HUMAN Ful	444345 1c	1QPD	1YHS	3BYM	34.33		Sc=6.19937, min distance = 2.50675
PIM1_HUMAN F	23653515	CDK2_HUMAN Ful	444345 1c	1AQ1	1YHS	2R3F	31.71		Sc=5.74093, min distance = 2.74113
PIM1_HUMAN F	23653516	CDK2_HUMAN Ful	444345 1c	1AQ1	1YHS	2R3G	31.71		Sc=6.09078, min distance = 3.14677
PIM1_HUMAN F	23653518	CDK2_HUMAN Ful	444345 1c	1AQ1	1YHS	2R3J	31.71		Sc=5.96034, min distance = 2.66341
PIM1_HUMAN F	23653522	CDK2_HUMAN Ful	444345 1c	1AQ1	1YHS	2R3N	31.71		Sc=6.02071, min distance = 2.51541
PIM1_HUMAN F	23653526	CDK2_HUMAN Ful	444345 1c	1AQ1	1YHS	2R3R	31.71		Sc=5.93749, min distance = 2.62811
PIM1_HUMAN F	23657800	CHK1_HUMAN Ful	444345 1c	1NVR	1YHS	2E9P	34.71		Sc=6.13476, min distance = 2.27013
PIM1_HUMAN F	23727982	CDK2_HUMAN Ful	444345 1c	1AQ1	1YHS	3BHU	31.71		Sc=6.23711, min distance = 2.83500
PIM1_HUMAN F	2403 1uvr	PDPK1_HUMAN F	444345 1c	1OKY	1YHS	1UVR	25.47		Sc=6.37213, min distance = 2.26385
PIM1_HUMAN F	24752838	CHK1_HUMAN Ful	444345 1c	1NVR	1YHS	2QHM	34.71		Sc=6.05605, min distance = 2.20082
PIM1_HUMAN F	24756824	CSK2A_MAIZE F	33113 gam	1LP4	1XR1	3BE9	26.34		Sc=6.35121, min distance = 2.33331
PIM1_HUMAN F	24779674	CSK2A_MAIZE F	33113 gam	1LP4	1XR1	2PVH	26.34		Sc=5.84254, min distance = 2.84434
PIM1_HUMAN F	24779676	CSK2A_MAIZE F	33113 gam	1LP4	1XR1	2PVK	26.34		Sc=6.27663, min distance = 2.16895
PIM1_HUMAN F	24779677	CSK2A_MAIZE F	33113 gam	1LP4	1XR1	2PVL	26.34		Sc=6.0179, min distance = 2.6589738
PIM1_HUMAN F	24779678	CSK2A_MAIZE F	33113 gam	1LP4	1XR1	2PVM	26.34		Sc=6.29757, min distance = 2.18524
PIM1_HUMAN F	24864081	CDK2_HUMAN Ful	444345 1c	1AQ1	1YHS	2VTS	31.71		Sc=6.19212, min distance = 2.24733
PIM1_HUMAN F	24901723	CDK2_HUMAN Ful	444345 1c	1AQ1	1YHS	2W06	31.71		Sc=6.05915, min distance = 2.11147
PIM1_HUMAN F	24905142	SRC_CHICK Ful	6083 aden	3DQX	1YXU	3EN4	32.35		Sc=6.38378, min distance = 0.72507
PIM1_HUMAN F	24905143	SRC_CHICK Ful	6083 aden	3DQX	1YXU	3EN7	32.35		Sc=6.19882, min distance = 0.94141
PIM1_HUMAN F	24905152	SRC_CHICK Ful	6083 aden	3DQX	1YXU	3EN6	32.35		Sc=6.30149, min distance = 1.28391
PIM1_HUMAN F	24905153	SRC_CHICK Ful	6083 aden	3DQX	1YXU	3EN5	32.35		Sc=6.08596, min distance = 2.59706
PIM1_HUMAN F	24916751	CDK2_HUMAN Ful	444345 1c	1AQ1	1YHS	3DOG	31.71		Sc=6.02911, min distance = 2.63951
PIM1_HUMAN F	25021197	CDK2_HUMAN Ful	444345 1c	1AQ1	1YHS	3EOC	31.71		Sc=5.94101, min distance = 2.21367
PIM1_HUMAN F	3543 1g5s	CDK2_HUMAN Ful	444345 1c	1AQ1	1YHS	1G5S	31.71		Sc=6.2321, min distance = 2.342946

PIM1_HUMAN F	3547 Fasu	ROCK1_HUMAN F	33113 gam	2V55	1XR1	2ESM	32.89		Sc=5.8712, min distance = 2.635911
PIM1_HUMAN F	4369136 C	CDK2_HUMAN Fu	444345 1c	1AQ1	1YHS	1DM2	31.71		Sc=6.14216, min distance = 3.04842
PIM1_HUMAN F	439176 Me	MTAP_SULSO Fu	60961 ade	1JDV	1YI4	1JDT		0.91	Sc=5.97033, min distance = 2.00835
PIM1_HUMAN F	444367 CI	CSK2A_MAIZE F	33113 gam	1LP4	1XR1	1OM1	26.34		Sc=6.12588, min distance = 2.20889
PIM1_HUMAN F	444564 AD	MYS2_DICDI Fu	33113 gam	1MMN	1XR1	1W9I		0.91	Sc=5.80761, min distance = 2.33450
PIM1_HUMAN F	445840 di	CDK2_HUMAN Fu	444345 1c	1AQ1	1YHS	1GII	31.71		Sc=5.99141, min distance = 2.22833
PIM1_HUMAN F	446724 AM	NUDT5_HUMAN F	6083 aden	2DSD	1YXU	3BM4		0.92	Sc=5.84203, min distance = 1.46374
PIM1_HUMAN F	447649 1c	CDK2_HUMAN Fu	444345 1c	1AQ1	1YHS	1OI9	31.71		Sc=6.1409, min distance = 2.652222
PIM1_HUMAN F	447654 CI	CDK2_HUMAN Fu	444345 1c	1AQ1	1YHS	1OIT	31.71		Sc=6.04107, min distance = 2.07622
PIM1_HUMAN F	447655 1c	CDK2_HUMAN Fu	444345 1c	1AQ1	1YHS	1OIU	31.71		Sc=6.03183, min distance = 2.18419
PIM1_HUMAN F	447722 9-	DEOD_ECOLI Fu	60961 ade	1PK7	1YI4	1OUM		0.9	Sc=5.9922, min distance = 1.379211
PIM1_HUMAN F	447832 AD	ACSA_SALTY Fu	6083 aden	2P2F	1YXU	1PG4		0.99	Sc=6.36476, min distance = 1.08362
PIM1_HUMAN F	448005 CI	GSK3B_HUMAN F	33113 gam	1J1B	1XR1	1Q3W	32.04		Sc=6.13001, min distance = 2.89314
PIM1_HUMAN F	448043 2g	KAPCA_BOVIN F	444345 1c	1STC	1YHS	1Q8U	30.06		Sc=5.95001, min distance = 2.03637
PIM1_HUMAN F	456214 pu	KS6A1_HUMAN F	444345 1c	2Z7R	1YHS	2Z7S			Sc=6.25043, min distance = 2.01784
PIM1_HUMAN F	4565 1h1r	CDK2_HUMAN Fu	444345 1c	1AQ1	1YHS	1H1R	31.71		Sc=6.00246, min distance = 2.32026
PIM1_HUMAN F	4566 1h1s	CDK2_HUMAN Fu	444345 1c	1AQ1	1YHS	1H1S	31.71		Sc=6.058, min distance = 2.4769136
PIM1_HUMAN F	5288016 6	CDK2_HUMAN Fu	444345 1c	1AQ1	1YHS	2B53	31.71		Sc=6.03565, min distance = 2.44459
PIM1_HUMAN F	5288708 1	CDK2_HUMAN Fu	444345 1c	1AQ1	1YHS	1KE5	31.71		Sc=6.00804, min distance = 2.13412
PIM1_HUMAN F	5326978 1	CSK2A_MAIZE F	33113 gam	1LP4	1XR1	1ZOH	26.34		Sc=5.61111, min distance = 2.29080
PIM1_HUMAN F	5327122 C	CHK1_HUMAN Fu	444345 1c	1NVR	1YHS	2C3K	34.71		Sc=6.38294, min distance = 2.13893
PIM1_HUMAN F	5327123 I	CHK1_HUMAN Fu	444345 1c	1NVR	1YHS	2C3L	34.71		Sc=5.98389, min distance = 2.47527
PIM1_HUMAN F	5327130 C	CDK2_HUMAN Fu	444345 1c	1AQ1	1YHS	2C68	31.71		Sc=5.97128, min distance = 2.62679
PIM1_HUMAN F	5327131 T	CDK2_HUMAN Fu	444345 1c	1AQ1	1YHS	2C69	31.71		Sc=5.94964, min distance = 2.62418
PIM1_HUMAN F	5327132 C	CDK2_HUMAN Fu	444345 1c	1AQ1	1YHS	2C6I	31.71		Sc=5.93421, min distance = 2.02211
PIM1_HUMAN F	5327133 C	CDK2_HUMAN Fu	444345 1c	1AQ1	1YHS	2C6K	31.71		Sc=5.88994, min distance = 2.09940
PIM1_HUMAN F	5327134 C	CDK2_HUMAN Fu	444345 1c	1AQ1	1YHS	2C6L	31.71		Sc=6.06182, min distance = 2.18215
PIM1_HUMAN F	5327135 D	CDK2_HUMAN Fu	444345 1c	1AQ1	1YHS	2C6M	31.71		Sc=5.90158, min distance = 2.01803
PIM1_HUMAN F	5327148 C	KAPCA_MOUSE F	60961 ade	1FMO	1YI4	2ERZ	31.25		Sc=6.05208, min distance = 2.30283
PIM1_HUMAN F	5331010 c	CDK2_HUMAN Fu	444345 1c	1AQ1	1YHS	2BKZ	31.71		Sc=6.19059, min distance = 2.67037
PIM1_HUMAN F	5687 1di8	CDK2_HUMAN Fu	444345 1c	1AQ1	1YHS	1DI8	31.71		Sc=6.1091, min distance = 2.661407
PIM1_HUMAN F	6419789 C	HASP_HUMAN Fu	6083 aden	3DLZ	1YXU	3E7V			Sc=6.31461, min distance = 2.19579
PIM1_HUMAN F	6420139 C	CDK2_HUMAN Fu	444345 1c	1AQ1	1YHS	2C5V	31.71		Sc=5.9861, min distance = 2.553361
PIM1_HUMAN F	6540273 2	CHK1_HUMAN Fu	444345 1c	1NVR	1YHS	2CGV	34.71		Sc=6.10671, min distance = 2.28921
PIM1_HUMAN F	656968 1y	PDE4B_HUMAN F	6083 aden	1ROR	1YXU	1Y2H			Sc=5.95476, min distance = 1.30353
PIM1_HUMAN F	657072 RY	CDK2_HUMAN Fu	444345 1c	1AQ1	1YHS	2BHH	31.71		Sc=6.36554, min distance = 2.64497
PIM1_HUMAN F	6852207 M	KAPCA_BOVIN F	444345 1c	1STC	1YHS	2GNI	30.06		Sc=5.89227, min distance = 2.15523

PIM1_HUMAN F	72271 7-H	CDK2_HUMAN Fu	444345 1c	1AQ1	1YHS	1PKD	31.71	0.91	Sc=6.35367, min distance = 2.04108
PIM1_HUMAN F	72443 for	MTAP_SULSO Fu	60961 ade	1JDV	1YI4	1JDZ			Sc=5.96895, min distance = 2.08993
PIM1_HUMAN F	9547890 I	CDK2_HUMAN Fu	444345 1c	1AQ1	1YHS	1W8C	31.71		Sc=5.75958, min distance = 2.84098
PIM1_HUMAN F	97184 6-M	DEOD_ECOLI Fu	60961 ade	1PK7	1YI4	1OVG		0.9	Sc=5.90568, min distance = 1.46560
PIM1_HUMAN F	9817550 V	CDK2_HUMAN Fu	444345 1c	1AQ1	1YHS	3BHV	31.71		Sc=6.36399, min distance = 2.37918
PIM2_HUMAN F	10109823	PIM1_HUMAN Fu	11986113	2BZH	2IWI	3CY3			Sc=6.36703, min distance = 2.80807
PIM2_HUMAN F	2396 bisi	PIM1_HUMAN Fu	11986113	2BZH	2IWI	1XWS		0.76	Sc=6.40238, min distance = 2.46303
PIM2_HUMAN F	24180721	PIM1_HUMAN Fu	11986113	2BZH	2IWI	3C4E			Sc=5.85843, min distance = 2.99106
PIM2_HUMAN F	444345 1c	PIM1_HUMAN Fu	11986113	2BZH	2IWI	1YHS		0.76	Sc=6.35117, min distance = 2.10010
PIM2_HUMAN F	611002 Op	PIM1_HUMAN Fu	11986113	2BZH	2IWI	1YXX			Sc=6.00356, min distance = 3.05821
PIMA_MYCLE F	24978488	TBB2B_BOVIN F	8977 1dar	1Z2B	2GEK	3DU7			Sc=5.62514, min distance = 2.28993
PIMA_MYCLE F	439912 GD	GMD2_ARATH Fu	8977 1dar	1N7H	2GEK	1N7G		0.92	Sc=6.10509, min distance = 2.35444
PIMA_MYCLE F	445463 B-	TGM3_HUMAN Fu	8977 1dar	1VJJ	2GEK	1L9N			Sc=6.11735, min distance = 2.18075
PIMA_MYCLE F	6022 Ader	PARM_ECOLX Fu	8977 1dar	2ZGY	2GEK	1MWM		0.8	Sc=5.61535, min distance = 2.25832
PIMA_MYCLE F	6031 Uric	RIR1_YEAST Fu	8977 1dar	2CVW	2GEK	2CVV			Sc=5.73619, min distance = 2.13302
PIMA_MYCLE F	6804 guar	EF1A_YEAST Fu	8977 1dar	2B7B	2GEK	1G7C		0.99	Sc=5.88452, min distance = 2.14732
PIMA_MYCLE F	6804 guar	GBP1_HUMAN Fu	8977 1dar	2B92	2GEK	2B8W		0.99	Sc=5.86698, min distance = 2.01109
PIMT_DROME F	13711 dec	MTH1_HAEPH Fu	439155 Ad	2C7P	1R18	2Z6Q			Sc=6.00878, min distance = 2.09089
PIMT_DROME F	188380 Ac	MCES_ENCCU Fu	439155 Ad	1RI1	1R18	1Z3C		0.91	Sc=6.54986, min distance = 2.21794
PIMT_DROME F	446535 CI	HNMT_HUMAN Fu	439155 Ad	2AOT	1R18	1JQE		0.95	Sc=6.50881, min distance = 2.60003
PIMT_DROME F	60961 ade	PIMT_PYRFU Fu	439155 Ad	1JG1	1R18	1JG2		0.84	Sc=6.27193, min distance = 2.67139
PIMT_DROME F	65482 sir	ERM_BACSU Ful	439155 Ad	1QAN	1R18	1QAQ		0.88	Sc=6.54332, min distance = 2.45526
PIMT_DROME F	65482 sir	MTR1_RHOSH Fu	439155 Ad	1NW7	1R18	1NW6		0.88	Sc=6.53247, min distance = 2.02016
PIMT_HUMAN F	188380 Ac	MCES_ENCCU Fu	439155 Ad	1RI1	1I1N	1Z3C		0.91	Sc=6.60848, min distance = 2.51207
PIMT_HUMAN F	446535 CI	HNMT_HUMAN Fu	439155 Ad	2AOT	1I1N	1JQE		0.95	Sc=6.56277, min distance = 2.20148
PIMT_HUMAN F	60961 ade	PIMT_PYRFU Fu	439155 Ad	1JG1	1I1N	1JG2		0.84	Sc=6.34067, min distance = 2.37771
PIMT_PYRFU F	10041129	SAHH_MYCTU Fu	60961 ade	3CE6	1JG2	3DHY		0.89	Sc=6.37282, min distance = 1.93625
PIMT_PYRFU F	439176 Me	MTAP_SULSO Fu	60961 ade	1JDV	1JG2	1JDT		0.91	Sc=6.35603, min distance = 1.32801
PIMT_PYRFU F	446535 CI	HNMT_HUMAN Fu	439155 Ad	2AOT	1JG1	1JQE		0.95	Sc=6.56731, min distance = 2.15970
PIMT_PYRFU F	447722 9-	DEOD_ECOLI Fu	60961 ade	1PK7	1JG2	1OUM		0.9	Sc=6.36333, min distance = 2.36195
PIMT_PYRFU F	6021 inos	DEOD_ECOLI Fu	60961 ade	1PK7	1JG2	1PR0		0.79	Sc=6.3464, min distance = 1.678100
PIMT_PYRFU F	65482 sir	MTR1_RHOSH Fu	439155 Ad	1NW7	1JG1	1NW6		0.88	Sc=6.58684, min distance = 2.14866
PIMT_PYRFU F	72443 for	DEOD_ECOLI Fu	60961 ade	1PK7	1JG2	1A69			Sc=6.38616, min distance = 2.10984
PIMT_PYRFU F	8975 2-Fl	SAHH_MYCTU Fu	60961 ade	3CE6	1JG2	2ZJ0		0.99	Sc=6.3637, min distance = 2.158682
PIMT_PYRFU F	97184 6-M	DEOD_ECOLI Fu	60961 ade	1PK7	1JG2	1OVG		0.9	Sc=6.28344, min distance = 1.81982
PIMT_THEMA F	188380 Ac	MCES_ENCCU Fu	439155 Ad	1RI1	1DL5	1Z3C		0.91	Sc=6.58035, min distance = 2.04677
PIMT_THEMA F	446535 CI	HNMT_HUMAN Fu	439155 Ad	2AOT	1DL5	1JQE		0.95	Sc=6.53741, min distance = 2.47687

PIMT_THEMA Fu	60961 ade	PIMT_PYRFU Fu	439155 Ad	1JG1	1DL5	1JG2		0.84	Sc=6.30972, min distance = 2.624353
PIMT_THEMA Fu	65482 sir	MTR1_RHOSH Fu	439155 Ad	1NW7	1DL5	1NW6		0.88	Sc=6.56303, min distance = 2.067970
PIP_THEAC Fu	6305 L-tr	APX_STRGR Ful	6140 L-ph	1F2P	1XRM	1TF8			Sc=6.23306, min distance = 1.058740
PK3CG_HUMAN I	10109823	PIM1_HUMAN Fu	11986114	2BZI	3CSF	3CY3			Sc=6.23472, min distance = 2.433800
PK3CG_HUMAN I	11348631	CHK1_HUMAN Fu	444345 1c	1NVR	1E8Z	2E9U			Sc=6.21179, min distance = 2.270318
PK3CG_HUMAN I	11502647	CHK1_HUMAN Fu	444345 1c	1NVR	1E8Z	2GDO			Sc=6.26014, min distance = 2.369178
PK3CG_HUMAN I	11986113	PIM1_HUMAN Fu	444345 1c	1YHS	1E8Z	2BZH		0.76	Sc=6.41226, min distance = 1.999826
PK3CG_HUMAN I	11986115	PIM1_HUMAN Fu	444345 1c	1YHS	1E8Z	2BZJ			Sc=6.59117, min distance = 2.181321
PK3CG_HUMAN I	1587957 2	CHK1_HUMAN Fu	444345 1c	1NVR	1E8Z	2BRB			Sc=6.16464, min distance = 2.080478
PK3CG_HUMAN I	15942652	CDK2_HUMAN Fu	444345 1c	1AQ1	1E8Z	2DUV			Sc=6.2904, min distance = 2.0920719
PK3CG_HUMAN I	16058647	PIM1_HUMAN Fu	444345 1c	1YHS	1E8Z	2OI4		0.75	Sc=6.43701, min distance = 2.361531
PK3CG_HUMAN I	16122608	PDPK1_HUMAN F	444345 1c	1OKY	1E8Z	2PE1			Sc=6.32871, min distance = 2.352264
PK3CG_HUMAN I	16122643	CHK1_HUMAN Fu	444345 1c	1NVR	1E8Z	2YWP			Sc=6.26922, min distance = 2.492743
PK3CG_HUMAN I	16214825	CDK2_HUMAN Fu	444345 1c	1AQ1	1E8Z	2UZE			Sc=6.24783, min distance = 2.444473
PK3CG_HUMAN I	17754396	PDPK1_HUMAN F	444345 1c	1OKY	1E8Z	2R7B			Sc=6.26424, min distance = 2.625282
PK3CG_HUMAN I	1907917 2	CHK1_HUMAN Fu	444345 1c	1NVR	1E8Z	2CGW			Sc=6.13456, min distance = 2.570668
PK3CG_HUMAN I	23653515	CDK2_HUMAN Fu	444345 1c	1AQ1	1E8Z	2R3F			Sc=5.95826, min distance = 2.130316
PK3CG_HUMAN I	23653518	CDK2_HUMAN Fu	444345 1c	1AQ1	1E8Z	2R3J			Sc=6.18542, min distance = 2.185305
PK3CG_HUMAN I	23653519	CDK2_HUMAN Fu	444345 1c	1AQ1	1E8Z	2R3K			Sc=6.0678, min distance = 2.0905755
PK3CG_HUMAN I	23653520	CDK2_HUMAN Fu	444345 1c	1AQ1	1E8Z	2R3L			Sc=6.07163, min distance = 2.254866
PK3CG_HUMAN I	23657800	CHK1_HUMAN Fu	444345 1c	1NVR	1E8Z	2E9P			Sc=6.24576, min distance = 2.084684
PK3CG_HUMAN I	23727982	CDK2_HUMAN Fu	444345 1c	1AQ1	1E8Z	3BHU			Sc=6.25434, min distance = 2.277001
PK3CG_HUMAN I	2403 1uvr	PDPK1_HUMAN F	444345 1c	1OKY	1E8Z	1UVR			Sc=6.35372, min distance = 2.046245
PK3CG_HUMAN I	24963033	CDK2_HUMAN Fu	444345 1c	1AQ1	1E8Z	2W05			Sc=6.29896, min distance = 2.091824
PK3CG_HUMAN I	3547 Fasu	KAPCA_BOVIN F	444345 1c	1STC	1E8Z	1Q8W			Sc=5.89227, min distance = 2.270481
PK3CG_HUMAN I	3973 nche	PIM1_HUMAN Fu	11986114	2BZI	3CSF	1YI3			Sc=5.82145, min distance = 2.416870
PK3CG_HUMAN I	4369136 C	CDK2_HUMAN Fu	444345 1c	1AQ1	1E8Z	1DM2			Sc=6.24757, min distance = 2.204166
PK3CG_HUMAN I	4369433 2	CHK1_HUMAN Fu	444345 1c	1NVR	1E8Z	2BRM			Sc=6.25624, min distance = 2.159222
PK3CG_HUMAN I	4369434 2	CHK1_HUMAN Fu	444345 1c	1NVR	1E8Z	2BRN			Sc=6.3877, min distance = 2.4356058
PK3CG_HUMAN I	445966 O6	CDK2_HUMAN Fu	444345 1c	1AQ1	1E8Z	1H0V			Sc=6.05419, min distance = 2.553069
PK3CG_HUMAN I	447446 1n	CHK1_HUMAN Fu	444345 1c	1NVR	1E8Z	1NVS		0.84	Sc=6.2174, min distance = 2.7985944
PK3CG_HUMAN I	447766 1p	CDK2_HUMAN Fu	444345 1c	1AQ1	1E8Z	1P2A			Sc=6.2901, min distance = 2.3294211
PK3CG_HUMAN I	447962 1p	CDK2_HUMAN Fu	444345 1c	1AQ1	1E8Z	2C5N			Sc=6.08317, min distance = 2.845035
PK3CG_HUMAN I	449240 1y	KAPCA_BOVIN F	444345 1c	1STC	1E8Z	1YDR			Sc=5.86942, min distance = 2.207227
PK3CG_HUMAN I	4565 1h1r	CDK2_HUMAN Fu	444345 1c	1AQ1	1E8Z	1H1R			Sc=6.02535, min distance = 2.091302
PK3CG_HUMAN I	4566 1h1s	CDK2_HUMAN Fu	444345 1c	1AQ1	1E8Z	1H1S			Sc=6.32677, min distance = 1.887532
PK3CG_HUMAN I	5288711 1	CDK2_HUMAN Fu	444345 1c	1AQ1	1E8Z	1KE8			Sc=6.17103, min distance = 2.047217

PK3CG_HUMAN	5288712	CDK2_HUMAN Fu	444345 1c	1AQ1	1E8Z	1KE9			Sc=6.11023, min distance = 2.36286
PK3CG_HUMAN	5326844	CDK2_HUMAN Fu	444345 1c	1AQ1	1E8Z	1URW			Sc=6.44892, min distance = 2.38076
PK3CG_HUMAN	5327121	PIM1_HUMAN Fu	444345 1c	1YHS	1E8Z	2C3I			Sc=6.24016, min distance = 2.39583
PK3CG_HUMAN	5327123	CHK1_HUMAN Fu	444345 1c	1NVR	1E8Z	2C3L			Sc=6.10454, min distance = 2.46146
PK3CG_HUMAN	5331010	CDK2_HUMAN Fu	444345 1c	1AQ1	1E8Z	2BKZ			Sc=6.25806, min distance = 2.45448
PK3CG_HUMAN	6420138	CDK2_HUMAN Fu	444345 1c	1AQ1	1E8Z	2UUE			Sc=6.26622, min distance = 2.76988
PK3CG_HUMAN	6420139	CDK2_HUMAN Fu	444345 1c	1AQ1	1E8Z	2C5V			Sc=6.07813, min distance = 2.64671
PK3CG_HUMAN	848641 2b	CHK1_HUMAN Fu	444345 1c	1NVR	1E8Z	2BRH			Sc=6.23243, min distance = 2.21318
PK3CG_HUMAN	9547890	CDK2_HUMAN Fu	444345 1c	1AQ1	1E8Z	1W8C			Sc=6.09821, min distance = 2.19428
PK3CG_HUMAN	9817550	CDK2_HUMAN Fu	444345 1c	1AQ1	1E8Z	3BHV			Sc=6.25748, min distance = 2.28369
PK3CG_PIG Fu	11175137	KAPCA_BOVIN F	5957 Aden	1Q24	1E8X	2UW7			Sc=6.15073, min distance = 2.28958
PK3CG_PIG Fu	11708454	KAPCA_BOVIN F	5957 Aden	1Q24	1E8X	2VNW			Sc=6.10977, min distance = 2.38366
PK3CG_PIG Fu	15602982	KAPCA_BOVIN F	5957 Aden	1Q24	1E8X	2UW6			Sc=6.0346, min distance = 2.279292
PK3CG_PIG Fu	15602983	KAPCA_BOVIN F	5957 Aden	1Q24	1E8X	2UW5			Sc=6.08912, min distance = 2.19697
PK3CG_PIG Fu	16122633	KAPCA_BOVIN F	5957 Aden	1Q24	1E8X	2UW0			Sc=6.4194, min distance = 2.253989
PK3CG_PIG Fu	16122642	KAPCA_BOVIN F	5957 Aden	1Q24	1E8X	2UZW			Sc=6.38773, min distance = 2.03582
PK3CG_PIG Fu	17751819	KAPCA_BOVIN F	5957 Aden	1Q24	1E8X	2VO6			Sc=6.25032, min distance = 2.42563
PK3CG_PIG Fu	17754396	PDPK1_HUMAN F	5957 Aden	2BIY	1E8X	2R7B			Sc=6.29127, min distance = 2.49559
PK3CG_PIG Fu	24180721	PIM1_HUMAN Fu	3973 nche	1YI3	1E7V	3C4E			Sc=5.73329, min distance = 2.22680
PK3CG_PIG Fu	24820122	PIM1_HUMAN Fu	3973 nche	1YI3	1E7V	3BWF			Sc=6.45382, min distance = 2.08331
PK3CG_PIG Fu	3547 Fasu	KAPCA_BOVIN F	5957 Aden	1Q24	1E8X	1Q8W			Sc=5.89401, min distance = 2.03149
PK3CG_PIG Fu	440317 AT	PURT_ECOLI Fu	5957 Aden	1KJ8	1E8X	1KJJ		0.99	Sc=5.66827, min distance = 2.07811
PK3CG_PIG Fu	449240 1y	KAPCA_BOVIN F	5957 Aden	1Q24	1E8X	1YDR			Sc=5.87469, min distance = 2.07748
PK3CG_PIG Fu	5280863 k	O22304_VITVI	5280343 q	2C9Z	1E8W	2C1Z		0.94	Sc=6.26327, min distance = 2.44724
PK3CG_PIG Fu	6022 Ader	CLCN5_HUMAN F	5957 Aden	2J9L	1E8X	2JA3		0.99	Sc=5.90213, min distance = 2.33538
PK3CG_PIG Fu	6022 Ader	DDL_THET8 Ful	5957 Aden	2ZDQ	1E8X	2ZDH		0.99	Sc=5.91339, min distance = 2.07608
PK3CG_PIG Fu	6022 Ader	MUTS_ECOLI Fu	5957 Aden	1W7A	1E8X	1OH7		0.99	Sc=5.60976, min distance = 2.19596
PK3CG_PIG Fu	6022 Ader	RK_BOVIN Full	5957 Aden	3C4W	1E8X	3C4Z		0.99	Sc=5.91339, min distance = 2.14993
PK3CG_PIG Fu	611002 Op	PIM1_HUMAN Fu	5281672 m	2O63	1E90	1YXX			Sc=6.04435, min distance = 2.41496
PK3CG_PIG Fu	657135 Op	PIM1_HUMAN Fu	5281672 m	2O63	1E90	1YXV			Sc=5.87164, min distance = 2.24087
PK3CG_PIG Fu	667639 pi	ATPG_BOVIN Fu	5280343 q	2JJ2	1E8W	2JJ1			Sc=6.15459, min distance = 2.06584
PKHA1_HUMAN	51 2-Oxop	SERA_ECOLI Fu	311 citri	2P9E	1EAZ	1YBA		0.76	Sc=5.66422, min distance = 1.38874
PKNB_MYCTU F	24905142	SRC_CHICK Ful	440317 AT	3DQW	1MRU	3EN4	29.8		Sc=6.35676, min distance = 1.73958
PKNB_MYCTU F	24905143	SRC_CHICK Ful	440317 AT	3DQW	1MRU	3EN7	29.8		Sc=6.19161, min distance = 2.28165
PKNB_MYCTU F	33113 gam	CHEA_THEMA Fu	91532 AMF	1I58	1O6Y	1I59		0.99	Sc=5.72497, min distance = 2.07958
PKNB_MYCTU F	443200 C1	ACK1_HUMAN Fu	91532 AMF	1U54	1O6Y	1U4D	24.89		Sc=6.23092, min distance = 2.34133
PKNB_MYCTU F	444564 AD	BIOD_ECOLI Fu	91532 AMF	1DAG	1O6Y	1BS1		0.91	Sc=6.35352, min distance = 2.10010

PKNB_MYCTU F	444564 AD	MYS2_DICDI Fu	440317 AT	1MMG	1MRU	1W9I		0.91	Sc=5.91756, min distance = 2.06901
PKNB_MYCTU F	5287416 C	INSR_HUMAN Fu	91532 AMP	1I44	1O6Y	1RQQ	28.12	0.93	Sc=6.46186, min distance = 2.08163
PKNB_MYCTU F	5957 Aden	BIOD_ECOLI Fu	91532 AMP	1DAG	1O6Y	1A82		0.99	Sc=5.95734, min distance = 2.15374
PKNB_MYCTU F	5957 Aden	MYS2_DICDI Fu	440317 AT	1MMG	1MRU	1FMW		0.99	Sc=5.98029, min distance = 0
PKNB_MYCTU F	5957 Aden	NIFH1_AZOVI F	91532 AMP	2AFK	1O6Y	2C8V		0.99	Sc=5.97528, min distance = 2.42490
PKNB_MYCTU F	6022 Aden	BIOD_ECOLI Fu	91532 AMP	1DAG	1O6Y	1DAD		0.99	Sc=6.35458, min distance = 2.29964
PKNB_MYCTU F	6022 Aden	FTSK_PSEAE Fu	440317 AT	2IUT	1MRU	2IUU		0.99	Sc=6.36505, min distance = 2.37846
PKNB_MYCTU F	6022 Aden	MYS2_DICDI Fu	440317 AT	1MMG	1MRU	1VOM		0.99	Sc=5.87768, min distance = 2.25897
PKNB_MYCTU F	6083 aden	SRC_CHICK Ful	440317 AT	3DQW	1MRU	3DQX	29.8	0.97	Sc=6.27224, min distance = 2.72605
PLK4_HUMAN F	11553058	LCK_HUMAN Ful	448170 CI	1QPC	3COK	2OF2			Sc=6.09415, min distance = 2.35908
PMA2_ARATH F	5957 Aden	SYK2_ECOLI Fu	91532 AMP	1E22	3B8C	1E24		0.99	Sc=5.62958, min distance = 2.37494
PMG1_YEAST F	439168 Tr	TPIS_PLAFA Fu	439183 1i	1M7O	1QHF	1M7P		0.84	Sc=5.6426, min distance = 1.138465
PMG1_YEAST F	439278 ph	GPMI_BACST Fu	439183 1i	1EJJ	1QHF	1O98	1		Sc=5.65858, min distance = 2.02419
PMG1_YEAST F	447550 ph	TPIS_TRYBB Fu	439183 1i	1IIH	1QHF	4TIM	1		Sc=5.66437, min distance = 2.64824
PMS2_HUMAN F	447955 1p	CDK2_HUMAN Fu	6022 Aden	1GY3	1EA6	1PXI			Sc=5.61196, min distance = 2.30289
PMS2_HUMAN F	5326935 1	HS90A_HUMAN F	6022 Aden	1BYQ	1EA6	1YC4			Sc=6.29839, min distance = 2.61410
PMS2_HUMAN F	6323491 M	PDK3_HUMAN Fu	6022 Aden	1Y8O	1EA6	2Q8I			Sc=6.30357, min distance = 2.57587
PMS2_HUMAN F	6323491 M	TOP6B_SULSH F	6022 Aden	1Z5B	1EA6	2HKJ			Sc=6.31855, min distance = 2.12311
PMS2_HUMAN F	72194 2-C	ENPL_CANFA Fu	6022 Aden	1TC6	1EA6	1QYE		0.89	Sc=6.07636, min distance = 2.34388
PNMT_HUMAN F	188380 Ac	MCES_ENCCU Fu	439155 Ad	1RI1	2G8N	1Z3C		0.91	Sc=6.58328, min distance = 1.95408
PNMT_HUMAN F	439176 Me	MTR1_RHOSH Fu	34756 Acy	1NW5	2G72	1EG2		0.91	Sc=6.26843, min distance = 2.16371
PNMT_HUMAN F	439182 5'	Q70GK9_STRCT	34756 Acy	1RQP	2G72	2CC2		0.86	Sc=6.27634, min distance = 1.83417
PNMT_HUMAN F	446535 CI	HNMT_HUMAN Fu	439155 Ad	2AOT	2G8N	1JQE		0.95	Sc=6.5575, min distance = 1.962062
PNMT_HUMAN F	5326531 C	CHOMT_MEDSA F	34756 Acy	1FPQ	2G72	1FP1		0.92	Sc=6.55519, min distance = 2.04559
PNMT_HUMAN F	60961 ade	PIMT_PYRFU Fu	439155 Ad	1JG1	2G8N	1JG2		0.84	Sc=6.27869, min distance = 2.58260
PNPH_BOVIN F	447992 1c	Q8I3X4_PLAF7	6021 inos	2BSX	1A9S	1Q1G			Sc=6.35829, min distance = 2.57138
PNPH_HUMAN F	100684 9-	TGT_ZYMMO Ful	764 guani	2PWU	1V2H	1Q2R			Sc=5.86412, min distance = 2.06068
PNPH_HUMAN F	108714 9-	URIC_ASPFL Fu	764 guani	1XT4	1V2H	1R4S			Sc=5.95799, min distance = 2.07317
PNPH_HUMAN F	1188 xant	XAPA_ECOLI Fu	764 guani	1YQQ	1V2H	1YR3	46.08	0.92	Sc=5.82604, min distance = 2.25637
PNPH_HUMAN F	1188 xant	XGPT_ECOLI Fu	764 guani	1A95	1V2H	1A96		0.92	Sc=5.81604, min distance = 2.50297
PNPH_HUMAN F	15113 Xar	URIC_ASPFL Fu	764 guani	1XT4	1V2H	2IBA			Sc=5.78932, min distance = 2.21093
PNPH_HUMAN F	242831 2-	TGT_ZYMMO Ful	764 guani	2PWU	1V2H	1S39			Sc=5.92919, min distance = 2.14106
PNPH_HUMAN F	439713 (F	URAD_DANRE Fu	764 guani	2O74	1V2H	2O73			Sc=5.81927, min distance = 1.85624
PNPH_HUMAN F	446221 9-	PUNA_MYCTU Fu	447268 DA	1N3I	1RSZ	1I80	39.31	0.78	Sc=5.75163, min distance = 2.65846
PNPH_HUMAN F	446728 CI	Q9GPQ4_TRYVI	6021 inos	1KIC	1RCT	1KIE			Sc=6.27463, min distance = 2.04926
PNPH_HUMAN F	447864 2'	DEOD_ECOLI Fu	6021 inos	1PR0	1RCT	1PKE		0.75	Sc=6.23494, min distance = 2.31812
PNPH_HUMAN F	448488 1s	TGT_ZYMMO Ful	764 guani	2PWU	1V2H	1S38			Sc=6.00198, min distance = 2.08276

PNPH_HUMAN Fu	4604 Oxor	URIC_ASPFL Ful	764 guani	1XT4	1V2H	1R4U			Sc=5.78234, min distance = 2.36542
PNPH_HUMAN Fu	5326622 C	URIC_ASPFL Ful	764 guani	1XT4	1V2H	3BJP			Sc=5.8845, min distance = 2.036155
PNPH_HUMAN Fu	60961 ade	DEOD_ECOLI Ful	6021 inos	1PR0	1RCT	1PK7		0.79	Sc=6.27945, min distance = 1.96403
PNPH_HUMAN Fu	76726 5,6	URIC_ASPFL Ful	764 guani	1XT4	1V2H	1WS2			Sc=5.72885, min distance = 1.81698
PNPH_HUMAN Fu	97184 6-M	DEOD_ECOLI Ful	6021 inos	1PR0	1RCT	1OVG			Sc=6.23989, min distance = 1.54924
PNPO_HUMAN Fu	5326566 l	BLVRB_HUMAN Fu	444243 FA	1HE4	1NRG	1HE5		0.82	Sc=6.16763, min distance = 2.52553
PNPO_HUMAN Fu	5326566 l	Q9WZW1_THEMA l	444243 FA	1T6Y	1NRG	1S4M		0.82	Sc=6.19851, min distance = 1.99133
PNTAA_RHORT l	5957 Ader	PPNK_ARCFU Ful	5893 nadi	1Z0Z	1L7E	1Z0S			Sc=6.43744, min distance = 0.71255
PNTAA_RHORT l	6022 Ader	PNTAA_RHORU Fu	5893 nadi	2FSV	1L7E	2FR8			Sc=6.38293, min distance = 2.26152
PNTAA_RHORU l	448826 AE	PNTAA_RHORT Fu	5893 nadi	1L7E	2FSV	1U2G			Sc=6.16472, min distance = 1.81196
PNTB_RHORT Fu	15942680	6PGD_LACLM Ful	5886 NADF	2IZ0	1PNO	2IZ1			Sc=6.27439, min distance = 2.09479
PNTB_RHORT Fu	16040277	PNTB_RHORU Ful	5886 NADF	2FSV	1PNO	2OOR		0.79	Sc=6.82883, min distance = 2.22840
PNTB_RHORT Fu	440141 9i	DHB1_HUMAN Ful	5886 NADF	1QYV	1PNO	1QYW			Sc=6.47291, min distance = 2.09195
PNTB_RHORU Fu	15942680	6PGD_LACLM Ful	5886 NADF	2IZ0	2FSV	2IZ1			Sc=6.34618, min distance = 0.74059
PNTB_RHORU Fu	3080718 C	NNTM_HUMAN Ful	5886 NADF	1DJL	2FSV	1PT9		0.9	Sc=6.81757, min distance = 2.67215
PNTB_RHORU Fu	440141 9i	DHB1_HUMAN Ful	5886 NADF	1QYV	2FSV	1QYW			Sc=6.45927, min distance = 2.25650
POLG_CXA21 Fu	65052 4-H	INS_HUMAN Full	69492 Myr	1XDA	1Z7S	1BEN			Sc=5.61464, min distance = 1.75196
POLG_CXB3W Fu	175468 12	LACB_BOVIN Ful	985 palmi	1GXA	1COV	1BSO		0.83	Sc=5.77273, min distance = 3.11985
POLG_CXB3W Fu	5496506 s	POLG_POL1M Ful	985 palmi	1HXS	1COV	1AL2			Sc=6.14751, min distance = 2.76532
POLG_CXB3W Fu	91486 Sph	POLG_POL1M Ful	985 palmi	1HXS	1COV	2PLV			Sc=6.13845, min distance = 2.15544
POLG_DEN2P Fu	3059 difl	ALBU_HUMAN Ful	311 citri	1TF0	2P41	2BXE			Sc=5.84628, min distance = 2.26499
POLG_DEN2P Fu	447532 1d	SRC_HUMAN Full	311 citri	1O4L	2P41	1O4H			Sc=5.98826, min distance = 2.52041
POLG_DEN2P Fu	8582 Inos	RNAS1_BOVIN Fu	6804 guan	1RNC	2P3Q	1Z6D		0.99	Sc=6.3486, min distance = 2.299149
POLG_DEN2T Fu	439680 be	HEMA_I63A3 Ful	82313 1rd	1MQL	1TG8	1MQM			Sc=5.65341, min distance = 2.02189
POLG_DEN4T Fu	446090 1h	NDKC_DICDI Ful	6022 Aden	1KDN	2JLX	1HIY		0.97	Sc=6.43556, min distance = 1.71279
POLG_DEN4T Fu	447916 ad	RIO1_ARCFU Ful	6022 Aden	1ZTH	2JLX	1ZTF		0.95	Sc=6.18281, min distance = 1.81918
POLG_DEN4T Fu	6083 ader	PURP_METJA Ful	6022 Aden	2R7N	2JLX	2R7M		0.99	Sc=5.9546, min distance = 2.603224
POLG_DEN4T Fu	6804 guar	NDK_PYRHO Full	6022 Aden	2DYA	2JLX	2DXF		0.8	Sc=6.0035, min distance = 2.130187
POLG_DEN4T Fu	8977 1dar	PARM_ECOLX Ful	6022 Aden	1MWM	2JLX	2ZGY		0.8	Sc=6.44121, min distance = 2.18571
POLG_EC01F Fu	151008 N-	CPXB_BACME Ful	985 palmi	1FAG	1EV1	1JPZ			Sc=6.27735, min distance = 2.07578
POLG_EC01F Fu	175468 12	LACB_BOVIN Ful	985 palmi	1GXA	1EV1	1BSO		0.83	Sc=5.81768, min distance = 2.61029
POLG_EC01F Fu	448769 1t	FABP4_HUMAN Fu	985 palmi	2HNX	1EV1	1TOW			Sc=5.98339, min distance = 2.15501
POLG_EC01F Fu	5280934 l	NLTP_MAIZE Ful	985 palmi	1MZM	1EV1	1FK6		0.75	Sc=5.99435, min distance = 2.38329
POLG_HRV14 Fu	5496506 s	POLG_POL1M Ful	91486 Sph	2PLV	1K5M	1AL2	66.07		Sc=6.01519, min distance = 2.20314
POLG_HRV16 Fu	127504 AI	POLG_HRV14 Ful	1684 Plec	1NCQ	1NCR	1R09	55.31		Sc=5.99657, min distance = 2.14644
POLG_HRV16 Fu	1360 AIDS	POLG_HRV14 Ful	1684 Plec	1NCQ	1NCR	1HRI	55.31		Sc=5.8752, min distance = 2.208655
POLG_HRV16 Fu	448417 W8	POLG_HRV14 Ful	1684 Plec	1NCQ	1NCR	1RUD	55.31		Sc=6.1583, min distance = 2.527740

POLG_HRV16 Fu	449079 SD	POLG_HRV14 Ful	1684 Plec	1NCQ	1NCR	1VRH	55.31		Sc=6.11128, min distance = 1.89751
POLG_HRV16 Fu	449391 W4	POLG_HRV14 Ful	1684 Plec	1NCQ	1NCR	2RM2	55.31		Sc=6.16961, min distance = 2.05745
POLG_HRV16 Fu	449395 W8	POLG_HRV14 Ful	1684 Plec	1NCQ	1NCR	2RR1	55.31		Sc=6.15527, min distance = 2.46504
POLG_HRV16 Fu	449396 W5	POLG_HRV14 Ful	1684 Plec	1NCQ	1NCR	2RS3	55.31		Sc=6.18583, min distance = 2.13224
POLG_HRV16 Fu	449397 Co	POLG_HRV14 Ful	1684 Plec	1NCQ	1NCR	2RS5	55.31		Sc=6.09451, min distance = 1.74006
POLG_HRV16 Fu	73181 AID	POLG_HRV14 Ful	1684 Plec	1NCQ	1NCR	2HWC	55.31		Sc=6.10276, min distance = 2.66243
POLG_HRV1A Fu	1360 AIDS	POLG_HRV14 Ful	449352 CI	2HWB	2HWD	1HRI	43.92		Sc=5.78796, min distance = 2.78171
POLG_HRV1A Fu	1360 AIDS	POLG_HRV14 Ful	73181 AID	2HWC	2HWE	1HRI	43.92		Sc=5.78118, min distance = 2.22670
POLG_HRV1A Fu	1684 Plec	POLG_HRV14 Ful	449352 CI	2HWB	2HWD	1NCQ	43.92		Sc=5.91647, min distance = 2.59423
POLG_HRV1A Fu	448416 W3	POLG_HRV14 Ful	449352 CI	2HWB	2HWD	1RUE	43.92	0.9	Sc=5.83992, min distance = 2.90141
POLG_HRV1A Fu	448416 W3	POLG_HRV14 Ful	73181 AID	2HWC	2HWE	1RUE	43.92	0.92	Sc=5.84585, min distance = 2.80611
POLG_HRV1A Fu	449397 Co	POLG_HRV14 Ful	73181 AID	2HWC	2HWE	2RS5	43.92		Sc=5.89372, min distance = 2.66392
POLG_KUNJM Fu	188380 Ad	MCES_ENCCU Ful	439155 Ad	1RI1	2OY0	1Z3C		0.91	Sc=6.22601, min distance = 2.54341
POLG_KUNJM Fu	21945395	SETD7_HUMAN Fu	439155 Ad	2F69	2OY0	1XQH		0.88	Sc=6.18322, min distance = 1.85128
POLG_KUNJM Fu	446535 CI	HNMT_HUMAN Ful	439155 Ad	2AOT	2OY0	1JQE		0.95	Sc=6.49376, min distance = 2.25896
POLG_KUNJM Fu	60961 ade	PIMT_PYRFU Ful	439155 Ad	1JG1	2OY0	1JG2		0.84	Sc=6.27193, min distance = 2.54073
POLG_KUNJM Fu	65482 sin	ERM_BACSU Full	439155 Ad	1QAN	2OY0	1QAQ		0.88	Sc=6.55441, min distance = 2.51525
POLG_KUNJM Fu	65482 sin	MCES_ENCCU Ful	439155 Ad	1RI1	2OY0	2HV9		0.88	Sc=6.52461, min distance = 2.61669
POLG_KUNJM Fu	65482 sin	PRMA_THET8 Ful	439155 Ad	3EGV	2OY0	2ZBR		0.88	Sc=6.54918, min distance = 2.40188
POLG_MVEV5 Fu	12771338	MCES_HUMAN Ful	439155 Ad	3BGV	2PX2	3EPP		0.88	Sc=6.54452, min distance = 2.49554
POLG_MVEV5 Fu	188380 Ad	MCES_ENCCU Ful	439155 Ad	1RI1	2PX2	1Z3C		0.91	Sc=6.54168, min distance = 2.67616
POLG_MVEV5 Fu	445971 CI	COMT_RAT Full=	34756 Acy	2CL5	2PXC	1H1D		0.92	Sc=6.53097, min distance = 2.30170
POLG_MVEV5 Fu	446535 CI	HNMT_HUMAN Ful	439155 Ad	2AOT	2PX2	1JQE		0.95	Sc=6.49214, min distance = 2.36632
POLG_MVEV5 Fu	60961 ade	PIMT_PYRFU Ful	439155 Ad	1JG1	2PX2	1JG2		0.84	Sc=6.2837, min distance = 2.448278
POLG_MVEV5 Fu	65482 sin	ERM_BACSU Full	439155 Ad	1QAN	2PX2	1QAQ		0.88	Sc=6.56065, min distance = 2.02028
POLG_MVEV5 Fu	65482 sin	MCES_ENCCU Ful	439155 Ad	1RI1	2PX2	2HV9		0.88	Sc=6.51645, min distance = 2.53869
POLG_MVEV5 Fu	65482 sin	PRMA_THET8 Ful	439155 Ad	3EGV	2PX2	2ZBR		0.88	Sc=6.55, min distance = 2.27682190
POLG_MVEV5 Fu	6852187 2	Q70GK9_STRCT I	34756 Acy	1RQP	2PXC	2CBX		0.84	Sc=6.17859, min distance = 1.26692
POLH_YEAST Fu	119119 Di	DPO42_SULSO Fu	65091 dCT	2ASD	2R8J	1S97		0.93	Sc=6.15478, min distance = 2.20241
POLH_YEAST Fu	15942678	DPOLB_HUMAN Fu	65091 dCT	1MQ3	2R8J	2ISO			Sc=5.9854, min distance = 2.361951
POLH_YEAST Fu	187790 de	DPOL_BPR69 Ful	65091 dCT	1Q9Y	2R8J	1Q9X			Sc=6.10761, min distance = 2.41870
POLH_YEAST Fu	64968 dTT	DPO42_SULSO Fu	65091 dCT	2ASD	2R8J	1S0O		0.75	Sc=5.63416, min distance = 2.75959
POLH_YEAST Fu	64968 dTT	DPOLL_HUMAN Fu	65091 dCT	2PFP	2R8J	2BCV		0.75	Sc=5.6228, min distance = 2.695279
POLH_YEAST Fu	64968 dTT	DPOL_BPR69 Ful	65091 dCT	1Q9Y	2R8J	3CQ8		0.75	Sc=5.62891, min distance = 2.59706
POLH_YEAST Fu	64968 dTT	POLI_HUMAN Ful	65091 dCT	2DPI	2R8J	1T3N		0.75	Sc=5.63907, min distance = 2.53235
POLH_YEAST Fu	65103 dGT	DPO42_SULSO Fu	65091 dCT	2ASD	2R8J	2JEJ			Sc=5.94245, min distance = 2.58275
POLI_HUMAN Fu	65103 dGT	DPO42_SULSO Fu	65091 dCT	2ASD	2DPI	2JEJ			Sc=5.63523, min distance = 2.65855

POLI_HUMAN Fu	656930 DD	DPO42_SULSO Fu	64968 dTT	1S0O	1T3N	1S9F			Sc=6.09005, min distance = 2.35014
POLK_HUMAN Fu	5481180 E	POL_HV1B1 Ful	64968 dTT	1RTD	2OH2	1T05			Sc=5.90035, min distance = 1.88376
POLK_HUMAN Fu	65051 ddT	DPOLL_HUMAN Fu	64968 dTT	2BCV	2OH2	1XSN		0.94	Sc=5.62537, min distance = 2.42197
POLK_HUMAN Fu	656930 DD	DPO42_SULSO Fu	64968 dTT	1S0O	2OH2	1S9F			Sc=6.1728, min distance = 2.448480
POL_HV192 Fu	16122613	POL_HV1BR Ful	5362440 i	1SDU	2R5P	2PK5			Sc=6.20297, min distance = 2.62235
POL_HV192 Fu	16122614	POL_HV1BR Ful	5362440 i	1SDU	2R5P	2PK6		0.76	Sc=6.33327, min distance = 2.32429
POL_HV192 Fu	392622 ri	POL_HV1BR Ful	5362440 i	1SDU	2R5P	1RL8			Sc=6.07904, min distance = 1.75051
POL_HV192 Fu	392622 ri	POL_HV1BR Ful	64143 nel	2PYN	2R5Q	1RL8			Sc=5.90661, min distance = 2.33161
POL_HV192 Fu	468045 1i	POL_HV1BR Ful	5362440 i	1SDU	2R5P	1IZI			Sc=6.19527, min distance = 1.96324
POL_HV1A2 Fu	65023 1hi	POL_HV2RO Ful	213039 Da	3EBZ	1T3R	1HII	50		Sc=6.20578, min distance = 2.45120
POL_HV1B1 Fu	119119 Di	DPO42_SULSO Fu	64968 dTT	1S0O	1RTD	1S97			Sc=5.74463, min distance = 1.85491
POL_HV1B1 Fu	15993 dAT	DPOL_BPR69 Ful	64968 dTT	3CQ8	1RTD	2OZS			Sc=5.92247, min distance = 2.32075
POL_HV1B1 Fu	64689 bet	P74325_SYNY3 I	5988 sucr	1TJ5	2BE2	1U2S		0.87	Sc=5.76862, min distance = 2.53053
POL_HV1B1 Fu	65091 dCT	DPO42_SULSO Fu	64968 dTT	1S0O	1RTD	2ASD		0.75	Sc=5.82044, min distance = 2.28037
POL_HV1B1 Fu	656930 DD	DPO42_SULSO Fu	64968 dTT	1S0O	1RTD	1S9F			Sc=6.13604, min distance = 2.43101
POL_HV1B5 Fu	65023 1hi	POL_HV2RO Ful	213039 Da	3EBZ	3D20	1HII	57.58		Sc=6.18438, min distance = 2.76322
POL_HV1BR Fu	65023 1hi	POL_HV2RO Ful	457409 AI	3ECG	2QD7	1HII	58.35		Sc=6.02573, min distance = 2.83907
POL_HV1BR Fu	65023 1hi	POL_HV2RO Ful	5362440 i	1HSH	1SDU	1HII	58.35		Sc=6.25904, min distance = 2.69351
POL_HV1N5 Fu	4775 Benz	THER_BACTH Fu	12136 Ben	1KRO	9HVP	1THL			Sc=5.716, min distance = 2.0369180
POL_HV1N5 Fu	51 2-Oxop	SERA_ECOLI Fu	311 citri	2P9E	2GON	1YBA		0.76	Sc=5.73246, min distance = 1.01248
POL_HV1Y2 Fu	392622 ri	POL_HV1A2 Ful	213039 Da	1T3R	1T7I	1N49			Sc=5.90131, min distance = 2.18047
POL_HV1Y2 Fu	392622 ri	POL_HV1BR Ful	5362440 i	1SDU	1K6C	1RL8			Sc=6.07132, min distance = 1.40210
POL_HV1Y2 Fu	444480 1b	POL_HV1A2 Ful	213039 Da	1T3R	1T7I	1B6L			Sc=5.93139, min distance = 2.50958
POL_HV2RO Fu	148192 At	POL_HV1B1 Ful	65023 1hi	1HIH	1HII	2AQU	60		Sc=6.08712, min distance = 2.60555
POL_HV2RO Fu	16122613	POL_HV1BR Ful	5362440 i	1SDU	1HSH	2PK5	58.35		Sc=6.30641, min distance = 2.67416
POL_HV2RO Fu	16122614	POL_HV1BR Ful	457409 AI	2QD7	3ECG	2PK6	58.35		Sc=6.39814, min distance = 2.08421
POL_HV2RO Fu	16122614	POL_HV1BR Ful	5362440 i	1SDU	1HSH	2PK6	58.35	0.76	Sc=6.31824, min distance = 2.52476
POL_HV2RO Fu	392622 ri	POL_HV1A2 Ful	213039 Da	1T3R	3EBZ	1N49	50		Sc=6.17574, min distance = 1.47785
POL_HV2RO Fu	392622 ri	POL_HV1BR Ful	457409 AI	2QD7	3ECG	1RL8	58.35		Sc=6.03646, min distance = 2.13409
POL_HV2RO Fu	392622 ri	POL_HV1BR Ful	5362440 i	1SDU	1HSH	1RL8	58.35		Sc=6.12676, min distance = 1.77754
POL_HV2RO Fu	441243 Fo	O09893_9HIV1 I	5362440 i	1C6Y	1HSH	1C6Z	49.49	0.77	Sc=6.1549, min distance = 2.133271
POL_HV2RO Fu	444192 1g	POL_HV1B1 Ful	65023 1hi	1HIH	1HII	1AXA	60		Sc=5.9039, min distance = 2.304364
POL_HV2RO Fu	444192 1g	POL_HV1B1 Ful	9547976 2	2HB3	3EC0	1AXA	60		Sc=6.69056, min distance = 2.50484
POL_HV2RO Fu	444480 1b	POL_HV1A2 Ful	213039 Da	1T3R	3EBZ	1B6L	50		Sc=6.0354, min distance = 2.456536
POL_HV2RO Fu	444756 2b	O09893_9HIV1 I	5362440 i	1C6Y	1HSH	1C6X	49.49	0.77	Sc=5.80283, min distance = 2.11195
POL_HV2RO Fu	445303 IN	POL_HV1B1 Ful	65023 1hi	1HIH	1HII	1EBW	60		Sc=5.66432, min distance = 2.35404
POL_HV2RO Fu	445308 1e	POL_HV1B1 Ful	65023 1hi	1HIH	1HII	1EC2	60		Sc=5.61317, min distance = 2.35194

POL_HV2RO Fu	445309 1e	POL_HV1B1 Ful	65023 1hi	1HIH	1HII	1EC3	60		Sc=5.71822, min distance = 2.33033
POL_SIVM1 Fu	392622 ri	POL_HV1BR Ful	444196 1a	1AAQ	1SIV	1RL8			Sc=5.972, min distance = 1.0350173
POL_SIVM1 Fu	448921 CI	POL_HV1BR Ful	444196 1a	1AAQ	1SIV	1UPJ			Sc=5.92304, min distance = 2.334268
POXB_LACPL F	124687 1r	ODP1_ECOLI Fu	1132 thia	1L8A	2EZ4	1RP7		0.79	Sc=6.02172, min distance = 2.098200
POXB_LACPL F	24836827	BZNB_PSEFL Fu	1132 thia	2UZ1	2EZ4	3D7K			Sc=5.94705, min distance = 1.885306
POXB_LACPL F	444421 CI	MDLC_PSEPU Fu	1132 thia	1BFD	2EZ4	2FWN	24.86	0.82	Sc=6.40926, min distance = 2.566281
POXB_LACPL F	448671 2-	ILVB_YEAST Fu	1132 thia	1N0H	2EZ4	1T9B	26.02		Sc=5.72421, min distance = 2.914337
POXB_LACPL F	448673 PE	ILVB_YEAST Fu	1132 thia	1N0H	2EZ4	1T9B	26.02		Sc=5.84383, min distance = 2.203907
POXB_LACPL F	448722 1t	TKT1_YEAST Fu	1132 thia	1TRK	2EZ4	1TKB		0.78	Sc=6.42957, min distance = 2.427400
POXB_LACPL F	6802 guar	RNAS1_BOVIN F	6022 Aden	1O0H	1Y9D	1EOW		0.75	Sc=6.20436, min distance = 1.626279
PP1B_HUMAN F	439353 be	O96048_LUMTE	8172 Trig	2DRY	1S70	2DS0			Sc=5.69804, min distance = 1.639053
PPAC_BOVIN F	125409 be	XYNA_BACSU Fu	23831 HEP	2B45	1DG9	2QZ3			Sc=5.60724, min distance = 2.093978
PPAC_BOVIN F	164795 N(	RBCMT_PEA Ful	23831 HEP	1MLV	1DG9	2H2J			Sc=5.81535, min distance = 2.574485
PPAC_BOVIN F	445939 1g	PPTA_ECOLI Fu	23831 HEP	1GYX	1DG9	1GYY			Sc=5.90396, min distance = 2.272750
PPAC_BOVIN F	854023 Ep	Q8WSF8_APLCA	23831 HEP	2BR7	1DG9	2BYQ			Sc=5.72819, min distance = 1.934840
PPAC_STRA5 F	6140 L-ph	THER_BACTH Fu	6305 L-tr	1THL	2ENX	1OS0			Sc=5.80323, min distance = 2.158798
PPAL_YEAST F	100684 9-	RICI_RICCO Fu	190 adeni	2P8N	1D2A	1IL4			Sc=5.65237, min distance = 2.469740
PPAL_YEAST F	11979 5-M	COBT_SALTY Fu	190 adeni	1JH8	1D2A	1JHM			Sc=5.65339, min distance = 2.490355
PPAL_YEAST F	164795 N(	RBCMT_PEA Ful	23831 HEP	1MLV	1D1P	2H2J			Sc=5.86794, min distance = 1.814613
PPAL_YEAST F	445939 1g	PPTA_ECOLI Fu	23831 HEP	1GYX	1D1P	1GYY			Sc=5.89451, min distance = 2.133929
PPAL_YEAST F	446312 1i	RICI_RICCO Fu	190 adeni	2P8N	1D2A	1IL9			Sc=5.67725, min distance = 3.030796
PPAL_YEAST F	5287547 6	PURR_ECOLI Fu	190 adeni	2PUB	1D2A	2PUA		0.9	Sc=5.72381, min distance = 2.731667
PPAL_YEAST F	5287830 6	Q8RLY5_LACHE	190 adeni	1S2D	1D2A	1S2I		0.93	Sc=5.68688, min distance = 2.192215
PPAL_YEAST F	78598 5-M	COBT_SALTY Fu	190 adeni	1JH8	1D2A	1JHP			Sc=5.71485, min distance = 2.129349
PPAL_YEAST F	8200 TETR	Q8WSF8_APLCA	23831 HEP	2BR7	1D1P	2BYN			Sc=5.72488, min distance = 1.093572
PPAP_HUMAN F	854023 Ep	Q8WSF8_APLCA	62551 Pen	2BYN	1ND6	2BYQ			Sc=5.77382, min distance = 1.255766
PPARA_HUMAN	445655 ro	PPARG_HUMAN F	208901 Te	1I7I	1I7G	1ZGY	64.06		Sc=6.31231, min distance = 2.648534
PPARA_HUMAN	447458 Ra	PPARG_HUMAN F	208901 Te	1I7I	1I7G	1NYX	64.06		Sc=6.24391, min distance = 1.936802
PPARA_HUMAN	9549225 2	PPARG_HUMAN F	208901 Te	1I7I	1I7G	2GTK	64.06		Sc=6.3229, min distance = 1.710282
PPARG_HUMAN	9956726	PPARA_HUMAN F	208901 Te	1I7G	1I7I	2P54	64.06		Sc=6.41883, min distance = 0.659740
PPB1_HUMAN F	5962 L-ly	THER_BACTH Fu	6140 L-ph	1OS0	1ZEF	1KEI			Sc=5.65103, min distance = 2.201592
PPB1_HUMAN F	6305 L-tr	THER_BACTH Fu	6140 L-ph	1OS0	1ZEF	1THL			Sc=6.01085, min distance = 2.007333
PPB1_HUMAN F	71083 D-H	CAH2_HUMAN Fu	6140 L-ph	2FMG	1ZEF	2EZ7			Sc=5.82924, min distance = 1.902584
PPCKC_HUMAN	36735 Gpp	FTSY_THEAQ Fu	93082 Gab	1RJ9	1KHB	2J7P		0.99	Sc=6.49234, min distance = 2.462650
PPCKC_HUMAN	36735 Gpp	RAC3_HUMAN Fu	93082 Gab	2QME	1KHB	2IC5		0.99	Sc=6.49679, min distance = 2.570999
PPCKC_HUMAN	446248 CI	RASH_HUMAN Fu	93082 Gab	121P	1KHB	1IAQ			Sc=6.48532, min distance = 2.392575
PPCKC_HUMAN	8977 1dar	FTSY_THEAQ Fu	93082 Gab	1RJ9	1KHB	2IYL		0.99	Sc=6.41775, min distance = 2.102174

PPCKC_RAT Fu	164795 N(	RBCMT_PEA Ful	23831 HEP	1MLV	3DTB	2H2J		Sc=5.68874, min distance = 1.89686
PPCKC_RAT Fu	37792 gam	GNAI1_RAT Ful	8977 1dar	1SVK	3DTB	1AS0	0.99	Sc=6.5042, min distance = 2.534577
PPCKC_RAT Fu	37792 gam	GNAT1_BOVIN F	8977 1dar	1TAD	3DTB	1TND	0.99	Sc=6.51479, min distance = 2.20027
PPCKC_RAT Fu	37792 gam	RAC1_HUMAN Fu	8977 1dar	1RYF	3DTB	2FJU	0.99	Sc=6.52415, min distance = 2.46182
PPCKC_RAT Fu	37792 gam	RHOA_HUMAN Fu	8977 1dar	1TX4	3DTB	1CXZ	0.99	Sc=6.51705, min distance = 2.40537
PPCKC_RAT Fu	439710 rh	IGKC_MOUSE Fu	23831 HEP	1P7K	3DTB	3BZ4		Sc=5.74159, min distance = 0.87661
PPCKC_RAT Fu	444845 1c	RASH_HUMAN Fu	8977 1dar	2CE2	3DTB	1CLU	0.97	Sc=6.55509, min distance = 1.55062
PPCKC_RAT Fu	445871 CI	RASH_HUMAN Fu	8977 1dar	2CE2	3DTB	1GNP		Sc=6.70536, min distance = 2.29541
PPCKC_RAT Fu	446248 CI	RAN_HUMAN Ful	8977 1dar	3CH5	3DTB	1IBR		Sc=6.53083, min distance = 2.28614
PPCKC_RAT Fu	448405 CI	ARF1_RAT Full	8977 1dar	1RRG	3DTB	1RRF		Sc=6.45364, min distance = 2.43880
PPCKC_RAT Fu	5962 L-ly	RBCMT_PEA Ful	23831 HEP	1MLV	3DTB	2H2E		Sc=5.60731, min distance = 1.68438
PPCKC_RAT Fu	6802 guar	DPOL_BPR69 Fu	8977 1dar	1CLQ	3DTB	1IH7	0.94	Sc=6.27945, min distance = 2.06283
PPCKC_RAT Fu	6804 guar	EF1A_YEAST Fu	8977 1dar	2B7B	3DTB	1G7C	0.99	Sc=6.39535, min distance = 2.65098
PPCKC_RAT Fu	8582 Inos	PURA1_MOUSE F	8977 1dar	1LON	3DTB	1IWE	0.99	Sc=6.38508, min distance = 2.62541
PPCKC_RAT Fu	93082 Gab	CDC42_HUMAN F	8977 1dar	2NGR	3DTB	2ODB	0.99	Sc=6.54922, min distance = 2.57084
PPCKC_RAT Fu	93082 Gab	EFG_THETH Ful	8977 1dar	2BM0	3DTB	2J7K	0.99	Sc=6.53811, min distance = 1.85645
PPCKC_RAT Fu	93082 Gab	FTSY_THEAQ Fu	8977 1dar	2IYL	3DTB	1RJ9	0.99	Sc=6.58952, min distance = 2.02344
PPCKC_RAT Fu	93082 Gab	RAC3_HUMAN Fu	8977 1dar	2C2H	3DTB	2QME	0.99	Sc=6.55157, min distance = 2.36780
PPCKC_RAT Fu	93082 Gab	RASH_HUMAN Fu	6830 guan	2CL7	3DT4	121P	0.99	Sc=6.54206, min distance = 2.24137
PPCKC_RAT Fu	93082 Gab	RASH_HUMAN Fu	8977 1dar	2CE2	3DTB	121P	0.99	Sc=6.54319, min distance = 2.20101
PPCKM_CHICK	37792 gam	GNAI1_RAT Ful	8977 1dar	1SVK	2FAH	1AS0	0.99	Sc=6.51057, min distance = 2.47216
PPCKM_CHICK	37792 gam	RAB6B_HUMAN F	8977 1dar	2E9S	2FAH	2FFQ	0.99	Sc=6.52116, min distance = 2.48834
PPCKM_CHICK	37792 gam	RAC1_HUMAN Fu	8977 1dar	1RYF	2FAH	2FJU	0.99	Sc=6.533, min distance = 2.4338284
PPCKM_CHICK	37792 gam	RB11A_HUMAN F	8977 1dar	1OIX	2FAH	1OIW	0.99	Sc=6.06948, min distance = 2.55318
PPCKM_CHICK	37792 gam	RHOA_HUMAN Fu	8977 1dar	1TX4	2FAH	1CXZ	0.99	Sc=6.52457, min distance = 2.39860
PPCKM_CHICK	446248 CI	RAN_HUMAN Ful	8977 1dar	3CH5	2FAH	1IBR		Sc=6.54024, min distance = 2.31665
PPCKM_CHICK	6802 guar	DPOL_BPR69 Fu	8977 1dar	1CLQ	2FAH	1IH7	0.94	Sc=6.28916, min distance = 2.03403
PPCKM_CHICK	6804 guar	EF1A_YEAST Fu	8977 1dar	2B7B	2FAH	1G7C	0.99	Sc=6.39217, min distance = 2.62102
PPCKM_CHICK	8582 Inos	PURA1_MOUSE F	8977 1dar	1LON	2FAH	1IWE	0.99	Sc=6.38203, min distance = 2.59584
PPCKM_CHICK	93082 Gab	CDC42_HUMAN F	8977 1dar	2NGR	2FAH	2ODB	0.99	Sc=6.55438, min distance = 2.56565
PPCK_ANASU F	444564 AD	MYS2_DICDI Fu	5957 Aden	1FMW	1YTM	1W9I	0.91	Sc=6.01482, min distance = 0
PPCK_ANASU F	447916 ac	RIO1_ARCFU Fu	5957 Aden	1ZP9	1YTM	1ZTF	0.94	Sc=6.26717, min distance = 2.43532
PPCK_ANASU F	6022 Ader	ARSA1_ECOLX F	5957 Aden	1II0	1YTM	1IHU	0.99	Sc=6.0267, min distance = 2.178008
PPCK_ANASU F	6022 Ader	BIOD_ECOLI Fu	5957 Aden	1A82	1YTM	1DAD	0.99	Sc=6.01482, min distance = 2.53882
PPCK_ANASU F	6022 Ader	HSP7F_YEAST F	5957 Aden	3D2F	1YTM	3C7N	0.99	Sc=6.01546, min distance = 2.25178
PPCK_ANASU F	6022 Ader	KTHY_HUMAN Fu	5957 Aden	1E2Q	1YTM	1NN3	0.99	Sc=6.01776, min distance = 2.32225
PPCK_ANASU F	6022 Ader	Q72H90_THET2	5957 Aden	2BEK	1YTM	2BEJ	0.99	Sc=6.0094, min distance = 2.277014

PPCK_ANASU F	6338561 C	MYS2_DICDI Fu	5957 Aden	1FMW	1YTM	1D0X		Sc=6.2536, min distance = 0
PPCK_ANASU F	6338562 C	MYS2_DICDI Fu	5957 Aden	1FMW	1YTM	1D0Y		Sc=6.2583, min distance = 0
PPCK_ANASU F	6338566 C	MYS2_DICDI Fu	5957 Aden	1FMW	1YTM	1D1C		Sc=6.2528, min distance = 0
PPCK_ECOLI F	11708454	KAPCA_BOVIN F	5957 Aden	1Q24	2OLR	2VNW		Sc=6.1587, min distance = 2.144384
PPCK_ECOLI F	444564 AD	BIOD_ECOLI Fu	5957 Aden	1A82	2OLR	1BS1	0.91	Sc=6.46828, min distance = 2.35772
PPCK_ECOLI F	444564 AD	MYS2_DICDI Fu	5957 Aden	1FMW	2OLR	1W9I	0.91	Sc=6.10431, min distance = 0
PPCK_ECOLI F	444564 AD	MYS2_DICDI Fu	6022 Aden	1VOM	1K3C	1W9I	0.91	Sc=6.44264, min distance = 2.23684
PPCK_ECOLI F	444852 CI	ATPB_BOVIN Fu	5957 Aden	2V7Q	2OLR	1COW	0.91	Sc=6.53845, min distance = 2.13731
PPCK_ECOLI F	445940 O6	CDK2_HUMAN Fu	6022 Aden	1GY3	1K3C	1GZ8		Sc=6.16452, min distance = 2.61558
PPCK_ECOLI F	445966 O6	CDK2_HUMAN Fu	6022 Aden	1GY3	1K3C	1H0V		Sc=5.83032, min distance = 2.35802
PPCK_ECOLI F	447916 ac	RIO1_ARCFU Fu	5957 Aden	1ZP9	2OLR	1ZTF	0.94	Sc=6.27543, min distance = 2.50475
PPCK_ECOLI F	4565 1h1r	CDK2_HUMAN Fu	6022 Aden	1GY3	1K3C	1H1R		Sc=6.14004, min distance = 2.12083
PPCK_ECOLI F	6031 Uric	DCK_HUMAN Ful	6022 Aden	1P5Z	1K3C	2ZIA		Sc=5.90111, min distance = 2.34914
PPCK_ECOLI F	6031 Uric	ECX1_PYRAB Fu	6022 Aden	2PO0	1K3C	2PNZ		Sc=5.92039, min distance = 2.31041
PPCK_ECOLI F	6083 aden	Y059_METJA Fu	5957 Aden	2J9C	2OLR	2J9D	0.98	Sc=6.3787, min distance = 2.151924
PPCK_ECOLI F	60961 ade	IPKA_RABIT Fu	6022 Aden	1JBP	1K3C	1FMO	0.95	Sc=5.87576, min distance = 2.48686
PPCK_ECOLI F	6102784 A	PSPF_ECOLI Fu	6022 Aden	2C98	1K3C	2C99	0.99	Sc=6.50524, min distance = 2.17335
PPCK_ECOLI F	6132 Cyti	ECX1_PYRAB Fu	6022 Aden	2PO0	1K3C	2PO2		Sc=6.36194, min distance = 2.44942
PPCK_ECOLI F	6338561 C	MYS2_DICDI Fu	5957 Aden	1FMW	2OLR	1D0X		Sc=6.24138, min distance = 0
PPCK_ECOLI F	6338565 D	MYS2_DICDI Fu	5957 Aden	1FMW	2OLR	1D1B		Sc=5.8836, min distance = 0
PPCK_ECOLI F	91532 AME	AROK_MYCTU Fu	6022 Aden	2IYV	1K3C	1ZYU	0.99	Sc=6.52946, min distance = 2.42781
PPCK_ECOLI F	91532 AME	KIF1A_MOUSE F	6022 Aden	1I5S	1K3C	1I6I	0.99	Sc=6.52736, min distance = 2.14434
PPCK_ECOLI F	91532 AME	NIFH1_AZOVI F	6022 Aden	1FP6	1K3C	2AFK	0.99	Sc=6.54206, min distance = 2.29364
PPCK_THET8 F	24871491	INSR_HUMAN Fu	5957 Aden	3BU5	1XKV	2Z8C		Sc=5.8644, min distance = 2.163937
PPCK_THET8 F	4369183 C	NADE_BACSU Fu	5957 Aden	1NSY	1XKV	1IFX		Sc=6.27651, min distance = 2.11978
PPCK_THET8 F	6022 Aden	MUTS_ECOLI Fu	5957 Aden	1W7A	1XKV	1OH7	0.99	Sc=5.99637, min distance = 2.36387
PPCK_THET8 F	6022 Aden	PDXK_SHEEP Fu	5957 Aden	1LHR	1XKV	1RFU	0.99	Sc=5.9203, min distance = 1.956652
PPK_ECOLI Fu	11608401	KAPCA_BOVIN F	5957 Aden	1Q24	1XDP	2UVY		Sc=6.06197, min distance = 2.11089
PPK_ECOLI Fu	6022 Aden	MALK_ECOLI Fu	5957 Aden	1Q12	1XDP	2AWN	0.99	Sc=5.89093, min distance = 1.26340
PPK_ECOLI Fu	6022 Aden	RIO1_ARCFU Fu	5957 Aden	1ZP9	1XDP	1ZTH	0.99	Sc=5.87489, min distance = 1.43282
PPNK1_LISMO	447532 1c	SRC_HUMAN Ful	311 citri	1O4L	2I29	1O4H		Sc=5.93544, min distance = 2.34829
PPNK1_LISMO	448573 FI	CASP7_HUMAN F	311 citri	2QL9	2I29	1SHL		Sc=6.01778, min distance = 1.42810
PPNK_ARCFU F	11814433	PPNK1_LISMO F	5893 nadi	2I2F	1Z0Z	2I2D		Sc=6.26298, min distance = 2.21376
PPNK_ARCFU F	16741199	PPNK1_LISMO F	5886 NADF	2I2A	1Z0U	2I2C		Sc=6.22812, min distance = 2.01011
PPNK_ARCFU F	16741199	PPNK1_LISMO F	5893 nadi	2I2F	1Z0Z	2I2C		Sc=6.22149, min distance = 2.50327
PPNK_ARCFU F	6022 Aden	Y059_METJA Fu	5957 Aden	2J9C	1Z0S	2J9D	0.99	Sc=5.87831, min distance = 1.91408
PPNK_ARCFU F	6083 aden	LPLA_THEAC Fu	5957 Aden	2ARU	1Z0S	2ART	0.98	Sc=5.86917, min distance = 2.23478

PPNK_MYCTU Fı	16741199	PPNK1_LISMO Fı	5893 nadi	2I2F	1Y3I	2I2C			Sc=6.67739, min distance = 1.72031(
PPNK_MYCTU Fı	5957 Ader	PPNK_ARCFU Fu	5893 nadi	1Z0Z	1Y3I	1Z0S			Sc=5.60057, min distance = 2.53201!
PPOM_TOBAC Fı	11987634	TYTR_TRYCR Fu	444188 CI	1BZL	1SEZ	1AOG		0.97	Sc=6.42394, min distance = 2.22587:
PPOM_TOBAC Fı	16740985	FRDA_SHEFR Fu	444188 CI	1M64	1SEZ	1E39		0.93	Sc=6.41514, min distance = 2.13637(
PPOM_TOBAC Fı	16750117	LSD1_HUMAN Fu	444188 CI	2DW4	1SEZ	2UXX		0.88	Sc=6.48554, min distance = 2.41423:
PPOM_TOBAC Fı	23631921	LSD1_HUMAN Fu	444188 CI	2DW4	1SEZ	2Z3Y		0.91	Sc=6.48614, min distance = 2.78630!
PPOM_TOBAC Fı	24757937	LSD1_HUMAN Fu	444188 CI	2DW4	1SEZ	2Z5U			Sc=6.48282, min distance = 2.55187!
PPOM_TOBAC Fı	444502 CI	FRDA_SHEFR Fu	444188 CI	1M64	1SEZ	1QJD		0.98	Sc=6.41616, min distance = 2.03371!
PPOM_TOBAC Fı	444502 CI	GSHR_HUMAN Fu	444188 CI	3DK9	1SEZ	1BWC		0.98	Sc=6.41217, min distance = 2.29230(
PPOM_TOBAC Fı	446013 1,	O28603_ARCFU I	444188 CI	1JNR	1SEZ	1JNZ		0.94	Sc=6.42332, min distance = 2.14821(
PPOM_TOBAC Fı	449465 CI	GSHR_HUMAN Fu	444188 CI	3DK9	1SEZ	3GRT		0.98	Sc=6.41913, min distance = 2.02188:
PPOM_TOBAC Fı	6420174 C	O28603_ARCFU I	444188 CI	1JNR	1SEZ	2FJB		0.92	Sc=6.42938, min distance = 2.07647:
PPOM_TOBAC Fı	6420174 C	O28604_ARCFU I	444188 CI	1JNR	1SEZ	2FJB		0.92	Sc=6.42774, min distance = 2.13186'
PPOX_MYXXA Fı	16740985	FRDA_SHEFR Fu	444188 CI	1M64	2IVD	1E39		0.93	Sc=6.1283, min distance = 2.334872:
PPOX_MYXXA Fı	16741253	LSD1_HUMAN Fu	444188 CI	2DW4	2IVD	2UXN		0.95	Sc=6.85374, min distance = 2.20230:
PPOX_MYXXA Fı	444502 CI	FRDA_SHEFR Fu	444188 CI	1M64	2IVD	1QJD		0.98	Sc=6.1289, min distance = 2.294451'
PPT1_BOVIN Fı	185698 al	Q9HYN5_PSEAE I	24139 ace	1W8F	1EH5	1OVS			Sc=5.7024, min distance = 0.870554
PPT2_HUMAN Fı	60961 ade	TRFL_BOVIN Fu	444205 CI	2G93	1PJA	2DOJ			Sc=5.84681, min distance = 2.30990:
PPTA_ECOLI Fı	164795 N(	RBCMT_PEA Ful	23831 HEF	1MLV	1GYX	2H2J			Sc=5.78318, min distance = 2.03968!
PPTA_ECOLI Fı	854023 Ep	Q8WSF8_APLCA I	23831 HEF	2BR7	1GYX	2BYQ			Sc=5.81305, min distance = 0.81539:
PQQC_KLEPN Fı	11987682	DHM1_METME Fu	5326460 F	2AD8	1OTW	2AD6		0.83	Sc=6.52045, min distance = 2.32077
PRDC1_HUMAN I	8582 Inos	HPRT_ECOLI Fu	6804 guan	1G9T	2JBH	1G9S		0.99	Sc=6.34357, min distance = 2.23542:
PRGR_HUMAN Fı	10472685	MCR_HUMAN Ful	5994 prog	2AA6	1A28	2A3I	56.85	0.77	Sc=6.33288, min distance = 2.74328:
PRGR_HUMAN Fı	11522441	ANDR_HUMAN Fu	261000 Me	1XOW	1E3K	2PIP	55.69		Sc=5.90086, min distance = 2.10710(
PRGR_HUMAN Fı	11560224	ANDR_HUMAN Fu	261000 Me	1XOW	1E3K	2HVC	55.69		Sc=6.04164, min distance = 1.66452!
PRGR_HUMAN Fı	11987686	P71278_ENTCL I	5994 prog	2ABA	1A28	1H61			Sc=6.4203, min distance = 2.301273:
PRGR_HUMAN Fı	11987810	MCR_HUMAN Ful	5994 prog	2AA6	1A28	2AA2	56.85		Sc=6.3457, min distance = 2.828064!
PRGR_HUMAN Fı	11987811	MCR_HUMAN Ful	5994 prog	2AA6	1A28	2ABI	56.85	0.83	Sc=6.23745, min distance = 2.65553
PRGR_HUMAN Fı	13472 Bol	P71278_ENTCL I	5994 prog	2ABA	1A28	1H62		0.96	Sc=6.01925, min distance = 2.54280:
PRGR_HUMAN Fı	445033 CI	P71278_ENTCL I	5994 prog	2ABA	1A28	1H60		0.8	Sc=6.08159, min distance = 2.73187(
PRGR_HUMAN Fı	6166 Desc	MCR_HUMAN Ful	5994 prog	2AA6	1A28	1Y9R	56.85	0.85	Sc=6.23745, min distance = 2.45726'
PRIM_BPT7 Fu	5789 thym	RMLA1_ECOLI Fı	164628 de	1H5T	1CR4	1H5R		0.9	Sc=6.03853, min distance = 2.02464:
PRIM_BPT7 Fu	6338561 C	MYS2_DICDI Fu	33113 gam	1MMN	1E0J	1D0X			Sc=6.18677, min distance = 2.30894(
PRIM_BPT7 Fu	65103 dGT	RIR1_YEAST Fu	33113 gam	2EUD	1E0J	2CVX		0.78	Sc=5.86446, min distance = 1.82472:
PRIM_BPT7 Fu	656930 DI	DPO42_SULSO Fı	15993 dAT	2AGQ	1CR2	1S9F			Sc=5.76911, min distance = 2.41958!
PRIM_BPT7 Fu	766948 2c	MK10_HUMAN Fu	33113 gam	1PMQ	1E0J	2O2U			Sc=5.98909, min distance = 1.19515
PRMA_THET8 Fı	12771338	MCES_HUMAN Fu	439155 Ad	3BGV	3EGV	3EPP		0.88	Sc=6.52701, min distance = 2.53777:

PRMA_THET8 Fu	16741210	MTTA_THEAQ Ful	34756 Acy	2ADM	2NXE	2JG3			Sc=6.38658, min distance = 2.44788(
PRMA_THET8 Fu	439176 Me	MTR1_RHOSH Ful	34756 Acy	1NW5	2NXE	1EG2		0.91	Sc=6.23182, min distance = 2.16409(
PRMA_THET8 Fu	445971 CI	COMT_RAT Full=	34756 Acy	2CL5	2NXE	1H1D		0.92	Sc=6.52093, min distance = 2.52089(
PRMA_THET8 Fu	446535 CI	HNMT_HUMAN Ful	439155 Ad	2AOT	3EGV	1JQE		0.95	Sc=6.46759, min distance = 2.34970(
PRMA_THET8 Fu	446768 CI	CFA1_MYCTU Ful	439155 Ad	1KPG	3EGV	1KPH		0.91	Sc=6.48709, min distance = 2.26347(
PRMA_THET8 Fu	60961 ade	PIMT_PYRFU Ful	439155 Ad	1JG1	3EGV	1JG2		0.84	Sc=6.175, min distance = 2.48938480
PROB_CAMJE Fu	13373715	HS90A_HUMAN Fu	6022 Aden	1BYQ	2AKO	2QF6			Sc=6.13394, min distance = 2.03247(
PROB_CAMJE Fu	3044933 A	ENPL_CANFA Ful	6022 Aden	1TC6	2AKO	1U0Y		0.81	Sc=6.36274, min distance = 2.28324(
PROB_CAMJE Fu	444842 CI	CDK2_HUMAN Ful	6022 Aden	1GY3	2AKO	1CKP			Sc=5.63079, min distance = 2.09898(
PROB_CAMJE Fu	447955 1p	CDK2_HUMAN Ful	6022 Aden	1GY3	2AKO	1PXI			Sc=5.71804, min distance = 2.23737(
PROB_CAMJE Fu	4565 1h1r	CDK2_HUMAN Ful	6022 Aden	1GY3	2AKO	1H1R			Sc=6.11845, min distance = 2.12887(
PROB_CAMJE Fu	6083 aden	PURP_METJA Ful	6022 Aden	2R7N	2AKO	2R7M		0.99	Sc=6.35869, min distance = 2.08168(
PROB_CAMJE Fu	60961 ade	IPKA_RABIT Ful	6022 Aden	1JBP	2AKO	1FMO		0.95	Sc=6.2262, min distance = 2.3410775
PRPB_ECOLI Fu	444212 tr	ACON_BOVIN Ful	5318532 i	1C97	1XG4	1ACO			Sc=5.83952, min distance = 2.32719(
PRPB_ECOLI Fu	444591 CI	IDH_ECOLI Full	5318532 i	1P8F	1XG4	1BL5			Sc=5.62017, min distance = 2.33722(
PRPB_ECOLI Fu	445607 AT	ACON_BOVIN Ful	5318532 i	1C97	1XG4	1FGH			Sc=5.91718, min distance = 2.17358(
PRPB_ECOLI Fu	447376 2-	ACON_BOVIN Ful	5318532 i	1C97	1XG4	1NIS			Sc=5.82788, min distance = 2.19252(
PRPB_ECOLI Fu	449604 NI	ACON_BOVIN Ful	5318532 i	1C97	1XG4	8ACN			Sc=5.82002, min distance = 2.41863(
PRTK_TRIAL Fu	180559 at	PA28_DABRR Ful	323 couma	2PMJ	2PWB	2OTF			Sc=6.11441, min distance = 2.11126(
PRTK_TRIAL Fu	75846 Alc	CP2A6_HUMAN Fu	323 couma	1Z10	2PWB	2FDY			Sc=5.67401, min distance = 2.34515(
PRTK_TRIAL Fu	802 indol	PA28_DABRR Ful	323 couma	2PMJ	2PWB	2OYF			Sc=5.71627, min distance = 2.17911(
PRTN3_HUMAN I	446578 1r	MBL1_RAT Full=	439554 fu	3KMB	1FUJ	1KWW		0.96	Sc=5.66913, min distance = 2.51313(
PRTN3_HUMAN I	94214 Met	MBL1_RAT Full=	439554 fu	3KMB	1FUJ	1AFA		0.96	Sc=5.76236, min distance = 2.01000(
PRXC_CALFU Fu	11957330	CCPR_YEAST Ful	444098 HE	2EUT	2CIW	1BEQ		0.81	Sc=6.71483, min distance = 2.17445(
PRXC_CALFU Fu	11957330	CCPR_YEAST Ful	444207 He	2CCP	1CPO	1BEQ		0.84	Sc=6.70339, min distance = 2.23935(
PRXC_CALFU Fu	11957353	HBA_HORSE Full	444124 HE	1Y8I	2CPO	1IWH		0.87	Sc=6.75636, min distance = 2.02147(
PRXC_CALFU Fu	11957364	HMOX1_HUMAN Fu	444124 HE	1OZW	2CPO	1S13		0.86	Sc=6.65217, min distance = 1.75764(
PRXC_CALFU Fu	11957370	HMOX1_HUMAN Fu	444124 HE	1OZW	2CPO	1TWN			Sc=6.57774, min distance = 2.25166(
PRXC_CALFU Fu	11957371	HMOX1_HUMAN Fu	444124 HE	1OZW	2CPO	1TWR			Sc=6.59283, min distance = 2.39667(
PRXC_CALFU Fu	11957373	CATE_ECOLI Ful	444124 HE	1IPH	2CPO	1P81		0.79	Sc=6.76846, min distance = 1.88127(
PRXC_CALFU Fu	11957385	MYG_PHYCA Full	444098 HE	1A6M	2CIW	2CMM			Sc=6.21296, min distance = 2.70847(
PRXC_CALFU Fu	11957385	MYG_PHYCA Full	444124 HE	1U7R	2CPO	2CMM			Sc=6.19118, min distance = 2.69351(
PRXC_CALFU Fu	11957385	MYG_PHYCA Full	444207 He	1VXG	1CPO	2CMM			Sc=6.19863, min distance = 2.58950(
PRXC_CALFU Fu	11970219	CCPR_YEAST Ful	444098 HE	2EUT	2CIW	1BEM		0.83	Sc=6.71909, min distance = 2.23091(
PRXC_CALFU Fu	11970220	CCPR_YEAST Ful	444098 HE	2EUT	2CIW	1BEP		0.8	Sc=6.74997, min distance = 2.07676(
PRXC_CALFU Fu	11970220	CCPR_YEAST Ful	444207 He	2CCP	1CPO	1BEP		0.83	Sc=6.73904, min distance = 2.15599(
PRXC_CALFU Fu	11970221	CCPR_YEAST Ful	444098 HE	2EUT	2CIW	1BES		0.83	Sc=6.66412, min distance = 2.02238(

PRXC_CALFU F	11970221	CCPR_YEAST Fu	444207 He	2CCP	1CPO	1BES		0.87	Sc=6.64778, min distance = 2.10131
PRXC_CALFU F	11970242	CCPR_YEAST Fu	444098 HE	2EUT	2CIW	1CPE		0.83	Sc=6.71874, min distance = 2.16788
PRXC_CALFU F	11970242	CCPR_YEAST Fu	444207 He	2CCP	1CPO	1CPE		0.87	Sc=6.70231, min distance = 2.23975
PRXC_CALFU F	16214774	CP51_MYCTU Fu	444124 HE	2CIB	2CPO	2CI0		0.95	Sc=6.54925, min distance = 2.52975
PRXC_CALFU F	16741183	CPXA_PSEPU Fu	444098 HE	1YRC	2CIW	2ZAW		0.97	Sc=6.54705, min distance = 2.23299
PRXC_CALFU F	16741183	CPXA_PSEPU Fu	444207 He	1PHC	1CPO	2ZAW		0.93	Sc=6.52858, min distance = 2.34945
PRXC_CALFU F	444522 HE	CCPR_YEAST Fu	444207 He	2CCP	1CPO	3E2O	1		Sc=6.70634, min distance = 2.05790
PRXC_CALFU F	444522 HE	CP2C9_HUMAN F	444124 HE	1OG2	2CPO	1R9O		0.99	Sc=6.71026, min distance = 2.09199
PRXC_CALFU F	444522 HE	HBA_HORSE Ful	444124 HE	1Y8I	2CPO	1Y8K		0.99	Sc=6.76289, min distance = 2.26848
PRXC_CALFU F	444522 HE	HBA_HORSE Ful	444207 He	2ZLW	1CPO	1Y8K	1		Sc=6.76109, min distance = 2.38231
PRXC_CALFU F	444522 HE	HBA_PAGBE Ful	444124 HE	1S5X	2CPO	1PBX		0.99	Sc=6.76035, min distance = 2.23077
PRXC_CALFU F	444522 HE	HBB_HORSE Ful	444207 He	2ZLW	1CPO	1Y8K	1		Sc=6.76289, min distance = 2.03156
PRXC_CALFU F	444522 HE	PER_COPCI Ful	444207 He	1LY8	1CPO	1H3J	1		Sc=6.70713, min distance = 1.92884
PRXC_CALFU F	448845 CI	CCPR_YEAST Fu	444207 He	2CCP	1CPO	2B11		0.99	Sc=6.75477, min distance = 2.01572
PRXC_CALFU F	6420167 M	CPXA_PSEPU Fu	444207 He	1PHC	1CPO	2FE6		0.9	Sc=6.76456, min distance = 2.16400
PSD10_MOUSE	445939 1g	PPTA_ECOLI Fu	23831 HEP	1GYX	2DWZ	1GYY			Sc=5.74123, min distance = 2.42751
PSD7_HUMAN F	439353 be	O96048_LUMTE	8172 Trig	2DRY	2O95	2DS0			Sc=5.68216, min distance = 0.64654
PSPF_ECOLI F	11608401	KAPCA_BOVIN F	5957 Aden	1Q24	2C96	2UVY			Sc=6.13021, min distance = 2.46300
PSPF_ECOLI F	11957393	FAK1_HUMAN Fu	5957 Aden	2IJM	2C96	2ETM			Sc=6.23298, min distance = 2.11725
PSPF_ECOLI F	1540 1h1q	CDK2_HUMAN Fu	6022 Aden	1GY3	2C98	1H1Q			Sc=6.1259, min distance = 2.392282
PSPF_ECOLI F	1707 1fvt	CDK2_HUMAN Fu	6022 Aden	1GY3	2C98	1FVT			Sc=5.94712, min distance = 1.79921
PSPF_ECOLI F	24864080	CDK2_HUMAN Fu	6022 Aden	1GY3	2C98	2VTR			Sc=5.83182, min distance = 2.65592
PSPF_ECOLI F	24864081	CDK2_HUMAN Fu	6022 Aden	1GY3	2C98	2VTS			Sc=6.13713, min distance = 2.61888
PSPF_ECOLI F	2608 1jsv	CDK2_HUMAN Fu	6022 Aden	1GY3	2C98	1JSV			Sc=5.83407, min distance = 1.48666
PSPF_ECOLI F	3547 Fasu	KAPCA_BOVIN F	5957 Aden	1Q24	2C96	1Q8W			Sc=5.91836, min distance = 2.05263
PSPF_ECOLI F	444852 CI	ATPA1_BOVIN F	5957 Aden	2V7Q	2C96	1COW		0.91	Sc=6.04974, min distance = 2.02528
PSPF_ECOLI F	444852 CI	ATPB_BOVIN Fu	5957 Aden	2V7Q	2C96	1COW		0.91	Sc=5.75195, min distance = 2.09506
PSPF_ECOLI F	445940 O6	CDK2_HUMAN Fu	6022 Aden	1GY3	2C98	1GZ8			Sc=5.96309, min distance = 2.51705
PSPF_ECOLI F	447649 1c	CDK2_HUMAN Fu	6022 Aden	1GY3	2C98	1OI9			Sc=6.23523, min distance = 2.29646
PSPF_ECOLI F	447654 CI	CDK2_HUMAN Fu	6022 Aden	1GY3	2C98	1OIT			Sc=6.10119, min distance = 2.45708
PSPF_ECOLI F	447821 CI	CDK2_HUMAN Fu	6022 Aden	1GY3	2C98	1PF8			Sc=6.07576, min distance = 2.39247
PSPF_ECOLI F	448055 To	PDE4D_HUMAN F	6083 aden	1TB7	2VII	1Q9M			Sc=5.97219, min distance = 1.90652
PSPF_ECOLI F	4566 1h1s	CDK2_HUMAN Fu	6022 Aden	1GY3	2C98	1H1S			Sc=6.18239, min distance = 2.13758
PSPF_ECOLI F	5281680 Q	PIM1_HUMAN Fu	6083 aden	1YXU	2VII	2O64			Sc=6.42382, min distance = 2.14604
PSPF_ECOLI F	5327097 2	CDK2_HUMAN Fu	6022 Aden	1GY3	2C98	2BTS			Sc=5.87269, min distance = 2.38681
PSPF_ECOLI F	6338566 C	MYS2_DICDI Fu	5957 Aden	1FMW	2C96	1D1C			Sc=6.23474, min distance = 0
PSPF_ECOLI F	656966 1y	PDE4D_HUMAN F	6083 aden	1TB7	2VII	1Y2E			Sc=5.94592, min distance = 2.10519

PSPF_ECOLI Fu	656969 1y	PDE4B_HUMAN Fu	6083 aden	1ROR	2VII	1Y2J			Sc=6.18811, min distance = 2.08406
PSPF_ECOLI Fu	656971 CI	CDK2_HUMAN Ful	6022 Aden	1GY3	2C98	1Y8Y			Sc=5.92292, min distance = 2.29669
PSPF_ECOLI Fu	657135 Op	PIM1_HUMAN Ful	6083 aden	1YXU	2VII	1YXV			Sc=5.84525, min distance = 2.29009
PSPF_ECOLI Fu	6852187 2	O57883_PYRHO		6022 Aden	1WNL	2C98	2DTH		0.89	Sc=6.03908, min distance = 2.09984
PSPF_ECOLI Fu	9578243 F	PDE4B_HUMAN Fu	6083 aden	1ROR	2VII	1XLZ			Sc=6.06433, min distance = 2.23808
PTEN_HUMAN Fu	439710 rh	PGRP_CAMDR Ful	444305 L-	3C2X	1D5R	3CG9			Sc=5.77455, min distance = 2.40179
PTGD2_HUMAN		115260 S-	GSTM2_HUMAN Fu	124886 gl	1XW5	2CVD	2AB6	28.43	0.94	Sc=6.33582, min distance = 1.59862
PTGD2_HUMAN		444352 GT	GSTM1_RAT Full	124886 gl	6GST	2CVD	4GST	29.44		Sc=5.70765, min distance = 1.88809
PTGD2_HUMAN		449471 GG	GSTT2_HUMAN Fu	124886 gl	1LJR	2CVD	3LJR			Sc=6.63168, min distance = 1.74538
PTGD2_HUMAN		97536 Hex	GSTP_ONCVO Ful	124886 gl	1TU7	2CVD	1TU8		0.9	Sc=6.46492, min distance = 2.49640
PTGIS_HUMAN		11957361	CY1_BOVIN Full	444098 HE	1L0N	3B6H	1NTZ		0.8	Sc=6.41457, min distance = 2.38290
PTGIS_HUMAN		11957385	MYG_PHYCA Full	444098 HE	1A6M	3B6H	2CMM			Sc=6.23786, min distance = 2.57724
PTGIS_HUMAN		16741183	CPXA_PSEPU Ful	444098 HE	1YRC	3B6H	2ZAW		0.97	Sc=6.5861, min distance = 2.513721
PTGIS_HUMAN		5326592 C	HMOX1_RAT Full	444098 HE	1DVG	3B6H	1J2C		0.79	Sc=6.65772, min distance = 1.90230
PTGR1_CAVPO		440334 Ni	DAPB_ECOLI Ful	5886 NADP	1DIH	1V3V	1DRW		0.91	Sc=5.99358, min distance = 2.10117
PTGR2_HUMAN		11970225	ETR1_CANTR Ful	5886 NADP	1GUF	2ZB4	1N9G		0.78	Sc=6.75741, min distance = 2.02907
PTGR2_HUMAN		440334 Ni	DAPB_ECOLI Ful	5886 NADP	1DIH	2ZB4	1DRW		0.91	Sc=5.98512, min distance = 2.38965
PTGR2_MOUSE		11970225	ETR1_CANTR Ful	5886 NADP	1GUF	2ZB3	1N9G		0.78	Sc=6.74894, min distance = 2.26708
PTGR2_MOUSE		440334 Ni	DAPB_ECOLI Ful	5886 NADP	1DIH	2ZB3	1DRW		0.91	Sc=5.97846, min distance = 2.44983
PTPA_HUMAN Fu	23653515	CDK2_HUMAN Ful	6022 Aden	1GY3	2HV7	2R3F			Sc=6.1423, min distance = 1.431031
PTPA_HUMAN Fu	36735 Gpp	PARM_ECOLX Ful	6022 Aden	1MWM	2HV7	2ZGZ		0.79	Sc=5.66832, min distance = 2.33921
PTPA_HUMAN Fu	445966 O6	CDK2_HUMAN Ful	6022 Aden	1GY3	2HV7	1H0V			Sc=6.21059, min distance = 2.06408
PTPA_HUMAN Fu	447962 1p	CDK2_HUMAN Ful	6022 Aden	1GY3	2HV7	2C5N			Sc=6.29353, min distance = 1.80028
PTPA_HUMAN Fu	448505 AD	NDKC_DICDI Ful	6022 Aden	1KDN	2HV7	1S5Z		0.93	Sc=5.73417, min distance = 2.13082
PTPA_HUMAN Fu	5289411 1	CDK2_HUMAN Ful	6022 Aden	1GY3	2HV7	1OGU			Sc=6.10302, min distance = 2.03833
PTPA_HUMAN Fu	5957 Aden	HSP7F_YEAST Fu	6022 Aden	3C7N	2HV7	3D2F		0.99	Sc=5.64795, min distance = 2.01091
PTR1_LEIMA Fu	15942680	6PGD_LACLM Ful	5886 NADP	2IZ0	1E7W	2IZ1			Sc=6.22534, min distance = 2.37901
PTR1_LEIMA Fu	165230 1a	FABG_ECOLI Ful	5886 NADP	1Q7B	1E7W	1Q7C			Sc=5.78497, min distance = 2.57639
PTR1_LEIMA Fu	2429 BROM	DYR_MYCTU Full	126941 me	1DF7	1E7W	1DG7			Sc=6.08853, min distance = 2.15356
PTR1_LEIMA Fu	440141 9i	DHB1_HUMAN Ful	5886 NADP	1QYV	1E7W	1QYW			Sc=6.38384, min distance = 2.57365
PTR1_LEIMA Fu	440141 9i	G6PD_LEUME Ful	5886 NADP	1H9A	1E7W	1E7Y			Sc=6.40273, min distance = 2.43955
PTR1_LEIMA Fu	445109 2,	NOS3_BOVIN Ful	444951 CI	1Q2O	2BFP	1DMK			Sc=6.10486, min distance = 2.02982
PTR1_LEIMA Fu	446753 1k	DYR_HUMAN Full	126941 me	1U72	1E7W	1KMV			Sc=6.23651, min distance = 0.85252
PTR1_LEIMA Fu	447022 CI	DYR_PNECA Full	126941 me	3CD2	1E7W	1LY4			Sc=6.19256, min distance = 2.03085
PTR1_LEIMA Fu	447229 AI	DYR_HUMAN Full	126941 me	1U72	1E7W	1MVT			Sc=6.07676, min distance = 2.02979
PTR1_LEIMA Fu	6022 Aden	HMDH_HUMAN Ful	5886 NADP	1DQA	1E7W	1HW8			Sc=5.98759, min distance = 2.15552
PTR1_LEITA Fu	15942680	6PGD_LACLM Ful	5886 NADP	2IZ0	1P33	2IZ1			Sc=6.24342, min distance = 2.47164

PTR1_LEITA	Fı	165230 1a	G6PD_LEUME Fu	5886 NADF	1H9A	1P33	2DPG			Sc=6.43274, min distance = 2.23289(
PTR1_LEITA	Fı	440141 9i	DHB1_HUMAN Fu	5886 NADF	1QYV	1P33	1QYW			Sc=6.40508, min distance = 2.40275
PULA_KLEPN	Fı	193758 Va	AMY1_HUMAN Fu	79025 alp	3DHP	2FHF	1MFU			Sc=5.98285, min distance = 0.79829(
PULA_KLEPN	Fı	193758 Va	CDGT2_BACCI Fı	79025 alp	1D3C	2FHF	1DTU			Sc=6.00004, min distance = 1.15929
PULA_KLEPN	Fı	23831 HEI	CDGT2_BACCI Fı	79025 alp	1D3C	2FHF	1OT1			Sc=5.75044, min distance = 1.07353!
PULA_KLEPN	Fı	29435 1-I	CDGT_BACS0 Fu	79025 alp	1UKS	2FHF	1I75			Sc=5.87327, min distance = 0.75966!
PULA_KLEPN	Fı	444915 Gl	CDGT2_BACCI Fı	79025 alp	1D3C	2FHF	1CXL		0.85	Sc=5.80993, min distance = 0.84829!
PULA_KLEPN	Fı	447607 1c	PYGM_RABIT Fu	79025 alp	1H5U	2FHF	2G9V			Sc=5.70694, min distance = 2.37355
PULA_KLEPN	Fı	448009 TI	AMY1_HUMAN Fu	79025 alp	3DHP	2FHF	1Q4N			Sc=5.62672, min distance = 1.50304
PULA_KLEPN	Fı	64960 Pol	XYLA_STRRU Fu	79025 alp	1XIF	2FHF	1XIE		0.89	Sc=5.73005, min distance = 2.19733
PULA_THEMA	Fı	444139 CI	CDGT2_BACCI Fı	79025 alp	1D3C	2J73	1DTU			Sc=5.79943, min distance = 0.96227
PULA_THEMA	Fı	444809 Th	AMY1_HORVU Fu	79025 alp	1RP8	2J73	1P6W		0.78	Sc=5.92023, min distance = 0.72834
PULA_THEMA	Fı	444915 Gl	CDGT2_BACCI Fı	79025 alp	1D3C	2J73	1CXL		0.85	Sc=5.6204, min distance = 1.0198362
PULA_THEMA	Fı	448009 TI	AMY1_HUMAN Fu	79025 alp	3DHP	2J73	1Q4N			Sc=5.7665, min distance = 1.420921
PULA_THEMA	Fı	449571 CI	CDGT1_BACCI Fı	79025 alp	1CGU	2J73	6CGT			Sc=5.83964, min distance = 0.911008
PUNA_MYCTU	Fı	2022 acyc	PNPH_HUMAN Fu	447268 DA	1RSZ	1N3I	1PWY	39.31		Sc=6.10216, min distance = 2.43662
PUNA_MYCTU	Fı	444498 im	PNPH_BOVIN Fu	444499 Fc	1B8O	1G2O	1B8N	41.28	0.98	Sc=6.40753, min distance = 2.48864
PUNA_MYCTU	Fı	448999 1v	PNPH_BOVIN Fu	444499 Fc	1B8O	1G2O	1V48	41.28		Sc=6.41124, min distance = 2.07628(
PUNA_MYCTU	Fı	5327012 C	PNPH_BOVIN Fu	444499 Fc	1B8O	1G2O	2AI1	41.28		Sc=6.51092, min distance = 2.22459
PUNA_MYCTU	Fı	5327013 C	PNPH_BOVIN Fu	444499 Fc	1B8O	1G2O	2AI2	41.28	0.81	Sc=6.50021, min distance = 2.07810
PUNA_MYCTU	Fı	5327014 I	PNPH_BOVIN Fu	444499 Fc	1B8O	1G2O	2AI3	41.28		Sc=6.46859, min distance = 2.17833
PUNA_MYCTU	Fı	764 guani	PNPH_HUMAN Fu	444499 Fc	1RT9	1G2O	1V2H	39.31		Sc=5.83999, min distance = 2.04987
PUNA_MYCTU	Fı	8646 Guar	PNPH_HUMAN Fu	444499 Fc	1RT9	1G2O	1V41	39.31		Sc=5.81927, min distance = 2.14846
PUNA_MYCTU	Fı	8646 Guar	PNPH_HUMAN Fu	447268 DA	1RSZ	1N3I	1V41	39.31		Sc=5.79816, min distance = 2.49056(
PUR1_BACSU	Fı	164628 de	PMS2_HUMAN Fu	6022 Aden	1EA6	1AO0	1H7U			Sc=5.95812, min distance = 1.83802
PUR1_BACSU	Fı	24832037	PGK1_HUMAN Fu	6022 Aden	2ZGV	1AO0	3C3C			Sc=5.79462, min distance = 2.54336
PUR1_BACSU	Fı	444564 AI	BIOD_ECOLI Fu	6022 Aden	1DAD	1AO0	1BS1		0.91	Sc=6.34281, min distance = 2.26816
PUR1_BACSU	Fı	445966 O6	CDK2_HUMAN Fu	6022 Aden	1GY3	1AO0	1H0V			Sc=6.27415, min distance = 1.77499!
PUR1_BACSU	Fı	5957 Ader	BIOD_ECOLI Fu	6022 Aden	1DAD	1AO0	1A82		0.99	Sc=5.93143, min distance = 2.22406
PUR1_BACSU	Fı	5957 Ader	MALK_ECOLI Fu	6022 Aden	2AWN	1AO0	1Q12		0.99	Sc=5.96313, min distance = 1.67165
PUR1_BACSU	Fı	5957 Ader	RK_BOVIN Full-	6022 Aden	3C4Z	1AO0	3C4W		0.99	Sc=5.61812, min distance = 2.39285
PUR1_BACSU	Fı	8977 1dar	PARM_ECOLX Fu	6022 Aden	1MWM	1AO0	2ZGY		0.8	Sc=6.33233, min distance = 2.43382
PUR1_BACSU	Fı	91532 AMI	BIOD_ECOLI Fu	6022 Aden	1DAD	1AO0	1DAG		0.99	Sc=5.66731, min distance = 2.13280!
PUR1_BACSU	Fı	91532 AMI	MTNK_BACSU Fu	6022 Aden	2OLC	1AO0	2PUL		0.99	Sc=5.67043, min distance = 2.37430
PUR1_BACSU	Fı	91532 AMI	PURP_METJA Fu	6022 Aden	2R7N	1AO0	2R7K		0.99	Sc=5.68914, min distance = 2.17870
PUR1_BACSU	Fı	91532 AMI	RBSK_ECOLI Fu	6022 Aden	1RKD	1AO0	1GQT		0.99	Sc=6.03279, min distance = 1.98580
PUR1_BACSU	Fı	9817550 V	CDK2_HUMAN Fu	6022 Aden	1GY3	1AO0	3BHV			Sc=6.21229, min distance = 1.92769

PUR1_ECOLI F	24316 cyc	PDE10_HUMAN F	6804 guan	2OUQ	1ECB	2OUU		0.97	Sc=6.20286, min distance = 0
PUR1_ECOLI F	5327121 I	PIM1_HUMAN Fu	6083 aden	1YXU	1ECJ	2C3I			Sc=6.26491, min distance = 1.384243
PUR1_ECOLI F	6030 Uric	RNMC_MOMCH Fu	6804 guan	1J1F	1ECB	1UCD			Sc=6.16799, min distance = 2.176918
PUR1_ECOLI F	60961 ade	Q84CU3_THETH I	6083 aden	1V8S	1ECJ	1V8Y		0.96	Sc=6.14611, min distance = 2.209267
PUR1_ECOLI F	657135 Op	PIM1_HUMAN Fu	6083 aden	1YXU	1ECJ	1YXV			Sc=5.78227, min distance = 2.198019
PUR1_ECOLI F	8582 Inos	GMPR2_HUMAN F	6804 guan	2A7R	1ECB	2C6Q		0.99	Sc=6.28955, min distance = 2.144142
PUR1_ECOLI F	8582 Inos	PYGM_RABIT Fu	6083 aden	8GPB	1ECJ	2QN7		0.79	Sc=6.28066, min distance = 2.205835
PUR1_ECOLI F	8582 Inos	RNAS1_BOVIN F	6804 guan	1RNC	1ECB	1Z6D		0.99	Sc=6.31605, min distance = 1.695663
PUR2_AQUAE F	11708454	KAPCA_BOVIN F	5957 Aden	1Q24	2YW2	2VNW			Sc=6.12522, min distance = 2.320874
PUR2_AQUAE F	3540 1yds	KAPCA_BOVIN F	5957 Aden	1Q24	2YW2	1YDS			Sc=5.87585, min distance = 2.191269
PUR2_AQUAE F	3547 Fasu	KAPCA_BOVIN F	5957 Aden	1Q24	2YW2	1Q8W			Sc=5.94983, min distance = 2.344768
PUR2_AQUAE F	444564 AD	BIOD_ECOLI Fu	5957 Aden	1A82	2YW2	1BS1		0.91	Sc=5.93013, min distance = 2.024993
PUR2_AQUAE F	444564 AD	MYS2_DICDI Fu	5957 Aden	1FMW	2YW2	1W9I		0.91	Sc=5.9208, min distance = 0
PUR2_AQUAE F	444852 CI	ATPA1_BOVIN F	5957 Aden	2V7Q	2YW2	1COW		0.91	Sc=6.45055, min distance = 1.433277
PUR2_AQUAE F	447840 CI	PHKG1_RABIT F	5957 Aden	1QL6	2YW2	1PHK			Sc=5.68741, min distance = 1.970379
PUR2_AQUAE F	447916 ac	RIO1_ARCFU Fu	5957 Aden	1ZP9	2YW2	1ZTF		0.94	Sc=6.15065, min distance = 2.439309
PUR2_AQUAE F	448042 2e	KAPCA_BOVIN F	5957 Aden	1Q24	2YW2	1Q8T			Sc=6.00356, min distance = 2.504158
PUR2_AQUAE F	448043 2g	KAPCA_BOVIN F	5957 Aden	1Q24	2YW2	1Q8U			Sc=6.00119, min distance = 1.976258
PUR2_AQUAE F	449240 1y	KAPCA_BOVIN F	5957 Aden	1Q24	2YW2	1YDR			Sc=5.94462, min distance = 2.323839
PUR2_AQUAE F	6022 Aden	ARSA1_ECOLX F	5957 Aden	1II0	2YW2	1IHU		0.99	Sc=5.92906, min distance = 2.161022
PUR2_AQUAE F	6022 Aden	HSP7F_YEAST F	5957 Aden	3D2F	2YW2	3C7N		0.99	Sc=5.89278, min distance = 2.115373
PUR2_AQUAE F	6022 Aden	MUTS_ECOLI Fu	5957 Aden	1W7A	2YW2	1OH7		0.99	Sc=5.93109, min distance = 2.040465
PUR2_AQUAE F	6022 Aden	PURK_ECOLI Fu	5957 Aden	3ETH	2YW2	3ETJ		0.99	Sc=5.93109, min distance = 2.566996
PUR2_AQUAE F	6022 Aden	REX_BACSU Ful	5957 Aden	2VT3	2YW2	2VT2		0.99	Sc=6.39047, min distance = 0.996780
PUR2_AQUAE F	6022 Aden	RK_BOVIN Full=	5957 Aden	3C4W	2YW2	3C4Z		0.99	Sc=5.94478, min distance = 1.871389
PUR2_AQUAE F	6083 aden	Y059_METJA Fu	5957 Aden	2J9C	2YW2	2J9D		0.98	Sc=6.33093, min distance = 1.979026
PUR2_AQUAE F	6852207 M	KAPCA_BOVIN F	5957 Aden	1Q24	2YW2	2GNI			Sc=5.9329, min distance = 2.5137257
PUR2_AQUAE F	91532 AMF	INSR_HUMAN Fu	5957 Aden	3BU5	2YW2	1I44		0.99	Sc=5.99183, min distance = 2.164213
PUR2_AQUAE F	91532 AMF	Q5SJV7_THET8 I	5957 Aden	2Z08	2YW2	2Z09		0.99	Sc=5.99571, min distance = 2.029963
PUR2_HUMAN F	444852 CI	ATPB_BOVIN Fu	5957 Aden	2V7Q	2QK4	1COW		0.91	Sc=5.93252, min distance = 1.618394
PUR2_HUMAN F	6022 Aden	ARSA1_ECOLX F	5957 Aden	1II0	2QK4	1IHU		0.99	Sc=6.28562, min distance = 1.978720
PUR2_HUMAN F	6022 Aden	FAK1_HUMAN Fu	5957 Aden	2IJM	2QK4	1MP8		0.99	Sc=6.24556, min distance = 1.177657
PUR2_HUMAN F	6022 Aden	Q72H90_THET2 I	5957 Aden	2BEK	2QK4	2BEJ		0.99	Sc=6.2676, min distance = 2.0624415
PUR2_HUMAN F	6022 Aden	Y059_METJA Fu	5957 Aden	2J9C	2QK4	2J9D		0.99	Sc=6.29928, min distance = 1.416210
PUR2_HUMAN F	6083 aden	ASSY_THET8 Fu	5957 Aden	1J1Z	2QK4	1J20		0.98	Sc=5.7569, min distance = 2.2082375
PUR3_ECOLI F	439905 Ph	PUR2_HUMAN Fu	160913 Gl	1MEN	1C2T	1ZLY	43.01	0.77	Sc=5.77962, min distance = 2.478848
PUR4_SALTY F	444503 FU	NDKC_DICDI Fu	6022 Aden	1KDN	1T3T	1B99			Sc=6.26983, min distance = 2.229418

PUR4_SALTY F	444564 AD	BIOD_ECOLI Fu	6022 Aden	1DAD	1T3T	1BS1		0.91	Sc=5.96627, min distance = 2.72959
PUR4_SALTY F	446090 1h	NDKC_DICDI Fu	6022 Aden	1KDN	1T3T	1HIY		0.97	Sc=6.41927, min distance = 2.66188
PUR4_SALTY F	447916 ac	RIO1_ARCFU Fu	6022 Aden	1ZTH	1T3T	1ZTF		0.95	Sc=6.25934, min distance = 2.61497
PUR4_SALTY F	448222 NE	ENPL_CANFA Fu	6022 Aden	1TC6	1T3T	1QY5		0.81	Sc=6.40752, min distance = 2.38933
PUR4_SALTY F	5957 Aden	ARSA1_ECOLX F	6022 Aden	1IHU	1T3T	1II0		0.99	Sc=6.47311, min distance = 1.97482
PUR4_SALTY F	5957 Aden	PUR7_METJA Fu	6022 Aden	2YZL	1T3T	2Z02		0.99	Sc=6.01011, min distance = 2.01618
PUR4_SALTY F	5957 Aden	Q72H90_THET2 I	6022 Aden	2BEJ	1T3T	2BEK		0.99	Sc=6.01818, min distance = 2.07780
PUR4_SALTY F	6031 Uric	DCK_HUMAN Ful	6022 Aden	1P5Z	1T3T	2ZIA			Sc=5.86282, min distance = 2.08943
PUR4_SALTY F	6083 ader	NDK_PYRHO Ful	6022 Aden	2DYA	1T3T	2DXD		0.99	Sc=5.98059, min distance = 2.24041
PUR4_SALTY F	6804 guar	NDK_PYRHO Ful	6022 Aden	2DYA	1T3T	2DXF		0.8	Sc=6.38783, min distance = 2.14181
PUR4_SALTY F	72194 2-C	ENPL_CANFA Fu	6022 Aden	1TC6	1T3T	1QYE		0.89	Sc=6.17997, min distance = 2.75201
PUR4_SALTY F	8977 1dar	NDK_PYRHO Ful	6022 Aden	2DYA	1T3T	2DXE		0.8	Sc=6.43482, min distance = 2.33726
PUR4_SALTY F	8977 1dar	NDK_THET8 Ful	6022 Aden	1WKL	1T3T	1WKK		0.8	Sc=6.00956, min distance = 2.08630
PUR4_SALTY F	9543433 T	NDKC_DICDI Fu	6022 Aden	1KDN	1T3T	1F6T			Sc=6.35023, min distance = 1.89397
PUR7_ECOLI F	445940 O6	CDK2_HUMAN Fu	6022 Aden	1GY3	2GQR	1GZ8			Sc=6.02888, min distance = 2.47945
PUR7_ECOLI F	445966 O6	CDK2_HUMAN Fu	6022 Aden	1GY3	2GQR	1H0V			Sc=6.1319, min distance = 2.357251
PUR7_ECOLI F	447916 ac	RIO1_ARCFU Fu	6022 Aden	1ZTH	2GQR	1ZTF		0.95	Sc=6.1319, min distance = 2.262558
PUR7_ECOLI F	448238 CI	GSK3B_HUMAN F	6022 Aden	1J1C	2GQR	1R0E			Sc=6.481, min distance = 1.9988831
PUR7_ECOLI F	448505 AD	NDKC_DICDI Fu	6022 Aden	1KDN	2GQR	1S5Z		0.93	Sc=5.93209, min distance = 1.76363
PUR7_ECOLI F	65059 Dec	RNAS1_BOVIN F	6022 Aden	1O0H	2GQR	2QCA		0.77	Sc=6.17404, min distance = 1.69303
PUR7_ECOLI F	656946 CI	RNAS1_BOVIN F	6022 Aden	1O0H	2GQR	1W4Q			Sc=6.31607, min distance = 0.94892
PUR7_METJA F	11708454	KAPCA_BOVIN F	5957 Aden	1Q24	2Z02	2VNW			Sc=6.11694, min distance = 2.10884
PUR7_METJA F	15602983	KAPCA_BOVIN F	5957 Aden	1Q24	2Z02	2UW5			Sc=6.13359, min distance = 1.96056
PUR7_METJA F	164628 de	PMS2_HUMAN Fu	6022 Aden	1EA6	2YZL	1H7U			Sc=5.67406, min distance = 2.02848
PUR7_METJA F	165388 Ca	PUR7_ECOLI Fu	6022 Aden	2GQR	2YZL	2GQS			Sc=5.73465, min distance = 2.38896
PUR7_METJA F	24836816	HS90A_HUMAN F	6022 Aden	1BYQ	2YZL	3BMY			Sc=6.32316, min distance = 2.18635
PUR7_METJA F	25021197	CDK2_HUMAN Fu	6022 Aden	1GY3	2YZL	3EOC			Sc=6.38294, min distance = 1.37079
PUR7_METJA F	3540 1yds	KAPCA_BOVIN F	5957 Aden	1Q24	2Z02	1YDS			Sc=5.83015, min distance = 2.31255
PUR7_METJA F	398148 1e	CDK2_HUMAN Fu	6022 Aden	1GY3	2YZL	1E1X			Sc=6.19844, min distance = 2.08529
PUR7_METJA F	444564 AD	MYS2_DICDI Fu	5957 Aden	1FMW	2Z02	1W9I		0.91	Sc=5.60274, min distance = 0
PUR7_METJA F	445940 O6	CDK2_HUMAN Fu	6022 Aden	1GY3	2YZL	1GZ8			Sc=6.12246, min distance = 1.58653
PUR7_METJA F	447004 CI	MYS2_DICDI Fu	5957 Aden	1FMW	2Z02	1LVK			Sc=5.96444, min distance = 0
PUR7_METJA F	447955 1p	CDK2_HUMAN Fu	6022 Aden	1GY3	2YZL	1PXI			Sc=5.71163, min distance = 2.14661
PUR7_METJA F	448043 2g	KAPCA_BOVIN F	5957 Aden	1Q24	2Z02	1Q8U			Sc=5.96132, min distance = 2.00941
PUR7_METJA F	449240 1y	KAPCA_BOVIN F	5957 Aden	1Q24	2Z02	1YDR			Sc=5.91095, min distance = 2.31338
PUR7_METJA F	4565 1h1r	CDK2_HUMAN Fu	6022 Aden	1GY3	2YZL	1H1R			Sc=6.34032, min distance = 1.73236
PUR7_METJA F	5288016 6	CDK2_HUMAN Fu	6022 Aden	1GY3	2YZL	2B53			Sc=6.32552, min distance = 1.55500

PUR7_METJA Fu	6031 Uric	DCK_HUMAN Ful	6022 Aden	1P5Z	2YZL	2ZIA			Sc=5.84898, min distance = 2.20378
PUR7_METJA Fu	6082103 2	HS90A_HUMAN Fu	6022 Aden	1BYQ	2YZL	2BT0			Sc=6.34876, min distance = 1.94099
PUR7_METJA Fu	6083 aden	PURP_METJA Fu	5957 Aden	2R7L	2Z02	2R7M		0.98	Sc=5.83918, min distance = 2.07560
PUR7_METJA Fu	6083 aden	PURP_METJA Fu	6022 Aden	2R7N	2YZL	2R7M		0.99	Sc=5.85636, min distance = 2.18640
PUR7_METJA Fu	6176 Cyti	CCA_ARCFU Ful	5957 Aden	1R8B	2Z02	1R89			Sc=5.88221, min distance = 1.27399
PUR7_METJA Fu	6852207 M	KAPCA_BOVIN Fu	5957 Aden	1Q24	2Z02	2GNI			Sc=5.91374, min distance = 2.33970
PUR7_METJA Fu	8977 1dar	NDK_PYRHO Ful	6022 Aden	2DYA	2YZL	2DXE		0.8	Sc=5.96313, min distance = 2.17756
PUR7_METJA Fu	8977 1dar	PARM_ECOLX Fu	6022 Aden	1MWM	2YZL	2ZGY		0.8	Sc=6.42476, min distance = 2.11951
PUR7_METJA Fu	91532 AMP	PURP_METJA Fu	5957 Aden	2R7L	2Z02	2R7K		0.99	Sc=6.00433, min distance = 2.08941
PUR8_ECOLI Fu	6022 Aden	HSP71_HUMAN Fu	6083 aden	1XQS	2PTQ	1S3X		0.99	Sc=6.36524, min distance = 2.12854
PUR8_ECOLI Fu	611002 Op	PIM1_HUMAN Fu	6083 aden	1YXU	2PTQ	1YXX			Sc=6.12521, min distance = 0.76995
PUR8_HUMAN Fu	166760 Fa	PURP_METJA Fu	6083 aden	2R7M	2J91	2R7N		0.76	Sc=5.90339, min distance = 1.89985
PUR8_HUMAN Fu	6804 guar	PDE10_HUMAN Fu	6083 aden	2OUN	2J91	2OUQ		0.8	Sc=5.81712, min distance = 0
PUR9_CHICK Fu	100252 Ri	IMDH_TRIFO Fu	73323 Xan	1MEW	1M9N	1ME8			Sc=6.32844, min distance = 2.19768
PUR9_CHICK Fu	8582 Inos	IMDH_TRIFO Fu	73323 Xan	1MEW	1M9N	1MEH		0.94	Sc=6.45486, min distance = 2.02160
PUR9_HUMAN Fu	100252 Ri	IMDH_TRIFO Fu	73323 Xan	1MEW	1PKX	1ME8			Sc=6.32435, min distance = 2.18993
PUR9_HUMAN Fu	8582 Inos	IMDH_TRIFO Fu	73323 Xan	1MEW	1PKX	1MEH		0.94	Sc=6.44761, min distance = 2.18868
PUR9_THEMA Fu	445562 is	UPAR_HUMAN Fu	8200 TETR	2FD6	1ZCZ	1YWH			Sc=5.61822, min distance = 1.65282
PUR9_THEMA Fu	446506 DE	ACES_TORCA Fu	8200 TETR	1DX6	1ZCZ	1JJB		0.82	Sc=5.68177, min distance = 2.23450
PURA1_MOUSE I	100252 Ri	IMDH_TRIFO Fu	8582 Inos	1MEH	1IWE	1ME8			Sc=6.27516, min distance = 2.01580
PURA1_MOUSE I	188347 pp	ARL5A_HUMAN Fu	8977 1dar	2H17	1LON	1ZJ6		0.99	Sc=6.54081, min distance = 2.17251
PURA1_MOUSE I	444825 1d	PURA_ECOLI Fu	8582 Inos	1CIB	1IWE	1CH8	42.72	0.93	Sc=6.51113, min distance = 2.09138
PURA1_MOUSE I	444845 1d	RASH_HUMAN Fu	6830 guan	2CL7	1LOO	1CLU		0.98	Sc=6.05111, min distance = 2.35854
PURA1_MOUSE I	445708 AM	NADE_ECOLI Fu	6083 aden	1WXI	1MF0	1WXE		0.91	Sc=6.34357, min distance = 2.31767
PURA1_MOUSE I	445708 AM	PURA_ECOLI Fu	444818 6-	1CG0	1LON	1SON	42.72	0.83	Sc=6.36062, min distance = 2.40831
PURA1_MOUSE I	445708 AM	PURA_ECOLI Fu	8582 Inos	1CIB	1IWE	1SON	42.72	0.81	Sc=6.35869, min distance = 2.05357
PURA1_MOUSE I	445708 AM	PURA_ECOLI Fu	9549204 C	2GCQ	2DGN	1SON	42.72	0.83	Sc=6.35869, min distance = 2.71918
PURA1_MOUSE I	446248 CI	RASH_HUMAN Fu	6830 guan	2CL7	1LOO	1IAQ			Sc=5.72632, min distance = 2.34784
PURA1_MOUSE I	447942 MZ	IMDH_TRIFO Fu	8582 Inos	1MEH	1IWE	1PVN			Sc=6.33074, min distance = 1.78787
PURA1_MOUSE I	447979 5G	KGUA_ECOLI Fu	8977 1dar	2AN9	1LON	2ANB			Sc=6.4265, min distance = 2.131546
PURA1_MOUSE I	448089 RE	PURA_ECOLI Fu	444818 6-	1CG0	1LON	1QF5	42.72		Sc=6.1111, min distance = 2.456173
PURA1_MOUSE I	448089 RE	PURA_ECOLI Fu	8582 Inos	1CIB	1IWE	1QF5	42.72		Sc=6.55319, min distance = 2.27640
PURA1_MOUSE I	448089 RE	PURA_ECOLI Fu	9549204 C	2GCQ	2DGN	1QF5	42.72		Sc=5.77056, min distance = 2.38639
PURA1_MOUSE I	5326748 F	PURA_ECOLI Fu	444818 6-	1CG0	1LON	1QF4	42.72		Sc=6.56553, min distance = 2.27041
PURA1_MOUSE I	5326748 F	PURA_ECOLI Fu	8582 Inos	1CIB	1IWE	1QF4	42.72		Sc=6.10475, min distance = 2.15539
PURA1_MOUSE I	5326748 F	PURA_ECOLI Fu	8977 1dar	1QF5	1LON	1QF4	42.72		Sc=6.56276, min distance = 2.13578
PURA1_MOUSE I	5326748 F	PURA_ECOLI Fu	9549204 C	2GCQ	2DGN	1QF4	42.72		Sc=5.77717, min distance = 2.43073

PURA1_MOUSE I	6022 Aden	NDK_THET8 Ful	8977 1dar	1WKK	1LON	1WKL		0.8	Sc=6.04519, min distance = 2.22426
PURA1_MOUSE I	611002 Op	PIM1_HUMAN Fu	6083 aden	1YXU	1MF0	1YXX			Sc=6.14238, min distance = 0.87276
PURA1_MOUSE I	65110 AIC	AAKG1_HUMAN Fu	6083 aden	2UV4	1MF0	2UV5		0.79	Sc=6.26097, min distance = 2.30171
PURA1_MOUSE I	65110 AIC	PURO_METTH Fu	8582 Inos	2NTK	1IWE	2NTL		0.97	Sc=6.33322, min distance = 1.20165
PURA1_MOUSE I	65533 cor	O57693_THETE I	6083 aden	1UXU	1MF0	1UXT			Sc=5.96673, min distance = 2.18556
PURA1_MOUSE I	657135 Op	PIM1_HUMAN Fu	6083 aden	1YXU	1MF0	1YXV			Sc=5.82235, min distance = 2.66730
PURA1_MOUSE I	6804 guar	DPOL_BPR69 Fu	8977 1dar	1CLQ	1LON	1WAJ		0.99	Sc=6.43458, min distance = 2.35034
PURA1_MOUSE I	6804 guar	EF1A_YEAST Fu	8977 1dar	2B7B	1LON	1G7C		0.99	Sc=6.43401, min distance = 2.63288
PURA1_MOUSE I	6804 guar	GMPR2_HUMAN Fu	8582 Inos	2C6Q	1IWE	2A7R		0.99	Sc=6.43155, min distance = 1.89062
PURA1_MOUSE I	73323 Xar	HGXR_TOXGO Fu	8582 Inos	1QK4	1IWE	1QK5		0.94	Sc=6.39741, min distance = 2.38161
PURA1_MOUSE I	93082 Gak	RASH_HUMAN Fu	6830 guan	2CL7	1LOO	121P		0.99	Sc=6.04454, min distance = 2.44326
PURA2_HUMAN I	188347 pp	ARF1_BOVIN Fu	8977 1dar	1R8S	2V40	1R8Q		0.99	Sc=5.75923, min distance = 2.25324
PURA_ARATH Fu	188347 pp	ARF1_BOVIN Fu	8977 1dar	1R8S	1DJ2	1R8Q		0.99	Sc=6.51765, min distance = 2.30077
PURA_ARATH Fu	188347 pp	ARL5A_HUMAN Fu	8977 1dar	2H17	1DJ2	1ZJ6		0.99	Sc=6.519, min distance = 2.1707201
PURA_ARATH Fu	24978488	TBB2B_BOVIN Fu	8977 1dar	1Z2B	1DJ2	3DU7			Sc=6.03889, min distance = 1.65168
PURA_ARATH Fu	37792 gam	ARF6_HUMAN Fu	8977 1dar	1E0S	1DJ2	2J5X		0.99	Sc=6.07664, min distance = 1.81588
PURA_ARATH Fu	37792 gam	GNAI1_RAT Ful	8977 1dar	1SVK	1DJ2	1AS0		0.99	Sc=6.07532, min distance = 2.33377
PURA_ARATH Fu	37792 gam	GNAT1_BOVIN Fu	8977 1dar	1TAD	1DJ2	1TND		0.99	Sc=6.08361, min distance = 1.83498
PURA_ARATH Fu	37792 gam	RAB6B_HUMAN Fu	8977 1dar	2E9S	1DJ2	2FFQ		0.99	Sc=6.08088, min distance = 2.02872
PURA_ARATH Fu	37792 gam	RAC1_HUMAN Fu	8977 1dar	1RYF	1DJ2	2FJU		0.99	Sc=6.0876, min distance = 2.141612
PURA_ARATH Fu	37792 gam	RB11A_HUMAN Fu	8977 1dar	1OIX	1DJ2	1OIW		0.99	Sc=6.07963, min distance = 2.06961
PURA_ARATH Fu	445708 AM	PURA_ECOLI Fu	8977 1dar	1QF5	1DJ2	1SON		0.8	Sc=6.32041, min distance = 2.22579
PURA_ARATH Fu	445871 CI	RASH_HUMAN Fu	8977 1dar	2CE2	1DJ2	1GNP			Sc=6.72202, min distance = 2.04568
PURA_ARATH Fu	446248 CI	RASH_HUMAN Fu	8977 1dar	2CE2	1DJ2	1IAQ			Sc=6.08747, min distance = 2.28557
PURA_ARATH Fu	447979 5G	KGUA_ECOLI Fu	8977 1dar	2AN9	1DJ2	2ANB			Sc=6.39741, min distance = 2.19176
PURA_ARATH Fu	6802 guar	DPOL_BPR69 Fu	8977 1dar	1CLQ	1DJ2	1IH7		0.94	Sc=6.32324, min distance = 2.32557
PURA_ARATH Fu	6804 guar	DPOL_BPR69 Fu	8977 1dar	1CLQ	1DJ2	1WAJ		0.99	Sc=6.41524, min distance = 2.31017
PURA_ARATH Fu	6804 guar	EF1A_YEAST Fu	8977 1dar	2B7B	1DJ2	1G7C		0.99	Sc=5.96585, min distance = 2.56230
PURA_ARATH Fu	93082 Gak	CDC42_HUMAN Fu	8977 1dar	2NGR	1DJ2	2ODB		0.99	Sc=6.12168, min distance = 2.15444
PURA_ARATH Fu	93082 Gak	EFG_THETH Ful	8977 1dar	2BM0	1DJ2	2J7K		0.99	Sc=6.11406, min distance = 2.12897
PURA_ECOLI Fu	446248 CI	RASH_HUMAN Fu	8977 1dar	2CE2	1QF5	1IAQ			Sc=6.09225, min distance = 1.92362
PURA_ECOLI Fu	447145 As	PURA1_MOUSE Fu	444818 6-	1LON	1CG0	1MEZ	42.72	0.83	Sc=6.61178, min distance = 2.27899
PURA_ECOLI Fu	447145 As	PURA1_MOUSE Fu	8582 Inos	1IWE	1CIB	1MEZ	42.72	0.8	Sc=6.60486, min distance = 2.23562
PURA_ECOLI Fu	447145 As	PURA1_MOUSE Fu	9549204 C	2DGN	2GCQ	1MEZ	42.72	0.83	Sc=6.61582, min distance = 2.07836
PURA_ECOLI Fu	447979 5G	KGUA_ECOLI Fu	8977 1dar	2AN9	1QF5	2ANB			Sc=6.39894, min distance = 2.16172
PURA_ECOLI Fu	6022 Aden	NDK_THET8 Ful	8977 1dar	1WKK	1QF5	1WKL		0.8	Sc=6.03387, min distance = 2.27147
PURA_ECOLI Fu	6083 aden	NADE_ECOLI Fu	445708 AM	1WXE	1SON	1WXI		0.91	Sc=6.35869, min distance = 2.19047

PURA_ECOLI Fı	6083 aden	PYGM_RABIT Fu	8582	Inos	2QN7	1CIB	8GPB		0.79	Sc=6.37056, min distance = 2.28627
PURA_ECOLI Fı	65110 AIC	PURO_METTH Fu	8582	Inos	2NTK	1CIB	2NTL		0.97	Sc=6.32225, min distance = 1.63588
PURA_ECOLI Fı	6804 guar	DPOL_BPR69 Fu	8977	1dar	1CLQ	1QF5	1WAJ		0.99	Sc=5.66186, min distance = 2.57244
PURA_ECOLI Fı	6804 guar	EF1A_YEAST Fu	8977	1dar	2B7B	1QF5	1G7C		0.99	Sc=5.66539, min distance = 2.04409
PURK_ECOLI Fı	11608401	KAPCA_BOVIN Fı	5957	Aden	1Q24	3ETH	2UVY			Sc=6.2451, min distance = 2.065654
PURK_ECOLI Fı	11708454	KAPCA_BOVIN Fı	5957	Aden	1Q24	3ETH	2VNW			Sc=6.19798, min distance = 1.94390
PURK_ECOLI Fı	15602983	KAPCA_BOVIN Fı	5957	Aden	1Q24	3ETH	2UW5			Sc=6.17989, min distance = 2.08007
PURK_ECOLI Fı	17751819	KAPCA_BOVIN Fı	5957	Aden	1Q24	3ETH	2VO6			Sc=6.33407, min distance = 1.89213
PURK_ECOLI Fı	3547 Fasu	KAPCA_BOVIN Fı	5957	Aden	1Q24	3ETH	1Q8W			Sc=5.9603, min distance = 2.164080
PURK_ECOLI Fı	440317 AT	PURT_ECOLI Fu	5957	Aden	1KJ8	3ETH	1KJJ		0.99	Sc=6.47448, min distance = 2.55199
PURK_ECOLI Fı	444564 AD	BIOD_ECOLI Fu	5957	Aden	1A82	3ETH	1BS1		0.91	Sc=6.43746, min distance = 2.08345
PURK_ECOLI Fı	445940 O6	CDK2_HUMAN Fu	6022	Aden	1GY3	3ETJ	1GZ8			Sc=6.21253, min distance = 1.64842
PURK_ECOLI Fı	447840 CI	PHKG1_RABIT Fı	5957	Aden	1QL6	3ETH	1PHK			Sc=6.03987, min distance = 2.41060
PURK_ECOLI Fı	447916 ac	RIO1_ARCFU Fu	5957	Aden	1ZP9	3ETH	1ZTF		0.94	Sc=6.23012, min distance = 2.44204
PURK_ECOLI Fı	448042 2e	KAPCA_BOVIN Fı	5957	Aden	1Q24	3ETH	1Q8T			Sc=6.14764, min distance = 2.14380
PURK_ECOLI Fı	448043 2g	KAPCA_BOVIN Fı	5957	Aden	1Q24	3ETH	1Q8U			Sc=6.01358, min distance = 2.16559
PURK_ECOLI Fı	449240 1y	KAPCA_BOVIN Fı	5957	Aden	1Q24	3ETH	1YDR			Sc=5.96713, min distance = 2.07709
PURK_ECOLI Fı	5327148 C	IPKA_RABIT Fu	6022	Aden	1JBP	3ETJ	2ERZ			Sc=6.17987, min distance = 2.21735
PURK_ECOLI Fı	6083 aden	AAKG1_RAT Ful	5957	Aden	2V92	3ETH	2V8Q		0.98	Sc=6.36522, min distance = 1.97216
PURK_ECOLI Fı	6083 aden	PURP_METJA Fu	5957	Aden	2R7L	3ETH	2R7M		0.98	Sc=6.3548, min distance = 2.308293
PURK_ECOLI Fı	6083 aden	PURP_METJA Fu	6022	Aden	2R7N	3ETJ	2R7M		0.99	Sc=6.38356, min distance = 2.54801
PURK_ECOLI Fı	6083 aden	PURP_PYRFU Fu	5957	Aden	2R86	3ETH	2R85		0.98	Sc=6.3548, min distance = 2.607971
PURK_ECOLI Fı	60961 ade	IPKA_RABIT Fu	6022	Aden	1JBP	3ETJ	1FMO		0.95	Sc=6.25934, min distance = 2.36183
PURK_ECOLI Fı	60961 ade	SKY1_YEAST Fu	6022	Aden	1Q8Y	3ETJ	1Q97		0.95	Sc=6.27292, min distance = 1.68178
PURK_ECOLI Fı	6804 guar	NDK_PYRHO Ful	6022	Aden	2DYA	3ETJ	2DXF		0.8	Sc=6.40204, min distance = 2.32968
PURK_ECOLI Fı	8977 1dar	NDK_THET8 Ful	6022	Aden	1WKL	3ETJ	1WKK		0.8	Sc=6.02364, min distance = 1.29890
PURK_ECOLI Fı	91532 AMF	BIOD_ECOLI Fu	6022	Aden	1DAD	3ETJ	1DAG		0.99	Sc=6.01874, min distance = 2.28643
PURK_ECOLI Fı	91532 AMF	MTNK_BACSU Fu	6022	Aden	2OLC	3ETJ	2PUL		0.99	Sc=6.51962, min distance = 2.34178
PURK_ECOLI Fı	91532 AMF	RBSK_ECOLI Fu	6022	Aden	1RKD	3ETJ	1GQT		0.99	Sc=6.54469, min distance = 1.01571
PURK_ECOLI Fı	9547944 2	RNAS1_BOVIN Fı	6022	Aden	1O0H	3ETJ	2G8R			Sc=6.46276, min distance = 1.75510
PURL_THEMA Fı	11314340	KAPCA_BOVIN Fı	5957	Aden	1Q24	2HS0	2UZT			Sc=6.39916, min distance = 2.13054
PURL_THEMA Fı	11708454	KAPCA_BOVIN Fı	5957	Aden	1Q24	2HS0	2VNW			Sc=6.18562, min distance = 2.24104
PURL_THEMA Fı	16122632	KAPCA_BOVIN Fı	5957	Aden	1Q24	2HS0	2UVZ			Sc=6.38839, min distance = 2.08714
PURL_THEMA Fı	33113 gam	PURT_ECOLI Fu	91532	AMF	1KJI	2HS4	1EYZ		0.99	Sc=6.06412, min distance = 1.74868
PURL_THEMA Fı	3540 1yds	KAPCA_BOVIN Fı	5957	Aden	1Q24	2HS0	1YDS			Sc=5.90112, min distance = 2.32044
PURL_THEMA Fı	444503 FU	NDKC_DICDI Fu	6022	Aden	1KDN	2HRU	1B99			Sc=5.91655, min distance = 1.85034
PURL_THEMA Fı	444564 AD	BIOD_ECOLI Fu	5957	Aden	1A82	2HS0	1BS1		0.91	Sc=6.39694, min distance = 2.63356

PURL_THEMA F	444564 AD	BIOD_ECOLI Fu	6022 Aden	1DAD	2HRU	1BS1		0.91	Sc=5.9388, min distance = 2.2537688
PURL_THEMA F	444564 AD	MYS2_DICDI Fu	5957 Aden	1FMW	2HS0	1W9I		0.91	Sc=6.40423, min distance = 0
PURL_THEMA F	446090 1h	NDKC_DICDI Fu	6022 Aden	1KDN	2HRU	1HIY		0.97	Sc=6.40913, min distance = 1.968136
PURL_THEMA F	447916 ad	RIO1_ARCFU Fu	5957 Aden	1ZP9	2HS0	1ZTF		0.94	Sc=6.26145, min distance = 2.632391
PURL_THEMA F	447916 ad	RIO1_ARCFU Fu	6022 Aden	1ZTH	2HRU	1ZTF		0.95	Sc=6.25098, min distance = 2.040867
PURL_THEMA F	449240 1y	KAPCA_BOVIN F	5957 Aden	1Q24	2HS0	1YDR			Sc=5.95819, min distance = 2.022682
PURL_THEMA F	5327103 2	HS90A_HUMAN F	6022 Aden	1BYQ	2HRU	2BYH			Sc=6.20348, min distance = 2.015458
PURL_THEMA F	6083 aden	PURP_METJA Fu	5957 Aden	2R7L	2HS0	2R7M		0.98	Sc=6.33814, min distance = 2.877282
PURL_THEMA F	6083 aden	PURP_PYRFU Fu	5957 Aden	2R86	2HS0	2R85		0.98	Sc=6.3529, min distance = 2.3913880
PURL_THEMA F	6133 uric	CCA_ARCFU Ful	5957 Aden	1R8B	2HS0	1R8C			Sc=6.35382, min distance = 2.422954
PURL_THEMA F	6852207 M	KAPCA_BOVIN F	5957 Aden	1Q24	2HS0	2GNI			Sc=5.97731, min distance = 2.012821
PURO_METTH F	100252 Ri	IMDH_TRIFO Fu	8582 Inos	1MEH	2NTK	1ME8			Sc=6.30218, min distance = 2.068704
PURO_METTH F	6083 aden	AAKG1_HUMAN F	65110 AIC	2UV5	2NTL	2UV4		0.79	Sc=6.42726, min distance = 1.949194
PURP_METJA F	11708454	KAPCA_BOVIN F	5957 Aden	1Q24	2R7L	2VNW			Sc=6.18678, min distance = 2.275316
PURP_METJA F	1400 nche	RET_HUMAN Ful	6083 aden	2IVT	2R7M	2IVV			Sc=6.02205, min distance = 2.245795
PURP_METJA F	16122632	KAPCA_BOVIN F	5957 Aden	1Q24	2R7L	2UVZ			Sc=6.37475, min distance = 2.174288
PURP_METJA F	16122635	KAPCA_BOVIN F	5957 Aden	1Q24	2R7L	2UW8			Sc=5.69628, min distance = 2.052180
PURP_METJA F	24762195	KAPCA_BOVIN F	5957 Aden	1Q24	2R7L	2VO0			Sc=6.31461, min distance = 2.274707
PURP_METJA F	36735 Gpp	Q9RVK2_DEIRA	5957 Aden	1SU2	2R7L	1SZ3		0.8	Sc=5.94371, min distance = 2.156442
PURP_METJA F	41211 3'-	CSKP_HUMAN Fu	6083 aden	3C0H	2R7M	3C0I	1		Sc=5.82692, min distance = 2.403517
PURP_METJA F	440317 AT	FTSK_PSEAE Fu	6022 Aden	2IUU	2R7N	2IUT		0.99	Sc=6.00673, min distance = 1.970514
PURP_METJA F	440317 AT	SRC_CHICK Ful	6083 aden	3DQX	2R7M	3DQW		0.97	Sc=5.97251, min distance = 2.476181
PURP_METJA F	444564 AD	MYS2_DICDI Fu	5957 Aden	1FMW	2R7L	1W9I		0.91	Sc=5.94395, min distance = 0
PURP_METJA F	444852 CI	ATPA1_BOVIN F	5957 Aden	2V7Q	2R7L	1COW		0.91	Sc=5.96529, min distance = 2.363396
PURP_METJA F	445840 di	CDK2_HUMAN Fu	6022 Aden	1GY3	2R7N	1GII			Sc=6.2645, min distance = 1.6659948
PURP_METJA F	446090 1h	NDKC_DICDI Fu	6022 Aden	1KDN	2R7N	1HIY		0.97	Sc=6.4349, min distance = 2.1237638
PURP_METJA F	447840 CI	PHKG1_RABIT F	5957 Aden	1QL6	2R7L	1PHK			Sc=5.97392, min distance = 2.627558
PURP_METJA F	447916 ad	RIO1_ARCFU Fu	5957 Aden	1ZP9	2R7L	1ZTF		0.94	Sc=6.2582, min distance = 2.3549411
PURP_METJA F	447916 ad	RIO1_ARCFU Fu	6022 Aden	1ZTH	2R7N	1ZTF		0.95	Sc=6.27543, min distance = 2.236855
PURP_METJA F	448222 NE	ENPL_CANFA Fu	6022 Aden	1TC6	2R7N	1QY5		0.81	Sc=6.37962, min distance = 2.207484
PURP_METJA F	449240 1y	KAPCA_BOVIN F	5957 Aden	1Q24	2R7L	1YDR			Sc=5.98801, min distance = 2.308819
PURP_METJA F	60961 ade	IPKA_RABIT Fu	6022 Aden	1JBP	2R7N	1FMO		0.95	Sc=6.2582, min distance = 2.3729926
PURP_METJA F	611002 Op	PIM1_HUMAN Fu	6083 aden	1YXU	2R7M	1YXX			Sc=6.1096, min distance = 2.1355931
PURP_METJA F	6176 Cyti	PYRI_ECOLI Fu	5957 Aden	7AT1	2R7L	2FZC			Sc=5.87802, min distance = 1.793352
PURP_METJA F	656964 1y	PDE4D_HUMAN F	6083 aden	1TB7	2R7M	1Y2C			Sc=5.81761, min distance = 2.278716
PURP_METJA F	656966 1y	PDE4D_HUMAN F	6083 aden	1TB7	2R7M	1Y2E			Sc=5.973, min distance = 1.9490728
PURP_METJA F	656969 1y	PDE4B_HUMAN F	6083 aden	1ROR	2R7M	1Y2J			Sc=6.18446, min distance = 2.156228

PURP_METJA Fı 6804 guar	PDE10_HUMAN Fı 6083 aden 2OUN	2R7M	2OUQ		0.8	Sc=6.37789, min distance = 0
PURP_METJA Fı 6852207 M	KAPCA_BOVIN Fı 5957 Aden 1Q24	2R7L	2GNI			Sc=5.97535, min distance = 2.23493(
PURP_METJA Fı 8582 Inos	PYGM_RABIT Ful 6083 aden 8GPB	2R7M	2QN7		0.79	Sc=6.3392, min distance = 2.589738!
PURP_METJA Fı 8977 1dar	NDK_PYRHO Full 6022 Aden 2DYA	2R7N	2DXE		0.8	Sc=6.00373, min distance = 2.16275⁄
PURP_METJA Fı 9994066 4	CDK2_HUMAN Ful 6022 Aden 1GY3	2R7N	2VTJ			Sc=5.88168, min distance = 2.37913!
PURP_PYRFU Fı 11608401	KAPCA_BOVIN Fı 5957 Aden 1Q24	2R86	2UVY			Sc=6.19023, min distance = 2.07381(
PURP_PYRFU Fı 11708454	KAPCA_BOVIN Fı 5957 Aden 1Q24	2R86	2VNW			Sc=6.12681, min distance = 2.47740(
PURP_PYRFU Fı 15342951	PYGM_RABIT Ful 6083 aden 8GPB	2R85	3BCR			Sc=6.01955, min distance = 2.10083⁄
PURP_PYRFU Fı 15942652	CDK2_HUMAN Ful 6022 Aden 1GY3	2R87	2DUV			Sc=6.3647, min distance = 1.629543:
PURP_PYRFU Fı 16122632	KAPCA_BOVIN Fı 5957 Aden 1Q24	2R86	2UVZ			Sc=6.35409, min distance = 2.24715⁄
PURP_PYRFU Fı 24836816	HS90A_HUMAN Fı 6022 Aden 1BYQ	2R87	3BMY			Sc=6.37197, min distance = 1.99743!
PURP_PYRFU Fı 3540 1yds	KAPCA_BOVIN Fı 5957 Aden 1Q24	2R86	1YDS			Sc=5.83364, min distance = 2.39065:
PURP_PYRFU Fı 3547 Fasu	KAPCA_BOVIN Fı 5957 Aden 1Q24	2R86	1Q8W			Sc=5.90625, min distance = 2.31630:
PURP_PYRFU Fı 3973 nche	PIM1_HUMAN Ful 6083 aden 1YXU	2R85	1YI3			Sc=5.95721, min distance = 1.96864:
PURP_PYRFU Fı 440317 AT	SRC_CHICK Full 6083 aden 3DQX	2R85	3DQW		0.97	Sc=5.60028, min distance = 1.11317(
PURP_PYRFU Fı 444564 AD	BIOD_ECOLI Fu 6022 Aden 1DAD	2R87	1BS1		0.91	Sc=6.45364, min distance = 2.24755⁄
PURP_PYRFU Fı 444842 CI	CDK2_HUMAN Ful 6022 Aden 1GY3	2R87	1CKP			Sc=5.60554, min distance = 2.21978⁄
PURP_PYRFU Fı 445840 di	CDK2_HUMAN Ful 6022 Aden 1GY3	2R87	1GII			Sc=6.27545, min distance = 1.63473⁄
PURP_PYRFU Fı 445841 py	CDK2_HUMAN Ful 6022 Aden 1GY3	2R87	1GIJ			Sc=6.44664, min distance = 1.95937!
PURP_PYRFU Fı 447654 CI	CDK2_HUMAN Ful 6022 Aden 1GY3	2R87	1OIT			Sc=6.18474, min distance = 2.00561{
PURP_PYRFU Fı 447916 ac	RIO1_ARCFU Ful 5957 Aden 1ZP9	2R86	1ZTF		0.94	Sc=6.17057, min distance = 2.78815⁄
PURP_PYRFU Fı 447955 1p	CDK2_HUMAN Ful 6022 Aden 1GY3	2R87	1PXI			Sc=5.78991, min distance = 2.11899{
PURP_PYRFU Fı 449240 1y	KAPCA_BOVIN Fı 5957 Aden 1Q24	2R86	1YDR			Sc=5.89401, min distance = 2.29463(
PURP_PYRFU Fı 4566 1h1s	CDK2_HUMAN Ful 6022 Aden 1GY3	2R87	1H1S			Sc=6.34795, min distance = 1.47337{
PURP_PYRFU Fı 60961 ade	IPKA_RABIT Ful 6022 Aden 1JBP	2R87	1FMO		0.95	Sc=6.25934, min distance = 2.50464{
PURP_PYRFU Fı 6338561 C	MYS2_DICDI Ful 6022 Aden 1VOM	2R87	1D0X			Sc=6.21582, min distance = 1.89628{
PURP_PYRFU Fı 65051 ddT	NDKC_DICDI Ful 6022 Aden 1KDN	2R87	1F3F			Sc=6.43092, min distance = 1.35116:
PURP_PYRFU Fı 656964 1y	PDE4D_HUMAN Fı 6083 aden 1TB7	2R85	1Y2C			Sc=5.78973, min distance = 2.09343!
PURP_PYRFU Fı 656969 1y	PDE4B_HUMAN Fı 6083 aden 1ROR	2R85	1Y2J			Sc=6.16964, min distance = 2.31198'
PURP_PYRFU Fı 6852207 M	KAPCA_BOVIN Fı 5957 Aden 1Q24	2R86	2GNI			Sc=5.89227, min distance = 2.16055:
PURP_PYRFU Fı 91557 1tm	Q94M05_9VIRU I 6022 Aden 1W44	2R87	1W48		0.99	Sc=6.08049, min distance = 1.18077:
PURP_PYRFU Fı 9817550 V	CDK2_HUMAN Ful 6022 Aden 1GY3	2R87	3BHV			Sc=6.40106, min distance = 1.65825!
PURP_PYRFU Fı 9994066 4	CDK2_HUMAN Ful 6022 Aden 1GY3	2R87	2VTJ			Sc=5.95377, min distance = 2.48460(
PURR_BACSU Fı 164795 N(	RBCMT_PEA Ful 23831 HEF 1MLV	1O57	2H2J			Sc=5.74887, min distance = 1.25526:
PURR_BACSU Fı 2014 CID2	TRY1_BOVIN Fu 17472 Hex 2G81	1O57	1BJU			Sc=6.08675, min distance = 2.24835:
PURR_BACSU Fı 378 4-nit	PPAL_YEAST Ful 23831 HEF 1D1P	1O57	1D1Q			Sc=5.79098, min distance = 2.20928⁄
PURR_BACSU Fı 6102772 C	ACES_MOUSE Ful 17472 Hex 2HA2	1O57	2GYU		0.81	Sc=5.81768, min distance = 2.49897:

PURR_ECOLI Fu	100684 9-	RICI_RICCO Ful	190 adeni	2P8N	2PUB	1IL4			Sc=5.78164, min distance = 2.41016
PURR_ECOLI Fu	100684 9-	TGT_ZYMMO Ful	764 guani	2PWU	1WET	1Q2R			Sc=5.89242, min distance = 2.81513
PURR_ECOLI Fu	1188 xant	XGPT_ECOLI Fu	764 guani	1A95	1WET	1A96		0.92	Sc=5.85163, min distance = 2.62334
PURR_ECOLI Fu	11979 5-M	COBT_SALTY Fu	190 adeni	1JH8	2PUB	1JHM			Sc=5.67711, min distance = 2.50636
PURR_ECOLI Fu	242831 2-	TGT_ZYMMO Ful	764 guani	2PWU	1WET	1S39			Sc=5.95951, min distance = 2.44955
PURR_ECOLI Fu	439713 (F	URAD_DANRE Fu	764 guani	2O74	1WET	2O73			Sc=5.83442, min distance = 2.45279
PURR_ECOLI Fu	446312 1i	RICI_RICCO Fu	190 adeni	2P8N	2PUB	1IL9			Sc=5.79566, min distance = 2.25987
PURR_ECOLI Fu	5078682 C	PNPH_BOVIN Fu	790 hypox	1A9Q	1JFT	2QPL			Sc=5.92682, min distance = 2.01183
PURR_ECOLI Fu	5287830 6	Q8RLY5_LACHE I	190 adeni	1S2D	2PUB	1S2I		0.93	Sc=5.64481, min distance = 2.97539
PURR_ECOLI Fu	675 Dimed	COBT_SALTY Fu	190 adeni	1JH8	2PUB	1D0S			Sc=5.73504, min distance = 2.63462
PURR_ECOLI Fu	76635 4,5	COBT_SALTY Fu	190 adeni	1JH8	2PUB	1L4F			Sc=5.6648, min distance = 2.079094
PURR_ECOLI Fu	8646 Guar	PNPH_HUMAN Fu	764 guani	1V2H	1WET	1V41			Sc=5.85809, min distance = 2.26013
PURR_ECOLI Fu	96253 7-D	RICI_RICCO Fu	190 adeni	2P8N	2PUB	1IL3			Sc=5.79423, min distance = 2.49755
PURT_ECOLI Fu	11608401	KAPCA_BOVIN Fu	5957 Aden	1Q24	1KJ8	2UVY			Sc=6.21679, min distance = 1.94813
PURT_ECOLI Fu	11708454	KAPCA_BOVIN Fu	5957 Aden	1Q24	1KJ8	2VNW			Sc=6.15352, min distance = 2.12506
PURT_ECOLI Fu	11957393	FAK1_HUMAN Fu	6022 Aden	1MP8	1KJQ	2ETM			Sc=6.31416, min distance = 2.20168
PURT_ECOLI Fu	129236 2c	CKI1_SCHPO Fu	5957 Aden	1CSN	1KJ8	2CSN			Sc=5.82028, min distance = 1.92726
PURT_ECOLI Fu	16122607	PDPK1_HUMAN Fu	5957 Aden	2BIY	1KJ8	2PE0			Sc=6.15325, min distance = 2.18890
PURT_ECOLI Fu	16122608	PDPK1_HUMAN Fu	5957 Aden	2BIY	1KJ8	2PE1			Sc=6.36143, min distance = 2.18731
PURT_ECOLI Fu	16214826	CDK2_HUMAN Fu	6022 Aden	1GY3	1KJQ	2UZL			Sc=6.11487, min distance = 2.06606
PURT_ECOLI Fu	1707 1fvt	CDK2_HUMAN Fu	6022 Aden	1GY3	1KJQ	1FVT			Sc=6.11218, min distance = 1.96645
PURT_ECOLI Fu	17754396	PDPK1_HUMAN Fu	5957 Aden	2BIY	1KJ8	2R7B			Sc=6.30136, min distance = 2.15799
PURT_ECOLI Fu	23653523	CDK2_HUMAN Fu	6022 Aden	1GY3	1KJQ	2R3O			Sc=6.37211, min distance = 2.03769
PURT_ECOLI Fu	24779674	CSK2A_MAIZE Fu	33113 gam	1LP4	1EYZ	2PVH			Sc=6.21996, min distance = 2.44077
PURT_ECOLI Fu	2608 1jsv	CDK2_HUMAN Fu	6022 Aden	1GY3	1KJQ	1JSV			Sc=6.01955, min distance = 1.45466
PURT_ECOLI Fu	3044933 A	ENPL_CANFA Fu	6022 Aden	1TC6	1KJQ	1U0Y		0.81	Sc=6.37755, min distance = 2.27897
PURT_ECOLI Fu	3540 1yds	KAPCA_BOVIN Fu	5957 Aden	1Q24	1KJ8	1YDS			Sc=5.85757, min distance = 2.44000
PURT_ECOLI Fu	36735 Gpp	CSK2A_MAIZE Fu	33113 gam	1LP4	1EYZ	1DAY		0.8	Sc=5.98265, min distance = 2.09016
PURT_ECOLI Fu	444345 1c	PDPK1_HUMAN Fu	5957 Aden	2BIY	1KJ8	1OKY			Sc=6.34086, min distance = 2.31091
PURT_ECOLI Fu	444564 AD	BIOD_ECOLI Fu	5957 Aden	1A82	1KJ8	1BS1		0.91	Sc=6.41531, min distance = 2.13427
PURT_ECOLI Fu	444564 AD	BIOD_ECOLI Fu	91532 AMF	1DAG	1KJI	1BS1		0.91	Sc=6.04837, min distance = 2.13935
PURT_ECOLI Fu	444564 AD	MYS2_DICDI Fu	33113 gam	1MMN	1EYZ	1W9I		0.91	Sc=5.95851, min distance = 2.00144
PURT_ECOLI Fu	445940 O6	CDK2_HUMAN Fu	6022 Aden	1GY3	1KJQ	1GZ8			Sc=6.10488, min distance = 1.77040
PURT_ECOLI Fu	445998 CI	SYP_RHOPA Ful	5957 Aden	2I4O	1KJ8	2I4M		0.88	Sc=6.5933, min distance = 0.931011
PURT_ECOLI Fu	446704 LS	CDK2_HUMAN Fu	6022 Aden	1GY3	1KJQ	1KE6			Sc=6.26869, min distance = 1.91137
PURT_ECOLI Fu	447377 1r	SYP_RHOPA Ful	5957 Aden	2I4O	1KJ8	2I4N		0.84	Sc=6.49894, min distance = 1.37257
PURT_ECOLI Fu	447766 1p	CDK2_HUMAN Fu	6022 Aden	1GY3	1KJQ	1P2A			Sc=6.35847, min distance = 1.60752

PURT_ECOLI Fu	447821 CI	CDK2_HUMAN Fu	6022 Aden	1GY3	1KJQ	1PF8			Sc=6.13673, min distance = 2.22482
PURT_ECOLI Fu	447840 CI	PHKG1_RABIT Fu	5957 Aden	1QL6	1KJ8	1PHK			Sc=6.46801, min distance = 2.35421
PURT_ECOLI Fu	447955 1p	CDK2_HUMAN Fu	6022 Aden	1GY3	1KJQ	1PXI			Sc=5.76545, min distance = 2.25462
PURT_ECOLI Fu	448042 2e	KAPCA_BOVIN Fu	5957 Aden	1Q24	1KJ8	1Q8T			Sc=6.01809, min distance = 2.31131
PURT_ECOLI Fu	448043 2g	KAPCA_BOVIN Fu	5957 Aden	1Q24	1KJ8	1Q8U			Sc=5.98475, min distance = 2.29617
PURT_ECOLI Fu	448222 NE	ENPL_CANFA Fu	6022 Aden	1TC6	1KJQ	1QY5		0.81	Sc=6.35811, min distance = 2.16255
PURT_ECOLI Fu	448965 1u	HS90A_HUMAN Fu	6022 Aden	1BYQ	1KJQ	1UY6			Sc=6.18155, min distance = 2.40858
PURT_ECOLI Fu	448974 1u	HS90A_HUMAN Fu	6022 Aden	1BYQ	1KJQ	1UYH			Sc=6.17607, min distance = 2.06245
PURT_ECOLI Fu	449240 1y	KAPCA_BOVIN Fu	5957 Aden	1Q24	1KJ8	1YDR			Sc=5.93726, min distance = 2.23742
PURT_ECOLI Fu	5288708 1	CDK2_HUMAN Fu	6022 Aden	1GY3	1KJQ	1KE5			Sc=6.12512, min distance = 1.85824
PURT_ECOLI Fu	5288710 C	CDK2_HUMAN Fu	6022 Aden	1GY3	1KJQ	1KE7			Sc=6.32987, min distance = 1.64132
PURT_ECOLI Fu	5327097 2	CDK2_HUMAN Fu	6022 Aden	1GY3	1KJQ	2BTS			Sc=5.89747, min distance = 1.99706
PURT_ECOLI Fu	5327132 C	CDK2_HUMAN Fu	6022 Aden	1GY3	1KJQ	2C6I			Sc=6.26795, min distance = 2.03331
PURT_ECOLI Fu	5327148 C	IPKA_RABIT Fu	33113 gam	1CDK	1EYZ	2ERZ			Sc=6.1463, min distance = 2.371389
PURT_ECOLI Fu	5327148 C	IPKA_RABIT Fu	6022 Aden	1JBP	1KJQ	2ERZ			Sc=6.14764, min distance = 2.51023
PURT_ECOLI Fu	5496922 C	HS90A_HUMAN Fu	6022 Aden	1BYQ	1KJQ	1UY7			Sc=6.22967, min distance = 2.33003
PURT_ECOLI Fu	6083 aden	AAKG1_RAT Full	5957 Aden	2V92	1KJ8	2V8Q		0.98	Sc=6.32622, min distance = 1.94346
PURT_ECOLI Fu	6083 aden	HSP71_HUMAN Fu	33113 gam	2E8A	1EYZ	1XQS		0.97	Sc=6.28344, min distance = 1.87693
PURT_ECOLI Fu	6083 aden	PURP_METJA Fu	91532 AMP	2R7K	1KJI	2R7M		0.97	Sc=5.93519, min distance = 2.23299
PURT_ECOLI Fu	6083 aden	PURP_PYRFU Fu	5957 Aden	2R86	1KJ8	2R85		0.98	Sc=6.33495, min distance = 2.65609
PURT_ECOLI Fu	6083 aden	SRC_CHICK Full	440317 AT	3DQW	1KJJ	3DQX		0.97	Sc=6.30011, min distance = 1.75321
PURT_ECOLI Fu	60961 ade	SKY1_YEAST Fu	33113 gam	1Q99	1EYZ	1Q97		0.93	Sc=6.23841, min distance = 1.44342
PURT_ECOLI Fu	60961 ade	SKY1_YEAST Fu	6022 Aden	1Q8Y	1KJQ	1Q97		0.95	Sc=6.15213, min distance = 2.56873
PURT_ECOLI Fu	60961 ade	STK6_HUMAN Fu	33113 gam	2DWB	1EYZ	1MUO		0.93	Sc=6.17584, min distance = 2.56869
PURT_ECOLI Fu	60961 ade	STK6_HUMAN Fu	6022 Aden	1MQ4	1KJQ	1MUO		0.95	Sc=6.19516, min distance = 2.24623
PURT_ECOLI Fu	6830 guar	Q381M1_9TRYP I	5957 Aden	2Q0D	1KJ8	2Q0E		0.8	Sc=6.02028, min distance = 2.11359
PURT_ECOLI Fu	6852207 M	KAPCA_BOVIN Fu	5957 Aden	1Q24	1KJ8	2GNI			Sc=5.93852, min distance = 1.90629
PURT_ECOLI Fu	8977 1dar	NDK_THET8 Full	5957 Aden	1WKL	1KJ8	1WKK		0.8	Sc=5.98308, min distance = 1.61897
PURT_ECOLI Fu	9549303 2	STK6_HUMAN Fu	33113 gam	2DWB	1EYZ	2NP8			Sc=6.51952, min distance = 2.07347
PURT_PYRHO Fu	1707 1fvt	CDK2_HUMAN Fu	6022 Aden	1GY3	2DWC	1FVT			Sc=6.25133, min distance = 1.50778
PURT_PYRHO Fu	23653523	CDK2_HUMAN Fu	6022 Aden	1GY3	2DWC	2R3O			Sc=6.42147, min distance = 2.03886
PURT_PYRHO Fu	444842 CI	CDK2_HUMAN Fu	6022 Aden	1GY3	2DWC	1CKP			Sc=5.63672, min distance = 2.17524
PURT_PYRHO Fu	444852 CI	ATPB_BOVIN Fu	6022 Aden	2CK3	2DWC	1COW		0.9	Sc=5.99374, min distance = 1.88933
PURT_PYRHO Fu	445940 O6	CDK2_HUMAN Fu	6022 Aden	1GY3	2DWC	1GZ8			Sc=6.22156, min distance = 1.95520
PURT_PYRHO Fu	445966 O6	CDK2_HUMAN Fu	6022 Aden	1GY3	2DWC	1H0V			Sc=6.30607, min distance = 2.22009
PURT_PYRHO Fu	446090 1h	NDKC_DICDI Fu	6022 Aden	1KDN	2DWC	1HIY		0.97	Sc=6.40273, min distance = 2.09820
PURT_PYRHO Fu	446704 LS	CDK2_HUMAN Fu	6022 Aden	1GY3	2DWC	1KE6			Sc=6.37042, min distance = 2.04317

PURT_PYRHO F	447654 CI	CDK2_HUMAN Fu	6022 Aden	1GY3	2DWC	1OIT		Sc=6.28901, min distance = 2.02775
PURT_PYRHO F	447916 ac	RIO1_ARCFU Fu	6022 Aden	1ZTH	2DWC	1ZTF	0.95	Sc=6.2582, min distance = 2.829450
PURT_PYRHO F	447955 1p	CDK2_HUMAN Fu	6022 Aden	1GY3	2DWC	1PXI		Sc=5.79875, min distance = 2.178090
PURT_PYRHO F	448008 CI	GSK3B_HUMAN F	6022 Aden	1J1C	2DWC	1Q4L		Sc=6.44727, min distance = 1.68663
PURT_PYRHO F	448965 1u	HS90A_HUMAN F	6022 Aden	1BYQ	2DWC	1UY6		Sc=6.35113, min distance = 2.04558
PURT_PYRHO F	5327097 2	CDK2_HUMAN Fu	6022 Aden	1GY3	2DWC	2BTS		Sc=5.94044, min distance = 1.92444
PURT_PYRHO F	5327148 C	IPKA_RABIT Fu	6022 Aden	1JBP	2DWC	2ERZ		Sc=6.17763, min distance = 2.01446
PURT_PYRHO F	5957 Aden	HSP7F_YEAST F	6022 Aden	3C7N	2DWC	3D2F	0.99	Sc=5.99876, min distance = 1.94476
PURT_PYRHO F	5957 Aden	MALK_ECOLI Fu	6022 Aden	2AWN	2DWC	1Q12	0.99	Sc=5.96438, min distance = 1.45962
PURT_PYRHO F	5957 Aden	MUTS_ECOLI Fu	6022 Aden	1OH7	2DWC	1W7A	0.99	Sc=5.99947, min distance = 2.08657
PURT_PYRHO F	5957 Aden	Y059_METJA Fu	6022 Aden	2J9D	2DWC	2J9C	0.99	Sc=6.46412, min distance = 2.12654
PURT_PYRHO F	6083 aden	PURP_METJA Fu	6022 Aden	2R7N	2DWC	2R7M	0.99	Sc=5.99424, min distance = 2.46440
PURT_PYRHO F	60961 ade	SKY1_YEAST Fu	6022 Aden	1Q8Y	2DWC	1Q97	0.95	Sc=6.24707, min distance = 2.41991
PURT_PYRHO F	6132 Cyti	RIR1_YEAST Fu	6022 Aden	2CVX	2DWC	2CVU		Sc=6.26848, min distance = 1.48029
PURT_PYRHO F	6420140 2	CDK2_HUMAN Fu	6022 Aden	1GY3	2DWC	2C5Y		Sc=6.54107, min distance = 1.98624
PURT_PYRHO F	6804 guar	NDK_PYRHO Ful	6022 Aden	2DYA	2DWC	2DXF	0.8	Sc=6.02783, min distance = 2.50646
PURT_PYRHO F	8977 1dar	NDK_THET8 Ful	6022 Aden	1WKL	2DWC	1WKK	0.8	Sc=6.41519, min distance = 2.14244
PURT_PYRHO F	91532 AMI	PURP_METJA Fu	6022 Aden	2R7N	2DWC	2R7K	0.99	Sc=6.01543, min distance = 2.24292
PURT_PYRHO F	91532 AMI	PURT_ECOLI Fu	6022 Aden	1KJQ	2DWC	1KJI	0.99	Sc=6.01874, min distance = 2.31033
PURT_PYRHO F	9991833 S	CDK2_HUMAN Fu	6022 Aden	1GY3	2DWC	2R3H		Sc=6.05783, min distance = 1.95900
PURT_PYRHO F	9994066 4	CDK2_HUMAN Fu	6022 Aden	1GY3	2DWC	2VTJ		Sc=5.95256, min distance = 2.11624
PUS10_HUMAN I	164795 N	RBCMT_PEA Ful	23831 HEF	1MLV	2V9K	2H2J		Sc=5.61167, min distance = 2.45758
PUS10_HUMAN I	444305 L-	XYNA_BACSU Fu	23831 HEF	2B45	2V9K	2DCY		Sc=5.79517, min distance = 0.90134
PUS10_HUMAN I	64689 bet	GUB_PAEMA Ful	23831 HEF	1AJK	2V9K	1U0A		Sc=5.93054, min distance = 0.63468
PUS10_HUMAN I	8200 TETF	Q8WSF8_APLCA I	23831 HEF	2BR7	2V9K	2BYN		Sc=5.73968, min distance = 1.10850
PUS10_HUMAN I	82313 1rc	LYSC_CHICK Fu	23831 HEF	2HUB	2V9K	1LSZ		Sc=5.9366, min distance = 2.316083
PUTA_ECOLI F	854023 Ep	Q8WSF8_APLCA I	62551 Pen	2BYN	1K87	2BYQ		Sc=5.6617, min distance = 1.912366
PYGL_HUMAN F	1052 pyri	PDXK_SHEEP Fu	95687 Dec	1RFU	1XOI	1RFT	0.92	Sc=5.91376, min distance = 1.64553
PYGL_HUMAN F	2153 theo	Q873X9_ASPFU I	2519 caff	2A3B	1L5Q	2A3A	0.91	Sc=6.02736, min distance = 0.70783
PYGL_HUMAN F	24905143	SRC_CHICK Ful	6083 aden	3DQX	1FA9	3EN7		Sc=6.06417, min distance = 2.22722
PYGL_HUMAN F	445916 be	XYLA_STRRU Fu	79025 alp	1XIF	2ATI	1GW9	0.93	Sc=5.61389, min distance = 2.83396
PYGL_HUMAN F	448895 AI	UMPK_YEAST Fu	6083 aden	1UKZ	1FA9	1UKY	0.99	Sc=5.80138, min distance = 2.50779
PYGL_HUMAN F	449180 XI	XYLA_STRRU Fu	79025 alp	1XIF	2ATI	4XIS		Sc=5.71461, min distance = 2.17421
PYGL_HUMAN F	449193 Rc	PDE4B_HUMAN F	6083 aden	1ROR	1FA9	1XMU		Sc=5.90099, min distance = 2.28002
PYGL_HUMAN F	449225 3-	XYLA_STROL Fu	79025 alp	1S5M	2ATI	1XYC		Sc=5.95073, min distance = 1.62876
PYGL_HUMAN F	449343 2g	XYLA_STROL Fu	79025 alp	1S5M	2ATI	2GYI		Sc=5.7325, min distance = 2.259708
PYGL_HUMAN F	4740 pent	Q873X9_ASPFU I	2519 caff	2A3B	1L5Q	2A3C	0.89	Sc=6.25524, min distance = 2.15556

PYGL_HUMAN F	5289590 D	XYLA_STRRU Fu	79025 alp	1XIF	2ATI	1XII		Sc=5.72465, min distance = 2.460188
PYGL_HUMAN F	5326566 l	Q9WZW1_THEMA	444658 2a	1T6Z	1L5R	1S4M	0.85	Sc=6.3327, min distance = 2.4940064
PYGL_HUMAN F	5327121 I	PIM1_HUMAN Fu	6083 aden	1YXU	1FA9	2C3I		Sc=5.94104, min distance = 2.368983
PYGL_HUMAN F	5780 D-Sc	XYLA_STRRU Fu	79025 alp	1XIF	2ATI	1XIH	0.78	Sc=5.94196, min distance = 2.391918
PYGL_HUMAN F	64960 Pol	XYLA_STRRU Fu	79025 alp	1XIF	2ATI	1XIE	0.89	Sc=5.75873, min distance = 2.513526
PYGL_HUMAN F	892 myo-i	SFTPD_HUMAN F	79025 alp	1PWB	2ATI	2OS9		Sc=5.90909, min distance = 1.838217
PYGL_HUMAN F	95259 L-x	XYLA_STRRU Fu	79025 alp	1XIF	2ATI	1XIC		Sc=5.70633, min distance = 2.004463
PYGL_HUMAN F	9578243 E	PDE4B_HUMAN F	6083 aden	1ROR	1FA9	1XLZ		Sc=5.98564, min distance = 2.549131
PYGM_HUMAN F	100684 9-	RICI_RICCO Fu	190 adeni	2P8N	1Z8D	1IL4		Sc=5.71748, min distance = 2.981034
PYGM_HUMAN F	11550797	PYGM_RABIT Fu	79025 alp	1H5U	1Z8D	2FET		Sc=6.31976, min distance = 2.621750
PYGM_HUMAN F	124823 6g	PYGM_RABIT Fu	79025 alp	1H5U	1Z8D	6GPB	0.78	Sc=6.09081, min distance = 2.506365
PYGM_HUMAN F	15042826	PYGM_RABIT Fu	79025 alp	1H5U	1Z8D	3BCS		Sc=6.31779, min distance = 2.669192
PYGM_HUMAN F	15613364	PYGM_RABIT Fu	79025 alp	1H5U	1Z8D	3BD7		Sc=6.35922, min distance = 2.635292
PYGM_HUMAN F	21678292	PYGM_RABIT Fu	79025 alp	1H5U	1Z8D	3BD8		Sc=6.32013, min distance = 2.620119
PYGM_HUMAN F	24180721	PIM1_HUMAN Fu	6083 aden	1YXU	1Z8D	3C4E		Sc=5.99648, min distance = 2.318523
PYGM_HUMAN F	24851683	PYGM_RABIT Fu	79025 alp	1H5U	1Z8D	2QLM		Sc=6.5302, min distance = 2.3336518
PYGM_HUMAN F	24856350	PYGM_RABIT Fu	79025 alp	1H5U	1Z8D	2QN7		Sc=6.52318, min distance = 2.628495
PYGM_HUMAN F	24856351	PYGM_RABIT Fu	79025 alp	1H5U	1Z8D	2QN8		Sc=6.56796, min distance = 2.056687
PYGM_HUMAN F	24856352	PYGM_RABIT Fu	79025 alp	1H5U	1Z8D	2QN9		Sc=6.5153, min distance = 2.7236910
PYGM_HUMAN F	24860521	PYGM_RABIT Fu	79025 alp	1H5U	1Z8D	2QRM		Sc=6.44957, min distance = 2.587701
PYGM_HUMAN F	24860522	PYGM_RABIT Fu	79025 alp	1H5U	1Z8D	2QRP		Sc=6.43502, min distance = 2.516644
PYGM_HUMAN F	24864075	PYGM_RABIT Fu	79025 alp	1H5U	1Z8D	2QRG		Sc=6.36264, min distance = 2.597335
PYGM_HUMAN F	24864076	PYGM_RABIT Fu	79025 alp	1H5U	1Z8D	2QRH		Sc=6.31878, min distance = 2.620583
PYGM_HUMAN F	2519 caff	PYGL_HUMAN Fu	79025 alp	2ATI	1Z8D	1L5Q		Sc=5.86511, min distance = 2.756822
PYGM_HUMAN F	3758 IBMX	PDE4D_HUMAN F	6083 aden	1TB7	1Z8D	1ZKN		Sc=6.01127, min distance = 2.189304
PYGM_HUMAN F	4369162 1	PYGM_RABIT Fu	79025 alp	1H5U	1Z8D	1HLF		Sc=6.16762, min distance = 2.609298
PYGM_HUMAN F	440115 N-	SFTPD_HUMAN F	79025 alp	1PWB	1Z8D	2ORJ		Sc=6.14954, min distance = 1.805857
PYGM_HUMAN F	444185 1a	PYGM_RABIT Fu	79025 alp	1H5U	1Z8D	1A8I		Sc=6.19693, min distance = 2.525873
PYGM_HUMAN F	445382 2p	PYGL_HUMAN Fu	79025 alp	2ATI	1Z8D	1L5R		Sc=6.10599, min distance = 2.396246
PYGM_HUMAN F	445382 2p	PYGM_RABIT Fu	79025 alp	1H5U	1Z8D	1WW2		Sc=6.09945, min distance = 2.570282
PYGM_HUMAN F	445717 GI	PYGM_RABIT Fu	79025 alp	1H5U	1Z8D	1FTQ		Sc=6.19095, min distance = 2.550221
PYGM_HUMAN F	445718 GI	PYGM_RABIT Fu	79025 alp	1H5U	1Z8D	1FTW		Sc=6.23107, min distance = 2.544330
PYGM_HUMAN F	445720 GI	PYGM_RABIT Fu	79025 alp	1H5U	1Z8D	1FTY		Sc=6.2368, min distance = 2.6191985
PYGM_HUMAN F	445722 1-	PYGM_RABIT Fu	79025 alp	1H5U	1Z8D	1FU7		Sc=6.12768, min distance = 2.516091
PYGM_HUMAN F	445723 1-	PYGM_RABIT Fu	79025 alp	1H5U	1Z8D	1P4H		Sc=6.2668, min distance = 2.0537256
PYGM_HUMAN F	445825 CI	PYGM_RABIT Fu	79025 alp	1H5U	1Z8D	1GG8		Sc=5.99781, min distance = 2.697434
PYGM_HUMAN F	445916 be	XYLA_STRRU Fu	79025 alp	1XIF	1Z8D	1GW9	0.93	Sc=5.60935, min distance = 2.863012

PYGM_HUMAN F	446801 1k	PYGM_RABIT Fu	79025 alp	1H5U	1Z8D	1KTI			Sc=6.27387, min distance = 2.72114
PYGM_HUMAN F	447413 NT	PYGM_RABIT Fu	79025 alp	1H5U	1Z8D	1NOJ			Sc=5.99708, min distance = 2.57327
PYGM_HUMAN F	447607 1c	PYGM_RABIT Fu	79025 alp	1H5U	1Z8D	2G9V			Sc=5.71337, min distance = 2.55234
PYGM_HUMAN F	447770 CG	PYGM_RABIT Fu	79025 alp	1H5U	1Z8D	1P4G			Sc=6.14023, min distance = 2.48775
PYGM_HUMAN F	447771 CI	PYGM_RABIT Fu	79025 alp	1H5U	1Z8D	1P4J			Sc=6.05716, min distance = 2.62906
PYGM_HUMAN F	449343 2g	XYLA_STROL Fu	79025 alp	1S5M	1Z8D	2GYI			Sc=5.73594, min distance = 2.11451
PYGM_HUMAN F	449515 CI	PYGM_RABIT Fu	79025 alp	1H5U	1Z8D	4GPB		0.83	Sc=5.9839, min distance = 2.049301
PYGM_HUMAN F	449554 CI	PYGM_RABIT Fu	79025 alp	1H5U	1Z8D	5GPB			Sc=6.08154, min distance = 2.09167
PYGM_HUMAN F	5287547 6	PURR_ECOLI Fu	190 adeni	2PUB	1Z8D	2PUA		0.9	Sc=5.60768, min distance = 3.24695
PYGM_HUMAN F	5287830 6	Q8RLY5_LACHE	190 adeni	1S2D	1Z8D	1S2I		0.93	Sc=5.63076, min distance = 2.48527
PYGM_HUMAN F	5289590 D	XYLA_STRRU Fu	79025 alp	1XIF	1Z8D	1XII			Sc=5.73619, min distance = 2.17603
PYGM_HUMAN F	5326622 C	PYGL_HUMAN Fu	79025 alp	2ATI	1Z8D	1L5S			Sc=5.90403, min distance = 2.80370
PYGM_HUMAN F	5327121 I	PIM1_HUMAN Fu	6083 aden	1YXU	1Z8D	2C3I			Sc=6.02626, min distance = 2.16080
PYGM_HUMAN F	5780 D-Sc	XYLA_STRRU Fu	79025 alp	1XIF	1Z8D	1XIH		0.78	Sc=5.94468, min distance = 2.09858
PYGM_HUMAN F	6076 Cycl	PDE10_HUMAN F	6083 aden	2OUN	1Z8D	2OUR		0.99	Sc=6.32163, min distance = 2.26293
PYGM_HUMAN F	611002 Op	PIM1_HUMAN Fu	6083 aden	1YXU	1Z8D	1YXX			Sc=6.23486, min distance = 1.84543
PYGM_HUMAN F	64960 Pol	XYLA_STRRU Fu	79025 alp	1XIF	1Z8D	1XIE		0.89	Sc=5.74384, min distance = 2.53099
PYGM_HUMAN F	65533 cor	PYGM_RABIT Fu	79025 alp	1H5U	1Z8D	3GPB		0.87	Sc=6.03187, min distance = 2.12073
PYGM_HUMAN F	656969 1y	PDE4D_HUMAN F	6083 aden	1TB7	1Z8D	1Y2K			Sc=6.17438, min distance = 1.91666
PYGM_HUMAN F	657042 CH	PYGM_RABIT Fu	79025 alp	1H5U	1Z8D	1XKX			Sc=6.34728, min distance = 2.60056
PYGM_HUMAN F	657043 CH	PYGM_RABIT Fu	79025 alp	1H5U	1Z8D	1XL0			Sc=6.18187, min distance = 2.60907
PYGM_HUMAN F	657044 CH	PYGM_RABIT Fu	79025 alp	1H5U	1Z8D	1XL1			Sc=6.2915, min distance = 2.580816
PYGM_HUMAN F	675 Dimed	COBT_SALTY Fu	190 adeni	1JH8	1Z8D	1D0S			Sc=5.67688, min distance = 2.57036
PYGM_HUMAN F	6852179 M	PYGM_RABIT Fu	79025 alp	1H5U	1Z8D	2F3Q			Sc=6.23776, min distance = 2.27537
PYGM_HUMAN F	6852183 N	PYGM_RABIT Fu	79025 alp	1H5U	1Z8D	2F3U			Sc=6.37829, min distance = 2.25899
PYGM_HUMAN F	6914619 C	PYGM_RABIT Fu	79025 alp	1H5U	1Z8D	2FFR			Sc=6.22194, min distance = 2.43414
PYGM_HUMAN F	76635 4,5	COBT_SALTY Fu	190 adeni	1JH8	1Z8D	1L4F			Sc=5.62477, min distance = 2.75355
PYGM_HUMAN F	78598 5-M	COBT_SALTY Fu	190 adeni	1JH8	1Z8D	1JHP			Sc=5.64546, min distance = 2.88303
PYGM_HUMAN F	892 myo-i	SFTPD_HUMAN F	79025 alp	1PWB	1Z8D	2OS9			Sc=5.91587, min distance = 1.87303
PYGM_HUMAN F	95054 Pte	RICI_RICCO Fu	6083 aden	1OBT	1Z8D	1BR6			Sc=5.80162, min distance = 2.31918
PYGM_HUMAN F	95687 Dec	PYGL_HUMAN Fu	79025 alp	2ATI	1Z8D	1XOI			Sc=5.99598, min distance = 2.71600
PYGM_HUMAN F	9578243 E	PDE4B_HUMAN F	6083 aden	1ROR	1Z8D	1XLZ			Sc=6.10033, min distance = 2.17065
PYGM_HUMAN F	96253 7-D	RICI_RICCO Fu	190 adeni	2P8N	1Z8D	1IL3			Sc=5.80142, min distance = 2.95401
PYGM_RABIT F	11957431	BGLA_THEMA Fu	447413 NT	2J7B	1NOJ	2J7D			Sc=6.28284, min distance = 1.99708
PYGM_RABIT F	11957433	BGLA_THEMA Fu	447413 NT	2J7B	1NOJ	2J7F			Sc=6.24547, min distance = 2.02003
PYGM_RABIT F	11957434	BGLA_THEMA Fu	447413 NT	2J7B	1NOJ	2J7G			Sc=6.32499, min distance = 1.83627
PYGM_RABIT F	11957434	BGLA_THEMA Fu	447607 1c	1OIF	2G9V	2J7G			Sc=6.30319, min distance = 1.25370

PYGM_RABIT Fı 11957435	BGLA_THEMA Fu 447413 NI	2J7B	1NOJ	2J7H			Sc=5.68936, min distance = 1.97590
PYGM_RABIT Fı 11957435	BGLA_THEMA Fu 447607 1c	1OIF	2G9V	2J7H			Sc=5.67557, min distance = 2.47409
PYGM_RABIT Fı 11986113	PIM1_HUMAN Fu 6083 aden	1YXU	8GPB	2BZH			Sc=6.44717, min distance = 2.15898
PYGM_RABIT Fı 11986115	PIM1_HUMAN Fu 6083 aden	1YXU	8GPB	2BZJ			Sc=6.2382, min distance = 2.030687
PYGM_RABIT Fı 11987879	BGLA_THEMA Fu 447413 NI	2J7B	1NOJ	2J75			Sc=5.81167, min distance = 1.97423
PYGM_RABIT Fı 124434 Ca	BGLA_THEMA Fu 447607 1c	1OIF	2G9V	2CBV			Sc=5.86672, min distance = 2.03668
PYGM_RABIT Fı 1400 nche	RET_HUMAN Ful 6083 aden	2IVT	8GPB	2IVV			Sc=5.8753, min distance = 2.149772
PYGM_RABIT Fı 2153 thec	Q873X9_ASPFU I 2519 caff	2A3B	1GFZ	2A3A		0.91	Sc=5.92998, min distance = 0.77793
PYGM_RABIT Fı 24905143	SRC_CHICK Ful 6083 aden	3DQX	8GPB	3EN7			Sc=6.2355, min distance = 1.271161
PYGM_RABIT Fı 439217 1m	GLMS_ECOLI Fu 439284 Rc	1MOR	1GPY	1MOQ			Sc=5.91718, min distance = 2.12914
PYGM_RABIT Fı 439236 ri	ALGC_PSEAE Fu 439284 Rc	1P5G	1GPY	2H4L		0.87	Sc=5.63797, min distance = 2.47151
PYGM_RABIT Fı 440102 2h	ALGC_PSEAE Fu 439284 Rc	1P5G	1GPY	2H5A		0.94	Sc=5.7653, min distance = 2.105509
PYGM_RABIT Fı 444345 1c	PIM1_HUMAN Fu 6083 aden	1YXU	8GPB	1YHS			Sc=6.28079, min distance = 2.14295
PYGM_RABIT Fı 445248 nc	MYRA_SINAL Fu 447413 NI	1E6Q	1NOJ	1E6S			Sc=5.95702, min distance = 2.43726
PYGM_RABIT Fı 445257 1i	MYRA_SINAL Fu 447413 NI	1E6Q	1NOJ	1E70			Sc=5.61822, min distance = 2.34599
PYGM_RABIT Fı 445999 CI	Q79G13_MYCTU I 447607 1c	1UP2	2G9V	1UOZ			Sc=5.65049, min distance = 0.65348
PYGM_RABIT Fı 447651 CI	BGLA_THEMA Fu 447413 NI	2J7B	1NOJ	2J77			Sc=5.81359, min distance = 2.07929
PYGM_RABIT Fı 448895 AI	UMPK_YEAST Fu 6083 aden	1UKZ	8GPB	1UKY		0.99	Sc=5.82063, min distance = 2.26707
PYGM_RABIT Fı 448980 nc	BGLA_THEMA Fu 447413 NI	2J7B	1NOJ	1UZ1			Sc=5.80766, min distance = 2.18083
PYGM_RABIT Fı 448980 nc	BGLA_THEMA Fu 447607 1c	1OIF	2G9V	1UZ1		0.79	Sc=5.78241, min distance = 2.68781
PYGM_RABIT Fı 449101 nc	BGLA_THEMA Fu 447413 NI	2J7B	1NOJ	1W3J			Sc=5.61389, min distance = 2.13792
PYGM_RABIT Fı 449101 nc	BGLA_THEMA Fu 447607 1c	1OIF	2G9V	1W3J		0.76	Sc=5.60036, min distance = 2.61070
PYGM_RABIT Fı 449180 XI	XYLA_STRRU Fu 79025 alp	1XIF	1H5U	4XIS			Sc=5.73055, min distance = 2.25529
PYGM_RABIT Fı 449343 2g	XYLA_STROL Fu 79025 alp	1S5M	1H5U	2GYI			Sc=5.72294, min distance = 2.27089
PYGM_RABIT Fı 5326892 C	BGLA_THEMA Fu 447413 NI	2J7B	1NOJ	2J7C			Sc=6.10137, min distance = 2.26441
PYGM_RABIT Fı 6022 Aden	PURP_METJA Fu 6083 aden	2R7M	8GPB	2R7N		0.99	Sc=5.80912, min distance = 2.09940
PYGM_RABIT Fı 611002 Op	PIM1_HUMAN Fu 6083 aden	1YXU	8GPB	1YXX			Sc=5.97495, min distance = 1.53950
PYGM_RABIT Fı 6419789 C	HASP_HUMAN Fu 6083 aden	3DLZ	8GPB	3E7V			Sc=6.0126, min distance = 2.983118
PYGM_RABIT Fı 64689 bet	Q79G13_MYCTU I 447607 1c	1UP2	2G9V	1UP0			Sc=5.82924, min distance = 2.39500
PYGM_RABIT Fı 656964 1y	PDE4D_HUMAN Fı 6083 aden	1TB7	8GPB	1Y2C			Sc=5.91095, min distance = 2.53406
PYGM_RABIT Fı 656966 1y	PDE4D_HUMAN Fı 6083 aden	1TB7	8GPB	1Y2E			Sc=6.03643, min distance = 2.34537
PYGM_RABIT Fı 657135 Op	PIM1_HUMAN Fu 6083 aden	1YXU	8GPB	1YXV			Sc=5.74641, min distance = 3.01569
PYGM_RABIT Fı 7048614 Z	XYLA_STRRU Fu 79025 alp	1XIF	1H5U	1XID			Sc=5.82551, min distance = 2.08802
PYGM_RABIT Fı 81462 Bis	GLCM_HUMAN Fu 447607 1c	2NSX	2G9V	2V3F			Sc=6.02633, min distance = 2.01577
PYGM_RABIT Fı 91532 AMI	PURP_METJA Fu 6083 aden	2R7M	8GPB	2R7K		0.97	Sc=5.96303, min distance = 2.06450
PYGM_RABIT Fı 95259 L-x	XYLA_STRRU Fu 79025 alp	1XIF	1H5U	1XIC			Sc=5.70204, min distance = 2.08158
PYGM_RABIT Fı 9549193 C	BGLA_THEMA Fu 447607 1c	1OIF	2G9V	2CES			Sc=6.0068, min distance = 2.588945

PYLS_METMA Fu	36735 Gpp	Q04230_ECOLX I	33113 gam	1GL7	2Q7E	1GL6		0.8	Sc=6.0289, min distance = 1.6812596
PYLS_METMA Fu	445439 CI	SYT_STAAW Full	5957 Aden	1NYR	2Q7G	1NYQ		0.86	Sc=6.55457, min distance = 1.684022
PYLS_METMA Fu	447377 1r	SYP_RHOPA Full	5957 Aden	2I4O	2Q7G	2I4N		0.84	Sc=6.49493, min distance = 2.354317
PYLS_METMA Fu	447378 1r	SYP_ENTFA Full	5957 Aden	2J3M	2Q7G	2J3L		0.83	Sc=6.54959, min distance = 2.455470
PYLS_METMA Fu	91532 AMP	SYK2_ECOLI Fu	5957 Aden1E24		2Q7G	1E22	28.3	0.99	Sc=6.50347, min distance = 2.439825
PYR5_HUMAN Fu	150959 11	A5KE56_PLAVI I	6030 Urid	2FFC	2V30	2GUU		0.77	Sc=5.84238, min distance = 2.618009
PYR5_HUMAN Fu	445210 9-	KITH_HHV11 Fu	449489 Id	3VTK	2QCH	1E2I			Sc=5.91557, min distance = 0
PYR5_HUMAN Fu	9700 thym	KITH_HHV11 Fu	449489 Id	3VTK	2QCH	1VTK		0.91	Sc=5.87307, min distance = 2.210016
PYRB_ECOLI Fu	444212 tr	ACON_BOVIN Fu	311 citri	1C96	1R0B	1ACO			Sc=5.87857, min distance = 1.328453
PYRB_ECOLI Fu	445607 AT	ACON_BOVIN Fu	311 citri	1C96	1R0B	1FGH			Sc=5.92684, min distance = 2.449714
PYRB_ECOLI Fu	446944 CI	MIF_HUMAN Full	311 citri	1GD0	1R0B	1LJT			Sc=6.20083, min distance = 0.707899
PYRB_ECOLI Fu	447376 2-	ACON_BOVIN Fu	311 citri	1C96	1R0B	1NIS			Sc=5.83567, min distance = 2.554602
PYRB_ECOLI Fu	447550 ph	TPIS_TRYBB Fu	311 citri	2VEK	1R0B	4TIM			Sc=5.66437, min distance = 1.723757
PYRB_ECOLI Fu	449604 NI	ACON_BOVIN Fu	311 citri	1C96	1R0B	8ACN			Sc=5.87827, min distance = 2.053207
PYRB_ECOLI Fu	51 2-Oxop	SERA_ECOLI Fu	311 citri	2P9E	1R0B	1YBA		0.76	Sc=5.83812, min distance = 0.954838
PYRC_ECOHS Fu	16214780	PYRC_ECOLI Fu	439216 L-	2Z26	2Z27	2EG7			Sc=5.99439, min distance = 2.572505
PYRDA_LACLC I	127 4-hyd	PCXA_PSEPU Fu	72 protoc	1YKN	2BSL	3PCG		0.79	Sc=5.81276, min distance = 1.299775
PYRDA_LACLC I	127 4-hyd	PCXB_PSEPU Fu	72 protoc	1YKN	2BSL	3PCG		0.79	Sc=5.81111, min distance = 1.899884
PYRDA_LACLC I	135 p-Hyd	PCXA_PSEPU Fu	72 protoc	1YKN	2BSL	3PCC		0.89	Sc=5.69396, min distance = 2.135258
PYRDA_LACLC I	135 p-Hyd	PHHY_PSEFL Fu	72 protoc	1PHH	2BSL	1PBE		0.89	Sc=5.69586, min distance = 2.193653
PYRDA_LACLC I	160456 3p	PCXA_PSEPU Fu	72 protoc	1YKN	2BSL	3PCF		0.87	Sc=5.72493, min distance = 2.145437
PYRDA_LACLC I	193479 Hi	PCXA_PSEPU Fu	72 protoc	1YKN	2BSL	3PCJ			Sc=5.78503, min distance = 2.036193
PYRDA_LACLC I	3505109 4	PCXA_ACIAD Fu	72 protoc	2BUV	2BSL	2BUU			Sc=5.79566, min distance = 2.187536
PYRDA_LACLC I	3505109 4	PCXB_ACIAD Fu	72 protoc	2BUV	2BSL	2BUU			Sc=5.80345, min distance = 2.177444
PYRDA_LACLC I	446995 FM	CYB2_YEAST Fu	444243 FA	1KBI	1JUB	1LTD		0.77	Sc=6.17114, min distance = 2.096583
PYRDA_LACLC I	448076 CI	CYB2_YEAST Fu	444243 FA	1KBI	1JUB	1SZG		0.76	Sc=6.19415, min distance = 1.656377
PYRDA_LACLC I	7420 3-Hy	PCXA_PSEPU Fu	72 protoc	1YKN	2BSL	3PCB		0.89	Sc=5.69586, min distance = 2.564352
PYRDB_LACLM I	72 protoc	PYRDA_LACLC Fu	967 Oroti	1JQX	1EP2	2BSL	30		Sc=5.80476, min distance = 2.118966
PYRDB_LACLM I	7424 alph	PYRDA_LACLC Fu	967 Oroti	1JQX	1EP2	2BX7	30		Sc=5.79322, min distance = 2.231011
PYRD_ECOLI Fu	15942658	PYRD_HUMAN Fu	444243 FA	1D3G	1F76	2FPY			Sc=6.30948, min distance = 2.106977
PYRD_ECOLI Fu	24794393	PYRD_HUMAN Fu	444243 FA	1D3G	1F76	2PRL			Sc=6.19863, min distance = 1.972974
PYRD_ECOLI Fu	72 protoc	PYRDA_LACLC Fu	967 Oroti	1JQX	1F76	2BSL			Sc=5.80653, min distance = 2.409922
PYRD_ECOLI Fu	7424 alph	PYRDA_LACLC Fu	967 Oroti	1JQX	1F76	2BX7			Sc=5.78911, min distance = 2.539640
PYRD_HUMAN Fu	16741044	PYRDA_LACLC Fu	444243 FA	1JUB	1D3G	1JRB	32.22		Sc=6.20746, min distance = 2.357210
PYRD_HUMAN Fu	446995 FM	CYB2_YEAST Fu	444243 FA	1KBI	1D3G	1LTD		0.77	Sc=6.20107, min distance = 2.346006
PYRD_HUMAN Fu	448076 CI	CYB2_YEAST Fu	444243 FA	1KBI	1D3G	1SZG		0.76	Sc=6.21636, min distance = 1.963074
PYRD_PLAF7 Fu	16741044	PYRDA_LACLC Fu	444243 FA	1JUB	1TV5	1JRB			Sc=6.19869, min distance = 2.546907

PYRD_PLAF7 Fı	448076 CI	CYB2_YEAST Fu	444243 FA	1KBI	1TV5	1SZG		0.76	Sc=6.21027, min distance = 2.20317
PYRD_PLAF7 Fı	72 protoc	PYRDA_LACLC Fı	967 Oroti	1JQX	1TV5	2BSL			Sc=5.8504, min distance = 2.196552
PYRD_PLAF7 Fı	7424 alph	PYRDA_LACLC Fı	967 Oroti	1JQX	1TV5	2BX7			Sc=5.83816, min distance = 2.39390
PYRD_RAT Ful	446995 FM	CYB2_YEAST Fu	444243 FA	1KBI	1UUM	1LTD		0.77	Sc=6.19047, min distance = 2.24545
PYRD_RAT Ful	448076 CI	CYB2_YEAST Fu	444243 FA	1KBI	1UUM	1SZG		0.76	Sc=6.19963, min distance = 2.21923
PYRE_SALTY Fı	160617 Or	PYRE_YEAST Fu	7339 PRPF	2PS1	1LH0	2PRZ			Sc=6.44264, min distance = 2.52903
PYRE_SALTY Fı	7424 alph	PYRDA_LACLC Fı	967 Oroti	1JQX	1LH0	2BX7			Sc=5.82056, min distance = 2.15600
PYRE_SALTY Fı	8582 Inos	Q4DRC4_TRYCR I	7339 PRPF	1I0L	1LH0	1P19			Sc=6.40716, min distance = 1.36602
PYRE_YEAST Fı	445132 1x	PYR5_HUMAN Fu	160617 Or	2QCL	2PRZ	3BGG			Sc=5.86996, min distance = 1.93587
PYRE_YEAST Fı	445132 1x	PYRF_METTH Fu	160617 Or	1KM6	2PRZ	1X1Z			Sc=5.87627, min distance = 1.93595
PYRE_YEAST Fı	72 protoc	PYRDA_LACLC Fı	967 Oroti	1JQX	2PS1	2BSL			Sc=5.8551, min distance = 2.098176
PYRE_YEAST Fı	7424 alph	PYRDA_LACLC Fı	967 Oroti	1JQX	2PS1	2BX7			Sc=5.81357, min distance = 2.10612
PYRF_BACSU Fı	150856 5-	PYR5_HUMAN Fu	6030 Urid	2V30	1DBT	2QCF		0.94	Sc=6.24529, min distance = 2.54321
PYRF_BACSU Fı	150959 11	A5KE56_PLAVI I	6030 Urid	2FFC	1DBT	2GUU		0.77	Sc=6.20823, min distance = 2.51318
PYRF_BACSU Fı	150973 5-	PYR5_HUMAN Fu	6030 Urid	2V30	1DBT	2QCG		0.91	Sc=6.25303, min distance = 2.27198
PYRF_BACSU Fı	160617 Or	PYR5_HUMAN Fu	6030 Urid	2V30	1DBT	2QCL		0.86	Sc=6.38455, min distance = 2.27479
PYRF_BACSU Fı	17749721	PYR5_HUMAN Fu	6030 Urid	2V30	1DBT	2QCM		0.88	Sc=6.3106, min distance = 2.512440
PYRF_BACSU Fı	445132 1x	PYR5_HUMAN Fu	6030 Urid	2V30	1DBT	3BGG		0.81	Sc=6.26683, min distance = 2.40477
PYRF_BACSU Fı	449489 Id	PYR5_HUMAN Fu	6030 Urid	2V30	1DBT	2QCH		0.9	Sc=6.21355, min distance = 2.15451
PYRF_BACSU Fı	6029 uric	GLMU_HAEIN Fu	6030 Urid	2V0J	1DBT	2V0L		0.92	Sc=6.0225, min distance = 2.068084
PYRF_BACSU Fı	65063 dUM	PYR5_HUMAN Fu	6030 Urid	2V30	1DBT	2QCH		0.97	Sc=6.19531, min distance = 2.01233
PYRF_ECOLI Fı	150856 5-	PYR5_HUMAN Fu	445132 1x	3BGG	1EIX	2QCF		0.81	Sc=6.24174, min distance = 2.51416
PYRF_ECOLI Fı	150973 5-	PYR5_HUMAN Fu	445132 1x	3BGG	1EIX	2QCG		0.78	Sc=6.32623, min distance = 1.15962
PYRF_ECOLI Fı	160617 Or	PYR5_HUMAN Fu	445132 1x	3BGG	1EIX	2QCL			Sc=6.42454, min distance = 1.37570
PYRF_ECOLI Fı	17749721	PYR5_HUMAN Fu	445132 1x	3BGG	1EIX	2QCM		0.78	Sc=6.30589, min distance = 2.17101
PYRF_ECOLI Fı	6030 Urid	PYR5_HUMAN Fu	445132 1x	3BGG	1EIX	2V30		0.81	Sc=6.29542, min distance = 1.51515
PYRF_ECOLI Fı	65063 dUM	PYR5_HUMAN Fu	445132 1x	3BGG	1EIX	2QCH		0.78	Sc=6.26898, min distance = 1.23166
PYRF_GEOKA Fı	445132 1x	PYRF_METTH Fu	6131 cyti	1LP6	2YYU	1X1Z			Sc=6.3385, min distance = 1.359506
PYRF_METTH Fı	150856 5-	PYR5_HUMAN Fu	445132 1x	3BGG	1X1Z	2QCF		0.81	Sc=6.22683, min distance = 2.24539
PYRF_METTH Fı	150973 5-	PYR5_HUMAN Fu	160617 Or	2QCL	1KM6	2QCG		0.8	Sc=6.19818, min distance = 1.97840
PYRF_METTH Fı	150973 5-	PYR5_HUMAN Fu	445132 1x	3BGG	1X1Z	2QCG		0.78	Sc=6.28244, min distance = 2.11245
PYRF_METTH Fı	17749721	PYR5_HUMAN Fu	160617 Or	2QCL	1KM6	2QCM		0.93	Sc=6.25006, min distance = 2.18267
PYRF_METTH Fı	17749721	PYR5_HUMAN Fu	445132 1x	3BGG	1X1Z	2QCM		0.78	Sc=6.29108, min distance = 2.28129
PYRF_METTH Fı	449489 Id	PYR5_HUMAN Fu	160617 Or	2QCL	1KM6	2QCH		0.79	Sc=6.15429, min distance = 2.14989
PYRF_METTH Fı	449489 Id	PYR5_HUMAN Fu	445132 1x	3BGG	1X1Z	2QCH		0.77	Sc=6.2325, min distance = 1.957439
PYRF_METTH Fı	65063 dUM	PYR5_HUMAN Fu	160617 Or	2QCL	1KM6	2QCH		0.83	Sc=6.1468, min distance = 2.294111
PYRF_METTH Fı	65063 dUM	PYR5_HUMAN Fu	445132 1x	3BGG	1X1Z	2QCH		0.78	Sc=5.839, min distance = 2.4218509

PYRF_PYRHO Fı	150856 5-	PYR5_HUMAN Fu	6030 Urid	2V30	2CZE	2QCF		0.94	Sc=6.25402, min distance = 2.59548
PYRF_PYRHO Fı	150959 1l	A5KE56_PLAVI]	6030 Urid	2FFC	2CZE	2GUU		0.77	Sc=5.85011, min distance = 2.59585
PYRF_PYRHO Fı	150973 5-	PYR5_HUMAN Fu	6030 Urid	2V30	2CZE	2QCG		0.91	Sc=6.3377, min distance = 0.796070
PYRF_PYRHO Fı	160617 Or	PYR5_HUMAN Fu	6030 Urid	2V30	2CZE	2QCL		0.86	Sc=6.04049, min distance = 2.53959
PYRF_PYRHO Fı	17749721	PYR5_HUMAN Fu	6030 Urid	2V30	2CZE	2QCM		0.88	Sc=5.96997, min distance = 2.61326
PYRF_PYRHO Fı	3713 Indo	TRPA_THET2 Fu	311 citri	1UJP	2CZ5	1WXJ			Sc=6.01085, min distance = 2.19052
PYRF_PYRHO Fı	39981 Spa	PYRB_ECOLI Fu	311 citri	1R0B	2CZ5	1EKX			Sc=6.08301, min distance = 0.60171
PYRF_PYRHO Fı	439459 Hc	NIFD_KLEPN Fu	311 citri	1H1L	2CZ5	1QGU		0.92	Sc=5.90081, min distance = 2.23028
PYRF_PYRHO Fı	444212 tr	ACON_BOVIN Fu	311 citri	1C96	2CZ5	1ACO			Sc=5.753, min distance = 2.2655811
PYRF_PYRHO Fı	445132 1x	PYR5_HUMAN Fu	6030 Urid	2V30	2CZE	3BGG		0.81	Sc=6.27435, min distance = 2.29678
PYRF_PYRHO Fı	445607 AT	ACON_BOVIN Fu	311 citri	1C96	2CZ5	1FGH			Sc=5.82301, min distance = 2.10316
PYRF_PYRHO Fı	445905 1g	AROQ_HELPY Fu	311 citri	2C4V	2CZ5	2C57			Sc=5.82175, min distance = 1.02534
PYRF_PYRHO Fı	51 2-Oxop	SERA_ECOLI Fu	311 citri	2P9E	2CZ5	1YBA		0.76	Sc=5.61496, min distance = 1.94606
PYRF_PYRHO Fı	6031 Uric	PYRH_ECOLI Fu	6030 Urid	2BNE	2CZE	2BND		0.99	Sc=6.35095, min distance = 2.04898
PYRF_PYRHO Fı	6102777 2	AROQ_HELPY Fu	311 citri	2C4V	2CZ5	2C4W			Sc=6.19496, min distance = 2.11047
PYRF_PYRHO Fı	65063 dUM	PYR5_HUMAN Fu	6030 Urid	2V30	2CZE	2QCH		0.97	Sc=5.86248, min distance = 2.38092
PYRF_PYRHO Fı	8200 TETR	PPNK1_LISMO Fı	311 citri	2I29	2CZ5	2I2C			Sc=5.6162, min distance = 2.134973
PYRF_YEAST Fı	150856 5-	PYR5_HUMAN Fu	445132 1x	3BGG	1DQX	2QCF		0.81	Sc=6.25303, min distance = 2.55999
PYRF_YEAST Fı	150973 5-	PYR5_HUMAN Fu	445132 1x	3BGG	1DQX	2QCG		0.78	Sc=6.29956, min distance = 1.10555
PYRF_YEAST Fı	160617 Or	PYR5_HUMAN Fu	445132 1x	3BGG	1DQX	2QCL			Sc=6.41806, min distance = 1.30695
PYRF_YEAST Fı	17749721	PYR5_HUMAN Fu	445132 1x	3BGG	1DQX	2QCM		0.78	Sc=6.31685, min distance = 2.40988
PYRF_YEAST Fı	449489 Ic	PYR5_HUMAN Fu	445132 1x	3BGG	1DQX	2QCH		0.77	Sc=6.24547, min distance = 1.41752
PYRF_YEAST Fı	6030 Uric	PYR5_HUMAN Fu	445132 1x	3BGG	1DQX	2V30		0.81	Sc=6.28662, min distance = 1.55531
PYRF_YEAST Fı	65063 dUM	PYR5_HUMAN Fu	445132 1x	3BGG	1DQX	2QCH		0.78	Sc=6.23357, min distance = 1.29479
PYRG_ECOLI Fı	447821 CI	CDK2_HUMAN Fu	6022 Aden	1GY3	2AD5	1PF8			Sc=6.06861, min distance = 2.37985
PYRG_ECOLI Fı	5957 Ader	BIOD_ECOLI Fu	6022 Aden	1DAD	2AD5	1A82		0.99	Sc=5.98423, min distance = 2.29152
PYRG_ECOLI Fı	5957 Ader	KTHY_HUMAN Fu	6022 Aden	1NN3	2AD5	1E2Q		0.99	Sc=5.96741, min distance = 2.21330
PYRG_ECOLI Fı	91532 AMI	BIOD_ECOLI Fu	6022 Aden	1DAD	2AD5	1DAG		0.99	Sc=5.99892, min distance = 2.79410
PYRH_ECOLI Fı	150959 1l	A5KE56_PLAVI]	6030 Urid	2FFC	2BNE	2GUU		0.77	Sc=6.30037, min distance = 2.24586
PYRH_ECOLI Fı	17749721	PYR5_HUMAN Fu	6030 Urid	2V30	2BNE	2QCM		0.88	Sc=6.40362, min distance = 2.09971
PYRH_ECOLI Fı	449489 Ic	PYR5_HUMAN Fu	6030 Urid	2V30	2BNE	2QCH		0.9	Sc=6.30386, min distance = 1.87987
PYRH_ECOLI Fı	6029 uric	GLMU_HAEIN Fu	6030 Urid	2V0J	2BNE	2V0L		0.92	Sc=6.16096, min distance = 2.14880
PYRH_ECOLI Fı	65063 dUM	PYR5_HUMAN Fu	6030 Urid	2V30	2BNE	2QCH		0.97	Sc=6.28376, min distance = 1.97866
PYRH_PYRFU Fı	150959 1l	A5KE56_PLAVI]	6030 Urid	2FFC	2BMU	2GUU		0.77	Sc=6.29315, min distance = 2.22582
PYRH_PYRFU Fı	150973 5-	PYR5_HUMAN Fu	6030 Urid	2V30	2BMU	2QCG		0.91	Sc=6.33968, min distance = 2.05778
PYRH_PYRFU Fı	188966 dA	HSLU_ECOLI Fu	33113 gam	1E94	2BMU	1G4A		0.97	Sc=6.4092, min distance = 1.822797
PYRH_PYRFU Fı	449489 Ic	PYR5_HUMAN Fu	6030 Urid	2V30	2BMU	2QCH		0.9	Sc=6.3031, min distance = 1.889567

PYRH_PYRFU Fı	6029 uric	GLMU_HAEIN Fu:	6030 Urid	2V0J	2BMU	2V0L		0.92	Sc=6.16429, min distance = 1.88211{
PYRH_PYRFU Fı	6031 Uric	O87988_BORBR]	6030 Urid	2PZL	2BMU	2PZM		0.99	Sc=6.39838, min distance = 1.81626:
PYRH_PYRFU Fı	6031 Uric	PYRH_ECOLI Fu:	6030 Urid	2BNE	2BMU	2BND		0.99	Sc=6.40184, min distance = 2.49976'
PYRH_PYRFU Fı	60961 ade	SKY1_YEAST Fu:	33113 gam	1Q99	2BMU	1Q97		0.93	Sc=6.24461, min distance = 2.25308:
PYRH_PYRFU Fı	65063 dUM	PYR5_HUMAN Fu:	6030 Urid	2V30	2BMU	2QCH		0.97	Sc=6.27772, min distance = 2.33443'
PYRH_SULSO Fı	150973 5-	PYR5_HUMAN Fu:	6030 Urid	2V30	2J4J	2QCG		0.91	Sc=6.30887, min distance = 1.93985{
PYRH_SULSO Fı	17749721	PYR5_HUMAN Fu:	6030 Urid	2V30	2J4J	2QCM		0.88	Sc=6.37101, min distance = 2.05255(
PYRH_SULSO Fı	18068 Udp	GAL7_ECOLI Fu:	6030 Urid	1HXP	2J4J	1GUP		0.88	Sc=5.87658, min distance = 1.65502(
PYRH_SULSO Fı	33113 gam	IGF1R_HUMAN Fı	91532 AMP	1K3A	2J4J	1JQH		0.99	Sc=5.97627, min distance = 1.49059!
PYRH_SULSO Fı	33113 gam	KIF1A_MOUSE Fı	91532 AMP	1I6I	2J4J	1VFV		0.99	Sc=6.47174, min distance = 1.18068(
PYRH_SULSO Fı	33113 gam	PURT_ECOLI Fu:	91532 AMP	1KJI	2J4J	1EYZ		0.99	Sc=6.48998, min distance = 1.73230{
PYRH_SULSO Fı	444564 AI	BIOD_ECOLI Fu:	91532 AMP	1DAG	2J4J	1BS1		0.91	Sc=6.42696, min distance = 1.21627:
PYRH_SULSO Fı	445132 1x	PYR5_HUMAN Fu:	6030 Urid	2V30	2J4J	3BGG		0.81	Sc=6.32606, min distance = 2.07304:
PYRH_SULSO Fı	446048 AM	PGK1_PIG Full:	91532 AMP	1KF0	2J4J	1HDI		0.97	Sc=6.34357, min distance = 2.29801(
PYRH_SULSO Fı	5957 Aden	BIOD_ECOLI Fu:	91532 AMP	1DAG	2J4J	1A82		0.99	Sc=6.48195, min distance = 1.39746!
PYRH_SULSO Fı	5957 Aden	NIFH1_AZOVI Fı	91532 AMP	2AFK	2J4J	2C8V		0.99	Sc=6.48623, min distance = 1.83345!
PYRH_SULSO Fı	6022 Aden	BIOD_ECOLI Fu:	91532 AMP	1DAG	2J4J	1DAD		0.99	Sc=6.42175, min distance = 2.23693{
PYRH_SULSO Fı	6031 Uric	O87988_BORBR]	6030 Urid	2PZL	2J4J	2PZM		0.99	Sc=5.90525, min distance = 2.16839:
PYRH_SULSO Fı	65063 dUM	PYR5_HUMAN Fu:	6030 Urid	2V30	2J4J	2QCH		0.97	Sc=6.24912, min distance = 2.21719!
PYRI_ECOLI Fı	11608401	KAPCA_BOVIN Fı	5957 Aden	1Q24	7AT1	2UVY			Sc=6.14453, min distance = 2.33250(
PYRI_ECOLI Fı	6132 Cyti	KPSU5_ECOLX Fı	6176 Cyti	1H7G	2FZC	1H7H		0.99	Sc=5.6442, min distance = 2.0703038
PYRI_METJA Fı	11608401	KAPCA_BOVIN Fı	5957 Aden	1Q24	2YWW	2UVY			Sc=6.16054, min distance = 2.01988'
PYRI_METJA Fı	3540 1yds	KAPCA_BOVIN Fı	5957 Aden	1Q24	2YWW	1YDS			Sc=5.84248, min distance = 1.59945:
PYRI_METJA Fı	6176 Cyti	PYRI_ECOLI Fu:	5957 Aden	7AT1	2YWW	2FZC			Sc=5.65955, min distance = 1.90507!
PYRR_BACCL Fı	150856 5-	PYR5_HUMAN Fu:	6030 Urid	2V30	2IGB	2QCF		0.94	Sc=6.27197, min distance = 2.09296:
PYRR_BACCL Fı	150959 1l	A5KE56_PLAVI]	6030 Urid	2FFC	2IGB	2GUU		0.77	Sc=6.2582, min distance = 2.102293!
PYRR_BACCL Fı	17749721	PYR5_HUMAN Fu:	6030 Urid	2V30	2IGB	2QCM		0.88	Sc=6.33538, min distance = 2.13421:
PYRR_BACCL Fı	445132 1x	PYR5_HUMAN Fu:	6030 Urid	2V30	2IGB	3BGG		0.81	Sc=5.83382, min distance = 2.19722!
PYRR_BACCL Fı	6029 uric	GLMU_HAEIN Fu:	6030 Urid	2V0J	2IGB	2V0L		0.92	Sc=6.12232, min distance = 2.14212(
PYRR_BACCL Fı	65063 dUM	PYR5_HUMAN Fu:	6030 Urid	2V30	2IGB	2QCH		0.97	Sc=6.25808, min distance = 2.32546!
Q02SD6_PSEAB	11957385	MYG_PHYCA Ful:	444098 HE	1A6M	1TU9	2CMM			Sc=6.18658, min distance = 2.25418{
Q04230_ECOLX	11957417	CDK2_HUMAN Fu:	6022 Aden	1GY3	1GKI	2I40			Sc=6.20381, min distance = 2.23715(
Q04230_ECOLX	1540 1h1q	CDK2_HUMAN Fu:	6022 Aden	1GY3	1GKI	1H1Q			Sc=5.96812, min distance = 1.55750(
Q04230_ECOLX	160355 rc	CDK2_HUMAN Fu:	6022 Aden	1GY3	1GKI	3DDQ			Sc=5.85174, min distance = 2.07594!
Q04230_ECOLX	23727982	CDK2_HUMAN Fu:	6022 Aden	1GY3	1GKI	3BHU			Sc=6.05241, min distance = 2.26290:
Q04230_ECOLX	24756824	CSK2A_MAIZE Fı	36735 Gpp	1DAY	1GL6	3BE9			Sc=6.17401, min distance = 2.01129:
Q04230_ECOLX	4369136 C	CDK2_HUMAN Fu:	6022 Aden	1GY3	1GKI	1DM2			Sc=5.86032, min distance = 2.72295(

Q04230_ECOLX	445940 O6	CDK2_HUMAN Fu	6022 Aden	1GY3	1GKI	1GZ8		Sc=5.87388, min distance = 1.85783
Q04230_ECOLX	4565 1h1r	CDK2_HUMAN Fu	6022 Aden	1GY3	1GKI	1H1R		Sc=5.96571, min distance = 1.79035
Q04230_ECOLX	611002 Op	PIM1_HUMAN Fu	33113 gam	1XR1	1GL7	1YXX		Sc=5.77021, min distance = 1.869128
Q04230_ECOLX	6918710 5	CDK2_HUMAN Fu	6022 Aden	1GY3	1GKI	3EJ1		Sc=5.95319, min distance = 1.874859
Q04230_ECOLX	72194 2-C	ENPL_CANFA Fu	6022 Aden	1TC6	1GKI	1QYE	0.89	Sc=5.91939, min distance = 2.028514
Q04230_ECOLX	854023 Ep	Q8WSF8_APLCA	23831 HEP	2BR7	1GL6	2BYQ		Sc=5.73951, min distance = 2.189320
Q04230_ECOLX	8977 1dar	NDK_PYRHO Ful	6022 Aden	2DYA	1GKI	2DXE	0.8	Sc=6.30493, min distance = 2.197024
Q04416_9MICC	72 protoc	PCXB_PSEPU Fu	135 p-Hyd	3PCC	1Q4S	1YKN	0.89	Sc=5.71884, min distance = 2.32285
Q05KQ5_COMTE	127 4-hyd	PCXB_PSEPU Fu	7420 3-Hy	3PCB	2DKH	3PCG	0.86	Sc=5.96969, min distance = 0.618654
Q05KQ5_COMTE	16741253	LSD1_HUMAN Fu	444188 CI	2DW4	2DKH	2UXN	0.95	Sc=6.87737, min distance = 2.040739
Q068W2_SERMA	104904 Me	IGKC_MOUSE Fu	24139 ace	3BZ4	1EIB	2AJZ		Sc=5.87386, min distance = 0.76671
Q068W2_SERMA	119404 Ma	Q54276_SERMA	24139 ace	1UR9	1EIB	1W1Y		Sc=6.18811, min distance = 1.00551
Q068W2_SERMA	126154 Cy	Q54276_SERMA	24139 ace	1UR9	1EIB	1W1P		Sc=5.83482, min distance = 0.85155
Q068W2_SERMA	16058615	LYSC_CHICK Fu	24139 ace	1LZB	1EIB	2H9J		Sc=6.0335, min distance = 1.6249000
Q068W2_SERMA	185698 al	CONA_CANEN Fu	24139 ace	1TEI	1EIB	1CVN		Sc=6.05074, min distance = 1.159964
Q068W2_SERMA	4369268 C	F17AG_ECOLX Fu	24139 ace	2BSC	1EIB	1O9V	0.88	Sc=5.94579, min distance = 2.70293
Q068W2_SERMA	439353 be	LYSC_CHICK Fu	24139 ace	1LZB	1EIB	1UC0		Sc=5.69574, min distance = 2.315298
Q068W2_SERMA	439680 be	LYSC_HUMAN Fu	24139 ace	1LZR	1EIB	1REM		Sc=5.89869, min distance = 0.978112
Q068W2_SERMA	444205 CI	DPP4_HUMAN Fu	24139 ace	2RGU	1EIB	3BJM	0.91	Sc=5.89269, min distance = 2.855478
Q068W2_SERMA	445595 CI	CHIA_SERMA Fu	24139 ace	1K9T	1EIB	1FFQ		Sc=6.01457, min distance = 2.26168
Q068W2_SERMA	449094 Cy	Q54276_SERMA	24139 ace	1UR9	1EIB	1W1T		Sc=6.19798, min distance = 2.11390
Q068W2_SERMA	449095 1w	Q54276_SERMA	24139 ace	1UR9	1EIB	1W1V		Sc=6.24607, min distance = 2.204914
Q06A48_STRPY	11957385	MYG_PHYCA Ful	444098 HE	1A6M	2Q7A	2CMM		Sc=6.06387, min distance = 2.237365
Q07387_SIVCZ	392622 ri	POL_HV1BR Ful	444196 1a	1AAQ	1SIV	1RL8		Sc=5.972, min distance = 1.0350173
Q07387_SIVCZ	448921 CI	POL_HV1BR Ful	444196 1a	1AAQ	1SIV	1UPJ		Sc=5.92304, min distance = 2.334268
Q09LX0_BACST	8172 Trig	LMBL1_HUMAN Fu	78165 MES	1OZ2	2EXH	2RI5		Sc=5.72691, min distance = 1.11567
Q0PB48_CAMJE	11957355	CY1_BOVIN Ful	444098 HE	1L0N	2IG3	1L0L	0.77	Sc=6.64365, min distance = 2.102422
Q0PB48_CAMJE	11957385	MYG_PHYCA Ful	444098 HE	1A6M	2IG3	2CMM		Sc=6.08163, min distance = 2.04043
Q0PBL7_CAMJE	11957399	PYRB_ECOLI Fu	311 citri	1R0B	2HXW	2H3E		Sc=6.14025, min distance = 0.881858
Q0PBL7_CAMJE	126892 1t	TPIS_TRYBB Fu	311 citri	2VEK	2HXW	1TSI		Sc=5.74838, min distance = 1.57920
Q0PBL7_CAMJE	439183 1i	TPIS_TRYBB Fu	311 citri	2VEK	2HXW	1IIH		Sc=5.70644, min distance = 1.662088
Q0PBL7_CAMJE	439276 3-	TPIS_TRYBB Fu	311 citri	2VEK	2HXW	6TIM		Sc=5.61353, min distance = 1.660492
Q0PBL7_CAMJE	444212 tr	ACON_BOVIN Fu	311 citri	1C96	2HXW	1ACO		Sc=5.91587, min distance = 2.838068
Q0PBL7_CAMJE	445496 TE	A2NHM3_MOUSE	311 citri	1ZEA	2HXW	1F3D		Sc=6.096, min distance = 0.5401194
Q0PBL7_CAMJE	445607 AT	ACON_BOVIN Fu	311 citri	1C96	2HXW	1FGH		Sc=5.98364, min distance = 2.644488
Q0PBL7_CAMJE	447376 2-	ACON_BOVIN Fu	311 citri	1C96	2HXW	1NIS		Sc=5.89614, min distance = 2.663938
Q0PBL7_CAMJE	447550 ph	TPIS_TRYBB Fu	311 citri	2VEK	2HXW	4TIM		Sc=5.70106, min distance = 1.879959

Q0PBL7_CAMJE	449604 N	ACON_BOVIN Fu	311 citri	1C96	2HXW	8ACN		Sc=5.89885, min distance = 2.73914
Q0PBL7_CAMJE	4797 1tp	TPIS_TRYBB Fu	311 citri	2VEK	2HXW	1TTJ		Sc=5.60113, min distance = 2.32506
Q11S56_CYTH3	378 4-nit	PPAL_YEAST Fu	23831 HE	1D1P	2HX1	1D1Q		Sc=5.73102, min distance = 2.53889
Q11S56_CYTH3	445939 1	PPTA_ECOLI Fu	23831 HE	1GYX	2HX1	1GYY		Sc=5.78844, min distance = 1.43159
Q11S56_CYTH3	854023 E	Q8WSF8_APLCA	23831 HE	2BR7	2HX1	2BYQ		Sc=5.76758, min distance = 0.87861
Q17091_ASCSU	11957385	MYG_PHYCA Ful	444098 HE	1A6M	1X3X	2CMM		Sc=6.36729, min distance = 1.95617
Q18664_CAEEL	11987635	ADH_DROLE Ful	5893 nadi	1SBY	1VKO	1B2L	0.76	Sc=6.87614, min distance = 2.02624
Q1CU05_HELPH	5957 Ade	MYS2_DICDI Fu	440317 AT	1MMG	1NLY	1FMW	0.99	Sc=6.46533, min distance = 0
Q1CU05_HELPH	6083 ade	SRC_CHICK Ful	440317 AT	3DQW	1NLY	3DQX	0.97	Sc=6.3071, min distance = 1.765023
Q1JBR0_STRPB	11987634	GLPD_ECOLI Fu	444188 CI	2QCU	2BCP	2R4E	0.97	Sc=6.41641, min distance = 2.14786
Q1JBR0_STRPB	444502 C	TYTR_TRYCR Fu	444188 CI	1BZL	2BCP	1GXF	0.98	Sc=6.42418, min distance = 1.67788
Q1JBR0_STRPB	445794 A	PHHY_PSEFL Fu	444188 CI	1PBE	2BCP	2PHH		Sc=6.65619, min distance = 2.05899
Q1JBR0_STRPB	446013 1,	O28603_ARCFU	444188 CI	1JNR	2BCP	1JNZ	0.94	Sc=6.42555, min distance = 1.92801
Q1JBR0_STRPB	446013 1,	O28604_ARCFU	444188 CI	1JNR	2BCP	1JNZ	0.94	Sc=6.42584, min distance = 1.87716
Q1JLP2_STRPC	444502 C	TYTR_TRYCR Fu	444188 CI	1BZL	2BC0	1GXF	0.98	Sc=6.13778, min distance = 1.92761
Q1JLP2_STRPC	445794 A	PHHY_PSEFL Fu	444188 CI	1PBE	2BC0	2PHH		Sc=6.6635, min distance = 2.056560
Q1JLP2_STRPC	446013 1,	O28603_ARCFU	444188 CI	1JNR	2BC0	1JNZ	0.94	Sc=6.13797, min distance = 1.99166
Q24973_GIALA	11287125	TGT_ZYMMO Ful	100684 9-	1Q2R	1DQP	2BBF		Sc=6.13196, min distance = 1.98119
Q24973_GIALA	171 preQ	TGT_ZYMMO Ful	100684 9-	1Q2R	1DQP	3BLD		Sc=5.92497, min distance = 0.88291
Q24973_GIALA	190 aden	RICI_RICCO Fu	100684 9-	1IL4	1DQP	2P8N		Sc=5.60499, min distance = 2.90744
Q24973_GIALA	242831 2-	TGT_ZYMMO Ful	100684 9-	1Q2R	1DQP	1S39		Sc=5.89095, min distance = 2.28976
Q24973_GIALA	24741806	TGT_ZYMMO Ful	100684 9-	1Q2R	1DQP	2Z7K		Sc=6.1976, min distance = 1.838631
Q24973_GIALA	445390 C	TGT_ZYMMO Ful	100684 9-	1Q2R	1DQP	1ENU		Sc=6.0111, min distance = 1.869360
Q24973_GIALA	445497 C	TGT_ZYMMO Ful	100684 9-	1Q2R	1DQP	1F3E		Sc=6.03462, min distance = 1.52741
Q24973_GIALA	447262 C	TGT_ZYMMO Ful	100684 9-	1Q2R	1DQP	2Z1W		Sc=6.08083, min distance = 1.81238
Q24973_GIALA	448010 1	TGT_ZYMMO Ful	100684 9-	1Q2R	1DQP	1R5Y		Sc=6.002, min distance = 1.8549388
Q24973_GIALA	448488 1s	TGT_ZYMMO Ful	100684 9-	1Q2R	1DQP	1S38		Sc=5.93635, min distance = 2.47424
Q24973_GIALA	6804 gua	HPRT_HUMAN Fu	444699 1	1BZY	1DQN	1HMP		Sc=6.00568, min distance = 1.96454
Q24973_GIALA	764 guan	TGT_ZYMMO Ful	100684 9-	1Q2R	1DQP	2PWU		Sc=5.7284, min distance = 2.593694
Q24973_GIALA	96253 7-	RICI_RICCO Fu	100684 9-	1IL4	1DQP	1IL3		Sc=5.80142, min distance = 2.54669
Q25BW5_PHACH	445257 1i	MYRA_SINAL Fu	7027 gluc	1E6X	2E40	1E70		Sc=5.71012, min distance = 2.15636
Q25BW5_PHACH	447413 N	MYRA_SINAL Fu	7027 gluc	1E6X	2E40	1E6Q		Sc=6.00403, min distance = 2.19223
Q26000_PLAFA	446248 C	RAN_HUMAN Ful	8977 1dar	3CH5	1D5C	1IBR		Sc=6.54904, min distance = 2.02456
Q26000_PLAFA	6804 gua	EF1A_YEAST Fu	8977 1dar	2B7B	1D5C	1G7C	0.99	Sc=6.42792, min distance = 2.62988
Q26966_TRYCR	123619 E	TRFL_BOVIN Fu	84571 lac	2H4I	1MS0	3CFL		Sc=5.90912, min distance = 2.59849
Q26966_TRYCR	439353 b	LECG_ARAHY Fu	6134 lact	2PEL	1MS9	1V6I	0.87	Sc=5.6153, min distance = 2.019240
Q26966_TRYCR	439353 b	LECG_ARAHY Fu	84571 lac	2PEL	1MS0	1V6I	0.87	Sc=5.63826, min distance = 2.39903

Q26966_TRYCR	439353 be	LEG1_HUMAN Fu	6134 lact	1W6O	1MS9	1GZW	0.87	Sc=5.62042, min distance = 1.71570
Q26966_TRYCR	439353 be	LEG2_CONMY Fu	6134 lact	1IS3	1MS9	1WLD	0.87	Sc=5.62287, min distance = 2.12062
Q27198_TETTH	164795 N(	RBCMT_PEA Ful	23831 HEP	1MLV	1QST	2H2J		Sc=5.69139, min distance = 2.12264
Q27198_TETTH	449233 CI	O53831_MYCTU I	87642 coe	1P0H	1QSN	2C27		Sc=5.93239, min distance = 2.27225
Q27198_TETTH	82313 1ro	LYSC_CHICK Fu	23831 HEP	2HUB	1QST	1LSZ		Sc=5.886, min distance = 2.4102817
Q27679_LEIDO	2519 caff	PYGM_RABIT Fu	6083 aden	8GPB	1QB8	1GFZ		Sc=5.79875, min distance = 2.29007
Q27679_LEIDO	6102712 C	PYRB_ECOLI Fu	311 citri	1R0B	1QB7	2AIR		Sc=5.77572, min distance = 1.21833
Q27679_LEIDO	611002 Op	PIM1_HUMAN Fu	6083 aden	1YXU	1QB8	1YXX		Sc=6.10454, min distance = 1.20751
Q27679_LEIDO	656964 1y	PDE4D_HUMAN Fu	6083 aden	1TB7	1QB8	1Y2C		Sc=5.90171, min distance = 2.58238
Q27679_LEIDO	656966 1y	PDE4D_HUMAN Fu	6083 aden	1TB7	1QB8	1Y2E		Sc=6.01978, min distance = 2.43462
Q27679_LEIDO	656968 1y	PDE4B_HUMAN Fu	6083 aden	1ROR	1QB8	1Y2H		Sc=5.92628, min distance = 2.03890
Q27679_LEIDO	6804 guar	PDE10_HUMAN Fu	6083 aden	2OUN	1QB8	2OUQ	0.8	Sc=6.38867, min distance = 0
Q27679_LEIDO	6804 guar	PUR1_ECOLI Fu	6083 aden	1ECJ	1QB8	1ECB	0.8	Sc=6.36004, min distance = 2.06153
Q27679_LEIDO	6961 PYRO	FUMC_ECOLI Fu	311 citri	1FUO	1QB7	1FUP		Sc=5.68112, min distance = 2.61484
Q27679_LEIDO	8582 Inos	PURA1_MOUSE Fu	6083 aden	1MF0	1QB8	1IWE	0.79	Sc=5.99424, min distance = 2.25971
Q27679_LEIDO	8582 Inos	PYGM_RABIT Fu	6083 aden	8GPB	1QB8	2QN7	0.79	Sc=6.00721, min distance = 2.10722
Q28KC0_JANSC	8158 Pela	ALBU_HUMAN Fu	311 citri	1TF0	3BB5	1E7E		Sc=5.80318, min distance = 1.17300
Q28VU1_JANSC	5326566 l	BLVRB_HUMAN Fu	444243 FA	1HE4	2OU5	1HE5	0.82	Sc=6.13555, min distance = 2.56758
Q28VU1_JANSC	5326566 l	Q9WZW1_THEMA I	444243 FA	1T6Y	2OU5	1S4M	0.82	Sc=6.17894, min distance = 1.97312
Q29U70_MEDTR	188380 Ac	MCES_ENCCU Fu	439155 Ad	1RI1	1ZGJ	1Z3C	0.91	Sc=6.18328, min distance = 2.18711
Q29U70_MEDTR	446535 CI	HNMT_HUMAN Fu	439155 Ad	2AOT	1ZGJ	1JQE	0.95	Sc=6.45849, min distance = 2.10316
Q29U70_MEDTR	60961 ade	PIMT_PYRFU Fu	439155 Ad	1JG1	1ZGJ	1JG2	0.84	Sc=6.24461, min distance = 2.69358
Q29U70_MEDTR	65482 sir	ERM_BACSU Ful	439155 Ad	1QAN	1ZGJ	1QAQ	0.88	Sc=6.53751, min distance = 2.11892
Q29U70_MEDTR	65482 sir	MTR1_RHOSH Fu	439155 Ad	1NW7	1ZGJ	1NW6	0.88	Sc=6.50081, min distance = 2.18800
Q2PS28_9PSED	11957435	BGLA_THEMA Fu	54445 cas	2CBU	2PWG	2J7H		Sc=5.71663, min distance = 2.41333
Q2PS28_9PSED	124434 Ca	BGLA_THEMA Fu	54445 cas	2CBU	2PWG	2CBV	0.79	Sc=5.92497, min distance = 2.30888
Q2PS28_9PSED	445238 O1	GUN2_THEFU Fu	64689 bet	2BOF	2PWF	2BOG	0.94	Sc=5.90669, min distance = 1.82611
Q2PS28_9PSED	445248 nc	BGLA_THEMA Fu	54445 cas	2CBU	2PWG	2J78		Sc=6.01157, min distance = 2.07787
Q2PS28_9PSED	445999 CI	Q79G13_MYCTU I	64689 bet	1UP0	2PWF	1UOZ	0.77	Sc=5.70271, min distance = 2.33920
Q2PS28_9PSED	447607 1c	BGLA_THEMA Fu	54445 cas	2CBU	2PWG	1OIF		Sc=5.72788, min distance = 2.40318
Q2PS28_9PSED	447607 1c	Q79G13_MYCTU I	64689 bet	1UP0	2PWF	1UP2		Sc=5.77268, min distance = 2.41777
Q2PS28_9PSED	447651 CI	BGLA_THEMA Fu	54445 cas	2CBU	2PWG	2J77		Sc=5.85029, min distance = 2.37905
Q2PS28_9PSED	448963 2j	BGLA_THEMA Fu	54445 cas	2CBU	2PWG	2J79		Sc=5.99439, min distance = 2.20971
Q2PS28_9PSED	449101 nc	BGLA_THEMA Fu	54445 cas	2CBU	2PWG	1W3J		Sc=5.64101, min distance = 2.49668
Q2PS28_9PSED	6027 xylo	Q8XXK6_RALSO I	64689 bet	2BS5	2PWF	2BS6	0.93	Sc=5.72828, min distance = 0.93889
Q309D1_9AGAR	152217 2,	DYR_CANAL Ful	78165 MES	1M79	2BWR	1M78		Sc=5.82902, min distance = 1.64785
Q309D1_9AGAR	1881 GW34	DYR_CANAL Ful	78165 MES	1M79	2BWR	1AOE		Sc=6.03058, min distance = 1.94652

Q309D1_9AGAR	439353 be	LEG2_CONMY Fu	78165 MES	1IS3	2BWR	1WLD		Sc=5.66077, min distance = 2.07929
Q309D1_9AGAR	447099 CI	DYR_CANAL Ful	78165 MES	1M79	2BWR	1M7A		Sc=6.07509, min distance = 1.82578
Q309D1_9AGAR	656981 CU	LYSC_CHICK Fu	24139 ace	1LZB	2C4D	1YIL		Sc=5.91746, min distance = 2.34290
Q309D1_9AGAR	8761 Bici	MLTB_ECOLI Fu	24139 ace	1QUT	2C4D	1QUS		Sc=5.73406, min distance = 1.96370
Q31163_MOUSE	441481 ri	TRFL_BOVIN Fu	444205 CI	2G93	1FNE	2PX1		Sc=5.75827, min distance = 0.83308
Q381M1_9TRYP	10126464	KAPCA_BOVIN Fu	5957 Aden	1Q24	2Q0D	2OH0		Sc=6.44661, min distance = 2.04652
Q381M1_9TRYP	10172943	KAPCA_BOVIN Fu	5957 Aden	1Q24	2Q0D	2JDS		Sc=6.46413, min distance = 2.03422
Q381M1_9TRYP	11175137	KAPCA_BOVIN Fu	5957 Aden	1Q24	2Q0D	2UW7		Sc=6.17421, min distance = 2.25714
Q381M1_9TRYP	11608401	KAPCA_BOVIN Fu	5957 Aden	1Q24	2Q0D	2UVY		Sc=6.17797, min distance = 2.36282
Q381M1_9TRYP	11696113	KAPCA_BOVIN Fu	5957 Aden	1Q24	2Q0D	2VO3		Sc=6.23091, min distance = 2.46805
Q381M1_9TRYP	11708454	KAPCA_BOVIN Fu	5957 Aden	1Q24	2Q0D	2VNW		Sc=6.10614, min distance = 2.16310
Q381M1_9TRYP	150856 5-	PYR5_HUMAN Fu	6030 Urid	2V30	2Q0F	2QCF	0.94	Sc=5.82546, min distance = 2.49183
Q381M1_9TRYP	150959 1l	A5KE56_PLAVI	6030 Urid	2FFC	2Q0F	2GUU	0.77	Sc=5.66328, min distance = 2.46088
Q381M1_9TRYP	150973 5-	PYR5_HUMAN Fu	6030 Urid	2V30	2Q0F	2QCG	0.91	Sc=5.83687, min distance = 2.29072
Q381M1_9TRYP	160617 Or	PYR5_HUMAN Fu	6030 Urid	2V30	2Q0F	2QCL	0.86	Sc=6.30234, min distance = 2.39733
Q381M1_9TRYP	16122633	KAPCA_BOVIN Fu	5957 Aden	1Q24	2Q0D	2UW0		Sc=6.41837, min distance = 2.41793
Q381M1_9TRYP	16741197	DUT_ECOLI Ful	65070 dUT	1SYL	2NOM	2HRM	0.98	Sc=5.87454, min distance = 2.08068
Q381M1_9TRYP	17749721	PYR5_HUMAN Fu	6030 Urid	2V30	2Q0F	2QCM	0.88	Sc=6.23181, min distance = 2.48219
Q381M1_9TRYP	17751819	KAPCA_BOVIN Fu	5957 Aden	1Q24	2Q0D	2VO6		Sc=6.23651, min distance = 2.14132
Q381M1_9TRYP	24762195	KAPCA_BOVIN Fu	5957 Aden	1Q24	2Q0D	2VO0		Sc=6.23388, min distance = 2.05260
Q381M1_9TRYP	3540 1yds	KAPCA_BOVIN Fu	5957 Aden	1Q24	2Q0D	1YDS		Sc=5.78319, min distance = 2.07979
Q381M1_9TRYP	444564 AD	MYS2_DICDI Fu	5957 Aden	1FMW	2Q0D	1W9I	0.91	Sc=6.33916, min distance = 0
Q381M1_9TRYP	444852 CI	ATPA1_BOVIN Fu	5957 Aden	2V7Q	2Q0D	1COW	0.91	Sc=6.46386, min distance = 1.82604
Q381M1_9TRYP	445132 1x	PYR5_HUMAN Fu	6030 Urid	2V30	2Q0F	3BGG	0.81	Sc=6.19607, min distance = 2.49563
Q381M1_9TRYP	449108 CI	FTSZ1_METJA Fu	6830 guan	1W5B	2Q0E	1W58	0.99	Sc=6.02831, min distance = 2.04775
Q381M1_9TRYP	449489 Id	PYR5_HUMAN Fu	6030 Urid	2V30	2Q0F	2QCH	0.9	Sc=6.13763, min distance = 2.23572
Q381M1_9TRYP	6022 Aden	ARSA1_ECOLX Fu	5957 Aden	1II0	2Q0D	1IHU	0.99	Sc=6.36205, min distance = 2.00339
Q381M1_9TRYP	6022 Aden	DDL_THET8 Ful	5957 Aden	2ZDQ	2Q0D	2ZDH	0.99	Sc=6.32314, min distance = 2.53305
Q381M1_9TRYP	6022 Aden	FAK1_HUMAN Fu	5957 Aden	2IJM	2Q0D	1MP8	0.99	Sc=6.32314, min distance = 2.75505
Q381M1_9TRYP	6022 Aden	MUTS_ECOLI Fu	5957 Aden	1W7A	2Q0D	1OH7	0.99	Sc=6.38, min distance = 1.54089065
Q381M1_9TRYP	6022 Aden	PDK2_HUMAN Fu	5957 Aden	2BU2	2Q0D	2BU8	0.99	Sc=5.89525, min distance = 2.70198
Q381M1_9TRYP	6022 Aden	PURK_ECOLI Fu	5957 Aden	3ETH	2Q0D	3ETJ	0.99	Sc=6.39232, min distance = 2.12656
Q381M1_9TRYP	6022 Aden	RK_BOVIN Full	5957 Aden	3C4W	2Q0D	3C4Z	0.99	Sc=6.3416, min distance = 2.158238
Q381M1_9TRYP	6022 Aden	Y059_METJA Fu	5957 Aden	2J9C	2Q0D	2J9D	0.99	Sc=6.40759, min distance = 2.24022
Q381M1_9TRYP	6083 aden	AAKG1_RAT Ful	5957 Aden	2V92	2Q0D	2V8Q	0.98	Sc=6.28824, min distance = 2.42114
Q381M1_9TRYP	6083 aden	PURP_METJA Fu	5957 Aden	2R7L	2Q0D	2R7M	0.98	Sc=6.27911, min distance = 2.75652
Q381M1_9TRYP	6083 aden	PURP_PYRFU Fu	5957 Aden	2R86	2Q0D	2R85	0.98	Sc=6.26791, min distance = 2.52501

Q381M1_9TRYP	6083 aden	Y059_METJA Fu:	5957 Aden	2J9C	2Q0D	2J9D	0.98	Sc=6.3137, min distance = 2.233593
Q381M1_9TRYP	6131 cyti	KPSU5_ECOLX Fu	6176 Cyti	1H7G	2Q0C	1H7F	0.98	Sc=6.23161, min distance = 2.16536
Q381M1_9TRYP	6132 Cyti	KPSU5_ECOLX Fu	6176 Cyti	1H7G	2Q0C	1H7H	0.99	Sc=5.85994, min distance = 1.79252
Q381M1_9TRYP	65063 dUM	PYR5_HUMAN Fu:	6030 Urid	2V30	2Q0F	2QCH	0.97	Sc=6.08359, min distance = 2.28192
Q381M1_9TRYP	65103 dGT	Y938_MYCTU Fu:	6830 guan	2IRX	2Q0E	2IRY	0.98	Sc=6.38432, min distance = 2.36981
Q381M1_9TRYP	91532 AMF	Q5SJV7_THET8 I	5957 Aden	2Z08	2Q0D	2Z09	0.99	Sc=6.00445, min distance = 2.03544
Q38420_BPP1 I	8778 Laur	CTXA3_NAJAT Fu	311 citri	1XT3	1ZX4	1H0J		Sc=5.68511, min distance = 1.40682
Q38784_ALLSA	24139 ace	MBL2_RAT Full=	185698 al	1RDL	1BWU	1KZD		Sc=5.76797, min distance = 2.56897
Q3HTL7_STRAT	6030 Uric	O87988_BORBR I	6031 Urid	2PZM	2IYA	2PZL	0.99	Sc=6.31736, min distance = 2.01617
Q3HTL7_STRAT	6030 Uric	PYRH_ECOLI Fu:	6031 Urid	2BND	2IYA	2BNE	0.99	Sc=6.31956, min distance = 1.874542
Q3HTL7_STRAT	6132 Cyti	ECX1_PYRAB Fu:	6031 Urid	2PNZ	2IYA	2PO2	0.83	Sc=6.38469, min distance = 2.04311
Q3HTL7_STRAT	8629 UDP-	GSTA_BPT4 Ful:	6031 Urid	1XV5	2IYA	1Y6F	0.89	Sc=6.33006, min distance = 2.18095
Q3J677_RHOS4	5326566 I	BLVRB_HUMAN Fu	444243 FA	1HE4	2IYG	1HE5	0.82	Sc=6.30792, min distance = 2.40542
Q3XZW8_ENTFC	439183 li	TPIS_TRYBB Fu:	311 citri	2VEK	2JFV	1IIH		Sc=5.6666, min distance = 1.884787
Q3XZW8_ENTFC	444212 tr	ACON_BOVIN Fu:	311 citri	1C96	2JFV	1ACO		Sc=5.83287, min distance = 2.05189
Q3XZW8_ENTFC	445607 AT	ACON_BOVIN Fu:	311 citri	1C96	2JFV	1FGH		Sc=5.87513, min distance = 2.19832
Q3XZW8_ENTFC	447550 ph	TPIS_TRYBB Fu:	311 citri	2VEK	2JFV	4TIM		Sc=5.64399, min distance = 2.02297
Q3XZW8_ENTFC	449604 NI	ACON_BOVIN Fu:	311 citri	1C96	2JFV	8ACN		Sc=5.76165, min distance = 2.12366
Q3XZW8_ENTFC	6102712 C	PYRB_ECOLI Fu:	311 citri	1R0B	2JFV	2AIR		Sc=5.67626, min distance = 1.24582
Q3XZW8_ENTFC	6961 PYRC	FUMC_ECOLI Fu:	311 citri	1FUO	2JFV	1FUP		Sc=5.89291, min distance = 1.74050
Q3ZDR0_STEMA	16740985	FRDA_SHEFR Fu:	444188 CI	1M64	2GAG	1E39	0.93	Sc=6.45735, min distance = 2.23465
Q3ZDR0_STEMA	444502 CI	FRDA_SHEFR Fu:	444188 CI	1M64	2GAG	1QJD	0.98	Sc=6.4583, min distance = 2.151811
Q3ZDR0_STEMA	444502 CI	TYTR_TRYCR Fu:	444188 CI	1BZL	2GAG	1GXF	0.98	Sc=6.8978, min distance = 2.067892
Q3ZDR0_STEMA	446013 1,	O28604_ARCFU I	444188 CI	1JNR	2GAG	1JNZ	0.94	Sc=6.89716, min distance = 2.00614
Q40069_HORVU	11957353	HBA_HORSE Ful:	444124 HE	1Y8I	1BGP	1IWH	0.87	Sc=6.76456, min distance = 2.24576
Q40069_HORVU	11957353	HBB_HORSE Ful:	444124 HE	1Y8I	1BGP	1IWH	0.87	Sc=6.77079, min distance = 1.94338
Q40069_HORVU	11957370	HMOX1_HUMAN Fu	444124 HE	1OZW	1BGP	1TWN		Sc=6.52769, min distance = 1.74513
Q40069_HORVU	11957373	CATE_ECOLI Fu:	444124 HE	1IPH	1BGP	1P81	0.79	Sc=6.7724, min distance = 1.960881
Q40069_HORVU	444207 He	COX1_THET8 Fu:	444124 HE	1EHK	1BGP	2QPE	0.99	Sc=6.80337, min distance = 2.18946
Q40069_HORVU	444522 HE	Q83WG3_9ACTO I	444124 HE	2Z3U	1BGP	2Z3T	0.99	Sc=6.80791, min distance = 2.08905
Q40309_MEDSA	440141 9i	IDH_ECOLI Ful:	5886 NADF	1AI2	1ZGD	1BL5		Sc=6.39955, min distance = 2.01957
Q40423_9ASPA	82313 1rc	Q9HYN5_PSEAE I	185698 al	1OVS	1NPL	1W8H		Sc=6.11052, min distance = 1.83055
Q41290_SORBI	29435 1-I	Q2PS28_9PSED I	64689 bet	2PWF	1V03	2PWD		Sc=5.86412, min distance = 2.01306
Q41290_SORBI	447607 1c	Q79G13_MYCTU I	64689 bet	1UP0	1V03	1UP2		Sc=5.77268, min distance = 2.49228
Q41290_SORBI	6027 xylc	O93715_SULSO I	64689 bet	2CDB	1V03	2CDC	0.93	Sc=5.65485, min distance = 2.01494
Q43758_SOYBN	11957353	HBA_HORSE Ful:	444522 HE	1Y8K	2GHK	1IWH	0.87	Sc=6.72218, min distance = 2.18509
Q43758_SOYBN	11957360	CYB_BOVIN Ful:	444522 HE	1BGY	2GHK	1NTM	0.78	Sc=6.80562, min distance = 2.12672

Q43758_SOYBN	11957361	CYB_BOVIN Ful	444522 HE	1BGY	2GHK	1NTZ		0.84	Sc=6.77274, min distance = 2.01300
Q43758_SOYBN	11957363	HBB_HUMAN Ful	444098 HE	1J40	2GHC	1RQA		0.77	Sc=6.74445, min distance = 2.02356
Q43758_SOYBN	11957385	MYG_PHYCA Ful	444098 HE	1A6M	2GHC	2CMM			Sc=6.02705, min distance = 2.28722
Q43758_SOYBN	11957385	MYG_PHYCA Ful	444522 HE	1VXD	2GHK	2CMM			Sc=6.22497, min distance = 2.24890
Q43758_SOYBN	126994 CI	CCPR_YEAST Fu	444098 HE	2EUT	2GHC	1Z53	40.15	0.77	Sc=6.70102, min distance = 2.59322
Q43758_SOYBN	126994 CI	CCPR_YEAST Fu	444522 HE	3E2O	2GHK	1Z53	40.15	0.81	Sc=6.72175, min distance = 2.68344
Q43758_SOYBN	15342951	ALBU_HUMAN Fu	444098 HE	1N5U	2GHC	3B9L			Sc=6.17038, min distance = 2.12547
Q43758_SOYBN	444044 CI	CCPR_YEAST Fu	444522 HE	3E2O	2GHK	1DSE	40.15	0.99	Sc=6.72253, min distance = 2.51667
Q43758_SOYBN	444207 He	CCPR_YEAST Fu	444522 HE	3E2O	2GHK	2CCP	40.15	1	Sc=6.72857, min distance = 2.01413
Q43758_SOYBN	446332 CI	MYG_PHYCA Ful	444522 HE	1VXD	2GHK	1IOP		0.79	Sc=6.63794, min distance = 2.33104
Q44048_ARTGO	999 Benze	PAC_ECOLX Ful	547 Dopac	1AI4	1F1V	1FXH			Sc=5.70244, min distance = 2.14815
Q44244_9PSEU	5287432 S	MENC_ECOLI Fu	955 2-Suc	1FHV	1SJB	1R6W			Sc=6.29056, min distance = 1.98295
Q44467_9LACT	16741044	PYRDA_LACLC Fu	444243 FA	1JUB	2NLI	1JRB			Sc=6.21027, min distance = 2.57454
Q44467_9LACT	446995 FM	CYB2_YEAST Fu	444243 FA	1KBI	2NLI	1LTD		0.77	Sc=6.21217, min distance = 2.47801
Q44467_9LACT	448076 CI	CYB2_YEAST Fu	444243 FA	1KBI	2NLI	1SZG		0.76	Sc=6.21433, min distance = 2.32519
Q46Y90_RALEJ	1800 5-am	TRY1_BOVIN Fu	8172 Trig	2G81	2PRR	1C2K			Sc=5.61756, min distance = 2.81984
Q46Y90_RALEJ	5287519 1	TRY1_BOVIN Fu	8172 Trig	2G81	2PRR	1TX7			Sc=5.6474, min distance = 2.474637
Q46Y90_RALEJ	5496535 2	TRY1_BOVIN Fu	8172 Trig	2G81	2PRR	1G3B			Sc=5.88632, min distance = 2.40717
Q47450_ECOLX	101798 Me	LECA_ARTIN Fu	440552 1b	1UH1	1J8R	1WS5			Sc=5.73529, min distance = 2.02478
Q47450_ECOLX	64947 alp	LECA_ARTIN Fu	440552 1b	1UH1	1J8R	1WS4			Sc=5.7139, min distance = 1.976329
Q47450_ECOLX	84265 1ax	CGL2_COPCI Fu	439357 ga	1ULE	1J8R	1ULF			Sc=5.90663, min distance = 2.15181
Q47644_ECOLX	84265 1ax	CGL2_COPCI Fu	439357 ga	1ULE	2BOS	1ULF			Sc=5.97377, min distance = 1.68088
Q47785_ENTFC	12793 Phe	SRC_HUMAN Ful	311 citri	1O4L	2BL8	1O4O			Sc=5.67897, min distance = 1.14601
Q47785_ENTFC	3893 laur	CO8G_HUMAN Fu	311 citri	1LF7	2BL8	2OVD			Sc=5.6908, min distance = 2.508436
Q47785_ENTFC	446127 3-	RENI_HUMAN Fu	311 citri	2IL2	2BL8	1HRN			Sc=5.66434, min distance = 2.43167
Q47785_ENTFC	447536 1c	SRC_HUMAN Ful	311 citri	1O4L	2BL8	1O4Q			Sc=5.7235, min distance = 0.809535
Q4AE26_9RHIZ	11987682	DHM1_METME Fu	5326460 F	2AD8	2D0V	2AD6		0.83	Sc=6.56796, min distance = 2.17367
Q4CT43_TRYCR	444502 CI	GSHR_HUMAN Fu	444188 CI	3DK9	1BZL	1BWC		0.98	Sc=6.86491, min distance = 2.51178
Q4CT43_TRYCR	449465 CI	GSHR_HUMAN Fu	444188 CI	3DK9	1BZL	3GRT		0.98	Sc=6.8696, min distance = 2.354156
Q4D3W2_TRYCR	16741044	PYRDA_LACLC Fu	444243 FA	1JUB	2DJL	1JRB			Sc=6.17412, min distance = 2.64657
Q4D3W2_TRYCR	448076 CI	CYB2_YEAST Fu	444243 FA	1KBI	2DJL	1SZG		0.76	Sc=6.18434, min distance = 2.00037
Q4DRC4_TRYCR	445708 AM	PURA_ECOLI Fu	8582 Inos	1CIB	1P19	1SON		0.81	Sc=5.95595, min distance = 2.08376
Q4DRC4_TRYCR	6083 ader	PURA1_MOUSE Fu	8582 Inos	1IWE	1P19	1MF0		0.79	Sc=5.94205, min distance = 2.04287
Q4DRC4_TRYCR	6083 ader	PYGM_RABIT Fu	8582 Inos	2QN7	1P19	8GPB		0.79	Sc=5.95818, min distance = 2.53351
Q4DRC4_TRYCR	65110 AIC	PURO_METTH Fu	8582 Inos	2NTK	1P19	2NTL		0.97	Sc=6.17958, min distance = 2.05616
Q4DRC4_TRYCR	6804 guar	GMPR2_HUMAN Fu	8582 Inos	2C6Q	1P19	2A7R		0.99	Sc=6.38141, min distance = 1.62357
Q4DRC4_TRYCR	6804 guar	Q5SLS3_THET8	8582 Inos	2YWU	1P19	2YWT	42.6	0.99	Sc=6.36311, min distance = 2.07872

Q4DRC4_TRYCR	73323 Xan	IMDH_TRIFO Fu	8582 Inos	1MEH	1P19	1MEW		0.94	Sc=5.89278, min distance = 1.08502
Q4E4E1_TRYCR	24139 ace	LEG3_HUMAN Fu	64689 bet	2NMO	2Q2R	1KJL			Sc=6.07738, min distance = 2.13653
Q4E4E1_TRYCR	445564 4-	GUNG_CLOCE Fu	64689 bet	1GA2	2Q2R	1KFG		0.88	Sc=5.81309, min distance = 0.94571
Q4E4E1_TRYCR	445999 CI	Q79G13_MYCTU	64689 bet	1UP0	2Q2R	1UOZ		0.77	Sc=5.64458, min distance = 2.50803
Q4E4E1_TRYCR	447607 1c	Q79G13_MYCTU	64689 bet	1UP0	2Q2R	1UP2			Sc=5.7048, min distance = 2.454812
Q4E4E1_TRYCR	447955 1p	CDK2_HUMAN Fu	6022 Aden	1GY3	2Q2R	1PXI			Sc=5.70247, min distance = 1.80081
Q4E4E1_TRYCR	5957 Aden	HSLU_ECOLI Fu	6022 Aden	1HQY	2Q2R	1DO0		0.99	Sc=5.93475, min distance = 1.91912
Q4E4E1_TRYCR	5957 Aden	MUTS_ECOLI Fu	6022 Aden	1OH7	2Q2R	1W7A		0.99	Sc=5.92722, min distance = 2.01080
Q4E4E1_TRYCR	8977 1dar	PARM_ECOLX Fu	6022 Aden	1MWM	2Q2R	2ZGY		0.8	Sc=5.63266, min distance = 2.07019
Q4PRK9_PLAVI	11987786	LDH_PLAFD Ful	5893 nadi	1T2D	2A92	1T2E		0.77	Sc=6.75682, min distance = 2.45664
Q4PRK9_PLAVI	16129587	G3PA_SPIOL Fu	5893 nadi	1NBO	2A92	2PKR			Sc=6.36351, min distance = 1.86297
Q4PRK9_PLAVI	169266 1,	GALE_HUMAN Fu	5893 nadi	1HZJ	2A92	1I3K		0.79	Sc=6.76564, min distance = 2.11331
Q4PRK9_PLAVI	439153 Di	ADH1B_HUMAN F	5893 nadi	1U3U	2A92	1DEH		0.79	Sc=6.75832, min distance = 1.85127
Q4PRK9_PLAVI	439153 Di	GALE_ECOLI Fu	5893 nadi	1UDC	2A92	1UDB		0.79	Sc=6.76187, min distance = 2.22844
Q4PRK9_PLAVI	445794 AD	ADHX_HUMAN Fu	5893 nadi	2FZW	2A92	2FZE			Sc=6.61578, min distance = 1.40663
Q4PRK9_PLAVI	6083 aden	NADE_ECOLI Fu	5893 nadi	1WXH	2A92	1WXI			Sc=6.32965, min distance = 2.04047
Q4PRK9_PLAVI	6102709 A	LDH_PLAFD Ful	5893 nadi	1T2D	2A92	2A94		0.88	Sc=6.30612, min distance = 2.52133
Q4W5X6_DESVU	5326566 l	BLVRB_HUMAN F	444243 FA	1HE4	2D5M	1HE5		0.82	Sc=6.25598, min distance = 2.21669
Q4W5X6_DESVU	5326566 l	Q9WZW1_THEMA	444243 FA	1T6Y	2D5M	1S4M		0.82	Sc=6.28837, min distance = 2.31579
Q50549_MYCTU	447530	SRC_HUMAN Ful	311 citri	1O4L	1UZR	1O4F			Sc=5.93635, min distance = 2.27474
Q50549_MYCTU	447536 1c	SRC_HUMAN Ful	311 citri	1O4L	1UZR	1O4Q			Sc=5.73401, min distance = 1.62251
Q50549_MYCTU	448573 FI	CASP7_HUMAN F	311 citri	2QL9	1UZR	1SHL			Sc=5.82541, min distance = 1.90484
Q50549_MYCTU	51 2-Oxop	SERA_ECOLI Fu	311 citri	2P9E	1UZR	1YBA		0.76	Sc=5.71862, min distance = 1.28173
Q50565_METTH	164795 N(	RBCMT_PEA Ful	23831 HEP	1MLV	1G5C	2H2J			Sc=5.66843, min distance = 1.15874
Q50565_METTH	5962 L-ly	RBCMT_PEA Ful	23831 HEP	1MLV	1G5C	2H2E			Sc=5.65341, min distance = 0.68493
Q50565_METTH	854023 Ep	Q8WSF8_APLCA	23831 HEP	2BR7	1G5C	2BYQ			Sc=5.76954, min distance = 1.53254
Q50CM2_9HIV1	65023 1hi	POL_HV2RO Ful	5362440 i	1HSH	2R5P	1HII	50.51		Sc=6.19681, min distance = 2.43392
Q50LF2_9CORY	444502 CI	FRDA_SHEFR Fu	444188 CI	1M64	1X31	1QJD		0.98	Sc=6.89608, min distance = 2.10887
Q50LF2_9CORY	444502 CI	GSHR_HUMAN Fu	444188 CI	3DK9	1X31	1BWC		0.98	Sc=6.45324, min distance = 2.15309
Q50LF2_9CORY	444502 CI	TYTR_TRYCR Fu	444188 CI	1BZL	1X31	1GXF		0.98	Sc=6.45554, min distance = 2.06503
Q50LF2_9CORY	449465 CI	GSHR_HUMAN Fu	444188 CI	3DK9	1X31	3GRT		0.98	Sc=6.45424, min distance = 2.14299
Q50LF2_9CORY	5326566 l	Q9WZW1_THEMA	444243 FA	1T6Y	1X31	1S4M		0.82	Sc=6.02124, min distance = 2.45499
Q52060_PSEUF	169266 1,	GALE_HUMAN Fu	5893 nadi	1HZJ	1NVM	1I3K		0.79	Sc=6.33019, min distance = 2.32212
Q52060_PSEUF	439153 Di	Q9BJJ9_PLAFA	5893 nadi	1UH5	1NVM	1V35		0.79	Sc=6.32839, min distance = 1.99942
Q52060_PSEUF	440334 Ni	DAPB_ECOLI Fu	5893 nadi	1DRU	1NVM	1DRW		0.91	Sc=6.795, min distance = 2.0076237
Q52060_PSEUF	6022 Aden	Q5SI02_THET8	5893 nadi	2BJK	1NVM	2BJA			Sc=6.10459, min distance = 2.09172
Q52078_PSEPU	123927 A3	Q9GT92_CRYPV	5893 nadi	2FM3	2DPH	2EWD		0.97	Sc=6.8007, min distance = 2.131827

Q52078_PSEPU	439153 Di	ADH1E_HORSE Fu	5893 nadi	1MGO	2DPH	2JHF	0.79	Sc=6.79361, min distance = 1.99296
Q52078_PSEPU	439153 Di	CTBP1_RAT Ful	5893 nadi	1HKU	2DPH	1HL3	0.79	Sc=6.79127, min distance = 2.19100
Q52078_PSEPU	445794 AD	ADHX_HUMAN Fu	5893 nadi	2FZW	2DPH	2FZE		Sc=6.65041, min distance = 2.22473
Q52437_PSES1	444502 CI	TYTR_TRYCR Fu	444188 CI	1BZL	2GR3	1GXF	0.98	Sc=6.43876, min distance = 1.86461
Q52L64_MOUSE	104904 Me	IGKC_MOUSE Fu	445569 CH	1Q9W	1Q9R	2AJZ		Sc=5.68376, min distance = 2.66071
Q53119_RHOSH	449551 FM	FLAV_DESVH Fu	444243 FA	1F4P	1YRX	5FX2	0.77	Sc=5.97656, min distance = 2.08292
Q53119_RHOSH	5326566 l	BLVRB_HUMAN Fu	444243 FA	1HE4	1YRX	1HE5	0.82	Sc=6.27599, min distance = 2.30715
Q53119_RHOSH	5326566 l	Q9WZW1_THEMA I	444243 FA	1T6Y	1YRX	1S4M	0.82	Sc=6.29035, min distance = 2.53692
Q53W52_THET8	444658 2a	RIFK_HUMAN Fu	444243 FA	1P4M	1VCG	1NB9	0.97	Sc=6.06223, min distance = 2.44427
Q53W68_THET8	16129590	Y5224_ARATH Fu	5886 NADP	1XQ6	2YUT	2Q4B	0.99	Sc=6.80198, min distance = 1.90795
Q53W83_THET8	11314340	KAPCA_BOVIN Fu	5957 Aden	1Q24	1V1B	2UZT		Sc=6.37013, min distance = 2.23785
Q53W83_THET8	11608401	KAPCA_BOVIN Fu	5957 Aden	1Q24	1V1B	2UVY		Sc=6.22181, min distance = 2.11212
Q53W83_THET8	11708454	KAPCA_BOVIN Fu	5957 Aden	1Q24	1V1B	2VNW		Sc=6.11694, min distance = 2.47480
Q53W83_THET8	1540 1h1q	CDK2_HUMAN Fu	6022 Aden	1GY3	1V1A	1H1Q		Sc=6.43169, min distance = 1.83736
Q53W83_THET8	15942652	CDK2_HUMAN Fu	6022 Aden	1GY3	1V1A	2DUV		Sc=6.34799, min distance = 2.24665
Q53W83_THET8	23727981	CDK2_HUMAN Fu	6022 Aden	1GY3	1V1A	3BHT		Sc=6.02613, min distance = 2.81175
Q53W83_THET8	23727982	CDK2_HUMAN Fu	6022 Aden	1GY3	1V1A	3BHU		Sc=6.1214, min distance = 2.934595
Q53W83_THET8	24820111	KIME_RAT Full	5957 Aden	1KVK	1V1B	2R42		Sc=6.00428, min distance = 2.39832
Q53W83_THET8	3246006 m	A4XF23_NOVAD I	161227 2-	2QJN	1V1A	2QJM	0.98	Sc=5.98364, min distance = 1.89333
Q53W83_THET8	3540 1yds	KAPCA_BOVIN Fu	5957 Aden	1Q24	1V1B	1YDS		Sc=5.88671, min distance = 2.23028
Q53W83_THET8	3547 Fasu	KAPCA_BOVIN Fu	5957 Aden	1Q24	1V1B	1Q8W		Sc=5.95366, min distance = 2.11990
Q53W83_THET8	444564 AD	BIOD_ECOLI Fu	5957 Aden	1A82	1V1B	1BS1	0.91	Sc=5.94562, min distance = 2.10542
Q53W83_THET8	444564 AD	MYS2_DICDI Fu	5957 Aden	1FMW	1V1B	1W9I	0.91	Sc=5.97855, min distance = 0
Q53W83_THET8	444852 CI	ATPB_BOVIN Fu	6022 Aden	2CK3	1V1A	1COW	0.9	Sc=6.47363, min distance = 2.04234
Q53W83_THET8	446090 1h	NDKC_DICDI Fu	6022 Aden	1KDN	1V1A	1HIY	0.97	Sc=6.40192, min distance = 2.32724
Q53W83_THET8	447840 CI	PHKG1_RABIT Fu	5957 Aden	1QL6	1V1B	1PHK		Sc=6.00342, min distance = 2.44846
Q53W83_THET8	448043 2g	KAPCA_BOVIN Fu	5957 Aden	1Q24	1V1B	1Q8U		Sc=6.05525, min distance = 2.04780
Q53W83_THET8	4565 1h1r	CDK2_HUMAN Fu	6022 Aden	1GY3	1V1A	1H1R		Sc=6.42697, min distance = 1.52616
Q53W83_THET8	6031 Uric	DCK_HUMAN Ful	6022 Aden	1P5Z	1V1A	2ZIA		Sc=5.84279, min distance = 2.13922
Q53W83_THET8	6031 Uric	O33839_THEMA I	6022 Aden	1XJK	1V1A	1XJG		Sc=5.84898, min distance = 2.12908
Q53W83_THET8	6031 Uric	RIR1_YEAST Fu	6022 Aden	2CVX	1V1A	2CVV		Sc=6.3154, min distance = 2.011775
Q53W83_THET8	6083 aden	PSPF_ECOLI Fu	5957 Aden	2C96	1V1B	2VII	0.98	Sc=6.33814, min distance = 2.40228
Q53W83_THET8	6083 aden	PSPF_ECOLI Fu	6022 Aden	2C98	1V1A	2VII	0.99	Sc=6.32842, min distance = 2.37211
Q53W83_THET8	6083 aden	PURP_METJA Fu	5957 Aden	2R7L	1V1B	2R7M	0.98	Sc=5.89538, min distance = 2.17092
Q53W83_THET8	6083 aden	PURP_METJA Fu	6022 Aden	2R7N	1V1A	2R7M	0.99	Sc=5.86994, min distance = 2.40291
Q53W83_THET8	6083 aden	PURP_PYRFU Fu	5957 Aden	2R86	1V1B	2R85	0.98	Sc=6.36062, min distance = 1.95535
Q53W83_THET8	60961 ade	IPKA_RABIT Fu	6022 Aden	1JBP	1V1A	1FMO	0.95	Sc=6.25481, min distance = 2.55319

Q53W83_THET8	60961 ade	SYQ_ECOLI Ful	5957 Aden	1GTR	1V1B	1O0B	0.94	Sc=6.29417, min distance = 2.02787
Q53W83_THET8	6102809 E	O57883_PYRHO	5957 Aden	2DTO	1V1B	2E41	0.82	Sc=6.02543, min distance = 2.26734
Q53W83_THET8	6420140 2	CDK2_HUMAN Fu	6022 Aden	1GY3	1V1A	2C5Y		Sc=6.57749, min distance = 1.49424
Q53W83_THET8	6804 guar	NDK_PYRHO Ful	6022 Aden	2DYA	1V1A	2DXF	0.8	Sc=5.88675, min distance = 1.61959
Q53W83_THET8	6852187 2	O57883_PYRHO	6022 Aden	1WNL	1V1A	2DTH	0.89	Sc=6.18187, min distance = 1.26546
Q53W83_THET8	6918710 5	CDK2_HUMAN Fu	6022 Aden	1GY3	1V1A	3EJ1		Sc=6.13206, min distance = 2.98623
Q53W83_THET8	8977 1dar	O33839_THEMA	6022 Aden	1XJK	1V1A	1XJE	0.8	Sc=6.42552, min distance = 2.22851
Q53W83_THET8	8977 1dar	PARM_ECOLX Fu	6022 Aden	1MWM	1V1A	2ZGY	0.8	Sc=6.40901, min distance = 2.13189
Q53W83_THET8	91532 AMF	F263_HUMAN Fu	6022 Aden	2AXN	1V1A	2DWP	0.99	Sc=6.03231, min distance = 1.68537
Q53W83_THET8	91532 AMF	KIF1A_MOUSE F	6022 Aden	1I5S	1V1A	1I6I	0.99	Sc=6.48826, min distance = 1.97289
Q53W83_THET8	9817550 V	CDK2_HUMAN Fu	6022 Aden	1GY3	1V1A	3BHV		Sc=6.30995, min distance = 3.01358
Q54527_9ACTO	11349402	PNMT_HUMAN Fu	34756 Acy	2G72	1XDS	2G71		Sc=5.8389, min distance = 1.690992
Q54527_9ACTO	12606920	PNMT_HUMAN Fu	34756 Acy	2G72	1XDS	2ONY		Sc=5.84969, min distance = 1.56011
Q54527_9ACTO	445762 5'	MTTA_THEAQ Fu	34756 Acy	2ADM	1XDS	2IH2	0.93	Sc=6.34125, min distance = 2.40870
Q57329_YERPS	11987635	ADH_DROLE Ful	5893 nadi	1SBY	1RKX	1B2L	0.76	Sc=6.41089, min distance = 1.86085
Q57329_YERPS	16129587	G3PA_SPIOL Fu	5893 nadi	1NBO	1RKX	2PKR		Sc=6.8152, min distance = 1.612093
Q57329_YERPS	169266 1,	GALE_HUMAN Fu	5893 nadi	1HZJ	1RKX	1I3K	0.79	Sc=6.77549, min distance = 2.59024
Q57329_YERPS	439153 Di	GALE_ECOLI Fu	5893 nadi	1UDC	1RKX	1UDB	0.79	Sc=6.33523, min distance = 2.60305
Q57329_YERPS	445794 AD	ADHX_HUMAN Fu	5893 nadi	2FZW	1RKX	2FZE		Sc=6.19733, min distance = 1.95837
Q57U83_9TRYP	16741044	PYRDA_LACLC F	444243 FA	1JUB	2B4G	1JRB		Sc=6.17171, min distance = 2.47463
Q57U83_9TRYP	446995 FM	CYB2_YEAST Fu	444243 FA	1KBI	2B4G	1LTD	0.77	Sc=6.17114, min distance = 1.83944
Q57U83_9TRYP	72 protoc	PYRDA_LACLC F	967 Oroti	1JQX	2B4G	2BSL		Sc=5.82581, min distance = 2.38575
Q57U83_9TRYP	7424 alph	PYRDA_LACLC F	967 Oroti	1JQX	2B4G	2BX7		Sc=5.80653, min distance = 2.38704
Q59096_ACICA	5893 nadi	GALE_HUMAN Fu	169266 1,	1I3K	1F8F	1HZJ	0.79	Sc=6.71084, min distance = 1.95494
Q59718_9PSED	16741204	INHA_MYCTU Fu	5893 nadi	2H7I	2DKN	2IDZ	0.91	Sc=6.42096, min distance = 2.05285
Q59718_9PSED	169266 1,	GALE_HUMAN Fu	5893 nadi	1HZJ	2DKN	1I3K	0.79	Sc=6.79018, min distance = 2.32874
Q59718_9PSED	439153 Di	GALE_ECOLI Fu	5893 nadi	1UDC	2DKN	1UDB	0.79	Sc=6.78732, min distance = 2.29854
Q59718_9PSED	439153 Di	Q9BJJ9_PLAFA	5893 nadi	1UH5	2DKN	1V35	0.79	Sc=6.78476, min distance = 2.43112
Q59718_9PSED	449263 IN	INHA_MYCTU Fu	5893 nadi	2H7I	2DKN	2NV6	0.93	Sc=6.86511, min distance = 2.09051
Q59771_RHOSO	16129587	G3PA_SPIOL Fu	5893 nadi	1NBO	1C1D	2PKR		Sc=6.75436, min distance = 2.19357
Q59771_RHOSO	378 4-nit	APEH_AERPE Fu	6140 L-ph	2HU5	1C1D	1VE7		Sc=5.88842, min distance = 2.05684
Q59771_RHOSO	439153 Di	GALE_ECOLI Fu	5893 nadi	1UDC	1C1D	1UDB	0.79	Sc=6.71508, min distance = 2.12273
Q59771_RHOSO	439153 Di	Q9BJJ9_PLAFA	5893 nadi	1UH5	1C1D	1V35	0.79	Sc=6.71229, min distance = 1.92145
Q59771_RHOSO	445794 AD	ADHX_HUMAN Fu	5893 nadi	2FZW	1C1D	2FZE		Sc=6.53415, min distance = 1.80697
Q59771_RHOSO	446050 CI	G3P_HUMAN Ful	5893 nadi	1U8F	1C1D	3GPD	0.95	Sc=6.67933, min distance = 1.31911
Q59771_RHOSO	6022 Ader	Q5SI02_THET8	5893 nadi	2BJK	1C1D	2BJA		Sc=6.25773, min distance = 1.95075
Q59771_RHOSO	71083 D-F	CAH2_HUMAN Fu	6140 L-ph	2FMG	1C1D	2EZ7		Sc=5.70801, min distance = 2.36836

Q59771_RHOSO	9547960 A	NPD_THEMA Ful	5893 nadi	2H4H	1C1D	2H59			Sc=5.79233, min distance = 2.16720
Q5AU62_EMENI	440141 9i	DHB1_HUMAN Fu	5886 NADP	1QYV	1TI7	1QYW			Sc=5.92216, min distance = 2.61171
Q5AU62_EMENI	440141 9i	G6PD_LEUME Fu	5886 NADP	1H9A	1TI7	1E7Y			Sc=5.89418, min distance = 2.17052
Q5CRJ8_CRYPV	5326843 A	CDK5_HUMAN Fu	5326739 i	1UNH	2QKR	1UNG			Sc=6.03787, min distance = 1.86950
Q5CTR0_CRYPV	23831 HEE	GSTP1_HUMAN Fu	78165 MES	2A2R	2PD0	1PGT		0.81	Sc=5.6016, min distance = 2.201724
Q5CTR0_CRYPV	23831 HEE	MMP8_HUMAN Fu	78165 MES	1ZS0	2PD0	1BZS		0.81	Sc=5.69423, min distance = 1.42840
Q5FYV5_SPHEL	24864076	PYGM_RABIT Fu	65533 cor	3GPB	2UX8	2QRH			Sc=6.17763, min distance = 1.85406
Q5FYV5_SPHEL	4369162 1	PYGM_RABIT Fu	65533 cor	3GPB	2UX8	1HLF			Sc=6.03399, min distance = 1.87911
Q5FYV5_SPHEL	444185 1a	PYGM_RABIT Fu	65533 cor	3GPB	2UX8	1A8I			Sc=5.93104, min distance = 2.65817
Q5FYV5_SPHEL	445825 CI	PYGM_RABIT Fu	65533 cor	3GPB	2UX8	1GG8			Sc=5.90537, min distance = 1.92470
Q5FYV5_SPHEL	657043 CH	PYGM_RABIT Fu	65533 cor	3GPB	2UX8	1XL0			Sc=6.03334, min distance = 1.67922
Q5IFH7_MEDTR	445675 UI	EXTL2_MOUSE Fu	6031 Urid	1ON8	2ACV	1ON6		0.82	Sc=6.20579, min distance = 1.97609
Q5IFH7_MEDTR	6030 Uric	GLMU_HAEIN Fu	6031 Urid	2V0K	2ACV	2V0J		0.99	Sc=6.30493, min distance = 2.06333
Q5IFH7_MEDTR	6030 Uric	O87988_BORBR I	6031 Urid	2PZM	2ACV	2PZL		0.99	Sc=6.31372, min distance = 2.41211
Q5IFH7_MEDTR	6030 Uric	PYRH_ECOLI Fu	6031 Urid	2BND	2ACV	2BNE		0.99	Sc=6.30573, min distance = 2.28243
Q5JD28_PYRKO	3540 1yds	KAPCA_BOVIN Fu	5957 Aden	1Q24	2PBZ	1YDS			Sc=5.95616, min distance = 2.13174
Q5JD28_PYRKO	6022 Ader	CLCN5_HUMAN Fu	5957 Aden	2J9L	2PBZ	2JA3		0.99	Sc=5.69269, min distance = 1.44900
Q5JD28_PYRKO	6022 Ader	PPCK_ECOLI Fu	5957 Aden	2OLR	2PBZ	1K3C		0.99	Sc=5.66361, min distance = 2.47805
Q5JD28_PYRKO	6852207 M	KAPCA_BOVIN Fu	5957 Aden	1Q24	2PBZ	2GNI			Sc=6.01664, min distance = 2.02276
Q5KST5_PSEFR	5893 nadi	GALE_ECOLI Fu	439153 Di	1UDB	1X1T	1UDC		0.79	Sc=6.75279, min distance = 2.51675
Q5KST5_PSEFR	5893 nadi	Q9BJJ9_PLAFA I	439153 Di	1V35	1X1T	1UH5		0.79	Sc=6.76064, min distance = 2.45592
Q5L1S0_GEOKA	11957353	HBA_HORSE Ful	444098 HE	2D5X	2BKM	1IWH		0.83	Sc=6.70374, min distance = 2.25308
Q5L1S0_GEOKA	11957360	CY1_BOVIN Ful	444098 HE	1L0N	2BKM	1NTM			Sc=6.70561, min distance = 2.06560
Q5L1S0_GEOKA	11957363	HBB_HUMAN Ful	444098 HE	1J40	2BKM	1RQA		0.77	Sc=6.75164, min distance = 2.16598
Q5L1S0_GEOKA	11957385	MYG_PHYCA Ful	444098 HE	1A6M	2BKM	2CMM			Sc=6.1963, min distance = 2.227731
Q5L1S0_GEOKA	16741061	MYG_PHYCA Ful	444098 HE	1A6M	2BKM	1MBC		0.83	Sc=6.70056, min distance = 2.06084
Q5P5I2_AZOSE	11957385	MYG_PHYCA Ful	444098 HE	1A6M	2IVF	2CMM			Sc=6.32345, min distance = 2.07145
Q5P5I2_AZOSE	16741183	CPXA_PSEPU Fu	444098 HE	1YRC	2IVF	2ZAW		0.97	Sc=6.59891, min distance = 2.13178
Q5P5I4_AZOSE	11987635	ADH_DROLE Ful	5893 nadi	1SBY	2EWM	1B2L		0.76	Sc=6.53819, min distance = 1.98072
Q5P5I4_AZOSE	169266 1,	GALE_HUMAN Fu	5893 nadi	1HZJ	2EWM	1I3K		0.79	Sc=6.76739, min distance = 2.79651
Q5P5I4_AZOSE	439153 Di	GALE_ECOLI Fu	5893 nadi	1UDC	2EWM	1UDB		0.79	Sc=6.76703, min distance = 2.77708
Q5P5I4_AZOSE	6022 Ader	Q5SI02_THET8 I	5893 nadi	2BJK	2EWM	2BJA			Sc=6.27328, min distance = 2.02526
Q5SH33_THET8	444502 CI	TYTR_TRYCR Fu	444188 CI	1BZL	2CUL	1GXF		0.98	Sc=6.15197, min distance = 2.10135
Q5SH33_THET8	446013 1,	O28603_ARCFU I	444188 CI	1JNR	2CUL	1JNZ		0.94	Sc=5.98672, min distance = 2.18596
Q5SH33_THET8	446013 1,	O28604_ARCFU I	444188 CI	1JNR	2CUL	1JNZ		0.94	Sc=5.98645, min distance = 2.20142
Q5SH33_THET8	6420174 C	O28603_ARCFU I	444188 CI	1JNR	2CUL	2FJB		0.92	Sc=5.91307, min distance = 2.25220
Q5SH33_THET8	6420174 C	O28604_ARCFU I	444188 CI	1JNR	2CUL	2FJB		0.92	Sc=5.91327, min distance = 2.26317

Q5SH92_THET8	11957393	FAK1_HUMAN Fu	6022 Aden	1MP8	2D2F	2ETM		Sc=5.9232, min distance = 2.052478
Q5SH92_THET8	440317 AT	RECA_MYCTU Fu	6022 Aden	1MO3	2D2F	1MO4	0.99	Sc=5.86196, min distance = 2.052668
Q5SH92_THET8	445940 O6	CDK2_HUMAN Fu	6022 Aden	1GY3	2D2F	1GZ8		Sc=5.80086, min distance = 2.526008
Q5SH92_THET8	446090 1h	NDKC_DICDI Fu	6022 Aden	1KDN	2D2F	1HIY	0.97	Sc=6.35786, min distance = 1.392434
Q5SH92_THET8	447700 NS	ABL1_HUMAN Fu	6022 Aden	2G2I	2D2F	2G2H		Sc=5.94725, min distance = 2.372098
Q5SH92_THET8	447962 1p	CDK2_HUMAN Fu	6022 Aden	1GY3	2D2F	2C5N		Sc=5.9116, min distance = 2.187867
Q5SH92_THET8	5331010 c	CDK2_HUMAN Fu	6022 Aden	1GY3	2D2F	2BKZ		Sc=6.05404, min distance = 2.140037
Q5SH92_THET8	6420139 C	CDK2_HUMAN Fu	6022 Aden	1GY3	2D2F	2C5V		Sc=5.89372, min distance = 2.502750
Q5SI02_THET8	23727982	CDK2_HUMAN Fu	6022 Aden	1GY3	2BJA	3BHU		Sc=6.19356, min distance = 2.114124
Q5SI02_THET8	4369136 C	CDK2_HUMAN Fu	6022 Aden	1GY3	2BJA	1DM2		Sc=6.11119, min distance = 2.888284
Q5SI02_THET8	439276 3-	TPIS_TRYBB Fu	311 citri	2VEK	2BJK	6TIM		Sc=5.65657, min distance = 2.058414
Q5SI02_THET8	447916 ad	RIO1_ARCFU Fu	6022 Aden	1ZTH	2BJA	1ZTF	0.95	Sc=6.08088, min distance = 2.173395
Q5SI02_THET8	447955 1p	CDK2_HUMAN Fu	6022 Aden	1GY3	2BJA	1PXI		Sc=5.61489, min distance = 2.536893
Q5SI02_THET8	4565 1h1r	CDK2_HUMAN Fu	6022 Aden	1GY3	2BJA	1H1R		Sc=6.19635, min distance = 2.165172
Q5SI02_THET8	4797 1tpw	TPIS_TRYBB Fu	311 citri	2VEK	2BJK	1TTJ		Sc=5.63416, min distance = 2.418909
Q5SI02_THET8	51 2-Oxop	SERA_ECOLI Fu	311 citri	2P9E	2BJK	1YBA	0.76	Sc=5.75199, min distance = 1.606769
Q5SI02_THET8	5687 1di8	CDK2_HUMAN Fu	6022 Aden	1GY3	2BJA	1DI8		Sc=6.01672, min distance = 2.073646
Q5SI76_THET8	445871 CI	RASH_HUMAN Fu	6830 guan	2CL7	2DY1	1GNP		Sc=5.9754, min distance = 2.232696
Q5SI76_THET8	60961 ade	NUD16_XENLA Fu	6830 guan	2A8S	2DY1	2A8T		Sc=6.24332, min distance = 2.585458
Q5SI76_THET8	8582 Inos	PURA1_MOUSE Fu	6830 guan	1LOO	2DY1	1IWE	0.98	Sc=6.34773, min distance = 2.543844
Q5SI76_THET8	93082 Gab	RASH_HUMAN Fu	6830 guan	2CL7	2DY1	121P	0.99	Sc=6.53762, min distance = 2.136416
Q5SID9_THET8	24180721	PIM1_HUMAN Fu	60961 ade	1YI4	1ODI	3C4E		Sc=6.05465, min distance = 1.678244
Q5SID9_THET8	5327148 C	KAPCA_MOUSE Fu	60961 ade	1FMO	1ODI	2ERZ		Sc=6.17209, min distance = 1.962019
Q5SID9_THET8	657135 Op	PIM1_HUMAN Fu	60961 ade	1YI4	1ODI	1YXV		Sc=5.896, min distance = 2.2974912
Q5SID9_THET8	764 guani	PNPH_HUMAN Fu	6802 guan	1RFG	1ODJ	1V2H		Sc=5.80524, min distance = 2.199741
Q5SID9_THET8	8646 Guar	PNPH_HUMAN Fu	6802 guan	1RFG	1ODJ	1V41		Sc=5.77151, min distance = 2.992486
Q5SIM6_THET8	1005 phos	F16PA_ECOLI Fu	311 citri	2OWZ	1IXE	2OX3		Sc=5.62317, min distance = 2.168469
Q5SIM6_THET8	439276 3-	GGGPS_ARCFU Fu	311 citri	2F6U	1IXE	2F6X		Sc=5.64422, min distance = 1.993301
Q5SIM6_THET8	444390 2-	HS3SB_HUMAN Fu	311 citri	1T8T	1IXE	1T8U		Sc=5.7881, min distance = 1.4478667
Q5SIM6_THET8	51 2-Oxop	SERA_ECOLI Fu	311 citri	2P9E	1IXE	1YBA	0.76	Sc=5.8686, min distance = 0.5794186
Q5SIM6_THET8	6131 cyti	UCK2_HUMAN Fu	311 citri	1UEJ	1IXE	1UJ2		Sc=6.1515, min distance = 2.0271097
Q5SIM6_THET8	6852187 2	PPNK1_LISMO Fu	311 citri	2I29	1IXE	2I2B		Sc=6.041, min distance = 2.6030501
Q5SIT4_THET8	12771338	MCES_HUMAN Fu	439155 Ad	3BGV	2CWW	3EPP	0.88	Sc=6.5518, min distance = 2.0468270
Q5SIT4_THET8	188380 Ac	MCES_ENCCU Fu	439155 Ad	1RI1	2CWW	1Z3C	0.91	Sc=6.54451, min distance = 2.240885
Q5SIT4_THET8	446535 CI	HNMT_HUMAN Fu	439155 Ad	2AOT	2CWW	1JQE	0.95	Sc=6.50944, min distance = 2.521886
Q5SIT4_THET8	60961 ade	PIMT_PYRFU Fu	439155 Ad	1JG1	2CWW	1JG2	0.84	Sc=6.26717, min distance = 2.754085
Q5SIT4_THET8	65482 sir	ERM_BACSU Ful	439155 Ad	1QAN	2CWW	1QAQ	0.88	Sc=6.5599, min distance = 2.072589

Q5SIT4_THET8	65482 sir	MCES_ENCCU Ful	439155 Ad	1RI1	2CWW	2HV9	0.88	Sc=6.54193, min distance = 2.329451
Q5SIT4_THET8	65482 sir	PRMA_THET8 Ful	439155 Ad	3EGV	2CWW	2ZBR	0.88	Sc=6.55513, min distance = 2.313960
Q5SJ05_THET8	1881 GW34	DYR_CANAL Full	78165 MES	1M79	1WK4	1AOE		Sc=6.25716, min distance = 1.639437
Q5SJ05_THET8	447099 CI	DYR_CANAL Full	78165 MES	1M79	1WK4	1M7A		Sc=6.26121, min distance = 1.681645
Q5SJ05_THET8	8200 TETR	LMBL1_HUMAN Fu	78165 MES	1OZ2	1WK4	2RHI		Sc=5.60714, min distance = 2.016425
Q5SJP9_THET8	6022 Aden	Q5SI02_THET8 I	5893 nadi	2BJK	2D4E	2BJA		Sc=6.18561, min distance = 2.341925
Q5SJQ7_THET8	175468 12	LACB_BOVIN Ful	985 palmi	1GXA	2DT8	1BSO	0.83	Sc=5.89621, min distance = 2.343139
Q5SJV7_THET8	3540 1yds	KAPCA_BOVIN Fu	5957 Aden	1Q24	2Z08	1YDS		Sc=5.97865, min distance = 2.056087
Q5SJV7_THET8	444564 AD	BIOD_ECOLI Ful	5957 Aden	1A82	2Z08	1BS1	0.91	Sc=6.48088, min distance = 2.208821
Q5SJV7_THET8	444564 AD	BIOD_ECOLI Ful	91532 AMP	1DAG	2Z09	1BS1	0.91	Sc=6.47235, min distance = 2.241207
Q5SJV7_THET8	444852 CI	ATPA1_BOVIN Fu	5957 Aden	2V7Q	2Z08	1COW	0.91	Sc=6.54539, min distance = 1.835652
Q5SJV7_THET8	447916 ad	RIO1_ARCFU Ful	5957 Aden	1ZP9	2Z08	1ZTF	0.94	Sc=6.32578, min distance = 2.149226
Q5SJV7_THET8	6022 Aden	ARSA1_ECOLX Fu	5957 Aden	1II0	2Z08	1IHU	0.99	Sc=6.47813, min distance = 2.018285
Q5SJV7_THET8	6022 Aden	BIOD_ECOLI Ful	5957 Aden	1A82	2Z08	1DAD	0.99	Sc=6.4884, min distance = 2.0524105
Q5SJV7_THET8	6022 Aden	BIOD_ECOLI Ful	91532 AMP	1DAG	2Z09	1DAD	0.99	Sc=6.48431, min distance = 2.114063
Q5SJV7_THET8	6022 Aden	MUTS_ECOLI Ful	5957 Aden	1W7A	2Z08	1OH7	0.99	Sc=6.46586, min distance = 2.269192
Q5SJV7_THET8	6022 Aden	MYS2_DICDI Ful	5957 Aden	1FMW	2Z08	1VOM	0.99	Sc=6.42187, min distance = 0
Q5SJV7_THET8	6022 Aden	PURP_METJA Ful	5957 Aden	2R7L	2Z08	2R7N	0.99	Sc=6.47124, min distance = 2.083767
Q5SJV7_THET8	6022 Aden	PURP_METJA Ful	91532 AMP	2R7K	2Z09	2R7N	0.99	Sc=6.47927, min distance = 2.132396
Q5SJV7_THET8	6022 Aden	Y059_METJA Ful	5957 Aden	2J9C	2Z08	2J9D	0.99	Sc=6.49963, min distance = 1.721301
Q5SJV7_THET8	6083 aden	AAKG1_RAT Full	5957 Aden	2V92	2Z08	2V8Q	0.98	Sc=6.42465, min distance = 2.090757
Q5SJV7_THET8	6083 aden	PURP_METJA Ful	5957 Aden	2R7L	2Z08	2R7M	0.98	Sc=6.41736, min distance = 2.166219
Q5SJV7_THET8	6083 aden	PURP_METJA Ful	91532 AMP	2R7K	2Z09	2R7M	0.97	Sc=6.41554, min distance = 2.299080
Q5SJV7_THET8	6083 aden	Y059_METJA Ful	5957 Aden	2J9C	2Z08	2J9D	0.98	Sc=6.44118, min distance = 2.486547
Q5SKH6_THET8	16750069	DPHB_PYRHO Ful	439155 Ad	2Z6R	1V9A	2PCI	0.92	Sc=6.04325, min distance = 2.311320
Q5SKH6_THET8	188380 Ad	RBCMT_PEA Full	439155 Ad	2H23	1V9A	2H2E	0.91	Sc=6.52731, min distance = 1.870701
Q5SKH6_THET8	4369099 C	CBIF_BACME Ful	439155 Ad	1CBF	1V9A	2CBF		Sc=6.48638, min distance = 2.622690
Q5SKH6_THET8	4369533 C	SETD8_HUMAN Fu	439155 Ad	1ZKK	1V9A	2BQZ	0.95	Sc=6.47343, min distance = 2.151564
Q5SKH6_THET8	447532 1d	SRC_HUMAN Full	311 citri	1O4L	1V9A	1O4H		Sc=5.91273, min distance = 2.160781
Q5SKH6_THET8	448573 FI	CASP7_HUMAN Fu	311 citri	2QL9	1V9A	1SHL		Sc=5.90383, min distance = 2.184632
Q5SKI6_THET8	21945395	SETD7_HUMAN Fu	34756 Acy	1N6A	2Z0Y	1XQH	0.88	Sc=6.47274, min distance = 2.237838
Q5SKI6_THET8	21945395	SETD7_HUMAN Fu	439155 Ad	2F69	2CX8	1XQH	0.88	Sc=6.02544, min distance = 1.870975
Q5SKI6_THET8	65482 sir	MTTA_THEAQ Ful	439155 Ad	1AQI	2CX8	1AQJ	0.88	Sc=6.06427, min distance = 1.626438
Q5SKI6_THET8	6852187 2	Q70GK9_STRCT I	34756 Acy	1RQP	2Z0Y	2CBX	0.84	Sc=6.17859, min distance = 1.289934
Q5SKQ2_THET8	439153 Di	Q9BJJ9_PLAFA I	5893 nadi	1UH5	2P5Y	1V35	0.79	Sc=6.79475, min distance = 2.316662
Q5SL11_THET8	10822243	PNMT_HUMAN Ful	439155 Ad	2G8N	2GS9	2G70		Sc=6.04385, min distance = 1.827835
Q5SL11_THET8	188380 Ad	MCES_ENCCU Ful	439155 Ad	1RI1	2GS9	1Z3C	0.91	Sc=6.55766, min distance = 2.130409

Q5SL11_THET8	446535 CI	HNMT_HUMAN Fu	439155 Ad	2AOT	2GS9	1JQE		0.95	Sc=6.52378, min distance = 2.39569
Q5SL11_THET8	60961 ade	PIMT_PYRFU Fu	439155 Ad	1JG1	2GS9	1JG2		0.84	Sc=6.29108, min distance = 2.61406
Q5SL11_THET8	65482 sir	MCES_ENCCU Fu	439155 Ad	1RI1	2GS9	2HV9		0.88	Sc=6.10694, min distance = 2.09155
Q5SL11_THET8	65482 sir	PRMA_THET8 Fu	439155 Ad	3EGV	2GS9	2ZBR		0.88	Sc=6.5636, min distance = 2.384188
Q5SLC3_THET8	444502 CI	TYTR_TRYCR Fu	444188 CI	1BZL	2YWL	1GXF		0.98	Sc=6.12096, min distance = 2.10547
Q5SLS0_THET8	16741210	MTTA_THEAQ Fu	34756 Acy	2ADM	2YQZ	2JG3			Sc=6.33694, min distance = 2.26561
Q5SLS0_THET8	445971 CI	COMT_RAT Full	34756 Acy	2CL5	2YQZ	1H1D		0.92	Sc=6.50242, min distance = 2.31681
Q5SLS0_THET8	6852187 2	Q70GK9_STRCT I	34756 Acy	1RQP	2YQZ	2CBX		0.84	Sc=6.06375, min distance = 1.60231
Q5SLS3_THET8	168120 Po	HINT1_RABIT Fu	6804 guan	3RHN	2YWT	5RHN		0.78	Sc=6.38049, min distance = 2.02977
Q5SLS3_THET8	3758 IBMX	PDE5A_HUMAN Fu	6804 guan	1T9S	2YWT	1RKP			Sc=6.06507, min distance = 2.44234
Q5SLS3_THET8	6083 ader	PURA1_MOUSE Fu	8582 Inos	1IWE	2YWU	1MF0		0.79	Sc=6.34047, min distance = 2.01591
Q5SLS3_THET8	6083 ader	PYGM_RABIT Fu	8582 Inos	2QN7	2YWU	8GPB		0.79	Sc=5.98282, min distance = 2.46386
Q5SLS3_THET8	73323 Xar	HGXR_TOXGO Fu	8582 Inos	1QK4	2YWU	1QK5	31.82	0.94	Sc=6.39894, min distance = 2.14554
Q5SLS3_THET8	7339 PRPE	Q4DRC4_TRYCR I	8582 Inos	1P19	2YWU	1I0L	42.6		Sc=5.61713, min distance = 1.48353
Q5SM86_THET8	11608401	KAPCA_BOVIN Fu	5957 Aden	1Q24	1V3S	2UVY			Sc=5.66096, min distance = 3.01695
Q5SM86_THET8	444564 AD	BIOD_ECOLI Fu	5957 Aden	1A82	1V3S	1BS1		0.91	Sc=6.22223, min distance = 2.20424
Q5SM86_THET8	444852 CI	ATPA1_BOVIN Fu	5957 Aden	2V7Q	1V3S	1COW		0.91	Sc=6.39232, min distance = 1.54594
Q5SM86_THET8	447916 ac	RIO1_ARCFU Fu	5957 Aden	1ZP9	1V3S	1ZTF		0.94	Sc=5.92645, min distance = 2.27477
Q5SM86_THET8	449240 1y	KAPCA_BOVIN Fu	5957 Aden	1Q24	1V3S	1YDR			Sc=5.65475, min distance = 2.20923
Q5SM86_THET8	6022 Ader	CLCN5_HUMAN Fu	5957 Aden	2J9L	1V3S	2JA3		0.99	Sc=6.35212, min distance = 1.49965
Q5SM86_THET8	6022 Ader	FAK1_HUMAN Fu	5957 Aden	2IJM	1V3S	1MP8		0.99	Sc=6.23012, min distance = 2.00549
Q5SM86_THET8	6022 Ader	MUTS_ECOLI Fu	5957 Aden	1W7A	1V3S	1OH7		0.99	Sc=6.23841, min distance = 2.72971
Q5SM86_THET8	6022 Ader	PURK_ECOLI Fu	5957 Aden	3ETH	1V3S	3ETJ		0.99	Sc=6.26791, min distance = 2.42511
Q5SM86_THET8	6022 Ader	PURT_ECOLI Fu	5957 Aden	1KJ8	1V3S	1KJQ		0.99	Sc=6.28863, min distance = 2.41850
Q5SM86_THET8	6022 Ader	Q72H90_THET2 I	5957 Aden	2BEK	1V3S	2BEJ		0.99	Sc=6.25481, min distance = 2.02721
Q5SM86_THET8	6022 Ader	RIO1_ARCFU Fu	5957 Aden	1ZP9	1V3S	1ZTH		0.99	Sc=6.30345, min distance = 2.10632
Q5SM86_THET8	6022 Ader	RK_BOVIN Full	5957 Aden	3C4W	1V3S	3C4Z		0.99	Sc=6.2334, min distance = 1.445469
Q5SM86_THET8	6022 Ader	SKY1_YEAST Fu	5957 Aden	1Q97	1V3S	1Q8Y		0.99	Sc=6.21985, min distance = 2.47270
Q5SM86_THET8	6022 Ader	Y059_METJA Fu	5957 Aden	2J9C	1V3S	2J9D		0.99	Sc=6.30457, min distance = 2.66252
Q5SM86_THET8	6083 ader	AAKG1_RAT Ful	5957 Aden	2V92	1V3S	2V8Q		0.98	Sc=6.16907, min distance = 2.05251
Q5SM86_THET8	6083 ader	Y059_METJA Fu	5957 Aden	2J9C	1V3S	2J9D		0.98	Sc=6.15526, min distance = 3.01838
Q5XAY9_STRP6	892 myo-i	SFTPD_HUMAN Fu	79025 alp	1PWB	2ESR	2OS9			Sc=5.78845, min distance = 2.22907
Q65YW7_STRLA	445719 EE	DGDA_BURCE Fu	445008 DE	1D7U	1VFS	1M0Q		0.88	Sc=5.88618, min distance = 2.34817
Q65YW7_STRLA	447036 CI	DGDA_BURCE Fu	445008 DE	1D7U	1VFS	1M0N		0.89	Sc=5.96992, min distance = 2.31099
Q65YW7_STRLA	447037 ME	DGDA_BURCE Fu	445008 DE	1D7U	1VFS	1M0O		0.89	Sc=5.94664, min distance = 2.39471
Q65YW7_STRLA	447038 CI	DGDA_BURCE Fu	445008 DE	1D7U	1VFS	1M0P		0.84	Sc=6.04023, min distance = 2.37280
Q6A8X5_PROAC	444502 CI	GSHR_HUMAN Fu	444188 CI	3DK9	2B9W	1BWC		0.98	Sc=6.87685, min distance = 2.49914

Q6A8X5_PROAC	444502 CI	TYTR_TRYCR Fu	444188 CI	1BZL	2B9W	1GXF		0.98	Sc=6.88028, min distance = 2.35208(
Q6A8X5_PROAC	446013 1,	O28603_ARCFU I	444188 CI	1JNR	2B9W	1JNZ		0.94	Sc=6.88034, min distance = 2.243858
Q6A8X5_PROAC	446013 1,	O28604_ARCFU I	444188 CI	1JNR	2B9W	1JNZ		0.94	Sc=6.88051, min distance = 2.26311!
Q6A8X5_PROAC	6420174 C	O28604_ARCFU I	444188 CI	1JNR	2B9W	2FJB		0.92	Sc=6.88983, min distance = 2.01397(
Q6DPL2_9INFA	154234 Pe	NRAM_I75A5 Fu	449381 Os	2QWH	2HU4	1L7F			Sc=6.4453, min distance = 2.097154!
Q6DPL2_9INFA	16058615	LYSC_CHICK Fu	82313 1rd	1LSZ	2HTY	2H9J			Sc=5.81139, min distance = 1.78655:
Q6DPL2_9INFA	656981 CU	LYSC_CHICK Fu	82313 1rd	1LSZ	2HTY	1YIL			Sc=5.97688, min distance = 1.97541:
Q6L8F0_THETH	15993 dAT	PRIM_BPT7 Ful	33113 gam	1E0J	1V25	1CR2		0.97	Sc=6.4884, min distance = 1.996816
Q6L8F0_THETH	16750062	CSK2A_MAIZE Fu	33113 gam	1LP4	1V25	2OXD			Sc=5.7389, min distance = 2.0403622
Q6L8F0_THETH	16750063	CSK2A_MAIZE Fu	33113 gam	1LP4	1V25	2OXX			Sc=5.61744, min distance = 2.13176
Q6L8F0_THETH	188966 dA	HSLU_ECOLI Fu	33113 gam1E94		1V25	1G4A		0.97	Sc=6.43215, min distance = 1.92639
Q6L8F0_THETH	24316 cyc	PDE10_HUMAN Fu	6083 aden	2OUN	1V26	2OUU		0.81	Sc=6.36711, min distance = 2.05617:
Q6L8F0_THETH	31291 Myr	FHUA_ECOLI Fu	11005 Tet	1QFG	1V26	2GRX			Sc=5.80654, min distance = 2.37249:
Q6L8F0_THETH	439459 Ho	NIFD_KLEPN Fu	311 citri	1H1L	1ULT	1QGU		0.92	Sc=5.94988, min distance = 2.37220(
Q6L8F0_THETH	439459 Ho	NIFK_KLEPN Fu	311 citri	1H1L	1ULT	1QGU		0.92	Sc=5.97221, min distance = 2.311008
Q6L8F0_THETH	445708 AM	NADE_ECOLI Fu	6083 aden	1WXI	1V26	1WXE		0.91	Sc=6.39465, min distance = 2.10317
Q6L8F0_THETH	447832 AD	ACSA_SALTY Fu	6083 aden	2P2F	1V26	1PG4		0.99	Sc=6.47725, min distance = 2.553458
Q6L8F0_THETH	51 2-Oxop	SERA_ECOLI Fu	311 citri	2P9E	1ULT	1YBA		0.76	Sc=5.88325, min distance = 0.82150(
Q6L8F0_THETH	5326739 i	GSK3B_HUMAN Fu	33113 gam	1J1B	1V25	1Q41			Sc=6.39376, min distance = 2.02464
Q6L8F0_THETH	5326977 1	CSK2A_MAIZE Fu	33113 gam	1LP4	1V25	1ZOG			Sc=5.74218, min distance = 2.08719!
Q6L8F0_THETH	5327121 I	PIM1_HUMAN Fu	6083 aden	1YXU	1V26	2C3I			Sc=6.33655, min distance = 1.28565:
Q6L8F0_THETH	6030 Uric	ECX2_SULSO Fu	6083 aden	2C38	1V26	2C37			Sc=6.30263, min distance = 2.333018
Q6L8F0_THETH	6076 Cycl	PDE10_HUMAN Fu	6083 aden	2OUN	1V26	2OUR		0.99	Sc=6.33354, min distance = 1.92935!
Q6L8F0_THETH	6338563 C	MYS2_DICDI Fu	33113 gam	1MMN	1V25	1D0Z			Sc=6.25199, min distance = 2.00369:
Q6L8F0_THETH	6540255 C	DAPK1_HUMAN Fu	33113 gam	1JKL	1V25	1WVX			Sc=6.35608, min distance = 2.15717(
Q6NIF5_CORDI	439182 5'	Q70GK9_STRCT I	34756 Acy	1RQP	2NPN	2CC2		0.86	Sc=6.14726, min distance = 2.56927!
Q6PYX1_HUMAN	101798 Me	LEC1_LATOC Fu	185698 al	1LOG	1ZLS	1LOB		0.94	Sc=5.87043, min distance = 2.45459
Q6PYX1_HUMAN	101798 Me	LEC_GALNI Ful	185698 al	1JPC	1ZLS	1MSA		0.94	Sc=5.87705, min distance = 2.32835!
Q6PYX1_HUMAN	151504 D-	CONA_CANEN Fu	185698 al	1CVN	1ZLS	1I3H			Sc=5.7881, min distance = 2.6224082
Q6PYX1_HUMAN	151504 D-	CVN_NOSEL Ful	185698 al	2RDK	1ZLS	2PYS			Sc=5.79727, min distance = 2.46270(
Q6PYX1_HUMAN	15600 DEC	BACR_HALSA Fu	185698 al	1IW6	1ZLS	1XJI			Sc=5.61886, min distance = 0.92256
Q6PYX1_HUMAN	16741034	Q9XEI3_HORVD I	185698 al	1X38	1ZLS	1IEV		0.82	Sc=5.73005, min distance = 2.08834
Q6PYX1_HUMAN	446578 1r	MBL1_RAT Full	439554 fu	3KMB	1S3K	1KWW		0.96	Sc=5.60974, min distance = 2.11537{
Q6PYX1_HUMAN	446578 1r	Q8XXK6_RALSO I	439554 fu	2BS6	1S3K	2BT9		0.96	Sc=5.60728, min distance = 2.41317{
Q6PYX1_HUMAN	447147 Ab	TSPE_BPP22 Fu	185698 al	1TYU	1ZLS	1TYX		0.98	Sc=5.65518, min distance = 1.11794{
Q6PYX1_HUMAN	64947 alp	LEC1_LATOC Fu	185698 al	1LOG	1ZLS	1LOA		0.94	Sc=5.87312, min distance = 2.30826(
Q6Q004_9HIV1	24764433	POL_HV1BR Ful	5481249 c	2P3B	2P3C	2QNQ			Sc=6.06425, min distance = 2.25045:

Q6Q004_9HIV1	392622 ri	POL_HV1BR Ful	5481249 c	2P3B	2P3C	1RL8			Sc=6.03277, min distance = 2.02714
Q6RYA0_TOBAC	8158 Pela	ALBU_HUMAN Fu	338 salic	2I2Z	1Y7I	1E7E			Sc=5.75955, min distance = 0.75893
Q6T6J0_9FABA	14253 Cet	FABP4_MOUSE F	1369 Phen	2ANS	1TXC	1LIC			Sc=5.72195, min distance = 0.81884
Q6XV46_9INFA	11963507	NEUR2_HUMAN F	447208 DA	2F25	2HTW	2F13		0.89	Sc=6.2874, min distance = 2.075017
Q6XV46_9INFA	154234 Pe	NRAM_I63A3 Fu	447208 DA	2HTR	2HTW	2HTU			Sc=6.43802, min distance = 2.00529
Q6XV46_9INFA	5288452 C	NEUR2_HUMAN F	447208 DA	2F25	2HTW	2F0Z		0.86	Sc=5.99042, min distance = 2.12773
Q700S9_PENSQ	24139 ace	CONA_CANEN Fu	185698 al	1CVN	1TG7	1TEI			Sc=6.08225, min distance = 0.78865
Q700S9_PENSQ	439731 al	ARAF_ECOLI Fu	439353 be	8ABP	1XC6	6ABP		0.93	Sc=5.66215, min distance = 2.51018
Q700S9_PENSQ	7027 gluc	Q72KX2_THET2	439353 be	2B3F	1XC6	2B3B			Sc=5.92682, min distance = 1.93529
Q70DK5_CLOTM	29435 1-D	CDGT_BACS0 Fu	64689 bet	1UKS	2CN3	1I75			Sc=5.70048, min distance = 2.07498
Q70DK5_CLOTM	444279 1s	OMPG_ECOLI Fu	64689 bet	2IWW	2CN3	2IWV			Sc=5.94202, min distance = 1.31585
Q70DK5_CLOTM	445564 4-	GUNG_CLOCE Fu	64689 bet	1GA2	2CN3	1KFG		0.88	Sc=5.62862, min distance = 1.05641
Q70DK5_CLOTM	445999 CI	Q79G13_MYCTU	64689 bet	1UP0	2CN3	1UOZ		0.77	Sc=5.71284, min distance = 1.31792
Q70DK5_CLOTM	62852 B-C	PGH1_SHEEP Fu	64689 bet	2OYU	2CN3	1Q4G		0.85	Sc=6.22749, min distance = 1.25423
Q70GK9_STRCT	439155 Ac	RBCMT_PEA Ful	188380 Ad	2H2E	2C4T	2H23		0.91	Sc=6.41857, min distance = 2.07363
Q71Z85_LISMF	16741210	MTTA_THEAQ Fu	34756 Acy	2ADM	2F8L	2JG3			Sc=6.53255, min distance = 1.62287
Q71Z85_LISMF	439176 Me	MTR1_RHOSH Fu	34756 Acy	1NW5	2F8L	1EG2		0.91	Sc=6.24147, min distance = 2.57918
Q71Z85_LISMF	445971 CI	COMT_RAT Full	34756 Acy	2CL5	2F8L	1H1D		0.92	Sc=6.55172, min distance = 2.22700
Q72H90_THET2	3547 Fasu	KAPCA_BOVIN F	5957 Aden	1Q24	2BEK	1Q8W			Sc=5.90944, min distance = 2.31275
Q72H90_THET2	449240 1y	KAPCA_BOVIN F	5957 Aden	1Q24	2BEK	1YDR			Sc=5.8712, min distance = 2.136187
Q72H90_THET2	6083 aden	AAKG1_RAT Ful	5957 Aden	2V92	2BEK	2V8Q		0.98	Sc=5.96259, min distance = 2.38230
Q72H90_THET2	6083 aden	Y059_METJA Fu	5957 Aden	2J9C	2BEK	2J9D		0.98	Sc=5.8145, min distance = 1.936934
Q72H90_THET2	6338561 C	MYS2_DICDI Fu	5957 Aden	1FMW	2BEK	1D0X			Sc=6.15342, min distance = 0
Q72H90_THET2	91532 AMP	DNAA_AQUAE Fu	6022 Aden	1L8Q	2BEJ	2HCB		0.99	Sc=5.97142, min distance = 1.58462
Q72H90_THET2	91532 AMP	KIF1A_MOUSE F	6022 Aden	1I5S	2BEJ	1I6I		0.99	Sc=6.18302, min distance = 2.38345
Q72H90_THET2	91532 AMP	NIFH1_AZOVI F	5957 Aden	2C8V	2BEK	2AFK		0.99	Sc=6.1805, min distance = 2.562655
Q72H90_THET2	9817550 V	CDK2_HUMAN Fu	6022 Aden	1GY3	2BEJ	3BHV			Sc=6.36382, min distance = 1.61844
Q72K25_THET2	6022 Aden	Q5SI02_THET8	5893 nadi	2BJK	2D4E	2BJA			Sc=6.19516, min distance = 2.36913
Q72KX2_THET2	15942683	FCN3_HUMAN Fu	439353 be	2J5Z	2B3F	2J60		0.77	Sc=5.71012, min distance = 1.31489
Q72KX2_THET2	439680 be	Q9C171_PIREQ	7027 gluc	1W8T	2B3B	1W8U			Sc=5.94398, min distance = 1.48928
Q72KX2_THET2	445257 1i	MYRA_SINAL Fu	7027 gluc	1E6X	2B3B	1E70			Sc=5.73277, min distance = 2.27606
Q72KX2_THET2	445564 4-	GUNG_CLOCE Fu	64689 bet	1GA2	2B3B	1KFG		0.88	Sc=5.7543, min distance = 1.511861
Q72KX2_THET2	445999 CI	Q79G13_MYCTU	64689 bet	1UP0	2B3B	1UOZ		0.77	Sc=5.74542, min distance = 2.38098
Q72KX2_THET2	447607 1c	Q79G13_MYCTU	64689 bet	1UP0	2B3B	1UP2			Sc=5.87591, min distance = 1.19992
Q72KX2_THET2	6027 xylo	Q8XXK6_RALSO	64689 bet	2BS5	2B3B	2BS6		0.93	Sc=5.71487, min distance = 1.87399
Q72KX2_THET2	6027 xylo	Q9ZB17_9LACT	439353 be	1NSX	2B3F	1MN0		0.93	Sc=5.71776, min distance = 2.06323
Q72KX2_THET2	6102765 C	Q9RX51_DEIRA	64689 bet	2BY0	2B3B	2BY2			Sc=5.64326, min distance = 2.13422

Q72KX2_THET2	79025 alp	Q9C171_PIREQ	7027 gluc	1W8T	2B3B	1GWM			Sc=5.84213, min distance = 2.16750
Q746J6_THET2	11957385	MYG_PHYCA Ful	444098 HE	1A6M	1N97	2CMM			Sc=6.31993, min distance = 2.14433
Q746J6_THET2	446406 CZ	MYG_PHYCA Ful	444098 HE	1A6M	1N97	1J3F			Sc=6.40059, min distance = 2.01724
Q75WK2_THETH	43 2-hydr	AAT_ECOLI Ful	107 hydrc	1TOI	1V2F	1AIB			Sc=5.61496, min distance = 1.92500
Q76IQ9_VIBPR	185698 al	MBL1_RAT Full	82313 1rd	1KWV	1V7W	2MSB			Sc=5.84578, min distance = 0.66864
Q76IQ9_VIBPR	185698 al	MBL2_RAT Full	24139 ace	1KZD	1V7W	1RDL			Sc=5.80338, min distance = 2.15507
Q76IQ9_VIBPR	4369268 C	F17AG_ECOLX F	24139 ace	2BSC	1V7W	1O9V		0.88	Sc=6.0566, min distance = 2.553229
Q76IQ9_VIBPR	4369359 S	DPP4_HUMAN Fu	24139 ace	2RGU	1V7W	1X70			Sc=6.07542, min distance = 2.03579
Q76IQ9_VIBPR	439353 be	MBL2_RAT Full	24139 ace	1KZD	1V7W	1RDK			Sc=5.74566, min distance = 2.34357
Q76IQ9_VIBPR	444205 CI	DPP4_HUMAN Fu	24139 ace	2RGU	1V7W	3BJM		0.91	Sc=6.01572, min distance = 2.14684
Q76IQ9_VIBPR	444863 is	MBL1_RAT Full	82313 1rd	1KWV	1V7W	1KWX			Sc=5.60481, min distance = 1.30211
Q76IQ9_VIBPR	444863 is	MBL2_RAT Full	24139 ace	1KZD	1V7W	1RDJ			Sc=5.68635, min distance = 2.41817
Q76IQ9_VIBPR	84571 lac	LECG_ARAHY Fu	82313 1rd	1CIW	1V7W	2PEL			Sc=6.27916, min distance = 2.02606
Q76IQ9_VIBPR	94214 Met	LECG_ARAHY Fu	82313 1rd	1CIW	1V7W	1QF3			Sc=5.64122, min distance = 2.43950
Q79FX6_MYCTU	16958 Dic	CFA1_MYCTU Fu	439155 Ad	1KPG	1TPY	1KPH			Sc=6.00043, min distance = 2.63082
Q79FX6_MYCTU	188380 Ac	MCES_ENCCU Fu	439155 Ad	1RI1	1TPY	1Z3C		0.91	Sc=6.59241, min distance = 2.18486
Q79FX6_MYCTU	446535 CI	HNMT_HUMAN Fu	439155 Ad	2AOT	1TPY	1JQE		0.95	Sc=6.54274, min distance = 2.19307
Q79FX6_MYCTU	60961 ade	PIMT_PYRFU Fu	439155 Ad	1JG1	1TPY	1JG2		0.84	Sc=6.32085, min distance = 2.32218
Q79FX8_MYCTU	439176 Me	MTR1_RHOSH Fu	34756 Acy	1NW5	2FK8	1EG2		0.91	Sc=6.21966, min distance = 2.33531
Q79FX8_MYCTU	445971 CI	COMT_RAT Full	34756 Acy	2CL5	2FK8	1H1D		0.92	Sc=6.53308, min distance = 2.12955
Q79FX8_MYCTU	5326531 C	CHOMT_MEDSA F	34756 Acy	1FPQ	2FK8	1FP1		0.92	Sc=6.5186, min distance = 2.456024
Q79G13_MYCTU	11957432	BGLA_THEMA Fu	447607 1c	1OIF	1UP2	2J7E			Sc=6.22322, min distance = 2.30535
Q79G13_MYCTU	11957435	BGLA_THEMA Fu	447607 1c	1OIF	1UP2	2J7H			Sc=5.64286, min distance = 2.64627
Q79G13_MYCTU	16741013	CARP_YEAST Fu	64689 bet	1FQ4	1UP0	1FQ6			Sc=5.70923, min distance = 2.38437
Q79G13_MYCTU	445037 D-	UGL_BACGL Ful	64689 bet	2FV0	1UP0	2FV1			Sc=5.80655, min distance = 2.22933
Q79G13_MYCTU	445248 nc	BGLA_THEMA Fu	447607 1c	1OIF	1UP2	2J78			Sc=5.89886, min distance = 2.65175
Q79G13_MYCTU	445723 1-	PYGM_RABIT Fu	447607 1c	2G9V	1UP2	1P4H			Sc=6.20469, min distance = 2.50854
Q79G13_MYCTU	447413 NT	BGLA_THEMA Fu	447607 1c	1OIF	1UP2	2J7B			Sc=5.91307, min distance = 2.39481
Q79G13_MYCTU	447601 CI	GUX6_HUMIN Fu	64689 bet	1OCN	1UP0	1OCB	29.32		Sc=5.68216, min distance = 2.56164
Q79G13_MYCTU	447606 IE	GUX6_HUMIN Fu	444809 Th	1OC7	1UOZ	1OCN	29.32		Sc=5.69101, min distance = 2.06823
Q79G13_MYCTU	447651 CI	BGLA_THEMA Fu	447607 1c	1OIF	1UP2	2J77			Sc=5.76396, min distance = 2.59586
Q79G13_MYCTU	447770 CG	PYGM_RABIT Fu	447607 1c	2G9V	1UP2	1P4G			Sc=6.06736, min distance = 2.43988
Q79G13_MYCTU	448963 2j	BGLA_THEMA Fu	447607 1c	1OIF	1UP2	2J79			Sc=5.88856, min distance = 2.34513
Q79G13_MYCTU	448980 nc	BGLA_THEMA Fu	447607 1c	1OIF	1UP2	1UZ1		0.79	Sc=5.76396, min distance = 2.29800
Q79G13_MYCTU	5327083 C	GUN2_THEFU Fu	444809 Th	2BOG	1UOZ	2BOD	42.36	0.9	Sc=5.71748, min distance = 1.72393
Q79G13_MYCTU	5327083 C	GUN2_THEFU Fu	64689 bet	2BOF	1UP0	2BOD	42.36		Sc=5.73786, min distance = 1.37981
Q79G13_MYCTU	79025 alp	AMY1_HORVU Fu	444809 Th	1P6W	1UOZ	1RP8		0.78	Sc=5.77106, min distance = 1.79449

Q79G13_MYCTU	9549193 (	BGLA_THEMA Fu	447607 1c	1OIF	1UP2	2CES		Sc=5.94232, min distance = 2.41777
Q7BK04_HELPY	1540 1h1c	CDK2_HUMAN Fu	6022 Aden	1GY3	1G6O	1H1Q		Sc=6.17103, min distance = 1.89391
Q7BK04_HELPY	23653516	CDK2_HUMAN Fu	6022 Aden	1GY3	1G6O	2R3G		Sc=6.27648, min distance = 1.48931
Q7BK04_HELPY	23653523	CDK2_HUMAN Fu	6022 Aden	1GY3	1G6O	2R3O		Sc=6.34771, min distance = 1.67616
Q7BK04_HELPY	24864080	CDK2_HUMAN Fu	6022 Aden	1GY3	1G6O	2VTR		Sc=5.99875, min distance = 2.09850
Q7BK04_HELPY	24864081	CDK2_HUMAN Fu	6022 Aden	1GY3	1G6O	2VTS		Sc=6.34755, min distance = 1.49099
Q7BK04_HELPY	444564 AD	BIOD_ECOLI Fu	6022 Aden	1DAD	1G6O	1BS1	0.91	Sc=6.42525, min distance = 2.60246
Q7BK04_HELPY	445940 O6	CDK2_HUMAN Fu	6022 Aden	1GY3	1G6O	1GZ8		Sc=6.01807, min distance = 2.22227
Q7BK04_HELPY	445966 O6	CDK2_HUMAN Fu	6022 Aden	1GY3	1G6O	1H0V		Sc=6.16759, min distance = 1.92361
Q7BK04_HELPY	447652 Im	CDK2_HUMAN Fu	6022 Aden	1GY3	1G6O	1OIQ		Sc=6.18335, min distance = 2.00972
Q7BK04_HELPY	447655 1c	CDK2_HUMAN Fu	6022 Aden	1GY3	1G6O	1OIU		Sc=6.19106, min distance = 1.72246
Q7BK04_HELPY	4564 1e1v	CDK2_HUMAN Fu	6022 Aden	1GY3	1G6O	1E1V		Sc=6.08416, min distance = 2.42349
Q7BK04_HELPY	4565 1h1r	CDK2_HUMAN Fu	6022 Aden	1GY3	1G6O	1H1R		Sc=6.16444, min distance = 1.65626
Q7BK04_HELPY	5327087 3	CDK2_HUMAN Fu	6022 Aden	1GY3	1G6O	2BPM		Sc=6.38349, min distance = 1.64640
Q7BK04_HELPY	5327096 2	CDK2_HUMAN Fu	6022 Aden	1GY3	1G6O	2BTR		Sc=6.13673, min distance = 1.77929
Q7BK04_HELPY	5957 Aden	MALK_ECOLI Fu	6022 Aden	2AWN	1G6O	1Q12	0.99	Sc=6.46674, min distance = 1.41857
Q7BK04_HELPY	5957 Aden	MUTS_ECOLI Fu	6022 Aden	1OH7	1G6O	1W7A	0.99	Sc=6.4463, min distance = 1.058642
Q7BK04_HELPY	6031 Uric	DCK_HUMAN Ful	6022 Aden	1P5Z	1G6O	2ZIA		Sc=5.90657, min distance = 2.10247
Q7BK04_HELPY	91532 AMP	BIOD_ECOLI Fu	6022 Aden	1DAD	1G6O	1DAG	0.99	Sc=6.49966, min distance = 2.39089
Q7BK04_HELPY	9547890 I	CDK2_HUMAN Fu	6022 Aden	1GY3	1G6O	1W8C		Sc=6.17684, min distance = 1.98618
Q7BK04_HELPY	9991833 S	CDK2_HUMAN Fu	6022 Aden	1GY3	1G6O	2R3H		Sc=6.09743, min distance = 1.95390
Q7CKD7_YERPE	79639 9-M	FOLB_STAAU Fu	69736 Ran	2DHN	2QX0	1RRW		Sc=5.8609, min distance = 2.780862
Q7CKD7_YERPE	95034 8-A	FOLB_STAAU Fu	69736 Ran	2DHN	2QX0	1RS2		Sc=5.94804, min distance = 2.39360
Q7CRW4_AGRT5	91557 1tm	NMNA3_HUMAN Fu	5893 nadi	1NUU	1Z2I	1NUT		Sc=5.87228, min distance = 1.48887
Q7D8I1_MYCTU	8209 Myri	ABL1_MOUSE Fu	11005 Tet	1OPK	1TED	1OPJ		Sc=5.8546, min distance = 2.173630
Q7D9R5_MYCTU	188380 Ad	MCES_ENCCU Fu	439155 Ad	1RI1	1L1E	1Z3C	0.91	Sc=6.54776, min distance = 2.30358
Q7D9R5_MYCTU	446535 CI	HNMT_HUMAN Fu	439155 Ad	2AOT	1L1E	1JQE	0.95	Sc=6.50507, min distance = 2.00222
Q7D9R5_MYCTU	60961 ade	PIMT_PYRFU Fu	439155 Ad	1JG1	1L1E	1JG2	0.84	Sc=6.21918, min distance = 2.47640
Q7D9R5_MYCTU	65482 sir	MTR1_RHOSH Fu	439155 Ad	1NW7	1L1E	1NW6	0.88	Sc=6.10016, min distance = 2.13853
Q7DDI9_NEIMB	12136 Ber	POL_HV1N5 Ful	311 citri	2GON	2IS5	9HVP		Sc=5.65702, min distance = 0.57296
Q7DDI9_NEIMB	444568 HI	RENI_HUMAN Fu	311 citri	2IL2	2IS5	1BIL		Sc=5.64022, min distance = 2.09714
Q7DDI9_NEIMB	8778 Laur	CTXA3_NAJAT Fu	311 citri	1XT3	2IS5	1H0J		Sc=5.60067, min distance = 0.95050
Q7KIF2_9DIPT	115260 S-	GSTM2_HUMAN Fu	124886 gl	1XW5	1JLV	2AB6	0.94	Sc=6.28333, min distance = 2.63658
Q7KIF2_9DIPT	5288475 C	GSTM2_HUMAN Fu	124886 gl	1XW5	1JLV	2C4J		Sc=6.10447, min distance = 2.01290
Q7KIF2_9DIPT	97536 Hex	GSTP_ONCVO Fu	124886 gl	1TU7	1JLV	1TU8	0.9	Sc=6.45278, min distance = 2.17593
Q7LYW4_THELI	444175 CI	MALE_ECOLI Fu	6255 malt	1IUD	2F5T	1A7L	0.94	Sc=6.40328, min distance = 2.26761
Q7LYW4_THELI	447139 CI	MALE_ECOLI Fu	6255 malt	1IUD	2F5T	1MDQ	0.91	Sc=6.35058, min distance = 2.54675

Q7LYW4_THELI	79025 alp	MALE_ECOLI Fu	6255 malt	1IUD	2F5T	1ANF		0.87	Sc=5.76222, min distance = 2.542124
Q7LYW7_THELI	64689 bet	P74325_SYNY3	7427 treh	2B1Q	1EU8	1U2S		0.87	Sc=5.90022, min distance = 2.512521
Q7LYW7_THELI	657130 CI	CONA_CANLI Fu	7427 treh	2CY6	1EU8	2CYF	1		Sc=6.40926, min distance = 2.145874
Q7LYW7_THELI	82313 1rc	PGRP_CAMDR Fu	7427 treh	3CXA	1EU8	3C93			Sc=6.07341, min distance = 2.049657
Q7M1T4_ARTIN	151504 D-	CONA_CANEN Fu	101798 Me	5CNA	1J4U	1I3H			Sc=5.69341, min distance = 2.311579
Q7M1T4_ARTIN	151504 D-	CONA_CANEN Fu	185698 al	1CVN	1VBO	1I3H			Sc=5.82145, min distance = 1.460824
Q7M1T4_ARTIN	16741014	CARP_YEAST Fu	185698 al	1DPJ	1VBO	1FQ7			Sc=5.70029, min distance = 1.896616
Q7M1T4_ARTIN	24310 fru	Q9HYN5_PSEAE	101798 Me	2JDN	1J4U	1OVP		0.9	Sc=5.72108, min distance = 2.900792
Q7M1T4_ARTIN	439554 fu	Q9HYN5_PSEAE	101798 Me	2JDN	1J4U	1UZV		0.96	Sc=5.64084, min distance = 1.700198
Q7M1T4_ARTIN	439680 be	Q9HYN5_PSEAE	101798 Me	2JDN	1J4U	1OUR		0.94	Sc=5.64084, min distance = 2.871032
Q7M1T4_ARTIN	445184 1,	EGFR_HUMAN Fu	185698 al	1MOX	1VBO	3C09		0.89	Sc=5.7048, min distance = 1.3919791
Q7M1T4_ARTIN	445184 1,	Q6PYX1_HUMAN	185698 al	1ZLS	1VBO	1ZLW		0.89	Sc=5.78651, min distance = 1.321525
Q7M1T4_ARTIN	446972 Mu	LECB_LATOC Fu	185698 al	1LOG	1VBO	1LOD			Sc=6.05292, min distance = 2.367491
Q7M1T4_ARTIN	87330 M97	CONA_CANEN Fu	101798 Me	5CNA	1J4U	1CJP			Sc=6.35659, min distance = 0.698331
Q7M421_9DIPT	11957353	HBB_HORSE Ful	444098 HE	2D5X	1X46	1IWH		0.83	Sc=6.72105, min distance = 2.020572
Q7M421_9DIPT	11957363	HBB_HUMAN Ful	444098 HE	1J40	1X46	1RQA		0.77	Sc=6.71957, min distance = 1.966676
Q7M421_9DIPT	11957385	MYG_PHYCA Ful	444098 HE	1A6M	1X46	2CMM			Sc=6.32423, min distance = 2.068079
Q7M422_9DIPT	11957355	CYB_BOVIN Ful	444522 HE	1BGY	1X3K	1L0L		0.81	Sc=6.66271, min distance = 2.082583
Q7M422_9DIPT	11957363	HBB_HUMAN Ful	444522 HE	2DN1	1X3K	1RQA		0.81	Sc=6.66708, min distance = 2.495913
Q7M422_9DIPT	11957385	MYG_PHYCA Ful	444522 HE	1VXD	1X3K	2CMM			Sc=6.20676, min distance = 2.474819
Q7M422_9DIPT	16741062	MYG_PHYCA Ful	444522 HE	1VXD	1X3K	1MBN		0.88	Sc=6.65504, min distance = 2.237054
Q7M422_9DIPT	16741183	MYG_PHYCA Ful	444522 HE	1VXD	1X3K	2EKT		0.93	Sc=6.59457, min distance = 2.369449
Q7M422_9DIPT	444097 He	MYG_PHYCA Ful	444522 HE	1VXD	1X3K	2EVK		0.96	Sc=6.7309, min distance = 2.1453514
Q7M422_9DIPT	444124 HE	GLB1_GLYDI Fu	444522 HE	1HBG	1X3K	1JF3		0.99	Sc=6.6709, min distance = 2.4668419
Q7M422_9DIPT	444124 HE	MYG_PHYCA Ful	444522 HE	1VXD	1X3K	1U7R		0.99	Sc=6.71852, min distance = 2.156488
Q7M422_9DIPT	444207 He	MYG_PHYCA Ful	444522 HE	1VXD	1X3K	1VXG	1		Sc=6.66332, min distance = 2.132781
Q7M422_9DIPT	446332 CI	MYG_PHYCA Ful	444522 HE	1VXD	1X3K	1IOP		0.79	Sc=6.64586, min distance = 2.350445
Q7M422_9DIPT	446409 HN	HBA_HUMAN Ful	444522 HE	2DN1	1X3K	1J40		0.98	Sc=6.65721, min distance = 2.459311
Q7NX84_CHRVO	24310 fru	Q9HYN5_PSEAE	446578 1r	2JDP	2BOI	1OVP		0.9	Sc=5.90839, min distance = 1.196206
Q7NX84_CHRVO	439554 fu	Q8XXK6_RALSO	446578 1r	2BT9	2BOI	2BS6		0.96	Sc=5.83078, min distance = 0.689369
Q7NX84_CHRVO	439680 be	Q9HYN5_PSEAE	446578 1r	2JDP	2BOI	1OUR		0.94	Sc=5.81081, min distance = 1.224720
Q7NX84_CHRVO	447053 CI	Q9HYN5_PSEAE	446578 1r	2JDP	2BOI	1W8H		0.81	Sc=5.72207, min distance = 1.067146
Q7NX84_CHRVO	82313 1rc	MBL1_RAT Full=	446578 1r	1KWW	2BOI	1KWV			Sc=6.04782, min distance = 1.854612
Q7SI98_STROI	23831 HEF	XYNA_BACSU Fu	125409 be	2QZ3	2D20	2B45			Sc=5.68317, min distance = 1.150866
Q7SI98_STROI	446394 1c	GUX_CELFI Ful	125409 be	2XYL	2D20	1J01	70.61		Sc=6.19765, min distance = 2.599891
Q7SI98_STROI	447315 Me	XYNA_BACSU Fu	125409 be	2QZ3	2D20	1XXN			Sc=5.79391, min distance = 0.633395
Q7SI98_STROI	69275 4-N	GCAA_MOUSE Fu	980 p-nit	1YEK	2D20	1YEG		0.77	Sc=5.60236, min distance = 2.632607

Q7SI98_STROI	69275 4-N	Q58EU8_MOUSE I	980 p-nit	1YEK	2D20	1YEG		0.77	Sc=5.60974, min distance = 2.78778
Q7SIA1_THETH	10041129	SAHH_MYCTU Fu	60961 ade	3CE6	1UAY	3DHY		0.89	Sc=6.33577, min distance = 2.30244
Q7SIA1_THETH	6802 guar	A5KE01_PLAVI I	60961 ade	2PGF	1UAY	2QVN		0.79	Sc=6.34884, min distance = 1.99826
Q7SIC5_THETH	51 2-Oxop	SERA_ECOLI Fu	311 citri	2P9E	1J3N	1YBA		0.76	Sc=5.75562, min distance = 0.44775
Q7SIE2_BACAG	439692 D-	XYLA_STRRU Fu	6027 xylc	3XIS	1H4H	4XIS			Sc=5.63076, min distance = 2.05972
Q7SIE2_BACAG	445564 4-	GUX2_TRIRE Fu	125409 be	1QK0	1H4H	1QJW		0.82	Sc=5.70271, min distance = 0.64435
Q7SIE9_THESP	444915 Gl	CDGT2_BACCI Fu	23831 HEF	1OT1	1GKP	1CXL			Sc=5.78284, min distance = 1.09262
Q7SMT3_9HIV1	65023 1hi	POL_HV2RO Ful	5362440 i	1HSH	2B7Z	1HII	50.51		Sc=6.12567, min distance = 2.74112
Q7SPG9_9HIV1	24883482	POL_HV1BR Ful	5743186 E	2FDE	2FDD	3BGB			Sc=6.06064, min distance = 2.57831
Q7SPG9_9HIV1	392622 ri	POL_HV1BR Ful	5743186 E	2FDE	2FDD	1RL8			Sc=5.9524, min distance = 2.615154
Q7W0A0_BORPE	24820116	GGT_ECOLI Ful	33032 L-g	2DG5	2DVZ	2Z8I			Sc=5.72302, min distance = 2.49225
Q7W0A0_BORPE	24820117	GGT_ECOLI Ful	33032 L-g	2DG5	2DVZ	2Z8J			Sc=5.86141, min distance = 2.18716
Q7W0A0_BORPE	24820118	GGT_ECOLI Ful	33032 L-g	2DG5	2DVZ	2Z8K			Sc=5.71633, min distance = 2.38938
Q7W0A0_BORPE	449178 gl	GLMS_ECOLI Fu	33032 L-g	1XFF	2DVZ	1XFG		0.81	Sc=5.87272, min distance = 1.99707
Q7W0A0_BORPE	5961 L-gl	SYQ_ECOLI Ful	33032 L-g	1O0C	2DVZ	1ZJW		0.84	Sc=5.81534, min distance = 2.18680
Q7YYW6_CRYPV	37792 gam	GNAI1_RAT Ful	8977 1dar	1SVK	2RHD	1AS0		0.99	Sc=6.49058, min distance = 2.33078
Q7YYW6_CRYPV	37792 gam	RHOA_HUMAN Fu	8977 1dar	1TX4	2RHD	1CXZ		0.99	Sc=6.51801, min distance = 2.23574
Q7YYW6_CRYPV	444845 1c	RASH_HUMAN Fu	8977 1dar	2CE2	2RHD	1CLU		0.97	Sc=6.22291, min distance = 2.42534
Q7YYW6_CRYPV	445871 CI	RASH_HUMAN Fu	8977 1dar	2CE2	2RHD	1GNP			Sc=6.69595, min distance = 2.22788
Q7YYW6_CRYPV	445873 CA	RASH_HUMAN Fu	8977 1dar	2CE2	2RHD	2CL6			Sc=6.41471, min distance = 2.17010
Q7YYW6_CRYPV	446248 CI	RAN_HUMAN Ful	8977 1dar	3CH5	2RHD	1IBR			Sc=6.53121, min distance = 2.28784
Q7YYW6_CRYPV	446248 CI	RASH_HUMAN Fu	8977 1dar	2CE2	2RHD	1IAQ			Sc=6.15088, min distance = 2.38604
Q7YYW6_CRYPV	448405 CI	ARF1_RAT Full=	8977 1dar	1RRG	2RHD	1RRF			Sc=6.45435, min distance = 2.58019
Q7YYW6_CRYPV	5496554 C	RASH_HUMAN Fu	8977 1dar	2CE2	2RHD	1GNQ			Sc=6.71558, min distance = 2.22869
Q7YYW6_CRYPV	8582 Inos	PURA1_MOUSE Fu	8977 1dar	1LON	2RHD	1IWE		0.99	Sc=6.39134, min distance = 2.85407
Q7YYW6_CRYPV	93082 Gab	CDC42_HUMAN Fu	8977 1dar	2NGR	2RHD	2ODB		0.99	Sc=6.54571, min distance = 2.42952
Q7YYW6_CRYPV	93082 Gab	RAC3_HUMAN Fu	8977 1dar	2C2H	2RHD	2QME		0.99	Sc=6.52958, min distance = 2.52490
Q7YYW6_CRYPV	93082 Gab	RASH_HUMAN Fu	8977 1dar	2CE2	2RHD	121P		0.99	Sc=6.21256, min distance = 2.48459
Q816V9_BACCR	11987634	GLPD_ECOLI Fu	444188 CI	2QCU	2I0Z	2R4E		0.97	Sc=6.43158, min distance = 2.05857
Q816V9_BACCR	16740985	FRDA_SHEFR Fu	444188 CI	1M64	2I0Z	1E39		0.93	Sc=6.43641, min distance = 2.48389
Q816V9_BACCR	16741253	LSD1_HUMAN Fu	444188 CI	2DW4	2I0Z	2UXN		0.95	Sc=6.4369, min distance = 2.269613
Q816V9_BACCR	444502 CI	FRDA_SHEFR Fu	444188 CI	1M64	2I0Z	1QJD		0.98	Sc=6.43692, min distance = 2.55102
Q816V9_BACCR	444502 CI	TYTR_TRYCR Fu	444188 CI	1BZL	2I0Z	1GXF		0.98	Sc=6.44027, min distance = 2.49771
Q816V9_BACCR	446013 1,	O28603_ARCFU I	444188 CI	1JNR	2I0Z	1JNZ		0.94	Sc=6.43774, min distance = 2.12798
Q818H0_BACCR	445939 1c	PPTA_ECOLI Fu	23831 HEF	1GYX	2GX8	1GYY			Sc=5.63172, min distance = 2.95686
Q818H0_BACCR	447026 NA	LYSC_CHICK Fu	23831 HEF	2HUB	2GX8	1LZG			Sc=6.13288, min distance = 0.43308
Q818H0_BACCR	8200 TETF	Q8WSF8_APLCA I	23831 HEF	2BR7	2GX8	2BYN			Sc=5.7103, min distance = 1.261660

Q81B18_BACCR	8172 Trig	LMBL1_HUMAN Fu	78165 MES	1OZ2	2B81	2RI5			Sc=5.71823, min distance = 0.84428
Q81F54_BACCR	8778 Laur	CTXA3_NAJAT Fu	311 citri	1XT3	1SS4	1H0J			Sc=5.67172, min distance = 0.73010
Q81IL5_BACCR	127370 N2	ASTB_ECOLI Ful	439968 N2	1YNI	2P8C	1YNH		0.92	Sc=6.21483, min distance = 2.16530
Q81K34_BACAN	11987635	ADH_DROLE Full	5893 nadi	1SBY	2C20	1B2L		0.76	Sc=6.86847, min distance = 1.71984
Q81K34_BACAN	439153 Di	GALE_ECOLI Ful	5893 nadi	1UDC	2C20	1UDB		0.79	Sc=6.80395, min distance = 2.43062
Q81K34_BACAN	444427 CI	ADH_DROLE Full	5893 nadi	1SBY	2C20	1B15		0.76	Sc=6.84242, min distance = 1.69440
Q81K34_BACAN	445794 AD	ADHX_HUMAN Ful	5893 nadi	2FZW	2C20	2FZE			Sc=6.66314, min distance = 1.96840
Q81K34_BACAN	449409 CI	GALE_ECOLI Ful	5893 nadi	1UDC	2C20	2UDP			Sc=6.03532, min distance = 2.01199
Q81K34_BACAN	6031 Uric	GALE_ECOLI Ful	5893 nadi	1UDC	2C20	1NAH			Sc=5.83741, min distance = 2.10261
Q81K34_BACAN	8629 UDP-	GALE_ECOLI Ful	5893 nadi	1UDC	2C20	1LRL			Sc=6.15766, min distance = 1.68827
Q81LX0_BACAN	1694 TBBt	CDK2_HUMAN Ful	6022 Aden	1GY3	2JCB	1P5E			Sc=5.71804, min distance = 1.59090
Q81LX0_BACAN	445940 O6	CDK2_HUMAN Ful	6022 Aden	1GY3	2JCB	1GZ8			Sc=6.14555, min distance = 2.45647
Q81LX0_BACAN	445966 O6	CDK2_HUMAN Ful	6022 Aden	1GY3	2JCB	1H0V			Sc=6.16353, min distance = 2.39559
Q81LX0_BACAN	446090 1h	NDKC_DICDI Ful	6022 Aden	1KDN	2JCB	1HIY		0.97	Sc=6.00714, min distance = 1.60470
Q81LX0_BACAN	4564 1e1v	CDK2_HUMAN Ful	6022 Aden	1GY3	2JCB	1E1V			Sc=6.0291, min distance = 2.424300
Q81LX0_BACAN	4565 1h1r	CDK2_HUMAN Ful	6022 Aden	1GY3	2JCB	1H1R			Sc=6.02813, min distance = 2.27145
Q81LX0_BACAN	5957 Ader	PUR7_METJA Ful	6022 Aden	2YZL	2JCB	2Z02		0.99	Sc=6.02483, min distance = 1.39576
Q81LX0_BACAN	6132 Cyti	ECX1_PYRAB Ful	6022 Aden	2PO0	2JCB	2PO2			Sc=5.90027, min distance = 1.46213
Q81LX0_BACAN	65051 ddT	NDKC_DICDI Ful	6022 Aden	1KDN	2JCB	1F3F			Sc=6.43599, min distance = 1.47263
Q82500_9INFA	101798 Me	MBL1_RAT Full	82313 1rd	1KWV	1RV0	1KWU			Sc=5.74414, min distance = 1.38745
Q82500_9INFA	444863 is	MBL1_RAT Full	82313 1rd	1KWV	1RV0	1KWX			Sc=5.62522, min distance = 1.86465
Q82500_9INFA	445692 CI	CARP_YEAST Ful	82313 1rd	1FMU	1RV0	1FQ5			Sc=5.89879, min distance = 0.87675
Q82UW4_NITEU	3713 Indo	TRPA_THET2 Ful	311 citri	1UJP	2A6C	1WXJ			Sc=5.97033, min distance = 2.11760
Q82UW4_NITEU	447532 1o	SRC_HUMAN Full	311 citri	1O4L	2A6C	1O4H			Sc=5.92061, min distance = 2.58470
Q82UW4_NITEU	51 2-Oxop	SERA_ECOLI Ful	311 citri	2P9E	2A6C	1YBA		0.76	Sc=5.62282, min distance = 1.30632
Q836H1_ENTFA	23624247	WRBA_ECOLI Ful	62551 Pen	3B6K	2AZW	3B6I			Sc=5.70013, min distance = 2.08595
Q836T6_ENTFA	162636 1f	UROK_HUMAN Ful	8200 TETR	2O8U	2HV2	1FV9			Sc=5.6966, min distance = 1.299237
Q836T6_ENTFA	448610 1s	UROK_HUMAN Ful	8200 TETR	2O8U	2HV2	1SQO			Sc=6.18464, min distance = 1.39988
Q836T6_ENTFA	449186 oc	OCTC_MOUSE Ful	23831 HEP	1XL7	2HV2	1XL8			Sc=6.13904, min distance = 2.12706
Q836T6_ENTFA	854023 Ep	Q8WSF8_APLCA I	23831 HEP	2BR7	2HV2	2BYQ			Sc=6.01204, min distance = 0.82643
Q837K0_ENTFA	100684 9-	RICI_RICCO Ful	190 adeni	2P8N	2ICS	1IL4			Sc=5.63036, min distance = 2.94626
Q837K0_ENTFA	446312 1i	RICI_RICCO Ful	190 adeni	2P8N	2ICS	1IL9			Sc=5.71068, min distance = 2.10313
Q83WG3_9ACTO	11957370	HMOX1_HUMAN Fu	444124 HE	1OZW	2Z3U	1TWN			Sc=6.65823, min distance = 2.16730
Q83WG3_9ACTO	11957371	HMOX1_HUMAN Fu	444124 HE	1OZW	2Z3U	1TWR			Sc=6.6812, min distance = 2.033203
Q83WG3_9ACTO	11957385	MYG_PHYCA Full	444124 HE	1U7R	2Z3U	2CMM			Sc=6.30099, min distance = 2.66127
Q83WG3_9ACTO	11957385	MYG_PHYCA Full	444522 HE	1VXD	2Z3T	2CMM			Sc=6.30602, min distance = 2.16643
Q83WG3_9ACTO	16214774	CP51_MYCTU Ful	444124 HE	2CIB	2Z3U	2CI0		0.95	Sc=6.67558, min distance = 2.57172

Q83WG3_9ACTO	444207 He	CP119_SULTO Fu	444124 HE	3B4X	2Z3U	1UE8		0.99	Sc=6.80007, min distance = 2.61782
Q83WG3_9ACTO	444207 He	PRXC_CALFU Ful	444124 HE	2CPO	2Z3U	1CPO		0.99	Sc=6.809, min distance = 2.2019650
Q83WG3_9ACTO	446332 CI	MYG_PHYCA Ful	444522 HE	1VXD	2Z3T	1IOP		0.79	Sc=6.69726, min distance = 1.93388
Q83WG3_9ACTO	446406 CZ	MYG_PHYCA Ful	444124 HE	1U7R	2Z3U	1J3F			Sc=6.39285, min distance = 2.44228
Q840M4_ECOLX	447026 NA	LYSC_CHICK Fu	5988 sucr	1JJ0	1YLP	1LZG			Sc=5.89076, min distance = 2.10542
Q84BR0_THETH	16758212	GSK3B_HUMAN Fu	6022 Aden	1J1C	1UC9	2O5K			Sc=6.44892, min distance = 1.97341
Q84BR0_THETH	188966 dA	HSLU_ECOLI Fu	6022 Aden	1HQY	1UC9	1G4A		0.98	Sc=5.80262, min distance = 2.05240
Q84BR0_THETH	447916 ac	RIO1_ARCFU Fu	6022 Aden	1ZTH	1UC9	1ZTF		0.95	Sc=5.79363, min distance = 2.50917
Q84BR0_THETH	5327097 2	CDK2_HUMAN Fu	6022 Aden	1GY3	1UC9	2BTS			Sc=5.86066, min distance = 1.98558
Q84BR0_THETH	5957 Aden	HSP7F_YEAST Fu	6022 Aden	3C7N	1UC9	3D2F		0.99	Sc=5.75578, min distance = 2.00607
Q84BR0_THETH	5957 Aden	MUTS_ECOLI Fu	6022 Aden	1OH7	1UC9	1W7A		0.99	Sc=5.8355, min distance = 2.330290
Q84BR0_THETH	6083 aden	HSP71_HUMAN Fu	6022 Aden	1S3X	1UC9	1XQS		0.99	Sc=6.28955, min distance = 2.00367
Q84BR0_THETH	9994066 4	CDK2_HUMAN Fu	6022 Aden	1GY3	1UC9	2VTJ			Sc=5.84904, min distance = 2.35917
Q84EX5_LACBR	3080718 C	NNTM_HUMAN Fu	5886 NADP	1DJL	1ZK4	1PT9		0.9	Sc=6.3787, min distance = 1.870150
Q84EX5_LACBR	440141 9i	DHB1_HUMAN Fu	5886 NADP	1QYV	1ZK4	1QYW			Sc=5.66031, min distance = 2.43861
Q84HQ2_BIFAD	24139 ace	LYSC_CHICK Fu	5988 sucr	1JJ0	2GDU	1LZB			Sc=5.97902, min distance = 2.18488
Q84HQ2_BIFAD	447026 NA	LYSC_CHICK Fu	5988 sucr	1JJ0	2GDU	1LZG			Sc=5.98457, min distance = 2.18204
Q84HQ2_BIFAD	6251 D-ma	TRFL_BOVIN Fu	5988 sucr	2DVC	2GDU	2Q8J			Sc=5.86664, min distance = 2.10883
Q868M7_9ECHN	439357 ga	ELBP_ECOLX Fu	84265 1ax	1LTI	2Z48	1DJR			Sc=6.04957, min distance = 0.65678
Q868M7_9ECHN	444863 is	MBL1_RAT Full	440552 1b	1AFB	2Z48	1KWX			Sc=5.7534, min distance = 1.556250
Q868M7_9ECHN	445579 BA	ELBP_ECOLX Fu	84265 1ax	1LTI	2Z48	1FD7			Sc=5.89774, min distance = 0.94081
Q868M7_9ECHN	6305 L-tr	TETX_CLOTE Fu	84265 1ax	1D0H	2Z48	1YXW			Sc=5.84626, min distance = 1.80436
Q868M7_9ECHN	6927060 Z	HYSA_STRPN Fu	440552 1b	1OJM	2Z48	1F9G			Sc=5.87651, min distance = 0.76636
Q868M7_9ECHN	79025 alp	LECG_ARAHY Fu	440552 1b	2TEP	2Z48	1V6I			Sc=5.77102, min distance = 2.27036
Q86C09_9TRYP	1195 isop	FPPS_HUMAN Fu	130956 Mi	3B7L	2EWG	1ZW5	37.65		Sc=5.73246, min distance = 1.55716
Q86C09_9TRYP	2088 alen	FPPS_HUMAN Fu	130956 Mi	3B7L	2EWG	2F92	37.65		Sc=5.74513, min distance = 2.15920
Q86C09_9TRYP	24764446	FPPS_HUMAN Fu	16750060	2OPN	2OGD	3CP6	37.65		Sc=6.05557, min distance = 2.07854
Q86C09_9TRYP	4674 pami	FPPS_HUMAN Fu	130956 Mi	3B7L	2EWG	2F89	37.65		Sc=5.63926, min distance = 2.41246
Q86C09_9TRYP	5245 Rise	FPPS_HUMAN Fu	130956 Mi	3B7L	2EWG	2QIS	37.65		Sc=6.03994, min distance = 2.37715
Q86C09_9TRYP	68740 Zol	FPPS_HUMAN Fu	130956 Mi	3B7L	2EWG	2F9K	37.65		Sc=5.85519, min distance = 2.32951
Q86C09_9TRYP	68740 Zol	GGPPS_YEAST Fu	130956 Mi2E92		2EWG	2E91			Sc=5.86305, min distance = 1.58413
Q86MV5_9TRYP	160617 Or	PYR5_HUMAN Fu	6030 Urid	2V30	2B56	2QCL		0.86	Sc=5.60557, min distance = 1.88787
Q86MV5_9TRYP	6131 cyti	UCK2_HUMAN Fu	6133 urid	1UEI	2B56	1UJ2		0.82	Sc=6.16634, min distance = 2.31934
Q86MV5_9TRYP	65063 dUM	PYR5_HUMAN Fu	6030 Urid	2V30	2B56	2QCH		0.97	Sc=6.13456, min distance = 2.28883
Q86MV5_9TRYP	6830 guar	Q381M1_9TRYP	6133 urid	2IKF	2B56	2Q0E			Sc=5.94617, min distance = 2.30598
Q86PR3_AEDAE	175468 12	LACB_BOVIN Fu	985 palmi	1GXA	1PZ4	1BSO		0.83	Sc=5.81956, min distance = 2.74518
Q873X9_ASPFU	119404 Ma	Q54276_SERMA	445246 N-	1E6R	2A3E	1W1Y	28.95		Sc=6.36143, min distance = 0.72368

Q873X9_ASPFU	119404 Ma	Q54276_SERMA	445247 CI	1E6R	2A3E	1W1Y	28.95		Sc=6.20222, min distance = 2.38896
Q873X9_ASPFU	126154 Cy	Q54276_SERMA	445247 CI	1E6R	2A3E	1W1P	28.95		Sc=5.7466, min distance = 2.063300
Q874E9_9TREE	444305 L-	Q3XZW8_ENTFC	311 citri	2JFV	2CZQ	2JFW			Sc=5.64969, min distance = 2.01301
Q88H32_PSEPK	5893 nadi	ADH1E_HORSE Fu	439153 Di	2JHF	1X7D	1MGO		0.79	Sc=6.76756, min distance = 2.13555
Q88N37_PSEPK	446966 [2	A2NHM3_MOUSE	311 citri	1ZEA	1RE5	1LO2			Sc=6.02396, min distance = 2.48452
Q88N37_PSEPK	5326566 l	Q9WZW1_THEMA	311 citri	1MRZ	1RE5	1S4M			Sc=5.85885, min distance = 2.29591
Q88NF7_PSEPK	16741044	PYRDA_LACLC Fu	444243 FA	1JUB	2H8Z	1JRB			Sc=5.86192, min distance = 2.62325
Q88NF7_PSEPK	446995 FM	CYB2_YEAST Fu	444243 FA	1KBI	2H8Z	1LTD		0.77	Sc=6.15682, min distance = 2.32197
Q88NF7_PSEPK	448076 CI	CYB2_YEAST Fu	444243 FA	1KBI	2H8Z	1SZG		0.76	Sc=5.87205, min distance = 2.11154
Q8A013_BACTN	16214827	CDK2_HUMAN Fu	6022 Aden	1GY3	2Q14	2UZN			Sc=5.91558, min distance = 1.72442
Q8A013_BACTN	445940 O6	CDK2_HUMAN Fu	6022 Aden	1GY3	2Q14	1GZ8			Sc=6.05197, min distance = 2.48062
Q8A013_BACTN	445966 O6	CDK2_HUMAN Fu	6022 Aden	1GY3	2Q14	1H0V			Sc=5.82243, min distance = 2.57229
Q8A013_BACTN	447962 1p	CDK2_HUMAN Fu	6022 Aden	1GY3	2Q14	2C5N			Sc=5.95277, min distance = 1.62664
Q8A013_BACTN	5288641 i	CDK2_HUMAN Fu	6022 Aden	1GY3	2Q14	1E9H			Sc=6.1705, min distance = 1.234005
Q8A013_BACTN	5327097 2	CDK2_HUMAN Fu	6022 Aden	1GY3	2Q14	2BTS			Sc=5.82133, min distance = 1.54199
Q8A013_BACTN	5327124	CDK2_HUMAN Fu	6022 Aden	1GY3	2Q14	2C4G			Sc=6.07794, min distance = 0.86986
Q8A013_BACTN	5957 Ader	Y059_METJA Fu	6022 Aden	2J9D	2Q14	2J9C		0.99	Sc=5.70329, min distance = 2.09118
Q8A013_BACTN	6852187 2	O57883_PYRHO	6022 Aden	1WNL	2Q14	2DTH		0.89	Sc=5.85736, min distance = 2.21560
Q8A013_BACTN	9926933 i	CDK2_HUMAN Fu	6022 Aden	1GY3	2Q14	2VTI			Sc=5.94231, min distance = 1.85875
Q8AAK6_BACTN	3758 IBMX	PDE4D_HUMAN Fu	125132 Bi	1TB7	2JE8	1ZKN			Sc=6.0041, min distance = 2.005343
Q8AYM0_THUTH	11957385	MYG_PHYCA Ful	444098 HE	1A6M	1V4X	2CMM	24.66		Sc=6.09299, min distance = 2.54666
Q8AYM1_THUTH	11957385	MYG_PHYCA Ful	444098 HE	1A6M	1V4X	2CMM			Sc=6.07504, min distance = 2.47138
Q8DG54_THEEB	588715 In	1A02_HUMAN Fu	8172 Trig	2PYE	2C41	2GJ6			Sc=5.66462, min distance = 1.67876
Q8DMN3_THEEB	444243 FA	Q9WZW1_THEMA	444658 2a	1T6Z	1X0P	1T6Y		0.97	Sc=6.15992, min distance = 1.80141
Q8DMN3_THEEB	5326566 l	Q9HPW4_HALSA	444658 2a	2CCB	1X0P	2CC6		0.85	Sc=6.28617, min distance = 2.96948
Q8DMN3_THEEB	6323394 C	RIB4_SCHPO Fu	444658 2a	1KYV	1X0P	2A59			Sc=5.84153, min distance = 2.10251
Q8DQ16_STRR6	188350 5'	MTAP_HUMAN Fu	446609 1y	1K27	1ZOS	1SD2			Sc=6.30309, min distance = 2.06186
Q8DQ16_STRR6	439176 Me	MTAP_HUMAN Fu	446609 1y	1K27	1ZOS	1CG6			Sc=6.33521, min distance = 2.44156
Q8DQ16_STRR6	656970 MT	MTNN_ECOLI Fu	446609 1y	1Y6R	1ZOS	1Y6Q		0.89	Sc=6.32448, min distance = 2.39534
Q8DSE5_STRMU	119119 Di	DPOLB_HUMAN Fu	65091 dCT	1MQ3	2HVW	2FMP		0.93	Sc=6.12669, min distance = 2.80924
Q8DSE5_STRMU	13945 Dec	Q93IG4_MYCMS	65091 dCT	2JAQ	2HVW	2JAT		0.97	Sc=6.11344, min distance = 2.29093
Q8DSE5_STRMU	15993 dAT	DPO42_SULSO Fu	65091 dCT	2ASD	2HVW	2AGQ			Sc=5.69152, min distance = 2.02724
Q8DSE5_STRMU	15993 dAT	NRDD_BPT4 Ful	65091 dCT	1H78	2HVW	1H7A			Sc=5.84538, min distance = 2.25401
Q8DSE5_STRMU	64968 dTT	DPO42_SULSO Fu	65091 dCT	2ASD	2HVW	1S0O		0.75	Sc=5.63665, min distance = 2.69425
Q8DSE5_STRMU	64968 dTT	NRDD_BPT4 Ful	65091 dCT	1H78	2HVW	1H79		0.75	Sc=5.87545, min distance = 2.44596
Q8DUC1_STRMU	12136 Ber	POL_HV1N5 Ful	311 citri	2GON	2HJ0	9HVP			Sc=5.67658, min distance = 0.81136
Q8DUC1_STRMU	439459 Ho	NIFD_KLEPN Fu	311 citri	1H1L	2HJ0	1QGU		0.92	Sc=6.00128, min distance = 2.18623

Q8DUC1_STRMU	439459 Hc	NIFK_KLEPN Fu	311 citri	1H1L	2HJ0	1QGU	0.92	Sc=6.01015, min distance = 2.189382
Q8DUC1_STRMU	447534 IS	SRC_HUMAN Ful	311 citri	1O4L	2HJ0	1O4J		Sc=5.92766, min distance = 2.815437
Q8DUC1_STRMU	51 2-Oxop	SERA_ECOLI Fu	311 citri	2P9E	2HJ0	1YBA	0.76	Sc=5.73619, min distance = 1.671798
Q8DUC1_STRMU	93072 Ure	PYRB_ECOLI Fu	311 citri	1R0B	2HJ0	1R0C		Sc=5.95761, min distance = 0.638070
Q8DY32_STRA5	37792 gam	TGM3_HUMAN Fu	8977 1dar	1VJJ	2OGI	1RLE	0.99	Sc=6.0196, min distance = 1.9494622
Q8DY32_STRA5	445463 B-	TGM3_HUMAN Fu	8977 1dar	1VJJ	2OGI	1L9N		Sc=6.11735, min distance = 2.191492
Q8DY32_STRA5	447979 5G	KGUA_ECOLI Fu	8977 1dar	2AN9	2OGI	2ANB		Sc=5.85406, min distance = 2.075486
Q8DY32_STRA5	6022 Ader	PARM_ECOLX Fu	8977 1dar	2ZGY	2OGI	1MWM	0.8	Sc=5.93974, min distance = 2.419974
Q8DY32_STRA5	6804 guar	DPOL_BPR69 Fu	8977 1dar	1CLQ	2OGI	1WAJ	0.99	Sc=5.85442, min distance = 2.088235
Q8DY32_STRA5	6804 guar	EF1A_YEAST Fu	8977 1dar	2B7B	2OGI	1G7C	0.99	Sc=5.82063, min distance = 2.391515
Q8DY32_STRA5	8582 Inos	PURA1_MOUSE Fu	8977 1dar	1LON	2OGI	1IWE	0.99	Sc=6.47815, min distance = 1.152984
Q8EEC8_SHEON	16741044	PYRDA_LACLC Fu	444243 FA	1JUB	2GOU	1JRB		Sc=6.19616, min distance = 2.448181
Q8EEC8_SHEON	446995 FM	CYB2_YEAST Fu	444243 FA	1KBI	2GOU	1LTD	0.77	Sc=6.19261, min distance = 2.378208
Q8EEC8_SHEON	448076 CI	CYB2_YEAST Fu	444243 FA	1KBI	2GOU	1SZG	0.76	Sc=6.20107, min distance = 2.192150
Q8EIU4_SHEON	11957353	HBB_HORSE Ful	444098 HE	2D5X	2IIZ	1IWH	0.83	Sc=6.72898, min distance = 2.006260
Q8EIU4_SHEON	11957355	CY1_BOVIN Ful	444098 HE	1L0N	2IIZ	1L0L	0.77	Sc=6.76743, min distance = 1.974724
Q8EIU4_SHEON	11957385	MYG_PHYCA Ful	444098 HE	1A6M	2IIZ	2CMM		Sc=6.1963, min distance = 2.4169124
Q8EIU4_SHEON	16741061	MYG_PHYCA Ful	444098 HE	1A6M	2IIZ	1MBC	0.83	Sc=6.74553, min distance = 2.068027
Q8EIU4_SHEON	4369122 C	GLB1_LUCPE Fu	444098 HE	1FLP	2IIZ	1B0B	0.96	Sc=6.7479, min distance = 2.2529920
Q8F8Y3_LEPIN	188380 Ac	MCES_ENCCU Fu	439155 Ad	1RI1	2HNK	1Z3C	0.91	Sc=6.58253, min distance = 2.124901
Q8F8Y3_LEPIN	446535 CI	HNMT_HUMAN Fu	439155 Ad	2AOT	2HNK	1JQE	0.95	Sc=6.551, min distance = 2.3600631
Q8F8Y3_LEPIN	60961 ade	PIMT_PYRFU Fu	439155 Ad	1JG1	2HNK	1JG2	0.84	Sc=6.30787, min distance = 3.032111
Q8F8Y3_LEPIN	65482 sir	ERM_BACSU Ful	439155 Ad	1QAN	2HNK	1QAQ	0.88	Sc=6.59138, min distance = 2.231550
Q8F8Y3_LEPIN	65482 sir	MCES_ENCCU Fu	439155 Ad	1RI1	2HNK	2HV9	0.88	Sc=6.58201, min distance = 2.409044
Q8F8Y3_LEPIN	65482 sir	PRMA_THET8 Fu	439155 Ad	3EGV	2HNK	2ZBR	0.88	Sc=6.59241, min distance = 2.379047
Q8G9L3_RHOOP	66644 Sal	PER_ARTRA Ful	10313 N-H	1HSR	2BOY	1CK6	0.76	Sc=5.85789, min distance = 2.150170
Q8GAX0_THITH	11005 Tet	ALBU_HUMAN Fu	311 citri	1TF0	2D4V	1N5U		Sc=5.83475, min distance = 1.711205
Q8GAX0_THITH	11869260	DGAL_ECOLI Fu	311 citri	2FW0	2D4V	2QW1		Sc=5.62959, min distance = 2.525431
Q8GAX0_THITH	16750041	PYRB_ECOLI Fu	311 citri	1R0B	2D4V	2IPO		Sc=6.05392, min distance = 2.121753
Q8GAX0_THITH	25023707	DNLJ_ENTFA Fu	5893 nadi	1TAE	2D4V	3BAB		Sc=6.03953, min distance = 2.004037
Q8GAX0_THITH	439183 1i	TPIS_TRYBB Fu	311 citri	2VEK	2D4V	1IIH		Sc=5.61992, min distance = 1.496281
Q8GAX0_THITH	444212 tr	ACON_BOVIN Fu	311 citri	1C96	2D4V	1ACO		Sc=5.7785, min distance = 2.0106939
Q8GAX0_THITH	445607 AT	ACON_BOVIN Fu	311 citri	1C96	2D4V	1FGH		Sc=5.82558, min distance = 2.103609
Q8GAX0_THITH	6961 PYRC	FUMC_ECOLI Fu	311 citri	1FUO	2D4V	1FUP		Sc=6.17737, min distance = 2.095199
Q8GAX0_THITH	8778 Laur	CTXA3_NAJAT Fu	311 citri	1XT3	2D4V	1H0J		Sc=5.72847, min distance = 0.830202
Q8GIP9_STRSU	5893 nadi	GALE_ECOLI Fu	439153 Di	1UDB	1OC2	1UDC	0.79	Sc=6.78884, min distance = 2.365824
Q8GR61_GLUOX	15942690	INHA_MYCTU Fu	5893 nadi	2H7I	1ZEM	2NTJ		Sc=6.14115, min distance = 1.954257

Q8GR61_GLUOX	439153 Di	GALE_ECOLI Fu	5893 nadi	1UDC	1ZEM	1UDB	0.79	Sc=6.30605, min distance = 2.58196
Q8GR61_GLUOX	449263 IN	INHA_MYCTU Fu	5893 nadi	2H7I	1ZEM	2NV6	0.93	Sc=6.39979, min distance = 2.11257
Q8GR64_PSEPU	11987682	DHM1_METME Fu	5326460 F	2AD8	1KV9	2AD6	0.83	Sc=6.55968, min distance = 2.38622
Q8GR64_PSEPU	164795 N(	RBCMT_PEA Ful	23831 HEF	1MLV	1KV9	2H2J		Sc=5.65264, min distance = 2.65026
Q8GR64_PSEPU	854023 Ep	Q8WSF8_APLCA	23831 HEF	2BR7	1KV9	2BYQ		Sc=5.81483, min distance = 1.24772
Q8GSD2_PTEAG	151504 D-	CONA_CANEN Fu	101798 Me	5CNA	1S1A	1I3H		Sc=5.82145, min distance = 0.74639
Q8GSD2_PTEAG	151504 D-	CVN_NOSEL Ful	185698 al	2RDK	1S1A	2PYS		Sc=5.75163, min distance = 2.47577
Q8GSD2_PTEAG	16741013	CARP_YEAST Fu	185698 al	1DPJ	1S1A	1FQ6		Sc=5.66326, min distance = 2.09264
Q8GSD2_PTEAG	16741034	Q9XEI3_HORVD	185698 al	1X38	1S1A	1IEV	0.82	Sc=5.69574, min distance = 2.63110
Q8GSD2_PTEAG	445184 1,	Q6P5R5_HUMAN	185698 al	1OP3	1S1A	1ZLW	0.89	Sc=5.72381, min distance = 2.65376
Q8GSD2_PTEAG	445184 1,	Q6PYX1_HUMAN	185698 al	1ZLS	1S1A	1ZLW	0.89	Sc=5.75684, min distance = 2.11340
Q8GSD2_PTEAG	446972 Mu	LEC1_LATOC Fu	185698 al	1LOG	1S1A	1LOD		Sc=6.07699, min distance = 2.38903
Q8GSD2_PTEAG	446972 Mu	LECB_LATOC Fu	185698 al	1LOG	1S1A	1LOD		Sc=6.07266, min distance = 2.40653
Q8GSD2_PTEAG	64947 alp	LEC1_LATOC Fu	185698 al	1LOG	1S1A	1LOA	0.94	Sc=5.82581, min distance = 2.35321
Q8GZD5_9ROSI	445948 D-	Q9HYN5_PSEAE	64689 bet	1W8F	1UMZ	1OXC		Sc=5.85704, min distance = 0.57412
Q8GZD5_9ROSI	445999 CI	GUX6_HUMIN Fu	64689 bet	1OCN	1UMZ	1OC7	0.77	Sc=5.61221, min distance = 1.86671
Q8GZD5_9ROSI	446220 cc	Q5XKG4_MOUSE	439353 be	1UZ8	1UMZ	1Q72		Sc=5.87839, min distance = 1.87811
Q8GZD5_9ROSI	447607 1c	Q79G13_MYCTU	64689 bet	1UP0	1UMZ	1UP2		Sc=5.74009, min distance = 1.29187
Q8GZD5_9ROSI	6102754 2	Q9HYN5_PSEAE	64689 bet	1W8F	1UMZ	2BOJ	0.88	Sc=5.72207, min distance = 0.52078
Q8I2M1_PLAF7	164795 N(	RBCMT_PEA Ful	23831 HEF	1MLV	1Z6G	2H2J		Sc=5.73948, min distance = 2.01961
Q8I2M1_PLAF7	5962 L-ly	RBCMT_PEA Ful	23831 HEF	1MLV	1Z6G	2H2E		Sc=5.64592, min distance = 2.42303
Q8I2M1_PLAF7	854023 Ep	Q8WSF8_APLCA	23831 HEF	2BR7	1Z6G	2BYQ		Sc=5.8025, min distance = 0.684988
Q8I3X4_PLAF7	122671 9-	PNPH_BOVIN Fu	444499 Fc	1B8O	1NW4	1A9P	0.89	Sc=6.3107, min distance = 2.704296
Q8I3X4_PLAF7	2022 acyc	PNPH_HUMAN Fu	444499 Fc	1RT9	1NW4	1PWY		Sc=6.08523, min distance = 2.42416
Q8I3X4_PLAF7	23190 Dea	Q9GPQ4_TRYVI	6021 inos	1KIC	2BSX	1HP0		Sc=6.20975, min distance = 2.58298
Q8I3X4_PLAF7	444498 im	PNPH_BOVIN Fu	6021 inos	1A9S	2BSX	1B8N		Sc=5.90772, min distance = 2.58470
Q8I3X4_PLAF7	446728 CI	Q9GPQ4_TRYVI	444499 Fc	2FF1	1NW4	1KIE		Sc=6.26619, min distance = 2.35699
Q8I3X4_PLAF7	446728 CI	Q9GPQ4_TRYVI	6021 inos	1KIC	2BSX	1KIE		Sc=6.21246, min distance = 2.44160
Q8I3X4_PLAF7	447001 1l	PNPH_BOVIN Fu	6021 inos	1A9S	2BSX	1LVU		Sc=6.28746, min distance = 1.96929
Q8I3X4_PLAF7	50599 dic	PNPH_HUMAN Fu	444499 Fc	1RT9	1NW4	1V3Q		Sc=6.13759, min distance = 2.84929
Q8I3X4_PLAF7	5078682 C	PNPH_BOVIN Fu	6021 inos	1A9S	2BSX	2QPL		Sc=5.84152, min distance = 2.16024
Q8I3X4_PLAF7	6802 guar	PNPH_HUMAN Fu	444499 Fc	1RT9	1NW4	1RFG		Sc=6.33741, min distance = 2.61538
Q8I3X4_PLAF7	6802 guar	PNPH_HUMAN Fu	6021 inos	1RCT	2BSX	1RFG	0.99	Sc=6.30932, min distance = 2.81084
Q8I3X4_PLAF7	764 guani	PNPH_HUMAN Fu	444499 Fc	1RT9	1NW4	1V2H		Sc=5.82456, min distance = 2.49927
Q8I3X4_PLAF7	764 guani	PNPH_HUMAN Fu	6021 inos	1RCT	2BSX	1V2H		Sc=5.77739, min distance = 3.07719
Q8I3X4_PLAF7	8646 Guar	PNPH_HUMAN Fu	444499 Fc	1RT9	1NW4	1V41		Sc=5.80864, min distance = 2.25306
Q8I3X4_PLAF7	8646 Guar	PNPH_HUMAN Fu	6021 inos	1RCT	2BSX	1V41		Sc=5.75149, min distance = 2.71662

Q8IEL9_PLAF7	445971 CI	COMT_RAT Full	34756 Acy	2CL5	2PLW	1H1D	0.92	Sc=6.5628, min distance = 2.749121
Q8IFJ9_RIFPA	11957353	HBA_HORSE Ful	444124 HE	1Y8I	1YHU	1IWH	0.87	Sc=6.6039, min distance = 2.080648
Q8IFJ9_RIFPA	11957371	HMOX1_HUMAN Fu	444124 HE	1OZW	1YHU	1TWR		Sc=6.44179, min distance = 1.81997
Q8IFJ9_RIFPA	11957385	MYG_PHYCA Ful	444124 HE	1U7R	1YHU	2CMM		Sc=5.89717, min distance = 2.28113
Q8IFJ9_RIFPA	444207 He	HBB_HORSE Ful	444124 HE	1Y8I	1YHU	2ZLW	0.99	Sc=6.60064, min distance = 2.02502
Q8IFJ9_RIFPA	444207 He	PRXC_CALFU Fu	444124 HE	2CPO	1YHU	1CPO	0.99	Sc=6.58549, min distance = 2.13356
Q8IFJ9_RIFPA	446189 CI	HBG1_HUMAN Fu	444124 HE	1I3E	1YHU	1I3D	0.89	Sc=6.60282, min distance = 1.84224
Q8II73_PLAF7	24250 Oct	FMS1_YEAST Fu	1102 sper	3CN8	2PWP	3BI5		Sc=5.70563, min distance = 0.39206
Q8II73_PLAF7	656970 MT	MTNN_ECOLI Fu	439176 Me	1Z5O	2HTE	1Y6Q		Sc=6.3278, min distance = 1.521904
Q8IKK7_PLAF7	15942665	INHA_MYCTU Fu	5893 nadi	2H7I	2B4R	2H9I	0.88	Sc=6.41376, min distance = 1.803468
Q8IKK7_PLAF7	439153 Di	GALE_ECOLI Fu	5893 nadi	1UDC	2B4R	1UDB	0.79	Sc=6.73957, min distance = 1.834568
Q8IKK7_PLAF7	439153 Di	Q9BJJ9_PLAFA	5893 nadi	1UH5	2B4R	1V35	0.79	Sc=6.74693, min distance = 1.872415
Q8IKK7_PLAF7	445794 AD	ADHX_HUMAN Fu	5893 nadi	2FZW	2B4R	2FZE		Sc=5.65502, min distance = 2.06145
Q8IKK7_PLAF7	446050 CI	G3P_HUMAN Ful	5893 nadi	1U8F	2B4R	3GPD	0.95	Sc=6.77514, min distance = 1.82662
Q8IKK7_PLAF7	446288 NA	G3P_PALVE Ful	5893 nadi	1DSS	2B4R	1IHX		Sc=6.30706, min distance = 2.02621
Q8IKK7_PLAF7	449263 IN	INHA_MYCTU Fu	5893 nadi	2H7I	2B4R	2NV6	0.93	Sc=6.39721, min distance = 2.00443
Q8IKK7_PLAF7	6022 Ader	Q5SI02_THET8	5893 nadi	2BJK	2B4R	2BJA		Sc=6.27138, min distance = 2.502618
Q8J0D2_9ASCO	16741014	CARP_YEAST Fu	64689 bet	1FQ4	2EBS	1FQ7		Sc=5.63307, min distance = 2.027049
Q8J0D2_9ASCO	29435 1-D	CDGT_BACS0 Fu	64689 bet	1UKS	2EBS	1I75		Sc=5.84332, min distance = 2.02232
Q8J0D2_9ASCO	444809 Th	GUN2_THEFU Fu	64689 bet	2BOF	2EBS	2BOG	0.78	Sc=5.77993, min distance = 0.97459
Q8J0D2_9ASCO	445237 1,	AMY1_HORVU Fu	64689 bet	1RP9	2EBS	1P6W	0.77	Sc=5.82491, min distance = 0.63551
Q8J0D2_9ASCO	445999 CI	GUX6_HUMIN Fu	64689 bet	1OCN	2EBS	1OC7	0.77	Sc=5.78694, min distance = 0.96901
Q8J0D2_9ASCO	445999 CI	Q79G13_MYCTU	64689 bet	1UP0	2EBS	1UOZ	0.77	Sc=5.69038, min distance = 0.65288
Q8J0D2_9ASCO	447607 1d	GUN5_BACAG Fu	64689 bet	1W3L	2EBS	1OCQ		Sc=5.73598, min distance = 0.85602
Q8J0D2_9ASCO	447607 1d	Q79G13_MYCTU	64689 bet	1UP0	2EBS	1UP2		Sc=5.75684, min distance = 0.631495
Q8J0D2_9ASCO	5327083 C	GUN2_THEFU Fu	64689 bet	2BOF	2EBS	2BOD		Sc=5.77823, min distance = 0.968129
Q8J0D2_9ASCO	82313 1rd	Q9HYN5_PSEAE	64689 bet	1W8F	2EBS	1W8H		Sc=5.98919, min distance = 1.673942
Q8J307_METMP	444845 1d	RASH_HUMAN Fu	36735 Gpp	1CTQ	1WB3	1CLU	0.99	Sc=6.1964, min distance = 2.076161
Q8J307_METMP	445871 CI	RASH_HUMAN Fu	36735 Gpp	1CTQ	1WB3	1GNP		Sc=5.82151, min distance = 2.26748
Q8J307_METMP	6022 Ader	PARM_ECOLX Fu	36735 Gpp	2ZGZ	1WB3	1MWM	0.79	Sc=6.41376, min distance = 2.264598
Q8J307_METMP	6022 Ader	PARM_ECOLX Fu	8977 1dar	2ZGY	1WB1	1MWM	0.8	Sc=6.02534, min distance = 1.849009
Q8J307_METMP	93082 Gab	EFG_THETH Ful	8977 1dar	2BM0	1WB1	2J7K	0.99	Sc=6.03831, min distance = 2.15799
Q8J307_METMP	93082 Gab	FTSY_THEAQ Fu	36735 Gpp	2J7P	1WB3	1RJ9	0.99	Sc=6.54152, min distance = 1.910558
Q8J307_METMP	93082 Gab	RASH_HUMAN Fu	36735 Gpp	1CTQ	1WB3	121P	0.99	Sc=6.2028, min distance = 2.111165
Q8KES3_CHLTE	15942680	6PGD_LACLM Fu	5886 NADF	2IZ0	2BD0	2IZ1		Sc=6.29104, min distance = 2.086195
Q8KES3_CHLTE	440141 9i	G6PD_LEUME Fu	5886 NADF	1H9A	2BD0	1E7Y		Sc=6.46179, min distance = 2.023247
Q8KGE0_CHLTE	439176 Me	MTR1_RHOSH Fu	34756 Acy	1NW5	1X1A	1EG2	0.91	Sc=6.24607, min distance = 2.50493

Q8KGE0_CHLTE	446535 CI	HNMT_HUMAN Ful	439155 Ad	2AOT	1X1B	1JQE		0.95	Sc=6.50107, min distance = 2.38525
Q8KGE0_CHLTE	60961 ade	PIMT_PYRFU Ful	439155 Ad	1JG1	1X1B	1JG2		0.84	Sc=6.25591, min distance = 2.64341
Q8KGE0_CHLTE	65482 sir	ERM_BACSU Full	439155 Ad	1QAN	1X1B	1QAQ		0.88	Sc=6.54269, min distance = 2.34315
Q8KGE0_CHLTE	65482 sir	MCES_ENCCU Ful	439155 Ad	1RI1	1X1B	2HV9		0.88	Sc=6.53495, min distance = 2.52915
Q8KHZ8_NOCAE	159961 Le	SAHH_RAT Full=	60961 ade	1D4F	2OA1	1K0U		0.84	Sc=6.20603, min distance = 2.19657
Q8KHZ8_NOCAE	447864 2'	DEOD_ECOLI Ful	60961 ade	1PK7	2OA1	1PKE		0.96	Sc=6.23615, min distance = 0.99815
Q8KHZ8_NOCAE	5962 L-ly	THER_BACTH Ful	6305 L-tr	1THL	2E4G	1KEI			Sc=5.74975, min distance = 2.15059
Q8KHZ8_NOCAE	6021 inos	TMPC_TREPA Ful	60961 ade	2FQY	2OA1	2FQW		0.79	Sc=6.25226, min distance = 2.43727
Q8KHZ8_NOCAE	6057 L-ty	CHMU_YEAST Ful	6305 L-tr	5CSM	2E4G	4CSM			Sc=6.00072, min distance = 1.98291
Q8KHZ8_NOCAE	6083 aden	SYQ_ECOLI Full	60961 ade	1O0B	2OA1	1ZJW		0.96	Sc=6.33495, min distance = 2.25538
Q8KHZ8_NOCAE	6140 L-ph	APX_STRGR Full	6305 L-tr	1TF8	2E4G	1F2P			Sc=6.00501, min distance = 0.84454
Q8KHZ8_NOCAE	6140 L-ph	THER_BACTH Ful	6305 L-tr	1THL	2E4G	1OS0			Sc=5.85141, min distance = 2.21944
Q8KHZ8_NOCAE	6802 guar	A5KE01_PLAVI	60961 ade	2PGF	2OA1	2QVN		0.79	Sc=6.31685, min distance = 2.32172
Q8KHZ8_NOCAE	6802 guar	TMPC_TREPA Ful	60961 ade	2FQY	2OA1	2FQX		0.79	Sc=6.30932, min distance = 2.44840
Q8KHZ8_NOCAE	8975 2-Fl	SAHH_MYCTU Ful	60961 ade	3CE6	2OA1	2ZJ0		0.99	Sc=6.27292, min distance = 1.78773
Q8KHZ8_NOCAE	9570 6-Me	DEOD_ECOLI Ful	60961 ade	1PK7	2OA1	1PR4		0.88	Sc=6.28095, min distance = 1.93823
Q8KHZ8_NOCAE	97184 6-M	DEOD_ECOLI Ful	60961 ade	1PK7	2OA1	1OVG		0.9	Sc=6.19686, min distance = 1.81879
Q8KHZ8_NOCAE	9842606 2	ADK_HUMAN Full	60961 ade	1BX4	2OA1	2I6A			Sc=6.16412, min distance = 2.37096
Q8KI25_NOCAE	444502 CI	GSHR_HUMAN Ful	444188 CI	3DK9	2R0C	1BWC		0.98	Sc=6.13795, min distance = 2.15974
Q8KI25_NOCAE	444502 CI	TYTR_TRYCR Ful	444188 CI	1BZL	2R0C	1GXF		0.98	Sc=6.41008, min distance = 2.23728
Q8KN66_PSEAE	169266 1,	GALE_HUMAN Ful	5893 nadi	1HZJ	1SB8	1I3K	26.09	0.79	Sc=6.7945, min distance = 2.675307
Q8KN66_PSEAE	439153 Di	GALE_ECOLI Ful	5893 nadi	1UDC	1SB8	1UDB	29.32	0.79	Sc=6.34629, min distance = 2.62407
Q8KN66_PSEAE	439153 Di	Q9BJJ9_PLAFA	5893 nadi	1UH5	1SB8	1V35		0.79	Sc=6.78688, min distance = 2.35704
Q8KN66_PSEAE	444428 CI	ADH_DROLE Full	5893 nadi	1SBY	1SB8	1B16		0.77	Sc=6.84722, min distance = 1.92468
Q8KN66_PSEAE	445794 AD	ADHX_HUMAN Ful	5893 nadi	2FZW	1SB8	2FZE			Sc=6.20684, min distance = 2.29630
Q8KN66_PSEAE	449409 CI	GALE_ECOLI Ful	8629 UDP-	1LRL	1SB9	2UDP	29.32		Sc=6.49843, min distance = 2.14062
Q8KN66_PSEAE	6031 Uric	GALE_ECOLI Ful	8629 UDP-	1LRL	1SB9	1NAH	29.32	0.89	Sc=5.86376, min distance = 2.26708
Q8LGR3_MORNI	151504 D-	CONA_CANEN Ful	185698 al	1CVN	1XXR	1I3H			Sc=5.76396, min distance = 2.59431
Q8LPE0_CHLRE	446995 FM	CYB2_YEAST Ful	444243 FA	1KBI	1N9L	1LTD		0.77	Sc=6.13645, min distance = 1.96884
Q8MPM3_LEIME	124687 1r	ODP1_ECOLI Ful	1132 thia	1L8A	1R9J	1RP7		0.79	Sc=5.95531, min distance = 1.82598
Q8MPM3_LEIME	13294447	DCIP_AZOBR Ful	1132 thia	2NXW	1R9J	2Q5Q			Sc=6.11617, min distance = 1.01123
Q8MPM3_LEIME	444421 CI	MDLC_PSEPU Ful	1132 thia	1BFD	1R9J	2FWN		0.82	Sc=6.37109, min distance = 1.95421
Q8MPM3_LEIME	445886 CI	TKT1_YEAST Ful	1132 thia	1TRK	1R9J	1GPU		0.91	Sc=5.71864, min distance = 2.37767
Q8MPM3_LEIME	448721 N3	TKT1_YEAST Ful	1132 thia	1TRK	1R9J	1TKA			Sc=6.3729, min distance = 2.407336
Q8MPM3_LEIME	448722 1t	TKT1_YEAST Ful	1132 thia	1TRK	1R9J	1TKB		0.78	Sc=6.37953, min distance = 2.70990
Q8MPM3_LEIME	448723 1t	TKT1_YEAST Ful	1132 thia	1TRK	1R9J	1TKC		0.77	Sc=6.09103, min distance = 2.26812
Q8RBX6_THETN	11957363	HBA_HUMAN Full	444124 HE	1NQP	1U55	1RQA		0.81	Sc=6.66087, min distance = 1.86482

Q8RBX6_THETN	11957364	HMOX1_HUMAN Fu	444124 HE	1OZW	1U55	1S13	0.86	Sc=6.49883, min distance = 1.31800
Q8RBX6_THETN	11957371	HMOX1_HUMAN Fu	444124 HE	1OZW	1U55	1TWR		Sc=6.52176, min distance = 1.84643
Q8RBX6_THETN	11957385	MYG_PHYCA Ful	444124 HE	1U7R	1U55	2CMM		Sc=6.0605, min distance = 2.130189
Q8RBX6_THETN	444522 HE	NOS3_BOVIN Fu	444124 HE	1ZZS	1U55	2HX2	0.99	Sc=6.70795, min distance = 1.98038
Q8RLY5_LACHE	119182 Cl	DCK_HUMAN Ful	13730 dec	2ZI3	1S2G	2A7Q	0.97	Sc=6.19469, min distance = 1.85269
Q8RLY5_LACHE	13711 dec	DCK_HUMAN Ful	13730 dec	2ZI3	1S2G	2NO1		Sc=5.9681, min distance = 2.385561
Q8RLY5_LACHE	159354 To	DCK_HUMAN Ful	13730 dec	2ZI3	1S2G	2NO7		Sc=5.99243, min distance = 2.04831
Q8RLY5_LACHE	454194 Tr	DCK_HUMAN Ful	13730 dec	2ZI3	1S2G	2NO9		Sc=5.82051, min distance = 2.52968
Q8RLY5_LACHE	60750 gem	DCK_HUMAN Ful	13730 dec	2ZI3	1S2G	2NO0		Sc=6.00563, min distance = 1.97162
Q8RLY5_LACHE	60825 lam	DCK_HUMAN Ful	13730 dec	2ZI3	1S2G	2NOA		Sc=5.80598, min distance = 2.39141
Q8RLY5_LACHE	60877 Emt	DCK_HUMAN Ful	13730 dec	2ZI3	1S2G	2NO6		Sc=5.82993, min distance = 2.37035
Q8T8E9_9TRYP	18068 Udp	GALE_ECOLI Fu	6031 Urid	1NAH	1GY8	1A9Z	0.89	Sc=5.89804, min distance = 2.15683
Q8T8E9_9TRYP	6030 Urid	O87988_BORBR	6031 Urid	2PZM	1GY8	2PZL	0.99	Sc=6.28746, min distance = 2.50507
Q8T8E9_9TRYP	6131 cyti	KCY_DICDI Ful	6031 Urid	4UKD	1GY8	1QF9	0.82	Sc=6.27197, min distance = 2.05319
Q8T9Z7_PLAFA	23190 Dea	Q9GPQ4_TRYVI	6021 inos	1KIC	2BSX	1HP0		Sc=6.20975, min distance = 2.58298
Q8T9Z7_PLAFA	444498 in	PNPH_BOVIN Fu	6021 inos	1A9S	2BSX	1B8N		Sc=5.90772, min distance = 2.58470
Q8T9Z7_PLAFA	444499 Fo	PNPH_BOVIN Fu	6021 inos	1A9S	2BSX	1B8O		Sc=6.32468, min distance = 2.55170
Q8T9Z7_PLAFA	444499 Fo	Q9GPQ4_TRYVI	6021 inos	1KIC	2BSX	2FF1		Sc=6.31286, min distance = 1.91034
Q8T9Z7_PLAFA	446728 CI	Q9GPQ4_TRYVI	6021 inos	1KIC	2BSX	1KIE		Sc=6.21246, min distance = 2.44160
Q8T9Z7_PLAFA	447001 1l	PNPH_BOVIN Fu	6021 inos	1A9S	2BSX	1LVU		Sc=6.28746, min distance = 1.96929
Q8T9Z7_PLAFA	5078682 C	PNPH_BOVIN Fu	6021 inos	1A9S	2BSX	2QPL		Sc=5.84152, min distance = 2.16024
Q8T9Z7_PLAFA	6802 guar	PNPH_HUMAN Fu	6021 inos	1RCT	2BSX	1RFG	0.99	Sc=6.30932, min distance = 2.81084
Q8T9Z7_PLAFA	764 guani	PNPH_HUMAN Fu	6021 inos	1RCT	2BSX	1V2H		Sc=5.77739, min distance = 3.07719
Q8T9Z7_PLAFA	8646 Guar	PNPH_HUMAN Fu	6021 inos	1RCT	2BSX	1V41		Sc=5.75149, min distance = 2.71662
Q8U1K9_PYRFU	16740985	FRDA_SHEFR Fu	444188 CI	1M64	1XHC	1E39	0.93	Sc=6.4354, min distance = 2.442968
Q8U1K9_PYRFU	444502 CI	FRDA_SHEFR Fu	444188 CI	1M64	1XHC	1QJD	0.98	Sc=6.43576, min distance = 2.38258
Q8U1K9_PYRFU	444502 CI	TYTR_TRYCR Fu	444188 CI	1BZL	1XHC	1GXF	0.98	Sc=6.44184, min distance = 2.04408
Q8U1K9_PYRFU	448054 CI	FRDA_SHEFR Fu	444188 CI	1M64	1XHC	1Y0P	0.97	Sc=6.43459, min distance = 2.47755
Q8U306_PYRFU	11957393	FAK1_HUMAN Fu	6022 Aden	1MP8	1YQT	2ETM		Sc=6.04905, min distance = 2.63798
Q8U306_PYRFU	11957417	CDK2_HUMAN Fu	6022 Aden	1GY3	1YQT	2I40		Sc=6.26449, min distance = 1.68953
Q8U306_PYRFU	16214823	CDK2_HUMAN Fu	6022 Aden	1GY3	1YQT	2UZB		Sc=5.99345, min distance = 2.06134
Q8U306_PYRFU	16214825	CDK2_HUMAN Fu	6022 Aden	1GY3	1YQT	2UZE		Sc=6.16441, min distance = 2.34429
Q8U306_PYRFU	16214826	CDK2_HUMAN Fu	6022 Aden	1GY3	1YQT	2UZL		Sc=6.02378, min distance = 2.38590
Q8U306_PYRFU	23727982	CDK2_HUMAN Fu	6022 Aden	1GY3	1YQT	3BHU		Sc=6.12054, min distance = 1.82357
Q8U306_PYRFU	3543 1g5s	CDK2_HUMAN Fu	6022 Aden	1GY3	1YQT	1G5S		Sc=6.21294, min distance = 2.26257
Q8U306_PYRFU	398148 1e	CDK2_HUMAN Fu	6022 Aden	1GY3	1YQT	1E1X		Sc=5.78226, min distance = 2.09595
Q8U306_PYRFU	445940 O6	CDK2_HUMAN Fu	6022 Aden	1GY3	1YQT	1GZ8		Sc=5.99526, min distance = 1.76942

Q8U306_PYRFU	446090 1h	NDKC_DICDI Fu	6022 Aden	1KDN	1YQT	1HIY		0.97	Sc=6.3991, min distance = 1.917899
Q8U306_PYRFU	447962 1p	CDK2_HUMAN Fu	6022 Aden	1GY3	1YQT	2C5N			Sc=6.05388, min distance = 1.79934
Q8U306_PYRFU	4565 1h1r	CDK2_HUMAN Fu	6022 Aden	1GY3	1YQT	1H1R			Sc=6.17431, min distance = 1.71076
Q8U306_PYRFU	5957 Aden	Y059_METJA Fu	6022 Aden	2J9D	1YQT	2J9C		0.99	Sc=6.47235, min distance = 1.46126
Q8U306_PYRFU	6083 aden	PURP_METJA Fu	6022 Aden	2R7N	1YQT	2R7M		0.99	Sc=6.24091, min distance = 2.32523
Q8U306_PYRFU	6420139 C	CDK2_HUMAN Fu	6022 Aden	1GY3	1YQT	2C5V			Sc=5.89865, min distance = 2.10673
Q8U306_PYRFU	6918710 5	CDK2_HUMAN Fu	6022 Aden	1GY3	1YQT	3EJ1			Sc=5.96228, min distance = 2.69655
Q8U306_PYRFU	72194 2-C	ENPL_CANFA Fu	6022 Aden	1TC6	1YQT	1QYE		0.89	Sc=5.91707, min distance = 2.02117
Q8U306_PYRFU	8977 1dar	NDK_PYRHO Ful	6022 Aden	2DYA	1YQT	2DXE		0.8	Sc=6.39336, min distance = 2.20486
Q8U3I1_PYRFU	33113 gam	CHEA_THEMA Fu	91532 AMP	1I58	1G3R	1I59		0.99	Sc=6.00075, min distance = 2.03011
Q8U3I1_PYRFU	444564 AD	BIOD_ECOLI Fu	6022 Aden	1DAD	1G3Q	1BS1		0.91	Sc=6.01648, min distance = 2.43266
Q8U3I1_PYRFU	444842 CI	CDK2_HUMAN Fu	6022 Aden	1GY3	1G3Q	1CKP			Sc=5.62455, min distance = 2.06193
Q8U3I1_PYRFU	445966 O6	CDK2_HUMAN Fu	6022 Aden	1GY3	1G3Q	1H0V			Sc=6.25346, min distance = 2.01370
Q8U3I1_PYRFU	5957 Aden	BIOD_ECOLI Fu	6022 Aden	1DAD	1G3Q	1A82		0.99	Sc=6.05949, min distance = 2.44118
Q8U3I1_PYRFU	5957 Aden	NIFH1_AZOVI Fu	6022 Aden	1FP6	1G3Q	2C8V	37.5	0.99	Sc=6.05359, min distance = 2.30163
Q8U3I1_PYRFU	5957 Aden	PUR7_METJA Fu	6022 Aden	2YZL	1G3Q	2Z02		0.99	Sc=6.05169, min distance = 2.04980
Q8U3I1_PYRFU	5957 Aden	Q72H90_THET2 I	6022 Aden	2BEJ	1G3Q	2BEK		0.99	Sc=6.05688, min distance = 2.33917
Q8U3I1_PYRFU	6420138 2	CDK2_HUMAN Fu	6022 Aden	1GY3	1G3Q	2UUE			Sc=6.43793, min distance = 1.25138
Q8VP74_9GAMM	23831 HEE	XYNA_BACSU Fu	125409 be	2QZ3	1GQJ	2B45			Sc=5.63719, min distance = 1.84447
Q8VPD4_RHOOP	11987634	GLPD_ECOLI Fu	444188 CI	2QCU	2JAE	2R4E		0.97	Sc=6.89225, min distance = 2.12470
Q8VPD4_RHOOP	16741253	LSD1_HUMAN Fu	444188 CI	2DW4	2JAE	2UXN		0.95	Sc=6.89396, min distance = 2.43401
Q8VPD4_RHOOP	446013 1,	O28603_ARCFU I	444188 CI	1JNR	2JAE	1JNZ		0.94	Sc=6.8981, min distance = 2.387318
Q8VPD4_RHOOP	446013 1,	O28604_ARCFU I	444188 CI	1JNR	2JAE	1JNZ		0.94	Sc=6.89869, min distance = 2.41993
Q8VPD4_RHOOP	6420174 C	O28603_ARCFU I	444188 CI	1JNR	2JAE	2FJB		0.92	Sc=6.90316, min distance = 2.24687
Q8VPD4_RHOOP	6420174 C	O28604_ARCFU I	444188 CI	1JNR	2JAE	2FJB		0.92	Sc=6.90342, min distance = 2.23386
Q8VQF6_CITBR	11957360	CY1_BOVIN Ful	444098 HE	1L0N	1T2B	1NTM			Sc=6.79908, min distance = 2.26115
Q8VQF6_CITBR	11957385	MYG_PHYCA Ful	444098 HE	1A6M	1T2B	2CMM			Sc=6.19212, min distance = 2.37586
Q8VQF6_CITBR	16741183	CPXA_PSEPU Fu	444098 HE	1YRC	1T2B	2ZAW		0.97	Sc=6.58892, min distance = 2.27351
Q8VQF6_CITBR	446406 CZ	MYG_PHYCA Ful	444098 HE	1A6M	1T2B	1J3F			Sc=6.38624, min distance = 2.26897
Q8VQF6_CITBR	5326592 C	HMOX1_RAT Ful	444098 HE	1DVG	1T2B	1J2C		0.79	Sc=6.73506, min distance = 2.15344
Q8VQF6_CITBR	6420167 M	CPXA_PSEPU Fu	444098 HE	1YRC	1T2B	2FE6		0.86	Sc=6.76952, min distance = 2.06291
Q8WZK8_GEOCN	11957370	HMOX1_HUMAN Fu	444124 HE	1OZW	2D3Q	1TWN			Sc=6.65157, min distance = 1.74309
Q8WZK8_GEOCN	11957371	HMOX1_HUMAN Fu	444124 HE	1OZW	2D3Q	1TWR			Sc=6.65858, min distance = 1.89730
Q8WZK8_GEOCN	11957385	MYG_PHYCA Ful	444124 HE	1U7R	2D3Q	2CMM			Sc=6.22571, min distance = 2.23644
Q8WZK8_GEOCN	444207 He	PER_COPCI Ful	444124 HE	1LY9	2D3Q	1LY8		0.99	Sc=6.78058, min distance = 2.01091
Q8WZK8_GEOCN	444522 HE	BFR_ECOLI Ful	444124 HE	1BFR	2D3Q	1BCF		0.99	Sc=6.77956, min distance = 2.02761
Q8WZK8_GEOCN	444522 HE	HBA_PAGBE Ful	444124 HE	1S5X	2D3Q	1PBX		0.99	Sc=6.7742, min distance = 2.366667

Q8WZK8_GEOCN	444522 HE	HBB_PAGBE Full	444124 HE	1S5X	2D3Q	1PBX	0.99	Sc=6.77611, min distance = 2.22048
Q8X123_9AGAR	445340 1e	ELBP_ECOLX Fu	439353 be	1LT3	2IHO	1EFI		Sc=6.25014, min distance = 2.16583
Q8X123_9AGAR	446996 GA	Q9HYN5_PSEAE	439353 be	1W8F	2IHO	1W8H	0.89	Sc=5.69574, min distance = 1.83537
Q8X1D8_FUSOX	11492134	FMS1_YEAST Fu	1103 sper	1XPQ	2C12	3CNP		Sc=6.10788, min distance = 1.53757
Q8XUA5_RALSO	151504 D-	CONA_CANEN Fu	101798 Me	5CNA	1UQX	1I3H		Sc=5.75163, min distance = 2.38018
Q8XUA5_RALSO	24310 fru	Q9HYN5_PSEAE	101798 Me	2JDN	1UQX	1OVP	0.9	Sc=5.75522, min distance = 2.39224
Q8XUA5_RALSO	439554 fu	Q9HYN5_PSEAE	101798 Me	2JDN	1UQX	1UZV	0.96	Sc=5.66077, min distance = 2.32365
Q8XUA5_RALSO	445948 D-	Q9HYN5_PSEAE	101798 Me	2JDN	1UQX	1OXC		Sc=5.75873, min distance = 1.82099
Q8XUA5_RALSO	64947 alp	LEC_PEA Full=	185698 al	1RIN	2CHH	1HKD	0.94	Sc=5.7749, min distance = 2.636743
Q8XUA5_RALSO	84265 1ax	LECA_ARTIN Fu	101798 Me	1WS5	1UQX	1M26		Sc=6.04124, min distance = 1.91572
Q8ZJW4_SALTY	449233 CI	O53831_MYCTU	87642 coe	1P0H	2CNT	2C27		Sc=6.41762, min distance = 1.81900
Q8ZKR2_SALTY	33113 gam	IGF1R_HUMAN F	91532 AMF	1K3A	1TZ6	1JQH	0.99	Sc=6.46705, min distance = 2.04044
Q8ZKR2_SALTY	33113 gam	KIF1A_MOUSE F	91532 AMF	1I6I	1TZ6	1VFV	0.99	Sc=6.47866, min distance = 2.02597
Q8ZKR2_SALTY	33113 gam	PURT_ECOLI Fu	91532 AMF	1KJI	1TZ6	1EYZ	0.99	Sc=6.47599, min distance = 1.67594
Q8ZKR2_SALTY	443200 C1	ACK1_HUMAN Fu	91532 AMF	1U54	1TZ6	1U4D		Sc=6.21812, min distance = 2.89332
Q8ZKR2_SALTY	444564 AD	BIOD_ECOLI Fu	91532 AMF	1DAG	1TZ6	1BS1	0.91	Sc=6.41775, min distance = 2.13729
Q8ZKR2_SALTY	5957 Aden	BIOD_ECOLI Fu	91532 AMF	1DAG	1TZ6	1A82	0.99	Sc=6.46205, min distance = 2.09048
Q8ZKR2_SALTY	6022 Aden	BIOD_ECOLI Fu	91532 AMF	1DAG	1TZ6	1DAD	0.99	Sc=6.41927, min distance = 2.09015
Q8ZKR2_SALTY	6022 Aden	GLPK_ECOLI Fu	91532 AMF	1GLL	1TZ6	1GLB	0.99	Sc=5.96788, min distance = 1.65077
Q8ZKR2_SALTY	6022 Aden	PURP_METJA Fu	91532 AMF	2R7K	1TZ6	2R7N	0.99	Sc=6.41454, min distance = 2.19709
Q8ZKR2_SALTY	6022 Aden	RBSK_ECOLI Fu	91532 AMF	1GQT	1TZ6	1RKD	0.99	Sc=6.44468, min distance = 1.39416
Q8ZKR2_SALTY	6083 ader	PURP_METJA Fu	91532 AMF	2R7K	1TZ6	2R7M	0.97	Sc=6.35386, min distance = 2.59504
Q8ZVF7_PYRAE	10041129	SAHH_MYCTU Fu	60961 ade	3CE6	2GL0	3DHY	0.89	Sc=5.94822, min distance = 2.37495
Q8ZVF7_PYRAE	24180721	PIM1_HUMAN Fu	60961 ade	1YI4	2GL0	3C4E		Sc=5.78541, min distance = 1.95474
Q8ZVF7_PYRAE	24851689	PIM1_HUMAN Fu	60961 ade	1YI4	2GL0	3CY2		Sc=6.17936, min distance = 1.81370
Q8ZVF7_PYRAE	5327148 C	KAPCA_MOUSE F	60961 ade	1FMO	2GL0	2ERZ		Sc=5.93185, min distance = 1.89407
Q8ZVF7_PYRAE	6802 guar	A5KE01_PLAVI	60961 ade	2PGF	2GL0	2QVN	0.79	Sc=6.08716, min distance = 2.36235
Q8ZVF7_PYRAE	9570 6-Me	ADK_TOXGO Full	60961 ade	1DGM	2GL0	2AA0	0.88	Sc=6.0163, min distance = 2.258819
Q8ZYK1_PYRAE	3044933 A	ENPL_CANFA Fu	6022 Aden	1TC6	1FNN	1U0Y	0.81	Sc=6.42117, min distance = 2.07867
Q8ZYK1_PYRAE	444564 AD	BIOD_ECOLI Fu	6022 Aden	1DAD	1FNN	1BS1	0.91	Sc=6.44573, min distance = 2.42134
Q8ZYK1_PYRAE	444852 CI	ATPB_BOVIN Fu	6022 Aden	2CK3	1FNN	1COW	0.9	Sc=6.02572, min distance = 2.00254
Q8ZYK1_PYRAE	4565 1h1r	CDK2_HUMAN Fu	6022 Aden	1GY3	1FNN	1H1R		Sc=6.41036, min distance = 1.27227
Q8ZYK1_PYRAE	5957 Aden	MUTS_ECOLI Fu	6022 Aden	1OH7	1FNN	1W7A	0.99	Sc=6.55847, min distance = 0.90853
Q8ZYK1_PYRAE	6132 Cyti	ECX2_PYRAB Fu	6022 Aden	2PO0	1FNN	2PO2		Sc=6.33113, min distance = 2.13203
Q8ZYK1_PYRAE	91532 AMF	BIOD_ECOLI Fu	6022 Aden	1DAD	1FNN	1DAG	0.99	Sc=6.06032, min distance = 2.54791
Q90282_CROAT	4369384 1	LEF_BACAN Full	132519 Ga	1PWU	2DW1	1YQY		Sc=6.13198, min distance = 2.09503
Q920E6_MOUSE	11368987	ESR1_HUMAN Fu	5757 estr	1QKT	1JGL	2QGW		Sc=5.72298, min distance = 2.17497

Q92456_TRIRE	101798 Me	LEC1_LATOC Fu	185698 al	1LOG	1SZN	1LOB	0.94	Sc=5.73279, min distance = 2.49699
Q92456_TRIRE	151504 D-	CVN_NOSEL Ful	185698 al	2RDK	1SZN	2PYS		Sc=5.673, min distance = 2.6431233
Q92456_TRIRE	439764 be	ARAF_ECOLI Fu	439353 be	8ABP	1T0O	6ABP	0.93	Sc=5.6567, min distance = 2.183693
Q92456_TRIRE	445184 1,	Q6PYX1_HUMAN	185698 al	1ZLS	1SZN	1ZLW	0.89	Sc=5.64592, min distance = 2.75004
Q92456_TRIRE	7027 gluc	Q72KX2_THET2	439353 be	2B3F	1T0O	2B3B		Sc=5.9108, min distance = 2.425837
Q93EK7_NEIME	18068 Udp	GGTA1_BOVIN Fu	445795 1g	2VFZ	1G9R	2VS5	0.98	Sc=6.67354, min distance = 2.19092
Q93EK7_NEIME	6030 Urid	GGTA1_BOVIN Fu	445795 1g	2VFZ	1G9R	1G8O	0.86	Sc=6.32687, min distance = 2.35578
Q93IG4_MYCMS	119119 Di	DPOLB_HUMAN Fu	65091 dCT	1MQ3	2JAQ	2FMP	0.93	Sc=6.28429, min distance = 2.38825
Q93IG4_MYCMS	15993 dAT	DPOL_BPR69 Fu	65091 dCT	1Q9Y	2JAQ	2OZS		Sc=6.47986, min distance = 2.16367
Q93IG4_MYCMS	448381 2'	DPOLL_HUMAN Fu	65091 dCT	2PFP	2JAQ	2PFN	0.82	Sc=6.40442, min distance = 2.30757
Q93IG4_MYCMS	454194 Tr	DCK_HUMAN Ful	13945 Dec	2QRN	2JAT	2NO9		Sc=5.91376, min distance = 2.43260
Q93IG4_MYCMS	60877 Emt	DCK_HUMAN Ful	13945 Dec	2QRN	2JAT	2NO6		Sc=5.96567, min distance = 2.32818
Q93IG4_MYCMS	64968 dTT	DPO42_SULSO Fu	65091 dCT	2ASD	2JAQ	1S0O	0.75	Sc=6.461, min distance = 1.9058840
Q93IG4_MYCMS	64968 dTT	DPOLL_HUMAN Fu	65091 dCT	2PFP	2JAQ	2BCV	0.75	Sc=6.45867, min distance = 2.34746
Q93IG4_MYCMS	64968 dTT	DPOL_BPR69 Fu	65091 dCT	1Q9Y	2JAQ	3CQ8	0.75	Sc=6.45867, min distance = 2.36087
Q93IG4_MYCMS	64968 dTT	POLI_HUMAN Fu	65091 dCT	2DPI	2JAQ	1T3N	0.75	Sc=6.46298, min distance = 2.00879
Q93IG4_MYCMS	656930 DD	DPO42_SULSO Fu	65091 dCT	2ASD	2JAQ	1S9F	0.92	Sc=6.31653, min distance = 1.86114
Q93KV2_STRVR	6030 Urid	GLMU_HAEIN Fu	6031 Urid	2V0K	2IV3	2V0J	0.99	Sc=6.33087, min distance = 1.89337
Q93KV2_STRVR	6030 Urid	O87988_BORBR	6031 Urid	2PZM	2IV3	2PZL	0.99	Sc=6.32623, min distance = 2.09676
Q93KV2_STRVR	6030 Urid	PYRH_ECOLI Fu	6031 Urid	2BND	2IV3	2BNE	0.99	Sc=6.32623, min distance = 2.06867
Q93KV2_STRVR	8629 UDP-	Q89399_PBCV1	6031 Urid	2P73	2IV3	2P72	0.89	Sc=5.90358, min distance = 1.94760
Q93LY7_9ACTO	11987634	GLPD_ECOLI Fu	444188 CI	2QCU	2QA1	2R4E	0.97	Sc=6.87337, min distance = 2.02756
Q93LY7_9ACTO	16740985	FRDA_SHEFR Fu	444188 CI	1M64	2QA1	1E39	0.93	Sc=6.87358, min distance = 2.24736
Q93LY7_9ACTO	444502 CI	FRDA_SHEFR Fu	444188 CI	1M64	2QA1	1QJD	0.98	Sc=6.87405, min distance = 2.09733
Q93LY7_9ACTO	444502 CI	GSHR_HUMAN Fu	444188 CI	3DK9	2QA1	1BWC	0.98	Sc=6.87844, min distance = 2.23024
Q93LY7_9ACTO	444502 CI	TYTR_TRYCR Fu	444188 CI	1BZL	2QA1	1GXF	0.98	Sc=6.43595, min distance = 2.44584
Q93LY7_9ACTO	445794 AD	PHHY_PSEFL Fu	444188 CI	1PBE	2QA1	2PHH		Sc=6.6578, min distance = 2.129669
Q93LY7_9ACTO	446013 1,	O28604_ARCFU	444188 CI	1JNR	2QA1	1JNZ	0.94	Sc=6.43876, min distance = 2.05943
Q93LY7_9ACTO	448054 CI	FRDA_SHEFR Fu	444188 CI	1M64	2QA1	1Y0P	0.97	Sc=6.87125, min distance = 2.07307
Q93LY7_9ACTO	449465 CI	GSHR_HUMAN Fu	444188 CI	3DK9	2QA1	3GRT	0.98	Sc=6.43876, min distance = 2.34774
Q94KL8_PODPE	169266 1,	GALE_HUMAN Fu	5893 nadi	1HZJ	2BGM	1I3K	0.79	Sc=6.29069, min distance = 2.61503
Q94KL8_PODPE	439153 Di	Q9BJJ9_PLAFA	5893 nadi	1UH5	2BGM	1V35	0.79	Sc=5.98666, min distance = 2.36409
Q94KL8_PODPE	447767 pi	INHA_MYCTU Fu	5893 nadi	2H7I	2BGM	1P44		Sc=6.06754, min distance = 1.65898
Q94KL8_PODPE	449263 IN	INHA_MYCTU Fu	5893 nadi	2H7I	2BGM	2NV6	0.93	Sc=6.08176, min distance = 2.19905
Q94M05_9VIRU	23653516	CDK2_HUMAN Fu	6022 Aden	1GY3	1W44	2R3G		Sc=6.2183, min distance = 2.017012
Q94M05_9VIRU	2608 1jsv	CDK2_HUMAN Fu	6022 Aden	1GY3	1W44	1JSV		Sc=5.93108, min distance = 2.00746
Q94M05_9VIRU	398148 1e	CDK2_HUMAN Fu	6022 Aden	1GY3	1W44	1E1X		Sc=6.12035, min distance = 1.29100

Q94M05_9VIRU	445966 O6	CDK2_HUMAN Fu	6022 Aden	1GY3	1W44	1H0V			Sc=6.17756, min distance = 1.365745
Q94M05_9VIRU	447916 ac	RIO1_ARCFU Fu	6022 Aden	1ZTH	1W44	1ZTF		0.95	Sc=5.71164, min distance = 2.27498
Q94M05_9VIRU	447955 1p	CDK2_HUMAN Fu	6022 Aden	1GY3	1W44	1PXI			Sc=5.6805, min distance = 1.997113
Q94M05_9VIRU	5327097 2	CDK2_HUMAN Fu	6022 Aden	1GY3	1W44	2BTS			Sc=5.74093, min distance = 2.02047
Q94M05_9VIRU	6132 Cyti	ECX1_PYRAB Fu	6022 Aden	2PO0	1W44	2PO2			Sc=6.18432, min distance = 2.23556
Q94M05_9VIRU	6338562 C	MYS2_DICDI Fu	6022 Aden	1VOM	1W44	1D0Y			Sc=5.92869, min distance = 2.49078
Q94M05_9VIRU	6338566 C	MYS2_DICDI Fu	6022 Aden	1VOM	1W44	1D1C			Sc=5.95605, min distance = 2.50243
Q94M05_9VIRU	91532 AMI	MTNK_BACSU Fu	6022 Aden	2OLC	1W44	2PUL		0.99	Sc=5.82701, min distance = 2.61293
Q94M05_9VIRU	91532 AMI	PDXK_SHEEP Fu	6022 Aden	1RFU	1W44	1RFT		0.99	Sc=5.63584, min distance = 2.47076
Q95VY4_AEDAE	3744 Indo	AAT_ECOLI Ful	1053 pyri	2Q7W	1YIY	1AHF			Sc=5.97665, min distance = 0.75664
Q95VY4_AEDAE	4369488 E	AAT_ECOLI Ful	1053 pyri	2Q7W	1YIY	1X29		0.89	Sc=5.7773, min distance = 1.544435
Q95VY4_AEDAE	444292 PI	AAT_ECOLI Ful	1053 pyri	2Q7W	1YIY	1ARG		0.9	Sc=6.02056, min distance = 1.73392
Q95VY4_AEDAE	444293 PI	AAT_ECOLI Ful	1053 pyri	2Q7W	1YIY	1X28		0.89	Sc=6.02164, min distance = 1.44595
Q95VY4_AEDAE	444293 PI	HIS8_ECOLI Fu	1053 pyri	1FG7	1YIY	1GEY		0.89	Sc=5.73352, min distance = 2.39264
Q95VY4_AEDAE	6140 L-ph	KAT1_HUMAN Fu	1053 pyri	1W7N	1YIY	1W7M			Sc=5.80916, min distance = 1.88066
Q95WL3_TRYCR	11266583	FPPS_HUMAN Fu	2088 alen	2F92	1YHM	2OPM	39.07		Sc=6.02126, min distance = 2.15070
Q95WL3_TRYCR	193703 Di	IDI1_HUMAN Fu	1195 isop	2ICK	1YHM	2I6K			Sc=5.61741, min distance = 2.23325
Q95WL3_TRYCR	68740 Zol	FPPS_HUMAN Fu	2088 alen	2F92	1YHM	2F9K	39.07		Sc=5.84709, min distance = 2.19953
Q95ZD7_LEIME	11987747	PGMB_LACLA Fu	447467 G1	1O03	2I55	1O08			Sc=6.14103, min distance = 2.07829
Q95ZD7_LEIME	444212 tr	ACON_BOVIN Fu	311 citri	1C96	2I54	1ACO			Sc=5.74788, min distance = 2.17634
Q967M2_GIALA	6030 Uric	UPP_TOXGO Ful	7339 PRPF	1JLS	1L1R	1UPU			Sc=6.28824, min distance = 2.07179
Q96UT7_TRAVE	439554 fu	FIBB_HUMAN Fu	444205 CI	1FZF	1KYA	2OYH			Sc=5.73638, min distance = 0.79519
Q96XN9_SULTO	101544 2'	RNAS1_BOVIN F	6022 Aden	1O0H	2DCN	1ROB			Sc=6.34633, min distance = 0.96833
Q96XN9_SULTO	23727981	CDK2_HUMAN Fu	6022 Aden	1GY3	2DCN	3BHT			Sc=6.14616, min distance = 1.86924
Q96XN9_SULTO	445840 di	CDK2_HUMAN Fu	6022 Aden	1GY3	2DCN	1GII			Sc=6.38925, min distance = 1.80804
Q96XN9_SULTO	4565 1h1r	CDK2_HUMAN Fu	6022 Aden	1GY3	2DCN	1H1R			Sc=6.42499, min distance = 1.34108
Q96XN9_SULTO	6031 Uric	DCK_HUMAN Ful	6022 Aden	1P5Z	2DCN	2ZIA			Sc=5.88541, min distance = 2.41777
Q96XN9_SULTO	6031 Uric	RIR1_YEAST Fu	6022 Aden	2CVX	2DCN	2CVV			Sc=6.33041, min distance = 2.10012
Q96XN9_SULTO	6083 ader	PURP_METJA Fu	6022 Aden	2R7N	2DCN	2R7M		0.99	Sc=6.32934, min distance = 2.87895
Q96XN9_SULTO	66535 3'-	RNAS1_BOVIN F	6022 Aden	1O0H	2DCN	1RPF			Sc=6.33643, min distance = 1.08052
Q96XN9_SULTO	6852187 2	O57883_PYRHO	6022 Aden	1WNL	2DCN	2DTH		0.89	Sc=6.14196, min distance = 1.45459
Q96XN9_SULTO	91532 AMI	NIFH1_AZOVI F	6022 Aden	1FP6	2DCN	2AFK		0.99	Sc=6.52335, min distance = 1.77456
Q96Y14_SULTO	440317 AT	BCKD_RAT Full	6022 Aden	1GKZ	2E2P	1GJV		0.99	Sc=5.80915, min distance = 2.07965
Q96Y14_SULTO	444564 AD	MYS2_DICDI Fu	6022 Aden	1VOM	2E2P	1W9I		0.91	Sc=6.42391, min distance = 1.96703
Q96Y14_SULTO	445940 O6	CDK2_HUMAN Fu	6022 Aden	1GY3	2E2P	1GZ8			Sc=6.23395, min distance = 1.57473
Q96Y14_SULTO	446521 1j	RNAS1_BOVIN F	6022 Aden	1O0H	2E2P	1JN4		0.87	Sc=5.94933, min distance = 2.08892
Q96Y14_SULTO	447004 CI	MYS2_DICDI Fu	6022 Aden	1VOM	2E2P	1LVK			Sc=6.48239, min distance = 2.13429

Q96Y14_SULTO	447955 1p	CDK2_HUMAN Fu	6022 Aden	1GY3	2E2P	1PXI		Sc=5.77922, min distance = 1.98546
Q96Y14_SULTO	5957 Ader	ARP2_BOVIN Fu	6022 Aden	2P9I	2E2P	1TYQ	0.99	Sc=5.81929, min distance = 1.99886
Q96Y14_SULTO	5957 Ader	ARP3_BOVIN Fu	6022 Aden	2P9I	2E2P	1TYQ	0.99	Sc=6.01094, min distance = 2.39192
Q96Y14_SULTO	5957 Ader	DDL_THET8 Ful	6022 Aden	2ZDH	2E2P	2ZDQ	0.99	Sc=5.80666, min distance = 1.93297
Q96Y14_SULTO	5957 Ader	HSP7F_YEAST Fu	6022 Aden	3C7N	2E2P	3D2F	0.99	Sc=5.82401, min distance = 2.71378
Q96Y14_SULTO	5957 Ader	MALK_ECOLI Fu	6022 Aden	2AWN	2E2P	1Q12	0.99	Sc=5.79483, min distance = 2.09981
Q96Y14_SULTO	5957 Ader	MUTS_ECOLI Fu	6022 Aden	1OH7	2E2P	1W7A	0.99	Sc=5.69748, min distance = 2.43473
Q96Y14_SULTO	5957 Ader	PDK2_HUMAN Fu	6022 Aden	2BU8	2E2P	2BU2	0.99	Sc=5.83102, min distance = 2.16476
Q96Y14_SULTO	6031 Uric	DCK_HUMAN Ful	6022 Aden	1P5Z	2E2P	2ZIA		Sc=5.8339, min distance = 2.301821
Q96Y14_SULTO	6083 ader	PURP_METJA Fu	6022 Aden	2R7N	2E2P	2R7M	0.99	Sc=6.39065, min distance = 2.24040
Q96Y14_SULTO	656946 CI	RNAS1_BOVIN Fu	6022 Aden	1O0H	2E2P	1W4Q		Sc=6.10997, min distance = 2.17034
Q96Y14_SULTO	6804 guar	NDK_PYRHO Ful	6022 Aden	2DYA	2E2P	2DXF	0.8	Sc=6.41806, min distance = 2.29759
Q96Y14_SULTO	8977 1dar	NDK_PYRHO Ful	6022 Aden	2DYA	2E2P	2DXE	0.8	Sc=5.99611, min distance = 2.04827
Q96Y14_SULTO	8977 1dar	PARM_ECOLX Fu	6022 Aden	1MWM	2E2P	2ZGY	0.8	Sc=5.99204, min distance = 2.38485
Q96Y14_SULTO	91532 AMP	PURP_METJA Fu	6022 Aden	2R7N	2E2P	2R7K	0.99	Sc=5.87339, min distance = 2.12674
Q96Y14_SULTO	91557 1tm	Q94M05_9VIRU	6022 Aden	1W44	2E2P	1W48	0.99	Sc=6.12631, min distance = 2.13330
Q96Z61_SULTO	15942680	6PGD_LACLM Fu	5886 NADP	2IZ0	2GGS	2IZ1		Sc=6.27697, min distance = 1.12943
Q96Z61_SULTO	440141 9i	DHB1_HUMAN Fu	5886 NADP	1QYV	2GGS	1QYW		Sc=6.44521, min distance = 2.09010
Q973K9_SULTO	444502 CI	PHR_SYNP6 Ful	444188 CI	1TEZ	2E0I	1OWM	0.98	Sc=6.43156, min distance = 1.96854
Q974C9_SULTO	5326566 l	Q9WZW1_THEMA	444243 FA	1T6Y	2D37	1S4M	0.82	Sc=6.27262, min distance = 1.45639
Q977U7_HALME	15342951	ALBU_HUMAN Fu	311 citri	1TF0	2B5W	3B9L		Sc=6.36847, min distance = 0.45568
Q97DM1_CLOAB	854023 Ep	Q8WSF8_APLCA	23831 HEP	2BR7	1Y7B	2BYQ		Sc=6.01283, min distance = 0.68391
Q97G05_CLOAB	24850913	NOS1_RAT Full	444243 FA	1TLL	2IG6	3B3M		Sc=6.17335, min distance = 0.11802
Q97G05_CLOAB	5326566 l	BLVRB_HUMAN Fu	444243 FA	1HE4	2IG6	1HE5	0.82	Sc=6.23265, min distance = 2.27495
Q97G05_CLOAB	5326566 l	Q9WZW1_THEMA	444243 FA	1T6Y	2IG6	1S4M	0.82	Sc=6.27488, min distance = 2.39987
Q97GQ1_CLOAB	169266 1,	GALE_HUMAN Fu	5893 nadi	1HZJ	1VL0	1I3K	0.79	Sc=6.75312, min distance = 2.17544
Q97GQ1_CLOAB	444428 CI	ADH_DROLE Ful	5893 nadi	1SBY	1VL0	1B16	0.77	Sc=6.8301, min distance = 1.933130
Q97NR6_STRPN	24901727	RHAMD_SALTY Fu	444305 L-	3D46	1SQS	3CXO		Sc=5.75061, min distance = 2.29983
Q97NR6_STRPN	24901728	RHAMD_SALTY Fu	444305 L-	3D46	1SQS	3CXO		Sc=5.87458, min distance = 1.89761
Q97NW2_STRPN	444915 Gl	CDGT2_BACCI Fu	79025 alp	1D3C	2I58	1CXL	0.85	Sc=5.72902, min distance = 0.69485
Q97NW2_STRPN	445237 1,	AMY1_HORVU Fu	79025 alp	1RP8	2I58	1P6W	0.77	Sc=5.95114, min distance = 0.58103
Q97NW2_STRPN	446685 Va	CDGT2_BACCI Fu	79025 alp	1D3C	2I58	1KCK		Sc=5.95505, min distance = 0.85020
Q97NW2_STRPN	446685 Va	NEPU1_THEVU Fu	79025 alp	1UH4	2I58	1UH3		Sc=5.85651, min distance = 2.10072
Q97NW2_STRPN	5326754 S	PHSM_ECOLI Fu	79025 alp	1L5W	2I58	1QM5	0.88	Sc=5.60997, min distance = 2.35855
Q97NW2_STRPN	7027 gluc	Q9C171_PIREQ	79025 alp	1GWM	2I58	1W8T		Sc=5.82924, min distance = 2.29401
Q97NW2_STRPN	84265 1ax	TRFL_BOVIN Fu	79025 alp	2DWJ	2I58	2AYS		Sc=6.09579, min distance = 2.32061
Q97S03_STRPN	16741044	PYRDA_LACLC Fu	444243 FA	1JUB	2B67	1JRB		Sc=5.79959, min distance = 2.17387

Q97T10_STRPN	29435 1-D	Q2PS28_9PSED I	64689 bet	2PWF	2POK	2PWD		Sc=5.74087, min distance = 2.82455!
Q97T10_STRPN	445999 CI	Q79G13_MYCTU I	64689 bet	1UP0	2POK	1UOZ	0.77	Sc=5.75206, min distance = 0.590618
Q97T10_STRPN	447607 1c	Q79G13_MYCTU I	64689 bet	1UP0	2POK	1UP2		Sc=5.60208, min distance = 2.84412(
Q97U29_SULSO	10109823	PIM1_HUMAN Fu!	6083 aden	1YXU	2VAR	3CY3		Sc=6.45062, min distance = 1.62054!
Q97U29_SULSO	11986113	PIM1_HUMAN Fu!	33113 gam	1XR1	2VAR	2BZH		Sc=6.54539, min distance = 1.813328
Q97U29_SULSO	11986113	PIM1_HUMAN Fu!	6083 aden	1YXU	2VAR	2BZH		Sc=6.49431, min distance = 1.47133!
Q97U29_SULSO	11986114	PIM1_HUMAN Fu!	6083 aden	1YXU	2VAR	2BZI		Sc=6.55074, min distance = 2.54532!
Q97U29_SULSO	1400 nche	RET_HUMAN Full	6083 aden	2IVT	2VAR	2IVV		Sc=6.14422, min distance = 1.24442(
Q97U29_SULSO	15993 dAT	PRIM_BPT7 Full	33113 gam	1E0J	2VAR	1CR2	0.97	Sc=5.96313, min distance = 2.38798!
Q97U29_SULSO	16058647	PIM1_HUMAN Fu!	6083 aden	1YXU	2VAR	2OI4		Sc=6.51032, min distance = 2.43554(
Q97U29_SULSO	16750062	CSK2A_MAIZE Fu	33113 gam	1LP4	2VAR	2OXD		Sc=5.67564, min distance = 2.12596!
Q97U29_SULSO	188966 dA	HSLU_ECOLI Fu!	33113 gam	1E94	2VAR	1G4A	0.97	Sc=6.36681, min distance = 2.238538
Q97U29_SULSO	2396 bisi	PIM1_HUMAN Fu!	6083 aden	1YXU	2VAR	1XWS		Sc=6.34637, min distance = 2.49816!
Q97U29_SULSO	24820122	PIM1_HUMAN Fu!	6083 aden	1YXU	2VAR	3BWF		Sc=6.56344, min distance = 2.51059(
Q97U29_SULSO	2519 caff	PYGM_RABIT Fu!	6083 aden	8GPB	2VAR	1GFZ		Sc=5.69752, min distance = 2.17022!
Q97U29_SULSO	3064778 H	ROCK1_HUMAN Fu	33113 gam	2V55	2VAR	2ETK		Sc=6.05783, min distance = 2.21001!
Q97U29_SULSO	3547 Fasu	ROCK1_HUMAN Fu	33113 gam	2V55	2VAR	2ESM		Sc=5.973, min distance = 2.3172196!
Q97U29_SULSO	3973 nche	PIM1_HUMAN Fu!	33113 gam	1XR1	2VAR	1YI3		Sc=5.93862, min distance = 1.77224!
Q97U29_SULSO	440317 AT	SRC_CHICK Full	6083 aden	3DQX	2VAR	3DQW	0.97	Sc=5.97561, min distance = 1.37531!
Q97U29_SULSO	444564 AD	MYS2_DICDI Fu!	33113 gam	1MMN	2VAR	1W9I	0.91	Sc=6.39313, min distance = 2.77336!
Q97U29_SULSO	444852 CI	ATPB_BOVIN Fu!	33113 gam	2JDI	2VAR	1COW	0.91	Sc=6.45745, min distance = 2.18782!
Q97U29_SULSO	445708 AM	NADE_ECOLI Fu!	6083 aden	1WXI	2VAR	1WXE	0.91	Sc=6.31254, min distance = 2.77346!
Q97U29_SULSO	446724 AM	NUDT5_HUMAN Fu	6083 aden	2DSD	2VAR	3BM4	0.92	Sc=5.76443, min distance = 2.04064(
Q97U29_SULSO	447832 AD	ACSA_SALTY Fu!	6083 aden	2P2F	2VAR	1PG4	0.99	Sc=6.38098, min distance = 2.089978
Q97U29_SULSO	448042 2e	ROCK1_HUMAN Fu	33113 gam	2V55	2VAR	2ETR		Sc=6.09899, min distance = 2.07161!
Q97U29_SULSO	448895 AD	UMPK_YEAST Fu!	6083 aden	1UKZ	2VAR	1UKY	0.99	Sc=6.41066, min distance = 2.232118
Q97U29_SULSO	5326976 1	CSK2A_MAIZE Fu	33113 gam	1LP4	2VAR	1ZOE		Sc=5.72082, min distance = 2.124558
Q97U29_SULSO	5326977 1	CSK2A_MAIZE Fu	33113 gam	1LP4	2VAR	1ZOG		Sc=5.65524, min distance = 2.04444(
Q97U29_SULSO	5326978 1	CSK2A_MAIZE Fu	33113 gam	1LP4	2VAR	1ZOH		Sc=5.71726, min distance = 2.06532!
Q97U29_SULSO	5327121 I	PIM1_HUMAN Fu!	6083 aden	1YXU	2VAR	2C3I		Sc=6.23651, min distance = 2.02587!
Q97U29_SULSO	5327148 C	IPKA_RABIT Fu!	33113 gam	1CDK	2VAR	2ERZ		Sc=6.21035, min distance = 1.67017(
Q97U29_SULSO	6076 Cycl	PDE10_HUMAN Fu	6083 aden	2OUN	2VAR	2OUR	0.99	Sc=6.27415, min distance = 2.62181(
Q97U29_SULSO	60961 ade	PIM1_HUMAN Fu!	33113 gam	1XR1	2VAR	1YI4	0.93	Sc=6.27193, min distance = 2.12141!
Q97U29_SULSO	611002 Op	PIM1_HUMAN Fu!	33113 gam	1XR1	2VAR	1YXX		Sc=6.1463, min distance = 1.599775(
Q97U29_SULSO	611002 Op	PIM1_HUMAN Fu!	6083 aden	1YXU	2VAR	1YXX		Sc=6.14112, min distance = 2.27366!
Q97U29_SULSO	6338561 C	MYS2_DICDI Fu!	33113 gam	1MMN	2VAR	1D0X		Sc=6.21158, min distance = 2.40200(
Q97U29_SULSO	65110 AIC	PURP_METJA Fu!	6083 aden	2R7M	2VAR	2R7K	0.79	Sc=5.79123, min distance = 2.95496!

Q97U29_SULSO	65110 AIC	YL28_SCHPO Fu	6083 aden	2OOX	2VAR	2QRE	0.79	Sc=6.288, min distance = 2.2315685
Q97U29_SULSO	656964 1y	PDE4D_HUMAN F	6083 aden	1TB7	2VAR	1Y2C		Sc=5.90171, min distance = 2.91648
Q97U29_SULSO	656969 1y	PDE4B_HUMAN F	6083 aden	1ROR	2VAR	1Y2J		Sc=6.18335, min distance = 2.42181
Q97U29_SULSO	657135 Op	PIM1_HUMAN Fu	6083 aden	1YXU	2VAR	1YXV		Sc=5.82952, min distance = 2.64357
Q97U29_SULSO	6804 guar	NDK_PYRHO Ful	6083 aden	2DXD	2VAR	2DXF	0.8	Sc=6.34281, min distance = 2.60401
Q97U29_SULSO	8582 Inos	PYGM_RABIT Fu	6083 aden	8GPB	2VAR	2QN7	0.79	Sc=6.32965, min distance = 2.55713
Q97UY8_SULSO	1540 1h1c	CDK2_HUMAN Fu	6022 Aden	1GY3	1OXU	1H1Q		Sc=5.9805, min distance = 1.616666
Q97UY8_SULSO	23727981	CDK2_HUMAN Fu	6022 Aden	1GY3	1OXU	3BHT		Sc=5.88629, min distance = 2.24777
Q97UY8_SULSO	3973 nche	PIM1_HUMAN Fu	33113 gam	1XR1	1OXV	1YI3		Sc=5.80723, min distance = 2.51200
Q97UY8_SULSO	445940 O6	CDK2_HUMAN Fu	6022 Aden	1GY3	1OXU	1GZ8		Sc=5.77462, min distance = 2.61703
Q97UY8_SULSO	447649 1c	CDK2_HUMAN Fu	6022 Aden	1GY3	1OXU	1OI9		Sc=6.1139, min distance = 2.005715
Q97UY8_SULSO	4565 1h1r	CDK2_HUMAN Fu	6022 Aden	1GY3	1OXU	1H1R		Sc=5.98786, min distance = 2.03507
Q97UY8_SULSO	5327133 C	CDK2_HUMAN Fu	6022 Aden	1GY3	1OXU	2C6K		Sc=5.95417, min distance = 1.86212
Q97UY8_SULSO	5327135 D	CDK2_HUMAN Fu	6022 Aden	1GY3	1OXU	2C6M		Sc=5.94964, min distance = 1.79088
Q97UY8_SULSO	6420138 2	CDK2_HUMAN Fu	6022 Aden	1GY3	1OXU	2UUE		Sc=6.15791, min distance = 1.29431
Q97UY8_SULSO	72194 2-C	ENPL_CANFA Fu	6022 Aden	1TC6	1OXU	1QYE	0.89	Sc=5.85344, min distance = 2.26659
Q97Y08_SULSO	444564 AD	BIOD_ECOLI Fu	6022 Aden	1DAD	2FNA	1BS1	0.91	Sc=6.48248, min distance = 2.26341
Q97Y08_SULSO	444842 CI	CDK2_HUMAN Fu	6022 Aden	1GY3	2FNA	1CKP		Sc=5.6204, min distance = 1.737930
Q97Y08_SULSO	447916 ac	RIO1_ARCFU Fu	6022 Aden	1ZTH	2FNA	1ZTF	0.95	Sc=6.30411, min distance = 2.44368
Q97Y08_SULSO	448505 AD	NDKC_DICDI Fu	6022 Aden	1KDN	2FNA	1S5Z	0.93	Sc=6.51341, min distance = 1.93871
Q97Y08_SULSO	5957 Aden	ARP3_BOVIN Fu	6022 Aden	2P9I	2FNA	1TYQ	0.99	Sc=6.533, min distance = 2.0677219
Q97Y08_SULSO	5957 Aden	BIOD_ECOLI Fu	6022 Aden	1DAD	2FNA	1A82	0.99	Sc=6.52632, min distance = 1.18771
Q97Y08_SULSO	5957 Aden	MUTS_ECOLI Fu	6022 Aden	1OH7	2FNA	1W7A	0.99	Sc=6.54477, min distance = 1.31997
Q97Y08_SULSO	5957 Aden	RK_BOVIN Full	6022 Aden	3C4Z	2FNA	3C4W	0.99	Sc=6.514, min distance = 1.9150271
Q97Y08_SULSO	60961 ade	IPKA_RABIT Fu	6022 Aden	1JBP	2FNA	1FMO	0.95	Sc=6.30607, min distance = 2.12114
Q97Y08_SULSO	60961 ade	SKY1_YEAST Fu	6022 Aden	1Q8Y	2FNA	1Q97	0.95	Sc=6.31289, min distance = 2.00664
Q97Y08_SULSO	91532 AMP	BIOD_ECOLI Fu	6022 Aden	1DAD	2FNA	1DAG	0.99	Sc=6.55253, min distance = 1.24794
Q97Y08_SULSO	91532 AMP	NIFH1_AZOVI F	6022 Aden	1FP6	2FNA	2AFK	0.99	Sc=6.57034, min distance = 1.87269
Q9A1S9_STRP1	15942680	6PGD_LACLM Fu	5886 NADP	2IZ0	2AHR	2IZ1		Sc=6.32102, min distance = 1.25228
Q9ADP7_STRCO	439709 be	SCNB_THITI Fu	444305 L-	2DXC	2D6Y	2DD4		Sc=5.75061, min distance = 0.86713
Q9AE70_PSEPU	439153 Di	GALE_ECOLI Fu	5893 nadi	1UDC	2Q2V	1UDB	0.79	Sc=6.77582, min distance = 2.47828
Q9AE70_PSEPU	439153 Di	Q9BJJ9_PLAFA	5893 nadi	1UH5	2Q2V	1V35	0.79	Sc=6.77582, min distance = 2.41897
Q9AE70_PSEPU	444427 CI	ADH_DROLE Ful	5893 nadi	1SBY	2Q2V	1B15	0.76	Sc=6.82418, min distance = 1.66096
Q9AE70_PSEPU	444428 CI	ADH_DROLE Ful	5893 nadi	1SBY	2Q2V	1B16	0.77	Sc=6.85221, min distance = 1.88789
Q9AE70_PSEPU	449263 IN	INHA_MYCTU Fu	5893 nadi	2H7I	2Q2V	2NV6	0.93	Sc=6.87608, min distance = 1.94754
Q9AE70_PSEPU	6022 Aden	Q5SI02_THET8	5893 nadi	2BJK	2Q2V	2BJA		Sc=6.27417, min distance = 1.79174
Q9AVR2_9DIPS	444260 FC	RICI_RICCO Fu	95054 Pte	1BR6	1HWP	1FMP		Sc=5.90339, min distance = 2.50305

Q9AVR2_9DIPS	446280 CI	RICI_RICCO Fu	95054 Pte	1BR6	1HWP	1IFU			Sc=6.20657, min distance = 2.53308
Q9BH77_PLAFA	169266 1,	GALE_HUMAN Fu	5893 nadi	1HZJ	2O2Y	1I3K		0.79	Sc=6.33994, min distance = 2.17103
Q9BH77_PLAFA	447049 OA	NPD_THEMA Ful	5893 nadi	2H4H	2O2Y	2H4J			Sc=6.68606, min distance = 1.63886
Q9BHB0_9DIPT	97538 s-(	GSTP1_MOUSE Fu	444104 II	1GLP	1V2A	1GLQ			Sc=5.79656, min distance = 1.69263
Q9BIH3_ANOGA	241868 4-	AAUB_ALCFA Fu	1150 tryp	2AGY	2PQL	2HKR			Sc=5.8508, min distance = 2.255500
Q9BIH3_ANOGA	29147 4-N	AAUB_ALCFA Fu	1150 tryp	2AGY	2PQL	2HJ4			Sc=5.75114, min distance = 1.28728
Q9BIH3_ANOGA	4657 Homo	AAUB_ALCFA Fu	1150 tryp	2AGY	2PQL	2HKR			Sc=5.61499, min distance = 2.56634
Q9BIH3_ANOGA	681 dopam	PH4H_HUMAN Fu	5814 nore	4PAH	2QEO	5PAH		0.93	Sc=5.84946, min distance = 2.21750
Q9BJJ9_PLAFA	169266 1,	GALE_HUMAN Fu	5893 nadi	1HZJ	1UH5	1I3K		0.79	Sc=6.3198, min distance = 2.378424
Q9BJJ9_PLAFA	444427 CI	ADH_DROLE Ful	5893 nadi	1SBY	1UH5	1B15		0.76	Sc=6.81721, min distance = 2.14195
Q9BJJ9_PLAFA	446288 NA	G3P_PALVE Ful	5893 nadi	1DSS	1UH5	1IHX			Sc=6.34497, min distance = 0.67202
Q9BJJ9_PLAFA	447049 OA	NPD_THEMA Ful	5893 nadi	2H4H	1UH5	2H4J			Sc=6.19716, min distance = 2.03710
Q9BJJ9_PLAFA	5274976 5	INHA_MYCTU Fu	5564 tric	2B35	1UH5	2B36	32.62		Sc=5.95438, min distance = 0.94756
Q9F1X5_LACSN	159354 To	DCK_HUMAN Ful	6022 Aden	1P5Z	2CDU	2NO7			Sc=5.89597, min distance = 2.73817
Q9F1X5_LACSN	20279 Cla	DCK_HUMAN Ful	6022 Aden	1P5Z	2CDU	2ZIA		0.91	Sc=6.04323, min distance = 2.81573
Q9F1X5_LACSN	444564 AD	BIOD_ECOLI Fu	6022 Aden	1DAD	2CDU	1BS1		0.91	Sc=6.41614, min distance = 2.03855
Q9F1X5_LACSN	447955 1p	CDK2_HUMAN Fu	6022 Aden	1GY3	2CDU	1PXI			Sc=5.62666, min distance = 2.07031
Q9F1X5_LACSN	4565 1h1r	CDK2_HUMAN Fu	6022 Aden	1GY3	2CDU	1H1R			Sc=6.07177, min distance = 2.26606
Q9F1X5_LACSN	489519 AI	DCK_HUMAN Ful	6022 Aden	1P5Z	2CDU	2ZI4		0.92	Sc=6.03862, min distance = 2.69880
Q9F1X5_LACSN	6031 Uric	DCK_HUMAN Ful	6022 Aden	1P5Z	2CDU	2ZIA			Sc=6.33113, min distance = 2.06776
Q9F1X5_LACSN	8977 1dar	PARM_ECOLX Fu	6022 Aden	1MWM	2CDU	2ZGY		0.8	Sc=5.99741, min distance = 1.66723
Q9F1X5_LACSN	91532 AMP	BIOD_ECOLI Fu	6022 Aden	1DAD	2CDU	1DAG		0.99	Sc=6.04631, min distance = 2.06014
Q9F5K6_STRVR	21945395	SETD7_HUMAN Fu	34756 Acy	1N6A	1X7P	1XQH		0.88	Sc=6.49446, min distance = 2.09790
Q9F5K6_STRVR	445762 5'	MTTA_THEAQ Fu	34756 Acy	2ADM	1X7P	2IH2		0.93	Sc=6.40452, min distance = 2.02507
Q9F5K6_STRVR	445971 CI	METJ_ECOLI Fu	34756 Acy	1CMC	1X7P	1MJL		0.92	Sc=6.0746, min distance = 2.133797
Q9FCA6_STRCO	11957363	HBA_HUMAN Ful	444124 HE	1NQP	1S1F	1RQA		0.81	Sc=6.73737, min distance = 1.63247
Q9FCA6_STRCO	11957370	HMOX1_HUMAN Fu	444124 HE	1OZW	1S1F	1TWN			Sc=6.61549, min distance = 1.97352
Q9FCA6_STRCO	11957371	HMOX1_HUMAN Fu	444124 HE	1OZW	1S1F	1TWR			Sc=6.65858, min distance = 1.85638
Q9FCA6_STRCO	11957385	MYG_PHYCA Ful	444124 HE	1U7R	1S1F	2CMM			Sc=6.1386, min distance = 2.157318
Q9FCA6_STRCO	125409 be	Q09LX0_BACST		78165 MES	2EXH	1SE6	2EXJ			Sc=5.70268, min distance = 0.25041
Q9FCA6_STRCO	444207 He	CP119_SULTO Fu	444124 HE	3B4X	1S1F	1UE8		0.99	Sc=6.72444, min distance = 2.30793
Q9FCA6_STRCO	444207 He	HBA_HORSE Ful	444124 HE	1Y8I	1S1F	2ZLW		0.99	Sc=6.73651, min distance = 2.17066
Q9FCA6_STRCO	444522 HE	C5B3_AMYOR Fu	444124 HE	1LGF	1S1F	1LFK	33.67	0.99	Sc=6.73366, min distance = 2.13668
Q9FCA6_STRCO	444522 HE	HBA_PAGBE Ful	444124 HE	1S5X	1S1F	1PBX		0.99	Sc=6.71957, min distance = 2.06055
Q9FCA6_STRCO	444522 HE	MYG_PIG Full=M	444124 HE	1MYH	1S1F	1MNI		0.99	Sc=6.75217, min distance = 2.02921
Q9FCA6_STRCO	444522 HE	NOS3_BOVIN Fu	444124 HE	1ZZS	1S1F	2HX2		0.99	Sc=6.67249, min distance = 2.42640
Q9FCA6_STRCO	446676 CI	MMP8_HUMAN Fu	78165 MES	1ZS0	1SE6	1KBC			Sc=5.66744, min distance = 2.06601

Q9FCA6_STRCO	5326592 C	HMOX1_RAT Ful	444124 HE	1J02	1S1F	1J2C		0.84	Sc=6.67722, min distance = 2.33895
Q9FVF8_ULEEU	446578 1r	MBL1_RAT Full	439554 fu	3KMB	1QOT	1KWW		0.96	Sc=5.62916, min distance = 2.48005
Q9FVF8_ULEEU	448294 CI	BGAT_HUMAN Fu	439353 be	1ZIZ	1DZQ	1R7U		0.9	Sc=5.94991, min distance = 2.15951
Q9GPQ4_TRYVI	11673379	PNPH_HUMAN Fu	6021 inos	1RCT	1KIC	3BGS			Sc=6.28617, min distance = 2.02435
Q9GPQ4_TRYVI	122671 9-	PNPH_BOVIN Fu	444499 Fc	1B8O	2FF1	1A9P		0.89	Sc=6.35612, min distance = 2.27093
Q9GPQ4_TRYVI	122671 9-	PNPH_BOVIN Fu	6021 inos	1A9S	1KIC	1A9P			Sc=6.29634, min distance = 2.60817
Q9GPQ4_TRYVI	21704 Vic	DEOD_ECOLI Fu	6021 inos	1PR0	1KIC	1PW7		0.79	Sc=6.23092, min distance = 1.88860
Q9GPQ4_TRYVI	444498 im	PNPH_BOVIN Fu	6021 inos	1A9S	1KIC	1B8N			Sc=6.36301, min distance = 2.15979
Q9GPQ4_TRYVI	445732 CI	PNPH_BOVIN Fu	444499 Fc	1B8O	2FF1	1FXU			Sc=6.32877, min distance = 1.88322
Q9GPQ4_TRYVI	445732 CI	PNPH_BOVIN Fu	6021 inos	1A9S	1KIC	1FXU		0.82	Sc=6.26431, min distance = 2.15787
Q9GPQ4_TRYVI	447722 9-	DEOD_ECOLI Fu	6021 inos	1PR0	1KIC	1OUM			Sc=6.33424, min distance = 1.66868
Q9GPQ4_TRYVI	447916 ac	DEOD_ECOLI Fu	6021 inos	1PR0	1KIC	1PR6		0.79	Sc=6.27292, min distance = 2.18510
Q9GPQ4_TRYVI	50599 dic	PNPH_HUMAN Fu	444499 Fc	1RT9	2FF1	1V3Q			Sc=6.12312, min distance = 2.54490
Q9GPQ4_TRYVI	50599 dic	PNPH_HUMAN Fu	6021 inos	1RCT	1KIC	1V3Q		0.96	Sc=6.05182, min distance = 2.57625
Q9GPQ4_TRYVI	5078682 C	PNPH_BOVIN Fu	444499 Fc	1B8O	2FF1	2QPL			Sc=5.79273, min distance = 2.15699
Q9GPQ4_TRYVI	5078682 C	PNPH_BOVIN Fu	6021 inos	1A9S	1KIC	2QPL			Sc=5.73591, min distance = 2.28176
Q9GPQ4_TRYVI	60961 ade	DEOD_ECOLI Fu	6021 inos	1PR0	1KIC	1PK7		0.79	Sc=6.27072, min distance = 1.87356
Q9GPQ4_TRYVI	60961 ade	TMPC_TREPA Fu	6021 inos	2FQW	1KIC	2FQY		0.79	Sc=6.29064, min distance = 2.06653
Q9GPQ4_TRYVI	6102683 C	PNPH_HUMAN Fu	444499 Fc	1RT9	2FF1	1YRY			Sc=6.33985, min distance = 1.65088
Q9GPQ4_TRYVI	72443 for	DEOD_ECOLI Fu	6021 inos	1PR0	1KIC	1A69			Sc=6.30124, min distance = 2.40021
Q9GPQ4_TRYVI	764 guani	PNPH_HUMAN Fu	6021 inos	1RCT	1KIC	1V2H			Sc=5.61226, min distance = 2.69661
Q9GPQ4_TRYVI	8646 Guar	PNPH_HUMAN Fu	6021 inos	1RCT	1KIC	1V41			Sc=5.64403, min distance = 2.18419
Q9GPQ4_TRYVI	8975 2-Fl	DEOD_ECOLI Fu	6021 inos	1PR0	1KIC	1PK9		0.78	Sc=6.28698, min distance = 2.56632
Q9GPQ4_TRYVI	97184 6-M	DEOD_ECOLI Fu	6021 inos	1PR0	1KIC	1OVG			Sc=6.1719, min distance = 2.349370
Q9GT92_CRYPV	11987786	LDH_PLAFD Ful	5893 nadi	1T2D	2FM3	1T2E	44.04	0.77	Sc=6.76327, min distance = 2.77944
Q9GT92_CRYPV	165230 1a	G6PD_LEUME Fu	5893 nadi	1H94	2FM3	2DPG			Sc=5.6782, min distance = 2.040460
Q9GT92_CRYPV	439153 Di	ADH1B_HUMAN Fu	5893 nadi	1U3U	2FM3	1DEH		0.79	Sc=6.75993, min distance = 2.34381
Q9GT92_CRYPV	439153 Di	ADH1E_HORSE Fu	5893 nadi	1MGO	2FM3	2JHF		0.79	Sc=6.75027, min distance = 2.55422
Q9GT92_CRYPV	439153 Di	CTBP1_RAT Ful	5893 nadi	1HKU	2FM3	1HL3		0.79	Sc=6.76446, min distance = 2.10254
Q9GT92_CRYPV	439153 Di	GALE_ECOLI Fu	5893 nadi	1UDC	2FM3	1UDB		0.79	Sc=6.77675, min distance = 2.07108
Q9GT92_CRYPV	440516 CI	MDH_THETH Ful	5893 nadi	1BMD	2FM3	1BDM		0.79	Sc=6.76812, min distance = 2.60672
Q9GT92_CRYPV	446288 NA	G3P_PALVE Ful	5893 nadi	1DSS	2FM3	1IHX			Sc=6.7634, min distance = 2.064048
Q9GT92_CRYPV	6102709 A	LDH_PLAFD Ful	5893 nadi	1T2D	2FM3	2A94	44.04	0.88	Sc=6.74897, min distance = 2.86521
Q9HJ28_THEAC	446857 7-	COBT_SALTY Fu	121992 ni	1L5O	1YTK	1L5L			Sc=5.81261, min distance = 2.12302
Q9HJ28_THEAC	446858 7-	COBT_SALTY Fu	121992 ni	1L5O	1YTK	1L5M			Sc=5.81799, min distance = 2.10194
Q9HK51_THEAC	11987635	ADH_DROLE Ful	5893 nadi	1SBY	2DTE	1B2L	26.55	0.76	Sc=6.41006, min distance = 2.14884
Q9HK51_THEAC	439153 Di	Q9BJJ9_PLAFA I	5893 nadi	1UH5	2DTE	1V35		0.79	Sc=6.32675, min distance = 2.52082

Q9HK51_THEAC	445794 AI	ADHX_HUMAN Fu	5893 nadi	2FZW	2DTE	2FZE			Sc=6.61113, min distance = 2.14986(
Q9HU22_PSEAE	124823 6g	PYGM_RABIT Fu	65533 cor	3GPB	1G0R	6GPB		0.91	Sc=6.02126, min distance = 2.16350
Q9HU22_PSEAE	13711 dec	DNK_DROME Ful	5789 thym	1OT3	1G0R	2VP5			Sc=6.09218, min distance = 2.70828
Q9HU22_PSEAE	13712 dec	TYSY_ECOLI Fu	9700 thym	1TYS	1FXO	1BDU		0.83	Sc=6.12058, min distance = 2.04240
Q9HU22_PSEAE	15993 dAT	NRDD_BPT4 Ful	64968 dTT	1H79	1G2V	1H7A			Sc=6.4769, min distance = 2.001356
Q9HU22_PSEAE	164628 de	RMLA1_ECOLI F	65533 cor	1H5R	1G0R	1H5T	77.11		Sc=6.03467, min distance = 0.88450(
Q9HU22_PSEAE	1865 6-hy	KITH_HHV11 Fu	9700 thym	1VTK	1FXO	1E2M			Sc=5.96318, min distance = 2.13061
Q9HU22_PSEAE	24860521	PYGM_RABIT Fu	65533 cor	3GPB	1G0R	2QRM			Sc=6.31821, min distance = 2.02985
Q9HU22_PSEAE	24860522	PYGM_RABIT Fu	65533 cor	3GPB	1G0R	2QRP			Sc=6.32555, min distance = 2.27843
Q9HU22_PSEAE	24860523	PYGM_RABIT Fu	65533 cor	3GPB	1G0R	2QRQ			Sc=6.22288, min distance = 2.43765(
Q9HU22_PSEAE	24864075	PYGM_RABIT Fu	65533 cor	3GPB	1G0R	2QRG			Sc=6.23452, min distance = 1.91152
Q9HU22_PSEAE	24864076	PYGM_RABIT Fu	65533 cor	3GPB	1G0R	2QRH			Sc=6.16963, min distance = 2.55087
Q9HU22_PSEAE	444320 2'	TYSY_ECOLI Fu	9700 thym	1TYS	1FXO	1AOB		0.81	Sc=6.04399, min distance = 1.98475
Q9HU22_PSEAE	445211 9-	KITH_HHV11 Fu	5789 thym	1P7C	1G0R	1E2I			Sc=5.98089, min distance = 1.96276
Q9HU22_PSEAE	445213 HY	KITH_HHV11 Fu	5789 thym	1P7C	1G0R	1E2N			Sc=6.41183, min distance = 1.88402
Q9HU22_PSEAE	445723 1-	PYGM_RABIT Fu	65533 cor	3GPB	1G0R	1P4H			Sc=6.16463, min distance = 2.06044(
Q9HU22_PSEAE	446600 1k	PYGM_RABIT Fu	65533 cor	3GPB	1G0R	1K06			Sc=6.26681, min distance = 2.14381
Q9HU22_PSEAE	447413 NT	PYGM_RABIT Fu	65533 cor	3GPB	1G0R	1NOJ			Sc=5.86465, min distance = 2.50998(
Q9HU22_PSEAE	447770 CG	PYGM_RABIT Fu	65533 cor	3GPB	1G0R	1P4G			Sc=5.97621, min distance = 2.24095
Q9HU22_PSEAE	449489 Io	KITH_HHV11 Fu	5789 thym	1P7C	1G0R	3VTK		0.84	Sc=6.26593, min distance = 1.18782
Q9HU22_PSEAE	449554 CI	PYGM_RABIT Fu	65533 cor	3GPB	1G0R	5GPB		0.82	Sc=5.99765, min distance = 2.17949
Q9HU22_PSEAE	514815 BE	DNK_DROME Ful	64968 dTT	1ZM7	1G2V	2VQS		0.82	Sc=6.25106, min distance = 1.64766
Q9HU22_PSEAE	65091 dCT	DPOLL_HUMAN F	64968 dTT	2BCV	1G2V	2PFP		0.75	Sc=6.37566, min distance = 1.89856
Q9HU22_PSEAE	65103 dGT	RIR1_YEAST Fu	64968 dTT	2CVW	1G2V	2CVX			Sc=6.48327, min distance = 1.72448
Q9HU22_PSEAE	656930 DI	DPO42_SULSO F	64968 dTT	1S0O	1G2V	1S9F			Sc=6.28746, min distance = 1.71018
Q9HU22_PSEAE	657042 CH	PYGM_RABIT Fu	65533 cor	3GPB	1G0R	1XKX			Sc=6.11408, min distance = 1.78815
Q9HU22_PSEAE	657043 CH	PYGM_RABIT Fu	65533 cor	3GPB	1G0R	1XL0			Sc=5.94699, min distance = 1.89402
Q9HV14_PSEAE	164795 N(	RBCMT_PEA Ful	23831 HEP	1MLV	2I6C	2H2J			Sc=5.71884, min distance = 1.94883
Q9HV14_PSEAE	854023 Ep	Q8WSF8_APLCA	23831 HEP	2BR7	2I6C	2BYQ			Sc=5.81832, min distance = 1.86900
Q9HYN5_PSEAE	151504 D-	CONA_CANEN Fu	101798 Me	5CNA	2JDN	1I3H			Sc=5.81999, min distance = 2.35387
Q9HYN5_PSEAE	151504 D-	CONA_CANEN Fu	185698 al	1CVN	1OVS	1I3H			Sc=5.73005, min distance = 2.08150
Q9HYN5_PSEAE	151504 D-	CVN_NOSEL Ful	185698 al	2RDK	1OVS	2PYS			Sc=5.72381, min distance = 2.44899
Q9HYN5_PSEAE	16741034	Q9XEI3_HORVD	185698 al	1X38	1OVS	1IEV		0.82	Sc=5.673, min distance = 2.0810619
Q9HYN5_PSEAE	17106 L-f	ACES_MOUSE Fu	447053 CI	2HA2	1W8H	2HA0		0.85	Sc=5.61221, min distance = 2.24468
Q9HYN5_PSEAE	445184 1,	NRAM_I56A2 Fu	439680 be	1V0Z	1OUR	1W1X		0.89	Sc=5.79423, min distance = 1.11512(
Q9HYN5_PSEAE	445184 1,	Q6PYX1_HUMAN	185698 al	1ZLS	1OVS	1ZLW		0.89	Sc=5.74975, min distance = 2.33550
Q9I4V0_PSEAE	16741044	PYRDA_LACLC F	444243 FA	1JUB	2GJL	1JRB			Sc=6.63267, min distance = 2.48031

Q9I4V0_PSEAE	446995 FM	CYB2_YEAST Fu	444243 FA	1KBI	2GJL	1LTD	0.77	Sc=6.62501, min distance = 2.31580
Q9I4V0_PSEAE	448076 CI	CYB2_YEAST Fu	444243 FA	1KBI	2GJL	1SZG	0.76	Sc=6.63699, min distance = 2.04435
Q9I509_PSEAE	16741044	PYRDA_LACLC Fu	444243 FA	1JUB	1ZWL	1JRB		Sc=6.1574, min distance = 2.492376
Q9I509_PSEAE	448076 CI	CYB2_YEAST Fu	444243 FA	1KBI	1ZWL	1SZG	0.76	Sc=6.15102, min distance = 1.371998
Q9I509_PSEAE	90207 Hex	WRBA_ECOLI Fu	444243 FA	3B6I	1ZWL	3B6M		Sc=5.64483, min distance = 2.09795
Q9JL75_MOUSE	854023 Ep	Q8WSF8_APLCA	62551 Pen	2BYN	1P7K	2BYQ		Sc=5.78163, min distance = 2.227038
Q9K1N1_NEIMB	440141 9i	DHB1_HUMAN Fu	5886 NADF	1QYV	2AG8	1QYW		Sc=5.9177, min distance = 2.189150
Q9K1N1_NEIMB	440141 9i	G6PD_LEUME Fu	5886 NADF	1H9A	2AG8	1E7Y		Sc=5.91877, min distance = 2.30948
Q9KFR4_BACHD	15600 DEC	BACR_HALSA Fu	79025 alp	1DZE	2C3W	1XJI		Sc=5.60337, min distance = 0.545488
Q9KFR4_BACHD	21678292	PYGM_RABIT Fu	79025 alp	1H5U	2C3W	3BD8		Sc=6.08072, min distance = 2.18425
Q9KFR4_BACHD	444139 CI	AMYP_PIG Full	79025 alp	1HX0	2C3W	1PIG		Sc=5.64458, min distance = 1.41243
Q9KFR4_BACHD	444139 CI	CDGT2_BACCI Fu	79025 alp	1D3C	2C3W	1DTU		Sc=5.80993, min distance = 1.08634
Q9KFR4_BACHD	444456 1b	PYGM_RABIT Fu	79025 alp	1H5U	2C3W	1B4D		Sc=6.11876, min distance = 1.97473
Q9KFR4_BACHD	445382 2p	PYGM_RABIT Fu	79025 alp	1H5U	2C3W	1WW2		Sc=5.9098, min distance = 2.364908
Q9KFR4_BACHD	445722 1-	PYGM_RABIT Fu	79025 alp	1H5U	2C3W	1FU7		Sc=5.95472, min distance = 1.96280
Q9KFR4_BACHD	446564 LI	BACR_HALSA Fu	79025 alp	1DZE	2C3W	1JV7		Sc=5.78222, min distance = 0.72270
Q9KFR4_BACHD	447413 NT	PYGM_RABIT Fu	79025 alp	1H5U	2C3W	1NOJ		Sc=5.82301, min distance = 1.93721
Q9KFR4_BACHD	656959 GI	PYGM_RABIT Fu	79025 alp	1H5U	2C3W	1XC7		Sc=5.98708, min distance = 2.25513
Q9KFR4_BACHD	657042 CH	PYGM_RABIT Fu	79025 alp	1H5U	2C3W	1XKX		Sc=6.15905, min distance = 2.123768
Q9KFR4_BACHD	657043 CH	PYGM_RABIT Fu	79025 alp	1H5U	2C3W	1XL0		Sc=6.00531, min distance = 1.90442
Q9KFR4_BACHD	6914619 C	PYGM_RABIT Fu	79025 alp	1H5U	2C3W	2FFR		Sc=6.05246, min distance = 2.41035
Q9KFR4_BACHD	82313 1rc	LEC_ERYCO Ful	79025 alp	1FYU	2C3W	1AX2		Sc=5.96611, min distance = 1.99499
Q9KFR4_BACHD	82313 1rc	NAGK_HUMAN Fu	79025 alp	2CH6	2C3W	2CH5		Sc=6.01471, min distance = 0.99440
Q9KG76_BACHD	445237 1,	AMY1_HORVU Fu	64689 bet	1RP9	1W9W	1P6W	0.77	Sc=5.73786, min distance = 2.64409
Q9KG76_BACHD	445999 CI	GUN5_BACAG Fu	64689 bet	1W3L	1W9W	1H5V	0.77	Sc=5.6718, min distance = 2.478565
Q9KG76_BACHD	445999 CI	GUX6_HUMIN Fu	64689 bet	1OCN	1W9W	1OC7	0.77	Sc=5.75889, min distance = 2.02861
Q9KG76_BACHD	5326754 S	PHSM_ECOLI Fu	64689 bet	2ASV	1W9W	1QM5	0.88	Sc=5.65594, min distance = 0.54688
Q9KVB4_VIBCH	24901727	RHAMD_SALTY Fu	444305 L-	3D46	1XPJ	3CXO		Sc=5.88106, min distance = 1.75673
Q9KZR7_STRCO	11957363	HBA_HUMAN Ful	444124 HE	1NQP	1ODO	1RQA	0.81	Sc=6.80401, min distance = 2.24379
Q9KZR7_STRCO	11957363	HBB_HUMAN Ful	444124 HE	1NQP	1ODO	1RQA	0.81	Sc=6.80401, min distance = 2.04798
Q9KZR7_STRCO	11957364	HMOX1_HUMAN Fu	444124 HE	1OZW	1ODO	1S13	0.86	Sc=6.74465, min distance = 2.00462
Q9KZR7_STRCO	11957370	HMOX1_HUMAN Fu	444124 HE	1OZW	1ODO	1TWN		Sc=6.67497, min distance = 1.50837
Q9KZR7_STRCO	11957371	HMOX1_HUMAN Fu	444124 HE	1OZW	1ODO	1TWR		Sc=6.6834, min distance = 1.923271
Q9KZR7_STRCO	11957385	MYG_PHYCA Ful	444124 HE	1U7R	1ODO	2CMM		Sc=6.3387, min distance = 2.104388
Q9KZR7_STRCO	24893347	CATE_ECOLI Fu	444124 HE	1IPH	1ODO	1GGJ		Sc=6.79153, min distance = 2.00968
Q9KZR7_STRCO	4369001 C	HBA_HUMAN Ful	444124 HE	1NQP	1ODO	4HHB	0.8	Sc=6.80662, min distance = 2.12412
Q9KZR7_STRCO	444207 He	CP119_SULTO Fu	444124 HE	3B4X	1ODO	1UE8	0.99	Sc=6.80221, min distance = 2.08015

Q9KZR7_STRCO	444207 He	PRXC_CALFU Fu	444124 HE	2CPO	1ODO	1CPO	0.99	Sc=6.79941, min distance = 2.26652
Q9KZR7_STRCO	444522 HE	HBA_PAGBE Ful	444124 HE	1S5X	1ODO	1PBX	0.99	Sc=6.7682, min distance = 2.008550
Q9KZR7_STRCO	446189 CI	HBG1_HUMAN Fu	444124 HE	1I3E	1ODO	1I3D	0.89	Sc=6.809, min distance = 2.0198673
Q9KZR7_STRCO	6420167 M	CPXA_PSEPU Fu	444124 HE	1RE9	1ODO	2FE6	0.89	Sc=6.79473, min distance = 2.05184
Q9L142_STRCO	11957353	HBA_HORSE Ful	444124 HE	1Y8I	1GWI	1IWH	0.87	Sc=6.81085, min distance = 2.00225
Q9L142_STRCO	11957353	HBB_HORSE Ful	444124 HE	1Y8I	1GWI	1IWH	0.87	Sc=6.79941, min distance = 2.04738
Q9L142_STRCO	11957363	HBA_HUMAN Ful	444124 HE	1NQP	1GWI	1RQA	0.81	Sc=6.80596, min distance = 2.26921
Q9L142_STRCO	11957363	HBB_HUMAN Ful	444124 HE	1NQP	1GWI	1RQA	0.81	Sc=6.80436, min distance = 2.21217
Q9L142_STRCO	11957385	MYG_PHYCA Ful	444124 HE	1U7R	1GWI	2CMM		Sc=6.29044, min distance = 2.45778
Q9L142_STRCO	16214774	CP51_MYCTU Fu	444124 HE	2CIB	1GWI	2CI0	0.95	Sc=6.67152, min distance = 2.39366
Q9L142_STRCO	444207 He	COX1_THET8 Fu	444124 HE	1EHK	1GWI	2QPE	0.99	Sc=6.8053, min distance = 2.011576
Q9L142_STRCO	444207 He	CP119_SULTO Fu	444124 HE	3B4X	1GWI	1UE8	0.99	Sc=6.79941, min distance = 2.38082
Q9L142_STRCO	444207 He	HBB_HORSE Ful	444124 HE	1Y8I	1GWI	2ZLW	0.99	Sc=6.81619, min distance = 1.79426
Q9L142_STRCO	444207 He	PRXC_CALFU Fu	444124 HE	2CPO	1GWI	1CPO	0.99	Sc=6.80363, min distance = 2.08042
Q9L142_STRCO	444522 HE	HBA_PAGBE Ful	444124 HE	1S5X	1GWI	1PBX	0.99	Sc=6.79908, min distance = 2.06995
Q9L142_STRCO	444522 HE	NOS3_BOVIN Fu	444124 HE	1ZZS	1GWI	2HX2	0.99	Sc=6.76035, min distance = 2.28162
Q9L142_STRCO	446189 CI	HBG1_HUMAN Fu	444124 HE	1I3E	1GWI	1I3D	0.89	Sc=6.80757, min distance = 2.15569
Q9L142_STRCO	446406 CZ	MYG_PHYCA Ful	444124 HE	1U7R	1GWI	1J3F		Sc=6.3909, min distance = 2.175091
Q9L5C7_ECOLX	447026 NA	LYSC_CHICK Fu	5988 sucr	1JJ0	1YLT	1LZG		Sc=5.88395, min distance = 2.05825
Q9L5C8_ECOLX	447026 NA	LYSC_CHICK Fu	5988 sucr	1JJ0	1YLJ	1LZG		Sc=5.89735, min distance = 2.06509
Q9LCV7_STRCL	24856362	DHI1_HUMAN Fu	5886 NADP	1XU9	2JAH	3D4N		Sc=6.26077, min distance = 1.25053
Q9LCV9_STRCL	124687 1r	ODP1_ECOLI Fu	1132 thia	1L8A	1UPA	1RP7	0.79	Sc=6.03133, min distance = 2.22673
Q9LCV9_STRCL	13294447	DCIP_AZOBR Fu	1132 thia	2NXW	1UPA	2Q5Q		Sc=6.08525, min distance = 0.77701
Q9LCV9_STRCL	445886 CI	TKT1_YEAST Fu	1132 thia	1TRK	1UPA	1GPU	0.91	Sc=5.80108, min distance = 2.39028
Q9LCV9_STRCL	448671 2-	ILVB_YEAST Fu	1132 thia	1N0H	1UPA	1T9B		Sc=5.75284, min distance = 2.53169
Q9LCV9_STRCL	448673 PE	ILVB_YEAST Fu	1132 thia	1N0H	1UPA	1T9B		Sc=5.85289, min distance = 1.72385
Q9LCV9_STRCL	448723 1t	TKT1_YEAST Fu	1132 thia	1TRK	1UPA	1TKC	0.77	Sc=6.48124, min distance = 2.42358
Q9LCV9_STRCL	6102647 C	ODBA_HUMAN Fu	1132 thia	2BFD	1UPA	1WCI	0.9	Sc=6.14162, min distance = 2.07865
Q9LCV9_STRCL	6102749 T	ODBA_HUMAN Fu	1132 thia	2BFD	1UPA	2J9F	0.92	Sc=6.11687, min distance = 2.08000
Q9LCV9_STRCL	6102751 T	ODBA_HUMAN Fu	1132 thia	2BFD	1UPA	2BEW	0.76	Sc=6.17925, min distance = 2.08240
Q9LCV9_STRCL	6518182 C	POXB_LACPL Fu	1132 thia	2EZ4	1UPA	2EZ9	0.94	Sc=6.11007, min distance = 2.29334
Q9LCV9_STRCL	6518187 2	POXB_LACPL Fu	1132 thia	2EZ4	1UPA	2EZ8	0.87	Sc=6.10831, min distance = 2.25956
Q9LCZ4_THETH	16750062	CSK2A_MAIZE Fu	33113 gam	1LP4	1IY0	2OXD		Sc=5.68392, min distance = 2.47385
Q9LCZ4_THETH	24779676	CSK2A_MAIZE Fu	33113 gam	1LP4	1IY0	2PVK		Sc=6.3533, min distance = 2.071116
Q9LCZ4_THETH	3064778 H	ROCK1_HUMAN Fu	33113 gam	2V55	1IY0	2ETK		Sc=6.13777, min distance = 2.37153
Q9LCZ4_THETH	3547 Fasu	ROCK1_HUMAN Fu	33113 gam	2V55	1IY0	2ESM		Sc=5.93726, min distance = 2.24497
Q9LCZ4_THETH	448042 2e	ROCK1_HUMAN Fu	33113 gam	2V55	1IY0	2ETR		Sc=6.20928, min distance = 1.96269

Q9LCZ4_THETH	5326976 1	CSK2A_MAIZE Fu	33113 gam	1LP4	1IY0	1ZOE		Sc=5.71555, min distance = 2.49101
Q9LCZ4_THETH	5326977 1	CSK2A_MAIZE Fu	33113 gam	1LP4	1IY0	1ZOG		Sc=5.64926, min distance = 2.33603
Q9LCZ4_THETH	5326978 1	CSK2A_MAIZE Fu	33113 gam	1LP4	1IY0	1ZOH		Sc=5.65336, min distance = 2.27890
Q9LCZ4_THETH	72194 2-C	ENPL_CANFA Ful	6022 Aden	1TC6	1IY1	1QYE	0.89	Sc=6.07993, min distance = 2.12390
Q9N5G4_CAEEL	440141 9i	DHB1_HUMAN Ful	5886 NADP	1QYV	1XKQ	1QYW		Sc=6.42327, min distance = 2.21833
Q9N5G4_CAEEL	440141 9i	G6PD_LEUME Ful	5886 NADP	1H9A	1XKQ	1E7Y		Sc=6.44188, min distance = 2.13697
Q9NAV8_9ANNE	11957363	HBA_HUMAN Full	444124 HE	1NQP	1EWA	1RQA	0.81	Sc=6.63676, min distance = 2.12445
Q9NAV8_9ANNE	11957363	HBB_HUMAN Full	444124 HE	1NQP	1EWA	1RQA	0.81	Sc=6.63392, min distance = 2.43345
Q9NAV8_9ANNE	11957364	HMOX1_HUMAN Fu	444124 HE	1OZW	1EWA	1S13	0.86	Sc=6.51871, min distance = 2.15107
Q9NAV8_9ANNE	11957370	HMOX1_HUMAN Fu	444124 HE	1OZW	1EWA	1TWN		Sc=6.44298, min distance = 2.23985
Q9NAV8_9ANNE	11957371	HMOX1_HUMAN Fu	444124 HE	1OZW	1EWA	1TWR		Sc=6.45305, min distance = 2.31257
Q9NAV8_9ANNE	11957373	CATE_ECOLI Ful	444124 HE	1IPH	1EWA	1P81	0.79	Sc=6.65289, min distance = 2.20689
Q9NAV8_9ANNE	11957385	MYG_PHYCA Full	444124 HE	1U7R	1EWA	2CMM		Sc=6.05614, min distance = 2.30314
Q9NAV8_9ANNE	16214774	CP51_MYCTU Ful	444124 HE	2CIB	1EWA	2CI0	0.95	Sc=6.47666, min distance = 2.57537
Q9NAV8_9ANNE	16741062	MYG_PHYCA Full	444124 HE	1U7R	1EWA	1MBN	0.87	Sc=6.63989, min distance = 2.12552
Q9NAV8_9ANNE	16741183	CPXA_PSEPU Ful	444124 HE	1RE9	1EWA	2ZAW	0.92	Sc=6.45683, min distance = 2.44465
Q9NAV8_9ANNE	444207 He	HBB_HORSE Full	444124 HE	1Y8I	1EWA	2ZLW	0.99	Sc=6.65289, min distance = 2.32538
Q9NAV8_9ANNE	444207 He	PER_COPCI Full	444124 HE	1LY9	1EWA	1LY8	0.99	Sc=6.58492, min distance = 2.11302
Q9NAV8_9ANNE	444668 CI	MYG_PHYCA Full	444124 HE	1U7R	1EWA	1BVD	0.84	Sc=6.00907, min distance = 2.24913
Q9NAV8_9ANNE	446189 CI	HBG1_HUMAN Ful	444124 HE	1I3E	1EWA	1I3D	0.89	Sc=6.6637, min distance = 2.451038
Q9Q288_9HIV1	392622 ri	POL_HV1BR Full	64143 nel	2PYN	1OHR	1RL8		Sc=5.91729, min distance = 1.89519
Q9RBY6_PSESY	3080614 C	HIF1N_HUMAN Fu	51 2-Oxop	1H2L	2FCT	1H2K		Sc=5.67388, min distance = 2.80267
Q9RBY6_PSESY	3080614 C	JHD3A_HUMAN Fu	51 2-Oxop	2Q8C	2FCT	2OX0		Sc=5.69341, min distance = 2.46684
Q9RFR0_RHOMR	37792 gam	RHOA_HUMAN Ful	8977 1dar	1TX4	2BO7	1CXZ	0.99	Sc=6.0553, min distance = 2.157811
Q9RFR0_RHOMR	444783 EN	MIF_HUMAN Full	311 citri	1GD0	2BO4	1CA7		Sc=6.1665, min distance = 0.639224
Q9RFR0_RHOMR	444845 1d	RASH_HUMAN Ful	8977 1dar	2CE2	2BO7	1CLU	0.97	Sc=5.83339, min distance = 2.05593
Q9RFR0_RHOMR	447979 5G	KGUA_ECOLI Ful	8977 1dar	2AN9	2BO7	2ANB		Sc=5.98777, min distance = 2.29115
Q9RFR0_RHOMR	449108 CI	FTSZ1_METJA Fu	8977 1dar	2VAP	2BO7	1W58	0.99	Sc=5.60701, min distance = 1.98406
Q9RFR0_RHOMR	5326975 5	KGUA_MYCTU Ful	8977 1dar	1ZNY	2BO7	1ZNX	0.99	Sc=5.98048, min distance = 2.41933
Q9RFR0_RHOMR	93082 Gab	RAC3_HUMAN Ful	8977 1dar	2C2H	2BO7	2QME	0.99	Sc=6.08194, min distance = 2.30969
Q9RFR0_RHOMR	93082 Gab	RASH_HUMAN Ful	8977 1dar	2CE2	2BO7	121P	0.99	Sc=5.81137, min distance = 2.04831
Q9RGX9_9FLAO	444635 O3	MBL1_RAT Full=	439353 be	2KMB	1URX	3KMB	0.9	Sc=5.64673, min distance = 1.60325
Q9RHG4_9CHRO	101798 Me	LEC_GALNI Full	185698 al	1JPC	1ZHS	1MSA	0.94	Sc=5.98132, min distance = 0.41745
Q9RHG4_9CHRO	126154 Cy	Q54276_SERMA I	24139 ace	1UR9	1ZHS	1W1P		Sc=5.69173, min distance = 1.75180
Q9RHG4_9CHRO	445184 1,	NRAM_I56A2 Ful	439680 be	1V0Z	1ZHS	1W1X	0.89	Sc=5.71526, min distance = 1.67277
Q9RHG4_9CHRO	445184 1,	Q6PYX1_HUMAN I	185698 al	1ZLS	1ZHS	1ZLW	0.89	Sc=5.93347, min distance = 0.72981
Q9RHZ6_ALIAC	79025 alp	CDGT2_BACCI Fu	439341 ma	1KCL	1URG	1D3C	0.87	Sc=5.84332, min distance = 2.18584

Q9RP17_NEIME	445987 1h	SQHC_ALIAC Fu	5414 Octy	2SQC	1P4T	1H36		Sc=5.99851, min distance = 1.48591
Q9RX51_DEIRA	29435 1-D	CDGT_BACS0 Fu	64689 bet	1UKS	2BY0	1I75		Sc=5.77335, min distance = 2.324355
Q9RX51_DEIRA	440115 N-	SFTPD_HUMAN Fu	79025 alp	1PWB	2BHY	2ORJ		Sc=5.85243, min distance = 2.553335
Q9RYU4_DEIRA	24850913	NOS1_RAT Full	444243 FA	1TLL	1YRH	3B3M		Sc=5.70117, min distance = 3.171955
Q9RYU4_DEIRA	449551 FM	FLAV_DESVH Fu	444243 FA	1F4P	1YRH	5FX2	0.77	Sc=6.10331, min distance = 2.19639
Q9S7B3_URTDI	16058615	LYSC_CHICK Fu	24139 ace	1LZB	1EHH	2H9J		Sc=5.77725, min distance = 2.24733
Q9SLV5_ARAHY	12136 Ber	POL_HV1N5 Ful	311 citri	2GON	1Z1E	9HVP		Sc=5.672, min distance = 11.834354
Q9SLV5_ARAHY	439459 Ho	NIFD_KLEPN Fu	311 citri	1H1L	1Z1E	1QGU	0.92	Sc=5.9995, min distance = 2.047388
Q9SLV5_ARAHY	439459 Ho	NIFK_KLEPN Fu	311 citri	1H1L	1Z1E	1QGU	0.92	Sc=6.0029, min distance = 2.194738
Q9SLV5_ARAHY	51 2-Oxop	SERA_ECOLI Fu	311 citri	2P9E	1Z1E	1YBA	0.76	Sc=5.69982, min distance = 1.61864
Q9SPV4_CLABR	446535 CI	HNMT_HUMAN Fu	439155 Ad	2AOT	1M6E	1JQE	0.95	Sc=6.54705, min distance = 2.32911
Q9SPV4_CLABR	60961 ade	PIMT_PYRFU Fu	439155 Ad	1JG1	1M6E	1JG2	0.84	Sc=6.2931, min distance = 2.570029
Q9SPV4_CLABR	65482 sir	MTR1_RHOSH Fu	439155 Ad	1NW7	1M6E	1NW6	0.88	Sc=6.59715, min distance = 2.09270
Q9VF15_DROME	11957363	HBB_HUMAN Ful	444098 HE	1J40	2G3H	1RQA	0.77	Sc=6.67506, min distance = 1.90061
Q9VF15_DROME	11957385	MYG_PHYCA Ful	444098 HE	1A6M	2G3H	2CMM		Sc=6.21685, min distance = 2.09422
Q9VF15_DROME	16741061	MYG_PHYCA Ful	444098 HE	1A6M	2G3H	1MBC	0.83	Sc=6.73559, min distance = 2.11010
Q9W3H4_DROME	440141 9i	G6PD_LEUME Fu	5886 NADF	1H9A	1SNY	1E7Y		Sc=6.02948, min distance = 2.514875
Q9W3H4_DROME	6022 Ader	HMDH_HUMAN Fu	5886 NADF	1DQA	1SNY	1HW8		Sc=5.97633, min distance = 2.22725
Q9WWU5_PSEPU	10365 Lut	JHD3A_HUMAN Fu	51 2-Oxop	2Q8C	1OIK	2VD7		Sc=5.82878, min distance = 1.60870
Q9WWU5_PSEPU	444591 CI	IDH_ECOLI Ful	51 2-Oxop	1CW4	1OIK	1BL5		Sc=5.64286, min distance = 2.20260
Q9WXN8_THEMA	444809 Th	GUN5_BACAG Fu	10712 cel	2A3H	2O7I	1H5V		Sc=5.84691, min distance = 2.005858
Q9WXN8_THEMA	445999 CI	GUN5_BACAG Fu	10712 cel	2A3H	2O7I	1H5V		Sc=5.61221, min distance = 2.486768
Q9WXV1_THEMA	16741044	PYRDA_LACLC Fu	444243 FA	1JUB	1VHN	1JRB		Sc=5.64709, min distance = 2.48833
Q9WXV1_THEMA	446995 FM	CYB2_YEAST Fu	444243 FA	1KBI	1VHN	1LTD	0.77	Sc=5.80324, min distance = 2.38538
Q9WXW9_THEMA	445564 4-	GUNG_CLOCE Fu	64689 bet	1GA2	2H3H	1KFG	0.88	Sc=5.80993, min distance = 1.17636
Q9WXW9_THEMA	447607 1c	Q79G13_MYCTU	64689 bet	1UP0	2H3H	1UP2		Sc=5.85704, min distance = 2.18014
Q9WXW9_THEMA	6102765 C	Q9RX51_DEIRA	64689 bet	2BY0	2H3H	2BY2		Sc=5.67766, min distance = 2.006768
Q9WYC6_THEMA	11957399	PYRB_ECOLI Fu	311 citri	1R0B	2P2W	2H3E		Sc=6.13033, min distance = 0.93516
Q9WYC6_THEMA	439276 3-	TPIS_TRYBB Fu	311 citri	2VEK	2P2W	6TIM		Sc=5.62129, min distance = 2.01067
Q9WYC6_THEMA	439554 fu	A2NHM3_MOUSE	311 citri	1ZEA	2P2W	1CLZ		Sc=5.79082, min distance = 0.93766
Q9WYC6_THEMA	4797 1tpw	TPIS_TRYBB Fu	311 citri	2VEK	2P2W	1TTJ		Sc=5.6133, min distance = 1.920079
Q9WYC6_THEMA	51 2-Oxop	SERA_ECOLI Fu	311 citri	2P9E	2P2W	1YBA	0.76	Sc=5.71244, min distance = 1.95770
Q9WYC6_THEMA	8778 Laur	CTXA3_NAJAT Fu	311 citri	1XT3	2P2W	1H0J		Sc=5.77231, min distance = 0.85318
Q9WYE8_THEMA	15942680	6PGD_LACLM Fu	5886 NADF	2IZ0	1ZH8	2IZ1		Sc=6.17514, min distance = 2.20901
Q9WYE8_THEMA	448155 AD	ILV5_SPIOL Fu	5886 NADF	1YVE	1ZH8	1QMG		Sc=6.67918, min distance = 1.77003
Q9WYS2_THEMA	15942680	6PGD_LACLM Fu	5886 NADF	2IZ0	1VL8	2IZ1		Sc=6.28951, min distance = 2.270038
Q9WYS2_THEMA	440141 9i	G6PD_LEUME Fu	5886 NADF	1H9A	1VL8	1E7Y		Sc=6.47927, min distance = 2.02291

Q9WYV8_THEMA	445762 5'	MTTA_THEAQ Ful	34756 Acy	2ADM	1VQ1	2IH2	0.93	Sc=6.38258, min distance = 2.02574
Q9WYV8_THEMA	445971 CI	COMT_RAT Full=	34756 Acy	2CL5	1VQ1	1H1D	0.92	Sc=6.55702, min distance = 2.83874
Q9WYV8_THEMA	5326531 C	CHOMT_MEDSA Fu	34756 Acy	1FPQ	1VQ1	1FP1	0.92	Sc=6.52717, min distance = 2.27064
Q9WZ12_THEMA	439153 Di	ADH1B_HUMAN Fu	5893 nadi	1U3U	2HAE	1DEH	0.79	Sc=6.29418, min distance = 2.07131
Q9WZ12_THEMA	440516 CI	MDH_THETH Ful	5893 nadi	1BMD	2HAE	1BDM	0.79	Sc=6.29856, min distance = 2.03522
Q9WZ12_THEMA	445794 AD	ADHX_HUMAN Fu	5893 nadi	2FZW	2HAE	2FZE		Sc=6.15322, min distance = 1.93371
Q9WZ40_THEMA	11957399	PYRB_ECOLI Fu	311 citri	1R0B	1VMA	2H3E		Sc=6.09789, min distance = 1.37718
Q9WZ40_THEMA	439459 Ho	NIFD_KLEPN Fu	311 citri	1H1L	1VMA	1QGU	0.92	Sc=6.03814, min distance = 1.79070
Q9WZ40_THEMA	439459 Ho	NIFK_KLEPN Fu	311 citri	1H1L	1VMA	1QGU	0.92	Sc=6.03814, min distance = 2.03203
Q9WZ40_THEMA	51 2-Oxop	SERA_ECOLI Fu	311 citri	2P9E	1VMA	1YBA	0.76	Sc=5.78653, min distance = 2.14131
Q9WZ49_THEMA	1694 TBBt	CDK2_HUMAN Fu	6022 Aden	1GY3	2CE7	1P5E		Sc=5.72073, min distance = 1.79061
Q9WZ49_THEMA	444564 AD	BIOD_ECOLI Fu	6022 Aden	1DAD	2CE7	1BS1	0.91	Sc=5.99586, min distance = 2.09404
Q9WZ49_THEMA	444564 AD	MYS2_DICDI Fu	6022 Aden	1VOM	2CE7	1W9I	0.91	Sc=6.4694, min distance = 2.008745
Q9WZ49_THEMA	444842 CI	CDK2_HUMAN Fu	6022 Aden	1GY3	2CE7	1CKP		Sc=5.62251, min distance = 1.99274
Q9WZ49_THEMA	445940 O6	CDK2_HUMAN Fu	6022 Aden	1GY3	2CE7	1GZ8		Sc=6.20512, min distance = 1.78565
Q9WZ49_THEMA	447004 CI	MYS2_DICDI Fu	6022 Aden	1VOM	2CE7	1LVK		Sc=6.54306, min distance = 1.80835
Q9WZ49_THEMA	447955 1p	CDK2_HUMAN Fu	6022 Aden	1GY3	2CE7	1PXI		Sc=5.77922, min distance = 1.71642
Q9WZ49_THEMA	4564 1e1v	CDK2_HUMAN Fu	6022 Aden	1GY3	2CE7	1E1V		Sc=6.18548, min distance = 2.30957
Q9WZ49_THEMA	5327148 C	IPKA_RABIT Fu	6022 Aden	1JBP	2CE7	2ERZ		Sc=6.20726, min distance = 2.08988
Q9WZ49_THEMA	5957 Aden	KTHY_HUMAN Fu	6022 Aden	1NN3	2CE7	1E2Q	0.99	Sc=6.47541, min distance = 2.23264
Q9WZ49_THEMA	5957 Aden	MUTS_ECOLI Fu	6022 Aden	1OH7	2CE7	1W7A	0.99	Sc=6.48915, min distance = 1.89187
Q9WZ49_THEMA	5957 Aden	PSPF_ECOLI Fu	6022 Aden	2C98	2CE7	2C96	0.99	Sc=6.47968, min distance = 2.26909
Q9WZ49_THEMA	5957 Aden	RK_BOVIN Full=	6022 Aden	3C4Z	2CE7	3C4W	0.99	Sc=6.48327, min distance = 1.77567
Q9WZ49_THEMA	5957 Aden	VG17_BPT4 Ful	6022 Aden	2O0J	2CE7	2O0H	0.99	Sc=6.47736, min distance = 2.21357
Q9WZ49_THEMA	5957 Aden	Y059_METJA Fu	6022 Aden	2J9D	2CE7	2J9C	0.99	Sc=6.51462, min distance = 1.62993
Q9WZ49_THEMA	91532 AMP	BIOD_ECOLI Fu	6022 Aden	1DAD	2CE7	1DAG	0.99	Sc=6.05614, min distance = 2.39088
Q9WZ57_THEMA	6083 ader	HSP71_HUMAN Fu	33113 gam	2E8A	1JCG	1XQS	0.97	Sc=6.37418, min distance = 2.50989
Q9WZC8_THEMA	24180721	PIM1_HUMAN Fu	60961 ade	1YI4	2ZBV	3C4E		Sc=6.01482, min distance = 1.90391
Q9WZC8_THEMA	24851689	PIM1_HUMAN Fu	60961 ade	1YI4	2ZBV	3CY2		Sc=6.20874, min distance = 2.21540
Q9WZC8_THEMA	8975 2-Fl	SAHH_MYCTU Fu	60961 ade	3CE6	2ZBV	2ZJ0	0.99	Sc=6.08888, min distance = 2.34829
Q9WZC8_THEMA	9842606 2	ADK_HUMAN Ful	60961 ade	1BX4	2ZBV	2I6A		Sc=5.99896, min distance = 2.28726
Q9WZU0_THEMA	11708454	KAPCA_BOVIN Fu	5957 Aden	1Q24	1E4G	2VNW		Sc=6.08846, min distance = 2.54284
Q9WZU0_THEMA	3540 1yds	KAPCA_BOVIN Fu	5957 Aden	1Q24	1E4G	1YDS		Sc=5.87269, min distance = 2.28746
Q9WZU0_THEMA	440317 AT	PURT_ECOLI Fu	5957 Aden	1KJ8	1E4G	1KJJ	0.99	Sc=6.01621, min distance = 1.21612
Q9WZU0_THEMA	444564 AD	BIOD_ECOLI Fu	5957 Aden	1A82	1E4G	1BS1	0.91	Sc=6.38867, min distance = 2.40440
Q9WZU0_THEMA	444564 AD	MYS2_DICDI Fu	5957 Aden	1FMW	1E4G	1W9I	0.91	Sc=5.9269, min distance = 0
Q9WZU0_THEMA	444852 CI	ATPA1_BOVIN Fu	5957 Aden	2V7Q	1E4G	1COW	0.91	Sc=6.03385, min distance = 1.48950

Q9WZU0_THEMA	448043 2g	KAPCA_BOVIN Fu	5957 Aden	1Q24	1E4G	1Q8U		Sc=6.0221, min distance = 2.192879
Q9WZU0_THEMA	449240 1y	KAPCA_BOVIN Fu	5957 Aden	1Q24	1E4G	1YDR		Sc=5.93439, min distance = 2.31010
Q9WZU0_THEMA	6022 Aden	ACT1_DROME Ful	5957 Aden	2HF4	1E4G	2HF3	0.99	Sc=6.42391, min distance = 2.50189
Q9WZU0_THEMA	6022 Aden	ARSA1_ECOLX Fu	5957 Aden	1II0	1E4G	1IHU	0.99	Sc=6.40423, min distance = 2.41962
Q9WZU0_THEMA	6022 Aden	BIOD_ECOLI Ful	5957 Aden	1A82	1E4G	1DAD	0.99	Sc=6.40838, min distance = 2.29307
Q9WZU0_THEMA	6022 Aden	CLCN5_HUMAN Fu	5957 Aden	2J9L	1E4G	2JA3	0.99	Sc=6.46179, min distance = 1.21615
Q9WZU0_THEMA	6022 Aden	DDL_THET8 Full	5957 Aden	2ZDQ	1E4G	2ZDH	0.99	Sc=5.96079, min distance = 1.80714
Q9WZU0_THEMA	6022 Aden	HSP7F_YEAST Fu	5957 Aden	3D2F	1E4G	3C7N	0.99	Sc=5.96467, min distance = 2.42520
Q9WZU0_THEMA	6022 Aden	MUTS_ECOLI Ful	5957 Aden	1W7A	1E4G	1OH7	0.99	Sc=5.95521, min distance = 1.18192
Q9WZU0_THEMA	6022 Aden	PUR7_METJA Ful	5957 Aden	2Z02	1E4G	2YZL	0.99	Sc=6.40913, min distance = 2.00828
Q9WZU0_THEMA	6022 Aden	PURK_ECOLI Ful	5957 Aden	3ETH	1E4G	3ETJ	0.99	Sc=5.95286, min distance = 2.43470
Q9WZU0_THEMA	6022 Aden	PURP_METJA Ful	5957 Aden	2R7L	1E4G	2R7N	0.99	Sc=5.95358, min distance = 2.09368
Q9WZU0_THEMA	6022 Aden	Q72H90_THET2 I	5957 Aden	2BEK	1E4G	2BEJ	0.99	Sc=6.42175, min distance = 2.18439
Q9WZU0_THEMA	6022 Aden	REX_BACSU Full	5957 Aden	2VT3	1E4G	2VT2	0.99	Sc=6.40838, min distance = 2.20268
Q9WZU0_THEMA	6022 Aden	RK_BOVIN Full=	5957 Aden	3C4W	1E4G	3C4Z	0.99	Sc=6.39694, min distance = 2.41843
Q9WZU0_THEMA	6083 aden	PURP_METJA Ful	5957 Aden	2R7L	1E4G	2R7M	0.98	Sc=5.85757, min distance = 2.65162
Q9WZU0_THEMA	6083 aden	PURP_PYRFU Ful	5957 Aden	2R86	1E4G	2R85	0.98	Sc=6.34466, min distance = 2.11127
Q9WZU0_THEMA	6083 aden	Y059_METJA Ful	5957 Aden	2J9C	1E4G	2J9D	0.98	Sc=6.32842, min distance = 2.21059
Q9WZU0_THEMA	6852207 M	KAPCA_BOVIN Fu	5957 Aden	1Q24	1E4G	2GNI		Sc=5.91836, min distance = 2.41993
Q9WZU0_THEMA	91532 AMP	BIOD_ECOLI Ful	5957 Aden	1A82	1E4G	1DAG	0.99	Sc=6.4948, min distance = 2.034151
Q9WZU0_THEMA	91532 AMP	NIFH1_AZOVI Fu	5957 Aden	2C8V	1E4G	2AFK	0.99	Sc=6.50232, min distance = 1.80004
Q9WZV7_THEMA	10403821	HS90A_HUMAN Fu	6022 Aden	1BYQ	2C2A	3BM9		Sc=6.20746, min distance = 2.36784
Q9WZV7_THEMA	164628 de	PMS2_HUMAN Ful	6022 Aden	1EA6	2C2A	1H7U		Sc=6.02227, min distance = 2.33332
Q9WZV7_THEMA	440317 AT	BCKD_RAT Full=	6022 Aden	1GKZ	2C2A	1GJV	0.99	Sc=6.02364, min distance = 2.59923
Q9WZV7_THEMA	444842 CI	CDK2_HUMAN Ful	6022 Aden	1GY3	2C2A	1CKP		Sc=5.61221, min distance = 2.26609
Q9WZV7_THEMA	446090 1h	NDKC_DICDI Ful	6022 Aden	1KDN	2C2A	1HIY	0.97	Sc=6.00057, min distance = 2.20945
Q9WZV7_THEMA	5327103 2	HS90A_HUMAN Fu	6022 Aden	1BYQ	2C2A	2BYH		Sc=6.53575, min distance = 2.24739
Q9WZV7_THEMA	5957 Aden	PDK2_HUMAN Ful	6022 Aden	2BU8	2C2A	2BU2	0.99	Sc=6.03284, min distance = 2.39650
Q9WZV7_THEMA	6031 Uric	O33839_THEMA I	6022 Aden	1XJK	2C2A	1XJG		Sc=5.92328, min distance = 1.85215
Q9WZV7_THEMA	6083 aden	PSPF_ECOLI Ful	6022 Aden	2C98	2C2A	2VII	0.99	Sc=6.36603, min distance = 2.21693
Q9WZV7_THEMA	6804 guar	NDK_PYRHO Full	6022 Aden	2DYA	2C2A	2DXF	0.8	Sc=5.95567, min distance = 2.37055
Q9WZV7_THEMA	72194 2-C	ENPL_CANFA Ful	6022 Aden	1TC6	2C2A	1QYE	0.89	Sc=6.16992, min distance = 2.37256
Q9WZV7_THEMA	8977 1dar	NDK_PYRHO Full	6022 Aden	2DYA	2C2A	2DXE	0.8	Sc=6.01229, min distance = 2.36886
Q9WZW1_THEMA	164628 de	PMS2_HUMAN Ful	6022 Aden	1EA6	1T6X	1H7U		Sc=5.98559, min distance = 1.62263
Q9WZW1_THEMA	24180721	PIM1_HUMAN Ful	6083 aden	1YXU	1T6Y	3C4E		Sc=6.01625, min distance = 2.31355
Q9WZW1_THEMA	2519 caff	PYGM_RABIT Ful	6083 aden	8GPB	1T6Y	1GFZ		Sc=5.88079, min distance = 2.24689
Q9WZW1_THEMA	36735 Gpp	PARM_ECOLX Ful	6022 Aden	1MWM	1T6X	2ZGZ	0.79	Sc=5.74434, min distance = 1.77952

Q9WZW1_THEMA	440317 AT	BCKD_RAT Full	6022 Aden	1GKZ	1T6X	1GJV	0.99	Sc=5.9796, min distance = 1.858088
Q9WZW1_THEMA	448222 NE	ENPL_CANFA Fu	6022 Aden	1TC6	1T6X	1QY5	0.81	Sc=5.99575, min distance = 2.14532
Q9WZW1_THEMA	51 2-Oxop	SERA_ECOLI Fu	311 citri	2P9E	1MRZ	1YBA	0.76	Sc=5.67797, min distance = 1.55285
Q9WZW1_THEMA	5327121 I	PIM1_HUMAN Fu	6083 aden	1YXU	1T6Y	2C3I		Sc=6.30014, min distance = 1.06287
Q9WZW1_THEMA	5957 Aden	Y059_METJA Fu	6022 Aden	2J9D	1T6X	2J9C	0.99	Sc=6.02958, min distance = 1.76213
Q9WZW1_THEMA	611002 Op	PIM1_HUMAN Fu	6083 aden	1YXU	1T6Y	1YXX		Sc=6.22823, min distance = 0.86847
Q9WZW1_THEMA	65110 AIC	YL28_SCHPO Fu	6083 aden	2OOX	1T6Y	2QRE	0.79	Sc=6.31269, min distance = 1.84936
Q9WZW1_THEMA	65533 cor	O57693_THETE	6083 aden	1UXU	1T6Y	1UXT		Sc=5.9636, min distance = 1.905449
Q9WZW1_THEMA	656966 1y	PDE4D_HUMAN F	6083 aden	1TB7	1T6Y	1Y2E		Sc=6.10296, min distance = 2.07423
Q9WZW1_THEMA	656968 1y	PDE4B_HUMAN F	6083 aden	1ROR	1T6Y	1Y2H		Sc=5.9783, min distance = 1.837792
Q9WZW1_THEMA	657135 Op	PIM1_HUMAN Fu	6083 aden	1YXU	1T6Y	1YXV		Sc=5.76418, min distance = 2.64067
Q9WZW1_THEMA	8158 Pela	ALBU_HUMAN Fu	311 citri	1TF0	1MRZ	1E7E		Sc=5.72966, min distance = 0.41969
Q9WZW1_THEMA	8180 UNDE	ALBU_HUMAN Fu	311 citri	1TF0	1MRZ	1E7F		Sc=5.62647, min distance = 2.06271
Q9WZW1_THEMA	8582 Inos	PYGM_RABIT Fu	6083 aden	8GPB	1T6Y	2QN7	0.79	Sc=6.35386, min distance = 2.01788
Q9WZW1_THEMA	8778 Laur	CTXA3_NAJAT F	311 citri	1XT3	1MRZ	1H0J		Sc=5.62433, min distance = 1.02557
Q9WZW1_THEMA	8977 1dar	PARM_ECOLX Fu	6022 Aden	1MWM	1T6X	2ZGY	0.8	Sc=6.45843, min distance = 1.89845
Q9X022_THEMA	15942680	6PGD_LACLM Fu	5886 NADF	2IZ0	1O2D	2IZ1		Sc=5.99693, min distance = 1.97128
Q9X0A2_THEMA	15942680	6PGD_LACLM Fu	5886 NADF	2IZ0	1VP5	2IZ1		Sc=6.20026, min distance = 2.27822
Q9X0M3_THEMA	6022 Aden	HSP7F_YEAST F	5957 Aden	3D2F	1JI0	3C7N	0.99	Sc=5.92172, min distance = 2.20067
Q9X0M3_THEMA	6022 Aden	REX_BACSU Ful	5957 Aden	2VT3	1JI0	2VT2	0.99	Sc=6.24707, min distance = 1.05647
Q9X0M3_THEMA	6022 Aden	Y059_METJA Fu	5957 Aden	2J9C	1JI0	2J9D	0.99	Sc=5.78073, min distance = 2.04599
Q9X0V0_THEMA	854023 Ep	Q8WSF8_APLCA	23831 HEF	2BR7	1VR5	2BYQ		Sc=5.964, min distance = 0.7158267
Q9X1F0_THEMA	11970 2-H	ALDR_HUMAN Fu	311 citri	1US0	1VKF	2INZ		Sc=5.77737, min distance = 2.51763
Q9X1F0_THEMA	12793 Phe	SRC_HUMAN Ful	311 citri	1O4L	1VKF	1O4O		Sc=5.73638, min distance = 1.34566
Q9X1F0_THEMA	447532 1c	SRC_HUMAN Ful	311 citri	1O4L	1VKF	1O4H		Sc=6.06405, min distance = 2.15502
Q9X1F0_THEMA	51 2-Oxop	SERA_ECOLI Fu	311 citri	2P9E	1VKF	1YBA	0.76	Sc=5.64043, min distance = 2.55011
Q9X1F0_THEMA	8158 Pela	ALBU_HUMAN Fu	311 citri	1TF0	1VKF	1E7E		Sc=5.81438, min distance = 1.14929
Q9X1F0_THEMA	999 Benze	ALDR_HUMAN Fu	311 citri	1US0	1VKF	2INE		Sc=5.66115, min distance = 1.21965
Q9X1T2_THEMA	108714 9-	URIC_ASPFL Fu	764 guani	1XT4	1VMK	1R4S		Sc=5.97471, min distance = 2.30165
Q9X1T2_THEMA	1188 xant	XGPT_ECOLI Fu	764 guani	1A95	1VMK	1A96	0.92	Sc=5.85034, min distance = 2.60809
Q9X1T2_THEMA	242831 2-	TGT_ZYMMO Ful	764 guani	2PWU	1VMK	1S39		Sc=5.96437, min distance = 2.26204
Q9X1T2_THEMA	4369374 5	URIC_ASPFL Fu	764 guani	1XT4	1VMK	1WRR		Sc=5.83842, min distance = 2.11915
Q9X1T2_THEMA	447262 CI	TGT_ZYMMO Ful	764 guani	2PWU	1VMK	2Z1W	0.76	Sc=6.15544, min distance = 2.01477
Q9X1T2_THEMA	448488 1s	TGT_ZYMMO Ful	764 guani	2PWU	1VMK	1S38		Sc=6.02165, min distance = 2.21768
Q9X1T2_THEMA	4604 Oxor	URIC_ASPFL Fu	764 guani	1XT4	1VMK	1R4U		Sc=5.81465, min distance = 2.36346
Q9X1T2_THEMA	5287547 6	PURR_ECOLI Fu	764 guani	1WET	1VMK	2PUA		Sc=5.77823, min distance = 2.70675
Q9X1T2_THEMA	5326622 C	URIC_ASPFL Fu	764 guani	1XT4	1VMK	3BJP		Sc=5.91482, min distance = 1.86823

Q9X1T2_THEMA	76726 5,6	URIC_ASPFL Fu	764 guani	1XT4	1VMK	1WS2			Sc=5.76239, min distance = 2.29829
Q9X1T2_THEMA	8646 Guar	PNPH_HUMAN Fu	764 guani	1V2H	1VMK	1V41			Sc=5.81634, min distance = 2.59730
Q9X264_THEMA	311 citri	ANT3_HUMAN Fu	82313 1rd	1JVQ	1PW5	2ZNH			Sc=5.8428, min distance = 2.085176
Q9X264_THEMA	439710 rh	UGL_BACGL Ful	82313 1rd	2FV1	1PW5	2FV0			Sc=5.65049, min distance = 1.59188
Q9X264_THEMA	446882 CI	PPAP_HUMAN Fu	82313 1rd	1ND5	1PW5	1ND6			Sc=5.76203, min distance = 2.16669
Q9X266_THEMA	1051 Code	HEM1_RHOCA Fu	445062 CI	2BWP	1LW5	2BWO		0.86	Sc=6.05697, min distance = 1.55176
Q9X2E8_THEMA	16741136	DYR_ECOLI Ful	5886 NADP	1RA2	2HXV	1RX9	1		Sc=5.70375, min distance = 2.37688
Q9X4K7_STRCO	446903 NC	AVID_CHICK Fu	171548 bi	2JGS	1XNY	1LDO		0.99	Sc=6.1082, min distance = 1.307895
Q9X4K7_STRCO	73318 8-C	AVID_CHICK Fu	171548 bi	2JGS	1XNY	2A5B			Sc=6.2931, min distance = 0.397016
Q9X5N2_MYXXA	3371 fluf	ANDR_HUMAN Fu	78165 MES	2PNU	2BKL	2PIX			Sc=5.9571, min distance = 2.349501
Q9X9I8_YEREN	8180 UNDE	ALBU_HUMAN Fu	338 salic	2I2Z	2FN1	1E7F			Sc=5.94246, min distance = 1.37251
Q9XB59_ERWCA	3080614 C	HIF1A_HUMAN Fu	51 2-Oxop	1H2L	1NX8	1H2K			Sc=5.65876, min distance = 2.70067
Q9XB59_ERWCA	3080614 C	HIF1N_HUMAN Fu	51 2-Oxop	1H2L	1NX8	1H2K			Sc=5.65876, min distance = 2.70919
Q9XB59_ERWCA	3080614 C	JHD3A_HUMAN Fu	51 2-Oxop	2Q8C	1NX8	2OX0			Sc=5.67011, min distance = 2.78654
Q9XB61_ERWCA	5957 Ader	PANC_MYCTU Fu	91557 1tm	1N2E	1Q19	2A84		0.99	Sc=6.5014, min distance = 1.951868
Q9XB61_ERWCA	6083 ader	BLS_STRCL Ful	91557 1tm	1JGT	1Q19	1MC1		0.97	Sc=6.37245, min distance = 2.45267
Q9XB61_ERWCA	6083 ader	NADE_BACAN Fu	91557 1tm	2PZ8	1Q19	2PZA		0.97	Sc=6.35869, min distance = 2.84557
Q9XB61_ERWCA	6083 ader	PANC_MYCTU Fu	91557 1tm	1N2E	1Q19	2A7X		0.97	Sc=6.40649, min distance = 1.84332
Q9XEI3_HORVD	6914591 E	A2NHM3_MOUSE	439554 fu	1CLZ	1X38	2CJU			Sc=6.2384, min distance = 2.240502
Q9XEI3_HORVD	79025 alp	MALE_ECOLI Fu	64960 Pol	1LAX	1EX1	1ANF		0.89	Sc=5.8653, min distance = 0.606172
Q9XEI3_HORVD	95259 L-x	XYLA_STRRU Fu	64960 Pol	1XIE	1EX1	1XIC			Sc=5.69982, min distance = 2.12309
Q9XFX3_CYNCA	445686 CI	CARP_YEAST Fu	185698 al	1DPJ	1B5F	1FQ4			Sc=5.69273, min distance = 1.46639
Q9YAQ8_AERPE	100684 9-	RICI_RICCO Fu	190 adeni	2P8N	1WTA	1IL4			Sc=5.87488, min distance = 2.47117
Q9YAQ8_AERPE	11979 5-M	COBT_SALTY Fu	190 adeni	1JH8	1WTA	1JHM			Sc=5.69643, min distance = 2.50410
Q9YAQ8_AERPE	5287547 6	PURR_ECOLI Fu	190 adeni	2PUB	1WTA	2PUA		0.9	Sc=5.78164, min distance = 2.31872
Q9YAQ8_AERPE	5287830 6	Q8RLY5_LACHE	190 adeni	1S2D	1WTA	1S2I		0.93	Sc=5.68859, min distance = 2.55369
Q9YAQ8_AERPE	675 Dimed	COBT_SALTY Fu	190 adeni	1JH8	1WTA	1D0S			Sc=5.76592, min distance = 2.28931
Q9YAQ8_AERPE	764 guani	PURR_ECOLI Fu	190 adeni	2PUB	1WTA	1WET			Sc=5.84608, min distance = 2.46498
Q9YAQ8_AERPE	790 hypox	PURR_ECOLI Fu	190 adeni	2PUB	1WTA	1JFT			Sc=5.75384, min distance = 2.52870
Q9YAQ8_AERPE	96253 7-D	RICI_RICCO Fu	190 adeni	2P8N	1WTA	1IL3			Sc=5.87958, min distance = 2.46513
Q9YFU8_AERPE	15993 dAT	PRIM_BPT7 Ful	33113 gam	1E0J	1W5T	1CR2		0.97	Sc=6.49236, min distance = 1.90761
Q9YFU8_AERPE	3064778 H	ROCK1_HUMAN Fu	33113 gam	2V55	1W5T	2ETK			Sc=6.13126, min distance = 1.67443
Q9YFU8_AERPE	3547 Fasu	ROCK1_HUMAN Fu	33113 gam	2V55	1W5T	2ESM			Sc=5.95819, min distance = 2.03050
Q9YFU8_AERPE	444564 AD	MYS2_DICDI Fu	33113 gam	1MMN	1W5T	1W9I		0.91	Sc=6.46237, min distance = 2.38461
Q9YFU8_AERPE	445966 O6	CDK2_HUMAN Fu	6022 Aden	1GY3	1W5S	1H0V			Sc=6.23707, min distance = 1.93361
Q9YFU8_AERPE	4564 1e1v	CDK2_HUMAN Fu	6022 Aden	1GY3	1W5S	1E1V			Sc=6.18548, min distance = 1.80125
Q9YFU8_AERPE	4565 1h1r	CDK2_HUMAN Fu	6022 Aden	1GY3	1W5S	1H1R			Sc=6.40755, min distance = 1.32063

Q9YFU8_AERPE	5957 Ader	BIOD_ECOLI Fu	6022 Aden	1DAD	1W5S	1A82		0.99	Sc=6.49477, min distance = 2.55411
Q9YFU8_AERPE	5957 Ader	RK_BOVIN Full	6022 Aden	3C4Z	1W5S	3C4W		0.99	Sc=6.48085, min distance = 1.97625
Q9YFU8_AERPE	5957 Ader	SECA_ECOLI Fu	6022 Aden	2FSI	1W5S	2FSG		0.99	Sc=6.48384, min distance = 2.14143
Q9YFU8_AERPE	6083 ader	ENTP2_RAT Ful	33113 gam	3CJA	1W5T	3CJ7		0.97	Sc=6.36062, min distance = 2.24570
Q9YFU8_AERPE	60961 ade	SKY1_YEAST Fu	33113 gam	1Q99	1W5T	1Q97		0.93	Sc=6.25098, min distance = 2.05750
Q9YFU8_AERPE	60961 ade	SKY1_YEAST Fu	6022 Aden	1Q8Y	1W5S	1Q97		0.95	Sc=6.2974, min distance = 2.268470
Q9YFU8_AERPE	6132 Cyti	ECX1_PYRAB Fu	6022 Aden	2PO0	1W5S	2PO2			Sc=6.33681, min distance = 2.09939
Q9YFU8_AERPE	6132 Cyti	ECX2_PYRAB Fu	6022 Aden	2PO0	1W5S	2PO2			Sc=6.33191, min distance = 2.36725
Q9YFU8_AERPE	91532 AME	BIOD_ECOLI Fu	6022 Aden	1DAD	1W5S	1DAG		0.99	Sc=6.52064, min distance = 2.22219
Q9YFU8_AERPE	91532 AME	KIF1A_MOUSE F	33113 gam	1VFV	1W5T	1I6I		0.99	Sc=6.51429, min distance = 2.19490
Q9YFU8_AERPE	91532 AME	MTNK_BACSU Fu	6022 Aden	2OLC	1W5S	2PUL		0.99	Sc=6.5119, min distance = 1.926571
Q9ZB17_9LACT	11550797	PYGM_RABIT Fu	79025 alp	1H5U	1NSZ	2FET			Sc=6.2135, min distance = 2.359210
Q9ZB17_9LACT	21678292	PYGM_RABIT Fu	79025 alp	1H5U	1NSZ	3BD8			Sc=6.11537, min distance = 2.39205
Q9ZB17_9LACT	24864076	PYGM_RABIT Fu	79025 alp	1H5U	1NSZ	2QRH			Sc=6.19496, min distance = 2.19191
Q9ZB17_9LACT	4369162 1	PYGM_RABIT Fu	79025 alp	1H5U	1NSZ	1HLF			Sc=6.07153, min distance = 2.37137
Q9ZB17_9LACT	444393 CI	PYGM_RABIT Fu	79025 alp	1H5U	1NSZ	1AXR			Sc=5.86672, min distance = 1.87670
Q9ZB17_9LACT	445722 1-	PYGM_RABIT Fu	79025 alp	1H5U	1NSZ	1FU7			Sc=6.04634, min distance = 2.24480
Q9ZB17_9LACT	445825 CI	PYGM_RABIT Fu	79025 alp	1H5U	1NSZ	1GG8			Sc=5.91148, min distance = 2.14779
Q9ZB17_9LACT	447413 NT	PYGM_RABIT Fu	79025 alp	1H5U	1NSZ	1NOJ			Sc=5.87857, min distance = 2.02379
Q9ZB17_9LACT	447771 CI	PYGM_RABIT Fu	79025 alp	1H5U	1NSZ	1P4J			Sc=5.97621, min distance = 2.32553
[truncated: 499,375 more chars]
